# Expression of Human Endogenous Retroviruses in Systemic Lupus Erythematosus: Multiomic Integration With Gene Expression

**DOI:** 10.3389/fimmu.2021.661437

**Published:** 2021-04-27

**Authors:** Nathaniel Stearrett, Tyson Dawson, Ali Rahnavard, Prathyusha Bachali, Matthew L. Bendall, Chen Zeng, Roberto Caricchio, Marcos Pérez-Losada, Amrie C. Grammer, Peter E. Lipsky, Keith A. Crandall

**Affiliations:** ^1^ Computational Biology Institute, George Washington University, Washington, DC, United States; ^2^ Department of Biostatistics & Bioinformatics, Milken Institute School of Public Health, George Washington University, Washington, DC, United States; ^3^ RILITE Research Institute and AMPEL BioSolutions, Charlottesville, VA, United States; ^4^ Division of Infectious Diseases, Department of Medicine, Weill Cornell Medicine, New York, NY, United States; ^5^ Department of Physics, The George Washington University, Washington, DC, United States; ^6^ Lewis Katz School of Medicine, Temple University, Philadelphia, PA, United States; ^7^ CIBIO-InBIO, Centro de Investigação em Biodiversidade e Recursos Genéticos, Universidade do Porto, Vairão, Portugal

**Keywords:** HERV human endogenous retroviruses, lupus (SLE), RNA-Seq, deep learning - artificial neural network (DL-ANN), retrovirus < virus classification

## Abstract

Systemic lupus erythematosus (SLE) is a chronic autoimmune disease characterized by the production of autoantibodies predominantly to nuclear material. Many aspects of disease pathology are mediated by the deposition of nucleic acid containing immune complexes, which also induce the type 1interferon response, a characteristic feature of SLE. Notably, SLE is remarkably heterogeneous, with a variety of organs involved in different individuals, who also show variation in disease severity related to their ancestries. Here, we probed one potential contribution to disease heterogeneity as well as a possible source of immunoreactive nucleic acids by exploring the expression of human endogenous retroviruses (HERVs). We investigated the expression of HERVs in SLE and their potential relationship to SLE features and the expression of biochemical pathways, including the interferon gene signature (IGS). Towards this goal, we analyzed available and new RNA-Seq data from two independent whole blood studies using Telescope. We identified 481 locus specific HERV encoding regions that are differentially expressed between case and control individuals with only 14% overlap of differentially expressed HERVs between these two datasets. We identified significant differences between differentially expressed HERVs and non-differentially expressed HERVs between the two datasets. We also characterized the host differentially expressed genes and tested their association with the differentially expressed HERVs. We found that differentially expressed HERVs were significantly more physically proximal to host differentially expressed genes than non-differentially expressed HERVs. Finally, we capitalized on locus specific resolution of HERV mapping to identify key molecular pathways impacted by differential HERV expression in people with SLE.

## Introduction

Systemic lupus erythematosus (SLE) is a prototypic autoimmune disease with the primary demographic affected being women of childbearing ages (1). At least 70% of lupus cases are systemic (2). SLE is characterized by dysregulation of both the innate and adaptive immune systems, resulting in the production of pathogenic autoantibodies and increased activity of the type I interferon system. Whereas a number of genetic regions have been identified as associated with risk for lupus (3, 4), host genetics alone has failed to fully explain the disease, suggesting an important role for environmental stimuli. Exogenous stimuli, such as ultraviolet light and cigarette smoking have been implicated in SLE pathogenesis. Recent work has suggested that endogenous elements, including transposable elements (5) and human endogenous retroviruses (HERVs) (6) may also play a pathogenic role in SLE.

HERVs make up between 5-8% of the human genome and are a subset of transposable genomic elements (7). HERVs are structurally similar to infectious retroviruses and contain *gag*, *pol*, and *env* genes in their genomes. These genes code for core viral proteins, reverse transcriptase, and envelope proteins, respectively. Their integration site preferences on chromosomes can vary widely as well (8). Generally speaking, most HERVs have accumulated mutations in important genes over the course of evolutionary time that have rendered them non-functional. However, it is increasingly clear from the analysis of RNA-Sequencing (RNA-Seq) data that functional HERVs, defined by the presence of 5’ and 3’ Long Terminal Repeat (LTR) regions with an open reading frame in between, remain in the human genome and their expression may have roles in a diversity of diseases (9, 10).

HERVs have been implicated in the pathology of a number of autoimmune diseases including type 1 diabetes and rheumatoid arthritis, by a number of proposed mechanisms (11). Active HERVs can insert themselves at different locations in the genome and if they insert into the regulatory sequence of a gene, expression of that gene can be altered (11). In SLE, the genes of most interest to researchers have been those of the immune/inflammatory systems, in particular the interferon response genes which are commonly upregulated in SLE (12). It has been suggested that HERVs may contribute, at least in part, to the characteristic production of anti-nuclear antibodies (ANAs) in SLE patients (13) by impacting the activation of the type I interferon pathway causing dysregulation of tolerance and the generation of autoantibodies (5). Since interferon related genes are expressed in response to viral infections, there has been speculation that their upregulation could be related to HERV-mediated dysregulation. This leads to the second proposed mechanism for HERVs, namely molecular mimicry.

HERVs have the structure of exogenous retroviruses even though the HERVs themselves are not infectious. However, certain human anti-nuclear antibodies may cross react with HERV-encoded proteins (5, 14). Earlier experiments on lupus-prone mice reported immune-complexes with the *gp70* endogenous retroviral envelope protein, a finding also observed in human patients (15–17). These were among the first indications that endogenous retroviruses could be involved in SLE, with later studies on elevated levels of antiretroviral antibodies adding to this evidence (18). Mouse models have also shown that the lupus susceptibility locus *Sgp3* codes for a Kruppel-associated box zinc finger protein (KRAB-ZFP) which represses the expression of HERVs (19). Certain HERVs such as HRES-1 are capable of protein expression and have been studied in the context of cross reactivity with antibodies to the HTLV-1 virus (20). HRES-1 was also found to be inducible with IFN-γ and proposed to be involved in lupus susceptibility as well as the perpetuation of the interferon response in SLE (21, 22). Studies on DNA methylation have posited that defects in methylation are a mechanism by which HERV expression is upregulated in SLE (23, 24).

Despite this evidence of a potential pathogenic role, the expression of HERV-encoded mRNAs has not been examined in detail in SLE using next-generation sequencing technologies, such as RNA-Seq, because effective analytical tools have not existed until very recently to assess such data (25). Using our computational pipeline Telescope (26), we characterized locus-specific HERV expression in SLE whole blood data and identified differentially expressed HERVs between case and control groups from two independent datasets. We also characterized the expression of annotated coding and non-coding RNAs. Finally, we employed a novel deep learning approach to integrate these different omics data types to identify biological pathways where locus specific HERV differential expression and host gene differential expression were significantly associated.

## Materials and Methods

### RNA-Seq Datasets

We analyzed two independent datasets to identify differentially expressed HERVs and differentially expressed host genes associated with SLE. Both datasets were generated from the analysis of whole blood RNA-Seq, one new to this study and one publicly available (GSE72420).

The first dataset includes RNA-Seq data from whole blood samples taken from 48 individuals, including 23 healthy female controls and 25 individuals (22 females and 3 males) at varying stages of SLE (Whole Blood 1 - WB 1). Data were collected through the Temple University Lupus Program with an approved IRB protocol #23022. The majority of the SLE samples were from patients whose SLE was not currently active (SLE Disease Activity Index, SLEDAI < 7). The samples were sequenced using the Illumina HiSeq2000 platform using low-input RNA-Seq with paired-end 100 base pair (bp) reads.

The second dataset (GSE72420) included whole blood data from 117 patients, including 99 SLE patients (93 females and 6 males) and another 18 female control individuals (27) (Whole Blood 2 - WB 2). This study provided limited clinical data beyond gender, including ethnicity, and high or low ISM (Interferon Score Metric). Sequence data were collected using the Illumina HiSeq platform with single-end 50 bp reads.

This study was conducted in accordance with the ethical principles that have their origin in the Declaration of Helsinki.

### RNA-Seq HERV Identification and Expression

Telescope was used to identify HERVs and quantify their expression from the RNA-Seq data (26). Telescope uses a Bayesian mixture model and expectation-maximization algorithm (28) to reassign ambiguously mapped RNA-Seq fragments to the most likely originating locus, enabling accurate locus-specific HERV quantification. Our software pipeline uses Flexbar (29) to trim reads then Bowtie2 (30) to align them to the Hg38 reference genome using the very-sensitive-local setting and allowing for a maximum of 100 alignments per reads (--very-sensitive-local -k 100 --score-min L,0,1.6). Telescope then takes the bam files generated by the alignment to use Bayesian reassignment and up to 200 iterations of an expectation-maximization algorithm which has been modified to identify transposable elements (TEs) (--max_iter 200 --theta_prior 200000). With the Telescope software, TEs are inferred when the hallmark genomic signatures of such elements are identified, including 5’ and 3’ LTRs with an open reading frame between, thus inferring a functional TE. This step reassigns the ambiguously mapped reads to a single TE using a reference TE annotation containing 14,968 HERVs that span 60 different HERV families and 18 family groups. The TE annotation can be found at https://github.com/mlbendall/telescope_annotation_db/tree/master/builds/retro.hg38.v1. The output generated by Telescope is a table of TEs (labeled by chromosomal location) and their relative expression, quantified by read counts which were then used in the downstream analyses.

### Locus-Specific HERV Characterization

The annotation used to examine the assigned HERVs was created by Luis P. Iniguez and can be found at https://github.com/LIniguez/Telescope_MetaAnnotations. The annotation includes Coding-Non-Coding Identifying Tool (CNIT) designations for protein coding potential of sequences (31). CNIT analyzes adjoining nucleotide triplets (ANTs) to determine coding potential for sequences. The annotation also includes analyses on the HERV database by FANTOM5, which identifies whether the HERVs are in enhancer regions of the genome (32, 33). This solves a common issue in HERV analysis where HERVs from a given family are very similar and many software packages treat them identically, whereas Telescope can map them to individual loci along the reference genome and assess coding/non-coding status. An R script was created to search for genes close to the differentially expressed HERVs on their chromosomes in the ENSEMBL Hg38 reference, release 99. The script starts by looking within 500bp upstream and downstream of the HERV and expanding until it finds a gene or hits a 10kb limit (34). The genes were then queried against the PubMed database to find their function, if they are protein coding genes. Furthermore, we generated a null distribution of HERV locations by mapping non-significantly DE HERVs to the human genome, calculating genomic distance to the nearest protein coding gene. Then we used this distribution to test (Wilcoxon Signed Rank test with ranks based on distance and sign based on DE HERV) against the distribution of DE HERV distances to ask whether these distributions have significantly different means. Our goal in this particular genome distance analysis was to identify potential targets where HERV expression might alter gene regulation. These targets can then be studied in more detail from a mechanistic perspective once they are placed in a biochemical pathway framework (see below).

### RNA-Seq Host Gene Expression

Analysis of the RNA-Seq data with respect to host gene expression commenced with quality control of the raw sequence reads. FastQC files were used to visualize the quality of the reads in each sample (35). MultiQC was used to summarize FastQC reports (36). When deemed necessary following visual inspection, Trimmomatic was used to eliminate low quality reads and bases in each sample (37). A sliding window of 4 bases was used with an average quality of 30 as the cutoff. The first 14 bases were trimmed from each sequence to eliminate highly duplicated bases from all reads from non-random primer selection during the amplification process of the RNA-Seq. The data were then aligned to the Hg38 reference genome (RefSeq Accession: GCF_000001405.39) using the STAR aligner (38). The resulting SAM files were sorted and converted to BAM format using SamTools (39). FeatureCounts was then used to obtain raw counts for transcripts that aligned to known genes in the human genome (40).

### Differential Gene Expression

The library DESeq2 (v1.24.0) (41) was used to evaluate differential gene expression on counts values. Results were plotted using ggPlot2(v3.2.1) (42). The BioConductor package HTSFilter, which uses the Jaccard similarity index to calculate a filtering threshold for replicated RNA sequencing data, was used to filter out transcripts with low signal (43, 44). The “pAdjustMethod = BH” argument was used to adjust the p-value and control the false discovery rate (45). A minimum filtering threshold of 1 (s.min = 1) and a maximum filtering threshold of 200 (s.max = 200) were considered with 100 tested thresholds total (s.len = 100). The DESeq normalization method within HTSFilter was used (normalization = “DESeq”). An alpha value of 0.05 was chosen as a threshold for significant p-values. Any NA values were replaced with zeroes. The BioConductor biomaRt package was used to identify gene symbols and gene loci (46).

We performed a permutation test to statistically assess the significance of the overlap in both DE genes and DE HERVs for the two data sets (47). The permutation test consisted of randomly choosing the same number of genes and HERVs as were differentially expressed in each dataset from the lists of all genes and HERVs which were found to be expressed in our data. The number of genes and HERVs which were found to be randomly picked from both datasets—i.e., the intersection of the two lists—was then recorded. This was repeated for 200,000 iterations for both genes and HERVs separately. We then tested for statistical significance by comparing the number of genes and HERVs found to overlap between the datasets from random chance—via the permutations—*versus* our actual overlap. We used PANTHER (48) to perform overrepresentation analysis and MaAsLin2 to test associations in gene expression (49). MaAsLin2 allowed us to test for individual associations between the expression of genes and HERVs instead of only testing for differences in disease vs control. It accomplishes this by using general linear models that account for the expression of the other HERVs and genes, as well as their correlations with each other, so that the correlation it generates for any given HERV/gene pair is less influenced by the noise of the data. This provides a more accurate correlation than other methods because it helps compensate for the considerable noise in SLE data. The resulting beta coefficients were also used as input for the pathway analysis.

### Omics Pathways Enrichment Analysis

To find enriched biochemical pathways (those pathways with an observed overabundance of differentially expressed genes), we used *deepath* (50) an open source R Package. *deepath* is a generic tool for pathway enrichment analysis that allows users to calculate importance scores for omics features (i.e., gene expression in our study for both host genes and HERVs) appropriate for their study design (e.g., adjusting for multivariable testing and confounding factors). Employing user reference databases for mapping omics features to pathways (e.g., KEGG and GO terms), *deepath* identifies which pathways have significant associations with the underlying features. It performs statistical tests (e.g., Kolmogorov–Smirnov test) using the feature scores in the pathways against all ranks to calculate a p-value and false discovery rate (FDR) for hypothesis testing. Here, beta coefficients from MaAsLin2 linear models (51) were used as importance scores for omics feature (i.e., genes and HERVs), and ontology gene sets from the Molecular Signatures Database (MSigDB) (52) were used to perform the enactment statistical test. The Wilcoxon Sum Rank test (Mann Whitney U test) was employed to calculate a p-value for the null hypothesis, that there is no difference between the distribution of the score of a given feature with the pathways of interest *vs.* all other features in the study. Benjamini-Hochberg FDR correction (q = 0.1) was used as a threshold to report significant pathways (53). To look specifically at the interferon response, we searched Ensembl gene ID in the Interferome database to determine association specifically with the interferon response (54).

## Results

### Datasets

We analyzed two datasets for this study, WB 1 (new to this study) and WB 2 (publicly available) as described in the methods. Our WB1 data set included 23 healthy female controls and 25 individuals (22 females and 3 males). The controls ranged in age from 20-54 years of age average age 32.6 whereas the cases ranged in age from 19 - 60 years old with an average age of 35.7 years old. The cases had SLE Disease Activity Index) ranging from 0 to 21 with an average score of 4.3. The RNA-Seq output resulted in a minimum and maximum number of input reads across the samples of 71,275,250 and 79,606,353, respectively. The minimum and maximum number of uniquely mapped reads for the gene expression analysis was 50,681,878 (64.03% of the total reads) and 69,654,926 (88.03% of the total reads), respectively. The WB 2 dataset was characterized previously (27). In WB 1 the average reads/kb for genes was 352.8 for all genes and 428.8 for only genes which were DE. For WB 2 the reads/kb for genes was 423.4 for all genes and 456.8 when only examining the DE genes. The HERV data reflected the same trend of DE HERVs having more reads/kb than when looking at all detected HERVs as a whole, albeit with much fewer numbers. In WB 1 and WB 2 the average reads/kb for all HERVs were 10.3 and 3.7, respectively. When looking at only HERVs identified as DE in WB 1 and WB 2 those numbers rose to 47.7 and 29.4 reads/kb.

### Differential HERV Expression in SLE 

**Figure 1:**
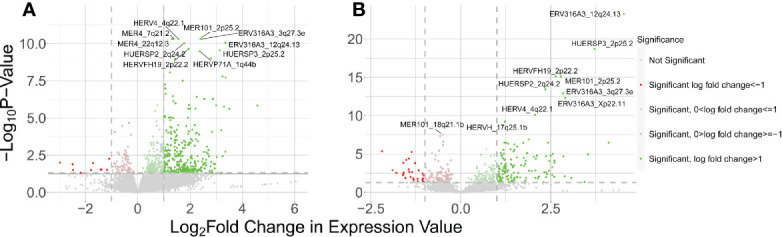
Results of host HERV DE analysis of control vs SLE. The positive LFC corresponds to HERVs upregulated in SLE and negative LFC corresponds to genes downregulated in SLE. **(A)** Volcano plot showing DE HERVs from the WB 1 dataset. **(B)** Volcano plot for WB 2.

**Figure 2:**
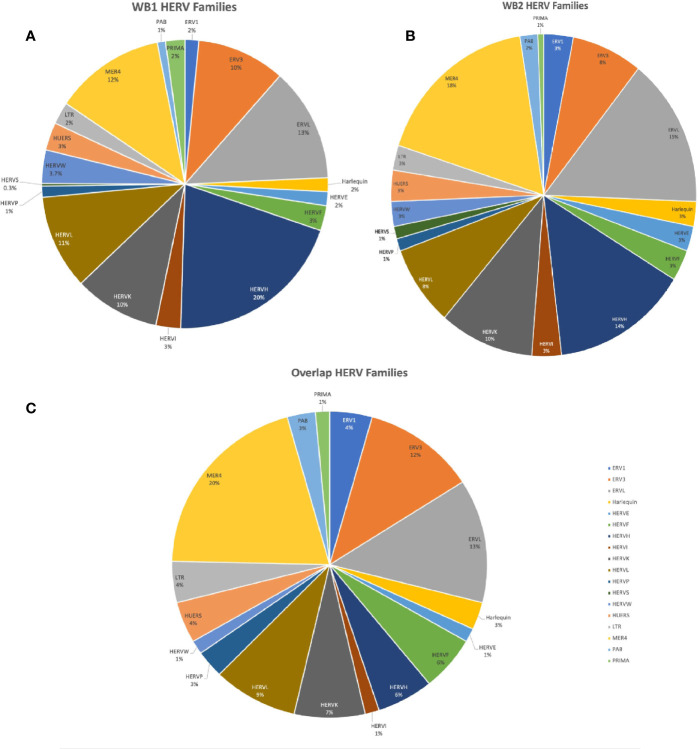
Charts of HERV family distributions of the DE HERVs in WB 1, WB 2, and of the HERVs DE in both. **(A)** Pie chart showing HERV families of the WB 1 DE HERVs. **(B)** Pie chart showing HERV families of the WB 2 DE HERVs. **(C)** Pie chart of the HERV families of HERVs which were DE in both WB 1 and WB 2.

In the WB 1 dataset, we identified 13,866 total expressed HERVs of which 321 HERVs were significantly differentially expressed between cases and controls in our DESeq2 with pAdjustMethod analysis with a FDR <0.05 and log2 fold change of ≥1 (**Figure 1A**) -see **Supplementary Table 1** for a complete list of all expressed and DE HERVs, their genomic locations, and distance to host genes, nearest host gene and associated P-values. Of these, there were 311 upregulated HERVs and only 10 downregulated HERVs. The HERV families of the DE HERVs were primarily of the ERV-L, ERV3, MER4, HERV-H, HERV-K, and HERV-L families (**Figure 2A**). Using the CNIT HERV annotation, we found that 35 of the 321 DE HERVs were protein coding and 286 were noncoding (31). The annotation also includes analyses on the HERV database by FANTOM5, which identifies whether the HERVs are in enhancer regions of the genome (32, 33). According to the annotation, 27 of the 321 total DE HERVs in WB 1 were in enhancer regions and 294 were not.

The WB 2 dataset yielded 12,376 expressed HERVs with 160 meeting the aforementioned criteria for significance (**Figure 1B**) with a similar skew towards upregulated DE HERVs -see **Supplementary Table 2** for a complete list of all expressed and DE HERVs, their genomic locations, and distance to host genes, nearest host gene and associated P-values. The HERV families of the DE HERVs in WB2 had similar representations as in WB1 (**Figure 2B**). The DE HERVs were predominantly upregulated in both datasets, although the differences in sample size for each set contributed to variation in the number of significant DE HERVs in each. There were 16 HERVs which were predicted as protein-coding and 144 that were noncoding. The number of HERVs which were in enhancer regions was 27, with 133 not being present in those regions.

Of the DE HERVs across the two datasets as well as their overlap (WB 1 = 321, WB 2 = 160, overlap = 69), there was higher representation in HERV families MER4, ERV-L, ERV-3, HERV-L, HERV-K, and HERV-H (**Figure 2**). Of the 471 differentially expressed HERVs across the two datasets, 69 HERVs were DE in both datasets. Using a permutation test for overlap (47), across 200,000 permutations the largest randomly generated overlap was of size 15, resulting in our inference of significant overlap of these 69 HERVs (p < 5e-6). Every family from the WB1 and WB2 DE HERVs was present in the overlap except the HERV-S family (**Figure 2C**). Among the 69 overlapping HERVs were 6 with a predicted protein product and 63 which were designated as noncoding. Between the two datasets, 15 of the 69 shared HERVs were in enhancer regions (**Supplementary Table 3**). We also tested for correlations among the identified HERVs between the two datasets. When all HERVs were analyzed, there was no significant correlation (Pearson correlation coefficient = 0.063; **Supplemental Figure 1A**), but when the analysis was restricted to just the DE HERVs, the two datasets were correlated (Pearson correlation coefficient = 0.602; **Supplemental Figure 1B**). This further supports consistency of inference relative to the impact of DE HERVs across these two datasets. Furthermore, we also explicitly tested the significance of the HERV DE and the association with each dataset by conducting a Fisher’s Exact Test (WB1/WB2 by DE/not). This test rejected the null hypothesis of no association with a P<0.00001, suggesting while there is overlap in DE HERVs, the DE HERVs in each dataset are independent.

### Host Gene Expression

**Figure 3:**
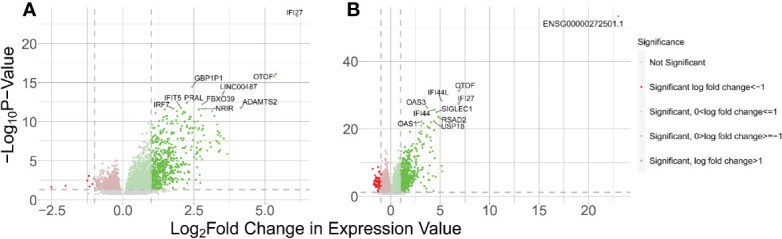
Results of host gene DE analysis of control vs SLE. The right side of each panel indicates genes upregulated in SLE and the left side indicates genes downregulated in SLE. **(A)** Volcano plot for the WB 1 dataset. **(B)** Volcano plot for the WB 2 dataset.

We identified 3,494 DE host genes in the WB 1 dataset (**Figure 3A**) (**Supplemental Table 4**). Of these genes, 1059 were downregulated and 2434 were upregulated. Imposing an absolute value log_2_ fold change of 1 to these results yielded 552 upregulated genes and 7 downregulated genes. We identified 4576 differentially expressed genes in the WB 2 dataset (**Figure 3B**) (**Supplemental Table 5**). Of these genes, 1604 were downregulated and 2972 were upregulated. Imposing an absolute value log_2_ fold change of 1 to these results yielded 662 upregulated genes and 64 downregulated genes. The two datasets were found to be independent using Fisher’s exact test comparing the numbers of DE genes between WB 1 and WB 2 (P < 0.00001). However, we observed 300 overlapping DE genes between the two data sets, but across 200,000 permutations the largest randomly generated overlap was 57 resulting in the inference of significant overlap in DE genes between the two datasets (p < 5e-6). Of the 10 most significantly DE host genes in both, most were interferon response related and have been implicated in lupus before including *IFI27*, *IFI44*, *IFI44L*, *OAS1*, *OAS3*, *OTOF*, and *RSAD2* (55). *SIGLEC-1* has been associated with the interferon signature as well as ancestry differences in kidney damage in SLE (56). *PRAL* is an lncRNA of interest in cancer research, including lung cancer, because of its modulation of the *p53* protein (57).

**Figure 4:**
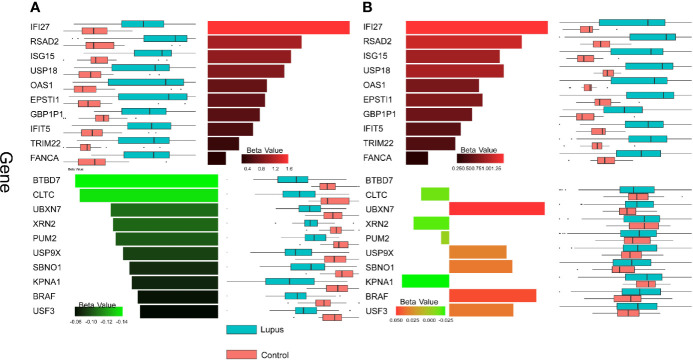
Gene expression patterns across datasets. Beta values calculated by MaAsLin2 are shown for up- and downregulated genes for the two datasets. Red bars represent upregulated genes and green bars represent downregulated genes. Box plots of log-transformed transcript counts are paired with each bar to show the distribution of counts in lupus (blue) and control (pink) samples. **(A)** The top 10 most DE genes in the up and down direction in WB 1 are shown. Top 10 genes were selected by FDR adjusted p-value and then sorted by magnitude of beta value. **(B)** The expression patterns for the same set of genes depicted in **(A)** are shown for WB 2.

The results obtained from MaAsLin2 provided insight into the consistency of the upregulated genes contrasted with the inconsistency of the downregulated genes. The top 10 most DE host genes from Whole Blood 1 according to MaAsLin2 were also uniformly upregulated in Whole Blood 2; whereas the top 10 downregulated genes did not show a consistent pattern (**Figure 4**).

### Integrated Analyses of HERV and Host Gene Expression

**Figure 5:**
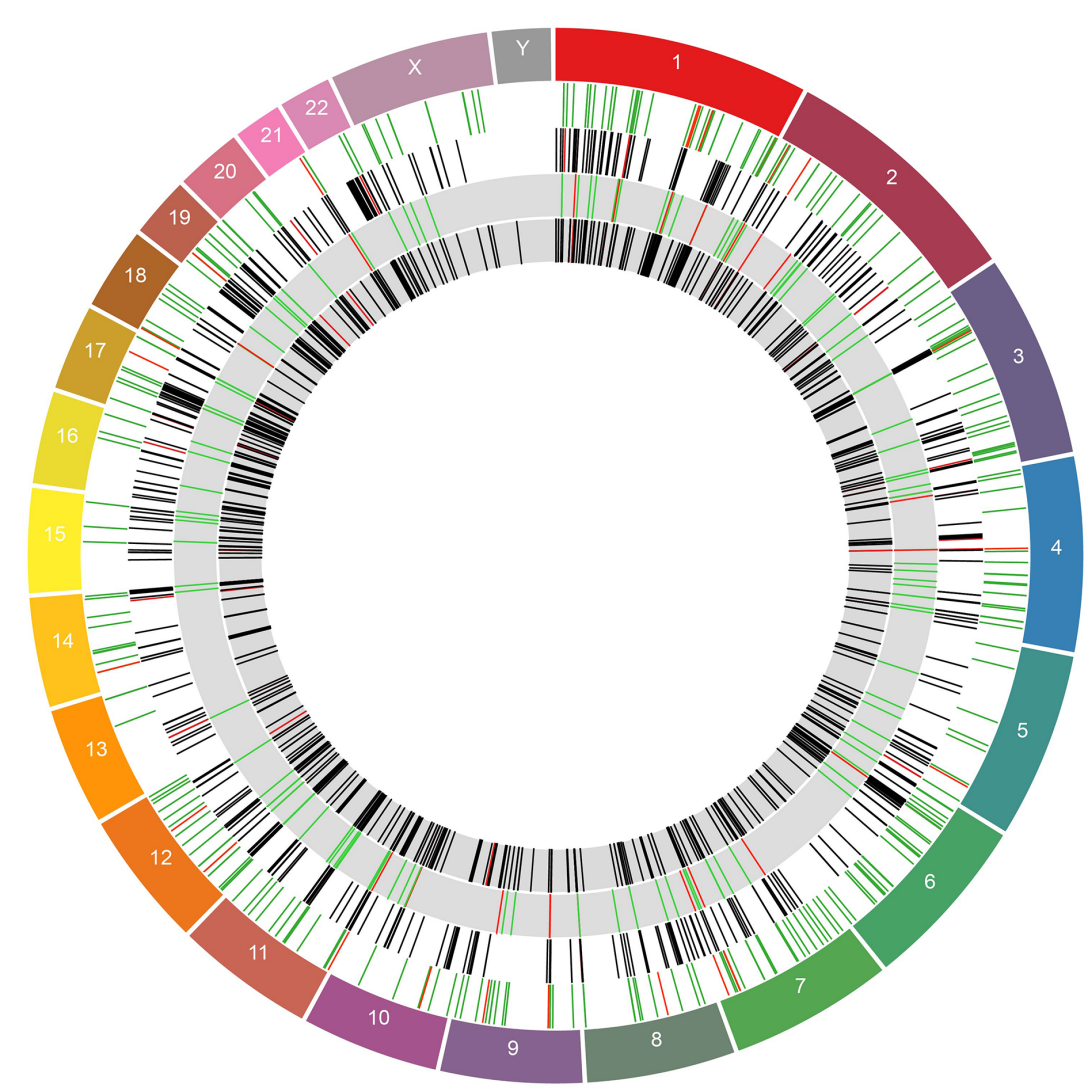
Circos plot showing DE genes and HERVs across the genome. The outermost ring represents the chromosomes and their boundaries. The inner rings show DE HERVs and genes in alternating order. The white rings are DE HERVs and genes from the WB 1, in that order. The grey rings represent the same for WB 2. The rings containing HERVs are colored by whether they were in an enhancer region, with red indicating HERVs in enhancer regions and green HERVs which were not. The rings containing genes are colored by whether the gene was among the 50 most DE genes for that dataset (determined by adjusted p-value). Genes which were among the top 50 most significantly DE are colored in red and genes which were not are colored black.

The DE host genes and HERVs had a relatively even spread throughout the genome and did not disproportionately originate from a small number of chromosomes (**Figure 5**). We tested for physical distance associations between DE HERVs and DE host genes, with the underlying hypothesis that a shorter physical distance allows for a greater opportunity for HERVs to impact regulation of gene expression for neighboring genes. Thus, we test for a shorter distance between DE HERVs and DE host genes against the null hypothesis of no difference in physical distance. To test for significance of reduced physical distance between DE HERVs and host genes relative to non-DE HERVs, we measured distances of all identified HERVs to host genes and then used the Wilcoxon Signed Rank test to test for differences between DE HERV distance to host genes relative to non-DE HERVs. We rejected the null hypothesis of no difference using this test with a P-value = 1.107e-07 (Supplemental **Figure 2**). We, therefore, proceeded to characterize the DE HERVs relative to host genes in greater detail. We found 284 genes in close proximity (<10kb) to the DE HERVs in the WB 1 dataset, including genes intersected by the HERVs. We determined that 162 of the 321 total HERVs in this dataset were intersecting genetic elements in either intronic or exonic gene regions. The genetic elements that the DE HERVs intersected were evenly split between protein coding genes and lncRNAs (82 *vs* 80, respectively). Some of the HERVs (39) intersected multiple genetic elements, such as an lncRNA and a protein-coding gene, with the remaining intersected genes being pseudogenes or other genetic elements. There were 94 HERVs located in intronic regions and 68 located in exons (**Supplemental Table 1**).

In WB 2, of the 160 DE HERVs, 103 intersected a host genetic element, with 50 and 54 of these being protein-coding genes and lncRNAs, respectively. The discrepancy between the total non-intergenic HERVs and total genetic elements that were intersected is caused by 25 HERVs that intersected multiple elements. There were 171 genes found to be in close proximity to the DE HERVs in the WB 2 dataset (**Supplemental Table 2**).

**Figure 6:**
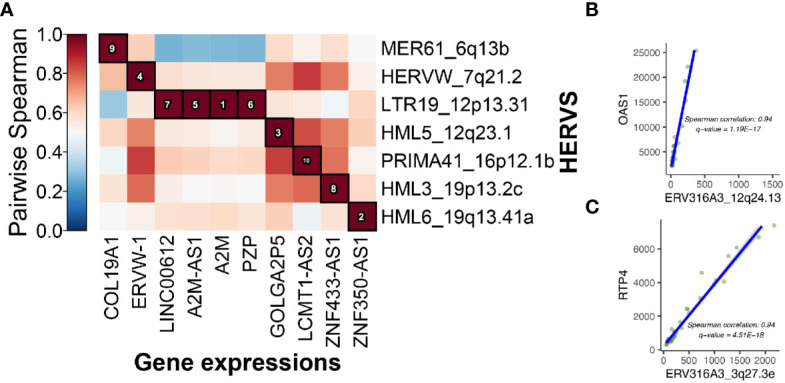
Integrative host gene and HERV associations. **(A)** 10 top associations between HERVs and host gene expression are shown. The associations are ranked by spearman correlation. **(B)** OAS1 is the closest gene to ERV316A3_12q24.13 and there is a significant correlation between their expression (coefficient = 0.94). **(C)**, The RTP4 gene codes for an interferon response protein and is the closest to ERV316A3_3q27.3e on chromosome 3.

Once we observed that some of the genes near the DE HERVs were involved in the immune response to viruses, we tested for associations between the expression levels of the genes and HERVs using MaAsLin2. The significant associations in the MaAsLin2 association testing were often HERVs and genes close to each other on their respective chromosomes. An example of that is the association of LTR19_12p13.31 with *LINC00612*, *A2M-AS1*, *A2M*, and *PZP* (**Figure 6A**). All four of those genes are located at the 12p13.31 locus with the DE HERV. Other examples are the association between ERV316A3_12q24.13 and *OAS1*, as well as the association of ERV316A3_3q27.3e with *RTP4* (**Figures 6B, C**). The latter two associations were highlighted because they were the most significant DE HERVs of the WB 1 and WB 2 datasets, respectively.

We next focused on the 69 HERVs that were DE in both whole blood datasets. A total of 83 host genes were found to be within 10 kb of them in the genome (**Supplementary Table 3**). Among the genes close to the HERVs, 52 were intersected by the HERVs. The HERVs that were intersecting genes were relatively evenly split between introns and exons (28 and 24, respectively). Only 21 of the total 52 intersected genes coded for a protein, of which 12 intersected an exon of a protein-coding gene. Whereas the other 31 were lncRNAs and other genetic elements. As with the results of the individual datasets, some of the HERVs (15) were intersecting multiple types of genomic elements.

When MaAsLin2 was used to test for significant associations between the 69 HERVs which were significant in both datasets and the 83 host genes found within 10kb of them, only 7 HERV/gene associations met the significance cutoff in WB1 (**Table 1**). The same association testing was then carried out on WB 2 to obtain the q-values and correlation coefficients for those HERVs and genes in that dataset (**Supplemental Tables 6** and **7**). Five of the resulting seven genes are part of the interferon response.

**Table 1:** Details of DE HERVs across both whole blood datasets, as well as the genes closest to the HERV and their MaAsLin2 correlation (closest within a 10kb expanding window).

HERV	Nearest Gene	WB1 q-value	WB1 Corr	WB2 q-val	WB2 Corr	Gene Description
ERV316A3_12q24.13	*OAS1*	1.19E-17	0.94080054	1.26E-44	0.91881796	Interferon response protein
ERV316A3_3q27.3e	*RTP4*	4.51E-18	0.94446275	5.07E-45	0.92023334	Interferon response protein
MER101_2p25.2	*RSAD2*	7.85E-18	0.94245529	9.91E-34	0.86730733	Interferon inducible antiviral binding protein
HERVFH19_2p22.2	*EIF2AK2*	5.01E-16	0.92348233	5.62E-47	0.92698783	Interferon inducible protein kinase
HERV4_4q22.1	*HERC6*	1.85E-17	0.93902961	7.68E-79	0.98104074	Ubiquitin ligase (IFN response)
HERVL18_3p21.31a	*LINC02009*	4.70E-25	0.97668767	5.47E-85	0.98533999	lncRNA
ERVLB4_17q25.3b	*RNF213*	2.61E-15	0.91444574	3.59E-15	0.67237303	Zinc finger protein

The q-value and correlation are for the beta value of the HERV/gene association in WB 1 and WB 2.

### Pathway Analysis

**Figure 7:**
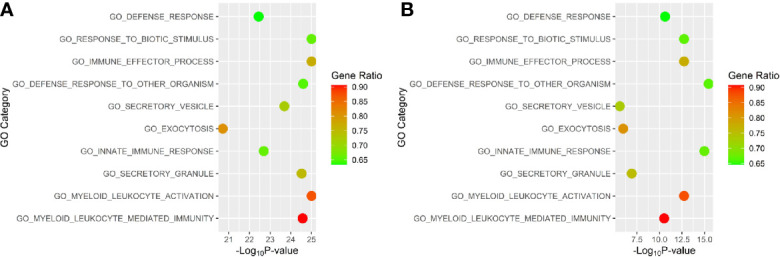
Top gene ontology (GO) terms for **(A)** WB 1 and **(B)** WB 2. Position on the horizontal axis is determined by significance and color is determined by the percentage of genes for the pathway that are differentially expressed.

The initial gene ontology enrichment analyses of the whole blood gene DESeq results yielded GO terms relevant to lupus pathology. The upregulated pathways in WB 1 were: immune system process (GO:0002376, adjusted p-value 8.34E-36), response to stress (GO:0006950, adjusted p-value 4.03E-33), immune response (GO:0006955, adjusted p-value 4.96E-28), and immune effector process (GO:0002252, adjusted p-value 1.65E-27). The top ten upregulated pathways were identified in WB 1 and WB 2 (**Figure 7**). Interestingly, the overlap was in the ‘go defense response to other organism’, ‘go response to biotic stimulus’, both suggesting a role of HERV interaction, and ‘go myeloid leukocyte activation and ‘go immune effector process’ both suggesting an immune response.

**Figure 8:**
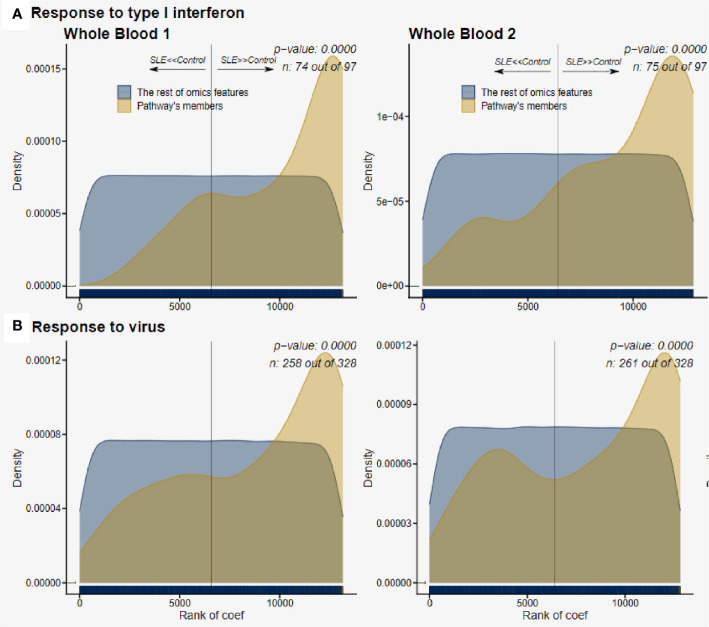
Pathway enrichment of host gene expression. **(A)**, Response to type I interferon was consistently upregulated in both WB 1 and WB 2. The increased pattern of the pathways is consistent across both datasets (WB 1 and WB 2 in that order), and we observe ~75% of the pathway genes in the samples. **(B)**, Response to virus was also upregulated significantly, again with over 75% of the pathway genes being observed in both datasets.

We then used *deepath* to assess pathway enrichment in greater detail. The WB1 data had 400 total pathways significantly enriched (FDR adjusted p-value of < 0.01). The WB2, likely due to its much larger sample size, had 184 total pathways that were enriched in SLE. The Response to Type I interferon pathway was highly significant in both datasets with a very consistent proportion (~75%) of genes in the pathway being DE and upregulated (**Figure 8A**). The broader category of Response to Virus was also highly significant in both datasets as well (**Figure 8B**). Other top pathways found by *deepath* were related to the innate immune response. GO terms such as Innate Immune Response and Defense Response to Virus were all observed to be significantly upregulated in lupus samples after adjustment for multiple testing (**Supplemental Tables 8** and **9**). These GO terms have some overlap with the response to type I interferon because of the nature of the biological processes they define.

**Figure 9:**
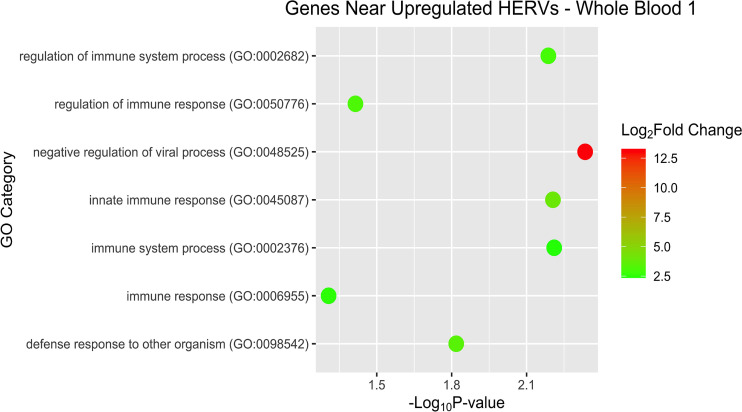
Overrepresentation analysis of the genes that were nearest to the significantly upregulated HERVs in WB 1. The position of the dot on the x-axis is based on significance and the color of the dot is based on the log2 foldchange of the pathway.

**Figure 10:**
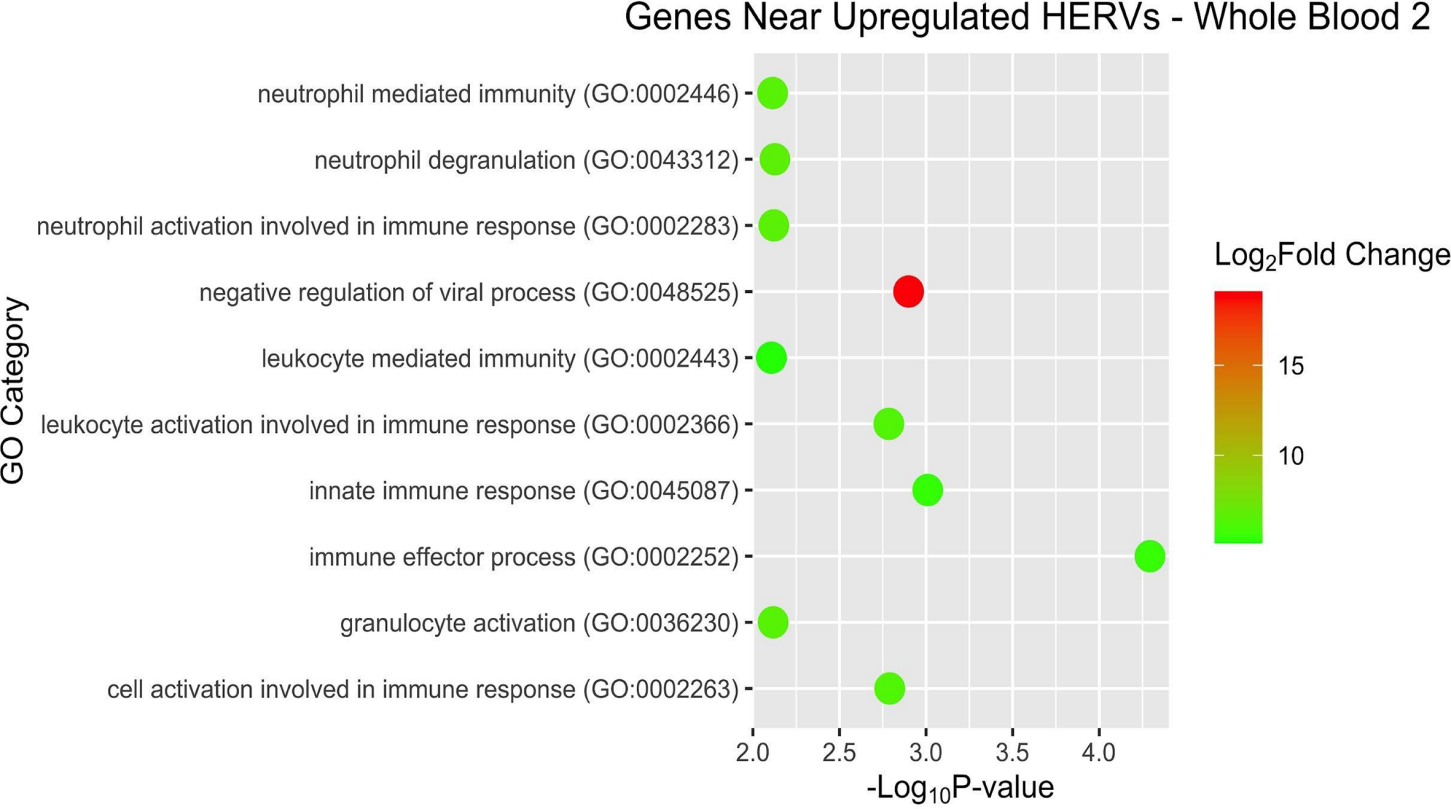
Overrepresentation analysis of the genes which were nearest to the significantly upregulated HERVs in WB 2. The position of the dot on the x-axis is based on significance and the color of the dot is based on the log2 foldchange of the pathway.

After examining pathways and overrepresentation for the gene expression data, we then sought to categorize the host genes near the DE HERVs by biological pathways as well. Some of the GO terms of interest for WB 1 were innate immune response and negative regulation of viral process (**Figure 9**). The nearby host genes in WB 2 showed much greater diversity of biological processes (42 *versus* 12) despite having only half (56 *versus* 95) of the number of proximal genes (**Figure 10**). The enriched pathways for WB 2 include neutrophil activation and degranulation, as well as leukocyte and neutrophil mediated immunity. The pathways for both sets were consistently innate immune response related and focused on the reaction of the immune system to a viral process.

Based on the results indicating GO term enrichment of response to a viral process, we analyzed the same host genes co-located with the DE HERVs with an interferon response database. Out of the 284 genes which were found to be in close proximity (<10kb) to the DE HERVs in the WB 1, 55 (19.3%) were associated with the interferon response including one associated with type I interferon response, 29 with both the type I and II interferon responses, and 25 with the type I/II/III response. Similarly, of the 171 host genes found to be in close proximity to the DE HERVs in WB 2, 40 (23.4%) were associated with the interferon response. Among those 40 genes, 16 were associated with the type I/II interferon response and 24 were associated with types I/II/III. Of the 83 genes in close proximity to the 69 shared HERVs of WB 1 and 2, 20 were part of the interferon response including 8 genes for type I/II and 12 genes with type I/II/III.

## Discussion

The goal of this study was to document the dysregulated expression of HERVs in SLE and to examine the interplay of HERVs and immune-related gene expression. The locus specific HERV identification provided by Telescope allowed us to look at the expression of HERVs as well as the genes nearby them. This was in contrast to previous studies on the role of transposable elements in lupus, where HERVs were simply grouped taxonomically based on a classification of repetitive elements with resolution to the superfamily, class, and type level (58). Limitations of previous approaches related to the high levels of sequence homology among HERVs, which can cause a single sequence to map onto many different HERVs, creating ambiguity in specifically assigning these reads. Telescope addresses that pervasive issue by using Bayesian reassignment to designate the multi-mapped reads to a single HERV. This improves the accuracy of the HERV assignment compared to other tools that randomly assign or discard ambiguous reads (26, 59, 60). As a result of using Telescope, we were able to accurately quantify the locus specific expression of HERVs. This allowed us to examine their neighboring genes, whether they were in enhancer regions, as well as assess their protein coding potential. The differences in the total number of HERVs and genes found to be significant in WB 1 and WB 2 may be related to imbalances in sample size between the two datasets. Whereas WB 2 had more samples overall, the sampling was skewed towards individuals with SLE, compared to the near 1:1 case/control ratio in WB 1. This imbalance in cases/controls in WB 2 may have impacted the output of our statistical approaches.

Whereas previous studies have looked at either host gene expression or HERV expression to identify significant associations with SLE, recent work has applied machine learning (ML) approaches to predict clinical outcomes based on gene expression (61). The integration of clinical data with multiomics data is particularly well suited for unsupervised ML approaches. However, multiomics data can present challenges to such approaches as there are significant sources of noise in the data, including different sequencing platforms and heterogeneity in feature profiles. Deep learning models have previously been employed to extract linear and non-linear relationships on the large, high-dimensional datasets of genomics (62–67). Taking advantage of the deep neural network allowed us to make effective use of the data and to account for some of the background noise inherent in the data. Therefore, a multi-resolution clustering approach was applied here, coupling clinical metadata and omics data to find significant clusters of omics data associated with phenotypes of interest (e.g., SLE status). We then employed a novel deep learning approach to identify biological pathways where locus specific HERV and host gene differential expression were significantly associated with SLE status.

The whole blood datasets demonstrated significant overlap in their differentially expressed HERVs and genes (p < 5e-6). Almost half [69] of the 160 significant HERVs in WB 2 were also significantly over-expressed in WB 1, which was a highly significant amount when compared with the amounts generated by our permutation testing. This pattern is very different from that observed in HERV expression in cancers (68). This points toward a more consistent set of HERV expression in SLE, not random differences in expression. The locus specific mapping of Telescope allowed us to determine where the HERVs fell in relation to protein-coding genes, lncRNAs, and other genetic elements. The majority of HERVs intersected at least one type of genetic element, sometimes multiple elements at once. But for the HERVs as a whole, the intersected or nearby protein-coding genes were associated with the host immune response to viruses. A number of HERV families including HERV-K, HERV-H, and MER4 were over-expressed in SLE. The HERV-K family specifically was moderately over-represented (WB 1 = 9.65%, WB 2 = 10%, and overlap = 7.2%) when compared to its total representation in the annotation (3.2%). The slightly elevated representation of the ERV-K family in the DE HERVs was interesting because previous research has suggested that the ERV-K family is the most biologically active family of HERVs capable of producing viral proteins (9). However, the low percentage of ERV-K relative to the sum of the other HERV families could mean that the contribution of HERVs to SLE pathology may be more related to dysregulation of immune gene expression as opposed to stimulation of autoantigens. Notably, the ‘protein-coding potential’ of the DE HERVs was approximately 10% for each dataset and that proportion was similar for the overlapping HERVs (WB 1 = 10.9%, WB 2 = 10%, and overlap = 8.7%). HERVs identified as protein coding could potentially produce intact viral proteins, which could stimulate the host immune system. The proportions of HERVs which were found in identified enhancer regions varied more between the two datasets (WB 1 = 8.4%, WB 2 = 16.9%, and overlap = 21.7%). Of note, the DE HERVs shared between both datasets were more frequently found in enhancer regions. The HERVs which were annotated as being in enhancer regions were split between intergenic and intragenic regions, both of which can harbor enhancers (69). Previous studies in multiple sclerosis found that some of the increased HERV expression was a byproduct of the activation of overlapping enhancers for genes which were involved in the immune response (70). In SLE, a similar phenomenon may be occurring, in which HERVs are upregulated as part of the response to type 1 interferon. On the other hand, retroelements themselves can also act as enhancers or promoters for certain interferon stimulated genes, as well as functioning in other regulatory capacities. As an example of this, the IFN inducibility of *AIM2* is conferred by retroelements and *ACE2* has co-opted an intronic HERV to regulate expression via alternative splicing (71). Interestingly, one of the HERVs that was DE in both of our datasets (MER61_1q23.1c) is next to the *AIM2* gene in an intergenic region. Whether the HERVs in enhancer regions are involved in the dysregulation of their neighboring genes requires further validation and testing, but our results provide clear targets for such follow-up work.

The biological pathways associated with the genes in close proximity to DE HERVs were mostly related in some way to the immune system or the innate immune response to viruses. This is in agreement with other analyses of gene expression in people with SLE and current understanding of lupus pathology. The two whole blood datasets overlapped in DE genes as well as overrepresentation for pathways related to immune response to viruses, suggesting a pathogenic role for HERVs in SLE (**Figure 7**). This was further supported by the results of the *deepath* analysis, which also showed significant pathways associated with ‘response to virus’ and ‘defense response to virus’, as well as the type I interferon response and innate immune response.

The proximity of the DE HERVs to genes involved in the innate immune system is consistent with the involvement of innate immunity in SLE. The correlation results showing only 7 total host genes that were significantly correlated with their closest neighboring HERVs was an unexpected result. We expected to find a more widespread correlation between the expression of the HERVs and the neighboring host genes. Whereas 5 of the 7 host genes with a significant correlation to the HERVs closest to them were interferon response genes, expression of many other genes involved in host immunity did not have a significant correlation with their HERV neighbors. This indicates that the DE of HERVs in lupus cannot be solely attributed to the increased or decreased expression of the nearest gene to them, even if those genes are immune related. HERVs have been shown to play roles in the immune regulatory networks of many mammals including humans. HERVs with *STAT1* (signal transducer and activator of transcription) and *IRF1* (interferon regulatory factor) binding sites have been found to be enriched near interferon stimulated genes in CD14+ macrophages as well (72). Many of the HERVs that were found to be DE near immune-involved genes or in their regulatory regions could also have direct or indirect roles in the dysregulation of their expression in SLE.

Additional information is required on HERV expression in other SLE datasets as well as the genes adjacent to them in order to obtain a more complete picture of the influence that HERVs may exert on the drivers of lupus pathology. Our study has identified a number of clear targets for further analysis of their impact on neighboring gene expression and on lupus immunopathology overall.

## Data Availability Statement

The datasets presented in this study can be found in online repositories. The names of the repository/repositories and accession number(s) can be found below: https://www.ncbi.nlm.nih.gov/genbank/, PRJNA717024.

## Author Contributions

PL, AG, and KC conceived of the project. RC provided sequence data for WB 1 samples. NS performed HERV analyses and TD performed host gene analyses. AR and NS performed integrative and multivariable association testing, and AR and TD performed pathway enrichment analyses. MB consulted on the HERV analyses and interpretations and provided novel HERV annotations. CZ, PB, MP-L, MB, AR, and KC all provided feedback on analyses. NS, TD, PL, and KC developed the initial version of the manuscript. All authors contributed to the article and approved the submitted version.

## Funding

This publication was supported in part by Award Number UL1TR001876 from the NIH National Center for Advancing Translational Sciences and Award Number CA206488 from the NIH National Cancer Institute. The project was also partially supported by the RILITE Foundation.

## Disclaimer

This publication was supported in part by Award Number UL1TR001876 from the NIH National Center for Advancing Translational Sciences and Award Number CA206488 from the NIH National Cancer Institute. Its contents are solely the responsibility of the authors and do not necessarily represent the official views of the National Center for Advancing Translational Sciences, the National Cancer Institute, or the National Institutes of Health.

## Conflict of Interest

PB, AG and PL were employed by AMPEL BioSolutions.

The remaining authors declare that the research was conducted in the absence of any commercial or financial relationships that could be construed as a potential conflict of interest.

## References

[1] Pons-Estel GJ Alarcón GS Scofield L Reinlib L Cooper GS. Understanding the Epidemiology and Progression of Systemic Lupus Erythematosus. Semin Arthritis Rheumatol (2010) 39:257–68. 10.1016/j.semarthrit.2008.10.007 PMC2813992 19136143

[2] Ugarte-Gil MF González LA Alarcón GS. Lupus: The New Epidemic. Lupus (2019) 28:1031–50. 10.1177/0961203319860907 31299878

[3] Kwon Y-C Chun S Kim K Mak A. Update on the Genetics of Systemic Lupus Erythematosus: Genome-Wide Association Studies and Beyond. Cells (2019) 8(10):1180. 10.3390/cells8101180 PMC6829439 31575058

[4] Oparina N Martínez-Bueno M Alarcón-Riquelme ME. An Update on the Genetics of Systemic Lupus Erythematosus. Curr Opin Rheumatol (2019) 31:659–68. 10.1097/BOR.0000000000000654 31436585

[5] Kelly M Lihua S Zhe Z Li S Yoselin P Michelle P. Transposable Element Dysregulation in Systemic Lupus Erythematosus and Regulation by Histone Conformation and Hsp90. Clin Immunol (2018) 197:6–18. 10.1016/j.clim.2018.08.011 30149120 PMC6258342

[6] Nelson P Rylance P Roden D Trela M Tugnet N. Viruses as Potential Pathogenic Agents in Systemic Lupus Erythematosus. Lupus (2014) 23:596–605. 10.1177/0961203314531637 24763543

[7] Belshaw R Pereira V Katzourakis A Talbot G Paces J Burt A. Long-Term Reinfection of the Human Genome by Endogenous Retroviruses. Proc Natl Acad Sci U S A (2004) 101:4894–9. 10.1073/pnas.0307800101 PMC387345 15044706

[8] Taruscio D Manuelidis L. Integration Site Preferences of Endogenous Retroviruses. Chromosoma (1991) 101:141–56. 10.1007/BF00355364 1790730

[9] Hohn O Hanke K Bannert N. Herv-K(HML-2), the Best Preserved Family of HERVs: Endogenization, Expression, and Implications in Health and Disease. Front Oncol (2013) 3:246. 10.3389/fonc.2013.00246 24066280 PMC3778440

[10] Bannert N Hofmann H Block A Hohn O. Hervs New Role in Cancer: From Accused Perpetrators to Cheerful Protectors. Front Microbiol (2018) 9:178. 10.3389/fmicb.2018.00178 29487579 PMC5816757

[11] Bengtsson A Blomberg J Nived O Pipkorn R Toth L Sturfelt G. Selective Antibody Reactivity With Peptides From Human Endogenous Retroviruses and Nonviral Poly(Amino Acids) in Patients With Systemic Lupus Erythematosus. Arthritis Rheumatol (1996) 39:1654–63. 10.1002/art.1780391007 8843855

[12] Trela M Nelson PN Rylance PB. The Role of Molecular Mimicry and Other Factors in the Association of Human Endogenous Retroviruses and Autoimmunity. APMIS (2016) 124:88–104. 10.1111/apm.12487 26818264

[13] Sukapan P Promnarate P Avihingsanon Y Mutirangura A Hirankarn N. Types of DNA Methylation Status of the Interspersed Repetitive Sequences for LINE-1, Alu, HERV-E and HERV-K in the Neutrophils From Systemic Lupus Erythematosus Patients and Healthy Controls. J Hum Genet (2014) 59:178–88. 10.1038/jhg.2013.140 24430577

[14] Wu Z Mei X Zhao D Sun Y Song J Pan W. DNA Methylation Modulates HERV-E Expression in CD4+ T Cells From Systemic Lupus Erythematosus Patients. J Dermatol Sci (2015) 77:110–6. 10.1016/j.jdermsci.2014.12.004 25595738

[15] Izui S McConahey PJ Theofilopoulos AN Dixon FJ. Association of Circulating Retroviral gp70-anti-gp70 Immune Complexes With Murine Systemic Lupus Erythematosus. J Exp Med (1979) 149:1099–116. 10.1084/jem.149.5.1099 PMC2184871 221610

[16] Mellors RC Mellors JW. Type C RNA Virus-Specific Antibody in Human Systemic Lupus Erythematosus Demonstrated by Enzymoimmunoassay. Proc Natl Acad Sci U S A (1978) 75:2463–7. 10.1073/pnas.75.5.2463 PMC392574 209465

[17] Yoshiki T Mellors RC Strand M August JT. The Viral Envelope Glycoprotein of Murine Leukemia Virus and the Pathogenesis of Immune Complex Glomerulonephritis of New Zealand Mice. J Exp Med (1974) 140:1011–27. 10.1084/jem.140.4.1011 PMC2139629 4279268

[18] Blomberg J Nived O Pipkorn R Bengtsson A Erlinge D Sturfelt G. Increased Antiretroviral Antibody Reactivity in Sera From a Defined Population of Patients With Systemic Lupus Erythematosus. Correlation With Autoantibodies and Clinical Manifestations. Arthritis Rheumatol (1994) 37:57–66. 10.1002/art.1780370109 7510483

[19] Suntsova M Garazha A Ivanova A Kaminsky D Zhavoronkov A Buzdin A. Molecular Functions of Human Endogenous Retroviruses in Health and Disease. Cell Mol Life Sci (2015) 72:3653–75. 10.1007/s00018-015-1947-6 PMC11113533 26082181

[20] Banki K Maceda J Hurley E Ablonczy E Mattson DH Szegedy L. Human T-cell Lymphotropic Virus (HTLV)-Related Endogenous Sequence, HRES-1, Encodes a 28-kDa Protein: A Possible Autoantigen for HTLV-I Gag-Reactive Autoantibodies. Proc Natl Acad Sci U S A (1992) 89:1939–43. 10.1073/pnas.89.5.1939 PMC48569 1347429

[21] Brookes SM Pandolfino YA Mitchell TJ Venables PJ Shattles WG Clark DA. The Immune Response to and Expression of Cross-Reactive Retroviral Gag Sequences in Autoimmune Disease. Br J Rheumatol (1992) 31:735–42. 10.1093/rheumatology/31.11.735 1280512

[22] Magistrelli C Samoilova E Agarwal RK Banki K Ferrante P Vladutiu A. Polymorphic Genotypes of the HRES-1 Human Endogenous Retrovirus Locus Correlate With Systemic Lupus Erythematosus and Autoreactivity. Immunogenetics (1999) 49:829–34. 10.1007/s002510050561 10436175

[23] Okada M Ogasawara H Kaneko H Hishikawa T Sekigawa I Hashimoto H. Role of DNA Methylation in Transcription of Human Endogenous Retrovirus in the Pathogenesis of Systemic Lupus Erythematosus. J Rheumatol (2002) 29:1678–82. 12180729

[24] Ogasawara H Naito T Kaneko H Hishikawa T Sekigawa I Hashimoto H. Quantitative Analyses of Messenger RNA of Human Endogenous Retrovirus in Patients With Systemic Lupus Erythematosus. J Rheumatol (2001) 28:533–8. 11296954

[25] Lanciano S Cristofari G. Measuring and Interpreting Transposable Element Expression. Nat Rev Genet (2020) 21:721–36. 10.1038/s41576-020-0251-y 32576954

[26] Bendall ML de Mulder M Iñiguez LP Lecanda-Sánchez A Pérez-Losada M Ostrowski MA. Telescope: Characterization of the Retrotranscriptome by Accurate Estimation of Transposable Element Expression. PloS Comput Biol (2019) 15:e1006453. 10.1371/journal.pcbi.1006453 31568525 PMC6786656

[27] Hung T Pratt GA Sundararaman B Townsend MJ Chaivorapol C Bhangale T. The Ro60 Autoantigen Binds Endogenous Retroelements and Regulates Inflammatory Gene Expression. Science (2015) 350:455–9. 10.1126/science.aac7442 PMC4691329 26382853

[28] Francis OE Bendall M Manimaran S Hong C Clement NL Castro-Nallar E. Pathoscope: Species Identification and Strain Attribution With Unassembled Sequencing Data. Genome Res (2013) 23:1721–9. 10.1101/gr.150151.112 PMC3787268 23843222

[29] Dodt M Roehr JT Ahmed R Dieterich C. Flexbar-Flexible Barcode and Adapter Processing for Next-Generation Sequencing Platforms. Biology (2012) 1:895–905. 10.3390/biology1030895 24832523 PMC4009805

[30] Langmead B Salzberg SL. Fast Gapped-Read Alignment With Bowtie 2. Nat Methods (2012) 9:357–9. 10.1038/nmeth.1923 PMC3322381 22388286

[31] Guo J-C Fang S-S Wu Y Zhang J-H Chen Y Liu J. CNIT: A Fast and Accurate Web Tool for Identifying Protein-Coding and Long non-Coding Transcripts Based on Intrinsic Sequence Composition. Nucleic Acids Res (2019) 47:W516–22. 10.1093/nar/gkz400 PMC6602462 31147700

[32] Lizio M Harshbarger J Shimoji H Severin J Kasukawa T Sahin S. Gateways to the FANTOM5 Promoter Level Mammalian Expression Atlas. Genome Biol (2015) 16:22. 10.1186/s13059-014-0560-6 25723102 PMC4310165

[33] Lizio M Abugessaisa I Noguchi S Kondo A Hasegawa A Hon CC. Update of the FANTOM Web Resource: Expansion to Provide Additional Transcriptome Atlases. Nucleic Acids Res (2019) 47:D752–8. 10.1093/nar/gky1099 PMC6323950 30407557

[34] Medstrand P van de Lagemaat LN Mager DL. Retroelement Distributions in the Human Genome: Variations Associated With Age and Proximity to Genes. Genome Res (2002) 12:1483–95. 10.1101/gr.388902 PMC187529 12368240

[35] Andrews S. Others. FastQC: A Quality Control Tool for High Throughput Sequence Data. (2010). Available at: https://www.bioinformatics.babraham.ac.uk/projects/fastqc/.

[36] Ewels P Magnusson M Lundin S Käller M. MultiQC: Summarize Analysis Results for Multiple Tools and Samples in a Single Report. Bioinformatics (2016) 32:3047–8. 10.1093/bioinformatics/btw354 PMC5039924 27312411

[37] Bolger AM Lohse M Usadel B. Trimmomatic: A Flexible Trimmer for Illumina Sequence Data. Bioinformatics (2014) 30:2114–20. 10.1093/bioinformatics/btu170 PMC4103590 24695404

[38] Dobin A Davis CA Schlesinger F Drenkow J Zaleski C Jha S. STAR: Ultrafast Universal RNA-seq Aligner. Bioinformatics (2013) 29:15–21. 10.1093/bioinformatics/bts635 23104886 PMC3530905

[39] Li H Handsaker B Wysoker A Fennell T Ruan J Homer N. The Sequence Alignment/Map Format and Samtools. Bioinformatics (2009) 25:2078–9. 10.1093/bioinformatics/btp352 PMC2723002 19505943

[40] Liao Y Smyth GK Shi W. featureCounts: An Efficient General Purpose Program for Assigning Sequence Reads to Genomic Features. Bioinformatics (2014) 30:923–30. 10.1093/bioinformatics/btt656 24227677

[41] Love MI Huber W Anders S. Moderated Estimation of Fold Change and Dispersion for RNA-seq Data With Deseq2. Genome Biol (2014) 15:550. 10.1186/s13059-014-0550-8 25516281 PMC4302049

[42] Wickham H. Ggplot2. WIREs Comp Stat (2011) 3:180–5. 10.1002/wics.147

[43] Rau A Gallopin M Celeux G Jaffrézic F. Htsfilter: Independent Data-Based Filtering for Replicated Transcriptome Sequencing Experiments (2013). Available at: http://citeseerx.ist.psu.edu/viewdoc/download?doi=10.1.1.592.1109&rep=rep1&type=pdf. 10.1093/bioinformatics/btt350 PMC3740625 23821648

[44] Rau A Gallopin M Celeux G Jaffrézic F. HTSFilter: Data-based filtering for replicated transcriptome. *bioconductor.statistik.tu-dortmund.de* . Available at: http://bioconductor.statistik.tu-dortmund.de/packages/3.1/bioc/vignettes/HTSFilter/inst/doc/HTSFilter.pdf. 10.1093/bioinformatics/btt350 PMC3740625 23821648

[45] Tibshirani R. Regression Shrinkage and Selection Via the Lasso. J R Stat Soc Ser B Stat Methodol (1996) 58:267–88. 10.1111/j.2517-6161.1996.tb02080.x

[46] Durinck S Spellman PT Birney E Huber W. Mapping Identifiers for the Integration of Genomic Datasets With the R/Bioconductor Package Biomart. Nat Protoc (2009) 4:1184–91. 10.1038/nprot.2009.97 PMC3159387 19617889

[47] Sorokin M Ignatev K Barbara V Vladimirova U Muraveva A Suntsova M. Molecular Pathway Activation Markers are Associated With Efficacy of Trastuzumab Therapy in Metastatic Her2-Positive Breast Cancer Better Than Individual Gene Expression Levels. Biochemistry (2020) 85:758–72. 10.1134/S0006297920070044 33040720

[48] Mi H Thomas P. PANTHER Pathway: An Ontology-Based Pathway Database Coupled With Data Analysis Tools. Methods Mol Biol (2009) 563:123–40. 10.1007/978-1-60761-175-2_7 PMC6608593 19597783

[49] Mallick H Rahnavard A McIver LJ Ma S Zhang Y Nguyen LH. ­Multivariable Association Discovery in Population-Scale Meta-Omics Studies. bioRxiv (2021) 2021.01.20.427420. 10.1101/2021.01.20.427420 PMC8714082 34784344

[50] Rahnavard A Mallick H. Deepath: Generic Omics Pathway Enrichment Analysis. R package version 1.1.1 (2020). Available at: https://github.com/omicsEye/deepath.

[51] Mallick H Rahnavard A McIver L. MaAsLin 2: Multivariable Association Discovery in Population-scale Meta-Omics Studies. Available at: http://huttenhower.sph.harvard.edu/maaslin2. 10.1371/journal.pcbi.1009442 PMC8714082 34784344

[52] Liberzon A Subramanian A Pinchback R Thorvaldsdóttir H Tamayo P Mesirov JP. Molecular Signatures Database (MsigDB) 3.0. Bioinformatics (2011) 27:1739–40. 10.1093/bioinformatics/btr260 PMC3106198 21546393

[53] Benjamini Y Hochberg Y. Controlling the False Discovery Rate: A Practical and Powerful Approach to Multiple Testing. J R Stat Soc Ser B Stat Methodol (1995) 57:289–300. 10.1111/j.2517-6161.1995.tb02031.x

[54] Rusinova I Forster S Yu S Kannan A Masse M Cumming H. Interferome v2.0: An Updated Database of Annotated Interferon-Regulated Genes. Nucleic Acids Res (2013) 41:D1040–6. 10.1093/nar/gks1215 PMC3531205 23203888

[55] Catalina MD Bachali P Geraci NS Grammer AC Lipsky PE. Gene Expression Analysis Delineates the Potential Roles of Multiple Type 1 Interferons in Systemic Lupus Erythematosus. Commun Biol (2019) 2:140. 10.1038/s42003-019-0382-x 31044165 PMC6478921

[56] Oliveira JJ Karrar S Rainbow DB Pinder CL Clarke P Rubio García A. The Plasma Biomarker Soluble SIGLEC-1 is Associated With the Type I Interferon Transcriptional Signature, Ethnic Background and Renal Disease in Systemic Lupus Erythematosus. Arthritis Res Ther (2018) 20:152. 10.1186/s13075-018-1649-1 30053827 PMC6062988

[57] Su P Wang F Qi B Wang T Zhang S. P53 Regulation-Association Long Non-Coding RNA (Lncrna PRAL) Inhibits Cell Proliferation by Regulation of P53 in Human Lung Cancer. Med Sci Monit (2017) 23:1751–8. 10.12659/MSM.900205 PMC5398470 28396580

[58] Perl A Fernandez D Telarico T Phillips PE. Endogenous Retroviral Pathogenesis in Lupus. Curr Opin Rheumatol (2010) 22:483–92. 10.1097/BOR.0b013e32833c6297 PMC5314925 20644481

[59] Tokuyama M Kong Y Song E Jayewickreme T Kang I Iwasaki A. Ervmap Analysis Reveals Genome-Wide Transcription of Human Endogenous Retroviruses. Proc Natl Acad Sci U S A (2018) 115:12565–72. 10.1073/pnas.1814589115 PMC6294949 30455304

[60] Iñiguez LP de Mulder Rougvie M Stearrett N Jones RB Ormsby CE Reyes-Terán G. Transcriptomic Analysis of Human Endogenous Retroviruses in Systemic Lupus Erythematosus. Proc Natl Acad Sci U S A (2019) 116:21350–1. 10.1073/pnas.1907705116 PMC6815141 31594853

[61] Kegerreis B Catalina MD Bachali P Geraci NS Labonte AC Zeng C. Machine Learning Approaches to Predict Lupus Disease Activity From Gene Expression Data. Sci Rep (2019) 9:9617. 10.1038/s41598-019-45989-0 31270349 PMC6610624

[62] Angermueller C Pärnamaa T Parts L Stegle O. Deep Learning for Computational Biology. Mol Syst Biol (2016) 12:878. 10.1371/journal.pcbi.1006484 27474269 PMC4965871

[63] Chen L Fish AE Capra JA. Prediction of gene regulatory enhancers across species reveals evolutionarily conserved sequence properties. PLoS Comput Biol (2018) 14:e1006484. 10.1371/journal.pcbi.1006484 30286077 PMC6191148

[64] Quang D Xie X. FactorNet: A Deep Learning Framework for Predicting Cell Type Specific Transcription Factor Binding From Nucleotide-Resolution Sequential Data. Methods (2019) 166:40–7. 10.1016/j.ymeth.2019.03.020 PMC6708499 30922998

[65] Tampuu A Bzhalava Z Dillner J Vicente R. Viraminer: Deep Learning on Raw DNA Sequences for Identifying Viral Genomes in Human Samples. PloS One (2019) 14:e0222271. 10.1371/journal.pone.0222271 31509583 PMC6738585

[66] Aoki G Sakakibara Y. Convolutional Neural Networks for Classification of Alignments of non-Coding RNA Sequences. Bioinformatics (2018) 34:i237–44. 10.1093/bioinformatics/bty228 PMC6022636 29949978

[67] Busia A Dahl GE Fannjiang C Alexander DH Dorfman E Poplin R. A Deep Learning Approach to Pattern Recognition for Short DNA Sequences. bioRxiv (2019). 10.1101/353474

[68] Kolbe AR Bendall ML Pearson AT Paul D Nixon DF Pérez-Losada M. Human Endogenous Retrovirus Expression is Associated With Head and Neck Cancer and Differential Survival. Viruses (2020) 12:956. 10.3390/v12090956 PMC7552064 32872377

[69] Kowalczyk MS Hughes JR Garrick D Lynch MD Sharpe JA Sloane-Stanley JA. Intragenic Enhancers Act as Alternative Promoters. Mol Cell (2012) 45:447–58. 10.1016/j.molcel.2011.12.021 22264824

[70] Azébi S Batsché E Michel F Kornobis E Muchardt C. Expression of Endogenous Retroviruses Reflects Increased Usage of Atypical Enhancers in T Cells. EMBO J (2019) 38(12):e101107. 10.15252/embj.2018101107 31068361 PMC6576159

[71] Ng KW Attig J Bolland W Young GR Major J Wrobel AG. Tissue-Specific and Interferon-Inducible Expression of Nonfunctional ACE2 Through Endogenous Retroelement Co-Option. Nat Genet (2020) 52:1294–302. 10.1038/s41588-020-00732-8 PMC7610354 33077915

[72] Chuong EB Elde NC Feschotte C. Regulatory Evolution of Innate Immunity Through Co-Option of Endogenous Retroviruses. Science (2016) 351:1083–7. 10.1126/science.aad5497 PMC4887275 26941318

