# Supplementary material for: Expression of Human Endogenous Retroviruses in Systemic Lupus Erythematosus: Multiomic Integration With Gene Expression
Source: Front Immunol. 2021 Apr 27;12:661437. doi: 10.3389/fimmu.2021.661437 (PMC8112243; doi:10.3389/fimmu.2021.661437)
Supplement: Supplementary file 1 [file DataSheet_1.pdf]

Supplementary Materials Index:

Supplementary Figure 1. Correlation analysis of expressed HERVs (1a) and differentially expressed HERVs (1b) showing a much higher correlation coefficient for the DE HERVs (1a Pearson Correlation Coefficient = 0.063; 1b Pearson Correlation Coefficient = 0.602.

Supplementary Figure 2. Box plot for HERV distance to nearest gene for DE HERVs versus all HERVs. This figure shows DE HERVs are significantly (p = 1.107e-07) closer to their nearest genes than non-DE HERVs.

Supplementary Table 1 - DE HERVs for WB1 from DESeq2 results

Supplementary Table 2 - DE HERVs for WB2 from DESeq2 results

Supplementary Table 3 - HERVs which were DE in WB1/WB2 from DESeq2 results

Supplementary Table 4 - DE genes for WB1 from DESeq2 results

Supplementary Table 5 - DE genes for WB2 from DESeq2 results

Supplementary Table 6 - Maaslin2 association testing of HERV and gene expression in WB1
Please request this file from corresponding author Keith Crandall kcrandall@gwu.edu as this file is too large to upload into the editorial system.

Supplementary Table 7 - Maaslin2 association testing of HERV and gene expression in WB2
Please request this file from corresponding author Keith Crandall kcrandall@gwu.edu as this file is too large to upload into the editorial system.

Supplementary Table 8 - *deepath* pathway enrichment results for WB1

Supplementary Table 9 - *deepath* pathway enrichment results for WB2

Note original file formats of all supplemental materials (jpeg for figures, .csv and .tsv for tables) are available from corresponding author Keith Crandall kcrandall@gwu.edu.

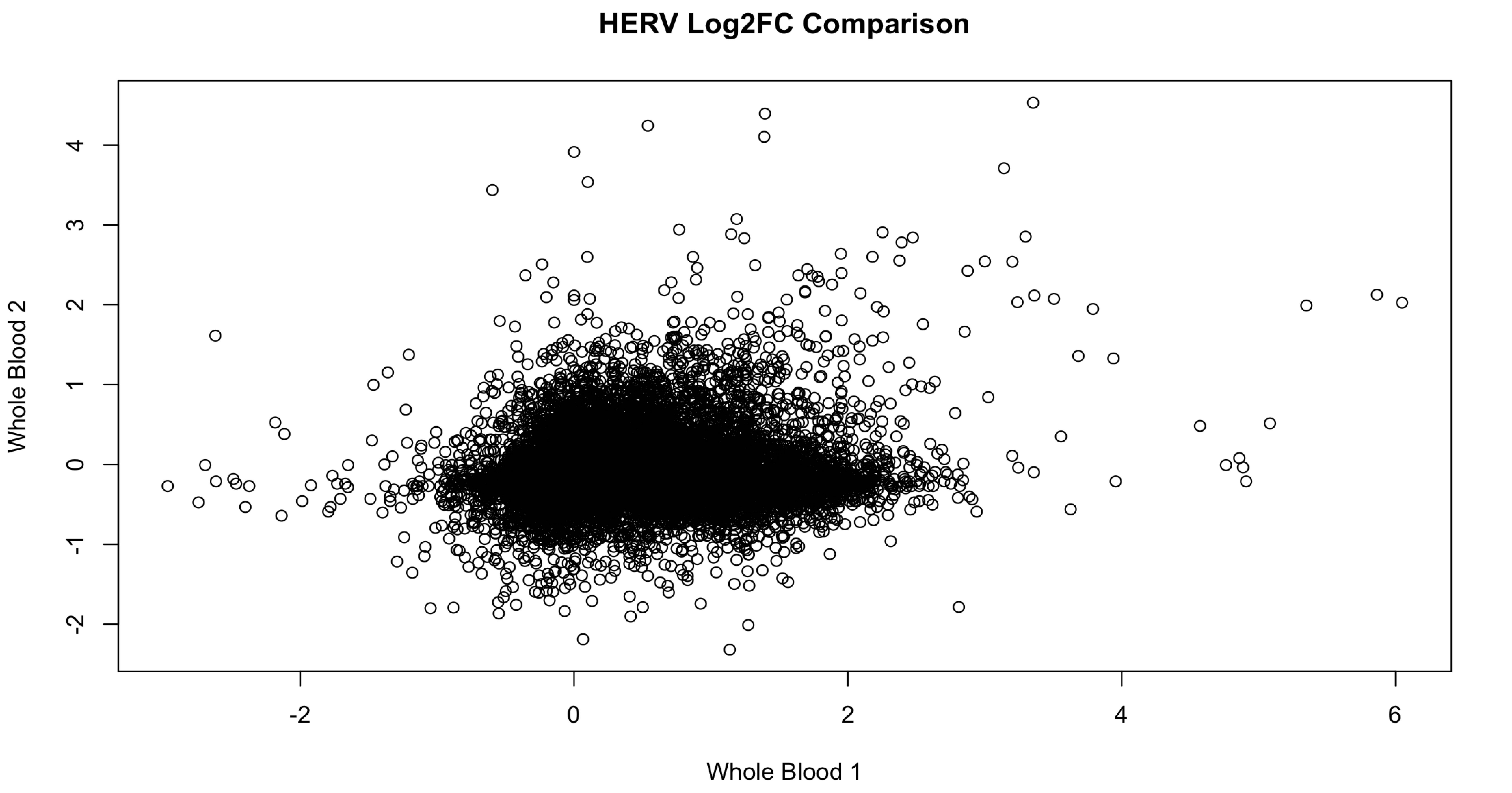
HERV Log2FC Comparison
Whole Blood 2
Whole Blood 1
-2
0
2
4
6
-2
-1
0
1
2
3
4

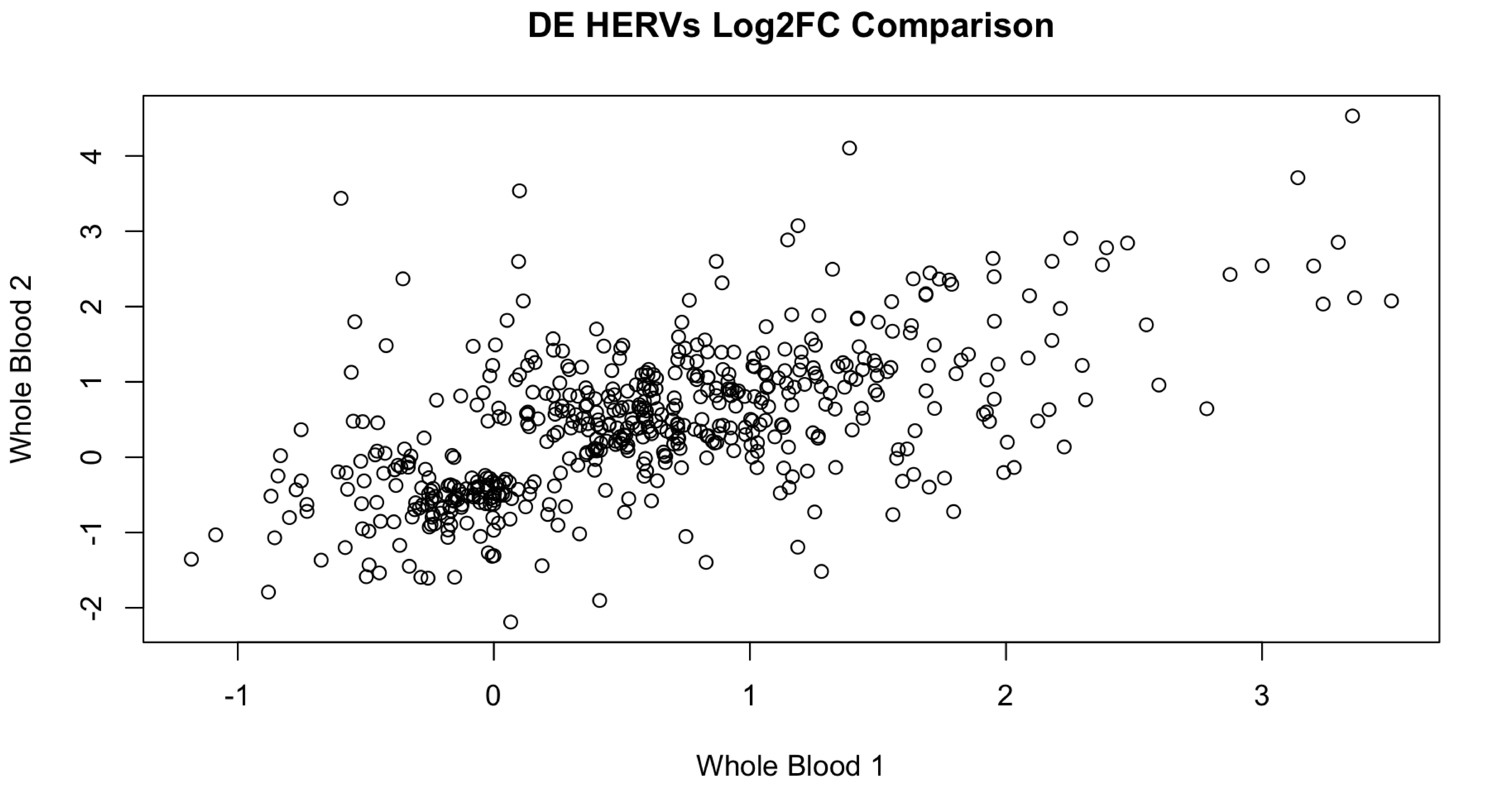
DE HERVs Log2FC Comparison
Whole Blood 2
Whole Blood 1
-2
-1
0
1
2
3
4
-1
0
1
2
3

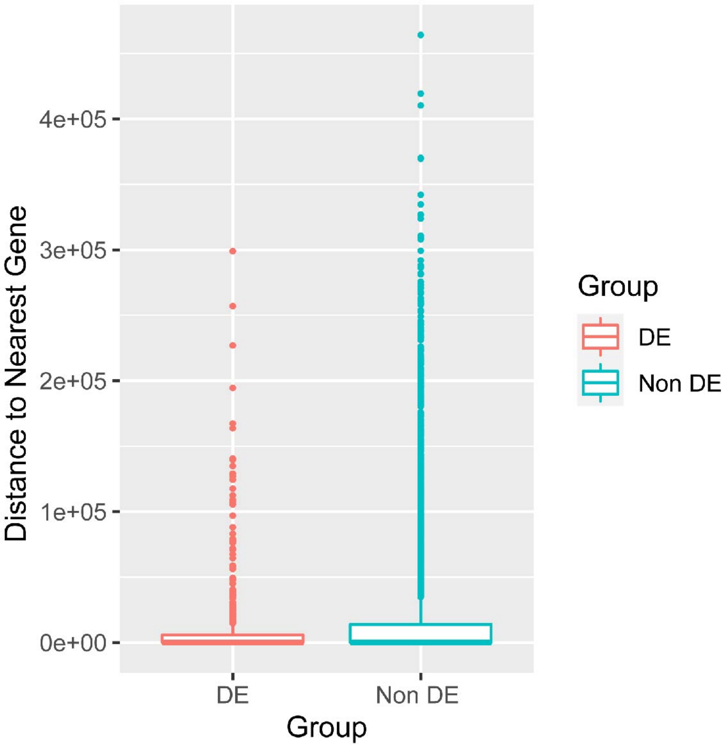

Distance to Nearest Gene
4e+05
3e+05
2e+05
1e+05
0e+00
DE
Non DE
Group
Group
DE
Non DE

Locus	log2FoldChar	padj	Class	Family	Category	Chrom
MER4_1q25.	4.57169891	1.46E-06	HERV	MER4	oneside	chr1
HML2_1p36.:	2.81815524	0.00904212	HERV	HML2	prototype	chr1
HERVL_1p21	2.38252652	0.02809185	HERV	HERVL	prototype	chr1
HERVP71A_1	2.37615982	3.31E-10	HERV	HERVP71A	prototype	chr1
ERV316A3_1	2.32338796	0.02935235	HERV	ERV316A3	internal	chr1
MER101_1p2	2.21224056	2.34E-06	HERV	MER101	oneside	chr1
HERVL_1p34	2.18128697	0.00787979	HERV	HERVL	prototype	chr1
HERVH_1p32	2.17983291	0.01090785	HERV	HERVH	prototype	chr1
HERVFH19_1	2.17938889	3.01E-05	HERV	HERVFH19	oneside	chr1
HERVIP10FH_	2.17858564	0.00072308	HERV	HERVIP10FH	oneside	chr1
PRIMA41_1q	2.15783082	0.02523547	HERV	PRIMA41	prototype	chr1
HERVFH21_1	2.00513702	2.27E-06	HERV	HERVFH21	prototype	chr1
HERVFH19_1	1.95325129	0.03295392	HERV	HERVFH19	oneside	chr1
HERVH_1q42	1.9255073	0.04496323	HERV	HERVH	prototype	chr1
MER4_1p34.	1.75213418	0.03280551	HERV	MER4	insert*	chr1
HARLEQUIN_	1.72691581	0.02522199	HERV	HARLEQUIN	internal	chr1
HERVH_1p34	1.69411175	0.01909716	HERV	HERVH	prototype	chr1
ERV316A3_1	1.68704038	0.00045675	HERV	ERV316A3	oneside	chr1
HERVL_1p22	1.67099879	0.03644493	HERV	HERVL	prototype	chr1
ERVLE_1q32.	1.55123658	0.00345305	HERV	ERVLE	internal	chr1
HERVH_1p34	1.50728862	0.01330278	HERV	HERVH	oneside	chr1
ERV316A3_1	1.49493187	0.00014606	HERV	ERV316A3	internal	chr1
ERV316A3_1	1.45585721	0.00018031	HERV	ERV316A3	oneside	chr1
HERVH_1p36	1.43628694	0.04860063	HERV	HERVH	oneside	chr1
MER101_1p2	1.43508197	1.06E-09	HERV	MER101	oneside	chr1
HERVL_1p31	1.41838967	0.00032792	HERV	HERVL	oneside	chr1
ERVLE_1q25.	1.2349026	0.00281343	HERV	ERVLE	internal	chr1
LTR19_1q24.	1.23050278	0.04443117	HERV	LTR19	internal	chr1
LTR25_1p22.	1.19383284	0.0024989	HERV	LTR25	internal	chr1
HERVH_1q42	1.1731602	0.00136129	HERV	HERVH	prototype	chr1
HML5_1q25.:	1.16861778	0.00345305	HERV	HML5	prototype	chr1
HML1_1p31.:	1.1660238	0.03505741	HERV	HML1	prototype	chr1
HERVL_1p22	1.16252199	0.00144275	HERV	HERVL	prototype	chr1
HUERSP3_1c	1.15309	0.00241316	HERV	HUERSP3	oneside	chr1
MER4_1q44k	1.1261769	0.04623809	HERV	MER4	internal	chr1
HUERSP1_1c	1.11568415	0.01142562	HERV	HUERSP1	prototype	chr1
MER34B_1q:	1.06053446	0.00308393	HERV	MER34B	internal	chr1
MER61_1q2:	1.04908767	8.08E-06	HERV	MER61	prototype	chr1
HERVIP10FH_	1.01176488	7.19E-06	HERV	HERVIP10FH	oneside	chr1
HERVK11D_1	-1.4213137	0.02916949	HERV	HERVK11D	oneside	chr1
HERVP71A_1	2.26088483	9.62E-05	HERV	HERVP71A	prototype	chr10
ERV316A3_1	2.13716489	0.0497064	HERV	ERV316A3	oneside	chr10

HERV9_10p1	1.85907372	0.03280551	HERV	HERV9	prototype	chr10
HERVH_10p1	1.7097201	0.03351005	HERV	HERVH	prototype	chr10
ERVL_10q21.	1.63978402	0.02931788	HERV	ERVL	oneside	chr10
HERVH_10p1	1.45889771	0.03846727	HERV	HERVH	prototype	chr10
HERVL_11q1	3.29731786	1.67E-06	HERV	HERVL	oneside	chr11
ERV316A3_1	2.85341483	0.04777409	HERV	ERV316A3	internal	chr11
HERVI_11p1!	2.53519928	0.00191435	HERV	HERVI	oneside	chr11
ERVLE_11q1:	2.22691018	0.03152544	HERV	ERVLE	internal	chr11
HERVH48_11	2.20140283	0.02441377	HERV	HERVH48	oneside	chr11
ERV316A3_1	1.95376458	0.0006388	HERV	ERV316A3	oneside	chr11
HUERSP1_11	1.79416542	0.00107642	HERV	HUERSP1	oneside	chr11
HERVH_11q1	1.74034318	0.04045318	HERV	HERVH	prototype	chr11
HUERSP2_11	1.70301073	0.00375046	HERV	HUERSP2	prototype	chr11
MER4_11q21	1.63801976	0.00632705	HERV	MER4	oneside	chr11
MER34B_11ǀ	1.63069199	0.00171918	HERV	MER34B	unknown	chr11
HERVH_11q2	1.52304339	0.04777409	HERV	HERVH	oneside	chr11
HML1_11q12	1.41382016	0.00256069	HERV	HML1	prototype	chr11
ERV316A3_1	1.11201541	0.00350299	HERV	ERV316A3	internal	chr11
HERVE_11q1	-1.0862824	0.00554386	HERV	HERVE	oneside	chr11
HERV9_11p1	-1.1701948	0.0307264	HERV	HERV9	prototype	chr11
ERV316A3_1	3.35296705	8.53E-11	HERV	ERV316A3	internal	chr12
HERVH_12p1	2.40876394	0.02223019	HERV	HERVH	prototype	chr12
HERVH48_12	2.29246068	0.00355878	HERV	HERVH48	prototype	chr12
HUERSP1_12	1.92347945	9.57E-06	HERV	HUERSP1	prototype	chr12
HML5_12p13	1.82466553	0.00041625	HERV	HML5	internal	chr12
ERVLB4_12q	1.78224158	0.01915726	HERV	ERVLB4	prototype	chr12
MER4_12q12	1.71972247	3.10E-08	HERV	MER4	oneside	chr12
HARLEQUIN_	1.71718616	0.04777409	HERV	HARLEQUIN	prototype	chr12
HERVW_12q	1.66521506	0.0083973	HERV	HERVW	prototype	chr12
ERVLB4_12q	1.62619379	0.00281343	HERV	ERVLB4	internal	chr12
HERVH_12q2	1.57289441	0.01671178	HERV	HERVH	prototype	chr12
ERV316A3_1	1.51090535	0.0156061	HERV	ERV316A3	internal	chr12
HERVW_12q	1.49724253	0.0497064	HERV	HERVW	oneside	chr12
MER4_12q12	1.44746644	1.04E-06	HERV	MER4	oneside	chr12
HERVH_12q2	1.41717299	0.02078942	HERV	HERVH	oneside	chr12
MER4_12q12	1.36356449	1.00E-05	HERV	MER4	internal	chr12
MER4_12q12	1.34369659	3.36E-07	HERV	MER4	prototype	chr12
HERVL_12p1	1.33511926	0.04376255	HERV	HERVL	prototype	chr12
HERVL_12p1	1.27350797	0.00023937	HERV	HERVL	prototype	chr12
MER4_12q12	1.25523382	4.06E-05	HERV	MER4	oneside	chr12
HERV4_12p1	1.25394691	8.53E-05	HERV	HERV4	oneside	chr12
HERVFH19_1	1.13638805	0.00375046	HERV	HERVFH19	oneside	chr12
HERVL_13q3	2.31102196	0.00806956	HERV	HERVL	prototype	chr13

LTR25_13q3:	2.26350335	0.04777409	HERV	LTR25	internal	chr13
HERVH_13q3	2.16811288	0.02485986	HERV	HERVH	prototype	chr13
ERVLE_13q3:	2.12405098	0.03189626	HERV	ERVLE	internal	chr13
HERVH_14q2	3.35800789	0.00182298	HERV	HERVH	prototype	chr14
HERVL18_14	3.24480701	0.00366065	HERV	HERVL18	oneside	chr14
ERV316A3_1	2.29272143	0.03721589	HERV	ERV316A3	oneside	chr14
ERV316A3_1	2.10601497	0.04631837	HERV	ERV316A3	oneside	chr14
HERVL40_14	2.06602357	0.02761071	HERV	HERVL40	prototype	chr14
HML3_14q21	1.88144438	0.04700409	HERV	HML3	prototype	chr14
HERVIP10F_:	1.80461898	0.00067803	HERV	HERVIP10F	tandem*	chr14
ERVLE_14q3:	1.36245665	0.03721589	HERV	ERVLE	internal	chr14
HML6_14q24	1.26591131	0.04295387	HERV	HML6	prototype	chr14
ERVLE_14q3	1.05597101	0.02217887	HERV	ERVLE	internal	chr14
HERVW_15q	2.63447848	0.00199685	HERV	HERVW	internal	chr15
ERV316A3_1	1.44584428	0.04777409	HERV	ERV316A3	oneside	chr15
HERVL_15q2	1.01594251	0.02078942	HERV	HERVL	oneside	chr15
HERVH_16q2	2.27803311	0.03424113	HERV	HERVH	prototype	chr16
MER4B_16q:	2.02299059	0.00418508	HERV	MER4B	prototype	chr16
HERVL_16q2	1.01869542	0.00848757	HERV	HERVL	prototype	chr16
MER4_17q21	2.72768665	0.00366065	HERV	MER4	oneside	chr17
HERV9_17q1	2.653927	0.00082686	HERV	HERV9	prototype	chr17
ERVLE_17q2	2.22709972	0.00026341	HERV	ERVLE	internal	chr17
ERV316A3_1	1.97620701	0.04175373	HERV	ERV316A3	oneside	chr17
HERVK11_17	1.8040665	0.01372398	HERV	HERVK11	prototype	chr17
HML2_17p1:	1.73107539	0.03822638	HERV	HML2	oneside	chr17
HARLEQUIN_	1.41947202	0.00048107	HERV	HARLEQUIN	oneside	chr17
ERVLB4_17q	1.24052682	8.45E-09	HERV	ERVLB4	internal	chr17
HERVFRD_1{	2.80268466	9.69E-10	HERV	HERVFRD	oneside	chr18
ERVLB4_18p	2.55791863	0.02498203	HERV	ERVLB4	oneside	chr18
MER61_18q1	2.33877681	0.00012772	HERV	MER61	prototype	chr18
HUERSP3B_1	1.9030691	0.01733322	HERV	HUERSP3B	oneside	chr18
ERV316A3_1	1.80202471	0.01441429	HERV	ERV316A3	internal	chr18
HERVH_18q2	1.38386194	0.04111645	HERV	HERVH	prototype	chr18
ERV316A3_1	1.09703604	6.91E-05	HERV	ERV316A3	oneside	chr18
HUERSP3B_:	2.75228543	0.03721589	HERV	HUERSP3B	oneside	chr19
ERVLE_19q1:	2.5969329	0.01034617	HERV	ERVLE	internal	chr19
HERVH_19p1	1.91598607	0.0065639	HERV	HERVH	prototype	chr19
HERVH_19p1	1.77166557	0.02321446	HERV	HERVH	prototype	chr19
LTR57_19p1:	1.64455911	0.03680578	HERV	LTR57	internal	chr19
HML3_19p12	1.55468451	0.04606108	HERV	HML3	oneside	chr19
MER4_19p1:	1.4421347	0.00366065	HERV	MER4	internal	chr19
HERVH_19q1	1.42594464	0.00029445	HERV	HERVH	prototype	chr19
HERVS71_19	1.2983836	0.04454666	HERV	HERVS71	prototype	chr19

HERVH_19q1	1.24594621	0.02154823	HERV	HERVH	prototype	chr19
ERVLE_19q1	1.00905062	0.00397702	HERV	ERVLE	internal	chr19
HERVH_19p1	1.00237964	0.01041612	HERV	HERVH	oneside	chr19
HUERSP3_2p	3.13965477	2.82E-10	HERV	HUERSP3	internal	chr2
HERVH_2p24	2.42045099	0.03442744	HERV	HERVH	prototype	chr2
MER101_2p2	2.3926546	4.79E-11	HERV	MER101	oneside	chr2
HERVH_2q14	2.14949682	0.03241515	HERV	HERVH	prototype	chr2
HERVH_2p13	2.0318118	0.00505051	HERV	HERVH	prototype	chr2
LTR25_2q12.	1.95445178	1.31E-06	HERV	LTR25	internal	chr2
MER101_2q3	1.95390548	7.84E-05	HERV	MER101	internal	chr2
HERVFH19_2	1.94899884	2.21E-10	HERV	HERVFH19	oneside	chr2
HERVIP10FH	1.92571123	0.00011677	HERV	HERVIP10FH	oneside	chr2
HERVH_2p12	1.81537277	0.03900716	HERV	HERVH	prototype	chr2
HUERSP2_2c	1.77805291	9.44E-11	HERV	HUERSP2	prototype	chr2
HML6_2q21.	1.72087343	0.00107489	HERV	HML6	prototype	chr2
HERVL_2q37	1.65442402	0.04543828	HERV	HERVL	prototype	chr2
PABLA_2p25	1.49193449	0.00030445	HERV	PABLA	internal	chr2
MER34B_2q3	1.48892473	6.17E-05	HERV	MER34B	internal	chr2
ERVLE_2p16.	1.42662343	0.00147721	HERV	ERVLE	internal	chr2
ERV316A3_2	1.33466746	0.00593983	HERV	ERV316A3	prototype	chr2
MER41_2p16	1.33204689	0.00517923	HERV	MER41	oneside	chr2
MER41_2p16	1.26258402	0.02747276	HERV	MER41	oneside	chr2
HERV9_2q35	1.15163712	0.00063679	HERV	HERV9	oneside	chr2
HERVL_2q14	1.1323017	0.00318042	HERV	HERVL	prototype	chr2
HERV9_2q35	1.11157294	0.00171918	HERV	HERV9	oneside	chr2
HML3_2q35	1.07293369	0.00146401	HERV	HML3	prototype	chr2
HERVH_2q33	1.02920725	0.00281895	HERV	HERVH	prototype	chr2
HERVH_2p14	1.02881592	0.00146313	HERV	HERVH	prototype	chr2
HERVH_20p1	1.82259427	0.02588858	HERV	HERVH	prototype	chr20
HERVIP10F_	1.68759329	0.04020337	HERV	HERVIP10F	prototype	chr20
ERV316A3_2	1.49828232	0.00185258	HERV	ERV316A3	internal	chr20
HERVH_20q1	1.13197435	0.00060611	HERV	HERVH	prototype	chr20
HERVL40_20	1.11839476	0.01368116	HERV	HERVL40	oneside	chr20
MER101_20c	1.04290896	0.04002832	HERV	MER101	internal	chr20
MER4B_21q2	2.20611585	0.01117355	HERV	MER4B	unknown	chr21
ERV316A3_2	1.50126729	0.00101075	HERV	ERV316A3	oneside	chr21
HERV3_21q2	1.25965875	0.02139448	HERV	HERV3	prototype	chr21
MER4_22q12	1.31279602	4.79E-11	HERV	MER4	prototype	chr22
ERVLE_22q1	1.16216192	0.02159639	HERV	ERVLE	internal	chr22
MER41_22q1	1.03963076	5.52E-08	HERV	MER41	prototype	chr22
HERVL18_3p	3.36123798	1.90E-08	HERV	HERVL18	oneside	chr3
HERVL18_3p	3.23839538	1.56E-08	HERV	HERVL18	oneside	chr3
ERVLE_3p14.	2.95534019	0.01246461	HERV	ERVLE	internal	chr3

HML6_3p21.:	2.87509621	5.15E-07	HERV	HML6	prototype	chr3
HML2_3q27.:	2.78398232	0.01799654	HERV	HML2	prototype	chr3
ERVLB4_3p2	2.54815047	5.19E-07	HERV	ERVLB4	internal	chr3
ERV316A3_3	2.47437026	4.79E-11	HERV	ERV316A3	oneside	chr3
HERVH_3q26	2.34574798	0.03212641	HERV	HERVH	prototype	chr3
HERVL_3q26	2.21725556	0.00011066	HERV	HERVL	prototype	chr3
HERVEA_3q2	2.16135496	0.04492972	HERV	HERVEA	prototype	chr3
HERVH_3q24	1.91327671	0.04045318	HERV	HERVH	prototype	chr3
HERVH_3q22	1.8707933	0.0225048	HERV	HERVH	prototype	chr3
HERVH_3p12	1.86418676	0.03112323	HERV	HERVH	prototype	chr3
ERV316A3_3	1.85303044	3.49E-05	HERV	ERV316A3	oneside	chr3
HERVH_3q25	1.75961791	0.01425015	HERV	HERVH	prototype	chr3
HML3_3q13.:	1.69973204	0.0497064	HERV	HML3	oneside	chr3
HERVFRD_3p	1.63913089	0.00235402	HERV	HERVFRD	oneside	chr3
HERVH_3p21	1.59612699	0.00851523	HERV	HERVH	oneside	chr3
HERVH_3p22	1.55843308	0.02898305	HERV	HERVH	prototype	chr3
HML3_3q13.	1.50163089	0.00936279	HERV	HML3	prototype	chr3
HERVH_3p21	1.26918853	0.0006776	HERV	HERVH	prototype	chr3
HERVH_3p21	1.26742881	0.04976582	HERV	HERVH	oneside	chr3
MER61_3p21	1.2382238	0.00150815	HERV	MER61	prototype	chr3
HERVH_3p22	1.22438517	0.04907696	HERV	HERVH	prototype	chr3
MER101_3q2	1.14229306	0.00340185	HERV	MER101	internal	chr3
HERVL_3p26	1.06444508	0.01419199	HERV	HERVL	prototype	chr3
HERVH_4q22	2.6008915	0.01548828	HERV	HERVH	prototype	chr4
PRIMA41_4q	2.52853813	0.01127006	HERV	PRIMA41	tandem*	chr4
HML3_4q28.	2.48570798	0.0056419	HERV	HML3	prototype	chr4
HERVH_4q24	2.44951316	0.02018248	HERV	HERVH	prototype	chr4
HERVH_4q28	2.00462064	0.02931788	HERV	HERVH	prototype	chr4
HERVH_4q26	1.86553088	0.00994164	HERV	HERVH	oneside	chr4
HERVH_4q32	1.78337779	0.01126267	HERV	HERVH	oneside	chr4
HERVL40_4q	1.76902285	0.00033486	HERV	HERVL40	internal	chr4
HERVL_4p16	1.73854685	0.01498797	HERV	HERVL	oneside	chr4
HML3_4p12	1.71236406	0.00363162	HERV	HML3	prototype	chr4
HERV4_4q22	1.55339619	4.79E-11	HERV	HERV4	prototype	chr4
MER34B_4q3	1.55008367	3.02E-05	HERV	MER34B	internal	chr4
ERVLE_4q31	1.48933659	0.02921432	HERV	ERVLE	oneside	chr4
HML3_4q31.:	1.42153623	9.00E-05	HERV	HML3	prototype	chr4
ERVLB4_4p1	1.41289444	0.04095416	HERV	ERVLB4	opposite*	chr4
HERVK11_4q	1.27705943	0.02835377	HERV	HERVK11	prototype	chr4
ERVLB4_4q3	1.19954308	9.57E-06	HERV	ERVLB4	oneside	chr4
ERVLE_4p16.	1.02763064	0.01860602	HERV	ERVLE	internal	chr4
ERVL_4q26b	-1.3771094	0.02898305	HERV	ERVL	internal	chr4
ERVLE_4q26	-1.7064642	0.01081344	HERV	ERVLE	internal	chr4

HERVP71A_5	3.20023228	0.04777409	HERV	HERVP71A	oneside	chr5
HERVH_5q34	2.84280936	0.03278178	HERV	HERVH	prototype	chr5
PRIMA4_5p1	2.27532877	0.03680578	HERV	PRIMA4	internal	chr5
HERVH_5p15	1.77829376	0.02309086	HERV	HERVH	prototype	chr5
ERVLE_5q22.	1.68719244	0.00177051	HERV	ERVLE	internal	chr5
HERVL_5q33	1.43982137	0.0037587	HERV	HERVL	prototype	chr5
HERVIP10FH_	1.01200552	0.02334172	HERV	HERVIP10FH	internal	chr5
ERVLE_5q35.	1.0084893	0.04230053	HERV	ERVLE	internal	chr5
PABLA_6p12	3.50499617	2.37E-06	HERV	PABLA	internal	chr6
MER41_6p12	3.20135606	6.63E-05	HERV	MER41	oneside	chr6
HERVW_6q2	3.02534565	0.00417878	HERV	HERVW	internal	chr6
HERVH_6q11	2.94092662	0.02458082	HERV	HERVH	prototype	chr6
HERVH_6q22	2.8102844	0.04367305	HERV	HERVH	prototype	chr6
HERVW_6q2	2.70392913	0.00144275	HERV	HERVW	oneside	chr6
HML3_6p12.	2.68612735	0.00261067	HERV	HML3	prototype	chr6
HERVH_6p25	2.52151718	0.01226351	HERV	HERVH	prototype	chr6
HUERSP3B_6	2.45195994	0.04149784	HERV	HUERSP3B	prototype	chr6
HML3_6q21a	2.29757686	0.00036455	HERV	HML3	prototype	chr6
HERVH_6q12	2.20440341	0.0171637	HERV	HERVH	prototype	chr6
HERVH_6q27	2.17812141	0.04943289	HERV	HERVH	prototype	chr6
HERVL32_6p	2.08444993	0.0272011	HERV	HERVL32	oneside	chr6
MER41_6q21	2.0806821	0.00530692	HERV	MER41	oneside	chr6
HERVIP10FH_	2.0747184	0.03407969	HERV	HERVIP10FH	prototype	chr6
HERVH_6q22	2.031695	0.01766236	HERV	HERVH	prototype	chr6
HERVEA_6q2	2.02802749	0.00045675	HERV	HERVEA	prototype	chr6
HERVH_6q14	1.9908014	0.02982926	HERV	HERVH	prototype	chr6
HERVH_6p22	1.93525261	2.37E-06	HERV	HERVH	prototype	chr6
MER101_6q2	1.79531312	0.02989164	HERV	MER101	oneside	chr6
HML6_6p22.	1.69690645	6.10E-10	HERV	HML6	prototype	chr6
HERVH_6p22	1.60627232	0.02690271	HERV	HERVH	prototype	chr6
HML1_6q23.	1.36946297	7.31E-07	HERV	HML1	prototype	chr6
HML5_6p22.	1.2957634	4.52E-05	HERV	HML5	prototype	chr6
HERVH_6q22	1.25259273	0.02453345	HERV	HERVH	prototype	chr6
MER61_6q21	1.15058655	0.03351005	HERV	MER61	oneside	chr6
HERVP71A_6	1.13633788	0.00060152	HERV	HERVP71A	oneside	chr6
ERV316A3_6	1.04819499	0.0045507	HERV	ERV316A3	internal	chr6
ERV316A3_6	1.01539681	8.54E-06	HERV	ERV316A3	oneside	chr6
PRIMA41_7p	2.61740809	0.01445724	HERV	PRIMA41	oneside	chr7
PRIMA41_7p	2.45612702	0.00837749	HERV	PRIMA41	oneside	chr7
HERVL_7q36	2.09164104	0.00032792	HERV	HERVL	internal	chr7
ERVLE_7q11.	2.08609855	6.17E-05	HERV	ERVLE	oneside	chr7
HERVL_7p15	2.04873372	0.04544222	HERV	HERVL	oneside	chr7
HERVH_7q33	1.97506306	0.03688211	HERV	HERVH	oneside	chr7

HERVK11_7p	1.85370743	0.04668336	HERV	HERVK11	prototype	chr7
HML3_7p11.2	1.83149393	0.04161602	HERV	HML3	prototype	chr7
ERVLE_7q22.	1.73899718	0.00101953	HERV	ERVLE	internal	chr7
ERV316A3_7	1.61643193	0.0225048	HERV	ERV316A3	prototype	chr7
HERVE_7p22	1.61379749	0.00346458	HERV	HERVE	oneside	chr7
HML3_7q11.2	1.57988122	0.0006388	HERV	HML3	oneside	chr7
ERV316A3_7	1.55856632	0.04045318	HERV	ERV316A3	internal	chr7
HERVH_7q22	1.55670132	0.00259206	HERV	HERVH	prototype	chr7
HERV9_7q21	1.53605434	0.00894372	HERV	HERV9	oneside	chr7
PRIMA41_7q	1.49681361	0.00336313	HERV	PRIMA41	oneside	chr7
HERVH_7p14	1.39931408	0.00663415	HERV	HERVH	prototype	chr7
HERVFH21_7	1.39597216	0.03189626	HERV	HERVFH21	prototype	chr7
ERVLE_7q34	1.39355208	7.84E-05	HERV	ERVLE	oneside	chr7
MER4_7q21.	1.37591003	4.79E-11	HERV	MER4	oneside	chr7
HERV3_7q33	1.27872779	9.27E-06	HERV	HERV3	internal	chr7
HARLEQUIN_	1.24755061	1.41E-05	HERV	HARLEQUIN	oneside	chr7
MER4_7q22.	1.21353628	0.0036095	HERV	MER4	internal	chr7
ERV316A3_7	1.20218804	0.00725743	HERV	ERV316A3	oneside	chr7
MER41_7q22	1.06312621	0.01108746	HERV	MER41	prototype	chr7
HARLEQUIN_	1.01759945	0.03442744	HERV	HARLEQUIN	oneside	chr7
ERVLE_8q12.	3.00006779	1.41E-06	HERV	ERVLE	internal	chr8
HERVH_8q21	2.11488967	0.0178641	HERV	HERVH	prototype	chr8
HERVL18_8q	2.02401695	0.01042788	HERV	HERVL18	prototype	chr8
PRIMA41_8q	1.96847138	0.00045675	HERV	PRIMA41	internal	chr8
HERVL_8q21	1.57938237	0.01330278	HERV	HERVL	oneside	chr8
HML2_8p22	1.16343869	0.00813671	HERV	HML2	oneside	chr8
HERVE_8p23	1.0570919	0.03442744	HERV	HERVE	unknown	chr8
HERVL_8q24	1.0040476	0.03846727	HERV	HERVL	oneside	chr8
ERVLE_8p11.	-1.7784649	0.03112323	HERV	ERVLE	internal	chr8
HERVIP10FH_	-2.1827263	0.04757299	HERV	HERVIP10FH	oneside	chr8
ERVLE_8q21.	-2.4685298	0.03115536	HERV	ERVLE	internal	chr8
HERVL_8q21	-2.4884781	0.01287498	HERV	HERVL	prototype	chr8
ERV316A3_8	-2.9681177	0.00994164	HERV	ERV316A3	oneside	chr8
ERV316A3_9	2.90749106	0.01226027	HERV	ERV316A3	internal	chr9
ERV316A3_9	2.50655668	0.01357325	HERV	ERV316A3	prototype	chr9
ERVLE_9q31.	2.13771222	0.04206422	HERV	ERVLE	internal	chr9
HERVH_9p21	2.12856889	0.04976582	HERV	HERVH	oneside	chr9
ERV316A3_9	1.93627237	0.02809185	HERV	ERV316A3	internal	chr9
HERVFH19_9	1.92402468	0.02319862	HERV	HERVFH19	internal	chr9
MER4B_9q22	1.91182643	0.00060152	HERV	MER4B	internal	chr9
ERVLE_9p24.	1.90750376	0.01816344	HERV	ERVLE	internal	chr9
HERVL40_9p	1.88894401	0.02588858	HERV	HERVL40	prototype	chr9
ERVLE_9q32	1.86824394	0.04321398	HERV	ERVLE	internal	chr9

MER101_9q3	1.16309199	9.66E-05	HERV	MER101	internal	chr9
HERVL_9q21	1.12328188	0.00345305	HERV	HERVL	prototype	chr9
LTR23_9q22.	1.06589592	2.36E-07	HERV	LTR23	internal	chr9
ERV316A3_9	1.06392237	0.0009514	HERV	ERV316A3	oneside	chr9
LTR23_9q22.	1.05689609	1.02E-07	HERV	LTR23	oneside	chr9
HERV4_9p21	1.03245555	0.00972589	HERV	HERV4	internal	chr9
ERVLB4_9q2	1.01081486	0.04777409	HERV	ERVLB4	oneside	chr9
HERVL_Xp22	2.84017568	8.53E-05	HERV	HERVL	prototype	chrX
ERVLB4_Xq2	2.61594747	0.00637804	HERV	ERVLB4	internal	chrX
ERV316A3_X	2.25317119	3.75E-07	HERV	ERV316A3	oneside	chrX
ERVLB4_Xq2	2.22330081	0.01505999	HERV	ERVLB4	internal	chrX
ERVLE_Xq24	2.06566749	0.00340185	HERV	ERVLE	internal	chrX
MER61_Xq25	2.04159336	0.00026341	HERV	MER61	prototype	chrX
HML3_Xq21.	1.7522032	0.02357765	HERV	HML3	prototype	chrX
HERVW_Xp2	1.66799152	0.04777409	HERV	HERVW	prototype	chrX
ERVLB4_Xp2	1.48528559	0.00375046	HERV	ERVLB4	internal	chrX
HERVL_Xq24	1.40754739	0.00084834	HERV	HERVL	prototype	chrX
HERVH_Xq26	1.29658156	0.03351005	HERV	HERVH	prototype	chrX
PABLB_Xp21	1.25658437	0.02498203	HERV	PABLB	internal	chrX
LTR25_Xq21.	1.06766576	0.0497064	HERV	LTR25	internal	chrX
ERVLB4_Xp2	1.0272496	0.03212641	HERV	ERVLB4	internal	chrX

Start	End	Strand	TE_CODING	IntersectedG	IntersectedG	IntersectedO
173674381	173676802	-	noncoding	None	NA	NA
13012447	13021988	-	noncoding	None	NA	NA
106585294	106591567	-	noncoding	None	NA	NA
247354022	247360572	+	coding	None	NA	NA
38155702	38159588	+	noncoding	AL390839.1	lncRNA	SENSE
89429509	89431206	-	noncoding	AL596214.1	lncRNA	SENSE
38144395	38150922	+	noncoding	AL390839.1	lncRNA	SENSE
55022708	55028369	+	noncoding	None	NA	NA
9088458	9096029	+	noncoding	SLC2A5	protein_codi	ANTISENSE
173683772	173688508	-	noncoding	None	NA	NA
175269253	175277820	+	noncoding	None	NA	NA
157273298	157279886	-	noncoding	LINC02772	lncRNA	ANTISENSE
9086800	9088393	+	noncoding	SLC2A5	protein_codi	ANTISENSE
226833503	226838404	+	noncoding	AL359732.1	lncRNA	SENSE
35127220	35130565	-	noncoding	None	NA	NA
91206976	91211402	-	noncoding	None	NA	NA
43085534	43091157	+	noncoding	None	NA	NA
153470032	153474206	-	noncoding	None	NA	NA
94161627	94167221	-	noncoding	ARHGAP29	protein_codi	SENSE
207685206	207685768	-	noncoding	CR1L	protein_codi	ANTISENSE
35132025	35138025	-	noncoding	None	NA	NA
65945013	65946329	-	noncoding	PDE4B	protein_codi	ANTISENSE
65946472	65950867	-	noncoding	PDE4B	protein_codi	ANTISENSE
20041281	20045782	-	noncoding	PLA2G5	protein_codi	ANTISENSE
89048082	89049754	+	noncoding	None	NA	NA
65927540	65930375	-	coding	PDE4B	protein_codi	ANTISENSE
185499736	185501896	-	noncoding	None	NA	NA
172847553	172851416	-	noncoding	AL031599.1	lncRNA	ANTISENSE
84603880	84611675	+	noncoding	AL359762.2	lncRNA	SENSE
228947424	228951887	+	noncoding	None	NA	NA
185581069	185586238	-	noncoding	LINC01350	lncRNA	SENSE
62178464	62183789	-	noncoding	PATJ	protein_codi	ANTISENSE
88595833	88602999	+	noncoding	PKN2-AS1	lncRNA	ANTISENSE
246529011	246538336	+	noncoding	None	NA	NA
248773924	248775499	+	noncoding	None	NA	NA
169437009	169440502	-	noncoding	CCDC181	protein_codi	SENSE
161596130	161598811	+	noncoding	AL590385.2,I	lncRNA,polyr	SENSE,SENS
159056048	159059052	-	noncoding	None	NA	NA
159022297	159025513	-	noncoding	IFI16	protein_codi	ANTISENSE
242457057	242460442	+	coding	PLD5	protein_codi	ANTISENSE
102193133	102204851	-	coding	None	NA	NA
33580589	33586855	-	noncoding	LINC02628	lncRNA	SENSE

22138857	22146509	+		noncoding	None	NA	NA
32133699	32138852	-		noncoding	None	NA	NA
62356338	62360585	+		noncoding	AC024597.1	lncRNA	ANTISENSE
1349016	1354570	+		noncoding	ADARB2	protein_codi	ANTISENSE
57650629	57651896	+		noncoding	AP000662.1,(	lncRNA,prote	SENSE,SENS
4550034	4550543	+		noncoding	None	NA	NA
5808038	5809640	-		noncoding	TRIM5	protein_codi	SENSE
76725656	76729039	-		noncoding	None	NA	NA
55583344	55587752	+		noncoding	None	NA	NA
4554797	4558978	+		noncoding	None	NA	NA
105074657	105077688	+		noncoding	CARD16	protein_codi	ANTISENSE
67759685	67765364	+		noncoding	OR7E11P	lncRNA	ANTISENSE
4550544	4554796	+		noncoding	None	NA	NA
94809643	94812759	-		noncoding	AMOTL1	protein_codi	ANTISENSE
339634	342458	-		noncoding	None	NA	NA
127543108	127548460	-		noncoding	None	NA	NA
56327845	56333225	-		coding	FAM8A2P	unprocessed_	SENSE
111545120	111548379	-		noncoding	LAYN	protein_codi	ANTISENSE
67845158	67853485	-		noncoding	ENPP7P7	unprocessed_	SENSE
39560214	39567289	+		coding	None	NA	NA
112974379	112975753	+		noncoding	AC004551.1	lncRNA	ANTISENSE
4018541	4023694	+		noncoding	AC084375.1	lncRNA	SENSE
88227290	88235601	+		noncoding	None	NA	NA
93248752	93255087	-		coding	None	NA	NA
8558451	8562470	-		noncoding	AC092746.1	lncRNA	SENSE
102653258	102661243	+		noncoding	None	NA	NA
40160628	40165076	-		noncoding	LINC02471	lncRNA	ANTISENSE
68540228	68547978	+		noncoding	None	NA	NA
123548418	123557734	+		noncoding	None	NA	NA
55642877	55643624	+		noncoding	AC009779.6	lncRNA	ANTISENSE
80783123	80788892	-		noncoding	None	NA	NA
25437598	25442371	-		noncoding	LMNTD1,TUE	protein_codi	SENSE,SENS
131875335	131880952	-		noncoding	None	NA	NA
40187163	40191467	-		noncoding	LINC02471,LI	lncRNA,lncRI	ANTISENSE,S
127153655	127158069	+		noncoding	AC079949.3,.	lncRNA,lncRI	SENSE,ANTIS
40191686	40193336	-		noncoding	LINC02471,LI	lncRNA,lncRI	ANTISENSE,S
40210828	40213880	+		noncoding	LINC02471,LI	lncRNA,lncRI	SENSE,ANTIS
5818088	5826421	+		noncoding	ANO2	protein_codi	ANTISENSE
25307152	25313857	+		noncoding	None	NA	NA
40193379	40194785	-		noncoding	LINC02471,LI	lncRNA,lncRI	ANTISENSE,S
31936771	31945711	-		noncoding	LINC02422	lncRNA	SENSE
31906332	31909833	+		noncoding	LINC02422	lncRNA	ANTISENSE
109218916	109225445	-		noncoding	None	NA	NA

78664894	78669286	-		coding	None	NA	NA
109265090	109271116	+		noncoding	AL161431.1	lncRNA	SENSE
109236056	109238572	+		noncoding	None	NA	NA
41515871	41521881	+		noncoding	None	NA	NA
20600302	20607318	+		noncoding	AL163195.3,I	protein_codi	ANTISENSE,A
100762499	100766490	-		noncoding	None	NA	NA
29299954	29303478	-		noncoding	AL135878.1,A	lncRNA,lncRI	ANTISENSE,S
44591977	44596311	-		noncoding	None	NA	NA
42307103	42314189	-		noncoding	None	NA	NA
20710435	20719019	-		noncoding	None	NA	NA
103989420	103990330	-		noncoding	TDRD9	protein_codi	ANTISENSE
69811463	69816023	+		noncoding	None	NA	NA
80968503	80971945	-		noncoding	TSHR	protein_codi	ANTISENSE
51357366	51360808	+		noncoding	GLDN	protein_codi	SENSE
67932524	67936765	+		noncoding	None	NA	NA
94492346	94493815	+		noncoding	AC009432.2	lncRNA	ANTISENSE
65275256	65279034	-		noncoding	AC009055.1	lncRNA	ANTISENSE
58307595	58317295	+		noncoding	GINS3	protein_codi	SENSE
84855240	84861709	-		noncoding	AC025280.3,(	lncRNA,prote	SENSE,ANTIS
41089014	41095763	+		coding	AC100808.1	lncRNA	SENSE
31762039	31772128	+		noncoding	AC007923.1	lncRNA	SENSE
44292787	44299505	-		noncoding	None	NA	NA
62178517	62185522	-		noncoding	None	NA	NA
27686099	27696448	-		noncoding	None	NA	NA
8056337	8063901	+		coding	AC129492.2	lncRNA	ANTISENSE
80397328	80399793	+		noncoding	RNF213,RNF	protein_codi	SENSE,ANTIS
80265360	80268548	+		noncoding	RNF213	protein_codi	SENSE
4057062	4062956	+		noncoding	DLGAP1	protein_codi	ANTISENSE
12934879	12940618	-		noncoding	None	NA	NA
39795158	39800435	-		noncoding	MIR924HG	lncRNA	SENSE
52784756	52789686	-		noncoding	DCC	protein_codi	ANTISENSE
2625765	2630527	+		noncoding	None	NA	NA
58641222	58646708	-		noncoding	None	NA	NA
45977352	45982497	+		noncoding	None	NA	NA
43226418	43230569	-		noncoding	PSG9,CEACAI	protein_codi	SENSE,SENSI
43364281	43366105	-		noncoding	AC005392.3	lncRNA	SENSE
23549493	23555054	+		noncoding	ZNF675	protein_codi	ANTISENSE
5548576	5553212	+		noncoding	None	NA	NA
15604143	15605745	-		noncoding	None	NA	NA
21672129	21673402	-		noncoding	None	NA	NA
21454300	21456510	-		noncoding	AC010615.4,,	protein_codi	ANTISENSE,S
54894297	54900597	-		noncoding	None	NA	NA
21688200	21697956	-		noncoding	None	NA	NA

55122805	55129538	+	noncoding	AC010327.8,I	lncRNA,miRI	SENSE,ANTIS
36161029	36168089	-	noncoding	None	NA	NA
15828738	15834725	+	noncoding	LINC01764,U	lncRNA,lncRI	ANTISENSE,S
6838364	6846887	-	noncoding	NRIR,CMPK2	lncRNA,prote	SENSE,SENSI
16791945	16797703	-	noncoding	None	NA	NA
6869773	6876489	+	noncoding	RSAD2	protein_codi	SENSE
117130629	117135078	+	noncoding	None	NA	NA
70620550	70626409	+	noncoding	ADD2	protein_codi	ANTISENSE
102408621	102413726	+	coding	SDR42E1P5	processed_p	SENSE
218050832	218052950	+	noncoding	RUFY4	protein_codi	SENSE
37128668	37135454	+	noncoding	EIF2AK2	protein_codi	ANTISENSE
118949646	118953399	-	coding	MARCO,AC01	protein_codi	ANTISENSE,S
79211346	79217707	-	noncoding	CTNNA2	protein_codi	ANTISENSE
162324003	162329299	-	noncoding	GCA	protein_codi	ANTISENSE
136071818	136077261	-	coding	None	NA	NA
230119588	230123285	+	noncoding	AC009950.1	lncRNA	SENSE
10199041	10202321	-	noncoding	RRM2	protein_codi	ANTISENSE
218042417	218047874	-	noncoding	RUFY4	protein_codi	ANTISENSE
48062145	48064068	+	noncoding	AC092650.1	lncRNA	ANTISENSE
11395773	11400825	-	noncoding	LINC00570	lncRNA	ANTISENSE
48058827	48062129	-	noncoding	AC092650.1	lncRNA	SENSE
48051040	48052882	-	noncoding	AC092650.1	lncRNA	SENSE
218108692	218112255	-	noncoding	None	NA	NA
126645902	126652426	-	noncoding	AC013474.2	lncRNA	SENSE
218117007	218122161	-	coding	None	NA	NA
218112256	218117006	+	noncoding	None	NA	NA
197578410	197587726	+	noncoding	RFTN2	protein_codi	ANTISENSE
64252414	64257646	+	noncoding	AC012368.1	lncRNA	SENSE
172772	178853	+	noncoding	None	NA	NA
791798	800945	+	noncoding	None	NA	NA
46432835	46435517	-	noncoding	ELMO2,ZNF6	protein_codi	SENSE,SENSI
44670229	44676774	+	noncoding	AL139352.1	lncRNA	ANTISENSE
34231890	34234436	-	noncoding	ASIP	protein_codi	ANTISENSE
44668371	44670228	+	noncoding	AL139352.1	lncRNA	ANTISENSE
42600909	42605973	-	noncoding	LINC01671	lncRNA	SENSE
42598743	42600906	-	noncoding	LINC01671	lncRNA	SENSE
38224043	38229805	-	noncoding	KCNJ15	protein_codi	ANTISENSE
35662326	35668403	+	noncoding	APOL6	protein_codi	SENSE
41026681	41029740	+	noncoding	None	NA	NA
35668700	35673866	+	coding	None	NA	NA
46419310	46423900	-	coding	LINC02009	lncRNA	SENSE
46417556	46419247	-	noncoding	LINC02009	lncRNA	SENSE
57178398	57179278	+	noncoding	None	NA	NA

46426543	46433630	+	noncoding	None	NA	NA
185562548	185571727	-	coding	None	NA	NA
46414268	46416315	+	noncoding	None	NA	NA
187374111	187378966	-	noncoding	None	NA	NA
176321619	176327363	-	noncoding	None	NA	NA
179994615	180001424	+	noncoding	PEX5L,AC092	protein_codi	ANTISENSE,S
157579228	157588006	-	coding	SLC66A1L,AC	protein_codi	ANTISENSE,S
146353817	146359463	+	noncoding	None	NA	NA
130059102	130064974	+	noncoding	None	NA	NA
86537069	86543930	-	noncoding	None	NA	NA
12706372	12713168	-	noncoding	None	NA	NA
152597016	152603153	+	coding	None	NA	NA
121395494	121399876	-	noncoding	STXBP5L,AC0	protein_codi	ANTISENSE,S
42896033	42900433	+	noncoding	AC099329.2,A	lncRNA,prote	SENSE,SENSE
44855024	44859382	-	noncoding	KIF15	protein_codi	ANTISENSE
40560896	40566678	+	noncoding	ZNF621	protein_codi	SENSE
103208941	103215975	+	noncoding	None	NA	NA
46387685	46393402	-	noncoding	CCR5AS	lncRNA	SENSE
48723255	48730147	-	noncoding	IP6K2	protein_codi	SENSE
44768308	44774152	-	noncoding	KIF15	protein_codi	ANTISENSE
32067467	32076087	+	noncoding	OSBPL10	protein_codi	ANTISENSE
178050183	178053655	+	noncoding	AC110992.1	lncRNA	ANTISENSE
3828106	3835001	-	noncoding	SUMF1,LRRN	protein_codi	SENSE,ANTIS
91623640	91629699	+	noncoding	None	NA	NA
175444248	175456817	-	noncoding	None	NA	NA
127271438	127277471	-	noncoding	AC093772.1,A	lncRNA,lncRI	SENSE,SENSE
103941268	103947029	+	noncoding	None	NA	NA
128640894	128646609	-	noncoding	None	NA	NA
117600122	117603729	-	noncoding	LINC01378	lncRNA	ANTISENSE
156103922	156109304	-	noncoding	None	NA	NA
168144146	168148595	-	noncoding	ANXA10	protein_codi	ANTISENSE
3453754	3460384	+	coding	None	NA	NA
45042048	45049024	+	coding	AC108467.1	lncRNA	SENSE
88442739	88453401	+	noncoding	HERC6	protein_codi	SENSE
153696581	153699612	+	noncoding	TLR2	protein_codi	SENSE
139639594	139642728	-	noncoding	None	NA	NA
153688368	153694427	-	noncoding	TLR2	protein_codi	ANTISENSE
21639764	21643956	+	noncoding	KCNIP4	protein_codi	ANTISENSE
139632366	139639045	+	coding	None	NA	NA
168434326	168439585	+	noncoding	DDX60L	protein_codi	ANTISENSE
7750967	7752779	-	noncoding	None	NA	NA
117897885	117898825	-	noncoding	None	NA	NA
117898903	117900122	+	noncoding	None	NA	NA

85647002	85648160	-		noncoding	None	NA	NA
162275428	162281160	-		noncoding	None	NA	NA
25010097	25019988	-		noncoding	None	NA	NA
5515523	5521164	+		noncoding	None	NA	NA
112547505	112549086	+		noncoding	AC137549.1	processed_p	SENSE
159233488	159238427	-		noncoding	AC134043.2,l	lncRNA,lncRI	ANTISENSE,A
119435608	119439169	+		noncoding	None	NA	NA
179668380	179669547	-		noncoding	None	NA	NA
49717615	49721439	-		noncoding	AL121974.1	lncRNA	ANTISENSE
49721416	49722594	-		noncoding	AL121974.1,A	lncRNA,TEC	ANTISENSE,A
137720646	137722702	-		noncoding	AL356234.2	lncRNA	ANTISENSE
62585932	62591724	+		noncoding	None	NA	NA
121620422	121626300	-		noncoding	None	NA	NA
137718928	137720499	-		noncoding	AL356234.2	lncRNA	ANTISENSE
47312692	47316775	-		noncoding	None	NA	NA
6027810	6033600	-		noncoding	None	NA	NA
66561896	66569647	-		noncoding	None	NA	NA
110499174	110506068	+		coding	None	NA	NA
67867628	67874456	-		noncoding	None	NA	NA
164338769	164344779	-		noncoding	None	NA	NA
21355378	21361143	-		noncoding	None	NA	NA
110488048	110491199	+		noncoding	None	NA	NA
66258645	66264857	+		noncoding	None	NA	NA
123556844	123562344	+		noncoding	TRDN,AL445	protein_codi	ANTISENSE,S
122741515	122749262	-		coding	RN7SL564P	misc_RNA	ANTISENSE
77658021	77666441	-		coding	MEI4	protein_codi	ANTISENSE
16259012	16264893	-		noncoding	GMPR,AL009	protein_codi	ANTISENSE,S
170426568	170430754	-		noncoding	None	NA	NA
26288022	26296266	-		noncoding	None	NA	NA
29245477	29252808	-		noncoding	AL672167.1	transcribed_	ANTISENSE
134600811	134605423	-		noncoding	AL078590.2,l	lncRNA,proce	SENSE,SENSI
26257219	26260461	+		noncoding	None	NA	NA
117658797	117665200	-		noncoding	None	NA	NA
109876944	109882777	+		coding	None	NA	NA
29962489	29970366	+		noncoding	None	NA	NA
6688741	6691684	+		noncoding	None	NA	NA
29946920	29950412	+		noncoding	None	NA	NA
41547054	41549561	-		noncoding	None	NA	NA
41553289	41559722	-		noncoding	None	NA	NA
148575970	148579927	-		noncoding	None	NA	NA
66304013	66309489	-		noncoding	TPST1	protein_codi	ANTISENSE
26996592	27002445	+		noncoding	None	NA	NA
138364700	138368299	+		noncoding	None	NA	NA

50576977	50583954	+	noncoding	None	NA	NA
56144893	56153451	+	coding	None	NA	NA
106548063	106548761	-	noncoding	AC004917.1	lncRNA	SENSE
135525117	135526758	+	noncoding	None	NA	NA
6950409	6958745	-	coding	AC079804.3	lncRNA	SENSE
64948007	64950796	+	noncoding	AC073210.3	lncRNA	SENSE
22099082	22102480	-	noncoding	None	NA	NA
106527752	106533658	-	noncoding	AC004917.1	lncRNA	SENSE
91447505	91456909	-	noncoding	AC079760.2,.	lncRNA,lncRI	ANTISENSE,S
93148971	93154061	+	noncoding	None	NA	NA
32832561	32838271	+	noncoding	DPY19L1P2	unprocessed_	SENSE
12588442	12595509	-	coding	SCIN	protein_codi	ANTISENSE
140290502	140295513	-	noncoding	PPP1R2P6,SL	processed_p:	ANTISENSE,S
93127516	93129355	+	noncoding	None	NA	NA
135175955	135180551	+	coding	None	NA	NA
135171264	135175958	+	coding	AC083862.3	lncRNA	SENSE
106451448	106455433	-	noncoding	AC004917.1,.	lncRNA,lncRI	SENSE,ANTIS
106475939	106477621	+	noncoding	AC004917.1,.	lncRNA,lncRI	ANTISENSE,S
106455435	106461177	-	noncoding	AC004917.1,.	lncRNA,lncRI	SENSE,ANTIS
129578164	129583087	+	coding	None	NA	NA
64811247	64815461	-	noncoding	AC104232.3,.	lncRNA,lncRI	ANTISENSE,S
83543715	83550668	+	noncoding	None	NA	NA
53482820	53488834	+	noncoding	AC022034.3	lncRNA	ANTISENSE
143050063	143053296	+	noncoding	C8orf31	lncRNA	SENSE
81242463	81243446	-	noncoding	None	NA	NA
17908096	17916431	-	coding	FGL1	protein_codi	SENSE
11907117	11913562	-	coding	None	NA	NA
143195432	143196865	+	noncoding	None	NA	NA
37200874	37202357	-	noncoding	None	NA	NA
89233917	89240391	-	noncoding	None	NA	NA
89330815	89335376	-	noncoding	None	NA	NA
89448797	89455259	+	noncoding	None	NA	NA
89208679	89212564	-	noncoding	None	NA	NA
135378186	135382106	-	noncoding	None	NA	NA
81128337	81134257	+	noncoding	None	NA	NA
102017314	102020177	+	noncoding	None	NA	NA
32164364	32169199	-	noncoding	None	NA	NA
81000385	81001884	-	noncoding	None	NA	NA
129588167	129594670	-	noncoding	None	NA	NA
98041983	98045194	-	noncoding	None	NA	NA
6652653	6654143	+	noncoding	AL162411.1	lncRNA	SENSE
32371344	32377156	-	noncoding	None	NA	NA
112982027	112984145	-	noncoding	ZNF883	transcribed_	SENSE

105055371	105056952	+		noncoding	AL591506.1	lncRNA	ANTISENSE
83843916	83847951	+		noncoding	AL354733.2,I	lncRNA,prote	SENSE,ANTIS
98111494	98115852	+		noncoding	TRIM14	protein_codi	ANTISENSE
34158413	34168326	-		noncoding	None	NA	NA
98116034	98117804	+		noncoding	TRIM14,AL13	protein_codi	ANTISENSE,S
29130143	29131215	+		noncoding	None	NA	NA
92779111	92783192	+		noncoding	None	NA	NA
9809093	9815987	-		noncoding	SHROOM2	protein_codi	ANTISENSE
121635051	121636749	+		noncoding	None	NA	NA
23748936	23751401	-		noncoding	ACOT9	protein_codi	SENSE
131906090	131909121	-		noncoding	None	NA	NA
121538387	121541566	-		noncoding	None	NA	NA
121903416	121909275	+		noncoding	None	NA	NA
80042634	80048272	+		noncoding	None	NA	NA
7699169	7704068	-		noncoding	STS	protein_codi	ANTISENSE
37475533	37478048	-		noncoding	AF241726.2	protein_codi	ANTISENSE
121449325	121455728	+		noncoding	None	NA	NA
138264358	138270335	-		noncoding	None	NA	NA
37466596	37469950	+		noncoding	AF241726.2	protein_codi	SENSE
80312695	80317563	+		coding	CHMP1B2P	transcribed_	ANTISENSE
37461988	37465035	-		noncoding	AF241726.2	protein_codi	ANTISENSE

IntersectedG	TE_type	TE_5UTR_Int	TE_3UTR_Int	ClosestUpstr	ClosestUpstr	DistUpstrear
None	INTERGENIC	NA	NA	KLHL20	ENSG00000C	38139
None	INTERGENIC	NA	NA	PRAMEF28P	ENSG000002	7351
None	INTERGENIC	NA	NA	AL596327.1	ENSG000002	188656
None	INTERGENIC	NA	NA	AC104335.2	ENSG000002	5946
ENSG000002	INTRONIC	NA	NA	NA	NA	NA
ENSG000002	INTRONIC	NA	NA	NA	NA	NA
ENSG000002	EXONIC	FALSE	FALSE	NA	NA	NA
None	INTERGENIC	NA	NA	BSND	ENSG000001	5536
ENSG000001	EXONIC	TRUE	FALSE	NA	NA	NA
None	INTERGENIC	NA	NA	KLHL20	ENSG00000C	26433
None	INTERGENIC	NA	NA	AL008626.1	ENSG000002	64404
ENSG000002	EXONIC	FALSE	FALSE	NA	NA	NA
ENSG000001	EXONIC	TRUE	FALSE	NA	NA	NA
ENSG000002	EXONIC	FALSE	FALSE	NA	NA	NA
None	INTERGENIC	NA	NA	AL590434.2	ENSG000002	10950
None	INTERGENIC	NA	NA	HFM1	ENSG000001	49364
None	INTERGENIC	NA	NA	AL161637.2	ENSG000002	61897
None	INTERGENIC	NA	NA	RN7SL44P	ENSG000002	26257
ENSG000001	INTRONIC	NA	NA	NA	NA	NA
ENSG000001	INTRONIC	NA	NA	NA	NA	NA
None	INTERGENIC	NA	NA	AL590434.2	ENSG000002	3490
ENSG000001	INTRONIC	NA	NA	NA	NA	NA
ENSG000001	INTRONIC	NA	NA	NA	NA	NA
ENSG000001	INTRONIC	NA	NA	NA	NA	NA
None	INTERGENIC	NA	NA	GBP3	ENSG000001	25188
ENSG000001	INTRONIC	NA	NA	NA	NA	NA
None	INTERGENIC	NA	NA	AL133383.4	ENSG000002	16755
ENSG000002	INTRONIC	NA	NA	NA	NA	NA
ENSG000002	EXONIC	FALSE	FALSE	NA	NA	NA
None	INTERGENIC	NA	NA	AL078624.1	ENSG000002	88868
ENSG000002	EXONIC	FALSE	FALSE	NA	NA	NA
ENSG000001	EXONIC	FALSE	TRUE	NA	NA	NA
ENSG000002	INTRONIC	NA	NA	NA	NA	NA
None	INTERGENIC	NA	NA	AL356583.3	ENSG000002	170
None	INTERGENIC	NA	NA	DPY19L4P1	ENSG000002	132
ENSG000001	INTRONIC	NA	NA	NA	NA	NA
ENSG000002	EXONIC	FALSE	FALSE	NA	NA	NA
None	INTERGENIC	NA	NA	AIM2	ENSG000001	3432
ENSG000001	INTRONIC	NA	NA	NA	NA	NA
ENSG000001	INTRONIC	NA	NA	NA	NA	NA
None	INTERGENIC	NA	NA	ELOVL3	ENSG000001	21448
ENSG000002	INTRONIC	NA	NA	NA	NA	NA

None	INTERGENIC	NA	NA	DNAJC1	ENSG000001	135088
None	INTERGENIC	NA	NA	RNU7-22P	ENSG000002	34832
ENSG000002	INTRONIC	NA	NA	NA	NA	NA
ENSG000001	INTRONIC	NA	NA	NA	NA	NA
ENSG000002	EXONIC	FALSE	FALSE	NA	NA	NA
None	INTERGENIC	NA	NA	OR52M1	ENSG000001	3890
ENSG000001	INTRONIC	NA	NA	NA	NA	NA
None	INTERGENIC	NA	NA	AP003119.1	ENSG000002	30877
None	INTERGENIC	NA	NA	OR4C16	ENSG000002	10284
None	INTERGENIC	NA	NA	OR52M1	ENSG000001	8653
ENSG000002	INTRONIC	NA	NA	NA	NA	NA
ENSG000002	EXONIC	FALSE	FALSE	NA	NA	NA
None	INTERGENIC	NA	NA	OR52M1	ENSG000001	4400
ENSG000001	INTRONIC	NA	NA	NA	NA	NA
None	INTERGENIC	NA	NA	B4GALNT4	ENSG000001	27041
None	INTERGENIC	NA	NA	AP003532.1	ENSG000002	109108
ENSG000002	EXONIC	FALSE	FALSE	NA	NA	NA
ENSG000002	INTRONIC	NA	NA	NA	NA	NA
ENSG000002	INTRONIC	NA	NA	NA	NA	NA
None	INTERGENIC	NA	NA	RNU6-99P	ENSG000002	299001
ENSG000002	INTRONIC	NA	NA	NA	NA	NA
ENSG000002	EXONIC	FALSE	FALSE	NA	NA	NA
None	INTERGENIC	NA	NA	TMTC3	ENSG000001	27403
None	INTERGENIC	NA	NA	AC126172.1	ENSG000002	10930
ENSG000002	INTRONIC	NA	NA	NA	NA	NA
None	INTERGENIC	NA	NA	AC010202.1	ENSG000002	139603
ENSG000002	INTRONIC	NA	NA	NA	NA	NA
None	INTERGENIC	NA	NA	AC008033.2	ENSG000002	52365
None	INTERGENIC	NA	NA	MIR3908	ENSG000002	11884
ENSG000002	INTRONIC	NA	NA	NA	NA	NA
None	INTERGENIC	NA	NA	LIN7A	ENSG000001	3628
ENSG000001	EXONIC	FALSE	FALSE	NA	NA	NA
None	INTERGENIC	NA	NA	ULK1	ENSG000001	13670
ENSG000002	EXONIC	FALSE	FALSE	NA	NA	NA
ENSG000002	INTRONIC	NA	NA	NA	NA	NA
ENSG000002	INTRONIC	NA	NA	NA	NA	NA
ENSG000002	EXONIC	FALSE	FALSE	NA	NA	NA
ENSG000000	INTRONIC	NA	NA	NA	NA	NA
None	INTERGENIC	NA	NA	KRAS	ENSG000001	56216
ENSG000002	INTRONIC	NA	NA	NA	NA	NA
ENSG000002	INTRONIC	NA	NA	NA	NA	NA
ENSG000002	INTRONIC	NA	NA	NA	NA	NA
None	INTERGENIC	NA	NA	AL161431.1	ENSG000002	44189

None	INTERGENIC	NA	NA	RPL21P111	ENSG000002	91889
ENSG000002	EXONIC	FALSE	FALSE	NA	NA	NA
None	INTERGENIC	NA	NA	MYO16	ENSG00000C	28051
None	INTERGENIC	NA	NA	AL356596.1	ENSG000002	521
ENSG000002	EXONIC	FALSE	FALSE	NA	NA	NA
None	INTERGENIC	NA	NA	MEG3	ENSG000002	12920
ENSG000002	INTRONIC	NA	NA	NA	NA	NA
None	INTERGENIC	NA	NA	LINC02302	ENSG000002	166842
None	INTERGENIC	NA	NA	AL442163.1	ENSG000002	48794
None	INTERGENIC	NA	NA	RANBP20P	ENSG000002	1723
ENSG000001	INTRONIC	NA	NA	NA	NA	NA
None	INTERGENIC	NA	NA	AL157789.1	ENSG000002	7132
ENSG000001	INTRONIC	NA	NA	NA	NA	NA
ENSG000001	EXONIC	FALSE	FALSE	NA	NA	NA
None	INTERGENIC	NA	NA	AC009292.2	ENSG000002	58658
ENSG000002	INTRONIC	NA	NA	NA	NA	NA
ENSG000002	INTRONIC	NA	NA	NA	NA	NA
ENSG000001	INTRONIC	NA	NA	NA	NA	NA
ENSG000002	EXONIC	FALSE	FALSE	NA	NA	NA
ENSG000002	EXONIC	FALSE	FALSE	NA	NA	NA
ENSG000002	EXONIC	FALSE	FALSE	NA	NA	NA
None	INTERGENIC	NA	NA	RUNDC3A-AS	ENSG000002	69
None	INTERGENIC	NA	NA	AC053481.3	ENSG000002	45948
None	INTERGENIC	NA	NA	AC130289.1	ENSG000002	10730
ENSG000002	EXONIC	FALSE	FALSE	NA	NA	NA
ENSG000001	EXONIC	FALSE	TRUE	NA	NA	NA
ENSG000001	INTRONIC	NA	NA	NA	NA	NA
ENSG000001	INTRONIC	NA	NA	NA	NA	NA
None	INTERGENIC	NA	NA	SEH1L	ENSG00000C	6515
ENSG000002	EXONIC	FALSE	FALSE	NA	NA	NA
ENSG000001	INTRONIC	NA	NA	NA	NA	NA
None	INTERGENIC	NA	NA	AP005061.1	ENSG000002	4564
None	INTERGENIC	NA	NA	AC104971.3	ENSG000002	13150
None	INTERGENIC	NA	NA	EPG5	ENSG000001	10023
ENSG000001	EXONIC	FALSE	FALSE	NA	NA	NA
ENSG000002	INTRONIC	NA	NA	NA	NA	NA
ENSG000001	INTRONIC	NA	NA	NA	NA	NA
None	INTERGENIC	NA	NA	ZNRF4	ENSG000001	91720
None	INTERGENIC	NA	NA	AD000091.1	ENSG000002	5878
None	INTERGENIC	NA	NA	MTDHP3	ENSG000002	4148
ENSG000002	EXONIC	FALSE	FALSE	NA	NA	NA
None	INTERGENIC	NA	NA	NCR1	ENSG000001	5551
None	INTERGENIC	NA	NA	MTDHP4	ENSG000002	11566

ENSG000002	EXONIC	FALSE	FALSE	NA	NA	NA
None	INTERGENIC	NA	NA	AC002984.1	ENSG000002	5317
ENSG000002	EXONIC	FALSE	FALSE	NA	NA	NA
ENSG000002	EXONIC	FALSE	FALSE	NA	NA	NA
None	INTERGENIC	NA	NA	AC008069.1	ENSG000002	56117
ENSG000001	INTRONIC	NA	NA	NA	NA	NA
None	INTERGENIC	NA	NA	MTCO1P43	ENSG000002	103605
ENSG000000	INTRONIC	NA	NA	NA	NA	NA
ENSG000002	EXONIC	FALSE	FALSE	NA	NA	NA
ENSG000001	INTRONIC	NA	NA	NA	NA	NA
ENSG000000	INTRONIC	NA	NA	NA	NA	NA
ENSG000000	EXONIC	FALSE	FALSE	NA	NA	NA
ENSG000000	INTRONIC	NA	NA	NA	NA	NA
ENSG000001	INTRONIC	NA	NA	NA	NA	NA
None	INTERGENIC	NA	NA	AC068492.1	ENSG000002	631
ENSG000002	EXONIC	FALSE	FALSE	NA	NA	NA
ENSG000001	EXONIC	FALSE	TRUE	NA	NA	NA
ENSG000001	INTRONIC	NA	NA	NA	NA	NA
ENSG000002	INTRONIC	NA	NA	NA	NA	NA
ENSG000002	EXONIC	FALSE	FALSE	NA	NA	NA
ENSG000002	INTRONIC	NA	NA	NA	NA	NA
ENSG000002	INTRONIC	NA	NA	NA	NA	NA
None	INTERGENIC	NA	NA	CXCR2	ENSG000001	13034
ENSG000002	EXONIC	FALSE	FALSE	NA	NA	NA
None	INTERGENIC	NA	NA	CXCR2	ENSG000001	3128
None	INTERGENIC	NA	NA	RUFY4	ENSG000001	21675
ENSG000001	INTRONIC	NA	NA	NA	NA	NA
ENSG000002	EXONIC	FALSE	FALSE	NA	NA	NA
None	INTERGENIC	NA	NA	DEFB127	ENSG000000	13609
None	INTERGENIC	NA	NA	SLC52A3	ENSG000001	15783
ENSG000000	EXONIC	FALSE	FALSE	NA	NA	NA
ENSG000001	INTRONIC	NA	NA	NA	NA	NA
ENSG000001	INTRONIC	NA	NA	NA	NA	NA
ENSG000001	INTRONIC	NA	NA	NA	NA	NA
ENSG000002	INTRONIC	NA	NA	NA	NA	NA
ENSG000002	EXONIC	FALSE	FALSE	NA	NA	NA
ENSG000001	INTRONIC	NA	NA	NA	NA	NA
ENSG000002	EXONIC	FALSE	TRUE	NA	NA	NA
None	INTERGENIC	NA	NA	RBX1	ENSG000001	53372
None	INTERGENIC	NA	NA	APOL6	ENSG000002	296
ENSG000002	EXONIC	FALSE	FALSE	NA	NA	NA
ENSG000002	EXONIC	FALSE	FALSE	NA	NA	NA
None	INTERGENIC	NA	NA	IL17RD	ENSG000001	8092

None	INTERGENIC	NA	NA	LINC02009	ENSG000002	2952
None	INTERGENIC	NA	NA	SENP2	ENSG000001	10769
None	INTERGENIC	NA	NA	CCRL2	ENSG000001	1271
None	INTERGENIC	NA	NA	AC068299.2	ENSG000002	4135
None	INTERGENIC	NA	NA	LINC01208	ENSG000002	88076
ENSG000001	INTRONIC	NA	NA	NA	NA	NA
ENSG000001	EXONIC	FALSE	FALSE	NA	NA	NA
None	INTERGENIC	NA	NA	AC130509.1	ENSG000002	5337
None	INTERGENIC	NA	NA	AC083906.4	ENSG000002	3182
None	INTERGENIC	NA	NA	AC108706.1	ENSG000002	272810
None	INTERGENIC	NA	NA	TMEM40	ENSG000000	20360
None	INTERGENIC	NA	NA	AC026347.2	ENSG000002	100203
ENSG000001	EXONIC	FALSE	FALSE	NA	NA	NA
ENSG000002	EXONIC	FALSE	FALSE	NA	NA	NA
ENSG000001	INTRONIC	NA	NA	NA	NA	NA
ENSG000001	INTRONIC	NA	NA	NA	NA	NA
None	INTERGENIC	NA	NA	RNU1-43P	ENSG000002	48817
ENSG000002	EXONIC	FALSE	FALSE	NA	NA	NA
ENSG000000	EXONIC	TRUE	FALSE	NA	NA	NA
ENSG000001	INTRONIC	NA	NA	NA	NA	NA
ENSG000001	INTRONIC	NA	NA	NA	NA	NA
ENSG000002	INTRONIC	NA	NA	NA	NA	NA
ENSG000001	INTRONIC	NA	NA	NA	NA	NA
None	INTERGENIC	NA	NA	AC110774.1	ENSG000002	18348
None	INTERGENIC	NA	NA	AC131094.1	ENSG000002	909
ENSG000002	INTRONIC	NA	NA	NA	NA	NA
None	INTERGENIC	NA	NA	RNU6-635P	ENSG000002	16627
None	INTERGENIC	NA	NA	JADRR	ENSG000002	121619
ENSG000002	EXONIC	FALSE	FALSE	NA	NA	NA
None	INTERGENIC	NA	NA	AC096736.3	ENSG000002	476675
ENSG000001	INTRONIC	NA	NA	NA	NA	NA
None	INTERGENIC	NA	NA	AL590235.2	ENSG000002	646
ENSG000002	INTRONIC	NA	NA	NA	NA	NA
ENSG000001	EXONIC	FALSE	TRUE	NA	NA	NA
ENSG000001	INTRONIC	NA	NA	NA	NA	NA
None	INTERGENIC	NA	NA	MGST2	ENSG000000	23040
ENSG000001	EXONIC	FALSE	FALSE	NA	NA	NA
ENSG000001	INTRONIC	NA	NA	NA	NA	NA
None	INTERGENIC	NA	NA	AC112236.1	ENSG000002	9134
ENSG000001	INTRONIC	NA	NA	NA	NA	NA
None	INTERGENIC	NA	NA	AFAP1-AS1	ENSG000002	1298
None	INTERGENIC	NA	NA	NDST3	ENSG000001	134793
None	INTERGENIC	NA	NA	LINC02264	ENSG000002	28943

None	INTERGENIC	NA	NA	AC010595.1	ENSG000002	15072
None	INTERGENIC	NA	NA	AC113414.1	ENSG000002	142882
None	INTERGENIC	NA	NA	LINC02228	ENSG000002	67462
None	INTERGENIC	NA	NA	ICE1	ENSG000001	25303
ENSG000002	EXONIC	FALSE	FALSE	NA	NA	NA
ENSG000002	INTRONIC	NA	NA	NA	NA	NA
None	INTERGENIC	NA	NA	TNFAIP8	ENSG000001	35920
None	INTERGENIC	NA	NA	CBY3	ENSG000002	9013
ENSG000002	INTRONIC	NA	NA	NA	NA	NA
ENSG000002	EXONIC	FALSE	FALSE	NA	NA	NA
ENSG000002	INTRONIC	NA	NA	NA	NA	NA
None	INTERGENIC	NA	NA	AL355375.1	ENSG000002	37660
None	INTERGENIC	NA	NA	AL603865.2	ENSG000002	14709
ENSG000002	INTRONIC	NA	NA	NA	NA	NA
None	INTERGENIC	NA	NA	B3GNTL1P2	ENSG000002	52168
None	INTERGENIC	NA	NA	F13A1	ENSG000001	110484
None	INTERGENIC	NA	NA	AL450336.1	ENSG000002	140595
None	INTERGENIC	NA	NA	AC002464.1	ENSG000002	19738
None	INTERGENIC	NA	NA	AL713998.1	ENSG000002	3860
None	INTERGENIC	NA	NA	AL358972.1	ENSG000002	1710
None	INTERGENIC	NA	NA	AL031767.2	ENSG000002	8547
None	INTERGENIC	NA	NA	AC002464.1	ENSG000002	8612
None	INTERGENIC	NA	NA	NUFIP1P1	ENSG000002	163736
ENSG000001	EXONIC	FALSE	FALSE	NA	NA	NA
ENSG000002	EXONIC	FALSE	FALSE	NA	NA	NA
ENSG000002	INTRONIC	NA	NA	NA	NA	NA
ENSG000001	EXONIC	FALSE	FALSE	NA	NA	NA
None	INTERGENIC	NA	NA	PSMB1	ENSG000000	104366
None	INTERGENIC	NA	NA	H3C9P	ENSG000002	25610
ENSG000001	INTRONIC	NA	NA	NA	NA	NA
ENSG000002	EXONIC	FALSE	FALSE	NA	NA	NA
None	INTERGENIC	NA	NA	H2BC9	ENSG000002	3509
None	INTERGENIC	NA	NA	NUS1	ENSG000001	10269
None	INTERGENIC	NA	NA	AL512303.1	ENSG000002	48159
None	INTERGENIC	NA	NA	HLA-W	ENSG000002	3919
None	INTERGENIC	NA	NA	AL031123.3	ENSG000002	1757
None	INTERGENIC	NA	NA	HLA-A	ENSG000002	1036
None	INTERGENIC	NA	NA	INHBA	ENSG000001	117607
None	INTERGENIC	NA	NA	INHBA	ENSG000001	107446
None	INTERGENIC	NA	NA	AC005229.2	ENSG000002	474
ENSG000001	INTRONIC	NA	NA	NA	NA	NA
None	INTERGENIC	NA	NA	SKAP2	ENSG000000	1353
None	INTERGENIC	NA	NA	AC008155.1	ENSG000002	66216

None	INTERGENIC	NA	NA	DDC	ENSG000001	11572
None	INTERGENIC	NA	NA	NUPR2	ENSG000001	28476
ENSG000002	INTRONIC	NA	NA	NA	NA	NA
None	INTERGENIC	NA	NA	CNOT4	ENSG000000	14990
ENSG000002	EXONIC	FALSE	FALSE	NA	NA	NA
ENSG000002	EXONIC	FALSE	FALSE	NA	NA	NA
None	INTERGENIC	NA	NA	RAPGEF5	ENSG000001	15756
ENSG000002	INTRONIC	NA	NA	NA	NA	NA
ENSG000002	EXONIC	FALSE	FALSE	NA	NA	NA
None	INTERGENIC	NA	NA	SAMD9L	ENSG000001	586
ENSG000002	INTRONIC	NA	NA	NA	NA	NA
ENSG000000	EXONIC	TRUE	FALSE	NA	NA	NA
ENSG000002	EXONIC	FALSE	TRUE	NA	NA	NA
None	INTERGENIC	NA	NA	SAMD9	ENSG000002	9493
None	INTERGENIC	NA	NA	AC083862.3	ENSG000002	3541
ENSG000002	EXONIC	FALSE	FALSE	NA	NA	NA
ENSG000002	INTRONIC	NA	NA	NA	NA	NA
ENSG000002	INTRONIC	NA	NA	NA	NA	NA
ENSG000002	INTRONIC	NA	NA	NA	NA	NA
None	INTERGENIC	NA	NA	SMKR1	ENSG000002	65246
ENSG000002	INTRONIC	NA	NA	NA	NA	NA
None	INTERGENIC	NA	NA	LINC01419	ENSG000002	134815
ENSG000002	EXONIC	FALSE	FALSE	NA	NA	NA
ENSG000001	EXONIC	FALSE	FALSE	NA	NA	NA
None	INTERGENIC	NA	NA	AC009902.1	ENSG000002	710
ENSG000001	EXONIC	TRUE	FALSE	NA	NA	NA
None	INTERGENIC	NA	NA	OR7E158P	ENSG000002	6338
None	INTERGENIC	NA	NA	LY6H	ENSG000001	34721
None	INTERGENIC	NA	NA	AC091182.1	ENSG000002	124218
None	INTERGENIC	NA	NA	RPSAP74	ENSG000002	3010
None	INTERGENIC	NA	NA	KRT8P4	ENSG000002	77245
None	INTERGENIC	NA	NA	KRT8P4	ENSG000002	33726
None	INTERGENIC	NA	NA	RPSAP74	ENSG000002	30837
None	INTERGENIC	NA	NA	SOCS5P2	ENSG000002	65340
None	INTERGENIC	NA	NA	AL138749.1	ENSG000002	257042
None	INTERGENIC	NA	NA	MTND3P4	ENSG000002	223558
None	INTERGENIC	NA	NA	RNA5SP281	ENSG000002	124359
None	INTERGENIC	NA	NA	RPS20P25	ENSG000002	408395
None	INTERGENIC	NA	NA	NTMT1	ENSG000001	14214
None	INTERGENIC	NA	NA	NANS	ENSG000000	11538
ENSG000002	INTRONIC	NA	NA	NA	NA	NA
None	INTERGENIC	NA	NA	ACO1	ENSG000001	7447
ENSG000002	EXONIC	FALSE	FALSE	NA	NA	NA

ENSG000002	INTRONIC	NA	NA	NA	NA	NA
ENSG000002	EXONIC	FALSE	FALSE	NA	NA	NA
ENSG000001	INTRONIC	NA	NA	NA	NA	NA
None	INTERGENIC	NA	NA	UBAP1	ENSG000001	10679
ENSG000001	EXONIC	FALSE	FALSE	NA	NA	NA
None	INTERGENIC	NA	NA	MIR873	ENSG000002	241188
None	INTERGENIC	NA	NA	BICD2	ENSG000001	14278
ENSG000001	INTRONIC	NA	NA	NA	NA	NA
None	INTERGENIC	NA	NA	MIR3672	ENSG000002	263998
ENSG000001	INTRONIC	NA	NA	NA	NA	NA
None	INTERGENIC	NA	NA	AL008629.1	ENSG000002	28549
None	INTERGENIC	NA	NA	TPT1P13	ENSG000002	322132
None	INTERGENIC	NA	NA	AL359851.1	ENSG000002	26551
None	INTERGENIC	NA	NA	TBX22	ENSG000001	10860
ENSG000001	INTRONIC	NA	NA	NA	NA	NA
ENSG000002	INTRONIC	NA	NA	NA	NA	NA
None	INTERGENIC	NA	NA	MIR3672	ENSG000002	78272
None	INTERGENIC	NA	NA	RN7SKP31	ENSG000002	127016
ENSG000002	INTRONIC	NA	NA	NA	NA	NA
ENSG000002	INTRONIC	NA	NA	NA	NA	NA
ENSG000002	INTRONIC	NA	NA	NA	NA	NA

ClosestDown	ClosestDown	DistDownstr	FANTOM_en	FANTOM_CAGE
ANKRD45	ENSG000001	4530	None	None
PRAMEF6	ENSG000002	64867	None	None
AL499605.1	ENSG000002	40550	None	None
AC104335.3	ENSG000002	29671	chr1:247357	chr1:247359352-247359363,chr1:24736
NA	NA	NA	None	None
NA	NA	NA	None	None
NA	NA	NA	None	None
PCSK9	ENSG000001	11078	None	None
NA	NA	NA	None	chr1:9088436-9088458,chr1:9094181-9
ANKRD45	ENSG000001	13921	None	None
RPS29P4	ENSG000002	19260	None	None
NA	NA	NA	chr1:157276	chr1:157274870-157274893,chr1:15727
NA	NA	NA	None	None
NA	NA	NA	None	None
EFCAB14P1	ENSG000002	4736	None	None
RPL5P6	ENSG000002	182172	None	None
AL161637.1	ENSG000002	12929	None	None
S100A7	ENSG000001	9381	None	None
NA	NA	NA	None	None
NA	NA	NA	None	None
EFCAB14P1	ENSG000002	9541	None	None
NA	NA	NA	None	None
NA	NA	NA	None	None
NA	NA	NA	None	None
GBP1	ENSG000001	2565	None	None
NA	NA	NA	None	None
MCRIP2P2	ENSG000002	63689	None	None
NA	NA	NA	chr1:172850	chr1:172851371-172851383
NA	NA	NA	None	None
LINC02815	ENSG000002	70886	None	None
NA	NA	NA	None	None
NA	NA	NA	None	chr1:62178587-62178591
NA	NA	NA	None	None
TFB2M	ENSG000001	2225	None	None
SH3BP5L	ENSG000001	34947	None	None
NA	NA	NA	None	None
NA	NA	NA	None	None
IFI16	ENSG000001	893	None	chr1:159056714-159056718
NA	NA	NA	chr1:159024	chr1:159024957-159024959
NA	NA	NA	None	None
NOLC1	ENSG000001	29262	None	chr10:102193241-102193246
NA	NA	NA	None	None

ADIPOR1P1	ENSG000002		16483	None	None
AL161932.2	ENSG000002		21910	chr10:32137(	None
NA	NA	NA		None	None
NA	NA	NA		None	None
NA	NA	NA		None	None
C11orf40	ENSG000001		20880	None	None
NA	NA	NA		None	chr11:5809247-5809258
GUCY2EP	ENSG000002		5855	None	None
OR4C11	ENSG000001		14608	None	None
C11orf40	ENSG000001		12445	None	None
NA	NA	NA		None	None
NA	NA	NA		None	None
C11orf40	ENSG000001		16627	None	None
NA	NA	NA		None	None
AC136475.9	ENSG000002		5946	chr11:34147	None
RN7SKP279	ENSG000002		134960	None	None
NA	NA	NA		None	None
NA	NA	NA		None	None
NA	NA	NA		None	None
AC021749.1	ENSG000002		306493	None	None
NA	NA	NA		None	None
NA	NA	NA		None	chr12:4018922-4018949,chr12:4021088
AC129416.1	ENSG000001		194841	chr12:88227	None
AC138123.1	ENSG000002		33073	None	None
NA	NA	NA		None	None
LINC00485	ENSG000002		148037	None	None
NA	NA	NA		None	None
RPSAP12	ENSG000002		5017	None	None
TMED2-DT	ENSG000002		18157	None	None
NA	NA	NA		None	chr12:55642940-55642962
LINC01490	ENSG000002		12406	None	None
NA	NA	NA		None	None
AC131009.2	ENSG000002		10797	None	None
NA	NA	NA		None	None
NA	NA	NA		None	None
NA	NA	NA		None	None
NA	NA	NA		None	None
NA	NA	NA		None	None
AC087239.1	ENSG000002		71813	None	None
NA	NA	NA		None	None
NA	NA	NA		None	None
NA	NA	NA		chr12:31906(	chr12:31906983-31906986
MYO16	ENSG00000C		10911	None	None

OBI1	ENSG000001	5739	None	None
NA	NA	NA		None	chr13:109265485-109265518,chr13:109
AL161431.1	ENSG000002	31062	None	None
AL121821.3	ENSG000002	33781	None	None
NA	NA	NA		None	None
DLK1	ENSG000001	24275	None	None
NA	NA	NA		None	None
AL356022.1	ENSG000002	24510	None	None
AL445074.1	ENSG000002	38517	None	None
AL163636.2	ENSG000002	3315	chr14:20714(	None
NA	NA	NA		None	None
SMOC1	ENSG000001	38108	None	chr14:69811822-69811836,chr14:69811
NA	NA	NA		None	None
NA	NA	NA		None	chr15:51360221-51360224,chr15:51360
AC022254.1	ENSG000002	37626	None	None
NA	NA	NA		None	None
NA	NA	NA		None	None
NA	NA	NA		None	None
NA	NA	NA		None	chr16:84855483-84855489
NA	NA	NA		None	None
NA	NA	NA		None	chr17:31762435-31762442,chr17:31769
RN7SL507P	ENSG000002	1636	None	None
POLRMTP1	ENSG000002	37878	chr17:62178(	chr17:62179765-62179772
AC015688.1	ENSG000002	1955	None	None
NA	NA	NA		None	chr17:8056882-8056886
NA	NA	NA		None	chr17:80397675-80397680
NA	NA	NA		None	None
NA	NA	NA		None	None
PTPN2	ENSG000001	5236	None	None
NA	NA	NA		None	chr18:39800266-39800319
NA	NA	NA		None	None
RNU6-340P	ENSG000002	19234	chr18:26279(	None
RPL9P31	ENSG000002	9102	None	None
PSTPIP2	ENSG000001	1039	None	None
NA	NA	NA		None	None
NA	NA	NA		None	None
NA	NA	NA		None	chr19:23549663-23549703,chr19:23549
TINCR	ENSG000002	4955	None	chr19:5551290-5551321
CYP4F23P	ENSG000002	19434	None	None
AC123912.3	ENSG000002	15829	None	None
NA	NA	NA		None	None
AC245128.3	ENSG000002	2877	None	None
MTDHP3	ENSG000002	9979	None	None

NA	NA	NA		None	None
COX7A1	ENSG000001		8580	None	None
NA	NA	NA		chr19:15833!	chr19:15828985-15829004,chr19:15829
NA	NA	NA		None	None
AC008164.1	ENSG000002		22378	None	None
NA	NA	NA		None	None
RNU7-190P	ENSG000002		4637	None	None
NA	NA	NA		None	None
NA	NA	NA		None	None
NA	NA	NA		None	None
NA	NA	NA		chr2:371317(	None
NA	NA	NA		None	chr2:118952981-118952985
NA	NA	NA		None	None
NA	NA	NA		None	chr2:162329084-162329086
DARS-AS1	ENSG000002		49225	None	None
NA	NA	NA		None	None
NA	NA	NA		chr2:102013!	chr2:10201360-10201365,chr2:1020140
NA	NA	NA		chr2:218046:	None
NA	NA	NA		None	None
NA	NA	NA		None	chr2:11399039-11399057
NA	NA	NA		None	None
NA	NA	NA		None	None
RUFY4	ENSG000001		18111	None	None
NA	NA	NA		None	chr2:126646167-126646169
RUFY4	ENSG000001		26426	None	None
CXCR2	ENSG000001		8283	None	None
NA	NA	NA		None	None
NA	NA	NA		None	chr2:64252792-64252794
DEFB128	ENSG000001		9000	None	None
FAM110A	ENSG000001		32770	None	chr20:792169-792179
NA	NA	NA		None	chr20:46433270-46433280,chr20:46433
NA	NA	NA		None	chr20:44671726-44671763,chr20:44674
NA	NA	NA		None	None
NA	NA	NA		None	None
NA	NA	NA		None	None
NA	NA	NA		None	None
NA	NA	NA		chr21:38228:	chr21:38224363-38224367,chr21:38229
NA	NA	NA		None	chr22:35663531-35663539
AL080243.3	ENSG000002		15757	None	None
AL049748.1	ENSG000002		12074	None	None
NA	NA	NA		chr3:4642088	chr3:46423626-46423645,chr3:4642366
NA	NA	NA		None	None
HESX1	ENSG000001		18560	None	None

LTF	ENSG00000C		2015	None	chr3:46426843-46426848
LIPH	ENSG000001		9960	None	None
LINC02009	ENSG000002		209	None	None
RTP4	ENSG000001		2035	None	None
AC117434.1	ENSG000002		207082	None	chr3:176327058-176327066
NA	NA	NA		None	None
NA	NA	NA		None	chr3:157583528-157583573
AC069528.1	ENSG000002		31900	None	None
ALG1L2	ENSG000002		16857	None	None
LINC02070	ENSG000002		40073	None	None
CRIP1P1	ENSG000002		32489	None	None
AC092924.1	ENSG000002		44993	None	None
NA	NA	NA		None	None
NA	NA	NA		None	None
NA	NA	NA		None	None
NA	NA	NA		None	None
NDUFA4P2	ENSG000002		25012	None	None
NA	NA	NA		chr3:463889:	None
NA	NA	NA		None	None
NA	NA	NA		None	None
NA	NA	NA		None	chr3:32074319-32074329
NA	NA	NA		None	None
NA	NA	NA		None	None
KRT19P6	ENSG000002		255347	None	None
AC095050.1	ENSG000002		41138	None	chr4:175451471-175451508
NA	NA	NA		None	None
LINC02503	ENSG000002		14587	None	None
AC078850.2	ENSG000002		39487	None	None
NA	NA	NA		None	None
FTH1P21	ENSG000002		96934	None	None
NA	NA	NA		None	None
DOK7	ENSG000001		2922	None	None
NA	NA	NA		None	None
NA	NA	NA		chr4:884473(	None
NA	NA	NA		None	None
AC112236.1	ENSG000002		16362	None	None
NA	NA	NA		None	None
NA	NA	NA		None	None
MGST2	ENSG00000C		26723	None	None
NA	NA	NA		None	None
SORCS2	ENSG000001		8131	None	None
LINC02264	ENSG000002		27925	None	None
NDST3	ENSG000001		133496	None	None

AC026700.1	ENSG000002	97050	None	chr5:85647755-85647773,chr5:8564781
GABRG2	ENSG000001	112451	chr5:162280(	None
AC106821.1	ENSG000001	99402	None	None
AC026736.1	ENSG000002	167641	None	None
NA	NA	NA	None	None
NA	NA	NA	None	None
RNA5SP190	ENSG000002	6547	None	None
AC136604.3	ENSG000002	3244	None	None
NA	NA	NA	None	None
NA	NA	NA	None	None
NA	NA	NA	None	None
AL355375.2	ENSG000002	19429	None	None
RNU2-8P	ENSG000002	39901	None	chr6:121625951-121625954
NA	NA	NA	None	None
TNFRSF21	ENSG000001	2787	None	None
NRN1	ENSG000001	20205	None	chr6:6032314-6032327
NUFIP1P1	ENSG000002	466987	None	None
AL512430.3	ENSG000002	56107	None	None
AL049860.1	ENSG000002	242164	None	None
AL078602.1	ENSG000002	107159	None	None
AL031767.1	ENSG000002	82	None	None
AL512430.3	ENSG000002	70976	None	None
AL450336.1	ENSG000002	445385	None	chr6:66259048-66259055,chr6:6625929
NA	NA	NA	None	None
NA	NA	NA	None	None
NA	NA	NA	None	None
NA	NA	NA	None	chr6:16263613-16263637,chr6:1626400
AL008628.1	ENSG000002	7243	None	None
H4C8	ENSG000001	2384	None	None
NA	NA	NA	None	None
NA	NA	NA	None	None
H3C8	ENSG000002	8944	None	chr6:26257493-26257505,chr6:2625752
NEPNP	ENSG000002	13710	None	None
GPR6	ENSG000001	95479	None	None
MICD	ENSG000002	435	chr6:299649:	chr6:29965020-29965040,chr6:2996506
AL031123.1	ENSG000002	1057	None	None
HLA-W	ENSG000002	6184	None	None
LINC01449	ENSG000002	413547	None	None
LINC01449	ENSG000002	419782	None	None
AC006974.2	ENSG000002	3793	chr7:148579	chr7:148579470-148579478
NA	NA	NA	None	None
TPM3P4	ENSG000002	21292	None	None
PTMAP10	ENSG000002	35896	None	None

GRB10	ENSG000001	6109	None	chr7:50579334-50579337
AC006970.1	ENSG000002	9694	None	None
NA	NA	NA	None	None
NUP205	ENSG000001	31159	None	None
NA	NA	NA	None	None
NA	NA	NA	None	None
CDCA7L	ENSG000001	153179	None	None
NA	NA	NA	None	None
NA	NA	NA	None	None
HEPACAM2	ENSG000001	34473	None	None
NA	NA	NA	None	None
NA	NA	NA	None	chr7:12589342-12589351,chr7:1258853
NA	NA	NA	None	None
SAMD9L	ENSG000001	701	None	None
WDR91	ENSG000001	3288	chr7:135177	None
NA	NA	NA	chr7:135174	chr7:135171872-135171886,chr7:13517
NA	NA	NA	None	None
NA	NA	NA	None	None
NA	NA	NA	None	chr7:106460712-106460751
AC078846.1	ENSG000002	21461	None	None
NA	NA	NA	None	None
AC015522.1	ENSG000002	362045	None	None
NA	NA	NA	chr8:534836	chr8:53483949-53483990
NA	NA	NA	None	None
AC079209.1	ENSG000002	77197	None	None
NA	NA	NA	None	chr8:17910357-17910383,chr8:1791588
AC025857.1	ENSG000002	10247	None	None
GPIHBP1	ENSG000002	16353	None	None
SMARCE1P4	ENSG000002	105484	None	None
AC090578.1	ENSG000002	346344	None	None
RPSAP74	ENSG000002	87022	None	None
AF117829.1	ENSG000002	130613	None	None
AC090578.1	ENSG000002	321106	None	None
AL353615.1	ENSG000002	23902	None	None
RPS20P25	ENSG000002	276022	None	None
ARL2BPP7	ENSG000002	35075	None	None
HMGB3P23	ENSG000002	519204	None	None
AL138749.1	ENSG000002	129090	None	None
AL391056.1	ENSG000002	3611	None	None
RNU6-918P	ENSG000002	9648	None	None
NA	NA	NA	None	None
SLC25A5P8	ENSG000002	36990	None	None
NA	NA	NA	None	None

NA	NA	NA		chr9:1050559	chr9:105056239-105056243,chr9:10505
NA	NA	NA		None	None
NA	NA	NA		None	None
AL354989.2	ENSG000002		23717	chr9:3415849	None
NA	NA	NA		None	None
AL162388.2	ENSG000002		83107	None	None
AL136981.3	ENSG000002		22239	None	None
NA	NA	NA		None	None
TPT1P13	ENSG000002		226949	None	None
NA	NA	NA		None	None
FIRRE	ENSG000002		75228	None	None
MIR3672	ENSG000002		167334	None	None
AL441985.1	ENSG000002		101701	None	None
CHMP1B2P	ENSG000002		180217	None	None
NA	NA	NA		None	None
NA	NA	NA		None	None
TPT1P13	ENSG000002		407970	None	None
Z96074.1	ENSG000002		282578	None	None
NA	NA	NA		None	None
NA	NA	NA		None	None
NA	NA	NA		None	chrX:37462820-37462830

0453-247360458,chr1:247360182-247360201,chr1:247360228-247360239

)94226

5089-157275108,chr1:157275128-157275140,chr1:157279404-157279413

-4021119

268986-109269006

846-69811853

232-51360246

237-31769243,chr17:31762590-31762597

859-23549887,chr19:23549900-23549909,chr19:23550355-23550374

010-15829025,chr19:15829031-15829050,chr19:15829153-15829171,chr19:15829222-158292

5-10201408,chr2:10201467-10201478

290-46433297,chr20:46433368-46433381,chr20:46433390-46433405,chr20:46433576-464335
007-44674011

723-38229734,chr21:38229786-38229822,chr21:38228619-38228622,chr21:38229593-382296

1-46423672,chr3:46423588-46423598

2-85647827

9-66259314,chr6:66259323-66259347,chr6:66259361-66259366

8-16264014

6-26257527

6-29965081,chr6:29964964-29964986,chr6:29966121-29966133

6-12588543,chr7:12588953-12588961,chr7:12589047-12589059,chr7:12590836-12590843,chr

2468-135172470

8-17915911,chr8:17915979-17915993,chr8:17916001-17916013,chr8:17916077-17916083,chr

6132-105056139

235,chr19:15828931-15828941,chr19:15828970-15828981

686,chr20:46433594-46433626,chr20:46433637-46433649,chr20:46433651-46433661,chr20:46

630

ʳ7:12590937-12590940,chr7:12591400-12591409,chr7:12591411-12591422,chr7:12591655-12

ʳ8:17916265-17916275

[illegible]433002-46433007,chr20:46433056-46433070,chr20:46433093-46433104,chr20:46433107-46[illegible]

:591664,chr7:12591881-12591891,chr7:12592413-12592429,chr7:12592549-12592574,chr7:12

433124,chr20:46433129-46433132,chr20:46433210-46433219

2592637-12592640,chr7:12592646-12592649,chr7:12592753-12592764,chr7:12592767-12592[illegible]

780,chr7:12593085-12593095,chr7:12594512-12594515,chr7:12594572-12594578,chr7:12594[illegible]

629-12594635,chr7:12595189-12595228,chr7:12595229-12595238,chr7:12594556-12594566

Locus	log2FoldChar	padj	Class	Family	Category	Chrom
ERV316A3_1	4.53069971	5.41E-24	HERV	ERV316A3	internal	chr12
HERVH_4q22	4.10443288	3.33E-07	HERV	HERVH	oneside	chr4
HUERSP3_2p	3.71072402	2.09E-19	HERV	HUERSP3	internal	chr2
HARLEQUIN_	3.53737511	1.11E-05	HERV	HARLEQUIN	oneside	chr17
HML5_14q32	3.43739877	0.04439933	HERV	HML5	prototype	chr14
HML3_4q28.:	3.0741428	0.01366527	HERV	HML3	internal	chr4
ERV316A3_X	2.90742125	5.05E-13	HERV	ERV316A3	oneside	chrX
HERV3_19p1	2.88437387	0.00794011	HERV	HERV3	prototype	chr19
HERVL_11q1	2.85308002	1.99E-05	HERV	HERVL	oneside	chr11
ERV316A3_3	2.84323469	1.27E-13	HERV	ERV316A3	oneside	chr3
MER101_2p2	2.78069825	7.59E-16	HERV	MER101	oneside	chr2
HERVFH19_2	2.6393664	7.59E-16	HERV	HERVFH19	oneside	chr2
HERVH_1p32	2.60088967	7.21E-05	HERV	HERVH	prototype	chr1
HERVL_1p22	2.59976653	0.00013694	HERV	HERVL	oneside	chr1
HML2_15q25	2.59867175	0.00932584	HERV	HML2	internal	chr15
HERVP71A_1	2.55410722	9.91E-06	HERV	HERVP71A	prototype	chr1
ERVLE_8q12.	2.54299831	0.00053745	HERV	ERVLE	internal	chr8
MER41_6p12	2.53939219	0.00550707	HERV	MER41	oneside	chr6
PABLB_8q24	2.4948627	1.61E-05	HERV	PABLB	internal	chr8
ERVLB4_11p	2.46173929	8.00E-05	HERV	ERVLB4	internal	chr11
HUERSP2_11	2.44654852	0.00150894	HERV	HUERSP2	prototype	chr11
HML6_3p21.:	2.42515257	3.33E-07	HERV	HML6	prototype	chr3
HERVFH19_1	2.39627784	0.00404456	HERV	HERVFH19	oneside	chr1
HML3_6p25.	2.36821285	0.01043028	HERV	HML3	oneside	chr6
HERVH_13q1	2.36795249	1.88E-05	HERV	HERVH	prototype	chr13
ERVLE_7q22.	2.36441405	0.00249464	HERV	ERVLE	internal	chr7
HUERSP2_2c	2.3520363	3.71E-14	HERV	HUERSP2	prototype	chr2
HERVH_7q34	2.31490323	0.00234386	HERV	HERVH	prototype	chr7
ERVLE_1p36.	2.29514145	0.0465174	HERV	ERVLE	internal	chr1
ERVLE_5q22.	2.17020894	0.00861311	HERV	ERVLE	internal	chr5
ERV316A3_1	2.1544372	0.00932584	HERV	ERV316A3	oneside	chr1
HERVL_7q36	2.1444005	0.01508642	HERV	HERVL	internal	chr7
HERVL18_3p	2.11649519	1.61E-05	HERV	HERVL18	oneside	chr3
HERVH_17q2	2.08436917	4.12E-05	HERV	HERVH	prototype	chr17
PABLA_6p12	2.07523581	0.0014148	HERV	PABLA	internal	chr6
ERV316A3_3	2.07495771	0.01283044	HERV	ERV316A3	oneside	chr3
HERV4_4q22	2.06545689	7.38E-11	HERV	HERV4	prototype	chr4
HERVL18_3p	2.03275144	2.10E-05	HERV	HERVL18	oneside	chr3
MER101_1p2	1.97365967	0.00165279	HERV	MER101	oneside	chr1
MER101_9q3	1.89281256	0.00152552	HERV	MER101	internal	chr9
HERVH_3p21	1.88215921	1.29E-07	HERV	HERVH	prototype	chr3
HML3_4q31.:	1.84744275	8.00E-06	HERV	HML3	prototype	chr4

HARLEQUIN_	1.83562068	8.11E-06	HERV	HARLEQUIN	oneside	chr17
HARLEQUIN_	1.81667787	0.01257176	HERV	HARLEQUIN	prototype	chr2
LTR25_2q12.	1.80485716	7.68E-05	HERV	LTR25	internal	chr2
ERVLB4_3p2	1.79819652	0.04483878	HERV	ERVLB4	oneside	chr3
ERV316A3_2	1.79351782	0.00018562	HERV	ERV316A3	oneside	chr21
HML1_1q23.:	1.79196144	0.02663856	HERV	HML1	internal	chr1
ERVLB4_3p2	1.75762153	0.00034864	HERV	ERVLB4	internal	chr3
MER34B_11p	1.74777913	3.22E-05	HERV	MER34B	unknown	chr11
MER41_7q22	1.733213	3.49E-07	HERV	MER41	prototype	chr7
MER4_4q25b	1.70106432	0.04930606	HERV	MER4	oneside	chr4
HERVH_7q22	1.67182696	0.00028859	HERV	HERVH	prototype	chr7
ERVLB4_12q	1.65095791	0.0114867	HERV	ERVLB4	internal	chr12
HERVH_1p33	1.59729084	0.00300361	HERV	HERVH	prototype	chr1
HUERSP1_11	1.57277456	0.01031666	HERV	HUERSP1	oneside	chr11
ERVLB4_17q	1.56843672	1.04E-06	HERV	ERVLB4	internal	chr17
HERVH48_1c	1.55703136	2.73E-05	HERV	HERVH48	prototype	chr1
HERVFH19_1	1.55070525	0.01014057	HERV	HERVFH19	oneside	chr1
MER41_19q1	1.49326593	0.02536324	HERV	MER41	internal	chr19
ERVLE_7q34	1.49109205	0.01369621	HERV	ERVLE	internal	chr7
MER4_12q12	1.48880345	5.65E-07	HERV	MER4	oneside	chr12
ERV316A3_1	1.48842298	2.77E-06	HERV	ERV316A3	oneside	chr18
MER4_12q12	1.48690842	0.00028859	HERV	MER4	oneside	chr12
HML6_3p21.:	1.4817927	0.01464502	HERV	HML6	prototype	chr3
ERVLE_2q24.	1.47469037	0.00292788	HERV	ERVLE	internal	chr2
ERVLB4_8p2	1.47255862	0.0145723	HERV	ERVLB4	internal	chr8
HERVH_19q1	1.46636894	4.28E-06	HERV	HERVH	prototype	chr19
MER101_2q3	1.44642555	0.03585766	HERV	MER101	oneside	chr2
ERVLB4_17q	1.44538801	0.00053745	HERV	ERVLB4	internal	chr17
HERVFH19_1	1.43352017	0.00034067	HERV	HERVFH19	oneside	chr12
HERVI_18q2	1.42136422	0.02763671	HERV	HERVI	prototype	chr18
HERVH_2q37	1.40972041	0.00546625	HERV	HERVH	prototype	chr2
ERV316A3_8	1.4038861	0.03668967	HERV	ERV316A3	internal	chr8
ERVLE_20q1	1.39866844	0.0112322	HERV	ERVLE	oneside	chr20
MER41_12p1	1.39525971	0.0121835	HERV	MER41	prototype	chr12
ERVLB4_4q3	1.39359909	5.13E-05	HERV	ERVLB4	oneside	chr4
MER41_19q1	1.39327109	0.00021414	HERV	MER41	oneside	chr19
MER61_1q23	1.38248191	2.42E-06	HERV	MER61	prototype	chr1
HERVL_9q21	1.33312611	0.04439933	HERV	HERVL	prototype	chr9
ERV316A3_6	1.31615172	0.00135347	HERV	ERV316A3	oneside	chr6
ERVLE_7q11.	1.31599752	0.01977539	HERV	ERVLE	oneside	chr7
MER4_12q12	1.31544941	4.42E-07	HERV	MER4	oneside	chr12
MER4_Xp22.	1.31223185	0.00660277	HERV	MER4	oneside	chrX
ERVLE_17q1	1.29763015	0.01970851	HERV	ERVLE	internal	chr17

HML5_12p13	1.28880899	0.00200794	HERV	HML5	internal	chr12
ERVLB4_11p	1.27215031	0.00932584	HERV	ERVLB4	internal	chr11
ERV316A3_7	1.26395727	0.00043494	HERV	ERV316A3	oneside	chr7
HERVW_2q1	1.25690485	3.33E-07	HERV	HERVW	prototype	chr2
HERVW_3q2	1.25572478	0.00018562	HERV	HERVW	oneside	chr3
MER4_12q12	1.25497395	0.00100121	HERV	MER4	internal	chr12
PRIMA41_8q	1.23535692	0.00186353	HERV	PRIMA41	internal	chr8
PABLA_2p25	1.22203453	0.00062896	HERV	PABLA	internal	chr2
MER4_7q21.	1.22076375	5.56E-05	HERV	MER4	oneside	chr7
HML6_6p22.:	1.22034789	0.00300361	HERV	HML6	prototype	chr6
HERVH_17q2	1.22010079	6.08E-10	HERV	HERVH	prototype	chr17
MER4B_19q1	1.21866132	0.00028859	HERV	MER4B	internal	chr19
HERVIP10FH_	1.20951963	3.33E-07	HERV	HERVIP10FH	oneside	chr1
HERVK11D_2	1.20949108	5.56E-05	HERV	HERVK11D	prototype	chr2
MER4_12q12	1.20484773	0.00047403	HERV	MER4	prototype	chr12
HERVL_16q2	1.20015774	0.00071909	HERV	HERVL	prototype	chr16
ERVL_16p12.	1.19580828	9.16E-06	HERV	ERVL	oneside	chr16
MER34B_4q3	1.19038629	0.00174106	HERV	MER34B	internal	chr4
HARLEQUIN_	1.18807469	2.77E-06	HERV	HARLEQUIN	oneside	chr7
HERVH_10q2	1.16592292	3.52E-05	HERV	HERVH	prototype	chr10
HML3_15q26	1.16467433	0.0015298	HERV	HML3	prototype	chr15
HERVL_5q33	1.16318032	0.0118904	HERV	HERVL	prototype	chr5
HML2_10q24	1.1627862	0.00425073	HERV	HML2	prototype	chr10
LTR25_1p22.	1.15861952	0.00126972	HERV	LTR25	internal	chr1
MER4_2q14.	1.15026091	0.0422917	HERV	MER4	oneside	chr2
HERVP71A_6	1.14966661	1.55E-05	HERV	HERVP71A	oneside	chr6
HML2_3q13.:	1.12668564	0.02536324	HERV	HML2	prototype	chr3
ERVLE_1q32.	1.12516568	0.00017791	HERV	ERVLE	internal	chr1
LTR23_9q22.	1.12494586	1.61E-05	HERV	LTR23	oneside	chr9
HERVIP10FH_	1.1181135	0.01784703	HERV	HERVIP10FH	mergeable*	chr7
HERV4_12p1	1.11460835	7.49E-06	HERV	HERV4	oneside	chr12
ERVLE_10q2!	1.1034062	0.02847309	HERV	ERVLE	internal	chr10
HERVE_12p1	1.09694453	0.01366925	HERV	HERVE	internal	chr12
HERVH48_1p	1.09660115	0.00852498	HERV	HERVH48	oneside	chr1
ERVLB4_1q2	1.09485364	0.04247046	HERV	ERVLB4	prototype	chr1
ERV316A3_9	1.09218657	0.02370446	HERV	ERV316A3	oneside	chr9
HML2_1q32.:	1.091204	0.01858677	HERV	HML2	oneside	chr1
HERVIP10FH_	1.08571551	0.00097323	HERV	HERVIP10FH	oneside	chr4
HERVL_11q1	1.08028586	0.00892899	HERV	HERVL	prototype	chr11
HERV3_21q2	1.06494783	0.00445981	HERV	HERV3	prototype	chr21
ERVLE_7q34.	1.05870079	0.00071732	HERV	ERVLE	oneside	chr7
HERVL_12p1	1.05682345	0.03973029	HERV	HERVL	oneside	chr12
HERV9_2q35	1.05211964	0.00200794	HERV	HERV9	oneside	chr2

MER4_5q35.	1.04666598	0.00035806	HERV	MER4	oneside	chr5
HML1_11q12	1.03304013	0.0065271	HERV	HML1	prototype	chr11
HERVH_4q31	1.03064428	0.03430942	HERV	HERVH	prototype	chr4
HERVIP10FH_	1.0276367	0.00932584	HERV	HERVIP10FH	oneside	chrX
HERVH_6q12	1.00580614	0.01732217	HERV	HERVH	prototype	chr6
ERVLB4_5q2	-1.0182271	0.04421439	HERV	ERVLB4	internal	chr5
HERVE_11q1	-1.0314616	0.00609724	HERV	HERVE	oneside	chr11
MER41_11q2	-1.0519427	0.03273307	HERV	MER41	oneside	chr11
HERVH_18q2	-1.0535804	0.01577279	HERV	HERVH	prototype	chr18
LTR25_4q34.	-1.0655764	0.00354237	HERV	LTR25	internal	chr4
MER4_17q21	-1.0703942	0.02252542	HERV	MER4	oneside	chr17
HERV4_15q2	-1.1703961	0.00131459	HERV	HERV4	oneside	chr15
HERVFC1_2p	-1.195069	0.03496999	HERV	HERVFC1	prototype	chr2
MER41_9p13	-1.2003762	0.01709953	HERV	MER41	prototype	chr9
HERVS71_4p	-1.2689803	0.00015982	HERV	HERVS71	prototype	chr4
HERVL_5q12	-1.3084137	0.02631427	HERV	HERVL	oneside	chr5
HERVH_16q2	-1.3167253	0.01575487	HERV	HERVH	tandem*	chr16
HERVL_1p35	-1.3551385	0.00225269	HERV	HERVL	oneside	chr1
MER61_4p16	-1.3675711	5.37E-06	HERV	MER61	prototype	chr4
HERVH_4p16	-1.396804	0.01012063	HERV	HERVH	prototype	chr4
HERVL18_1q	-1.4305437	0.00225269	HERV	HERVL18	prototype	chr1
HERVH_14q3	-1.4416261	3.96E-05	HERV	HERVH	prototype	chr14
HERVE_11q1	-1.4486694	0.00101068	HERV	HERVE	oneside	chr11
HERVH_19q1	-1.5173804	5.83E-05	HERV	HERVH	prototype	chr19
HERVE_8p23	-1.5361966	0.01378813	HERV	HERVE	oneside	chr8
HERVW_2p2	-1.5871944	0.00256279	HERV	HERVW	prototype	chr2
HERVH_11q1	-1.5941273	0.00387157	HERV	HERVH	oneside	chr11
MER4_22q13	-1.5946501	0.00014012	HERV	MER4	prototype	chr22
HERVS71_11	-1.6058692	0.00550707	HERV	HERVS71	prototype	chr11
HUERSP2_4q	-1.7921707	0.002959	HERV	HUERSP2	prototype	chr4
HERVH_10q2	-1.9021485	0.00125646	HERV	HERVH	prototype	chr10
ERV316A3_1	-2.1890506	4.28E-06	HERV	ERV316A3	oneside	chr15

Start	End	Strand	TE_CODING	IntersectedG	IntersectedG	IntersectedO
112974379	112975753	+	noncoding	AC004551.1	lncRNA	ANTISENSE
88344162	88349212	-	noncoding	None	NA	NA
6838364	6846887	-	noncoding	NRIR,CMPK2	lncRNA,prote	SENSE,SENS
80408187	80413516	+	noncoding	RNF213-AS1	lncRNA	ANTISENSE
106744465	106749566	+	noncoding	None	NA	NA
130941760	130945512	+	noncoding	None	NA	NA
23748936	23751401	-	noncoding	ACOT9	protein_codi	SENSE
22524807	22531806	+	noncoding	ZNF98,AC01	protein_codi	ANTISENSE,A
57650629	57651896	+	noncoding	AP000662.1,	lncRNA,prote	SENSE,SENS
187374111	187378966	-	noncoding	None	NA	NA
6869773	6876489	+	noncoding	RSAD2	protein_codi	SENSE
37128668	37135454	+	noncoding	EIF2AK2	protein_codi	ANTISENSE
55022708	55028369	+	noncoding	None	NA	NA
89437349	89443684	-	noncoding	AL596214.1	lncRNA	SENSE
84160268	84163612	+	noncoding	None	NA	NA
247354022	247360572	+	coding	None	NA	NA
64811247	64815461	-	noncoding	AC104232.3,	lncRNA,lncRI	ANTISENSE,S
49721416	49722594	-	noncoding	AL121974.1,	lncRNA,TEC	ANTISENSE,A
143062997	143065669	-	coding	None	NA	NA
3582916	3583518	+	noncoding	None	NA	NA
4550544	4554796	+	noncoding	None	NA	NA
46426543	46433630	+	noncoding	None	NA	NA
9086800	9088393	+	noncoding	SLC2A5	protein_codi	ANTISENSE
6064962	6068594	-	noncoding	None	NA	NA
42868004	42873158	-	noncoding	None	NA	NA
106548063	106548761	-	noncoding	AC004917.1	lncRNA	SENSE
162324003	162329299	-	noncoding	GCA	protein_codi	ANTISENSE
143105117	143110504	+	coding	None	NA	NA
27070419	27074352	+	noncoding	None	NA	NA
112547505	112549086	+	noncoding	AC137549.1	processed_p	SENSE
153470032	153474206	-	noncoding	None	NA	NA
148575970	148579927	-	noncoding	None	NA	NA
46419310	46423900	-	coding	LINC02009	lncRNA	SENSE
80399794	80408186	+	coding	RNF213-AS1	lncRNA	ANTISENSE
49717615	49721439	-	noncoding	AL121974.1	lncRNA	ANTISENSE
183114642	183118015	-	noncoding	MCCC1	protein_codi	SENSE
88442739	88453401	+	noncoding	HERC6	protein_codi	SENSE
46417556	46419247	-	noncoding	LINC02009	lncRNA	SENSE
89429509	89431206	-	noncoding	AL596214.1	lncRNA	SENSE
105055371	105056952	+	noncoding	AL591506.1	lncRNA	ANTISENSE
46387685	46393402	-	noncoding	CCR5AS	lncRNA	SENSE
153688368	153694427	-	noncoding	TLR2	protein_codi	ANTISENSE

80397328	80399793	+	noncoding	RNF213,RNF	protein_codi	SENSE,ANTIS
13525956	13533609	-	coding	None	NA	NA
102408621	102413726	+	coding	SDR42E1P5	processed_p	SENSE
46057514	46059437	+	noncoding	None	NA	NA
42598743	42600906	-	noncoding	LINC01671	lncRNA	SENSE
161489309	161491458	+	noncoding	None	NA	NA
46414268	46416315	+	noncoding	None	NA	NA
339634	342458	-	noncoding	None	NA	NA
106455435	106461177	-	noncoding	AC004917.1,	lncRNA,lncRI	SENSE,ANTIS
107886507	107892905	-	noncoding	SGMS2,AC09	protein_codi	ANTISENSE,S
106527752	106533658	-	noncoding	AC004917.1	lncRNA	SENSE
55642877	55643624	+	noncoding	AC009779.6	lncRNA	ANTISENSE
48209002	48214821	-	noncoding	None	NA	NA
6039059	6042072	+	noncoding	None	NA	NA
80265360	80268548	+	noncoding	RNF213	protein_codi	SENSE
247286141	247290862	+	noncoding	None	NA	NA
9088458	9096029	+	noncoding	SLC2A5	protein_codi	ANTISENSE
42492710	42494666	-	noncoding	LIPE-AS1	lncRNA	ANTISENSE
143154803	143160197	-	noncoding	None	NA	NA
40160628	40165076	-	noncoding	LINC02471	lncRNA	ANTISENSE
46275058	46278556	+	noncoding	None	NA	NA
40193379	40194785	-	noncoding	LINC02471,LI	lncRNA,lncRI	ANTISENSE,S
46046154	46054474	-	noncoding	None	NA	NA
165779881	165783164	-	noncoding	GALNT3	protein_codi	SENSE
23336642	23337699	-	noncoding	LOXL2,AC09C	protein_codi	SENSE,ANTIS
54894297	54900597	-	noncoding	None	NA	NA
196851115	196854775	+	noncoding	PGAP1	protein_codi	ANTISENSE
36131939	36132906	+	noncoding	AC243829.2	lncRNA	SENSE
31906332	31909833	+	noncoding	LINC02422	lncRNA	ANTISENSE
46868193	46877872	+	noncoding	PIAS2	protein_codi	ANTISENSE
233214800	233217821	-	noncoding	ATG16L1	protein_codi	ANTISENSE
89987046	89989040	-	noncoding	NBN	protein_codi	SENSE
40488907	40491323	-	noncoding	None	NA	NA
31847621	31852561	+	noncoding	None	NA	NA
168434326	168439585	+	noncoding	DDX60L	protein_codi	ANTISENSE
42487086	42492400	-	noncoding	LIPE-AS1	lncRNA	ANTISENSE
159056048	159059052	-	noncoding	None	NA	NA
85775592	85779684	-	noncoding	AL353743.4	unprocessed	ANTISENSE
29946920	29950412	+	noncoding	None	NA	NA
66304013	66309489	-	noncoding	TPST1	protein_codi	ANTISENSE
40187163	40191467	-	noncoding	LINC02471,LI	lncRNA,lncRI	ANTISENSE,S
12935112	12937470	-	noncoding	None	NA	NA
36143059	36144653	+	noncoding	AC243829.2	lncRNA	SENSE

8558451	8562470	-	noncoding	AC092746.1	lncRNA	SENSE
5649261	5651245	-	noncoding	AC104389.5	protein_codi	SENSE
106475939	106477621	+	noncoding	AC004917.1,.	lncRNA,lncRI	ANTISENSE,S
96217144	96223328	-	noncoding	STARD7-AS1	lncRNA	ANTISENSE
142438179	142443654	+	noncoding	XRN1	protein_codi	ANTISENSE
40191686	40193336	-	noncoding	LINC02471,LI	lncRNA,lncRI	ANTISENSE,S
143050063	143053296	+	noncoding	C8orf31	lncRNA	SENSE
10199041	10202321	-	noncoding	RRM2	protein_codi	ANTISENSE
93127516	93129355	+	noncoding	None	NA	NA
26288022	26296266	-	noncoding	None	NA	NA
75252621	75258281	+	noncoding	GGA3	protein_codi	ANTISENSE
54618131	54620746	+	noncoding	LILRB1	protein_codi	SENSE
159022297	159025513	-	noncoding	IFI16	protein_codi	ANTISENSE
101237832	101245098	+	coding	TBC1D8	protein_codi	ANTISENSE
40210828	40213880	+	noncoding	LINC02471,LI	lncRNA,lncRI	SENSE,ANTIS
84855240	84861709	-	noncoding	AC025280.3,(	lncRNA,prote	SENSE,ANTIS
23992260	23998394	+	noncoding	PRKCB	protein_codi	SENSE
153696581	153699612	+	noncoding	TLR2	protein_codi	SENSE
135171264	135175958	+	coding	AC083862.3	lncRNA	SENSE
89284720	89290887	+	noncoding	LIPA,IFIT2,AL	protein_codi	ANTISENSE,S
89233714	89240303	-	noncoding	None	NA	NA
159233488	159238427	-	noncoding	AC134043.2,I	lncRNA,lncRI	ANTISENSE,A
99820812	99827988	-	coding	ABCC2	protein_codi	ANTISENSE
84603880	84611675	+	noncoding	AL359762.2	lncRNA	SENSE
112857065	112863541	+	noncoding	None	NA	NA
29962489	29970366	+	noncoding	None	NA	NA
113024277	113033435	-	coding	AC078785.1	lncRNA	ANTISENSE
207512891	207513462	-	noncoding	CR1	protein_codi	ANTISENSE
98116034	98117804	+	noncoding	TRIM14,AL13	protein_codi	ANTISENSE,S
43812642	43823897	+	noncoding	None	NA	NA
31936771	31945711	-	noncoding	LINC02422	lncRNA	SENSE
114802955	114807340	+	noncoding	None	NA	NA
8441913	8443743	-	noncoding	None	NA	NA
86339370	86343589	+	noncoding	None	NA	NA
173333146	173340404	+	noncoding	None	NA	NA
34158413	34168326	-	noncoding	None	NA	NA
207635112	207639291	-	noncoding	CR1	protein_codi	ANTISENSE
15693941	15695719	-	noncoding	FAM200B	protein_codi	ANTISENSE
60216680	60223953	+	noncoding	MS4A4A,MS4	protein_codi	SENSE,ANTIS
38224043	38229805	-	noncoding	KCNJ15	protein_codi	ANTISENSE
140290502	140295513	-	noncoding	PPP1R2P6,SL	processed_p	ANTISENSE,S
7996848	8005194	+	noncoding	None	NA	NA
218117007	218122161	-	coding	None	NA	NA

180781518	180783272	-		noncoding	None	NA	NA
56327845	56333225	-		coding	FAM8A2P	unprocessed_	SENSE
138927836	138933543	+		noncoding	AC109927.1	lncRNA	ANTISENSE
44999167	45004021	-		noncoding	KDM6A	protein_codi	ANTISENSE
63950227	63956522	+		noncoding	EYS	protein_codi	ANTISENSE
126500832	126502841	-		noncoding	AC093535.1	lncRNA	SENSE
67845158	67853485	-		noncoding	ENPP7P7	unprocessed_	SENSE
118866008	118867768	+		noncoding	None	NA	NA
74293089	74299036	+		noncoding	AC090398.2	lncRNA	SENSE
173282514	173289284	-		noncoding	GALNT7	protein_codi	ANTISENSE
41109018	41116054	+		noncoding	None	NA	NA
55477977	55482210	+		noncoding	DNAAF4-CCP	lncRNA,prote	ANTISENSE,A
83873933	83879494	-		noncoding	None	NA	NA
33703617	33708862	+		noncoding	AL356489.3	lncRNA	SENSE
9599829	9607243	+		coding	AC105916.2	lncRNA	ANTISENSE
61908053	61913030	-		noncoding	AC008877.1	lncRNA	ANTISENSE
70616524	70626119	-		noncoding	IL34	protein_codi	ANTISENSE
31517845	31518912	+		noncoding	LINC01226	lncRNA	SENSE
94966	101337	+		noncoding	None	NA	NA
9638973	9644753	+		noncoding	AC105916.2	lncRNA	ANTISENSE
207420882	207425638	-		noncoding	None	NA	NA
98985205	98992939	+		noncoding	None	NA	NA
71741115	71749426	+		noncoding	AP003498.1,I	lncRNA,trans	ANTISENSE,S
38750366	38755295	+		noncoding	CAPN12	protein_codi	ANTISENSE
8172261	8180584	+		coding	AC105233.5,I	lncRNA,trans	SENSE,SENSI
153396	160782	-		noncoding	None	NA	NA
71749427	71752695	+		noncoding	AP003498.1,I	lncRNA,trans	ANTISENSE,S
47578322	47585857	+		noncoding	None	NA	NA
71705986	71713426	+		coding	AP003498.1,I	lncRNA,trans	ANTISENSE,S
117831374	117836043	-		noncoding	LINC02264	lncRNA	ANTISENSE
90294983	90300722	+		noncoding	None	NA	NA
95814469	95816820	-		noncoding	AC015574.1	lncRNA	ANTISENSE

IntersectedG	TE_type	TE_5UTR_Int	TE_3UTR_Int	ClosestUpstr	ClosestUpstr	DistUpstrean
ENSG000002	INTRONIC	NA	NA	NA	NA	NA
None	INTERGENIC	NA	NA	AC107067.3	ENSG000002	3010
ENSG000002	EXONIC	FALSE	FALSE	NA	NA	NA
ENSG000002	INTRONIC	NA	NA	NA	NA	NA
None	INTERGENIC	NA	NA	IGHV1-69-2	ENSG000002	6918
None	INTERGENIC	NA	NA	AC093831.1	ENSG000002	56119
ENSG000001	INTRONIC	NA	NA	NA	NA	NA
ENSG000001	EXONIC	FALSE	FALSE	NA	NA	NA
ENSG000002	EXONIC	FALSE	FALSE	NA	NA	NA
None	INTERGENIC	NA	NA	AC068299.2	ENSG000002	4135
ENSG000001	INTRONIC	NA	NA	NA	NA	NA
ENSG00000C	INTRONIC	NA	NA	NA	NA	NA
None	INTERGENIC	NA	NA	BSND	ENSG000001	5536
ENSG000002	INTRONIC	NA	NA	NA	NA	NA
None	INTERGENIC	NA	NA	EFL1P1	ENSG000002	33664
None	INTERGENIC	NA	NA	AC104335.2	ENSG000002	5946
ENSG000002	INTRONIC	NA	NA	NA	NA	NA
ENSG000002	EXONIC	FALSE	FALSE	NA	NA	NA
None	INTERGENIC	NA	NA	AC083982.1	ENSG000002	246
None	INTERGENIC	NA	NA	AC127526.2	ENSG000002	1705
None	INTERGENIC	NA	NA	OR52M1	ENSG000001	4400
None	INTERGENIC	NA	NA	LINC02009	ENSG000002	2952
ENSG000001	EXONIC	TRUE	FALSE	NA	NA	NA
None	INTERGENIC	NA	NA	F13A1	ENSG000001	75490
None	INTERGENIC	NA	NA	EPSTI1	ENSG000001	13230
ENSG000002	INTRONIC	NA	NA	NA	NA	NA
ENSG000001	INTRONIC	NA	NA	NA	NA	NA
None	INTERGENIC	NA	NA	OR6W1P	ENSG000001	41532
None	INTERGENIC	NA	NA	TENT5B	ENSG000001	57569
ENSG000002	EXONIC	FALSE	FALSE	NA	NA	NA
None	INTERGENIC	NA	NA	RN7SL44P	ENSG000002	26257
None	INTERGENIC	NA	NA	AC005229.2	ENSG000002	474
ENSG000002	EXONIC	FALSE	FALSE	NA	NA	NA
ENSG000002	INTRONIC	NA	NA	NA	NA	NA
ENSG000002	INTRONIC	NA	NA	NA	NA	NA
ENSG00000C	EXONIC	TRUE	FALSE	NA	NA	NA
ENSG000001	EXONIC	FALSE	TRUE	NA	NA	NA
ENSG000002	EXONIC	FALSE	FALSE	NA	NA	NA
ENSG000002	INTRONIC	NA	NA	NA	NA	NA
ENSG000002	INTRONIC	NA	NA	NA	NA	NA
ENSG000002	EXONIC	FALSE	FALSE	NA	NA	NA
ENSG000001	EXONIC	FALSE	FALSE	NA	NA	NA

ENSG000001	EXONIC	FALSE	TRUE	NA	NA	NA	
None	INTERGENIC	NA	NA	AC073062.1	ENSG000002		4064
ENSG000002	EXONIC	FALSE	FALSE	NA	NA	NA	
None	INTERGENIC	NA	NA	XCR1	ENSG000001		29772
ENSG000002	EXONIC	FALSE	FALSE	NA	NA	NA	
None	INTERGENIC	NA	NA	AL592295.3	ENSG000002		18786
None	INTERGENIC	NA	NA	CCRL2	ENSG000001		1271
None	INTERGENIC	NA	NA	B4GALNT4	ENSG000001		27041
ENSG000002	INTRONIC	NA	NA	NA	NA	NA	
ENSG000001	EXONIC	FALSE	FALSE	NA	NA	NA	
ENSG000002	INTRONIC	NA	NA	NA	NA	NA	
ENSG000002	INTRONIC	NA	NA	NA	NA	NA	
None	INTERGENIC	NA	NA	SLC5A9	ENSG000001		7864
None	INTERGENIC	NA	NA	OR56A1	ENSG000001		4721
ENSG000001	INTRONIC	NA	NA	NA	NA	NA	
None	INTERGENIC	NA	NA	VN1R5	ENSG000001		28931
ENSG000001	EXONIC	TRUE	FALSE	NA	NA	NA	
ENSG000002	INTRONIC	NA	NA	NA	NA	NA	
None	INTERGENIC	NA	NA	TAS2R39	ENSG000002		23222
ENSG000002	INTRONIC	NA	NA	NA	NA	NA	
None	INTERGENIC	NA	NA	C18orf25	ENSG000001		8066
ENSG000002	INTRONIC	NA	NA	NA	NA	NA	
None	INTERGENIC	NA	NA	FLT1P1	ENSG000002		87884
ENSG000001	INTRONIC	NA	NA	NA	NA	NA	
ENSG000001	EXONIC	FALSE	FALSE	NA	NA	NA	
None	INTERGENIC	NA	NA	NCR1	ENSG000001		5551
ENSG000001	INTRONIC	NA	NA	NA	NA	NA	
ENSG000002	INTRONIC	NA	NA	NA	NA	NA	
ENSG000002	INTRONIC	NA	NA	NA	NA	NA	
ENSG000000	INTRONIC	NA	NA	NA	NA	NA	
ENSG000000	INTRONIC	NA	NA	NA	NA	NA	
ENSG000001	INTRONIC	NA	NA	NA	NA	NA	
None	INTERGENIC	NA	NA	MAFB	ENSG000002		194525
None	INTERGENIC	NA	NA	AC023050.2	ENSG000002		36431
ENSG000001	INTRONIC	NA	NA	NA	NA	NA	
ENSG000002	INTRONIC	NA	NA	NA	NA	NA	
None	INTERGENIC	NA	NA	AIM2	ENSG000001		3432
ENSG000002	INTRONIC	NA	NA	NA	NA	NA	
None	INTERGENIC	NA	NA	HLA-A	ENSG000002		1036
ENSG000001	INTRONIC	NA	NA	NA	NA	NA	
ENSG000002	EXONIC	FALSE	FALSE	NA	NA	NA	
None	INTERGENIC	NA	NA	TMSB4X	ENSG000002		37640
ENSG000002	INTRONIC	NA	NA	NA	NA	NA	

ENSG000002	INTRONIC	NA	NA	NA	NA	NA
ENSG000002	INTRONIC	NA	NA	NA	NA	NA
ENSG000002	INTRONIC	NA	NA	NA	NA	NA
ENSG000002	INTRONIC	NA	NA	NA	NA	NA
ENSG000001	INTRONIC	NA	NA	NA	NA	NA
ENSG000002	INTRONIC	NA	NA	NA	NA	NA
ENSG000001	EXONIC	FALSE	FALSE	NA	NA	NA
ENSG000001	EXONIC	FALSE	TRUE	NA	NA	NA
None	INTERGENIC	NA	NA	SAMD9	ENSG000002	9493
None	INTERGENIC	NA	NA	H3C9P	ENSG000002	25610
ENSG000001	INTRONIC	NA	NA	NA	NA	NA
ENSG000001	INTRONIC	NA	NA	NA	NA	NA
ENSG000001	INTRONIC	NA	NA	NA	NA	NA
ENSG000002	INTRONIC	NA	NA	NA	NA	NA
ENSG000002	EXONIC	FALSE	FALSE	NA	NA	NA
ENSG000002	EXONIC	FALSE	FALSE	NA	NA	NA
ENSG000001	INTRONIC	NA	NA	NA	NA	NA
ENSG000001	INTRONIC	NA	NA	NA	NA	NA
ENSG000002	EXONIC	FALSE	FALSE	NA	NA	NA
ENSG000001	EXONIC	TRUE	FALSE	NA	NA	NA
None	INTERGENIC	NA	NA	FANCI	ENSG000001	3646
ENSG000002	INTRONIC	NA	NA	NA	NA	NA
ENSG000000	INTRONIC	NA	NA	NA	NA	NA
ENSG000002	EXONIC	FALSE	FALSE	NA	NA	NA
None	INTERGENIC	NA	NA	XIAPP3	ENSG000001	2943
None	INTERGENIC	NA	NA	HLA-W	ENSG000002	3919
ENSG000002	INTRONIC	NA	NA	NA	NA	NA
ENSG000002	INTRONIC	NA	NA	NA	NA	NA
ENSG000001	EXONIC	FALSE	FALSE	NA	NA	NA
None	INTERGENIC	NA	NA	BLVRA	ENSG000001	5300
ENSG000002	INTRONIC	NA	NA	NA	NA	NA
None	INTERGENIC	NA	NA	AL137025.1	ENSG000002	8611
None	INTERGENIC	NA	NA	CLEC6A	ENSG000002	12219
None	INTERGENIC	NA	NA	LINC02795	ENSG000002	18659
None	INTERGENIC	NA	NA	TNFSF4	ENSG000001	125815
None	INTERGENIC	NA	NA	UBAP1	ENSG000001	10679
ENSG000002	INTRONIC	NA	NA	NA	NA	NA
ENSG000002	INTRONIC	NA	NA	NA	NA	NA
ENSG000001	INTRONIC	NA	NA	NA	NA	NA
ENSG000001	INTRONIC	NA	NA	NA	NA	NA
ENSG000002	EXONIC	FALSE	TRUE	NA	NA	NA
None	INTERGENIC	NA	NA	AC006511.2	ENSG000002	1764
None	INTERGENIC	NA	NA	CXCR2	ENSG000001	3128

None	INTERGENIC	NA	NA	MGAT1	ENSG000001	1510
ENSG000002	EXONIC	FALSE	FALSE	NA	NA	NA
ENSG000002	INTRONIC	NA	NA	NA	NA	NA
ENSG000001	INTRONIC	NA	NA	NA	NA	NA
ENSG000001	INTRONIC	NA	NA	NA	NA	NA
ENSG000002	INTRONIC	NA	NA	NA	NA	NA
ENSG000002	INTRONIC	NA	NA	NA	NA	NA
None	INTERGENIC	NA	NA	AP004609.1	ENSG000002	29897
ENSG000002	EXONIC	FALSE	FALSE	NA	NA	NA
ENSG000001	INTRONIC	NA	NA	NA	NA	NA
None	INTERGENIC	NA	NA	KRTAP4-9	ENSG000002	2530
ENSG000002	INTRONIC	NA	NA	NA	NA	NA
None	INTERGENIC	NA	NA	AC016908.1	ENSG000002	151646
ENSG000002	EXONIC	FALSE	FALSE	NA	NA	NA
ENSG000002	INTRONIC	NA	NA	NA	NA	NA
ENSG000002	INTRONIC	NA	NA	NA	NA	NA
ENSG000001	INTRONIC	NA	NA	NA	NA	NA
ENSG000002	EXONIC	FALSE	FALSE	NA	NA	NA
None	INTERGENIC	NA	NA	ZNF595	ENSG000002	6758
ENSG000002	INTRONIC	NA	NA	NA	NA	NA
None	INTERGENIC	NA	NA	CR2	ENSG000001	28592
None	INTERGENIC	NA	NA	AL162151.2	ENSG000002	11734
ENSG000002	EXONIC	FALSE	FALSE	NA	NA	NA
ENSG000001	INTRONIC	NA	NA	NA	NA	NA
ENSG000002	EXONIC	FALSE	FALSE	NA	NA	NA
None	INTERGENIC	NA	NA	AC079779.1	ENSG000002	36787
ENSG000002	INTRONIC	NA	NA	NA	NA	NA
None	INTERGENIC	NA	NA	LINC01644	ENSG000002	91211
ENSG000002	EXONIC	FALSE	FALSE	NA	NA	NA
ENSG000002	EXONIC	FALSE	FALSE	NA	NA	NA
None	INTERGENIC	NA	NA	LINC01374	ENSG000002	69540
ENSG000002	INTRONIC	NA	NA	NA	NA	NA

ClosestDown	ClosestDown	DistDownstr	FANTOM_en	FANTOM_CAGE
NA	NA	NA	None	None
PPM1K-DT	ENSG000002	12741	None	None
NA	NA	NA	None	None
NA	NA	NA	None	None
AC245369.2	ENSG000002	723	None	None
AC093902.1	ENSG000002	18413	None	None
NA	NA	NA	None	None
NA	NA	NA	None	None
NA	NA	NA	None	None
RTP4	ENSG000001	2035	None	None
NA	NA	NA	None	None
NA	NA	NA	chr2:371317	None
PCSK9	ENSG000001	11078	None	None
NA	NA	NA	None	None
DNM1P41	ENSG000002	2935	None	None
AC104335.3	ENSG000002	29671	chr1:247357	chr1:247359352-247359363,chr1:24736
NA	NA	NA	None	None
NA	NA	NA	None	None
C8orf31	ENSG000001	2313	chr8:143063	None
AP006294.1	ENSG000002	7759	None	None
C11orf40	ENSG000001	16627	None	None
LTF	ENSG000000	2015	None	chr3:46426843-46426848
NA	NA	NA	None	None
NRN1	ENSG000001	57357	None	None
LINC00428	ENSG000002	25117	None	None
NA	NA	NA	None	None
NA	NA	NA	None	chr2:162329084-162329086
PIP	ENSG000001	21573	None	None
SLC9A1	ENSG000000	24457	chr1:270742	None
NA	NA	NA	None	None
S100A7	ENSG000001	9381	None	None
AC006974.2	ENSG000002	3793	chr7:148579	chr7:148579470-148579478
NA	NA	NA	chr3:464208	chr3:46423626-46423645,chr3:4642366
NA	NA	NA	None	None
NA	NA	NA	None	None
NA	NA	NA	None	chr3:183116158-183116202
NA	NA	NA	chr4:884473	None
NA	NA	NA	None	None
NA	NA	NA	None	None
NA	NA	NA	chr9:105055	chr9:105056239-105056243,chr9:10505
NA	NA	NA	chr3:463889	None
NA	NA	NA	None	None

NA	NA	NA	None	chr17:80397675-80397680
AC093912.1	ENSG000002	194273	None	None
NA	NA	NA	None	None
FLT1P1	ENSG000002	82921	None	None
NA	NA	NA	None	None
FCGR2A	ENSG000001	13972	None	None
LINC02009	ENSG000002	209	None	None
AC136475.9	ENSG000002	5946	chr11:34147	None
NA	NA	NA	None	chr7:106460712-106460751
NA	NA	NA	None	None
NA	NA	NA	None	None
NA	NA	NA	None	chr12:55642940-55642962
AL109659.1	ENSG000002	2145	None	None
OR56A7P	ENSG000002	3735	None	chr11:6040290-6040309
NA	NA	NA	None	None
AC104335.1	ENSG000002	3973	None	chr1:247286229-247286235
NA	NA	NA	None	chr1:9088436-9088458,chr1:9094181-9
NA	NA	NA	None	None
PIP	ENSG000001	15064	None	None
NA	NA	NA	None	None
RNF165	ENSG000001	48253	None	None
NA	NA	NA	None	None
XCR1	ENSG000001	18412	None	None
NA	NA	NA	None	None
NA	NA	NA	None	None
AC245128.3	ENSG000002	2877	None	None
NA	NA	NA	None	None
NA	NA	NA	chr17:36132	None
NA	NA	NA	chr12:31906	chr12:31906983-31906986
NA	NA	NA	chr18:46871	chr18:46868644-46868649,chr18:46868
NA	NA	NA	None	None
NA	NA	NA	None	None
AL009050.1	ENSG000002	362550	None	None
AC023050.3	ENSG000002	460	None	None
NA	NA	NA	None	None
NA	NA	NA	None	None
IFI16	ENSG000001	893	None	chr1:159056714-159056718
NA	NA	NA	None	None
HLA-W	ENSG000002	6184	None	None
NA	NA	NA	None	None
NA	NA	NA	None	None
TLR8	ENSG000001	11943	None	None
NA	NA	NA	None	None

NA	NA	NA		None	None
NA	NA	NA		None	None
NA	NA	NA		None	None
NA	NA	NA		None	None
NA	NA	NA		None	None
NA	NA	NA		None	None
NA	NA	NA		None	None
NA	NA	NA		chr2:102013!	chr2:10201360-10201365,chr2:1020140
SAMD9L	ENSG000001		701	None	None
H4C8	ENSG000001		2384	None	None
NA	NA	NA		None	None
NA	NA	NA		None	None
NA	NA	NA		chr1:159024!	chr1:159024957-159024959
NA	NA	NA		None	chr2:101238559-101238560
NA	NA	NA		None	None
NA	NA	NA		None	chr16:84855483-84855489
NA	NA	NA		None	None
NA	NA	NA		None	None
NA	NA	NA		chr7:135174	chr7:135171872-135171886,chr7:13517
NA	NA	NA		chr10:89285	None
RLBP1	ENSG000001		12100	None	None
NA	NA	NA		None	None
NA	NA	NA		None	None
NA	NA	NA		None	None
IL37	ENSG000001		49430	None	None
MICD	ENSG000002		435	chr6:299649	chr6:29965020-29965040,chr6:2996506
NA	NA	NA		None	None
NA	NA	NA		None	None
NA	NA	NA		None	None
MRPS24	ENSG000000		42661	chr7:438148	None
NA	NA	NA		None	None
TAF9BP2	ENSG000002		2035	None	None
OR7E149P	ENSG000001		3488	None	None
ODF2L	ENSG000001		3235	chr1:863394	None
AL645568.3	ENSG000002		11285	None	None
AL354989.2	ENSG000002		23717	chr9:341584	None
NA	NA	NA		None	None
NA	NA	NA		chr4:156940	None
NA	NA	NA		None	None
NA	NA	NA		chr21:38228	chr21:38224363-38224367,chr21:38229
NA	NA	NA		None	None
AC006511.5	ENSG000002		8899	None	None
RUFY4	ENSG000001		26426	None	None

OR2Y1	ENSG000001	41419	None	None
NA	NA	NA	None	None
NA	NA	NA	None	None
NA	NA	NA	None	None
NA	NA	NA	None	None
NA	NA	NA	None	None
NA	NA	NA	None	None
CXCR5	ENSG000001	16124	None	None
NA	NA	NA	None	None
NA	NA	NA	None	None
KRTAP4-11	ENSG000002	1127	None	None
NA	NA	NA	None	None
RNU6-1312P	ENSG000001	216091	chr2:838791	None
NA	NA	NA	chr9:337050	None
NA	NA	NA	chr4:960409	None
NA	NA	NA	None	None
NA	NA	NA	None	None
NA	NA	NA	None	None
AC253576.1	ENSG000002	4622	None	None
NA	NA	NA	None	None
AL391597.1	ENSG000002	4646	chr1:207423	None
AL162151.3	ENSG000002	6632	None	None
NA	NA	NA	None	None
NA	NA	NA	None	None
NA	NA	NA	None	None
FAM110C	ENSG000001	106526	None	chr2:160522-160528
NA	NA	NA	None	None
LINC00898	ENSG000002	26374	None	None
NA	NA	NA	None	None
NA	NA	NA	None	None
LINC02653	ENSG000002	101799	None	chr10:90295307-90295310
NA	NA	NA	None	None

0453-247360458,chr1:247360182-247360201,chr1:247360228-247360239

1-46423672,chr3:46423588-46423598

6132-105056139

)94226

843-46868847,chr18:46868874-46868899,chr18:46875003-46875048

5-10201408,chr2:10201467-10201478

2468-135172470

6-29965081,chr6:29964964-29964986,chr6:29966121-29966133

723-38229734,chr21:38229786-38229822,chr21:38228619-38228622,chr21:38229593-38229[illegible]

Locus	WB1-padj	WB-2padj	Class	Family	Category	Chrom
ERV316A3_1	8.53E-11	5.41E-24	HERV	ERV316A3	internal	chr12
ERV316A3_1	0.00045675	0.00932584	HERV	HUERSP3	internal	chr2
ERV316A3_2	0.00101075	0.00018562	HERV	ERV316A3	oneside	chrX
ERV316A3_3	4.79E-11	1.27E-13	HERV	HERVL	oneside	chr11
ERV316A3_6	8.54E-06	0.00135347	HERV	ERV316A3	oneside	chr3
ERV316A3_7	0.00725743	0.00043494	HERV	MER101	oneside	chr2
ERV316A3_9	0.0009514	0.02370446	HERV	HERVFH19	oneside	chr2
ERV316A3_X	3.75E-07	5.05E-13	HERV	HERVH	prototype	chr1
ERVLB4_12q	0.00281343	0.0114867	HERV	HERVP71A	prototype	chr1
ERVLB4_17q	8.45E-09	1.04E-06	HERV	ERVLE	internal	chr8
ERVLB4_3p2	5.19E-07	0.00034864	HERV	MER41	oneside	chr6
ERVLB4_4q3	9.57E-06	5.13E-05	HERV	HUERSP2	prototype	chr11
ERVLE_5q22	0.00177051	0.00861311	HERV	HML6	prototype	chr3
ERVLE_7q11	6.17E-05	0.01977539	HERV	HERVFH19	oneside	chr1
ERVLE_7q22	0.00101953	0.00249464	HERV	ERVLE	internal	chr7
ERVLE_7q34	7.84E-05	0.00071732	HERV	HUERSP2	prototype	chr2
ERVLE_8q12	1.41E-06	0.00053745	HERV	ERVLE	internal	chr5
HARLEQUIN_	0.00048107	8.11E-06	HERV	ERV316A3	oneside	chr1
HARLEQUIN_	1.41E-05	2.77E-06	HERV	HERVL	internal	chr7
HERV3_21q2	0.02139448	0.00445981	HERV	HERVL18	oneside	chr3
HERV4_12p1	8.53E-05	7.49E-06	HERV	PABLA	internal	chr6
HERV4_4q22	4.79E-11	7.38E-11	HERV	HERV4	prototype	chr4
HERV9_2q35	0.00171918	0.00200794	HERV	HERVL18	oneside	chr3
HERVE_11q1	0.00554386	0.00609724	HERV	MER101	oneside	chr1
HERVFH19_1	0.00375046	0.00034067	HERV	MER101	internal	chr9
HERVFH19_1	0.03295392	0.00404456	HERV	HERVH	prototype	chr3
HERVFH19_1	3.01E-05	0.01014057	HERV	HML3	prototype	chr4
HERVFH19_2	2.21E-10	7.59E-16	HERV	HARLEQUIN	oneside	chr17
HERVH_19q1	0.00029445	4.28E-06	HERV	LTR25	internal	chr2
HERVH_1p32	0.01090785	7.21E-05	HERV	ERV316A3	oneside	chr21
HERVH_3p21	0.0006776	1.29E-07	HERV	ERVLB4	internal	chr3
HERVH_7q22	0.00259206	0.00028859	HERV	MER34B	unknown	chr11
HERVIP10FH_	7.19E-06	3.33E-07	HERV	MER41	prototype	chr7
HERVL_11q1	1.67E-06	1.99E-05	HERV	HERVH	prototype	chr7
HERVL_16q2	0.00848757	0.00071909	HERV	ERVLB4	internal	chr12
HERVL_5q33	0.0037587	0.0118904	HERV	ERVLB4	internal	chr17
HERVL_7q36	0.00032792	0.01508642	HERV	HERVFH19	oneside	chr1
HERVL18_3p	1.56E-08	2.10E-05	HERV	MER4	oneside	chr12
HERVL18_3p	1.90E-08	1.61E-05	HERV	MER4	oneside	chr12
HERVP71A_1	3.31E-10	9.91E-06	HERV	HERVH	prototype	chr19
HERVP71A_6	0.00060152	1.55E-05	HERV	HERVFH19	oneside	chr12
HML1_11q12	0.00256069	0.0065271	HERV	ERVLB4	oneside	chr4

HML3_4q31.:	9.00E-05	8.00E-06	HERV	MER61	prototype	chr1
HML5_12p13	0.00041625	0.00200794	HERV	ERV316A3	oneside	chr6
HML6_3p21.:	5.15E-07	3.33E-07	HERV	ERVLE	oneside	chr7
HML6_6p22.:	6.10E-10	0.00300361	HERV	MER4	oneside	chr12
HUERSP2_11	0.00375046	0.00150894	HERV	HML5	internal	chr12
HUERSP2_2c	9.44E-11	3.71E-14	HERV	ERV316A3	oneside	chr7
HUERSP3_2p	2.82E-10	2.09E-19	HERV	MER4	internal	chr12
LTR23_9q22.	1.02E-07	1.61E-05	HERV	PRIMA41	internal	chr8
LTR25_1p22.	0.0024989	0.00126972	HERV	PABLA	internal	chr2
LTR25_2q12.	1.31E-06	7.68E-05	HERV	MER4	oneside	chr7
MER101_1p2	2.34E-06	0.00165279	HERV	HML6	prototype	chr6
MER101_2p2	4.79E-11	7.59E-16	HERV	HERVIP10FH	oneside	chr1
MER101_9q3	9.66E-05	0.00152552	HERV	MER4	prototype	chr12
MER34B_11p	0.00171918	3.22E-05	HERV	HERVL	prototype	chr16
MER34B_4q3	3.02E-05	0.00174106	HERV	MER34B	internal	chr4
MER4_12q12	3.10E-08	5.65E-07	HERV	HARLEQUIN	oneside	chr7
MER4_12q12	1.04E-06	4.42E-07	HERV	HERVL	prototype	chr5
MER4_12q12	1.00E-05	0.00100121	HERV	LTR25	internal	chr1
MER4_12q12	4.06E-05	0.00028859	HERV	HERVP71A	oneside	chr6
MER4_12q12	3.36E-07	0.00047403	HERV	LTR23	oneside	chr9
MER4_7q21.	4.79E-11	5.56E-05	HERV	HERV4	oneside	chr12
MER41_6p12	6.63E-05	0.00550707	HERV	ERV316A3	oneside	chr9
MER41_7q22	0.01108746	3.49E-07	HERV	HERV3	prototype	chr21
MER61_1q23	8.08E-06	2.42E-06	HERV	ERVLE	oneside	chr7
PABLA_2p25	0.00030445	0.00062896	HERV	HERV9	oneside	chr2
PABLA_6p12	2.37E-06	0.0014148	HERV	HML1	prototype	chr11
PRIMA41_8q	0.00045675	0.00186353	HERV	HERVE	oneside	chr11

Start	End	Strand	TE_CODING	IntersectedG	IntersectedG	IntersectedO
112974379	112975753	+	noncoding	AC004551.1	lncRNA	ANTISENSE
6838364	6846887	-	noncoding	NRIR,CMPK2	lncRNA,prote	SENSE,SENSI
23748936	23751401	-	noncoding	ACOT9	protein_codi	SENSE
57650629	57651896	+	noncoding	AP000662.1,(	lncRNA,prote	SENSE,SENSI
187374111	187378966	-	noncoding	None	NA	NA
6869773	6876489	+	noncoding	RSAD2	protein_codi	SENSE
37128668	37135454	+	noncoding	EIF2AK2	protein_codi	ANTISENSE
55022708	55028369	+	noncoding	None	NA	NA
247354022	247360572	+	coding	None	NA	NA
64811247	64815461	-	noncoding	AC104232.3,.	lncRNA,lncRI	ANTISENSE,S
49721416	49722594	-	noncoding	AL121974.1,/	lncRNA,TEC	ANTISENSE,/
4550544	4554796	+	noncoding	None	NA	NA
46426543	46433630	+	noncoding	None	NA	NA
9086800	9088393	+	noncoding	SLC2A5	protein_codi	ANTISENSE
106548063	106548761	-	noncoding	AC004917.1	lncRNA	SENSE
162324003	162329299	-	noncoding	GCA	protein_codi	ANTISENSE
112547505	112549086	+	noncoding	AC137549.1	processed_p:	SENSE
153470032	153474206	-	noncoding	None	NA	NA
148575970	148579927	-	noncoding	None	NA	NA
46419310	46423900	-	coding	LINC02009	lncRNA	SENSE
49717615	49721439	-	noncoding	AL121974.1	lncRNA	ANTISENSE
88442739	88453401	+	noncoding	HERC6	protein_codi	SENSE
46417556	46419247	-	noncoding	LINC02009	lncRNA	SENSE
89429509	89431206	-	noncoding	AL596214.1	lncRNA	SENSE
105055371	105056952	+	noncoding	AL591506.1	lncRNA	ANTISENSE
46387685	46393402	-	noncoding	CCR5AS	lncRNA	SENSE
153688368	153694427	-	noncoding	TLR2	protein_codi	ANTISENSE
80397328	80399793	+	noncoding	RNF213,RNF	protein_codi	SENSE,ANTIS
102408621	102413726	+	coding	SDR42E1P5	processed_p:	SENSE
42598743	42600906	-	noncoding	LINC01671	lncRNA	SENSE
46414268	46416315	+	noncoding	None	NA	NA
339634	342458	-	noncoding	None	NA	NA
106455435	106461177	-	noncoding	AC004917.1,.	lncRNA,lncRI	SENSE,ANTIS
106527752	106533658	-	noncoding	AC004917.1	lncRNA	SENSE
55642877	55643624	+	noncoding	AC009779.6	lncRNA	ANTISENSE
80265360	80268548	+	noncoding	RNF213	protein_codi	SENSE
9088458	9096029	+	noncoding	SLC2A5	protein_codi	ANTISENSE
40160628	40165076	-	noncoding	LINC02471	lncRNA	ANTISENSE
40193379	40194785	-	noncoding	LINC02471,LI	lncRNA,lncRI	ANTISENSE,S
54894297	54900597	-	noncoding	None	NA	NA
31906332	31909833	+	noncoding	LINC02422	lncRNA	ANTISENSE
168434326	168439585	+	noncoding	DDX60L	protein_codi	ANTISENSE

159056048	159059052	-		noncoding	None	NA	NA
29946920	29950412	+		noncoding	None	NA	NA
66304013	66309489	-		noncoding	TPST1	protein_codi	ANTISENSE
40187163	40191467	-		noncoding	LINC02471,LI	lncRNA,lncRI	ANTISENSE,S
8558451	8562470	-		noncoding	AC092746.1	lncRNA	SENSE
106475939	106477621	+		noncoding	AC004917.1,/	lncRNA,lncRI	ANTISENSE,S
40191686	40193336	-		noncoding	LINC02471,LI	lncRNA,lncRI	ANTISENSE,S
143050063	143053296	+		noncoding	C8orf31	lncRNA	SENSE
10199041	10202321	-		noncoding	RRM2	protein_codi	ANTISENSE
93127516	93129355	+		noncoding	None	NA	NA
26288022	26296266	-		noncoding	None	NA	NA
159022297	159025513	-		noncoding	IFI16	protein_codi	ANTISENSE
40210828	40213880	+		noncoding	LINC02471,LI	lncRNA,lncRI	SENSE,ANTIS
84855240	84861709	-		noncoding	AC025280.3,(	lncRNA,prote	SENSE,ANTIS
153696581	153699612	+		noncoding	TLR2	protein_codi	SENSE
135171264	135175958	+		coding	AC083862.3	lncRNA	SENSE
159233488	159238427	-		noncoding	AC134043.2,I	lncRNA,lncRI	ANTISENSE,/
84603880	84611675	+		noncoding	AL359762.2	lncRNA	SENSE
29962489	29970366	+		noncoding	None	NA	NA
98116034	98117804	+		noncoding	TRIM14,AL13	protein_codi	ANTISENSE,S
31936771	31945711	-		noncoding	LINC02422	lncRNA	SENSE
34158413	34168326	-		noncoding	None	NA	NA
38224043	38229805	-		noncoding	KCNJ15	protein_codi	ANTISENSE
140290502	140295513	-		noncoding	PPP1R2P6,SL	processed_p:	ANTISENSE,S
218117007	218122161	-		coding	None	NA	NA
56327845	56333225	-		coding	FAM8A2P	unprocessed_	SENSE
67845158	67853485	-		noncoding	ENPP7P7	unprocessed_	SENSE

IntersectedG	TE_type	TE_5UTR_Int	TE_3UTR_Int	ClosestUpstr	ClosestUpstr	DistUpstrear
ENSG000002	INTRONIC	NA	NA	NA	NA	NA
ENSG000002	EXONIC	FALSE	FALSE	NA	NA	NA
ENSG000001	INTRONIC	NA	NA	NA	NA	NA
ENSG000002	EXONIC	FALSE	FALSE	NA	NA	NA
None	INTERGENIC	NA	NA	AC068299.2	ENSG000002	4135
ENSG000001	INTRONIC	NA	NA	NA	NA	NA
ENSG000000	INTRONIC	NA	NA	NA	NA	NA
None	INTERGENIC	NA	NA	BSND	ENSG000001	5536
None	INTERGENIC	NA	NA	AC104335.2	ENSG000002	5946
ENSG000002	INTRONIC	NA	NA	NA	NA	NA
ENSG000002	EXONIC	FALSE	FALSE	NA	NA	NA
None	INTERGENIC	NA	NA	OR52M1	ENSG000001	4400
None	INTERGENIC	NA	NA	LINC02009	ENSG000002	2952
ENSG000001	EXONIC	TRUE	FALSE	NA	NA	NA
ENSG000002	INTRONIC	NA	NA	NA	NA	NA
ENSG000001	INTRONIC	NA	NA	NA	NA	NA
ENSG000002	EXONIC	FALSE	FALSE	NA	NA	NA
None	INTERGENIC	NA	NA	RN7SL44P	ENSG000002	26257
None	INTERGENIC	NA	NA	AC005229.2	ENSG000002	474
ENSG000002	EXONIC	FALSE	FALSE	NA	NA	NA
ENSG000002	INTRONIC	NA	NA	NA	NA	NA
ENSG000001	EXONIC	FALSE	TRUE	NA	NA	NA
ENSG000002	EXONIC	FALSE	FALSE	NA	NA	NA
ENSG000002	INTRONIC	NA	NA	NA	NA	NA
ENSG000002	INTRONIC	NA	NA	NA	NA	NA
ENSG000002	EXONIC	FALSE	FALSE	NA	NA	NA
ENSG000001	EXONIC	FALSE	FALSE	NA	NA	NA
ENSG000001	EXONIC	FALSE	TRUE	NA	NA	NA
ENSG000002	EXONIC	FALSE	FALSE	NA	NA	NA
ENSG000002	EXONIC	FALSE	FALSE	NA	NA	NA
None	INTERGENIC	NA	NA	CCRL2	ENSG000001	1271
None	INTERGENIC	NA	NA	B4GALNT4	ENSG000001	27041
ENSG000002	INTRONIC	NA	NA	NA	NA	NA
ENSG000002	INTRONIC	NA	NA	NA	NA	NA
ENSG000002	INTRONIC	NA	NA	NA	NA	NA
ENSG000001	INTRONIC	NA	NA	NA	NA	NA
ENSG000001	EXONIC	TRUE	FALSE	NA	NA	NA
ENSG000002	INTRONIC	NA	NA	NA	NA	NA
ENSG000002	INTRONIC	NA	NA	NA	NA	NA
None	INTERGENIC	NA	NA	NCR1	ENSG000001	5551
ENSG000002	INTRONIC	NA	NA	NA	NA	NA
ENSG000001	INTRONIC	NA	NA	NA	NA	NA

None	INTERGENIC	NA	NA	AIM2	ENSG000001	3432
None	INTERGENIC	NA	NA	HLA-A	ENSG000002	1036
ENSG000001	INTRONIC	NA	NA	NA	NA	NA
ENSG000002	EXONIC	FALSE	FALSE	NA	NA	NA
ENSG000002	INTRONIC	NA	NA	NA	NA	NA
ENSG000002	INTRONIC	NA	NA	NA	NA	NA
ENSG000002	INTRONIC	NA	NA	NA	NA	NA
ENSG000001	EXONIC	FALSE	FALSE	NA	NA	NA
ENSG000001	EXONIC	FALSE	TRUE	NA	NA	NA
None	INTERGENIC	NA	NA	SAMD9	ENSG000002	9493
None	INTERGENIC	NA	NA	H3C9P	ENSG000002	25610
ENSG000001	INTRONIC	NA	NA	NA	NA	NA
ENSG000002	EXONIC	FALSE	FALSE	NA	NA	NA
ENSG000002	EXONIC	FALSE	FALSE	NA	NA	NA
ENSG000001	INTRONIC	NA	NA	NA	NA	NA
ENSG000002	EXONIC	FALSE	FALSE	NA	NA	NA
ENSG000002	INTRONIC	NA	NA	NA	NA	NA
ENSG000002	EXONIC	FALSE	FALSE	NA	NA	NA
None	INTERGENIC	NA	NA	HLA-W	ENSG000002	3919
ENSG000001	EXONIC	FALSE	FALSE	NA	NA	NA
ENSG000002	INTRONIC	NA	NA	NA	NA	NA
None	INTERGENIC	NA	NA	UBAP1	ENSG000001	10679
ENSG000001	INTRONIC	NA	NA	NA	NA	NA
ENSG000002	EXONIC	FALSE	TRUE	NA	NA	NA
None	INTERGENIC	NA	NA	CXCR2	ENSG000001	3128
ENSG000002	EXONIC	FALSE	FALSE	NA	NA	NA
ENSG000002	INTRONIC	NA	NA	NA	NA	NA

ClosestDown	ClosestDown	DistDownstre	FANTOM_en	FANTOM_CAGE
NA	NA	NA	None	None
NA	NA	NA	None	None
NA	NA	NA	None	None
NA	NA	NA	None	None
RTP4	ENSG000001	2035	None	None
NA	NA	NA	None	None
NA	NA	NA	chr2:371317(	None
PCSK9	ENSG000001	11078	None	None
AC104335.3	ENSG000002	29671	chr1:247357{	chr1:247359352-247359363,chr1:24736
NA	NA	NA	None	None
NA	NA	NA	None	None
C11orf40	ENSG000001	16627	None	None
LTF	ENSG00000C	2015	None	chr3:46426843-46426848
NA	NA	NA	None	None
NA	NA	NA	None	None
NA	NA	NA	None	chr2:162329084-162329086
NA	NA	NA	None	None
S100A7	ENSG000001	9381	None	None
AC006974.2	ENSG000002	3793	chr7:148579	chr7:148579470-148579478
NA	NA	NA	chr3:4642088	chr3:46423626-46423645,chr3:4642366
NA	NA	NA	None	None
NA	NA	NA	chr4:884473(	None
NA	NA	NA	None	None
NA	NA	NA	None	None
NA	NA	NA	chr9:105055	chr9:105056239-105056243,chr9:10505
NA	NA	NA	chr3:463889	None
NA	NA	NA	None	None
NA	NA	NA	None	chr17:80397675-80397680
NA	NA	NA	None	None
NA	NA	NA	None	None
LINC02009	ENSG000002	209	None	None
AC136475.9	ENSG000002	5946	chr11:34147	None
NA	NA	NA	None	chr7:106460712-106460751
NA	NA	NA	None	None
NA	NA	NA	None	chr12:55642940-55642962
NA	NA	NA	None	None
NA	NA	NA	None	chr1:9088436-9088458,chr1:9094181-9(
NA	NA	NA	None	None
NA	NA	NA	None	None
AC245128.3	ENSG000002	2877	None	None
NA	NA	NA	chr12:31906(	chr12:31906983-31906986
NA	NA	NA	None	None

IFI16	ENSG000001		893	None	chr1:159056714-159056718
HLA-W	ENSG000002		6184	None	None
NA	NA	NA		None	None
NA	NA	NA		None	None
NA	NA	NA		None	None
NA	NA	NA		None	None
NA	NA	NA		None	None
NA	NA	NA		None	None
NA	NA	NA		chr2:102013	chr2:10201360-10201365,chr2:1020140
SAMD9L	ENSG000001		701	None	None
H4C8	ENSG000001		2384	None	None
NA	NA	NA		chr1:159024	chr1:159024957-159024959
NA	NA	NA		None	None
NA	NA	NA		None	chr16:84855483-84855489
NA	NA	NA		None	None
NA	NA	NA		chr7:135174	chr7:135171872-135171886,chr7:13517
NA	NA	NA		None	None
NA	NA	NA		None	None
MICD	ENSG000002		435	chr6:299649	chr6:29965020-29965040,chr6:2996506
NA	NA	NA		None	None
NA	NA	NA		None	None
AL354989.2	ENSG000002		23717	chr9:341584	None
NA	NA	NA		chr21:38228	chr21:38224363-38224367,chr21:38229
NA	NA	NA		None	None
RUFY4	ENSG000001		26426	None	None
NA	NA	NA		None	None
NA	NA	NA		None	None

0453-247360458,chr1:247360182-247360201,chr1:247360228-247360239

1-46423672,chr3:46423588-46423598

6132-105056139

)94226

5-10201408,chr2:10201467-10201478

2468-135172470

6-29965081,chr6:29964964-29964986,chr6:29966121-29966133

723-38229734,chr21:38229786-38229822,chr21:38228619-38228622,chr21:38229593-38229[illegible]

630

	baseMean	log2FoldChai	lfcSE	stat	pvalue	padj
ENSG00000C	842.307044	0.2531775	0.1155619	2.19083881	0.02846346	0.09688921
ENSG00000C	992.126257	0.36942908	0.11114653	3.32380233	0.00088799	0.00894923
ENSG00000C	3805.38453	0.12175235	0.06233178	1.95329506	0.05078464	0.14309036
ENSG00000C	1280.28404	-0.1593684	0.0604374	-2.6369166	0.00836634	0.04248463
ENSG00000C	475.181147	0.19911518	0.07685355	2.59083915	0.00957422	0.04639579
ENSG00000C	7803.61034	1.43387202	0.18977268	7.55573452	4.17E-14	2.73E-11
ENSG00000C	176.030946	-0.3497441	0.18302142	-1.9109461	0.05601151	0.15254508
ENSG00000C	279.450065	0.91179631	0.16345757	5.57818333	2.43E-08	1.84E-06
ENSG00000C	5676.87143	0.38802052	0.071619	5.4178433	6.03E-08	4.14E-06
ENSG00000C	63195.9617	0.29513173	0.13451172	2.19409676	0.02822845	0.09636934
ENSG00000C	363.50997	-0.1917747	0.06746149	-2.8427283	0.00447292	0.02768345
ENSG00000C	516.912103	-0.2690344	0.12863663	-2.0914295	0.03648958	0.11407429
ENSG00000C	2470.47612	0.32877082	0.08057318	4.08040014	4.50E-05	0.00095614
ENSG00000C	1984.70761	0.9820555	0.19084353	5.1458673	2.66E-07	1.42E-05
ENSG00000C	380.091578	0.84274907	0.25244532	3.33834301	0.0008428	0.0086455
ENSG00000C	15.4994369	0.616733	0.33087576	1.86394132	0.06232995	0.16436541
ENSG00000C	376.180827	0.28656543	0.15531106	1.84510642	0.06502206	0.16928655
ENSG00000C	6387.39769	0.24906351	0.11190253	2.22571829	0.02603306	0.09130781
ENSG00000C	61042.5383	0.93631714	0.33863874	2.76494398	0.00569326	0.03249298
ENSG00000C	5836.98167	0.31033892	0.17708286	1.75250687	0.07968669	0.19354505
ENSG00000C	1424.66425	0.2777611	0.09444245	2.94106184	0.00327089	0.02234905
ENSG00000C	125.651705	0.3362155	0.14438251	2.32864419	0.01987792	0.07627856
ENSG00000C	5261.96728	0.52053934	0.16272685	3.1988535	0.00137975	0.01221588
ENSG00000C	481.545416	2.21616023	0.38966598	5.68733315	1.29E-08	1.07E-06
ENSG00000C	530.25216	-0.513527	0.26349053	-1.948939	0.05130272	0.14401826
ENSG00000C	20036.0081	0.27473406	0.13396766	2.05074916	0.04029138	0.12187009
ENSG00000C	4936.42939	0.63656567	0.20802336	3.06006817	0.00221287	0.01693158
ENSG00000C	21.3088883	0.98266582	0.460324	2.13472646	0.03278336	0.1066565
ENSG00000C	280.932416	0.47694096	0.15495052	3.078021	0.0020838	0.01622417
ENSG00000C	332.42207	-0.3482373	0.15137055	-2.3005617	0.02141642	0.08046862
ENSG00000C	732.038666	0.28689607	0.10036389	2.85855855	0.00425571	0.02675198
ENSG00000C	18648.5852	0.29281502	0.12806406	2.28647307	0.0222266	0.08223139
ENSG00000C	1942.04184	0.31674645	0.13145081	2.40961958	0.01596916	0.06594928
ENSG00000C	1671.36484	-0.1749552	0.08560835	-2.0436699	0.04098617	0.12313123
ENSG00000C	828.885508	-0.209897	0.08438324	-2.4874257	0.01286713	0.05705311
ENSG00000C	387.698412	0.30209516	0.10576312	2.85633735	0.0042856	0.02687224
ENSG00000C	363.397849	0.16007252	0.07711794	2.07568473	0.03792312	0.11710123
ENSG00000C	217.021629	0.47380302	0.1487246	3.18577443	0.00144367	0.01263848
ENSG00000C	1821.3153	0.17154087	0.07256069	2.364102	0.01807384	0.07142879
ENSG00000C	199.731837	-0.3002038	0.16627278	-1.8054895	0.07099811	0.17932163
ENSG00000C	456.296871	0.18479601	0.09356892	1.97497207	0.04827132	0.1379774
ENSG00000C	1011.47815	0.2138957	0.06056067	3.53192405	0.00041255	0.00514037

ENSG00000(	744.181154	0.53911819	0.15899557	3.39077492	0.00069695	0.00755816
ENSG00000(	6834.5442	0.38814514	0.09228406	4.2059825	2.60E-05	0.00061685
ENSG00000(	1074.21808	1.35302917	0.26706024	5.06638187	4.05E-07	2.01E-05
ENSG00000(	16216.4242	0.48627343	0.14698915	3.30822675	0.00093889	0.00931333
ENSG00000(	617.280428	-0.3208712	0.08164652	-3.9300041	8.49E-05	0.00158097
ENSG00000(	864.033531	-0.3066822	0.09947136	-3.0831203	0.00204842	0.01604011
ENSG00000(	372.530423	0.3043925	0.15680718	1.94118976	0.05223526	0.14586199
ENSG00000(	231.102095	0.77894775	0.28678302	2.71615711	0.00660446	0.03602571
ENSG00000(	1096.5055	0.75903194	0.22093787	3.43549941	0.00059146	0.00666086
ENSG00000(	10846.8698	0.20675999	0.11064143	1.86873932	0.06165909	0.16327909
ENSG00000(	6747.31369	-0.1622376	0.04998119	-3.245973	0.0011705	0.01086173
ENSG00000(	624.984101	-0.2066653	0.05338506	-3.8712198	0.00010829	0.00191757
ENSG00000(	243.380587	-0.2554933	0.07828344	-3.2636955	0.00109969	0.01040488
ENSG00000(	2029.39974	0.14602957	0.07726781	1.88991486	0.05876935	0.15828531
ENSG00000(	2443.55527	0.27169302	0.10163322	2.67326988	0.00751158	0.03936345
ENSG00000(	689.664293	2.44069653	0.36003332	6.77908518	1.21E-11	2.68E-09
ENSG00000(	3807.52771	0.17160643	0.07506933	2.28597251	0.02225587	0.08230254
ENSG00000(	1813.22879	0.40372396	0.09813759	4.11385635	3.89E-05	0.00085194
ENSG00000(	1405.20236	0.1448259	0.07824765	1.85086594	0.06418884	0.16767635
ENSG00000(	73.8018627	0.89916012	0.23887902	3.76408163	0.00016716	0.00265597
ENSG00000(	660.44227	0.2586345	0.10762978	2.40300137	0.01626113	0.06675258
ENSG00000(	2498.44361	0.20913474	0.088278	2.36904711	0.01783398	0.07078765
ENSG00000(	2871.89875	-0.3712368	0.13939475	-2.6632052	0.00774002	0.04014981
ENSG00000(	189.569461	-0.2455349	0.12042403	-2.0389193	0.04145809	0.12407159
ENSG00000(	5571.35119	0.15382324	0.08878174	1.73259986	0.08316681	0.19948599
ENSG00000(	664.976205	0.3974966	0.1100638	3.61151068	0.00030442	0.00402544
ENSG00000(	12246.9006	-0.1622479	0.08917054	-1.8195238	0.06883156	0.17585402
ENSG00000(	8856.41288	-0.2002695	0.06978845	-2.8696659	0.00410906	0.02613507
ENSG00000(	2367.9656	0.8205345	0.26956072	3.04396911	0.00233479	0.01760813
ENSG00000(	320.876223	-0.2645732	0.05712352	-4.631599	3.63E-06	0.00012068
ENSG00000(	1157.4334	0.15113617	0.06035451	2.50414045	0.01227493	0.05521866
ENSG00000(	12662.4171	0.33709253	0.18562052	1.8160305	0.06936569	0.17677794
ENSG00000(	2182.96525	0.28684707	0.08472919	3.38545746	0.0007106	0.00766053
ENSG00000(	3550.06975	-0.1470366	0.04967278	-2.9601045	0.00307535	0.02137621
ENSG00000(	4855.37965	-0.1282292	0.04902621	-2.6155242	0.00890906	0.04425383
ENSG00000(	2988.74493	-0.2128256	0.04989694	-4.2653035	2.00E-05	0.00048929
ENSG00000(	4411.93908	0.49374097	0.15545756	3.17605002	0.00149295	0.01295235
ENSG00000(	288.986614	1.9291104	0.29745913	6.4852957	8.86E-11	1.53E-08
ENSG00000(	3832.21369	-0.1128471	0.03814942	-2.9580293	0.00309613	0.02145697
ENSG00000(	14656.8057	0.30313459	0.13506053	2.2444351	0.02480442	0.08847467
ENSG00000(	1410.20496	0.82419441	0.17586246	4.68658522	2.78E-06	9.55E-05
ENSG00000(	165.264643	0.24336835	0.09295491	2.61813336	0.00884122	0.0440164
ENSG00000(	2736.31293	2.12040734	0.41322916	5.13131103	2.88E-07	1.51E-05

ENSG00000(	6391.86494	0.20249303	0.0881294	2.29767836	0.0215801	0.08093525
ENSG00000(	2126.85393	0.12291546	0.0619731	1.98336783	0.04732636	0.13618994
ENSG00000(	104202.303	0.93914724	0.34992141	2.68388043	0.00727731	0.0385794
ENSG00000(	5191.94464	0.61595196	0.10225176	6.02387657	1.70E-09	1.97E-07
ENSG00000(	1597.74567	-0.2011763	0.08009082	-2.5118523	0.01200993	0.05441472
ENSG00000(	848.453326	-0.2786295	0.06135582	-4.5412074	5.59E-06	0.00017543
ENSG00000(	1149.14062	-0.2623205	0.09739966	-2.6932385	0.00707616	0.03789283
ENSG00000(	819.392889	0.25918438	0.09604217	2.69865172	0.0069621	0.03742049
ENSG00000(	105.375221	0.94029134	0.27267863	3.4483499	0.00056402	0.00641796
ENSG00000(	3129.40798	-0.3501014	0.15636096	-2.2390587	0.0251521	0.08932618
ENSG00000(	309.095756	-0.1998746	0.08840339	-2.2609382	0.02376308	0.08622334
ENSG00000(	2913.37831	-0.1061047	0.05831199	-1.8196037	0.06881937	0.17585028
ENSG00000(	2611.52279	-0.1696087	0.08283176	-2.0476289	0.04059638	0.12236375
ENSG00000(	1336.02545	0.31230103	0.13633847	2.2906304	0.0219848	0.08189113
ENSG00000(	2050.76917	-0.2175647	0.07514236	-2.8953663	0.00378716	0.02466168
ENSG00000(	5.82615523	1.11903213	0.55035433	2.03329395	0.04202283	0.12515575
ENSG00000(	1001.86144	1.12060188	0.22207699	5.04600619	4.51E-07	2.20E-05
ENSG00000(	8027.66813	0.2863056	0.1573059	1.8200563	0.06875043	0.17575629
ENSG00000(	2866.83648	-0.2036673	0.07742748	-2.6304271	0.00852776	0.04307884
ENSG00000(	1263.80927	-0.183664	0.09246215	-1.9863691	0.04699236	0.13558572
ENSG00000(	425.254298	-0.2922616	0.13285495	-2.1998551	0.02781717	0.09558219
ENSG00000(	1085.68311	0.38824645	0.15931243	2.43701293	0.01480915	0.06243909
ENSG00000(	488.577349	-0.2138253	0.1024035	-2.0880665	0.03679184	0.1147459
ENSG00000(	639.829919	0.5967889	0.15865868	3.76146395	0.00016892	0.00267356
ENSG00000(	10149.5565	0.38873729	0.16255594	2.39140626	0.01678397	0.06816093
ENSG00000(	3843.77979	0.32654909	0.10982393	2.97338747	0.00294532	0.02072612
ENSG00000(	1338.69722	0.29975136	0.12896657	2.32425633	0.02011176	0.07692253
ENSG00000(	7762.64872	-0.1094289	0.05103502	-2.1441928	0.03201744	0.10495602
ENSG00000(	7477.8358	0.3099592	0.11333896	2.73479835	0.00624185	0.03466384
ENSG00000(	49210.4001	1.0722771	0.29534485	3.63059354	0.00028277	0.00381858
ENSG00000(	801.883086	0.18987946	0.06327341	3.00093619	0.00269151	0.01944171
ENSG00000(	1098.32402	0.77746499	0.14216066	5.46891788	4.53E-08	3.21E-06
ENSG00000(	9490.23051	0.21767689	0.12323786	1.7663151	0.077343	0.18957522
ENSG00000(	4243.1452	0.17206889	0.05073539	3.39149647	0.00069512	0.00755049
ENSG00000(	1485.46826	0.26453526	0.07705422	3.43310553	0.00059671	0.00669695
ENSG00000(	6167.07877	-0.2650937	0.07715293	-3.4359504	0.00059048	0.00665435
ENSG00000(	492.292524	0.20111458	0.10549944	1.90630956	0.05661006	0.15373221
ENSG00000(	9464.76912	0.17033569	0.06580106	2.58864675	0.00963539	0.0466189
ENSG00000(	7818.6919	0.17782146	0.08704476	2.04287389	0.04106492	0.12327773
ENSG00000(	1169.20019	0.21886206	0.11851107	1.84676481	0.06478123	0.16882039
ENSG00000(	55.67703	1.76536462	0.33894498	5.20841053	1.90E-07	1.07E-05
ENSG00000(	1906.52944	0.22295604	0.09298635	2.39772877	0.01649708	0.06742337
ENSG00000(	2833.50064	0.60917641	0.18171476	3.35237719	0.00080121	0.00836083

ENSG00000(	868.814637	-0.2454291	0.0933465	-2.629227	0.00855792	0.04315014
ENSG00000(	5499.41297	-0.1108997	0.04618695	-2.4011052	0.01634564	0.06698824
ENSG00000(	14142.5614	0.67230106	0.14957945	4.49460839	6.97E-06	0.00021047
ENSG00000(	2390.90717	-0.1242521	0.0610403	-2.0355748	0.04179307	0.12466453
ENSG00000(	4320.20186	0.27075263	0.13136495	2.06107211	0.03929616	0.11992167
ENSG00000(	19505.1569	-0.2847253	0.06663346	-4.2730078	1.93E-05	0.000476
ENSG00000(	2740.38924	0.36329558	0.14159802	2.56568258	0.0102973	0.04877833
ENSG00000(	17929.0029	0.53161642	0.09754678	5.44986137	5.04E-08	3.56E-06
ENSG00000(	16440.8666	0.55684596	0.19275041	2.8889482	0.00386533	0.02499202
ENSG00000(	1579.41081	0.31655051	0.07827583	4.04403898	5.25E-05	0.00107751
ENSG00000(	2671.97419	-0.1184215	0.06461044	-1.8328534	0.06682438	0.17231556
ENSG00000(	152.864521	0.54258988	0.16134818	3.36285096	0.00077142	0.00815537
ENSG00000(	15422.7138	-0.1257263	0.06108384	-2.0582587	0.0395653	0.12047396
ENSG00000(	10064.0675	1.23552367	0.29386829	4.20434498	2.62E-05	0.00062043
ENSG00000(	1061.16663	-0.162537	0.0708593	-2.2937993	0.02180203	0.08154376
ENSG00000(	115.820698	0.70147563	0.19070383	3.678351	0.00023475	0.00334999
ENSG00000(	2716.94274	-0.1707187	0.05376727	-3.1751425	0.00149763	0.01297919
ENSG00000(	1581.11147	0.58760147	0.11308919	5.19591195	2.04E-07	1.12E-05
ENSG00000(	7682.35134	0.39525388	0.14761706	2.67756242	0.007416	0.03905572
ENSG00000(	1617.76214	-0.1952598	0.10059811	-1.9409891	0.0522596	0.1459051
ENSG00000(	107.737527	0.27058251	0.15233792	1.77619928	0.07570008	0.18707674
ENSG00000(	2286.32973	0.22377665	0.1106944	2.02157149	0.04322064	0.12771469
ENSG00000(	236.546725	-0.2439464	0.09623214	-2.5349783	0.01124543	0.05192659
ENSG00000(	3120.12402	0.22146156	0.07290205	3.03779618	0.00238315	0.01785131
ENSG00000(	257.119302	-0.300635	0.14698418	-2.0453558	0.04081979	0.12281232
ENSG00000(	6060.55362	0.34529003	0.15977011	2.16116787	0.03068237	0.10201631
ENSG00000(	3062.16065	0.57994668	0.15649231	3.70591169	0.00021063	0.00312707
ENSG00000(	10818.6311	0.16358268	0.0843427	1.93950015	0.05244047	0.14614641
ENSG00000(	5633.67901	-0.3074964	0.097051	-3.1684	0.0015328	0.01323507
ENSG00000(	9076.49222	0.70453575	0.22153532	3.18024121	0.00147153	0.01279228
ENSG00000(	30241.428	0.28119884	0.08943386	3.14421006	0.00166536	0.01396057
ENSG00000(	9666.506	0.2744085	0.10862113	2.52629032	0.01152742	0.05287135
ENSG00000(	2502.27665	-0.3055916	0.10757792	-2.8406532	0.00450212	0.02781512
ENSG00000(	127.634403	1.41708525	0.28863322	4.90964011	9.12E-07	3.89E-05
ENSG00000(	4830.93864	0.16468702	0.08524662	1.9318891	0.05337319	0.14775625
ENSG00000(	1403.07133	0.34629252	0.1516875	2.28293385	0.02243427	0.08264539
ENSG00000(	3679.56279	0.24743592	0.11006369	2.24811586	0.0245688	0.08805809
ENSG00000(	3722.55419	-0.1627563	0.08780679	-1.8535731	0.06380026	0.16692721
ENSG00000(	200.595696	0.23885816	0.09834197	2.42885271	0.01514668	0.06342598
ENSG00000(	111.578411	0.29276711	0.15571674	1.8801261	0.06009089	0.16055245
ENSG00000(	157.37586	-0.4224763	0.2317506	-1.8229781	0.06830671	0.17484512
ENSG00000(	3753.69232	-0.1527952	0.06000897	-2.5462063	0.01089008	0.05070216
ENSG00000(	3142.76932	0.18504115	0.08517584	2.17246062	0.02982094	0.10005605

ENSG00000(	1614.38801	0.54329631	0.11446799	4.74627268	2.07E-06	7.49E-05
ENSG00000(	9851.35595	0.30956017	0.12005864	2.57840811	0.00992567	0.04756026
ENSG00000(	4242.96211	-0.3527146	0.10775178	-3.2733993	0.00106262	0.01015259
ENSG00000(	3784.92919	0.40482577	0.13179765	3.07157043	0.00212936	0.01648494
ENSG00000(	540.058463	0.81157556	0.24210384	3.35217957	0.00080178	0.00836083
ENSG00000(	2196.83381	0.45494203	0.14110122	3.2242246	0.00126314	0.01150604
ENSG00000(	320.772341	0.21584406	0.08441652	2.55689357	0.01056115	0.04966195
ENSG00000(	13996.7474	-0.1404073	0.05488983	-2.5579837	0.0105281	0.04953494
ENSG00000(	1959.34934	0.34515383	0.10919878	3.16078454	0.00157345	0.01346547
ENSG00000(	2030.2114	-0.1464579	0.07162079	-2.0449071	0.04086402	0.1228543
ENSG00000(	1038.34562	0.3787553	0.13628144	2.77921416	0.00544906	0.03163889
ENSG00000(	2034.24427	-0.1597263	0.0769363	-2.0760855	0.03788605	0.11707267
ENSG00000(	3480.65829	0.60585672	0.14660591	4.13255325	3.59E-05	0.00080253
ENSG00000(	3269.19136	0.72621713	0.18066818	4.01961843	5.83E-05	0.00117487
ENSG00000(	1186.61232	-0.2380448	0.08049733	-2.957176	0.00310471	0.02149827
ENSG00000(	823.293315	0.59440481	0.16665221	3.56673823	0.00036145	0.00461943
ENSG00000(	3423.99036	-0.1814692	0.0772989	-2.34763	0.01889328	0.07363753
ENSG00000(	852.100866	-0.2950328	0.10376735	-2.8432145	0.0044661	0.02766552
ENSG00000(	4426.75902	-0.2308253	0.07326051	-3.1507459	0.00162854	0.01374685
ENSG00000(	2310.41105	0.06264291	0.0354801	1.76557859	0.07746657	0.18978327
ENSG00000(	8241.81152	-0.1207569	0.05232804	-2.3076902	0.02101637	0.07940219
ENSG00000(	12670.1801	0.22745696	0.09355288	2.43131955	0.01504394	0.06315296
ENSG00000(	18583.1223	-0.1710756	0.06089394	-2.8094019	0.00496336	0.0297566
ENSG00000(	5759.35531	0.58509856	0.30420106	1.92339424	0.05443056	0.14978081
ENSG00000(	628.101117	-0.2602154	0.1331536	-1.9542496	0.0506717	0.14295662
ENSG00000(	5230.70055	0.3105436	0.14412824	2.15463398	0.03119049	0.10313177
ENSG00000(	161.090538	0.34517889	0.11098312	3.1101927	0.00186965	0.01514485
ENSG00000(	3136.45219	-0.2988526	0.12198189	-2.4499755	0.01428659	0.06090932
ENSG00000(	1661.12385	0.45339071	0.09728273	4.6605468	3.15E-06	0.00010662
ENSG00000(	5297.73381	0.25725305	0.1315475	1.95559052	0.05051341	0.14268189
ENSG00000(	267.417983	1.43534865	0.35707795	4.01970678	5.83E-05	0.00117487
ENSG00000(	13275.3605	-0.2092465	0.06553823	-3.1927393	0.0014093	0.01241715
ENSG00000(	9514.7724	0.19007224	0.07769373	2.44642971	0.01442789	0.06133125
ENSG00000(	363.022875	1.26507965	0.22739006	5.56347814	2.64E-08	1.98E-06
ENSG00000(	2286.09131	0.1707127	0.08513853	2.00511679	0.04495056	0.13133542
ENSG00000(	4564.85376	0.22022955	0.11073681	1.98876549	0.04672709	0.13503422
ENSG00000(	463.745417	-0.4559542	0.20756389	-2.1966935	0.02804234	0.09597377
ENSG00000(	1765.90417	0.49371977	0.24130054	2.04607818	0.04074868	0.12271003
ENSG00000(	982.279735	0.25401251	0.08679152	2.92669745	0.00342582	0.02305013
ENSG00000(	13510.4958	-0.197253	0.05875653	-3.3571246	0.00078758	0.00826753
ENSG00000(	3506.00162	0.30504585	0.08777401	3.47535503	0.00051018	0.0059937
ENSG00000(	961.512495	0.58466486	0.23222286	2.51768868	0.01181277	0.05374416
ENSG00000(	63.8167179	0.39685051	0.21003328	1.88946493	0.05882956	0.15836219

ENSG00000C	400.635472	-0.1936073	0.10441305	-1.8542447	0.06370417	0.16683552
ENSG00000C	4249.32988	0.26800023	0.09349391	2.86649922	0.00415039	0.02628581
ENSG00000C	950.481373	-0.2182045	0.09562799	-2.2818058	0.02250081	0.08278431
ENSG00000C	618.015065	-0.3251809	0.11305484	-2.8763111	0.00402353	0.02571076
ENSG00000C	682.608003	-0.2547173	0.12199399	-2.0879496	0.03680238	0.1147459
ENSG00000C	2476.76101	0.19170943	0.10971343	1.74736515	0.08057402	0.19497819
ENSG00000C	99.6814395	0.94010869	0.18114661	5.189767	2.11E-07	1.15E-05
ENSG00000C	489.262399	1.61346698	0.27167392	5.93898375	2.87E-09	3.00E-07
ENSG00000C	23997.0391	0.35937595	0.11179523	3.21459103	0.0013063	0.01179097
ENSG00000C	746.991925	-0.3949026	0.11779599	-3.3524281	0.00080106	0.00836083
ENSG00000C	7457.53238	0.5252807	0.16299678	3.22264469	0.00127013	0.01153174
ENSG00000C	378.473073	-0.3606669	0.15793312	-2.2836687	0.02239101	0.08252313
ENSG00000C	10310.6937	-0.1030505	0.04500294	-2.2898612	0.02202936	0.0819291
ENSG00000C	343.538062	-0.2428598	0.08922435	-2.7219005	0.00649077	0.03558313
ENSG00000C	402.55657	-0.3394639	0.13246607	-2.5626477	0.01038774	0.04906108
ENSG00000C	2284.19756	0.24385501	0.0662055	3.68330435	0.00023023	0.00330857
ENSG00000C	553.773181	0.29944244	0.07585628	3.94749712	7.90E-05	0.00149361
ENSG00000C	11123.1818	0.16401933	0.07973862	2.05696225	0.03968986	0.12074623
ENSG00000C	50.806786	1.329436	0.30563858	4.34969964	1.36E-05	0.0003617
ENSG00000C	5745.29929	0.31615768	0.16036791	1.97145228	0.04867217	0.13886854
ENSG00000C	4094.17798	-0.1271286	0.0707746	-1.7962466	0.0724553	0.18185843
ENSG00000C	42830.1068	-0.214554	0.09638326	-2.2260503	0.02601081	0.09128838
ENSG00000C	173.596139	-0.2223183	0.08123645	-2.7366821	0.00620622	0.03450778
ENSG00000C	3562.24962	0.11078529	0.05946708	1.86296825	0.06246673	0.16454089
ENSG00000C	370.269048	0.88353329	0.29306122	3.01484207	0.00257113	0.01882091
ENSG00000C	16169.5218	-0.2298672	0.07018829	-3.2750073	0.00105659	0.01012563
ENSG00000C	12641.4787	1.72648285	0.23474302	7.35477811	1.91E-13	9.22E-11
ENSG00000C	1260.90291	-0.1858772	0.10500966	-1.7700963	0.0767111	0.18851853
ENSG00000C	439.688553	-0.1582683	0.07415182	-2.1343826	0.03281147	0.10668556
ENSG00000C	2622.449	-0.2514299	0.14079572	-1.7857781	0.07413519	0.18465666
ENSG00000C	285.618968	-0.1929919	0.06488946	-2.974164	0.00293788	0.02068374
ENSG00000C	136.852054	0.46121041	0.22176748	2.07970262	0.03755282	0.11631095
ENSG00000C	2210.47139	0.26924451	0.10505334	2.56293131	0.01037926	0.04904573
ENSG00000C	7150.51889	0.56343388	0.18846959	2.9895215	0.00279415	0.01997195
ENSG00000C	743.902754	-0.2949098	0.12597856	-2.3409522	0.01923463	0.07447231
ENSG00000C	668.628748	-0.4269516	0.11641374	-3.6675365	0.0002449	0.00346161
ENSG00000C	3063.81798	0.30647182	0.15372618	1.99362158	0.04619343	0.13389316
ENSG00000C	7956.76686	0.09448531	0.0517322	1.82643134	0.06778531	0.17404883
ENSG00000C	1052.51311	-0.2107787	0.0904942	-2.3291952	0.01984873	0.0762129
ENSG00000C	1269.01837	0.38234433	0.14956236	2.55642074	0.01057552	0.04971523
ENSG00000C	1690.34483	0.28099033	0.08608047	3.26427512	0.00109745	0.01039562
ENSG00000C	9047.29677	1.20357332	0.18295605	6.57848347	4.75E-11	8.96E-09
ENSG00000C	3151.92894	-0.2296954	0.09883249	-2.3240876	0.0201208	0.07692253

ENSG00000(	63595.3406	0.44103188	0.18291152	2.41117602	0.01590117	0.06577485
ENSG00000(	7563.97059	0.3835317	0.08187624	4.68428551	2.81E-06	9.64E-05
ENSG00000(	43018.4323	0.42759857	0.22175473	1.9282501	0.05382403	0.14860289
ENSG00000(	190.148103	0.77450981	0.23833375	3.24968585	0.00115533	0.01075135
ENSG00000(	28706.0329	0.3797563	0.19100158	1.9882364	0.04678555	0.13515551
ENSG00000(	7272.17107	0.4323365	0.09879505	4.3760945	1.21E-05	0.00032855
ENSG00000(	1218.28431	0.10992504	0.04785642	2.29697569	0.02162016	0.08102572
ENSG00000(	918.710148	0.17214823	0.06135285	2.80587177	0.00501807	0.03002964
ENSG00000(	3262.90892	0.16796023	0.09676799	1.73570028	0.08261684	0.19850707
ENSG00000(	4775.63646	-0.1227892	0.0391835	-3.1336969	0.00172619	0.01429978
ENSG00000(	3178.46103	0.45628725	0.1004178	4.54388792	5.52E-06	0.00017414
ENSG00000(	8702.51763	0.36340438	0.10856658	3.34729495	0.00081604	0.00846342
ENSG00000(	1870.03988	-0.1877275	0.05957011	-3.1513701	0.00162506	0.01374184
ENSG00000(	3738.64334	0.12467175	0.07183615	1.73550166	0.08265199	0.19854155
ENSG00000(	20330.8671	-0.3175524	0.08865796	-3.5817702	0.00034127	0.0044062
ENSG00000(	24224.9658	-0.1680225	0.08981941	-1.8706704	0.06139078	0.1628052
ENSG00000(	283.153188	0.31087238	0.12613492	2.46460201	0.01371655	0.05942148
ENSG00000(	1333.69556	0.27161562	0.08772627	3.09617205	0.00196037	0.01557518
ENSG00000(	4236.78505	-0.2509902	0.05757777	-4.3591518	1.31E-05	0.00035097
ENSG00000(	179.372836	-0.2617509	0.14513165	-1.803541	0.07130329	0.17984311
ENSG00000(	11158.0446	0.92898859	0.25289906	3.6733572	0.00023938	0.00340138
ENSG00000(	15976.8272	0.19955728	0.09231636	2.16166767	0.0306438	0.10194084
ENSG00000(	2169.37196	0.07932796	0.03721651	2.13152622	0.03304581	0.10725499
ENSG00000(	1956.07768	0.33185775	0.1336795	2.48248797	0.01304685	0.05754673
ENSG00000(	90.7734424	-0.738806	0.22182957	-3.3305118	0.00086687	0.00882308
ENSG00000(	4021.4921	0.18882166	0.10002717	1.88770369	0.05906574	0.1586926
ENSG00000(	6336.72307	0.13182504	0.07183191	1.83518768	0.06647789	0.17174065
ENSG00000(	1405.08898	0.4390232	0.09061916	4.84470587	1.27E-06	5.11E-05
ENSG00000(	1300.15892	-0.2277202	0.08236433	-2.7647914	0.00569592	0.03249687
ENSG00000(	251.045878	-0.2486381	0.09600514	-2.5898419	0.009602	0.04647108
ENSG00000(	6150.22898	0.43714941	0.17769517	2.46010851	0.0138895	0.05987018
ENSG00000(	4521.76423	0.37618205	0.14065393	2.67452209	0.00748358	0.03926667
ENSG00000(	7031.41681	0.31695032	0.1406757	2.25305669	0.02425557	0.08733233
ENSG00000(	929.691597	0.1588005	0.06509232	2.43961969	0.01470273	0.06219832
ENSG00000(	228.64341	0.40940695	0.15607879	2.62307869	0.00871391	0.04354833
ENSG00000(	2418.69555	-0.1599555	0.08779973	-1.8218224	0.06848194	0.17520649
ENSG00000(	3529.8938	-0.2118763	0.07601596	-2.7872603	0.00531558	0.03120641
ENSG00000(	8899.34261	0.26355477	0.13359967	1.97272024	0.04852744	0.13860033
ENSG00000(	125.68245	1.77770278	0.29641108	5.99742345	2.00E-09	2.22E-07
ENSG00000(	5959.40453	0.15607547	0.06041685	2.58331049	0.00978572	0.04709612
ENSG00000(	279.974752	0.26605452	0.13661629	1.94745825	0.05147982	0.14428953
ENSG00000(	1803.60065	0.56534832	0.1817793	3.11008079	0.00187036	0.01514485
ENSG00000(	1848.63568	0.23396521	0.06066247	3.85683648	0.00011486	0.00201221

ENSG00000C	8353.17179	0.17451609	0.09669308	1.80484564	0.07109884	0.17945754
ENSG00000C	384.836965	0.37102425	0.16055645	2.31086473	0.02084033	0.07893713
ENSG00000C	626.522134	0.62055579	0.16116682	3.85039428	0.00011793	0.00204404
ENSG00000C	1036.70288	-0.1854362	0.09624837	-1.9266423	0.05402422	0.14900507
ENSG00000C	2135.63277	-0.1560642	0.08218445	-1.8989506	0.05757097	0.15569705
ENSG00000C	13326.9402	-0.0701949	0.02677423	-2.6217348	0.00874835	0.04364061
ENSG00000C	1311.83586	0.38534014	0.0945334	4.07623265	4.58E-05	0.00096715
ENSG00000C	2378.70522	-0.374082	0.13935453	-2.6843904	0.00726622	0.03856906
ENSG00000C	36680.849	0.71420883	0.23032223	3.10091142	0.00192926	0.01546142
ENSG00000C	7878.27326	0.3405716	0.13972073	2.43751657	0.01478854	0.06241638
ENSG00000C	1631.80177	-0.1029655	0.05560844	-1.8516169	0.06408085	0.16742092
ENSG00000C	19034.4874	0.27691198	0.11667814	2.37329779	0.01763004	0.07036997
ENSG00000C	842.357919	1.69029461	0.31329347	5.39524368	6.84E-08	4.65E-06
ENSG00000C	7714.64333	-0.2408838	0.08853292	-2.7208385	0.00651166	0.03566187
ENSG00000C	3537.94955	0.33320431	0.15760208	2.11421264	0.03449711	0.11030599
ENSG00000C	398.441316	0.1723439	0.07192728	2.39608522	0.01657124	0.06762533
ENSG00000C	39.8121935	-0.7127604	0.31631935	-2.2532936	0.02424065	0.08729933
ENSG00000C	31.0991022	1.91450567	0.40245728	4.75704074	1.96E-06	7.17E-05
ENSG00000C	146.612645	-0.7143211	0.3126138	-2.2849954	0.02231309	0.08242122
ENSG00000C	682.212049	-0.1369763	0.07067862	-1.9380165	0.05262121	0.1464658
ENSG00000C	4711.00653	-0.127195	0.07047401	-1.8048498	0.07109819	0.17945754
ENSG00000C	61.1879454	1.17328498	0.31583748	3.71483771	0.00020333	0.0030584
ENSG00000C	2031.25175	-0.1574623	0.05429596	-2.9000739	0.00373075	0.0244399
ENSG00000C	1814.58655	-0.3563734	0.08556143	-4.1651179	3.11E-05	0.00071868
ENSG00000C	4968.03231	-0.2859745	0.08598969	-3.3256835	0.00088202	0.00892257
ENSG00000C	1925.57776	0.45371307	0.1622425	2.79651179	0.00516575	0.03067832
ENSG00000C	30828.0775	0.74514066	0.12496501	5.96279459	2.48E-09	2.66E-07
ENSG00000C	21375.2524	0.30877559	0.13696538	2.25440609	0.02417063	0.0872196
ENSG00000C	507.176119	-0.3560282	0.13797014	-2.5804726	0.00986652	0.04733215
ENSG00000C	8152.20766	0.18273292	0.09669792	1.88972964	0.05879413	0.15830005
ENSG00000C	428.514956	0.41884952	0.08087728	5.17882824	2.23E-07	1.20E-05
ENSG00000C	2909.38562	-0.0922906	0.05157834	-1.7893287	0.07356189	0.1836346
ENSG00000C	3492.84087	-0.1243258	0.05369566	-2.3153798	0.02059216	0.0782502
ENSG00000C	197.24762	0.7327668	0.31787551	2.30520053	0.02115535	0.07974349
ENSG00000C	744.407379	0.94151328	0.1956713	4.81170855	1.50E-06	5.80E-05
ENSG00000C	3498.01054	0.15292258	0.05555094	2.75283509	0.00590816	0.03333661
ENSG00000C	3578.34045	1.3904483	0.20467498	6.79344536	1.09E-11	2.53E-09
ENSG00000C	4778.18644	0.21954998	0.11383294	1.92870342	0.05376769	0.14854716
ENSG00000C	2029.6001	0.21728119	0.09478568	2.29234179	0.02188593	0.08171987
ENSG00000C	3523.50894	0.36077445	0.13423084	2.68771647	0.00719425	0.03834982
ENSG00000C	551.731376	0.22728451	0.10720283	2.12013535	0.03399463	0.1092668
ENSG00000C	10631.4251	0.25614534	0.14524048	1.76359469	0.07780024	0.19023122
ENSG00000C	1161.45215	0.34449691	0.06657601	5.17448993	2.29E-07	1.23E-05

ENSG00000C	1991.38396	0.39298399	0.08086495	4.85975707	1.18E-06	4.81E-05
ENSG00000C	162.450667	0.38681023	0.11351037	3.40770831	0.00065511	0.00723355
ENSG00000C	2645.31955	0.17227203	0.08356572	2.06151555	0.03925388	0.11981495
ENSG00000C	2475.02765	0.12886893	0.03963099	3.25172113	0.00114709	0.01069895
ENSG00000C	3359.41915	0.45055799	0.09949552	4.52842491	5.94E-06	0.00018385
ENSG00000C	636.286546	0.27423472	0.08182323	3.35155111	0.0008036	0.00836614
ENSG00000C	1477.52403	0.25912172	0.10480414	2.47243798	0.0134195	0.05856788
ENSG00000C	1949.15228	0.4939002	0.20373152	2.42426991	0.0153392	0.06404809
ENSG00000C	232.860163	0.31645563	0.13193899	2.39849971	0.01646239	0.06731516
ENSG00000C	1931.24643	-0.3170364	0.11945429	-2.6540397	0.00795345	0.04088172
ENSG00000C	233.868538	-0.5343136	0.26429835	-2.0216305	0.04321454	0.12771469
ENSG00000C	4180.03627	0.19101231	0.10612749	1.79983819	0.07188619	0.18101746
ENSG00000C	306.220268	0.32159954	0.08340931	3.85567923	0.00011541	0.00201851
ENSG00000C	1764.89097	0.1417146	0.06100589	2.32296565	0.020181	0.07706284
ENSG00000C	1651.98473	-0.1796936	0.10375929	-1.7318311	0.08330364	0.19972655
ENSG00000C	6068.12508	0.23385524	0.13073917	1.78871588	0.07366058	0.18378159
ENSG00000C	1968.31675	0.19058774	0.07428564	2.56560668	0.01029955	0.04877833
ENSG00000C	105.069159	-0.4723825	0.15286405	-3.0902129	0.00200013	0.01580086
ENSG00000C	3919.62685	-0.1827523	0.07217047	-2.532231	0.01133393	0.05223228
ENSG00000C	10254.4377	0.73508606	0.29962661	2.45334038	0.01415363	0.06053133
ENSG00000C	11732.963	0.43474761	0.11767908	3.69434907	0.00022045	0.00320739
ENSG00000C	3730.06033	0.2676647	0.09759199	2.74269139	0.00609379	0.03406748
ENSG00000C	1738.93693	-0.2240718	0.08439794	-2.6549438	0.00793216	0.04084915
ENSG00000C	820.583552	1.02750939	0.23420088	4.38729951	1.15E-05	0.00031432
ENSG00000C	179.284888	0.3974359	0.13143482	3.02382515	0.00249601	0.01837783
ENSG00000C	1115.68268	0.13598921	0.07221426	1.8831352	0.05968205	0.1597725
ENSG00000C	2070.07056	0.18375848	0.08827708	2.08161041	0.03737807	0.1160114
ENSG00000C	1693.2967	0.43274133	0.10406973	4.15818648	3.21E-05	0.00073456
ENSG00000C	2542.34054	0.44111728	0.12700357	3.47326672	0.00051416	0.00602196
ENSG00000C	3741.65889	0.42587919	0.16958067	2.51136634	0.01202648	0.0544596
ENSG00000C	641.623088	0.53285461	0.17382744	3.06542291	0.00217362	0.01668582
ENSG00000C	23666.69	-0.1998664	0.09551849	-2.0924367	0.03639947	0.11396472
ENSG00000C	760.441561	0.10905288	0.06016581	1.8125389	0.06990297	0.17731958
ENSG00000C	951.463765	-0.1596092	0.05990782	-2.6642458	0.00771612	0.04008908
ENSG00000C	1326.58118	-0.195428	0.1105188	-1.7682781	0.07701442	0.18903847
ENSG00000C	16123.3738	-0.1278714	0.07077091	-1.8068358	0.07078788	0.17895603
ENSG00000C	12611.5873	-0.1697396	0.07989354	-2.1245726	0.0336223	0.10854994
ENSG00000C	871.8704	-0.2838863	0.10335927	-2.7465973	0.0060217	0.03375653
ENSG00000C	697.011823	-0.2949804	0.13309948	-2.2162399	0.02667507	0.09288409
ENSG00000C	675.958169	-0.5055201	0.19039077	-2.6551715	0.00792681	0.0408344
ENSG00000C	78.6522516	0.94926719	0.23637314	4.01596897	5.92E-05	0.00118672
ENSG00000C	3240.04892	0.2697294	0.08967357	3.00790293	0.00263057	0.01911098
ENSG00000C	2042.47789	0.28380838	0.15230558	1.8634142	0.06240401	0.16444027

ENSG00000(	1102.84861	0.19297062	0.0589681	3.27245763	0.00106617	0.01016393
ENSG00000(	831.817148	0.16746692	0.07974124	2.10012936	0.03571746	0.11269179
ENSG00000(	3386.10019	0.48404891	0.22212329	2.17919022	0.02931754	0.09896794
ENSG00000(	4000.06703	-0.0936885	0.05281837	-1.7737858	0.07609858	0.18749638
ENSG00000(	4855.61203	0.28026887	0.10072633	2.78247876	0.00539454	0.031456
ENSG00000(	2671.22326	-0.1460838	0.05934414	-2.461638	0.01383042	0.05974584
ENSG00000(	7182.48796	0.48765577	0.15838493	3.0789279	0.00207747	0.01618255
ENSG00000(	125.810161	-0.4062788	0.15617652	-2.6014076	0.00928421	0.04558223
ENSG00000(	1258.48479	0.15460024	0.07680723	2.01283436	0.04413206	0.1295673
ENSG00000(	80.4734032	1.69916122	0.31464252	5.40029123	6.65E-08	4.55E-06
ENSG00000(	1886.3803	0.3090702	0.1713039	1.80422166	0.07119657	0.17962919
ENSG00000(	9356.75084	0.18446987	0.10458758	1.7637836	0.07776842	0.19018177
ENSG00000(	2212.5963	-0.1192098	0.06023827	-1.9789709	0.04781929	0.13710313
ENSG00000(	5800.92247	0.14893296	0.07263182	2.05051943	0.04031377	0.12191532
ENSG00000(	4769.97683	-0.0696609	0.0398272	-1.7490795	0.08027728	0.19438286
ENSG00000(	6050.11374	0.23909771	0.06519677	3.66732444	0.0002451	0.00346161
ENSG00000(	2288.32709	0.10590253	0.05222466	2.02782636	0.04257797	0.12640793
ENSG00000(	252.286261	0.3276291	0.16098224	2.03518784	0.04183198	0.12475425
ENSG00000(	261.015036	-0.272645	0.1405337	-1.9400687	0.05237135	0.14601818
ENSG00000(	549.63649	0.56499306	0.12668134	4.45995492	8.20E-06	0.00024132
ENSG00000(	245.344425	0.45140776	0.1980322	2.27946652	0.02263935	0.08312393
ENSG00000(	1779.19862	-0.2351794	0.07006774	-3.3564574	0.00078948	0.00827691
ENSG00000(	1220.26378	0.57148297	0.25022808	2.28384832	0.02238045	0.08250274
ENSG00000(	696.084566	-0.2830862	0.12074576	-2.3444819	0.01905354	0.0739983
ENSG00000(	1178.14206	-0.1622136	0.07713555	-2.1029684	0.03546853	0.11216539
ENSG00000(	3451.093	1.11697647	0.1965995	5.68148186	1.34E-08	1.10E-06
ENSG00000(	4175.4642	1.05306275	0.3379311	3.11620546	0.00183195	0.01492963
ENSG00000(	47327.8507	0.30733161	0.15440563	1.99041714	0.046545	0.13465039
ENSG00000(	238.294006	-0.2168933	0.11670711	-1.8584416	0.06310634	0.16571983
ENSG00000(	3490.08495	0.2449316	0.11452249	2.1387205	0.03245831	0.10589314
ENSG00000(	46.7337421	0.72839013	0.24946516	2.9198071	0.00350248	0.02337401
ENSG00000(	64.4465146	-0.4786602	0.27270862	-1.7552074	0.07922384	0.19264916
ENSG00000(	1307.4756	-0.2520928	0.07854976	-3.2093391	0.0013304	0.01194015
ENSG00000(	1198.82029	0.47739707	0.1284135	3.71765471	0.00020108	0.00303882
ENSG00000(	890.122146	-0.2193674	0.10796064	-2.0319206	0.04216169	0.12539911
ENSG00000(	4314.59968	0.20952535	0.08876845	2.3603583	0.01825729	0.07189781
ENSG00000(	79.247108	1.3555707	0.25224424	5.37404017	7.70E-08	5.09E-06
ENSG00000(	933.487431	0.31905164	0.14600181	2.18525815	0.02886992	0.09784625
ENSG00000(	1966.81213	0.51508286	0.13923675	3.69933131	0.00021617	0.00318179
ENSG00000(	2353.69658	0.21181353	0.08177635	2.59015649	0.00959323	0.04645343
ENSG00000(	313.570566	-0.3596167	0.1874346	-1.9186247	0.05503184	0.15099555
ENSG00000(	121.04942	-0.2274627	0.12620554	-1.8023195	0.07149515	0.18021615
ENSG00000(	650.710366	0.38188726	0.12132098	3.14774302	0.00164536	0.0138283

ENSG00000(	13402.7274	0.49371441	0.14842031	3.32646124	0.00087956	0.00891359
ENSG00000(	14766.5069	0.26727936	0.13025476	2.05197382	0.04017221	0.12170838
ENSG00000(	1644.33885	0.38992567	0.15646853	2.49203891	0.01270121	0.05653143
ENSG00000(	83.3925777	0.68125224	0.16643507	4.09320118	4.25E-05	0.00091552
ENSG00000(	4192.28512	-0.1363848	0.06488466	-2.1019576	0.03555699	0.1123368
ENSG00000(	3703.00723	-0.1095243	0.05285534	-2.0721513	0.03825134	0.11769697
ENSG00000(	19497.6315	-0.1983798	0.08793113	-2.2560819	0.0240655	0.08697423
ENSG00000(	1224.23373	0.17935378	0.07359933	2.43689436	0.01481401	0.06244351
ENSG00000(	148.270257	-0.3363872	0.12869555	-2.6138217	0.00895357	0.04439424
ENSG00000(	707.784673	0.34691979	0.18421366	1.8832468	0.05966693	0.15975811
ENSG00000(	271.541049	0.4628291	0.15955208	2.90080272	0.00372208	0.02439287
ENSG00000(	722.98746	-0.4706373	0.12221905	-3.8507688	0.00011775	0.00204308
ENSG00000(	408.997117	-0.246238	0.1390962	-1.7702715	0.07668192	0.18851853
ENSG00000(	23151.3755	0.18149175	0.09303582	1.95077278	0.05108408	0.14365043
ENSG00000(	8178.43588	0.31737404	0.13133471	2.41652824	0.01566931	0.06502903
ENSG00000(	1366.4253	0.39549791	0.11698443	3.38077384	0.00072282	0.00776676
ENSG00000(	1713.83396	0.44264185	0.15543322	2.84779446	0.00440233	0.0273532
ENSG00000(	466.179047	-0.2778397	0.0753114	-3.6892118	0.00022495	0.00325552
ENSG00000(	10306.194	-0.1743069	0.06288125	-2.7720012	0.00557128	0.03203619
ENSG00000(	89.3302989	0.40993533	0.16684813	2.45693695	0.01401273	0.06013261
ENSG00000(	7630.74329	0.22709441	0.10328748	2.19866349	0.02790186	0.09568947
ENSG00000(	2216.14157	0.15952098	0.08950114	1.78233466	0.07469467	0.1854586
ENSG00000(	2174.96324	0.17950887	0.09053088	1.98284685	0.04738454	0.13626171
ENSG00000(	418.996624	0.26934699	0.07807104	3.45002429	0.00056054	0.00638272
ENSG00000(	1693.09762	0.16814151	0.07386164	2.27643897	0.02281975	0.08359594
ENSG00000(	66.0311253	0.32568579	0.17695455	1.84050528	0.06569409	0.17033301
ENSG00000(	1191.43538	-0.1739937	0.07765055	-2.2407276	0.02504373	0.08911501
ENSG00000(	289.43658	1.87507688	0.25289544	7.41443543	1.22E-13	6.68E-11
ENSG00000(	54.169563	2.04076134	0.29645164	6.88396049	5.82E-12	1.47E-09
ENSG00000(	3793.68435	-0.2229798	0.11866526	-1.8790651	0.0602356	0.16080806
ENSG00000(	12850.0399	0.22562139	0.12162522	1.85505435	0.06358847	0.16659151
ENSG00000(	3003.70445	-0.2993137	0.08396121	-3.5649049	0.00036399	0.0046446
ENSG00000(	1331.56936	-0.1865789	0.08090025	-2.306283	0.02109483	0.07961754
ENSG00000(	2053.83614	0.15481929	0.07096118	2.18174612	0.02912827	0.09849512
ENSG00000(	2218.8458	0.31524882	0.09852292	3.19975123	0.00137546	0.01219106
ENSG00000(	2204.61161	-0.1865702	0.08139889	-2.2920482	0.02190286	0.08171987
ENSG00000(	113.482799	1.20595474	0.33312769	3.62009762	0.00029449	0.00393544
ENSG00000(	344.937615	1.08572008	0.25144466	4.31792855	1.58E-05	0.00040289
ENSG00000(	559.79456	0.236735	0.12462205	1.89962373	0.05748252	0.15551777
ENSG00000(	6373.282	0.58131145	0.12083162	4.8109214	1.50E-06	5.80E-05
ENSG00000(	18607.6706	0.62849766	0.28609032	2.19685048	0.02803113	0.0959554
ENSG00000(	4947.88813	-0.227579	0.07195458	-3.162814	0.00156252	0.01339993
ENSG00000(	248.484792	0.49068526	0.14838455	3.30684869	0.00094352	0.00933668

ENSG00000(	1519.84113	1.41034218	0.20457453	6.89402608	5.42E-12	1.40E-09
ENSG00000(	2185.2367	0.11724329	0.06677279	1.7558544	0.07911327	0.19243788
ENSG00000(	60.3151209	1.74472207	0.36722558	4.7510908	2.02E-06	7.34E-05
ENSG00000(	2537.96874	0.19044243	0.08966189	2.12400634	0.03366961	0.10861315
ENSG00000(	9960.66281	0.19023524	0.07158108	2.65761917	0.00786948	0.0405773
ENSG00000(	800.565291	-0.3023341	0.15460666	-1.9555048	0.05052352	0.14268586
ENSG00000(	111.637274	0.73162313	0.23421124	3.12377461	0.00178547	0.01465745
ENSG00000(	1545.29508	0.13594473	0.05833723	2.3303255	0.01978895	0.07605947
ENSG00000(	109.240106	0.30928215	0.14960312	2.067351	0.03870109	0.11861342
ENSG00000(	2255.26272	-0.2512206	0.10112105	-2.4843548	0.01297864	0.05737687
ENSG00000(	332.294073	-0.3202477	0.17662753	-1.8131245	0.06981262	0.17729017
ENSG00000(	16943.8824	-0.2263967	0.0485415	-4.6639816	3.10E-06	0.00010507
ENSG00000(	2223.49687	-0.3032431	0.09535597	-3.180116	0.00147216	0.01279228
ENSG00000(	302.940854	-0.7013193	0.18673044	-3.7557844	0.0001728	0.00271361
ENSG00000(	12199.366	0.2122754	0.09935905	2.13644758	0.03264295	0.10630515
ENSG00000(	1892.78985	0.29382038	0.15103302	1.94540489	0.05172627	0.14473645
ENSG00000(	1039.64265	-0.2269661	0.08710309	-2.6057185	0.00916818	0.04521222
ENSG00000(	342.590515	0.97541337	0.17351118	5.62161695	1.89E-08	1.47E-06
ENSG00000(	601.677839	-0.1997767	0.09426817	-2.1192379	0.03407037	0.10936801
ENSG00000(	690.748466	-0.2938897	0.13175025	-2.2306578	0.0257038	0.09066209
ENSG00000(	5725.59673	-0.3918789	0.15862482	-2.4704764	0.01349332	0.05876494
ENSG00000(	4330.52421	0.40784274	0.11167703	3.65198426	0.00026022	0.00361292
ENSG00000(	2747.15754	0.23486053	0.12235627	1.91948097	0.05492349	0.15090156
ENSG00000(	562.077584	-0.1884697	0.08614606	-2.1877921	0.02868475	0.09739984
ENSG00000(	44916.3145	0.43459345	0.13940471	3.11749466	0.00182395	0.01488668
ENSG00000(	4622.07624	-0.1538726	0.06391753	-2.4073622	0.01606822	0.06622456
ENSG00000(	2306.15945	-0.1673206	0.0653666	-2.5597267	0.01047545	0.04932969
ENSG00000(	790.287098	-0.2359585	0.11722668	-2.0128398	0.04413149	0.1295673
ENSG00000(	401.040811	0.44728039	0.15871959	2.81805416	0.00483157	0.02923998
ENSG00000(	1199.92795	0.40298863	0.12121991	3.32444268	0.00088595	0.00894519
ENSG00000(	2180.96523	-0.1292495	0.06425714	-2.0114421	0.04427878	0.12988178
ENSG00000(	69184.1829	0.85873647	0.32003029	2.68329746	0.00729001	0.03863424
ENSG00000(	6495.87536	0.12552954	0.06880891	1.82432093	0.06810357	0.17453801
ENSG00000(	172.73689	-0.3382358	0.12345203	-2.7398152	0.00614737	0.03426189
ENSG00000(	1294.79992	-0.2188075	0.08876827	-2.4649295	0.01370402	0.05938288
ENSG00000(	1626.34765	-0.6358705	0.28792031	-2.2084949	0.02720979	0.09412636
ENSG00000(	4890.00301	-0.3712836	0.11760843	-3.1569474	0.0015943	0.01357308
ENSG00000(	1545.15484	-0.190765	0.08143386	-2.3425759	0.01915114	0.07427182
ENSG00000(	2913.53122	-0.2222894	0.07374513	-3.0142931	0.00257579	0.01882978
ENSG00000(	9697.68244	0.32732125	0.09651979	3.39123466	0.00069579	0.00755049
ENSG00000(	8874.77735	0.39870666	0.14180353	2.81168358	0.0049283	0.02958962
ENSG00000(	5482.11895	-0.209362	0.07423119	-2.8204046	0.00479631	0.02912783
ENSG00000(	719.144965	-0.2666667	0.09849559	-2.7073972	0.00678131	0.0367095

ENSG00000(	27930.9338	0.29525571	0.1396493	2.11426564	0.03449259	0.11030599
ENSG00000(	1618.27287	-0.1695136	0.08811843	-1.9237017	0.05439198	0.14976757
ENSG00000(	2451.7093	0.33328433	0.14346287	2.32314	0.02017163	0.07704502
ENSG00000(	1673.69416	-0.1921119	0.10023117	-1.9166885	0.05527752	0.15135761
ENSG00000(	1583.41287	-0.1405076	0.06705943	-2.0952694	0.03614705	0.11358622
ENSG00000(	443.541681	-0.2663188	0.09451346	-2.8177871	0.00483559	0.02923998
ENSG00000(	5828.1895	-0.1401113	0.05428905	-2.5808392	0.00985605	0.04732506
ENSG00000(	219.406043	0.94647586	0.33083466	2.86087273	0.00422477	0.02661394
ENSG00000(	1266.75363	0.23763762	0.10445593	2.27500355	0.02290572	0.08382255
ENSG00000(	18721.9864	0.36326385	0.20421701	1.77881288	0.07527045	0.18640832
ENSG00000(	2680.52886	0.3854986	0.11432709	3.37189206	0.00074654	0.00795384
ENSG00000(	1478.81815	-0.1113535	0.05359301	-2.077761	0.03773138	0.11673767
ENSG00000(	5181.46984	-0.0861808	0.04917183	-1.7526463	0.07966273	0.19353665
ENSG00000(	6342.33385	0.44571265	0.18853373	2.36410028	0.01807392	0.07142879
ENSG00000(	565.986464	-0.1343122	0.06555756	-2.0487671	0.0404849	0.12222995
ENSG00000(	8387.29852	0.1751799	0.08358414	2.09585084	0.03609542	0.11355654
ENSG00000(	142.685162	-0.2509979	0.1229114	-2.042104	0.04114121	0.12346129
ENSG00000(	50838.6998	0.35805529	0.14806438	2.41824057	0.01559576	0.06483867
ENSG00000(	1267.63484	0.22961204	0.11723541	1.9585554	0.05016488	0.14194193
ENSG00000(	2451.52583	0.22485665	0.08899919	2.52650232	0.01152046	0.05286903
ENSG00000(	1670.15322	-0.1290396	0.07197558	-1.7928252	0.07300088	0.18283113
ENSG00000(	1097.02435	-0.214352	0.0750618	-2.8556731	0.00429457	0.02688347
ENSG00000(	1157.66465	-0.1560844	0.0714791	-2.1836369	0.02898894	0.09808743
ENSG00000(	2998.23526	0.47250285	0.08672806	5.44809672	5.09E-08	3.56E-06
ENSG00000(	142.601148	-0.2695893	0.1450502	-1.8585934	0.0630848	0.16568981
ENSG00000(	1181.50438	0.28223147	0.09919701	2.84516094	0.0044389	0.02755949
ENSG00000(	3692.77839	-0.3089199	0.08647188	-3.57249	0.0003536	0.00454035
ENSG00000(	1423.24537	0.30770391	0.13650857	2.25409966	0.0241899	0.08723236
ENSG00000(	58.4862192	0.39612853	0.19629793	2.01799642	0.04359163	0.128533
ENSG00000(	1081.64722	-0.5384637	0.15561535	-3.4602221	0.00053973	0.00624115
ENSG00000(	668.40348	0.37853457	0.09901948	3.82282922	0.00013193	0.00223938
ENSG00000(	2936.30873	-0.1834065	0.1050646	-1.7456542	0.08087105	0.195351
ENSG00000(	161.367666	0.60229697	0.19980278	3.01445736	0.00257439	0.01882978
ENSG00000(	10906.5671	0.17491314	0.06978331	2.50651835	0.01219267	0.05499952
ENSG00000(	148.8997	0.94304142	0.19842448	4.75264648	2.01E-06	7.30E-05
ENSG00000(	1409.97243	-0.1159661	0.05309551	-2.1841035	0.02895464	0.0980523
ENSG00000(	1251.99431	-0.1563235	0.08364353	-1.868925	0.06163325	0.16327879
ENSG00000(	5317.54928	0.4128128	0.1356542	3.04312577	0.00234134	0.01762673
ENSG00000(	1773.52932	1.25277556	0.21074072	5.94462986	2.77E-09	2.91E-07
ENSG00000(	12348.463	0.48894183	0.25375281	1.926843	0.0539992	0.14896111
ENSG00000(	433.57092	0.3366414	0.19356074	1.73920293	0.08199907	0.19740696
ENSG00000(	80.803759	0.80548785	0.20631219	3.90421833	9.45E-05	0.00173186
ENSG00000(	4641.99686	0.31268996	0.14130842	2.21281911	0.02691012	0.09338523

ENSG00000(	1544.49773	0.28796974	0.08626697	3.33812276	0.00084346	0.0086455
ENSG00000(	729.767762	0.98239136	0.24116881	4.07345947	4.63E-05	0.00097373
ENSG00000(	835.581567	0.59647078	0.14640822	4.07402507	4.62E-05	0.00097333
ENSG00000(	341.149733	2.6417705	0.42199842	6.26014306	3.85E-10	5.13E-08
ENSG00000(	5364.29315	0.27223011	0.08629694	3.15457442	0.00160732	0.01366267
ENSG00000(	4765.3481	0.30374644	0.15014643	2.02300136	0.04307301	0.12739324
ENSG00000(	53.0748975	0.48770249	0.21651464	2.2525151	0.02428974	0.08736541
ENSG00000(	8367.99382	0.38542653	0.13268952	2.90472479	0.00367576	0.02416658
ENSG00000(	319.407818	-0.2032652	0.09447586	-2.1515039	0.03143645	0.10367326
ENSG00000(	3293.56739	0.25889763	0.13889293	1.86400867	0.06232049	0.16436541
ENSG00000(	262.72235	0.29526659	0.08053748	3.66620093	0.00024618	0.00347386
ENSG00000(	142.964187	0.76546723	0.34305443	2.2313288	0.02565936	0.09055886
ENSG00000(	212310.012	0.98849215	0.32129426	3.0765945	0.0020938	0.01627882
ENSG00000(	1286.72678	0.20016065	0.06437818	3.10913821	0.00187634	0.01517078
ENSG00000(	110.368764	4.14715828	0.51564198	8.04270879	8.79E-16	1.80E-12
ENSG00000(	378.294591	0.99288985	0.15674637	6.3343722	2.38E-10	3.49E-08
ENSG00000(	943.780961	-0.1402606	0.07322321	-1.9155204	0.05542617	0.15157357
ENSG00000(	277.507229	-0.1874217	0.10701899	-1.7512943	0.07989523	0.19385056
ENSG00000(	1364.81903	-0.1683249	0.07512616	-2.2405629	0.0250544	0.08913366
ENSG00000(	27686.3318	0.37964101	0.12597085	3.0137212	0.00258065	0.0188485
ENSG00000(	3004.31528	0.26666443	0.10966417	2.43164601	0.01503039	0.0631284
ENSG00000(	349.28371	0.77974918	0.15559264	5.01147861	5.40E-07	2.53E-05
ENSG00000(	3141.31426	-0.2254918	0.12155793	-1.8550151	0.06359407	0.16659151
ENSG00000(	11.982364	1.75635329	0.45298653	3.87727488	0.00010563	0.00188268
ENSG00000(	3391.36657	0.30453338	0.08606098	3.53857657	0.00040229	0.00503155
ENSG00000(	361.664292	1.46482073	0.25480829	5.74871695	8.99E-09	7.93E-07
ENSG00000(	5276.96003	-0.358108	0.12857918	-2.7851169	0.00535084	0.03135732
ENSG00000(	2493.90493	-0.2403225	0.06618895	-3.6308553	0.00028248	0.00381853
ENSG00000(	1834.94936	1.06017993	0.23152705	4.57907578	4.67E-06	0.00015134
ENSG00000(	1723.8838	2.7088965	0.4638412	5.84013777	5.22E-09	4.97E-07
ENSG00000(	1210.60618	-0.185361	0.08882971	-2.0867005	0.03691522	0.11496655
ENSG00000(	600.782557	-0.1625454	0.0871254	-1.8656487	0.06209054	0.16413272
ENSG00000(	9661.40411	-0.2892184	0.07821852	-3.6975687	0.00021767	0.00318964
ENSG00000(	1499.83689	-0.0889364	0.05019559	-1.7717967	0.0764283	0.18811346
ENSG00000(	6298.33613	0.99438812	0.22215944	4.47601116	7.61E-06	0.00022631
ENSG00000(	473.665508	-0.2638792	0.14954523	-1.7645445	0.07764035	0.19009304
ENSG00000(	2517.32608	-0.1341194	0.0611271	-2.1941077	0.02822767	0.09636934
ENSG00000(	181.042099	-0.2284554	0.10470443	-2.1819076	0.02911635	0.09849512
ENSG00000(	1602.60028	-0.1504451	0.08445227	-1.7814215	0.07484362	0.18565975
ENSG00000(	18331.0738	2.34237145	0.33542022	6.98339373	2.88E-12	8.01E-10
ENSG00000(	124.152309	1.6911834	0.3457664	4.89111545	1.00E-06	4.19E-05
ENSG00000(	2922.67604	0.22162864	0.07974049	2.77937381	0.00544638	0.03163889
ENSG00000(	3349.2904	0.18447346	0.08052931	2.29076169	0.0219772	0.08188387

ENSG00000(	7242.5183	-0.1893285	0.08092599	-2.3395272	0.01930816	0.07462986
ENSG00000(	1963.15662	0.18946925	0.07516558	2.52069159	0.01171245	0.05341427
ENSG00000(	2948.70516	0.22544386	0.11830228	1.90565938	0.05669441	0.15388483
ENSG00000(	490.492427	0.31110841	0.14577188	2.13421421	0.03282525	0.10668556
ENSG00000(	150.08367	1.60315976	0.28273985	5.67008784	1.43E-08	1.16E-06
ENSG00000(	4455.65292	-0.2816613	0.10661884	-2.641759	0.00824767	0.04202519
ENSG00000(	3318.23059	0.18555569	0.05574338	3.32874844	0.00087237	0.00887362
ENSG00000(	8101.85862	0.16935553	0.0689273	2.45701663	0.01400962	0.06013261
ENSG00000(	808.763393	-0.1384469	0.07512733	-1.8428301	0.06535383	0.16988058
ENSG00000(	11827.2008	-0.0903671	0.03635875	-2.4854291	0.01293954	0.05726574
ENSG00000(	24812.1553	0.90371237	0.27024737	3.34401914	0.00082574	0.00850481
ENSG00000(	352.366594	0.30057873	0.13808654	2.17674177	0.02949984	0.09930381
ENSG00000(	3845.06472	0.11960141	0.05774727	2.07111802	0.03834777	0.1179273
ENSG00000(	127.741422	0.8034758	0.21787639	3.68775979	0.00022624	0.00326579
ENSG00000(	7520.1557	0.68988786	0.19948675	3.45831423	0.00054357	0.00625769
ENSG00000(	16298.0429	0.65564514	0.23571926	2.78146618	0.0054114	0.03150947
ENSG00000(	119421.214	0.33788622	0.15018844	2.24974846	0.02446492	0.08783693
ENSG00000(	1009.21118	-0.1694487	0.08139594	-2.0817833	0.03736227	0.11600628
ENSG00000(	1290.24265	0.18274324	0.07630086	2.39503512	0.01661877	0.06775187
ENSG00000(	1884.34019	0.17299392	0.07086759	2.44108666	0.01464314	0.06204739
ENSG00000(	2627.2954	-0.340624	0.08944996	-3.807984	0.0001401	0.00232991
ENSG00000(	1076.84003	0.56650383	0.21395474	2.64777412	0.00810236	0.0414264
ENSG00000(	1233.1948	-0.2390094	0.13642305	-1.751972	0.07977863	0.19373174
ENSG00000(	10970.091	-0.1732315	0.06454289	-2.6839755	0.00727524	0.0385794
ENSG00000(	150.336437	1.67442371	0.27861772	6.00975309	1.86E-09	2.13E-07
ENSG00000(	310.661222	0.25686203	0.11441388	2.24502505	0.02476652	0.08837795
ENSG00000(	239.848861	-0.8253072	0.26849585	-3.0738173	0.00211339	0.01640778
ENSG00000(	6694.76429	-0.1497654	0.05542051	-2.7023454	0.00688522	0.03717384
ENSG00000(	1756.82557	0.25794426	0.12571496	2.0518184	0.04018731	0.12171248
ENSG00000(	1467.58147	-0.834549	0.24237207	-3.4432555	0.00057476	0.00651297
ENSG00000(	74.136704	0.80381748	0.21329631	3.76854842	0.0001642	0.0026165
ENSG00000(	24976.1224	0.35501882	0.1514749	2.3437469	0.01909112	0.07410187
ENSG00000(	6617.38357	-0.2665625	0.10370128	-2.5704842	0.01015565	0.0483093
ENSG00000(	5815.84582	0.19567974	0.09991636	1.95843552	0.05017893	0.1419572
ENSG00000(	791.20221	-0.2832595	0.09797636	-2.8911009	0.00383895	0.02485658
ENSG00000(	915.915419	-0.3410103	0.11776821	-2.8956054	0.00378428	0.02466005
ENSG00000(	13171.8663	0.50842082	0.11213646	4.53394742	5.79E-06	0.00018012
ENSG00000(	2026.93406	0.39002172	0.13987297	2.78839959	0.00529692	0.03115263
ENSG00000(	548.639574	0.92212143	0.27998514	3.29346556	0.0009896	0.00967594
ENSG00000(	4762.05971	-0.109252	0.05188977	-2.1054639	0.03525094	0.11180072
ENSG00000(	1589.27863	0.14040998	0.06306457	2.22644807	0.02598418	0.09121675
ENSG00000(	850.574617	-0.3305531	0.09461155	-3.4937925	0.00047621	0.00568299
ENSG00000(	699.489446	0.19352573	0.09679124	1.99941367	0.04556361	0.13255972

ENSG00000(	16495.9782	0.78529661	0.22653046	3.46662694	0.00052703	0.00613761
ENSG00000(	5533.99677	-0.1576758	0.07080731	-2.2268293	0.02595869	0.09121675
ENSG00000(	227.904038	1.18949551	0.2113352	5.62847798	1.82E-08	1.43E-06
ENSG00000(	6127.07577	0.29911449	0.10901205	2.74386626	0.00607203	0.03396896
ENSG00000(	1413.14862	0.28594507	0.08853255	3.22982991	0.00123864	0.01133368
ENSG00000(	1480.55784	-0.1385882	0.07547332	-1.8362543	0.06632006	0.17141394
ENSG00000(	58.3294118	1.42060361	0.25152186	5.64803247	1.62E-08	1.31E-06
ENSG00000(	6312.49042	0.33290832	0.19025639	1.74978787	0.08015492	0.194222
ENSG00000(	5341.36762	0.1855927	0.10460857	1.77416353	0.07603611	0.18747406
ENSG00000(	121.649246	-0.3237667	0.10649491	-3.0402076	0.00236415	0.01775881
ENSG00000(	2670.61309	0.31447097	0.12137888	2.59082112	0.00957472	0.04639579
ENSG00000(	147.111891	1.21854454	0.2397774	5.08198257	3.74E-07	1.90E-05
ENSG00000(	1001.8125	-0.203189	0.07178159	-2.830657	0.00464525	0.02844219
ENSG00000(	3897.75311	-0.2657119	0.09853864	-2.6965249	0.00700672	0.03760691
ENSG00000(	4705.41252	0.22338896	0.10495164	2.12849416	0.03329613	0.10774752
ENSG00000(	547.680863	-0.1713289	0.08319753	-2.0593032	0.0394652	0.12025849
ENSG00000(	1550.16352	0.28999681	0.07608711	3.8113787	0.00013819	0.0023075
ENSG00000(	1310.76102	0.29777796	0.09587643	3.10585162	0.00189732	0.01528764
ENSG00000(	2301.29959	0.09341212	0.04459003	2.09491033	0.03617896	0.11360139
ENSG00000(	204.780982	-0.1980182	0.09086556	-2.1792436	0.02931358	0.09896794
ENSG00000(	11662.688	-0.1297194	0.05297622	-2.4486347	0.01433988	0.06107299
ENSG00000(	114.013291	0.47318804	0.20687127	2.287355	0.02217511	0.08215615
ENSG00000(	69.119795	0.51926915	0.24241643	2.14205426	0.03218912	0.10539236
ENSG00000(	15966.8142	0.20693102	0.09728451	2.12707054	0.03341422	0.10802307
ENSG00000(	609.47156	0.38217516	0.09191569	4.15788833	3.21E-05	0.00073456
ENSG00000(	690.782787	1.85168584	0.38696185	4.78518962	1.71E-06	6.43E-05
ENSG00000(	921.401634	3.28988774	0.51743554	6.358063	2.04E-10	3.16E-08
ENSG00000(	11765.8497	1.21031552	0.3151394	3.84057195	0.00012275	0.00211196
ENSG00000(	374.329893	0.21732262	0.07030231	3.09125864	0.0019931	0.015765
ENSG00000(	956.880566	-0.1898483	0.09030489	-2.1023038	0.03552667	0.11230593
ENSG00000(	198.639417	1.30706521	0.55102984	2.37204072	0.01769014	0.07050687
ENSG00000(	12675.1796	0.5416348	0.12777228	4.23906339	2.24E-05	0.00054363
ENSG00000(	1478.70533	0.25848565	0.08893556	2.9064375	0.0036557	0.02412173
ENSG00000(	1068.8604	-0.1556374	0.07804087	-1.9943069	0.04611853	0.13369971
ENSG00000(	12606.0284	0.30217548	0.1458726	2.07150274	0.03831184	0.11783891
ENSG00000(	614.382345	0.19280899	0.10093805	1.91017158	0.05611113	0.1526554
ENSG00000(	274.077979	-0.2354719	0.13276668	-1.7735766	0.07613321	0.18755353
ENSG00000(	1442.18092	-0.2198069	0.09459955	-2.3235506	0.02014959	0.07701034
ENSG00000(	313.724704	0.58767767	0.18260994	3.2182129	0.00128992	0.01169846
ENSG00000(	310.699276	0.29733168	0.08962043	3.31767752	0.00090769	0.00909189
ENSG00000(	10806.3224	-0.3159641	0.13233471	-2.3876132	0.01695818	0.06869152
ENSG00000(	3002.94781	-0.1562953	0.06707487	-2.3301612	0.01979764	0.07605947
ENSG00000(	295.274237	0.22547561	0.10582724	2.13060099	0.03312203	0.10743855

ENSG00000C	2535.54529	0.21115777	0.06273933	3.36563656	0.00076367	0.00809434
ENSG00000C	568.394093	0.15392929	0.07480713	2.05768208	0.03962066	0.12059773
ENSG00000C	280.066551	0.22276105	0.10288587	2.16512774	0.0303779	0.10134415
ENSG00000C	10285.1042	0.64749805	0.19179121	3.37605689	0.00073533	0.0078805
ENSG00000C	906.604281	0.22996308	0.07563283	3.04051936	0.00236171	0.01775881
ENSG00000C	17101.3699	0.70353915	0.24473686	2.8746759	0.00404442	0.02582415
ENSG00000C	2029.1128	-0.1664257	0.09180894	-1.81274	0.06987194	0.17731958
ENSG00000C	2105.67175	0.79204486	0.1311213	6.04055087	1.54E-09	1.79E-07
ENSG00000C	6532.58372	0.23231698	0.10426501	2.22813946	0.02587122	0.0910518
ENSG00000C	205.468192	0.41076462	0.12262051	3.34988513	0.00080845	0.00840594
ENSG00000C	1033.39277	0.15868674	0.06813097	2.32914253	0.01985152	0.0762129
ENSG00000C	1874.78861	-0.1650515	0.07061156	-2.3374577	0.0194154	0.0749162
ENSG00000C	273.079314	-0.2089889	0.0990036	-2.1109224	0.03477898	0.11097973
ENSG00000C	44.1780802	1.09954362	0.379393	2.89816528	0.00375353	0.02453033
ENSG00000C	4144.23353	0.26569831	0.13398076	1.983108	0.04735537	0.13622561
ENSG00000C	34.8713885	-1.2495512	0.34228867	-3.6505771	0.00026165	0.0036297
ENSG000001	410.594386	-0.1999219	0.11135654	-1.7953319	0.07260084	0.1821549
ENSG000001	137.369362	0.92449356	0.34587064	2.67294605	0.00751883	0.03936345
ENSG000001	817.265787	-0.1578047	0.06477611	-2.4361551	0.01484432	0.0625552
ENSG000001	229.855525	0.50874089	0.20217391	2.51635279	0.01185764	0.0539184
ENSG000001	969.703337	0.1412018	0.0797732	1.77004056	0.07672038	0.18851853
ENSG000001	5826.91439	-0.2212528	0.09222828	-2.3989695	0.01644128	0.06724562
ENSG000001	675.300486	0.17772236	0.08781181	2.02390048	0.04298039	0.1272111
ENSG000001	6261.4344	0.41513819	0.12214781	3.3986543	0.00067718	0.00741239
ENSG000001	13517.4719	-0.1471114	0.08285466	-1.7755357	0.07580948	0.18716398
ENSG000001	2412.44941	0.70994472	0.21813257	3.25464791	0.00113533	0.01062429
ENSG000001	3745.93366	0.21547268	0.10851522	1.98564482	0.04707278	0.1357461
ENSG000001	382.799512	0.97949214	0.29977415	3.26743362	0.00108527	0.01031009
ENSG000001	7913.58832	-0.3932695	0.09643771	-4.0779635	4.54E-05	0.00096246
ENSG000001	459.39546	0.22918989	0.08683564	2.63935271	0.00830645	0.04227215
ENSG000001	695.818627	0.14457006	0.05134739	2.81552882	0.0048697	0.02939931
ENSG000001	1189.03127	-0.2146735	0.07089362	-3.0281072	0.00246091	0.01823385
ENSG000001	521.535227	0.21465778	0.07038288	3.0498578	0.0022895	0.01738004
ENSG000001	356.310116	-0.5163948	0.13878136	-3.7209236	0.0001985	0.00301085
ENSG000001	65.8349756	0.72359546	0.20786592	3.48106822	0.00049942	0.00589558
ENSG000001	543.511439	-0.4727519	0.24251539	-1.9493685	0.05125143	0.1439454
ENSG000001	42.6934945	1.24502848	0.27766626	4.48390259	7.33E-06	0.0002197
ENSG000001	7916.41837	0.37485395	0.13506612	2.77533662	0.00551446	0.03188733
ENSG000001	2504.1839	0.19301614	0.07581559	2.54586366	0.01090078	0.05072078
ENSG000001	90660.878	0.87396905	0.25896996	3.37478926	0.00073872	0.00789624
ENSG000001	5890.89985	0.63379968	0.12628379	5.01885223	5.20E-07	2.45E-05
ENSG000001	3419.72034	0.11991609	0.05855852	2.04779919	0.04057968	0.12236375
ENSG000001	1070.13617	0.28106309	0.13283483	2.11588404	0.03435467	0.11004367

ENSG000001	373.037262	0.27662224	0.07305679	3.78640024	0.00015285	0.0024966
ENSG000001	916.006977	-0.0837206	0.04524907	-1.8502175	0.06428221	0.16786677
ENSG000001	3725.1945	0.41460888	0.11607602	3.57187381	0.00035444	0.00454749
ENSG000001	81.2820991	0.7304197	0.19760132	3.69643133	0.00021865	0.00319254
ENSG000001	818.835205	0.22387516	0.11145888	2.00858973	0.04458066	0.13048717
ENSG000001	816.044288	0.15322288	0.07974709	1.92136008	0.05468633	0.15040116
ENSG000001	182.009072	-0.3566739	0.17544999	-2.0329095	0.04206167	0.12523792
ENSG000001	31336.287	-0.2761504	0.11460296	-2.4096271	0.01596883	0.06594928
ENSG000001	385.4287	0.38945435	0.0909112	4.28389825	1.84E-05	0.00045764
ENSG000001	23821.2646	0.90451043	0.22929469	3.94475089	7.99E-05	0.0015073
ENSG000001	3528.69923	0.25033747	0.14173609	1.76622249	0.07735853	0.18957522
ENSG000001	1176.78011	-0.1461519	0.07034431	-2.077665	0.03774022	0.11673767
ENSG000001	75.1033012	0.82839041	0.22895415	3.61814979	0.00029672	0.00396105
ENSG000001	1684.56298	0.89277657	0.15962534	5.5929502	2.23E-08	1.72E-06
ENSG000001	436.615062	0.3289009	0.1511087	2.17658477	0.02951156	0.09932288
ENSG000001	2640.5937	0.1783339	0.09810998	1.81769381	0.06911095	0.17632055
ENSG000001	633.075699	-0.1206419	0.06152036	-1.9610082	0.04987806	0.14130107
ENSG000001	1515.1425	0.33957416	0.13943847	2.43529761	0.01487955	0.06260711
ENSG000001	9318.58974	0.27920457	0.11846656	2.35682184	0.01843209	0.07238581
ENSG000001	447.23862	0.26660637	0.10777493	2.47373266	0.01337097	0.05844943
ENSG000001	8343.61596	-0.1705367	0.05505504	-3.0975665	0.00195117	0.01553826
ENSG000001	2798.59322	-0.1163637	0.06070067	-1.9170089	0.0552368	0.15133131
ENSG000001	2780.50594	-0.2214713	0.05020305	-4.4115103	1.03E-05	0.00028772
ENSG000001	579.250589	0.63365608	0.16203068	3.91071666	9.20E-05	0.00168969
ENSG000001	1603.86886	0.23470035	0.11049428	2.12409507	0.03366219	0.10861315
ENSG000001	9003.93026	0.16874434	0.05598116	3.01430588	0.00257568	0.01882978
ENSG000001	356.367875	3.39553414	0.50152362	6.77043715	1.28E-11	2.81E-09
ENSG000001	2261.09107	0.39071339	0.22293412	1.75259572	0.07967143	0.19353665
ENSG000001	2478.6683	0.61812824	0.19583199	3.15642117	0.00159718	0.01359054
ENSG000001	790.7753	0.19950818	0.10230327	1.95016428	0.05115654	0.14375558
ENSG000001	367.457208	0.64134052	0.18746603	3.42110269	0.00062368	0.0069615
ENSG000001	33211.6934	0.16772453	0.09051458	1.85301119	0.06388075	0.16705785
ENSG000001	250.327465	-0.5750585	0.21993914	-2.6146254	0.00893254	0.04433014
ENSG000001	96.5774054	0.72473621	0.23511089	3.08252927	0.0020525	0.01604136
ENSG000001	1205.35151	-0.1788147	0.0868825	-2.0581217	0.03957846	0.12049164
ENSG000001	2524.59626	0.23790977	0.10415824	2.28411852	0.02236457	0.08249223
ENSG000001	1663.01115	0.67976262	0.31445805	2.1616957	0.03064164	0.10194084
ENSG000001	61.3926955	0.53321006	0.2332701	2.28580541	0.02226564	0.0823037
ENSG000001	16324.7079	0.88495128	0.17538115	5.04587462	4.51E-07	2.20E-05
ENSG000001	2593.32628	0.26379143	0.12058871	2.18753005	0.02870385	0.09742436
ENSG000001	2918.34548	0.10629035	0.05415019	1.96288042	0.04966007	0.1408295
ENSG000001	2557.7544	0.36472834	0.14181646	2.57183366	0.01011615	0.04820531
ENSG000001	21291.8476	0.76147764	0.24526529	3.10471015	0.00190466	0.01533168

ENSG000001	11228.3665	0.23446979	0.1151802	2.03567787	0.04178271	0.12465632
ENSG000001	18292.6576	-0.0757608	0.04077443	-1.8580471	0.06316232	0.16584028
ENSG000001	10876.0571	0.2031923	0.09934565	2.04530657	0.04082464	0.12281232
ENSG000001	2101.737	-0.1538429	0.05919462	-2.5989332	0.0093514	0.04570637
ENSG000001	1184.63002	0.60695385	0.18358652	3.30609164	0.00094607	0.00935065
ENSG000001	2079.9892	1.13157182	0.24299024	4.65686111	3.21E-06	0.00010788
ENSG000001	1062.11107	0.11999342	0.05537173	2.16705208	0.03023088	0.10105929
ENSG000001	6990.19531	-0.3317728	0.11026191	-3.0089516	0.00262151	0.01907048
ENSG000001	590.805854	0.32041035	0.06709568	4.77542454	1.79E-06	6.68E-05
ENSG000001	1078.41954	0.1038038	0.05615486	1.8485274	0.06452608	0.16836954
ENSG000001	1603.9992	-0.2194775	0.10353445	-2.1198498	0.03401871	0.10930919
ENSG000001	1442.71337	0.12256887	0.04343995	2.82157026	0.00477892	0.02905447
ENSG000001	998.690526	0.17326283	0.0572239	3.02780547	0.00246337	0.01824246
ENSG000001	2827.81083	0.13788387	0.0635107	2.17103375	0.02992862	0.10031317
ENSG000001	269.233313	0.44429487	0.12525312	3.54717611	0.00038938	0.00491511
ENSG000001	4088.22718	0.75171589	0.15517845	4.84420286	1.27E-06	5.11E-05
ENSG000001	542.438318	0.25435704	0.06879922	3.69709162	0.00021808	0.00319224
ENSG000001	11947.6819	0.59301863	0.17727212	3.34524479	0.0008221	0.00849329
ENSG000001	65.1572269	0.64366959	0.15207373	4.23261519	2.31E-05	0.00055536
ENSG000001	1371.44559	0.59553548	0.11687305	5.09557579	3.48E-07	1.79E-05
ENSG000001	312.374046	0.93543544	0.1604519	5.8300055	5.54E-09	5.19E-07
ENSG000001	3758.70852	-0.0906845	0.04758313	-1.9058116	0.05667465	0.15385666
ENSG000001	73.9953455	0.38882814	0.16995424	2.28784023	0.02214683	0.08214592
ENSG000001	2881.34869	1.34125221	0.36920459	3.63281565	0.00028035	0.00379807
ENSG000001	530.106258	-0.2169199	0.10082991	-2.151345	0.03144898	0.10367574
ENSG000001	63.7329325	0.96349778	0.18504624	5.20679476	1.92E-07	1.08E-05
ENSG000001	1638.61953	-0.1981047	0.08732198	-2.268669	0.02328846	0.08485799
ENSG000001	4826.04789	0.24368389	0.06658663	3.65965185	0.00025256	0.00354251
ENSG000001	1052.44272	0.41947934	0.17056372	2.4593702	0.0139181	0.05989025
ENSG000001	582.287767	1.40373107	0.27587445	5.08829672	3.61E-07	1.84E-05
ENSG000001	831.261015	0.45048128	0.13559318	3.32230034	0.00089279	0.00898098
ENSG000001	7742.26267	-0.1766402	0.03846584	-4.5921319	4.39E-06	0.00014331
ENSG000001	1666.10402	-0.0618221	0.03390861	-1.8231989	0.06827327	0.17484433
ENSG000001	1710.55944	-0.0728232	0.03676904	-1.9805572	0.04764095	0.13675418
ENSG000001	8886.16798	0.38191052	0.11718177	3.2591293	0.00111755	0.01050712
ENSG000001	9863.9746	0.25226821	0.10782183	2.33967657	0.01930045	0.07462986
ENSG000001	2166.66685	0.23078573	0.09008423	2.56188834	0.01041048	0.04911277
ENSG000001	1241.73285	0.18125192	0.0792768	2.28631737	0.0222357	0.0822465
ENSG000001	4369.36387	0.19313015	0.06324646	3.05361195	0.00226104	0.01720386
ENSG000001	55.3108804	0.847193	0.22082988	3.83640563	0.00012485	0.0021436
ENSG000001	98.7256283	0.98687208	0.22259999	4.43338776	9.28E-06	0.00026499
ENSG000001	612.919796	0.21347729	0.07258581	2.9410335	0.00327119	0.02234905
ENSG000001	500.115314	0.25134988	0.11845784	2.12185092	0.03385026	0.10898148

ENSG000001	85.9667401	-0.4695463	0.24285285	-1.9334603	0.05317952	0.14748301
ENSG000001	6975.55182	-0.0897703	0.04970463	-1.8060753	0.07090658	0.17914565
ENSG000001	4345.15834	0.09918288	0.04965857	1.99729635	0.045793	0.1330024
ENSG000001	202.157823	0.46268996	0.21396472	2.16245911	0.03058281	0.10188262
ENSG000001	285.452075	0.3704393	0.12206927	3.03466465	0.00240803	0.01794823
ENSG000001	4904.83854	0.13570866	0.07684	1.76611996	0.07737573	0.18958903
ENSG000001	19851.8897	0.1394227	0.07994242	1.74403912	0.08115227	0.19591486
ENSG000001	592.344338	0.13869336	0.07590249	1.82725709	0.06766112	0.17386184
ENSG000001	692.336185	0.23762188	0.10748373	2.21077076	0.02705172	0.09373774
ENSG000001	2277.72396	-0.1303496	0.04325103	-3.0137906	0.00258006	0.0188485
ENSG000001	337.628649	0.55450015	0.20295341	2.73215483	0.00629216	0.0348438
ENSG000001	2216.52675	0.22545125	0.10685169	2.10994566	0.03486304	0.11115833
ENSG000001	364.008331	2.13237018	0.42621934	5.00298779	5.64E-07	2.61E-05
ENSG000001	107.962872	0.41362511	0.13326256	3.10383591	0.00191029	0.0153635
ENSG000001	1160.69009	-0.1522359	0.07333763	-2.0758221	0.03791041	0.11710123
ENSG000001	275.40489	0.50700688	0.15883934	3.19194781	0.00141317	0.01244454
ENSG000001	1170.73191	0.15145877	0.04566733	3.31656748	0.00091131	0.00911695
ENSG000001	1279.67837	-0.1158597	0.05059387	-2.2899954	0.02202158	0.0819291
ENSG000001	444.270482	-0.2143561	0.1111237	-1.9289861	0.05373259	0.14847692
ENSG000001	1762.93806	0.21843864	0.08625421	2.5324984	0.01132529	0.05220713
ENSG000001	12764.7484	0.41940726	0.14237289	2.9458366	0.00322083	0.02208777
ENSG000001	120552.467	0.5086149	0.1435376	3.54342639	0.00039496	0.00497375
ENSG000001	15046.8598	0.35461413	0.09566012	3.70702175	0.00020971	0.00311753
ENSG000001	6487.39415	0.17168205	0.09447379	1.8172454	0.06917955	0.17644066
ENSG000001	849.480733	0.89283211	0.19633724	4.54744146	5.43E-06	0.00017156
ENSG000001	600.214176	-0.3672644	0.1469527	-2.4992018	0.01244734	0.05567861
ENSG000001	909.617486	0.18115322	0.10349606	1.7503393	0.08005978	0.19419235
ENSG000001	768.391796	0.37082331	0.11722036	3.16347173	0.00155899	0.01338614
ENSG000001	949.362805	0.38850278	0.11543856	3.36545079	0.00076419	0.00809456
ENSG000001	2724.49181	0.36519442	0.15390154	2.37290946	0.01764859	0.07040971
ENSG000001	452.897568	0.53283874	0.16373693	3.25423676	0.00113697	0.01062519
ENSG000001	3637.4952	-0.3181607	0.10189562	-3.1224175	0.00179372	0.01469397
ENSG000001	906.160306	0.26055232	0.06141873	4.2422292	2.21E-05	0.0005376
ENSG000001	2753.81192	0.38198516	0.10327022	3.69888974	0.00021654	0.00318281
ENSG000001	722.747934	0.40064595	0.10130962	3.95466843	7.66E-05	0.00146296
ENSG000001	1946.27743	0.23792097	0.11872811	2.00391431	0.04507924	0.13159414
ENSG000001	625.293801	0.98127886	0.32328953	3.03529425	0.00240301	0.01794823
ENSG000001	684.220973	0.56175994	0.11322934	4.96125792	7.00E-07	3.11E-05
ENSG000001	1957.58755	0.23356573	0.11046389	2.11440792	0.03448044	0.11030599
ENSG000001	450.292004	-0.2999767	0.10178649	-2.9471166	0.00320752	0.02202132
ENSG000001	436.05395	-0.4704754	0.12956356	-3.631232	0.00028207	0.00381611
ENSG000001	5447.89389	0.33670244	0.09020364	3.73269222	0.00018944	0.00290853
ENSG000001	4494.46892	0.22332703	0.11798636	1.8928207	0.05838172	0.15744819

ENSG000001	55.5953227	0.88502222	0.28866858	3.06587652	0.00217033	0.01666833
ENSG000001	2829.86244	0.74314274	0.24098485	3.08377371	0.00204393	0.01602432
ENSG000001	1724.36969	0.31087944	0.07501489	4.14423628	3.41E-05	0.00077324
ENSG000001	7013.59607	0.37964301	0.1537719	2.46887118	0.013554	0.05899786
ENSG000001	1448.80648	0.1729678	0.06458636	2.67808579	0.00740443	0.0390354
ENSG000001	191.731088	0.52336298	0.25379371	2.062159	0.0391926	0.11967245
ENSG000001	883.525425	-0.5566622	0.15354157	-3.625482	0.00028842	0.00387963
ENSG000001	382.747204	-0.1300631	0.07326448	-1.7752541	0.07585595	0.18723618
ENSG000001	125.394901	-0.2858172	0.12353771	-2.3136027	0.02068952	0.07849285
ENSG000001	220.716866	0.78223798	0.20810206	3.75891512	0.00017065	0.00269314
ENSG000001	1236.71523	0.25637471	0.09508007	2.69640839	0.00700917	0.03760776
ENSG000001	1376.57974	0.13146687	0.05390862	2.43869866	0.01474025	0.06226068
ENSG000001	5063.3576	-0.2688707	0.13556151	-1.9833852	0.04732441	0.13618994
ENSG000001	9735.21335	0.85425719	0.19331121	4.41907732	9.91E-06	0.00028169
ENSG000001	5246.22455	0.17312832	0.06369385	2.71813239	0.00656516	0.035899
ENSG000001	14.3696846	2.33422655	0.47182756	4.9472026	7.53E-07	3.30E-05
ENSG000001	373.632556	0.27344834	0.08935444	3.06026572	0.00221141	0.01692831
ENSG000001	240.755043	0.7780468	0.19688258	3.95183164	7.76E-05	0.00147526
ENSG000001	1645.57928	0.64584919	0.19184947	3.36643727	0.00076146	0.0080761
ENSG000001	3765.91267	-0.1689822	0.06426989	-2.6292593	0.00855711	0.04315014
ENSG000001	11188.679	0.23712922	0.09410939	2.51971898	0.01174486	0.0534797
ENSG000001	3355.31822	-0.1622323	0.0800677	-2.0261896	0.04274536	0.12662974
ENSG000001	2979.99918	0.21245692	0.11061506	1.9206871	0.05477117	0.15055897
ENSG000001	1086.77045	2.96763334	0.50857681	5.83517233	5.37E-09	5.09E-07
ENSG000001	2775.47488	0.13955615	0.05837245	2.39078803	0.01681226	0.06823529
ENSG000001	33067.3614	0.22053953	0.08684099	2.53957871	0.01109861	0.05140777
ENSG000001	296.851815	0.23249947	0.06844435	3.3969126	0.00068151	0.00744978
ENSG000001	1249.28542	0.47040366	0.1028274	4.57469162	4.77E-06	0.00015394
ENSG000001	57.5602827	0.7887614	0.22860938	3.45025829	0.00056005	0.00638272
ENSG000001	360.73224	-0.2323876	0.0785026	-2.9602535	0.00307386	0.02137493
ENSG000001	117.083564	-0.3206696	0.14028797	-2.2857956	0.02226622	0.0823037
ENSG000001	711.471027	-0.1954826	0.06564425	-2.9779095	0.00290222	0.02052076
ENSG000001	626.839807	-0.129498	0.06035891	-2.1454659	0.03191561	0.1047269
ENSG000001	2525.98246	-0.1734517	0.07900496	-2.1954532	0.0281311	0.09613707
ENSG000001	3088.14385	0.1083787	0.04978549	2.17691327	0.02948704	0.09930381
ENSG000001	650.218277	-0.5454645	0.18054677	-3.021181	0.00251791	0.01848909
ENSG000001	1924.63384	-0.3249664	0.10922028	-2.9753304	0.00292673	0.0206141
ENSG000001	379.993645	-0.2035443	0.10199563	-1.9956176	0.04597556	0.13342678
ENSG000001	2862.44878	0.25150546	0.09818075	2.56165754	0.0104174	0.04911277
ENSG000001	6479.67728	0.7615648	0.27705406	2.74879496	0.00598148	0.03361148
ENSG000001	2367.7206	-0.1539096	0.07752312	-1.9853379	0.04710689	0.13579671
ENSG000001	706.060492	0.15134324	0.06305899	2.40002628	0.0163939	0.06711878
ENSG000001	67.1221932	0.73931471	0.32665287	2.26330393	0.02361696	0.08586415

ENSG000001	6588.80051	0.35779804	0.12856996	2.7829055	0.00538745	0.03144245
ENSG000001	7202.48345	0.49342389	0.22277611	2.21488688	0.02676783	0.09309119
ENSG000001	1036.34677	0.39074756	0.12169399	3.21090272	0.00132319	0.01190796
ENSG000001	595.409983	-0.2190369	0.11120025	-1.969752	0.0488668	0.13918167
ENSG000001	767.750983	0.94051186	0.27059899	3.47566649	0.00050959	0.0059937
ENSG000001	17460.3574	0.35226806	0.14712454	2.39435281	0.01664972	0.06782357
ENSG000001	201.269841	-0.2667041	0.12075765	-2.2085894	0.02720321	0.09412346
ENSG000001	911.85213	0.23685846	0.10544949	2.24617917	0.02469253	0.0883061
ENSG000001	48805.8618	0.76582793	0.29174304	2.62500838	0.00866468	0.04340163
ENSG000001	5580.63626	0.72332423	0.23167898	3.12209689	0.00179568	0.01469982
ENSG000001	5497.87932	-0.2392532	0.11772721	-2.0322674	0.0421266	0.12534019
ENSG000001	146.699583	-0.2683047	0.13651096	-1.9654441	0.04936286	0.14025348
ENSG000001	89.3402987	0.28356163	0.14126328	2.00732728	0.04471483	0.13080981
ENSG000001	3248.83917	0.29693233	0.12349198	2.40446646	0.01619609	0.0665709
ENSG000001	1604.51509	0.22314989	0.06928401	3.22079933	0.00127834	0.01159982
ENSG000001	5386.08998	0.46738564	0.14577373	3.2062406	0.00134481	0.01202341
ENSG000001	985.677586	-0.277774	0.15220863	-1.8249557	0.06800771	0.17437411
ENSG000001	129.282471	-0.5675413	0.28811386	-1.9698508	0.04885547	0.13917359
ENSG000001	248.26502	1.07380454	0.2145578	5.00473327	5.59E-07	2.60E-05
ENSG000001	1878.8354	0.28184429	0.10375466	2.71644957	0.00659862	0.03601772
ENSG000001	4603.18072	0.29412066	0.10149395	2.89791328	0.00375654	0.02454026
ENSG000001	128.763473	-0.2890985	0.1213302	-2.3827412	0.01718427	0.06921407
ENSG000001	1978.93765	0.23406603	0.09943426	2.35397774	0.01857372	0.07272047
ENSG000001	94.687302	0.39345457	0.16772505	2.34583073	0.01898473	0.07380099
ENSG000001	660.01348	0.25742264	0.13475589	1.91028853	0.05609607	0.15263978
ENSG000001	597.789077	-0.2759302	0.11688576	-2.3606829	0.01824132	0.07186519
ENSG000001	449.549641	-0.2195946	0.12136185	-1.80942	0.07038578	0.1782418
ENSG000001	1698.22863	0.17940499	0.08049122	2.22887652	0.02582212	0.0909491
ENSG000001	4372.07315	0.69592733	0.19381189	3.59073595	0.00032975	0.00430138
ENSG000001	1560.41083	0.29414063	0.10687988	2.75206727	0.00592204	0.03338041
ENSG000001	1057.70882	-0.2071153	0.09844047	-2.1039649	0.03538151	0.11196472
ENSG000001	20752.4843	0.22852072	0.07413302	3.08257687	0.00205217	0.01604136
ENSG000001	2771.15004	0.15833639	0.07483674	2.1157573	0.03436545	0.11004409
ENSG000001	471.368256	-0.336347	0.12593176	-2.6708668	0.00756557	0.03953238
ENSG000001	3656.20195	0.21332632	0.11879707	1.79572042	0.07253899	0.18203579
ENSG000001	5909.79704	0.39207342	0.16986247	2.30818158	0.02098904	0.07931719
ENSG000001	1134.41432	-0.111123	0.05540107	-2.0057909	0.04487856	0.13119519
ENSG000001	1392.77074	0.25322517	0.07286468	3.47527988	0.00051032	0.0059937
ENSG000001	7500.96807	0.39065199	0.11399059	3.42705483	0.00061017	0.00682462
ENSG000001	125.496173	-0.4141108	0.19318301	-2.1436192	0.03206341	0.10508162
ENSG000001	7342.71485	0.13616364	0.07010974	1.94215014	0.05211893	0.14563634
ENSG000001	4704.36385	0.26682482	0.13696165	1.94817169	0.05139442	0.14420163
ENSG000001	4829.97839	0.16020473	0.08080094	1.98270882	0.04739996	0.13628217

ENSG000001	185.424627	-0.3414198	0.13305896	-2.5659287	0.01029	0.04877833
ENSG000001	1114.70341	-0.3287573	0.1432828	-2.2944646	0.02176382	0.08141944
ENSG000001	568.734077	0.31040485	0.15299821	2.02881361	0.04247728	0.12617752
ENSG000001	14789.7737	-0.1392211	0.04869776	-2.8588808	0.00425139	0.02673954
ENSG000001	2695.26364	0.31282634	0.06743739	4.63876694	3.50E-06	0.00011705
ENSG000001	3462.08513	1.04360164	0.30790925	3.38931568	0.00070067	0.00757346
ENSG000001	3246.27177	-0.0867938	0.04501752	-1.9280009	0.05385502	0.1486384
ENSG000001	233.374023	0.26636025	0.11620916	2.2920762	0.02190125	0.08171987
ENSG000001	4969.1152	0.22990396	0.11198755	2.05294217	0.04007819	0.12158409
ENSG000001	1531.52173	-0.1972391	0.09404537	-2.0972756	0.03596919	0.11322456
ENSG000001	4761.02912	-0.208457	0.09494867	-2.1954705	0.02812987	0.09613707
ENSG000001	964.921255	0.78338433	0.16577898	4.7254745	2.30E-06	8.11E-05
ENSG000001	22051.2747	0.25975541	0.13818325	1.87978938	0.06013679	0.16064889
ENSG000001	3869.76381	0.4498194	0.13676411	3.28901637	0.00100538	0.00977773
ENSG000001	744.128242	0.13361416	0.07467439	1.78929032	0.07356807	0.1836346
ENSG000001	229.763056	0.30939645	0.08820175	3.50782675	0.00045178	0.00544297
ENSG000001	1536.14468	0.16302509	0.07054998	2.31077461	0.02084531	0.07893776
ENSG000001	658.457175	0.46114592	0.13781539	3.34611347	0.00081953	0.00849329
ENSG000001	1558.22886	0.2701811	0.05700156	4.73988944	2.14E-06	7.71E-05
ENSG000001	900.189828	1.17574723	0.27377809	4.29452638	1.75E-05	0.00043692
ENSG000001	5715.26253	0.23923697	0.09182826	2.60526519	0.00918032	0.04525847
ENSG000001	316.275907	0.19244439	0.08378344	2.29692625	0.02162298	0.08102572
ENSG000001	10071.5826	0.96004257	0.17022296	5.63991223	1.70E-08	1.35E-06
ENSG000001	123637.221	1.03976658	0.24845186	4.18498207	2.85E-05	0.00067091
ENSG000001	3776.79561	0.41151624	0.12817954	3.21046753	0.00132519	0.01191293
ENSG000001	774.189982	2.75650782	0.46369102	5.94470819	2.77E-09	2.91E-07
ENSG000001	2040.40039	0.40615239	0.12820189	3.16806867	0.00153455	0.01323622
ENSG000001	291.780945	0.21681868	0.10058925	2.15548557	0.03112386	0.10296544
ENSG000001	3800.50819	0.12809266	0.06900028	1.85640783	0.06339545	0.16626382
ENSG000001	3530.76382	0.15837431	0.07526141	2.10432279	0.0353503	0.11196424
ENSG000001	3865.82763	0.23938002	0.12063207	1.98438136	0.04721334	0.13593629
ENSG000001	145.302757	-0.3417617	0.12199401	-2.8014629	0.00508715	0.03033236
ENSG000001	685.957473	-0.1682477	0.09552577	-1.7612803	0.07819098	0.19090195
ENSG000001	1279.40269	0.15788446	0.05249887	3.00738773	0.00263503	0.01913492
ENSG000001	98.0318449	0.52483842	0.17695098	2.96601027	0.0030169	0.02105925
ENSG000001	4057.09052	-0.4255644	0.2212483	-1.9234697	0.05442109	0.14978081
ENSG000001	1328.37451	-0.2070279	0.11295508	-1.8328339	0.06682728	0.17231556
ENSG000001	262.199276	0.28170509	0.10577901	2.66314728	0.00774135	0.04014981
ENSG000001	7114.35421	0.32190845	0.08891791	3.62028831	0.00029427	0.00393544
ENSG000001	596.174818	0.16254533	0.08786184	1.85001052	0.06431203	0.16789117
ENSG000001	3661.92328	0.61487187	0.26865972	2.28866412	0.02209888	0.08203651
ENSG000001	2962.36172	0.46967788	0.11217955	4.18684046	2.83E-05	0.0006664
ENSG000001	612.639776	0.32910673	0.11373517	2.89362316	0.00380825	0.02477884

ENSG000001	3671.99114	0.33448397	0.14085642	2.37464491	0.01756584	0.070219
ENSG000001	1144.56017	0.25594376	0.09428814	2.7144852	0.00663789	0.03614793
ENSG000001	1805.7357	0.26855433	0.10858414	2.47323702	0.01338953	0.05851084
ENSG000001	25991.9657	0.30707025	0.16029787	1.91562271	0.05541314	0.15157357
ENSG000001	846.041888	0.91605696	0.23139119	3.9589102	7.53E-05	0.00144563
ENSG000001	3627.87383	0.23992706	0.13519014	1.77473779	0.07594119	0.18736198
ENSG000001	215.476399	-0.5779007	0.3105426	-1.8609387	0.06275283	0.16507091
ENSG000001	8902.36032	-0.3298679	0.12080549	-2.7305701	0.00632249	0.03492919
ENSG000001	1213.57615	0.32726792	0.14513007	2.25499737	0.02413349	0.08712393
ENSG000001	497.789738	0.17209315	0.0770294	2.23412283	0.02547499	0.09004386
ENSG000001	1510.22862	0.75858159	0.18766659	4.04217702	5.30E-05	0.00108272
ENSG000001	12142.7734	0.19246147	0.08942496	2.15221205	0.03138066	0.10359344
ENSG000001	4898.18305	0.11746685	0.05073958	2.31509301	0.02060784	0.07827352
ENSG000001	1964.5233	0.9835172	0.16740626	5.87503229	4.23E-09	4.20E-07
ENSG000001	577.197422	0.2475232	0.07961705	3.10892212	0.00187771	0.01517439
ENSG000001	331.907393	0.22367245	0.07704576	2.9031116	0.00369475	0.02424282
ENSG000001	99.5594928	0.78497567	0.22402405	3.50397946	0.00045836	0.00550604
ENSG000001	181.367143	0.85885264	0.21104482	4.06952724	4.71E-05	0.00098526
ENSG000001	57.7351423	0.52334862	0.19632948	2.665665	0.00768362	0.03997092
ENSG000001	905.734464	-0.2655521	0.09665655	-2.7473779	0.00600739	0.03369004
ENSG000001	448.03673	0.19880205	0.08056543	2.46758517	0.01360279	0.05906909
ENSG000001	912.230945	0.28303607	0.14032812	2.01695905	0.04369979	0.12871302
ENSG000001	3268.3778	-0.1748214	0.07223074	-2.4203184	0.01550692	0.06460607
ENSG000001	5234.88513	1.1965029	0.26397394	4.53265539	5.82E-06	0.00018088
ENSG000001	2060.44413	0.55466906	0.10013998	5.5389373	3.04E-08	2.25E-06
ENSG000001	184.172545	0.15971498	0.087	1.83580437	0.06638659	0.17153183
ENSG000001	161.512425	0.22382421	0.07817533	2.86310547	0.00419511	0.02651781
ENSG000001	3391.40187	0.14747572	0.07937857	1.85787818	0.06318632	0.16585017
ENSG000001	2773.89533	0.23824633	0.07813879	3.04901482	0.00229593	0.0174017
ENSG000001	1514.16359	0.34490275	0.08399431	4.10626341	4.02E-05	0.00087099
ENSG000001	1028.29376	0.15587457	0.06724297	2.3180797	0.02044499	0.07783527
ENSG000001	122097.833	1.27466808	0.27077801	4.70742843	2.51E-06	8.79E-05
ENSG000001	181.89961	0.69816059	0.18192072	3.83771894	0.00012418	0.0021344
ENSG000001	161.665211	-0.2554198	0.13024119	-1.9611288	0.049864	0.14128563
ENSG000001	645.03917	0.23264026	0.08562723	2.71689574	0.00658974	0.0360053
ENSG000001	730.301219	-0.1537712	0.07785982	-1.974975	0.04827098	0.1379774
ENSG000001	209.384418	0.8135971	0.17848087	4.55845557	5.15E-06	0.00016407
ENSG000001	3155.11941	-0.1089014	0.04621935	-2.356186	0.01846367	0.07246262
ENSG000001	1225.62407	-0.1313451	0.06286472	-2.0893288	0.03667813	0.11453273
ENSG000001	386.01327	-0.2645176	0.1039834	-2.5438449	0.01096398	0.05091371
ENSG000001	299.969318	-0.7575717	0.22548788	-3.3597005	0.00078027	0.00821187
ENSG000001	17850.0058	0.20814703	0.11950594	1.74172963	0.08155577	0.19661336
ENSG000001	398.70169	-0.6782114	0.15236294	-4.4512885	8.54E-06	0.00024904

ENSG000001	25567.5159	0.59481064	0.13589272	4.37706026	1.20E-05	0.00032816
ENSG000001	2803.56357	0.13245071	0.07560308	1.75192222	0.07978718	0.19373174
ENSG000001	1048.81424	0.24419473	0.1024577	2.38337122	0.01715489	0.06918743
ENSG000001	2887.64447	0.7350728	0.17203235	4.27287542	1.93E-05	0.000476
ENSG000001	44.3305151	1.65997123	0.3430268	4.83918824	1.30E-06	5.18E-05
ENSG000001	226.71522	0.51140863	0.15958799	3.20455593	0.00135271	0.01207424
ENSG000001	963.049378	-0.2602643	0.12924046	-2.0137991	0.04403063	0.12943523
ENSG000001	314.491868	-0.2766085	0.15755682	-1.7556111	0.07915484	0.19250993
ENSG000001	1540.32698	1.38731205	0.3722788	3.72654056	0.00019413	0.00296653
ENSG000001	1661.95585	-0.1711896	0.08104459	-2.1122885	0.03466171	0.11069856
ENSG000001	1351.24531	0.19517778	0.09007668	2.16679592	0.03025042	0.1010708
ENSG000001	3262.53245	-0.2349544	0.06264312	-3.7506809	0.00017636	0.00275137
ENSG000001	4482.55857	0.26434509	0.10638081	2.4848945	0.01295898	0.05731341
ENSG000001	79.9736719	0.57694257	0.26980975	2.13833107	0.03248988	0.10591864
ENSG000001	2308.37182	0.18384713	0.07219412	2.54656664	0.01087884	0.05069754
ENSG000001	909.425725	0.34306909	0.09453864	3.62887684	0.00028466	0.00383527
ENSG000001	284.592363	0.16822808	0.06782341	2.48038357	0.01312411	0.05775525
ENSG000001	1072.96262	-0.267588	0.08242753	-3.246342	0.00116898	0.0108538
ENSG000001	6335.95589	0.17517643	0.0882737	1.98446905	0.04720357	0.13593629
ENSG000001	534.066097	0.59342549	0.15809869	3.75351298	0.00017437	0.00272825
ENSG000001	414.213661	0.79579046	0.26316963	3.02386895	0.00249565	0.01837783
ENSG000001	1706.30736	-0.174215	0.08323252	-2.0931116	0.03633919	0.11392995
ENSG000001	910.241808	0.14701776	0.07023215	2.09331139	0.03632137	0.11389854
ENSG000001	336.613957	0.26446413	0.09902622	2.67064744	0.00757051	0.03953302
ENSG000001	5797.81985	0.72612366	0.13607282	5.33628733	9.49E-08	6.08E-06
ENSG000001	678.067656	0.12756209	0.06560504	1.9443947	0.05184787	0.14505196
ENSG000001	1601.97071	0.2975286	0.0733008	4.05900918	4.93E-05	0.00102158
ENSG000001	411.431011	0.28314681	0.07430376	3.81066569	0.00013859	0.00231181
ENSG000001	712.523663	0.41769443	0.14621999	2.85661637	0.00428183	0.0268616
ENSG000001	566.12917	-0.2001022	0.10640721	-1.8805323	0.06003557	0.16045692
ENSG000001	1767.93384	-0.2439526	0.07608216	-3.2064365	0.0013439	0.01202237
ENSG000001	714.501695	-0.2433779	0.08050612	-3.0230984	0.00250201	0.01839706
ENSG000001	2982.7558	0.40009409	0.13305771	3.00692144	0.00263908	0.01915582
ENSG000001	1086.82624	0.6429473	0.1357881	4.73493112	2.19E-06	7.85E-05
ENSG000001	1285.03864	-0.2028578	0.09822494	-2.065237	0.03890058	0.11905928
ENSG000001	642.699664	-0.1499522	0.06591367	-2.2749793	0.02290717	0.08382255
ENSG000001	1487.72749	0.30768191	0.10848588	2.8361471	0.00456614	0.02807312
ENSG000001	2613.76393	0.41735384	0.08395966	4.97088551	6.66E-07	2.99E-05
ENSG000001	2731.80906	0.1191339	0.05607596	2.12450925	0.03362758	0.10854994
ENSG000001	1383.8906	-0.2063676	0.09025086	-2.2865996	0.02221921	0.08223139
ENSG000001	1419.78083	-0.1924414	0.05319365	-3.6177514	0.00029717	0.00396159
ENSG000001	4468.65872	-0.1895147	0.07609935	-2.4903595	0.01276139	0.05675307
ENSG000001	591.827784	0.53822689	0.2458302	2.18942545	0.02856593	0.09712182

ENSG000001	22588.7832	0.51771093	0.20933117	2.47316698	0.01339216	0.05851084
ENSG000001	2372.25375	0.60152714	0.12517525	4.80547968	1.54E-06	5.93E-05
ENSG000001	937.918403	0.24695403	0.11890819	2.0768463	0.03781575	0.11690513
ENSG000001	10655.8457	0.2758988	0.11365655	2.42747815	0.0152042	0.06363025
ENSG000001	1745.76565	0.28001607	0.08358421	3.3501072	0.0008078	0.00840453
ENSG000001	10433.7878	0.24059284	0.09619242	2.50116226	0.01237865	0.05554513
ENSG000001	3095.99587	0.30528365	0.08409966	3.63002228	0.0002834	0.00382143
ENSG000001	61.019883	0.3258433	0.16030488	2.03264746	0.04208815	0.12528748
ENSG000001	505.644968	0.1589486	0.07461706	2.130191	0.03315585	0.10750572
ENSG000001	8869.43425	-0.1730072	0.07961497	-2.1730486	0.02977666	0.09992794
ENSG000001	2108.16306	-0.1371314	0.06194145	-2.2138868	0.02683656	0.09316941
ENSG000001	697.28529	0.13650758	0.05910204	2.30969309	0.02090515	0.07907307
ENSG000001	21426.9124	1.31925708	0.19628118	6.72126127	1.80E-11	3.74E-09
ENSG000001	5009.51784	0.1311916	0.0655981	1.99992986	0.04550784	0.13244445
ENSG000001	133.936613	-0.5325523	0.18860879	-2.8235816	0.00474903	0.02895868
ENSG000001	18433.7664	0.91105102	0.24286585	3.75125212	0.00017595	0.00274773
ENSG000001	367.034827	0.4990604	0.17130214	2.9133343	0.00357592	0.02371005
ENSG000001	2151.98826	0.22527421	0.1015111	2.21920774	0.0264726	0.09237522
ENSG000001	11773.053	0.36993978	0.13572111	2.72573491	0.00641585	0.03530225
ENSG000001	1168.5805	-0.1198922	0.04903327	-2.4451182	0.01448047	0.06146421
ENSG000001	241.419931	-0.2959039	0.16908531	-1.7500271	0.08011364	0.19420734
ENSG000001	9799.03288	0.65210705	0.21178027	3.07916811	0.0020758	0.01617719
ENSG000001	2144.42301	0.88048245	0.21688287	4.0597141	4.91E-05	0.00102037
ENSG000001	11880.7532	-0.125041	0.05329682	-2.3461245	0.01896977	0.07377782
ENSG000001	2031.19482	-0.387282	0.12953173	-2.9898618	0.00279104	0.01995841
ENSG000001	2707.60847	-0.1674999	0.07087748	-2.3632313	0.01811636	0.07152755
ENSG000001	2522.85698	-0.1667833	0.06662927	-2.5031539	0.0123092	0.05531214
ENSG000001	1879.60663	-0.0894356	0.04110007	-2.1760456	0.02955185	0.09939728
ENSG000001	4501.35723	-0.3697735	0.12160003	-3.0408995	0.00235873	0.01774944
ENSG000001	2964.71298	0.29448531	0.12747996	2.31005185	0.02088528	0.07905263
ENSG000001	37117.0933	0.3163616	0.15808988	2.0011502	0.04537621	0.13213171
ENSG000001	6805.01545	-0.1672869	0.07301793	-2.291038	0.02196122	0.08187768
ENSG000001	616.778113	-0.3228219	0.13800369	-2.3392268	0.0193237	0.07465851
ENSG000001	5190.56617	0.36248128	0.11658471	3.1091666	0.00187616	0.01517078
ENSG000001	746.358931	0.21741667	0.10001458	2.17384968	0.02971643	0.09984326
ENSG000001	917.877622	0.11865403	0.06498908	1.82575326	0.06788744	0.17412018
ENSG000001	2325.04311	0.16568557	0.0569374	2.90996035	0.00361475	0.02392854
ENSG000001	6587.51785	-0.1330766	0.05330461	-2.49653	0.01254151	0.0559573
ENSG000001	2741.0729	-0.0930891	0.03751699	-2.4812516	0.01309219	0.05764573
ENSG000001	757.926696	-0.3563353	0.12722456	-2.8008372	0.00509702	0.03038018
ENSG000001	2638.72394	-0.2559776	0.07372959	-3.4718432	0.0005169	0.00604104
ENSG000001	10921.0892	0.28505771	0.13606711	2.09497881	0.03617288	0.11360139
ENSG000001	2289.22992	-0.1142234	0.06087288	-1.8764246	0.060597	0.16135254

ENSG000001	7892.02801	-0.2152657	0.07544337	-2.8533415	0.00432621	0.02699272
ENSG000001	625.055811	0.56782594	0.11200033	5.06985947	3.98E-07	1.98E-05
ENSG000001	592.648831	0.16785998	0.08224977	2.04085646	0.0412651	0.1237651
ENSG000001	3514.59534	1.19141293	0.29788704	3.99954602	6.35E-05	0.00124648
ENSG000001	5520.874	0.28233791	0.12636448	2.23431383	0.02546243	0.09001886
ENSG000001	186.236354	0.39661439	0.15396456	2.57601101	0.00999475	0.04782138
ENSG000001	106.607764	1.21311651	0.20953282	5.78962524	7.05E-09	6.43E-07
ENSG000001	1688.34488	-0.1589247	0.05726035	-2.7754748	0.00551212	0.03188733
ENSG000001	2267.55826	0.46121071	0.13800873	3.34189525	0.00083208	0.00855404
ENSG000001	12585.5244	0.38849701	0.11403958	3.40668561	0.00065757	0.00725094
ENSG000001	4324.8197	-0.1591019	0.07698871	-2.066561	0.03877554	0.11879717
ENSG000001	961.336535	0.15499226	0.07262489	2.13414783	0.03283068	0.10668556
ENSG000001	964.868719	-0.325109	0.10200388	-3.1872221	0.00143646	0.01260118
ENSG000001	1620.48328	-0.1235991	0.07137956	-1.7315761	0.08334907	0.19973865
ENSG000001	19477.2642	0.21245122	0.09507267	2.23461911	0.02544237	0.08998673
ENSG000001	429.961943	-0.2520139	0.12995	-1.939314	0.05246312	0.14614981
ENSG000001	1287.88806	-0.1522066	0.08267898	-1.8409349	0.0656311	0.17029384
ENSG000001	5902.60952	0.14710298	0.06685105	2.20045878	0.02777436	0.09545508
ENSG000001	57877.7633	-0.1177033	0.04243654	-2.7736319	0.00554344	0.03196052
ENSG000001	540.493697	1.58520343	0.2449509	6.47151499	9.70E-11	1.66E-08
ENSG000001	60.9298445	2.5043679	0.45970133	5.44781521	5.10E-08	3.56E-06
ENSG000001	2947.43682	1.53791789	0.22583245	6.80999514	9.76E-12	2.35E-09
ENSG000001	541.391217	0.25065317	0.10938994	2.29137321	0.02194184	0.08182404
ENSG000001	1413.61282	0.25122436	0.09815601	2.55943933	0.01048412	0.0493443
ENSG000001	2719.12231	0.18866171	0.09050893	2.0844541	0.03711888	0.11537856
ENSG000001	5796.48194	0.17913249	0.07323718	2.44592286	0.01444819	0.06138801
ENSG000001	817.168802	0.14183729	0.07215361	1.96576835	0.04932538	0.14019556
ENSG000001	921.362205	0.56697755	0.11106703	5.10482333	3.31E-07	1.72E-05
ENSG000001	2474.60827	0.15868329	0.05734142	2.76734125	0.00565156	0.03239027
ENSG000001	578.706969	0.14428755	0.07569937	1.90606022	0.05664239	0.15379456
ENSG000001	1814.88654	0.50307008	0.12222813	4.11582886	3.86E-05	0.0008491
ENSG000001	3273.09999	0.29479556	0.16293166	1.80932027	0.07040126	0.17825347
ENSG000001	6673.84948	0.6356206	0.16847171	3.7728625	0.00016139	0.00259153
ENSG000001	1214.7347	0.24344557	0.08744266	2.78405949	0.00536832	0.03141482
ENSG000001	14791.7708	0.41861051	0.11252849	3.72004026	0.00019919	0.00301582
ENSG000001	486.074167	0.28758262	0.1053119	2.73077036	0.00631865	0.03491974
ENSG000001	277.845278	-0.2361321	0.10514132	-2.2458544	0.02471333	0.08831247
ENSG000001	4111.24598	0.15592995	0.08826957	1.76651995	0.07730866	0.18953799
ENSG000001	3106.11738	0.38132467	0.1209854	3.15182396	0.00162254	0.01373505
ENSG000001	2295.8008	0.31530421	0.13216064	2.38576493	0.01704364	0.06890187
ENSG000001	37.499235	1.69149709	0.43199156	3.91557904	9.02E-05	0.00166345
ENSG000001	36035.1342	0.29584539	0.12904595	2.29255841	0.02187344	0.08171766
ENSG000001	9726.47953	0.19898952	0.11117027	1.7899526	0.07346153	0.18350834

ENSG000001	813.61347	0.11862617	0.05569229	2.13002855	0.03316926	0.10750668
ENSG000001	2518.90675	-0.3297008	0.13175086	-2.5024562	0.01233349	0.05539091
ENSG000001	9251.6133	-0.3536914	0.14695355	-2.4068244	0.0160919	0.06629622
ENSG000001	3224.94242	0.36054838	0.12902743	2.79435441	0.00520035	0.03079633
ENSG000001	751.107031	0.18891986	0.09764167	1.93482821	0.05301138	0.14720196
ENSG000001	3132.54377	0.27919134	0.12699101	2.19851273	0.02791259	0.09568947
ENSG000001	315.195925	-0.5249602	0.14709713	-3.5687996	0.00035862	0.0045904
ENSG000001	996.826275	1.91756741	0.36796623	5.21125926	1.88E-07	1.07E-05
ENSG000001	734.744673	-0.2794139	0.12658056	-2.2074	0.02728613	0.09429097
ENSG000001	3261.34559	-0.1150261	0.05563014	-2.0676938	0.03866883	0.1185519
ENSG000001	1682.92516	0.16602978	0.07556115	2.19729026	0.02799972	0.09586792
ENSG000001	4436.46092	0.57064124	0.1820473	3.13457678	0.00172102	0.01429556
ENSG000001	986.772314	-0.2702181	0.12345415	-2.188813	0.02861043	0.09722804
ENSG000001	369.721062	-0.5033319	0.11579207	-4.3468599	1.38E-05	0.00036582
ENSG000001	2414.41363	-0.2343958	0.06750189	-3.4724326	0.00051576	0.0060364
ENSG000001	312.099491	1.66319373	0.2976538	5.58767841	2.30E-08	1.76E-06
ENSG000001	2431.88595	0.44378738	0.12528851	3.5421234	0.00039692	0.00498298
ENSG000001	1866.52696	1.31830449	0.29698129	4.43901535	9.04E-06	0.00025971
ENSG000001	2882.61224	-0.1923273	0.08834917	-2.1768999	0.02948803	0.09930381
ENSG000001	1258.31688	0.12017028	0.06435405	1.86733039	0.06185546	0.16369336
ENSG000001	2482.66247	-0.2285436	0.07114484	-3.2123707	0.00131644	0.01185378
ENSG000001	157.410212	-0.3506448	0.15330406	-2.2872505	0.02218121	0.08215615
ENSG000001	615.391099	0.23955297	0.11483382	2.08608376	0.03697104	0.11504874
ENSG000001	1640.09138	0.38306041	0.13357006	2.8678614	0.00413257	0.0262139
ENSG000001	7858.50808	0.1461079	0.08218686	1.77775255	0.07544451	0.18669839
ENSG000001	3051.16748	-0.0775141	0.03903037	-1.9859952	0.04703385	0.13568158
ENSG000001	7029.30468	0.7921627	0.15659444	5.05868965	4.22E-07	2.08E-05
ENSG000001	493.412551	0.22425262	0.06482585	3.4593086	0.00054156	0.00624914
ENSG000001	362.732522	0.55151541	0.21307765	2.58833056	0.00964424	0.0466342
ENSG000001	2581.58476	-0.2067519	0.11161171	-1.8524211	0.06396537	0.1672258
ENSG000001	196.888926	-0.2780945	0.13487972	-2.0617961	0.03922715	0.11975566
ENSG000001	2068.73374	-0.174808	0.08871924	-1.9703506	0.0487982	0.13905877
ENSG000001	4658.89881	-0.7873007	0.3845952	-2.0470891	0.04064934	0.12247835
ENSG000001	216.547632	-0.3984311	0.146359	-2.7222865	0.00648319	0.03555347
ENSG000001	5225.01047	-0.0988025	0.05335352	-1.8518465	0.06404787	0.16736143
ENSG000001	967.099991	0.20972944	0.09971501	2.1032885	0.03544056	0.11212017
ENSG000001	1997.31615	-0.4202506	0.17448573	-2.4085096	0.01601781	0.06607396
ENSG000001	2507.752	0.22690202	0.08039148	2.82246356	0.00476562	0.02902746
ENSG000001	2054.90952	0.12792176	0.06861428	1.86436063	0.06227108	0.16436541
ENSG000001	323.953271	0.3123264	0.14428015	2.16472197	0.03040898	0.10140657
ENSG000001	6111.56787	0.28178093	0.09225937	3.05422543	0.00225642	0.01717669
ENSG000001	2924.48531	0.79519024	0.21923788	3.62706592	0.00028666	0.00385909
ENSG000001	1282.49279	0.19391628	0.07416367	2.61470723	0.0089304	0.04433014

ENSG000001	12496.9856	0.23332669	0.11630317	2.00619375	0.04483558	0.13111628
ENSG000001	1326.66802	0.13448185	0.05948391	2.26081055	0.02377099	0.08623295
ENSG000001	516.766072	0.24597604	0.07532786	3.26540609	0.00109307	0.01036617
ENSG000001	254.011493	-0.3410451	0.15512035	-2.1985836	0.02790754	0.09568947
ENSG000001	697.7213	0.57058589	0.15386038	3.7084653	0.00020852	0.00310544
ENSG000001	2132.40789	-0.0640424	0.03606997	-1.7755053	0.07581449	0.18716398
ENSG000001	2847.77286	0.38689549	0.12713322	3.04322891	0.00234054	0.01762673
ENSG000001	540.358969	0.25975581	0.09332789	2.78326014	0.00538156	0.03143766
ENSG000001	19474.7977	0.23436638	0.12434026	1.88487928	0.05944614	0.15954451
ENSG000001	16911.1956	0.22526034	0.0938301	2.40072568	0.0163626	0.06702411
ENSG000001	227.805825	-0.2425218	0.11872408	-2.042735	0.04107868	0.1232962
ENSG000001	4745.22556	0.61400795	0.24362707	2.52027804	0.01172622	0.05343935
ENSG000001	271.767887	-0.1965341	0.11066287	-1.7759713	0.07573764	0.18714137
ENSG000001	1126.46195	0.36070704	0.17387955	2.07446502	0.03803614	0.11725487
ENSG000001	76.798407	0.92541852	0.22649546	4.08581484	4.39E-05	0.00093787
ENSG000001	1685.32985	-0.2290695	0.09649662	-2.3738601	0.01760322	0.07028001
ENSG000001	146.889478	0.51026026	0.16752379	3.04589728	0.00231987	0.01751332
ENSG000001	2345.73047	0.41112099	0.10579756	3.88592129	0.00010194	0.00183687
ENSG000001	69.8857424	0.87043936	0.20219035	4.30504893	1.67E-05	0.0004205
ENSG000001	413.450203	0.41818494	0.22195396	1.88410671	0.05955054	0.15962157
ENSG000001	227.459845	-0.2702529	0.10379444	-2.6037318	0.00922149	0.04541538
ENSG000001	32.2417691	0.79719675	0.33775983	2.36024736	0.01826275	0.07189781
ENSG000001	2695.42204	-0.1378925	0.07879379	-1.7500424	0.08011099	0.19420734
ENSG000001	21144.0589	2.64990984	0.38214351	6.93433157	4.08E-12	1.10E-09
ENSG000001	22647.0032	2.03688838	0.29127447	6.99302068	2.69E-12	7.74E-10
ENSG000001	74.3277494	1.45941582	0.30560312	4.77552651	1.79E-06	6.68E-05
ENSG000001	23860.0175	-0.189582	0.07925839	-2.391949	0.01675917	0.06816093
ENSG000001	384.127976	0.36862251	0.12033147	3.06339243	0.00218843	0.01676807
ENSG000001	2502.28023	-0.1876334	0.05204599	-3.6051457	0.00031198	0.00410884
ENSG000001	237.309972	-0.5411886	0.202316	-2.6749667	0.00747367	0.03925232
ENSG000001	7228.11718	-0.1265788	0.07202088	-1.7575298	0.07882756	0.19197365
ENSG000001	932.176777	0.25649379	0.08756655	2.92912991	0.00339912	0.02295759
ENSG000001	33471.4667	0.26230155	0.10671006	2.45807712	0.01396832	0.06004233
ENSG000001	713.931248	0.38196019	0.16458934	2.32068598	0.0203038	0.07745356
ENSG000001	1505.16181	0.19982229	0.06282374	3.18068144	0.00146929	0.01278767
ENSG000001	69.0961822	0.99711418	0.18775956	5.31059082	1.09E-07	6.84E-06
ENSG000001	5720.13336	0.90221609	0.27288738	3.30618473	0.00094576	0.00935065
ENSG000001	1013.92195	-0.311449	0.12135693	-2.5663879	0.01027638	0.04875632
ENSG000001	347.794945	0.3483258	0.14487035	2.40439685	0.01619918	0.0665709
ENSG000001	516.152744	-0.2593794	0.12233847	-2.1201785	0.03399099	0.1092668
ENSG000001	881.862548	0.32606778	0.11170992	2.91887943	0.00351292	0.02340874
ENSG000001	1320.0593	-0.1983547	0.0860532	-2.3050235	0.02116526	0.07976251
ENSG000001	2850.65053	0.09267798	0.03539915	2.6180852	0.00884247	0.0440164

ENSG000001	5997.65151	0.48947896	0.16447839	2.97594683	0.00292085	0.02058155
ENSG000001	6374.90711	0.19991817	0.09668742	2.06767515	0.03867058	0.1185519
ENSG000001	55.1115758	0.82485426	0.21663108	3.80764506	0.0001403	0.00233074
ENSG000001	11281.4803	0.30615189	0.13736007	2.22882735	0.02582539	0.0909491
ENSG000001	378.527309	-0.3547576	0.19256636	-1.8422615	0.06543691	0.16991572
ENSG000001	271.34688	-0.3850273	0.16588646	-2.3210289	0.02028528	0.07740698
ENSG000001	8670.93315	0.28799569	0.16133153	1.78511727	0.0742423	0.1848392
ENSG000001	17327.9745	0.31704269	0.14278961	2.22034853	0.02639512	0.0921637
ENSG000001	1573.25266	0.47557988	0.11924815	3.98815321	6.66E-05	0.00129215
ENSG000001	3516.98847	0.27769179	0.07875451	3.52604302	0.00042182	0.00522004
ENSG000001	578.952261	-0.3108525	0.15028342	-2.0684418	0.0385985	0.11843181
ENSG000001	353.167438	0.42438015	0.11831447	3.58688303	0.00033465	0.00435629
ENSG000001	870.246094	1.01290819	0.27177308	3.72703648	0.00019374	0.00296346
ENSG000001	28694.896	0.31997501	0.16074451	1.99058131	0.04652694	0.13462188
ENSG000001	373.686663	1.51469315	0.30558934	4.95662951	7.17E-07	3.17E-05
ENSG000001	1632.9164	0.77267412	0.16096145	4.80036757	1.58E-06	6.01E-05
ENSG000001	1403.5916	-0.0891133	0.04778791	-1.8647671	0.06221406	0.16432654
ENSG000001	8360.12688	0.75411816	0.21244478	3.54971382	0.00038565	0.00487173
ENSG000001	9095.2194	-0.2485605	0.10204012	-2.4359096	0.0148544	0.06256837
ENSG000001	1283.96892	-0.4676943	0.19738084	-2.3695018	0.01781207	0.07075205
ENSG000001	204.553659	0.43924325	0.17211445	2.5520417	0.01070937	0.05017187
ENSG000001	175.42015	-0.4356451	0.2305717	-1.8894129	0.05883652	0.15836219
ENSG000001	2408.78576	0.36135491	0.15252699	2.36912107	0.01783042	0.07078765
ENSG000001	83.286858	0.83215108	0.15957399	5.21482911	1.84E-07	1.05E-05
ENSG000001	55.7489665	-0.4269928	0.20570641	-2.0757388	0.03791811	0.11710123
ENSG000001	14505.7711	0.13540537	0.07193025	1.88245364	0.05977445	0.15992504
ENSG000001	4344.53763	0.70934875	0.33135071	2.14077931	0.03229184	0.10560217
ENSG000001	45029.4635	0.46197731	0.22582679	2.04571523	0.0407844	0.12277249
ENSG000001	34393.3843	0.42070402	0.16620373	2.53125501	0.01136552	0.05231904
ENSG000001	16697.687	0.59846824	0.17547729	3.41051676	0.0006484	0.0071788
ENSG000001	203.697172	0.60116332	0.13899508	4.32506904	1.52E-05	0.00039251
ENSG000001	4059.47611	0.21660092	0.12146816	1.78319091	0.07455523	0.18528071
ENSG000001	13015.9579	0.55932352	0.10419148	5.36822706	7.95E-08	5.24E-06
ENSG000001	2382.11697	-0.1505507	0.07758139	-1.9405517	0.05231268	0.14597773
ENSG000001	456.272642	-0.7266117	0.18091907	-4.0162254	5.91E-05	0.00118672
ENSG000001	13806.8686	0.24800313	0.10546422	2.35153804	0.01869598	0.07307698
ENSG000001	67.5466207	-0.5278833	0.23111542	-2.284068	0.02236753	0.08249223
ENSG000001	4005.69992	0.35909322	0.09253542	3.88060312	0.0001042	0.00186521
ENSG000001	707.322345	0.22500665	0.07326184	3.07126672	0.00213153	0.01649393
ENSG000001	175.307021	-0.5216963	0.19190047	-2.7185773	0.00655633	0.0358706
ENSG000001	1050.93231	0.19208995	0.08192079	2.34482526	0.019036	0.07394771
ENSG000001	19235.812	0.23960347	0.12304622	1.94726394	0.0515031	0.14430901
ENSG000001	13369.019	0.43817673	0.12189693	3.59464944	0.00032483	0.004244

ENSG000001	1106.36423	0.26389782	0.12119099	2.17753654	0.02944056	0.09920609
ENSG000001	565.265602	-0.1804692	0.06265864	-2.8801967	0.00397427	0.02545552
ENSG000001	4881.32666	0.17978339	0.08263607	2.17560435	0.02958486	0.09948788
ENSG000001	237.521921	0.1800891	0.09229126	1.95131256	0.05101988	0.14354374
ENSG000001	1263.38294	0.36556664	0.15338624	2.38330797	0.01715783	0.06918743
ENSG000001	654.26863	0.58793547	0.1516497	3.87693131	0.00010578	0.00188329
ENSG000001	6852.37883	-0.1440393	0.0491871	-2.9283965	0.00340715	0.02297633
ENSG000001	108.348303	1.38169859	0.27702302	4.987667	6.11E-07	2.80E-05
ENSG000001	4157.0981	0.31092219	0.1048442	2.96556417	0.00302128	0.02108084
ENSG000001	5126.09922	0.71356922	0.12639232	5.64566932	1.65E-08	1.32E-06
ENSG000001	255.470109	-0.4961265	0.18690863	-2.65438	0.00794543	0.04086948
ENSG000001	116.57544	-0.6417421	0.28211453	-2.2747572	0.0229205	0.08385261
ENSG000001	1778.58967	-0.1445297	0.08131293	-1.7774499	0.07549425	0.18673696
ENSG000001	1758.53271	0.28593792	0.14220117	2.0107987	0.04434672	0.13003455
ENSG000001	196.270091	0.68305899	0.1536474	4.44562672	8.76E-06	0.00025343
ENSG000001	5139.74676	-0.1167005	0.0583038	-2.0015931	0.04532851	0.13207127
ENSG000001	1961.10719	-0.1524785	0.074734	-2.0402829	0.04132216	0.12386827
ENSG000001	80.8602953	0.39442834	0.18440864	2.13888213	0.03244522	0.10588728
ENSG000001	797.819952	0.18549849	0.08265782	2.24417357	0.02482124	0.08851283
ENSG000001	6886.34167	0.12130291	0.0575477	2.10786707	0.03504249	0.1114295
ENSG000001	6639.54688	0.2104133	0.1043217	2.0169658	0.04369908	0.12871302
ENSG000001	15000.7191	-0.2087967	0.11018017	-1.8950475	0.05808611	0.15672839
ENSG000001	2336.67567	0.2221556	0.08457693	2.62666904	0.00862252	0.04335584
ENSG000001	339.185793	-0.3755467	0.12785026	-2.9373951	0.00330982	0.0225285
ENSG000001	1790.3099	0.78026989	0.21781293	3.58229377	0.00034059	0.00440431
ENSG000001	2082.86767	0.16834928	0.05015419	3.35663456	0.00078897	0.0082769
ENSG000001	2274.80283	-0.3733174	0.15512076	-2.4066241	0.01610073	0.06631593
ENSG000001	1832.02508	-0.1790313	0.08891988	-2.0134001	0.04407256	0.12953177
ENSG000001	4463.95353	0.51663129	0.18502498	2.79222463	0.0052347	0.03093178
ENSG000001	20840.4218	0.54661577	0.15299151	3.57285028	0.00035312	0.00453766
ENSG000001	4371.3776	0.09589724	0.04106413	2.3353042	0.01952754	0.07523332
ENSG000001	2744.49525	0.14382959	0.074448	1.93194686	0.05336607	0.14775625
ENSG000001	980.436581	-0.1596435	0.07367724	-2.1667954	0.03025046	0.1010708
ENSG000001	322.078109	0.49475198	0.16805243	2.94403343	0.00323965	0.02218903
ENSG000001	370.411514	-0.1773743	0.07838633	-2.2628217	0.02364668	0.08594098
ENSG000001	5430.83909	0.12386763	0.06759281	1.83255639	0.06686857	0.17231556
ENSG000001	1872.45806	0.19536592	0.05662667	3.45006881	0.00056044	0.00638272
ENSG000001	3119.05648	0.27153669	0.14954896	1.81570431	0.06941574	0.17682305
ENSG000001	1757.36855	0.16863275	0.09184416	1.83607493	0.06634658	0.17145545
ENSG000001	1485.65253	0.21116225	0.11573767	1.82449018	0.068078	0.17452704
ENSG000001	21368.8604	0.32482284	0.10522064	3.0870639	0.00202144	0.01590479
ENSG000001	3570.17521	-0.1696547	0.07361913	-2.304492	0.02119504	0.07983806
ENSG000001	2445.11524	-0.1581574	0.09075233	-1.742737	0.08137956	0.19631907

ENSG000001	6482.32817	0.110746	0.05131346	2.1582254	0.03091031	0.10251545
ENSG000001	162.20564	0.77674944	0.22747399	3.41467368	0.00063858	0.0070917
ENSG000001	8741.58363	0.37625104	0.11946496	3.14946773	0.00163568	0.01378934
ENSG000001	11814.2249	0.49096423	0.16103959	3.04871757	0.0022982	0.01740585
ENSG000001	402.180829	0.33429781	0.12201395	2.73983273	0.00614705	0.03426189
ENSG000001	1126.85932	0.47274468	0.12761844	3.7043601	0.00021193	0.00313906
ENSG000001	503.612395	0.60182128	0.15773107	3.81548967	0.00013591	0.00228103
ENSG000001	2419.24465	0.33374515	0.12788643	2.6096994	0.00906218	0.04485137
ENSG000001	2461.72911	0.63941723	0.23757959	2.69138119	0.00711568	0.03806304
ENSG000001	1510.04673	0.13748143	0.06834427	2.01160138	0.04426197	0.12986401
ENSG000001	572.563095	-0.1760132	0.07980061	-2.2056622	0.02740766	0.09449188
ENSG000001	1218.62041	0.11217133	0.06177679	1.81575202	0.06940842	0.17682305
ENSG000001	17241.3857	0.45218086	0.10582811	4.27278591	1.93E-05	0.000476
ENSG000001	2593.07399	0.42619932	0.18057364	2.36025218	0.01826252	0.07189781
ENSG000001	6666.33728	0.41122449	0.17802577	2.30991556	0.02089283	0.07906294
ENSG000001	102.043299	0.46071986	0.16979802	2.71334058	0.00666086	0.03623694
ENSG000001	79.3667348	-0.6582116	0.20572234	-3.1995145	0.00137659	0.01219448
ENSG000001	11026.505	0.21367026	0.10853687	1.96864211	0.0489942	0.13947187
ENSG000001	686.745734	0.60695322	0.18444053	3.29077998	0.0009991	0.00974356
ENSG000001	2854.18326	0.07281655	0.04143139	1.75752146	0.07882897	0.19197365
ENSG000001	1004.7847	0.10559087	0.05618035	1.87949822	0.0601765	0.16070261
ENSG000001	5470.75189	0.66519647	0.13527102	4.9175092	8.77E-07	3.75E-05
ENSG000001	688.188239	-0.1843094	0.07090798	-2.5992764	0.00934205	0.04568495
ENSG000001	1379.16154	-0.1761801	0.09458701	-1.8626245	0.06251511	0.16461542
ENSG000001	806.002153	0.37724219	0.16380005	2.30306522	0.02127517	0.08001124
ENSG000001	450.988244	-0.3095336	0.16114472	-1.920842	0.05475163	0.1505555
ENSG000001	11322.8873	0.42639495	0.1606882	2.65355487	0.00796488	0.0409093
ENSG000001	314.461899	0.29271469	0.12993322	2.2528088	0.02427121	0.08735184
ENSG000001	167.515328	1.65953943	0.35273827	4.70473313	2.54E-06	8.87E-05
ENSG000001	1608.0881	0.15146676	0.07284137	2.07940569	0.03758008	0.11635207
ENSG000001	1907.9441	0.23454427	0.07760078	3.02244726	0.0025074	0.01842842
ENSG000001	1837.96261	0.48604952	0.18693578	2.6000882	0.00931998	0.04562458
ENSG000001	676.143389	-0.3593157	0.12306676	-2.9196816	0.00350389	0.02337401
ENSG000001	5638.38767	0.16458235	0.07625129	2.15842061	0.03089515	0.10248588
ENSG000001	23540.952	-0.2443378	0.06109204	-3.9995035	6.35E-05	0.00124648
ENSG000001	13622.7621	-0.1882424	0.05880483	-3.2011375	0.00136886	0.01214569
ENSG000001	1098.02118	0.17032306	0.08440687	2.01788132	0.04360362	0.12854524
ENSG000001	307.048705	-0.6236656	0.23751064	-2.6258426	0.00864348	0.04337763
ENSG000001	20444.298	0.17432513	0.08813173	1.97800641	0.04792798	0.13731874
ENSG000001	2754.35108	-0.1142363	0.06233504	-1.8326174	0.06685948	0.17231556
ENSG000001	150.7278	0.36047418	0.17552973	2.053636	0.04001094	0.12142501
ENSG000001	914.310044	0.29403424	0.08361173	3.51666249	0.00043701	0.00532258
ENSG000001	75.8380796	-0.7288734	0.26584564	-2.7417166	0.0061119	0.03413145

ENSG000001	889.068896	0.47721134	0.1671012	2.85582231	0.00429255	0.02688347
ENSG000001	382.407989	0.51019666	0.11762609	4.33744476	1.44E-05	0.00037637
ENSG000001	428.130769	-0.1561154	0.07155712	-2.1816886	0.02913252	0.09849512
ENSG000001	126.243694	0.65724783	0.2122846	3.09606933	0.00196105	0.01557518
ENSG000001	4282.4685	0.30719867	0.10281912	2.98775826	0.00281032	0.02005256
ENSG000001	1276.1944	5.39432122	0.57746876	9.34132127	9.51E-21	7.80E-17
ENSG000001	3273.65334	0.51407256	0.11800027	4.35653723	1.32E-05	0.00035307
ENSG000001	31.8179919	1.62168695	0.27017521	6.00235291	1.94E-09	2.20E-07
ENSG000001	24441.3348	0.2242524	0.08541702	2.6253831	0.00865515	0.04338536
ENSG000001	1212.047	0.22167982	0.09979171	2.22142517	0.02632218	0.09198737
ENSG000001	8121.85872	1.54633405	0.20656762	7.48584906	7.11E-14	4.48E-11
ENSG000001	3428.09537	0.17630315	0.0530043	3.32620475	0.00088037	0.00891628
ENSG000001	1027.6934	0.27150996	0.0717395	3.78466499	0.00015392	0.0025112
ENSG000001	535.422907	0.26597851	0.11896126	2.23584132	0.02536217	0.08976116
ENSG000001	2218.77963	0.34205237	0.10426515	3.28060103	0.00103586	0.00998255
ENSG000001	2595.38908	0.5345152	0.13573806	3.93784311	8.22E-05	0.00154247
ENSG000001	1680.84247	-0.1247134	0.07158501	-1.7421724	0.08147828	0.19652829
ENSG000001	2319.7664	-0.1941371	0.08299656	-2.3390987	0.01933033	0.07466652
ENSG000001	603.00062	0.63604435	0.16171191	3.93319423	8.38E-05	0.00156724
ENSG000001	42001.2236	1.08173199	0.16380471	6.6037906	4.01E-11	7.64E-09
ENSG000001	13575.563	0.19649864	0.0701309	2.80188382	0.00508052	0.03032041
ENSG000001	328.249662	-0.4312098	0.1512017	-2.8518843	0.00434609	0.02708585
ENSG000001	1908.92316	0.19068546	0.05103742	3.73618952	0.00018683	0.00288189
ENSG000001	485.809798	-0.2437769	0.08092845	-3.0122519	0.00259317	0.01890629
ENSG000001	678.703928	0.28182382	0.12232729	2.30385084	0.02123102	0.07991691
ENSG000001	2215.20333	0.25455668	0.10090545	2.52272493	0.01164495	0.05327627
ENSG000001	835.650796	0.18864715	0.0967767	1.94930336	0.05125921	0.1439454
ENSG000001	10371.1104	-0.1647645	0.05569161	-2.9585156	0.00309125	0.02144127
ENSG000001	4300.17887	0.207122	0.09696799	2.13598328	0.03268078	0.10636496
ENSG000001	727.117026	-0.211503	0.10042671	-2.1060432	0.0352006	0.11170127
ENSG000001	12089.1903	1.67611189	0.38581896	4.34429633	1.40E-05	0.00036833
ENSG000001	5730.5413	1.70448539	0.29730911	5.73304133	9.86E-09	8.51E-07
ENSG000001	9624.15339	0.91289624	0.26410394	3.45657936	0.00054708	0.00628391
ENSG000001	3975.32574	0.49076828	0.18013989	2.7243731	0.00644237	0.03541251
ENSG000001	2675.48332	-0.2262503	0.08748789	-2.5860754	0.00970757	0.04684373
ENSG000001	44.6250437	0.45689706	0.18733253	2.43896271	0.01472949	0.06224204
ENSG000001	1848.0881	0.42384275	0.11204476	3.78279863	0.00015507	0.00252008
ENSG000001	13579.0878	0.20526019	0.0986802	2.0800544	0.03752054	0.11625555
ENSG000001	1851.05934	0.25325276	0.11837701	2.13937444	0.03240536	0.10583687
ENSG000001	12446.7409	0.54268618	0.17592826	3.08470159	0.00203756	0.01600093
ENSG000001	40012.4653	-0.1157002	0.05099455	-2.2688729	0.02327605	0.08483853
ENSG000001	788.613757	-0.177365	0.0774266	-2.2907503	0.02197786	0.08188387
ENSG000001	4910.28326	-0.1586209	0.05434568	-2.9187397	0.0035145	0.02340874

ENSG000001	7644.26566	0.57401163	0.22623324	2.53725591	0.01117253	0.05164812
ENSG000001	2886.84869	0.16554194	0.08356771	1.98093185	0.04759892	0.13671036
ENSG000001	5303.27911	-0.1466413	0.06192999	-2.3678555	0.01789152	0.07087878
ENSG000001	58.104136	2.63391996	0.46301815	5.68858904	1.28E-08	1.07E-06
ENSG000001	2714.27492	0.27690986	0.11293415	2.45195866	0.0142081	0.06069772
ENSG000001	5523.48876	0.15703914	0.08093405	1.94033468	0.05233903	0.14597773
ENSG000001	6861.83934	-0.1261504	0.05318133	-2.3720809	0.01768822	0.07050687
ENSG000001	11031.1008	-0.2942312	0.09325004	-3.1552932	0.00160337	0.01363612
ENSG000001	891.108202	0.20471182	0.09874336	2.07317046	0.03815642	0.11753725
ENSG000001	1473.67809	-0.3487955	0.08412818	-4.1460011	3.38E-05	0.00076837
ENSG000001	260.205256	0.3068038	0.17515811	1.75158204	0.0798457	0.19378301
ENSG000001	2644.96711	0.25580452	0.12903576	1.98243126	0.04743099	0.13634748
ENSG000001	2034.25623	0.25704785	0.10752086	2.39067883	0.01681726	0.06823869
ENSG000001	7801.4696	0.17715321	0.0895916	1.97734171	0.04800302	0.13748567
ENSG000001	4124.21422	-0.153945	0.03950923	-3.896431	9.76E-05	0.00177396
ENSG000001	1923.97444	-0.3957652	0.14227766	-2.7816396	0.00540851	0.03150383
ENSG000001	8809.56058	-0.2539151	0.10476293	-2.4237112	0.01536282	0.06413038
ENSG000001	1315.10069	-0.3012932	0.12396481	-2.4304738	0.0150791	0.06321962
ENSG000001	1272.05888	-0.1636169	0.07203733	-2.2712799	0.02313004	0.08448339
ENSG000001	8886.61233	0.25177008	0.12320687	2.04347439	0.0410055	0.12316673
ENSG000001	273.25339	0.1787835	0.06798383	2.62979455	0.00854365	0.0431422
ENSG000001	3356.77345	-0.21902	0.09370096	-2.3374365	0.0194165	0.0749162
ENSG000001	2191.042	0.2804661	0.12812465	2.18900969	0.02859614	0.09719959
ENSG000001	6116.18162	0.19788604	0.10665948	1.8553067	0.06355244	0.16654457
ENSG000001	509.657208	-0.2024837	0.0655315	-3.0898685	0.00200245	0.01580086
ENSG000001	5723.83548	-0.4986306	0.13340933	-3.7375993	0.00018579	0.0028739
ENSG000001	462.617079	-0.4356177	0.15104756	-2.8839773	0.00392687	0.02528633
ENSG000001	8961.71104	0.18029855	0.07618397	2.3666207	0.01795132	0.07104702
ENSG000001	810.514357	-0.1486157	0.08484733	-1.7515657	0.07984851	0.19378301
ENSG000001	15422.1313	0.43600681	0.14964606	2.91358698	0.00357302	0.02370978
ENSG000001	3230.11349	0.29476096	0.12427171	2.37190725	0.01769653	0.07051164
ENSG000001	18581.2056	-0.0944264	0.04181314	-2.2582937	0.02392735	0.08665281
ENSG000001	1041.9108	0.2002593	0.06728106	2.97645863	0.00291598	0.02056766
ENSG000001	1148.47465	-0.286819	0.10237229	-2.8017252	0.00508301	0.03032041
ENSG000001	1454.71322	1.27873195	0.17341734	7.37372619	1.66E-13	8.78E-11
ENSG000001	1954.34881	0.4704177	0.14592368	3.22372421	0.00126535	0.01150698
ENSG000001	973.476633	0.3317571	0.08242898	4.02476274	5.70E-05	0.00115738
ENSG000001	1219.30058	0.52214034	0.25939553	2.01291181	0.04412391	0.1295673
ENSG000001	3631.05876	0.24833166	0.09812805	2.53068989	0.01138384	0.05238481
ENSG000001	10154.9758	0.32977028	0.16878316	1.95381027	0.05072366	0.14307829
ENSG000001	3121.88798	0.59060899	0.14040602	4.20643638	2.59E-05	0.0006165
ENSG000001	2744.41973	-0.2156375	0.0749094	-2.878644	0.00399389	0.02555122
ENSG000001	562.698757	1.15880725	0.26214566	4.42047091	9.85E-06	0.00028036

ENSG000001	4211.34305	-0.6254828	0.22967381	-2.7233525	0.00646231	0.03546855
ENSG000001	6365.18294	-0.1510396	0.04693811	-3.2178462	0.00129157	0.01170695
ENSG000001	34104.9671	0.47217125	0.20015503	2.35902765	0.01832289	0.07206535
ENSG000001	6678.66036	0.19305919	0.08712579	2.21586724	0.0267006	0.09293349
ENSG000001	772.416163	0.24100566	0.0656207	3.67270773	0.00023999	0.00340414
ENSG000001	10031.381	-0.1145743	0.06096561	-1.8793265	0.06019992	0.16073899
ENSG000001	2055.61016	0.2475912	0.0736569	3.36141236	0.00077545	0.00817162
ENSG000001	2651.96053	0.33313737	0.15512838	2.14749464	0.03175392	0.10436342
ENSG000001	2711.44918	0.29654341	0.13312022	2.22763607	0.02590479	0.09110795
ENSG000001	1398.16117	-0.1862615	0.09149608	-2.0357319	0.04177728	0.12465632
ENSG000001	5034.6081	0.18042423	0.09427109	1.91388722	0.05563457	0.15185742
ENSG000001	1755.43997	0.27042102	0.0824105	3.28139048	0.00103297	0.009975
ENSG000001	730.176405	0.22022902	0.08128186	2.70944858	0.00673952	0.03656778
ENSG000001	1745.59126	0.18508613	0.09371764	1.97493362	0.04827568	0.1379774
ENSG000001	942.818446	0.30367392	0.10760436	2.82213406	0.00477052	0.02903573
ENSG000001	6649.59808	-0.1861769	0.08715078	-2.1362622	0.03265804	0.10631208
ENSG000001	1122.16914	-0.2623812	0.14523784	-1.8065624	0.07083053	0.17898108
ENSG000001	6523.885	0.63685756	0.23114294	2.75525424	0.00586465	0.03314813
ENSG000001	359.895034	1.31957129	0.21268793	6.20426025	5.50E-10	6.99E-08
ENSG000001	955.533181	-0.3779683	0.1170332	-3.2295819	0.00123971	0.01133719
ENSG000001	3198.82329	-0.0621449	0.0306323	-2.0287375	0.04248503	0.1261777
ENSG000001	1653.42975	-0.3107723	0.13042081	-2.3828425	0.01717954	0.06921407
ENSG000001	3235.29568	0.22659894	0.09021092	2.51187913	0.01200902	0.05441472
ENSG000001	1017.83011	0.33013116	0.11410115	2.89332028	0.00381192	0.02477884
ENSG000001	3618.69845	0.1494197	0.06650031	2.24690219	0.02464628	0.08819839
ENSG000001	1028.76106	0.26552047	0.14820435	1.79158354	0.0731997	0.18310744
ENSG000001	3322.25441	0.84075173	0.1246331	6.7458141	1.52E-11	3.20E-09
ENSG000001	13464.5304	1.5243153	0.20026426	7.61151942	2.71E-14	2.04E-11
ENSG000001	3859.40177	0.12290045	0.06291497	1.95343746	0.05076778	0.14307998
ENSG000001	8554.00605	0.25319205	0.13582423	1.86411539	0.0623055	0.16436541
ENSG000001	1386.1087	0.18130757	0.05566671	3.25701968	0.00112589	0.01056735
ENSG000001	5689.82462	0.16406197	0.06005703	2.73176959	0.00629952	0.03485929
ENSG000001	7316.90716	0.97390903	0.23609941	4.12499555	3.71E-05	0.00082484
ENSG000001	185.772292	0.65566732	0.18901157	3.46892683	0.00052254	0.00609787
ENSG000001	11944.3524	0.27917711	0.13156779	2.12192595	0.03384396	0.10898148
ENSG000001	87.9736559	0.49499045	0.15116727	3.27445518	0.00105866	0.01013929
ENSG000001	644.984477	-0.1856136	0.10100872	-1.8376	0.06612136	0.17106216
ENSG000001	1872.982	0.12565432	0.06113565	2.05533624	0.03984654	0.12101569
ENSG000001	2373.63437	0.31001154	0.11171468	2.77502951	0.00551967	0.03188733
ENSG000001	1805.46727	0.33704816	0.09049423	3.7245266	0.00019568	0.0029792
ENSG000001	107.386746	0.27810836	0.11150719	2.49408461	0.01262824	0.05629835
ENSG000001	955.881687	-0.1297234	0.05645271	-2.2979123	0.02156678	0.0809038
ENSG000001	5118.74828	0.16975914	0.08846492	1.91894298	0.05499155	0.15099555

ENSG000001	7823.30837	0.19136174	0.07027583	2.72300936	0.00646902	0.03548764
ENSG000001	19349.8716	-0.0952396	0.0533192	-1.7862162	0.07406426	0.1845641
ENSG000001	315.244992	0.39779748	0.15380928	2.58630352	0.00970115	0.04683335
ENSG000001	2138.04862	-0.2304647	0.10753236	-2.1432129	0.03209601	0.10515049
ENSG000001	4031.13259	-0.4572234	0.13834053	-3.3050576	0.00094957	0.00937957
ENSG000001	2659.42156	0.27521186	0.09668109	2.84659455	0.00441896	0.0274461
ENSG000001	16081.1136	0.20772759	0.10125237	2.0515824	0.04021026	0.12173702
ENSG000001	1178.4173	0.40439976	0.13541417	2.98639178	0.00282291	0.02011613
ENSG000001	651.572375	1.14786629	0.22070647	5.20087287	1.98E-07	1.10E-05
ENSG000001	214.164208	0.78695629	0.25667061	3.06601637	0.00216931	0.01666833
ENSG000001	6921.31265	0.19935324	0.07819269	2.54951248	0.01078737	0.05043639
ENSG000001	589.312434	0.15676616	0.07485659	2.0942198	0.0362404	0.11372898
ENSG000001	4459.07365	0.4222848	0.1340592	3.1499874	0.00163278	0.01377192
ENSG000001	8986.34658	0.48208955	0.16084232	2.99728054	0.002724	0.01960754
ENSG000001	8991.03356	-0.2626669	0.09882293	-2.6579546	0.00786165	0.04054969
ENSG000001	1512.63949	2.71871134	0.4950732	5.49153405	3.98E-08	2.89E-06
ENSG000001	149.640057	0.86353777	0.13858533	6.23109066	4.63E-10	6.08E-08
ENSG000001	460.521267	1.51231929	0.29660338	5.09879319	3.42E-07	1.76E-05
ENSG000001	467.059507	-0.2190923	0.12564341	-1.7437628	0.08120045	0.19600233
ENSG000001	2271.69094	-0.1880349	0.08438588	-2.2282743	0.02586223	0.0910518
ENSG000001	137.655066	-0.2552016	0.12301587	-2.0745424	0.03802896	0.11725478
ENSG000001	934.609407	0.25114379	0.11773357	2.13315365	0.03291212	0.10692691
ENSG000001	659.378176	-0.3285449	0.10476353	-3.1360613	0.00171233	0.0142596
ENSG000001	79.806603	0.70085538	0.32362982	2.16560818	0.03034114	0.1012421
ENSG000001	37.7168684	1.30039947	0.37382435	3.47863764	0.00050397	0.00593649
ENSG000001	3163.85234	0.52744505	0.17044327	3.09454903	0.00197112	0.01562132
ENSG000001	2917.44498	0.38411476	0.10627156	3.61446411	0.00030097	0.00399919
ENSG000001	2338.40684	0.32118903	0.14547007	2.20793886	0.02724854	0.09420077
ENSG000001	680.660428	0.8128709	0.17197066	4.72679991	2.28E-06	8.09E-05
ENSG000001	1847.64732	2.05036071	0.40965761	5.00505953	5.58E-07	2.60E-05
ENSG000001	38611.748	0.42164742	0.15297596	2.75629849	0.00584596	0.03308811
ENSG000001	8925.83549	0.29178957	0.0738053	3.95350446	7.70E-05	0.00146783
ENSG000001	4952.5161	-0.224994	0.10119616	-2.2233453	0.02619252	0.09167104
ENSG000001	424.99625	0.19992817	0.10578613	1.88992803	0.05876759	0.15828531
ENSG000001	10736.3919	0.35906042	0.13362559	2.68706334	0.00720833	0.03839992
ENSG000001	25794.808	0.73430991	0.26454085	2.77579028	0.00550677	0.03188569
ENSG000001	658.930586	-0.3507112	0.10423344	-3.3646704	0.00076635	0.00811224
ENSG000001	7001.54994	0.12749651	0.06538852	1.94983007	0.05119638	0.14382551
ENSG000001	1155.64316	0.40699162	0.08443255	4.82031672	1.43E-06	5.60E-05
ENSG000001	432.664071	0.58816498	0.2790718	2.10757582	0.0350677	0.1114665
ENSG000001	50.3267283	0.53805083	0.19149765	2.80969938	0.00495878	0.02973998
ENSG000001	145.94221	-0.4373507	0.1179119	-3.7091314	0.00020797	0.00310292
ENSG000001	8433.91263	0.20057007	0.11115363	1.80444007	0.07116235	0.17957048

ENSG000001	1120.77082	0.4906174	0.1944639	2.5229228	0.01163839	0.05327604
ENSG000001	8794.40963	0.32666345	0.15894845	2.0551534	0.03986419	0.12104688
ENSG000001	9511.84495	-0.2631419	0.11974705	-2.1974815	0.02798607	0.0958412
ENSG000001	2441.22032	0.2131565	0.08849903	2.40857431	0.01601497	0.06607396
ENSG000001	357.074048	0.27823931	0.12727135	2.18618958	0.02880174	0.09767572
ENSG000001	1863.45326	0.84433794	0.21969659	3.84319996	0.00012144	0.00210048
ENSG000001	262.711355	-0.2659826	0.13972859	-1.9035662	0.0569667	0.15452159
ENSG000001	427.955554	0.51282243	0.22385979	2.29081971	0.02197384	0.08188387
ENSG000001	4551.17036	0.45203824	0.21393487	2.11297129	0.03460323	0.11055906
ENSG000001	1902.51337	0.42201512	0.16544117	2.55084714	0.01074614	0.05030104
ENSG000001	429.022104	-0.1567574	0.08677083	-1.8065676	0.07082973	0.17898108
ENSG000001	4618.87629	-0.2114782	0.08726963	-2.4232741	0.01538131	0.06417491
ENSG000001	244.495598	0.26578806	0.12703019	2.09232195	0.03640973	0.11396472
ENSG000001	2562.32271	0.31982112	0.10708016	2.98674486	0.00281965	0.02010169
ENSG000001	2897.09953	0.16597038	0.05821296	2.85108988	0.00435697	0.02713299
ENSG000001	939.752378	-0.2156691	0.11772507	-1.8319722	0.06695557	0.17245844
ENSG000001	1533.1338	0.15410755	0.07232243	2.1308403	0.0331023	0.10741209
ENSG000001	2255.77127	-0.2039256	0.06845438	-2.979	0.00289191	0.02046552
ENSG000001	218.332044	-0.3256189	0.13875529	-2.3467132	0.01893983	0.07373999
ENSG000001	3018.34269	0.40712929	0.13110783	3.10530106	0.00190085	0.0153086
ENSG000001	78011.3262	0.29093399	0.16499894	1.76324763	0.07785873	0.19031748
ENSG000001	2218.25191	0.17909532	0.05519139	3.24498652	0.00117456	0.01089326
ENSG000001	5443.95478	-0.2982892	0.07964093	-3.7454259	0.00018009	0.00279366
ENSG000001	396.432112	-0.3994916	0.12364497	-3.2309572	0.00123376	0.01132067
ENSG000001	180.962738	0.51554712	0.14645294	3.52022379	0.00043118	0.0052989
ENSG000001	1153.84126	0.82609583	0.30418868	2.71573492	0.00661288	0.03604769
ENSG000001	247.515824	-0.1743084	0.07935652	-2.1965232	0.02805451	0.09599538
ENSG000001	3911.16659	0.33298109	0.1031382	3.22849428	0.00124444	0.01136138
ENSG000001	805.650738	0.31586463	0.14665108	2.15385143	0.03125182	0.10329292
ENSG000001	127.015149	-0.2446616	0.1240916	-1.9716212	0.04865286	0.13883762
ENSG000001	799.104341	-0.2124837	0.08478089	-2.5062694	0.01220126	0.05502313
ENSG000001	363.574824	-0.1803768	0.07909761	-2.2804324	0.02258206	0.08302197
ENSG000001	225.51134	-0.3402098	0.08601564	-3.9552093	7.65E-05	0.00146296
ENSG000001	464.723259	-0.2137174	0.10981911	-1.9460857	0.05164445	0.14458153
ENSG000001	119.207982	0.39559408	0.22846116	1.73155947	0.08335203	0.19973865
ENSG000001	7728.52235	0.47565117	0.16405617	2.89931893	0.00373974	0.0244695
ENSG000001	9537.66943	0.20130551	0.08612663	2.3373202	0.01942254	0.0749162
ENSG000001	1519.02714	0.45069616	0.18367562	2.45376156	0.01413707	0.06049204
ENSG000001	4357.65959	-0.5043081	0.16422777	-3.0707846	0.00213497	0.01651279
ENSG000001	1235.74452	0.18933671	0.06371688	2.9715314	0.00296319	0.02077517
ENSG000001	23434.2064	0.35539255	0.1353979	2.62480109	0.00866996	0.04340224
ENSG000001	43092.0173	2.64477615	0.35953868	7.35602671	1.89E-13	9.22E-11
ENSG000001	50538.6852	2.04312823	0.31371806	6.5126254	7.38E-11	1.36E-08

ENSG000001	937.908119	-0.1764392	0.09273555	-1.9026063	0.05709193	0.15481295
ENSG000001	566.812738	-0.2466068	0.09417601	-2.618574	0.00882981	0.04398009
ENSG000001	615.014027	-0.2070835	0.09261926	-2.2358581	0.02536107	0.08976116
ENSG000001	3690.76387	0.27418016	0.12107404	2.26456608	0.02353932	0.08567713
ENSG000001	5992.18798	-0.2431229	0.07084586	-3.4317166	0.00059977	0.00672214
ENSG000001	376.098087	0.4873998	0.18121145	2.6896744	0.00715218	0.03815037
ENSG000001	17638.3588	0.1811672	0.10171456	1.78113341	0.07489066	0.18572022
ENSG000001	24625.2254	0.90846534	0.24574712	3.69674874	0.00021838	0.00319224
ENSG000001	1509.26199	0.345043	0.11625949	2.9678694	0.00299872	0.02095906
ENSG000001	403.330027	0.51607227	0.12463907	4.14053373	3.46E-05	0.0007815
ENSG000001	716.104929	-0.1860151	0.06300179	-2.9525358	0.00315175	0.02175971
ENSG000001	1957.16126	0.5351374	0.19809106	2.70147167	0.00690334	0.03723487
ENSG000001	2145.71599	-0.1153172	0.06120127	-1.8842281	0.05953413	0.15962157
ENSG000001	414.836137	0.39792543	0.18171901	2.18978433	0.02853988	0.09708889
ENSG000001	2497.50336	0.51567357	0.14732204	3.50031529	0.00046471	0.00556598
ENSG000001	1077.7581	-0.4481759	0.21615104	-2.0734388	0.03813146	0.11750453
ENSG000001	1964.668	0.69643738	0.13675975	5.09241475	3.54E-07	1.81E-05
ENSG000001	2928.14943	0.71175145	0.25125022	2.83283906	0.00461366	0.02828045
ENSG000001	2120.59782	0.08168994	0.04700911	1.73774691	0.08225542	0.19787883
ENSG000001	384.350753	0.20018332	0.09086341	2.20312353	0.02758604	0.09496711
ENSG000001	1139.90551	-0.3104704	0.10614734	-2.9249003	0.00344567	0.02315385
ENSG000001	256.533229	0.14203328	0.0610838	2.32522014	0.02006019	0.07678232
ENSG000001	925.61684	1.02009109	0.17770345	5.74041255	9.44E-09	8.19E-07
ENSG000001	15900.5881	0.4162587	0.15471533	2.69048129	0.0071349	0.03812735
ENSG000001	1443.40462	0.31795188	0.09640423	3.29811144	0.00097337	0.00955714
ENSG000001	800.862706	0.1436235	0.07661155	1.87469785	0.0608343	0.16182675
ENSG000001	632.833976	0.26830197	0.1024967	2.61766439	0.00885338	0.04405734
ENSG000001	18401.1442	0.42489676	0.09708118	4.37671622	1.20E-05	0.00032816
ENSG000001	198.730685	-0.2197926	0.10021519	-2.1932065	0.0282925	0.09648755
ENSG000001	4183.64586	0.20630605	0.07746301	2.66328462	0.00773819	0.04014981
ENSG000001	828.857498	0.17156043	0.07034045	2.43900088	0.01472793	0.06224204
ENSG000001	2383.27553	0.25941211	0.12858519	2.01743376	0.04365027	0.12861337
ENSG000001	9138.3475	0.2177288	0.09569683	2.27519354	0.02289432	0.08382255
ENSG000001	1177.89577	-0.165009	0.08739636	-1.8880536	0.05901875	0.15861834
ENSG000001	2421.84136	-0.2478136	0.0958632	-2.5850756	0.00973577	0.04695216
ENSG000001	535.516452	-0.2604213	0.11058603	-2.3549202	0.01852668	0.07263869
ENSG000001	867.16335	0.33098909	0.14473647	2.28683968	0.02220518	0.08222637
ENSG000001	177.960217	-0.2813603	0.14789644	-1.9024145	0.05711698	0.15485238
ENSG000001	10813.472	0.24559933	0.10881692	2.25699566	0.02400835	0.08684422
ENSG000001	1295.83622	-0.2132394	0.11330554	-1.8819854	0.059838	0.16005932
ENSG000001	3333.28844	-0.1472897	0.05359398	-2.7482499	0.00599143	0.03364436
ENSG000001	2948.27285	-0.1775578	0.06750177	-2.630417	0.00852802	0.04307884
ENSG000001	1036.53681	-0.2296302	0.10500814	-2.1867849	0.02875823	0.09756854

ENSG000001	699.785364	-0.1132096	0.06141212	-1.8434412	0.06526462	0.16972116
ENSG000001	21913.6796	0.64254088	0.17255758	3.72363167	0.00019638	0.00298701
ENSG000001	1418.12684	0.19582902	0.05875907	3.33274556	0.00085994	0.00878527
ENSG000001	4546.60394	0.69418733	0.19506343	3.55877741	0.00037259	0.00473221
ENSG000001	192.393855	1.30633587	0.24747596	5.27863744	1.30E-07	7.87E-06
ENSG000001	164.907317	1.75763831	0.24262264	7.24432948	4.35E-13	1.74E-10
ENSG000001	43.8203223	0.76777438	0.24306872	3.158672	0.0015849	0.01352111
ENSG000001	8742.43322	0.22666003	0.07988612	2.83728904	0.00454984	0.02802292
ENSG000001	1453.7761	-0.2765856	0.10449289	-2.6469317	0.00812258	0.04150605
ENSG000001	847.816874	-0.2773265	0.13257987	-2.0917691	0.03645918	0.1140642
ENSG000001	1835.79267	-0.1908766	0.06753957	-2.8261445	0.0047112	0.02877078
ENSG000001	721.824837	-0.3485118	0.08523596	-4.0887888	4.34E-05	0.00092945
ENSG000001	1812.23022	0.4160821	0.12627669	3.29500328	0.00098421	0.00964039
ENSG000001	1001.85405	0.6191794	0.24116614	2.56743917	0.01024528	0.04863688
ENSG000001	1604.35276	0.44405359	0.13821499	3.21277438	0.0013146	0.01184364
ENSG000001	271.489685	1.39392248	0.27458622	5.07644732	3.85E-07	1.93E-05
ENSG000001	1723.99427	0.11882993	0.05182335	2.29298059	0.02184912	0.08168264
ENSG000001	16230.9304	-0.3262318	0.14319943	-2.278164	0.02271681	0.08331191
ENSG000001	457.040395	0.28928678	0.16006862	1.80726729	0.07072061	0.17886872
ENSG000001	51.9721907	0.6492839	0.2945196	2.20455244	0.02748552	0.09470057
ENSG000001	204.659467	-0.2066025	0.09845774	-2.0983874	0.03587094	0.11304393
ENSG000001	477.017615	1.15793876	0.18409164	6.29001284	3.17E-10	4.49E-08
ENSG000001	8567.00396	0.18264024	0.10455133	1.74689538	0.08065549	0.19503142
ENSG000001	532.189787	0.26314376	0.0847874	3.10357154	0.001912	0.01536817
ENSG000001	18193.4134	0.90180347	0.1733168	5.20320849	1.96E-07	1.09E-05
ENSG000001	294.226529	-0.1921308	0.08571466	-2.2415159	0.02499268	0.0889913
ENSG000001	1164.01567	0.16669007	0.09193052	1.81321801	0.0697982	0.17729017
ENSG000001	160.896176	1.77432515	0.39206892	4.52554396	6.02E-06	0.00018602
ENSG000001	1040.46022	-0.1410531	0.0726014	-1.9428429	0.05203514	0.14547658
ENSG000001	1087.70926	0.20469678	0.1097325	1.86541614	0.0621231	0.16418351
ENSG000001	27508.3055	0.44769127	0.13147096	3.40524835	0.00066104	0.00727942
ENSG000001	1151.52926	-0.2772371	0.09587906	-2.8915294	0.00383372	0.02485658
ENSG000001	611.302458	0.63924512	0.26713247	2.39298923	0.01671173	0.06801248
ENSG000001	12620.435	0.68342476	0.28715858	2.37995588	0.01731471	0.06956468
ENSG000001	3632.51964	0.2715364	0.1116769	2.43144644	0.01503867	0.06314702
ENSG000001	159.339693	-0.6442595	0.18547527	-3.4735602	0.0005136	0.00601968
ENSG000001	2154.2246	-0.1863275	0.10760861	-1.7315292	0.08335742	0.19973865
ENSG000001	7617.20054	0.18916883	0.08211199	2.30379063	0.0212344	0.07991691
ENSG000001	2687.68594	0.33532348	0.1472836	2.27671971	0.02280297	0.08355313
ENSG000001	2506.31497	-0.2365668	0.10802884	-2.1898484	0.02853523	0.09708889
ENSG000001	2948.92619	-0.3418677	0.11951944	-2.8603525	0.0042317	0.02664641
ENSG000001	825.036716	0.16979004	0.07824043	2.17010608	0.02999881	0.10048836
ENSG000001	14374.9614	-0.1897551	0.1073714	-1.7672777	0.07718173	0.1892551

ENSG000001	104.606436	1.84142199	0.40300024	4.56928263	4.89E-06	0.0001572
ENSG000001	4974.07027	0.69772154	0.18837822	3.70383346	0.00021237	0.00314274
ENSG000001	2057.48913	-0.255654	0.09436769	-2.7091266	0.00674606	0.03657908
ENSG000001	4439.84561	0.16372754	0.05113679	3.20175661	0.00136592	0.01213932
ENSG000001	3651.40924	0.22255173	0.10414207	2.1370012	0.03259789	0.10620061
ENSG000001	161.713656	3.34154287	0.5014782	6.66338605	2.68E-11	5.29E-09
ENSG000001	327.739657	0.54391615	0.17909395	3.03704366	0.00238911	0.01787966
ENSG000001	3688.52028	0.81219521	0.1582888	5.13109732	2.88E-07	1.51E-05
ENSG000001	1599.62047	0.35216235	0.20026816	1.758454	0.07867029	0.19184366
ENSG000001	706.065605	0.35683769	0.12283651	2.90498076	0.00367276	0.02416155
ENSG000001	11812.999	0.42928188	0.11817262	3.63266785	0.00028051	0.00379807
ENSG000001	1217.6216	0.30069536	0.11560642	2.60102641	0.00929453	0.04558852
ENSG000001	2535.95176	0.17131996	0.0572489	2.99254617	0.00276661	0.01981829
ENSG000001	656.162433	-0.3278916	0.08218442	-3.9897054	6.62E-05	0.0012883
ENSG000001	9464.55091	1.31933791	0.19419093	6.79402433	1.09E-11	2.53E-09
ENSG000001	4660.68763	0.33016341	0.12456952	2.65043505	0.00803882	0.04121717
ENSG000001	3047.87493	0.53814418	0.12121255	4.4396737	9.01E-06	0.00025963
ENSG000001	67330.732	0.58914178	0.22723292	2.59267794	0.00952319	0.04626718
ENSG000001	129.255105	-0.4717492	0.21149425	-2.2305535	0.02571072	0.09066209
ENSG000001	1893.44283	-0.1528917	0.08096065	-1.8884693	0.05896297	0.1585204
ENSG000001	184.326789	0.91000301	0.21079751	4.31695331	1.58E-05	0.00040404
ENSG000001	231.776096	1.18429298	0.20455999	5.78946532	7.06E-09	6.43E-07
ENSG000001	2489.06975	-0.2006903	0.11410631	-1.758801	0.07861131	0.19172835
ENSG000001	303.029799	0.90524819	0.22103526	4.09549228	4.21E-05	0.0009077
ENSG000001	3992.07251	-0.2198208	0.10021264	-2.1935438	0.02826823	0.0964528
ENSG000001	3814.53374	0.71025895	0.11866698	5.98531259	2.16E-09	2.38E-07
ENSG000001	530.807996	0.4383246	0.1309466	3.34735396	0.00081587	0.00846342
ENSG000001	2950.50474	0.28878822	0.07398569	3.90329857	9.49E-05	0.00173652
ENSG000001	1445.67138	0.15654133	0.07188311	2.17772076	0.02942683	0.09918022
ENSG000001	399.080044	0.28488623	0.11471121	2.48350817	0.01300954	0.05742055
ENSG000001	323.313691	0.36087544	0.17595168	2.05099166	0.04026776	0.12182111
ENSG000001	9315.14854	0.83970554	0.2149948	3.90570166	9.40E-05	0.0017232
ENSG000001	7506.68038	0.25347129	0.12323861	2.0567523	0.03971006	0.12075776
ENSG000001	3808.59264	0.65891545	0.23365276	2.82006279	0.00480143	0.0291373
ENSG000001	301.403219	0.29735658	0.14485613	2.05277182	0.04009471	0.12161173
ENSG000001	809.521794	0.12923069	0.05153058	2.50784458	0.01214701	0.05485389
ENSG000001	261.321489	0.20124736	0.11566691	1.73988698	0.08187886	0.19720443
ENSG000001	17388.0962	0.61747501	0.16419631	3.76059011	0.00016951	0.00268033
ENSG000001	1368.02078	0.12026167	0.04097383	2.93508478	0.00333457	0.02263266
ENSG000001	135.616734	-0.6458851	0.24704912	-2.6143994	0.00893845	0.04434606
ENSG000001	4103.68461	1.62024529	0.27674736	5.85460063	4.78E-09	4.64E-07
ENSG000001	128.960062	1.6683476	0.27786452	6.00417646	1.92E-09	2.19E-07
ENSG000001	1601.53741	-0.3306369	0.07617706	-4.3403734	1.42E-05	0.00037317

ENSG000001	1256.22955	-0.3253523	0.0981402	-3.3151788	0.00091585	0.00914562
ENSG000001	4731.41241	0.75142061	0.14794968	5.07889304	3.80E-07	1.92E-05
ENSG000001	2296.10472	1.43914413	0.275424	5.22519508	1.74E-07	1.01E-05
ENSG000001	3676.30306	0.45871508	0.16075315	2.85353716	0.00432355	0.02698637
ENSG000001	112.263635	1.09980192	0.25808655	4.26136857	2.03E-05	0.0004965
ENSG000001	15155.9908	0.41257573	0.20354674	2.02693359	0.0426692	0.12649809
ENSG000001	2009.81928	0.47023713	0.13781914	3.41198712	0.00064491	0.00714984
ENSG000001	1405.18442	0.28227554	0.07055309	4.00089523	6.31E-05	0.00124513
ENSG000001	3568.70369	1.3946938	0.30139376	4.62748069	3.70E-06	0.00012286
ENSG000001	5017.37051	0.24025039	0.1287763	1.86564136	0.06209156	0.16413272
ENSG000001	117.755991	0.80522189	0.20482789	3.9312122	8.45E-05	0.00157483
ENSG000001	3420.18921	0.37449806	0.12080802	3.09994362	0.00193557	0.01549688
ENSG000001	169.685606	-0.2587275	0.13964479	-1.8527545	0.06391756	0.16712745
ENSG000001	2138.85673	-0.8673594	0.29762136	-2.914305	0.00356481	0.02366488
ENSG000001	421.770095	-0.1603779	0.07966002	-2.0132797	0.04408522	0.12954575
ENSG000001	3094.72327	0.27981856	0.0984253	2.84295349	0.00446976	0.02767773
ENSG000001	38290.3956	0.31774769	0.14381966	2.20934805	0.02715044	0.09398053
ENSG000001	14857.848	0.11600111	0.05713229	2.03039475	0.04231643	0.12579088
ENSG000001	968.639821	-0.1453988	0.07611264	-1.9103104	0.05609327	0.15263978
ENSG000001	3159.00097	-0.2124296	0.04053609	-5.2405043	1.60E-07	9.41E-06
ENSG000001	8051.2466	-0.2372662	0.08805464	-2.6945342	0.00704871	0.03778284
ENSG000001	5121.23243	-0.1073311	0.05371066	-1.9983195	0.04568204	0.13276291
ENSG000001	23772.5922	0.56712209	0.13256399	4.27810062	1.88E-05	0.00046759
ENSG000001	3940.04616	-0.3320179	0.12395645	-2.6785041	0.00739518	0.0390354
ENSG000001	3856.90622	-0.082843	0.03854038	-2.1495114	0.03159389	0.10404599
ENSG000001	3882.3958	0.32956006	0.15949025	2.06633357	0.03879699	0.11884071
ENSG000001	1913.48329	0.21926846	0.12099318	1.81223817	0.0699494	0.17738329
ENSG000001	5179.4611	0.21831249	0.05058492	4.31576218	1.59E-05	0.0004056
ENSG000001	965.011754	-0.2614513	0.12552739	-2.082823	0.03726736	0.11577735
ENSG000001	212.818801	-0.3863237	0.11157834	-3.4623541	0.00053547	0.00620943
ENSG000001	6224.10993	1.68370532	0.2322941	7.24816215	4.22E-13	1.74E-10
ENSG000001	9959.9552	0.45988574	0.13467431	3.41479939	0.00063829	0.0070917
ENSG000001	619.387709	-0.4153387	0.1433617	-2.8971388	0.00376583	0.02458539
ENSG000001	4735.87355	-0.107413	0.06180734	-1.7378685	0.08223398	0.19785629
ENSG000001	902.103848	3.06635814	0.48973774	6.26122489	3.82E-10	5.13E-08
ENSG000001	25.2615674	1.85892597	0.47634126	3.90250886	9.52E-05	0.00174025
ENSG000001	3183.11035	-0.2217427	0.08572943	-2.5865408	0.00969447	0.04682186
ENSG000001	1127.97945	0.40744694	0.09706681	4.19759282	2.70E-05	0.00063829
ENSG000001	1198.80253	0.32594763	0.11046559	2.95067111	0.00317084	0.02183634
ENSG000001	279.192497	0.68181907	0.13917669	4.89894575	9.64E-07	4.05E-05
ENSG000001	86.6359701	0.52388895	0.16837892	3.11136892	0.00186222	0.01511626
ENSG000001	2009.99088	-0.0812727	0.04547113	-1.7873479	0.07388127	0.18424809
ENSG000001	204.779282	-0.1239977	0.06004392	-2.0651172	0.03891192	0.11905928

ENSG000001	57.2480658	1.30249865	0.34561033	3.76869132	0.00016411	0.0026165
ENSG000001	2334.99141	0.80508144	0.16148093	4.98561306	6.18E-07	2.81E-05
ENSG000001	1058.11599	0.16205942	0.06020789	2.69166396	0.00710965	0.03804732
ENSG000001	47.7243985	1.16457154	0.22048669	5.28182233	1.28E-07	7.77E-06
ENSG000001	3154.5673	0.17972543	0.07052407	2.5484268	0.010821	0.05052539
ENSG000001	1407.68007	0.68942331	0.10269033	6.71361447	1.90E-11	3.89E-09
ENSG000001	1853.7512	-0.0905122	0.04876601	-1.8560504	0.06344638	0.16631978
ENSG000001	1057.66578	0.19430966	0.05568449	3.48947534	0.00048397	0.00574215
ENSG000001	1023.65117	0.40444406	0.15846944	2.55218955	0.01070483	0.05016491
ENSG000001	99.3202479	1.27469997	0.31530202	4.04279034	5.28E-05	0.00108124
ENSG000001	1281.51716	0.18440422	0.07983673	2.30976666	0.02090108	0.07907307
ENSG000001	5527.02331	0.24717112	0.09215467	2.68213334	0.00731543	0.03874389
ENSG000001	6799.03444	0.35496956	0.1735256	2.04563225	0.04079257	0.12277454
ENSG000001	160.044859	0.28081172	0.10919452	2.571665	0.01012108	0.04820563
ENSG000001	1913.94634	-0.2825172	0.07973167	-3.5433499	0.00039508	0.00497375
ENSG000001	1663.76985	1.62115192	0.24995946	6.48565935	8.83E-11	1.53E-08
ENSG000001	188.9711	-0.6060443	0.15347978	-3.9486914	7.86E-05	0.00148956
ENSG000001	2005.67101	-0.2957016	0.07543067	-3.920177	8.85E-05	0.00163571
ENSG000001	308.417792	-0.4276578	0.14622285	-2.9246988	0.0034479	0.02315385
ENSG000001	4109.94686	0.386362	0.18441039	2.09512056	0.03616028	0.11358622
ENSG000001	51.5718437	1.20148568	0.2437611	4.92894759	8.27E-07	3.56E-05
ENSG000001	27231.1183	0.36017396	0.10476956	3.43777284	0.00058652	0.00661883
ENSG000001	6089.91361	-0.2232128	0.10586149	-2.1085361	0.03498464	0.11130058
ENSG000001	1061.1136	0.33812323	0.12487991	2.70758714	0.00677743	0.03670062
ENSG000001	4182.4262	0.17725141	0.10091072	1.75651719	0.07900014	0.19227836
ENSG000001	115.621399	0.2480976	0.09679443	2.56313911	0.01037305	0.04904331
ENSG000001	891.869391	0.26400978	0.10797332	2.44513901	0.01447963	0.06146421
ENSG000001	443.634121	0.15456056	0.07669082	2.01537242	0.04386564	0.12904185
ENSG000001	1389.27075	-0.221237	0.06407343	-3.4528661	0.00055466	0.00635114
ENSG000001	1029.83077	-0.2095202	0.10824032	-1.9356943	0.05290515	0.14708135
ENSG000001	2155.68013	0.14489965	0.07789777	1.86012574	0.06286774	0.16525205
ENSG000001	183.040205	0.29216981	0.13655783	2.13953166	0.03239263	0.10583687
ENSG000001	131.058352	1.03947196	0.19759357	5.26065688	1.44E-07	8.56E-06
ENSG000001	903.446484	0.83329259	0.2363128	3.52622701	0.00042153	0.00522004
ENSG000001	113.161477	0.27123304	0.130637	2.0762345	0.03787227	0.11705779
ENSG000001	1141.27248	0.1604651	0.06415822	2.50108411	0.01238138	0.05554513
ENSG000001	4658.03619	0.24651512	0.07062991	3.49023671	0.00048259	0.00572996
ENSG000001	6612.78319	0.42956098	0.1952846	2.19966642	0.02783057	0.09560819
ENSG000001	3635.49317	0.63080258	0.11177573	5.64346659	1.67E-08	1.33E-06
ENSG000001	3425.92074	0.18515709	0.07593172	2.43846836	0.01474965	0.06228432
ENSG000001	1343.83249	0.1559882	0.07703706	2.02484609	0.04288317	0.12696919
ENSG000001	3819.93762	0.23389856	0.08412405	2.78040074	0.00542919	0.03158846
ENSG000001	1313.88317	0.31781896	0.08389745	3.78818367	0.00015175	0.00248829

ENSG000001	7815.67498	0.20286717	0.0714017	2.8412091	0.00449428	0.02777714
ENSG000001	6464.6371	0.14741598	0.07485503	1.96935285	0.04891259	0.13928789
ENSG000001	370.028477	0.25114782	0.07769757	3.23237661	0.00122765	0.01128352
ENSG000001	3400.96038	0.14027945	0.06126674	2.2896508	0.02204157	0.08193124
ENSG000001	529.559957	0.35654867	0.11492526	3.10243942	0.00191933	0.01540442
ENSG000001	198.040476	-0.3322455	0.13502867	-2.460555	0.01387223	0.05984293
ENSG000001	6339.50024	-0.0784769	0.04499401	-1.7441625	0.08113076	0.19589179
ENSG000001	1809.21885	0.58751021	0.15011366	3.91376913	9.09E-05	0.00167409
ENSG000001	593.782576	-0.273185	0.1088068	-2.5107344	0.01204803	0.0545324
ENSG000001	1323.53342	0.37984875	0.14246776	2.66620838	0.00767121	0.03993171
ENSG000001	320.020419	-0.3091157	0.17552747	-1.7610675	0.07822698	0.19094999
ENSG000001	218.351465	0.3752108	0.11587021	3.23819906	0.00120287	0.01109305
ENSG000001	11079.3119	0.60916524	0.18728952	3.25253246	0.00114382	0.01067868
ENSG000001	4762.52622	0.34473855	0.09736002	3.54086367	0.00039882	0.00499576
ENSG000001	5419.34961	0.7022936	0.1937875	3.62403976	0.00029004	0.00388858
ENSG000001	2570.58442	0.24991355	0.06708265	3.72545721	0.00019496	0.00297107
ENSG000001	605.381346	0.19598647	0.09289603	2.10974002	0.03488076	0.1111858
ENSG000001	4534.24113	0.22615914	0.10140329	2.23029387	0.02572794	0.0907033
ENSG000001	2171.2684	0.10520333	0.05165343	2.03671528	0.04167858	0.124563
ENSG000001	15252.9925	2.46396773	0.35158012	7.00826813	2.41E-12	7.06E-10
ENSG000001	1169.86913	0.18335919	0.10183441	1.80056221	0.07177191	0.18080258
ENSG000001	7200.67239	-0.1418561	0.0599068	-2.3679465	0.01788712	0.07087848
ENSG000001	243.990684	1.75811871	0.30554196	5.754099	8.71E-09	7.72E-07
ENSG000001	5634.07903	-0.5434846	0.20038789	-2.7121627	0.00668458	0.03632981
ENSG000001	5133.02903	0.60228629	0.15289917	3.9391077	8.18E-05	0.00153788
ENSG000001	2209.62779	1.0555363	0.2636886	4.00296527	6.26E-05	0.00123726
ENSG000001	19.1061316	0.92907161	0.34185644	2.71772447	0.00657326	0.03592722
ENSG000001	6288.38639	0.19600794	0.10776416	1.81886015	0.06893277	0.1759755
ENSG000001	4863.5762	0.27809924	0.12381757	2.24604022	0.02470143	0.08831247
ENSG000001	275.735443	-0.2984696	0.10723574	-2.7833037	0.00538084	0.03143766
ENSG000001	9891.92543	0.19436649	0.09866858	1.9698925	0.04885069	0.13917359
ENSG000001	1712.00874	0.10700437	0.04320056	2.47692055	0.01325214	0.05810588
ENSG000001	9217.68776	-0.2853062	0.13535528	-2.1078317	0.03504555	0.1114295
ENSG000001	164.47879	-0.7169784	0.18997188	-3.7741293	0.00016057	0.00259136
ENSG000001	42.6858274	1.16078693	0.32401707	3.58248696	0.00034034	0.00440431
ENSG000001	9817.87911	-0.3321719	0.12426003	-2.6732001	0.00751314	0.03936345
ENSG000001	309.689551	-0.2990855	0.12278975	-2.4357526	0.01486085	0.06256837
ENSG000001	429.747849	-0.2773231	0.12499695	-2.2186394	0.02651127	0.09246201
ENSG000001	777.12784	0.22631913	0.07658708	2.95505623	0.00312612	0.02162827
ENSG000001	1542.63262	-0.1630373	0.06757085	-2.4128342	0.01582902	0.06555908
ENSG000001	1397.10962	-0.1184575	0.06350623	-1.8652897	0.06214081	0.16418351
ENSG000001	6259.77353	0.14341203	0.08241003	1.74022547	0.08181943	0.1971192
ENSG000001	122.264063	0.38090475	0.18550806	2.05330561	0.04004295	0.12149967

ENSG000001	958.080232	0.34680714	0.12601334	2.75214613	0.00592061	0.03338041
ENSG000001	305.226186	0.17652149	0.09000747	1.96118722	0.04985719	0.14128563
ENSG000001	67081.2534	-0.2542611	0.08495369	-2.9929383	0.00276306	0.0198015
ENSG000001	948.321928	0.26959745	0.13450412	2.00438063	0.0450293	0.13147177
ENSG000001	1127.65827	-0.2116532	0.09373076	-2.2580976	0.02393958	0.08665281
ENSG000001	8102.06671	0.26892998	0.12555324	2.14195974	0.03219672	0.10539622
ENSG000001	604.368688	0.23697797	0.08472205	2.79712282	0.00515599	0.03063146
ENSG000001	3303.68388	0.09060049	0.0466633	1.94157912	0.05218807	0.14575504
ENSG000001	38055.9825	0.38659846	0.12423485	3.11183578	0.00185928	0.01509985
ENSG000001	850.704118	0.28261302	0.09625312	2.93614392	0.0033232	0.02258498
ENSG000001	123.848943	0.32166555	0.14951911	2.15133401	0.03144985	0.10367574
ENSG000001	540.878475	-0.4941433	0.14740195	-3.3523528	0.00080128	0.00836083
ENSG000001	2175.38139	-0.2108093	0.11575914	-1.8211026	0.06859126	0.17540407
ENSG000001	140.152037	-0.223063	0.09010778	-2.4755131	0.01330449	0.05827325
ENSG000001	256.675556	0.48552206	0.16575818	2.92909866	0.00339946	0.02295759
ENSG000001	3973.67257	0.22899522	0.11940781	1.91775738	0.05514178	0.15119729
ENSG000001	328.463946	0.27828732	0.08551708	3.2541723	0.00113723	0.01062519
ENSG000001	498.336606	0.4702771	0.13127603	3.5823531	0.00034051	0.00440431
ENSG000001	923.690527	0.17955238	0.10197056	1.76082571	0.07826791	0.19100445
ENSG000001	7065.54599	0.39207481	0.08091347	4.84560628	1.26E-06	5.10E-05
ENSG000001	76.1186999	1.41231802	0.62976172	2.24262285	0.02492115	0.08879444
ENSG000001	5438.02756	0.26120165	0.09287434	2.81241985	0.00491703	0.02953278
ENSG000001	1320.22518	0.34574027	0.11579334	2.98583885	0.00282802	0.02013503
ENSG000001	7499.78413	0.54868322	0.120917	4.53768463	5.69E-06	0.00017763
ENSG000001	7772.76338	1.11614285	0.20078596	5.5588689	2.72E-08	2.01E-06
ENSG000001	313.954223	0.39680448	0.11860782	3.34551692	0.00082129	0.00849329
ENSG000001	1128.46733	0.12807799	0.04455993	2.87428613	0.00404942	0.02583592
ENSG000001	17078.7421	0.83841774	0.1899669	4.41349374	1.02E-05	0.00028608
ENSG000001	462.387647	0.24786998	0.09609814	2.57934204	0.00989887	0.04745959
ENSG000001	551.182049	-0.3841125	0.15755312	-2.4379875	0.01476929	0.06235118
ENSG000001	1124.52583	0.13797614	0.04393743	3.14028722	0.00168782	0.01410647
ENSG000001	933.665304	-0.1614768	0.06687474	-2.4146154	0.01575183	0.06532189
ENSG000001	71.8607305	0.84690212	0.23254369	3.64190536	0.00027063	0.00370855
ENSG000001	2308.19042	0.43744786	0.11472192	3.81311505	0.00013723	0.00229602
ENSG000001	146.745182	0.17225464	0.09217335	1.86881166	0.06164902	0.16327879
ENSG000001	19.2101604	0.67854907	0.28519815	2.37921972	0.01734933	0.06963941
ENSG000001	676.866768	-0.2046727	0.09714453	-2.1068884	0.03512725	0.1116027
ENSG000001	1104.68951	0.27541971	0.15108338	1.82296492	0.0683087	0.17484512
ENSG000001	3152.43812	-0.1673529	0.08696253	-1.9244248	0.05430135	0.14959687
ENSG000001	454.749374	-0.2382749	0.10977045	-2.1706654	0.02995647	0.10036703
ENSG000001	6956.56943	0.48226846	0.11688799	4.12590249	3.69E-05	0.00082271
ENSG000001	3064.61813	0.19885895	0.10064781	1.97579011	0.04817855	0.13780151
ENSG000001	556.784516	0.43963571	0.13074433	3.36256041	0.00077223	0.00815646

ENSG000001	1086.02976	0.13811726	0.07267661	1.90043606	0.05737592	0.15537037
ENSG000001	35249.686	-0.2181098	0.07187308	-3.0346525	0.00240813	0.01794823
ENSG000001	91.3303917	-0.339961	0.11461205	-2.9661888	0.00301515	0.02105599
ENSG000001	1143.23522	0.17017023	0.06178705	2.75414061	0.00588465	0.03323823
ENSG000001	870.755627	-0.2140771	0.08493299	-2.5205411	0.01171746	0.05341427
ENSG000001	359.326673	-0.3845393	0.10975504	-3.5036138	0.00045899	0.00550957
ENSG000001	48.1430019	1.23984449	0.25814616	4.80287785	1.56E-06	5.96E-05
ENSG000001	1935.81095	-0.1700784	0.08837929	-1.9244145	0.05430265	0.14959687
ENSG000001	662.440506	0.18839237	0.10486338	1.7965506	0.07240699	0.18178801
ENSG000001	78.1064936	1.84691678	0.32245652	5.7276459	1.02E-08	8.70E-07
ENSG000001	1325.31285	0.10878534	0.06217384	1.74969628	0.08017074	0.19423161
ENSG000001	1210.21647	0.14116394	0.07548049	1.87020434	0.06145545	0.162924
ENSG000001	1092.96256	0.18114608	0.07011319	2.5836233	0.00977685	0.04709612
ENSG000001	574.296131	0.86187987	0.28100267	3.06715906	0.00216104	0.01662813
ENSG000001	6267.90137	0.6416506	0.19418406	3.30434232	0.000952	0.00939787
ENSG000001	275.069483	0.37255406	0.10122561	3.68043284	0.00023284	0.00333728
ENSG000001	67.811173	1.0237382	0.22405826	4.56907153	4.90E-06	0.0001572
ENSG000001	10504.8934	0.49462006	0.27359393	1.80786196	0.07062799	0.17868957
ENSG000001	4769.90383	0.20887053	0.10006696	2.08730757	0.03686034	0.11486108
ENSG000001	4848.57328	0.21620918	0.1033949	2.09110115	0.036519	0.11412274
ENSG000001	78.1188332	1.56224678	0.35800527	4.36375353	1.28E-05	0.00034423
ENSG000001	117.318132	0.29415945	0.11370708	2.58699318	0.00968175	0.04677421
ENSG000001	3371.57337	0.36148912	0.09378616	3.8543972	0.00011602	0.00202235
ENSG000001	302.703225	0.55740887	0.2283914	2.440586	0.01466345	0.06209624
ENSG000001	4609.9992	0.23306421	0.087029	2.6780064	0.00740618	0.0390354
ENSG000001	3559.47347	0.1124555	0.06202023	1.81320691	0.06979991	0.17729017
ENSG000001	1784.2731	0.42089354	0.11122577	3.78413696	0.00015424	0.00251232
ENSG000001	56.5918591	0.68466409	0.16381093	4.17959948	2.92E-05	0.0006836
ENSG000001	11370.4454	-0.0896855	0.05122569	-1.7507911	0.0799819	0.19403213
ENSG000001	688.518225	-0.1402426	0.07923684	-1.769917	0.07674097	0.18854085
ENSG000001	2345.64722	0.30062486	0.08252391	3.64288214	0.0002696	0.0037024
ENSG000001	738.695056	0.12941239	0.06135582	2.10921134	0.03492634	0.11120141
ENSG000001	355.943915	0.47177729	0.14742674	3.20007944	0.0013739	0.01218377
ENSG000001	6084.64752	0.13694249	0.0696735	1.96548892	0.04935768	0.14025348
ENSG000001	149.130552	-0.2530467	0.1330006	-1.9025982	0.057093	0.15481295
ENSG000001	2868.23219	-0.2286693	0.09214438	-2.4816411	0.01307789	0.05762757
ENSG000001	389.39883	0.31937333	0.12736595	2.50752516	0.01215799	0.05487327
ENSG000001	22207.248	0.77030547	0.16395829	4.69817938	2.62E-06	9.06E-05
ENSG000001	119.426448	-0.3915488	0.20494364	-1.9105192	0.0560664	0.15263502
ENSG000001	5333.50498	0.2643486	0.13255137	1.99431064	0.04611812	0.13369971
ENSG000001	825.017285	0.65973595	0.16416352	4.0187732	5.85E-05	0.00117556
ENSG000001	2135.68685	1.00444101	0.22018324	4.56184126	5.07E-06	0.00016207
ENSG000001	218.468222	0.55697165	0.20870885	2.66865374	0.00761559	0.03973047

ENSG000001	181.174689	0.62265019	0.29506148	2.11023882	0.03483779	0.11111364
ENSG000001	74.7706097	0.53731087	0.18201679	2.95198513	0.00315738	0.02177444
ENSG000001	10921.7357	0.54202401	0.14640137	3.70231523	0.00021364	0.00315592
ENSG000001	840.019265	0.12315752	0.06770437	1.81904819	0.06890408	0.1759755
ENSG000001	3875.38003	-0.0821218	0.04058035	-2.0236829	0.04300279	0.12725443
ENSG000001	109.319927	0.28178072	0.1358156	2.07473007	0.03801156	0.1172485
ENSG000001	366.319539	0.78663802	0.28063684	2.80304613	0.00506224	0.03024694
ENSG000001	3919.07053	1.04031333	0.15880954	6.55069779	5.73E-11	1.07E-08
ENSG000001	1275.30489	0.22372451	0.07241216	3.08959861	0.00200427	0.01580762
ENSG000001	453.284768	0.16532685	0.06932009	2.38497745	0.01708017	0.06896969
ENSG000001	1356.31383	-0.2648681	0.09832521	-2.6937963	0.00706433	0.0378542
ENSG000001	1532.69473	-0.1787956	0.09983258	-1.7909542	0.07330064	0.18324602
ENSG000001	1131.01331	0.27487711	0.10042211	2.73721714	0.00619614	0.03446339
ENSG000001	66.6395869	-0.6076735	0.33921346	-1.7914193	0.07322604	0.18310744
ENSG000001	490.57974	1.92226426	0.26496429	7.25480511	4.02E-13	1.74E-10
ENSG000001	93.0294268	0.32309545	0.15938343	2.02715835	0.04264622	0.12649604
ENSG000001	5657.26949	0.49616396	0.18825032	2.63566066	0.00839737	0.04257626
ENSG000001	66.392463	-0.8981114	0.31162344	-2.8820408	0.00395109	0.02538635
ENSG000001	2329.0939	0.56368455	0.1556646	3.62114792	0.0002933	0.00392589
ENSG000001	12993.7549	1.36407658	0.21231328	6.42482929	1.32E-10	2.16E-08
ENSG000001	67.2346917	1.0038876	0.24705984	4.06333787	4.84E-05	0.0010079
ENSG000001	589.858819	-0.2432447	0.09712779	-2.5043782	0.01226668	0.05519671
ENSG000001	109.071157	0.65958356	0.28448418	2.31852452	0.02042083	0.07776135
ENSG000001	982.887935	0.24999371	0.07242919	3.45156005	0.00055736	0.0063729
ENSG000001	895.192858	0.14079571	0.05704677	2.46807498	0.01358419	0.05901958
ENSG000001	2039.40084	0.443735	0.1622916	2.73418332	0.00625352	0.03471191
ENSG000001	584.801574	0.44583852	0.10714734	4.16098528	3.17E-05	0.00072975
ENSG000001	690.952779	0.28930849	0.08028585	3.60348024	0.00031398	0.00412533
ENSG000001	15363.8733	-0.1532589	0.0582864	-2.6294115	0.00855328	0.04315014
ENSG000001	6128.5689	0.39996291	0.15261543	2.62072389	0.00877433	0.04374359
ENSG000001	309.524275	0.36508132	0.10763052	3.39198688	0.00069388	0.00754477
ENSG000001	209.500513	0.26920304	0.07273256	3.70127273	0.00021452	0.00316606
ENSG000001	3104.56854	-0.1156681	0.04443969	-2.6028105	0.0092463	0.0454744
ENSG000001	1155.8572	0.96894287	0.13779496	7.03177283	2.04E-12	6.34E-10
ENSG000001	5411.72709	0.84421465	0.26997151	3.12705082	0.00176569	0.01452689
ENSG000001	1698.80282	-0.1591807	0.0643112	-2.4751626	0.01331755	0.05830919
ENSG000001	43359.996	0.94330975	0.29204879	3.22997316	0.00123802	0.01133368
ENSG000001	932.999161	-0.2504469	0.10645742	-2.3525542	0.01864497	0.07291237
ENSG000001	231.700541	0.46287642	0.1882074	2.45939542	0.01391712	0.05989025
ENSG000001	217.743649	0.20030524	0.10291637	1.9462913	0.05161976	0.14453769
ENSG000001	461.612429	-0.2915769	0.13694866	-2.1290967	0.03324626	0.1076924
ENSG000001	315.178531	0.21714648	0.11668824	1.86091138	0.06275669	0.16507091
ENSG000001	450.44183	-0.584632	0.22487172	-2.5998469	0.00932654	0.04562458

ENSG000001	1586.4444	-0.2196351	0.09911674	-2.215923	0.02669678	0.09293349
ENSG000001	5120.41032	0.32317138	0.10592252	3.05101654	0.00228068	0.01732915
ENSG000001	373.704138	0.15423033	0.08279596	1.86277602	0.06249378	0.1645857
ENSG000001	613.884799	0.0945948	0.04548641	2.07962759	0.0375597	0.11631095
ENSG000001	5482.01512	0.78849048	0.18060336	4.36586823	1.27E-05	0.00034204
ENSG000001	226.432385	0.33932954	0.09308773	3.64526591	0.00026712	0.00367749
ENSG000001	3123.22481	0.30017733	0.08966574	3.34773711	0.00081474	0.00846342
ENSG000001	10938.1323	0.34601407	0.15677708	2.20704495	0.02731092	0.09430372
ENSG000001	657.944369	0.38344315	0.1746111	2.19598378	0.0280931	0.09606729
ENSG000001	905.198094	-0.2548268	0.09613075	-2.6508349	0.00802931	0.04118271
ENSG000001	7391.83942	-0.1796093	0.08476298	-2.1189595	0.03409389	0.1094097
ENSG000001	2635.03535	-0.1834166	0.06725898	-2.7270206	0.00639091	0.03521226
ENSG000001	5255.66032	-0.2918612	0.16381684	-1.7816312	0.07480939	0.18565343
ENSG000001	4791.80161	-0.1358063	0.05808842	-2.337924	0.01939119	0.07488398
ENSG000001	4899.84077	-0.2968579	0.11875259	-2.4998014	0.01242629	0.05565527
ENSG000001	2393.8138	0.23981184	0.08235065	2.91208186	0.00359029	0.02378582
ENSG000001	733.640388	-0.2408401	0.1079674	-2.2306739	0.02570274	0.09066209
ENSG000001	13788.9288	0.24286005	0.08677416	2.7987602	0.00512992	0.03048761
ENSG000001	271.833028	0.35202493	0.09303344	3.78385358	0.00015442	0.00251232
ENSG000001	1037.93479	0.19312564	0.07444895	2.59406803	0.00948477	0.04620715
ENSG000001	833.476676	0.41640197	0.14452559	2.8811644	0.00396209	0.02541906
ENSG000001	836.323384	-0.1385636	0.07184554	-1.9286312	0.05377666	0.14854716
ENSG000001	3408.11868	0.15062404	0.07896116	1.90757122	0.05644666	0.15336691
ENSG000001	1892.52804	-0.3178713	0.17385389	-1.8283817	0.06749229	0.1735138
ENSG000001	3829.88647	0.35053717	0.15075503	2.32521046	0.02006071	0.07678232
ENSG000001	389.249322	-0.4126316	0.12110332	-3.4072696	0.00065616	0.00724031
ENSG000001	1176.49293	0.14263098	0.05799504	2.45936508	0.0139183	0.05989025
ENSG000001	623.411459	1.50224984	0.26341357	5.70300845	1.18E-08	9.85E-07
ENSG000001	1816.77499	0.25997982	0.07395687	3.51528981	0.00043927	0.00533481
ENSG000001	169.375088	-0.2298968	0.10169801	-2.2605832	0.02378508	0.08626496
ENSG000001	1078.51457	0.13872458	0.07369296	1.88246733	0.05977259	0.15992504
ENSG000001	1162.17215	-0.2272953	0.08657021	-2.6255599	0.00865066	0.04337763
ENSG000001	514.958127	0.14927374	0.06638108	2.24873933	0.02452909	0.08795834
ENSG000001	181.406147	0.34560191	0.1328187	2.60205753	0.00926663	0.04552238
ENSG000001	2359.71849	0.27574847	0.13159751	2.09539274	0.03613609	0.11358565
ENSG000001	3154.94582	0.45591913	0.09073278	5.02485564	5.04E-07	2.41E-05
ENSG000001	5323.90021	0.75604731	0.28337291	2.66802961	0.00762975	0.03979169
ENSG000001	104.093464	0.26142883	0.12186035	2.14531487	0.03192768	0.10474553
ENSG000001	4848.27199	0.69401939	0.1335124	5.19816446	2.01E-07	1.11E-05
ENSG000001	6723.17167	-0.2272181	0.10775809	-2.1085946	0.03497959	0.11130058
ENSG000001	3417.65442	1.00286065	0.1554042	6.45324032	1.09E-10	1.85E-08
ENSG000001	45648.2257	1.47594999	0.18575732	7.94558188	1.93E-15	2.88E-12
ENSG000001	1962.77159	-0.171379	0.07197803	-2.3809909	0.01726614	0.0694585

ENSG000001	7322.119	0.19630667	0.06657599	2.94861065	0.00319206	0.02195477
ENSG000001	5665.10527	-0.1927107	0.07179275	-2.6842648	0.00726895	0.03856906
ENSG000001	1307.62773	0.63412368	0.15701967	4.03849829	5.38E-05	0.00109574
ENSG000001	5209.32437	0.51395696	0.16472469	3.12009669	0.00180792	0.01478456
ENSG000001	2663.62448	-0.1613412	0.08337495	-1.9351282	0.05297457	0.14717566
ENSG000001	861.253178	0.19265486	0.09848431	1.95619842	0.05044178	0.14252868
ENSG000001	210.695195	-0.8616911	0.24880835	-3.4632724	0.00053365	0.00619266
ENSG000001	6447.97086	1.63363054	0.43486673	3.75662341	0.00017222	0.00270969
ENSG000001	3592.62685	0.53587281	0.18309048	2.92681972	0.00342447	0.02305013
ENSG000001	59139.2067	0.27413334	0.1341901	2.04287303	0.04106501	0.12327773
ENSG000001	1602.56844	0.23271234	0.13145119	1.77033263	0.07667175	0.18851853
ENSG000001	3213.16648	0.36567214	0.1633986	2.23791479	0.02522661	0.08949389
ENSG000001	9861.14616	2.13816396	0.27314361	7.82798455	4.96E-15	5.81E-12
ENSG000001	651.765811	-0.2059632	0.05965617	-3.4525048	0.00055541	0.00635521
ENSG000001	2394.40087	0.24760499	0.09424271	2.62731197	0.00860624	0.04331384
ENSG000001	480.689681	0.28397206	0.12317555	2.30542553	0.02114275	0.07971435
ENSG000001	1056.55469	0.10005118	0.05531954	1.80860476	0.07051243	0.17842473
ENSG000001	1145.21303	0.25030122	0.12492128	2.00367154	0.04510526	0.1315998
ENSG000001	463.702542	0.17177769	0.09246705	1.85771781	0.0632091	0.16588342
ENSG000001	1584.54643	0.35459311	0.09174475	3.86499625	0.00011109	0.00195656
ENSG000001	977.137251	0.31976157	0.11575838	2.76231902	0.00573924	0.03267579
ENSG000001	3969.75698	0.24045538	0.07825646	3.07265842	0.00212161	0.01646383
ENSG000001	2810.16383	0.41019884	0.13266823	3.0919145	0.0019887	0.01573781
ENSG000001	1622.78004	-0.540385	0.22854768	-2.3644302	0.01805783	0.07139961
ENSG000001	355.410053	0.27386233	0.12830784	2.13441619	0.03280873	0.10668556
ENSG000001	36119.9804	0.52297205	0.23293164	2.24517392	0.02475697	0.08836309
ENSG000001	4014.32705	0.28216561	0.14587632	1.93427978	0.05307874	0.1472586
ENSG000001	2605.23108	0.33013994	0.13252712	2.49111236	0.01273438	0.05664832
ENSG000001	302.955226	-0.1549874	0.08245491	-1.879663	0.06015402	0.16066876
ENSG000001	5415.84916	-0.3424996	0.1306783	-2.6209371	0.00876884	0.04372954
ENSG000001	4971.38617	0.37059432	0.15194143	2.43906042	0.01472551	0.06224204
ENSG000001	67.8397555	-0.3289753	0.17931422	-1.8346303	0.06656048	0.17191784
ENSG000001	1543.84786	0.16316999	0.07864927	2.07465352	0.03801866	0.1172485
ENSG000001	4649.57942	-0.1816962	0.08056679	-2.2552249	0.02411922	0.08709156
ENSG000001	1280.1666	0.32873266	0.09931315	3.31006181	0.00093275	0.00927221
ENSG000001	11254.0448	0.85948058	0.20629021	4.16636635	3.09E-05	0.00071677
ENSG000001	90.6392935	0.73900001	0.32762461	2.25563035	0.02409379	0.08701892
ENSG000001	9396.56443	-0.2892414	0.1041067	-2.7783165	0.00546414	0.03169276
ENSG000001	2840.3066	0.51500561	0.27203013	1.89319327	0.05833217	0.15734045
ENSG000001	949.372566	-0.3420974	0.14773965	-2.3155424	0.02058327	0.07823455
ENSG000001	547.185328	1.00020054	0.26290078	3.80447917	0.0001421	0.00235598
ENSG000001	5490.94069	2.20795744	0.36357267	6.07294662	1.26E-09	1.54E-07
ENSG000001	57.7964901	1.91736108	0.38612475	4.96565185	6.85E-07	3.06E-05

ENSG000001	2913.3058	0.2528924	0.07162301	3.5308819	0.00041418	0.0051527
ENSG000001	12726.9834	2.36604725	0.31635605	7.47906423	7.49E-14	4.55E-11
ENSG000001	332.458089	-0.2481042	0.09034896	-2.7460656	0.00603147	0.03379973
ENSG000001	70.0796009	0.53058189	0.2001045	2.65152404	0.00801294	0.04112306
ENSG000001	430.691693	-0.3434062	0.1374056	-2.4992154	0.01244686	0.05567861
ENSG000001	121.619692	0.61034714	0.19293912	3.16341825	0.00155928	0.01338614
ENSG000001	39.5000259	2.2965256	0.51174288	4.48765523	7.20E-06	0.00021705
ENSG000001	7806.40352	0.39413026	0.1908111	2.06555202	0.0388708	0.11900009
ENSG000001	939.284746	0.14754666	0.08504208	1.73498412	0.08274362	0.19873255
ENSG000001	189.919818	1.23215363	0.23191579	5.31293547	1.08E-07	6.78E-06
ENSG000001	2892.60491	-0.1581822	0.04950066	-3.195558	0.00139561	0.01232962
ENSG000001	858.998014	0.52039715	0.15298365	3.40165197	0.0006698	0.0073512
ENSG000001	5877.71191	0.16699718	0.08296431	2.01287978	0.04412728	0.1295673
ENSG000001	2160.27759	0.33045584	0.10523917	3.14004604	0.00168921	0.01411005
ENSG000001	30.6616083	0.8217278	0.41184276	1.99524646	0.04601601	0.13351658
ENSG000001	324.828579	0.30937355	0.13621517	2.27121219	0.02313414	0.08448339
ENSG000001	676.766201	-0.1014901	0.05384538	-1.8848438	0.05945093	0.15954451
ENSG000001	802.281949	-0.2918774	0.07098217	-4.1119816	3.92E-05	0.00085534
ENSG000001	1854.15542	-0.1840274	0.09075518	-2.027734	0.0425874	0.12641305
ENSG000001	502.149382	-0.5210918	0.25167131	-2.0705253	0.03840318	0.11800916
ENSG000001	34.1916457	1.06018396	0.37497635	2.82733552	0.00469371	0.02867889
ENSG000001	155.504775	0.67512425	0.30148316	2.23934314	0.0251336	0.08929329
ENSG000001	194.216701	1.26090171	0.31211157	4.03990691	5.35E-05	0.00109053
ENSG000001	22280.0007	0.31085822	0.08861372	3.50801457	0.00045146	0.00544297
ENSG000001	108116.259	0.6289257	0.29155151	2.15716837	0.03099255	0.10271192
ENSG000001	2371.68937	-0.1379417	0.06549608	-2.1061064	0.03519511	0.11170127
ENSG000001	359.768752	-0.1708706	0.06731664	-2.5383111	0.01113889	0.05155078
ENSG000001	9531.78696	0.16884364	0.08974339	1.88140469	0.05991689	0.16021808
ENSG000001	6281.60308	-0.193288	0.06628337	-2.9160854	0.00354454	0.02355887
ENSG000001	1330.82329	0.95429467	0.4198222	2.27309241	0.02302061	0.08414377
ENSG000001	4420.19899	-0.0968343	0.04305804	-2.2489243	0.02451731	0.08794802
ENSG000001	5386.33179	-0.1388798	0.07340753	-1.8919019	0.05850405	0.15767437
ENSG000001	1292.20711	0.15166032	0.0700209	2.1659293	0.03031659	0.10122198
ENSG000001	216.278879	0.47709913	0.15780138	3.02341541	0.00249939	0.01838604
ENSG000001	10380.0947	0.77187475	0.3956218	1.95104198	0.05105205	0.14358499
ENSG000001	3552.86759	-0.2924427	0.1115105	-2.6225576	0.00872725	0.04358841
ENSG000001	5830.13571	-0.1939462	0.08151111	-2.3793835	0.01734162	0.06962551
ENSG000001	21279.3613	0.40203306	0.20694917	1.94266578	0.05205655	0.14548684
ENSG000001	432.393543	0.34859203	0.12386316	2.81433182	0.00488787	0.02943912
ENSG000001	11491.7546	0.26894867	0.11384926	2.36232257	0.01816083	0.07165139
ENSG000001	86.9069298	-0.4233302	0.21591806	-1.9606058	0.04992503	0.14140969
ENSG000001	1412.80443	-0.0824847	0.04457444	-1.8504933	0.06424249	0.16778976
ENSG000001	205.487592	-0.2421158	0.11870158	-2.0397018	0.04138003	0.12395112

ENSG000001	4277.26352	0.11466923	0.06433963	1.78224881	0.07470867	0.18546525
ENSG000001	406.742897	0.20502662	0.11238254	1.82436354	0.06809713	0.17453801
ENSG000001	368.005866	1.31577976	0.2757527	4.77159332	1.83E-06	6.78E-05
ENSG000001	632.824607	0.25847054	0.08421331	3.06923619	0.00214607	0.01655322
ENSG000001	719.039559	0.356652	0.09012428	3.95733547	7.58E-05	0.00145179
ENSG000001	1595.47102	-0.243152	0.09882502	-2.4604299	0.01387707	0.05984806
ENSG000001	4023.98443	0.22916725	0.07955727	2.88053174	0.00397005	0.02543842
ENSG000001	55.0584644	-1.1701618	0.39261981	-2.9803941	0.00287878	0.02041666
ENSG000001	1087.55954	0.17100155	0.06788383	2.51903208	0.01176779	0.05356927
ENSG000001	244.040987	-0.4199813	0.2156631	-1.9473954	0.05148735	0.14428953
ENSG000001	6226.0467	0.24792133	0.10046225	2.46780593	0.0135944	0.05904831
ENSG000001	256.650585	0.32845605	0.12899903	2.54618999	0.01089059	0.05070216
ENSG000001	2763.6175	0.28430224	0.11333559	2.5084992	0.01212452	0.05481274
ENSG000001	3884.78684	0.45519615	0.15610861	2.91589383	0.00354671	0.02356379
ENSG000001	288.348659	-0.2159391	0.09908524	-2.1793271	0.02930738	0.09896794
ENSG000001	744.574222	0.21231733	0.07331525	2.89595051	0.00378012	0.02465498
ENSG000001	13817.1022	0.18566598	0.09201768	2.01772077	0.04362035	0.12857143
ENSG000001	4940.32702	0.11438842	0.06599703	1.73323592	0.08305375	0.19927309
ENSG000001	4103.82507	0.1939368	0.06724805	2.88390207	0.00392781	0.02528633
ENSG000001	38186.2149	3.34307754	0.52884181	6.32150757	2.59E-10	3.76E-08
ENSG000001	10666.1486	2.68454895	0.38289923	7.01111093	2.36E-12	7.05E-10
ENSG000001	3434.8323	0.26788423	0.087353	3.06668602	0.00216446	0.01664665
ENSG000001	441.856148	-0.2613691	0.14160305	-1.8457875	0.06492306	0.16913624
ENSG000001	858.354785	0.17476013	0.07002341	2.49573863	0.01256952	0.05606703
ENSG000001	3967.40776	0.18358105	0.072138	2.54485898	0.01093219	0.05083668
ENSG000001	3054.4276	-0.0897894	0.04231119	-2.1221187	0.03382777	0.10898148
ENSG000001	578.220635	1.87680053	0.37451817	5.01124021	5.41E-07	2.53E-05
ENSG000001	632.182805	0.60929099	0.1140265	5.34341569	9.12E-08	5.87E-06
ENSG000001	530.631688	-0.6759612	0.23246627	-2.9077819	0.00364002	0.02404604
ENSG000001	209.109381	0.18181438	0.10324342	1.76102634	0.07823395	0.19094999
ENSG000001	230.606461	1.26142491	0.2397448	5.26153197	1.43E-07	8.55E-06
ENSG000001	366.036002	0.63644853	0.15882475	4.00723785	6.14E-05	0.00122247
ENSG000001	463.372621	0.22419276	0.07944466	2.8219991	0.00477253	0.02903718
ENSG000001	4299.93516	0.17701193	0.0759408	2.33092008	0.01975757	0.07601242
ENSG000001	427.519635	0.70508398	0.18505563	3.81011895	0.0001389	0.00231458
ENSG000001	5675.20805	-0.1050497	0.05222264	-2.0115746	0.0442648	0.12986401
ENSG000001	8801.76027	0.65119657	0.23676879	2.75034804	0.0059532	0.03350999
ENSG000001	80.4172726	0.62890235	0.17402796	3.61380071	0.00030174	0.00400619
ENSG000001	76.8398357	1.05694492	0.21848794	4.83754355	1.31E-06	5.21E-05
ENSG000001	5930.92661	-0.2260739	0.06663114	-3.3929167	0.00069153	0.00752919
ENSG000001	1724.16017	0.28845817	0.10303361	2.79965109	0.00511579	0.03045877
ENSG000001	4615.64172	-0.2802857	0.08456379	-3.3144885	0.00091811	0.00916266
ENSG000001	123.407935	-1.0712571	0.32634315	-3.2826093	0.00102851	0.00994955

ENSG000001	92.9824932	-0.406398	0.21697591	-1.8730097	0.06106705	0.16215651
ENSG000001	448.514399	0.16307326	0.08448528	1.93019741	0.05358238	0.14817786
ENSG000001	441.928578	1.15507751	0.17903065	6.45184224	1.10E-10	1.85E-08
ENSG000001	571.50484	0.5661372	0.25484804	2.22146968	0.02631917	0.09198737
ENSG000001	1284.64632	0.23133001	0.08220593	2.81403067	0.00489246	0.02943912
ENSG000001	825.656347	1.50660101	0.31642495	4.76132186	1.92E-06	7.06E-05
ENSG000001	4890.08902	0.26236306	0.1076429	2.43734665	0.01479549	0.06242965
ENSG000001	3391.81579	-0.1761059	0.05901019	-2.9843307	0.00284199	0.02020823
ENSG000001	1067.81646	-0.1532368	0.05041653	-3.0394167	0.00237037	0.01778806
ENSG000001	10944.3702	-0.1666532	0.07816766	-2.1319971	0.03300709	0.10717172
ENSG000001	715.433775	-0.5510202	0.12389548	-4.44746	8.69E-06	0.00025172
ENSG000001	26920.3069	-0.2648588	0.11084131	-2.3895315	0.01686988	0.06841834
ENSG000001	259.39168	-0.2017763	0.09722196	-2.0754196	0.03794767	0.11713628
ENSG000001	2858.84184	0.21726353	0.10818563	2.00824761	0.04461699	0.13057018
ENSG000001	151.09503	-0.2421827	0.09281588	-2.6092812	0.00907327	0.04486561
ENSG000001	10425.4586	0.21506059	0.10990992	1.95669855	0.05038292	0.14241144
ENSG000001	608.573145	0.140929	0.07717278	1.82614905	0.06782781	0.1740756
ENSG000001	1892.00016	-0.1640713	0.06487782	-2.5289276	0.01144116	0.05257868
ENSG000001	11293.994	0.37806318	0.19587284	1.93014601	0.05358875	0.14817786
ENSG000001	604.282965	0.58023927	0.19272403	3.01072607	0.00260624	0.01897624
ENSG000001	202.370442	0.30852856	0.13752546	2.2434287	0.02486918	0.08864782
ENSG000001	1835.18879	0.45109476	0.10067625	4.48064699	7.44E-06	0.00022267
ENSG000001	8609.01023	-0.1053551	0.04936654	-2.1341399	0.03283133	0.10668556
ENSG000001	2522.33395	-0.1893733	0.09135908	-2.0728459	0.03818662	0.1176082
ENSG000001	8782.18412	2.13941777	0.32928945	6.49707358	8.19E-11	1.48E-08
ENSG000001	192.227601	-0.8723615	0.27655732	-3.1543604	0.0016085	0.01366561
ENSG000001	1588.12985	0.12751684	0.06560188	1.9437987	0.05191973	0.14517869
ENSG000001	5617.80626	0.67289477	0.13381558	5.02852355	4.94E-07	2.38E-05
ENSG000001	12148.6271	-0.1253411	0.04615289	-2.7157802	0.00661198	0.03604769
ENSG000001	2654.91687	-0.1417495	0.06409681	-2.2114912	0.02700184	0.09364761
ENSG000001	656.142913	0.47149409	0.09455133	4.98664698	6.14E-07	2.80E-05
ENSG000001	10320.8388	0.46716631	0.19589511	2.38477785	0.01708944	0.06898462
ENSG000001	1175.93917	0.30531458	0.10235069	2.98302414	0.00285415	0.02027711
ENSG000001	5716.11895	-0.151234	0.07369371	-2.0521963	0.04015059	0.12169116
ENSG000001	1130.67201	-0.3323414	0.14613248	-2.2742471	0.02295113	0.08394595
ENSG000001	2319.68858	0.39168583	0.10512698	3.72583538	0.00019467	0.00297107
ENSG000001	3386.62529	-0.2684719	0.13666441	-1.9644607	0.04947669	0.1404552
ENSG000001	3989.11266	0.59632206	0.12392632	4.81190814	1.49E-06	5.80E-05
ENSG000001	1975.46981	-0.1950186	0.10204591	-1.9110871	0.05599339	0.15253756
ENSG000001	2404.4015	0.6213433	0.21646445	2.87041731	0.0040993	0.02608315
ENSG000001	1189.0038	0.16491161	0.05439663	3.03165109	0.0024322	0.01807834
ENSG000001	6322.78185	0.41113106	0.1339147	3.0700966	0.0021399	0.01653528
ENSG000001	1089.46471	-0.3109791	0.09178008	-3.3883071	0.00070325	0.00759136

ENSG000001	63.9208084	0.82852056	0.21254756	3.8980478	9.70E-05	0.00176657
ENSG000001	118.22814	-0.3313903	0.1616846	-2.0496096	0.04040255	0.12204874
ENSG000001	487.617093	0.35352936	0.12208983	2.89564962	0.00378375	0.02466005
ENSG000001	104.521194	1.18764866	0.22733705	5.22417559	1.75E-07	1.01E-05
ENSG000001	339.918555	-0.2172481	0.09711635	-2.2369881	0.02528712	0.08963698
ENSG000001	76.7716778	1.33608583	0.22932486	5.82617081	5.67E-09	5.28E-07
ENSG000001	1313.53088	0.24767277	0.1166282	2.12360975	0.03370279	0.10869879
ENSG000001	4547.49636	0.22118426	0.09523074	2.32261414	0.02019989	0.07711702
ENSG000001	18526.932	0.45436848	0.09216819	4.92977543	8.23E-07	3.56E-05
ENSG000001	157.681818	0.33081069	0.13944597	2.3723216	0.0176767	0.07048755
ENSG000001	3344.74713	-0.2452833	0.06635229	-3.696682	0.00021844	0.00319224
ENSG000001	7189.40823	0.26366653	0.13332044	1.97769029	0.04796366	0.13739694
ENSG000001	770.499875	-0.2169988	0.09897182	-2.1925316	0.02834115	0.0965731
ENSG000001	674.947816	-0.2869734	0.13362928	-2.1475337	0.03175082	0.10436342
ENSG000001	302.260391	-0.3853659	0.17268999	-2.2315474	0.02564489	0.09052729
ENSG000001	262.367778	-0.2679004	0.13100772	-2.0449208	0.04086267	0.1228543
ENSG000001	10915.8932	0.39346167	0.22227544	1.77015362	0.07670155	0.18851853
ENSG000001	105.649427	-0.3705059	0.15598595	-2.3752515	0.017537	0.07018651
ENSG000001	45288.7204	0.50823492	0.1902991	2.67071629	0.00756896	0.03953302
ENSG000001	667.614852	0.38209128	0.17825123	2.14355479	0.03206858	0.10508162
ENSG000001	11175.9569	0.38991548	0.15737022	2.4776955	0.01322339	0.05806749
ENSG000001	6836.98313	-0.173939	0.06706316	-2.5936602	0.00949603	0.04622542
ENSG000001	628.80414	-0.2042681	0.09419917	-2.1684702	0.03012293	0.1007806
ENSG000001	8082.3303	0.21914475	0.12619559	1.73654834	0.08246692	0.19829512
ENSG000001	239.012957	0.19337433	0.09598561	2.01461801	0.04394469	0.12922544
ENSG000001	3762.14766	-0.3892891	0.132382	-2.9406494	0.00327525	0.02236746
ENSG000001	3022.54474	0.40968867	0.14712304	2.78466697	0.00535827	0.03137843
ENSG000001	1583.28846	-0.37083	0.09671829	-3.8341246	0.00012601	0.00215681
ENSG000001	84053.9045	0.47902774	0.19347313	2.47593935	0.01328861	0.05822913
ENSG000001	6667.33435	0.24993681	0.10000871	2.49915053	0.01244914	0.05567861
ENSG000001	12312.4285	-0.1053288	0.04678178	-2.2514915	0.02435442	0.08751687
ENSG000001	8060.83342	0.42470197	0.16028445	2.64967668	0.00805688	0.04123243
ENSG000001	3235.47248	0.4433661	0.21180228	2.09330181	0.03632222	0.11389854
ENSG000001	466.151821	-0.4701987	0.18436514	-2.5503666	0.01076097	0.05034588
ENSG000001	7991.03953	0.55137149	0.12986868	4.24560805	2.18E-05	0.00053114
ENSG000001	1563.46264	0.28398089	0.07115685	3.9909142	6.58E-05	0.00128328
ENSG000001	19800.6749	-0.279318	0.10783676	-2.5901933	0.00959221	0.04645343
ENSG000001	1024.7828	-0.1587461	0.08820736	-1.7996922	0.07190926	0.18103732
ENSG000001	216.234108	-0.2471842	0.08644778	-2.8593471	0.00424514	0.0267105
ENSG000001	1179.013	0.45628781	0.23010523	1.98295277	0.04737271	0.13625158
ENSG000001	188.495861	-0.2885519	0.15480028	-1.8640272	0.06231788	0.16436541
ENSG000001	7018.72329	0.76772896	0.18432149	4.16516254	3.11E-05	0.00071868
ENSG000001	10148.5789	0.29037676	0.10832172	2.6806882	0.00734709	0.03884314

ENSG000001	3173.29202	0.29756177	0.09203577	3.23311006	0.0012245	0.0112609
ENSG000001	1548.12981	0.37837882	0.15933156	2.37478882	0.01755899	0.070219
ENSG000001	287.451938	-0.5913541	0.20348524	-2.9061276	0.00365932	0.02413534
ENSG000001	1811.67022	-0.1168493	0.06162255	-1.8962094	0.05793236	0.15641642
ENSG000001	360.03787	-0.4775496	0.16719761	-2.8561987	0.00428747	0.0268737
ENSG000001	7525.00469	0.38893237	0.11862655	3.27862843	0.00104313	0.01002003
ENSG000001	171.51866	-0.2278664	0.12683041	-1.7966229	0.07239551	0.18178701
ENSG000001	43.5232292	0.53975	0.23240927	2.32241164	0.02021078	0.07714064
ENSG000001	266.380515	-0.3787772	0.16826286	-2.2511039	0.02437896	0.0875576
ENSG000001	6478.95186	0.27961461	0.13503401	2.07069769	0.03838706	0.11798173
ENSG000001	3699.94929	0.4565545	0.17720569	2.57640995	0.00998322	0.04779412
ENSG000001	1275.55797	0.42541779	0.15143779	2.8091918	0.0049666	0.02976514
ENSG000001	18552.798	0.2799219	0.11705028	2.39146725	0.01678118	0.06816093
ENSG000001	156.433047	-0.2582808	0.13767497	-1.8760187	0.06065272	0.16142227
ENSG000001	3855.77213	-0.1223936	0.04756378	-2.5732516	0.01007479	0.04807811
ENSG000001	4028.4854	-0.2826211	0.13380252	-2.1122252	0.03466714	0.11069856
ENSG000001	2065.00271	-0.1541739	0.06197262	-2.4877745	0.01285452	0.05702802
ENSG000001	2003.25488	0.24393012	0.07895649	3.08942473	0.00200545	0.01580927
ENSG000001	4427.2607	0.74841122	0.11810625	6.33676241	2.35E-10	3.47E-08
ENSG000001	776.50563	-0.4565614	0.11060577	-4.1278261	3.66E-05	0.00081808
ENSG000001	79512.6014	0.22630103	0.12863344	1.7592707	0.07853154	0.19156228
ENSG000001	137.467017	0.2874968	0.13621349	2.11063384	0.0348038	0.11102682
ENSG000001	2137.40195	0.29573192	0.11515337	2.56815696	0.01022408	0.0485644
ENSG000001	1907.1625	0.15006275	0.0670669	2.23750823	0.02525314	0.08956863
ENSG000001	534.052353	0.16774824	0.0866145	1.93672238	0.05277929	0.14678121
ENSG000001	8050.60667	0.12234138	0.07035392	1.73894177	0.08204501	0.19748855
ENSG000001	1651.63931	-0.1821038	0.06219619	-2.9278928	0.00341268	0.02298056
ENSG000001	1578.41532	0.34497958	0.1577166	2.18733841	0.02871783	0.09745162
ENSG000001	4809.27323	0.1198853	0.05958095	2.01214139	0.04420504	0.12975832
ENSG000001	1956.46105	0.4629835	0.12702055	3.64494963	0.00026744	0.00367893
ENSG000001	18874.3665	0.15774836	0.06457586	2.44283794	0.01457228	0.06180591
ENSG000001	480.478642	0.65537919	0.16294777	4.02202003	5.77E-05	0.0011695
ENSG000001	7362.29775	-0.1786614	0.04851723	-3.6824326	0.00023102	0.003317
ENSG000001	1876.47761	2.10022935	0.2716406	7.73164749	1.06E-14	1.02E-11
ENSG000001	14118.2298	-0.1109806	0.05168464	-2.1472652	0.03177218	0.10440248
ENSG000001	178.952175	0.59240728	0.24588499	2.40928603	0.01598377	0.06598333
ENSG000001	10496.0706	0.39193989	0.13348979	2.93610393	0.00332363	0.02258498
ENSG000001	628.940363	-0.4074205	0.20013696	-2.0357086	0.04177963	0.12465632
ENSG000001	2919.01536	0.25499332	0.09360517	2.72413712	0.00644697	0.03541408
ENSG000001	994.336906	0.55154306	0.18031548	3.05876704	0.0022225	0.01698151
ENSG000001	1302.22881	0.2028609	0.07588876	2.67313516	0.0075146	0.03936345
ENSG000001	3497.18635	0.87897202	0.21225837	4.14104769	3.46E-05	0.00078083
ENSG000001	7297.25632	-0.1914651	0.05017775	-3.8157367	0.00013578	0.00228103

ENSG000001	61613.576	0.84842559	0.24328976	3.48730495	0.00048791	0.00578059
ENSG000001	10230.3648	0.17377166	0.07545599	2.30295377	0.02128144	0.08001647
ENSG000001	9553.18933	0.14763389	0.06286724	2.34834376	0.01885711	0.0735665
ENSG000001	1085.1824	0.21243827	0.07317349	2.90321352	0.00369355	0.02424282
ENSG000001	691.207923	0.22692258	0.12907304	1.75809435	0.07873146	0.19190449
ENSG000001	1821.28924	0.76085366	0.24093849	3.15787515	0.00158924	0.01354402
ENSG000001	4144.32807	1.07456587	0.18426471	5.8316423	5.49E-09	5.17E-07
ENSG000001	20287.9301	1.02487814	0.31897104	3.21307587	0.00131322	0.01184187
ENSG000001	1014.8004	0.19574305	0.10987075	1.78157573	0.07481844	0.18565343
ENSG000001	2547.32726	0.50069276	0.1074811	4.65842605	3.19E-06	0.00010728
ENSG000001	1206.02218	-0.2809189	0.07677559	-3.6589604	0.00025324	0.00354602
ENSG000001	1634.33582	0.2019551	0.10984872	1.83848389	0.06599113	0.1708061
ENSG000001	1843.05675	0.709951	0.24615271	2.88418928	0.00392423	0.02528633
ENSG000001	2121.23849	0.93610129	0.34531224	2.71088365	0.00671042	0.03643401
ENSG000001	192.318345	-0.4082411	0.15157113	-2.693396	0.00707282	0.0378873
ENSG000001	155.19256	-0.4146228	0.1749618	-2.3697907	0.01779816	0.07071951
ENSG000001	44448.4542	0.60013572	0.20541003	2.92164763	0.00348185	0.02328382
ENSG000001	5307.60071	0.16909307	0.06262422	2.70012261	0.00693139	0.03734934
ENSG000001	2772.92189	0.33883446	0.12244874	2.76715348	0.00565481	0.03239761
ENSG000001	2429.67127	-0.1500486	0.0802614	-1.8694984	0.06155351	0.16313063
ENSG000001	163.208479	-0.3316837	0.13917316	-2.3832448	0.01716078	0.06918743
ENSG000001	3613.6363	0.84632384	0.23030045	3.67486833	0.00023797	0.00338424
ENSG000001	962.633836	-0.2167411	0.07268921	-2.9817506	0.00286605	0.02034402
ENSG000001	2487.45308	0.10922041	0.04317393	2.52977674	0.01141351	0.05248103
ENSG000001	2898.98278	-0.1246319	0.0546638	-2.2799714	0.02260939	0.08308519
ENSG000001	1268.35693	-0.2045149	0.08330253	-2.4550864	0.01408507	0.0603483
ENSG000001	5293.30264	-0.1594069	0.06266007	-2.5439952	0.01095926	0.05090623
ENSG000001	1921.09912	0.47132749	0.19390973	2.43065418	0.01507159	0.06320968
ENSG000001	1254.44714	0.14805647	0.08110157	1.82556842	0.0679153	0.17416439
ENSG000001	1149.48968	0.10683642	0.05750186	1.85796456	0.06317405	0.16584452
ENSG000001	160.303617	-0.4793801	0.12018348	-3.9887349	6.64E-05	0.00129052
ENSG000001	872.010837	0.94644448	0.21965516	4.30877417	1.64E-05	0.0004157
ENSG000001	869.887654	-0.2476123	0.10459593	-2.3673228	0.0179173	0.07094661
ENSG000001	3705.04891	0.5015476	0.11730404	4.27562094	1.91E-05	0.00047211
ENSG000001	1287.14457	-0.1177314	0.04854333	-2.4252853	0.01529636	0.06393434
ENSG000001	180.787847	-0.3994715	0.15717099	-2.5416363	0.01103349	0.05119302
ENSG000001	7022.19883	-0.1364398	0.07060334	-1.9324835	0.05329986	0.1476779
ENSG000001	764.986538	0.14232979	0.07650414	1.86041948	0.0628262	0.16519583
ENSG000001	42181.7215	0.87709064	0.1812439	4.83928355	1.30E-06	5.18E-05
ENSG000001	10280.2396	1.13439621	0.33209741	3.41585385	0.00063582	0.0070778
ENSG000001	6748.04666	0.75494624	0.11995624	6.29351391	3.10E-10	4.43E-08
ENSG000001	1972.06219	0.41323517	0.12989829	3.18122104	0.00146656	0.01278767
ENSG000001	4161.13225	0.57321353	0.24851518	2.30655336	0.02107973	0.07958655

ENSG000001	172.953569	-0.2518756	0.09581384	-2.6288017	0.00856863	0.04319085
ENSG000001	219.467811	-0.2520405	0.10246362	-2.4598047	0.01390126	0.05988939
ENSG000001	3328.47339	0.10598604	0.05542869	1.91211537	0.0558614	0.15230451
ENSG000001	128.962041	1.14248394	0.4459596	2.56185526	0.01041147	0.04911277
ENSG000001	4845.71473	0.30078527	0.15220912	1.97613176	0.04813986	0.13775728
ENSG000001	662.406416	0.2055288	0.09495304	2.16453101	0.03042362	0.10143475
ENSG000001	9493.13627	0.39854472	0.19936067	1.99911406	0.04559601	0.13260692
ENSG000001	2872.05662	0.22226724	0.09180144	2.42117376	0.01547048	0.06448131
ENSG000001	12456.032	0.84965112	0.16244425	5.23041659	1.69E-07	9.90E-06
ENSG000001	2932.24259	0.57337779	0.1593117	3.59909408	0.00031933	0.00418212
ENSG000001	228.137308	1.14642767	0.26874257	4.26589532	1.99E-05	0.00048872
ENSG000001	51983.8067	0.90141786	0.23268538	3.87397714	0.00010707	0.00190009
ENSG000001	575.48507	-0.2534385	0.06808917	-3.7221551	0.00019753	0.00299897
ENSG000001	765.516158	-0.2652641	0.08604526	-3.0828434	0.00205033	0.01604136
ENSG000001	124.340615	1.07069638	0.25306063	4.23098763	2.33E-05	0.00055776
ENSG000001	96.8192747	0.49290474	0.15897818	3.10045521	0.00193223	0.0154777
ENSG000001	16431.9652	1.91344904	0.25110653	7.62006871	2.54E-14	2.04E-11
ENSG000001	126.004225	-0.5017773	0.27277766	-1.8395101	0.06584019	0.17052308
ENSG000001	12399.164	0.48650991	0.13024264	3.73541191	0.00018741	0.00288809
ENSG000001	968.718402	-0.2762732	0.07241795	-3.814982	0.00013619	0.00228339
ENSG000001	3491.49459	0.56111985	0.12992193	4.31890007	1.57E-05	0.00040175
ENSG000001	616.663138	0.32782492	0.07351962	4.45901239	8.23E-06	0.00024195
ENSG000001	236.658585	1.1456525	0.24007082	4.77214396	1.82E-06	6.78E-05
ENSG000001	382.948121	-0.2138267	0.06147826	-3.4780868	0.00050501	0.00594443
ENSG000001	882.390574	1.60601997	0.28881344	5.56075212	2.69E-08	2.00E-06
ENSG000001	985.713963	0.24355934	0.13221267	1.84217857	0.06544903	0.16991572
ENSG000001	1649.90577	0.37142352	0.07725306	4.80788115	1.53E-06	5.87E-05
ENSG000001	136.826491	-0.2939094	0.14323705	-2.0519087	0.04017854	0.12170838
ENSG000001	2144.2756	0.25861916	0.10840498	2.38567606	0.01704776	0.06890187
ENSG000001	42963.8519	3.32589083	0.46200355	7.1988426	6.07E-13	2.37E-10
ENSG000001	152.015048	0.80029592	0.26257442	3.04788223	0.0023046	0.01744624
ENSG000001	13617.9897	2.82305174	0.39535283	7.14058811	9.29E-13	3.39E-10
ENSG000001	46.1266139	-2.0178733	0.65751112	-3.0689569	0.00214808	0.01655947
ENSG000001	1816.41687	0.13925406	0.05988545	2.32534029	0.02005377	0.07678232
ENSG000001	601.953771	-0.1671655	0.09377654	-1.7825937	0.07465247	0.18541587
ENSG000001	2015.12933	0.169016	0.08446658	2.00098074	0.04539447	0.13216141
ENSG000001	6461.86649	0.15509682	0.0683327	2.26973081	0.02322392	0.08468988
ENSG000001	1908.93261	0.97767215	0.14334559	6.82038545	9.08E-12	2.22E-09
ENSG000001	4188.89273	0.16838596	0.09123492	1.84563055	0.06494587	0.16916877
ENSG000001	2152.22658	-0.2112066	0.07485813	-2.8214255	0.00478107	0.02905681
ENSG000001	5095.44276	-0.2069871	0.0830407	-2.4925986	0.01268121	0.05647306
ENSG000001	712.439367	-0.2122901	0.08634519	-2.4586212	0.01394717	0.05997685
ENSG000001	322.046142	0.32680177	0.11154026	2.92989965	0.00339071	0.02291737

ENSG000001	487.665604	-0.1321539	0.06316102	-2.0923324	0.03640879	0.11396472
ENSG000001	491.57833	1.0035655	0.21309634	4.70944497	2.48E-06	8.73E-05
ENSG000001	1141.36543	0.33933576	0.16681303	2.03422811	0.0419286	0.12495516
ENSG000001	96.1642241	1.40457371	0.26780244	5.24481287	1.56E-07	9.26E-06
ENSG000001	709.420856	0.3876315	0.16951971	2.28664555	0.02221652	0.08223139
ENSG000001	3589.07015	0.32236711	0.15077651	2.13804598	0.03251301	0.10596618
ENSG000001	610.914689	-0.2712188	0.10971103	-2.4721198	0.01343145	0.05860443
ENSG000001	8847.50022	0.20120632	0.09076811	2.21670717	0.02664311	0.09279249
ENSG000001	121.030265	-0.5408908	0.23683053	-2.2838727	0.02237902	0.08250274
ENSG000001	736.884721	0.4749597	0.17691116	2.68473572	0.00725872	0.03856811
ENSG000001	652.985319	-0.2731141	0.10686961	-2.5555824	0.01060103	0.0498066
ENSG000001	1280.10134	0.39511311	0.11973776	3.29982048	0.00096747	0.00951091
ENSG000001	659.616772	0.43703717	0.18951997	2.30602179	0.02110941	0.07962528
ENSG000001	1054.22624	0.16950803	0.08228009	2.06013421	0.03938571	0.12010555
ENSG000001	3288.71711	0.18492727	0.09711246	1.90425894	0.05687647	0.15432789
ENSG000001	1069.40108	0.74272807	0.23985831	3.09652844	0.00195801	0.01557008
ENSG000001	2679.73653	0.27591603	0.09635512	2.86353277	0.00418945	0.0265025
ENSG000001	2686.38142	0.26818837	0.11506833	2.33068796	0.01976982	0.07604169
ENSG000001	8904.1393	0.2103446	0.11858491	1.77378893	0.07609807	0.18749638
ENSG000001	2638.41093	-0.3043098	0.08795323	-3.4599052	0.00054037	0.0062441
ENSG000001	1422.40172	-0.300593	0.11798184	-2.5477903	0.01084076	0.05059948
ENSG000001	10395.0315	0.50058691	0.17665246	2.83373866	0.00460069	0.02823263
ENSG000001	618.436922	0.15816847	0.0638067	2.47886921	0.01317996	0.0579233
ENSG000001	18764.0466	1.23531785	0.19383441	6.37305748	1.85E-10	2.92E-08
ENSG000001	6072.57265	0.10823059	0.04780798	2.26386032	0.02358271	0.08579413
ENSG000001	4336.24331	0.19638305	0.07857257	2.49938437	0.01244093	0.05567861
ENSG000001	120.698538	-0.2190096	0.12531898	-1.7476174	0.08053031	0.19490117
ENSG000001	2821.60644	0.20553035	0.09619784	2.13653812	0.03263558	0.10630226
ENSG000001	3089.09262	0.70624928	0.15658705	4.51026624	6.47E-06	0.00019733
ENSG000001	3603.55494	0.20451458	0.10091163	2.02667002	0.04269617	0.12652975
ENSG000001	937.75654	-0.1893184	0.08688887	-2.1788571	0.02934229	0.09897665
ENSG000001	5010.11556	1.56492017	0.21559161	7.25872481	3.91E-13	1.73E-10
ENSG000001	13913.1311	2.38345479	0.33867854	7.03751338	1.96E-12	6.34E-10
ENSG000001	5739.7752	-0.1086633	0.04446089	-2.4440188	0.01452467	0.06163587
ENSG000001	3206.3612	0.37654236	0.18495035	2.03591046	0.04175935	0.12465632
ENSG000001	588.839132	0.24996098	0.12481355	2.00267494	0.04521219	0.13184142
ENSG000001	40246.2548	0.29305908	0.09663883	3.03251882	0.00242522	0.01804905
ENSG000001	967.461182	0.85534421	0.28399047	3.01187642	0.00259638	0.01892128
ENSG000001	3836.96503	-0.2197812	0.11884869	-1.8492522	0.06442141	0.16814993
ENSG000001	7077.88404	0.27740818	0.11071427	2.50562269	0.0122236	0.05504816
ENSG000001	3235.85121	-0.2598254	0.09439925	-2.7524095	0.00591585	0.03336849
ENSG000001	4108.60917	0.43464395	0.08579625	5.06600176	4.06E-07	2.01E-05
ENSG000001	11967.9006	-0.1337427	0.07266016	-1.840661	0.06567125	0.17032767

ENSG000001	4012.28577	0.98752049	0.33768804	2.9243573	0.00345168	0.02315385
ENSG000001	367.260696	1.31658028	0.27256921	4.8302605	1.36E-06	5.37E-05
ENSG000001	488.791604	0.18027833	0.05760245	3.12969926	0.00174985	0.01442551
ENSG000001	985.86212	-0.2665895	0.08075236	-3.3013218	0.0009623	0.00947682
ENSG000001	2801.85868	-0.2646312	0.11899099	-2.2239603	0.02615112	0.09156521
ENSG000001	4545.41245	-0.1374172	0.07633924	-1.8000865	0.07184699	0.18096391
ENSG000001	3196.12867	0.20261715	0.07452486	2.71878621	0.00655219	0.03585992
ENSG000001	29633.7781	0.42180642	0.19827019	2.12743237	0.03338418	0.1079685
ENSG000001	1573.87289	0.27331649	0.0833263	3.28007488	0.0010378	0.00998634
ENSG000001	25.0839681	0.9305554	0.33660355	2.76454424	0.00570024	0.03249889
ENSG000001	832.224085	-0.3190171	0.07155547	-4.4583193	8.26E-06	0.0002423
ENSG000001	4463.2907	0.57594198	0.16449187	3.50134003	0.00046292	0.00555273
ENSG000001	802.953209	-0.296873	0.14498221	-2.0476513	0.04059418	0.12236375
ENSG000001	6677.98937	0.53445691	0.19235523	2.77848907	0.00546123	0.03169127
ENSG000001	1811.26721	-0.1734973	0.079799	-2.1741785	0.02969174	0.09979735
ENSG000001	480.936755	0.36242231	0.11087167	3.26884518	0.00107987	0.0102707
ENSG000001	413.2166	0.23754549	0.07997337	2.97030724	0.00297502	0.02082912
ENSG000001	250.459359	-0.5033682	0.13959934	-3.6058067	0.00031118	0.00410168
ENSG000001	242.173976	-0.2837677	0.15654478	-1.8126934	0.06987913	0.17731958
ENSG000001	434.985491	0.27899529	0.14963007	1.86456695	0.06224213	0.16436541
ENSG000001	2161.66841	-0.3889312	0.1760659	-2.2090093	0.027174	0.09404222
ENSG000001	2737.8429	0.28508022	0.07830278	3.640742	0.00027185	0.00372085
ENSG000001	110.082121	0.19366301	0.10365703	1.86830558	0.06171949	0.16341264
ENSG000001	19460.2516	0.1241897	0.06929508	1.79218644	0.07310311	0.1830033
ENSG000001	267.657113	-0.2734634	0.14597549	-1.8733512	0.06101991	0.16211008
ENSG000001	184.678823	1.0041723	0.25534979	3.93253628	8.41E-05	0.00156879
ENSG000001	86.4482607	0.83907806	0.19252032	4.35838689	1.31E-05	0.00035162
ENSG000001	387.85237	-0.1909235	0.09868637	-1.9346496	0.05303331	0.147213
ENSG000001	7718.89662	0.26387518	0.10538651	2.50388018	0.01228396	0.05524414
ENSG000001	6484.60356	0.31552245	0.11208895	2.81492915	0.0048788	0.02943912
ENSG000001	114.648049	1.13336804	0.29197298	3.88175665	0.0001037	0.00186248
ENSG000001	217.961139	0.3084881	0.08716488	3.53913297	0.00040144	0.00502479
ENSG000001	1122.23372	1.05052863	0.16622103	6.32007035	2.61E-10	3.76E-08
ENSG000001	1910.15499	-0.0806165	0.04169634	-1.9334194	0.05318455	0.14748301
ENSG000001	983.227186	0.35115385	0.12168994	2.88564406	0.00390614	0.02521611
ENSG000001	2701.04479	0.09446146	0.05288914	1.78602757	0.07409479	0.18458407
ENSG000001	8147.54439	-0.2984069	0.11171652	-2.6711086	0.00756012	0.0395165
ENSG000001	4152.34949	-0.1368686	0.0563069	-2.43076	0.01506719	0.06320968
ENSG000001	211.019743	-0.193913	0.1077407	-1.799812	0.07189033	0.18101746
ENSG000001	4789.54064	0.15121481	0.07460564	2.02685495	0.04267725	0.12649809
ENSG000001	7491.56969	-0.1625969	0.04769733	-3.40893	0.00065218	0.00721094
ENSG000001	1848.02539	0.08688635	0.0468236	1.85561019	0.06350914	0.1664577
ENSG000001	109.1211	1.59124257	0.29037851	5.47989093	4.26E-08	3.06E-06

ENSG000001	5386.83241	-0.1864555	0.06572096	-2.837078	0.00455285	0.02802292
ENSG000001	3151.32358	-0.1750941	0.0839583	-2.0854886	0.03702498	0.11513342
ENSG000001	3094.97884	0.21946904	0.12246888	1.79203931	0.07312667	0.18303435
ENSG000001	798.492858	0.39614846	0.18788877	2.10842014	0.03499466	0.11131086
ENSG000001	571.850612	0.58634527	0.17051837	3.43860473	0.00058472	0.00660307
ENSG000001	2458.28929	0.31033088	0.10340116	3.00123213	0.00268889	0.01943138
ENSG000001	8289.61503	0.28381841	0.12509396	2.26884175	0.02327795	0.08483853
ENSG000001	319.129694	-0.291383	0.11027098	-2.6424269	0.00823142	0.04195544
ENSG000001	3103.31922	0.2383956	0.11018543	2.1635855	0.03049618	0.10163532
ENSG000001	92.1105338	-0.4230402	0.12914852	-3.2756104	0.00105434	0.01011585
ENSG000001	2558.86849	0.8009	0.21769997	3.67891646	0.00023423	0.0033484
ENSG000001	1015.54309	0.10868637	0.05179583	2.09836128	0.03587324	0.11304393
ENSG000001	1793.64153	0.08451187	0.04151062	2.03590953	0.04175944	0.12465632
ENSG000001	4862.72694	-0.1873955	0.0653462	-2.8677332	0.00413424	0.0262139
ENSG000001	20819.2498	0.43501036	0.16165109	2.69104506	0.00712286	0.03808069
ENSG000001	1268.0054	0.51272867	0.11561608	4.43475211	9.22E-06	0.00026378
ENSG000001	85.1777674	0.73114861	0.2494203	2.93139177	0.00337447	0.02281698
ENSG000001	457.819287	-0.7723916	0.24809492	-3.1132909	0.00185014	0.01504795
ENSG000001	682.520993	0.26854986	0.06948755	3.86471907	0.00011122	0.00195668
ENSG000001	6774.64726	0.24716519	0.08556502	2.88862411	0.00386931	0.02499479
ENSG000001	3113.35848	0.19234853	0.10316749	1.86442965	0.06226139	0.16436541
ENSG000001	1440.43032	1.21039345	0.27044093	4.47562967	7.62E-06	0.00022631
ENSG000001	1928.46133	-0.1535873	0.0707969	-2.1694074	0.03005176	0.10060408
ENSG000001	2654.34006	-0.1811812	0.09012703	-2.0102867	0.04440085	0.130142
ENSG000001	1388.9878	-0.1147913	0.05309253	-2.1620991	0.03061054	0.1019129
ENSG000001	19484.2772	0.19591942	0.10454656	1.87399211	0.06093151	0.1619511
ENSG000001	2835.79394	0.477557	0.22947125	2.08111909	0.03742301	0.11608981
ENSG000001	70.7289825	0.71385978	0.1758457	4.05958047	4.92E-05	0.00102037
ENSG000001	8373.6543	0.20164116	0.09215793	2.18799572	0.02866991	0.09736963
ENSG000001	977.801912	-0.0844901	0.04509682	-1.8735274	0.06099559	0.16207174
ENSG000001	1466.71861	0.9708292	0.16496353	5.88511407	3.98E-09	4.03E-07
ENSG000001	252.525482	-0.537125	0.16143797	-3.3271291	0.00087746	0.00890596
ENSG000001	1070.38776	0.48497418	0.1501891	3.22909033	0.00124185	0.01134459
ENSG000001	15881.7491	-0.280622	0.12240436	-2.2925816	0.0218721	0.08171766
ENSG000001	57.2796378	1.32958336	0.2810481	4.73080361	2.24E-06	7.95E-05
ENSG000001	312.503319	-0.355471	0.10407785	-3.4154334	0.00063681	0.00708393
ENSG000001	2060.93018	0.2332054	0.0969449	2.40554582	0.01614833	0.06646188
ENSG000001	6396.96649	0.67581097	0.18518772	3.64932925	0.00026293	0.00363606
ENSG000001	2074.55406	0.12353941	0.06086907	2.02959254	0.04239797	0.12601044
ENSG000001	4304.8561	0.19578438	0.06548021	2.98997766	0.00278998	0.01995841
ENSG000001	3044.0592	-0.2852331	0.09816234	-2.9057288	0.00366399	0.02413534
ENSG000001	3755.83814	0.20032685	0.08562708	2.33952676	0.01930819	0.07462986
ENSG000001	642.422393	0.75838706	0.17927647	4.2302655	2.33E-05	0.00055873

ENSG000001	1089.24879	0.24773107	0.07642599	3.24145063	0.00118923	0.01101062
ENSG000001	815.667818	-0.1725461	0.09656553	-1.7868295	0.07396505	0.1843729
ENSG000001	13012.2931	0.38275603	0.10518821	3.63877308	0.00027394	0.00374316
ENSG000001	517.975054	0.18033149	0.07738729	2.33024698	0.0197931	0.07605947
ENSG000001	483.313319	0.41661521	0.14651588	2.84348167	0.00446236	0.02765279
ENSG000001	2818.2042	0.21416356	0.07698836	2.7817655	0.00540641	0.0315028
ENSG000001	93.1135192	0.31812573	0.1084049	2.93460658	0.00333971	0.02265126
ENSG000001	4092.98787	0.51523072	0.15566003	3.30997439	0.00093305	0.00927221
ENSG000001	6573.10355	-0.1239778	0.06124688	-2.0242298	0.04294651	0.12713376
ENSG000001	1249.12498	0.19605492	0.07072632	2.7720222	0.00557092	0.03203619
ENSG000001	2524.823	0.2442302	0.11575337	2.10991871	0.03486536	0.11115833
ENSG000001	566.577544	0.33621535	0.1287132	2.61212801	0.00899806	0.04458783
ENSG000001	3010.76772	0.68042554	0.21392386	3.18069029	0.00146925	0.01278767
ENSG000001	249.013711	0.39177975	0.18734838	2.09118302	0.03651166	0.11412156
ENSG000001	2219.28209	0.41783238	0.14705085	2.8414142	0.00449139	0.02776975
ENSG000001	1217.98333	0.25898554	0.11207295	2.3108657	0.02084027	0.07893713
ENSG000001	1381.86419	-0.3401349	0.12330543	-2.7584749	0.00580718	0.0329596
ENSG000001	2760.08248	0.28960396	0.10984566	2.63646263	0.00837754	0.04251519
ENSG000001	1744.27003	-0.0805631	0.04634741	-1.7382434	0.08216794	0.1977264
ENSG000001	418.086436	0.14769983	0.07556711	1.95455163	0.05063602	0.14291031
ENSG000001	4053.44794	0.1943786	0.11066169	1.75651218	0.07900099	0.19227836
ENSG000001	2286.89741	0.20695597	0.10885713	1.90117054	0.05727968	0.15521647
ENSG000001	745.185382	-0.1039205	0.05783104	-1.796968	0.0723407	0.1816772
ENSG000001	3053.61805	-0.1377256	0.07470808	-1.8435173	0.06525352	0.16972116
ENSG000001	11487.3615	0.28527096	0.12568759	2.26968281	0.02322683	0.08468988
ENSG000001	306.616229	0.18763969	0.08741642	2.14650384	0.0318328	0.10451791
ENSG000001	9026.94897	0.25254163	0.09531935	2.64942685	0.00806284	0.04125006
ENSG000001	744.202428	0.20144634	0.07585548	2.65565959	0.00791535	0.04078817
ENSG000001	17425.944	0.40844336	0.14330176	2.85023258	0.00436873	0.02717527
ENSG000001	150.335155	-0.2199825	0.12438118	-1.7686158	0.07695801	0.18896083
ENSG000001	4137.08359	0.2335477	0.12365894	1.88864387	0.05893956	0.15850944
ENSG000001	744.28799	0.21382007	0.06760429	3.16281826	0.0015625	0.01339993
ENSG000001	1714.73876	-0.1791069	0.10087684	-1.7755005	0.07581528	0.18716398
ENSG000001	6431.83862	0.41264801	0.15519088	2.65897075	0.00783798	0.04046578
ENSG000001	4092.30872	-0.1453835	0.03857753	-3.7686055	0.00016416	0.0026165
ENSG000001	140.892061	0.68199058	0.20430671	3.33807229	0.00084362	0.0086455
ENSG000001	11169.3533	1.45489707	0.20880906	6.96759549	3.22E-12	8.81E-10
ENSG000001	524.519576	0.85730047	0.14039041	6.10654578	1.02E-09	1.26E-07
ENSG000001	325.985525	-0.4510697	0.1706469	-2.6432926	0.00821041	0.04187434
ENSG000001	200.578864	0.2544572	0.11419763	2.22821781	0.02586599	0.0910518
ENSG000001	1994.39201	0.10573812	0.0552401	1.91415498	0.05560036	0.15181999
ENSG000001	218.647521	1.16806367	0.20329448	5.74567332	9.16E-09	8.03E-07
ENSG000001	2801.0212	0.2426593	0.11476696	2.11436553	0.03448406	0.11030599

ENSG000001	11081.5411	0.16253172	0.08696809	1.86886625	0.06164143	0.16327879
ENSG000001	228.956796	0.22591918	0.0816492	2.76694925	0.00565836	0.03240247
ENSG000001	1491.69655	0.27454332	0.11156918	2.4607452	0.01386488	0.05983569
ENSG000001	769.851325	0.22684652	0.0940902	2.41094739	0.01591114	0.0657995
ENSG000001	996.628334	0.63664114	0.15477699	4.11328025	3.90E-05	0.00085238
ENSG000001	8948.52593	1.16650992	0.29023432	4.01920041	5.84E-05	0.00117487
ENSG000001	5622.87408	0.27788433	0.13693082	2.02937751	0.04241985	0.12603236
ENSG000001	554.409452	-0.4245649	0.19765748	-2.1479831	0.0317151	0.10429852
ENSG000001	1928.43443	-0.2272474	0.11484026	-1.9788132	0.04783704	0.1371061
ENSG000001	6076.62465	0.33646124	0.09720967	3.46119093	0.00053779	0.00623192
ENSG000001	738.453714	-0.15171	0.05068371	-2.9932694	0.00276006	0.01978902
ENSG000001	61.2038661	-0.7044588	0.27728193	-2.5405868	0.01106666	0.05131789
ENSG000001	1661.28319	0.27851274	0.09879121	2.81920568	0.00481427	0.02919686
ENSG000001	2423.33521	0.67813141	0.21653226	3.13177997	0.0017375	0.01435977
ENSG000001	1757.00999	0.33192237	0.10363428	3.20282427	0.00136087	0.0121233
ENSG000001	2601.9226	-0.6503194	0.1815983	-3.5810875	0.00034217	0.00441425
ENSG000001	788.785218	-0.1390523	0.06763355	-2.0559669	0.03978571	0.12089811
ENSG000001	37308.2126	0.23596275	0.11683116	2.01969021	0.04341553	0.1281059
ENSG000001	176.899842	0.51338495	0.15965259	3.21563814	0.00130155	0.01177137
ENSG000001	294.838374	1.30634886	0.27797403	4.69953559	2.61E-06	9.04E-05
ENSG000001	4377.00372	0.17782679	0.09453272	1.88111367	0.05995646	0.16029774
ENSG000001	519.242815	0.14955552	0.07343137	2.0366706	0.04168306	0.124563
ENSG000001	4568.92477	0.48796235	0.1320687	3.69476145	0.00022009	0.00320504
ENSG000001	205.827743	0.83503747	0.21098438	3.95781658	7.56E-05	0.00145057
ENSG000001	4268.43615	0.54443253	0.15633031	3.48257822	0.00049661	0.00586665
ENSG000001	836.936796	-0.3864055	0.14682817	-2.6316852	0.00849625	0.04298814
ENSG000001	76.8735723	0.46101239	0.19345992	2.38298658	0.01717282	0.06921407
ENSG000001	316.766702	-0.3725425	0.13626542	-2.7339474	0.006258	0.03471329
ENSG000001	121.409025	0.98647995	0.18383404	5.36614427	8.04E-08	5.28E-06
ENSG000001	210.26222	0.9623574	0.21500197	4.47603993	7.60E-06	0.00022631
ENSG000001	4002.81591	0.57372676	0.11016074	5.20808763	1.91E-07	1.07E-05
ENSG000001	1813.10604	0.1804801	0.06102902	2.95728327	0.00310363	0.02149827
ENSG000001	3598.4673	0.16843404	0.05653623	2.97922311	0.0028898	0.02045945
ENSG000001	4007.24247	0.30619406	0.10484304	2.9204996	0.00349471	0.02333664
ENSG000001	31360.6305	0.1891837	0.08987312	2.10500869	0.03529055	0.11186143
ENSG000001	3286.46241	-0.1654875	0.06191671	-2.6727434	0.00752338	0.03936858
ENSG000001	1997.68938	0.17357376	0.07887971	2.2004869	0.02777237	0.09545508
ENSG000001	1299.77302	0.45180297	0.17246632	2.6196592	0.00880177	0.04385372
ENSG000001	6091.82518	0.38718948	0.20630563	1.87677614	0.06054878	0.16125034
ENSG000001	4396.11231	0.3873538	0.16694154	2.32029608	0.02032487	0.07750391
ENSG000001	336.333848	-0.3128576	0.16069317	-1.9469254	0.05154368	0.14439559
ENSG000001	2009.15553	0.19253378	0.10258061	1.87690225	0.06053149	0.16123049
ENSG000001	1534.35936	0.16069779	0.07316642	2.19633247	0.02806816	0.09602202

ENSG000001	979.74672	0.5837315	0.15012807	3.88822364	0.00010098	0.00182154
ENSG000001	92.4822491	0.45418182	0.19261805	2.35794012	0.01837666	0.07220754
ENSG000001	649.388581	-0.1189806	0.06629736	-1.7946512	0.07270929	0.18234093
ENSG000001	575.414779	0.13014907	0.07064767	1.84222738	0.0654419	0.16991572
ENSG000001	1059.57759	-0.1431537	0.06796738	-2.1062116	0.03518598	0.11170127
ENSG000001	19057.5267	0.55591913	0.22608039	2.45894444	0.01393462	0.0599386
ENSG000001	55.8007141	0.70995635	0.24181981	2.93588991	0.00332592	0.0225912
ENSG000001	10259.4131	0.28191135	0.09006672	3.13002795	0.0017479	0.01442389
ENSG000001	299.498565	0.35703058	0.14745726	2.42124793	0.01546732	0.06448131
ENSG000001	291.137492	-0.5491352	0.19940831	-2.753823	0.00589036	0.03325904
ENSG000001	440.446985	0.8215854	0.17037313	4.82227086	1.42E-06	5.57E-05
ENSG000001	6263.51278	0.27016178	0.12837817	2.10442146	0.0353417	0.11195862
ENSG000001	3055.1096	0.61477368	0.18721117	3.28385142	0.00102399	0.00992924
ENSG000001	127115.103	0.35858087	0.13671682	2.62279991	0.00872105	0.04357069
ENSG000001	262.153116	-0.1783308	0.0817455	-2.1815361	0.02914379	0.09850973
ENSG000001	1939.93701	0.35748368	0.12519663	2.85537787	0.00429857	0.0268862
ENSG000001	19924.8602	0.87724431	0.33617969	2.60945065	0.00906877	0.04486561
ENSG000001	253.828736	1.68315369	0.28057174	5.99901354	1.99E-09	2.22E-07
ENSG000001	2934.22087	-0.1286594	0.0460099	-2.7963409	0.00516849	0.03068344
ENSG000001	448.366916	-0.2851463	0.09148944	-3.1167124	0.0018288	0.01491472
ENSG000001	2038.43455	-0.1748272	0.08249419	-2.1192662	0.03406798	0.10936801
ENSG000001	994.585803	0.45091345	0.12288204	3.66948216	0.00024304	0.00344439
ENSG000001	26893.639	1.2366653	0.36419703	3.39559414	0.0006848	0.00747008
ENSG000001	189.79554	-0.1513394	0.07445906	-2.032518	0.04210124	0.12528748
ENSG000001	633.687069	0.46976537	0.12367497	3.79838664	0.00014564	0.00240586
ENSG000001	2214.22877	0.22077262	0.06364607	3.46875477	0.00052288	0.00609787
ENSG000001	3269.54636	0.21817638	0.07207955	3.0268833	0.00247089	0.01825415
ENSG000001	6045.10021	0.27930336	0.12764856	2.18806519	0.02866485	0.09736963
ENSG000001	3599.05209	-0.1166574	0.04734285	-2.4640972	0.01373589	0.05945627
ENSG000001	622.898753	0.38154345	0.12499588	3.05244824	0.00226983	0.0172627
ENSG000001	2555.72007	0.21562903	0.10518922	2.04991563	0.04037266	0.12200796
ENSG000001	1292.93211	-0.1312702	0.06402808	-2.0501973	0.04034519	0.12196534
ENSG000001	4027.60744	0.33567614	0.09503093	3.53228307	0.00041199	0.00514037
ENSG000001	12885.1303	0.2618292	0.14622964	1.79053444	0.07336804	0.18335859
ENSG000001	2351.82301	0.27353383	0.11649055	2.34812034	0.01886842	0.07359314
ENSG000001	7518.60408	-0.1807641	0.09352439	-1.9328017	0.05326062	0.14759413
ENSG000001	53460.1803	0.88251575	0.22335702	3.95114405	7.78E-05	0.00147608
ENSG000001	840.166789	0.46094761	0.19734183	2.33578253	0.01950258	0.07515483
ENSG000001	206.145979	0.57396183	0.15168875	3.78381282	0.00015444	0.00251232
ENSG000001	7161.92178	0.78319611	0.26054039	3.00604493	0.0026467	0.01920262
ENSG000001	18599.7771	0.3102379	0.13848351	2.24025158	0.02507459	0.08915888
ENSG000001	944.717283	0.39839599	0.11768781	3.38519333	0.00071128	0.00766287
ENSG000001	504.657545	-0.5616433	0.15545099	-3.6129925	0.00030268	0.00401469

ENSG000001	3745.84581	0.251522	0.08571237	2.93448888	0.00334098	0.02265126
ENSG000001	145.107247	-0.3126102	0.08865617	-3.5260962	0.00042173	0.00522004
ENSG000001	3574.34585	0.38527038	0.10931554	3.52438784	0.00042446	0.00523469
ENSG000001	1508.75095	0.96200769	0.15316874	6.28070505	3.37E-10	4.61E-08
ENSG000001	11818.6173	-0.1297762	0.05382007	-2.4112968	0.0158959	0.06576966
ENSG000001	18653.1677	0.33110059	0.13224012	2.50378314	0.01228733	0.05524414
ENSG000001	224.219127	0.31843544	0.15345085	2.07516249	0.03797148	0.11716566
ENSG000001	1253.06217	-0.289941	0.06200352	-4.6762028	2.92E-06	9.96E-05
ENSG000001	2756.18133	0.19999453	0.10176562	1.96524638	0.04938573	0.14026984
ENSG000001	6410.14181	-0.1644409	0.05432321	-3.0270836	0.00246926	0.01825415
ENSG000001	357.157058	0.34794809	0.16849835	2.064994	0.03892357	0.11907271
ENSG000001	1834.4515	-0.2645579	0.09676459	-2.7340366	0.00625631	0.03471329
ENSG000001	10586.2699	-0.1808598	0.06700392	-2.6992419	0.00694976	0.0373869
ENSG000001	56.3821003	2.23633516	0.44706677	5.00223969	5.67E-07	2.61E-05
ENSG000001	1458.80312	-0.2942927	0.08427397	-3.4920956	0.00047925	0.00570262
ENSG000001	1835.16992	0.32600137	0.12528282	2.60212352	0.00926485	0.04552238
ENSG000001	9545.40638	0.35516191	0.16906897	2.1006925	0.03566797	0.11258243
ENSG000001	281.194104	-0.2540308	0.12078989	-2.1030802	0.03545876	0.11215611
ENSG000001	66.6038975	0.71351369	0.25523402	2.79552736	0.00518151	0.03073851
ENSG000001	260.9916	-0.585431	0.18342295	-3.1917	0.00141438	0.01244853
ENSG000001	999.308937	-0.2818851	0.09749691	-2.8912212	0.00383748	0.02485658
ENSG000001	2466.95572	-0.6530315	0.23879013	-2.734751	0.00624275	0.03466384
ENSG000001	1135.52478	-0.1688396	0.05542222	-3.0464236	0.00231581	0.0174988
ENSG000001	1475.85559	-0.2190367	0.11751001	-1.8639831	0.06232408	0.16436541
ENSG000001	540.776989	-0.8677402	0.30419337	-2.8525939	0.0043364	0.02703572
ENSG000001	2913.91243	0.28359119	0.11424416	2.48232554	0.0130528	0.05754953
ENSG000001	186.488356	0.67165208	0.18104439	3.70987507	0.00020736	0.00309863
ENSG000001	8116.64179	-0.1432239	0.05544021	-2.5833934	0.00978337	0.04709612
ENSG000001	112.200612	-0.3923102	0.14548395	-2.6965874	0.0070054	0.03760691
ENSG000001	75.4202152	0.56061933	0.1731476	3.23781178	0.0012045	0.01109563
ENSG000001	1494.09212	-0.1309813	0.04858093	-2.6961471	0.00701467	0.03762496
ENSG000001	100.404701	0.73193328	0.21214806	3.45010602	0.00056037	0.00638272
ENSG000001	2369.54624	-0.1251842	0.07022296	-1.7826672	0.0746405	0.18541587
ENSG000001	4298.34188	0.54022967	0.13107099	4.12165708	3.76E-05	0.00083462
ENSG000001	1735.67365	-0.1757962	0.09907524	-1.7743703	0.07600192	0.18747406
ENSG000001	744.089124	-0.2497138	0.0853967	-2.9241621	0.00345385	0.02315385
ENSG000001	1639.314	-0.2248526	0.09254643	-2.429619	0.0151147	0.06332032
ENSG000001	398.888442	-0.2424131	0.12800111	-1.893836	0.05824678	0.15713598
ENSG000001	1013.45267	-0.1101112	0.05985473	-1.8396401	0.06582109	0.17050053
ENSG000001	3542.43196	0.21285043	0.10791271	1.9724315	0.04856037	0.13867021
ENSG000001	8790.3363	0.482079	0.14381127	3.35216433	0.00080182	0.00836083
ENSG000001	488.877305	0.17905104	0.08874308	2.01763384	0.04362941	0.12857502
ENSG000001	1699.54798	0.21735887	0.05935249	3.66216958	0.00025009	0.00351689

ENSG000001	75.4628592	0.52676414	0.19195654	2.74418443	0.00606614	0.03394764
ENSG000001	1295.53049	-0.5837153	0.19836572	-2.9426217	0.00325446	0.02227185
ENSG000001	51.8678722	1.26102863	0.28000098	4.50365783	6.68E-06	0.00020282
ENSG000001	3494.85756	0.50618069	0.15339696	3.29980921	0.00096751	0.00951091
ENSG000001	2350.22122	0.47142748	0.15707115	3.00136264	0.00268774	0.01943138
ENSG000001	217.889914	0.89701895	0.27285744	3.28750042	0.00101081	0.00982469
ENSG000001	108.400911	-0.5277004	0.16736939	-3.1529086	0.00161652	0.01370941
ENSG000001	1126.86823	-0.1538977	0.08076667	-1.9054601	0.05672028	0.15392958
ENSG000001	5251.91819	0.20803117	0.08275717	2.51375406	0.01194537	0.05421189
ENSG000001	1033.54065	-0.238191	0.1102475	-2.1605117	0.03073308	0.10213422
ENSG000001	649.362254	-0.2987378	0.1673367	-1.7852495	0.07422086	0.18481389
ENSG000001	631.437482	0.27652971	0.1052455	2.62747301	0.00860217	0.04330664
ENSG000001	4052.86485	0.24195791	0.1085337	2.22933447	0.02579166	0.090874
ENSG000001	73.0117772	0.4714756	0.24356106	1.93575934	0.05289718	0.14708135
ENSG000001	493.799764	0.49833427	0.15989438	3.1166466	0.00182921	0.01491472
ENSG000001	147.247964	0.2964527	0.10494991	2.8247066	0.00473239	0.02887869
ENSG000001	1969.80385	0.4103024	0.115244	3.56029281	0.00037044	0.00470863
ENSG000001	1069.69024	0.21148353	0.06360026	3.32519884	0.00088355	0.00892646
ENSG000001	2276.9335	-0.2413036	0.08755217	-2.7561127	0.00584928	0.03309548
ENSG000001	892.362536	0.32246699	0.11294014	2.85520254	0.00430094	0.0268862
ENSG000001	1685.17531	0.1906332	0.07117841	2.67824462	0.00740091	0.0390354
ENSG000001	235.277815	-0.7127884	0.21729364	-3.2803006	0.00103697	0.00998422
ENSG000001	3267.78078	-0.1583707	0.08518847	-1.8590628	0.06301824	0.1655415
ENSG000001	7188.12276	0.24931885	0.07851575	3.17539936	0.0014963	0.01297456
ENSG000001	3759.02007	0.64968873	0.18859848	3.44482475	0.00057143	0.00648149
ENSG000001	60796.446	1.02603414	0.29123736	3.52301696	0.00042666	0.00525621
ENSG000001	1171.41548	0.36016715	0.1217187	2.95901236	0.00308627	0.02142486
ENSG000001	3866.8193	-0.2302035	0.10929218	-2.1063123	0.03517723	0.11169657
ENSG000001	1536.03247	0.22454614	0.10787655	2.0815102	0.03738723	0.11601787
ENSG000001	2146.5689	-0.2167311	0.09844073	-2.2016407	0.0276907	0.09528739
ENSG000001	299.626871	0.97239179	0.20534319	4.7354469	2.19E-06	7.85E-05
ENSG000001	2220.93375	-0.3007949	0.09470315	-3.1761872	0.00149225	0.01295235
ENSG000001	2283.87634	-0.3166121	0.07790392	-4.0641349	4.82E-05	0.00100574
ENSG000001	186.748992	1.19267825	0.27175941	4.38872843	1.14E-05	0.00031368
ENSG000001	3214.61383	0.74545186	0.23722898	3.14233051	0.00167609	0.01403022
ENSG000001	307.42913	-0.2556353	0.13651481	-1.8725829	0.06112601	0.16228678
ENSG000001	828.305755	0.45407014	0.20706969	2.19283727	0.02831911	0.09653812
ENSG000001	764.484972	0.21125685	0.06738665	3.1349957	0.00171857	0.01428248
ENSG000001	4189.63647	0.22851399	0.10938597	2.0890612	0.03670221	0.1145861
ENSG000001	1435.16516	-0.0831058	0.04695716	-1.769822	0.0767568	0.1885515
ENSG000001	97.410929	0.28592075	0.14636671	1.95345475	0.05076573	0.14307998
ENSG000001	1400.74379	0.36767948	0.20460193	1.797048	0.072328	0.18167313
ENSG000001	9496.28866	-0.1468486	0.05960361	-2.463753	0.01374908	0.05946813

ENSG000001	2743.28445	0.7874206	0.16146509	4.87672351	1.08E-06	4.45E-05
ENSG000001	3528.44695	-0.1098831	0.06260838	-1.7550855	0.07924468	0.19267128
ENSG000001	498.181201	-0.6542498	0.29644923	-2.2069538	0.02731728	0.09430372
ENSG000001	3004.8889	-0.1588044	0.0722569	-2.1977746	0.02796517	0.09578963
ENSG000001	1338.06019	-0.1516865	0.04836761	-3.1361167	0.00171201	0.0142596
ENSG000001	3293.69755	0.24580293	0.12307004	1.99726053	0.04579689	0.1330024
ENSG000001	1251.55285	-0.1609305	0.07183692	-2.2402201	0.02507664	0.08915888
ENSG000001	4769.14378	0.13879559	0.07534352	1.84217029	0.06545025	0.16991572
ENSG000001	1853.68016	0.15982674	0.0693828	2.30354993	0.02124792	0.07992709
ENSG000001	4469.83279	-0.1247817	0.04446372	-2.8063713	0.00501029	0.03000502
ENSG000001	8146.96065	0.15264972	0.07965704	1.91633698	0.05532222	0.15143879
ENSG000001	1085	-0.3057489	0.09954043	-3.0716053	0.00212911	0.01648494
ENSG000001	7826.54039	0.32892681	0.10124514	3.24881579	0.00115886	0.01077206
ENSG000001	10848.2422	-0.1546179	0.06369948	-2.4273026	0.01521156	0.0636448
ENSG000001	739.227903	-0.1582731	0.08487582	-1.8647605	0.06221499	0.16432654
ENSG000001	159.361938	-0.2535138	0.13864368	-1.8285278	0.06747038	0.17348469
ENSG000001	1060.01305	0.30677398	0.09778738	3.13715318	0.00170597	0.01422827
ENSG000001	2412.66064	0.27211138	0.11250677	2.41862241	0.0155794	0.06481996
ENSG000001	2325.21811	-0.1494384	0.08171629	-1.8287463	0.06743762	0.17345485
ENSG000001	7488.05915	1.08704899	0.25740697	4.22307518	2.41E-05	0.00057519
ENSG000001	1046.74996	-0.5091738	0.1596147	-3.190018	0.00142264	0.01250108
ENSG000001	1456.75299	0.09310825	0.05091582	1.82867014	0.06744904	0.17345701
ENSG000001	105.159034	0.42339613	0.2384405	1.77568883	0.07578422	0.18716398
ENSG000001	1130.98882	0.152319	0.066352	2.29562037	0.0216976	0.08122728
ENSG000001	553.036642	3.39498224	0.54579837	6.22021321	4.96E-10	6.41E-08
ENSG000001	62.2728748	1.19057823	0.45506934	2.61625673	0.00888997	0.04419916
ENSG000001	713.671932	-0.1726354	0.08333431	-2.0716006	0.03830271	0.11783292
ENSG000001	1544.78412	0.19929595	0.0928236	2.14703967	0.03179012	0.1044196
ENSG000001	663.695716	0.4466085	0.17355409	2.57331019	0.01007309	0.04807811
ENSG000001	2377.556	0.22656777	0.11745459	1.92898178	0.05373312	0.14847692
ENSG000001	6331.00853	-0.2611271	0.07996371	-3.2655698	0.00109244	0.01036617
ENSG000001	3633.70759	0.6517934	0.13620905	4.78524302	1.71E-06	6.43E-05
ENSG000001	1381.00858	0.41010909	0.12045809	3.40457905	0.00066266	0.00729102
ENSG000001	2077.07607	0.73556654	0.22240145	3.30738195	0.00094172	0.00932532
ENSG000001	856.397747	0.20343129	0.11506272	1.7680035	0.07706032	0.18903847
ENSG000001	286.163476	0.27673117	0.1167752	2.36977681	0.01779883	0.07071951
ENSG000001	56.3779482	1.15062372	0.26090402	4.4101417	1.03E-05	0.00028905
ENSG000001	6795.74164	0.1774229	0.08360827	2.12207348	0.03383157	0.10898148
ENSG000001	3200.43323	-0.1903199	0.0797977	-2.38503	0.01707773	0.06896969
ENSG000001	2240.261	0.21236507	0.10950465	1.93932464	0.05246182	0.14614981
ENSG000001	12201.8317	0.84159658	0.16180739	5.20122455	1.98E-07	1.10E-05
ENSG000001	4332.34639	0.15440861	0.05666649	2.72486622	0.00643275	0.03538339
ENSG000001	1550.49146	-0.1872275	0.07744276	-2.4176245	0.01562219	0.06489918

ENSG000001	1172.1444	0.553699	0.23241435	2.38237871	0.0172012	0.06926523
ENSG000001	17.3354646	1.70736943	0.46876246	3.64229131	0.00027022	0.00370781
ENSG000001	56.6393076	0.89730982	0.22429547	4.00057038	6.32E-05	0.00124534
ENSG000001	1938.43599	-0.1937097	0.06862691	-2.8226496	0.00476286	0.02902141
ENSG000001	366.564751	0.37379877	0.192827	1.93851888	0.05255995	0.14634496
ENSG000001	3154.96147	-0.2032557	0.07572338	-2.6841864	0.00727066	0.03856906
ENSG000001	1520.11422	-0.2537345	0.09442099	-2.6872677	0.00720392	0.03838891
ENSG000001	679.419897	-0.1703657	0.05951682	-2.8624799	0.0042034	0.02653952
ENSG000001	1894.50739	0.23200625	0.07361947	3.15142538	0.00162476	0.01374184
ENSG000001	1281.74273	-0.270593	0.06540312	-4.1373109	3.51E-05	0.0007893
ENSG000001	7090.37244	-0.129363	0.05957931	-2.1712746	0.02991042	0.10029471
ENSG000001	5369.23748	-0.1417873	0.047639	-2.9762857	0.00291763	0.02056766
ENSG000001	306.876973	0.34207475	0.08199	4.17215198	3.02E-05	0.00070278
ENSG000001	46318.3596	-0.1107762	0.04589733	-2.4135654	0.01579729	0.06546122
ENSG000001	1453.18507	0.19041706	0.04839105	3.93496427	8.32E-05	0.00155751
ENSG000001	1347.88353	0.19242849	0.09921703	1.93947049	0.05244407	0.14614641
ENSG000001	346.050983	-0.3196898	0.13309859	-2.4019023	0.01631006	0.06690921
ENSG000001	8479.24259	-0.1706534	0.06229883	-2.7392713	0.00615755	0.03429531
ENSG000001	1918.23002	-0.1937302	0.08216615	-2.3577856	0.01838431	0.07222029
ENSG000001	1453.69496	-0.2433477	0.08488361	-2.8668395	0.00414593	0.02626771
ENSG000001	701.206414	0.43397783	0.19083156	2.27414073	0.02295753	0.08395062
ENSG000001	29399.3483	-0.2002664	0.10351063	-1.9347426	0.05302189	0.14720621
ENSG000001	3723.2866	-0.1850484	0.05588523	-3.3112226	0.00092889	0.00924215
ENSG000001	1744.22068	0.15697584	0.08088706	1.94067934	0.05229718	0.14596033
ENSG000001	245023.864	0.7400317	0.41572815	1.78008564	0.07506195	0.18603246
ENSG000001	6758.96687	-0.2493323	0.10886273	-2.290337	0.02200179	0.0819172
ENSG000001	158.592171	0.1630618	0.0840356	1.94038961	0.05233236	0.14597773
ENSG000001	2580.72841	0.17811205	0.08194761	2.17348666	0.02974371	0.09989355
ENSG000001	7942.16321	0.11906048	0.04791055	2.48505784	0.01295304	0.05731003
ENSG000001	242.13449	0.4774173	0.2314988	2.06228839	0.03918029	0.11965714
ENSG000001	443.519843	-0.1979577	0.071043	-2.7864483	0.00532891	0.03125113
ENSG000001	363.210381	0.19538408	0.1018302	1.91872439	0.05501922	0.15099555
ENSG000001	96.9923968	0.28525428	0.13250669	2.15275376	0.03133804	0.10347357
ENSG000001	6219.87423	0.42311006	0.14088066	3.00332264	0.00267049	0.01933247
ENSG000001	104.701474	0.67852144	0.22266093	3.04733043	0.00230884	0.01745708
ENSG000001	3782.72227	0.70788528	0.20370187	3.47510443	0.00051065	0.0059937
ENSG000001	4629.60419	-0.1408124	0.07824922	-1.7995377	0.07193367	0.18106478
ENSG000001	14921.2007	0.55878023	0.21485746	2.60070198	0.00930332	0.04560436
ENSG000001	1275.0155	-0.1242295	0.06903621	-1.7994834	0.07194225	0.18106478
ENSG000001	1796.95804	0.14671757	0.08386411	1.74946783	0.08021019	0.19429848
ENSG000001	265.671348	-0.3664005	0.14111466	-2.5964736	0.00941861	0.04599077
ENSG000001	1602.38983	0.25642592	0.1122382	2.28465815	0.02233288	0.08245716
ENSG000001	3973.01548	0.31785262	0.13140395	2.41889694	0.01556765	0.06480041

ENSG000001	1188.80052	2.38529978	0.40612185	5.87335982	4.27E-09	4.22E-07
ENSG000001	1028.03071	0.16890097	0.07593583	2.22425918	0.02613101	0.09151435
ENSG000001	396.425437	0.20523583	0.09299585	2.20693538	0.02731857	0.09430372
ENSG000001	694.322467	0.60783688	0.15248798	3.98612971	6.72E-05	0.00130014
ENSG000001	6848.68982	0.34165053	0.15411586	2.21684215	0.02663388	0.09278006
ENSG000001	310.707212	0.28207406	0.16261771	1.73458394	0.08281453	0.1988446
ENSG000001	1349.41443	-0.1726844	0.09472285	-1.8230491	0.06829596	0.17484512
ENSG000001	2024.45989	1.56770539	0.24936788	6.28671746	3.24E-10	4.49E-08
ENSG000001	3019.75291	0.29433438	0.09886671	2.97708277	0.00291005	0.02054411
ENSG000001	8379.22547	0.34217019	0.14534276	2.35422931	0.01856116	0.07270559
ENSG000001	246.937874	-0.6802723	0.19668247	-3.4587339	0.00054272	0.00625769
ENSG000001	839.084132	0.20444535	0.08723205	2.34369532	0.01909376	0.07410187
ENSG000001	47.0042619	0.95088798	0.33082295	2.87431084	0.0040491	0.02583592
ENSG000001	4139.86663	0.15273936	0.06884513	2.2185936	0.02651439	0.09246201
ENSG000001	735.729436	2.09721306	0.32779854	6.39787179	1.58E-10	2.53E-08
ENSG000001	687.988011	0.24070425	0.12291974	1.95822287	0.05020387	0.14200324
ENSG000001	515.106918	-0.160439	0.08522984	-1.8824271	0.05977804	0.15992504
ENSG000001	12481.361	-0.099081	0.05278869	-1.8769359	0.06052688	0.16123049
ENSG000001	55.055708	-0.9197925	0.38340468	-2.3990122	0.01643937	0.06724562
ENSG000001	298.357908	-0.3013035	0.14526405	-2.0741777	0.03806281	0.11731502
ENSG000001	10061.724	0.23569464	0.12328461	1.91179287	0.05590277	0.15234793
ENSG000001	337.8835	0.31821136	0.13832936	2.30038918	0.02142618	0.08048685
ENSG000001	1471.97764	-0.3481924	0.12628185	-2.7572644	0.00582872	0.03301331
ENSG000001	56075.7972	-0.1350985	0.07192948	-1.8782074	0.0603528	0.16101608
ENSG000001	5733.55183	0.31631896	0.09924999	3.18709319	0.0014371	0.01260118
ENSG000001	1170.37838	-0.1689083	0.09473259	-1.7830012	0.0745861	0.18532935
ENSG000001	1301.17186	0.20215027	0.07302785	2.76812589	0.00563797	0.03233499
ENSG000001	244.505346	2.0907683	0.42808878	4.88395961	1.04E-06	4.33E-05
ENSG000001	1746.65034	-0.2139352	0.08945287	-2.391597	0.01677525	0.06816093
ENSG000001	247.834381	-0.5933738	0.23481703	-2.5269625	0.01150538	0.05282937
ENSG000001	816.994502	0.7080244	0.20402281	3.4703198	0.00051984	0.00607108
ENSG000001	196.214137	0.8112735	0.1611424	5.03451294	4.79E-07	2.32E-05
ENSG000001	110.114389	-0.360811	0.16468552	-2.1909091	0.02845837	0.09688921
ENSG000001	623.353029	0.33141743	0.14357627	2.30830222	0.02098233	0.07931719
ENSG000001	548.811242	0.37929784	0.08627235	4.39651658	1.10E-05	0.00030571
ENSG000001	1860.79006	-0.0765558	0.03956482	-1.9349455	0.05299699	0.14720196
ENSG000001	1387.70327	-0.1411307	0.06223709	-2.2676307	0.02335172	0.08506958
ENSG000001	785.845351	0.0966129	0.0515459	1.87430824	0.06088795	0.16191081
ENSG000001	7165.11137	0.2182441	0.12038722	1.81285112	0.06985479	0.17731958
ENSG000001	370.808266	0.20288219	0.1059233	1.91536884	0.05544548	0.15157379
ENSG000001	1601.68724	0.22322805	0.06354051	3.51316099	0.00044281	0.00536244
ENSG000001	1266.27028	-0.4729948	0.15379213	-3.0755459	0.00210118	0.01632843
ENSG000001	2403.99232	0.63263098	0.21250388	2.97703265	0.00291053	0.02054411

ENSG000001	1103.65976	-0.2028642	0.04101604	-4.9459732	7.58E-07	3.31E-05
ENSG000001	1212.6807	-0.1034544	0.05863164	-1.7644813	0.07765099	0.19009304
ENSG000001	1433.6566	0.21085953	0.10210357	2.06515333	0.03890849	0.11905928
ENSG000001	3428.03468	0.21944252	0.08835891	2.48353575	0.01300853	0.05742055
ENSG000001	9169.15837	-0.0995517	0.04456279	-2.2339658	0.02548532	0.09006095
ENSG000001	132.961358	-0.2096905	0.11508135	-1.8221064	0.06843884	0.17512355
ENSG000001	6712.13041	0.79738449	0.23165773	3.44208028	0.00057726	0.00653682
ENSG000001	2652.31731	-0.2285602	0.0535459	-4.2684919	1.97E-05	0.00048452
ENSG000001	816.619139	-0.1580879	0.05834594	-2.7094932	0.00673861	0.03656778
ENSG000001	328.56209	0.28758883	0.14715047	1.95438603	0.05065558	0.14293574
ENSG000001	395.151546	0.64296113	0.15741416	4.08451894	4.42E-05	0.00094055
ENSG000001	46376.4106	0.50818554	0.24806099	2.04863148	0.04049816	0.12224749
ENSG000001	5797.14254	-0.3048886	0.06276498	-4.8576227	1.19E-06	4.85E-05
ENSG000001	1346.31468	-0.2178207	0.07538648	-2.8893867	0.00385994	0.02496704
ENSG000001	4286.27399	0.35212837	0.12046407	2.92309871	0.00346567	0.02322294
ENSG000001	65975.6368	0.2829325	0.1019336	2.77565486	0.00550907	0.03188569
ENSG000001	14121.8766	-0.2703802	0.10082698	-2.6816253	0.00732655	0.03877809
ENSG000001	327.927262	0.36083386	0.20002498	1.80394397	0.07124009	0.17971135
ENSG000001	1184.19489	0.17340931	0.09481764	1.8288719	0.0674188	0.17343365
ENSG000001	1298.57737	0.21788993	0.06303792	3.45648962	0.00054726	0.00628391
ENSG000001	184.607086	-0.3269749	0.14431746	-2.2656646	0.02347193	0.08548849
ENSG000001	1745.52623	-0.4102916	0.11899664	-3.4479261	0.00056491	0.00642358
ENSG000001	4120.55009	0.10779632	0.05739003	1.87831091	0.06033864	0.16100452
ENSG000001	232.609877	-0.2613241	0.08631134	-3.0276914	0.0024643	0.01824246
ENSG000001	418.739618	0.39985084	0.14764807	2.70813461	0.00676626	0.03666791
ENSG000001	1399.38812	0.23677871	0.12209876	1.93923923	0.05247222	0.14615032
ENSG000001	550.102634	-0.4107697	0.17343332	-2.3684586	0.01786238	0.07083178
ENSG000001	1989.93221	0.23670742	0.12949496	1.82792771	0.0675604	0.17363447
ENSG000001	367.499782	-0.6414516	0.22344289	-2.8707632	0.00409482	0.02607487
ENSG000001	2197.00089	0.1888198	0.09821531	1.92250875	0.05454177	0.15005394
ENSG000001	3333.45263	-0.1894462	0.07304435	-2.593578	0.0094983	0.04622542
ENSG000001	1058.93151	-0.1704036	0.07086426	-2.4046483	0.01618804	0.06656619
ENSG000001	475.690539	-0.2785005	0.10420238	-2.6726881	0.00752462	0.03936858
ENSG000001	1929.07724	0.12253931	0.05501038	2.22756712	0.02590939	0.09110795
ENSG000001	1151.55228	0.16750345	0.09665066	1.73308126	0.08308123	0.19930986
ENSG000001	1226.19099	0.86140372	0.21319783	4.04039621	5.34E-05	0.00108961
ENSG000001	1853.24325	-0.1520256	0.07626719	-1.9933288	0.04622545	0.1339471
ENSG000001	5318.3442	0.40768008	0.15250175	2.67328134	0.00751132	0.03936345
ENSG000001	2037.50973	-0.0975851	0.04061466	-2.402705	0.01627431	0.06677924
ENSG000001	1024.15122	-0.3153029	0.13334313	-2.3645979	0.01804965	0.07138451
ENSG000001	2486.88187	-0.473378	0.15302287	-3.0935114	0.00197803	0.01566848
ENSG000001	1822.31986	0.10122109	0.05594096	1.809427	0.07038469	0.1782418
ENSG000001	866.480783	-0.219426	0.09105172	-2.4099054	0.01595666	0.06594928

ENSG000001	437.14294	0.27215487	0.10269742	2.65006533	0.00804762	0.04123243
ENSG000001	418.549284	-0.2724585	0.10376455	-2.6257376	0.00864614	0.04337763
ENSG000001	165.30366	0.46637317	0.1653552	2.82043235	0.0047959	0.02912783
ENSG000001	90750.4555	0.46424023	0.24270825	1.91275008	0.05578005	0.15215863
ENSG000001	799.595457	0.45229915	0.12666296	3.57088723	0.00035577	0.00455752
ENSG000001	800.255878	-0.2796868	0.10046969	-2.7837932	0.00537273	0.0314294
ENSG000001	1627.24127	-0.1487463	0.08270066	-1.798611	0.07208023	0.18118956
ENSG000001	27737.5999	0.18927321	0.10174123	1.86033932	0.06283753	0.16519914
ENSG000001	169.411629	-0.3916537	0.14924093	-2.624305	0.0086826	0.04344479
ENSG000001	811.382978	0.59388587	0.27662719	2.14688178	0.03180269	0.10443997
ENSG000001	4003.89487	-0.240986	0.05773888	-4.1737219	3.00E-05	0.00069894
ENSG000001	329.855467	-0.3278531	0.14013327	-2.3395811	0.01930538	0.07462986
ENSG000001	7566.38721	-0.18324	0.07538429	-2.4307452	0.01506781	0.06320968
ENSG000001	164.480969	-0.2212338	0.12064035	-1.833829	0.06667938	0.1720985
ENSG000001	1552.70449	0.54247146	0.09983628	5.43361058	5.52E-08	3.82E-06
ENSG000001	1231.50083	-0.5350518	0.18888352	-2.8327077	0.00461556	0.0282815
ENSG000001	4099.46776	0.32492695	0.13151037	2.47073259	0.01348366	0.05876494
ENSG000001	3471.73195	0.65727616	0.16943506	3.87922172	0.00010479	0.00187174
ENSG000001	359.008342	0.38656173	0.17149945	2.25401144	0.02419545	0.08723236
ENSG000001	7755.09411	0.29337434	0.07238078	4.05320772	5.05E-05	0.00104068
ENSG000001	7683.36679	-0.2233388	0.07300002	-3.0594347	0.00221755	0.01695951
ENSG000001	13864.6689	0.20273128	0.07603348	2.66634237	0.00766816	0.03993115
ENSG000001	300.464472	0.31893022	0.14224271	2.24215504	0.02495135	0.08886346
ENSG000001	428.271821	0.38470006	0.15603883	2.4654124	0.01368556	0.05933426
ENSG000001	174.532148	-0.3510806	0.12573547	-2.7922159	0.00523484	0.03093178
ENSG000001	528.76462	-0.2287643	0.1018629	-2.2458059	0.02471645	0.08831247
ENSG000001	410.703852	-0.2852751	0.1241996	-2.2969085	0.02162399	0.08102572
ENSG000001	3924.96315	0.10567155	0.0396535	2.66487311	0.00770174	0.04003976
ENSG000001	82.4197795	3.10118066	0.51269173	6.04882136	1.46E-09	1.71E-07
ENSG000001	1886.56762	-0.2886308	0.11211253	-2.5744741	0.01003926	0.04797335
ENSG000001	2908.31356	-0.2415583	0.13475616	-1.7925583	0.07304357	0.18289256
ENSG000001	9355.7937	-0.3685565	0.14598165	-2.524677	0.01158047	0.05302567
ENSG000001	22402.2964	1.06600207	0.1518684	7.01924875	2.23E-12	6.77E-10
ENSG000001	1013.90814	-0.3311251	0.12588106	-2.6304603	0.00852693	0.04307884
ENSG000001	50415.6506	0.11715505	0.06663956	1.75804049	0.07874062	0.19190449
ENSG000001	1057.37416	0.18972251	0.09500318	1.99701228	0.04582385	0.13305714
ENSG000001	47.1648877	1.55047193	0.37696054	4.1130881	3.90E-05	0.00085238
ENSG000001	427.633596	1.85344651	0.30601607	6.0566966	1.39E-09	1.64E-07
ENSG000001	799.004899	-0.2148821	0.07888897	-2.7238546	0.00645249	0.03543252
ENSG000001	9209.00238	2.03884175	0.25297924	8.0593243	7.67E-16	1.80E-12
ENSG000001	800.291938	0.23295798	0.1188587	1.95995731	0.05000078	0.14152646
ENSG000001	7473.65621	-0.2102646	0.06566016	-3.2023163	0.00136327	0.01212827
ENSG000001	289.575029	-0.4485676	0.23523068	-1.9069265	0.0565301	0.15354051

ENSG000001	4724.58827	0.7564995	0.2066656	3.66050041	0.00025172	0.00353382
ENSG000001	130.398647	-0.423511	0.19979888	-2.1196865	0.0340325	0.10933206
ENSG000001	28.5442488	0.64421922	0.29307553	2.19813374	0.02793958	0.09573725
ENSG000001	890.233157	-0.1273826	0.07036695	-1.810262	0.07025517	0.17799352
ENSG000001	5116.84848	0.28314581	0.06049864	4.6802015	2.87E-06	9.81E-05
ENSG000001	1205.55797	0.11680501	0.05864756	1.99164318	0.04641022	0.13433158
ENSG000001	3606.04059	0.55283423	0.14775018	3.74168215	0.00018279	0.00283026
ENSG000001	1832.19852	0.34560361	0.12456894	2.77439631	0.00553043	0.03190795
ENSG000001	3514.73565	-0.4167694	0.19775895	-2.1074616	0.03507759	0.1114665
ENSG000001	2340.34775	0.32296007	0.11325174	2.85170068	0.0043486	0.0270912
ENSG000001	120.236723	1.1192971	0.25829245	4.33344874	1.47E-05	0.00038205
ENSG000001	3101.57272	-0.22031	0.09356113	-2.3547172	0.01853681	0.07264532
ENSG000001	431.789142	0.39412587	0.18466789	2.13424146	0.03282302	0.10668556
ENSG000001	38982.1853	-0.1631024	0.03760863	-4.336835	1.45E-05	0.00037682
ENSG000001	12893.0103	-0.1245889	0.05320266	-2.3417797	0.01919204	0.07436008
ENSG000001	7969.85041	-0.2442201	0.12221838	-1.998227	0.04569205	0.13276849
ENSG000001	8201.34197	0.37961199	0.16576707	2.29003254	0.02201943	0.0819291
ENSG000001	221.696673	0.31912912	0.09992261	3.19376287	0.00140431	0.01238652
ENSG000001	388.006819	0.45218503	0.12521467	3.61127823	0.00030469	0.00402581
ENSG000001	392.894343	-0.2040788	0.08581262	-2.3781913	0.0173978	0.06978439
ENSG000001	3118.17476	-0.1747247	0.0610497	-2.8620075	0.00420967	0.02655784
ENSG000001	4165.13991	0.54876828	0.26899359	2.04007939	0.04134242	0.12388371
ENSG000001	1186.80805	0.11809221	0.05240253	2.25355934	0.0242239	0.0872582
ENSG000001	175.726881	-0.1472134	0.08146187	-1.8071455	0.0707396	0.17888915
ENSG000001	805.299468	-0.533317	0.18861604	-2.8275274	0.0046909	0.02867889
ENSG000001	33519.002	0.33494689	0.1487911	2.25112177	0.02437783	0.0875576
ENSG000001	597.611288	-0.2993884	0.13294303	-2.2520055	0.02432192	0.08741923
ENSG000001	303.826363	-0.239994	0.10839317	-2.2141067	0.02682144	0.09315503
ENSG000001	1906.83035	-0.2856596	0.10366352	-2.7556427	0.00585769	0.03313102
ENSG000001	679.625341	0.26159692	0.12109999	2.16017293	0.03075928	0.10218121
ENSG000001	752.138453	-0.3951239	0.18823616	-2.0990858	0.03580934	0.11289478
ENSG000001	142.611721	-0.2789618	0.14789148	-1.8862601	0.05925991	0.15916212
ENSG000001	2045.42004	0.28335876	0.14929736	1.89794893	0.05770281	0.15589932
ENSG000001	7637.40541	-0.1168837	0.04393313	-2.6604909	0.00780268	0.040356
ENSG000001	2539.25609	0.12266602	0.06926519	1.77096189	0.07656704	0.18836798
ENSG000001	3648.01002	-0.2555613	0.0725118	-3.5244102	0.00042443	0.00523469
ENSG000001	3425.40832	-0.3401121	0.12658576	-2.6868117	0.00721376	0.03841638
ENSG000001	2062.01449	0.24606756	0.11988162	2.05258797	0.04011256	0.12162085
ENSG000001	143.956554	-0.8250931	0.22438586	-3.677117	0.00023588	0.00336331
ENSG000001	6296.65034	0.09373084	0.0390361	2.40113239	0.01634442	0.06698824
ENSG000001	367.044175	-0.2575867	0.10160833	-2.5350939	0.01124172	0.05192407
ENSG000001	1876.23229	0.14184228	0.05615578	2.52587141	0.01154117	0.05290483
ENSG000001	30022.7214	0.88693902	0.1645597	5.38977041	7.05E-08	4.76E-06

ENSG000001	5071.65067	-0.1822777	0.06813623	-2.6751953	0.00746857	0.03923812
ENSG000001	185.677702	-0.1833248	0.10523517	-1.7420485	0.08149996	0.19655168
ENSG000001	2496.18686	0.11484052	0.06496363	1.76776625	0.07709999	0.18908294
ENSG000001	280.29146	-0.397337	0.13313147	-2.984546	0.00284	0.02020823
ENSG000001	676.487983	0.16913283	0.07409561	2.2826295	0.0224522	0.08269289
ENSG000001	723.587378	0.59124151	0.24693888	2.39428277	0.0166529	0.06782357
ENSG000001	146.346183	0.25191878	0.10496047	2.40013	0.01638925	0.06711652
ENSG000001	2827.35213	0.21706859	0.11482233	1.8904737	0.05869464	0.15813604
ENSG000001	223.529076	0.18998165	0.07535654	2.52110374	0.01169874	0.0533883
ENSG000001	427.484605	0.32762857	0.13765772	2.38002333	0.01731154	0.06956468
ENSG000001	138.115706	-0.5526337	0.28358682	-1.9487284	0.05132786	0.14403953
ENSG000001	725.113931	-0.1966691	0.09025098	-2.1791356	0.0293216	0.09896794
ENSG000001	2806.25063	0.1899288	0.08952546	2.12150595	0.03387925	0.1090106
ENSG000001	177.440758	-0.5128802	0.2388046	-2.147698	0.03173775	0.10435209
ENSG000001	2866.93029	0.5558209	0.1223971	4.54112818	5.60E-06	0.00017543
ENSG000001	1100.06058	0.45111321	0.14612533	3.0871664	0.00202074	0.01590479
ENSG000001	11901.4646	-0.3141324	0.12076949	-2.6010906	0.00929279	0.04558852
ENSG000001	1394.16047	0.20462176	0.09400238	2.17677209	0.02949758	0.09930381
ENSG000001	856.579542	-0.1692451	0.06789234	-2.4928446	0.01267243	0.05644929
ENSG000001	1687.74411	0.08924172	0.04992675	1.78745295	0.07386431	0.18424809
ENSG000001	615.540961	-0.2729593	0.12090471	-2.2576397	0.02396813	0.08673701
ENSG000001	622.341309	1.02771539	0.23687094	4.33871453	1.43E-05	0.0003748
ENSG000001	7942.63617	0.28353338	0.1229441	2.30619759	0.02109959	0.07961754
ENSG000001	3795.41446	0.22374398	0.1262072	1.7728306	0.07625678	0.18780151
ENSG000001	3001.32981	0.41563865	0.16253452	2.5572331	0.01055085	0.04962773
ENSG000001	725.365852	0.16491686	0.06867902	2.40126971	0.01633829	0.06698824
ENSG000001	4037.89655	-0.1627168	0.06857643	-2.3727803	0.01765476	0.0704172
ENSG000001	3595.4336	0.12554689	0.05521233	2.27389246	0.02297245	0.08398647
ENSG000001	1412.71693	-0.1425771	0.06932616	-2.0566127	0.03972349	0.12077621
ENSG000001	1560.91421	0.20470204	0.04922082	4.15885052	3.20E-05	0.00073454
ENSG000001	1860.55742	0.36824725	0.12469417	2.95320351	0.00314495	0.0217269
ENSG000001	4235.4251	1.06389735	0.25135491	4.23265003	2.31E-05	0.00055536
ENSG000001	11855.4676	0.39429875	0.09252501	4.26153691	2.03E-05	0.0004965
ENSG000001	288.454531	0.26150651	0.12212698	2.14126731	0.03225249	0.10549452
ENSG000001	1792.08607	0.88367866	0.14998679	5.89170984	3.82E-09	3.92E-07
ENSG000001	865.211991	0.25862455	0.11001993	2.35070636	0.01873781	0.07318817
ENSG000001	5737.32518	0.16365707	0.06092909	2.68602516	0.00723076	0.03849443
ENSG000001	25732.1043	0.52069144	0.12954599	4.01935587	5.84E-05	0.00117487
ENSG000001	2136.42115	0.57118608	0.16269224	3.51083799	0.0004467	0.00540154
ENSG000001	3368.05729	0.29889274	0.13687414	2.18370496	0.02898394	0.09808743
ENSG000001	1635.20335	0.11433397	0.04502258	2.53948057	0.01110172	0.05140777
ENSG000001	1438.21476	0.27241007	0.10081503	2.70207796	0.00689076	0.03719151
ENSG000001	1808.90066	-0.1157728	0.05429027	-2.1324779	0.03296758	0.10708586

ENSG000001	1888.50874	0.12649481	0.06349726	1.99213031	0.04635676	0.13427017
ENSG000001	1426.882	0.25257935	0.1287209	1.96222485	0.04973631	0.14099694
ENSG000001	1022.24559	-0.1284864	0.05327956	-2.4115507	0.01588484	0.06574189
ENSG000001	39981.031	0.52018948	0.16416888	3.16862422	0.00153162	0.01323183
ENSG000001	992.179555	0.35729474	0.16311948	2.19038667	0.02849621	0.09698055
ENSG000001	1340.27176	0.81220928	0.24264216	3.34735426	0.00081587	0.00846342
ENSG000001	2266.20184	0.14440711	0.07521896	1.91982324	0.05488023	0.15083325
ENSG000001	295.973762	0.29206091	0.13081533	2.23261986	0.02557402	0.09030897
ENSG000001	3647.69635	-0.1576589	0.08917185	-1.7680343	0.07705518	0.18903847
ENSG000001	1616.22316	-0.2057931	0.07686699	-2.6772626	0.00742264	0.03907127
ENSG000001	139.805449	0.60680043	0.32271249	1.88031284	0.06006545	0.16051063
ENSG000001	542.786179	0.44303101	0.0968131	4.57614726	4.74E-06	0.00015317
ENSG000001	26.1752369	1.22074764	0.29240894	4.17479596	2.98E-05	0.00069665
ENSG000001	1067.11522	-0.3320665	0.11002223	-3.0181761	0.00254301	0.01864003
ENSG000001	35767.6295	0.22448768	0.12055102	1.86217981	0.06257775	0.16475391
ENSG000001	4548.50433	-0.2930107	0.13330356	-2.1980711	0.02794404	0.09573725
ENSG000001	2201.53254	1.00850925	0.30615474	3.2941161	0.00098732	0.0096651
ENSG000001	456.187007	-0.2706122	0.13025826	-2.0775052	0.03775495	0.11676122
ENSG000001	812.641716	0.37808073	0.15429604	2.4503592	0.01427138	0.06090386
ENSG000001	474.513293	0.65125712	0.22003785	2.95975036	0.00307888	0.02138706
ENSG000001	6076.43212	-0.1200151	0.06322698	-1.8981636	0.05767454	0.15584862
ENSG000001	429.716741	0.1839418	0.07390189	2.48899999	0.0128103	0.0568934
ENSG000001	22.5153876	1.13172296	0.29889454	3.78636206	0.00015287	0.0024966
ENSG000001	5518.00358	1.26342733	0.19455199	6.49403435	8.36E-11	1.49E-08
ENSG000001	5740.84744	0.12826649	0.05063246	2.53328551	0.01129989	0.05210468
ENSG000001	4590.06332	0.2327721	0.10775649	2.16016785	0.03075968	0.10218121
ENSG000001	663.176136	-0.2828795	0.07854296	-3.601589	0.00031628	0.00414881
ENSG000001	5824.26977	0.25128621	0.10635229	2.36277196	0.01813883	0.07159902
ENSG000001	2235.48878	0.23727064	0.13663789	1.73649223	0.08247684	0.19829512
ENSG000001	2512.41649	0.38831174	0.12747454	3.04619048	0.00231761	0.0175043
ENSG000001	1743.40417	0.55600063	0.16077504	3.4582522	0.00054369	0.00625769
ENSG000001	742.451584	0.17522313	0.07889787	2.22088533	0.02635873	0.09208283
ENSG000001	1923.55574	0.33531502	0.09895604	3.38852493	0.0007027	0.00759032
ENSG000001	1075.85336	0.11831008	0.05656592	2.09154322	0.0364794	0.1140642
ENSG000001	313.888589	0.23668192	0.1006684	2.35110448	0.01871778	0.07314476
ENSG000001	467.938866	0.1746726	0.09664254	1.80740894	0.07069854	0.17884048
ENSG000001	71.2118348	0.67764998	0.23636195	2.86700116	0.00414381	0.02626445
ENSG000001	203.952973	1.56184807	0.26934938	5.79859541	6.69E-09	6.16E-07
ENSG000001	981.848822	0.1001147	0.04020257	2.49025653	0.01276509	0.05675413
ENSG000001	3743.97094	0.09418336	0.05000723	1.8833949	0.05964687	0.15973048
ENSG000001	893.334691	-0.2483553	0.09994802	-2.4848448	0.01296079	0.05731341
ENSG000001	524.800414	-0.72198	0.15875902	-4.5476473	5.42E-06	0.00017156
ENSG000001	4119.37662	0.71431155	0.23277374	3.06869474	0.00214996	0.01656622

ENSG000001	1439.60036	-0.1505127	0.070903	-2.1227975	0.03377082	0.10887537
ENSG000001	1501.62202	0.44333306	0.17534571	2.52833709	0.01146043	0.0526377
ENSG000001	7613.82536	0.25998139	0.10466681	2.48389513	0.01299541	0.05742002
ENSG000001	634.119947	0.12774726	0.07107994	1.79723352	0.07229856	0.181627
ENSG000001	164.642303	-0.26801	0.10911937	-2.4561175	0.01404472	0.06020688
ENSG000001	169.36541	0.82523302	0.20943061	3.94036496	8.14E-05	0.00153336
ENSG000001	810.820588	-0.3368968	0.14987572	-2.2478413	0.02458631	0.08806286
ENSG000001	3604.75956	-0.1142665	0.05270002	-2.1682445	0.03014009	0.10081743
ENSG000001	20954.5421	0.53070683	0.21433098	2.47610881	0.01328231	0.05821706
ENSG000001	23735.9532	0.47438031	0.23030001	2.05983627	0.0394142	0.12014912
ENSG000001	138.367503	2.42053838	0.39958289	6.05766268	1.38E-09	1.64E-07
ENSG000001	112.543004	-0.3555785	0.13536068	-2.6268965	0.00861675	0.04334532
ENSG000001	27383.1996	2.31185348	0.34264006	6.74717792	1.51E-11	3.20E-09
ENSG000001	4050.80827	0.2975064	0.11896254	2.50084111	0.01238988	0.05556805
ENSG000001	4549.40297	0.23963509	0.0751817	3.18741237	0.00143552	0.01260076
ENSG000001	2743.71561	0.28161655	0.14702432	1.91544201	0.05543616	0.15157357
ENSG000001	28.3701042	0.94557667	0.32860519	2.87754636	0.00400781	0.0256203
ENSG000001	1238.4352	0.29148237	0.1186489	2.45667999	0.01402275	0.06015989
ENSG000001	5440.15473	-0.1622535	0.06991793	-2.3206278	0.02030694	0.07745356
ENSG000001	1269.91751	-0.186129	0.06668812	-2.7910375	0.00525394	0.03099994
ENSG000001	28.3655795	1.40963691	0.339895	4.14727168	3.36E-05	0.00076518
ENSG000001	2289.60202	0.17650583	0.06620642	2.66599289	0.00767613	0.03994463
ENSG000001	5151.40307	0.08180233	0.04635892	1.76454344	0.07764053	0.19009304
ENSG000001	1690.1087	-0.1964466	0.0972034	-2.0209849	0.04328133	0.12784795
ENSG000001	5482.59397	0.26860945	0.13939757	1.92693063	0.05398828	0.14895604
ENSG000001	3197.80073	-0.2567452	0.13759117	-1.8660007	0.06204126	0.1641056
ENSG000001	394.292007	-0.443932	0.20702474	-2.1443426	0.03200545	0.10493767
ENSG000001	265.529379	-0.181711	0.08089333	-2.2463045	0.02468451	0.08829667
ENSG000001	1367.23177	-0.1667323	0.07210902	-2.3122256	0.02076526	0.07870733
ENSG000001	114.353961	0.5603695	0.17769091	3.15361934	0.00161259	0.01369326
ENSG000001	567.289089	-0.4289512	0.11901164	-3.6042797	0.00031302	0.00411595
ENSG000001	1933.07873	-0.0985166	0.05246085	-1.8779078	0.06039377	0.16106236
ENSG000001	82.2326861	0.68151657	0.15090071	4.51632446	6.29E-06	0.00019285
ENSG000001	480.180484	-0.2453016	0.08290349	-2.9588809	0.00308758	0.02142493
ENSG000001	87.2476491	1.45828191	0.31605159	4.61406289	3.95E-06	0.00013028
ENSG000001	595.600715	-0.1889806	0.09513135	-1.986523	0.04697528	0.1355603
ENSG000001	166.683364	-0.4297836	0.20445814	-2.1020616	0.03554788	0.11232966
ENSG000001	8321.92433	0.32174354	0.17166492	1.87425325	0.06089553	0.16191081
ENSG000001	5994.32155	0.33396062	0.12083029	2.76388166	0.00571183	0.03255363
ENSG000001	204.217606	-0.1548865	0.08005115	-1.9348445	0.05300938	0.14720196
ENSG000001	112.310861	-0.3185648	0.14476815	-2.2005172	0.02777022	0.09545508
ENSG000001	1004.47627	-0.4890436	0.129975	-3.7625976	0.00016816	0.00266727
ENSG000001	106.70905	0.43507015	0.23580685	1.84502765	0.06503352	0.1692895

ENSG000001	4127.54244	0.47937359	0.12499841	3.83503757	0.00012555	0.00215106
ENSG000001	59.4518153	0.97434988	0.23891523	4.07822417	4.54E-05	0.00096246
ENSG000001	3051.80728	0.4935013	0.1156754	4.26625966	1.99E-05	0.00048866
ENSG000001	1844.96215	-0.1827612	0.07211939	-2.534148	0.01127211	0.05200585
ENSG000001	1311.94754	-0.2034078	0.06972106	-2.9174512	0.00352905	0.0234845
ENSG000001	11021.9692	0.40517249	0.19742814	2.05225305	0.04014508	0.12169116
ENSG000001	488.287332	-0.5396578	0.20578616	-2.6224201	0.00873077	0.04359272
ENSG000001	8264.02135	0.5755182	0.17971937	3.202316	0.00136327	0.01212827
ENSG000001	1313.50467	0.22122257	0.06784004	3.26094424	0.00111042	0.01048821
ENSG000001	4917.89051	0.07129317	0.03442913	2.070722	0.03838478	0.11798173
ENSG000001	13276.6998	0.14548487	0.06966777	2.08826644	0.03677381	0.1147459
ENSG000001	750.158982	-0.2205286	0.07144392	-3.0867377	0.00202366	0.01591461
ENSG000001	342.742469	-0.4695073	0.19933667	-2.3553484	0.01850535	0.07259143
ENSG000001	2908.27951	0.27256069	0.07940723	3.43244188	0.00059817	0.00670877
ENSG000001	6752.4806	0.30771048	0.08706196	3.53438495	0.00040873	0.00510813
ENSG000001	626.397958	0.22035889	0.10818341	2.03690094	0.04165997	0.1245605
ENSG000001	1291.03882	0.12598385	0.05777099	2.18074602	0.02920221	0.09866651
ENSG000001	4292.36422	0.11623769	0.05011751	2.3193028	0.02037862	0.07764462
ENSG000001	116.786244	0.43780195	0.24395409	1.79460793	0.07271619	0.18234093
ENSG000001	825.265545	0.61071894	0.22284933	2.74050156	0.00613455	0.03421367
ENSG000001	5499.10959	0.77881591	0.21871463	3.56087707	0.00036962	0.00470546
ENSG000001	16964.2172	1.08998201	0.24844634	4.38719286	1.15E-05	0.00031432
ENSG000001	2960.0062	0.14396757	0.04979014	2.89148752	0.00383423	0.02485658
ENSG000001	9379.55936	0.34311691	0.1385275	2.47688665	0.0132534	0.05810588
ENSG000001	531.33355	0.28196669	0.07793447	3.61799703	0.00029689	0.00396105
ENSG000001	328.926341	0.41973728	0.11536015	3.63849459	0.00027424	0.00374409
ENSG000001	2626.74421	-0.2018718	0.068157	-2.9618642	0.00305783	0.021293
ENSG000001	6329.10282	0.19113682	0.05779138	3.3073586	0.0009418	0.00932532
ENSG000001	442.515786	-0.1818487	0.07942737	-2.2894963	0.02205053	0.08193124
ENSG000001	4991.36581	0.26487081	0.1387106	1.9095211	0.0561949	0.15278193
ENSG000001	35451.3078	-0.1297622	0.0456142	-2.8447765	0.00444426	0.02758233
ENSG000001	808.342378	0.36430643	0.12696171	2.86941968	0.00411226	0.02613515
ENSG000001	2365.60479	-0.1165481	0.06552922	-1.7785668	0.07531082	0.18645199
ENSG000001	4337.82411	-0.2432273	0.06609599	-3.6799097	0.00023332	0.0033383
ENSG000001	663.485619	0.32058677	0.10263105	3.1236821	0.00178603	0.01465745
ENSG000001	81.1335572	-0.5797437	0.31886676	-1.8181379	0.06904306	0.17617476
ENSG000001	8916.71764	0.47017286	0.14365843	3.27285253	0.00106468	0.01016156
ENSG000001	90.0867507	0.23032987	0.10919268	2.10938935	0.03491099	0.11118755
ENSG000001	1021.91412	-0.1967411	0.10946068	-1.7973681	0.07227721	0.18160119
ENSG000001	2156.75632	0.26298645	0.11299949	2.32732416	0.01994802	0.07649384
ENSG000001	431.376633	0.93133889	0.20314729	4.58454996	4.55E-06	0.00014772
ENSG000001	2707.94458	1.00708691	0.25474479	3.95331701	7.71E-05	0.00146783
ENSG000001	176733.4	1.0509801	0.29775869	3.52963705	0.00041613	0.00516916

ENSG000001	5971.62675	0.37336092	0.12563924	2.97169029	0.00296165	0.02077517
ENSG000001	230.476772	0.34974825	0.1251533	2.79455878	0.00519706	0.03079633
ENSG000001	159.776768	0.34811904	0.14209031	2.4499844	0.01428624	0.06090932
ENSG000001	197.549667	0.26465719	0.11742306	2.25387747	0.02420388	0.08724356
ENSG000001	1537.01442	-0.4689802	0.15499474	-3.0257811	0.00247992	0.01829204
ENSG000001	1422.80119	-0.3442465	0.121047	-2.843908	0.00445639	0.02763671
ENSG000001	7523.85835	0.48412605	0.18228232	2.65591332	0.00790939	0.0407703
ENSG000001	1742.9522	0.2054227	0.05727174	3.58680706	0.00033475	0.00435629
ENSG000001	1741.73855	0.51713513	0.19625302	2.63504293	0.00841266	0.04262746
ENSG000001	560.082492	0.30377724	0.0999225	3.04012858	0.00236477	0.01775881
ENSG000001	6687.45968	0.31596306	0.10703097	2.95207131	0.0031565	0.02177444
ENSG000001	8575.53242	-0.0455669	0.02466809	-1.8472006	0.06471808	0.16870943
ENSG000001	1117.76388	0.41160088	0.16645102	2.47280485	0.01340573	0.05852337
ENSG000001	192.84523	-0.2956887	0.12700026	-2.3282527	0.01989869	0.07634039
ENSG000001	724.987537	0.32821136	0.18362739	1.7873769	0.07387659	0.18424809
ENSG000001	1767.63522	0.60690715	0.15150567	4.00583792	6.18E-05	0.00122527
ENSG000001	233.730027	0.43355617	0.11887452	3.64717505	0.00026514	0.00365335
ENSG000001	133.990937	-0.3519882	0.15209666	-2.3142402	0.02065455	0.0784146
ENSG000001	207.492232	-0.2192307	0.10707595	-2.0474322	0.04061567	0.1223994
ENSG000001	1416.63599	-0.1134049	0.05850444	-1.9383974	0.05257476	0.14636135
ENSG000001	3564.99591	-0.2355535	0.06366271	-3.7000231	0.00021558	0.00317598
ENSG000001	3347.70931	0.28701996	0.11310486	2.53764477	0.01116012	0.05161988
ENSG000001	1737.39069	-0.1459625	0.07898225	-1.8480412	0.06459638	0.1684727
ENSG000001	244.186585	0.23480567	0.08769556	2.67750923	0.00741718	0.03905572
ENSG000001	6629.56748	0.32990143	0.10479627	3.14802644	0.00164377	0.0138283
ENSG000001	2425.43356	0.16898705	0.05452682	3.0991549	0.00194074	0.0154969
ENSG000001	79.2629994	0.55875307	0.19209119	2.90879066	0.0036283	0.02399036
ENSG000001	3964.69629	0.85854026	0.16491017	5.20610874	1.93E-07	1.08E-05
ENSG000001	2161.77827	-0.413253	0.14906286	-2.7723405	0.00556548	0.03203619
ENSG000001	206.706304	0.37931427	0.19637139	1.93161683	0.05340682	0.14779942
ENSG000001	3930.96543	0.16504502	0.0896375	1.84124974	0.06558497	0.17021158
ENSG000001	2299.98178	0.3100636	0.15222847	2.03683047	0.04166703	0.1245605
ENSG000001	1537.88671	0.32450378	0.12024824	2.69861565	0.00696285	0.03742049
ENSG000001	3507.73037	0.22885048	0.08820471	2.59453824	0.00947181	0.04616802
ENSG000001	830.54573	0.20412334	0.10042725	2.03254938	0.04209807	0.12528748
ENSG000001	213.793496	0.72197883	0.17438623	4.14011367	3.47E-05	0.00078186
ENSG000001	3885.78672	0.29017854	0.12921823	2.24564696	0.02472663	0.08831247
ENSG000001	7471.47167	0.43090951	0.20141689	2.13939115	0.032404	0.10583687
ENSG000001	582.287484	0.33034061	0.11567865	2.85567483	0.00429455	0.02688347
ENSG000001	621.169817	-0.1405591	0.06147351	-2.2864983	0.02222512	0.08223139
ENSG000001	5789.31515	0.34286881	0.10416848	3.29148316	0.00099661	0.00973278
ENSG000001	5461.99269	-0.0644976	0.03164023	-2.0384666	0.04150329	0.12418422
ENSG000001	147.33271	-0.4678157	0.26534663	-1.7630361	0.0778944	0.1903479

ENSG000001	52197.8045	0.71604004	0.15226847	4.70248383	2.57E-06	8.95E-05
ENSG000001	820.01977	0.84902599	0.17496284	4.85260743	1.22E-06	4.96E-05
ENSG000001	644.828716	0.16147943	0.08347389	1.93449036	0.05305287	0.14721744
ENSG000001	1380.64351	0.10744647	0.04583797	2.34404948	0.01907564	0.07406661
ENSG000001	143.865864	0.34263332	0.13722104	2.49694448	0.01252686	0.05590715
ENSG000001	11105.7121	0.42401497	0.14908576	2.84410115	0.00445369	0.02763041
ENSG000001	292.589288	-0.2108711	0.11187249	-1.8849235	0.05944016	0.15954451
ENSG000001	3468.89393	-0.3457917	0.17153351	-2.0158841	0.0438121	0.12895118
ENSG000001	13071.2189	0.5310911	0.16614576	3.19653712	0.00139088	0.0123011
ENSG000001	1752.65148	0.29589968	0.0976318	3.0307716	0.0024393	0.01811465
ENSG000001	5044.44085	0.31295331	0.08566488	3.65322772	0.00025896	0.00359851
ENSG000001	2414.26294	2.54932601	0.36258393	7.03099561	2.05E-12	6.34E-10
ENSG000001	1743.77253	0.18447186	0.1030455	1.79019819	0.07342206	0.18346563
ENSG000001	545.147564	0.25663088	0.06804135	3.7716898	0.00016215	0.00259639
ENSG000001	127.210542	-0.2432041	0.13193725	-1.8433315	0.06528062	0.16972116
ENSG000001	6458.19475	0.28248238	0.11379229	2.4824387	0.01304865	0.05754673
ENSG000001	1064.02893	0.3171727	0.08524767	3.72060248	0.00019875	0.0030119
ENSG000001	1744.96281	0.22154154	0.08526083	2.59839754	0.009366	0.04576111
ENSG000001	1175.83948	0.16376407	0.09316665	1.75775407	0.07878936	0.19196273
ENSG000001	11.5140681	2.77963852	0.52235713	5.32133741	1.03E-07	6.52E-06
ENSG000001	9217.82714	0.51477733	0.17554712	2.93241688	0.00336335	0.02276996
ENSG000001	267.115302	-0.744257	0.19594535	-3.7982887	0.0001457	0.00240586
ENSG000001	263.357577	0.25418942	0.12304535	2.06581893	0.03884558	0.11896731
ENSG000001	3704.18082	0.08486111	0.04295085	1.97577272	0.04818052	0.13780151
ENSG000001	936.35787	-0.2850254	0.11385988	-2.5033	0.01230412	0.05530446
ENSG000001	215.839895	-0.1532783	0.08025581	-1.9098719	0.0561497	0.15270968
ENSG000001	179.781771	-0.2039391	0.09688203	-2.1050255	0.03528909	0.11186143
ENSG000001	324.358972	0.17766665	0.0742802	2.39184389	0.01676397	0.06816093
ENSG000001	163.591489	0.57179824	0.12800561	4.46697792	7.93E-06	0.0002348
ENSG000001	1563.4386	-0.3165705	0.13946238	-2.2699345	0.02321156	0.08467184
ENSG000001	746.472803	0.55933393	0.13758505	4.0653685	4.80E-05	0.00100171
ENSG000001	83.8126144	1.07010652	0.2224427	4.8107064	1.50E-06	5.80E-05
ENSG000001	307.105982	-0.6843902	0.35283795	-1.9396729	0.05241945	0.14612746
ENSG000001	311.683247	0.32057525	0.15060789	2.12854228	0.03329215	0.10774752
ENSG000001	563.89387	0.21943252	0.11434218	1.91908643	0.0549734	0.15098807
ENSG000001	250.46891	0.26552746	0.11825157	2.24544543	0.02473955	0.08833938
ENSG000001	817.789286	0.29174323	0.08436316	3.45818288	0.00054383	0.00625769
ENSG000001	57.6769849	1.14642119	0.27822419	4.12049431	3.78E-05	0.00083715
ENSG000001	103.741715	0.30503414	0.12312968	2.47734042	0.01323656	0.05809418
ENSG000001	447.253107	0.17732014	0.08679391	2.0430021	0.04105223	0.12327773
ENSG000001	1828.41883	-0.1557627	0.0735243	-2.1185198	0.03413107	0.1094641
ENSG000001	1346.07425	-0.2331386	0.06672525	-3.4940094	0.00047582	0.00568251
ENSG000001	1762.38532	-0.2291951	0.09806246	-2.3372354	0.01942695	0.0749162

ENSG000001	1601.20384	0.13585385	0.06362796	2.13512805	0.03275055	0.10657091
ENSG000001	123.937305	0.46047587	0.18656817	2.46813736	0.01358182	0.05901958
ENSG000001	1955.94675	0.72312698	0.25171221	2.87283233	0.0040681	0.02593945
ENSG000001	4620.57804	0.711246	0.23925628	2.97273704	0.00295157	0.0207534
ENSG000001	5097.15833	-0.160331	0.07822443	-2.0496284	0.04040071	0.12204874
ENSG000001	100.237835	1.6453458	0.48746191	3.37533204	0.00073727	0.00789568
ENSG000001	15322.7281	-0.1175704	0.0628631	-1.8702612	0.06144755	0.162924
ENSG000001	2018.01979	-0.1820821	0.08473162	-2.1489273	0.03164016	0.10413564
ENSG000001	729.634141	-0.1650756	0.0710525	-2.32329	0.02016358	0.0770322
ENSG000001	429.902326	0.71576946	0.22644813	3.16085389	0.00157307	0.01346547
ENSG000001	2385.54108	0.19817264	0.06394661	3.09903258	0.00194154	0.0154969
ENSG000001	320.537241	0.53964753	0.23467012	2.29960053	0.02147086	0.08063622
ENSG000001	99.5939693	-0.7863077	0.31780998	-2.474144	0.01335558	0.05841332
ENSG000001	2647.14998	0.25476157	0.10413878	2.446366	0.01443044	0.06133125
ENSG000001	576.874505	0.17903641	0.0748555	2.39176039	0.01676779	0.06816093
ENSG000001	437.247207	0.34053219	0.15751387	2.1619187	0.03062444	0.10193401
ENSG000001	31178.8804	0.50884347	0.1369463	3.71564225	0.00020269	0.00305186
ENSG000001	5695.69652	0.8369495	0.15438061	5.42133812	5.92E-08	4.08E-06
ENSG000001	25.6837051	1.7288238	0.39672342	4.35775583	1.31E-05	0.00035206
ENSG000001	992.110695	0.45184911	0.16561635	2.72828808	0.0063664	0.03510082
ENSG000001	27298.7108	0.25882091	0.12814757	2.01970992	0.04341349	0.1281059
ENSG000001	785.34855	0.59546221	0.14285727	4.16823175	3.07E-05	0.00071194
ENSG000001	162444.322	0.73802618	0.29951214	2.46409437	0.01373599	0.05945627
ENSG000001	508.750087	-0.1299111	0.07309672	-1.7772492	0.07552725	0.18676223
ENSG000001	81.4109943	0.92328919	0.19780999	4.66755582	3.05E-06	0.00010348
ENSG000001	5426.15055	-0.0789406	0.03916942	-2.0153641	0.04386652	0.12904185
ENSG000001	1272.27016	-0.3759534	0.13754174	-2.7333764	0.00626886	0.03475002
ENSG000001	5834.87023	0.25025099	0.1014599	2.46650128	0.01364402	0.05918547
ENSG000001	110.407317	1.37455036	0.304673	4.51155941	6.44E-06	0.0001965
ENSG000001	204.79443	-0.3131803	0.13867828	-2.258323	0.02392553	0.08665281
ENSG000001	4851.85225	0.29891138	0.10740116	2.78312985	0.00538373	0.03143766
ENSG000001	321.631414	-0.47568	0.17081441	-2.7847765	0.00535646	0.03137843
ENSG000001	1948.55978	-0.3184403	0.14767241	-2.1563967	0.0310527	0.10288364
ENSG000001	10984.1967	0.24880743	0.07640955	3.2562348	0.001129	0.01059054
ENSG000001	767.981109	0.14135424	0.07054235	2.00382082	0.04508926	0.1315998
ENSG000001	573.389718	0.17976813	0.10380219	1.73183361	0.08330319	0.19972655
ENSG000001	3854.65497	0.33616895	0.12564691	2.67550521	0.00746167	0.03921443
ENSG000001	381.938386	0.18720169	0.10588668	1.76794362	0.07707033	0.18903847
ENSG000001	298.37234	0.14402282	0.06626529	2.17342778	0.02974814	0.09989355
ENSG000001	317.693965	0.44518164	0.17765428	2.50588747	0.01221445	0.05503128
ENSG000001	744.367374	-0.2071191	0.0635905	-3.2570762	0.00112566	0.01056735
ENSG000001	277.230729	1.95997633	0.44001633	4.4543264	8.42E-06	0.00024641
ENSG000001	2892.53561	-0.2766867	0.09826118	-2.8158295	0.00486515	0.02938263

ENSG000001	239.929263	-0.2838783	0.13013706	-2.1813794	0.02915537	0.09852856
ENSG000001	103.474297	1.41220401	0.36847622	3.83255133	0.00012682	0.00216613
ENSG000001	1791.81683	-0.1383007	0.06062547	-2.2812313	0.02253477	0.08288527
ENSG000001	101012.098	0.26978761	0.13151183	2.05143225	0.04022487	0.12175876
ENSG000001	452.335278	0.41632372	0.14103709	2.95187409	0.00315852	0.02177444
ENSG000001	1166.39315	0.17931976	0.0490789	3.65370359	0.00025848	0.00359733
ENSG000001	12334.6734	0.35499127	0.12871096	2.75805015	0.00581473	0.03297962
ENSG000001	208153.653	0.85737452	0.26400311	3.24759246	0.00116386	0.01081234
ENSG000001	5213.17761	1.16634976	0.37586427	3.1031142	0.00191496	0.0153844
ENSG000001	744.149814	0.23037288	0.10401484	2.2148078	0.02677326	0.09309119
ENSG000001	463.781437	0.56099243	0.22272062	2.5188168	0.01177499	0.05358715
ENSG000001	199.398548	-0.5185913	0.17745971	-2.9223047	0.00347451	0.02326447
ENSG000001	2860.91656	0.32424141	0.11343402	2.85841425	0.00425764	0.02675198
ENSG000001	1839.0926	-0.1308711	0.06253468	-2.0927758	0.03636918	0.1139608
ENSG000001	5135.58246	0.72352542	0.19265353	3.75557831	0.00017294	0.00271361
ENSG000001	4554.22282	-0.2607724	0.09184038	-2.8394089	0.00451972	0.02788181
ENSG000001	1275.66936	-0.2443481	0.11772298	-2.0756193	0.03792917	0.11710123
ENSG000001	4566.81694	0.41771438	0.16572235	2.52056761	0.01171657	0.05341427
ENSG000001	534.912986	0.44552035	0.11223306	3.96960011	7.20E-05	0.00138716
ENSG000001	24417.7884	0.19215702	0.10564267	1.81893377	0.06892153	0.1759755
ENSG000001	5328.51422	0.1617549	0.07095662	2.27963102	0.02262958	0.08312393
ENSG000001	130.574352	-0.3553555	0.13748923	-2.5846064	0.00974902	0.04700227
ENSG000001	1724.77937	-0.4461444	0.17968142	-2.4829748	0.01302903	0.05749112
ENSG000001	991.596367	0.60051816	0.13280004	4.52197286	6.13E-06	0.00018848
ENSG000001	120.985934	-0.5376516	0.28273368	-1.9016184	0.05722106	0.15510889
ENSG000001	172.677387	0.25033254	0.14348206	1.74469576	0.08103784	0.19569624
ENSG000001	206.914301	0.93848595	0.22809097	4.11452476	3.88E-05	0.00085194
ENSG000001	5637.60783	-0.1673013	0.05777953	-2.8955117	0.00378541	0.02466005
ENSG000001	2356.93013	1.89601796	0.35274127	5.37509532	7.65E-08	5.08E-06
ENSG000001	84256.2878	0.41604958	0.17764774	2.34199199	0.01918113	0.07435294
ENSG000001	20701.2224	0.84975027	0.15498773	5.48269394	4.19E-08	3.03E-06
ENSG000001	1239.86581	0.84522525	0.2085033	4.05377401	5.04E-05	0.00103946
ENSG000001	950.991754	-0.3447973	0.09775247	-3.5272489	0.0004199	0.00520812
ENSG000001	5262.1758	0.29189988	0.13911878	2.09820621	0.03588693	0.1130461
ENSG000001	1038.20136	-0.4246532	0.12928603	-3.2846022	0.00102126	0.00990868
ENSG000001	3264.89894	-0.1472463	0.06429417	-2.2901962	0.02200994	0.08192896
ENSG000001	429.031301	-0.3646968	0.10026605	-3.6372909	0.00027552	0.00375277
ENSG000001	746.408701	-0.2818387	0.08181682	-3.4447528	0.00057158	0.00648149
ENSG000001	911.87558	-0.4120258	0.20231345	-2.0365713	0.04169302	0.12457006
ENSG000001	130.89743	-0.1912815	0.08223627	-2.3259998	0.02001856	0.0766747
ENSG000001	662.382161	0.37324656	0.10071988	3.70578836	0.00021073	0.00312707
ENSG000001	6940.20349	0.36755519	0.11053234	3.32531818	0.00088318	0.00892646
ENSG000001	155.184494	1.39201163	0.2773309	5.0193168	5.19E-07	2.45E-05

ENSG000001	138.478175	0.36510181	0.19048092	1.91673693	0.05527136	0.15135761
ENSG000001	53.1676395	2.1701685	0.50111224	4.33070344	1.49E-05	0.00038619
ENSG000001	220.915988	0.56276801	0.19080193	2.94948806	0.00318301	0.02191091
ENSG000001	136.378026	1.13725217	0.22254543	5.1102024	3.22E-07	1.68E-05
ENSG000001	310.982347	-0.5358403	0.25820947	-2.0752154	0.03796658	0.11716566
ENSG000001	1921.64496	0.33305117	0.15165636	2.19609108	0.02808542	0.09606106
ENSG000001	2525.66366	-0.138603	0.06705377	-2.0670429	0.03873011	0.11868017
ENSG000001	13788.4451	0.17631423	0.08504319	2.07323167	0.03815072	0.11753725
ENSG000001	1845.13868	0.0957563	0.05286556	1.81131706	0.07009179	0.17763447
ENSG000001	281.038728	1.79140841	0.32067402	5.58638461	2.32E-08	1.77E-06
ENSG000001	2942.57061	0.42440032	0.13548288	3.13250142	0.00173324	0.01433899
ENSG000001	9590.42181	0.92366538	0.21809818	4.2350898	2.28E-05	0.000551
ENSG000001	13788.3152	1.02658639	0.15957489	6.43325772	1.25E-10	2.07E-08
ENSG000001	360.298407	-0.3475338	0.13322767	-2.6085704	0.00909213	0.04490472
ENSG000001	1272.37485	0.1466583	0.07359349	1.9928161	0.04628159	0.1340776
ENSG000001	2052.76584	-0.1003044	0.05527707	-1.8145754	0.06958918	0.17707228
ENSG000001	2352.40364	0.26641121	0.06886536	3.86858091	0.00010947	0.00193426
ENSG000001	3499.35692	0.13283189	0.05604506	2.37009097	0.01778371	0.07071951
ENSG000001	1310.86864	-0.1094324	0.05385507	-2.0319793	0.04215575	0.12539911
ENSG000001	1133.05933	-0.2323154	0.10169522	-2.2844279	0.02234639	0.08246072
ENSG000001	7909.45103	-0.0937707	0.03744113	-2.5044851	0.01226298	0.05519517
ENSG000001	6764.52672	-0.1405134	0.06568097	-2.139332	0.03240879	0.10583687
ENSG000001	2916.73704	-0.0806119	0.04286149	-1.8807536	0.06000545	0.16040257
ENSG000001	2139.42275	1.25004949	0.47177149	2.64969275	0.0080565	0.04123243
ENSG000001	88.1442357	-0.3707521	0.1741026	-2.1295034	0.03321263	0.10760474
ENSG000001	419.098143	0.64203573	0.13476534	4.76410136	1.90E-06	7.01E-05
ENSG000001	311.999908	-0.1627179	0.07682953	-2.1179079	0.03418287	0.10957899
ENSG000001	1621.1871	0.97566407	0.22469216	4.34222571	1.41E-05	0.00037123
ENSG000001	586.081954	-0.1114537	0.05501643	-2.0258259	0.04278263	0.12671725
ENSG000001	1510.60343	-0.2466278	0.05966523	-4.1335267	3.57E-05	0.00080132
ENSG000001	76.1649759	1.58571352	0.32127303	4.93571939	7.99E-07	3.47E-05
ENSG000001	1416.53972	2.04224894	0.3794133	5.38264984	7.34E-08	4.91E-06
ENSG000001	1954.85837	-0.2733683	0.08858265	-3.0860255	0.00202851	0.01593749
ENSG000001	276.42473	0.20503269	0.09155687	2.23940256	0.02512974	0.08929329
ENSG000001	722.646923	0.25727153	0.05853529	4.39515222	1.11E-05	0.0003066
ENSG000001	16981.2349	1.06656641	0.17070492	6.24801199	4.16E-10	5.50E-08
ENSG000001	59.410547	0.60061995	0.20839978	2.88205658	0.00395089	0.02538635
ENSG000001	5845.57053	0.69386098	0.2315768	2.99624559	0.00273326	0.01966101
ENSG000001	2029.73442	0.11294748	0.06347448	1.77941576	0.07517163	0.18621984
ENSG000001	2515.67836	-0.1395991	0.07195313	-1.940139	0.0523628	0.14601818
ENSG000001	13072.3878	-0.148406	0.04378317	-3.3895673	0.00070003	0.00757339
ENSG000001	5451.00082	0.46327995	0.11954147	3.87547463	0.00010642	0.00189049
ENSG000001	4170.30867	1.24441644	0.25246487	4.92906779	8.26E-07	3.56E-05

ENSG000001	675.231974	1.51025539	0.28517758	5.29584197	1.18E-07	7.32E-06
ENSG000001	568.121665	0.92755239	0.26052161	3.56036647	0.00037034	0.00470863
ENSG000001	219.509801	0.3637436	0.13794987	2.6367811	0.00836968	0.04248845
ENSG000001	41.6855861	0.74109345	0.25916772	2.85951291	0.00424292	0.02670679
ENSG000001	4101.7014	-0.1203722	0.06638903	-1.8131333	0.06981126	0.17729017
ENSG000001	994.080793	0.287777	0.11711532	2.45721062	0.01400206	0.06013261
ENSG000001	262.202296	-0.3535266	0.1376689	-2.567948	0.01023025	0.04857962
ENSG000001	3169.7647	0.39390325	0.12877684	3.05880497	0.00222222	0.01698151
ENSG000001	4031.83821	-0.1091016	0.05149101	-2.1188473	0.03410337	0.1094097
ENSG000001	201.041873	-0.1528618	0.08712731	-1.7544649	0.07935088	0.19290087
ENSG000001	2544.85382	0.10859916	0.05857791	1.85392676	0.06374964	0.16688402
ENSG000001	554.932273	-0.1836044	0.09426364	-1.9477757	0.05144181	0.14425369
ENSG000001	21.9862016	1.23586681	0.64451324	1.91751967	0.05517194	0.15125213
ENSG000001	745.700457	-0.262458	0.11814241	-2.2215391	0.02631447	0.09198737
ENSG000001	1282.45954	-0.1519983	0.08031904	-1.8924322	0.05843342	0.15756171
ENSG000001	336.963175	-0.1625925	0.08989078	-1.8087785	0.07048542	0.17838392
ENSG000001	5763.6187	-0.2853345	0.08267262	-3.4513784	0.00055773	0.0063729
ENSG000001	2135.61654	0.41836162	0.13575525	3.08173427	0.00205798	0.01606129
ENSG000001	514.919	0.21316269	0.11373157	1.87426131	0.06089442	0.16191081
ENSG000001	28506.9452	0.27695242	0.1219699	2.27066207	0.02316744	0.08456743
ENSG000001	7616.87836	0.20535834	0.09812762	2.09276794	0.03636988	0.1139608
ENSG000001	540.793329	-0.4217771	0.20743904	-2.0332582	0.04202644	0.12515575
ENSG000001	155.69525	0.36420218	0.16945724	2.14922762	0.03161636	0.10409139
ENSG000001	984.427821	-0.1836748	0.07732844	-2.375255	0.01753683	0.07018651
ENSG000001	218.105995	-0.1667337	0.06759657	-2.4666002	0.01364026	0.05918478
ENSG000001	139.266285	-0.4086703	0.10572668	-3.8653466	0.00011093	0.00195585
ENSG000001	1585.50859	0.40562398	0.09117666	4.44877004	8.64E-06	0.00025108
ENSG000001	190.123564	0.36812657	0.11870427	3.10120751	0.00192733	0.01545352
ENSG000001	1844.63887	0.3264599	0.09285679	3.51573553	0.00043854	0.00533039
ENSG000001	17257.2028	0.27607963	0.11573425	2.38546181	0.01705769	0.06892435
ENSG000001	315.995837	-0.4780236	0.11729131	-4.0755244	4.59E-05	0.00096885
ENSG000001	2652.84804	-0.2099216	0.06999465	-2.9991095	0.0027077	0.01951567
ENSG000001	3662.22806	0.41585587	0.12969467	3.20642227	0.00134397	0.01202237
ENSG000001	272.216691	-0.1964137	0.10273896	-1.9117739	0.05590519	0.15234793
ENSG000001	442.294889	1.34892139	0.26857922	5.0224339	5.10E-07	2.42E-05
ENSG000001	317.83284	0.17350323	0.07800264	2.22432514	0.02612658	0.09151435
ENSG000001	80.7688346	0.7030635	0.19220177	3.65794502	0.00025425	0.00355705
ENSG000001	3087.94687	0.30769932	0.09933583	3.09756639	0.00195117	0.01553826
ENSG000001	119.379777	0.27454488	0.13456156	2.04029198	0.04132125	0.12386827
ENSG000001	14161.8725	0.18759615	0.08892356	2.10963386	0.03488991	0.11118755
ENSG000001	533.79995	-0.2818694	0.09691423	-2.9084418	0.00363235	0.02400629
ENSG000001	2875.56584	0.4987647	0.1321651	3.77380034	0.00016078	0.00259153
ENSG000001	21093.4322	0.58878544	0.19810483	2.97209029	0.0029578	0.02077517

ENSG000001	38.6182266	0.72135735	0.38277481	1.88454759	0.05949094	0.15960922
ENSG000001	123.038517	-0.27175	0.11483494	-2.3664404	0.01796007	0.07106448
ENSG000001	4921.98068	0.2066797	0.08700898	2.37538364	0.01753072	0.07018651
ENSG000001	599.192771	3.55203454	0.54648002	6.49984333	8.04E-11	1.46E-08
ENSG000001	3751.68627	0.3215647	0.14391403	2.23442211	0.02545531	0.0900131
ENSG000001	2813.05002	-0.2056645	0.08782344	-2.3417949	0.01919126	0.07436008
ENSG000001	4144.39071	0.16606808	0.07981824	2.08057803	0.03747255	0.11612877
ENSG000001	638.266675	0.53818137	0.23893235	2.25244249	0.02429432	0.08736541
ENSG000001	86.5917067	0.81822445	0.24207064	3.38010613	0.00072458	0.00778056
ENSG000001	24.5815005	0.9725879	0.27199095	3.57580983	0.00034915	0.00449367
ENSG000001	452.125163	0.20086299	0.07592902	2.64540479	0.00815932	0.04163973
ENSG000001	726.273903	0.16366384	0.06422217	2.54840075	0.01082181	0.05052539
ENSG000001	38428.3906	0.1137019	0.06263867	1.81520285	0.06949274	0.1769642
ENSG000001	4556.80913	-0.1318103	0.04472243	-2.9472973	0.00320565	0.02202053
ENSG000001	228.262245	-0.3221663	0.15132184	-2.1290137	0.03325312	0.10769336
ENSG000001	130.377286	-0.2704382	0.11612021	-2.32895	0.01986171	0.07623421
ENSG000001	4487.70288	0.35722407	0.15938825	2.2412196	0.02501185	0.08904025
ENSG000001	213.365809	-0.2578881	0.09728604	-2.6508232	0.00802959	0.04118271
ENSG000001	14188.0848	0.84728559	0.16992465	4.98624296	6.16E-07	2.80E-05
ENSG000001	3114.40316	0.6029821	0.19859411	3.03625366	0.00239538	0.01791017
ENSG000001	533.621893	-0.2062806	0.0926776	-2.2257865	0.02602848	0.09130781
ENSG000001	9837.88322	0.36507362	0.14138186	2.58218149	0.00981779	0.04719507
ENSG000001	1972.31271	-0.1821077	0.07599853	-2.3962	0.01656605	0.06762098
ENSG000001	1992.92276	0.52270537	0.21994347	2.37654417	0.01747567	0.07000942
ENSG000001	635.448381	0.18286054	0.07683167	2.38001512	0.01731193	0.06956468
ENSG000001	32871.5029	0.17791978	0.09873711	1.80195448	0.07155257	0.18033315
ENSG000001	508.36491	-0.3776686	0.1399131	-2.6993081	0.00694838	0.0373869
ENSG000001	42526.8265	0.32044837	0.13101741	2.44584567	0.01445128	0.06138801
ENSG000001	4610.80769	0.22599182	0.08757499	2.58055213	0.00986425	0.04733215
ENSG000001	5107.96864	0.6527567	0.17772048	3.67294034	0.00023978	0.00340398
ENSG000001	1137.18836	1.13963427	0.17955979	6.34682349	2.20E-10	3.34E-08
ENSG000001	2426.91472	-0.3611115	0.15388254	-2.3466698	0.01894203	0.07373999
ENSG000001	2465.08725	0.18830124	0.10351841	1.81901215	0.06890958	0.1759755
ENSG000001	408.400212	0.2555854	0.10297071	2.48211749	0.01306042	0.05756767
ENSG000001	281.623224	0.26905642	0.14731886	1.8263542	0.06779693	0.17405139
ENSG000001	1979.21539	0.57183276	0.15499392	3.68938835	0.00022479	0.00325552
ENSG000001	181.519166	1.62977982	0.28436357	5.73132434	9.96E-09	8.55E-07
ENSG000001	1390.34479	0.1766498	0.09587668	1.84246889	0.06540659	0.16991572
ENSG000001	22296.859	0.84740805	0.2689342	3.1509866	0.0016272	0.01374611
ENSG000001	171.07563	0.38192912	0.1454537	2.62577805	0.00864512	0.04337763
ENSG000001	2900.67815	0.16924535	0.08538082	1.98224085	0.04745229	0.13638479
ENSG000001	1476.04542	0.34164265	0.09090639	3.75818069	0.00017115	0.00269846
ENSG000001	535.00896	1.76180971	0.32837497	5.36523757	8.08E-08	5.28E-06

ENSG000001	2240.69853	0.21633264	0.07447123	2.90491553	0.00367352	0.02416155
ENSG000001	561.102378	0.7861986	0.188468	4.17152292	3.03E-05	0.00070372
ENSG000001	4616.88806	0.1923485	0.08807471	2.18392423	0.02896782	0.09807642
ENSG000001	438.665438	-0.5358429	0.15162844	-3.5339206	0.00040944	0.00511322
ENSG000001	186.488257	0.21111241	0.11633667	1.81466787	0.06957497	0.17706421
ENSG000001	193.219836	0.48763623	0.13867793	3.51632172	0.00043757	0.00532258
ENSG000001	1016.4178	0.39802881	0.1621681	2.45442108	0.01411116	0.0604285
ENSG000001	3421.63123	-0.1582567	0.08989974	-1.7603689	0.07834528	0.1911648
ENSG000001	2675.34642	0.49778473	0.18945324	2.62748063	0.00860197	0.04330664
ENSG000001	798.918755	-0.2350278	0.07997273	-2.9388496	0.00329433	0.02246034
ENSG000001	1553.62444	0.55733914	0.23813182	2.34046477	0.01925976	0.07455199
ENSG000001	15.6102763	2.33313097	0.53174246	4.38770865	1.15E-05	0.00031432
ENSG000001	933.85526	0.40484259	0.19093149	2.1203553	0.03397609	0.10925789
ENSG000001	2632.3311	-0.1973886	0.08512638	-2.3187712	0.02040744	0.07772842
ENSG000001	1893.13156	0.23289823	0.12944586	1.79919407	0.07198799	0.18108452
ENSG000001	291.837095	-0.2889989	0.15340104	-1.8839434	0.05957264	0.15962157
ENSG000001	1399.17427	0.37232969	0.10181137	3.65705422	0.00025513	0.00356335
ENSG000001	4136.81407	0.2373605	0.10735831	2.21091873	0.02704147	0.09372203
ENSG000001	1625.55359	0.229206	0.09709129	2.36072675	0.01823916	0.07186519
ENSG000001	6798.68919	0.46438705	0.16389431	2.8334544	0.00460479	0.02824718
ENSG000001	377.640363	0.28264459	0.15502851	1.82317818	0.06827641	0.17484433
ENSG000001	81.1153467	1.21970653	0.22803561	5.34875475	8.86E-08	5.72E-06
ENSG000001	359.05476	0.92007339	0.2283312	4.02955624	5.59E-05	0.00113544
ENSG000001	219.701719	-0.3961752	0.1945713	-2.0361441	0.04173589	0.1246527
ENSG000001	1712.87031	0.20627345	0.07017373	2.93946804	0.00328776	0.02243164
ENSG000001	5371.67688	-0.2923435	0.12935839	-2.2599498	0.02382437	0.08638836
ENSG000001	5855.44971	0.10885749	0.06288558	1.73104072	0.0834445	0.19988889
ENSG000001	56.4869169	0.98365873	0.19777874	4.97353124	6.57E-07	2.97E-05
ENSG000001	17259.8894	6.13251931	0.5548048	11.0534721	2.11E-28	3.46E-24
ENSG000001	4492.23925	0.33257225	0.15160771	2.19363673	0.02826154	0.0964528
ENSG000001	630.883999	-0.2371301	0.09188654	-2.5806834	0.0098605	0.04733097
ENSG000001	99.4186759	1.30603857	0.28397834	4.59907811	4.24E-06	0.00013917
ENSG000001	2218.03205	0.29575919	0.10752812	2.75052885	0.00594992	0.03350999
ENSG000001	4114.27337	0.31670703	0.11063607	2.86260195	0.00420178	0.02653952
ENSG000001	245.697383	0.21754615	0.10460523	2.07968712	0.03755424	0.11631095
ENSG000001	1561.89223	-0.4052916	0.15579978	-2.6013617	0.00928545	0.04558223
ENSG000001	13229.5941	0.43074726	0.12964226	3.32258366	0.00089188	0.00898098
ENSG000001	715.36955	0.4676083	0.14327196	3.26378091	0.00109936	0.01040488
ENSG000001	135.590465	0.42493264	0.22247166	1.91005286	0.05612641	0.15267165
ENSG000001	4655.76941	0.14651567	0.04276919	3.4257297	0.00061315	0.00685332
ENSG000001	141.350354	-0.4730151	0.18934907	-2.4981117	0.01248568	0.05576894
ENSG000001	1950.35537	0.60680948	0.14935307	4.06291939	4.85E-05	0.00100843
ENSG000001	474.729022	0.87140006	0.22685126	3.84128379	0.00012239	0.00210806

ENSG000001	2997.96391	-0.1746887	0.06151668	-2.8396961	0.00451565	0.02786721
ENSG000001	6590.47818	-0.0963429	0.04777404	-2.0166362	0.04373349	0.12878915
ENSG000001	1468.39069	-0.1711782	0.08579569	-1.9951842	0.04602279	0.13351658
ENSG000001	1009.43279	-0.3406429	0.11414359	-2.9843363	0.00284194	0.02020823
ENSG000001	22.2931878	1.03513575	0.41740074	2.47995669	0.01313983	0.05779342
ENSG000001	1274.77719	-0.3497262	0.16240251	-2.1534529	0.0312831	0.10335602
ENSG000001	878.115853	-0.2967264	0.08050558	-3.685786	0.000228	0.00327937
ENSG000001	5969.2676	-0.1720457	0.08397444	-2.0487864	0.04048301	0.12222995
ENSG000001	2038.89581	0.38420815	0.10233631	3.7543678	0.00017378	0.00272155
ENSG000001	244.60719	-0.3592483	0.19377958	-1.8539017	0.06375322	0.16688402
ENSG000001	436.08492	-0.1698456	0.05399573	-3.1455384	0.00165781	0.01390444
ENSG000001	6002.78117	-0.1700364	0.08172179	-2.0806742	0.03746374	0.11612343
ENSG000001	183.792556	0.26869669	0.12936893	2.07698008	0.0378034	0.11688899
ENSG000001	550.714003	-0.4440552	0.23084604	-1.9235989	0.05440488	0.14977783
ENSG000001	2228.00146	-0.2650121	0.08592958	-3.0840609	0.00204196	0.01602432
ENSG000001	135.144658	0.29115071	0.11882302	2.45028877	0.01427417	0.06090386
ENSG000001	2833.85134	0.14184845	0.07320738	1.93762502	0.05266899	0.14655617
ENSG000001	334.983432	0.1293234	0.06686063	1.93422343	0.05308566	0.1472586
ENSG000001	2961.35992	0.24365574	0.12693598	1.9195167	0.05491897	0.15090156
ENSG000001	2247.67862	0.19661841	0.06695391	2.93662336	0.00331807	0.02257526
ENSG000001	8368.46104	1.40781711	0.31532237	4.46469157	8.02E-06	0.0002369
ENSG000001	1839.49855	1.13437655	0.29874423	3.79714964	0.00014637	0.00241451
ENSG000001	222.337989	0.5222644	0.18981413	2.75145161	0.00593318	0.03343173
ENSG000001	1679.15682	0.19337276	0.10261892	1.8843772	0.05951397	0.15962157
ENSG000001	875.402623	0.31221017	0.15519393	2.01174209	0.04424713	0.12985864
ENSG000001	298.732822	-0.2097802	0.10644474	-1.9707899	0.04874791	0.13898948
ENSG000001	1153.95048	0.23480078	0.06289169	3.7334151	0.0001889	0.00290564
ENSG000001	1897.85971	-0.1915465	0.10012351	-1.9131019	0.05573501	0.15206735
ENSG000001	252.538807	-0.1768846	0.08575983	-2.0625583	0.03915462	0.11960102
ENSG000001	1926.75954	-0.078793	0.0449209	-1.754038	0.07942399	0.19304999
ENSG000001	135.943421	-0.3490538	0.15869944	-2.1994647	0.0278449	0.09563737
ENSG000001	216.254359	-0.2419276	0.13930551	-1.7366693	0.08244556	0.19827807
ENSG000001	143239.084	0.61592676	0.18704201	3.29298614	0.00099129	0.00968668
ENSG000001	5366.36177	0.20644729	0.1079389	1.91263114	0.05579529	0.15217488
ENSG000001	8.70668157	2.26850027	0.66889998	3.39138938	0.00069539	0.00755049
ENSG000001	125.92164	-0.5627315	0.16310868	-3.4500399	0.0005605	0.00638272
ENSG000001	15888.2079	0.30136933	0.15334369	1.96531936	0.04937729	0.14026984
ENSG000001	253.174773	-0.3291977	0.13796224	-2.3861437	0.0170261	0.06886455
ENSG000001	142.695874	-0.4674215	0.19494093	-2.3977598	0.01649568	0.06742337
ENSG000001	4297.83849	0.27197584	0.15598511	1.74360125	0.08122865	0.19604152
ENSG000001	510.437892	-0.1467944	0.0757719	-1.9373202	0.05270622	0.14660285
ENSG000001	112.625588	1.48935872	0.25491165	5.84264673	5.14E-09	4.93E-07
ENSG000001	2257.53762	0.30312809	0.10984176	2.75967971	0.00578581	0.03288119

ENSG000001	1002.95988	0.3073793	0.11446104	2.68544917	0.00724324	0.03853177
ENSG000001	6897.25931	0.27762336	0.06666646	4.16436316	3.12E-05	0.00072004
ENSG000001	32.5664064	0.95006283	0.25180037	3.77307961	0.00016124	0.00259153
ENSG000001	68.7518933	0.45224496	0.22788547	1.98452745	0.04719707	0.13593629
ENSG000001	6390.63734	0.20684921	0.0998149	2.07232808	0.03823486	0.11766834
ENSG000001	110.245127	-0.3807625	0.12814323	-2.9713824	0.00296462	0.02077517
ENSG000001	1044.09767	-0.2489479	0.09637739	-2.5830528	0.00979303	0.04711748
ENSG000001	2292.48551	0.45766968	0.12407034	3.68879193	0.00022532	0.00325802
ENSG000001	121.818876	0.37867584	0.16541348	2.28926837	0.02206376	0.08194327
ENSG000001	2167.68737	0.15487378	0.08268514	1.8730546	0.06106085	0.16215651
ENSG000001	1494.8379	0.20008721	0.07160458	2.79433558	0.00520065	0.03079633
ENSG000001	2589.64217	0.31338493	0.11365299	2.75738389	0.00582659	0.03301265
ENSG000001	1006.12572	-0.4764679	0.16518571	-2.8844381	0.00392113	0.02528303
ENSG000001	13805.2244	1.12577166	0.32116731	3.50524989	0.00045618	0.00548385
ENSG000001	3495.91289	-0.2414943	0.11293062	-2.1384307	0.03248181	0.10591864
ENSG000001	4583.52707	0.16210356	0.0658772	2.4606928	0.0138669	0.05983569
ENSG000001	564.372888	0.32176371	0.15463155	2.08084125	0.03744844	0.11609795
ENSG000001	232.913161	0.7449581	0.31226337	2.38567243	0.01704793	0.06890187
ENSG000001	665.989734	0.20934975	0.09544139	2.19349015	0.02827208	0.0964528
ENSG000001	484.964466	-0.226964	0.11829199	-1.9186757	0.05502539	0.15099555
ENSG000001	1120.23261	0.13113257	0.07131396	1.8388065	0.06594365	0.1707371
ENSG000001	83.347808	0.93344634	0.2216854	4.21068026	2.55E-05	0.00060591
ENSG000001	4501.34405	0.21251023	0.08672268	2.45045749	0.01426748	0.06090386
ENSG000001	718.649244	1.05363028	0.27890608	3.7777243	0.00015827	0.00255929
ENSG000001	537.534519	-0.2027339	0.09251291	-2.1914126	0.02842195	0.0967881
ENSG000001	557.633514	-0.1976452	0.07901699	-2.5012999	0.01237383	0.05554168
ENSG000001	197.119214	-0.3859341	0.16783836	-2.2994394	0.02148	0.08064187
ENSG000001	831.975998	0.71501344	0.26634991	2.6844891	0.00726408	0.03856906
ENSG000001	1530.41695	0.27876228	0.08570914	3.25242178	0.00114426	0.01067868
ENSG000001	2645.47518	0.21889915	0.08367449	2.61607997	0.00889457	0.04420864
ENSG000001	706.694291	0.14119573	0.07711099	1.83107146	0.06708988	0.17275011
ENSG000001	135.422603	0.27562938	0.11256983	2.44851922	0.01434448	0.06107671
ENSG000001	945.073417	0.20321658	0.09388944	2.16442417	0.03043181	0.10144143
ENSG000001	1540.38329	-0.2390837	0.12131393	-1.9707851	0.04874846	0.13898948
ENSG000001	630.199901	0.20798403	0.09447778	2.20140692	0.02770723	0.09532426
ENSG000001	107.560976	0.2294982	0.12562768	1.82681243	0.06772798	0.17395608
ENSG000001	62.0211379	0.4378675	0.14834689	2.95164601	0.00316085	0.02177667
ENSG000001	860.92726	0.14959711	0.08064674	1.85496774	0.06360083	0.16659151
ENSG000001	3830.49695	-0.1494252	0.06601696	-2.2634363	0.02360881	0.08585354
ENSG000001	672.133827	0.39200128	0.10178338	3.85132912	0.00011748	0.00204057
ENSG000001	1479.24264	0.19723422	0.06598037	2.98928624	0.0027963	0.01997862
ENSG000001	389.146032	0.25843041	0.10088432	2.56165103	0.01041759	0.04911277
ENSG000001	37158.8845	1.00557899	0.23723932	4.23866911	2.25E-05	0.00054378

ENSG000001	748.114324	0.41879218	0.18511375	2.26235051	0.02367576	0.08597283
ENSG000001	2591.7773	0.8295288	0.24232915	3.42314898	0.000619	0.00691401
ENSG000001	730.55396	0.18298669	0.09960782	1.83707149	0.06619934	0.17115589
ENSG000001	1700.33174	0.17265765	0.06722856	2.56821887	0.01022226	0.0485644
ENSG000001	886.889203	-0.1633559	0.08461452	-1.9305898	0.0535338	0.14807587
ENSG000001	2893.00542	0.65544255	0.26483664	2.47489378	0.01332758	0.05832195
ENSG000001	400.578425	-0.210287	0.10485491	-2.0055045	0.04490913	0.13126116
ENSG000001	9111.40559	0.42976802	0.13288964	3.23402205	0.0012206	0.0112313
ENSG000001	78.0469873	0.27929485	0.15996735	1.74594903	0.08081981	0.19528476
ENSG000001	142.786813	0.9789888	0.31284762	3.12928323	0.00175233	0.01443581
ENSG000001	163.80645	0.83537358	0.19816923	4.21545555	2.49E-05	0.00059409
ENSG000001	508.59746	0.15817004	0.07756584	2.03917138	0.04143293	0.12403951
ENSG000001	208.333396	-0.2504468	0.14208862	-1.76261	0.07796628	0.1904668
ENSG000001	2061.60281	0.09955192	0.05553702	1.79253247	0.07304771	0.18289256
ENSG000001	276.714981	-0.2524752	0.11348674	-2.2247114	0.02610062	0.09147931
ENSG000001	55.417115	0.65319776	0.26150463	2.49784393	0.01249512	0.05579588
ENSG000001	238.576359	1.11136299	0.24431654	4.54886512	5.39E-06	0.00017139
ENSG000001	2904.24281	0.28967716	0.1604601	1.80529093	0.07102917	0.17937244
ENSG000001	2803.77623	0.45056099	0.0916843	4.91426565	8.91E-07	3.81E-05
ENSG000001	17812.1032	0.32956821	0.14277813	2.30825408	0.02098501	0.07931719
ENSG000001	3768.11724	0.18580755	0.09938433	1.869586	0.06154133	0.16312532
ENSG000001	33377.0146	0.47745993	0.13442748	3.55180308	0.0003826	0.00484441
ENSG000001	572.404772	0.68404996	0.14448799	4.73430343	2.20E-06	7.85E-05
ENSG000001	2685.72015	-0.2909147	0.08610993	-3.3784107	0.00072906	0.00781845
ENSG000001	581.349926	0.34143175	0.11934581	2.86086086	0.00422492	0.02661394
ENSG000001	141.930034	2.25253715	0.31819844	7.07903269	1.45E-12	4.96E-10
ENSG000001	8275.34549	0.24913803	0.1064939	2.3394582	0.01931173	0.07462986
ENSG000001	7641.62302	-0.1673315	0.05595567	-2.9904294	0.00278586	0.01993875
ENSG000001	543.403961	0.96398542	0.1965538	4.90443549	9.37E-07	3.98E-05
ENSG000001	931.642355	0.43951766	0.21143764	2.07871055	0.03764396	0.11652786
ENSG000001	827.113866	-0.1816778	0.08029597	-2.2626016	0.02366026	0.08595754
ENSG000001	1355.32136	0.26358739	0.0731819	3.60181106	0.00031601	0.00414859
ENSG000001	3169.90028	0.07183899	0.0358367	2.00462077	0.0450036	0.13142014
ENSG000001	273.168165	-0.1583166	0.07360912	-2.1507749	0.03149397	0.10377948
ENSG000001	263.717251	0.37792257	0.09741286	3.87959641	0.00010463	0.0018709
ENSG000001	548.234198	-0.3289307	0.15607337	-2.1075388	0.0350709	0.1114665
ENSG000001	123.2235	0.24237741	0.09009477	2.69024965	0.00713986	0.03813429
ENSG000001	3507.9786	0.34317151	0.12643419	2.71423023	0.006643	0.0361612
ENSG000001	13562.7042	0.24590857	0.10274174	2.39346313	0.01669016	0.06794154
ENSG000001	10102.7769	0.39570958	0.16162338	2.44834367	0.01435147	0.06108196
ENSG000001	573.855115	-0.1749957	0.08421088	-2.0780657	0.03770331	0.11668953
ENSG000001	3108.66748	0.28096032	0.09904769	2.83661643	0.00455944	0.02804241
ENSG000001	584.817178	-0.357443	0.13001851	-2.7491699	0.00597464	0.03359609

ENSG000001	11494.3526	0.89091649	0.1440093	6.18652051	6.15E-10	7.76E-08
ENSG000001	1732.54015	-0.2077826	0.08791822	-2.3633619	0.01810997	0.071523
ENSG000001	5158.46137	0.48971071	0.11321931	4.32532867	1.52E-05	0.00039251
ENSG000001	5566.31246	-0.203398	0.11187074	-1.8181518	0.06904093	0.17617476
ENSG000001	14451.1703	0.37333313	0.15726422	2.37392281	0.01760023	0.07028001
ENSG000001	249.411778	0.2343599	0.10136958	2.31193525	0.02078125	0.07874975
ENSG000001	68.0749103	0.97459209	0.21476357	4.53797669	5.68E-06	0.00017763
ENSG000001	620.733619	0.41476054	0.11672014	3.55346163	0.0003802	0.00481769
ENSG000001	34.1306992	0.7271254	0.2495856	2.91333074	0.00357596	0.02371005
ENSG000001	1287.53109	0.1684644	0.08224213	2.04839534	0.04052128	0.12229475
ENSG000001	1330.86805	-0.1637838	0.06963661	-2.3519789	0.01867383	0.07300784
ENSG000001	2064.17361	0.3184985	0.1047591	3.04029421	0.00236347	0.01775881
ENSG000001	18398.0391	0.24906151	0.12896052	1.93130042	0.05344591	0.1478577
ENSG000001	1655.06284	0.25172196	0.12562673	2.00372929	0.04509907	0.1315998
ENSG000001	360.755517	-0.5557208	0.19221831	-2.8910918	0.00383906	0.02485658
ENSG000001	280.489789	-0.2717137	0.12502701	-2.1732402	0.02976224	0.09992046
ENSG000001	2986.54046	-0.2349135	0.08032854	-2.9244085	0.00345111	0.02315385
ENSG000001	482.828651	-0.2712618	0.07084949	-3.8287045	0.00012882	0.00219341
ENSG000001	46859.436	-0.3396743	0.12360338	-2.7480991	0.00599419	0.03364831
ENSG000001	660.513511	0.17991515	0.06709461	2.68151414	0.00732898	0.03877809
ENSG000001	3610.21934	-0.1395327	0.04279647	-3.2603781	0.00111264	0.0104953
ENSG000001	2133.19022	-0.1206105	0.06390057	-1.8874718	0.05909689	0.15875029
ENSG000001	999.849658	-0.2723065	0.14606862	-1.8642368	0.06228845	0.16436541
ENSG000001	1922.57318	-0.2180125	0.06574482	-3.3160402	0.00091303	0.0091286
ENSG000001	495.385519	-0.3940844	0.18681979	-2.1094358	0.03490698	0.11118755
ENSG000001	331.209956	-0.2697878	0.10532588	-2.5614576	0.0104234	0.04911277
ENSG000001	11433.0995	0.51439822	0.24593884	2.09156968	0.03647703	0.1140642
ENSG000001	1420.16245	-0.2408073	0.08029181	-2.9991521	0.00270732	0.01951567
ENSG000001	947.403226	-0.1604216	0.09009735	-1.7805361	0.07498827	0.18590607
ENSG000001	3683.7217	-0.1463492	0.06841852	-2.1390292	0.03243331	0.10587476
ENSG000001	284.118571	-0.3871184	0.19310362	-2.0047187	0.04499312	0.13142014
ENSG000001	2552.69476	0.11525401	0.04062849	2.83677796	0.00455713	0.02803874
ENSG000001	2039.18576	0.09470053	0.04526148	2.09229844	0.03641183	0.11396472
ENSG000001	4908.4081	0.13398555	0.05500736	2.43577505	0.01485992	0.06256837
ENSG000001	1734.66259	0.32497458	0.08514289	3.81681394	0.00013519	0.00227816
ENSG000001	372.468852	0.40597851	0.10994623	3.69251859	0.00022204	0.00322771
ENSG000001	485.035652	0.6936903	0.28099341	2.46870665	0.01356023	0.05900933
ENSG000001	206.863696	-0.2027763	0.09340403	-2.1709589	0.02993428	0.10031317
ENSG000001	5024.94089	0.19499794	0.09612389	2.02861046	0.04249798	0.1261933
ENSG000001	3044.08774	0.12370005	0.06759621	1.82998505	0.06725217	0.17311364
ENSG000001	1477.82536	0.92196133	0.1751608	5.26351405	1.41E-07	8.49E-06
ENSG000001	242.912769	0.3552311	0.14486234	2.45219781	0.01419866	0.06067641
ENSG000001	2467.24299	0.21056193	0.06189148	3.40211498	0.00066866	0.00734367

ENSG000001	705.397168	0.67651895	0.14152277	4.78028345	1.75E-06	6.55E-05
ENSG000001	389.397655	-0.5744158	0.16421493	-3.4979509	0.00046885	0.00560736
ENSG000001	1473.61568	-0.1727141	0.08978218	-1.9237014	0.05439202	0.14976757
ENSG000001	591.854569	-0.2445719	0.08416746	-2.9057768	0.00366343	0.02413534
ENSG000001	2921.99811	-0.1868388	0.09536055	-1.9592878	0.05007909	0.14172365
ENSG000001	638.367001	0.62493083	0.19035895	3.28290761	0.00102742	0.0099449
ENSG000001	12.418846	1.30636152	0.37820179	3.45413891	0.00055205	0.00632565
ENSG000001	3685.30291	0.20827966	0.06105625	3.41127519	0.0006466	0.00716369
ENSG000001	5545.13518	0.25429879	0.10844333	2.34499233	0.01902747	0.07393209
ENSG000001	6597.1988	0.22484546	0.07034631	3.19626527	0.00139219	0.01230606
ENSG000001	139.022862	-0.527066	0.22656163	-2.3263692	0.01999886	0.07664084
ENSG000001	860.779334	0.58037622	0.11759572	4.9353515	8.00E-07	3.47E-05
ENSG000001	2398.53732	0.23177838	0.09241435	2.50803464	0.01214048	0.05485389
ENSG000001	555.114611	0.17323675	0.09991934	1.73376602	0.08295961	0.19910549
ENSG000001	2190.18199	-0.2254463	0.09645555	-2.3373082	0.01942317	0.0749162
ENSG000001	1036.10231	0.17005826	0.07246793	2.3466694	0.01894205	0.07373999
ENSG000001	96.4067522	2.26562645	0.31676807	7.15231955	8.53E-13	3.18E-10
ENSG000001	434.148041	0.35938948	0.19764311	1.81837592	0.0690067	0.1761368
ENSG000001	94.2028926	0.92303414	0.18165303	5.08130322	3.75E-07	1.90E-05
ENSG000001	482.11986	-0.2030945	0.08078019	-2.5141617	0.01193157	0.05417891
ENSG000001	513.990224	0.50142509	0.19665664	2.54974909	0.01078005	0.05041655
ENSG000001	3422.84455	-0.2795094	0.05761443	-4.8513786	1.23E-06	4.98E-05
ENSG000001	1138.56663	0.68670643	0.35935891	1.91092084	0.05601475	0.15254508
ENSG000001	212.865503	1.73631467	0.47736945	3.63725552	0.00027556	0.00375277
ENSG000001	2568.91127	0.4775048	0.17390867	2.74572167	0.0060378	0.03382362
ENSG000001	595.286465	1.4222111	0.29486408	4.82327681	1.41E-06	5.55E-05
ENSG000001	557.416903	0.44711312	0.20565122	2.17413306	0.02969514	0.09979735
ENSG000001	137.48805	-0.5293881	0.2714369	-1.9503175	0.05113829	0.14375229
ENSG000001	2385.99487	0.23283844	0.11908071	1.95529937	0.05054775	0.1427297
ENSG000001	170.543446	-0.6780113	0.36159484	-1.8750581	0.06078473	0.16174736
ENSG000001	3836.36802	-0.3928065	0.11415765	-3.4409128	0.00057976	0.00655603
ENSG000001	318.515418	0.28481735	0.16025616	1.77726302	0.07552498	0.18676223
ENSG000001	140.377009	0.31019955	0.16680175	1.85968997	0.0629294	0.16538763
ENSG000001	410.11598	0.22871463	0.08454518	2.7052357	0.00682559	0.03688835
ENSG000001	105.172077	1.30323987	0.25706309	5.06972773	3.98E-07	1.98E-05
ENSG000001	2020.34735	-0.0797715	0.04549976	-1.75323	0.07956253	0.19335806
ENSG000001	362.141894	0.19446737	0.0894869	2.17313788	0.02976994	0.09992584
ENSG000001	572.565079	0.27200417	0.11228625	2.42241749	0.01541763	0.06431005
ENSG000001	1919.63955	-0.2390834	0.08668052	-2.7582133	0.00581183	0.03297457
ENSG000001	1017.20649	0.24166748	0.07225453	3.34466892	0.00082381	0.00849329
ENSG000001	511.440004	1.42466299	0.24221697	5.88176368	4.06E-09	4.06E-07
ENSG000001	664.38091	0.15698428	0.09020418	1.74032155	0.08180257	0.19710753
ENSG000001	1419.38173	0.27719344	0.11230072	2.46831408	0.01357512	0.05901958

ENSG000001	16190.3767	0.16329242	0.07671045	2.12868538	0.0332803	0.10773881
ENSG000001	71.9562597	1.09463066	0.25369273	4.31478926	1.60E-05	0.00040676
ENSG000001	391.234522	0.26463745	0.0876738	3.01843271	0.00254086	0.01863258
ENSG000001	4742.53873	0.18830414	0.06767255	2.78257805	0.00539289	0.031456
ENSG000001	1600.59209	-0.1514217	0.05255729	-2.8810791	0.00396316	0.02541906
ENSG000001	5135.91253	0.61163871	0.16768452	3.64755626	0.00026475	0.00365101
ENSG000001	123.188659	-0.6171264	0.30255099	-2.0397435	0.04137588	0.12395112
ENSG000001	10644.6793	0.56979267	0.09407716	6.05665282	1.39E-09	1.64E-07
ENSG000001	4348.8186	1.68517315	0.42224093	3.99102269	6.58E-05	0.00128328
ENSG000001	6283.20816	0.38104037	0.21516024	1.77096086	0.07656721	0.18836798
ENSG000001	31.8040433	1.10771747	0.39519565	2.8029597	0.0050636	0.03024694
ENSG000001	2789.88532	0.15578862	0.07691059	2.02558091	0.04280775	0.12676877
ENSG000001	9142.42068	0.40410153	0.15869501	2.54640344	0.01088393	0.05069995
ENSG000001	1481.31692	1.49116899	0.3557681	4.19140722	2.77E-05	0.00065431
ENSG000001	1247.73327	-0.2060782	0.08440924	-2.4414179	0.01462972	0.06201744
ENSG000001	1659.55176	-0.2939951	0.1686348	-1.7433833	0.08126668	0.19607559
ENSG000001	1850.64756	0.24033103	0.07608433	3.15874539	0.0015845	0.01352111
ENSG000001	1999.90359	-0.3284185	0.13095478	-2.5078768	0.0121459	0.05485389
ENSG000001	918.596303	0.25142092	0.14171288	1.77415715	0.07603716	0.18747406
ENSG000001	578.160779	0.19737493	0.07705541	2.56146766	0.01042309	0.04911277
ENSG000001	142.807278	0.40636679	0.10595949	3.83511468	0.00012551	0.00215106
ENSG000001	1796.24904	0.10165179	0.05594267	1.81707071	0.06920629	0.17645397
ENSG000001	8302.07744	-0.1603992	0.05119016	-3.1333997	0.00172794	0.01430238
ENSG000001	2163.27239	-0.3731554	0.07644257	-4.8815122	1.05E-06	4.37E-05
ENSG000001	43.349784	0.86239943	0.42532998	2.02760084	0.042601	0.12642286
ENSG000001	229.10021	0.97936456	0.30882754	3.17123453	0.00151793	0.01313426
ENSG000001	5629.19737	-0.121416	0.06304534	-1.9258514	0.05412293	0.14922712
ENSG000001	910.613596	0.17702356	0.07019497	2.52188386	0.01167282	0.05335916
ENSG000001	7014.71081	0.82507966	0.21608808	3.81825622	0.0001344	0.00227124
ENSG000001	158.946223	0.28250715	0.11733873	2.40762065	0.01605686	0.06620173
ENSG000001	127.330924	1.57702688	0.29600956	5.32762142	9.95E-08	6.35E-06
ENSG000001	66212.484	0.999828	0.24242085	4.12434823	3.72E-05	0.00082604
ENSG000001	2042.58836	-0.1209707	0.06380136	-1.8960526	0.05795309	0.15644663
ENSG000001	2560.97186	0.42796768	0.11225512	3.8124559	0.00013759	0.00229981
ENSG000001	5510.60066	0.64709928	0.17553067	3.68653102	0.00022733	0.00327553
ENSG000001	616.614387	-0.2212809	0.08746548	-2.5299225	0.01140877	0.05247395
ENSG000001	2337.16796	0.4141578	0.11116985	3.7254507	0.00019497	0.00297107
ENSG000001	226.469281	-0.2602316	0.13667491	-1.9040187	0.05690774	0.15438721
ENSG000001	949.713205	-0.2062109	0.09566265	-2.1556053	0.0311145	0.10296356
ENSG000001	1649.54547	-0.6552559	0.18761702	-3.4925183	0.00047849	0.00570188
ENSG000001	14875.503	0.27065959	0.14518039	1.8642986	0.06227978	0.16436541
ENSG000001	11124.9702	-0.0992126	0.03695798	-2.6844716	0.00726446	0.03856906
ENSG000001	824.248972	0.15111852	0.0703146	2.149177	0.03162037	0.10409139

ENSG000001	1464.46576	1.52225737	0.3811279	3.99408538	6.49E-05	0.00127076
ENSG000001	93.788774	0.39801206	0.18486123	2.15303151	0.0313162	0.10344314
ENSG000001	1503.44941	0.35723189	0.16871727	2.11734042	0.03423097	0.10969082
ENSG000001	376.793531	0.40365973	0.13478822	2.99477006	0.00274652	0.01971745
ENSG000001	6199.9627	0.21575555	0.12111934	1.78134685	0.07485581	0.18566187
ENSG000001	15083.4137	0.28054949	0.12833803	2.18601992	0.02881415	0.0976976
ENSG000001	78.8019008	1.72179242	0.27899266	6.17146129	6.77E-10	8.47E-08
ENSG000001	10584.014	1.01671169	0.15950638	6.37411303	1.84E-10	2.92E-08
ENSG000001	48.6577876	0.70036999	0.2706568	2.5876682	0.0096628	0.04669642
ENSG000001	1338.03507	0.16093222	0.05473786	2.94005333	0.00328156	0.02240121
ENSG000001	43.7285286	-1.1947216	0.29203474	-4.0910256	4.29E-05	0.00092173
ENSG000001	1616.41038	0.22370181	0.08914282	2.50947649	0.01209103	0.05467638
ENSG000001	8989.36665	0.24968314	0.0914492	2.73029321	0.0063278	0.03494677
ENSG000001	24.6232429	1.49708014	0.42934433	3.48689856	0.00048866	0.0057852
ENSG000001	8972.26241	0.34060373	0.12360707	2.75553601	0.00585961	0.03313102
ENSG000001	23981.4447	0.30386025	0.16306918	1.86338245	0.06240848	0.16444027
ENSG000001	1575.61176	0.4993159	0.20343497	2.45442506	0.01411101	0.0604285
ENSG000001	1038.4402	-0.1743103	0.08477184	-2.056229	0.03976045	0.12084375
ENSG000001	1643.19439	-0.1504922	0.08434736	-1.7841959	0.07439183	0.18507099
ENSG000001	2836.96676	0.20229405	0.10440423	1.93760399	0.05267155	0.14655617
ENSG000001	2365.71722	0.28324642	0.15084004	1.87779338	0.06040944	0.16106236
ENSG000001	1143.44096	0.32890832	0.08624287	3.81374517	0.00013688	0.00229251
ENSG000001	274.077195	0.5457752	0.16975227	3.21512751	0.00130387	0.01177933
ENSG000001	1647.62117	-0.3780503	0.10923806	-3.4607926	0.00053859	0.00623674
ENSG000001	3501.37071	-0.1635841	0.05141966	-3.1813527	0.00146589	0.01278767
ENSG000001	2401.85657	0.30306775	0.1747513	1.73428036	0.08286835	0.19891558
ENSG000001	18404.7265	0.35562035	0.18169994	1.95718472	0.05032575	0.14227438
ENSG000001	206.842193	-0.3064803	0.11736491	-2.6113452	0.00901868	0.04466304
ENSG000001	424.859399	-0.1485021	0.06875578	-2.1598493	0.03078434	0.10220472
ENSG000001	1167.41347	0.32421005	0.14840803	2.18458558	0.02891925	0.09799316
ENSG000001	354.619212	-0.2009851	0.07211232	-2.7871122	0.00531801	0.03120798
ENSG000001	925.551212	-0.2570474	0.13877619	-1.8522445	0.06399073	0.16723875
ENSG000001	1658.43055	0.35349581	0.11943689	2.95968695	0.00307952	0.02138706
ENSG000001	1982.19674	0.19404862	0.08171264	2.37476868	0.01755995	0.070219
ENSG000001	66.6975344	0.33578791	0.17605511	1.90728864	0.05648322	0.15343859
ENSG000001	1313.05097	0.21304193	0.0893608	2.38406466	0.01712259	0.06908444
ENSG000001	825.732768	0.13675536	0.05492708	2.48976197	0.01278287	0.05681775
ENSG000001	885.62567	0.22911241	0.0695625	3.29361953	0.00098906	0.00967594
ENSG000001	367.359195	0.2464488	0.10964256	2.24774754	0.02459229	0.08806286
ENSG000001	336.720944	-0.3697544	0.10591197	-3.4911489	0.00048095	0.00571457
ENSG000001	1168.25612	0.27876925	0.07373993	3.78043832	0.00015655	0.00254158
ENSG000001	6168.63916	0.38450633	0.19289917	1.99330212	0.04622837	0.1339471
ENSG000001	175.00095	1.09262883	0.23094319	4.73115852	2.23E-06	7.95E-05

ENSG000001	1825.98537	-0.2152572	0.10504414	-2.0492076	0.04044182	0.12214489
ENSG000001	1186.21725	-0.3409239	0.14538268	-2.3450104	0.01902655	0.07393209
ENSG000001	7078.94487	0.21081353	0.10366058	2.0336904	0.04198282	0.12507128
ENSG000001	134.860783	1.27694316	0.22753679	5.61202955	2.00E-08	1.55E-06
ENSG000001	1030.96493	-0.2193938	0.09254442	-2.3706861	0.0177551	0.07062843
ENSG000001	222.675538	-0.3670984	0.14622266	-2.5105442	0.01205452	0.05453865
ENSG000001	316.789675	0.17034272	0.09218201	1.84789546	0.06461747	0.16848734
ENSG000001	415.275434	0.4319932	0.12381117	3.48912934	0.0004846	0.00574543
ENSG000001	5036.51765	-0.2051263	0.07812255	-2.6256996	0.00864711	0.04337763
ENSG000001	3366.19628	-0.3152443	0.11338469	-2.7803077	0.00543074	0.03158846
ENSG000001	2924.04898	0.10512389	0.05595005	1.87888834	0.06025974	0.16084632
ENSG000001	70.8718067	0.89987889	0.27128708	3.31707242	0.00090966	0.00910604
ENSG000001	20093.1397	0.84666704	0.2689199	3.14839866	0.00164168	0.01382566
ENSG000001	268.573182	-0.2118198	0.10109011	-2.0953565	0.03613931	0.11358565
ENSG000001	131.366144	0.31980616	0.1655115	1.93222922	0.05333122	0.14771487
ENSG000001	624.754375	-0.4273613	0.12443908	-3.4343012	0.00059408	0.00667662
ENSG000001	446.884084	-0.2876741	0.11750234	-2.4482414	0.01435555	0.06108196
ENSG000001	37.9736859	1.71110621	0.36335373	4.7092023	2.49E-06	8.73E-05
ENSG000001	435.418111	-0.3278837	0.13339737	-2.457947	0.01397338	0.06004233
ENSG000001	6001.32828	0.26529194	0.10699488	2.47948255	0.01315732	0.05785479
ENSG000001	5.16695353	0.85965506	0.43289201	1.98584182	0.04705089	0.13570685
ENSG000001	766.629715	-0.1717019	0.08710932	-1.9711083	0.04871149	0.13895656
ENSG000001	727.828052	0.12362647	0.06919241	1.78670574	0.07398506	0.18439475
ENSG000001	75.6355847	-0.6567988	0.31741176	-2.0692326	0.03852426	0.11828797
ENSG000001	810.633928	0.30600057	0.10953818	2.79355166	0.00521327	0.03084799
ENSG000001	572.148073	-0.2912183	0.14341222	-2.0306377	0.04229176	0.12574033
ENSG000001	1415.94663	0.37701588	0.15255643	2.47132079	0.0134615	0.05870431
ENSG000001	531.849724	1.60851809	0.27346334	5.88202454	4.05E-09	4.06E-07
ENSG000001	2105.59	-0.1149779	0.06255465	-1.838039	0.06605667	0.17092175
ENSG000001	1198.11367	0.70653615	0.20188311	3.49972896	0.00046573	0.00557416
ENSG000001	10609.2965	0.5215357	0.16949132	3.07706446	0.0020905	0.01626088
ENSG000001	209.339973	-0.2068414	0.10228828	-2.0221419	0.0431617	0.12758651
ENSG000001	294.961936	0.94551672	0.22083417	4.28156898	1.86E-05	0.00046105
ENSG000001	1179.2538	0.20769792	0.08377862	2.47912787	0.01317041	0.05789683
ENSG000001	812.889043	0.66797939	0.15493343	4.31139597	1.62E-05	0.00041177
ENSG000001	650.126589	-0.1649269	0.07764655	-2.1240729	0.03366405	0.10861315
ENSG000001	239.67553	-0.295784	0.10538023	-2.806826	0.00500323	0.02997366
ENSG000001	540.786659	0.57781621	0.16860391	3.42706296	0.00061015	0.00682462
ENSG000001	274.594983	0.67723349	0.21594899	3.13608079	0.00171222	0.0142596
ENSG000001	344.760511	-0.2686206	0.09202218	-2.9190853	0.0035106	0.02340874
ENSG000001	2533.56673	0.69830015	0.16215875	4.30627485	1.66E-05	0.00041882
ENSG000001	5303.09546	0.15356939	0.07369899	2.0837381	0.037184	0.11554027
ENSG000001	5497.41125	-0.177612	0.06842248	-2.5958143	0.0094367	0.04606539

ENSG000001	6158.73759	0.32636727	0.11727809	2.78284944	0.00538838	0.03144245
ENSG000001	1106.45545	0.34212269	0.12850818	2.66226398	0.0077617	0.04021124
ENSG000001	2063.78905	0.1791576	0.08455408	2.11885225	0.03410296	0.1094097
ENSG000001	1996.07086	0.94459055	0.17089633	5.52727213	3.25E-08	2.38E-06
ENSG000001	619.17769	0.66306927	0.1508309	4.3961102	1.10E-05	0.00030577
ENSG000001	2207.46575	0.23499214	0.08135321	2.88854167	0.00387033	0.02499479
ENSG000001	6588.70498	-0.1422838	0.07331247	-1.9407851	0.05228435	0.14594934
ENSG000001	6831.65026	0.82308373	0.13562777	6.06869607	1.29E-09	1.56E-07
ENSG000001	3417.42853	0.20675058	0.09744951	2.12161737	0.03386988	0.10900186
ENSG000001	2062.53961	-0.2203307	0.10758244	-2.0480172	0.04055831	0.12233897
ENSG000001	6427.76673	0.29911196	0.15657213	1.9103781	0.05608455	0.15263978
ENSG000001	94.3202819	1.93655157	0.3967407	4.88115172	1.05E-06	4.37E-05
ENSG000001	21415.5326	0.43766015	0.15012406	2.91532305	0.00355321	0.02359737
ENSG000001	193.40267	-0.2423809	0.10629453	-2.2802768	0.02259128	0.08303725
ENSG000001	49564.6976	0.6513419	0.23363014	2.78791894	0.00530478	0.03116535
ENSG000001	211.505893	0.18063997	0.09588391	1.88394457	0.05957247	0.15962157
ENSG000001	8658.77994	0.68642299	0.26363698	2.60366729	0.00922322	0.04541538
ENSG000001	417.878779	0.29865145	0.11471627	2.60339232	0.00923063	0.04543278
ENSG000001	2767.47659	-0.1318083	0.07577764	-1.7394091	0.08196282	0.19734866
ENSG000001	218.505544	0.17554791	0.09722544	1.80557579	0.07098463	0.1793152
ENSG000001	9049.46978	0.21710496	0.11522982	1.88410401	0.05955091	0.15962157
ENSG000001	133.692617	0.34994928	0.20016674	1.74828884	0.08041402	0.19467721
ENSG000001	996.017655	0.15647954	0.06698576	2.33601225	0.0194906	0.07512633
ENSG000001	230.415175	0.57415827	0.11855371	4.84302231	1.28E-06	5.13E-05
ENSG000001	2669.84209	0.72977347	0.12449168	5.86202598	4.57E-09	4.49E-07
ENSG000001	926.378687	0.20404219	0.09828489	2.076028	0.03789136	0.11707267
ENSG000001	1139.16333	0.39033682	0.13556057	2.87942737	0.00398398	0.02550774
ENSG000001	13105.2311	0.40734928	0.12922829	3.1521679	0.00162063	0.01372597
ENSG000001	1478.47949	0.24434392	0.08443632	2.89382486	0.0038058	0.02477323
ENSG000001	338.99062	-0.3287784	0.14648092	-2.2445137	0.02479937	0.08847467
ENSG000001	286.80834	-0.2443542	0.13472327	-1.8137489	0.0697164	0.17723098
ENSG000001	6056.29445	-0.2103995	0.11899038	-1.7682057	0.07702652	0.18903847
ENSG000001	209.412568	0.16532305	0.0815797	2.02652201	0.04271132	0.12655177
ENSG000001	282.237192	-0.18329	0.10104158	-1.8140056	0.06967686	0.17715792
ENSG000001	2593.9155	0.23350138	0.10437032	2.2372393	0.0252707	0.08961153
ENSG000001	2969.26229	-0.1818017	0.07054909	-2.576953	0.00996755	0.04773304
ENSG000001	4536.03379	0.22099678	0.0954292	2.31581922	0.02056814	0.07819518
ENSG000001	1161.23248	0.10890999	0.05046811	2.15799637	0.03092811	0.10255375
ENSG000001	1957.33237	-0.153149	0.08264204	-1.8531608	0.06385932	0.16702843
ENSG000001	5083.63905	0.20787518	0.05552887	3.74355119	0.00018144	0.00281194
ENSG000001	407.73726	0.21171477	0.10533809	2.00985962	0.04444605	0.13016393
ENSG000001	5245.47271	0.30836311	0.09471907	3.25555464	0.00113171	0.01060506
ENSG000001	330.346532	0.15352999	0.08643067	1.77633684	0.07567741	0.18704892

ENSG000001	695.470174	0.27895758	0.1302663	2.14144088	0.0322385	0.10549086
ENSG000001	22589.5975	0.20549338	0.09780377	2.10107827	0.0356341	0.11253703
ENSG000001	9446.17014	-0.1234455	0.05630116	-2.1925934	0.02833669	0.0965731
ENSG000001	213.097851	-0.396963	0.12175798	-3.2602631	0.00111309	0.0104953
ENSG000001	169.140928	0.50580143	0.16321796	3.09893248	0.00194219	0.0154969
ENSG000001	1170.96747	-0.3184856	0.17244896	-1.8468396	0.06477039	0.16881896
ENSG000001	1205.25683	0.24247035	0.06617298	3.66418946	0.00024812	0.00349525
ENSG000001	73.2115085	0.81122344	0.21924406	3.70009319	0.00021552	0.00317598
ENSG000001	6140.18517	0.25601048	0.0970427	2.63812201	0.00833666	0.04237342
ENSG000001	6622.19596	0.3152815	0.14323369	2.20116867	0.02772409	0.09536225
ENSG000001	2700.50826	-0.1165992	0.05282446	-2.2072956	0.02729341	0.09429626
ENSG000001	3314.40976	0.30857163	0.13740212	2.24575596	0.02471964	0.08831247
ENSG000001	362.600288	0.39272043	0.18523804	2.12008528	0.03399885	0.1092668
ENSG000001	14254.5665	0.15081025	0.08618263	1.74989156	0.08013703	0.19420734
ENSG000001	1173.62376	0.10787166	0.05880637	1.83435334	0.06660156	0.17197887
ENSG000001	9647.18565	0.2775555	0.09000736	3.08369775	0.00204445	0.01602432
ENSG000001	2443.00387	-0.2775427	0.15080635	-1.8403916	0.06571077	0.17034933
ENSG000001	11089.5958	0.36463452	0.16613035	2.19486995	0.02817293	0.09623989
ENSG000001	2131.08106	0.18381436	0.06353202	2.89325547	0.00381271	0.02477884
ENSG000001	1926.96244	-0.3519328	0.19787211	-1.7785871	0.07530748	0.18645199
ENSG000001	597.680486	0.1034965	0.05681258	1.82171781	0.06849781	0.17521976
ENSG000001	167.501392	-0.3713037	0.1361654	-2.7268583	0.00639405	0.03521597
ENSG000001	60733.2911	1.19610529	0.18431244	6.48955268	8.61E-11	1.52E-08
ENSG000001	2339.91217	0.54152055	0.12524424	4.32371634	1.53E-05	0.00039431
ENSG000001	4131.07238	-0.2482867	0.11290083	-2.199157	0.02786676	0.09567237
ENSG000001	165.28493	0.35299392	0.13278246	2.65843798	0.00785038	0.0405043
ENSG000001	156.920195	0.75803551	0.25154156	3.01355971	0.00258202	0.01884882
ENSG000001	4223.02514	-0.2838839	0.06895556	-4.1169117	3.84E-05	0.00084626
ENSG000001	501.180082	0.1952986	0.09982874	1.95633641	0.05042554	0.14250733
ENSG000001	4671.89238	0.23068691	0.10239194	2.25297919	0.02426046	0.08733233
ENSG000001	1414.46091	-0.2829521	0.14750212	-1.9182918	0.05507402	0.151062
ENSG000001	954.269074	0.17286321	0.08341134	2.07241869	0.03822641	0.11766834
ENSG000001	1641.93942	-0.1919785	0.04841855	-3.9649782	7.34E-05	0.001411
ENSG000001	53.7424283	2.18637715	0.4418515	4.94821714	7.49E-07	3.29E-05
ENSG000001	4121.63853	0.18389032	0.09417581	1.952628	0.05086369	0.14325179
ENSG000001	2947.94767	0.53138613	0.15101201	3.51883351	0.00043345	0.00530788
ENSG000001	272.69562	3.43431731	0.51943475	6.6116433	3.80E-11	7.33E-09
ENSG000001	575.049774	-0.1778797	0.08639743	-2.0588542	0.03950821	0.12034481
ENSG000001	132.780904	0.43363037	0.1777934	2.43895658	0.01472974	0.06224204
ENSG000001	736.295263	-0.2174542	0.06789548	-3.2027791	0.00136108	0.0121233
ENSG000001	302.79281	0.19762213	0.07694449	2.56837284	0.01021772	0.0485623
ENSG000001	4256.87748	-0.1775237	0.05535372	-3.207078	0.00134091	0.01202237
ENSG000001	2757.60971	-0.1106464	0.05534153	-1.9993379	0.04557181	0.13256005

ENSG000001	784.23896	-0.3939267	0.22646616	-1.7394506	0.08195554	0.19734866
ENSG000001	2428.88539	0.31677431	0.11042241	2.86875008	0.00412097	0.02617025
ENSG000001	637.6627	-0.2150054	0.0780515	-2.7546611	0.00587529	0.03319683
ENSG000001	1736.97431	0.15516129	0.05098746	3.04312655	0.00234134	0.01762673
ENSG000001	1310.65857	-0.3318855	0.09961295	-3.3317503	0.00086302	0.00880578
ENSG000001	826.139539	0.18455535	0.09222343	2.00117638	0.04537339	0.13213171
ENSG000001	112.796391	0.3578041	0.13600877	2.63074288	0.00851985	0.04307737
ENSG000001	1451.89393	0.4612101	0.15528052	2.97017363	0.00297631	0.02082912
ENSG000001	131.519577	0.40113826	0.15581759	2.57440943	0.01004114	0.04797335
ENSG000001	10743.3797	0.28127403	0.12680236	2.21820812	0.02654065	0.09251424
ENSG000001	2430.07882	0.52236942	0.11254177	4.64156027	3.46E-06	0.00011571
ENSG000001	51.7654607	-0.6956505	0.38996818	-1.7838647	0.07444566	0.18509256
ENSG000001	374.790272	-0.2289202	0.08201016	-2.7913637	0.00524865	0.03097986
ENSG000001	95669.9588	0.95844023	0.16196108	5.91771929	3.26E-09	3.39E-07
ENSG000001	9535.87047	0.86076106	0.20007918	4.30210213	1.69E-05	0.00042483
ENSG000001	1646.42866	0.45036601	0.1809966	2.48825681	0.0128371	0.05696615
ENSG000001	992.0122	-0.1611985	0.06090223	-2.6468409	0.00812476	0.04150605
ENSG000001	1007.5884	0.37955357	0.17835807	2.12804256	0.03333355	0.10784734
ENSG000001	225.62175	0.19022077	0.07152939	2.65933729	0.00782945	0.04044837
ENSG000001	1940.68687	0.57111422	0.20445537	2.79334416	0.00521662	0.03084799
ENSG000001	2091.44462	-0.2425505	0.05582421	-4.3448974	1.39E-05	0.00036792
ENSG000001	1715.41703	0.48988315	0.12979934	3.77415743	0.00016055	0.00259136
ENSG000001	1952.2291	0.3989965	0.16242072	2.45656151	0.01402738	0.06016399
ENSG000001	547.023477	0.39277364	0.11775565	3.33549718	0.00085147	0.00870964
ENSG000001	497.582208	0.789783	0.19212918	4.1106874	3.94E-05	0.00085787
ENSG000001	4144.21862	0.1150357	0.05113561	2.24962028	0.02447306	0.08784693
ENSG000001	1994.3977	-0.231722	0.11533437	-2.0091321	0.04452312	0.13034202
ENSG000001	151.583977	-0.4691076	0.15987058	-2.9342959	0.00334305	0.02265126
ENSG000001	8318.80953	-0.2734926	0.09245204	-2.9582106	0.00309431	0.02145342
ENSG000001	4224.89864	-0.1795214	0.10249439	-1.7515247	0.07985557	0.19378301
ENSG000001	842.347477	-0.2436924	0.11778317	-2.0689917	0.03854687	0.11831767
ENSG000001	160.264902	-0.19927	0.08846964	-2.2524113	0.02429629	0.08736541
ENSG000001	130.833232	-0.9871101	0.35738469	-2.762038	0.00574418	0.03269257
ENSG000001	105.893692	1.12704149	0.25164518	4.47869297	7.51E-06	0.00022431
ENSG000001	5619.52284	-0.1900341	0.07240105	-2.6247426	0.00867145	0.04340224
ENSG000001	1213.18126	0.28543012	0.11356924	2.51326961	0.01196179	0.05425655
ENSG000001	4733.77633	-0.1293472	0.06457524	-2.0030464	0.04517231	0.13175589
ENSG000001	382.852652	0.66006731	0.20613092	3.20217508	0.00136394	0.01212827
ENSG000001	39318.0862	-0.2102839	0.10877118	-1.9332683	0.05320316	0.14750967
ENSG000001	64.144885	0.9435645	0.33710658	2.79900943	0.00512596	0.03048006
ENSG000001	5568.62446	0.18625166	0.08599102	2.16594325	0.03031552	0.10122198
ENSG000001	229.827127	-0.3801109	0.21554876	-1.7634566	0.07782351	0.19025975
ENSG000001	5007.50589	0.25098267	0.1143715	2.19445111	0.02820299	0.09632254

ENSG000001	63.0227391	0.63311815	0.28701181	2.20589584	0.02739129	0.09446237
ENSG000001	96387.6179	0.4677961	0.15215464	3.07447797	0.00210871	0.01637924
ENSG000001	3190.25755	-0.1807786	0.09137092	-1.9785132	0.04787084	0.13717898
ENSG000001	2036.89811	0.2499715	0.07155716	3.49331195	0.00047707	0.00568909
ENSG000001	605.710699	0.22600702	0.08131712	2.77932898	0.00544713	0.03163889
ENSG000001	3715.36585	-0.4483709	0.19357644	-2.3162475	0.02054476	0.0781244
ENSG000001	3490.79284	0.55052169	0.20555274	2.67825031	0.00740079	0.0390354
ENSG000001	499.817045	0.18057697	0.05620242	3.2129751	0.00131368	0.01184187
ENSG000001	896.516126	0.17195313	0.09907125	1.73565121	0.08262553	0.19850707
ENSG000001	517.157407	0.16907787	0.07195652	2.34972271	0.0187874	0.07332945
ENSG000001	184.241276	-0.4424203	0.17113938	-2.585146	0.00973378	0.04695216
ENSG000001	713.215815	-0.2582384	0.09037623	-2.8573709	0.00427166	0.02682592
ENSG000001	3517.39593	0.18056908	0.09646822	1.87179863	0.06123447	0.16244323
ENSG000001	2453.09276	0.28591879	0.13734945	2.08168865	0.03737092	0.11601116
ENSG000001	8991.83705	-0.1693354	0.07605662	-2.2264382	0.02598484	0.09121675
ENSG000001	3307.00361	0.39873342	0.15506545	2.5713879	0.01012918	0.04821137
ENSG000001	132.81709	1.21875561	0.2302128	5.29403938	1.20E-07	7.32E-06
ENSG000001	13167.8463	0.32559304	0.10328809	3.15228066	0.00162	0.01372597
ENSG000001	445.932255	-0.3052207	0.09302575	-3.2810343	0.00103427	0.00997585
ENSG000001	420.866542	-0.3133773	0.13287745	-2.3583932	0.01835424	0.07213675
ENSG000001	5667.86261	0.41276072	0.11126182	3.70981454	0.00020741	0.00309863
ENSG000001	6052.55385	0.17171927	0.07702784	2.22931451	0.02579299	0.090874
ENSG000001	127.672862	-0.3681968	0.12327696	-2.9867442	0.00281966	0.02010169
ENSG000001	236.846454	-0.2250763	0.09186311	-2.4501273	0.01428057	0.06090932
ENSG000001	262.561153	-0.1135546	0.05775499	-1.9661432	0.04928207	0.14014536
ENSG000001	629.830142	0.62931152	0.15187714	4.14355658	3.42E-05	0.00077441
ENSG000001	850.361424	-0.2958621	0.10082831	-2.9343157	0.00334284	0.02265126
ENSG000001	1835.77559	0.19197248	0.04929689	3.89421048	9.85E-05	0.00178697
ENSG000001	1442.36313	0.22934142	0.11655282	1.96770374	0.04910214	0.13975485
ENSG000001	2378.30185	0.12664453	0.06876079	1.84181328	0.06550247	0.17002437
ENSG000001	2386.7615	-0.3364616	0.12972128	-2.5937272	0.00949418	0.04622542
ENSG000001	713.370914	0.3448694	0.14687952	2.34797473	0.0188758	0.07360441
ENSG000001	290.905327	0.36343213	0.13323077	2.72783925	0.00637507	0.0351368
ENSG000001	311.236597	0.29505079	0.12824275	2.30072095	0.02140741	0.08045319
ENSG000001	7149.58304	-0.2362128	0.06834536	-3.4561639	0.00054792	0.00628711
ENSG000001	988.385999	-0.418926	0.13160298	-3.1832561	0.00145629	0.01272063
ENSG000001	2475.9198	0.76556351	0.19141317	3.99953412	6.35E-05	0.00124648
ENSG000001	9045.56496	0.13517851	0.06238727	2.16676443	0.03025282	0.1010708
ENSG000001	7552.18479	0.39324973	0.13773689	2.85507914	0.00430261	0.0268864
ENSG000001	859.791956	0.20850033	0.07407643	2.81466488	0.00488281	0.02943912
ENSG000001	105.633598	0.88631872	0.21544123	4.1139698	3.89E-05	0.00085194
ENSG000001	6840.04383	0.35475393	0.14464099	2.45265145	0.01418077	0.06061576
ENSG000001	1459.12171	0.70208008	0.12264583	5.72445136	1.04E-08	8.82E-07

ENSG000001	168.422895	-0.1533742	0.08661283	-1.7708027	0.07659352	0.18840443
ENSG000001	481.038092	0.3813963	0.11675527	3.26663024	0.00108836	0.01033341
ENSG000001	902.247905	0.29882081	0.09449872	3.16216779	0.00156599	0.01341567
ENSG000001	2201.06464	0.1699516	0.05008611	3.39318844	0.00069084	0.00752672
ENSG000001	277.707839	0.90448098	0.15207115	5.94774871	2.72E-09	2.89E-07
ENSG000001	902.621175	-0.1992693	0.07861662	-2.5346973	0.01125445	0.05195362
ENSG000001	2456.8386	0.29539358	0.08176234	3.61283191	0.00030287	0.00401469
ENSG000001	295.297257	-0.2410165	0.0830016	-2.9037576	0.00368714	0.02421224
ENSG000001	1688.53663	0.28837933	0.12827661	2.24810524	0.02456948	0.08805809
ENSG000001	1190.55387	-0.1552959	0.06989699	-2.2217825	0.02629801	0.09198737
ENSG000001	88.5837055	0.80797263	0.18484404	4.37110469	1.24E-05	0.00033559
ENSG000001	3009.76045	0.41652148	0.16936392	2.45932832	0.01391973	0.05989025
ENSG000001	2781.77791	0.23097022	0.08882198	2.6003724	0.00931226	0.04562091
ENSG000001	10816.6008	-0.5306722	0.19249141	-2.7568617	0.0058359	0.03304258
ENSG000001	414.425663	0.74648683	0.2999039	2.48908672	0.01280717	0.0568934
ENSG000001	4655.09182	0.99529042	0.2892105	3.44140486	0.0005787	0.00654864
ENSG000001	850.74815	0.26541203	0.0874551	3.03483781	0.00240665	0.01794823
ENSG000001	1147.18382	-0.2967979	0.06576986	-4.5126724	6.40E-06	0.00019583
ENSG000001	217.918965	0.35177607	0.15920407	2.20959217	0.02713348	0.09394165
ENSG000001	2150.51607	0.42629864	0.15806263	2.69702351	0.00699623	0.03757525
ENSG000001	750.232802	0.20805513	0.11180361	1.86089818	0.06275856	0.16507091
ENSG000001	1271.83218	0.23846682	0.06939007	3.43661284	0.00058904	0.00664267
ENSG000001	1281.83418	-0.2352479	0.05700053	-4.1271183	3.67E-05	0.00081949
ENSG000001	982.766175	0.20913767	0.09267269	2.25673455	0.02402467	0.086867
ENSG000001	3643.24481	-0.2418279	0.09427752	-2.5650646	0.01031566	0.04881554
ENSG000001	554.800258	0.20855455	0.07145431	2.91871188	0.00351481	0.02340874
ENSG000001	170.500738	-0.7080926	0.23383604	-3.0281587	0.00246049	0.01823385
ENSG000001	1312.13194	0.26843198	0.08496113	3.15946796	0.00158057	0.01350531
ENSG000001	261.413288	-0.3713813	0.16724359	-2.2206009	0.026378	0.09212356
ENSG000001	255.948598	0.41847749	0.18054234	2.31789118	0.02045524	0.0778562
ENSG000001	266.253837	-0.2156111	0.11885525	-1.8140646	0.06966778	0.17715792
ENSG000001	576.879176	-0.3083531	0.11541234	-2.6717516	0.00754565	0.03946603
ENSG000001	109.171513	1.48735005	0.30058373	4.9482054	7.49E-07	3.29E-05
ENSG000001	392.213809	-0.1307564	0.05903566	-2.2148711	0.02676891	0.09309119
ENSG000001	2841.49209	0.21214474	0.09399214	2.25704769	0.0240051	0.08684422
ENSG000001	140.327246	0.36686745	0.11738228	3.12540747	0.00177559	0.01458898
ENSG000001	808.657611	0.23453802	0.13522125	1.73447603	0.08283365	0.19886141
ENSG000001	2699.3449	0.15291911	0.08440948	1.81163435	0.07004271	0.17753754
ENSG000001	1292.93927	0.45745128	0.13853009	3.3021799	0.00095937	0.00945355
ENSG000001	1990.44101	-0.3565383	0.14819275	-2.4059094	0.01613227	0.06641244
ENSG000001	239.944002	0.23939298	0.11808858	2.02723233	0.04263866	0.12649604
ENSG000001	1723.82272	0.17874507	0.07285015	2.4535991	0.01414346	0.06050359
ENSG000001	1354.46006	0.36796106	0.12690623	2.89947204	0.00373792	0.0244695

ENSG000001	731.700004	0.21868756	0.10410323	2.10067998	0.03566907	0.11258243
ENSG000001	1161.93873	0.28979933	0.15250741	1.90023108	0.0574028	0.15537037
ENSG000001	256.424538	2.79444988	0.34045442	8.20799989	2.25E-16	6.15E-13
ENSG000001	497.207204	0.34205962	0.10491592	3.26032145	0.00111286	0.0104953
ENSG000001	1119.75807	0.10312576	0.05859008	1.76012337	0.07838689	0.19123789
ENSG000001	55.1072768	-0.7954246	0.38673743	-2.0567561	0.03970969	0.12075776
ENSG000001	1234.88421	0.28382653	0.08949222	3.17152201	0.00151642	0.01312819
ENSG000001	29314.4039	1.55375807	0.20417474	7.60994283	2.74E-14	2.04E-11
ENSG000001	4689.79968	0.0911486	0.0457756	1.9912052	0.04645833	0.1344471
ENSG000001	324.659448	-0.3796364	0.14406406	-2.6351915	0.00840898	0.04262198
ENSG000001	1187.78921	0.06470003	0.03629715	1.78250965	0.07466616	0.18541587
ENSG000001	3684.60805	1.09161865	0.24542196	4.44792571	8.67E-06	0.00025162
ENSG000001	131.888849	-0.4162799	0.13897513	-2.9953551	0.00274125	0.01969691
ENSG000001	657.355114	0.61585772	0.14092918	4.36998028	1.24E-05	0.00033677
ENSG000001	557.733896	0.35832739	0.13927783	2.57275245	0.01008933	0.04811949
ENSG000001	1589.81183	-0.1579759	0.07094607	-2.2267043	0.02596705	0.09121675
ENSG000001	1678.6196	0.52521533	0.16678592	3.14903866	0.00163809	0.01380251
ENSG000001	3204.02696	0.54251961	0.13977469	3.88138661	0.00010386	0.00186328
ENSG000001	1944.32372	0.21604733	0.12153748	1.777619	0.07546645	0.18672452
ENSG000001	78.0743451	0.34522971	0.17520875	1.97039075	0.0487936	0.13905877
ENSG000001	805.422787	0.14715231	0.08342126	1.7639665	0.07773762	0.19013481
ENSG000001	820.262326	-0.1498216	0.08417205	-1.7799452	0.07508493	0.18606128
ENSG000001	2921.26166	-0.1486201	0.07470594	-1.989401	0.04665696	0.1348791
ENSG000001	229.868786	-0.2796675	0.09857282	-2.8371661	0.00455159	0.02802292
ENSG000001	1214.94339	0.48305987	0.17019435	2.83828374	0.00453568	0.02795925
ENSG000001	238.475628	0.16192995	0.09354273	1.73108004	0.08343749	0.19988889
ENSG000001	228.680419	0.27256484	0.1050273	2.5951808	0.00945412	0.04613666
ENSG000001	650.339176	0.45380135	0.21332903	2.12723671	0.03340042	0.10799974
ENSG000001	1352.8356	0.36752454	0.1304586	2.81717375	0.00484483	0.02928149
ENSG000001	1716.00779	0.23425049	0.09660908	2.42472547	0.01531997	0.06400038
ENSG000001	4764.05419	0.11375219	0.05844567	1.94628951	0.05161997	0.14453769
ENSG000001	3003.15458	0.11398576	0.06461193	1.76415972	0.07770509	0.19013481
ENSG000001	2711.79606	0.82407514	0.14799402	5.56830028	2.57E-08	1.94E-06
ENSG000001	136.984471	-0.3101779	0.12566819	-2.4682292	0.01357833	0.05901958
ENSG000001	580.5466	-0.473489	0.16391856	-2.8885627	0.00387007	0.02499479
ENSG000001	1717.12795	0.24546058	0.10739246	2.28564062	0.02227529	0.08231867
ENSG000001	687.869302	0.17684261	0.09425519	1.87621075	0.06062635	0.16137828
ENSG000001	176.526045	0.15514303	0.08774932	1.76802547	0.07705665	0.18903847
ENSG000001	2155.77113	-0.3244326	0.09751992	-3.3268335	0.00087839	0.0089072
ENSG000001	1734.43055	-0.2070847	0.08883854	-2.3310228	0.01975216	0.07600941
ENSG000001	715.656881	-0.2176462	0.05419992	-4.0156178	5.93E-05	0.00118704
ENSG000001	4089.4642	0.12835869	0.0683557	1.87780518	0.06040782	0.16106236
ENSG000001	218.502909	-0.2336881	0.09755418	-2.3954694	0.0165991	0.06772218

ENSG000001	116.042021	0.31326982	0.15054089	2.08096161	0.03743742	0.11609051
ENSG000001	791.175944	-0.1742823	0.09226915	-1.8888468	0.05891235	0.15848825
ENSG000001	629.886178	0.75643889	0.37530894	2.01550988	0.04385125	0.12904185
ENSG000001	200.399947	-0.405033	0.11086076	-3.6535292	0.00025866	0.00359733
ENSG000001	462.326367	0.33491607	0.16503459	2.02936898	0.04242072	0.12603236
ENSG000001	1119.68311	-0.1765002	0.07302512	-2.416979	0.01564992	0.06499413
ENSG000001	184.14962	1.3214335	0.38885653	3.3982546	0.00067817	0.00741828
ENSG000001	391.426994	0.14972033	0.0744838	2.01010605	0.04441997	0.130142
ENSG000001	1327.98801	-0.1191206	0.0561759	-2.1204924	0.03396455	0.10924218
ENSG000001	161.25933	-0.6129141	0.18924634	-3.2387106	0.00120071	0.0110794
ENSG000001	5665.28093	-0.3378352	0.15930748	-2.1206488	0.03395137	0.10922123
ENSG000001	313.66135	-0.3218009	0.16521919	-1.9477212	0.05144833	0.14425369
ENSG000001	7536.89481	1.11613102	0.15865333	7.03503066	1.99E-12	6.34E-10
ENSG000001	2244.58747	0.26959703	0.11962931	2.25360342	0.02422113	0.0872582
ENSG000001	6421.91748	0.27769441	0.11490922	2.4166417	0.01566443	0.06502522
ENSG000001	3322.48732	0.71433686	0.20291201	3.52042679	0.00043085	0.0052989
ENSG000001	72.0491825	0.4748407	0.24377455	1.94786819	0.05143074	0.14425369
ENSG000001	689.142884	0.29387416	0.09378013	3.13365075	0.00172646	0.01429978
ENSG000001	509.171003	0.46966009	0.20797368	2.25826691	0.02392902	0.08665281
ENSG000001	328.652379	0.38289512	0.09241404	4.14325705	3.42E-05	0.00077441
ENSG000001	3487.58001	0.28924383	0.13805154	2.09518729	0.03615435	0.11358622
ENSG000001	1074.45101	0.22499811	0.086429	2.60327095	0.00923389	0.04543278
ENSG000001	781.706429	0.34674236	0.13744489	2.52277372	0.01164333	0.05327627
ENSG000001	3386.09848	0.18435716	0.09265962	1.98961717	0.04663312	0.1348791
ENSG000001	4494.93317	0.32340362	0.12245219	2.64106044	0.0082647	0.04208579
ENSG000001	2261.86294	0.17887169	0.07818456	2.28781345	0.02214839	0.08214592
ENSG000001	218.367367	2.07453439	0.29164617	7.1131893	1.13E-12	3.96E-10
ENSG000001	208.042237	-0.3269021	0.15843317	-2.0633437	0.03907999	0.11942744
ENSG000001	435.048761	-0.5364516	0.18004229	-2.9795864	0.00288638	0.02045945
ENSG000001	1921.51311	0.18732623	0.07601303	2.46439631	0.01372443	0.05943989
ENSG000001	407.330776	1.20333697	0.27276059	4.4116966	1.03E-05	0.00028772
ENSG000001	1351.31831	-0.1989618	0.09041196	-2.2006142	0.02776335	0.09545508
ENSG000001	5158.57573	0.3508532	0.0901299	3.89275039	9.91E-05	0.00179379
ENSG000001	935.726137	-0.1930682	0.09741128	-1.9819898	0.04748038	0.1364177
ENSG000001	1411.66726	0.53482607	0.19376106	2.76023505	0.00577598	0.03283936
ENSG000001	567.452408	0.32410728	0.1059899	3.05790705	0.00222889	0.01702238
ENSG000001	728.757038	0.57950894	0.23396205	2.47693562	0.01325158	0.05810588
ENSG000001	212.366085	0.52117367	0.1424327	3.65908714	0.00025312	0.00354602
ENSG000001	9334.62725	0.22736471	0.09982598	2.27761064	0.02274979	0.08341419
ENSG000001	299.323877	-0.5563071	0.21852733	-2.5457095	0.01090559	0.05072879
ENSG000001	1228.28817	0.21207564	0.08275726	2.56262264	0.01038849	0.04906108
ENSG000001	2065.26056	0.21656884	0.11426706	1.89528681	0.05805441	0.15669436
ENSG000001	709.183293	0.44990066	0.13156714	3.41955182	0.00062724	0.00699179

ENSG000001	1309.02826	-0.220476	0.11597569	-1.9010536	0.057295	0.15523233
ENSG000001	2364.18448	0.62241649	0.16037041	3.88111815	0.00010398	0.0018633
ENSG000001	2306.96476	-0.2452781	0.12410595	-1.9763603	0.04811399	0.13770729
ENSG000001	4193.41473	0.19446214	0.06177308	3.14800802	0.00164387	0.0138283
ENSG000001	5019.42511	-0.3072659	0.16508702	-1.861236	0.06271086	0.1650477
ENSG000001	112.714444	2.56819156	0.4560151	5.6318126	1.78E-08	1.41E-06
ENSG000001	238.382424	0.26591271	0.13365838	1.98949524	0.04664657	0.1348791
ENSG000001	273.323827	0.28212703	0.12527466	2.25206771	0.02431799	0.08741923
ENSG000001	9890.21851	0.44961525	0.17225655	2.61014896	0.00905028	0.04480599
ENSG000001	921.876912	-0.8598886	0.25322187	-3.3957913	0.00068431	0.00747008
ENSG000001	504.430717	0.13325026	0.06917695	1.92622349	0.05407647	0.14912411
ENSG000001	250.292144	1.78835059	0.47670866	3.75145397	0.00017581	0.00274773
ENSG000001	178.524929	0.23636361	0.09058572	2.60928113	0.00907327	0.04486561
ENSG000001	811.539637	0.20341205	0.11072331	1.83712044	0.06619212	0.17115589
ENSG000001	910.389048	-0.3071744	0.12290476	-2.4992881	0.01244431	0.05567861
ENSG000001	59.5839035	0.58408297	0.19048131	3.06635323	0.00216687	0.01665738
ENSG000001	3141.79751	3.33739502	0.48624907	6.86355051	6.72E-12	1.67E-09
ENSG000001	1434.08375	-0.5715286	0.18248477	-3.1319252	0.00173664	0.01435977
ENSG000001	2621.04629	0.26509236	0.08940721	2.96499994	0.00302683	0.02111056
ENSG000001	896.991895	-0.3152433	0.10660849	-2.9570189	0.00310629	0.02150015
ENSG000001	421.979389	-0.3012931	0.06668936	-4.5178583	6.25E-06	0.00019181
ENSG000001	2518.76564	0.19203766	0.09381541	2.0469736	0.04066068	0.12249001
ENSG000001	4296.0039	-0.2175193	0.06563069	-3.3142929	0.00091875	0.00916349
ENSG000001	180.887894	0.54114025	0.15962179	3.39014016	0.00069857	0.00756568
ENSG000001	133.351169	0.5523299	0.17224744	3.20660732	0.0013431	0.01202237
ENSG000001	1244.8389	0.09630684	0.04726229	2.03771017	0.04157893	0.12438782
ENSG000001	3443.29657	-0.2115252	0.05000799	-4.2298272	2.34E-05	0.00055901
ENSG000001	783.685771	0.58906634	0.15883418	3.70868748	0.00020834	0.00310544
ENSG000001	962.233796	-0.1160199	0.06682962	-1.7360557	0.08255399	0.19842243
ENSG000001	2123.37935	0.21214978	0.06766648	3.13522732	0.00171721	0.01427844
ENSG000001	305.338738	0.75023053	0.21850606	3.4334541	0.00059594	0.00669293
ENSG000001	192.631529	-0.2613073	0.09146592	-2.856882	0.00427825	0.02685698
ENSG000001	6836.09335	0.37554655	0.16931258	2.21806648	0.0265503	0.09252822
ENSG000001	4353.78188	-0.163855	0.07762385	-2.1108842	0.03478227	0.11097973
ENSG000001	125.438844	-0.200076	0.10839496	-1.8458055	0.06492046	0.16913624
ENSG000001	24.2533573	0.77867913	0.32203493	2.41799586	0.01560625	0.0648516
ENSG000001	2371.12207	0.10776351	0.04547204	2.36988493	0.01779362	0.07071951
ENSG000001	348.443155	0.45690974	0.11224209	4.07075219	4.69E-05	0.00098134
ENSG000001	377.02472	0.29788194	0.12274754	2.42678535	0.01523327	0.06371681
ENSG000001	23.879275	0.84262059	0.40991137	2.05561653	0.03981949	0.12095595
ENSG000001	28589.4751	0.20090827	0.07085209	2.83560109	0.00457395	0.02811061
ENSG000001	367.329752	-0.2998937	0.12317642	-2.4346682	0.01490545	0.06269704
ENSG000001	1843.02523	1.44189202	0.29950975	4.81417388	1.48E-06	5.76E-05

ENSG000001	45.1164438	1.59619489	0.38083324	4.19132235	2.77E-05	0.00065431
ENSG000001	829.865367	0.29861154	0.133625	2.23469813	0.02543717	0.08998673
ENSG000001	21621.1175	0.51344129	0.13068571	3.92882504	8.54E-05	0.00158694
ENSG000001	7253.546	0.42956663	0.16739748	2.56614745	0.01028351	0.04877602
ENSG000001	304.74985	0.3677701	0.15605507	2.35666876	0.01843969	0.07238581
ENSG000001	14864.7214	0.82349882	0.17609116	4.67654828	2.92E-06	9.96E-05
ENSG000001	6390.55509	0.32864425	0.16070354	2.0450343	0.04085148	0.1228543
ENSG000001	193.928506	0.37244249	0.21086923	1.76622495	0.07735812	0.18957522
ENSG000001	1673.73801	-0.1825745	0.10450162	-1.7470974	0.08062044	0.19503142
ENSG000001	401.328861	-0.7226151	0.14324867	-5.0444804	4.55E-07	2.21E-05
ENSG000001	8565.39293	0.38574033	0.13201403	2.92196469	0.00347831	0.02327912
ENSG000001	594.365248	-0.1818562	0.0871612	-2.0864348	0.03693926	0.11501956
ENSG000001	1798.02488	-0.2303536	0.09509762	-2.4222861	0.0154232	0.06431695
ENSG000001	318.618096	0.19497322	0.08365817	2.33059386	0.01977479	0.07604295
ENSG000001	3002.10753	0.27269352	0.09152867	2.97932362	0.00288885	0.02045945
ENSG000001	2125.75933	-0.2780961	0.10837846	-2.5659722	0.01028871	0.04877833
ENSG000001	772.643672	0.43728105	0.13936883	3.13758132	0.00170348	0.01421764
ENSG000001	1325.32675	-0.2111095	0.0582467	-3.6244036	0.00028963	0.00388629
ENSG000001	427.813974	0.19995519	0.10754513	1.85926764	0.06298921	0.16549176
ENSG000001	1290.84531	0.13490986	0.06059197	2.22653019	0.02597869	0.09121675
ENSG000001	599.594596	0.21115031	0.09223989	2.28914317	0.02207104	0.08195171
ENSG000001	254.784145	0.36350905	0.13316517	2.72976076	0.00633803	0.03498119
ENSG000001	560.976128	-0.2302748	0.12040427	-1.9125133	0.05581039	0.15219076
ENSG000001	2045.39677	0.15621443	0.07114465	2.19572982	0.02811129	0.09610942
ENSG000001	659.707772	0.19001365	0.08536328	2.22594144	0.0260181	0.09129443
ENSG000001	518.638109	0.64465916	0.24571773	2.62357605	0.0087012	0.04351132
ENSG000001	7.51876065	0.94334302	0.38065048	2.47823945	0.01320325	0.05799456
ENSG000001	4134.93847	0.23571655	0.10498083	2.24532938	0.024747	0.08834672
ENSG000001	1572.42082	0.23936839	0.12345961	1.93883972	0.05252086	0.14626096
ENSG000001	15048.4559	0.33319087	0.16570062	2.01080039	0.04434655	0.13003455
ENSG000001	365.76192	-0.194945	0.07091609	-2.7489529	0.0059786	0.03360681
ENSG000001	1021.82143	0.4309101	0.18300587	2.35462447	0.01854143	0.07264609
ENSG000001	40580.87	1.17088045	0.19594547	5.97554231	2.29E-09	2.51E-07
ENSG000001	232.216354	0.33784606	0.17855382	1.89212456	0.05847439	0.15762034
ENSG000001	41.304527	1.50914926	0.34729949	4.34538285	1.39E-05	0.0003677
ENSG000001	1767.77272	0.35302135	0.12638026	2.7933267	0.0052169	0.03084799
ENSG000001	157.654794	-0.4029229	0.13713487	-2.9381505	0.00330177	0.02249235
ENSG000001	371.800052	0.33592357	0.12925552	2.59891076	0.00935201	0.04570637
ENSG000001	90.8285055	0.67833529	0.20749534	3.26915917	0.00107868	0.0102707
ENSG000001	1963.44211	-0.346165	0.08949301	-3.868067	0.0001097	0.00193625
ENSG000001	7430.75417	-0.1568096	0.06010464	-2.6089438	0.00908221	0.04488278
ENSG000001	2259.17977	0.38506076	0.10937765	3.52047025	0.00043078	0.0052989
ENSG000001	787.14728	1.30494187	0.26242103	4.97270302	6.60E-07	2.97E-05

ENSG000001	1404.36541	-0.4286468	0.14562244	-2.943549	0.00324472	0.02221451
ENSG000001	426.273104	0.59245615	0.21049486	2.81458724	0.00488399	0.02943912
ENSG000001	6263.9918	0.1513987	0.06195611	2.44364438	0.01453975	0.0616839
ENSG000001	4156.23973	0.42248341	0.18183185	2.32348412	0.02015316	0.07701034
ENSG000001	2307.85325	-0.2004262	0.08999894	-2.2269835	0.02594838	0.0912059
ENSG000001	29.5183647	1.225753	0.31998874	3.83061294	0.00012782	0.002181
ENSG000001	1342.46246	0.5404001	0.16011059	3.37516781	0.00073771	0.00789568
ENSG000001	764.088917	-0.2631983	0.1176588	-2.2369622	0.02528881	0.08963698
ENSG000001	1911.52564	0.24009018	0.06911532	3.47376211	0.00051322	0.00601946
ENSG000001	126.870929	0.33387976	0.11139437	2.99727672	0.00272403	0.01960754
ENSG000001	235.871656	0.75601067	0.22529457	3.35565424	0.00079177	0.00829038
ENSG000001	23892.4912	0.28564138	0.15327876	1.86354186	0.06238607	0.16444027
ENSG000001	3060.27222	0.26676416	0.08489442	3.14230497	0.00167623	0.01403022
ENSG000001	376.573034	-0.2106793	0.08894614	-2.3686169	0.01785473	0.07082846
ENSG000001	2952.49374	0.14589836	0.06728077	2.16850003	0.03012066	0.1007806
ENSG000001	2811.75193	0.33033982	0.11856342	2.78618671	0.00533321	0.03126518
ENSG000001	969.833396	-0.340244	0.11203136	-3.0370428	0.00238912	0.01787966
ENSG000001	3739.20053	0.3459473	0.13847408	2.49828211	0.01247968	0.05575732
ENSG000001	397.947722	-0.319141	0.11491844	-2.7771082	0.00548449	0.03177711
ENSG000001	614.694717	0.23823576	0.07200409	3.30864185	0.0009375	0.00930517
ENSG000001	1646.5929	0.15947217	0.08067642	1.97668867	0.04807683	0.137649
ENSG000001	4251.25343	0.43626883	0.09082555	4.80337135	1.56E-06	5.96E-05
ENSG000001	2324.72564	0.17380384	0.09665844	1.79812374	0.0721574	0.18135575
ENSG000001	2414.96667	0.27789289	0.07958463	3.49179088	0.00047979	0.00570499
ENSG000001	55.3638495	-0.544625	0.2686777	-2.027057	0.04265658	0.12649809
ENSG000001	367.924022	-0.2949985	0.09602605	-3.0720672	0.00212582	0.01647309
ENSG000001	446.589397	-0.1474466	0.08082956	-1.8241669	0.06812685	0.1745704
ENSG000001	722.272385	0.38493227	0.12622074	3.04967533	0.00229089	0.01738101
ENSG000001	737.39194	0.61926946	0.17690871	3.50050302	0.00046438	0.00556598
ENSG000001	640.499025	0.17664654	0.06955952	2.53950222	0.01110103	0.05140777
ENSG000001	6506.39558	0.20757484	0.092498	2.24410085	0.02482591	0.08851283
ENSG000001	713.634813	-0.6830136	0.21472473	-3.1808799	0.00146828	0.01278767
ENSG000001	66.5649415	1.70191632	0.36068387	4.71858178	2.37E-06	8.37E-05
ENSG000001	4415.04005	0.4211529	0.08949876	4.70568406	2.53E-06	8.85E-05
ENSG000001	261.703147	0.41024533	0.13661894	3.00284375	0.0026747	0.01935236
ENSG000001	3252.6161	0.692616	0.18358895	3.77264534	0.00016153	0.00259153
ENSG000001	172.286374	-0.4220532	0.209954	-2.0102177	0.04440816	0.130142
ENSG000001	2052.25303	0.36824981	0.11024026	3.34042948	0.00083649	0.0085888
ENSG000001	6996.75971	0.25133643	0.14147668	1.77652188	0.07564694	0.18700179
ENSG000001	12868.7735	0.69881934	0.3243902	2.15425536	0.03122015	0.10320904
ENSG000001	303.624279	-0.3730811	0.15730187	-2.3717523	0.01770396	0.07051164
ENSG000001	8345.48021	-0.1696905	0.07532841	-2.2526754	0.02427962	0.08736296
ENSG000001	2404.36588	-0.1596301	0.07453222	-2.1417591	0.03221287	0.10542804

ENSG000001	1932.80885	-0.4095306	0.13087413	-3.1291945	0.00175286	0.01443581
ENSG000001	458.331662	0.21982742	0.11704858	1.87808698	0.06036927	0.16103382
ENSG000001	10789.936	-0.2908953	0.09682863	-3.0042276	0.00266256	0.01928357
ENSG000001	998.802934	-0.3439171	0.09593732	-3.5848105	0.00033732	0.00438569
ENSG000001	15360.2216	0.17319329	0.09853855	1.75761973	0.07881223	0.19197365
ENSG000001	555.451229	0.97298643	0.26623337	3.65463744	0.00025755	0.00359097
ENSG000001	98.3009861	0.32337869	0.16305782	1.98321485	0.04734344	0.13621518
ENSG000001	23.9853896	1.30661159	0.33039122	3.95474068	7.66E-05	0.00146296
ENSG000001	152.958698	0.64251289	0.28875296	2.22513003	0.02607251	0.09140709
ENSG000001	754.89891	-0.1181381	0.06495055	-1.8188936	0.06892766	0.1759755
ENSG000001	3369.76295	0.40773067	0.18827291	2.16563643	0.03033898	0.1012421
ENSG000001	136.881649	0.18756283	0.08709886	2.15344755	0.03128352	0.10335602
ENSG000001	989.869373	0.34390202	0.09298096	3.69862861	0.00021677	0.00318281
ENSG000001	4963.43941	0.36295145	0.08310523	4.36737217	1.26E-05	0.00034025
ENSG000001	10201.1366	0.3203435	0.07917545	4.04599525	5.21E-05	0.0010719
ENSG000001	2267.42063	-0.0803043	0.04428398	-1.8133942	0.06977104	0.17729017
ENSG000001	144.19207	-0.4486441	0.18760966	-2.3913701	0.01678562	0.06816093
ENSG000001	318.264377	-0.2862502	0.08113226	-3.5281919	0.00041841	0.00519353
ENSG000001	52.305567	0.98913827	0.29690234	3.3315274	0.00086371	0.00880735
ENSG000001	635.88469	0.27354751	0.11240172	2.43365958	0.01494705	0.06281053
ENSG000001	183.620797	0.51764753	0.24069786	2.15061122	0.0315069	0.10380121
ENSG000001	24.191302	1.07152395	0.36528605	2.93338317	0.0033529	0.02270858
ENSG000001	1725.57615	0.31834681	0.13129064	2.42474873	0.01531899	0.06400038
ENSG000001	5050.75675	-0.4388331	0.11924595	-3.6800674	0.00023317	0.0033383
ENSG000001	888.125772	-0.2065191	0.08438431	-2.4473637	0.01439055	0.06119344
ENSG000001	94.6296341	-0.5085341	0.26939168	-1.8877126	0.05906455	0.1586926
ENSG000001	407.388732	1.29793936	0.28788937	4.50846582	6.53E-06	0.00019864
ENSG000001	944.935548	-0.5976217	0.23048983	-2.5928334	0.00951889	0.04626295
ENSG000001	483.346368	0.37690389	0.16552986	2.27695411	0.02278897	0.08353916
ENSG000001	131.105375	-0.2795081	0.13337271	-2.0956917	0.03610955	0.11357919
ENSG000001	1261.62106	0.16217891	0.0689885	2.35081076	0.01873256	0.07318507
ENSG000001	1364.99148	-0.1831565	0.09062029	-2.0211417	0.0432651	0.12782302
ENSG000001	152.027333	0.95096262	0.26000289	3.6575078	0.00025468	0.00356009
ENSG000001	393.874462	0.28501222	0.07341536	3.88218791	0.00010352	0.00186122
ENSG000001	81.4357633	0.34275109	0.19189717	1.78611855	0.07408007	0.18457542
ENSG000001	142.38988	-0.2739941	0.11928626	-2.2969462	0.02162184	0.08102572
ENSG000001	293.344943	0.51789988	0.13017082	3.97861744	6.93E-05	0.00133717
ENSG000001	20307.1889	0.99879803	0.19177568	5.20815786	1.91E-07	1.07E-05
ENSG000001	2113.38563	-0.2158428	0.11273792	-1.9145537	0.05554945	0.15174175
ENSG000001	3463.34626	0.25966954	0.11953517	2.17232759	0.02983096	0.10006921
ENSG000001	5520.8325	0.83756883	0.18871098	4.43836839	9.06E-06	0.00025984
ENSG000001	848.087225	0.58135534	0.15556603	3.73703272	0.0001862	0.0028761
ENSG000001	683.460974	0.23544673	0.08721035	2.69975671	0.00693902	0.03736588

ENSG000001	2928.636	0.2894792	0.15439121	1.87497198	0.06079658	0.16175264
ENSG000001	512.078352	-0.2716018	0.08883537	-3.0573608	0.00223295	0.01703501
ENSG000001	818.007978	0.22522544	0.08933547	2.52112013	0.01169819	0.0533883
ENSG000001	240.956165	-0.3955886	0.14194002	-2.7870121	0.00531965	0.03120798
ENSG000001	97.0654982	-0.6217954	0.22253255	-2.7941773	0.00520319	0.03080028
ENSG000001	1298.30917	0.2090689	0.07488243	2.79196222	0.00523895	0.03094489
ENSG000001	290.729738	-0.6843454	0.27578266	-2.4814665	0.0130843	0.05762757
ENSG000001	3378.17104	0.3674972	0.13299031	2.76333814	0.00572135	0.03259656
ENSG000001	2201.62047	-0.0922881	0.0472896	-1.9515518	0.05099144	0.14348834
ENSG000001	5866.36375	0.2021304	0.09306477	2.17193251	0.02986075	0.10014866
ENSG000001	373.617193	0.47570978	0.13746905	3.46048644	0.0005392	0.00623943
ENSG000001	2859.62816	0.39223517	0.11151754	3.51725095	0.00043604	0.00532258
ENSG000001	228.428406	0.3949908	0.1925243	2.05164129	0.04020454	0.12173702
ENSG000001	403.890519	0.20739102	0.08330971	2.48939796	0.01279596	0.05686055
ENSG000001	996.810354	0.29037117	0.14508061	2.00144714	0.04534422	0.13208549
ENSG000001	109.965135	1.12939385	0.20535672	5.49966833	3.81E-08	2.77E-06
ENSG000001	3800.11182	0.16356986	0.07163587	2.28335128	0.02240968	0.08257339
ENSG000001	2580.40278	-0.3630443	0.17369053	-2.0901789	0.03660174	0.11433772
ENSG000001	256.204114	0.4903637	0.18439401	2.65932555	0.00782973	0.04044837
ENSG000001	3170.18888	-0.181977	0.05480608	-3.3203806	0.00089895	0.0090319
ENSG000001	61.6475218	0.98750788	0.42198793	2.340133	0.01927687	0.07460064
ENSG000001	43.2744012	1.69374545	0.32208527	5.2586865	1.45E-07	8.62E-06
ENSG000001	1069.8025	0.13751735	0.07301227	1.88348286	0.05963496	0.15973048
ENSG000001	921.395822	0.25817617	0.08817258	2.92807772	0.00341065	0.02297633
ENSG000001	646.36376	0.17496001	0.0694253	2.52011875	0.01173152	0.05344868
ENSG000001	464.932773	0.36112743	0.18775747	1.92337189	0.05443336	0.14978081
ENSG000001	13967.9224	0.20849731	0.10325291	2.01928754	0.04345734	0.12818313
ENSG000001	250.570974	-0.2226117	0.09926787	-2.2425351	0.02492681	0.08879533
ENSG000001	796.559477	0.32102336	0.09877431	3.25006923	0.00115377	0.01074296
ENSG000001	882.070559	0.28799392	0.08827357	3.26251588	0.00110428	0.01044226
ENSG000001	1916.3896	-0.206359	0.07915401	-2.6070572	0.00913241	0.04506293
ENSG000001	1053.19652	0.18592918	0.0654584	2.84041751	0.00450545	0.0278252
ENSG000001	978.583724	0.1137478	0.05195372	2.18940618	0.02856733	0.09712182
ENSG000001	2362.67678	0.20734507	0.06927056	2.99326417	0.00276011	0.01978902
ENSG000001	538.601406	-0.2902769	0.10895072	-2.6642951	0.00771499	0.04008908
ENSG000001	313.780636	0.26365087	0.13262942	1.98787624	0.04682538	0.13524674
ENSG000001	6371.9568	0.211674	0.11714569	1.80692944	0.07077328	0.17894672
ENSG000001	961.416155	0.60307022	0.1595595	3.77959448	0.00015708	0.00254516
ENSG000001	274.594595	-0.3024509	0.14734503	-2.0526709	0.04010451	0.12161894
ENSG000001	2212.21449	0.22392781	0.0635272	3.52491228	0.00042362	0.00523447
ENSG000001	139.932047	1.4860507	0.44200036	3.36210291	0.00077351	0.00815646
ENSG000001	200.259603	-0.4312336	0.1821119	-2.3679595	0.01788649	0.07087848
ENSG000001	741.71436	-0.2218244	0.09772067	-2.2699843	0.02320854	0.08467184

ENSG000001	3351.37919	1.23231935	0.2457733	5.01404886	5.33E-07	2.50E-05
ENSG000001	310.757205	0.67578581	0.19259341	3.50887296	0.00045001	0.00542959
ENSG000001	643.248185	-0.3402228	0.16630598	-2.0457639	0.0407796	0.12277249
ENSG000001	2931.73068	0.39522049	0.13480659	2.93175934	0.00337048	0.0228088
ENSG000001	1306.65887	-0.3872613	0.11116347	-3.4837099	0.00049452	0.00584612
ENSG000001	1793.0799	0.5168859	0.15951234	3.24041329	0.00119357	0.01102795
ENSG000001	11398.1014	1.27539095	0.26020329	4.90151732	9.51E-07	4.02E-05
ENSG000001	36.2622188	0.71988607	0.27965386	2.57420398	0.0100471	0.04798786
ENSG000001	395.129529	-0.2553769	0.14730316	-1.7336826	0.08297442	0.19911188
ENSG000001	10497.6749	0.29463113	0.11620752	2.5353877	0.01123229	0.05189516
ENSG000001	448.621848	0.24858397	0.09236547	2.69130857	0.00711723	0.03806304
ENSG000001	108.946346	1.46856215	0.28834133	5.09313784	3.52E-07	1.80E-05
ENSG000001	4756.72143	-0.2660395	0.09558844	-2.7831769	0.00538294	0.03143766
ENSG000001	8405.56991	0.44572101	0.09633679	4.62669557	3.72E-06	0.00012308
ENSG000001	3210.27292	0.46163925	0.16800949	2.74769749	0.00600154	0.03367802
ENSG000001	1091.98474	0.13935006	0.06973149	1.9983806	0.04567541	0.13276291
ENSG000001	7677.37707	1.02543796	0.31874907	3.21706971	0.00129507	0.01172572
ENSG000001	160.12742	0.54000964	0.16084684	3.35729102	0.0007871	0.00826753
ENSG000001	2331.86499	0.14200624	0.07260222	1.95594889	0.05047118	0.14258716
ENSG000001	835.506903	-0.2702762	0.13199166	-2.0476765	0.0405917	0.12236375
ENSG000001	1066.16574	0.42651246	0.10265028	4.15500542	3.25E-05	0.00074284
ENSG000001	856.092059	0.13954985	0.06910754	2.01931434	0.04345456	0.12818313
ENSG000001	649.65953	-0.4180223	0.12097251	-3.4555146	0.00054924	0.00629786
ENSG000001	3666.45466	0.37216394	0.14660913	2.5384772	0.01113361	0.05154086
ENSG000001	187.864124	0.2503997	0.14293707	1.75181777	0.07980515	0.19374667
ENSG000001	1419.52751	3.1421452	0.39959921	7.86324175	3.74E-15	4.74E-12
ENSG000001	271.745547	0.3768084	0.14807172	2.54476957	0.01093499	0.05083668
ENSG000001	169.53264	-0.2062504	0.10720193	-1.9239428	0.05436175	0.14973452
ENSG000001	1363.4628	-0.3145314	0.07752749	-4.05703	4.97E-05	0.00102897
ENSG000001	1226.96219	0.22314061	0.09685684	2.30381886	0.02123281	0.07991691
ENSG000001	2259.22025	0.28189104	0.13397741	2.10401912	0.03537678	0.11196472
ENSG000001	4675.90902	-0.2588195	0.09197096	-2.8141433	0.00489074	0.02943912
ENSG000001	615.385335	1.16931068	0.21674874	5.39477493	6.86E-08	4.65E-06
ENSG000001	170.616968	-0.3154426	0.17672631	-1.7849215	0.07427406	0.18486213
ENSG000001	1881.58097	0.27271429	0.06839477	3.9873558	6.68E-05	0.00129497
ENSG000001	70.7671133	0.53933771	0.21758749	2.47871653	0.01318561	0.05793257
ENSG000001	44658.4048	0.33491598	0.17437307	1.92068642	0.05477125	0.15055897
ENSG000001	16306.1332	0.21890392	0.12328573	1.77558196	0.07580185	0.18716398
ENSG000001	726.449534	0.66035488	0.171875	3.84206483	0.000122	0.00210578
ENSG000001	1724.72003	-0.1572338	0.07644044	-2.0569448	0.03969154	0.12074623
ENSG000001	2023.68342	0.56429867	0.13639068	4.13736979	3.51E-05	0.0007893
ENSG000001	86.9762588	0.45412492	0.19320481	2.3504845	0.01874899	0.07321438
ENSG000001	667.316908	0.14339972	0.06619777	2.16623176	0.03029348	0.10118603

ENSG000001	145.418421	0.96381709	0.32948669	2.925208	0.00344226	0.02315125
ENSG000001	143.756923	1.14293259	0.17140156	6.66815735	2.59E-11	5.18E-09
ENSG000001	279.794213	0.69157536	0.20136056	3.43451259	0.00059362	0.00667662
ENSG000001	2988.73012	-0.1550206	0.05606459	-2.7650366	0.00569164	0.03249298
ENSG000001	202.418446	-0.5150809	0.16157	-3.1879734	0.00143274	0.01258307
ENSG000001	2013.12299	0.22601472	0.07293066	3.09903582	0.00194152	0.0154969
ENSG000001	135.950243	0.31652814	0.14776327	2.14213011	0.03218301	0.10539236
ENSG000001	1553.96637	-0.2781818	0.0882929	-3.1506699	0.00162896	0.01374685
ENSG000001	918.685921	-0.3068892	0.08437032	-3.6374069	0.0002754	0.00375277
ENSG000001	4801.70407	0.26105215	0.07783812	3.35378264	0.00079715	0.00834133
ENSG000001	308.518905	0.41892116	0.14600702	2.86918498	0.00411531	0.02614442
ENSG000001	237.265013	0.67292799	0.18307975	3.67560028	0.00023729	0.00337748
ENSG000001	188.764299	0.56576938	0.13990712	4.04389277	5.26E-05	0.00107751
ENSG000001	2620.1413	1.77287458	0.2216181	7.99968324	1.25E-15	2.27E-12
ENSG000001	188.821401	-0.6198	0.26745526	-2.3173969	0.02048212	0.07792235
ENSG000001	225.987107	0.41664655	0.12918791	3.22512027	0.0012592	0.01148335
ENSG000001	262.883956	-0.3499878	0.10548261	-3.3179671	0.00090675	0.00908803
ENSG000001	6599.41033	0.12785733	0.06251347	2.04527667	0.04082759	0.12281232
ENSG000001	3692.33518	0.09964754	0.05670287	1.75736323	0.07885592	0.19201076
ENSG000001	845.276522	0.39767549	0.22414969	1.7741514	0.07603811	0.18747406
ENSG000001	3037.60303	0.12111246	0.04303842	2.81405424	0.0048921	0.02943912
ENSG000001	296.636208	-0.3017518	0.17164852	-1.7579633	0.07875375	0.19190449
ENSG000001	756.384268	0.22485319	0.08675077	2.59194448	0.00954352	0.04629735
ENSG000001	1350.20277	0.11063862	0.05482443	2.01805341	0.0435857	0.128533
ENSG000001	7514.24539	0.45635917	0.09875148	4.62128964	3.81E-06	0.00012607
ENSG000001	25361.364	2.82023213	0.40348761	6.98963753	2.76E-12	7.79E-10
ENSG000001	441.572163	-0.2109885	0.09325262	-2.2625472	0.02366362	0.08595754
ENSG000001	576.415577	0.40357528	0.14608038	2.76269323	0.00573266	0.03264969
ENSG000001	8243.58784	0.43712949	0.14964221	2.92116439	0.00348726	0.02331046
ENSG000001	382.140291	0.96852058	0.1373479	7.0515866	1.77E-12	5.92E-10
ENSG000001	11739.6228	1.25461862	0.17593678	7.13107656	9.96E-13	3.55E-10
ENSG000001	5891.15241	0.1579108	0.08659619	1.82353065	0.06822306	0.1747623
ENSG000001	83.6543834	0.96507138	0.3398419	2.83976575	0.00451467	0.02786721
ENSG000001	1243.3195	-0.1166804	0.04925483	-2.3689134	0.01784043	0.07079612
ENSG000001	11167.8398	0.19623371	0.09623281	2.03915606	0.04143445	0.12403951
ENSG000001	870.656294	0.98659773	0.19838367	4.9731801	6.59E-07	2.97E-05
ENSG000001	5359.22741	0.40054359	0.13244644	3.02419303	0.00249297	0.01837783
ENSG000001	3268.39903	0.91147846	0.26078401	3.49514702	0.0004738	0.00566247
ENSG000001	1025.47698	-0.2512883	0.10171525	-2.4705077	0.01349214	0.05876494
ENSG000001	489.684161	-0.346781	0.10567867	-3.2814667	0.00103269	0.009975
ENSG000001	751.229856	-0.3850331	0.09373181	-4.107817	3.99E-05	0.00086689
ENSG000001	500.170857	0.3409097	0.08274652	4.11992783	3.79E-05	0.00083751
ENSG000001	82.6594687	1.61536454	0.29515129	5.47300516	4.42E-08	3.15E-06

ENSG000001	2420.81479	0.75195735	0.24059907	3.12535436	0.00177591	0.01458898
ENSG000001	514.97911	-0.3338165	0.12321075	-2.7093127	0.00674228	0.03657066
ENSG000001	164.037011	-0.474683	0.12589475	-3.770475	0.00016294	0.00260529
ENSG000001	1305.22034	-0.3143303	0.11292885	-2.783437	0.00537863	0.03143766
ENSG000001	2425.19019	-0.2742759	0.12802724	-2.1423249	0.03216735	0.10536317
ENSG000001	1533.96941	0.16963214	0.06544551	2.5919601	0.00954308	0.04629735
ENSG000001	754.91965	-0.1701939	0.09507376	-1.790125	0.07343382	0.18346707
ENSG000001	489.392779	-0.1220886	0.07032836	-1.7359802	0.08256734	0.19842543
ENSG000001	122.181177	0.44313071	0.14716129	3.01119068	0.00260225	0.01895564
ENSG000001	323.525761	-0.3500874	0.11027185	-3.1747666	0.00149957	0.01298915
ENSG000001	559.131307	-0.1872197	0.08596763	-2.1777933	0.02942143	0.09918022
ENSG000001	32.659367	1.75868819	0.39425293	4.46081197	8.16E-06	0.00024079
ENSG000001	1001.32666	0.7014351	0.23863412	2.93937467	0.00328875	0.02243164
ENSG000001	4927.66938	-0.2727933	0.10076846	-2.7071302	0.00678676	0.03672692
ENSG000001	4465.80414	0.16812226	0.07995849	2.10261929	0.03549906	0.11224029
ENSG000001	10259.8476	0.26384757	0.08650153	3.05020691	0.00228684	0.01736789
ENSG000001	358.183969	-0.5133578	0.18519495	-2.7719857	0.00557155	0.03203619
ENSG000001	370.349599	0.76068023	0.16747453	4.54206515	5.57E-06	0.00017532
ENSG000001	1061.97884	0.18652283	0.06053342	3.0813199	0.00206085	0.01607601
ENSG000001	31599.9719	0.17883332	0.07437115	2.40460597	0.01618991	0.06656619
ENSG000001	9723.17484	0.65488296	0.24673028	2.65424645	0.00794858	0.04086948
ENSG000001	4356.16331	-0.1109753	0.04735585	-2.3434342	0.01910713	0.07413621
ENSG000001	525.538329	0.19803411	0.08360931	2.36856546	0.01785722	0.07082846
ENSG000001	665.459946	0.29580636	0.12301557	2.40462544	0.01618905	0.06656619
ENSG000001	93.3898689	0.57139302	0.19894465	2.87212059	0.00407727	0.02598331
ENSG000001	1139.0802	0.29732911	0.10215529	2.91055992	0.00360782	0.02389232
ENSG000001	3016.3584	-0.0681214	0.03874656	-1.7581291	0.07872555	0.19190449
ENSG000001	209.543736	0.55259851	0.1689609	3.27057039	0.00107331	0.01022605
ENSG000001	4280.0897	0.12946977	0.05220091	2.48022063	0.01313011	0.05776615
ENSG000001	1007.10316	-0.2779753	0.10351442	-2.6853779	0.00724478	0.03853177
ENSG000001	320.837631	0.34129145	0.1825646	1.86942841	0.06156324	0.16313063
ENSG000001	2947.1689	0.25995156	0.12628866	2.05839188	0.03955253	0.12045744
ENSG000001	5375.21392	-0.3347088	0.16030277	-2.0879786	0.03679976	0.1147459
ENSG000001	556.015445	-0.202935	0.08070695	-2.514467	0.01192125	0.05416256
ENSG000001	194.759902	0.31821978	0.16504084	1.9281275	0.05383927	0.14861995
ENSG000001	6555.05857	0.26623574	0.10961715	2.42877823	0.0151498	0.06342598
ENSG000001	462.036361	-0.2437709	0.10867529	-2.2431123	0.02488957	0.08870122
ENSG000001	1114.86234	-0.1967845	0.07157948	-2.7491745	0.00597456	0.03359609
ENSG000001	158.683375	1.42575215	0.27378678	5.20752729	1.91E-07	1.07E-05
ENSG000001	646.216014	1.12284197	0.36337324	3.09005131	0.00200122	0.01580086
ENSG000001	1826.10306	0.49338233	0.2108928	2.3394935	0.01930991	0.07462986
ENSG000001	1434.58942	0.25008083	0.06798698	3.67836368	0.00023474	0.00334999
ENSG000001	1173.16624	0.38209602	0.1481969	2.57829964	0.00992879	0.04756129

ENSG000001	6812.85594	-0.2737175	0.09896266	-2.7658671	0.00567717	0.03244633
ENSG000001	111.003893	0.49171677	0.13475885	3.64886442	0.0002634	0.00363606
ENSG000001	1931.46282	0.24122502	0.1122581	2.14884276	0.03164687	0.1041368
ENSG000001	2599.49455	0.21246501	0.10025375	2.11927248	0.03406745	0.10936801
ENSG000001	6772.03294	-0.1071928	0.04754031	-2.2547778	0.02414728	0.08715451
ENSG000001	847.470213	-0.4362528	0.13587434	-3.2107075	0.00132409	0.01190951
ENSG000001	69.4010474	0.7936933	0.3226525	2.45990132	0.01389752	0.05988901
ENSG000001	6367.96174	0.21395977	0.09574051	2.23478826	0.02543125	0.08998624
ENSG000001	297.146779	0.2648415	0.10730479	2.46812377	0.01358233	0.05901958
ENSG000001	620.724504	0.45382946	0.13748701	3.30088985	0.00096379	0.00948572
ENSG000001	924.765833	-0.1297507	0.07387753	-1.7562945	0.07903814	0.19231167
ENSG000001	127.520932	0.6997542	0.17726567	3.94748845	7.90E-05	0.00149361
ENSG000001	2879.85049	0.22205861	0.08698532	2.55282854	0.01068521	0.05013029
ENSG000001	273.911905	-0.2322803	0.1291087	-1.7991066	0.07200181	0.18108452
ENSG000001	370.456418	-0.3091549	0.10328284	-2.9932847	0.00275992	0.01978902
ENSG000001	19188.048	3.22265364	0.42348334	7.60987114	2.74E-14	2.04E-11
ENSG000001	1087.23054	0.08342437	0.04609018	1.81002491	0.07029193	0.17805912
ENSG000001	464.681768	-0.2403719	0.09613219	-2.500431	0.01240423	0.05561721
ENSG000001	2742.47812	0.12916375	0.07415972	1.74169683	0.08156151	0.19661336
ENSG000001	3242.90516	0.54494711	0.17490299	3.11571063	0.00183502	0.01493985
ENSG000001	26.9155111	0.51631931	0.24239211	2.13009949	0.0331634	0.10750668
ENSG000001	673.35808	0.90093552	0.12434585	7.24540104	4.31E-13	1.74E-10
ENSG000001	388.476025	-0.1876403	0.103389	-1.8148958	0.06953992	0.17702935
ENSG000001	426.4913	-0.1928748	0.09986487	-1.9313576	0.05343884	0.1478577
ENSG000001	15069.3251	0.23123244	0.10454412	2.21181672	0.02697933	0.09360561
ENSG000001	92.9582768	0.26541258	0.13843191	1.91727896	0.0552025	0.1512626
ENSG000001	50.0253349	1.09233362	0.29620702	3.68773707	0.00022626	0.00326579
ENSG000001	109.23264	0.84197297	0.41283684	2.03948118	0.04140203	0.12399435
ENSG000001	710.056403	0.22992405	0.050315	4.56969218	4.88E-06	0.0001572
ENSG000001	80.332828	1.76077462	0.30249899	5.8207619	5.86E-09	5.43E-07
ENSG000001	310.665138	-0.4631241	0.17751153	-2.6089803	0.00908125	0.04488278
ENSG000001	347.151595	0.17609282	0.09221782	1.90953125	0.05619359	0.15278193
ENSG000001	403.832908	1.6931773	0.29667672	5.70714579	1.15E-08	9.66E-07
ENSG000001	782.794784	0.37519691	0.18948291	1.98010948	0.04769123	0.13680774
ENSG000001	253.919928	0.73240013	0.29310433	2.49876944	0.01246254	0.05569589
ENSG000001	4276.0791	0.36297412	0.11990421	3.02720094	0.0024683	0.01825415
ENSG000001	733.113634	0.11806571	0.06750237	1.74906038	0.08028058	0.19438286
ENSG000001	426.238255	-0.4868882	0.16291239	-2.9886503	0.00280213	0.02000282
ENSG000001	4826.22474	0.3192657	0.10612455	3.00840582	0.00262622	0.01908784
ENSG000001	111.749897	1.03260059	0.25272992	4.0857868	4.39E-05	0.00093787
ENSG000001	371.138162	2.17609025	0.29585578	7.35523992	1.91E-13	9.22E-11
ENSG000001	395.641569	0.52741151	0.15155764	3.47994011	0.00050153	0.00591195
ENSG000001	14044.5317	1.47738451	0.23267521	6.34955695	2.16E-10	3.31E-08

ENSG000001	1792.79964	0.26422343	0.14114591	1.87198784	0.06120829	0.16244323
ENSG000001	1414.10379	-0.2454254	0.09646318	-2.5442394	0.0109516	0.05088506
ENSG000001	26.3783996	0.65536636	0.36580925	1.79155219	0.07320473	0.18310744
ENSG000001	157.529114	0.4592469	0.17024873	2.69750558	0.00698611	0.03753318
ENSG000001	71019.4848	0.64915314	0.15638867	4.15089633	3.31E-05	0.00075421
ENSG000001	261.799592	-0.3455251	0.16471229	-2.0977492	0.03592731	0.11311445
ENSG000001	559.435908	0.85523482	0.21277918	4.01935389	5.84E-05	0.00117487
ENSG000001	130.312382	0.33019452	0.10880036	3.03486617	0.00240642	0.01794823
ENSG000001	971.645705	-0.2190967	0.11084022	-1.9766892	0.04807677	0.137649
ENSG000001	16286.7712	0.84851671	0.33219606	2.55426487	0.01064123	0.04996684
ENSG000001	169.429662	0.33488641	0.10061787	3.32829959	0.00087378	0.00888242
ENSG000001	360.708956	0.20288926	0.08475729	2.39376776	0.0166763	0.067902
ENSG000001	41.9034594	1.01686409	0.37410914	2.71809478	0.0065659	0.035899
ENSG000001	243.157504	0.68646585	0.20387264	3.36713077	0.00075955	0.00807148
ENSG000001	488.879095	0.22060287	0.09041412	2.43991611	0.01469067	0.06218977
ENSG000001	3237.46982	0.17138442	0.05766806	2.97191214	0.00295951	0.02077517
ENSG000001	902.111111	-0.2136573	0.11943882	-1.7888432	0.07364008	0.18375839
ENSG000001	13683.7514	0.17418733	0.09536018	1.82662546	0.0677561	0.17400107
ENSG000001	119.885545	-0.973397	0.41348258	-2.3541426	0.01856549	0.07270559
ENSG000001	347.369617	1.44970924	0.32715186	4.43130374	9.37E-06	0.0002671
ENSG000001	227.84307	0.25396012	0.10636014	2.38773765	0.01695244	0.06868523
ENSG000001	910.116159	0.16684381	0.0789455	2.11340481	0.03456614	0.11046208
ENSG000001	214.943464	-0.1700258	0.0889322	-1.9118585	0.05589435	0.15234793
ENSG000001	335.365944	-0.4560145	0.1661067	-2.7453109	0.00604536	0.03385442
ENSG000001	243.94048	1.22397438	0.25578561	4.78515726	1.71E-06	6.43E-05
ENSG000001	164.930324	0.57776971	0.15546785	3.71632907	0.00020214	0.00304918
ENSG000001	1378.36467	-0.3014571	0.10398392	-2.899074	0.00374267	0.02447137
ENSG000001	3098.67069	0.19770711	0.10699247	1.84785995	0.06462261	0.16848734
ENSG000001	143.68632	0.53577779	0.16197665	3.307747	0.0009405	0.00932366
ENSG000001	854.268049	-0.3946026	0.15648943	-2.5215927	0.01168249	0.05337358
ENSG000001	420.329586	-0.3962707	0.19231768	-2.0605006	0.03935071	0.12003277
ENSG000001	66.943794	0.30659538	0.14286398	2.14606497	0.0318678	0.10461186
ENSG000001	5690.85632	0.54760935	0.21654823	2.52881014	0.01144499	0.05258155
ENSG000001	106272.219	0.43903468	0.18386872	2.3877617	0.01695133	0.06868523
ENSG000001	10447.3703	0.46568014	0.11199008	4.15822649	3.21E-05	0.00073456
ENSG000001	4816.75329	0.28608428	0.06152815	4.64964859	3.33E-06	0.00011149
ENSG000001	120.397472	-0.2477763	0.1320052	-1.8770191	0.06051547	0.16123049
ENSG000001	1158.74745	0.35948702	0.19803919	1.81523173	0.06948831	0.1769642
ENSG000001	173.756234	-0.2705624	0.09535502	-2.8374213	0.00454796	0.02802292
ENSG000001	1020.73798	0.173392	0.0883943	1.96157449	0.04981204	0.14118723
ENSG000001	68.7840091	1.84116592	0.38193456	4.82063191	1.43E-06	5.60E-05
ENSG000001	1859.43528	0.47956585	0.25589565	1.87406802	0.06092105	0.1619511
ENSG000001	1522.06719	0.34620969	0.14577019	2.37503769	0.01754716	0.07021004

ENSG000001	5884.69446	-0.1414554	0.05146817	-2.7484046	0.00598861	0.03364
ENSG000001	71.422349	0.86638112	0.19730963	4.39097221	1.13E-05	0.00031203
ENSG000001	630.460586	-0.3021902	0.10999308	-2.7473561	0.00600779	0.03369004
ENSG000001	501.892769	-0.1874375	0.10641627	-1.7613616	0.07817722	0.19089678
ENSG000001	11388.7097	0.20932922	0.11545334	1.81310666	0.06981537	0.17729017
ENSG000001	217.217413	1.36177495	0.49075879	2.77483556	0.00552296	0.03188733
ENSG000001	283.116606	-0.1830982	0.0942477	-1.9427341	0.05204829	0.14548684
ENSG000001	785.95211	-0.487223	0.19185371	-2.5395547	0.01109937	0.05140777
ENSG000001	5971.37786	0.19606101	0.07681205	2.55247713	0.01069599	0.05015627
ENSG000001	142.594233	-0.2560593	0.10542901	-2.4287368	0.01515153	0.06342598
ENSG000001	631.020374	-0.1483162	0.06220477	-2.3843229	0.01711058	0.06905297
ENSG000001	2733.5828	0.27066517	0.12273169	2.20534052	0.02743021	0.09452987
ENSG000001	359.929192	0.27471308	0.10534038	2.60786113	0.00911099	0.04498432
ENSG000001	2128.85133	-0.3018996	0.09777615	-3.0876607	0.00201739	0.01588813
ENSG000001	164.582153	0.36620798	0.1528276	2.39621621	0.01656532	0.06762098
ENSG000001	1058.18833	0.45912434	0.15064745	3.0476742	0.0023062	0.01745027
ENSG000001	4948.29272	0.96702438	0.15243433	6.34387537	2.24E-10	3.35E-08
ENSG000001	159.701397	0.42535707	0.12092798	3.51744137	0.00043573	0.00532258
ENSG000001	278.446441	0.58182497	0.18567294	3.13360138	0.00172675	0.01429978
ENSG000001	1371.4134	2.32402166	0.3114916	7.46094501	8.59E-14	5.03E-11
ENSG000001	848.844755	-0.1465225	0.06982863	-2.098315	0.03587733	0.11304393
ENSG000001	1240.91737	0.35087336	0.09751746	3.59805667	0.0003206	0.00419548
ENSG000001	22772.6885	0.29380171	0.11328394	2.59349838	0.0095005	0.04622542
ENSG000001	1070.2868	-0.2859704	0.084428	-3.3871516	0.00070622	0.00761839
ENSG000001	227.225467	0.7981011	0.38507934	2.07256277	0.03821299	0.1176673
ENSG000001	686.976087	-0.250118	0.10179426	-2.4570934	0.01400663	0.06013261
ENSG000001	1255.04511	-0.3105867	0.13553496	-2.2915611	0.02193098	0.08180216
ENSG000001	261.054168	-0.6520225	0.16598375	-3.9282308	8.56E-05	0.00158906
ENSG000001	144.817311	-0.4343406	0.19128371	-2.2706617	0.02316746	0.08456743
ENSG000001	157.939636	-0.251332	0.11875092	-2.1164637	0.03430539	0.10990728
ENSG000001	863.088622	-0.1050393	0.05789364	-1.8143491	0.06962399	0.17710589
ENSG000001	3135.6724	1.40887176	0.22412921	6.28598024	3.26E-10	4.49E-08
ENSG000001	7477.37074	-0.1812695	0.08289901	-2.1866305	0.02876951	0.09758659
ENSG000001	582.044159	0.77748194	0.16935878	4.59073882	4.42E-06	0.00014369
ENSG000001	1364.81083	0.29539345	0.10889494	2.7126463	0.00667483	0.0363009
ENSG000001	17.8497145	2.70121537	0.64631333	4.17942082	2.92E-05	0.0006836
ENSG000001	934.316579	0.33122616	0.12584315	2.63205564	0.008487	0.04297754
ENSG000001	2486.6399	-0.1260161	0.04979657	-2.5306174	0.0113862	0.05238481
ENSG000001	502.944735	0.13227858	0.05104706	2.59130633	0.00956123	0.04636958
ENSG000001	201.238878	0.22418497	0.08285374	2.70579196	0.00681417	0.03683877
ENSG000001	131.748792	0.4675682	0.12276605	3.80861164	0.00013975	0.00232636
ENSG000001	3826.24849	0.6061417	0.19891239	3.04727972	0.00230923	0.01745708
ENSG000001	8422.62708	0.11014204	0.06349132	1.7347574	0.08278379	0.1987999

ENSG000001	547.763457	0.67168523	0.28294437	2.37391266	0.01760071	0.07028001
ENSG000001	102.215075	0.58869838	0.2154075	2.73295208	0.00627695	0.03477131
ENSG000001	7416.67247	0.39848167	0.14535086	2.7415157	0.00611564	0.03413145
ENSG000001	214.823275	-0.2793865	0.15788982	-1.7695027	0.07681002	0.18865397
ENSG000001	60.7908707	1.28835778	0.48382032	2.66288479	0.00774739	0.04014981
ENSG000001	71.4985913	-0.7290912	0.30607802	-2.3820436	0.01721686	0.06931128
ENSG000001	89.9671099	0.60296259	0.15361166	3.92523982	8.66E-05	0.00160712
ENSG000001	423.772285	-0.4382705	0.21194409	-2.067859	0.03865328	0.1185519
ENSG000001	161.122982	-0.2241668	0.12251426	-1.8297203	0.06729177	0.17318838
ENSG000001	279.27365	0.29907276	0.10422556	2.86947609	0.00411152	0.02613515
ENSG000001	1460.74526	0.12960135	0.0510012	2.54114345	0.01104906	0.05125075
ENSG000001	1661.24294	0.25860132	0.14047747	1.84087404	0.06564002	0.17029384
ENSG000001	240.436011	0.43880476	0.18876434	2.32461681	0.02009246	0.07688589
ENSG000001	4688.59848	0.85634195	0.16497661	5.19068716	2.10E-07	1.15E-05
ENSG000001	3038.58342	-0.1789402	0.09114496	-1.9632484	0.04961731	0.14075694
ENSG000001	2048.00159	0.20365224	0.05919567	3.44032302	0.00058102	0.00656581
ENSG000001	1155.36993	0.86874209	0.20772887	4.18209598	2.89E-05	0.00067754
ENSG000001	949.516369	0.67040982	0.20838725	3.21713455	0.00129478	0.01172572
ENSG000001	2864.14588	0.6113699	0.15525417	3.93786463	8.22E-05	0.00154247
ENSG000001	2360.93144	0.22982722	0.12922582	1.77849296	0.07532293	0.18645381
ENSG000001	882.253107	-0.2671042	0.08351908	-3.1981219	0.00138326	0.0122403
ENSG000001	4659.3763	0.17625793	0.06441626	2.73623337	0.00621469	0.03453146
ENSG000001	5435.25052	-0.2187527	0.07812936	-2.7998782	0.00511219	0.03044844
ENSG000001	544.293838	-0.2308002	0.12639105	-1.82608	0.06783821	0.1740756
ENSG000001	235.92462	0.32768229	0.16382755	2.00016595	0.04548235	0.13239376
ENSG000001	685.214457	0.3873922	0.13592566	2.85002994	0.00437151	0.02718228
ENSG000001	15843.7489	1.02119391	0.28452043	3.58917602	0.00033172	0.00432376
ENSG000001	1472.87693	0.47693159	0.16157273	2.95180735	0.0031592	0.02177444
ENSG000001	191.081315	0.29994766	0.15504948	1.93452863	0.05304816	0.14721744
ENSG000001	3346.01357	-0.1845291	0.09295416	-1.9851625	0.0471264	0.13582906
ENSG000001	19458.4825	0.27208205	0.14616022	1.86153292	0.06266896	0.16496755
ENSG000001	7723.87799	0.43042093	0.20921289	2.05733468	0.03965404	0.12067694
ENSG000001	1116.9781	-0.150178	0.05632407	-2.6663198	0.00766867	0.03993115
ENSG000001	1624.94228	0.17441149	0.08849649	1.97082938	0.0487434	0.13898948
ENSG000001	4029.62954	0.61584091	0.16353695	3.76576	0.00016604	0.00264331
ENSG000001	1822.43483	0.46193648	0.20960731	2.2038186	0.0275371	0.09481854
ENSG000001	832.385859	0.22484453	0.0726242	3.09600021	0.0019615	0.01557518
ENSG000001	2892.04104	-0.232552	0.06118985	-3.8004987	0.00014441	0.00238932
ENSG000001	2213.42073	-0.1365284	0.06075302	-2.2472688	0.02462285	0.08815304
ENSG000001	1046.5405	0.43140862	0.15937511	2.70687576	0.00679197	0.03674295
ENSG000001	241.347361	0.2369216	0.12729682	1.86117452	0.06271954	0.1650477
ENSG000001	2416.24554	0.42668518	0.19863083	2.14813168	0.0317033	0.10428063
ENSG000001	755.006373	0.35995689	0.11847258	3.03831387	0.00237906	0.01783697

ENSG000001	16080.5203	-0.1628003	0.07773319	-2.0943475	0.03622903	0.11371506
ENSG000001	570.784412	0.24896249	0.08245132	3.01950863	0.00253185	0.01858315
ENSG000001	828.621686	-0.2717475	0.14139174	-1.9219477	0.05461234	0.15022288
ENSG000001	1172.7357	0.69168863	0.15659161	4.41714994	1.00E-05	0.00028274
ENSG000001	852.851995	-0.2283008	0.11052009	-2.0656953	0.03885726	0.11898085
ENSG000001	14.72907	1.26289834	0.43649372	2.89327953	0.00381242	0.02477884
ENSG000001	2073.05887	-0.1827785	0.09153436	-1.9968293	0.04584373	0.13309131
ENSG000001	786.660814	1.31749234	0.27064319	4.86800485	1.13E-06	4.63E-05
ENSG000001	114.785919	-0.1734855	0.09377342	-1.8500499	0.06430636	0.16789117
ENSG000001	494.802828	0.19162957	0.08872748	2.15975438	0.03079169	0.10220472
ENSG000001	2662.92668	-0.1711188	0.08024945	-2.1323362	0.03297922	0.10710245
ENSG000001	4353.69256	0.51940956	0.22007779	2.36011801	0.01826912	0.07190562
ENSG000001	8243.81422	-0.0758137	0.03839259	-1.974696	0.04830265	0.13803043
ENSG000001	481.061767	0.27910797	0.15426798	1.8092411	0.07041355	0.17825707
ENSG000001	145.273319	0.27851194	0.14353548	1.94037009	0.05233473	0.14597773
ENSG000001	89.2302496	0.4706702	0.18926764	2.48679709	0.01288989	0.05707997
ENSG000001	401.164123	-0.3327381	0.10764821	-3.0909768	0.00199499	0.01577237
ENSG000001	94.5141324	0.38093617	0.21231954	1.79416448	0.07278692	0.18248563
ENSG000001	1363.49123	0.13650553	0.07527921	1.81332298	0.06978202	0.17729017
ENSG000001	4849.70243	0.23127424	0.12355004	1.87190742	0.06121941	0.16244323
ENSG000001	6682.41829	0.35036355	0.15515716	2.25812037	0.02393816	0.08665281
ENSG000001	19484.5948	0.50164074	0.17549899	2.85836827	0.00425826	0.02675198
ENSG000001	411.161195	0.18729842	0.08484096	2.20764137	0.02726928	0.09425262
ENSG000001	1632.66649	-0.3670609	0.13204527	-2.7798108	0.00543906	0.03162352
ENSG000001	406.652299	-0.5584923	0.23129351	-2.4146477	0.01575044	0.06532189
ENSG000001	6553.65094	-0.1758208	0.07927094	-2.2179734	0.02655665	0.09253067
ENSG000001	40.6477848	1.00002064	0.38228033	2.61593538	0.00889834	0.04421396
ENSG000001	302.481767	0.51990088	0.16515942	3.14787291	0.00164463	0.0138283
ENSG000001	14114.4833	0.22393255	0.11204589	1.99857892	0.04565393	0.13272829
ENSG000001	7670.22272	0.17109917	0.05881736	2.90899115	0.00362597	0.02399036
ENSG000001	1384.79675	-0.2230392	0.12185685	-1.8303375	0.06719948	0.17300516
ENSG000001	204.04081	0.57336911	0.2123412	2.70022547	0.00692925	0.03734934
ENSG000001	68.955964	0.91008773	0.3854575	2.36105856	0.01822285	0.0718418
ENSG000001	173.587285	-0.3532554	0.16922541	-2.0874845	0.03684436	0.1148331
ENSG000001	28.6005978	0.88706882	0.39406727	2.25105936	0.02438178	0.0875576
ENSG000001	152.813361	-0.3746111	0.20677563	-1.8116792	0.07003579	0.17753754
ENSG000001	5406.2752	0.40156277	0.10588145	3.79256973	0.0001491	0.00245209
ENSG000001	5786.63842	0.2837097	0.10902278	2.60229754	0.00926015	0.04552238
ENSG000001	186.158729	2.61360289	0.45360277	5.76187589	8.32E-09	7.45E-07
ENSG000001	36311.0446	0.19227568	0.08897345	2.16104562	0.03069182	0.10201778
ENSG000001	2132.20992	-0.2814624	0.14417012	-1.9522933	0.0509034	0.14328978
ENSG000001	161.808893	0.51465248	0.17750479	2.89937243	0.0037391	0.0244695
ENSG000001	165.688487	-0.1988948	0.10313698	-1.9284526	0.05379885	0.1485584

ENSG000001	6689.22001	-0.1210751	0.05846281	-2.0709771	0.03836094	0.11794568
ENSG000001	1073.22387	0.82013083	0.23484188	3.49226817	0.00047894	0.00570262
ENSG000001	1752.65044	0.21707484	0.07689799	2.82289366	0.00475924	0.02901011
ENSG000001	38.0369045	0.73809893	0.25956021	2.84365208	0.00445997	0.02764845
ENSG000001	8055.49544	0.14656741	0.07806341	1.87754291	0.06044372	0.16110138
ENSG000001	281.203137	-0.3567072	0.1338378	-2.6652198	0.0076938	0.04001118
ENSG000001	300.110604	0.27166177	0.12566139	2.16185552	0.03062931	0.10193401
ENSG000001	364.405135	0.1696532	0.09340127	1.81639064	0.06931047	0.17666466
ENSG000001	201.951263	-0.2521845	0.10652317	-2.3674141	0.01791288	0.07094624
ENSG000001	119.834244	-0.5711416	0.20579612	-2.7752788	0.00551544	0.03188733
ENSG000001	350.802789	-0.1553221	0.05991361	-2.5924339	0.00952995	0.04628631
ENSG000001	2734.6443	-0.1145567	0.06598293	-1.7361564	0.08253619	0.19840871
ENSG000001	6063.60786	0.38347687	0.10170859	3.77034886	0.00016302	0.00260529
ENSG000001	877.397013	-0.275859	0.12106716	-2.2785615	0.02269314	0.08328099
ENSG000001	1755.47116	0.19997642	0.07712725	2.59281139	0.0095195	0.04626295
ENSG000001	627.10025	-0.1796581	0.08878851	-2.0234382	0.04302798	0.127306
ENSG000001	8444.25916	0.45498847	0.12452831	3.65369498	0.00025849	0.00359733
ENSG000001	492.524108	0.30055737	0.0812632	3.6985668	0.00021682	0.00318281
ENSG000001	623.460143	1.10697482	0.28879997	3.8330157	0.00012658	0.0021643
ENSG000001	1763.65058	0.52884675	0.21264889	2.48694805	0.01288442	0.05707997
ENSG000001	39595.9351	-0.2585932	0.11420548	-2.26428	0.0235569	0.08572181
ENSG000001	540.558823	0.39658059	0.11607071	3.41671531	0.00063382	0.00706024
ENSG000001	1877.63717	-0.2852115	0.09828658	-2.9018351	0.00370984	0.02433208
ENSG000001	156.980733	-0.5981857	0.23936828	-2.4990182	0.01245379	0.05567861
ENSG000001	9208.41717	0.20635095	0.07039087	2.93150179	0.00337327	0.02281698
ENSG000001	2886.77895	0.48535755	0.1545592	3.1402695	0.00168792	0.01410647
ENSG000001	65.8060157	0.37211303	0.15059451	2.4709601	0.01347509	0.05874793
ENSG000001	944.517246	-0.2034293	0.09757621	-2.0848246	0.03708523	0.11529892
ENSG000001	1153.27596	-0.320313	0.10229933	-3.1311348	0.00174132	0.01438411
ENSG000001	7606.86061	0.30340058	0.11140926	2.72329763	0.00646338	0.03546855
ENSG000001	1146.92549	0.24793338	0.13666877	1.81411877	0.06965944	0.17715792
ENSG000001	430.045943	-0.2046275	0.11427934	-1.7905908	0.07335899	0.18335859
ENSG000001	10728.1465	0.33403905	0.14218634	2.34930488	0.0188085	0.07339433
ENSG000001	754.913323	-0.2507806	0.10978039	-2.2843847	0.02234893	0.08246072
ENSG000001	262.138696	-0.1950785	0.06279091	-3.1067957	0.00189127	0.01525389
ENSG000001	1084.67182	0.50730708	0.23811433	2.1305189	0.0331288	0.10743926
ENSG000001	5453.47222	-0.0848652	0.0331239	-2.5620538	0.01040552	0.04911277
ENSG000001	190.110826	-0.3253237	0.11917725	-2.7297467	0.0063383	0.03498119
ENSG000001	1929.38738	0.27536	0.13781603	1.99802595	0.04571385	0.13280828
ENSG000001	12982.6702	-0.0878059	0.04890925	-1.7952827	0.07260867	0.1821549
ENSG000001	3980.31954	-0.183142	0.07121909	-2.5715296	0.01012503	0.04820563
ENSG000001	876.516413	1.4719736	0.32046259	4.59327754	4.36E-06	0.00014281
ENSG000001	139.396482	0.33256739	0.16343088	2.03491161	0.04185977	0.12479538

ENSG000001	4032.3381	0.19739547	0.10630678	1.85684748	0.06333286	0.16612844
ENSG000001	2493.38983	0.57089249	0.16223061	3.51901832	0.00043315	0.00530788
ENSG000001	8020.30031	0.598131	0.24952159	2.39711123	0.01652491	0.06750941
ENSG000001	121.84572	0.20577744	0.11847736	1.7368503	0.0824136	0.19823027
ENSG000001	2405.38235	-0.1697673	0.08013602	-2.1184889	0.03413368	0.1094641
ENSG000001	32539.346	0.19248127	0.07835798	2.45643493	0.01403232	0.06016945
ENSG000001	151.755698	0.57296943	0.14019518	4.08694092	4.37E-05	0.00093565
ENSG000001	15.757289	1.66844927	0.3754744	4.44357661	8.85E-06	0.00025541
ENSG000001	654.561376	-0.2240428	0.11335773	-1.9764231	0.04810687	0.13770729
ENSG000001	633.993367	-0.2650919	0.09698126	-2.7334347	0.00626775	0.03475002
ENSG000001	2051.79626	-0.1334857	0.07125921	-1.8732413	0.06103507	0.1621241
ENSG000001	1776.75042	0.31785077	0.11838108	2.68497943	0.00725343	0.03855249
ENSG000001	533.523809	-0.204346	0.07825279	-2.611358	0.00901834	0.04466304
ENSG000001	294.19507	0.22439785	0.12039741	1.86380966	0.06234844	0.16438774
ENSG000001	378.916598	0.43404217	0.1220412	3.55652174	0.0003758	0.00476562
ENSG000001	1158.21626	-0.1448365	0.06357139	-2.2783289	0.02270699	0.08331191
ENSG000001	1294.46113	-0.1963516	0.10764675	-1.8240366	0.06814653	0.17459355
ENSG000001	4316.49695	0.59577501	0.19010844	3.13386926	0.00172518	0.01429978
ENSG000001	19442.1662	-0.2510548	0.09457592	-2.6545324	0.00794184	0.04086948
ENSG000001	13181.1701	0.29339821	0.12776143	2.29645367	0.02164996	0.08108596
ENSG000001	6858.8058	0.15643285	0.06724417	2.32634082	0.02000038	0.07664084
ENSG000001	18879.8183	-0.0963877	0.04180507	-2.3056471	0.02113036	0.07968595
ENSG000001	1616.97413	-0.206467	0.10880359	-1.8976117	0.05774725	0.15599367
ENSG000001	6495.60403	0.15535207	0.05884329	2.64009849	0.00828819	0.04219234
ENSG000001	3256.66323	0.41863399	0.15086203	2.77494607	0.00552109	0.03188733
ENSG000001	3537.01528	-0.2004472	0.07764556	-2.5815672	0.00983529	0.04725144
ENSG000001	288.306719	-0.3715109	0.1202332	-3.0899198	0.00200211	0.01580086
ENSG000001	281.81448	0.24158305	0.10394675	2.32410402	0.02011992	0.07692253
ENSG000001	666.568572	-0.2266835	0.09187158	-2.4673955	0.01360999	0.05908474
ENSG000001	152.545282	0.52611866	0.14457256	3.63913216	0.00027356	0.00374106
ENSG000001	3834.38698	-0.1413462	0.03881208	-3.6418095	0.00027073	0.00370855
ENSG000001	10516.587	0.37834103	0.18181214	2.08094479	0.03743896	0.11609051
ENSG000001	8799.6031	-0.2073339	0.10184271	-2.0358248	0.04176795	0.12465632
ENSG000001	521.291777	0.19218191	0.07068026	2.71903249	0.00654732	0.0358452
ENSG000001	16918.086	-0.1878277	0.0956826	-1.9630283	0.04964288	0.14080511
ENSG000001	50030.149	-0.3615154	0.0927486	-3.8977984	9.71E-05	0.00176657
ENSG000001	3956.31836	0.33555335	0.16036116	2.09248512	0.03639514	0.11396472
ENSG000001	3940.44532	-0.129598	0.05365351	-2.4154625	0.01571524	0.06520315
ENSG000001	2376.61224	0.38206196	0.09549403	4.00089883	6.31E-05	0.00124513
ENSG000001	1785.39926	-0.1441111	0.06697425	-2.1517389	0.03141793	0.10365387
ENSG000001	70.4395182	0.48986001	0.18091466	2.70768552	0.00677542	0.03670062
ENSG000001	228.735846	0.872182	0.26225799	3.32566421	0.00088208	0.00892257
ENSG000001	8775.16948	0.3264919	0.08606945	3.79335421	0.00014863	0.00244681

ENSG000001	9546.30387	0.25890573	0.13493087	1.91880283	0.05500929	0.15099555
ENSG000001	334.71413	-0.237301	0.09812441	-2.4183686	0.01559027	0.06483867
ENSG000001	5318.4367	0.25182046	0.11859464	2.12337137	0.03372274	0.10874176
ENSG000001	664.530685	0.81794578	0.31879274	2.56576036	0.01029499	0.04877833
ENSG000001	40380.5479	0.8433223	0.20555051	4.10274968	4.08E-05	0.00088084
ENSG000001	1944.65127	0.15503341	0.07168552	2.16268815	0.03056517	0.10184457
ENSG000001	1367.05412	-0.1709236	0.07498016	-2.2795839	0.02263238	0.08312393
ENSG000001	6470.84617	0.47103489	0.18490205	2.54748337	0.0108503	0.0506296
ENSG000001	4030.07837	0.3844639	0.15584893	2.46690106	0.0136288	0.05915073
ENSG000001	572.124393	0.67714748	0.14747866	4.59149453	4.40E-06	0.00014346
ENSG000001	48.5419728	0.59420468	0.15056004	3.94662941	7.93E-05	0.00149725
ENSG000001	1498.47111	-0.1372604	0.05408083	-2.5380602	0.01114688	0.05157319
ENSG000001	665.539615	0.32100991	0.11323814	2.83482133	0.00458513	0.02815821
ENSG000001	1263.65659	0.4111555	0.15937086	2.57986617	0.00988386	0.04740148
ENSG000001	2119.55527	-0.3428521	0.14809351	-2.3151057	0.02060715	0.07827352
ENSG000001	678.921012	0.52184343	0.16754706	3.11460806	0.00184189	0.01498836
ENSG000001	279.911493	0.19781661	0.10968712	1.80346248	0.07131561	0.17984652
ENSG000001	4002.79875	0.13481751	0.07477106	1.80307057	0.07137713	0.17997399
ENSG000001	1070.36826	1.29028705	0.26382823	4.89063307	1.01E-06	4.19E-05
ENSG000001	3807.46564	0.20749499	0.07714004	2.6898482	0.00714845	0.03814292
ENSG000001	336.526685	0.44525144	0.23215182	1.91793218	0.05511961	0.15116178
ENSG000002	6156.97934	0.64732416	0.30230215	2.14131515	0.03224863	0.10549452
ENSG000002	384.554566	0.45047027	0.23731572	1.89818977	0.05767109	0.15584862
ENSG000002	113.990262	0.40524527	0.20647623	1.96267277	0.04968421	0.14087359
ENSG000002	1536.40358	0.8239825	0.44821511	1.83836393	0.0660088	0.17082485
ENSG000002	188.141186	0.48495219	0.21662627	2.23865825	0.02517816	0.08937453
ENSG000002	16095.9578	0.47773788	0.23653077	2.01977051	0.0434072	0.1281059
ENSG000002	99.5364071	0.5352527	0.2450966	2.18384381	0.02897373	0.09807642
ENSG000002	110.635451	-0.2397984	0.12645125	-1.8963706	0.05791106	0.15638465
ENSG000002	611.024601	0.57476804	0.21661656	2.65338921	0.00796879	0.0409093
ENSG000002	101.264226	0.414736	0.14746143	2.81250495	0.00491573	0.02953278
ENSG000002	128.386301	-0.1923623	0.09428163	-2.0402941	0.04132104	0.12386827
ENSG000002	984.402763	0.3988854	0.18140914	2.19881646	0.02789098	0.09568947
ENSG000002	223.277906	0.23130617	0.10299958	2.2457001	0.02472322	0.08831247
ENSG000002	743.743875	-0.2640059	0.05097738	-5.1788832	2.23E-07	1.20E-05
ENSG000002	936.845988	0.15395951	0.08399712	1.83291419	0.06681533	0.17231556
ENSG000002	260.963337	-0.2588523	0.1014756	-2.5508822	0.01074506	0.05030104
ENSG000002	405.183541	-0.232051	0.11211574	-2.0697451	0.03847622	0.11818932
ENSG000002	27010.0549	0.43080576	0.19072249	2.25880939	0.02389524	0.08658792
ENSG000002	35.2441608	1.0192356	0.34215801	2.97884475	0.00289337	0.02046706
ENSG000002	51.7256935	-0.4353191	0.18142214	-2.3994817	0.0164183	0.06718514
ENSG000002	677.262282	-0.1944502	0.08034445	-2.4202065	0.01551169	0.06460607
ENSG000002	69.198006	0.5787629	0.22745189	2.54455084	0.01094184	0.05085412

ENSG000002	3770.61204	0.52258623	0.14859627	3.51681916	0.00043675	0.00532258
ENSG000002	5804.34226	0.63689612	0.145911	4.36496302	1.27E-05	0.00034289
ENSG000002	837.25823	-0.2192635	0.082429	-2.6600286	0.0078134	0.04038977
ENSG000002	2790.18633	-0.2078403	0.08463026	-2.4558627	0.01405468	0.06023383
ENSG000002	3942.17859	1.04074235	0.3346213	3.11020946	0.00186955	0.01514485
ENSG000002	1571.56221	-0.1593579	0.06014024	-2.6497719	0.00805461	0.04123243
ENSG000002	2894.36707	-0.1540752	0.06349146	-2.4267073	0.01523654	0.06371681
ENSG000002	137.514914	0.18683709	0.10065587	1.85619658	0.06342554	0.16629175
ENSG000002	1108.20619	0.38721394	0.13708959	2.8245321	0.00473497	0.02888367
ENSG000002	376.052923	0.40214053	0.17603184	2.28447612	0.02234356	0.08246072
ENSG000002	2177.08665	0.32073307	0.10083422	3.18079598	0.00146871	0.01278767
ENSG000002	250.524517	0.1454029	0.08180394	1.77745592	0.07549326	0.18673696
ENSG000002	1221.5565	0.14258612	0.06711582	2.12447842	0.03363016	0.10854994
ENSG000002	5079.7484	0.43644075	0.09339964	4.67283106	2.97E-06	0.00010106
ENSG000002	2799.87222	0.23360672	0.11094096	2.10568502	0.03523172	0.11176137
ENSG000002	169.783697	0.4194874	0.14588689	2.87542912	0.00403479	0.02577266
ENSG000002	484.737157	0.40826509	0.13482186	3.02818177	0.0024603	0.01823385
ENSG000002	69.2680474	-0.6554985	0.18286323	-3.584638	0.00033755	0.00438569
ENSG000002	1473.69721	0.63440391	0.21568473	2.94134823	0.00326787	0.02234498
ENSG000002	225.752225	0.22915129	0.08490093	2.69904342	0.00695391	0.03739694
ENSG000002	1233.21829	0.19927593	0.07195419	2.76948331	0.00561453	0.03223439
ENSG000002	112.007101	-0.393963	0.15862392	-2.4836293	0.01300511	0.05742055
ENSG000002	30.5035944	2.07634322	0.54220294	3.82945771	0.00012843	0.00218898
ENSG000002	959.569822	0.36541146	0.10089157	3.62182342	0.00029253	0.00391885
ENSG000002	2594.64433	0.62108211	0.16762313	3.70522906	0.0002112	0.00313114
ENSG000002	191.475171	0.207678	0.08146234	2.54937445	0.01079164	0.05043656
ENSG000002	3424.574	0.6659835	0.20491039	3.25012076	0.00115356	0.01074296
ENSG000002	2298.19583	-0.1857622	0.07273185	-2.5540694	0.0106472	0.04998059
ENSG000002	549.662992	0.4263145	0.09785909	4.3564117	1.32E-05	0.00035307
ENSG000002	1269.16461	-0.2009114	0.09218842	-2.1793565	0.0293052	0.09896794
ENSG000002	210.554619	0.32841642	0.15686151	2.09367125	0.03628928	0.11383877
ENSG000002	2700.64661	-0.1163547	0.063232	-1.8401229	0.06575019	0.17041312
ENSG000002	790.203364	0.18685851	0.095665	1.95325895	0.05078892	0.14309036
ENSG000002	1924.56404	0.47429816	0.20156664	2.35305884	0.01861969	0.07283358
ENSG000002	112.813243	0.78418014	0.20668839	3.79402129	0.00014823	0.00244269
ENSG000002	68554.6181	0.39457003	0.10961912	3.59946349	0.00031887	0.00417952
ENSG000002	457.153364	1.50792297	0.28411561	5.30742739	1.11E-07	6.93E-06
ENSG000002	94.8471547	0.34228661	0.13410127	2.55244877	0.01069686	0.05015627
ENSG000002	2515.61422	0.236526	0.10969043	2.15630475	0.03105988	0.10288663
ENSG000002	6177.46936	0.32923011	0.09988619	3.29605244	0.00098054	0.00961595
ENSG000002	1135.79778	-0.1399064	0.07777995	-1.7987468	0.07205874	0.18116332
ENSG000002	39.3722557	0.38283816	0.20141952	1.90070043	0.05734126	0.15533201
ENSG000002	98.1438294	0.68752153	0.22150915	3.10380648	0.00191048	0.0153635

ENSG000002	198.110062	1.11559248	0.29925751	3.72786796	0.00019311	0.00295646
ENSG000002	98.4661649	0.96973786	0.26520039	3.65662308	0.00025556	0.00356631
ENSG000002	320.207097	-0.3642035	0.15258664	-2.3868638	0.01699278	0.06877584
ENSG000002	807.107075	1.93508211	0.51099198	3.78691288	0.00015253	0.00249605
ENSG000002	292.788375	-0.2588138	0.07331856	-3.5299907	0.00041557	0.00516617
ENSG000002	1101.17204	-0.4471568	0.25218456	-1.7731333	0.07620662	0.18770616
ENSG000002	1406.81374	-0.137067	0.07154451	-1.9158279	0.05538701	0.15156556
ENSG000002	112.985972	-0.3510726	0.14486685	-2.4234155	0.01537533	0.06416628
ENSG000002	1172.93943	0.41778454	0.17620975	2.37095012	0.01774243	0.07061227
ENSG000002	884.687395	0.27882388	0.10013699	2.7844244	0.00536228	0.03139069
ENSG000002	2523.43308	0.24945494	0.07597972	3.28317798	0.00102644	0.00994271
ENSG000002	3307.15369	0.26591324	0.09156153	2.90420254	0.0036819	0.02418754
ENSG000002	14178.9371	-0.2506965	0.06367312	-3.9372428	8.24E-05	0.00154456
ENSG000002	6099.0852	0.38112274	0.16508121	2.30869851	0.02096032	0.07926345
ENSG000002	811.460961	0.71842469	0.21478858	3.34479934	0.00082342	0.00849329
ENSG000002	737.22856	0.37348574	0.12844835	2.90767243	0.0036413	0.02404604
ENSG000002	20792.1457	1.79089757	0.26412346	6.78053194	1.20E-11	2.68E-09
ENSG000002	2571.9542	0.39836464	0.13771595	2.89265431	0.00382001	0.02480665
ENSG000002	5805.33759	0.53127553	0.17292858	3.0722251	0.00212469	0.01647216
ENSG000002	126885.276	0.26406576	0.0863682	3.05744207	0.00223235	0.01703501
ENSG000002	928.132532	0.77681837	0.25617159	3.03241422	0.00242606	0.01804905
ENSG000002	152.109325	0.22758941	0.1137056	2.00156731	0.04533129	0.13207127
ENSG000002	1106.32294	0.54572901	0.21047299	2.59286954	0.00951789	0.04626295
ENSG000002	1335.78345	0.20594694	0.10256448	2.00797515	0.04464593	0.13063158
ENSG000002	1378.75676	-0.1523704	0.04775823	-3.1904529	0.0014205	0.01249567
ENSG000002	109.032277	-0.4578274	0.22320765	-2.0511275	0.04025453	0.12180357
ENSG000002	124.462012	3.51094323	0.40828131	8.59932383	8.02E-18	3.29E-14
ENSG000002	113.138134	0.56768968	0.24876104	2.28206829	0.02248531	0.08275906
ENSG000002	233.746122	-0.5662756	0.25323054	-2.2362057	0.0253383	0.08974304
ENSG000002	58.927141	3.37239005	0.58445717	5.77012347	7.92E-09	7.14E-07
ENSG000002	1363.4136	-0.3091086	0.17109294	-1.8066708	0.07081363	0.17898108
ENSG000002	52.3694602	-0.7198045	0.26420384	-2.7244287	0.00644128	0.03541251
ENSG000002	445.870671	0.52688353	0.18804374	2.80192015	0.00507994	0.03032041
ENSG000002	3821.14267	0.85556489	0.27073603	3.16014413	0.00157691	0.01348806
ENSG000002	2699.64278	1.10380104	0.27094764	4.07385362	4.62E-05	0.00097333
ENSG000002	254.060953	0.42838204	0.11638052	3.68087402	0.00023244	0.00333443
ENSG000002	1480.68144	1.03211934	0.25126707	4.10765864	4.00E-05	0.00086689
ENSG000002	550.244314	-0.1351098	0.07663796	-1.7629614	0.07790701	0.19035035
ENSG000002	16251.445	0.55416842	0.1700015	3.25978556	0.00111496	0.01050091
ENSG000002	1622.5472	-0.1980257	0.05826442	-3.3987409	0.00067697	0.00741239
ENSG000002	704.504471	0.12308982	0.05369787	2.29226645	0.02189027	0.08171987
ENSG000002	30.7254111	0.63594824	0.35348436	1.79908454	0.07200531	0.18108452
ENSG000002	97.8654132	0.85287073	0.2944099	2.89688202	0.00376891	0.02459168

ENSG000002	355.928219	0.44710627	0.15683569	2.85079425	0.00436102	0.02714792
ENSG000002	136.171485	1.1780912	0.22248943	5.29504343	1.19E-07	7.32E-06
ENSG000002	61.228502	0.62470446	0.29857502	2.09228642	0.0364129	0.11396472
ENSG000002	33.3668152	0.46129605	0.24567543	1.87766459	0.06042706	0.16108317
ENSG000002	112.656869	0.28732065	0.10932068	2.62823704	0.00858287	0.04323989
ENSG000002	186.258069	0.38615928	0.18343292	2.10517982	0.03527566	0.11185747
ENSG000002	1004.20633	0.49003812	0.16768988	2.92228796	0.0034747	0.02326447
ENSG000002	29525.6295	1.27358956	0.36592458	3.48047008	0.00050053	0.00590451
ENSG000002	1918.92055	0.70679094	0.37052	1.90756488	0.05644748	0.15336691
ENSG000002	40.2230106	1.49055575	0.40372297	3.6920261	0.00022247	0.0032311
ENSG000002	56.2253551	1.12837224	0.34666979	3.25489061	0.00113436	0.01062256
ENSG000002	259.952317	0.97036855	0.44549183	2.17819604	0.02939144	0.09910169
ENSG000002	319.71196	1.61762763	0.41968595	3.85437637	0.00011602	0.00202235
ENSG000002	282.882068	1.33890407	0.43085145	3.10757706	0.00188628	0.01522111
ENSG000002	209.490684	1.43412294	0.40895597	3.5067906	0.00045355	0.0054602
ENSG000002	36.0054278	0.91362368	0.40628782	2.2487105	0.02453092	0.08795834
ENSG000002	216.673289	1.47573845	0.45578212	3.23781558	0.00120449	0.01109563
ENSG000002	81.5857574	1.17323418	0.41840946	2.8040336	0.00504676	0.03017936
ENSG000002	311.7049	1.7932891	0.41671261	4.30341934	1.68E-05	0.00042296
ENSG000002	35.9373064	1.57424682	0.48961353	3.21528455	0.00130315	0.01177933
ENSG000002	602.928047	1.25914374	0.39556809	3.18312768	0.00145693	0.01272063
ENSG000002	910.509329	1.48918195	0.37411702	3.9805245	6.88E-05	0.00132805
ENSG000002	1088.24947	1.4853768	0.42937018	3.45943165	0.00054132	0.00624914
ENSG000002	810.651384	1.64346157	0.38152668	4.30759277	1.65E-05	0.00041698
ENSG000002	51.9463542	1.08950917	0.3749646	2.90563211	0.00366512	0.02413534
ENSG000002	26.8125785	1.08701062	0.40031521	2.71538672	0.00661984	0.03607363
ENSG000002	2275.62313	1.18260591	0.36284396	3.25926857	0.001117	0.01050712
ENSG000002	625.193703	1.17969513	0.37824238	3.11888667	0.00181536	0.0148313
ENSG000002	80.7911337	1.30775599	0.45328341	2.8850736	0.00391322	0.02524197
ENSG000002	193.579226	0.57245102	0.22334771	2.56304853	0.01037575	0.04904331
ENSG000002	981.578977	0.92475555	0.39681523	2.33044366	0.01978271	0.07605561
ENSG000002	38.0858453	1.04234856	0.33537148	3.10804175	0.00188331	0.01521193
ENSG000002	4280.8988	1.25130524	0.38926565	3.21452778	0.00130659	0.01179097
ENSG000002	36.5981139	0.65403175	0.34448585	1.89857361	0.05762056	0.1557577
ENSG000002	2478.69875	1.11083062	0.39313691	2.82555665	0.00471985	0.0288129
ENSG000002	83.08957	0.89162218	0.46156252	1.93174736	0.0533907	0.14777975
ENSG000002	1665.56124	-0.3521878	0.18533363	-1.900291	0.05739494	0.15537037
ENSG000002	368.260505	-0.4955559	0.22196691	-2.2325666	0.02557754	0.09030897
ENSG000002	195.325142	-0.3732756	0.16974658	-2.1990166	0.02787674	0.0956866
ENSG000002	124.505836	-0.4132815	0.18268814	-2.2622242	0.02368356	0.08597283
ENSG000002	114.16652	-0.3370366	0.17873091	-1.8857208	0.05933259	0.15933122
ENSG000002	267.283012	-0.3062025	0.16770037	-1.8258901	0.06786681	0.17409766
ENSG000002	163.468457	-0.4161755	0.16345857	-2.5460613	0.01089461	0.05070646

ENSG000002	173.746175	-0.4675206	0.2017383	-2.3174605	0.02047866	0.07792235
ENSG000002	140.465297	-0.4014471	0.20002911	-2.0069436	0.04475567	0.13090595
ENSG000002	122.168605	-0.3904662	0.202385	-1.929324	0.05369065	0.14843461
ENSG000002	145.983953	-0.8012623	0.29332019	-2.7316984	0.00630088	0.03485929
ENSG000002	114.281634	-0.4265341	0.19786263	-2.1557085	0.03110643	0.10295787
ENSG000002	156.934645	-0.4869552	0.18068063	-2.6951155	0.00703642	0.03772931
ENSG000002	129.810379	-0.4987825	0.18362295	-2.7163407	0.0066008	0.03601772
ENSG000002	168.556368	-0.4948711	0.17604887	-2.8109869	0.00493898	0.02964292
ENSG000002	158.355847	-0.4640286	0.17369687	-2.6714849	0.00755165	0.03948481
ENSG000002	185.764899	-0.4959069	0.18844478	-2.6315768	0.00849896	0.04298814
ENSG000002	260.679948	-0.4501064	0.15536514	-2.8970871	0.00376645	0.02458539
ENSG000002	116.684684	-0.491092	0.17941093	-2.7372465	0.00619558	0.03446339
ENSG000002	128.277985	-0.4270271	0.17786809	-2.4008078	0.01635893	0.06702411
ENSG000002	187.670306	-0.4345514	0.1639763	-2.6500867	0.00804711	0.04123243
ENSG000002	235.842763	-0.3132994	0.17843144	-1.7558532	0.07911348	0.19243788
ENSG000002	235.867088	-0.4411527	0.16253843	-2.7141437	0.00664473	0.0361612
ENSG000002	209.77489	-0.4082881	0.17292626	-2.3610531	0.01822312	0.0718418
ENSG000002	232.273955	-0.4726633	0.17254468	-2.7393673	0.00615576	0.03429531
ENSG000002	261.710787	-0.3732016	0.15939414	-2.3413761	0.0192128	0.07440537
ENSG000002	283.133271	-0.3944537	0.16072598	-2.4542002	0.01411983	0.06044985
ENSG000002	137.658861	-0.3216161	0.17875745	-1.7991762	0.07199082	0.18108452
ENSG000002	253.714582	-0.4855365	0.16947268	-2.8649838	0.00417031	0.02640174
ENSG000002	271.960955	-0.4017672	0.15819876	-2.5396357	0.0110968	0.05140777
ENSG000002	232.904173	-0.4270772	0.16936236	-2.5216769	0.01167969	0.05337358
ENSG000002	246.041315	-0.3646153	0.16436647	-2.2183073	0.02653389	0.09251034
ENSG000002	292.347136	-0.4360942	0.16880868	-2.5833633	0.00978422	0.04709612
ENSG000002	224.687138	-0.347693	0.15570601	-2.2330092	0.02554834	0.09024474
ENSG000002	235.593512	-0.4048405	0.15103389	-2.6804613	0.00735208	0.03885015
ENSG000002	172.254826	-0.4932094	0.16203429	-3.0438582	0.00233565	0.01760813
ENSG000002	260.50693	-0.423367	0.15874208	-2.6670118	0.0076529	0.03988702
ENSG000002	356.001696	-0.4651251	0.14719946	-3.1598286	0.00157862	0.01349564
ENSG000002	332.263989	-0.3646376	0.15764008	-2.313102	0.02071703	0.07856086
ENSG000002	256.54354	-0.3572904	0.15311852	-2.333424	0.01962591	0.07555904
ENSG000002	252.05514	-0.3774084	0.15748311	-2.3965008	0.01655246	0.06759917
ENSG000002	187.179642	-0.4351666	0.15592999	-2.7907821	0.00525809	0.0310021
ENSG000002	173.6485	-0.3778989	0.15080678	-2.5058481	0.01221581	0.05503128
ENSG000002	239.637261	-0.359128	0.15936	-2.2535646	0.02422357	0.0872582
ENSG000002	220.154264	-0.4285078	0.16529905	-2.5923185	0.00953315	0.04628813
ENSG000002	230.658987	-0.4232957	0.15009333	-2.8202168	0.00479912	0.02913409
ENSG000002	184.795748	-0.2743668	0.1523326	-1.8011036	0.07168655	0.18061531
ENSG000002	176.21952	-0.4650673	0.16252014	-2.8615977	0.00421512	0.02657257
ENSG000002	30.752921	0.89935167	0.38327652	2.34648255	0.01895155	0.07375947
ENSG000002	88.5843448	0.81059285	0.4662422	1.73856601	0.08211113	0.1976187

ENSG000002	437.422456	0.88154036	0.42867036	2.05645279	0.03973889	0.12080063
ENSG000002	1375.34198	1.23547999	0.43250252	2.8565845	0.00428226	0.0268616
ENSG000002	1343.41593	1.73167613	0.44817473	3.86384156	0.00011162	0.00196162
ENSG000002	114.08353	0.97377319	0.42571632	2.28737575	0.0221739	0.08215615
ENSG000002	42.3123877	1.68995428	0.43193174	3.91254943	9.13E-05	0.00168068
ENSG000002	39.2361735	0.9098016	0.40933711	2.22262181	0.02624131	0.09182219
ENSG000002	86.8341351	0.83357354	0.34663017	2.40479226	0.01618166	0.06656619
ENSG000002	193.580246	0.93370433	0.34441012	2.71102469	0.00670756	0.03643058
ENSG000002	84.1615893	0.69893498	0.33944737	2.05903782	0.03949062	0.12031357
ENSG000002	18.9962854	1.02462756	0.50452852	2.03086151	0.04226904	0.1256956
ENSG000002	105.593046	0.93587945	0.36696297	2.55033757	0.01076187	0.05034588
ENSG000002	100.971496	0.77162299	0.34100498	2.262791	0.02364858	0.08594098
ENSG000002	24.3453151	0.96306018	0.49264134	1.9548911	0.05059593	0.14284115
ENSG000002	290.058039	1.35378489	0.41189721	3.28670568	0.00101367	0.00984662
ENSG000002	270.770998	0.6777146	0.3297804	2.05504815	0.03987435	0.12105532
ENSG000002	174.359068	0.83896377	0.43044903	1.94904325	0.05129026	0.14400794
ENSG000002	134.830489	0.54319573	0.30289105	1.79337001	0.07291378	0.18272463
ENSG000002	183.355406	0.6399341	0.32387557	1.97586408	0.04817017	0.13780151
ENSG000002	151.509644	0.6762819	0.34349025	1.96885327	0.04896994	0.13942701
ENSG000002	53.4378631	0.94180821	0.3523121	2.67322131	0.00751267	0.03936345
ENSG000002	46.4238646	0.89391815	0.34364562	2.60127903	0.00928769	0.04558223
ENSG000002	135.431699	0.35740771	0.19336015	1.84840419	0.06454389	0.16838921
ENSG000002	418.708207	-0.1967908	0.11021539	-1.7855111	0.07417846	0.18473636
ENSG000002	307.001818	-0.8564876	0.26711453	-3.2064432	0.00134387	0.01202237
ENSG000002	111.242332	-0.3801139	0.20815637	-1.826098	0.06783549	0.1740756
ENSG000002	3580.33101	0.23316224	0.09247613	2.52132335	0.01169144	0.05338471
ENSG000002	191.296307	-0.5284257	0.1874653	-2.8187922	0.00482047	0.02920021
ENSG000002	257.362878	-0.3298614	0.17790353	-1.8541586	0.06371648	0.16684112
ENSG000002	6751.87586	-0.1604729	0.06935629	-2.3137467	0.02068162	0.07848103
ENSG000002	1364.90815	-0.3340116	0.08517391	-3.9215255	8.80E-05	0.00162841
ENSG000002	69.0385281	0.61388167	0.18537113	3.31163574	0.00092752	0.00923411
ENSG000002	128.375759	2.14756818	0.79172841	2.71250615	0.00667765	0.03630421
ENSG000002	362.686101	-0.1967912	0.09510627	-2.0691717	0.03852998	0.11828797
ENSG000002	48.5211051	0.77154448	0.21142527	3.64925387	0.000263	0.00363606
ENSG000002	183.045876	-0.2376636	0.07360109	-3.2290766	0.00124191	0.01134459
ENSG000002	138.509247	1.21348904	0.32111496	3.77898633	0.00015747	0.00254887
ENSG000002	482.795764	0.35265706	0.14322214	2.46230823	0.0138046	0.05966895
ENSG000002	3396.36993	0.35580407	0.09237139	3.85188597	0.00011721	0.00203809
ENSG000002	28.4658456	0.63918011	0.32172913	1.98670261	0.04695536	0.13552668
ENSG000002	286.476929	-0.1760179	0.08430559	-2.0878562	0.03681081	0.11475035
ENSG000002	3807.37604	0.32362115	0.12169769	2.65922179	0.00783214	0.04044837
ENSG000002	1786.61243	0.40647919	0.15318705	2.65348275	0.00796658	0.0409093
ENSG000002	131.186934	1.53855856	0.31444052	4.89300345	9.93E-07	4.16E-05

ENSG000002	2726.6155	0.23642785	0.12370181	1.91127231	0.05596959	0.15249808
ENSG000002	223.123063	0.29333566	0.0951207	3.08382586	0.00204357	0.01602432
ENSG000002	151.675254	-0.2147584	0.12317894	-1.7434665	0.08125215	0.1960694
ENSG000002	5571.31555	0.40288332	0.16066284	2.50763231	0.01215431	0.05487174
ENSG000002	171.612621	0.57286968	0.1491246	3.84155034	0.00012226	0.00210798
ENSG000002	1752.68968	0.23230413	0.09724123	2.3889469	0.01689674	0.06851036
ENSG000002	1703.91371	0.17320498	0.06725075	2.57550997	0.01000924	0.04786278
ENSG000002	622.940822	0.20670361	0.07841012	2.63618554	0.00838439	0.0425236
ENSG000002	2755.66426	0.16904993	0.09528811	1.7740927	0.07604782	0.18747406
ENSG000002	11602.8681	0.39979472	0.22067998	1.81164925	0.07004041	0.17753754
ENSG000002	1045.94056	0.14710249	0.06641817	2.21479279	0.02677429	0.09309119
ENSG000002	118.394912	-0.2456913	0.1072974	-2.2898163	0.02203197	0.0819291
ENSG000002	13791.5609	0.38090731	0.14164785	2.68911466	0.00716418	0.03820197
ENSG000002	780.332895	0.5316069	0.13032342	4.07913573	4.52E-05	0.0009601
ENSG000002	172.367596	-0.3595402	0.13302099	-2.7028832	0.00687409	0.03712597
ENSG000002	292.636717	-0.5801249	0.29423196	-1.9716582	0.04864863	0.13883762
ENSG000002	1775.33076	-0.1945025	0.06668273	-2.916834	0.00353604	0.02352148
ENSG000002	239.210982	-0.2537237	0.10493195	-2.4179836	0.01560678	0.0648516
ENSG000002	1130.95344	0.2147463	0.12057929	1.78095504	0.0749198	0.18576439
ENSG000002	581.152219	0.27188454	0.11722879	2.31926436	0.02038071	0.07764462
ENSG000002	108.8741	0.71069669	0.26687581	2.66302405	0.00774419	0.04014981
ENSG000002	324.069226	-0.2849415	0.07694344	-3.703259	0.00021285	0.00314703
ENSG000002	359.472851	-0.168196	0.06986881	-2.4073112	0.01607047	0.06622456
ENSG000002	45.5843119	-0.4095599	0.19965683	-2.0513195	0.04023585	0.12176951
ENSG000002	363.737126	0.88641355	0.23971438	3.69779054	0.00021748	0.00318964
ENSG000002	1627.56893	-0.1782699	0.0980933	-1.8173506	0.06916344	0.17642703
ENSG000002	1022.55433	0.3642317	0.1678681	2.16974936	0.03002584	0.10054516
ENSG000002	649.320067	0.25186164	0.0840664	2.99598449	0.0027356	0.01966492
ENSG000002	1553.42952	-0.3196271	0.09691074	-3.2981598	0.00097321	0.00955714
ENSG000002	1293.63894	-0.1247801	0.06852266	-1.8210053	0.06860606	0.17541455
ENSG000002	1336.78364	-0.1155296	0.05407802	-2.1363506	0.03265085	0.10630976
ENSG000002	4644.48384	0.23823927	0.09788477	2.43387466	0.01493817	0.06278932
ENSG000002	69.5253964	0.65898812	0.1859846	3.54324022	0.00039524	0.00497375
ENSG000002	66.0400088	-0.492987	0.16605864	-2.9687528	0.00299011	0.02090782
ENSG000002	2834.55754	0.36427926	0.14104417	2.58273167	0.00980215	0.04714752
ENSG000002	303.054382	-0.2138513	0.09918112	-2.156169	0.03107047	0.10290094
ENSG000002	4431.73169	0.99053416	0.23339748	4.24397968	2.20E-05	0.00053422
ENSG000002	171.367143	-0.1940293	0.10961492	-1.7700989	0.07671066	0.18851853
ENSG000002	703.743722	0.40642089	0.13782558	2.9488059	0.00319004	0.02195012
ENSG000002	4659.17176	-0.1387348	0.05793458	-2.3946804	0.01663486	0.06780059
ENSG000002	674.986466	-0.1996291	0.08604994	-2.3199213	0.02034514	0.07756317
ENSG000002	471.231738	0.28951894	0.13849457	2.09047149	0.03657547	0.11427743
ENSG000002	110.739376	-0.6836327	0.21694169	-3.1512277	0.00162586	0.01374184

ENSG000002	887.689271	-0.3095081	0.09453499	-3.2740054	0.00106035	0.01014381
ENSG000002	206.604085	-0.3725209	0.10987429	-3.3904279	0.00069784	0.00756273
ENSG000002	94.4066351	0.50238491	0.19041496	2.63836893	0.00833059	0.0423687
ENSG000002	87.1996259	1.28016641	0.29430272	4.34982871	1.36E-05	0.0003617
ENSG000002	214.033922	0.60098959	0.17908966	3.35580276	0.00079135	0.00829038
ENSG000002	168.619919	-0.1983568	0.10780791	-1.8399097	0.0657815	0.17042491
ENSG000002	830.002028	-0.1730722	0.07053168	-2.4538217	0.01413471	0.06049204
ENSG000002	299.291787	0.45652259	0.16742284	2.72676405	0.00639588	0.03521597
ENSG000002	1528.49219	-0.4865225	0.19139804	-2.5419409	0.01102388	0.05116291
ENSG000002	442.446609	1.90622739	0.24243379	7.86287833	3.75E-15	4.74E-12
ENSG000002	1926.87816	-0.2730957	0.08615235	-3.1699155	0.00152483	0.01318012
ENSG000002	242.595231	-0.179072	0.10156317	-1.7631587	0.07787373	0.19032576
ENSG000002	279.614824	0.3588975	0.17600758	2.03910248	0.0414398	0.12403951
ENSG000002	1018.89544	-0.2449066	0.09508885	-2.5755554	0.01000793	0.04786278
ENSG000002	369.369368	0.72611351	0.14634899	4.96152052	6.99E-07	3.11E-05
ENSG000002	304.32935	0.27074772	0.06943179	3.89947788	9.64E-05	0.00175826
ENSG000002	7925.63155	0.31347354	0.16521078	1.89741573	0.05777309	0.15603778
ENSG000002	560.994326	0.33918768	0.17467876	1.94177981	0.05216376	0.14571196
ENSG000002	1470.83198	-0.3122166	0.09888607	-3.157336	0.00159218	0.01356204
ENSG000002	679.106742	-0.3890144	0.11680272	-3.3305256	0.00086682	0.00882308
ENSG000002	2661.69124	-0.3523406	0.14501881	-2.4296202	0.01511465	0.06332032
ENSG000002	199.712329	0.49037396	0.2748851	1.78392338	0.07443612	0.18509256
ENSG000002	2181.87181	0.11396919	0.04786889	2.38086112	0.01727222	0.06946594
ENSG000002	28.9022756	0.852959	0.31519314	2.7061471	0.00680689	0.03681153
ENSG000002	704.60757	0.52585562	0.10882256	4.83222986	1.35E-06	5.33E-05
ENSG000002	361.172745	-0.246231	0.06880473	-3.5786925	0.00034532	0.0044479
ENSG000002	7081.45481	-0.1516506	0.04782755	-3.1707788	0.00152031	0.01314794
ENSG000002	889.532451	-0.1839647	0.08775267	-2.0964003	0.0360467	0.113425
ENSG000002	509.994993	0.22035773	0.11595688	1.90034207	0.05738824	0.15537037
ENSG000002	226.676462	-0.2439176	0.07646248	-3.1900297	0.00142258	0.01250108
ENSG000002	739.741521	-0.1887112	0.07034006	-2.6828411	0.00729997	0.03867449
ENSG000002	120.563888	-0.2376278	0.10129034	-2.3460069	0.01897576	0.07378361
ENSG000002	207.685648	-0.741069	0.24874138	-2.9792749	0.00288931	0.02045945
ENSG000002	3539.69704	0.28995957	0.11018248	2.6316305	0.00849762	0.04298814
ENSG000002	21.9160136	2.47535275	0.38667644	6.40161247	1.54E-10	2.50E-08
ENSG000002	210.915441	-0.2387061	0.119662	-1.9948363	0.04606074	0.13360303
ENSG000002	375.758376	0.34587207	0.11081209	3.12124853	0.00180086	0.01473488
ENSG000002	41.2500361	0.94582967	0.30816713	3.06921016	0.00214626	0.01655322
ENSG000002	464.252062	-0.1799909	0.09739552	-1.8480408	0.06459644	0.1684727
ENSG000002	645.179896	-0.2675493	0.11324362	-2.3625996	0.01814726	0.07161508
ENSG000002	120.527527	0.41267576	0.22428375	1.83997176	0.06577238	0.17042491
ENSG000002	107.51961	1.56927397	0.24638709	6.36914038	1.90E-10	2.97E-08
ENSG000002	505.125561	-0.2738384	0.10229097	-2.6770532	0.00742728	0.03907127

ENSG000002	636.797984	0.22857954	0.12741073	1.79403688	0.07280728	0.18248563
ENSG000002	131.764311	-0.2554973	0.08723691	-2.928775	0.00340301	0.02297205
ENSG000002	493.842252	-0.3082576	0.11479122	-2.6853758	0.00724483	0.03853177
ENSG000002	264.780086	-0.3195111	0.09084968	-3.5169208	0.00043658	0.00532258
ENSG000002	135.822683	-0.2585596	0.1021193	-2.5319368	0.01134344	0.05226144
ENSG000002	245.655504	-0.3425204	0.11481036	-2.9833576	0.00285105	0.02026381
ENSG000002	111.258526	-0.3667512	0.12729918	-2.8810181	0.00396393	0.02541906
ENSG000002	115.838333	-0.5724801	0.20641338	-2.773464	0.0055463	0.03196578
ENSG000002	96.6319316	1.06269154	0.32895775	3.23048036	0.00123582	0.01133249
ENSG000002	287.210172	0.23414461	0.12162003	1.92521416	0.05420257	0.14939646
ENSG000002	165.949775	-0.3176284	0.09447127	-3.3621693	0.00077333	0.00815646
ENSG000002	825.843791	0.40899973	0.10270005	3.98246873	6.82E-05	0.00131878
ENSG000002	641.311912	-0.2528246	0.08854596	-2.8552926	0.00429972	0.0268862
ENSG000002	260.472713	-0.303363	0.12365892	-2.4532241	0.01415821	0.06053511
ENSG000002	2847.35673	0.34940274	0.14012388	2.49352739	0.01264808	0.05637146
ENSG000002	578.763798	0.30629944	0.14117	2.1697204	0.03002803	0.10054516
ENSG000002	511.318111	-0.288915	0.13067422	-2.2109564	0.02703885	0.09372203
ENSG000002	95.9398408	0.78254935	0.31818539	2.45941324	0.01391643	0.05989025
ENSG000002	202.758992	-0.1417875	0.07688969	-1.8440372	0.06517772	0.1695841
ENSG000002	368.374664	-0.1048566	0.05833343	-1.7975389	0.0722501	0.18156091
ENSG000002	81.5324502	-0.3905598	0.13294043	-2.9378558	0.00330491	0.02250439
ENSG000002	674.609975	-0.129911	0.06521441	-1.9920603	0.04636444	0.13427017
ENSG000002	450.54968	0.14872072	0.05720396	2.59983258	0.00932693	0.04562458
ENSG000002	237.122159	-0.2041927	0.09143276	-2.2332551	0.02553213	0.09020692
ENSG000002	7800.74169	0.75237558	0.2212289	3.40089188	0.00067166	0.00736674
ENSG000002	187.327431	0.12975384	0.07042385	1.84247001	0.06540643	0.16991572
ENSG000002	375.257164	0.15358058	0.08489123	1.80914528	0.07042844	0.17826722
ENSG000002	305.554059	-0.1725629	0.09366166	-1.8424074	0.06541558	0.16991572
ENSG000002	3658.93572	-0.163717	0.09405169	-1.740713	0.0817339	0.19697101
ENSG000002	26432.4578	1.01982887	0.14202515	7.18062144	6.94E-13	2.65E-10
ENSG000002	53312.9325	0.48949271	0.15798837	3.0982832	0.00194645	0.01552334
ENSG000002	298.033504	-0.3351949	0.11617648	-2.8852222	0.00391138	0.02523999
ENSG000002	129.636222	0.24297249	0.12461353	1.94980831	0.05119897	0.14382551
ENSG000002	167.98227	-0.4957821	0.1974042	-2.5115074	0.01202168	0.05445288
ENSG000002	444.027232	0.49741023	0.25222223	1.97211094	0.04859694	0.13872634
ENSG000002	35.2890844	0.77343617	0.23983383	3.22488352	0.00126024	0.01148646
ENSG000002	227.727477	-0.1509951	0.05637368	-2.6784674	0.00739599	0.0390354
ENSG000002	177.666783	0.99004023	0.36806328	2.68986413	0.00714811	0.03814292
ENSG000002	157.90206	-0.2946045	0.12379574	-2.3797626	0.0173238	0.06957097
ENSG000002	119.023471	0.69530175	0.26589582	2.61494046	0.0089243	0.0443161
ENSG000002	167.309576	0.45580088	0.19368807	2.35327287	0.01860897	0.07282371
ENSG000002	39.6379563	1.02672481	0.38385146	2.67479723	0.00747745	0.03925958
ENSG000002	67.8342294	1.02050207	0.28405443	3.59262863	0.00032736	0.00427365

ENSG000002	98.4795741	-0.4684264	0.22625634	-2.0703349	0.038421	0.1180418
ENSG000002	73.087751	-0.9256565	0.2470611	-3.7466702	0.0001792	0.00278248
ENSG000002	532.24807	0.95146499	0.25631739	3.71205792	0.00020558	0.00308409
ENSG000002	513.771928	-0.1502626	0.06658435	-2.2567254	0.02402524	0.086867
ENSG000002	79.9720073	0.48985051	0.17606875	2.78215484	0.00539993	0.03147622
ENSG000002	651.429155	0.24450784	0.10647942	2.29629205	0.02165919	0.08110201
ENSG000002	165.566753	0.49076172	0.15407783	3.18515475	0.00144677	0.01265882
ENSG000002	70.7944469	0.48835778	0.2416832	2.02065257	0.04331574	0.12790352
ENSG000002	111.990483	0.73802414	0.35677208	2.06861522	0.03858221	0.11840398
ENSG000002	28.1422783	1.16939162	0.33360805	3.50528597	0.00045612	0.00548385
ENSG000002	2629.23879	-0.1641022	0.09403996	-1.7450262	0.08098031	0.19558611
ENSG000002	34.5864433	1.63376612	0.68850462	2.37291962	0.0176481	0.07040971
ENSG000002	165.114147	0.22056005	0.1078849	2.04440143	0.04091391	0.12295919
ENSG000002	106.884339	-0.7251997	0.24358481	-2.977196	0.00290898	0.02054411
ENSG000002	192.569118	-0.3256115	0.0901365	-3.6124271	0.00030334	0.00401621
ENSG000002	114.250689	0.58363184	0.20131867	2.89904481	0.00374301	0.02447137
ENSG000002	1216.60266	0.3163392	0.10139196	3.11996342	0.00180874	0.01478456
ENSG000002	601.905432	0.98442771	0.19037379	5.17102556	2.33E-07	1.24E-05
ENSG000002	639.666479	0.66490909	0.21568433	3.08278813	0.00205071	0.01604136
ENSG000002	1579.34124	0.32420473	0.09480888	3.41956083	0.00062722	0.00699179
ENSG000002	286.634638	0.37228268	0.19981758	1.86311278	0.0624464	0.16451376
ENSG000002	115.815208	-0.3102894	0.11660469	-2.661037	0.00779004	0.04032781
ENSG000002	405.650713	-0.2119102	0.11345369	-1.8678121	0.06178826	0.16354192
ENSG000002	49.0118602	0.93604062	0.38406918	2.43716669	0.01480285	0.06243909
ENSG000002	277.317368	-0.3207763	0.07996033	-4.0116924	6.03E-05	0.00120401
ENSG000002	10617.1708	-0.2580782	0.10868189	-2.3746204	0.01756701	0.070219
ENSG000002	16.0034955	1.0987193	0.4157226	2.64291453	0.00821958	0.04190809
ENSG000002	42.543242	-0.8064338	0.30480709	-2.6457186	0.00815176	0.04161408
ENSG000002	320.156471	-0.2711781	0.10183412	-2.6629392	0.00774614	0.04014981
ENSG000002	323.744413	0.9278771	0.31207269	2.97327235	0.00294643	0.02072612
ENSG000002	215.5515	0.32771267	0.12318097	2.66041647	0.00780441	0.040356
ENSG000002	289.380182	-0.9623768	0.25684775	-3.7468765	0.00017905	0.00278248
ENSG000002	32.7456613	0.9511545	0.2378874	3.99833912	6.38E-05	0.00125112
ENSG000002	90.6399569	0.74788036	0.21255295	3.51856021	0.0004339	0.00530939
ENSG000002	288.319861	-0.4454105	0.18865477	-2.3609819	0.01822662	0.0718418
ENSG000002	183.090166	0.81382344	0.35666149	2.28178111	0.02250227	0.08278431
ENSG000002	103.439109	0.54916246	0.13706393	4.00661542	6.16E-05	0.00122421
ENSG000002	139.867636	0.15001145	0.07574103	1.98058361	0.04763799	0.13675418
ENSG000002	284.867276	-0.2381474	0.11318852	-2.1039892	0.03537939	0.11196472
ENSG000002	109.729425	-0.2717009	0.12526236	-2.1690551	0.0300785	0.10067304
ENSG000002	370.974167	-0.3430175	0.09763308	-3.5133334	0.00044252	0.00536244
ENSG000002	139.459577	-0.2900008	0.09819031	-2.9534568	0.00314237	0.02172233
ENSG000002	20.7226212	1.0348814	0.48769248	2.12199582	0.03383809	0.10898148

ENSG000002	243.434595	0.56256219	0.17453647	3.22317853	0.00126777	0.01151665
ENSG000002	146.393557	0.39246912	0.12681554	3.09480318	0.00196944	0.01561549
ENSG000002	662.300862	0.70030183	0.18179826	3.85208208	0.00011712	0.00203809
ENSG000002	945.071764	-0.2107394	0.09416377	-2.2380093	0.02522045	0.08949139
ENSG000002	60.6350568	0.87962623	0.38124454	2.3072494	0.02104092	0.07947661
ENSG000002	3104.25126	-0.1419296	0.05471368	-2.5940427	0.00948547	0.04620715
ENSG000002	32.2409554	0.79630169	0.25142895	3.16710424	0.00153965	0.01326624
ENSG000002	510.956652	-0.9724244	0.26688113	-3.6436611	0.00026879	0.0036943
ENSG000002	173.451603	-0.7587085	0.22555744	-3.3637039	0.00076904	0.00813545
ENSG000002	211.346619	0.79212785	0.24732647	3.2027622	0.00136116	0.0121233
ENSG000002	113.528733	1.71699097	0.25233168	6.80450011	1.01E-11	2.41E-09
ENSG000002	370.089133	-0.2927395	0.09138111	-3.2035015	0.00135767	0.01211195
ENSG000002	28.2213937	1.69966149	0.35103124	4.84190952	1.29E-06	5.13E-05
ENSG000002	132.242431	-0.2750929	0.13210012	-2.0824578	0.03730068	0.11585891
ENSG000002	1797.26112	0.78515146	0.20072311	3.91161472	9.17E-05	0.00168531
ENSG000002	585.973926	-0.173046	0.06723832	-2.5736209	0.01006405	0.0480548
ENSG000002	207.258454	-0.246714	0.13419335	-1.8384968	0.06598923	0.1708061
ENSG000002	1602.90207	0.57736428	0.24136596	2.39207006	0.01675364	0.06816093
ENSG000002	151.66181	0.13756958	0.0734124	1.87392829	0.06094031	0.1619511
ENSG000002	1665.63291	0.74701731	0.18579487	4.0206563	5.80E-05	0.00117484
ENSG000002	191.534159	0.63960647	0.30734028	2.08110196	0.03742457	0.11608981
ENSG000002	89.0621524	0.42365518	0.2140402	1.97932529	0.04777939	0.13703668
ENSG000002	880.171082	0.56215219	0.24832383	2.2637867	0.02358724	0.08579413
ENSG000002	591.071012	-0.2958608	0.09853024	-3.0027411	0.0026756	0.01935236
ENSG000002	2707.44017	0.27503281	0.09366454	2.93635992	0.00332089	0.02258498
ENSG000002	374.92526	-0.9460844	0.2656879	-3.5608864	0.0003696	0.00470546
ENSG000002	142.744483	-0.4654181	0.17859078	-2.6060592	0.00915906	0.04518086
ENSG000002	179.689632	2.44217073	0.27497819	8.88132506	6.61E-19	3.61E-15
ENSG000002	16.218145	0.93017683	0.33149729	2.80598625	0.00501628	0.03002964
ENSG000002	31.5853324	1.75829489	0.50099635	3.5095962	0.00044879	0.00542282
ENSG000002	20.4746859	1.49823106	0.62651132	2.39138707	0.01678485	0.06816093
ENSG000002	91.6224584	-0.444638	0.15947355	-2.7881617	0.00530081	0.03116434
ENSG000002	104.693275	-0.3144035	0.12333354	-2.5492131	0.01079663	0.05043656
ENSG000002	342.875739	0.19742528	0.08545243	2.31035301	0.02086862	0.07900779
ENSG000002	40.5737734	1.52182733	0.2874315	5.29457404	1.19E-07	7.32E-06
ENSG000002	625.206963	0.34415316	0.11218803	3.06764589	0.00215752	0.01661666
ENSG000002	178.878989	-0.2165396	0.11014338	-1.9659795	0.04930097	0.1401525
ENSG000002	6216.26112	-0.2106451	0.11885762	-1.7722474	0.0763535	0.18801147
ENSG000002	116.942482	0.21553196	0.08621897	2.4998207	0.01242562	0.05565527
ENSG000002	4294.49769	-0.1571312	0.04789835	-3.2805133	0.00103618	0.00998255
ENSG000002	559.224449	0.43572909	0.22637484	1.92481239	0.05425283	0.14950987
ENSG000002	200.709309	-0.3863654	0.20698333	-1.8666496	0.06195054	0.16389206
ENSG000002	456.31862	2.75123846	0.35917519	7.65987885	1.86E-14	1.70E-11

ENSG000002	2660.90948	0.40355583	0.17659005	2.28526936	0.02229704	0.08238047
ENSG000002	367.765232	-0.1495041	0.06384481	-2.3416795	0.01919719	0.07436248
ENSG000002	625.630193	-0.1745797	0.07805751	-2.2365528	0.02531558	0.08969308
ENSG000002	1401.85023	0.16022424	0.08055472	1.98901127	0.04669996	0.13497959
ENSG000002	57.0968387	0.51095031	0.25778657	1.98206721	0.04747172	0.13641672
ENSG000002	2356.19443	0.50531167	0.12787771	3.95152257	7.77E-05	0.00147546
ENSG000002	825.4011	2.67527838	0.33601203	7.96185305	1.69E-15	2.78E-12
ENSG000002	241.603651	0.23697111	0.09679371	2.44820767	0.01435689	0.06108196
ENSG000002	545.536247	-0.2610646	0.10816874	-2.4134939	0.01580039	0.06546122
ENSG000002	1594.17053	0.48801872	0.2489944	1.95995864	0.05000062	0.14152646
ENSG000002	47.1740674	1.17407375	0.27564435	4.25937904	2.05E-05	0.0005002
ENSG000002	351.530391	0.41906383	0.17431476	2.40406394	0.01621394	0.06659818
ENSG000002	237.170257	0.57064066	0.29805811	1.9145282	0.0555527	0.15174175
ENSG000002	104.854874	-0.3039297	0.1512196	-2.0098562	0.04444641	0.13016393
ENSG000002	895.114193	-0.2225684	0.12257907	-1.8157133	0.06941436	0.17682305
ENSG000002	122.436147	0.19971632	0.09282431	2.1515518	0.03143267	0.10367326
ENSG000002	275.943009	0.19868062	0.11253715	1.76546694	0.07748532	0.18980084
ENSG000002	237.383807	0.44973082	0.1966181	2.28733169	0.02217647	0.08215615
ENSG000002	123.201466	-0.3510983	0.11301657	-3.1066087	0.00189247	0.01525604
ENSG000002	77.3524161	0.61260253	0.18495462	3.31217746	0.00092573	0.00922185
ENSG000002	71.5999049	-0.8454124	0.22238617	-3.8015513	0.00014379	0.00238159
ENSG000002	1053.95714	0.22611878	0.09606272	2.35386602	0.01857931	0.07272497
ENSG000002	146.263824	0.82626584	0.19103015	4.32531652	1.52E-05	0.00039251
ENSG000002	238.445459	0.36675093	0.18930804	1.93732356	0.0527058	0.14660285
ENSG000002	178.002757	-0.1541215	0.08077462	-1.9080441	0.05638552	0.15327477
ENSG000002	203.207575	-0.4484421	0.13320587	-3.366534	0.00076119	0.0080761
ENSG000002	208.364525	-0.2357532	0.08263115	-2.8530794	0.00432978	0.02700472
ENSG000002	140.945172	-0.4634606	0.16663187	-2.7813446	0.00541342	0.03151009
ENSG000002	3115.93745	0.19640241	0.09536405	2.05950149	0.03944622	0.12022299
ENSG000002	130.22461	-0.404318	0.14085023	-2.8705529	0.00409755	0.02608209
ENSG000002	195.565117	-0.2047937	0.1096268	-1.8680985	0.06174834	0.16346262
ENSG000002	272.921687	-0.3817095	0.15315014	-2.4923875	0.01268875	0.0564913
ENSG000002	606.916299	1.04086836	0.22833667	4.55848096	5.15E-06	0.00016407
ENSG000002	56.3430201	0.83309264	0.25316811	3.29066973	0.00099949	0.00974356
ENSG000002	187.693968	-0.2491132	0.11075386	-2.2492507	0.02449655	0.08791201
ENSG000002	216.572957	-0.3184335	0.14803375	-2.1510871	0.03146933	0.10371911
ENSG000002	317.0283	-0.205502	0.08671842	-2.3697615	0.01779956	0.07071951
ENSG000002	133.593585	0.31874816	0.16988331	1.8762771	0.06061724	0.16137828
ENSG000002	212.615474	-0.2107744	0.12065048	-1.7469831	0.08064027	0.19503142
ENSG000002	498.283032	-0.2458913	0.07082321	-3.4719021	0.00051678	0.00604104
ENSG000002	79.7656334	0.52647863	0.28784317	1.82904676	0.0673926	0.17339346
ENSG000002	43.1224671	-0.8785607	0.30389208	-2.8910286	0.00383983	0.02485658
ENSG000002	196.508168	-0.432247	0.15575904	-2.7751008	0.00551846	0.03188733

ENSG000002	790.184981	0.31684202	0.161838	1.95777271	0.05025669	0.14212814
ENSG000002	101.474124	-0.4623714	0.15485454	-2.9858434	0.00282797	0.02013503
ENSG000002	142.317932	-0.1718564	0.09552007	-1.7991657	0.07199247	0.18108452
ENSG000002	126.530739	-0.2471868	0.10347502	-2.3888544	0.016901	0.06851067
ENSG000002	571.130677	0.40860047	0.12094425	3.37842005	0.00072904	0.00781845
ENSG000002	214.238161	-0.7593595	0.28465659	-2.6676338	0.00763875	0.03982593
ENSG000002	200.898636	-0.2867471	0.09792853	-2.9281265	0.00341011	0.02297633
ENSG000002	145.244135	-0.2840817	0.07783432	-3.6498259	0.00026242	0.00363606
ENSG000002	113.172547	-0.3219142	0.15107815	-2.1307794	0.03310732	0.10741209
ENSG000002	212.757922	-0.2156753	0.12106344	-1.7815064	0.07482976	0.18565343
ENSG000002	75.5925309	0.53414085	0.17330162	3.08214581	0.00205514	0.01604674
ENSG000002	156.283652	-0.3229975	0.14021291	-2.3036218	0.02124388	0.07992709
ENSG000002	502.488678	-0.353571	0.14236502	-2.4835528	0.01300791	0.05742055
ENSG000002	56.2840821	0.70045705	0.22216827	3.15282217	0.001617	0.01370941
ENSG000002	138.532573	-0.9702575	0.26859713	-3.6123151	0.00030348	0.00401621
ENSG000002	248.829754	0.45794763	0.19104403	2.39707895	0.01652636	0.06750941
ENSG000002	167.420798	-0.1600287	0.07837613	-2.0418042	0.04117096	0.12352795
ENSG000002	59.2021794	0.57092654	0.19675706	2.90168256	0.00371164	0.0243342
ENSG000002	191.412659	-0.3397479	0.14848459	-2.288102	0.02213158	0.08212075
ENSG000002	553.940574	-0.2654636	0.09599378	-2.7654246	0.00568487	0.03246774
ENSG000002	202.142131	0.28951326	0.13607621	2.12758177	0.03337178	0.10794969
ENSG000002	212.370254	-0.2376309	0.0976223	-2.4341862	0.01492531	0.06275138
ENSG000002	168.897789	0.25125699	0.08107353	3.09912468	0.00194093	0.0154969
ENSG000002	143.562095	-0.3106087	0.15693842	-1.9791756	0.04779624	0.13706104
ENSG000002	339.167273	1.77830844	0.26315482	6.75765099	1.40E-11	3.03E-09
ENSG000002	535.270029	0.29384544	0.13913418	2.11195716	0.03469012	0.11075038
ENSG000002	502.233805	0.18400115	0.09507589	1.93530812	0.0529525	0.14716307
ENSG000002	699.497179	2.30827287	0.34020945	6.78485808	1.16E-11	2.65E-09
ENSG000002	183.427102	-0.1851846	0.09673689	-1.9143118	0.05558033	0.15179057
ENSG000002	166.346426	0.35923856	0.16364849	2.19518409	0.0281504	0.09618296
ENSG000002	4931.42314	0.51398112	0.16269555	3.1591591	0.00158225	0.01351259
ENSG000002	119.453642	0.39534998	0.20244058	1.95291861	0.05082924	0.14317936
ENSG000002	208.19786	-0.2088656	0.10136841	-2.0604606	0.03935453	0.12003277
ENSG000002	707.910738	-0.0962983	0.04904871	-1.9633205	0.04960894	0.14075694
ENSG000002	152.894639	-0.2611915	0.12386091	-2.1087481	0.03496633	0.1112855
ENSG000002	291.715433	-0.3292211	0.13954099	-2.3593144	0.01830874	0.07202697
ENSG000002	7222.82493	-0.3455729	0.09786689	-3.5310499	0.00041391	0.0051527
ENSG000002	611.783232	-0.2580461	0.07882945	-3.2734738	0.00106234	0.01015259
ENSG000002	22.1880285	2.3302438	0.61031941	3.81807257	0.0001345	0.00227124
ENSG000002	61.6001993	0.74870797	0.21615636	3.46373332	0.00053273	0.00619082
ENSG000002	581.578959	0.32206134	0.12261856	2.62653001	0.00862604	0.04336026
ENSG000002	82.7458127	0.57745811	0.19217964	3.00478306	0.0026577	0.01926541
ENSG000002	1533.45494	-0.176773	0.07718026	-2.290391	0.02199866	0.0819172

ENSG000002	1991.01315	-0.1850566	0.08395752	-2.2041695	0.02751242	0.09477337
ENSG000002	121.818424	-0.3155555	0.17408953	-1.8126046	0.06989282	0.17731958
ENSG000002	177.535057	-0.169727	0.07281051	-2.3310789	0.0197492	0.07600941
ENSG000002	1176.22373	-0.339794	0.1017219	-3.3404213	0.00083651	0.0085888
ENSG000002	2162.53103	-0.3527566	0.14658869	-2.4064379	0.01610894	0.06633309
ENSG000002	148.674714	1.08876784	0.21049963	5.1723028	2.31E-07	1.24E-05
ENSG000002	434.283463	-0.2233696	0.06944688	-3.2164102	0.00129805	0.01174622
ENSG000002	284.611413	-0.2583188	0.12789298	-2.0198048	0.04340364	0.1281059
ENSG000002	115.419602	0.88555824	0.19469742	4.54838192	5.41E-06	0.00017145
ENSG000002	77.8938268	-0.581977	0.21702761	-2.6815806	0.00732753	0.03877809
ENSG000002	15.3951324	0.85521422	0.35582443	2.40347244	0.01624019	0.06668931
ENSG000002	299.29364	-0.1870475	0.09765177	-1.915454	0.05543463	0.15157357
ENSG000002	231.051675	-0.473954	0.18177539	-2.6073604	0.00912433	0.0450366
ENSG000002	3539.74015	0.51619051	0.22155207	2.32988347	0.01981231	0.07609802
ENSG000002	121.250257	-0.3201204	0.13667961	-2.3421229	0.0191744	0.07434444
ENSG000002	156.899523	-0.3067524	0.13340825	-2.299351	0.02148501	0.08064187
ENSG000002	221.329463	-0.4359096	0.12093493	-3.6044973	0.00031276	0.0041158
ENSG000002	113.513202	0.50924477	0.19395416	2.62559347	0.00864981	0.04337763
ENSG000002	103.103689	0.19884613	0.10178386	1.95361161	0.05074717	0.14307829
ENSG000002	592.07339	1.12080887	0.29018128	3.8624437	0.00011226	0.00197077
ENSG000002	188.931471	0.26634073	0.12169041	2.18867469	0.02862049	0.09724207
ENSG000002	485.618652	-0.3436572	0.1501155	-2.2892855	0.02206277	0.08194327
ENSG000002	1727.04488	0.36869292	0.12508002	2.94765632	0.00320193	0.02201342
ENSG000002	135.768153	0.52664616	0.11931863	4.41377983	1.02E-05	0.00028608
ENSG000002	187.187943	-0.1693804	0.09559843	-1.7717905	0.07642934	0.18811346
ENSG000002	348.233592	0.38596412	0.1197316	3.22357772	0.001266	0.01150698
ENSG000002	4165.86301	-0.2937497	0.11763427	-2.4971441	0.01251981	0.0558909
ENSG000002	46.0207025	0.92496736	0.31232958	2.96151062	0.00306134	0.02129689
ENSG000002	87.798219	-0.3949219	0.18124248	-2.17897	0.02933389	0.09896869
ENSG000002	363.031947	0.60644947	0.13970776	4.340843	1.42E-05	0.00037297
ENSG000002	192.037869	-0.2045853	0.09271662	-2.2065662	0.02734438	0.09433323
ENSG000002	647.837802	0.18315524	0.06105277	2.99995	0.00270024	0.0194876
ENSG000002	137.763993	0.481774	0.13050458	3.69162525	0.00022283	0.00323334
ENSG000002	33.2275912	0.81444675	0.2802744	2.90589066	0.00366209	0.02413534
ENSG000002	462.360287	0.40974452	0.18970541	2.15989902	0.03078049	0.10220472
ENSG000002	129.945595	-0.2116061	0.09833991	-2.1517826	0.03141448	0.10365387
ENSG000002	219.679287	-0.2927984	0.13880471	-2.1094273	0.03490771	0.11118755
ENSG000002	799.596683	0.38167419	0.12823584	2.97634578	0.00291706	0.02056766
ENSG000002	220.391566	-0.2694431	0.10989227	-2.451884	0.01421105	0.06069772
ENSG000002	1973.38878	1.20865371	0.30179733	4.00485228	6.21E-05	0.00122891
ENSG000002	124.308825	-0.2958194	0.10959225	-2.6992731	0.00694911	0.0373869
ENSG000002	135.170986	-0.1779976	0.09806699	-1.8150608	0.06951457	0.17699229
ENSG000002	30.6289592	1.03073067	0.33294574	3.09579178	0.00196288	0.01557859

ENSG000002	3077.94887	-0.2527942	0.11015994	-2.2947922	0.02174504	0.08136773
ENSG000002	1906.99945	-0.218896	0.06422158	-3.4084497	0.00065333	0.00721878
ENSG000002	154.309667	0.30754463	0.13025363	2.36112131	0.01821977	0.0718418
ENSG000002	533.560122	0.46083301	0.24533769	1.87836208	0.06033165	0.16100452
ENSG000002	188.927598	-0.2181965	0.07871592	-2.7719482	0.00557219	0.03203619
ENSG000002	100.757682	-0.6470076	0.26808179	-2.4134707	0.0158014	0.06546122
ENSG000002	542.869804	0.84171476	0.20428298	4.12033724	3.78E-05	0.00083715
ENSG000002	171.092952	-0.3715261	0.10277275	-3.6150259	0.00030032	0.003997
ENSG000002	261.707224	0.358164	0.1248921	2.86778748	0.00413353	0.0262139
ENSG000002	22.1572323	0.74513455	0.38190042	1.95112264	0.05104246	0.14358264
ENSG000002	181.852446	-0.2491889	0.12440639	-2.003023	0.04517483	0.13175589
ENSG000002	237.820913	-0.2835871	0.10229762	-2.7721764	0.00556828	0.03203619
ENSG000002	477.132685	1.12208087	0.27562349	4.07106395	4.68E-05	0.00098128
ENSG000002	53.8116027	-0.4680688	0.2005631	-2.3337732	0.0196076	0.0755063
ENSG000002	118.404276	2.57098643	0.68611177	3.74718312	0.00017883	0.00278248
ENSG000002	200.426393	-0.2206171	0.08998579	-2.451688	0.01421879	0.06071497
ENSG000002	8.85380673	1.12460505	0.50894135	2.20969477	0.02712635	0.09393681
ENSG000002	223.566675	-0.3911377	0.13387174	-2.9217345	0.00348088	0.02328382
ENSG000002	135.138599	0.72228677	0.18930083	3.81554997	0.00013588	0.00228103
ENSG000002	261.289574	0.65247556	0.16020461	4.07276399	4.65E-05	0.00097539
ENSG000002	41.2387188	-0.8726424	0.48146159	-1.8124861	0.06991112	0.17731958
ENSG000002	132.832352	-0.5065645	0.16996472	-2.98041	0.00287863	0.02041666
ENSG000002	158.565304	-0.2488216	0.11154811	-2.2306215	0.02570621	0.09066209
ENSG000002	869.428853	-0.1872565	0.08516098	-2.1988529	0.02788838	0.09568947
ENSG000002	2143.52667	0.22824606	0.10666176	2.13990531	0.03236242	0.1057866
ENSG000002	142.319647	0.56507087	0.16761147	3.3713139	0.00074811	0.00796538
ENSG000002	184.21584	-0.2483083	0.09303991	-2.668837	0.00761144	0.03972143
ENSG000002	1970.54326	0.50952658	0.25186283	2.02303209	0.04306984	0.12739324
ENSG000002	122.398386	-0.1833071	0.10475226	-1.7499107	0.08013372	0.19420734
ENSG000002	46.0293425	0.98107779	0.27698863	3.54194244	0.00039719	0.00498298
ENSG000002	209.361447	1.25007457	0.24883169	5.02377568	5.07E-07	2.42E-05
ENSG000002	205.455127	-0.2273331	0.0794332	-2.8619403	0.00421056	0.02655784
ENSG000002	168.306734	-0.2546733	0.08208572	-3.1025289	0.00191875	0.01540442
ENSG000002	185.704532	-0.3607924	0.12005251	-3.0052883	0.00265329	0.01924195
ENSG000002	55.6855286	-0.9302212	0.29885924	-3.1125731	0.00185464	0.01506965
ENSG000002	961.666509	-0.3462636	0.13292089	-2.6050349	0.00918649	0.04527529
ENSG000002	3267.63313	0.31597509	0.11477535	2.75298739	0.00590542	0.03333257
ENSG000002	411.391091	0.6140258	0.23285119	2.63698801	0.00836458	0.04248463
ENSG000002	196.373468	-0.2850023	0.11675812	-2.4409636	0.01464813	0.06204739
ENSG000002	164.215837	-0.4172653	0.16272654	-2.5642116	0.01034105	0.04892157
ENSG000002	154.004021	-0.2523176	0.09943788	-2.5374394	0.01116667	0.05163561
ENSG000002	86.923424	-0.795743	0.25317487	-3.1430567	0.00167193	0.01400854
ENSG000002	333.404698	0.36855667	0.11301014	3.26127072	0.00110914	0.01048218

ENSG000002	2073.89293	-0.3919051	0.10008833	-3.9155924	9.02E-05	0.00166345
ENSG000002	289.954166	-0.9261863	0.2674255	-3.4633431	0.00053351	0.00619266
ENSG000002	107.04414	-0.450661	0.1599366	-2.8177478	0.00483618	0.02923998
ENSG000002	321.312237	0.42836626	0.11528188	3.71581591	0.00020255	0.00305186
ENSG000002	126.092637	0.43128147	0.18184131	2.37174638	0.01770424	0.07051164
ENSG000002	118.543125	-0.2648886	0.14847349	-1.7840803	0.07441062	0.18508964
ENSG000002	270.912136	0.18612244	0.09533374	1.95232486	0.05089965	0.14328978
ENSG000002	78.2607672	1.81962168	0.33957392	5.3585437	8.39E-08	5.46E-06
ENSG000002	1581.70619	-0.3135501	0.11336355	-2.765881	0.00567692	0.03244633
ENSG000002	641.87218	-0.4205918	0.17661266	-2.3814365	0.01724526	0.06939156
ENSG000002	86.5034645	-0.5908911	0.22072312	-2.6770693	0.00742693	0.03907127
ENSG000002	130.579726	0.41800222	0.13200341	3.16660153	0.00154231	0.01327524
ENSG000002	655.018289	-0.155439	0.07087604	-2.1931102	0.02829944	0.09649115
ENSG000002	127.795528	0.69326359	0.30207342	2.29501687	0.02173216	0.08133809
ENSG000002	330.948797	0.53149316	0.22348182	2.37823894	0.01739555	0.06978439
ENSG000002	36.3907971	0.96533264	0.38369186	2.51590598	0.01187268	0.05397184
ENSG000002	418.74899	-0.8113382	0.25038525	-3.2403592	0.00119379	0.01102795
ENSG000002	240.348505	-0.3364022	0.12792378	-2.629708	0.00854582	0.0431422
ENSG000002	74.7446882	0.96705047	0.27750329	3.48482529	0.00049246	0.005826
ENSG000002	84.8733317	0.58875244	0.15678545	3.75514717	0.00017324	0.00271569
ENSG000002	362.208951	0.21668334	0.10905645	1.98689154	0.04693442	0.13549008
ENSG000002	186.319293	0.34530794	0.18531075	1.86339938	0.0624061	0.16444027
ENSG000002	86.6533614	0.65796071	0.15920252	4.13285347	3.58E-05	0.00080253
ENSG000002	172.915368	-0.418813	0.13666766	-3.0644633	0.00218061	0.01673161
ENSG000002	34.8493407	0.45036968	0.20420061	2.20552563	0.02741723	0.09450501
ENSG000002	1662.13511	-0.2401647	0.10605336	-2.2645649	0.0235394	0.08567713
ENSG000002	32.137909	1.25113418	0.68851743	1.81714235	0.06919532	0.17645345
ENSG000002	104.857607	0.39955531	0.15650181	2.55303957	0.01067874	0.05011426
ENSG000002	353.215838	-0.1399529	0.06651341	-2.1041306	0.03536706	0.11196472
ENSG000002	118.468341	-0.2935585	0.13257424	-2.214295	0.02680849	0.09313406
ENSG000002	134.611824	0.3815274	0.19540777	1.95246795	0.05088267	0.14328064
ENSG000002	238.807188	-0.1955883	0.07743769	-2.525751	0.01154513	0.05290818
ENSG000002	58.2668886	0.51554041	0.20094893	2.5655295	0.01030185	0.04877833
ENSG000002	165.400402	-0.3435819	0.09522694	-3.608032	0.00030853	0.00406994
ENSG000002	457.147668	-0.9640256	0.26144173	-3.6873442	0.00022661	0.00326796
ENSG000002	192.178402	-0.2552543	0.08857195	-2.8818863	0.00395302	0.02538885
ENSG000002	1949.12696	0.59563775	0.18804931	3.16745508	0.00153779	0.01325721
ENSG000002	265.367807	0.14660545	0.06780585	2.16213586	0.03060771	0.1019129
ENSG000002	95.4355169	-0.3228287	0.17003841	-1.8985635	0.05762189	0.1557577
ENSG000002	158.567982	-0.2223701	0.07905752	-2.8127633	0.00491178	0.02952289
ENSG000002	287.543705	-0.2282665	0.10489776	-2.1760857	0.02954885	0.09939728
ENSG000002	292.223984	-0.4358255	0.16927913	-2.5745965	0.01003571	0.04797335
ENSG000002	663.02669	0.84772585	0.21240905	3.99100623	6.58E-05	0.00128328

ENSG000002	187.118434	-0.2889629	0.12141881	-2.3798862	0.01731799	0.06956468
ENSG000002	112.993016	-0.475603	0.18040898	-2.6362492	0.00838281	0.0425236
ENSG000002	113.575229	-0.4451644	0.18820348	-2.3653359	0.01801373	0.07125961
ENSG000002	273.428934	-0.2902465	0.11609338	-2.5001122	0.0124154	0.05565207
ENSG000002	141.234024	0.43069407	0.1861181	2.31409017	0.02066278	0.07842767
ENSG000002	83.5520318	0.3720276	0.16559531	2.24660704	0.02466515	0.08824667
ENSG000002	1132.78096	-0.2140926	0.10042822	-2.1317976	0.03302349	0.10720376
ENSG000002	622.028618	0.20217256	0.07911677	2.5553693	0.01060752	0.04982283
ENSG000002	79.9281464	0.33286981	0.15032837	2.21428478	0.02680919	0.09313406
ENSG000002	31472.5441	0.80543066	0.20812697	3.86990054	0.00010888	0.00192589
ENSG000002	290.052799	-0.2540773	0.12096777	-2.1003718	0.03569614	0.11264621
ENSG000002	136.809073	-0.4452884	0.13624371	-3.2683225	0.00108187	0.01028372
ENSG000002	86.0530478	0.61595365	0.19189343	3.20987351	0.00132793	0.01193103
ENSG000002	2737.97005	-0.4802078	0.14083208	-3.4097896	0.00065013	0.00719311
ENSG000002	509.771775	-0.2689351	0.10341719	-2.6004873	0.00930915	0.04561927
ENSG000002	165.898944	-0.4964348	0.18331463	-2.7081023	0.00676692	0.03666791
ENSG000002	332.67421	0.46249317	0.17668884	2.61755739	0.00885616	0.04405778
ENSG000002	302.909141	-0.2163167	0.11298717	-1.9145243	0.05555319	0.15174175
ENSG000002	359.902208	0.27525166	0.10881037	2.52964557	0.01141778	0.05248594
ENSG000002	189.604152	0.42555371	0.17190768	2.47547815	0.01330579	0.05827325
ENSG000002	168.341421	-0.1171639	0.06604582	-1.7739788	0.07606665	0.18747406
ENSG000002	556.82999	-0.3015285	0.12173463	-2.4769329	0.01325168	0.05810588
ENSG000002	181.884402	0.26235844	0.07847827	3.34307115	0.00082857	0.00852857
ENSG000002	129.931704	-0.2878887	0.11073229	-2.5998621	0.00932612	0.04562458
ENSG000002	146.731328	0.44400546	0.20566579	2.15886877	0.03086035	0.10239117
ENSG000002	1304.13316	0.60240467	0.13095232	4.60018318	4.22E-06	0.00013871
ENSG000002	136.690332	-0.3485885	0.14791244	-2.3567216	0.01843706	0.07238581
ENSG000002	368.113127	-0.2651523	0.09588038	-2.7654493	0.00568444	0.03246774
ENSG000002	88.4370991	0.41202004	0.14822373	2.77971712	0.00544063	0.03162352
ENSG000002	149.449779	-0.3798121	0.14261473	-2.6632039	0.00774005	0.04014981
ENSG000002	231.793425	-0.3516707	0.11353702	-3.0974101	0.0019522	0.01553891
ENSG000002	140.337411	-0.4298284	0.1210397	-3.5511356	0.00038357	0.00485296
ENSG000002	152.400467	0.6339282	0.16427189	3.85901811	0.00011384	0.00199646
ENSG000002	5.65045617	0.94271633	0.46115888	2.04423329	0.04093051	0.12298653
ENSG000002	245.916049	0.45266848	0.14954562	3.02695902	0.00247027	0.01825415
ENSG000002	341.639834	0.2141086	0.10922148	1.96031596	0.04995887	0.14148111
ENSG000002	118.818515	0.85190387	0.24045589	3.54286969	0.0003958	0.00497692
ENSG000002	134.808228	0.35471284	0.14118513	2.51239513	0.01199147	0.05436113
ENSG000002	208.724814	-0.3647836	0.11614381	-3.1407921	0.00168492	0.0140957
ENSG000002	1568.92409	0.41734381	0.12575193	3.3187866	0.00090409	0.00906694
ENSG000002	63.7713828	0.49097455	0.24747144	1.98396448	0.0472598	0.13604616
ENSG000002	362.124457	-0.3242918	0.11751435	-2.7595927	0.00578735	0.03288119
ENSG000002	188.283239	-0.287347	0.14251402	-2.0162715	0.04377159	0.12885507

ENSG000002	32.450443	1.97362221	0.41274999	4.78164084	1.74E-06	6.52E-05
ENSG000002	351.071094	-0.6990981	0.24255741	-2.8821966	0.00394913	0.02538635
ENSG000002	1082.63369	0.67344472	0.25611617	2.62945021	0.00855231	0.04315014
ENSG000002	222.524594	0.59553771	0.21922229	2.71659292	0.00659577	0.03601772
ENSG000002	69.8782473	-0.8333383	0.31345597	-2.6585498	0.00784778	0.04050361
ENSG000002	506.834123	0.63183446	0.22819905	2.76878647	0.00562655	0.0322808
ENSG000002	152.667451	0.59432362	0.14830179	4.00752832	6.14E-05	0.00122245
ENSG000002	277.375266	0.40002524	0.10646025	3.75750807	0.00017161	0.00270312
ENSG000002	157.443401	-0.318248	0.1463187	-2.1750332	0.02962763	0.09961127
ENSG000002	1618.16204	-0.314261	0.13463743	-2.3341283	0.01958901	0.07545241
ENSG000002	1928.43749	-0.1949107	0.07602206	-2.5638704	0.01035122	0.04895557
ENSG000002	181.280453	0.20509576	0.08147658	2.51723568	0.01182797	0.05379839
ENSG000002	321.646753	-0.1616158	0.0726569	-2.2243693	0.02612361	0.09151435
ENSG000002	57.3525309	0.57269992	0.25735341	2.22534417	0.02605814	0.09137625
ENSG000002	182.155107	-0.1461046	0.07619904	-1.9174068	0.05518627	0.15125213
ENSG000002	158.878648	-0.3090657	0.10006692	-3.0885899	0.00201109	0.01584614
ENSG000002	49.9445673	-0.5377863	0.25164883	-2.1370506	0.03259388	0.10620061
ENSG000002	47.014148	0.52272786	0.29882589	1.74927231	0.08024396	0.19435158
ENSG000002	153.740463	-0.2417497	0.1319136	-1.8326365	0.06685665	0.17231556
ENSG000002	64.0310385	1.15974377	0.51868296	2.2359396	0.02535573	0.08976116
ENSG000002	21.9392897	0.54960254	0.27416283	2.00465738	0.04499969	0.13142014
ENSG000002	363.929424	0.44012416	0.11286222	3.89965889	9.63E-05	0.00175826
ENSG000002	90.4781228	3.42454782	0.58444033	5.85953375	4.64E-09	4.53E-07
ENSG000002	164.549049	-0.1802043	0.08229795	-2.1896566	0.02854915	0.09710027
ENSG000002	310.153779	0.60558008	0.27236017	2.22345316	0.02618526	0.09166518
ENSG000002	670.163649	-0.6088499	0.16770751	-3.6304274	0.00028295	0.00381858
ENSG000002	177.190066	-0.2018437	0.09013758	-2.2392848	0.02513739	0.08929329
ENSG000002	176.01452	0.97830155	0.17251002	5.67098375	1.42E-08	1.16E-06
ENSG000002	121.093367	0.77588971	0.21406159	3.62460973	0.0002894	0.00388629
ENSG000002	131.629411	-0.4356782	0.11993168	-3.6327204	0.00028045	0.00379807
ENSG000002	1483.955	-0.4158634	0.14795843	-2.8106771	0.00494374	0.02966062
ENSG000002	236.286662	0.24089612	0.11476116	2.09910837	0.03580735	0.11289478
ENSG000002	65.762498	1.36662985	0.28204438	4.84544263	1.26E-06	5.10E-05
ENSG000002	181.975391	-0.3332002	0.11008767	-3.0266803	0.00247255	0.01825415
ENSG000002	327.877265	-0.3463768	0.10219115	-3.3894987	0.00070021	0.00757339
ENSG000002	418.686123	0.65381273	0.11997985	5.44935462	5.06E-08	3.56E-06
ENSG000002	296.809426	-0.369766	0.1767347	-2.0922095	0.03641978	0.11396472
ENSG000002	1239.62207	-0.3444533	0.17352417	-1.985045	0.04713946	0.13584284
ENSG000002	63.0265441	-0.8415641	0.26288599	-3.2012514	0.00136832	0.01214569
ENSG000002	140.125589	0.34173598	0.10918693	3.12982509	0.0017491	0.01442551
ENSG000002	207.177813	-0.5336293	0.25808505	-2.0676489	0.03867305	0.1185519
ENSG000002	638.215874	-0.2615147	0.08066696	-3.2419066	0.00118733	0.01100465
ENSG000002	916.085129	-0.282325	0.09203909	-3.0674468	0.00215896	0.01661993

ENSG000002	706.903774	-0.408626	0.14124405	-2.8930491	0.00381522	0.0247853
ENSG000002	14317.1491	0.37018152	0.1161677	3.18661329	0.00143949	0.01261536
ENSG000002	275.157126	-0.4230697	0.16147213	-2.620079	0.00879094	0.04381308
ENSG000002	97.6582992	-0.2289397	0.12080951	-1.8950473	0.05808614	0.15672839
ENSG000002	387.937776	-0.1699529	0.08110092	-2.0955734	0.03612005	0.11358565
ENSG000002	454.57135	0.31031216	0.16890352	1.83721541	0.0661781	0.17115494
ENSG000002	125.96482	-0.2079625	0.11755129	-1.7691212	0.07687365	0.18878197
ENSG000002	208.291103	0.2560245	0.13681874	1.87126787	0.06130796	0.16261188
ENSG000002	254.350017	0.10933661	0.05544136	1.97211291	0.04859672	0.13872634
ENSG000002	154.510317	-0.3540759	0.15081859	-2.3476943	0.01889002	0.07363753
ENSG000002	803.577486	-0.22082	0.07746759	-2.8504823	0.0043653	0.02716424
ENSG000002	291.184457	-0.2226505	0.08733779	-2.5493032	0.01079384	0.05043656
ENSG000002	118.610133	-0.183625	0.0980908	-1.8719896	0.06120805	0.16244323
ENSG000002	1526.34478	-0.261413	0.09578557	-2.7291479	0.00634982	0.0350212
ENSG000002	186.840149	0.66454222	0.21440459	3.09947759	0.00193862	0.0154969
ENSG000002	158.138703	-0.3187871	0.11419923	-2.7914997	0.00524644	0.030978
ENSG000002	603.062884	-0.3092267	0.17340575	-1.7832551	0.07454479	0.18528071
ENSG000002	26.9223396	1.9010936	0.54094305	3.51440618	0.00044074	0.00534526
ENSG000002	268.591996	0.28184203	0.1225846	2.29916339	0.02149566	0.08065547
ENSG000002	386.387253	-0.3480793	0.13890654	-2.5058523	0.01221566	0.05503128
ENSG000002	69.4248752	-0.9264665	0.28044666	-3.3035389	0.00095473	0.00941917
ENSG000002	583.343015	0.43993199	0.19096163	2.30377158	0.02123547	0.07991691
ENSG000002	48.4652465	2.02496119	0.40960277	4.94371951	7.66E-07	3.34E-05
ENSG000002	299.242517	1.01187655	0.23242018	4.35365195	1.34E-05	0.00035697
ENSG000002	64.6428058	0.65292303	0.2141038	3.04956301	0.00229175	0.01738101
ENSG000002	75.2827498	-0.6544125	0.21498516	-3.0439893	0.00233463	0.01760813
ENSG000002	70.5961001	-0.6320524	0.31658192	-1.9964891	0.04588071	0.13317506
ENSG000002	154.789216	-0.3789095	0.13446757	-2.8178502	0.00483464	0.02923998
ENSG000002	1189.20029	0.73333851	0.26944862	2.72162648	0.00649615	0.03559289
ENSG000002	147.379586	0.30617768	0.1164968	2.6282067	0.00858363	0.04323989
ENSG000002	39.9275529	0.69271749	0.24403466	2.83860288	0.00453115	0.02794181
ENSG000002	272.589253	-0.3119264	0.14805927	-2.1067669	0.03513779	0.11161457
ENSG000002	536.954525	0.34836792	0.11840155	2.94225817	0.00325828	0.02228871
ENSG000002	101.88282	-0.3120937	0.16731135	-1.8653467	0.06213282	0.16418351
ENSG000002	209.618656	-0.5616617	0.15970811	-3.5168015	0.00043678	0.00532258
ENSG000002	156.618422	-0.3748529	0.11182959	-3.3520012	0.0008023	0.00836083
ENSG000002	293.125467	0.33149901	0.11942667	2.77575359	0.00550739	0.03188569
ENSG000002	46.0339339	1.52615799	0.31134378	4.90184191	9.49E-07	4.02E-05
ENSG000002	50.766415	0.6220675	0.18912981	3.28910338	0.00100507	0.00977773
ENSG000002	81.9000454	-0.2807062	0.1550617	-1.8102869	0.07025131	0.17799352
ENSG000002	1382.56673	-0.4155145	0.18635402	-2.2297051	0.02576703	0.09082156
ENSG000002	286.270458	-0.5666333	0.22335131	-2.5369596	0.01118199	0.05167729
ENSG000002	15.4102448	1.40655469	0.53420951	2.63296451	0.00846432	0.04287595

ENSG000002	1766.72798	-0.2455933	0.10288169	-2.387143	0.01697989	0.06874548
ENSG000002	217.757004	-0.2186154	0.06534255	-3.3456814	0.00082081	0.00849329
ENSG000002	196.795608	-0.2779597	0.08329532	-3.3370381	0.00084676	0.00866697
ENSG000002	106.402244	-0.3105808	0.11034997	-2.8145076	0.0048852	0.02943912
ENSG000002	106.985945	-0.3457408	0.1745825	-1.9803864	0.04766013	0.13676639
ENSG000002	21.324317	0.73552676	0.33940509	2.16710586	0.03022678	0.10105929
ENSG000002	477.102804	0.36992815	0.18811067	1.96654527	0.04923566	0.14008625
ENSG000002	427.450731	0.1453151	0.05693687	2.55221445	0.01070406	0.05016491
ENSG000002	105.757416	0.66187744	0.2336862	2.8323343	0.00462095	0.02830397
ENSG000002	112.531576	-0.4358716	0.20579821	-2.117956	0.03417879	0.10957899
ENSG000002	252.870576	0.72850653	0.20724927	3.51512236	0.00043955	0.00533481
ENSG000002	486.345314	0.6407994	0.22305921	2.87277718	0.00406881	0.02593945
ENSG000002	2352.51895	0.29438545	0.10777797	2.7314064	0.00630647	0.03486416
ENSG000002	70.0279768	0.33039861	0.14502585	2.27820497	0.02271437	0.08331191
ENSG000002	85.7679791	-0.6213672	0.25628868	-2.4244816	0.01533026	0.06402708
ENSG000002	19.3387112	0.99925438	0.50609995	1.97442103	0.04833389	0.13809562
ENSG000002	144.232955	-0.3554864	0.12740151	-2.7902838	0.00526619	0.03103715
ENSG000002	2055.83728	0.29764078	0.11343703	2.62384141	0.00869443	0.0434907
ENSG000002	380.718972	0.35671978	0.13720354	2.59993131	0.00932424	0.04562458
ENSG000002	199.534244	-0.4235586	0.12870551	-3.2909123	0.00099863	0.00974356
ENSG000002	925.449198	-0.1741454	0.06510988	-2.6746378	0.007481	0.03926568
ENSG000002	66.0613113	0.78709391	0.15871022	4.95931466	7.07E-07	3.14E-05
ENSG000002	210.363965	-0.5870723	0.15378852	-3.8174001	0.00013487	0.00227509
ENSG000002	65.6111323	1.46984555	0.25593512	5.74303976	9.30E-09	8.11E-07
ENSG000002	208.708322	-0.1834563	0.09581079	-1.914777	0.05552095	0.1517295
ENSG000002	426.683457	-0.2875128	0.08906853	-3.2279958	0.00124661	0.01137486
ENSG000002	454.053932	0.23604845	0.121981	1.93512483	0.05297498	0.14717566
ENSG000002	288.644049	-0.2605997	0.11833275	-2.2022615	0.02764684	0.09515644
ENSG000002	194.311906	-0.3175479	0.12093166	-2.6258461	0.00864339	0.04337763
ENSG000002	2078.89176	0.38021492	0.14600374	2.60414507	0.00921038	0.04537937
ENSG000002	179.181368	-0.2470322	0.1169168	-2.1128893	0.03461024	0.11055994
ENSG000002	293.550994	0.51564648	0.18224148	2.82946821	0.00466254	0.02853741
ENSG000002	1243.54177	0.70525994	0.2710414	2.60203774	0.00926717	0.04552238
ENSG000002	279.262399	-0.2983863	0.12958912	-2.3025566	0.02130381	0.08008219
ENSG000002	45.3575703	0.70154646	0.24580809	2.85404139	0.00431669	0.02695384
ENSG000002	584.797872	-0.4634897	0.15604602	-2.9702118	0.00297594	0.02082912
ENSG000002	2066.2329	-0.3019345	0.15023374	-2.0097649	0.04445608	0.13016898
ENSG000002	186.740832	0.43726392	0.17921711	2.43985584	0.01469312	0.06218977
ENSG000002	143.741322	0.55671265	0.21010373	2.64970382	0.00805624	0.04123243
ENSG000002	507.890616	0.42298595	0.21208638	1.99440412	0.04610791	0.13369971
ENSG000002	126.414886	0.75281674	0.24796637	3.03596306	0.00239769	0.01791927
ENSG000002	171.038395	2.51695014	0.34314964	7.33484712	2.22E-13	1.04E-10
ENSG000002	147.124451	-0.2606368	0.12446774	-2.094011	0.036259	0.11376557

ENSG000002	1125.42708	2.18321798	0.29789515	7.32881349	2.32E-13	1.06E-10
ENSG000002	1173.71954	-0.2644383	0.11768972	-2.2469107	0.02464573	0.08819839
ENSG000002	102.687838	-0.3231048	0.13041504	-2.4775119	0.0132302	0.05808182
ENSG000002	2649.7434	0.47942057	0.18477596	2.5946047	0.00946998	0.04616802
ENSG000002	113.289581	-0.3879739	0.16039772	-2.4188245	0.01557075	0.06480041
ENSG000002	1003.62974	-0.1584512	0.05916698	-2.6780338	0.00740557	0.0390354
ENSG000002	194.271203	0.41654071	0.18706877	2.2266716	0.02596923	0.09121675
ENSG000002	240.165052	-0.3122543	0.13179299	-2.3692788	0.01782281	0.07077758
ENSG000002	198.315406	-0.669312	0.22914589	-2.920899	0.00349023	0.02332082
ENSG000002	312.042215	0.45712679	0.17499481	2.61223056	0.00899536	0.04458783
ENSG000002	75.1854057	0.89924482	0.31862931	2.82222886	0.00476911	0.02903573
ENSG000002	117.694917	0.30996289	0.11052608	2.80443218	0.00504053	0.03015307
ENSG000002	71.9806052	0.5756908	0.12929111	4.45267111	8.48E-06	0.00024788
ENSG000002	66.1593097	0.85292628	0.25318674	3.36876363	0.00075506	0.00802902
ENSG000002	37.8122362	0.52975889	0.28789957	1.84008225	0.06575616	0.17041312
ENSG000002	217.25805	0.876509	0.25909316	3.38298785	0.00071702	0.00770947
ENSG000002	3095.38869	-0.2195634	0.08704606	-2.5223813	0.01165633	0.05331347
ENSG000002	330.415988	-0.2353421	0.07969239	-2.9531318	0.00314568	0.0217269
ENSG000002	444.45165	-0.3381716	0.09809789	-3.4472871	0.00056625	0.00642988
ENSG000002	50.2914293	1.20477399	0.29862935	4.03434555	5.48E-05	0.00111391
ENSG000002	4826.66385	-0.1502796	0.05791518	-2.5948224	0.00946398	0.04616548
ENSG000002	175.274187	0.31256787	0.13145747	2.37771093	0.01742048	0.06982243
ENSG000002	3068.64244	0.52613055	0.10076865	5.22117289	1.78E-07	1.02E-05
ENSG000002	152.836964	-0.2396331	0.10704212	-2.2386802	0.02517673	0.08937453
ENSG000002	2966.67926	2.61512447	0.54842905	4.76839157	1.86E-06	6.87E-05
ENSG000002	108.015007	0.99673783	0.3885473	2.56529344	0.01030886	0.04879744
ENSG000002	220.109005	0.59905179	0.22804897	2.62685597	0.00861778	0.04334532
ENSG000002	563.976605	-0.1769752	0.07449361	-2.37571	0.01751522	0.07015073
ENSG000002	188.648156	0.73504527	0.15487588	4.74602795	2.07E-06	7.49E-05
ENSG000002	68.4869998	0.72791341	0.19488456	3.73510047	0.00018764	0.00288896
ENSG000002	155.929083	-0.2326738	0.09295855	-2.5029843	0.0123151	0.05532348
ENSG000002	83.3114644	0.54279746	0.17312738	3.13524903	0.00171708	0.01427844
ENSG000002	1742.34234	1.13828165	0.36984542	3.07772268	0.00208589	0.01623271
ENSG000002	2140.82706	0.34510311	0.18166248	1.89969393	0.0574733	0.15551777
ENSG000002	186.746481	-0.2957218	0.09932238	-2.977393	0.00290711	0.02054411
ENSG000002	102.752577	-0.2968437	0.14850847	-1.9988331	0.04562641	0.1326718
ENSG000002	6373.10709	0.64469359	0.11979215	5.38176835	7.38E-08	4.92E-06
ENSG000002	247.490846	-0.2210895	0.08885306	-2.4882601	0.01283698	0.05696615
ENSG000002	167.501601	-0.314713	0.11153355	-2.821689	0.00477715	0.02905447
ENSG000002	111.043899	0.26263714	0.11129586	2.35981058	0.01828427	0.07194796
ENSG000002	212.912804	-0.2409594	0.12248231	-1.9673	0.04914864	0.13986293
ENSG000002	2488.19477	-0.0837961	0.0473107	-1.7711873	0.07652955	0.18833185
ENSG000002	82.3979653	0.89356203	0.20738985	4.30861017	1.64E-05	0.0004157

ENSG000002	59.4321998	3.38043466	0.58443216	5.78413525	7.29E-09	6.60E-07
ENSG000002	550.268972	-0.1924922	0.10214384	-1.8845212	0.05949451	0.15960922
ENSG000002	174.56548	1.85313535	0.38577621	4.80365381	1.56E-06	5.96E-05
ENSG000002	240.546352	-0.3085224	0.14775567	-2.0880579	0.03679261	0.1147459
ENSG000002	51.1452509	-0.7272051	0.34008475	-2.1383054	0.03249197	0.10591864
ENSG000002	319.629597	0.81226382	0.285754	2.84252823	0.00447573	0.02768345
ENSG000002	113.020661	1.27345684	0.36721111	3.46791483	0.00052451	0.00611261
ENSG000002	714.51763	0.23787732	0.0713938	3.33190456	0.00086254	0.00880578
ENSG000002	159.908543	-0.2615501	0.09043184	-2.8922341	0.00382513	0.02482019
ENSG000002	129.306528	-0.1848442	0.06963988	-2.6542868	0.00794763	0.04086948
ENSG000002	175.668343	0.78361523	0.37069881	2.11388655	0.03452496	0.11037351
ENSG000002	210.463922	0.38028608	0.15780634	2.40982759	0.01596006	0.06594928
ENSG000002	177.200635	-0.3710021	0.16700382	-2.2215187	0.02631585	0.09198737
ENSG000002	76.5369552	-0.3445347	0.18932707	-1.8197859	0.06879162	0.17580675
ENSG000002	123.396229	-0.28301	0.13505571	-2.095506	0.03612604	0.11358565
ENSG000002	196.444141	-0.217815	0.06594062	-3.3031992	0.00095588	0.00942492
ENSG000002	1092.31632	-0.2482872	0.11785974	-2.1066332	0.03514939	0.11162977
ENSG000002	51.34689	0.87933103	0.39782394	2.21035221	0.02708073	0.09381844
ENSG000002	1506.33154	0.12274222	0.06288299	1.95191467	0.05094834	0.14339167
ENSG000002	94.9779012	-0.447271	0.15272733	-2.928559	0.00340537	0.02297633
ENSG000002	72.9910877	1.1385949	0.37794994	3.01255478	0.00259059	0.01889584
ENSG000002	227.417023	-0.4277642	0.13673953	-3.1283144	0.00175812	0.01447184
ENSG000002	784.784918	1.27565905	0.35340891	3.60958371	0.00030669	0.00404893
ENSG000002	417.917752	0.21553235	0.0952932	2.26178108	0.02371093	0.08605316
ENSG000002	204.482315	-0.4272642	0.13631322	-3.13443	0.00172188	0.01429556
ENSG000002	133.925735	-0.2595018	0.10250486	-2.5316045	0.0113542	0.0522816
ENSG000002	247.665069	-0.397155	0.17410505	-2.2811226	0.02254119	0.08289031
ENSG000002	8865.5695	0.25345355	0.08875673	2.85559816	0.00429558	0.02688347
ENSG000002	22.781793	-0.6936361	0.31428666	-2.2070173	0.02731285	0.09430372
ENSG000002	902.985448	-0.2761004	0.07680079	-3.5950207	0.00032437	0.00424133
ENSG000002	59.2177794	0.66393651	0.1879819	3.53191723	0.00041256	0.00514037
ENSG000002	346.946351	0.14328978	0.05959788	2.40427654	0.01620451	0.06657613
ENSG000002	269.189309	1.32896822	0.43919799	3.02589776	0.00247896	0.01829204
ENSG000002	270.172663	0.4587831	0.1448748	3.16675572	0.0015415	0.01327518
ENSG000002	122.343333	-0.3807847	0.12920977	-2.9470273	0.00320845	0.02202132
ENSG000002	5103.64212	0.22649854	0.12396758	1.82707885	0.06768791	0.17388042
ENSG000002	4017.36247	-0.2030112	0.08652626	-2.3462386	0.01896396	0.07377274
ENSG000002	278.252985	-0.1706864	0.06932103	-2.4622607	0.01380643	0.05966895
ENSG000002	219.933531	-0.2652025	0.14455734	-1.8345834	0.06656744	0.17191784
ENSG000002	87.9817258	1.06960909	0.43337371	2.4680987	0.01358329	0.05901958
ENSG000002	65.5328783	1.21588289	0.2700842	4.50186602	6.74E-06	0.00020416
ENSG000002	175.475519	-0.4072619	0.12412169	-3.2811503	0.00103385	0.00997585
ENSG000002	54.4198025	1.40451456	0.27801546	5.05192966	4.37E-07	2.15E-05

ENSG000002	95.0593023	-0.7898512	0.18439405	-4.2834959	1.84E-05	0.00045777
ENSG000002	86.7374201	-0.4496985	0.19511108	-2.3048332	0.02117592	0.07978436
ENSG000002	254.82409	-0.409479	0.09978428	-4.1036422	4.07E-05	0.0008786
ENSG000002	215.031516	-0.173481	0.09211058	-1.8833997	0.05964622	0.15973048
ENSG000002	107.375637	1.09328375	0.24174496	4.52246765	6.11E-06	0.00018839
ENSG000002	213.221251	1.92857461	0.43804526	4.40268338	1.07E-05	0.00029765
ENSG000002	205.388323	2.70967879	0.46006564	5.88976564	3.87E-09	3.94E-07
ENSG000002	18.4779388	0.91512627	0.3859715	2.37096853	0.01774154	0.07061227
ENSG000002	132.978087	-0.1918093	0.08781677	-2.1841991	0.02894762	0.09804879
ENSG000002	74.3845233	0.46132553	0.20862961	2.21121787	0.02702075	0.09368985
ENSG000002	168.361267	-0.2276544	0.06972571	-3.2649992	0.00109464	0.01037507
ENSG000002	184.70759	-0.308956	0.10980672	-2.8136352	0.00489848	0.02946454
ENSG000002	2357.02384	0.28049075	0.0740146	3.78966768	0.00015085	0.00247595
ENSG000002	76.8411477	0.77500486	0.25578459	3.02991222	0.00244625	0.01815805
ENSG000002	578.273065	-0.3403938	0.08750537	-3.8899765	0.00010025	0.00181042
ENSG000002	12.3356219	1.06998974	0.44362316	2.4119339	0.01586816	0.06570459
ENSG000002	852.782214	0.72994727	0.28892833	2.52639559	0.01152396	0.05287029
ENSG000002	696.255757	0.16713577	0.07680078	2.17622495	0.02953844	0.09939295
ENSG000002	771.685395	0.16053055	0.0769171	2.08705913	0.03688279	0.1149092
ENSG000002	62.991524	1.11404944	0.38803781	2.87098164	0.00409199	0.02606698
ENSG000002	259.206355	1.70760432	0.37416357	4.56379091	5.02E-06	0.00016089
ENSG000002	94.459764	0.32565224	0.17645493	1.84552648	0.06496099	0.16918128
ENSG000002	2615.27448	-0.2722601	0.09237354	-2.9473821	0.00320477	0.02202053
ENSG000002	208.566878	0.23699027	0.12876112	1.84054217	0.06568868	0.17033301
ENSG000002	209.062483	-0.3303841	0.11324017	-2.9175521	0.00352791	0.0234845
ENSG000002	168.829716	0.23108374	0.12979858	1.78032569	0.07502268	0.18596324
ENSG000002	1200.55481	1.42928457	0.37879995	3.77319096	0.00016117	0.00259153
ENSG000002	240.414234	-0.2847148	0.11554918	-2.4640137	0.01373909	0.05945627
ENSG000002	373.833137	0.23900921	0.12966228	1.84332106	0.06528215	0.16972116
ENSG000002	509.542417	0.77324575	0.13210123	5.85343361	4.82E-09	4.64E-07
ENSG000002	109.345339	0.40347304	0.22258074	1.81270416	0.06987746	0.17731958
ENSG000002	190.953285	-0.1938807	0.08341743	-2.3242234	0.02011352	0.07692253
ENSG000002	774.803906	0.8669859	0.2787061	3.11075328	0.00186611	0.01514031
ENSG000002	172.715045	0.66590165	0.19606528	3.39632628	0.00068297	0.00746079
ENSG000002	646.021535	-0.2949418	0.10723645	-2.7503881	0.00595247	0.03350999
ENSG000002	828.768461	0.10802135	0.05107785	2.1148374	0.03444381	0.11024305
ENSG000002	326.951067	-0.1977037	0.08382961	-2.3583997	0.01835392	0.07213675
ENSG000002	87.1764413	0.59461583	0.22350219	2.6604474	0.00780369	0.040356
ENSG000002	74.0660091	0.78597421	0.35618714	2.20663273	0.02733973	0.09433323
ENSG000002	110.511213	-0.3624734	0.16275152	-2.2271584	0.02593669	0.09118437
ENSG000002	238.833825	0.12727146	0.07272158	1.75011962	0.08009767	0.19420734
ENSG000002	193.096024	-0.2992826	0.1228909	-2.4353521	0.0148773	0.06260711
ENSG000002	12.30691	0.65651558	0.24274151	2.70458727	0.00683893	0.03694825

ENSG000002	14.0758961	0.91798824	0.27707413	3.31315027	0.00092251	0.00919542
ENSG000002	318.034704	1.24805084	0.37399981	3.33703604	0.00084677	0.00866697
ENSG000002	358.813074	-0.2692343	0.10468062	-2.571959	0.01011249	0.04820531
ENSG000002	650.898379	0.67150306	0.2094032	3.20674686	0.00134245	0.01202237
ENSG000002	219.452015	1.53623086	0.40193071	3.8221286	0.0001323	0.00224343
ENSG000002	865.694498	0.60785101	0.22269978	2.72946395	0.00634374	0.03499942
ENSG000002	30.4118341	0.73382209	0.2948924	2.48844012	0.01283048	0.05696615
ENSG000002	4120.52738	0.39159871	0.21058079	1.85961269	0.06294034	0.16538987
ENSG000002	1045.36728	-0.3066017	0.071317	-4.2991391	1.71E-05	0.00042923
ENSG000002	117.102214	0.8846912	0.25909219	3.41458075	0.0006388	0.0070917
ENSG000002	28035.1954	0.63010582	0.35499843	1.77495383	0.07590551	0.18733032
ENSG000002	3963.87703	-0.3654293	0.15322318	-2.384948	0.01708153	0.06896969
ENSG000002	213.024356	-0.2118025	0.11152097	-1.8992168	0.05753598	0.15562809
ENSG000002	2534.80537	-0.5160283	0.19496246	-2.6468085	0.00812554	0.04150605
ENSG000002	157.896853	-0.2703519	0.11895298	-2.2727628	0.02304048	0.08417886
ENSG000002	160.288969	0.36590547	0.17346914	2.10934042	0.03491521	0.11118755
ENSG000002	6709.15853	-0.3393433	0.13785951	-2.4615153	0.01383515	0.05974584
ENSG000002	454.355474	-0.1530296	0.0705814	-2.168129	0.03014887	0.10082623
ENSG000002	1922.99097	-0.1260651	0.05941876	-2.1216387	0.03386809	0.10900186
ENSG000002	666.457742	-0.3432807	0.13091924	-2.6220796	0.0087395	0.04362302
ENSG000002	949.455773	0.2680585	0.08129747	3.29725512	0.00097635	0.00958059
ENSG000002	1629.76859	-0.3281899	0.15231414	-2.1546909	0.03118603	0.10313177
ENSG000002	780.00125	-0.1693337	0.08077118	-2.0964619	0.03604124	0.113425
ENSG000002	521.774714	-0.31941	0.17622901	-1.8124709	0.06991346	0.17731958
ENSG000002	560.659094	-0.3468308	0.12513939	-2.7715561	0.00557891	0.03205232
ENSG000002	359.212458	-0.2313156	0.10159597	-2.276819	0.02279704	0.08355007
ENSG000002	2655.21052	-0.337874	0.10426925	-3.2403991	0.00119362	0.01102795
ENSG000002	219.052551	-0.5543596	0.2253374	-2.4601313	0.01388862	0.05987018
ENSG000002	83.0206536	0.51916341	0.20775046	2.49897597	0.01245528	0.05567861
ENSG000002	615.140188	0.75009523	0.20987852	3.57394956	0.00035164	0.00452219
ENSG000002	878.905445	-0.2813655	0.1328864	-2.1173384	0.03423113	0.10969082
ENSG000002	166.572213	-0.4740722	0.14704677	-3.2239552	0.00126433	0.01150604
ENSG000002	132.990499	-0.3852945	0.14586105	-2.6415178	0.00825355	0.04204207
ENSG000002	1942.92244	-0.3929247	0.15217988	-2.5819755	0.00982366	0.04720941
ENSG000002	61.2865938	-0.6876909	0.24264206	-2.8341785	0.00459437	0.02820435
ENSG000002	1503.58323	0.42856782	0.14585848	2.938244	0.00330077	0.02249235
ENSG000002	192.850581	-0.5905908	0.26925449	-2.1934298	0.02827643	0.0964528
ENSG000002	274.676466	-0.2622986	0.08509908	-3.0822732	0.00205426	0.01604674
ENSG000002	1043.85032	-0.2847555	0.12048837	-2.3633441	0.01811084	0.071523
ENSG000002	149.680808	0.52817321	0.17784774	2.96980552	0.00297988	0.0208452
ENSG000002	1795.21745	0.72167838	0.19463462	3.70786235	0.00020902	0.00311002
ENSG000002	124.331446	-0.3163533	0.17678076	-1.7895232	0.07353059	0.1836346
ENSG000002	694.325001	-0.327277	0.13638732	-2.3996142	0.01641236	0.0671776

ENSG000002	458.259092	0.45022727	0.13348445	3.37288191	0.00074386	0.00794079
ENSG000002	681.187925	-0.2725406	0.1250762	-2.1789968	0.02933191	0.09896869
ENSG000002	71.0668759	1.55064024	0.30407924	5.09946112	3.41E-07	1.76E-05
ENSG000002	715.255099	-0.4279216	0.11451145	-3.7369324	0.00018628	0.0028761
ENSG000002	913.45103	-0.3021785	0.12681813	-2.3827706	0.0171829	0.06921407
ENSG000002	6303.22286	-0.2061918	0.09218729	-2.236662	0.02530844	0.08968716
ENSG000002	247.548488	-0.141962	0.0667867	-2.1256029	0.03353634	0.10839648
ENSG000002	1920.73525	-0.5483581	0.23999423	-2.2848805	0.02231984	0.08242756
ENSG000002	55.8328073	0.47742598	0.19384198	2.46296475	0.01377935	0.05958332
ENSG000002	183.390719	-0.3938628	0.15097366	-2.608818	0.00908555	0.04488576
ENSG000002	332.64394	-0.2679416	0.12371425	-2.16581	0.03032571	0.10123181
ENSG000002	287.830377	0.45248365	0.17455761	2.5921737	0.00953716	0.04629391
ENSG000002	150.268068	0.56790822	0.20279772	2.80036784	0.00510444	0.03041334
ENSG000002	12.0413928	-2.5124646	0.86937783	-2.889957	0.00385295	0.02493163
ENSG000002	369.035148	-0.5226867	0.16738507	-3.1226602	0.00179225	0.01469372
ENSG000002	296.049996	0.27037876	0.08126695	3.32704451	0.00087772	0.00890596
ENSG000002	195.831075	-0.1819852	0.09331387	-1.9502477	0.0511466	0.14375229
ENSG000002	92.3851246	-0.436807	0.19966647	-2.1876832	0.02869269	0.09740662
ENSG000002	21.2032062	2.11161339	0.56158296	3.76010943	0.00016984	0.0026829
ENSG000002	239.792789	-0.2943432	0.10280545	-2.8631088	0.00419507	0.02651781
ENSG000002	254.570881	0.41348276	0.09528707	4.33933771	1.43E-05	0.00037434
ENSG000002	152.342201	0.35930646	0.17061236	2.10598148	0.03520596	0.11170127
ENSG000002	260.264315	-0.2191539	0.09325337	-2.3500909	0.01876882	0.07327439
ENSG000002	145.8144	0.20235504	0.10469482	1.93280861	0.05325978	0.14759413
ENSG000002	301.10936	-0.2909011	0.05977695	-4.8664422	1.14E-06	4.66E-05
ENSG000002	179.926969	-0.2937535	0.12808507	-2.2934252	0.02182353	0.08160559
ENSG000002	117.557479	0.20124622	0.11318399	1.77804494	0.07539647	0.1866077
ENSG000002	1115.63869	-0.2387442	0.13358524	-1.7872047	0.07390441	0.18427776
ENSG000002	137.036275	0.83083874	0.239833	3.46423867	0.00053173	0.00618358
ENSG000002	142.604149	-0.3244964	0.12924539	-2.5106998	0.01204921	0.0545324
ENSG000002	295.43674	0.29576899	0.09542313	3.09955238	0.00193813	0.0154969
ENSG000002	445.567165	-0.3359769	0.10467644	-3.2096706	0.00132887	0.01193292
ENSG000002	154.467981	-0.2515136	0.09494309	-2.6490986	0.00807068	0.04127727
ENSG000002	813.745446	0.53560724	0.23393642	2.28954191	0.02204789	0.08193124
ENSG000002	61.2385654	0.62549589	0.25413153	2.46130763	0.01384316	0.0597647
ENSG000002	81.9846426	0.98695509	0.32212869	3.06385341	0.00218506	0.01675791
ENSG000002	199.357924	0.32168275	0.16999584	1.89229772	0.05845132	0.15758408
ENSG000002	112.11196	-0.4147794	0.2302073	-1.8017646	0.07158245	0.18038073
ENSG000002	371.697156	-0.4277743	0.10491841	-4.0772094	4.56E-05	0.00096434
ENSG000002	144.256649	-0.2163716	0.09658573	-2.2402021	0.0250778	0.08915888
ENSG000002	184.095394	0.34286494	0.13907867	2.46525907	0.01369142	0.05934397
ENSG000002	286.887302	0.73270775	0.26435717	2.77165834	0.00557715	0.03205232
ENSG000002	310.292035	-0.5822523	0.19660158	-2.961585	0.0030606	0.02129689

ENSG000002	99.1458129	0.44624113	0.12189668	3.66081442	0.00025141	0.00353252
ENSG000002	81.8063352	0.72113985	0.23785307	3.03187112	0.00243043	0.01807336
ENSG000002	44.018393	0.66266435	0.35756977	1.8532449	0.06384726	0.16702355
ENSG000002	109.829857	-0.4150762	0.15162269	-2.73756	0.00618968	0.03445086
ENSG000002	132.781134	-0.4858276	0.13641133	-3.5614897	0.00036876	0.00470179
ENSG000002	404.0383	1.39985618	0.18859461	7.42256737	1.15E-13	6.49E-11
ENSG000002	1036.2201	-0.1708428	0.07172404	-2.3819457	0.01722144	0.0693127
ENSG000002	197.359869	-0.4235676	0.16571193	-2.5560479	0.01058686	0.04975428
ENSG000002	432.604349	0.56727113	0.21377879	2.65354257	0.00796517	0.0409093
ENSG000002	129.32145	-0.4637354	0.19361814	-2.3951029	0.0166157	0.06775187
ENSG000002	327.730036	-0.2861002	0.11906244	-2.4029426	0.01626374	0.06675258
ENSG000002	63.8805733	1.15277593	0.28748632	4.00984622	6.08E-05	0.00121199
ENSG000002	153.730959	0.27448474	0.10244736	2.67927594	0.00737816	0.0389754
ENSG000002	102.853658	-0.3935669	0.19758896	-1.9918465	0.04638791	0.13429069
ENSG000002	30.5441137	0.80898953	0.37009847	2.18587647	0.02882464	0.09771298
ENSG000002	19070.9232	1.66051621	0.42153186	3.93924246	8.17E-05	0.00153788
ENSG000002	168.679949	-0.3327721	0.15486859	-2.1487387	0.03165512	0.10414305
ENSG000002	395.366606	-0.5067917	0.13943509	-3.6346068	0.0002784	0.00378839
ENSG000002	193.498123	-0.3751198	0.2067156	-1.8146663	0.06957521	0.17706421
ENSG000002	331.642098	-0.1866654	0.09753496	-1.9138303	0.05564185	0.15185742
ENSG000002	129.923877	2.18977458	0.28090268	7.79549196	6.42E-15	7.01E-12
ENSG000002	112.459963	0.72866311	0.35804353	2.03512435	0.04183836	0.12475425
ENSG000002	119.094838	0.73684684	0.1288765	5.71746453	1.08E-08	9.14E-07
ENSG000002	71.4763386	0.93962515	0.19569559	4.80146302	1.58E-06	5.99E-05
ENSG000002	689.78275	-0.4488066	0.11183308	-4.0131828	5.99E-05	0.00119789
ENSG000002	339.09043	-0.1595724	0.08098216	-1.9704634	0.04878529	0.13905877
ENSG000002	624.150461	-0.3606672	0.19981783	-1.8049801	0.0710778	0.17945754
ENSG000002	51.1665873	0.64666694	0.24573591	2.63155242	0.00849958	0.04298814
ENSG000002	103.998466	0.22872085	0.12605736	1.81441889	0.06961326	0.17710589
ENSG000002	274.920987	0.23944285	0.08734809	2.74124885	0.00612061	0.03414757
ENSG000002	75.8786946	0.40751211	0.13710473	2.97226871	0.00295608	0.02077517
ENSG000002	2298.15793	1.41791353	0.37583401	3.77271216	0.00016148	0.00259153
ENSG000002	129.982245	-0.2714571	0.10575223	-2.5669166	0.01026073	0.04869614
ENSG000002	234.432253	-0.8625719	0.2622111	-3.2896087	0.00100327	0.00976876
ENSG000002	541.105938	-0.2160312	0.07640931	-2.8272883	0.0046944	0.02867889
ENSG000002	388.09388	-0.1548686	0.07846793	-1.9736554	0.04842095	0.13832025
ENSG000002	390.122412	-0.1133888	0.06104254	-1.8575373	0.06323475	0.16589764
ENSG000002	1260.55462	0.75321963	0.18066109	4.16924105	3.06E-05	0.0007098
ENSG000002	127.787105	0.80412024	0.1832015	4.38926675	1.14E-05	0.00031343
ENSG000002	87.3896985	0.64348209	0.31226663	2.06068156	0.03933343	0.12001307
ENSG000002	142.312609	-0.1801016	0.0742594	-2.4253037	0.01529559	0.06393434
ENSG000002	258.898599	1.05774722	0.27273318	3.87832241	0.00010518	0.00187663
ENSG000002	263.804602	-0.2563646	0.12289492	-2.0860474	0.03697433	0.11504874

ENSG000002	139.027102	-0.2317337	0.10537098	-2.1992174	0.02786247	0.09567237
ENSG000002	408.149683	0.45330853	0.14159057	3.20154458	0.00136693	0.01214168
ENSG000002	1273.56006	-0.3475975	0.1107873	-3.1375215	0.00170383	0.01421764
ENSG000002	1164.64592	0.7643788	0.23479461	3.25552114	0.00113184	0.01060506
ENSG000002	195.815178	-0.2348256	0.09875953	-2.3777509	0.01741859	0.06982243
ENSG000002	162.753751	-0.6919276	0.22160397	-3.1223611	0.00179407	0.01469397
ENSG000002	527.559275	-0.4158402	0.09819022	-4.2350472	2.29E-05	0.000551
ENSG000002	288.764584	0.74177228	0.25763103	2.87920398	0.0039868	0.02551586
ENSG000002	483.320262	-0.2816316	0.09314287	-3.0236517	0.00249744	0.01837993
ENSG000002	191.524506	-0.3049229	0.17109687	-1.7821655	0.07472225	0.18547089
ENSG000002	71.1505044	-0.3550417	0.19241174	-1.8452184	0.06500578	0.16927104
ENSG000002	825.450822	0.4351468	0.2128303	2.04457164	0.04089711	0.12293123
ENSG000002	158.575447	-0.6807667	0.1974649	-3.4475325	0.00056573	0.00642849
ENSG000002	97.3642899	-0.3377212	0.18415273	-1.8339191	0.06666601	0.17209107
ENSG000002	1284.19794	-0.2273094	0.10592966	-2.1458524	0.03188476	0.10464659
ENSG000002	904.7857	-0.3571986	0.08250929	-4.3291927	1.50E-05	0.00038767
ENSG000002	56.7430441	2.03161706	0.45991226	4.41740136	9.99E-06	0.00028274
ENSG000002	1240407.46	0.16807743	0.0798601	2.10464828	0.03532194	0.11191764
ENSG000002	583.097986	0.64950024	0.13822338	4.69891741	2.62E-06	9.05E-05
ENSG000002	308.815671	-0.1943192	0.08775716	-2.2142827	0.02680934	0.09313406
ENSG000002	162.44844	-0.2360496	0.09067577	-2.6032273	0.00923507	0.04543278
ENSG000002	163.031105	-0.200466	0.11107405	-1.8047958	0.07110664	0.17945754
ENSG000002	36.3956479	0.66692332	0.27230158	2.44920847	0.01431706	0.06102333
ENSG000002	594.581471	0.59750885	0.16531028	3.61446887	0.00030096	0.00399919
ENSG000002	256.285661	1.04926905	0.25227799	4.15917791	3.19E-05	0.00073452
ENSG000002	2700.03896	0.69030823	0.1777187	3.88427447	0.00010264	0.00184733
ENSG000002	110.94064	-0.6656015	0.19813948	-3.3592571	0.00078152	0.00821978
ENSG000002	360.498107	0.50216094	0.15576104	3.22391889	0.00126449	0.01150604
ENSG000002	466.581703	-0.2854876	0.09417437	-3.0314788	0.00243359	0.01808046
ENSG000002	111.342518	0.39042189	0.1895407	2.05983147	0.03941465	0.12014912
ENSG000002	43.5011307	1.51519843	0.30922266	4.90002394	9.58E-07	4.04E-05
ENSG000002	146.417218	-0.4312735	0.19286256	-2.2361702	0.02534063	0.08974304
ENSG000002	111.282772	-0.3335601	0.17115636	-1.9488619	0.05131192	0.14401943
ENSG000002	211.166575	1.06299459	0.30149639	3.52572907	0.00042232	0.00522229
ENSG000002	41.2529481	-0.959186	0.54914856	-1.7466786	0.08069311	0.19503611
ENSG000002	259.067797	-0.2597597	0.09578859	-2.7118021	0.00669185	0.03635729
ENSG000002	3985.60034	-0.0979831	0.05071674	-1.931968	0.05336345	0.14775625
ENSG000002	17242.0548	-0.1746781	0.07743588	-2.2557767	0.02408462	0.08701892
ENSG000002	166.172629	-0.3030656	0.10286575	-2.9462244	0.00321679	0.02206933
ENSG000002	332.366063	0.24478422	0.11167357	2.1919619	0.02838226	0.09667301
ENSG000002	275.364222	-0.4103938	0.15634317	-2.6249554	0.00866603	0.04340163
ENSG000002	466.376089	1.41147647	0.2749348	5.13385895	2.84E-07	1.50E-05
ENSG000002	28.1927512	1.39857118	0.31507509	4.43885035	9.04E-06	0.00025971

ENSG000002	26.4585811	2.31591157	0.52870884	4.38031556	1.19E-05	0.00032386
ENSG000002	564.104934	0.29314925	0.1217218	2.40835447	0.01602461	0.06608541
ENSG000002	778.960512	-0.2441675	0.13319727	-1.8331269	0.0667837	0.1722864
ENSG000002	225.443361	1.231991	0.25890469	4.75847308	1.95E-06	7.14E-05
ENSG000002	210.026615	-0.3148423	0.11656659	-2.7009655	0.00691385	0.03727932
ENSG000002	383.194639	0.29969423	0.14633083	2.04805937	0.04055418	0.12233897
ENSG000002	251.964643	-0.5169917	0.18962374	-2.7264082	0.00640278	0.03524213
ENSG000002	211.496966	-0.2983481	0.12501597	-2.3864803	0.01701052	0.06881853
ENSG000002	108.14582	-0.4797859	0.1572002	-3.0520696	0.00227269	0.01727648
ENSG000002	345.516138	0.82238549	0.39744219	2.06919527	0.03852777	0.11828797
ENSG000002	486.889923	-0.2247143	0.07942691	-2.8291967	0.0046665	0.02855097
ENSG000002	483.500753	-0.2302941	0.10887136	-2.1152867	0.03440552	0.11014201
ENSG000002	11633.5303	0.75311709	0.17999493	4.18410169	2.86E-05	0.00067255
ENSG000002	392.308151	0.65003486	0.17633931	3.68627318	0.00022756	0.00327597
ENSG000002	58.3436152	0.951128	0.17378022	5.47316601	4.42E-08	3.15E-06
ENSG000002	192.056341	0.41472901	0.17976029	2.30712255	0.02104799	0.079485
ENSG000002	243.776531	-0.3211767	0.1098358	-2.9241535	0.00345394	0.02315385
ENSG000002	347.05908	0.41048189	0.16786197	2.44535375	0.01447101	0.06145589
ENSG000002	457.937385	-0.275438	0.1322785	-2.0822585	0.03731887	0.11589346
ENSG000002	968.86955	0.25309281	0.09806653	2.58082753	0.00985638	0.04732506
ENSG000002	101.726267	0.41081835	0.2194745	1.87182721	0.06123051	0.16244323
ENSG000002	1157.05435	0.39595342	0.12112612	3.268935	0.00107953	0.0102707
ENSG000002	88.0398859	-0.9112259	0.29993634	-3.0380644	0.00238103	0.01784358
ENSG000002	149.343325	0.29073224	0.1612909	1.80253347	0.07146151	0.18015905
ENSG000002	18570.0631	0.12605129	0.06574137	1.91738172	0.05518946	0.15125213
ENSG000002	196.104116	0.20557102	0.08986247	2.28761809	0.02215977	0.08215615
ENSG000002	361.878008	-0.2798605	0.13901985	-2.0130971	0.04410441	0.1295673
ENSG000002	219.267756	0.34922745	0.13854928	2.52060101	0.01171546	0.05341427
ENSG000002	191.753215	-0.8750109	0.29519425	-2.9641869	0.00303484	0.02115742
ENSG000002	127.423059	-0.1656552	0.07837048	-2.1137451	0.03453704	0.11039062
ENSG000002	251.137408	0.24364784	0.11852312	2.05569886	0.03981155	0.12095424
ENSG000002	634.543081	-0.2161499	0.08258403	-2.6173324	0.008862	0.04407347
ENSG000002	3477.96174	0.4744726	0.16311764	2.90877543	0.00362847	0.02399036
ENSG000002	549.359112	0.26961862	0.14793711	1.82252192	0.06837583	0.17498962
ENSG000002	249.562982	0.34817892	0.14709277	2.3670703	0.01792953	0.0709779
ENSG000002	58.396013	-0.7625979	0.35638056	-2.1398413	0.03236759	0.1057866
ENSG000002	110.350685	-0.4301014	0.17295533	-2.4867775	0.0128906	0.05707997
ENSG000002	97.0975884	0.36015064	0.16298454	2.20972274	0.02712441	0.09393681
ENSG000002	5592.26809	0.2309093	0.1119121	2.06330956	0.03908323	0.11942744
ENSG000002	106.158759	0.41356461	0.12687907	3.25951805	0.00111602	0.01050478
ENSG000002	123.972373	-0.8402327	0.29151008	-2.8823454	0.00394727	0.02538635
ENSG000002	733.577547	-0.257313	0.12646674	-2.0346297	0.04188815	0.12485729
ENSG000002	304.66913	-0.336959	0.11771357	-2.8625333	0.00420269	0.02653952

ENSG000002	383.076348	1.48451396	0.23837299	6.22769366	4.73E-10	6.16E-08
ENSG000002	234.673293	-0.5076443	0.19932847	-2.5467725	0.01087243	0.05069754
ENSG000002	2934.85759	0.21191318	0.1012469	2.09303383	0.03634613	0.11392995
ENSG000002	124.982181	1.42682265	0.29956432	4.76299266	1.91E-06	7.03E-05
ENSG000002	44.7533606	0.99203836	0.29881183	3.31994343	0.00090036	0.00904051
ENSG000002	72.3643422	-1.195893	0.6333075	-1.8883291	0.05898178	0.15854496
ENSG000002	149.870693	-0.4335338	0.11830701	-3.6644812	0.00024784	0.00349427
ENSG000002	323.210389	-0.387963	0.11426004	-3.3954392	0.00068519	0.00747008
ENSG000002	10.2277002	1.4854793	0.68009619	2.18421941	0.02894613	0.09804879
ENSG000002	119.257866	0.54093653	0.17855815	3.02946983	0.00244983	0.01817644
ENSG000002	273.152101	0.76430128	0.21399205	3.57163403	0.00035476	0.0045481
ENSG000002	793.432477	-0.4438028	0.17997689	-2.4658875	0.01366743	0.0592713
ENSG000002	413.245467	0.59933686	0.14164287	4.231324	2.32E-05	0.00055774
ENSG000002	312.678865	-0.5372498	0.17803617	-3.0176445	0.00254748	0.01866442
ENSG000002	1182.12351	1.41124269	0.35374643	3.98941887	6.62E-05	0.00128833
ENSG000002	94.3281505	-0.643347	0.2789707	-2.3061454	0.02110251	0.07961754
ENSG000002	358.856028	0.37238823	0.14414489	2.58342998	0.00978233	0.04709612
ENSG000002	129.216989	-0.6253737	0.16579355	-3.7720027	0.00016194	0.00259567
ENSG000002	90.3281188	-0.6857035	0.31544161	-2.173789	0.02972098	0.09984326
ENSG000002	386.306972	0.14679865	0.0797463	1.84082079	0.06564782	0.17029384
ENSG000002	943.878489	-0.6001384	0.20924899	-2.8680586	0.00412999	0.0262139
ENSG000002	64.3854794	0.81057171	0.29395822	2.75743849	0.00582562	0.03301265
ENSG000002	600.643605	-0.4971999	0.19146525	-2.5968151	0.00940925	0.04595876
ENSG000002	486.279629	-0.2630655	0.11063095	-2.3778649	0.01741321	0.06982243
ENSG000002	1515.38195	0.22097545	0.12482485	1.77028418	0.07667981	0.18851853
ENSG000002	1047.86666	0.42855512	0.14055928	3.04892786	0.0022966	0.0174017
ENSG000002	1087.6962	1.55145883	0.29930907	5.1834675	2.18E-07	1.18E-05
ENSG000002	759.516894	-0.1271986	0.06476171	-1.9641018	0.04951829	0.14054898
ENSG000002	98.2948631	0.38126446	0.16726145	2.27945206	0.02264021	0.08312393
ENSG000002	120.119523	0.57761557	0.18354743	3.14695543	0.0016498	0.0138585
ENSG000002	114.243431	-0.44	0.15950822	-2.7584786	0.00580711	0.0329596
ENSG000002	1998.62005	-0.1443667	0.0812584	-1.7766371	0.07562798	0.1869831
ENSG000002	579.901915	0.63010812	0.26894506	2.34288785	0.01913513	0.0742273
ENSG000002	405.48718	-0.2802097	0.15698014	-1.7850008	0.07426119	0.18485816
ENSG000002	1930.50784	0.21193452	0.07759112	2.73142729	0.00630607	0.03486416
ENSG000002	580.417282	-0.4515316	0.20339317	-2.219994	0.02641917	0.09222805
ENSG000002	729.821607	0.41491568	0.2325753	1.78400581	0.07442272	0.18509166
ENSG000002	222.932755	-0.2662889	0.13093521	-2.0337455	0.04197726	0.12507128
ENSG000002	438.685933	0.37595468	0.15068535	2.49496509	0.01259695	0.05617412
ENSG000002	464.855475	-0.5773338	0.20310583	-2.8425268	0.00447575	0.02768345
ENSG000002	569.148588	0.27200295	0.09921243	2.74162169	0.00611367	0.03413145
ENSG000002	910.396831	-0.4449133	0.20620366	-2.1576402	0.03095581	0.10262485
ENSG000002	24.7172049	1.02544053	0.34282848	2.99111828	0.00277958	0.01990251

ENSG000002	150.241808	0.97792922	0.21798466	4.48622949	7.25E-06	0.00021771
ENSG000002	568.51576	1.78886289	0.33430238	5.35103245	8.75E-08	5.67E-06
ENSG000002	6991.11874	-0.2042816	0.09447725	-2.1622305	0.03060041	0.1019129
ENSG000002	42.7179993	1.1342557	0.2619298	4.33038045	1.49E-05	0.00038619
ENSG000002	925.050054	-0.2845531	0.11879345	-2.3953602	0.01660405	0.06772551
ENSG000002	1580.8464	0.34578703	0.14067748	2.45801265	0.01397083	0.06004233
ENSG000002	291.724043	-0.3729041	0.08440718	-4.417919	9.97E-06	0.00028271
ENSG000002	506.120922	-0.2711747	0.11006318	-2.4638095	0.01374691	0.05946813
ENSG000002	357.505118	0.41965788	0.21067347	1.99198257	0.04637297	0.13427116
ENSG000002	1962.4265	0.35653359	0.11885491	2.99973797	0.00270212	0.01949259
ENSG000002	2595.83766	-0.3774248	0.10031127	-3.7625361	0.0001682	0.00266727
ENSG000002	291.348699	-0.3592213	0.12770074	-2.8129931	0.00490827	0.02951262
ENSG000002	1406.33692	-0.3470331	0.09458226	-3.6691138	0.00024339	0.00344638
ENSG000002	798.36899	0.39195077	0.17965582	2.18167592	0.02913346	0.09849512
ENSG000002	69.6405348	0.82871177	0.2312941	3.58293521	0.00033975	0.00440392
ENSG000002	526.004711	0.13773455	0.07309689	1.88427372	0.05952796	0.15962157
ENSG000002	131.252085	-0.4148235	0.11043053	-3.7564204	0.00017236	0.00270969
ENSG000002	502.627668	0.47180203	0.12591795	3.7469006	0.00017903	0.00278248
ENSG000002	237.949199	2.38645542	0.31477956	7.58135443	3.42E-14	2.34E-11
ENSG000002	88.085669	-0.5267192	0.25718143	-2.0480453	0.04055556	0.12233897
ENSG000002	334.47848	0.75192974	0.31617834	2.37818235	0.01739822	0.06978439
ENSG000002	180.083902	0.29388142	0.10425348	2.8189123	0.00481867	0.02920021
ENSG000002	291.054215	-0.179018	0.06406238	-2.7944319	0.0051991	0.03079633
ENSG000002	315.916623	0.73500373	0.22169822	3.31533439	0.00091534	0.00914562
ENSG000002	93.8668816	0.40965475	0.22353395	1.83262874	0.0668578	0.17231556
ENSG000002	3359.68765	0.17038832	0.09720019	1.75296279	0.07960839	0.19344085
ENSG000002	110.331058	-0.410121	0.16491916	-2.4867999	0.01288979	0.05707997
ENSG000002	164.430645	-0.3531942	0.09863645	-3.5807676	0.00034259	0.00441619
ENSG000002	417.804835	0.38594966	0.1869559	2.0643888	0.03898087	0.11922576
ENSG000002	188.237135	0.35470457	0.18392614	1.92851634	0.05379093	0.1485584
ENSG000002	172.992732	0.55549718	0.19920279	2.78860136	0.00529362	0.0311444
ENSG000002	71.8138507	0.76952124	0.3484713	2.20827725	0.02722495	0.09413909
ENSG000002	292.64469	-0.1834561	0.05976964	-3.0693865	0.00214499	0.01655322
ENSG000002	423.279371	0.62109489	0.24740237	2.51046457	0.01205724	0.05453865
ENSG000002	4110.5376	0.34594567	0.11829809	2.92435543	0.0034517	0.02315385
ENSG000002	684.821564	-0.2334051	0.08771429	-2.6609706	0.00779158	0.04032781
ENSG000002	100.871915	0.26337529	0.12089942	2.17846617	0.02937135	0.0990543
ENSG000002	236.053574	0.33642702	0.14700615	2.28852347	0.02210706	0.0820483
ENSG000002	101.14782	1.00329616	0.26648477	3.76492876	0.0001666	0.00264955
ENSG000002	1179.79087	1.22419416	0.37295628	3.28240661	0.00102925	0.00995084
ENSG000002	117.942036	0.75794198	0.20302901	3.73317091	0.00018908	0.00290573
ENSG000002	141.326673	0.3712481	0.12900107	2.87786834	0.00400372	0.02560415
ENSG000002	555.009294	-0.3312404	0.09823195	-3.372023	0.00074618	0.00795384

ENSG000002	91.1230296	1.05380632	0.3546541	2.97136368	0.0029648	0.02077517
ENSG000002	135.853856	0.40544747	0.21980808	1.84455213	0.06510272	0.16944273
ENSG000002	106.404798	0.93478413	0.36197173	2.58247829	0.00980935	0.04716832
ENSG000002	2081.94286	-0.232006	0.06254016	-3.7097119	0.0002075	0.00309863
ENSG000002	2401.72855	-0.2348185	0.11052098	-2.1246509	0.03361575	0.10854994
ENSG000002	205.225881	-0.2693328	0.09143962	-2.9454711	0.00322463	0.02210464
ENSG000002	290.196039	-0.3387544	0.15684802	-2.1597623	0.03079108	0.10220472
ENSG000002	122.877873	-0.2799454	0.11253947	-2.4875304	0.01286335	0.05705174
ENSG000002	220.82005	-0.3781457	0.09438928	-4.0062355	6.17E-05	0.0012247
ENSG000002	193.632321	-0.3881733	0.10083257	-3.8496822	0.00011827	0.00204783
ENSG000002	499.195101	-0.1502241	0.07116132	-2.111036	0.03476921	0.11097973
ENSG000002	145.865126	0.51262986	0.17896473	2.86441829	0.00417776	0.02643872
ENSG000002	127.06488	0.5814227	0.20369478	2.85438186	0.00431207	0.02693522
ENSG000002	156.74337	-0.3362536	0.17448896	-1.9270768	0.05397006	0.14893085
ENSG000002	137.683291	-0.2653399	0.09479956	-2.7989573	0.00512679	0.03048006
ENSG000002	398.471714	-0.1947298	0.09687678	-2.0100769	0.04442305	0.130142
ENSG000002	74.7841296	0.8366343	0.22835627	3.6637238	0.00024857	0.00349861
ENSG000002	915.416102	-0.3140434	0.13213385	-2.3767065	0.01746798	0.06999572
ENSG000002	352.063999	0.36614543	0.19724023	1.85634254	0.06340475	0.16626382
ENSG000002	498.634756	-0.3178495	0.12481722	-2.5465192	0.01088032	0.05069754
ENSG000002	177.265903	-0.3158493	0.1132415	-2.7891658	0.0052844	0.0311125
ENSG000002	172.231954	0.23610182	0.10905744	2.16493081	0.03039298	0.10137383
ENSG000002	362.421902	-0.1939122	0.06808694	-2.848009	0.00439937	0.02734512
ENSG000002	95.3337414	-0.6791402	0.19354472	-3.5089577	0.00044987	0.00542959
ENSG000002	143.570733	1.16506377	0.32504147	3.58435425	0.00033791	0.00438699
ENSG000002	84.4101069	-0.3314774	0.17844122	-1.857628	0.06322186	0.16589036
ENSG000002	347.255024	0.60638383	0.32171151	1.88486832	0.05944762	0.15954451
ENSG000002	179.499968	-0.3500018	0.15222011	-2.2993139	0.02148712	0.08064187
ENSG000002	162.826699	-0.3080227	0.08594218	-3.584069	0.00033828	0.00438831
ENSG000002	291.917742	-0.2610109	0.12460488	-2.0947086	0.03619691	0.11363598
ENSG000002	115.788736	-0.629319	0.2087249	-3.0150645	0.00256925	0.01881551
ENSG000002	532.365927	0.23214904	0.11118041	2.08803909	0.03679431	0.1147459
ENSG000002	160.342964	-0.3148165	0.11651441	-2.701953	0.00689335	0.03719324
ENSG000002	132.817285	-0.2016228	0.11429519	-1.7640531	0.07772304	0.19013481
ENSG000002	914.534115	0.28623592	0.14195013	2.01645401	0.04375252	0.12882207
ENSG000002	430.153045	-0.3300863	0.11832793	-2.7895892	0.00527749	0.031083
ENSG000002	143.196613	0.74514497	0.24562139	3.03371371	0.00241564	0.01799599
ENSG000002	110.099054	0.40092997	0.14717674	2.72413951	0.00644693	0.03541408
ENSG000002	316.852568	0.32553129	0.1444037	2.2543141	0.02417641	0.08722127
ENSG000002	284.04875	-0.3032276	0.12584322	-2.4095663	0.01597149	0.06594928
ENSG000002	165.678378	0.90276285	0.30272523	2.98211963	0.0028626	0.02032831
ENSG000002	402.856004	-0.2854322	0.10289847	-2.7739209	0.00553851	0.03194338
ENSG000002	682.292807	-0.3272065	0.10129257	-3.2303104	0.00123656	0.01133249

ENSG000002	159.932827	0.43188045	0.1544742	2.79580956	0.00517699	0.0307228
ENSG000002	261.044247	0.2857547	0.11668922	2.44885249	0.01433121	0.0610678
ENSG000002	3508.41756	-0.1158481	0.06316269	-1.8341219	0.06663591	0.17204046
ENSG000002	763.833497	-0.8062104	0.1957198	-4.1192072	3.80E-05	0.000839
ENSG000002	307.387964	0.36659563	0.18473515	1.98443897	0.04720693	0.13593629
ENSG000002	42.4778035	1.22042919	0.33595113	3.63275804	0.00028041	0.00379807
ENSG000002	101.92985	0.26395541	0.12545829	2.10392953	0.03538459	0.11196472
ENSG000002	811.063238	0.25027717	0.1316402	1.90122138	0.05727302	0.15521647
ENSG000002	956.840069	-0.2097453	0.0847484	-2.4749176	0.01332669	0.05832195
ENSG000002	1161.51783	0.48485526	0.18714451	2.59080667	0.00957513	0.04639579
ENSG000002	44.1938039	1.20284105	0.27841361	4.32033862	1.56E-05	0.00039976
ENSG000002	225.838012	-0.5456492	0.1428565	-3.8195613	0.00013369	0.00226457
ENSG000002	97.8231887	0.33295365	0.13481479	2.4697116	0.0135222	0.05887508
ENSG000002	1297.15099	1.50423905	0.31641866	4.75395182	1.99E-06	7.27E-05
ENSG000002	129.206631	0.17582213	0.08284071	2.12241212	0.03380315	0.10893982
ENSG000002	725.420634	0.24724606	0.10867698	2.2750545	0.02290266	0.08382255
ENSG000002	546.275605	-0.3342464	0.0836186	-3.9972734	6.41E-05	0.00125527
ENSG000002	264.065205	-0.3614753	0.11668722	-3.0978142	0.00194954	0.01553826
ENSG000002	75.8516752	-0.3720521	0.21090684	-1.7640589	0.07772206	0.19013481
ENSG000002	103.50551	0.81485182	0.20947014	3.89006191	0.00010022	0.00181042
ENSG000002	205.987061	-0.1578126	0.0748328	-2.1088697	0.03495583	0.1112737
ENSG000002	259.171288	-0.384731	0.20367949	-1.8889041	0.05890468	0.15848825
ENSG000002	203.316034	-0.2610419	0.13820953	-1.8887399	0.05892669	0.1585008
ENSG000002	98.8392993	1.06101916	0.38056116	2.78803853	0.00530282	0.03116502
ENSG000002	180.146263	0.32519533	0.1463837	2.22152686	0.0263153	0.09198737
ENSG000002	6546.6061	-0.3408527	0.09169564	-3.7172181	0.00020143	0.00304127
ENSG000002	764.858689	1.85725543	0.30605055	6.0684597	1.29E-09	1.56E-07
ENSG000002	289.361312	-0.2866122	0.10272164	-2.7901836	0.00526782	0.03103715
ENSG000002	632.142368	0.17030848	0.09749095	1.74691575	0.08065195	0.19503142
ENSG000002	114.030065	-0.2344757	0.11088701	-2.1145458	0.03446868	0.11030112
ENSG000002	637.186683	-0.1443922	0.06525227	-2.2128296	0.02690939	0.09338523
ENSG000002	202.771829	-0.775761	0.17346058	-4.472261	7.74E-06	0.00022949
ENSG000002	104.707119	-0.2498795	0.14227245	-1.7563447	0.07902956	0.19231167
ENSG000002	3302.59308	0.28552693	0.1288897	2.21528115	0.02674077	0.09305358
ENSG000002	135.838382	-0.4833275	0.20599137	-2.346348	0.01895839	0.07376858
ENSG000002	166.403914	1.22784394	0.26118594	4.70103375	2.59E-06	8.99E-05
ENSG000002	64.2966241	-0.8784124	0.27921179	-3.1460436	0.00165495	0.01389466
ENSG000002	132.00169	-0.6063936	0.16289432	-3.7226198	0.00019717	0.00299623
ENSG000002	285.229533	0.62540802	0.16922081	3.69581042	0.00021919	0.00319467
ENSG000002	96.6009956	0.77573987	0.23605776	3.28622909	0.00101538	0.00985746
ENSG000002	247.645566	0.16108632	0.09141368	1.76216852	0.07804083	0.19062051
ENSG000002	281.921599	-0.2551365	0.1229786	-2.0746414	0.03801978	0.1172485
ENSG000002	291.077381	-0.3548651	0.11352747	-3.1258081	0.00177317	0.0145811

ENSG000002	125.505365	-0.401792	0.11400609	-3.5243032	0.0004246	0.00523469
ENSG000002	635.913022	-0.2425996	0.10380774	-2.3370091	0.01943871	0.07494392
ENSG000002	157.709193	-0.4074136	0.10973841	-3.7125888	0.00020515	0.00308044
ENSG000002	60.8375851	0.97782801	0.30178224	3.24017746	0.00119455	0.01102877
ENSG000002	206.402137	0.16713963	0.08406141	1.98830386	0.04677809	0.13515551
ENSG000002	491.104948	-0.4359003	0.23217601	-1.8774563	0.06045558	0.16110679
ENSG000002	370.86793	-0.2530198	0.09174361	-2.7579005	0.00581739	0.03298332
ENSG000002	128.480435	0.65873618	0.18472961	3.56594793	0.00036254	0.00462977
ENSG000002	67.1046124	0.59843348	0.2537397	2.35845429	0.01835122	0.07213675
ENSG000002	240.861138	-0.1731024	0.0700413	-2.4714331	0.01345727	0.05870148
ENSG000002	21116.8399	-0.2860737	0.10702532	-2.6729533	0.00751867	0.03936345
ENSG000002	134.428718	-0.2227119	0.10084782	-2.2083961	0.02721667	0.0941303
ENSG000002	339.039105	0.21450202	0.07443833	2.88160723	0.00395653	0.02540139
ENSG000002	1554.20657	-0.3025364	0.08864056	-3.4130693	0.00064236	0.00712633
ENSG000002	138.07901	-0.1827804	0.10214258	-1.789463	0.07354028	0.1836346
ENSG000002	755.390903	-0.1690842	0.07041932	-2.4011045	0.01634567	0.06698824
ENSG000002	45.014822	0.60953346	0.24446221	2.49336482	0.01265388	0.05638196
ENSG000002	31.2744248	2.60169708	0.43381446	5.99725769	2.01E-09	2.22E-07
ENSG000002	139.95267	-0.4421247	0.13216586	-3.3452265	0.00082215	0.00849329
ENSG000002	165.147228	-0.2460511	0.11471359	-2.1449169	0.03195949	0.10482891
ENSG000002	159.929169	-0.5094282	0.10778386	-4.7263867	2.29E-06	8.09E-05
ENSG000002	111.188572	-0.545857	0.1426427	-3.8267433	0.00012985	0.00220637
ENSG000002	602.88495	-0.1673893	0.09664764	-1.7319539	0.08328177	0.19972655
ENSG000002	109.944596	0.39815925	0.14431108	2.7590345	0.00579724	0.032926
ENSG000002	850.763097	-0.2078511	0.07591572	-2.7379192	0.00618293	0.03442494
ENSG000002	36.7407951	0.5803797	0.28992301	2.00184075	0.04530186	0.13205593
ENSG000002	112.227142	0.50198773	0.233168	2.15290149	0.03132642	0.10345606
ENSG000002	33.0741869	0.68702031	0.22900892	2.99997189	0.00270005	0.0194876
ENSG000002	3273.08824	0.3201281	0.12732799	2.51420054	0.01193026	0.05417891
ENSG000002	101.476411	-0.4049504	0.20039317	-2.0207794	0.0433026	0.12788775
ENSG000002	149.883189	-0.4963509	0.21093984	-2.353045	0.01862038	0.07283358
ENSG000002	394.268109	-0.189059	0.10010891	-1.8885337	0.05895434	0.1585204
ENSG000002	198.852394	-0.3486887	0.12509941	-2.787293	0.00531504	0.03120641
ENSG000002	997.673444	0.34960353	0.18492608	1.8905042	0.05869056	0.15813604
ENSG000002	195.813358	-0.2138452	0.08144661	-2.625587	0.00864997	0.04337763
ENSG000002	641.209808	0.34772195	0.09242666	3.76213919	0.00016847	0.00266893
ENSG000002	3097.35189	0.42174556	0.18697388	2.2556389	0.02409325	0.08701892
ENSG000002	121.640725	-0.3146718	0.16015431	-1.964804	0.04943693	0.14038502
ENSG000002	347.422584	0.2919574	0.13509467	2.1611319	0.03068515	0.10201631
ENSG000002	1239.44622	0.42352011	0.15431353	2.74454297	0.00605952	0.03392215
ENSG000002	572.957713	-0.3491325	0.14302584	-2.4410448	0.01464484	0.06204739
ENSG000002	161.649198	0.23232183	0.11606796	2.00160176	0.04532758	0.13207127
ENSG000002	202.604063	-0.3192128	0.12101554	-2.6377838	0.00834498	0.04240241

ENSG000002	1510.12096	-0.2717309	0.10954504	-2.4805405	0.01311834	0.05774533
ENSG000002	94.226496	0.59552769	0.26359418	2.25925964	0.02386724	0.08650963
ENSG000002	1463.92671	-0.1555079	0.07521031	-2.067641	0.03867379	0.1185519
ENSG000002	1422.81183	0.99188275	0.19041501	5.20905755	1.90E-07	1.07E-05
ENSG000002	274.565675	0.35151404	0.10706654	3.28313629	0.00102659	0.00994271
ENSG000002	424.520485	-0.1759575	0.09971027	-1.7646878	0.07761624	0.19009304
ENSG000002	130.093189	-0.2201189	0.12014571	-1.8320997	0.06693656	0.17243658
ENSG000002	398.680721	-0.3106555	0.11131445	-2.7907923	0.00525792	0.0310021
ENSG000002	124.698055	0.24025206	0.10582536	2.27026924	0.02319125	0.08463542
ENSG000002	506.314369	-0.4239372	0.20187484	-2.1	0.03572884	0.11270601
ENSG000002	562.220687	1.01517423	0.2659984	3.81646738	0.00013538	0.00227901
ENSG000002	791.179291	0.25465725	0.11368736	2.23997862	0.02509231	0.08919112
ENSG000002	10454.7988	0.69507452	0.22169375	3.13529143	0.00171683	0.01427844
ENSG000002	176.32536	0.52113582	0.12674985	4.11152997	3.93E-05	0.00085588
ENSG000002	184.146396	-0.1602451	0.07906117	-2.0268498	0.04267777	0.12649809
ENSG000002	1055.83367	-0.3504599	0.09977236	-3.5125948	0.00044375	0.00536991
ENSG000002	15.0113788	1.06456583	0.28695049	3.70992857	0.00020732	0.00309863
ENSG000002	2212.59336	-0.2120192	0.06697145	-3.1658146	0.00154649	0.01329725
ENSG000002	120.04072	-0.2045041	0.10685318	-1.9138789	0.05563562	0.15185742
ENSG000002	1242.05603	0.23605774	0.08851563	2.66684797	0.00765663	0.03989379
ENSG000002	225.665647	0.27262534	0.10812584	2.52137089	0.01168986	0.05338471
ENSG000002	46.2114554	0.54711097	0.22261407	2.4576657	0.01398433	0.06007363
ENSG000002	296.979124	-0.4833726	0.1878791	-2.5727855	0.01008837	0.04811949
ENSG000002	133.951661	0.26844269	0.13662957	1.96474806	0.0494434	0.14038502
ENSG000002	252.250859	-0.2506648	0.14406655	-1.7399236	0.08187243	0.19720443
ENSG000002	124.028266	-0.3166665	0.11862659	-2.6694393	0.0075978	0.03966289
ENSG000002	181.728533	0.69279272	0.26389178	2.62529106	0.00865749	0.04338536
ENSG000002	118.871064	0.23832663	0.10274828	2.31951927	0.0203669	0.07762808
ENSG000002	165.742973	-0.3392711	0.1598525	-2.1224008	0.0338041	0.10893982
ENSG000002	121.579919	-0.2497551	0.14078599	-1.7740053	0.07606227	0.18747406
ENSG000002	84.771229	0.66273557	0.27983163	2.36833685	0.01786826	0.07083797
ENSG000002	542.364785	-0.2913937	0.11010114	-2.6465997	0.00813055	0.04151874
ENSG000002	112.909311	-0.3206573	0.15989749	-2.0053932	0.04492103	0.13127252
ENSG000002	128.531087	-0.2352909	0.09151851	-2.5709649	0.01014156	0.04825628
ENSG000002	1903.22168	0.28892881	0.10321035	2.79941717	0.00511949	0.03045877
ENSG000002	161.122053	-0.3320907	0.09813984	-3.3838515	0.00071477	0.00769031
ENSG000002	161.678686	0.35147067	0.13063789	2.69041901	0.00713624	0.03812735
ENSG000002	626.184874	0.20145469	0.11236004	1.79293898	0.07298268	0.18283022
ENSG000002	1439.52385	0.27004398	0.12931203	2.08831292	0.03676962	0.1147459
ENSG000002	61.7578257	0.71905414	0.32120731	2.23859829	0.02518207	0.08937453
ENSG000002	478.673105	0.39203044	0.22172737	1.76807423	0.07704849	0.18903847
ENSG000002	751.24141	0.45680795	0.14868578	3.07230418	0.00212413	0.01647216
ENSG000002	148.512307	-0.3504965	0.1133387	-3.0924699	0.00198498	0.01571597

ENSG000002	728.770425	0.82660905	0.33977516	2.43281188	0.01498208	0.06294164
ENSG000002	5305.34364	0.19719308	0.07869378	2.50582823	0.0122165	0.05503128
ENSG000002	111.936305	-0.2887715	0.14919733	-1.9355001	0.05292895	0.14712257
ENSG000002	915.815755	0.36437582	0.11260607	3.23584523	0.00121283	0.01116608
ENSG000002	245425.844	0.42810142	0.15729834	2.72158886	0.00649689	0.03559289
ENSG000002	101.771784	0.31488021	0.13415929	2.34706238	0.01892209	0.07371477
ENSG000002	205.832856	0.43697616	0.18614419	2.34751443	0.01889914	0.07364287
ENSG000002	2304.52692	0.79774803	0.15214288	5.24341348	1.58E-07	9.30E-06
ENSG000002	330.692771	0.42137028	0.15425346	2.73167476	0.00630133	0.03485929
ENSG000002	364.999668	-0.1270822	0.05315459	-2.3908035	0.01681155	0.06823529
ENSG000002	319.853857	0.49873398	0.21852438	2.28228071	0.02247277	0.08273149
ENSG000002	83.1737466	-0.546878	0.17272567	-3.1661649	0.00154463	0.01328821
ENSG000002	1778.63338	-0.4386847	0.15211389	-2.8839225	0.00392755	0.02528633
ENSG000002	143.142135	-0.2845349	0.16337751	-1.7415793	0.08158208	0.19663403
ENSG000002	85.1607285	0.32370444	0.14725558	2.19824904	0.02793136	0.09573382
ENSG000002	90.036774	0.60478183	0.22263296	2.71649728	0.00659767	0.03601772
ENSG000002	309.043476	0.56936881	0.12572787	4.52858062	5.94E-06	0.00018385
ENSG000002	98.1655673	0.51843097	0.13545683	3.82727811	0.00012957	0.00220387
ENSG000002	4072.80653	0.2144072	0.11349956	1.8890576	0.05888411	0.15846426
ENSG000002	237.911079	-0.2744904	0.14444078	-1.9003665	0.05738503	0.15537037
ENSG000002	154.961489	-0.4158416	0.12485733	-3.3305344	0.00086679	0.00882308
ENSG000002	51.4317214	1.35078177	0.24817833	5.44278687	5.25E-08	3.64E-06
ENSG000002	287.931495	-0.3382504	0.12064658	-2.8036471	0.00505282	0.03020454
ENSG000002	54.8438591	-0.4837622	0.23711745	-2.0401795	0.04133245	0.12387648
ENSG000002	191.458768	-0.3274113	0.14695396	-2.2279858	0.02588146	0.09106831
ENSG000002	312.833706	0.20680813	0.10443237	1.98030683	0.04766906	0.13676808
ENSG000002	545.96981	-0.2034449	0.07322252	-2.7784468	0.00546195	0.03169127
ENSG000002	340.557702	0.92479476	0.17707069	5.22274338	1.76E-07	1.02E-05
ENSG000002	197.855351	-0.1768816	0.09480852	-1.8656716	0.06208732	0.16413272
ENSG000002	1734.99068	0.3514572	0.0817573	4.29878703	1.72E-05	0.00042926
ENSG000002	125.617798	-0.2794684	0.15987672	-1.7480246	0.08045976	0.19475918
ENSG000002	1162.67346	-0.2778981	0.12269626	-2.2649274	0.02351714	0.08563413
ENSG000002	179.425886	-0.3199282	0.14736153	-2.1710429	0.02992793	0.10031317
ENSG000002	410.93362	-0.3467912	0.12322262	-2.8143467	0.00488765	0.02943912
ENSG000002	58.5210493	-0.6033021	0.29628359	-2.0362319	0.04172707	0.12464908
ENSG000002	144.123299	-0.244032	0.10096866	-2.416908	0.01565297	0.06499413
ENSG000002	79.4988054	0.40467048	0.22746903	1.77901352	0.07523754	0.18635499
ENSG000002	113.99058	0.6969224	0.23063686	3.02173037	0.00251334	0.01846384
ENSG000002	151.623349	0.36895087	0.15316534	2.40884051	0.01600329	0.0660473
ENSG000002	326.143531	0.1868175	0.0675731	2.76467287	0.00569799	0.03249737
ENSG000002	81.4288971	1.31234187	0.35178668	3.73050471	0.0001911	0.00292842
ENSG000002	368.469955	0.690469	0.22053614	3.13086559	0.00174292	0.01439005
ENSG000002	314.110371	-0.1876338	0.08704048	-2.1557076	0.0311065	0.10295787

ENSG000002	42.4622877	2.16384773	0.36219071	5.97433241	2.31E-09	2.51E-07
ENSG000002	349.357304	-0.4310497	0.11070458	-3.8936935	9.87E-05	0.0017888
ENSG000002	435.534664	-0.2668012	0.08853692	-3.0134459	0.00258299	0.01884882
ENSG000002	86.9776136	0.49289102	0.19513071	2.52595312	0.01153849	0.05290483
ENSG000002	153.71013	-0.4508878	0.11019328	-4.0917907	4.28E-05	0.0009199
ENSG000002	783.700465	-0.1740548	0.09779592	-1.7797755	0.0751127	0.18610199
ENSG000002	67.8839146	0.51193502	0.13851341	3.69592372	0.00021909	0.00319467
ENSG000002	131.212978	0.94615735	0.18907444	5.00415264	5.61E-07	2.60E-05
ENSG000002	226.305787	-0.1301422	0.06688457	-1.9457734	0.05168196	0.14463716
ENSG000002	1311.45835	-0.2011416	0.08484403	-2.3707216	0.0177534	0.07062843
ENSG000002	133.567953	0.32060344	0.1157876	2.76889279	0.00562471	0.0322808
ENSG000002	105.162642	-0.3307641	0.12615172	-2.6219545	0.00874271	0.04362575
ENSG000002	1772.21242	0.11944167	0.04644687	2.57157646	0.01012367	0.04820563
ENSG000002	128.795454	-0.4070645	0.1225202	-3.3224276	0.00089238	0.00898098
ENSG000002	185.310292	-0.2751911	0.1405814	-1.9575215	0.05028619	0.14218703
ENSG000002	1590.46198	-0.2117915	0.09900668	-2.1391641	0.03242238	0.10586017
ENSG000002	151.814877	-0.3571618	0.13764727	-2.5947616	0.00946566	0.04616548
ENSG000002	415.003097	-0.1892946	0.09224897	-2.0519966	0.04016999	0.12170838
ENSG000002	375.792911	-0.360347	0.18131561	-1.9874021	0.04687786	0.13537446
ENSG000002	118.024657	1.07453441	0.20172779	5.32665536	1.00E-07	6.36E-06
ENSG000002	36.9116864	0.72662121	0.32747095	2.21888754	0.02649438	0.09243155
ENSG000002	63.7798998	0.4804603	0.2734172	1.75724237	0.07887651	0.19203238
ENSG000002	178.446296	-0.5189276	0.20048506	-2.5883607	0.0096434	0.0466342
ENSG000002	132.21585	0.1574544	0.07923274	1.98723921	0.04689589	0.13540271
ENSG000002	101.865222	-0.6060063	0.18513807	-3.2732667	0.00106312	0.01015259
ENSG000002	256.947805	-0.3013918	0.1314956	-2.2920296	0.02190394	0.08171987
ENSG000002	1387.86338	0.4343078	0.1385689	3.1342371	0.00172302	0.01429772
ENSG000002	50.4212075	0.30821252	0.1524059	2.0223135	0.04314397	0.12757544
ENSG000002	1685.97903	-0.1677012	0.08766016	-1.9130839	0.05573732	0.15206735
ENSG000002	378.243063	0.74428525	0.30236377	2.46155567	0.01383359	0.05974584
ENSG000002	1422.80649	-0.2062113	0.07293392	-2.8273716	0.00469318	0.02867889
ENSG000002	154.917474	0.88680287	0.33138476	2.67605203	0.00744951	0.03917562
ENSG000002	23.2584835	1.28254054	0.38346535	3.34460606	0.000824	0.00849329
ENSG000002	173.0029	-0.3648356	0.11195089	-3.25889	0.00111849	0.01050996
ENSG000002	5457.39702	-0.3647492	0.09819241	-3.7146371	0.0002035	0.0030584
ENSG000002	358.608414	-0.2812994	0.11220304	-2.5070569	0.01217411	0.0549309
ENSG000002	1231.43965	0.78018077	0.364854	2.13833692	0.03248941	0.10591864
ENSG000002	276.704662	0.20069322	0.10977023	1.82830276	0.06750413	0.17351703
ENSG000002	912.769437	-0.8744951	0.24414286	-3.5818993	0.00034111	0.0044062
ENSG000002	36.1270124	1.31754791	0.3350485	3.93240949	8.41E-05	0.00156879
ENSG000002	222.329124	-0.3184098	0.13409829	-2.3744505	0.01757509	0.070219
ENSG000002	2074.37885	0.22875078	0.11709253	1.95358995	0.05074973	0.14307829
ENSG000002	309.140782	-0.2228995	0.09852987	-2.2622527	0.0236818	0.08597283

ENSG000002	480.735402	-0.3960865	0.17315804	-2.2874279	0.02217086	0.08215615
ENSG000002	660.089093	0.30315562	0.11381867	2.66349632	0.00773333	0.04014981
ENSG000002	55.5024563	0.55707027	0.18515862	3.00861102	0.00262445	0.01908341
ENSG000002	516.579077	0.23092321	0.08200139	2.81608897	0.00486122	0.02936972
ENSG000002	2005.50812	-0.1257626	0.05428075	-2.3168913	0.02050965	0.07800899
ENSG000002	676.093422	-0.209014	0.09472061	-2.2066364	0.02733947	0.09433323
ENSG000002	325.678645	-0.3035922	0.08853044	-3.4292408	0.00060527	0.00677913
ENSG000002	125.212155	-0.2035754	0.1036657	-1.9637686	0.04955693	0.14063431
ENSG000002	685.540027	-0.2514694	0.10310771	-2.4389	0.01473205	0.06224204
ENSG000002	14512.025	-0.2683388	0.08371564	-3.2053608	0.00134893	0.01204709
ENSG000002	171.102639	-0.2714305	0.15218704	-1.7835325	0.07449967	0.18519877
ENSG000002	634.766574	-0.2767565	0.13270404	-2.0855167	0.03702243	0.11513342
ENSG000002	31.9748374	1.45887675	0.35714764	4.08480021	4.41E-05	0.00094055
ENSG000002	137.191366	0.37493217	0.15705835	2.38721581	0.01697652	0.06874548
ENSG000002	176.86141	-0.242538	0.09006557	-2.6929044	0.00708326	0.03791843
ENSG000002	219.069495	-0.2156404	0.1232211	-1.7500286	0.08011338	0.19420734
ENSG000002	541.374526	0.25703023	0.13196948	1.94764911	0.05145696	0.14425369
ENSG000002	154.494691	-0.3191337	0.13895987	-2.2965888	0.02164224	0.08107557
ENSG000002	309.249274	-0.3051481	0.10049101	-3.0365711	0.00239286	0.01789949
ENSG000002	391.899104	-0.2488793	0.09867886	-2.5221139	0.01166519	0.05333915
ENSG000002	112.014465	0.64924524	0.23754705	2.73312273	0.0062737	0.03476505
ENSG000002	794.393996	0.13909655	0.07618047	1.82588198	0.06786804	0.17409766
ENSG000002	167.694954	-0.188931	0.10725258	-1.7615521	0.07814501	0.19084655
ENSG000002	64.9146477	0.4183962	0.21652421	1.93232984	0.05331881	0.14770544
ENSG000002	274.59902	-0.1470929	0.08082436	-1.8199085	0.06877294	0.17578643
ENSG000002	217.894558	-0.3827135	0.12804104	-2.988991	0.002799	0.01998922
ENSG000002	177.228717	-0.3450962	0.10679633	-3.231349	0.00123207	0.01131149
ENSG000002	161.474822	-0.3022634	0.16395579	-1.8435666	0.06524633	0.16972116
ENSG000002	1467.73617	-0.4146673	0.11963687	-3.4660495	0.00052817	0.00614644
ENSG000002	135.583729	-0.3198454	0.10567255	-3.0267598	0.0024719	0.01825415
ENSG000002	221.493526	0.30540085	0.14369081	2.12540282	0.03355301	0.10842901
ENSG000002	235.102584	-0.2107222	0.10074888	-2.0915586	0.03647801	0.1140642
ENSG000002	265.134116	-0.6159442	0.16433657	-3.7480656	0.0001782	0.00277757
ENSG000002	216.598322	-0.1886903	0.09081671	-2.0777045	0.03773658	0.11673767
ENSG000002	120.476276	0.33478541	0.14461987	2.31493372	0.02061656	0.0782885
ENSG000002	158.60202	-0.1797754	0.09073931	-1.9812299	0.04756549	0.1366383
ENSG000002	1135.67861	-0.1921058	0.06888131	-2.7889389	0.0052881	0.03112313
ENSG000002	269.475158	0.2297135	0.07457693	3.08022202	0.00206846	0.01612772
ENSG000002	39.6089662	0.39948452	0.22509072	1.77477118	0.07593568	0.18736198
ENSG000002	161.34194	0.72200487	0.16222273	4.45070104	8.56E-06	0.00024928
ENSG000002	130.111997	1.45587639	0.32349306	4.50048723	6.78E-06	0.00020511
ENSG000002	259.160283	-0.2177795	0.10366569	-2.1007864	0.03565972	0.11258243
ENSG000002	80.6121247	-0.4938041	0.24031299	-2.0548373	0.03989472	0.12109474

ENSG000002	115.772669	-0.3912056	0.17062652	-2.2927598	0.02186184	0.08171155
ENSG000002	228.755547	-0.3236327	0.15787728	-2.0499003	0.04037416	0.12200796
ENSG000002	272.497164	-0.251832	0.09065681	-2.7778605	0.00547181	0.03171484
ENSG000002	1864.72113	-0.1973864	0.10144149	-1.9458151	0.05167695	0.14463716
ENSG000002	107.593043	0.62496696	0.18587315	3.36233054	0.00077288	0.00815646
ENSG000002	43.0062417	1.0266876	0.3178423	3.23017927	0.00123713	0.01133249
ENSG000002	222.826964	-0.2589543	0.13248887	-1.9545361	0.05063785	0.14291031
ENSG000002	161.854628	-0.2424217	0.11621027	-2.0860609	0.03697311	0.11504874
ENSG000002	96.4817621	0.35797714	0.17459424	2.05033762	0.0403315	0.12194644
ENSG000002	2055.05565	-0.1252743	0.06480821	-1.9330005	0.05323613	0.14757614
ENSG000002	1230.52195	0.4248422	0.16859421	2.51990976	0.01173849	0.05346557
ENSG000002	651.304112	0.20700221	0.09349568	2.21402963	0.02682674	0.09315503
ENSG000002	1210.57209	0.56117848	0.13641286	4.11382396	3.89E-05	0.00085194
ENSG000002	395.673748	-0.1695932	0.06716529	-2.5250129	0.0115694	0.05298981
ENSG000002	415.770671	2.09922978	0.35189371	5.96552238	2.44E-09	2.63E-07
ENSG000002	188.231109	-0.3498635	0.09586435	-3.6495688	0.00026268	0.00363606
ENSG000002	975.709047	-0.3062592	0.12812102	-2.3903899	0.0168305	0.06827552
ENSG000002	90.1278922	0.35973919	0.11556377	3.11290627	0.00185255	0.01506011
ENSG000002	42.0557072	0.96199732	0.31813952	3.02382211	0.00249603	0.01837783
ENSG000002	356.1163	-0.1552417	0.07838519	-1.9804986	0.04764753	0.13675418
ENSG000002	65.9795623	2.78305395	0.40241266	6.91592043	4.65E-12	1.23E-09
ENSG000002	675.218651	-0.4112145	0.13278207	-3.0969125	0.00195548	0.01555746
ENSG000002	95.9286672	0.42306464	0.14567064	2.90425471	0.00368129	0.02418754
ENSG000002	203.172246	-0.2408262	0.07729074	-3.1158482	0.00183417	0.01493985
ENSG000002	113.147139	-0.2291362	0.12841555	-1.7843338	0.07436944	0.18504336
ENSG000002	365.061323	-0.2261606	0.09779165	-2.3126786	0.02074032	0.07863098
ENSG000002	106.405644	0.37087502	0.20022912	1.85225321	0.06398947	0.16723875
ENSG000002	131.196942	0.19420559	0.10227537	1.89884999	0.0575842	0.15570715
ENSG000002	234.48525	-0.1524135	0.0613074	-2.4860531	0.01291687	0.05718086
ENSG000002	277.171504	-0.3023139	0.09571533	-3.1584693	0.001586	0.01352347
ENSG000002	110.771954	0.54172092	0.17719146	3.05726313	0.00223368	0.01703501
ENSG000002	3264.03183	0.26032008	0.13003193	2.00197048	0.04528791	0.13203873
ENSG000002	107.743897	-0.3720024	0.14958449	-2.4869049	0.01288599	0.05707997
ENSG000002	82.9724697	-0.3331271	0.16140206	-2.0639582	0.03902168	0.11932834
ENSG000002	143.763147	0.31320425	0.11322758	2.76614794	0.00567228	0.03244633
ENSG000002	62.7974978	0.47208044	0.14060448	3.35750638	0.00078649	0.00826671
ENSG000002	568.269491	-0.3119874	0.11490402	-2.7151997	0.00662358	0.03608202
ENSG000002	79.1032757	-0.6030249	0.18935182	-3.1846798	0.00144914	0.01267285
ENSG000002	748.959444	0.22802977	0.1271676	1.79314363	0.07294996	0.18278736
ENSG000002	480.809919	-0.2559177	0.09699561	-2.6384458	0.0083287	0.0423687
ENSG000002	350.467199	-0.341757	0.09258787	-3.691164	0.00022323	0.00323634
ENSG000002	174.269023	-0.1769547	0.09650054	-1.8337175	0.06669594	0.17210761
ENSG000002	1865.1899	0.83532099	0.28276865	2.95407917	0.00313604	0.02168771

ENSG000002	159.278974	0.19139973	0.08228295	2.32611665	0.02001233	0.07666873
ENSG000002	417.778267	0.40198085	0.12439574	3.23146797	0.00123156	0.01131149
ENSG000002	27.5992959	1.44363725	0.31824656	4.53622265	5.73E-06	0.00017853
ENSG000002	725.617345	-0.4240141	0.14112792	-3.0044665	0.00266047	0.01927695
ENSG000002	514.686534	-0.4318272	0.14573032	-2.9631939	0.00304465	0.02121678
ENSG000002	266.338294	0.2417801	0.13644153	1.7720419	0.0763876	0.1880672
ENSG000002	173.872319	-0.5383575	0.16061252	-3.3519025	0.00080258	0.00836083
ENSG000002	457.888037	-0.3607798	0.20215891	-1.7846347	0.07432059	0.18494988
ENSG000002	62.4717381	-0.3914628	0.21761682	-1.7988625	0.07204043	0.18114505
ENSG000002	9.82598529	1.7567574	0.63628254	2.76097062	0.00576299	0.03277686
ENSG000002	111.631549	1.30201326	0.21160708	6.15297599	7.60E-10	9.45E-08
ENSG000002	216.236758	0.77238808	0.15380267	5.02194187	5.12E-07	2.42E-05
ENSG000002	92.6265825	0.44488568	0.14341063	3.10218053	0.00192101	0.01541035
ENSG000002	101.375031	0.23786033	0.10717062	2.21945464	0.02645581	0.09233629
ENSG000002	356.006383	-0.2417316	0.06204211	-3.8962498	9.77E-05	0.00177396
ENSG000002	134.137617	-0.2249345	0.12241756	-1.8374362	0.06614553	0.17109768
ENSG000002	2413.31403	0.07173499	0.03855727	1.860479	0.06281779	0.16519583
ENSG000002	201.616574	0.58804255	0.16605456	3.54126109	0.00039822	0.00499205
ENSG000002	137.954103	-0.4434322	0.12490728	-3.550091	0.0003851	0.00486851
ENSG000002	180.866837	-0.1817205	0.09803302	-1.8536659	0.06378697	0.16691907
ENSG000002	1464.58565	-0.2341869	0.11629177	-2.0137878	0.04403182	0.12943523
ENSG000002	131.56221	0.54320334	0.16237161	3.34543291	0.00082154	0.00849329
ENSG000002	184.18127	-0.2579801	0.11476899	-2.247821	0.02458761	0.08806286
ENSG000002	113.633727	0.39612211	0.18520571	2.13882239	0.03245006	0.10588728
ENSG000002	104.377584	0.22695421	0.12644172	1.79493133	0.07266464	0.18226742
ENSG000002	128.384834	-0.2823293	0.102039	-2.7668768	0.00565961	0.03240247
ENSG000002	1984.7366	0.31713751	0.10373711	3.05712702	0.0022347	0.01703501
ENSG000002	157.36072	0.46328989	0.15615089	2.96693719	0.00300782	0.02101376
ENSG000002	121.044692	1.05700811	0.19636003	5.38301065	7.33E-08	4.91E-06
ENSG000002	152.244635	-0.1732732	0.08258377	-2.098151	0.03589181	0.1130461
ENSG000002	2771.34164	-0.1721825	0.09624619	-1.7889802	0.073618	0.18373127
ENSG000002	1115.1023	-0.1076839	0.04730786	-2.276237	0.02283183	0.08362151
ENSG000002	311.710639	-0.1758739	0.08486533	-2.0723887	0.0382292	0.11766834
ENSG000002	1006.66356	-0.2847478	0.07411296	-3.8420785	0.000122	0.00210578
ENSG000002	164.360062	0.39938581	0.15759845	2.53419883	0.01127048	0.05200585
ENSG000002	181.996523	-0.2925377	0.10992756	-2.661186	0.00778659	0.04032747
ENSG000002	344.321238	-0.2414098	0.09626067	-2.5078759	0.01214593	0.05485389
ENSG000002	135.849246	-0.4420677	0.19989709	-2.2114764	0.02700286	0.09364761
ENSG000002	409.544607	-0.110981	0.05753041	-1.9290848	0.05372034	0.14847692
ENSG000002	142.353213	0.17835117	0.09669709	1.84443158	0.06512027	0.16946152
ENSG000002	224.892417	0.26186742	0.14991341	1.7467912	0.08067356	0.19503611
ENSG000002	307.648982	-0.3830394	0.1311584	-2.9204338	0.00349544	0.02333664
ENSG000002	747.309909	1.3654459	0.2653911	5.14503273	2.67E-07	1.42E-05

ENSG000002	2062.02758	0.50138714	0.15097751	3.32093927	0.00089715	0.00901936
ENSG000002	127.035979	0.86208326	0.25969157	3.31964277	0.00090133	0.00904471
ENSG000002	59.1547599	0.71618352	0.23376686	3.06366575	0.00218643	0.01676059
ENSG000002	1098.74531	1.41691456	0.29926693	4.73461793	2.19E-06	7.85E-05
ENSG000002	178.58173	0.7840123	0.18884148	4.15169533	3.30E-05	0.00075262
ENSG000002	62.2059019	0.83413058	0.1713391	4.86830258	1.13E-06	4.63E-05
ENSG000002	145.81216	-0.3129583	0.12450314	-2.5136577	0.01194864	0.05421189
ENSG000002	403.302955	0.36087044	0.18741795	1.92548495	0.05416872	0.14932825
ENSG000002	2382.46151	0.56453208	0.19725812	2.86189529	0.00421116	0.02655784
ENSG000002	116.467688	-0.2536795	0.13684741	-1.8537397	0.06377641	0.16691808
ENSG000002	214.338344	-0.221987	0.11588585	-1.9155664	0.05542031	0.15157357
ENSG000002	202.781668	0.46192496	0.21514284	2.14706173	0.03178837	0.1044196
ENSG000002	111.697332	0.46266013	0.0955402	4.84257049	1.28E-06	5.13E-05
ENSG000002	1448.77556	0.86443391	0.16929034	5.10622112	3.29E-07	1.71E-05
ENSG000002	141.911452	0.19238004	0.06295166	3.05599608	0.00224314	0.01709145
ENSG000002	119.83805	-0.206404	0.09806793	-2.1047043	0.03531706	0.11191764
ENSG000002	129.84646	-0.2577055	0.14087176	-1.8293622	0.06734536	0.1732991
ENSG000002	43559.7647	0.91623929	0.20757525	4.41401024	1.01E-05	0.00028608
ENSG000002	141.72754	-0.4992473	0.13454895	-3.7105252	0.00020683	0.00309863
ENSG000002	172.628507	-0.202869	0.10436585	-1.9438251	0.05191654	0.14517869
ENSG000002	141.602741	-0.2892853	0.11956413	-2.4194994	0.01554189	0.06471313
ENSG000002	1048.89475	1.3555654	0.2352977	5.76106527	8.36E-09	7.45E-07
ENSG000002	430.554979	0.63871814	0.16898781	3.77966989	0.00015704	0.00254516
ENSG000002	664.865978	0.49013815	0.09662404	5.07263139	3.92E-07	1.97E-05
ENSG000002	740.117674	0.28523319	0.14928514	1.91066038	0.05604824	0.15261093
ENSG000002	919.804195	1.30940689	0.2478176	5.28375266	1.27E-07	7.71E-06
ENSG000002	2296.60692	-0.1934757	0.08014428	-2.4140922	0.01577447	0.06539924
ENSG000002	765.255124	0.37787473	0.08421263	4.48715019	7.22E-06	0.00021717
ENSG000002	499.478673	-0.1304276	0.0621352	-2.099094	0.03580862	0.11289478
ENSG000002	516.861544	0.62241839	0.25152703	2.4745586	0.01334009	0.05836113
ENSG000002	1792.58472	-0.3767957	0.12678706	-2.9718784	0.00295984	0.02077517
ENSG000002	131.316217	-0.2815368	0.10160457	-2.7709065	0.00559005	0.03210508
ENSG000002	53.8905236	0.4075392	0.1582018	2.57607187	0.00999299	0.04782138
ENSG000002	230.077116	0.44517048	0.14503521	3.06939603	0.00214492	0.01655322
ENSG000002	1151.23497	0.36763421	0.13282005	2.76791212	0.00564167	0.0323449
ENSG000002	99.6500025	0.38156612	0.16395821	2.32721565	0.01995379	0.07649808
ENSG000002	969.924153	-0.1788568	0.08108235	-2.2058666	0.02739334	0.09446237
ENSG000002	103.690019	-0.2655371	0.12381374	-2.1446497	0.03198086	0.10487804
ENSG000002	82.388208	0.35950967	0.14472404	2.48410472	0.01298776	0.0574017
ENSG000002	2038.72768	0.93751793	0.17805124	5.26543891	1.40E-07	8.43E-06
ENSG000002	437.482882	0.47821636	0.14589967	3.27770696	0.00104654	0.0100469
ENSG000002	112.271064	-0.2375416	0.11992009	-1.9808323	0.04761009	0.1367185
ENSG000002	22.6934665	1.4361613	0.33398036	4.300137	1.71E-05	0.00042796

ENSG000002	108.333897	-0.4601769	0.15959456	-2.8834121	0.00393392	0.02531576
ENSG000002	132.799375	0.31331786	0.17948187	1.74567975	0.08086662	0.195351
ENSG000002	170.517527	0.43532377	0.15876508	2.74193648	0.00610782	0.03413145
ENSG000002	267.923113	0.21337626	0.0837701	2.54716486	0.01086021	0.05066142
ENSG000002	201.410732	0.31220874	0.14481525	2.15591066	0.03109064	0.10294694
ENSG000002	871.036033	0.31212877	0.11806989	2.64359319	0.00820312	0.04185019
ENSG000002	3037496.49	1.22145109	0.55419822	2.20399678	0.02752457	0.09479529
ENSG000002	1346.38752	0.43556278	0.11710877	3.71930105	0.00019977	0.00302187
ENSG000002	30.9255485	1.05819177	0.39549022	2.67564585	0.00745854	0.03921055
ENSG000002	243.826737	0.4415417	0.13283621	3.3239558	0.0008875	0.00894923
ENSG000002	407.163255	-0.1668094	0.07884266	-2.1157247	0.03436823	0.11004409
ENSG000002	619.315223	1.46978199	0.28985835	5.07069063	3.96E-07	1.98E-05
ENSG000002	439.984138	1.27591106	0.27381065	4.65982991	3.16E-06	0.00010677
ENSG000002	150.626897	-0.4800824	0.15034188	-3.1932711	0.00140671	0.01240096
ENSG000002	751.364085	-0.25392	0.06259222	-4.0567347	4.98E-05	0.00102897
ENSG000002	186.860716	-0.3259978	0.1313734	-2.4814596	0.01308455	0.05762757
ENSG000002	156.10503	-0.2433212	0.10329065	-2.3556945	0.01848811	0.07254118
ENSG000002	390.493602	-0.2918488	0.10300897	-2.8332364	0.00460793	0.02825588
ENSG000002	185.98436	-0.2193091	0.12224293	-1.7940432	0.07280627	0.18248563
ENSG000002	159.170005	-0.4948016	0.17106883	-2.8924124	0.00382296	0.02481593
ENSG000002	34.0322078	1.1622821	0.54575037	2.12969548	0.03319676	0.10757457
ENSG000002	231.056158	-0.3744166	0.17029666	-2.1986137	0.0279054	0.09568947
ENSG000002	969.886426	0.76499542	0.18870202	4.05398633	5.04E-05	0.00103946
ENSG000002	750.317286	1.27683243	0.22641754	5.6392823	1.71E-08	1.35E-06
ENSG000002	167.919155	-0.1994213	0.08899743	-2.2407531	0.02504207	0.08911501
ENSG000002	373.035567	0.4208804	0.15176441	2.77324833	0.00554998	0.03197573
ENSG000002	91.8107724	-0.304055	0.13489339	-2.2540396	0.02419368	0.08723236
ENSG000002	312.551181	-0.1572307	0.08777039	-1.7913868	0.07323125	0.18310744
ENSG000002	1917.13841	0.44112335	0.13479584	3.27252942	0.0010659	0.01016393
ENSG000002	581.008959	-0.1681292	0.0909334	-1.8489271	0.06446834	0.16824563
ENSG000002	424.681513	-0.1904555	0.08182672	-2.3275464	0.0199362	0.07646641
ENSG000002	1452.48376	0.56744432	0.17217995	3.29564693	0.00098195	0.00962408
ENSG000002	1629.21297	0.71329399	0.15503452	4.60087196	4.21E-06	0.00013853
ENSG000002	135.546408	-0.5805336	0.18272239	-3.1771342	0.00148738	0.01291769
ENSG000002	5242.23307	0.48506079	0.19071361	2.54339895	0.01097798	0.05096431
ENSG000002	601.436756	0.41959086	0.15904914	2.63812088	0.00833669	0.04237342
ENSG000002	367.84493	0.44031541	0.17076374	2.57850656	0.00992284	0.04756026
ENSG000002	862.048256	0.37854704	0.11948595	3.16813003	0.00153423	0.01323622
ENSG000002	66.7009336	1.71520097	0.25567139	6.70861508	1.96E-11	3.98E-09
ENSG000002	69.8019933	0.45500836	0.22441282	2.02755063	0.04260613	0.12642286
ENSG000002	1486.7795	1.32221992	0.23884874	5.53580457	3.10E-08	2.28E-06
ENSG000002	89.9869287	0.34796578	0.12980749	2.68062951	0.00734838	0.03884314
ENSG000002	190.207022	1.31657583	0.19099011	6.89342399	5.45E-12	1.40E-09

ENSG000002	793.808538	0.12826702	0.0698374	1.83665218	0.06626126	0.17128896
ENSG000002	496.932918	0.87853772	0.17478885	5.02628024	5.00E-07	2.40E-05
ENSG000002	326.595578	1.30480535	0.37991378	3.43447755	0.0005937	0.00667662
ENSG000002	714.355427	0.29894163	0.15063851	1.98449679	0.04720049	0.13593629
ENSG000002	22.7464286	0.3939183	0.21930227	1.79623453	0.07245723	0.18185843
ENSG000002	315.639597	-0.2454059	0.12985584	-1.8898332	0.05878028	0.15828875
ENSG000002	30.3426941	0.99192919	0.3113619	3.18577581	0.00144366	0.01263848
ENSG000002	129.408883	-0.5013279	0.14778481	-3.3922829	0.00069313	0.00754162
ENSG000002	416.693596	1.29519125	0.26326471	4.91972987	8.67E-07	3.72E-05
ENSG000002	203.896997	-0.2289164	0.09033936	-2.5339611	0.01127813	0.05201896
ENSG000002	16.7608711	1.23834931	0.37764543	3.27913229	0.00104127	0.01001388
ENSG000002	1089.19868	-0.2849684	0.09241676	-3.0835145	0.00204571	0.01602653
ENSG000002	1332.67025	0.92871536	0.2108409	4.40481595	1.06E-05	0.00029524
ENSG000002	436.337474	0.61129023	0.17247176	3.54429169	0.00039367	0.00496538
ENSG000002	188.566101	-0.6090705	0.17618409	-3.4570118	0.0005462	0.00628054
ENSG000002	1254.77615	-0.1512618	0.05107041	-2.9618283	0.00305818	0.021293
ENSG000002	178.6511	-0.2682245	0.12463872	-2.1520158	0.03139611	0.10362361
ENSG000002	85181.4915	0.74114165	0.247447	2.99515317	0.00274307	0.01970133
ENSG000002	203.766578	-0.3148598	0.13611634	-2.3131666	0.02071348	0.07856086
ENSG000002	5488.57119	0.64018589	0.20598659	3.10790082	0.00188421	0.01521193
ENSG000002	29.0648748	1.38291745	0.42671022	3.24088192	0.00119161	0.01102638
ENSG000002	538.300178	-0.431395	0.14848799	-2.9052516	0.00366958	0.024155
ENSG000002	675.719661	0.43032631	0.17032043	2.52656899	0.01151828	0.05286903
ENSG000002	609.459109	-0.494514	0.1451938	-3.4058891	0.00065949	0.00726724
ENSG000002	449.7126	0.33633349	0.13424793	2.5053161	0.0122342	0.05508077
ENSG000002	117.071034	-0.7067632	0.21556062	-3.2787215	0.00104278	0.01002003
ENSG000002	305.390772	0.31067762	0.09179521	3.38446452	0.00071317	0.00767819
ENSG000002	260.578123	-0.5607466	0.15435092	-3.6329331	0.00028022	0.00379807
ENSG000002	184.935052	-0.4739228	0.13318414	-3.5584022	0.00037312	0.0047353
ENSG000002	59.8921255	0.35451327	0.18662528	1.89959939	0.05748571	0.15551777
ENSG000002	257.598009	-0.338042	0.1013633	-3.3349548	0.00085313	0.00872121
ENSG000002	104.398064	-0.5277977	0.23220898	-2.2729427	0.02302964	0.084158
ENSG000002	518.482551	-0.1783866	0.07213296	-2.4730245	0.01339749	0.05851857
ENSG000002	35.8769903	2.58543121	0.48591718	5.32072402	1.03E-07	6.52E-06
ENSG000002	180.043451	-0.4888043	0.17702959	-2.7611447	0.00575991	0.03277076
ENSG000002	282.493301	-0.2786482	0.07103572	-3.9226496	8.76E-05	0.00162266
ENSG000002	103.589628	-0.461239	0.15813925	-2.9166639	0.00353797	0.02352476
ENSG000002	563.701134	-0.1910274	0.09969224	-1.9161711	0.05534332	0.15147129
ENSG000002	119.186223	-0.3433223	0.15971235	-2.1496293	0.03158455	0.10403613
ENSG000002	182.248087	0.2192973	0.10966346	1.99972979	0.04552945	0.13248383
ENSG000002	647.466603	-0.3532298	0.09161595	-3.8555491	0.00011547	0.00201851
ENSG000002	305.614072	-0.2257046	0.06923245	-3.2600986	0.00111373	0.01049535
ENSG000002	845.425474	1.1946202	0.18998939	6.28782575	3.22E-10	4.49E-08

ENSG000002	135.029074	-0.2070023	0.09851482	-2.1012301	0.03562077	0.11251663
ENSG000002	3838.60399	-0.2055559	0.06778441	-3.0324953	0.00242541	0.01804905
ENSG000002	420.31898	-0.4088441	0.15974065	-2.5594242	0.01048457	0.0493443
ENSG000002	153.25008	-0.2977332	0.15315001	-1.9440626	0.0518879	0.1451392
ENSG000002	120.676073	0.40707539	0.15518113	2.6232274	0.00871011	0.04354259
ENSG000002	423.538436	2.24181747	0.2707593	8.27974331	1.23E-16	4.05E-13
ENSG000002	188.393238	-0.3303206	0.13369945	-2.4706201	0.0134879	0.05876494
ENSG000002	791.48887	1.07762243	0.21575287	4.99470733	5.89E-07	2.71E-05
ENSG000002	241.936478	0.52245489	0.18885186	2.76647998	0.00566651	0.03243061
ENSG000002	468.121626	-0.3814043	0.10020011	-3.806426	0.00014099	0.00233988
ENSG000002	285.525525	-0.1712266	0.08817258	-1.9419481	0.05214338	0.14567984
ENSG000002	931.433497	-0.204765	0.052889	-3.8715996	0.00010812	0.00191665
ENSG000002	189.29158	-0.1604504	0.0895686	-1.7913694	0.07323404	0.18310744
ENSG000002	271.081452	-0.3198384	0.09768156	-3.2742964	0.00105925	0.01013929
ENSG000002	80.780731	0.87262215	0.25921004	3.36646742	0.00076138	0.0080761
ENSG000002	276.614172	-0.4020928	0.11964527	-3.3607076	0.00077743	0.00818724
ENSG000002	2088.52747	-0.2192485	0.11097463	-1.9756634	0.04819291	0.13781289
ENSG000002	292.883628	0.2011459	0.11225338	1.79189176	0.07315031	0.18306423
ENSG000002	145.376328	0.28827243	0.1511069	1.90773843	0.05642503	0.15335675
ENSG000002	128.229739	-0.3039034	0.10847078	-2.8017074	0.0050833	0.03032041
ENSG000002	1390.73996	-0.2563187	0.07282034	-3.5198779	0.00043175	0.0052989
ENSG000002	375.791034	-0.1762223	0.08314872	-2.1193623	0.03405986	0.10936801
ENSG000002	468.151742	-0.1995048	0.08151266	-2.4475318	0.01438384	0.06118077
ENSG000002	238.53584	-0.2649925	0.1478897	-1.7918254	0.07316094	0.18306423
ENSG000002	743.221191	-0.2130746	0.07396619	-2.8807032	0.00396789	0.02543452
ENSG000002	66.4401689	-0.8641913	0.31428843	-2.7496757	0.00596543	0.0335673
ENSG000002	860.264732	0.28326105	0.11929242	2.37451014	0.01757225	0.070219
ENSG000002	124.089904	-0.2617444	0.10751056	-2.4345926	0.01490856	0.06269704
ENSG000002	193.243354	-0.2274681	0.12421533	-1.8312403	0.06706468	0.17271236
ENSG000002	203.529768	-0.1945835	0.10888473	-1.7870599	0.0739278	0.18430807
ENSG000002	763.535631	-0.5597376	0.15224708	-3.6765078	0.00023645	0.00336842
ENSG000002	70.0563766	-0.8248742	0.23458229	-3.5163533	0.00043752	0.00532258
ENSG000002	120.713859	-0.2661217	0.14929467	-1.7825267	0.07466339	0.18541587
ENSG000002	789.811981	-0.1629604	0.07546874	-2.1593102	0.03082611	0.10229826
ENSG000002	303.830854	0.45916481	0.12868271	3.56819365	0.00035945	0.00459744
ENSG000002	215.946809	0.37887136	0.19082846	1.98540275	0.04709968	0.13579671
ENSG000002	950.495014	-0.318068	0.11282328	-2.8191699	0.0048148	0.02919686
ENSG000002	1191.37707	-0.2951596	0.09724952	-3.0350749	0.00240476	0.01794823
ENSG000002	153.974454	0.87014153	0.20114708	4.32589699	1.52E-05	0.00039251
ENSG000002	372.946588	0.17609327	0.08898787	1.9788458	0.04783337	0.1371061
ENSG000002	74.1518775	0.92990417	0.15726389	5.91301759	3.36E-09	3.46E-07
ENSG000002	217.072792	-0.2008251	0.08201381	-2.4486737	0.01433833	0.06107299
ENSG000002	234.132636	-0.2077324	0.11272991	-1.8427444	0.06536633	0.16988616

ENSG000002	273.19011	0.21792214	0.11670984	1.86721302	0.06187185	0.16371029
ENSG000002	257.924504	-0.3898968	0.12201819	-3.1953988	0.00139638	0.01232978
ENSG000002	417.323666	-0.447608	0.11817852	-3.7875576	0.00015214	0.00249207
ENSG000002	151.643268	-0.3839129	0.13111227	-2.9281232	0.00341015	0.02297633
ENSG000002	254.164588	0.58993414	0.14584149	4.04503634	5.23E-05	0.00107495
ENSG000002	333.279327	0.35815862	0.19713616	1.81680831	0.06924647	0.17652898
ENSG000002	227.072871	0.37074048	0.11892049	3.11754916	0.00182362	0.01488668
ENSG000002	199.038956	-0.3115799	0.09852427	-3.1624691	0.00156437	0.0134088
ENSG000002	163.164373	0.34175574	0.16082459	2.12502172	0.0335848	0.10851034
ENSG000002	724.497431	-0.2699276	0.11547407	-2.33756	0.01941009	0.0749162
ENSG000002	157.582701	-0.5315026	0.21866924	-2.4306235	0.01507287	0.06320968
ENSG000002	233.229931	-0.3428254	0.14558332	-2.3548399	0.01853069	0.07263869
ENSG000002	589.202311	-0.5634732	0.22080758	-2.5518745	0.01071451	0.05018162
ENSG000002	97.0521939	-0.4357482	0.16839385	-2.5876732	0.00966266	0.04669642
ENSG000002	348.45507	-0.1910742	0.06285233	-3.0400503	0.00236539	0.01775881
ENSG000002	182.977767	0.23950755	0.13061693	1.83366389	0.0667039	0.17210761
ENSG000002	81.1791234	-0.7758412	0.20013658	-3.8765584	0.00010594	0.00188413
ENSG000002	214.318694	1.02434822	0.19589394	5.22909597	1.70E-07	9.94E-06
ENSG000002	74.6843138	2.85018043	0.42849913	6.65154302	2.90E-11	5.66E-09
ENSG000002	120.212174	0.49042443	0.21806068	2.24902736	0.02451076	0.08794373
ENSG000002	2043.08656	-0.2410631	0.07148991	-3.371988	0.00074628	0.00795384
ENSG000002	356.306287	-0.4764115	0.15890831	-2.9980275	0.00271733	0.01957649
ENSG000002	186.974649	-0.3224528	0.18460435	-1.7467239	0.08068524	0.19503611
ENSG000002	980.300613	-0.2358338	0.08782881	-2.6851531	0.00724966	0.03854496
ENSG000002	454.565851	0.1990051	0.07172496	2.77455844	0.00552767	0.03190328
ENSG000002	285.963864	-0.3026038	0.1366417	-2.2145791	0.02678897	0.09312247
ENSG000002	189.922858	0.38738469	0.15415026	2.51303301	0.01196981	0.05427794
ENSG000002	92.9266638	0.29124293	0.11324319	2.57183617	0.01011607	0.04820531
ENSG000002	34.6183047	1.86162218	0.38874063	4.78885419	1.68E-06	6.35E-05
ENSG000002	1379.45544	0.15127685	0.07766403	1.94783667	0.05143451	0.14425369
ENSG000002	986.147794	0.15766504	0.06469528	2.43704077	0.01480801	0.06243909
ENSG000002	295.531039	-0.1796462	0.08776564	-2.0468858	0.0406693	0.12249349
ENSG000002	884.863381	-0.3453813	0.18000138	-1.9187704	0.0550134	0.15099555
ENSG000002	101.507274	-0.573546	0.23183077	-2.4739858	0.0133615	0.05842361
ENSG000002	251.659456	-0.3643435	0.10025284	-3.6342466	0.00027879	0.00379054
ENSG000002	866.75883	0.44365512	0.13406085	3.30935632	0.00093511	0.00928707
ENSG000002	2008.59524	-0.3009292	0.11512085	-2.6140283	0.00894816	0.04438083
ENSG000002	49.8698351	-0.8693818	0.28313362	-3.0705706	0.0021365	0.01651684
ENSG000002	1514.89899	0.8299988	0.26814034	3.09538956	0.00196555	0.01559219
ENSG000002	1301.90012	-0.1427509	0.06993422	-2.041216	0.04122937	0.12368056
ENSG000002	562.384862	0.61387424	0.18882737	3.25098118	0.00115007	0.01072074
ENSG000002	624.627064	0.39053587	0.10703053	3.64882681	0.00026344	0.00363606
ENSG000002	554.695647	1.02156548	0.23269195	4.39020545	1.13E-05	0.0003126

ENSG000002	182.832583	3.68087702	0.65420531	5.62648601	1.84E-08	1.44E-06
ENSG000002	19.0204519	1.59594873	0.69627255	2.29213219	0.02189802	0.08171987
ENSG000002	690.341984	-0.1642875	0.09251151	-1.7758601	0.07575598	0.18715847
ENSG000002	44.1667261	0.48612613	0.21625849	2.24789383	0.02458296	0.08806286
ENSG000002	250.059037	-0.2958388	0.10790181	-2.7417409	0.00611145	0.03413145
ENSG000002	638.532752	1.14630922	0.14754694	7.76911527	7.90E-15	8.10E-12
ENSG000002	138.85555	-0.8854668	0.22329142	-3.9655211	7.32E-05	0.00140944
ENSG000002	301.507125	-0.1488968	0.07572435	-1.9662996	0.04926402	0.14012004
ENSG000002	357.84856	1.33009491	0.21230548	6.26500516	3.73E-10	5.05E-08
ENSG000002	184.561331	-0.4016781	0.10948537	-3.6687833	0.00024371	0.00344786
ENSG000002	340.969431	0.30566292	0.15545126	1.96629431	0.04926462	0.14012004
ENSG000002	84.6539407	0.89284699	0.16042434	5.56553336	2.61E-08	1.97E-06
ENSG000002	116.206105	0.89573579	0.27228193	3.28973649	0.00100281	0.00976876
ENSG000002	171.688084	-0.5580582	0.1677253	-3.3272154	0.00087719	0.00890596
ENSG000002	40.2047144	1.174659	0.45352328	2.59007432	0.00959552	0.04645343
ENSG000002	341.231618	0.38425564	0.17529198	2.19208913	0.02837307	0.0966618
ENSG000002	341.108364	2.73222563	0.4307068	6.34358599	2.24E-10	3.35E-08
ENSG000002	95.4061179	-0.5163617	0.25688626	-2.0100791	0.04442282	0.130142
ENSG000002	1092.7515	-0.3754231	0.10059095	-3.7321757	0.00018983	0.00291178
ENSG000002	1788.67293	-0.0819204	0.0357768	-2.2897646	0.02203496	0.0819291
ENSG000002	54.901246	0.5413904	0.25807498	2.09780271	0.03592258	0.11311445
ENSG000002	326.333895	-1.005043	0.26075864	-3.8543036	0.00011606	0.00202235
ENSG000002	129.592597	1.12983262	0.22721927	4.972433	6.61E-07	2.97E-05
ENSG000002	877.68864	-0.3290068	0.1272137	-2.5862533	0.00970256	0.04683335
ENSG000002	291.018998	-0.0978667	0.05548058	-1.7639811	0.07773516	0.19013481
ENSG000002	523.310866	0.43322538	0.15369267	2.81877715	0.0048207	0.02920021
ENSG000002	340.063807	-0.2749771	0.13499002	-2.0370182	0.04164822	0.12454967
ENSG000002	57.3570801	0.74732218	0.20617189	3.62475299	0.00028924	0.00388629
ENSG000002	11.7933545	1.09853212	0.40689889	2.69976681	0.00693881	0.03736588
ENSG000002	97.8719246	-0.5154616	0.20618519	-2.4999931	0.01241957	0.05565527
ENSG000002	94.31764	0.3160011	0.16773673	1.88391113	0.059577	0.15962157
ENSG000002	339.623744	0.56695782	0.19462392	2.91309425	0.00357867	0.02371842
ENSG000002	119.2281	0.2216568	0.08897709	2.49116706	0.01273242	0.05664832
ENSG000002	429.947952	0.282363	0.0958751	2.94511287	0.00322837	0.022121
ENSG000002	704.635782	0.38692569	0.2104031	1.83897331	0.06591911	0.17070052
ENSG000002	152.85264	-0.1776682	0.08816092	-2.0152721	0.04387615	0.12904704
ENSG000002	667.900745	-0.1540318	0.06308747	-2.44156	0.01462396	0.06200906
ENSG000002	80.4600729	0.32034905	0.16714072	1.91664278	0.05528333	0.15135761
ENSG000002	114.436139	0.47053658	0.18477258	2.54657147	0.01087869	0.05069754
ENSG000002	59.702213	-0.7891622	0.25834434	-3.0546912	0.00225292	0.01715801
ENSG000002	1163.49459	0.1397122	0.07792538	1.7928972	0.07298936	0.18283022
ENSG000002	1919.2541	-0.0936573	0.04488224	-2.0867331	0.03691226	0.11496655
ENSG000002	31.4790837	1.21095881	0.35614958	3.40014107	0.00067351	0.00738206

ENSG000002	242.133588	0.42043958	0.17558721	2.39447719	0.01664408	0.06782131
ENSG000002	77.1229541	1.00509512	0.33289902	3.01921921	0.00253427	0.01859259
ENSG000002	307.543043	0.2908866	0.0897309	3.24176631	0.00118791	0.01100465
ENSG000002	301.094142	1.01905888	0.50803322	2.00589026	0.04486796	0.13118757
ENSG000002	177.140303	1.10009061	0.29010013	3.79210655	0.00014937	0.00245421
ENSG000002	272.920243	-0.1825332	0.10480015	-1.741726	0.0815564	0.19661336
ENSG000002	64.7016804	0.33983179	0.1933069	1.75799099	0.07874904	0.19190449
ENSG000002	133.206076	-0.4877963	0.16280612	-2.9961789	0.00273386	0.01966101
ENSG000002	394.518853	0.26059711	0.10318301	2.52558167	0.01155069	0.05291889
ENSG000002	123.663951	-0.2694619	0.12592299	-2.1398947	0.03236328	0.1057866
ENSG000002	109.475213	-0.3645996	0.14400845	-2.5317934	0.01134808	0.05226812
ENSG000002	1721.48067	0.46468475	0.14938732	3.11060365	0.00186705	0.01514049
ENSG000002	206.281235	-0.2318042	0.11825757	-1.9601636	0.04997667	0.14150706
ENSG000002	285.688423	-0.2437984	0.10034016	-2.4297194	0.01511052	0.06332032
ENSG000002	295.432754	-0.351585	0.10820604	-3.2492175	0.00115723	0.01076296
ENSG000002	1001.85223	0.54348883	0.20205059	2.68986509	0.00714809	0.03814292
ENSG000002	95.8540406	0.8566664	0.2539627	3.37319775	0.00074301	0.00793685
ENSG000002	310.818092	0.22659315	0.07456993	3.03866653	0.00237628	0.01782425
ENSG000002	895.470671	-0.2819065	0.08434391	-3.3423461	0.00083073	0.00854551
ENSG000002	38.0379487	0.9065076	0.27678677	3.27511175	0.0010562	0.01012563
ENSG000002	79.3082335	0.76793794	0.1634889	4.69718692	2.64E-06	9.09E-05
ENSG000002	716.396199	-0.3350483	0.17479407	-1.9168172	0.05526116	0.15135761
ENSG000002	655.41229	-0.1696573	0.07177007	-2.3639007	0.01808366	0.07145007
ENSG000002	102.805347	0.62002737	0.25073463	2.47284294	0.0134043	0.05852337
ENSG000002	287.850055	-0.797616	0.25049603	-3.1841464	0.00145182	0.01268946
ENSG000002	281.488229	-0.325427	0.13446319	-2.4201939	0.01551223	0.06460607
ENSG000002	54.5176723	1.03974691	0.27231028	3.81824328	0.00013441	0.00227124
ENSG000002	2561.38192	0.22022025	0.12716675	1.73174397	0.08331916	0.19973454
ENSG000002	800.018825	0.7506241	0.24934262	3.01041234	0.00260893	0.01898742
ENSG000002	842.817859	0.21396819	0.11468363	1.8657257	0.06207975	0.16413272
ENSG000002	511.743119	-0.3028433	0.06869102	-4.4087751	1.04E-05	0.00029039
ENSG000002	395.979353	0.61403385	0.1835913	3.34456948	0.0008241	0.00849329
ENSG000002	375.28101	0.24285983	0.10901559	2.22775328	0.02589697	0.09110334
ENSG000002	1438.20572	-0.308373	0.096196	-3.2056735	0.00134747	0.01204056
ENSG000002	317.561231	-0.2202441	0.09027765	-2.4396309	0.01470228	0.06219832
ENSG000002	217.474591	0.70206808	0.26994903	2.60074311	0.00930221	0.04560436
ENSG000002	231.371529	-0.3075991	0.09778822	-3.1455635	0.00165767	0.01390444
ENSG000002	141.309102	-0.4767692	0.15331289	-3.1097792	0.00187227	0.01515284
ENSG000002	140.94441	-0.2231726	0.11860286	-1.8816793	0.05987957	0.16014441
ENSG000002	349.285167	-0.2321732	0.11129954	-2.0860213	0.0369767	0.11504874
ENSG000002	210.186118	-0.5144708	0.12134119	-4.2398699	2.24E-05	0.00054248
ENSG000002	365.660718	-0.2519172	0.10449541	-2.4107971	0.0159177	0.06581001
ENSG000002	340.716494	0.54710422	0.24247613	2.25632195	0.02405048	0.08693908

ENSG000002	133.305709	0.27073359	0.12988639	2.08438767	0.03712492	0.11537856
ENSG000002	222.834537	-0.2635325	0.15085669	-1.7469065	0.08065355	0.19503142
ENSG000002	129.003081	-0.5288937	0.1819152	-2.907364	0.00364489	0.02406008
ENSG000002	75.4636835	1.28867692	0.37869547	3.40293726	0.00066666	0.00732651
ENSG000002	1657.11996	0.30930728	0.12076267	2.56128217	0.01042866	0.04912346
ENSG000002	1964.27332	1.49226723	0.28158845	5.29946187	1.16E-07	7.21E-06
ENSG000002	621.125336	-0.3675797	0.08956829	-4.1039049	4.06E-05	0.0008786
ENSG000002	249.812761	-0.2809344	0.10053099	-2.794505	0.00519792	0.03079633
ENSG000002	120.574063	-0.4213528	0.13651518	-3.0864901	0.00202535	0.01592024
ENSG000002	150.087816	-0.1467166	0.07255107	-2.0222535	0.04315017	0.12757544
ENSG000002	812.750283	0.36505874	0.11921451	3.06220054	0.00219716	0.01682712
ENSG000002	221.022419	-0.2441148	0.12538902	-1.9468592	0.05155161	0.14439559
ENSG000002	95.2833389	1.74409439	0.40096241	4.34977036	1.36E-05	0.0003617
ENSG000002	152.301379	-0.2790994	0.09969785	-2.7994528	0.00511893	0.03045877
ENSG000002	149.028813	-0.3294714	0.13662264	-2.4115429	0.01588519	0.06574189
ENSG000002	178.596924	-0.3375409	0.15856433	-2.1287316	0.03327647	0.10773881
ENSG000002	97.9988658	0.47849238	0.14394548	3.32412225	0.00088697	0.00894923
ENSG000002	2175.4722	-0.298397	0.11862369	-2.5154926	0.01188662	0.05402019
ENSG000002	371.363526	0.42189993	0.18997177	2.22085589	0.02636072	0.09208283
ENSG000002	86.5860077	-0.530616	0.23486466	-2.2592415	0.02386837	0.08650963
ENSG000002	164.692621	0.42147288	0.16764581	2.5140675	0.01193476	0.05417891
ENSG000002	86.5349053	-0.6224823	0.19574132	-3.1801275	0.0014721	0.01279228
ENSG000002	202.186172	-0.4073292	0.15211725	-2.6777317	0.00741226	0.03905488
ENSG000002	164.116711	-0.4681756	0.17066032	-2.7433186	0.00608216	0.03401407
ENSG000002	154.289596	-0.3337827	0.13025978	-2.5624391	0.01039398	0.04907289
ENSG000002	1340.42419	1.5679975	0.2763066	5.67484628	1.39E-08	1.14E-06
ENSG000002	284.352985	0.17064919	0.06641442	2.5694599	0.01018572	0.04843829
ENSG000002	177.618101	-0.1737396	0.09072652	-1.9149816	0.05549485	0.15168345
ENSG000002	26.1831621	2.72626951	0.80079656	3.40444706	0.00066298	0.00729102
ENSG000002	169.731097	-0.2885577	0.08146561	-3.5420804	0.00039698	0.00498298
ENSG000002	82.5314781	1.6095103	0.31373799	5.13010967	2.90E-07	1.52E-05
ENSG000002	732.756661	0.60274056	0.17859378	3.37492475	0.00073836	0.00789624
ENSG000002	70.8650857	0.49817383	0.17674579	2.81858949	0.00482352	0.0292065
ENSG000002	58.1188692	1.637564	0.2633119	6.21910368	5.00E-10	6.41E-08
ENSG000002	346.431428	-0.2783119	0.08551549	-3.2545202	0.00113584	0.01062429
ENSG000002	315.998001	-0.2047671	0.09926474	-2.0628382	0.039128	0.11954198
ENSG000002	159.316744	0.33513764	0.14683976	2.28233579	0.02246952	0.08273149
ENSG000002	101.873298	0.26328802	0.12759739	2.06342794	0.03907199	0.11942744
ENSG000002	2214.76569	0.40828923	0.0811099	5.0337783	4.81E-07	2.32E-05
ENSG000002	968.863824	-0.1795515	0.09358681	-1.9185558	0.05504058	0.15099555
ENSG000002	43.2783423	-0.5477553	0.21890974	-2.5021969	0.01234253	0.05541632
ENSG000002	617.916752	2.35651808	0.31063324	7.58617488	3.29E-14	2.34E-11
ENSG000002	229.978854	-0.2804613	0.12305813	-2.2790959	0.02266137	0.08318299

ENSG000002	4.36121737	1.11212529	0.51596271	2.15543735	0.03112763	0.10296544
ENSG000002	208.562762	-0.3944033	0.15353095	-2.5688844	0.01020265	0.04850473
ENSG000002	127.677407	-0.3087981	0.13112575	-2.3549768	0.01852386	0.07263869
ENSG000002	88.2081547	-0.4421237	0.24708464	-1.7893612	0.07355666	0.1836346
ENSG000002	106.434221	0.30134407	0.17257029	1.74621065	0.08077436	0.1952037
ENSG000002	320.46973	-0.2908094	0.12216634	-2.380438	0.01729207	0.06952872
ENSG000002	365.220644	0.85330892	0.30097064	2.83518992	0.00457984	0.02813628
ENSG000002	358.396565	-0.2840609	0.14278229	-1.9894688	0.04664948	0.1348791
ENSG000002	254.008894	-0.3088933	0.14694088	-2.1021607	0.0355392	0.11232387
ENSG000002	151.800339	-0.2014467	0.09638212	-2.090084	0.03661025	0.11434254
ENSG000002	128.254372	-0.5832652	0.18499406	-3.1528859	0.00161665	0.01370941
ENSG000002	231.653369	0.5461781	0.15520248	3.51913258	0.00043296	0.00530788
ENSG000002	147.549428	-0.7223761	0.21437306	-3.3697149	0.00075246	0.00800655
ENSG000002	1240.9844	-0.299106	0.10930959	-2.7363194	0.00621307	0.03453146
ENSG000002	126.060555	-0.353915	0.15908744	-2.2246571	0.02610427	0.09147931
ENSG000002	192.364684	-0.2730646	0.11515599	-2.3712583	0.01772764	0.07058769
ENSG000002	182.297311	-0.5239861	0.2730214	-1.9192125	0.05495745	0.15096955
ENSG000002	693.157237	0.36426829	0.16886617	2.15714193	0.03099461	0.10271192
ENSG000002	195.884999	-0.3542577	0.19332762	-1.8324216	0.06688863	0.17234018
ENSG000002	113.241301	0.96110784	0.27304022	3.52002289	0.00043151	0.0052989
ENSG000002	193.0977	0.56186393	0.15538942	3.61584427	0.00029937	0.00398763
ENSG000002	120.889501	-0.4317866	0.22101413	-1.9536605	0.05074138	0.14307829
ENSG000002	225.481094	-0.4752028	0.23026284	-2.0637409	0.03904229	0.11936908
ENSG000002	93.3857543	0.33474785	0.14024969	2.38679917	0.01699577	0.06877584
ENSG000002	110.797732	-0.432913	0.23770046	-1.8212545	0.06856818	0.17537239
ENSG000002	113.017996	0.63247936	0.26154141	2.41827612	0.01559424	0.06483867
ENSG000002	124.757455	-0.4502282	0.23083823	-1.9504058	0.05112777	0.14374865
ENSG000002	80.798589	0.96545375	0.20818719	4.63743107	3.53E-06	0.00011757
ENSG000002	159.5604	-0.3077251	0.14237856	-2.1613165	0.0306709	0.10201029
ENSG000002	948.038905	0.93727059	0.19681916	4.76209026	1.92E-06	7.04E-05
ENSG000002	4647.53793	0.27228175	0.13849717	1.96597343	0.04930168	0.1401525
ENSG000002	138.623582	0.74021198	0.23168804	3.19486493	0.00139896	0.01234596
ENSG000002	345.204333	-0.5034518	0.24709362	-2.0374942	0.04160055	0.1244298
ENSG000002	1470.8841	-0.4412317	0.16622636	-2.6544026	0.0079449	0.04086948
ENSG000002	149.448697	-0.2381098	0.08571112	-2.7780506	0.00546861	0.03170749
ENSG000002	435.05396	-0.3360568	0.11449726	-2.9350641	0.00333479	0.02263266
ENSG000002	561.840375	0.57757806	0.11446859	5.04573411	4.52E-07	2.20E-05
ENSG000002	54.0770971	0.95125493	0.30457185	3.12325297	0.00178864	0.0146715
ENSG000002	672.468015	-0.1550694	0.07121708	-2.1774182	0.02944938	0.09921543
ENSG000002	121.075024	-0.6260705	0.2414454	-2.5930106	0.00951399	0.04626295
ENSG000002	169.575265	-0.1645588	0.09006079	-1.8271975	0.06767008	0.17386184
ENSG000002	150.831965	-0.3651097	0.17504021	-2.085862	0.03699113	0.11507182
ENSG000002	125.902081	-0.3065251	0.10480803	-2.9246333	0.00344862	0.02315385

ENSG000002	620.690222	0.85942333	0.27165427	3.16366581	0.00155796	0.01338614
ENSG000002	78.2111357	0.34202187	0.19387874	1.76410202	0.0777148	0.19013481
ENSG000002	246.209983	-0.3048655	0.1251651	-2.4357073	0.01486271	0.06256837
ENSG000002	90.3160625	-0.6997596	0.16221053	-4.3138974	1.60E-05	0.00040777
ENSG000002	678.182965	-0.2119362	0.08203618	-2.5834485	0.00978181	0.04709612
ENSG000002	269.677998	-0.3573047	0.16816471	-2.1247305	0.0336091	0.10854994
ENSG000002	106.070629	0.1927946	0.10241436	1.88249569	0.05976874	0.15992504
ENSG000002	71.6176092	-0.8204878	0.30599813	-2.6813491	0.0073326	0.03878471
ENSG000002	2767.75931	0.39426957	0.14254783	2.76587563	0.00567702	0.03244633

entrez	symbol	chromosome	start	end
8813	DPM1	20	50934867	50958555
55732	C1orf112	1	169662007	169854080
4800	NFYA	6	41072945	41099976
5893	RAD52	12	911736	990053
572	BAD	11	64269830	64284704
51056	LAP3	4	17577192	17607972
51384	WNT16	7	121325367	121341104
3382	ICA1	7	8113184	8262687
843	CASP10	2	201182898	201229406
8837	CFLAR	2	201116154	201176687
9108	MTMR7	8	17296794	17413528
23098	SARM1	17	28364356	28404049
26073	POLDIP2	17	28346633	28357527
952	CD38	4	15778275	15853232
85413	SLC22A16	6	110424687	110476641
340273	ABCB5	7	20615207	20777038
7982	ST7	7	116953238	117230103
996	CDC27	17	47117703	47189422
6521	SLC4A1	17	44248390	44268141
8935	SKAP2	7	26667068	26995239
5439	POLR2J	7	102473118	102478907
81691	REXO5	16	20806429	20849668
80256	FAM214B	9	35104112	35116341
4353	MPO	17	58269855	58280935
5244	ABCB4	7	87401696	87480435
3920	LAMP2	X	120426148	120469365
51573	GDE1	16	19501693	19522123
26022	TMEM98	17	32927910	32945106
51330	TNFRSF12A	16	3018445	3022383
4293	MAP3K9	14	70722526	70809534
55971	BAIAP2L1	7	98291650	98401090
80853	KDM7A	7	140084746	140176983
221	ALDH3B1	11	68008578	68029282
9855	FARP2	2	241356285	241494841
1352	COX10	17	14069490	14231736
79602	ADIPOR2	12	1688574	1788674
57787	MARK4	19	45079288	45305284
115098	CCDC124	19	17933015	17943991
54879	ST7L	1	112523514	112620825
8277	TKTL1	X	154295795	154330350
113000	RPUSD1	16	784974	788397
115939	TSR3	16	1349240	1351878

9091	PIGQ		16	566995	584109
29116	MYLIP		6	16129086	16148248
1870	E2F2		1	23506438	23531233
65220	NADK		1	1751232	1780457
9265	CYTH3		7	6161776	6272644
53616	ADAM22		7	87934143	88202889
4257	MGST1		12	16347142	16609259
8993	PGLYRP1		19	46019153	46023053
4784	NFIX		19	12995608	13098796
6482	ST3GAL1		8	133454848	133571940
5980	REV3L		6	111299028	111483715
10747	MASP2		1	11026523	11047239
29954	POMT2		14	77274956	77320883
51534	VTA1		6	142147162	142224685
10048	RANBP9		6	13621498	13711835
51513	ETV7		6	36354091	36387800
7384	UQCRC1		3	48599002	48610976
83930	STARD3NL		7	38178222	38230671
25900	IFFO1		12	6538375	6556083
56920	SEMA3G		3	52433035	52445103
80199	FUZ		19	49806866	49817376
7752	ZNF200		16	3222325	3236221
920	CD4		12	6786858	6820799
10233	LRRC23		12	6873569	6914241
695	BTK	X		101349447	101390796
3077	HFE		6	26087281	26098343
2534	FYN		6	111660332	111873452
3097	HIVEP2		6	142751469	142956698
9902	MRC2		17	62627670	62693597
55727	BTBD7		14	93237550	93333092
9546	APBA3		19	3750772	3761692
51099	ABHD5		3	43690108	43734371
26993	AKAP8L		19	15380050	15419141
54799	MBTD1		17	51177425	51260163
54476	RNF216		7	5620047	5781696
54902	TTC19		17	15999784	16045015
5329	PLAUR		19	43646095	43670547
54443	ANLN		7	36389821	36453791
23637	RABGAP1		9	122940833	123104866
7305	TYROBP		19	35904401	35908295
672	BRCA1		17	43044295	43170245
4007	PRICKLE3	X		49174802	49186528
4057	LTF		3	46435645	46485234

57658	CALCOCO1		12	53708517	53727745
5704	PSMC4		19	39971165	39981764
51629	SLC25A39		17	44319625	44324870
51667	NUB1		7	151341699	151378449
5238	PGM3		6	83161150	83193936
55703	POLR3B		12	106357748	106510198
23357	ANGEL1		14	76786009	76826246
1774	DNASE1L1	X		154401236	154412112
10046	MAMLD1	X		150361422	150514178
923	CD6		11	60971680	61020377
23649	POLA2		11	65261920	65305589
10138	YAF2		12	42157104	42238349
10771	ZMYND11		10	134465	254637
7454	WAS	X		48676596	48691427
9782	MATR3		5	139293674	139331677
3479	IGF1		12	102395874	102481744
92745	SLC38A5	X		48458537	48470260
10928	RALBP1		18	9475009	9538114
22902	RUFY3		4	70704204	70808619
55622	TTC27		2	32628032	32821051
56981	PRDM11		11	45095806	45235110
3082	HGF		7	81699010	81770438
55734	ZFP64		20	52051663	52204308
23034	SAMD4A		14	54566117	54793315
1992	SERPINB1		6	2832332	2841959
6683	SPAST		2	32063556	32157637
114879	OSBPL5		11	3087107	3166739
9716	AQR		15	34851782	34969742
51507	RTF2		20	56468585	56519449
9921	RNF10		12	120533480	120577588
55778	ZNF839		14	102317377	102342702
57476	GRAMD1B		11	123358428	123627774
9821	RB1CC1		8	52622458	52745843
23071	ERP44		9	99979185	100099000
211	ALAS1		3	52198086	52214327
11215	AKAP11		13	42272152	42323261
1244	ABCC2		10	99782640	99852594
50619	DEF6		6	35297818	35321771
51177	PLEKHO1		1	150149183	150164720
2730	GCLM		1	93885199	93909456
55635	DEPDC1		1	68474152	68497221
25901	CCDC28A		6	138773509	138793319
58528	RRAGD		6	89364616	89412273

3298	HSF2	6	122399551	122433119
51230	PHF20	20	35771974	35950370
1890	TYMP	22	50525752	50530032
11231	SEC63	6	107867756	107958208
355	FAS	10	88990531	89017059
960	CD44	11	35138882	35232402
57823	SLAMF7	1	160739057	160754821
11119	BTN3A1	6	26402237	26415208
3459	IFNGR1	6	137197485	137219449
7444	VRK2	2	57907629	58159920
65980	BRD9	5	850291	892801
56938	ARNTL2	12	27332854	27425289
9774	BCLAF1	6	136256627	136289851
286	ANK1	8	41653220	41896762
51106	TFB1M	6	155256134	155314493
3149	HMGB3	X	150980509	150990771
23279	NUP160	11	47778087	47848555
578	BAK1	6	33572547	33580293
2896	GRN	17	44345246	44353106
57514	ARHGAP31	3	119294383	119420714
55166	CENPQ	6	49463370	49493107
10908	PNPLA6	19	7534004	7561764
10061	ABCF2	7	151211484	151227205
54480	CHPF2	7	151232489	151238827
57554	LRRC7	1	69567922	70151945
9846	GAB2	11	78215293	78418348
535	ATP6V0A1	17	42458844	42522582
8554	PIAS1	15	68054309	68198603
9497	SLC4A7	3	27372721	27484420
5606	MAP2K3	17	21284672	21315232
9168	TMSB10	2	84905656	84906671
25897	RNF19A	8	100257060	100336218
26751	SH3YL1	2	217730	266398
55789	DEPDC1B	5	60596912	60700190
8439	NSMAF	8	58583508	58659853
7077	TIMP2	17	78852977	78925387
55773	TBC1D23	3	100260992	100325251
26009	ZZZ3	1	77562416	77683419
27175	TUBG2	17	42659284	42667006
51121	RPL26L1	5	172958729	172969771
2324	FLT4	5	180601506	180649624
54888	NSUN2	5	6599239	6633291
4238	MFAP3	5	154038906	154220478

55300	PI4K2B		4	25160663	25279204
27430	MAT2B		5	163503114	163519354
7204	TRIO		5	14143342	14532128
23274	CLEC16A		16	10944564	11182186
999	CDH1		16	68737292	68835541
23035	PHLPP2		16	71637835	71724701
54896	SLC66A1		1	19312326	19329300
3631	INPP4A		2	98444854	98594392
5685	PSMA4		15	78540405	78552417
55341	LSG1		3	194640791	194672463
140876	RIPOR3		20	50586108	50691542
55775	TDP1		14	89954939	90044764
822	CAPG		2	85394753	85418432
1175	AP2S1		19	46838136	46850846
54802	TRIT1		1	39838110	39883511
54875	CNTLN		9	17134982	17503923
8481	OFD1	X		13734745	13769357
2824	GPM6B	X		13770939	13938638
64848	YTHDC2		5	113513694	113595285
51606	ATP6V1H		8	53715543	53843558
5431	POLR2B		4	56977722	57031158
116984	ARAP2		4	35948221	36244514
7175	TPR		1	186311652	186375693
7504	XK	X		37685791	37732130
6322	SCML1	X		17737449	17754988
55841	WWC3	X		10015254	10144474
27146	FAM184B		4	17629306	17781621
9734	HDAC9		7	18086949	19002416
26262	TSPAN17		5	176647387	176659054
50484	RRM2B		8	102204502	102238961
608	TNFRSF17		16	11965210	11968068
55187	VPS13D		1	12230030	12512047
23196	FAM120A		9	93451685	93566112
1298	COL9A2		1	40300489	40317813
9563	H6PD		1	9234774	9271337
9341	VAMP3		1	7771296	7781432
3604	TNFRSF9		1	7915871	7943165
4052	LTBP1		2	32946953	33399509
5982	RFC2		7	74231499	74254458
57492	ARID1B		6	156776020	157210779
81037	CLPTM1L		5	1317752	1345099
23327	NEDD4L		18	58044226	58401540
50943	FOXP3	X		49250438	49270477

89801	PPP1R3F	X	49269793	49301461
3074	HEXB	5	74640023	74722647
79810	PTCD2	5	72320367	72368395
51474	LIMA1	12	50175788	50283546
9498	SLC4A8	12	51391317	51515763
84067	FAM160A2	11	6211345	6234711
5888	RAD51	15	40694774	40732340
10721	POLQ	3	121431427	121546641
1535	CYBA	16	88643289	88651054
84321	THOC3	5	175917873	176034680
23593	HEBP2	6	138403531	138422197
54477	PLEKHA5	12	19129752	19376400
55761	TTC17	11	43358920	43494933
4826	NNAT	20	37521206	37523690
83714	NRIP2	12	2825348	2835544
27095	TRAPPC3	1	36136570	36156053
29085	PHPT1	9	136848724	136851027
51742	ARID4B	1	235131634	235328219
5798	PTPRN	2	219289623	219309648
23095	KIF1B	1	10210570	10381603
25771	TBC1D22A	22	46762617	47175699
23224	SYNE2	14	63761899	64226433
57475	PLEKHH1	14	67533290	67589612
2581	GALC	14	87837820	87993665
3757	KCNH2	7	150944961	150978321
26999	CYFIP2	5	157266079	157395595
5610	EIF2AK2	2	37099210	37157065
57602	USP36	17	78787381	78841441
55609	ZNF280C	X	130202707	130268899
7185	TRAF1	9	120902393	120929173
55540	IL17RB	3	53846568	53865794
5318	PKP2	12	32790755	32896777
639	PRDM1	6	105993463	106109939
57458	TMCC3	12	94567122	94650557
57504	MTA3	2	42494569	42756947
8975	USP13	3	179653040	179789401
5218	CDK14	7	90466424	91210590
29927	SEC61A1	3	128051641	128071683
55781	RIOK2	5	97160867	97183247
1608	DGKG	3	186105668	186362234
64718	UNKL	16	1363205	1414751
64761	PARP12	7	140023749	140062951
6894	TARBP1	1	234391313	234479179

4084	MXD1		2	69897688	69942945
5128	CDK17		12	96278261	96400480
8531	YBX3		12	10699089	10723323
55359	STYK1		12	10618923	10674318
65125	WNK1		12	752579	911452
11054	OGFR		20	62804835	62814000
55650	PIGV		1	26787054	26800659
51660	MPC1		6	166364919	166383013
30	ACAA1		3	38103129	38137242
6433	SFSWAP		12	131711081	131799738
10188	TNK2		3	195863364	195911945
65056	GPBP1		5	57173948	57264679
1431	CS		12	56271699	56300391
63916	ELMO2		20	46366050	46432985
1975	EIF4B		12	53006282	53042215
6141	RPL18		19	48615328	48619184
79763	ISOC2		19	55452985	55462343
29924	EPN1		19	55675226	55709858
8776	MTMR1	X		150692971	150765108
2817	GPC1		2	240435663	240468076
3029	HAGH		16	1795620	1827157
841	CASP8		2	201233443	201287711
51768	TM7SF3		12	26971579	27014434
10402	ST3GAL6		3	98732236	98821201
50937	CDON		11	125955796	126063335
28996	HIPK2		7	139561570	139777998
23534	TNPO3		7	128954180	129055173
8625	RFXANK		19	19192229	19201869
6558	SLC12A2		5	128083766	128189677
28966	SNX24		5	122843439	123029354
10347	ABCA7		19	1039997	1065572
55711	FAR2		12	29149103	29340980
22904	SBNO2		19	1107637	1174268
10362	HMG20B		19	3572777	3579088
10203	CALCRL		2	187341964	187448460
54887	UHRF1BP1		6	34792015	34883138
3843	IPO5		13	97953658	98024296
5586	PKN2		1	88684222	88836255
55388	MCM10		10	13161554	13211104
3735	KARS1		16	75627474	75648643
10954	PDIA5		3	123067025	123225227
55633	TBC1D22B		6	37257772	37332970
4710	NDUFB4		3	120596328	120602507

9748	SLK		10	103967140	104029233
1308	COL17A1		10	104031286	104085880
119391	GSTO2		10	104268873	104304950
55176	SEC61A2		10	12129637	12169961
51665	ASB1		2	238426742	238452250
8621	CDK13		7	39950121	40099580
10797	MTHFD2		2	74198610	74217565
84679	SLC9A7	X		46599251	46759118
4904	YBX1		1	42682418	42703805
5525	PPP2R5A		1	212285410	212361853
1994	ELAVL1		19	7958573	8005659
57609	DIP2B		12	50504985	50748657
259266	ASPM		1	197084127	197146694
8832	CD84		1	160541095	160579516
6688	SPI1		11	47354860	47378547
30834	ZNRD1		6	30058899	30064909
744	MPPED2		11	30384493	30586872
51208	CLDN18		3	137998735	138033655
2263	FGFR2		10	121478332	121598458
51015	ISOC1		5	129094749	129114028
55623	THUMPD1		16	20702816	20742084
26153	KIF26A		14	104138723	104180894
57623	ZFAT		8	134477788	134713049
9650	MTFR1		8	65644734	65771261
4649	MYO9A		15	71822291	72118577
3422	IDI1		10	1039152	1049170
6672	SP100		2	230415942	230545606
1316	KLF6		10	3775996	3785281
4756	NEO1		15	73051710	73305205
23471	TRAM1		8	70573218	70608416
9399	STOML1		15	73978926	73994622
7158	TP53BP1		15	43403061	43510728
55699	IARS2		1	220094132	220148041
89795	NAV3		12	77324641	78213010
79641	ROGDI		16	4796968	4802880
865	CBFB		16	67028984	67101058
3430	IFI35		17	43006740	43014456
4205	MEF2A		15	99565417	99716466
55593	OTUD5	X		48922028	48958386
7030	TFE3	X		49028726	49043410
4943	TBC1D25	X		48539714	48562609
2182	ACSL4	X		109624244	109733403
27238	GPKOW	X		49113407	49123735

56850	GRIPAP1	X		48973720	49002264
55771	PRR11		17	59155732	59206709
9741	LAPTM4A		2	20032650	20051628
51447	IP6K2		3	48688003	48740353
55133	SRBD1		2	45388680	45612165
22933	SIRT2		19	38878555	38899862
27248	ERLEC1		2	53787044	53818819
5526	PPP2R5B		11	64917553	64934475
5837	PYGM		11	64746389	64759974
375449	MAST4		5	66596361	67169595
54549	SDK2		17	73334384	73644445
10294	DNAJA2		16	46955362	46973674
139341	FUNDC1	X		44523639	44542859
483	ATP1B3		3	141876124	141926549
10681	GNB5		15	52115105	52191369
5873	RAB27A		15	55202966	55319113
7353	UFD1		22	19449911	19479202
4040	LRP6		12	12116025	12267044
8518	ELP1		9	108866898	108934328
6710	SPTB		14	64746283	64879907
27071	DAPP1		4	99816827	99870190
23446	SLC44A1		9	105244622	105439171
23293	SMG6		17	2059839	2303785
8218	CLTCL1		22	19179473	19291719
10272	FSTL3		19	676392	683392
55658	RNF126		19	647526	663233
23210	JMJD6		17	76712832	76726799
5423	POLB		8	42338454	42371808
55062	WIPI1		17	68420948	68457513
3340	NDST1		5	150485818	150558211
10610	ST6GALNAC2		17	76565377	76586956
26986	PABPC1		8	100685816	100722809
29105	CFAP20		16	58113592	58129381
1459	CSNK2A2		16	58157907	58198106
6949	TCOF1		5	150357629	150400308
490	ATP2B1		12	89588049	89709300
11320	MGAT4A		2	98619106	98731132
79658	ARHGAP10		4	147732063	148072776
6196	RPS6KA2		6	166409364	166906451
22846	VASH1		14	76761468	76783015
9319	TRIP13		5	892884	919357
537	ATP6AP1	X		154428645	154436516
6929	TCF3		19	1609290	1652615

9130	FAM50A	X		154444141	154450654
60343	FAM3A	X		154506159	154516242
79901	CYBRD1		2	171522247	171558129
5134	PDCD2		6	170575295	170584692
23503	ZFYVE26		14	67727374	67816590
224	ALDH3A2		17	19648136	19685760
7037	TFRC		3	196027183	196082096
7224	TRPC5	X		111774315	112082776
3028	HSD17B10	X		53431258	53434370
3161	HMMR		5	163460203	163491941
2213	FCGR2B		1	161663147	161678654
4775	NFATC3		16	68084751	68229259
51095	TRNT1		3	3126933	3150879
37	ACADVL		17	7217125	7225266
23291	FBXW11		5	171861549	172006873
9744	ACAP1		17	7336529	7351477
51009	DERL2		17	5471254	5486811
79041	TMEM38A		19	16661139	16690023
949	SCARB1		12	124776856	124882668
4171	MCM2		3	127598410	127622436
56666	PANX2		22	50170731	50180295
5151	PDE8A		15	84980440	85139145
1183	CLCN4	X		10156975	10237660
54475	NLE1		17	35128730	35142304
5523	PPP2R3A		3	135965728	136147894
10170	DHRS9		2	169064789	169096167
10644	IGF2BP2		3	185643130	185825042
8301	PICALM		11	85957175	86069882
54825	CDHR2		5	176542511	176595974
7251	TSG101		11	18468336	18526951
771	CA12		15	63321378	63381846
59277	NTN4		12	95657807	95791189
9187	SLC24A1		15	65611366	65660995
56005	MYDGF		19	4641374	4670362
51175	TUBE1		6	112070663	112087529
8672	EIF4G3		1	20806292	21176888
51512	GTSE1		22	46296870	46330810
10512	SEMA3C		7	80742538	80922359
55020	TTC38		22	46267961	46294008
23151	GRAMD4		22	46576012	46679790
9620	CELSR1		22	46360834	46537620
9462	RASAL2		1	178094104	178484147
9605	VPS9D1		16	89707134	89720898

4140	MARK3		14	103385392	103503831
64778	FNDC3B		3	172039578	172401669
5337	PLD1		3	171600404	171810950
284403	WDR62		19	36054881	36105108
1739	DLG1		3	197042560	197299330
9733	SART3		12	108522214	108561400
55843	ARHGAP15		2	143091362	143768352
10179	RBM7		11	114400030	114414203
113235	SLC46A1		17	28394642	28407197
5362	PLXNA2		1	208022242	208244384
10615	SPAG5		17	28577565	28599025
32	ACACB		12	109116595	109268226
9618	TRAF4		17	28744005	28750956
55824	PAG1		8	80967810	81112068
22978	NT5C2		10	103087185	103277605
6813	STXBP2		19	7636881	7647873
5609	MAP2K7		19	7903843	7914478
5915	RARB		3	25174332	25597932
7155	TOP2B		3	25597905	25664907
29089	UBE2T		1	202331657	202341980
23032	USP33		1	77695987	77759852
4034	LRCH4		7	100574011	100586129
143684	FAM76B		11	95768953	95790409
51548	SIRT6		19	4174109	4182566
7319	UBE2A	X		119574536	119591083
4133	MAP2		2	209424058	209734118
56922	MCCC1		3	183015218	183116075
27074	LAMP3		3	183122215	183163839
2191	FAP		2	162170684	162245151
27334	P2RY10	X		78945332	78963727
4898	NRDC		1	51789191	51878937
57690	TNRC6C		17	77959240	78108835
9139	CBFA2T2		20	33490075	33650036
51150	SDF4		1	1216908	1232067
54472	TOLLIP		11	1274371	1309654
9984	THOC1		18	214520	268050
114880	OSBPL6		2	178194481	178402893
6507	SLC1A3		5	36606355	36688334
56925	LXN		3	158645822	158672648
11262	SP140		2	230203110	230313215
7145	TNS1		2	217799588	218033982
8556	CDC14A		1	100345001	100520277
10411	RAPGEF3		12	47734363	47771040

634	CEACAM1	19	42507304	42561234
29843	SENP1	12	48042897	48106079
51207	DUSP13	10	75094432	75109221
2222	FDFT1	8	11795573	11839304
1785	DNM2	19	10718079	10833488
2037	EPB41L2	6	130839347	131063322
26002	MOXD1	6	132296055	132401475
9817	KEAP1	19	10486125	10503558
55510	DDX43	6	73394828	73417566
131544	CRYBG3	3	97822011	97944984
8671	SLC4A4	4	71062667	71572087
6595	SMARCA2	9	1980290	2193624
27244	SESN1	6	108986437	109094819
11043	MID2	X	107825755	107927193
5663	PSEN1	14	73136418	73223691
5933	RBL1	20	36996349	37095997
114819	CROCCP3	1	16467436	16499257
10403	NDC80	18	2571557	2616635
260425	MAGI3	1	113390515	113685923
1286	COL4A4	2	227002714	227164453
6932	TCF7	5	134114681	134151865
28962	OSTM1	6	108041409	108165854
57092	PCNP	3	101574180	101594465
55218	EXD2	14	69191511	69244020
9262	STK17B	2	196133583	196176503
440515	ZNF506	19	19785839	19821751
9812	DELE1	5	141923855	141942047
5205	ATP8B1	18	57646426	57803315
3595	IL12RB2	1	67307364	67398724
6604	SMARCD3	7	151238764	151277896
55100	WDR70	5	37379285	37753435
55437	STRADB	2	201387858	201480846
9689	BZW1	2	200810594	200827338
114899	C1QTNF3	5	34019448	34043832
57579	FAM135A	6	70412941	70561174
1310	COL19A1	6	69866556	70212468
7188	TRAF5	1	211326615	211374946
25929	GEMIN5	5	154887411	154938211
23085	ERC1	12	990509	1495933
7514	XPO1	2	61477849	61538626
11342	RNF13	3	149812770	149962139
23033	DOP1A	6	83067666	83171350
594	BCKDHB	6	80106647	80346270

79670	TUT7	9	86287733	86354454
9706	ULK2	17	19770829	19867936
5351	PLOD1	1	11934205	11975538
5019	OXCT1	5	41730065	41870425
79891	ZNF671	19	57719751	57727624
55659	ZNF416	19	57571566	57578911
9422	ZNF264	19	57191500	57222846
2195	FAT1	4	186587794	186726722
25978	CHMP2B	3	87227271	87255556
64744	SMAP2	1	40344850	40423326
10269	ZMPSTE24	1	40258041	40294180
84273	NOA1	4	56963350	56977606
5978	REST	4	56907876	56966808
3034	HAL	12	95972662	95996365
51729	WBP11	12	14784582	14803486
10890	RAB10	2	26034084	26137454
22924	MAPRE3	2	26970637	27027219
2219	FCN1	9	134903232	134917912
55892	MYNN	3	169772831	169789716
9522	SCAMP1	5	78360611	78480739
5550	PREP	6	105273218	105454062
57531	HACE1	6	104728094	104859919
81929	SEH1L	18	12947133	12987536
57590	WDFY1	2	223855716	223945357
5016	OVGP1	1	111414319	111427735
29957	SLC25A24	1	108134043	108200849
4216	MAP3K4	6	160991727	161117385
29992	PILRA	7	100367530	100400096
57549	IGSF9	1	159927039	159945613
5243	ABCB1	7	87503017	87713323
7760	ZNF213	16	3129777	3142804
23259	DDHD2	8	38225218	38275558
22996	TTC39A	1	51287258	51345116
2060	EPS15	1	51354263	51519266
4998	ORC1	1	52372829	52404423
55054	ATG16L1	2	233210051	233295674
55230	USP40	2	233475520	233566782
3301	DNAJA1	9	33025273	33039907
51510	CHMP5	9	33264879	33282070
27102	EIF2AK1	7	6022247	6059175
51314	NME8	7	37848597	37900397
54749	EPDR1	7	37683843	37951936
29887	SNX10	7	26291862	26374383

10573	MRPL28		16	366969	371289
3049	HBQ1		16	180459	181179
80271	ITPKC		19	40717112	40740860
4680	CEACAM6		19	41750977	41772211
10959	TMED2		12	123584533	123598582
54469	ZFAND6		15	80059568	80138393
5475	PPEF1	X		18675909	18827921
7462	LAT2		7	74199652	74229834
79796	ALG9		11	111782195	111871581
23645	PPP1R15A		19	48872421	48876058
7205	TRIP6		7	100867387	100873454
43	ACHE		7	100889994	100896974
2512	FTL		19	48965309	48966879
581	BAX		19	48954815	48961798
9509	ADAMTS2		5	179110853	179345461
1071	CETP		16	56961923	56983845
55239	OGFOD1		16	56451521	56479104
79944	L2HGDH		14	50237563	50312229
57542	KLHL42		12	27780048	27803040
2778	GNAS		20	58839718	58911192
51290	ERGIC2		12	29337352	29381189
6790	AURKA		20	56369389	56392337
57091	CASS4		20	56412112	56460387
8544	PIR	X		15384799	15493564
55741	EDEM2		20	35115364	35147336
22974	TPX2		20	31739271	31801805
23348	DOCK9		13	98793429	99086625
8455	ATRN		20	3471018	3651118
54498	SMOX		20	4120980	4187747
6614	SIGLEC1		20	3686970	3707128
25943	C20orf194		20	3249305	3407625
79175	ZNF343		20	2481817	2524702
22803	XRN2		20	21303331	21389825
55857	KIZ		20	21125983	21246622
54997	TESC		12	117038923	117099479
55423	SIRPG		20	1629152	1657779
8550	MAPKAPK5		12	111842228	111902222
55184	DZANK1		20	18383367	18467281
84678	KDM2B		12	121429096	121581023
4938	OAS1		12	112906783	112933222
22895	RPH3A		12	112570380	112898881
8315	BRAP		12	111642146	111685956
10961	ERP29		12	112013348	112023449

2521	FUS		16	31180138	31191605
3476	IGBP1	X		70133447	70166324
57655	GRAMD1A		19	34994784	35026471
113540	CMTM1		16	66566393	66579137
332	BIRC5		17	78214186	78225636
7597	ZBTB25		14	64449106	64505213
25977	NECAP1		12	8076939	8097881
393	ARHGAP4	X		153907367	153934999
55760	DHX32		10	125836337	125896436
55668	GPATCH2L		14	76151916	76254342
645	BLVRB		19	40447765	40465764
6548	SLC9A1		1	27098809	27166981
10558	SPTLC1		9	92000087	92115413
5996	RGS1		1	192575763	192580024
83719	YPEL3		16	30092314	30096915
11213	IRAK3		12	66189195	66254622
4069	LYZ		12	69348381	69354234
10081	PDCD7		15	65117379	65133808
51726	DNAJB11		3	186567403	186585800
84572	GNPTG		16	1351931	1364113
8761	PABPC4		1	39560816	39576790
57192	MCOLN1		19	7522624	7534009
55066	PDPR		16	70114332	70162537
2734	GLG1		16	74447427	74607144
24137	KIF4A	X		70290104	70420886
113878	DTX2		7	76461676	76505995
4897	NRCAM		7	108147623	108456717
23335	WDR7		18	56651343	57036606
9352	TXNL1		18	56597209	56651600
3568	IL5RA		3	3066324	3126613
368	ABCC6		16	16148928	16223522
54918	CMTM6		3	32481312	32502852
3655	ITGA6		2	172427354	172506459
54890	ALKBH5		17	18183078	18209954
2099	ESR1		6	151656691	152129619
285	ANGPT2		8	6499632	6563409
5720	PSME1		14	24136163	24138967
55012	PPP2R3C		14	35085467	35122517
1053	CEBPE		14	23117304	23119616
11198	SUPT16H		14	21351476	21384019
26277	TINF2		14	24239643	24242674
57556	SEMA6A		5	116443555	116574823
79968	WDR76		15	43826980	43868412

4637	MYL6		12	56158346	56163496
26523	AGO1		1	35869808	35930532
63967	CLSPN		1	35720218	35769978
201294	UNC13D		17	75827225	75844552
79157	MFSD11		17	76735865	76781449
55105	GPATCH2		1	217426992	217631090
8318	CDC45		22	19479457	19520612
55350	VNN3		6	132722787	132736935
9209	LRRFIP2		3	37052626	37183689
9942	XYLB		3	38346760	38421348
10013	HDAC6	X		48801377	48824982
990	CDC6		17	40287879	40304657
139596	UPRT	X		75156388	75304885
23468	CBX5		12	54230942	54280133
51430	SUCO		1	172532349	172611833
55151	TMEM38B		9	105694541	105776629
54187	NANS		9	98056732	98083077
55357	TBC1D2		9	98199011	98255649
55280	CWF19L1		10	100232298	100267680
57715	SEMA4G		10	100969518	100985871
9044	BTAF1		10	91923770	92030325
10580	SORBS1		10	95311771	95561414
25805	BAMBI		10	28677510	28682932
51322	WAC		10	28532493	28623112
1390	CREM		10	35126791	35212958
284422	SMIM24		19	3473986	3480525
10321	CRISP3		6	49727384	49744437
2289	FKBP5		6	35573585	35728583
55168	MRPS18A		6	43671303	43687791
114327	EFHC1		6	52362123	52529886
1832	DSP		6	7541617	7586714
3717	JAK2		9	4984390	5129948
3594	IL12RB1		19	18058995	18098944
25	ABL1		9	130713016	130887675
51100	SH3GLB1		1	86704570	86748184
8317	CDC7		1	91500851	91525764
84144	SYDE2		1	85156889	85201016
5125	PCSK5		9	75890644	76362975
6319	SCD		10	100347233	100364826
11018	TMED1		19	10832067	10836318
3983	ABLIM1		10	114431113	114768061
79956	ERMP1		9	5765076	5833117
57828	CATSPERG		19	38335775	38370943

5714	PSMD8	19	38374536	38383824
54620	FBXL19	16	30923055	30948783
513	ATP5F1D	19	1241746	1244825
51257	MARCHF2	19	8413270	8439017
4713	NDUFB7	19	14566078	14572066
997	CDC34	19	531760	542092
4507	MTAP	9	21802636	21937651
4616	GADD45B	19	2476122	2478259
2872	MKNK2	19	2037465	2051244
421	ARVCF	22	19969896	20016823
5902	RANBP1	22	20115938	20127355
84861	KLHL22	22	20441519	20495844
3053	SERPIND1	22	20774113	20787720
91319	DERL3	22	23834503	23839128
23786	BCL2L13	22	17628855	17730855
2687	GGT5	22	24219654	24245142
23541	SEC14L2	22	30396941	30425303
266629	SEC14L3	22	30447959	30472017
23384	SPECC1L	22	24270817	24417739
51733	UPB1	22	24494107	24528390
8220	ESS2	22	19130279	19144684
157	GRK3	22	25564675	25729294
7290	HIRA	22	19330698	19447450
3956	LGALS1	22	37675636	37679802
113791	PIK3IP1	22	31281594	31292534
402055	SRRD	22	26483877	26494658
11078	TRIOBP	22	37697048	37776556
23464	GCAT	22	37807905	37817176
51386	EIF3L	22	37848868	37889407
85377	MICALL1	22	37905657	37942822
5435	POLR2F	22	37952607	38041915
9681	DEPDC5	22	31753867	31908033
9463	PICK1	22	38056311	38075701
23331	TTC28	22	27978014	28679865
79019	CENPM	22	41938737	41947152
387590	TPTEP1	22	16601887	16698742
11144	DMC1	22	38518948	38570204
7494	XBP1	22	28794555	28800597
51493	RTCB	22	32387582	32412248
25793	FBXO7	22	32474676	32498829
9567	GTPBP1	22	38705742	38738299
9701	PPP6R2	22	50343304	50445090
1727	CYB5R3	22	42617840	42649392

25807	RHBDD3	22	29259872	29268209
10042	HMGXB4	22	35257452	35295807
10043	TOM1	22	35299275	35347992
164668	APOBEC3H	22	39097224	39104067
410	ARSA	22	50622754	50628173
23170	TTLL12	22	43166622	43187134
5155	PDGFB	22	39223359	39244982
6122	RPL3	22	39312882	39320389
55954	ZMAT5	22	29730956	29767011
84164	ASCC2	22	29788609	29838304
8897	MTMR3	22	29883169	30030868
54471	MIEF1	22	39499432	39518132
80832	APOL4	22	36189124	36204840
8542	APOL1	22	36253071	36267530
25828	TXN2	22	36467046	36481640
23313	KIAA0930	22	45190338	45240769
10478	SLC25A17	22	40769630	40819399
9978	RBX1	22	40951347	40973309
2033	EP300	22	41092592	41180077
84844	PHF5A	22	41459717	41468692
64781	CERK	22	46684410	46738252
23774	BRD1	22	49773283	49827512
9889	ZBED4	22	49853844	49890080
23209	MLC1	22	50059391	50085426
63874	ABHD4	14	22598237	22613215
23351	KHNYN	14	24429286	24441843
1511	CTSG	14	24573518	24576250
2999	GZMH	14	24606480	24609699
3002	GZMB	14	24630954	24634267
10419	PRMT5	14	22920525	22929408
5427	POLE2	14	49643555	49688422
51199	NIN	14	50719763	50831162
114088	TRIM9	14	50975262	51096061
1033	CDKN3	14	54396849	54420218
10175	CNIH1	14	54423561	54441391
51382	ATP6V1D	14	67294371	67360265
26499	PLEK2	14	67386984	67412167
161394	SAMD15	14	77376689	77391497
9517	SPTLC2	14	77505997	77616637
79890	RIN3	14	92513781	92688994
22938	SNW1	14	77717599	77761207
3705	ITPK1	14	92936914	93116320
5494	PPM1A	14	60245752	60299087

3091	HIF1A	14	61695513	61748259
6430	SRSF5	14	69726900	69772005
1983	EIF5	14	103333544	103345025
79882	ZC3H14	14	88562970	88627596
51527	GSKIP	14	96363452	96387288
7443	VRK1	14	96797382	96931722
5700	PSMC1	14	90256527	90275429
9252	RPS6KA5	14	90847861	91060641
60686	C14orf93	14	22985894	23010166
5693	PSMB5	14	23016543	23035230
328	APEX1	14	20455191	20457772
8106	PABPN1	14	23321289	23326185
51550	CINP	14	102341102	102362916
6729	SRP54	14	34981957	35029567
5106	PCK2	14	24094053	24110598
80344	DCAF11	14	24114195	24125242
5687	PSMA6	14	35278633	35317493
4792	NFKBIA	14	35401513	35404749
51016	EMC9	14	24138959	24141591
5721	PSME2	14	24143362	24147570
9985	REC8	14	24171853	24180257
5411	PNN	14	39175183	39183220
5360	PLTP	20	45898621	45912155
4318	MMP9	20	46008908	46016561
26090	ABHD12	20	25294742	25390835
9837	GINS1	20	25407673	25452628
55245	UQCC1	20	35302566	35412031
23613	ZMYND8	20	47209214	47356889
51098	IFT52	20	43590937	43647299
4605	MYBL2	20	43667019	43716495
55969	RAB5IF	20	36605779	36612557
23394	ADNP	20	50888916	50931437
1477	CSTF1	20	56392371	56406362
8480	RAE1	20	57351223	57379211
7165	TPD52L2	20	63865228	63891545
1522	CTSZ	20	58995185	59007254
51012	PRELID3B	20	59033145	59042809
26164	MTG2	20	62183029	62203568
5688	PSMA7	20	62136733	62143440
28231	SLCO4A1	20	62642503	62685785
4923	NTSR1	20	62708836	62762771
55257	MRGBP	20	62796473	62801729
54976	C20orf27	20	3753508	3767781

140862	ISM1	20	13221274	13300651
1457	CSNK2A1	20	472498	543835
10483	SEC23B	20	18507520	18561415
10398	MYL9	20	36541497	36551447
140711	TLDC2	20	36876121	36894235
51340	CRNKL1	20	20034368	20056046
25939	SAMHD1	20	36890229	36951843
63905	MANBAL	20	37289638	37317260
182	JAG1	20	10637684	10673999
9675	TTI1	20	37983007	38033461
1869	E2F1	20	33675477	33686385
128866	CHMP4B	20	33811348	33854366
671	BPI	20	38304150	38337505
81610	FAM83D	20	38926312	38953106
60625	DHX35	20	38962299	39039723
84557	MAP1LC3A	20	34546854	34560345
10005	ACOT8	20	45841721	45857405
22850	ADNP2	18	80109262	80147523
79863	RBFA	18	80034389	80050651
64863	METTL4	18	2537525	2571509
9663	LPIN2	18	2916994	3013315
23347	SMCHD1	18	2655726	2805017
10627	MYL12A	18	3247481	3256236
57534	MIB1	18	21704957	21870957
5932	RBBP8	18	22798261	23026488
340547	VSIG1	X	108044970	108079184
5716	PSMD10	X	108084207	108091549
115201	ATG4A	X	108091668	108154671
412	STS	X	7147237	7804358
10857	PGRMC1	X	119236245	119244466
55916	NXT2	X	109535781	109544690
79868	ALG13	X	111665811	111760649
5634	PRPS2	X	12791355	12824222
64743	WDR13	X	48590042	48608869
215	ABCD1	X	153724856	153744755
5355	PLP2	X	49171898	49175235
660	BMX	X	15464246	15556529
5973	RENBP	X	153935269	153944687
2000	ELF4	X	130063955	130110716
55613	MTMR8	X	64268081	64395452
340554	ZC3H12B	X	65366638	65507887
2332	FMR1	X	147911919	147951125
11040	PIM2	X	48913182	48919024

27344	PCSK1N	X		48831096	48835610
2623	GATA1	X		48786562	48794311
6611	SMS	X		21940709	21994837
6102	RP2	X		46837043	46882358
5127	CDK16	X		47217860	47229997
9468	PCYT1B	X		24558087	24672677
959	CD40LG	X		136648158	136660390
5303	PIN4	X		72181353	72302926
158866	ZDHHC15	X		75368427	75523502
2491	CENPI	X		101098218	101163681
2717	GLA	X		101393273	101408012
51566	ARMCX3	X		101622797	101627843
80183	RUBCNL		13	46334681	46438190
10673	TNFSF13B		13	108251240	108308484
22862	FNDC3A		13	48975912	49209779
2862	MLNR		13	49220338	49222377
81617	CAB39L		13	49308650	49444064
688	KLF5		13	73054976	73077541
54	ACP5		19	11574660	11579008
8874	ARHGEF7		13	111114559	111305737
143	PARP4		13	24420931	24512778
23078	VWA8		13	41566835	41961120
160851	DGKH		13	42040036	42256578
10562	OLFM4		13	53028813	53052057
8717	TRADD		16	67154185	67159909
11151	CORO1A		16	30182827	30189076
79767	ELMO3		16	67199111	67204029
57149	LYRM1		16	20899868	20925006
55815	TSNAXIP1		16	67806765	67832148
51090	PLLP		16	57248547	57284672
65009	NDRG4		16	58462846	58513628
79918	SETD6		16	58515479	58523842
3038	HAS3		16	69105653	69118719
79613	TANGO6		16	68843531	69085182
25839	COG4		16	70480568	70523560
55512	SMPD3		16	68358327	68448508
9057	SLC7A6		16	68264516	68301823
80004	ESRP2		16	68229033	68238102
8312	AXIN1		16	287440	352723
8131	NPRL3		16	84271	138677
23417	MLYCD		16	83899115	83951445
4350	MPG		16	77007	85851
54550	NECAB2		16	83968632	84002776

9100	USP10	16	84699986	84779922
83716	CRISPLD2	16	84819985	84920768
4833	NME4	16	396725	410367
64788	LMF1	16	853634	981318
8140	SLC7A5	16	87830023	87869507
4210	MEFV	16	3242027	3256633
29904	EEF2K	16	22206278	22288738
6650	CAPN15	16	527712	554636
2935	GSPT1	16	11868128	11916082
6923	ELOB	16	2771414	2777280
23062	GGA2	16	23463542	23521995
NA	RRN3P2	16	29074976	29116718
23475	QPRT	16	29663279	29698699
29108	PYCARD	16	31201486	31203450
6810	STX4	16	31032889	31042975
10423	CDIPT	16	29858357	29863414
50615	IL21R	16	27402174	27452042
51760	SYT17	16	19167971	19268332
79838	TMC5	16	19410496	19499113
9738	CCP110	16	19523811	19553408
9810	RNF40	16	30761745	30776307
64799	IQCH	15	67254786	67502260
114294	LACTB	15	63121833	63142061
64927	TTC23	15	99136323	99251223
2184	FAH	15	80152490	80186946
57214	CEMIP	15	80779343	80951776
26015	RPAP1	15	41517176	41544269
30844	EHD4	15	41895933	41972557
79895	ATP8B4	15	49858238	50182817
2553	GABPB1	15	50275392	50355408
90427	BMF	15	40087890	40108892
80208	SPG11	15	44562696	44663678
57617	VPS18	15	40894450	40903975
7782	SLC30A4	15	45479606	45522755
26258	BLOC1S6	15	45587214	45615945
51201	ZDHHC2	8	17156482	17224799
55756	INTS9	8	28767661	28890242
8767	RIPK2	8	89757806	89791064
4683	NBN	8	89933331	90003228
8989	TRPA1	8	72019917	72075584
1666	DECR1	8	90001405	90053633
55284	UBE2W	8	73780097	73878910
10397	NDRG1	8	133237175	133302022

3574	IL7	8	78675743	78805523
83988	NCALD	8	101686542	102124907
64089	SNX16	8	81799581	81842866
51366	UBR5	8	102252273	102412759
79792	GSDMD	8	143553207	143563062
7264	TSTA3	8	143612618	143618048
55174	INTS10	8	19817391	19852083
10671	DCTN6	8	30156319	30183639
2936	GSR	8	30678066	30727846
8797	TNFRSF10A	8	23190452	23225102
157697	ERICH1	8	614746	738106
4173	MCM4	8	47960185	47978160
427	ASAH1	8	18055992	18084998
4924	NUCB1	19	48900312	48923372
2997	GYS1	19	48968130	48993310
4793	NFKBIB	19	38899700	38908893
55011	PIH1D1	19	49446298	49453497
84444	DOT1L	19	2163933	2232578
55111	PLEKHJ1	19	2230084	2237704
147700	KLC3	19	45333434	45351520
951	CD37	19	49335171	49343335
8175	SF3A2	19	2236824	2248655
4066	LYL1	19	13099033	13103161
4946	OAZ1	19	2269509	2273490
8677	STX10	19	13144058	13150383
56729	RETN	19	7669049	7670455
81857	MED25	19	49818282	49840383
729440	CCDC61	19	45995461	46021318
51231	VRK3	19	49976468	50025946
26017	FAM32A	19	16185380	16192046
22870	PPP6R1	19	55229779	55259017
93145	OLFM2	19	9853718	9936552
79744	ZNF419	19	57487711	57496098
10775	POP4	19	29606283	29617237
898	CCNE1	19	29811991	29824312
6217	RPS16	19	39433137	39435949
2091	FBL	19	39834458	39846379
9149	DYRK1B	19	39825350	39834201
208	AKT2	19	40230317	40285536
55702	YJU2	19	4247080	4269088
6510	SLC1A5	19	46774883	46788594
25865	PRKD2	19	46674275	46717127
26093	CCDC9	19	47255980	47273701

51343	FZR1	19	3506273	3538330
27113	BBC3	19	47220822	47232766
7040	TGFB1	19	41301587	41353922
22898	DENND3	8	141117278	141195808
1089	CEACAM4	19	41618971	41627074
10226	PLIN3	19	4838341	4867694
27181	SIGLEC8	19	51450847	51458456
29997	NOP53	19	47745546	47757058
4818	NKG7	19	51371606	51372701
2109	ETFB	19	51345169	51366388
29086	BABAM1	19	17267376	17281249
7297	TYK2	19	10350529	10380572
11140	CDC37	19	10391133	10420121
8775	NAPA	19	47487637	47515091
10567	RABAC1	19	41956681	41959321
10945	KDELR1	19	48382575	48391551
6320	CLEC11A	19	50723364	50725718
8778	SIGLEC5	19	51611927	51645545
3036	HAS1	19	51713112	51723991
1628	DBP	19	48630030	48637379
374882	TMEM205	19	11342776	11346518
64748	PLPPR2	19	11355386	11365698
5518	PPP2R1A	19	52190048	52229518
10661	KLF1	19	12884422	12887201
1777	DNASE2	19	12875209	12881466
79165	LENG1	19	54155161	54159721
29844	TFPT	19	54107013	54115675
27106	ARRDC2	19	18001132	18014102
11316	COPE	19	18899514	18919387
54555	DDX49	19	18919705	18929189
93436	ARMC6	19	19033575	19060311
23770	FKBP8	19	18531751	18544077
6324	SCN1B	19	35030470	35040449
80714	PBX4	19	19561707	19618693
2931	GSK3A	19	42230190	42242625
64763	ZNF574	19	42068477	42081552
199731	CADM4	19	43622368	43639850
9512	PMPCB	7	103297435	103329511
27000	DNAJC2	7	103312289	103344830
5445	PON2	7	95404862	95435329
3696	ITGB8	7	20330702	20415754
136319	MTPN	7	135926760	135977359
1687	GSDME	7	24698355	24757940

56829	ZC3HAV1	7	139043515	139109720
4967	OGDH	7	44606572	44709066
11033	ADAP1	7	897900	955407
22797	TFEC	7	115935148	116159896
857	CAV1	7	116525001	116561184
3199	HOXA2	7	27100354	27102686
79974	CPED1	7	120988697	121297442
1124	CHN2	7	29146569	29514328
2887	GRB10	7	50590063	50793462
84725	PLEKHA8	7	30027404	30130483
10392	NOD1	7	30424527	30478784
835	CASP2	7	143288215	143307696
51142	CHCHD2	7	56101573	56106476
3315	HSPB1	7	76302673	76304295
11333	PDAP1	7	99392048	99408597
8896	BUD31	7	99408641	99419616
26024	PTCD1	7	99416739	99466163
1577	CYP3A5	7	99648194	99679998
7586	ZKSCAN1	7	100015572	100041689
4521	NUDT1	7	2242222	2251146
7036	TFR2	7	100620416	100642779
55131	RBM28	7	128297685	128343908
84132	USP42	7	6104884	6161564
1174	AP1S1	7	101154456	101161596
56913	C1GALT1	7	7156934	7248616
6119	RPA3	7	7636518	7718607
10467	ZNHIT1	7	101218165	101224190
24146	CLDN15	7	101232092	101238820
2146	EZH2	7	148807383	148884321
95681	CEP41	7	130393771	130442433
643641	ZNF862	7	149838375	149867479
653857	ACTR3C	7	150243916	150323725
26157	GIMAP2	7	150685697	150693641
644	BLVRA	7	43758680	43807342
55665	URGCP	7	43875894	43926411
64940	STAG3L4	7	67302621	67362950
6009	RHEB	7	151466012	151520120
51422	PRKAG2	7	151556124	151877125
10652	YKT6	7	44200968	44214294
2218	FKTN	9	105558122	105653820
10927	SPIN1	9	88388430	88478694
23731	TMEM245	9	109015135	109119947
158471	PRUNE2	9	76611376	76906114

1955	MEGF9	9	120600811	120714470
9830	TRIM14	9	98069275	98119222
7464	CORO2A	9	98120975	98192637
7046	TGFBR1	9	99104038	99154192
10952	SEC61B	9	99222064	99230615
7091	TLE4	9	79571773	79726882
944	TNFSF8	9	114893343	114930595
203	AK1	9	127866486	127877675
55848	PLGRKT	9	5357971	5437925
23081	KDM4C	9	6720863	7175648
8939	FUBP3	9	130578965	130638352
10488	CREB3	9	35732598	35736999
23586	DDX58	9	32455302	32526208
8721	EDF1	9	136862119	136866308
169792	GLIS3	9	3824127	4348392
573	BAG1	9	33247820	33264720
56654	NPDC1	9	137039463	137046179
20	ABCA2	9	137007234	137028922
7763	ZFAND5	9	72351413	72365235
54619	CCNJ	10	96043394	96060870
2625	GATA3	10	8045378	8075198
3257	HPS1	10	98416198	98446947
10613	ERLIN1	10	100150094	100186033
8661	EIF3A	10	119033670	119080823
8029	CUBN	10	16823966	17129811
1787	TRDMT1	10	17137336	17202054
5599	MAPK8	10	48306639	48439360
11196	SEC23IP	10	119892730	119944657
59338	PLEKHA1	10	122374696	122442602
64072	CDH23	10	71396920	71815947
64115	VSIR	10	71747556	71773520
54462	CCSER2	10	84328586	84518521
657	BMPR1A	10	86756601	86932825
3988	LIPA	10	89213569	89414557
22849	CPEB3	10	92046692	92291078
79004	CUEDC2	10	102423245	102432584
91452	ACBD5	10	27195214	27242130
23185	LARP4B	10	806914	931705
26098	EDRF1	10	125719515	125764143
9644	SH3PXD2A	10	103594027	103855543
79991	STN1	10	103877569	103918184
8036	SHOC2	10	110919593	111013666
8030	CCDC6	10	59788747	59906556

219771	CCNY	10	35247025	35572669
27338	UBE2S	19	55399745	55407788
64081	PBLD	10	68282660	68333049
10900	RUNDC3A	17	44308413	44318670
22794	CASC3	17	40140318	40172171
51195	RAPGEFL1	17	40177010	40195656
5414	SEPTIN4	17	58520250	58544368
4591	TRIM37	17	58982638	59106921
5023	P2RX1	17	3896592	3916476
3837	KPNB1	17	47649476	47685505
9570	GOSR2	17	46923075	46975524
80279	CDK5RAP3	17	47967810	47981781
9487	PIGL	17	16217191	16351797
57508	INTS2	17	61865367	61928016
9969	MED13	17	61942605	62065278
10743	RAI1	17	17681458	17811453
1819	DRG2	17	18087892	18107970
6603	SMARCD2	17	63832081	63843065
1655	DDX5	17	64498254	64508199
3959	LGALS3BP	17	78971238	78979947
6347	CCL2	17	34255218	34257203
79132	DHX58	17	42101404	42112714
2648	KAT2A	17	42113111	42121367
5878	RAB5C	17	42124978	42155044
6945	MLX	17	42567068	42573239
2145	EZH1	17	42700275	42745049
51264	MRPL27	17	50367857	50373207
10493	VAT1	17	43014607	43025123
55379	LRRC59	17	50375059	50397523
239	ALOX12	17	6996049	7010754
9120	SLC16A6	17	68267026	68291267
23531	MMD	17	55392622	55421924
5608	MAP2K6	17	69414697	69553865
25979	DHRS7B	17	21123364	21193265
26118	WSB1	17	27294076	27315926
90410	IFT20	17	28328325	28335489
230	ALDOC	17	28573115	28576948
57606	SLAIN2	4	48341529	48426201
26511	CHIC2	4	54009789	54064605
8649	LAMTOR3	4	99878336	99894428
374	AREG	4	74445136	74455005
7323	UBE2D3	4	102794383	102868896
1998	ELF2	4	139028112	139177218

4717	NDUFC1	4	139266880	139302551
23158	TBC1D9	4	140620782	140756385
8821	INPP4B	4	142023160	142847432
2549	GAB1	4	143336762	143474568
2483	FRG1	4	189940870	189963202
51809	GALNT7	4	173168811	173323967
23321	TRIM2	4	153152163	153339319
55247	NEIL3	4	177309874	177362936
55296	TBC1D19	4	26576437	26756223
57620	STIM2	4	26857601	27025381
10227	MFSD10	4	2930561	2934834
683	BST1	4	15703065	15738313
83891	SNX25	4	185204237	185370185
55805	LRP2BP	4	185363879	185395899
51088	KLHL5	4	39045039	39126857
64151	NCAPG	4	17810979	17844865
10553	HTATIP2	11	20363685	20383783
7704	ZBTB16	11	114059711	114256765
26610	ELP4	11	31509755	31790324
23788	MTCH2	11	47617315	47642607
23360	FNBP4	11	47716494	47767443
79864	JHY	11	122882683	122963862
3338	DNAJC4	11	64230278	64234286
23130	ATG2A	11	64894546	64917211
10938	EHD1	11	64851642	64888296
5007	OSBP	11	59574398	59615774
81622	UNC93B1	11	67991100	68004982
55572	FOXRED1	11	126269055	126278131
51338	MS4A4A	11	60185702	60317944
1374	CPT1A	11	68754620	68844410
79080	CCDC86	11	60842113	60851081
27339	PRPF19	11	60890547	60906585
2352	FOLR3	11	72114869	72139892
57562	CEP126	11	101915010	102001062
26273	FBXO3	11	33740939	33774543
8050	PDHX	11	34915829	35020591
921	CD5	11	61102489	61127852
55626	AMBRA1	11	46396414	46594125
833	CARS1	11	3000922	3057613
5002	SLC22A18	11	2899721	2925246
29125	C11orf21	11	2295628	2303049
55553	SOX6	11	15966449	16739591
4728	NDUFS8	11	68030617	68036644

10312	TCIRG1	11	68039025	68050895
2965	GTF2H1	11	18322295	18367045
5715	PSMD9	12	121888732	121918297
8496	PPFIBP1	12	27523431	27695564
969	CD69	12	9752486	9760901
11108	PRDM4	12	107732871	107761272
9976	CLEC2B	12	9852984	9870136
84274	COQ5	12	120503279	120534434
6404	SELPLG	12	108621895	108633894
51411	BIN2	12	51281038	51324668
51561	IL23A	12	56334174	56340410
8825	LIN7A	12	80792520	80937925
23371	TNS2	12	53046969	53064379
2735	GLI1	12	57459785	57472268
81029	WNT5B	12	1529891	1647212
55846	ITFG2	12	2812622	2859791
2305	FOXM1	12	2857680	2877174
57097	PARP11	12	3791047	3873448
10635	RAD51AP1	12	4538798	4560048
54682	MANSC1	12	12326056	12350242
80824	DUSP16	12	12473282	12562863
55507	GPRC5D	12	12940775	12952147
80018	NAA25	12	112026689	112108796
4940	OAS3	12	112938444	112973251
4939	OAS2	12	112978395	113011723
420	ART4	12	14825569	14843526
81539	SLC38A1	12	46183063	46270017
8909	ENDOU	12	47709734	47725567
79794	C12orf49	12	116710171	116738070
283383	ADGRD1	12	130953907	131141469
55832	CAND1	12	67269358	67319953
51258	MRPL51	12	6491886	6493841
2597	GAPDH	12	6534512	6538374
84519	ACRBP	12	6638075	6647433
51147	ING4	12	6650301	6663142
83461	CDCA3	12	6844793	6852066
56994	CHPT1	12	101696947	101744140
3945	LDHB	12	21635342	21757857
50856	CLEC4A	12	8123632	8138607
1911	PHC1	12	8913896	8941467
1337	COX6A1	12	120438090	120440737
55188	RIC8B	12	106774621	106889316
8683	SRSF9	12	120461672	120469748

26127	FGFR1OP2	12	26938470	26966650
10384	BTN3A3	6	26440472	26453415
2651	GCNT2	6	10492223	10629368
4739	NEDD9	6	11183298	11382348
84830	ADTRP	6	11712054	11807046
79632	FAM184A	6	118959763	119149387
4121	MAN1A1	6	119177205	119349761
57515	SERINC1	6	122443351	122471807
135114	HINT3	6	125956770	125980244
135112	NCOA7	6	125781161	125932034
10090	UST	6	148747030	149076990
26271	FBXO5	6	152970519	152983579
116369	SLC26A8	6	35943516	36024868
1432	MAPK14	6	36027677	36111236
6005	RHAG	6	49605175	49636839
221692	PHACTR1	6	12716554	13287843
22858	CILK1	6	53001279	53061824
26268	FBXO9	6	53051991	53100873
23195	MDN1	6	89642498	89819794
60468	BACH2	6	89926528	90296908
222642	TSPO2	6	41042467	41044337
202559	KHDRBS2	6	61679961	62286225
1871	E2F3	6	20401879	20493714
7803	PTP4A1	6	63521746	63583436
8936	WASF1	6	110099819	110180004
202	CRYBG1	6	106360717	106572017
8876	VNN1	6	132680849	132714055
8875	VNN2	6	132743870	132763459
6206	RPS12	6	132814569	132817564
81688	C6orf62	6	24704861	24719998
51053	GMNN	6	24774931	24786099
10767	HBS1L	6	134960378	135103056
10475	TRIM38	6	25962802	25991231
9841	ZBTB24	6	109462594	109483219
117247	SLC16A10	6	111087503	111231194
51696	HECA	6	139135080	139180802
57211	ADGRG6	6	142301854	142446266
9749	PHACTR2	6	143536845	143831185
7922	SLC39A7	6	33200445	33204439
1235	CCR6	6	167111807	167139696
5252	PHF1	6	33410399	33416453
9444	QKI	6	163414000	163578592
896	CCND3	6	41934934	42050357

705	BYSL	6	41921499	41933046
6908	TBP	6	170554302	170572870
23506	BICRAL	6	42746958	42868560
51069	MRPL2	6	43054029	43059438
1347	COX7A2	6	75237675	75250323
7422	VEGFA	6	43770184	43786487
8899	PRPF4B	6	4021267	4064983
7272	TTK	6	80003887	80042527
11120	BTN2A1	6	26457904	26476621
55603	TENT5A	6	81491439	81752774
53405	CLIC5	6	45880827	46080348
9037	SEMA5A	5	9035033	9546075
11044	TENT4A	5	6713432	6757044
1611	DAP	5	10679230	10761234
10112	KIF20A	5	138178719	138187723
23530	NNT	5	43602692	43707405
23107	MRPS27	5	72219403	72320646
1839	HBEGF	5	140332843	140346603
10307	APBB3	5	140558268	140564781
3550	IK	5	140647058	140662480
57396	CLK4	5	178602664	178630615
3702	ITK	5	157142933	157255185
411	ARSB	5	78777209	78986087
7060	THBS4	5	79991311	80083287
79908	BTNL8	5	180899077	180950906
7265	TTC1	5	160009113	160065543
5924	RASGRF2	5	80960363	81230162
29102	DROSHA	5	31400497	31532196
4001	LMNB1	5	126776623	126837020
57561	ARRDC3	5	91368631	91383317
64083	GOLPH3	5	32124716	32174319
6897	TARS1	5	33440696	33468091
5810	RAD1	5	34905260	34918989
5618	PRLR	5	35048756	35230487
10111	RAD50	5	132556019	132646349
5515	PPP2CA	5	134194332	134226073
56951	C5orf15	5	133955510	133968674
373	TRIM23	5	65589690	65625975
10802	SEC24A	5	134648785	134727909
23548	TTC33	5	40512333	40755963
9555	MACROH2A1	5	135333900	135399914
10915	TCERG1	5	146447311	146511961
4090	SMAD5	5	136132845	136188747

1452	CSNK1A1	5	149492982	149551471
5159	PDGFRB	5	150113839	150155872
8992	ATP6V0E1	5	172983771	173035445
80315	CPEB4	5	173888349	173961980
1627	DBN1	5	177456608	177474401
23567	ZNF346	5	177022696	177081189
90249	UNC5A	5	176810519	176880898
10051	SMC4	3	160399274	160434954
58515	SELENOK	3	53884417	53891962
51300	TIMMDC1	3	119498547	119525090
27031	NPHP3	3	132680609	132722414
9616	RNF7	3	141738249	141747560
54464	XRN1	3	142306607	142448062
11235	PDCD10	3	167683298	167734939
5210	PFKFB4	3	48517684	48562015
1294	COL7A1	3	48564073	48595267
3280	HES1	3	194136148	194138732
7375	USP4	3	49277144	49340712
1894	ECT2	3	172750682	172821474
10342	TFG	3	100709295	100748964
11068	CYB561D2	3	50350845	50358460
59345	GNB4	3	179396088	179451476
84223	IQCG	3	197889077	197960142
7372	UMPS	3	124730433	124749273
8723	SNX4	3	125446650	125520202
5361	PLXNA1	3	126988594	127037392
80325	ABTB1	3	127672935	127680926
79858	NEK11	3	131026850	131350465
57088	PLSCR4	3	146192335	146251179
51122	COMMD2	3	149738472	149752495
64689	GORASP1	3	39096659	39108363
10289	EIF1B	3	40309707	40312424
7433	VIPR1	3	42489299	42537573
6747	SSR3	3	156539553	156555149
4820	NKTR	3	42600655	42648735
27086	FOXP1	3	70952817	71583989
28972	SPCS1	3	52704955	52711148
8745	ADAM23	2	206443532	206621127
55233	MOB1A	2	74152528	74178898
55683	KANSL3	2	96593170	96642787
55240	STEAP3	2	119223831	119265652
57669	EPB41L5	2	120013077	120179119
29842	TFCP2L1	2	121216587	121285202

51639	SF3B6	2	24067586	24076373
9540	TP53I3	2	24077433	24085861
51277	DNAJC27	2	24943636	24972094
5443	POMC	2	25160853	25168903
10254	STAM2	2	152116801	152175763
9381	OTOF	2	26457203	26558756
2820	GPD2	2	156435290	156613735
1058	CENPA	2	26764289	26801067
9595	CYTIP	2	157414619	157488961
10213	PSMD14	2	161308425	161411717
64135	IFIH1	2	162267074	162318684
550	AUP1	2	74526645	74529760
27429	HTRA2	2	74529725	74533332
84695	LOXL3	2	74532258	74555690
1796	DOK1	2	74549026	74557554
2591	GALNT3	2	165747588	165794659
9801	MRPL19	2	75646783	75690851
84128	WDR75	2	189441446	189475552
55120	FANCL	2	58159243	58241372
6772	STAT1	2	190908460	191020960
2744	GLS	2	190880821	190965552
1768	DNAH6	2	84516455	84819589
84173	ELMOD3	2	85354394	85391752
3769	KCNJ13	2	232765802	232776565
10190	TXNDC9	2	99318982	99340702
80219	COQ10B	2	197453423	197475308
25843	MOB4	2	197515571	197553699
55818	KDM3A	2	86440647	86492716
51652	CHMP3	2	86503430	86563479
7701	ZNF142	2	218637916	218659655
7850	IL1R2	2	101991960	102028544
8809	IL18R1	2	102311529	102398775
8807	IL18RAP	2	102418689	102452565
27013	CNPPD1	2	219171897	219178106
80146	UXS1	2	106093308	106194301
10058	ABCB6	2	219209772	219218994
8576	STK16	2	219245455	219250337
3069	HDLBP	2	241227264	241317061
3241	HPCAL1	2	10302889	10427617
4953	ODC1	2	10439968	10448327
57448	BIRC6	2	32357028	32618899
26003	GORASP2	2	170928464	170967130
23683	PRKD3	2	37250502	37324833

25797	QPCT	2	37342827	37373322
1615	DARS1	2	135905881	135986100
6432	SRSF7	2	38743599	38751494
6382	SDC1	2	20200797	20225433
9167	COX7A2L	2	42333546	42425088
1386	ATF2	2	175072250	175168382
63892	THADA	2	43230836	43596046
22848	AAK1	2	69457997	69674349
54980	C2orf42	2	70149880	70248615
51449	PCYOX1	2	70257386	70281185
2034	EPAS1	2	46293667	46386697
1820	ARID3A	19	925781	975939
7341	SUMO1	2	202206182	202238599
4780	NFE2L2	2	177227595	177392697
2956	MSH6	2	47695530	47810063
2043	EPHA4	2	221418027	221574202
7840	ALMS1	2	73385758	73625166
9068	ANGPTL1	1	178849535	178871077
9731	CEP104	1	3812086	3857396
9528	TMEM59	1	54026681	54053504
115353	LRRC42	1	53946085	53968168
6146	RPL22	1	6185020	6209389
5768	QSOX1	1	180154869	180204030
6814	STXBP3	1	108746674	108809523
148479	PHF13	1	6613731	6624030
271	AMPD2	1	109616104	109632053
3749	KCNC4	1	110210314	110283100
80267	EDEM3	1	184690237	184754907
79084	WDR77	1	111439890	111449256
829	CAPZA1	1	112619805	112671616
127544	RNF19B	1	32936445	32964685
6421	SFPQ	1	35176378	35193145
28956	LAMTOR2	1	156054782	156058506
85440	DOCK7	1	62454298	62688386
26270	FBXO6	1	11664200	11674354
54823	SWT1	1	185157080	185291781
10459	MAD2L2	1	11674480	11691650
9829	DNAJC6	1	65248219	65415871
90231	KIAA2013	1	11919591	11926428
9927	MFN2	1	11980181	12015211
60672	MIIP	1	12019466	12032045
23169	SLC35D1	1	66999350	67054148
1647	GADD45A	1	67685201	67688334

79971	WLS	1	68098473	68233120
7799	PRDM2	1	13700198	13825079
5997	RGS2	1	192809039	192812275
6738	RO60	1	193059454	193091777
10286	BCAS2	1	114567557	114581629
9295	SRSF11	1	70205682	70253052
23207	PLEKHM2	1	15684320	15734769
10745	PHTF1	1	113696831	113759489
965	CD58	1	116514534	116571039
8458	TTF2	1	117060326	117107453
23046	KIF21B	1	200969390	201023700
54936	ADPRS	1	36088892	36093932
64960	MRPS15	1	36455718	36464384
7257	TSNAX	1	231528541	231566524
64121	RRAGC	1	38838198	38859772
4548	MTR	1	236795292	236921278
4610	MYCL	1	39895426	39902256
57568	SIPA1L2	1	232397965	232630571
127396	ZNF684	1	40531573	40548167
10000	AKT3	1	243488233	243851079
2117	ETV3	1	157121191	157138474
6504	SLAMF1	1	160608106	160647295
6390	SDHB	1	17018722	17054032
6675	UAP1	1	162561506	162599842
59349	KLHL12	1	202891116	202928636
117178	SSX2IP	1	84643707	84690803
2635	GBP3	1	89006679	89022894
2633	GBP1	1	89052319	89065230
8934	RAB29	1	205767986	205775482
1889	ECE1	1	21217247	21345572
3155	HMGCL	1	23801885	23838620
51107	APH1A	1	150265399	150269580
6513	SLC2A1	1	42925353	42958893
991	CDC20	1	43358981	43363203
533	ATP6V0B	1	43974487	43978295
8704	B4GALT2	1	43978943	43991170
79033	ERI3	1	44221070	44355260
10327	AKR1A1	1	45550543	45570049
5052	PRDX1	1	45511036	45523047
8548	BLZF1	1	169367970	169396540
57821	CCDC181	1	169394870	169460669
387338	NSUN4	1	46340177	46365152
50999	TMED5	1	93149742	93180516

1810	DR1	1	93345907	93369493
23215	PRRC2C	1	171485551	171593511
356	FASLG	1	172659103	172666876
27042	UTP25	1	209827972	209857565
11123	RCAN3	1	24502351	24541040
25949	SYF2	1	25222276	25232502
57035	RSRP1	1	25242249	25338213
3925	STMN1	1	25884181	25906991
1063	CENPF	1	214603195	214664571
200010	SLC5A9	1	48222685	48248644
114883	OSBPL9	1	51577179	51798427
51060	TXNDC12	1	52020131	52055191
1509	CTSD	11	1752755	1764573
6794	STK11	19	1177558	1228431
4297	KMT2A	11	118436490	118526832
4317	MMP8	11	102711796	102727050
11133	KPTN	19	47475150	47484265
9928	KIF14	1	200551497	200620751
23271	CAMSAP2	1	200739558	200860704
22868	FASTKD2	2	206765357	206796189
55259	CASC1	12	25108289	25195162
57558	USP35	11	78188812	78215232
90025	UBE3D	6	82892390	83065841
84251	SGIP1	1	66533383	66751139
79747	ADGB	6	146598967	146815462
84085	FBXO30	6	145793502	145814795
7128	TNFAIP3	6	137867214	137883312
10981	RAB32	6	146543833	146554953
4602	MYB	6	135181308	135219173
383	ARG1	6	131470832	131584332
26234	FBXL5	4	15604381	15681679
80306	MED28	4	17614641	17634105
9194	SLC16A7	12	59596029	59789855
64858	DCLRE1B	1	113905213	113914086
103910	MYL12B	18	3261479	3278461
2309	FOXO3	6	108559835	108684774
84071	ARMC2	6	108848416	108974476
6185	RPN2	20	37178410	37241619
5311	PKD2	4	88007635	88077777
9429	ABCG2	4	88090150	88231628
8987	STBD1	4	76306026	76311599
5918	RARRES1	3	158696892	158732489
64747	MFSD1	3	158732198	158829719

5493	PPL	16	4882507	4960741
29855	UBN1	16	4846665	4882401
11278	KLF12	13	73686089	73995056
64342	HS1BP3	2	20560448	20651130
NA	OTUD4P1	12	3839459	3842525
22936	ELL2	5	95885098	95961851
56171	DNAH7	2	195737703	196068837
4709	NDUFB3	2	201071433	201085750
7320	UBE2B	5	134371469	134392108
687	KLF9	9	70384597	70414624
80212	CCDC92	12	123918660	123972831
55127	HEATR1	1	236549005	236604516
89891	WDR34	9	128633661	128656787
5524	PTPA	9	129110950	129148946
2733	GLE1	9	128504719	128542288
22954	TRIM32	9	116687302	116701300
92400	RBM18	9	122237622	122264840
27130	INVS	9	100099243	100302175
79695	GALNT12	9	98807670	98850081
57706	DENND1A	9	123379654	123930152
1441	CSF3R	1	36466043	36483278
148223	C19orf25	19	1461143	1479219
56252	YLPM1	14	74763316	74859435
283571	PROX2	14	74852871	74876154
83982	IFI27L2	14	94127779	94130253
80127	BBOF1	14	74019349	74082863
112752	IFT43	14	75902136	76084585
10577	NPC2	14	74476192	74494177
4053	LTBP2	14	74498183	74612378
7043	TGFB3	14	75958097	75983011
4329	ALDH6A1	14	74056847	74084492
51004	COQ6	14	73949926	73963670
10936	GPR75	2	53852912	53859967
114818	KLHL29	2	23385179	23708611
2281	FKBP1B	2	24049701	24063681
51646	YPEL5	2	30146941	30160533
84272	YIPF4	2	32277904	32316594
29094	LGALSL	2	64453969	64461381
53335	BCL11A	2	60450520	60554467
26503	SLC17A5	6	73593379	73653992
79627	OGFRL1	6	71288811	71309059
3437	IFIT3	10	89327997	89340971
3433	IFIT2	10	89283694	89309271

57678	GPAM		10	112149865	112215377
84795	PYROXD2		10	98383565	98415182
26123	TCTN3		10	95663396	95694143
404636	DENND10		10	119104086	119137984
55717	WDR11		10	120851305	120909524
159686	CFAP58		10	104353833	104455102
10672	GNA13		17	65009289	65056740
1843	DUSP1		5	172768096	172771195
10171	RCL1		9	4792944	4885917
11172	INSL6		9	5123880	5185647
2315	MLANA		9	5890889	5910606
29126	CD274		9	5450503	5470566
348995	NUP43		6	149724315	149749665
80129	CCDC170		6	151494017	151621193
5110	PCMT1		6	149749443	149811420
57480	PLEKHG1		6	150599883	150843665
80231	CXorf21	X		30558809	30577766
84418	CYSTM1		5	140175156	140282052
54853	WDR55		5	140664676	140674124
91687	CENPL		1	173799550	173824720
79684	MSANTD2		11	124766498	124800706
51248	PDZD11	X		70286595	70290514
84930	MASTL		10	27154824	27187953
84898	PLXDC2		10	19816239	20289856
6883	TAF12		1	28589323	28643085
9617	MTRF1		13	41216369	41263577
11193	WBP4		13	41061509	41084006
1997	ELF1		13	40931924	41061440
4093	SMAD9		13	36844831	36920765
2107	ETF1		5	138506095	138543236
64374	SIL1		5	138946724	139293557
51247	PAIP2		5	139341587	139369720
27230	SERP1		3	150541998	150603228
7181	NR2C1		12	95020229	95073628
27340	UTP20		12	101280105	101386618
80298	MTERF2		12	106977277	106987160
8835	SOCS2		12	93569814	93583487
10174	SORBS3		8	22544986	22575788
2185	PTK2B		8	27311482	27459391
29914	UBIAD1		1	11273198	11296049
23435	TARDBP		1	11012344	11025739
51123	ZNF706		8	101177878	101206193
8165	AKAP1		17	57085092	57121346

8161	COIL	17	56938199	56961050
7706	TRIM25	17	56836387	56914080
10237	SLC35B1	17	49700934	49709014
81558	FAM117A	17	49710332	49789180
23397	NCAPH	2	96335766	96377091
117854	TRIM6	11	5596109	5612958
85320	ABCC11	16	48166910	48247568
113	ADCY7	16	50246137	50318135
256051	ZNF549	19	57527325	57557542
65982	ZSCAN18	19	58083838	58118427
10520	ZNF211	19	57630395	57644041
89884	LHX4	1	180230264	180278984
81627	TRMT1L	1	185118101	185157072
1475	CSTA	3	122325248	122341969
8702	B4GALT4	3	119211732	119240946
81930	KIF18A	11	28020619	28108156
9409	PEX16	11	45909663	45918812
847	CAT	11	34438934	34472060
29990	PILRB	7	100352176	100367733
10804	GJB6	13	20221962	20232365
2170	FABP3	1	31365253	31376850
9034	CCRL2	3	46407166	46412997
729230	CCR2	3	46353744	46360940
84265	POLR3GL	1	145964690	145978848
8743	TNFSF10	3	172505508	172523475
7579	ZSCAN20	1	33472645	33496507
55791	LRIF1	1	110947190	110963965
57413	TMIGD3	1	111483348	111563962
23155	CLCC1	1	108927361	108963504
29899	GPSM2	1	108875350	108934545
7852	CXCR4	2	136114349	136118149
92	ACVR2A	2	147844517	147930826
2322	FLT3	13	28003274	28100592
6144	RPL21	13	27251309	27256691
2971	GTF3A	13	27424619	27435823
NA	LINC00544	13	29935771	29950488
130916	MTERF4	2	241072169	241102332
54440	SASH3	X	129779949	129795201
57179	KIAA1191	5	176346062	176361807
51744	CD244	1	160830160	160862887
4063	LY9	1	160796074	160828261
84947	SERAC1	6	158109519	158168280
6125	RPL5	1	92832013	92841924

5737	PTGFR		1	78303884	78540701
80148	SLC66A2		18	79902420	79951657
27241	BBS9		7	33109557	33877180
64224	HERPUD2		7	35632659	35697459
11335	CBX3		7	26201162	26213607
3624	INHBA		7	41667168	41705834
11328	FKBP9		7	32957404	33006930
51251	NT5C3A		7	33014114	33062797
10124	ARL4A		7	12686856	12690958
51622	CCZ1		7	5898725	5926550
152007	GLIPR2		9	36136536	36163913
92014	SLC25A51		9	37879400	37904353
1211	CLTA		9	36190856	36304781
8434	RECK		9	36036913	36124455
48	ACO1		9	32384603	32454769
79269	DCAF10		9	37800554	37867666
78996	CYREN		7	135092363	135170795
5552	SRGN		10	69088103	69104805
NA	ARL4AP1		10	60684505	60685209
5033	P4HA1		10	73007217	73096974
11130	ZWINT		10	56357227	56361273
11113	CIT		12	119685791	119877320
84329	HVCN1		12	110627841	110704950
1031	CDKN2C		1	50960745	50974634
11059	WWP1		8	86342547	86478420
23597	ACOT9	X		23701055	23766475
10549	PRDX4	X		23664262	23686397
5585	PKN1		19	14433053	14471867
79002	TRIR		19	12730640	12734684
84650	EBPL		13	49660674	49691486
540	ATP7B		13	51930436	52012125
10133	OPTN		10	13099449	13138308
64333	ARHGAP9		12	57472264	57488814
5204	PFDN5		12	53295291	53299450
65244	SPATS2		12	49366584	49527425
29095	ORMDL2		12	55818041	55821879
5153	PDE1B		12	54549601	54579239
4778	NFE2		12	54292111	54301015
23583	SMUG1		12	54121277	54189008
1757	SARDH		9	133663560	133739955
6491	STIL		1	47250139	47314147
55355	HJURP		2	233833416	233854566
1300	COL10A1		6	116118909	116158747

79828	METTL8		2	171317405	171434802
9111	NMI		2	151270470	151289894
7130	TNFAIP6		2	151357592	151380046
206358	SLC36A1		5	151437046	151492379
50486	G0S2		1	209675412	209676390
3759	KCNJ2		17	70168673	70180044
57826	RAP2C	X		132203024	132219480
79934	COQ8B		19	40691530	40718207
5208	PFKFB2		1	207034366	207081024
10608	MXD4		4	2247432	2262109
1164	CKS2		9	89311195	89316703
2181	ACSL3		2	222860942	222944639
79843	FAM124B		2	224378698	224402107
5266	PI3		20	45174902	45176544
90203	SNX21		20	45833799	45843276
79183	TTPAL		20	44475874	44494603
57580	PREX1		20	48624252	48827999
8202	NCOA3		20	47501887	47656877
57727	NCOA5		20	46060991	46089962
9217	VAPB		20	58389229	58451101
84181	CHD6		20	41402083	41618384
10564	ARFGEF2		20	48921711	49036693
57169	ZNFX1		20	49237946	49278426
128611	ZNF831		20	59123381	59259113
57403	RAB22A		20	58309715	58367507
6780	STAU1		20	49113339	49188367
NA	PPP4R1L		20	58228940	58309451
55905	RNF114		20	49936336	49953885
55653	BCAS4		20	50794894	50882676
27296	TP53TG5		20	45372557	45407889
81030	ZBP1		20	57603846	57620576
55577	NAGK		2	71064344	71079808
400961	PAIP2B		2	71182738	71227103
23326	USP22		17	20999596	21043760
1088	CEACAM8		19	42580243	42595055
7180	CRISP2		6	49692358	49713590
55809	TRERF1		6	42224931	42452051
10385	BTN2A2		6	26383096	26394874
23408	SIRT5		6	13574529	13615158
54718	BTN2A3P		6	26421391	26432383
696	BTN1A1		6	26501221	26510422
221443	OARD1		6	41033627	41097787
6204	RPS10		6	34417454	34426069

4337	MOCS1		6	39899578	39934551
8970	H2BC11		6	27125897	27132795
9477	MED20		6	41905354	41921139
26212	OR2B6		6	27957241	27958182
6903	TBCC		6	42744498	42746103
9587	MAD2L1BP		6	43629540	43640941
116138	KLHDC3		6	43014103	43021298
4201	MEA1		6	43011143	43016868
1026	CDKN1A		6	36676460	36687337
51299	NRN1		6	5997999	6007605
51000	SLC35B3		6	8411463	8435611
7913	DEK		6	18223860	18264548
55831	EMC3		3	9962537	10011116
26231	LRRC29		16	67207139	67227048
583	BBS2		16	56466836	56520087
4502	MT2A		16	56608584	56609497
55715	DOK4		16	57471922	57487327
2841	GPR18		13	99254714	99261744
171425	CLYBL		13	99606669	99897134
10257	ABCC4		13	95019835	95301475
78995	HROB		17	44141906	44162476
3659	IRF1		5	132481609	132490777
23157	SEPTIN6	X		119615724	119693370
4694	NDUFA1	X		119871832	119876662
5732	PTGER2		14	52314305	52328598
2868	GRK4		4	2963571	3040760
51081	MRPS7		17	75261674	75266376
30833	NT5C		17	75130225	75131757
9329	GTF3C4		9	132670035	132694953
64794	DDX31		9	132592997	132670401
54776	PPP1R12C		19	55090914	55117637
4987	OPRL1		20	64080082	64100643
79025	FNDC11		20	63547891	63556695
79144	PPDPF		20	63520765	63522206
84269	CHCHD5		2	112584240	112589275
24139	EML2		19	45606994	45645629
80025	PANK2		20	3888839	3929882
9491	PSMF1		20	1113240	1189415
10616	RBCK1		20	407498	432139
6238	RRBP1		20	17613678	17682295
92667	MGME1		20	17969018	17991122
128637	TBC1D20		20	435480	462543
56926	NCLN		19	3185563	3209575

4149	MAX		14	65006174	65102695
22913	RALY		20	33993646	34108308
83658	DYNLRB1		20	34516409	34540958
8894	EIF2S2		20	34088309	34112243
140823	ROMO1		20	35699272	35700984
154796	AMOT	X		112774503	112840815
192669	AGO3		1	35930718	36072500
7389	UROD		1	45010950	45015575
6487	ST3GAL3		1	43705824	43931165
79654	HECTD3		1	45002540	45011324
23263	MCF2L		13	112894378	113099742
79713	IGFLR1		19	35738801	35742453
2867	FFAR2		19	35443907	35451767
1340	COX6B1		19	35648323	35658782
25824	PRDX5		11	64318121	64321811
3661	IRF3		19	49659569	49665875
435	ASL		7	66075800	66094697
8678	BECN1		17	42810134	42833350
22826	DNAJC8		1	28199456	28233029
2537	IFI6		1	27666064	27672198
10245	TIMM17B	X		48893447	48898143
64430	PCNX4		14	60091911	60169133
9787	DLGAP5		14	55148112	55191608
5858	PZP		12	9148840	9208395
55288	RHOT1		17	32142454	32253374
2123	EVI2A		17	31316410	31321749
83543	AIF1L		9	131096476	131123152
8021	NUP214		9	131125586	131234663
5605	MAP2K2		19	4090321	4124122
55906	ZC4H2	X		64915802	65034713
89846	FGD3		9	92947523	93036236
79717	PPCS		1	42456117	42473385
64919	BCL11B		14	99169287	99272197
84239	ATP13A4		3	193398967	193593111
144453	BEST3		12	69643360	69699476
8445	DYRK2		12	67648338	67665406
50831	TAS2R3		7	141764097	141765197
50832	TAS2R4		7	141776674	141781691
84286	TMEM175		4	932387	958656
23065	EMC1		1	19215660	19251527
79939	SLC35E1		19	16549831	16572415
10365	KLF2		19	16324826	16328685
9002	F2RL3		19	16888999	16892606

10975	UQCR11	19	1597169	1605473
63922	CHTF18	16	788046	800737
23499	MACF1	1	39081316	39487177
84190	METTL25	12	82358528	82479239
79066	METTL16	17	2405562	2511891
7277	TUBA4A	2	219249710	219278170
25953	PNKD	2	218270392	218346793
6049	RNF6	13	26132115	26222314
10875	FGL2	7	77193369	77199848
51657	STYXL1	7	75996338	76048004
2770	GNAI1	7	79768028	80226181
8910	SGCE	7	94524204	94656572
64921	CASD1	7	94509219	94557019
163131	ZNF780B	19	40028260	40056231
57622	LRFN1	19	39306566	39320863
7538	ZFP36	19	39406847	39409412
57168	ASPHD2	22	26429260	26445015
23753	SDF2L1	22	21642302	21644299
7533	YWHAH	22	31944522	31957603
468	ATF4	22	39519672	39522683
53947	A4GALT	22	42692121	42721298
8459	TPST2	22	26521996	26596717
4357	MPST	22	37019635	37029822
23780	APOL2	22	36226209	36239954
200315	APOBEC3A	22	38952741	38992778
200316	APOBEC3F	22	39040604	39055972
51234	EMC4	15	34225013	34230156
92521	SPECC1	17	20009344	20319026
9296	ATP6V1F	7	128862856	128865847
222256	CDHR3	7	105876796	106036432
79706	PRKRIP1	7	102363872	102426676
5420	PODXL	7	131500262	131558217
57464	STRIP2	7	129434432	129488399
3663	IRF5	7	128937457	128950038
6183	MRPS12	19	38930548	38933162
2571	GAD1	2	170813213	170861151
64172	OSGEPL1	2	189746660	189763227
8520	HAT1	2	171922448	171983686
8924	HERC2	15	28111040	28322179
6638	SNRPN	15	24823637	24978723
29767	TMOD2	15	51751597	51816363
54629	MINDY2	15	58771192	58861900
90417	KNSTRN	15	40382721	40394246

1854	DUT		15	48331011	48343373
54832	VPS13C		15	61852389	62060473
4016	LOXL1		15	73925989	73952137
5682	PSMA1		11	14504874	14643635
26297	SERGEF		11	17788048	18013047
3746	KCNC1		11	17734774	17856804
79733	E2F8		11	19224063	19241620
84268	RPAIN		17	5419641	5432876
84817	TXNDC17		17	6640985	6644541
482	ATP1B2		17	7646627	7657770
9513	FXR2		17	7591230	7614897
10749	KIF1C		17	4997950	5028401
9526	MPDU1		17	7583529	7592789
23639	LRRC6		8	132570416	132675592
1032	CDKN2D		19	10566460	10569059
11035	RIPK3		14	24336025	24340022
196883	ADCY4		14	24318349	24335093
84932	RAB2B		14	21459020	21476960
58533	SNX6		14	34561093	34630160
55320	MIS18BP1		14	45203190	45253540
6035	RNASE1		14	20801228	20802855
4738	NEDD8		14	24216857	24232367
1603	DAD1		14	22564907	22589224
64097	EPB41L4A		5	112142441	112419313
7905	REEP5		5	112876385	112922289
81533	ITFG1		16	47154387	47464149
79651	RHBDF2		17	76470891	76501790
15	AANAT		17	76453351	76470117
9459	ARHGEF6	X		136665547	136780932
79649	MAP7D3	X		136213220	136256482
9070	ASH2L		8	38105493	38144076
80185	TTI2		8	33473386	33513185
83475	DOHH		19	3490821	3500674
81926	ABHD17A		19	1876810	1885496
2593	GAMT		19	1397026	1401570
55274	PHF10		6	169703902	169725566
9754	STARD8	X		68647666	68725842
6303	SAT1	X		23783173	23786226
23677	SH3BP4		2	234952017	235055714
158747	MOSPD2	X		14873421	14922327
51295	ECSIT		19	11505929	11529172
84337	ELOF1		19	11551147	11559236
26526	TSPAN16		19	11296139	11326996

5819	NECTIN2	19	44846175	44889223
10912	GADD45G	9	89605012	89606555
23039	XPO7	8	21919662	22006585
147965	FAM98C	19	38403135	38409088
9667	SAFB2	19	5586999	5624046
148229	ATP8B3	19	1782075	1812276
83483	PLVAP	19	17351450	17377342
684	BST2	19	17402939	17405630
55695	NSUN5	7	73302516	73308826
79016	DDA1	19	17309518	17323298
51429	SNX9	6	157700387	157945077
55278	QRSL1	6	106629578	106668417
83861	RSPH3	6	158972871	159000202
59285	CACNG6	19	53992288	54012669
113730	KLHDC7B	22	50545899	50551023
9518	GDF15	19	18374731	18389176
3727	JUND	19	18279694	18281622
10439	OLFM1	9	135075422	135121180
10422	UBAC1	9	135932969	135961373
85441	HELZ2	20	63558086	63574239
7140	TNNT3	11	1919703	1938706
26152	ZNF337	20	25673195	25696853
3911	LAMA5	20	62307955	62367312
11047	ADRM1	20	62302093	62308862
83549	UCK1	9	131523801	131531264
9040	UBE2M	19	58555712	58558954
78992	YIPF2	19	10922185	10928681
84971	ATG4D	19	10543895	10553418
1968	EIF2S3	X	24054946	24078810
9535	GMFG	19	39328353	39342372
93974	ATP5IF1	1	28236109	28246906
54797	MED18	1	28329002	28335965
57693	ZNF317	19	9140397	9163424
55337	SHFL	19	10086122	10093252
6535	SLC6A8	X	153687926	153696588
1736	DKC1	X	154762742	154777689
4354	MPP1	X	154778684	154821007
55422	ZNF331	19	53519527	53580269
4037	LRP3	19	33177603	33208864
54897	CASZ1	1	10636604	10796650
22927	HABP4	9	96450169	96491336
11046	SLC35D2	9	96313444	96383711
9590	AKAP12	6	151239967	151358559

23229	ARHGEF9	X		63634967	63809274
529	ATP6V1E1		22	17592136	17628749
126299	ZNF428		19	43607224	43619629
10328	EMC8		16	85771758	85799608
23199	GSE1		16	85169525	85676204
51659	GINS2		16	85676198	85690073
5119	CHMP1A		16	89640816	89657738
6451	SH3BGRL	X		81202102	81298547
1349	COX7B	X		77899440	77907376
4772	NFATC1		18	79395856	79529325
84440	RAB11FIP4		17	31391675	31538211
7187	TRAF3		14	102777449	102911500
84658	ADGRE3		19	14619117	14690027
23473	CAPN7		3	15206152	15252916
23180	RFTN1		3	16313574	16514026
7376	NR1H2		19	50329653	50383388
11127	KIF3A		5	132692628	132737638
4245	MGAT1		5	180784780	180815652
7283	TUBG1		17	42609683	42615238
84313	VPS25		17	42773449	42779599
4695	NDUFA2		5	140638740	140647771
54882	ANKHD1		5	140401814	140539856
7322	UBE2D2		5	139526431	139628434
79652	TMEM204		16	1528688	1555580
4814	NINJ1		9	93121496	93134251
1160	CKMT2		5	81233320	81266398
84240	ZCCHC9		5	81301587	81313297
7153	TOP2A		17	40388525	40417896
10948	STARD3		17	39637090	39664201
9557	CHD1L		1	147242654	147295765
55829	SELENOS		15	101270817	101277500
6627	SNRPA1		15	101281510	101295282
55145	THAP1		8	42836674	42843325
91442	FAAP24		19	32972209	32978229
55860	ACTR10		14	58200080	58235636
2643	GCH1		14	54842008	54902826
3958	LGALS3		14	55124110	55145423
54862	CC2D1A		19	13906201	13930879
6737	TRIM21		11	4384897	4393702
23225	NUP210		3	13316235	13420322
85363	TRIM5		11	5663195	5938619
10346	TRIM22		11	5689697	5737089
23378	RRP8		11	6595072	6603616

23167	EFR3A	8	131904093	132013642
55037	PTCD3	2	86106223	86142157
84674	CARD6	5	40841308	40860175
51763	INPP5K	17	1494577	1516742
6117	RPA1	17	1829702	1900082
23480	SEC61G	7	54752250	54759974
10117	ENAM	4	70628744	70646824
3512	JCHAIN	4	70655541	70681817
23558	WBP2	17	75845699	75856507
3021	H3-3B	17	75776434	75785893
1984	EIF5A	17	7306999	7312463
23135	KDM6B	17	7839904	7854796
54739	XAF1	17	6755447	6775647
84105	PCBD2	5	134905120	135007959
6388	SDF2	17	28648346	28662189
26284	ERAL1	17	28855010	28861061
51388	NIP7	16	69337996	69343106
7014	TERF2	16	69355567	69408571
22903	BTBD3	20	11890723	11926609
5111	PCNA	20	5114953	5126626
54453	RIN2	20	19757606	20002459
5786	PTPRA	20	2864184	3039076
9826	ARHGEF11	1	156934840	157045742
79368	FCRL2	1	157745733	157777132
9633	TESMIN	11	68707440	68751520
55544	RBM38	20	57391396	57409333
51526	OSER1	20	44195939	44210771
5509	PPP1R3D	20	59936663	59940305
84327	ZBED3	5	77072072	77087285
10207	PATJ	1	61742477	62178675
51164	DCTN4	5	150708440	150759095
23281	MTUS2	13	28820348	29505947
9205	ZMYM5	13	19823482	19863649
64328	XPO4	13	20777329	20903048
51371	POMP	13	28659104	28678959
241	ALOX5AP	13	30713478	30764426
NA	HMGB1P5	3	22381819	22382929
23164	MPRIP	17	17042457	17217679
1116	CHI3L1	1	203178931	203186704
5287	PIK3C2B	1	204422628	204494724
1118	CHIT1	1	203212827	203273641
9911	TMCC2	1	205227946	205285632
8900	CCNA1	13	36431520	36442870

23111	SPART		13	36301638	36370180
94240	EPSTI1		13	42886388	42992271
5994	RFXAP		13	36819222	36829104
9365	KL		13	33016423	33066143
79710	MORC4	X		106813871	107000212
54885	TBC1D8B	X		106802673	106876150
55859	BEX1	X		103062651	103064171
84923	FAM104A		17	73207353	73236753
201266	SLC39A11		17	72645949	73092712
2048	EPHB2		1	22710839	22921500
10250	SRRM1		1	24631716	24673281
84106	PRAM1		19	8490056	8502640
1455	CSNK1G2		19	1941172	1981338
5920	PLAAT4		11	63536808	63546462
54979	PLAAT2		11	63552770	63563379
123811	CEP20		16	15865719	15888625
84246	MED10		5	6371874	6378571
23037	PDZD2		5	31639131	32110932
22880	MORC2		22	30925130	30968774
9215	LARGE1		22	33162226	33922841
84700	MYO18B		22	25742144	26031045
113828	FAM83F		22	39994954	40043534
284904	SEC14L4		22	30488902	30505711
55303	GIMAP4		7	150567369	150573953
23608	MKRN1		7	140453033	140479536
79970	ZNF767P		7	149547154	149624752
57180	ACTR3B		7	152759749	152855378
694	BTG1		12	92140278	92145846
79572	ATP13A3		3	194402672	194498364
83857	TMTC1		12	29500840	29784759
10526	IPO8		12	30628988	30695869
51520	LARS1		5	146110566	146182696
3612	IMPA1		8	81656914	81686331
85444	LRRCC1		8	85107215	85146080
759	CA1		8	85327608	85379014
23075	SWAP70		11	9664077	9752993
406	ARNTL		11	13276652	13387266
9645	MICAL2		11	12094008	12359144
79845	RNF122		8	33547754	33567128
80017	DGLUCY		14	91060333	91225632
79820	CATSPERB		14	91580696	91780707
10001	MED6		14	70581257	70600690
8748	ADAM20		14	70522358	70535015

8932	MBD2	18	54151606	54224669
92259	MRPS36	5	69217760	69230158
891	CCNB1	5	69167135	69178245
1022	CDK7	5	69234795	69277430
3656	IRAK2	3	10164919	10243745
8553	BHLHE40	3	4979437	4985323
55207	ARL8B	3	5122249	5180911
10752	CHL1	3	196763	409417
56851	EMC7	15	34084017	34101862
2947	GSTM3	1	109733932	109741038
10451	VAV3	1	107571161	107965180
84722	PSRC1	1	109279556	109283186
26191	PTPN22	1	113813811	113871753
6272	SORT1	1	109309568	109397918
7482	WNT2B	1	112466541	112530165
79022	TMEM106C	12	47963569	47968878
54407	SLC38A2	12	46358188	46372773
10971	YWHAQ	2	9583967	9630997
9475	ROCK2	2	11179759	11348330
91543	RSAD2	2	6865806	6898239
129607	CMPK2	2	6840570	6866635
3939	LDHA	11	18394560	18408425
89796	NAV1	1	201622885	201826969
10440	TIMM17A	1	201955503	201970664
84893	FBH1	10	5890203	5937594
84991	RBM17	10	6089034	6117457
79746	ECHDC3	10	11742366	11764070
3601	IL15RA	10	5943639	5978187
59340	HRH4	18	24460637	24479961
85019	TMEM241	18	23197144	23437961
2012	EMP1	12	13196723	13219941
4607	MYBPC3	11	47331406	47352702
53	ACP2	11	47239302	47248906
51765	STK26	X	132023302	132075943
143689	PIWIL4	11	94543840	94621421
9698	PUM1	1	30931506	31065991
1912	PHC2	1	33323623	33431052
55143	CDCA8	1	37692481	37709719
1573	CYP2J2	1	59893308	59926773
23318	TUT4	1	52408282	52553487
51444	RNF138	18	32091874	32131561
55250	ELP2	18	36129444	36180557
1823	DSC1	18	31129236	31162856

1837	DTNA	18	34493290	34891844
29015	SLC43A3	11	57406954	57427580
26519	TIMM10	11	57528464	57530803
9415	FADS2	11	61792980	61867354
746	TMEM258	11	61768501	61792802
6947	TCN1	11	59852800	59866489
55858	TMEM165	4	55395957	55453397
9575	CLOCK	4	55427903	55546909
2073	ERCC5	13	102845831	102875995
7174	TPP2	13	102596986	102679958
9743	ARHGAP32	11	128965060	129279324
2113	ETS1	11	128458761	128587558
152831	KLB	4	39406930	39451533
51014	TMED7	5	115613210	115632992
9315	NREP	5	111662621	111997464
26578	OSTF1	9	75088514	75147265
55312	RFK	9	76385526	76394517
60560	NAA35	9	85941146	86025462
301	ANXA1	9	73151865	73170393
1514	CTSL	9	87726109	87731469
29968	PSAT1	9	78297125	78330093
84868	HAVCR2	5	157085832	157142869
51347	TAOK3	12	118149801	118372907
23014	FBXO21	12	117141991	117190471
8638	OASL	12	121019111	121039242
8739	HRK	12	116856144	116881441
5025	P2RX4	12	121209861	121234106
10906	TRAFD1	12	112125538	112153604
9988	DMTF1	7	87152409	87196337
79161	TMEM243	7	87196160	87220587
85025	TMEM60	7	77793728	77798434
948	CD36	7	80369575	80679277
22832	CEP162	6	84124241	84227643
10492	SYNCRIP	6	85607785	85643792
4907	NT5E	6	85449584	85495791
55122	AKIRIN2	6	87674860	87702233
79899	PRR5L	11	36296288	36465204
4005	LMO2	11	33858576	33892076
55226	NAT10	11	34105617	34147670
79056	PRRG4	11	32829927	32858120
85406	DNAJC14	12	55820960	55830824
967	CD63	12	55725323	55729707
10220	GDF11	12	55743122	55757264

3679	ITGA7	12	55684568	55716043
84070	FAM186B	12	49582885	49605639
2647	BLOC1S1	12	55716037	55720087
10024	TROAP	12	49323236	49331731
93058	COQ10A	12	56266858	56270966
9700	ESPL1	12	53268299	53293638
91	ACVR1B	12	51951699	51997078
10956	OS9	12	57693955	57721557
8763	CD164	6	109366514	109382467
57224	NHSL1	6	138422043	138692571
85021	REPS1	6	138903493	138988261
8676	STX11	6	144150517	144191939
7006	TEC	4	48135783	48269838
10505	SEMA4F	2	74654228	74683853
26056	RAB11FIP5	2	73073382	73156721
10322	SMYD5	2	73214222	73227221
8291	DYSF	2	71453722	71686768
27345	KCNMB4	12	70366290	70434292
9958	USP15	12	62260338	62417431
1368	CPM	12	68842197	68971570
4193	MDM2	12	68808177	68850686
79786	KLHL36	16	84648511	84667686
10200	MPHOSPH6	16	82147798	82170224
9764	KIAA0513	16	85027751	85094230
55336	FBXL8	16	67159932	67164570
80003	PCNX2	1	232983435	233295725
23456	ABCB10	1	229516582	229558707
84284	NTPCR	1	232950605	232983882
2752	GLUL	1	182378098	182392206
6041	RNASEL	1	182573634	182589256
1660	DHX9	1	182839347	182887982
80896	NPL	1	182789293	182830384
5279	PIGC	1	172370189	172444086
3915	LAMC1	1	183023420	183145592
3431	SP110	2	230165186	230225729
65080	MRPL44	2	223957463	223967714
55619	DOCK10	2	224765090	225042445
9654	TTLL4	2	218710835	218755416
3357	HTR2B	2	231108230	231125042
81618	ITM2C	2	230864639	230879248
5270	SERPINE2	2	223975045	224039318
3300	DNAJB2	2	219279342	219286898
64114	TMBIM1	2	218274197	218292586

1329	COX5B	2	97646062	97648383
84804	MFSD9	2	102714630	102736888
10913	EDAR	2	108894471	108989372
9392	TGFBRAP1	2	105264391	105329735
50649	ARHGEF4	2	130836914	131047263
23479	ISCU	12	108562582	108569368
160428	ALDH1L2	12	105019784	105107643
55576	STAB2	12	103587273	103766719
84101	USP44	12	95516560	95551476
55681	SCYL2	12	100267140	100341715
10970	CKAP4	12	106237881	106304279
55332	DRAM1	12	101877580	102012130
23325	WASHC4	12	105107324	105169130
84102	SLC41A2	12	104802553	104958744
51028	VPS36	13	52412602	52450634
9882	TBC1D4	13	75283503	75482169
23143	LRCH1	13	46553168	46753040
55213	RCBTB1	13	49531946	49585558
51131	PHF11	13	49495610	49528981
4008	LMO7	13	75620434	75859870
9445	ITM2B	13	48232612	48270357
79020	C7orf25	7	42908726	42912305
55501	CHST12	7	2403588	2448484
11014	KDELR2	7	6445953	6484190
55146	ZDHHC4	7	6577434	6589374
83605	CCM2	7	44999475	45076469
91875	TTC5	14	20256558	20305960
53346	TM6SF1	15	83107572	83144854
7716	VEZF1	17	57971547	57988259
55852	TEX2	17	64147227	64263260
80774	LIMD2	17	63695888	63701172
83990	BRIP1	17	61679139	61863528
11143	KAT7	17	49788648	49835026
64108	RTP4	3	187368385	187372076
6434	TRA2B	3	185914558	185938103
11227	GALNT5	2	157257705	157318491
10010	TANK	2	161136908	161236230
640	BLK	8	11486894	11564599
6498	SKIL	3	170357678	170396835
3142	HLX	1	220879431	220885059
150472	CBWD2	2	113437691	113496204
3557	IL1RN	2	113107214	113134016
55339	WDR33	2	127701027	127811187

2995	GYPC	2	126656133	126696667
10006	ABI1	10	26746593	26861087
10730	YME1L1	10	27110111	27155266
64215	DNAJC1	10	21756548	22003769
25934	NIPSNAP3A	9	104747683	104760120
7295	TXN	9	110243810	110256507
27348	TOR1B	9	129803157	129811281
51759	C9orf78	9	129827290	129835863
10592	SMC2	9	104094260	104141419
1861	TOR1A	9	129812942	129824244
9649	RALGPS1	9	126914774	127223166
64855	NIBAN2	9	127505339	127578989
27090	ST6GALNAC4	9	127907886	127917041
7111	TMOD1	9	97501180	97601743
23452	ANGPTL2	9	127087348	127122635
7539	ZFP37	9	113038377	113056759
7099	TLR4	9	117704175	117724735
55014	STX17	9	99906654	99974534
9550	ATP6V1G1	9	114587769	114598879
54881	TEX10	9	100302077	100352942
84253	GARNL3	9	127224265	127393660
8818	DPM2	9	127935099	127937854
158427	TSTD2	9	97600080	97633368
5695	PSMB7	9	124353465	124415444
51531	TRMO	9	97904489	97922516
7507	XPA	9	97674909	97697340
4686	NCBP1	9	97633668	97673748
10541	ANP32B	9	97983341	98015943
5082	PDCL	9	122798389	122828588
81873	ARPC5L	9	124862130	124877733
5168	ENPP2	8	119557086	119673453
4856	CCN3	8	119416446	119424434
64425	POLR1E	9	37485948	37503697
55833	UBAP2	9	33921693	34048949
54840	APTX	9	32886601	33025130
51754	TMEM8B	9	35814451	35865518
55667	DENND4C	9	19230435	19373545
7746	ZSCAN9	6	28224886	28233487
5292	PIM1	6	37170152	37175428
2766	GMPR	6	16238587	16295549
23070	CMTR1	6	37433219	37482827
25844	YIPF3	6	43511832	43516985
55362	TMEM63B	6	44126914	44155519

55227	LRRC1	6	53794497	53924125
670	BPHL	6	3118374	3153578
8737	RIPK1	6	3063991	3115187
347733	TUBB2B	6	3224277	3231730
10211	FLOT1	6	30727709	30742732
7172	TPMT	6	18128311	18155077
255488	RNF144B	6	18387350	18468870
129685	TAF8	6	42050513	42087461
51439	FAM8A1	6	17600302	17611715
132864	CPEB2	4	15002674	15070153
85462	FHDC1	4	152936352	152979696
7097	TLR2	4	153684070	153706260
338699	ANKRD42	11	83193712	83260694
79731	NARS2	11	78435968	78574874
8836	GGH	8	63015079	63038806
81796	SLCO5A1	8	69667046	69834978
55601	DDX60	4	168216294	168318804
7225	TRPC6	11	101451564	101872562
834	CASP1	11	105025443	105035250
5607	MAP2K5	15	67542703	67807117
58472	SQOR	15	45631148	45691281
51103	NDUFAF1	15	41387353	41402519
9493	KIF23	15	69414246	69448427
22801	ITGA11	15	68296532	68432163
57082	KNL1	15	40594020	40664342
55177	RMDN3	15	40735884	40755851
80021	TMEM62	15	43123279	43185144
8412	BCAR3	1	93561741	93847150
2959	GTF2B	1	88852633	88891944
10964	IFI44L	1	78619922	78646145
9411	ARHGAP29	1	94148988	94275068
10561	IFI44	1	78649796	78664078
204962	SLC44A5	1	75202131	75611116
8634	RTCA	1	100266216	100292769
26160	IFT172	2	27444377	27489805
85465	SELENOI	2	26308547	26395891
3032	HADHB	2	26243170	26290465
87178	PNPT1	2	55634061	55693863
5861	RAB1A	2	65070696	65130331
9581	PREPL	2	44316281	44361862
10128	LRPPRC	2	43886224	43995989
1838	DTNB	2	25377198	25673647
79847	MFSD13A	10	102461395	102477045

84171	LOXL4	10	98247690	98268194
3832	KIF11	10	92593130	92655395
1847	DUSP5	10	110497907	110511533
55165	CEP55	10	93496612	93529092
9585	KIF20B	10	89701610	89774939
54536	EXOC6	10	92826831	93059493
51196	PLCE1	10	93993931	94332823
23317	DNAJC13	3	132417502	132539032
22891	ZNF365	10	62374192	62480288
1763	DNA2	10	68414064	68472121
471	ATIC	2	215311956	215349773
580	BARD1	2	214725646	214809683
54529	ASNSD1	2	189661385	189670831
6741	SSB	2	169791933	169812064
4664	NAB1	2	190646746	190692766
57520	HECW2	2	196189099	196593684
3417	IDH1	2	208236229	208266074
9541	CIR1	2	174348022	174395712
6744	ITPRID2	2	181891730	181930738
150864	FAM117B	2	202634969	202769757
10152	ABI2	2	203328280	203447728
30061	SLC40A1	2	189560590	189583758
10063	COX17	3	119654513	119677454
83666	PARP9	3	122527924	122564577
9101	USP8	15	50424380	50514421
84888	SPPL2A	15	50702266	50765709
90525	SHF	15	45167214	45201175
83464	APH1B	15	63276018	63309126
84993	UBL7	15	74445977	74461182
10144	FAM13A	4	88725955	89111398
8916	HERC3	4	88592434	88708541
55008	HERC6	4	88378739	88443097
51191	HERC5	4	88457119	88506163
3184	HNRNPD	4	82352498	82374503
84803	GPAT3	4	83535914	83605875
55212	BBS7	4	121824329	121870487
84162	KIAA1109	4	122152333	122362758
11107	PRDM5	4	120684919	120922870
27163	NAAA	4	75913660	75941013
55589	BMP2K	4	78776342	78916372
55752	SEPTIN11	4	76949703	77040384
950	SCARB2	4	76158737	76234536
246175	CNOT6L	4	77713387	77819615

306	ANXA3	4	78551747	78610451
1062	CENPE	4	103105349	103198445
57117	INTS12	4	105682627	105895986
3033	HADH	4	107989714	108035174
9061	PAPSS1	4	107590276	107720234
23162	MAPK8IP3	16	1706183	1770351
5998	RGS3	9	113444731	113597743
83606	GUCD1	22	24540423	24555935
91445	RNF185	22	31160182	31207019
5149	PDE6H	12	14973042	14981865
121506	ERP27	12	14914039	14938537
23710	GABARAPL1	12	10212458	10223128
55605	KIF21A	12	39293228	39443390
121512	FGD4	12	32399529	32646050
121536	AEBP2	12	19404045	19720801
254013	ETFBKMT	12	31647160	31673114
85437	ZCRB1	12	42312086	42326118
84216	TMEM117	12	43835967	44389762
144165	PRICKLE1	12	42456757	42590355
51279	C1RL	12	7089587	7109238
10219	KLRG1	12	8950044	9010760
55080	TAPBPL	12	6451690	6466517
196500	PIANP	12	6693792	6700800
9169	SCAF11	12	45919131	45992120
22822	PHLDA1	12	76025447	76033932
283431	GAS2L3	12	100573683	100628288
113675	SDSL	12	113422380	113438276
326625	MMAB	12	109553715	109573580
9818	NUP58	13	25301556	25349800
9107	MTMR6	13	25246201	25288009
53831	GPR84	12	54362445	54364487
79035	NABP2	12	56222015	56229854
675	BRCA2	13	32315086	32400268
91012	CERS5	12	50129289	50167533
51380	CSAD	12	53157663	53180909
55716	LMBR1L	12	49096551	49110900
23344	ESYT1	12	56118250	56144674
283337	ZNF740	12	53180704	53195142
144983	HNRNPA1L2	13	52642425	52643796
5925	RB1	13	48303744	48599436
55206	SBNO1	12	123289109	123364847
8562	DENR	12	122752824	122771064
81624	DIAPH3	13	59665583	60163928

64062	RBM26	13	79311824	79406477
10150	MBNL2	13	97221434	97394120
84945	ABHD13	13	108218392	108234243
55647	RAB20	13	110523066	110561722
161253	REM2	14	22883222	22887678
81542	TMX1	14	51240162	51257655
57161	PELI2	14	56117814	56301524
2100	ESR2	14	64084232	64338112
8477	GPR65	14	88005135	88014811
145482	PTGR2	14	73851844	73886827
122953	JDP2	14	75427716	75474111
81614	NIPA2	15	22838644	22869362
4236	MFAP1	15	43804492	43824690
6938	TCF12	15	56918623	57299281
10169	SERF2	15	43777087	43804427
256586	LYSMD2	15	51723011	51751585
11001	SLC27A2	15	50182196	50236385
3067	HDC	15	50241947	50265965
54939	COMMD4	15	75336020	75343224
9051	PSTPIP1	15	76993359	77037475
2108	ETFA	15	76215353	76311472
597	BCL2A1	15	79960892	79971196
10363	HMG20A	15	77420412	77485607
10099	TSPAN3	15	77041404	77083984
80349	WDR61	15	78277835	78299703
10499	NCOA2	8	70109782	70403808
55466	DNAJA4	15	78264086	78282196
80119	PIF1	15	64815632	64825668
9960	USP3	15	63504511	63594640
585	BBS4	15	72686179	72738475
5371	PML	15	73994673	74047827
170691	ADAMTS17	15	99971437	100342005
55215	FANCI	15	89243945	89317261
11057	ABHD2	15	89087459	89202355
90381	TICRR	15	89575469	89631056
55070	DET1	15	88494440	88546681
55898	UNC45A	15	90930180	90954093
5045	FURIN	15	90868588	90883458
23478	SEC11A	15	84669538	84716460
84656	GLYR1	16	4803203	4847288
79068	FTO	16	53701692	54158512
7385	UQCRC2	16	21953288	21983660
85407	NKD1	16	50548396	50649249

54957	TXNL4B	16	72044289	72094431
463	ZFHX3	16	72782885	73891871
84166	NLRC5	16	56989485	57083531
10300	KATNB1	16	57735739	57757244
3801	KIFC3	16	57758217	57863053
123920	CMTM3	16	66603874	66613892
8996	NOL3	16	67170154	67175735
81631	MAP1LC3B	16	87383953	87404779
8720	MBTPS1	16	84053761	84116942
4716	NDUFB10	16	1959538	1961975
8533	COPS3	17	17246616	17281273
84916	UTP4	16	69131291	69231130
57610	RANBP10	16	67723070	67806652
64180	DPEP3	16	67975663	67980549
81577	GFOD2	16	67674531	67719339
10140	TOB1	17	50862223	50867978
8631	SKAP1	17	48133442	48430275
22901	ARSG	17	68259182	68422731
51651	PTRH2	17	59674636	59707626
83608	C18orf21	18	35972625	35979286
2589	GALNT1	18	35581117	35711834
114799	ESCO1	18	21529284	21600884
80000	GREB1L	18	21242242	21525417
4864	NPC1	18	23506184	23586506
409	ARRB2	17	4710596	4721499
112483	SAT2	17	7626234	7627876
23533	PIK3R5	17	8878911	8965712
396	ARHGDIA	17	81867721	81871378
9123	SLC16A3	17	82228397	82261129
79092	CARD14	17	80169992	80209331
1453	CSNK1D	17	82239023	82273731
51529	ANAPC11	17	81890790	81900991
57521	RPTOR	17	80544819	80966371
6398	SECTM1	17	82321024	82334074
54808	DYM	18	49036387	49461347
8792	TNFRSF11A	18	62325287	62391288
54877	ZCCHC2	18	62523025	62587709
5366	PMAIP1	18	59899996	59904305
115024	NT5C3B	17	41825057	41836260
84299	MIEN1	17	39728496	39730532
10907	TXNL4A	18	79970813	80033949
773	CACNA1A	19	13206442	13633025
23476	BRD4	19	15235519	15332545

7409	VAV1	19	6772708	6857366
93343	MVB12A	19	17405722	17433724
6455	SH3GL1	19	4360370	4400547
90324	CCDC97	19	41310172	41324873
641649	TMEM91	19	41350911	41384083
10410	IFITM3	11	319676	327537
80162	PGGHG	11	289126	296107
1292	COL6A2	21	46098112	46132848
9875	URB1	21	32311018	32393012
2014	EMP3	19	48321509	48330553
NA	ERVK3-1	19	58305319	58315663
59283	CACNG8	19	53963040	53990215
126208	ZNF787	19	56087366	56121295
10498	CARM1	19	10871553	10923075
5694	PSMB6	17	4796144	4798502
89790	SIGLEC10	19	51410020	51417803
25888	ZNF473	19	50025714	50053414
6205	RPS11	19	49496365	49499708
57333	RCN3	19	49528003	49546962
6518	SLC2A5	1	9035106	9088478
473	RERE	1	8352397	8848921
127703	C1orf216	1	35713877	35718894
79932	KIAA0319L	1	35433492	35557950
10733	PLK4	4	127880893	127899224
23038	WDTC1	1	27234632	27308636
84224	NBPF3	1	21440128	21485005
3339	HSPG2	1	21822244	21937310
113451	AZIN2	1	33081104	33123492
11004	KIF2C	1	44739818	44767767
5792	PTPRF	1	43525187	43623666
148932	MOB3C	1	46607715	46616891
8543	LMO4	1	87328880	87348923
5686	PSMA5	1	109399042	109426448
128346	C1orf162	1	111473792	111478512
963	CD53	1	110871188	110899922
223	ALDH9A1	1	165662216	165698863
261726	TIPRL	1	168178962	168202109
25874	MPC2	1	167916675	167937072
8804	CREG1	1	167529117	167553805
55827	DCAF6	1	167935783	168075843
10223	GPA33	1	167052836	167166479
54499	TMCO1	1	165724293	165827755
51506	UFC1	1	161152776	161158856

5498	PPOX	1	161166056	161178013
83540	NUF2	1	163266576	163355764
5546	PRCC	1	156750610	156800815
51093	RRNAD1	1	156728442	156736960
93183	PIGM	1	160024953	160031990
3068	HDGF	1	156742109	156766925
6004	RGS16	1	182598623	182604389
26092	TOR1AIP1	1	179882042	179925000
23179	RGL1	1	183636085	183928532
6097	RORC	1	151806071	151831845
7286	TUFT1	1	151540305	151583583
81609	SNX27	1	151612006	151699091
54507	ADAMTSL4	1	150549369	150560937
4170	MCL1	1	150574551	150579738
1513	CTSK	1	150796208	150808260
81611	ANP32E	1	150218417	150236156
8991	SELENBP1	1	151364304	151372707
51514	DTL	1	212035553	212107400
1939	EIF2D	1	206571292	206612465
9015	TAF1A	1	222557902	222589933
56950	SMYD2	1	214281102	214337131
10899	JTB	1	153974269	153977674
6279	S100A8	1	153390032	153391073
91181	NUP210L	1	153992685	154155116
11000	SLC27A3	1	153774354	153780157
10456	HAX1	1	154272589	154275875
25912	C1orf43	1	154206696	154220637
6016	RIT1	1	155897808	155911404
79573	TTC13	1	230906243	230978875
51097	SCCPDH	1	246724409	246768137
6726	SRP9	1	225777813	225790468
4931	NVL	1	224227334	224330189
8560	DEGS1	1	224175756	224193441
375	ARF1	1	228082660	228099212
29097	CNIH4	1	224356858	224379459
3707	ITPKB	1	226631690	226739323
2987	GUK1	1	228139962	228148984
8476	CDC42BPA	1	226989865	227318474
149111	CNIH3	1	224434660	224740554
129642	MBOAT2	2	8852690	9003709
3930	LBR	1	225401502	225428925
127829	ARL8A	1	202133404	202144743
151449	GDF7	2	20667144	20679243

10130	PDIA6	2	10783391	10837977
245973	ATP6V1C2	2	10721100	10785110
92906	HNRNPLL	2	38561969	38603586
130589	GALM	2	38666081	38741237
27436	EML4	2	42169353	42332548
805	CALM2	2	47160083	47176921
494143	CHAC2	2	53767804	53775196
51057	WDPCP	2	63119559	63827843
51542	VPS54	2	63892146	64019428
55252	ASXL2	2	25733753	25878487
4211	MEIS1	2	66433452	66573869
84874	ZNF514	2	95145000	95165413
23020	SNRNP200	2	96274338	96321271
9027	NAT8	2	73640723	73642422
23233	EXOC6B	2	72175984	72826041
94097	SFXN5	2	72942036	73075619
5899	RALB	2	120240064	120294710
80775	TMEM177	2	119679167	119686507
284958	NT5DC4	2	112721486	112742879
129804	FBLN7	2	112138385	112188218
84524	ZC3H8	2	112211529	112255136
3899	AFF3	2	99545419	100192428
5433	POLR2D	2	127843553	127858155
8502	PKP4	2	158456952	158682879
57282	SLC4A10	2	161424332	161985282
80856	LNPK	2	175923882	176002839
83879	CDCA7	2	173354820	173368997
130507	UBR3	2	169827454	170084131
493911	PHOSPHO2	2	169694454	169701708
284992	CCDC150	2	196639554	196763490
151194	METTL21A	2	207580631	207625928
5746	PTH2R	2	208359714	208854503
65065	NBEAL1	2	203014879	203226378
84236	RHBDD1	2	226835581	226999215
51281	ANKMY1	2	240479422	240569209
64708	COPS7B	2	231781671	231809254
129563	DIS3L2	2	231961245	232344350
132001	TAMM41	3	11790442	11846919
9686	VGLL4	3	11556069	11771350
5868	RAB5A	3	19947097	19985175
79137	RETREG2	2	219176225	219185475
29926	GMPPA	2	219498884	219506989
85403	EAF1	3	15427598	15450635

27303	RBMS3	3	28574791	30010391
114884	OSBPL10	3	31657890	32077580
1238	ACKR2	3	42804752	42887974
64651	CSRNP1	3	39141855	39154562
54977	SLC25A38	3	39383370	39397351
3680	ITGA9	3	37452115	37823507
23066	CAND2	3	12796472	12871916
55164	SHQ1	3	72749277	72861914
7110	TMF1	3	69019827	69052339
26018	LRIG1	3	66378797	66501263
90102	PHLDB2	3	111732497	111976517
141	ADPRH	3	119579268	119589945
64422	ATG3	3	112532510	112562046
66000	TMEM108	3	133038391	133397792
116931	MED12L	3	151085697	151437072
93109	TMEM44	3	194587673	194633689
9711	RUBCN	3	197668867	197749727
6988	TCTA	3	49412206	49416475
9730	DCAF1	3	51395867	51500002
7873	MANF	3	51385291	51389397
81608	FIP1L1	4	53377641	53460862
10699	CORIN	4	47593999	47838106
57205	ATP10D	4	47485275	47593486
51316	PLAC8	4	83090048	83137075
57563	KLHL8	4	87160103	87240314
6622	SNCA	4	89700345	89838315
100996939	PYURF	4	88520998	88523776
817	CAMK2D	4	113451032	113761927
92610	TIFA	4	112274537	112285904
166378	SPATA5	4	122923070	123319433
890	CCNA2	4	121816444	121823883
54532	USP53	4	119212587	119295517
80854	SETD7	4	139495941	139606699
6423	SFRP2	4	153780591	153789083
285533	RNF175	4	153710160	153760024
56034	PDGFC	4	156760454	156971799
83853	ROPN1L	5	10441524	10472029
4726	NDUFS6	5	1801407	1816048
54491	OTULINL	5	14581792	14616180
6502	SKP2	5	36151989	36184319
10769	PLK2	5	58453982	58460139
3001	GZMA	5	55102646	55110252
5295	PIK3R1	5	68215756	68301821

10184	LHFPL2	5	78485215	78770021
23635	SSBP2	5	81413021	81751797
167410	LIX1	5	97091867	97142753
55814	BDP1	5	71455651	71567820
64839	FBXL17	5	107859035	108382098
56929	FEM1C	5	115520908	115544775
51397	COMMD10	5	116085016	116412762
9140	ATG12	5	115828200	115841837
81555	YIPF5	5	144158162	144170714
9879	DDX46	5	134758771	134855133
153830	RNF145	5	159157409	159210053
78991	PCYOX1L	5	149358037	149369653
10318	TNIP1	5	151029945	151093577
10146	G3BP1	5	151771045	151812785
91272	BOD1	5	173607145	173616659
3779	KCNMB1	5	170374671	170389634
51256	TBC1D7	6	13266542	13328583
54438	GFOD1	6	13357830	13487662
54901	CDKAL1	6	20534457	21232404
153527	ZMAT2	5	140698680	140706686
26249	KLHL3	5	137617500	137736089
90933	TRIM41	5	181222499	181235808
27242	TNFRSF21	6	47231532	47309905
4594	MMUT	6	49430360	49463253
23500	DAAM2	6	39792298	39904877
51557	LGSN	6	63275951	63319983
222663	SCUBE3	6	35213956	35253079
4794	NFKBIE	6	44258166	44265788
154214	RNF217	6	124962545	125092633
93663	ARHGAP18	6	129576132	129710177
257218	SHPRH	6	145864245	145964423
6993	DYNLT1	6	158636474	158644743
79624	ARMT1	6	151452258	151470101
2768	GNA12	7	2728105	2844308
84310	C7orf50	7	996986	1138260
NA	WASH2P	2	113588550	113599043
113130	CDCA5	11	65066300	65084164
5814	PURB	7	44876299	44885530
64418	TMEM168	7	112762377	112790423
55262	MAP11	7	100154420	100158723
55281	TMEM140	7	135148072	135166215
11011	TLK2	17	62458658	62615481
64434	NOM1	7	156949712	156973176

54892	NCAPG2		7	158631169	158704804
357	SHROOM2	X		9786429	9949443
401409	RAB19		7	140404058	140427974
51631	LUC7L2		7	139340359	139423457
347404	LANCL3	X		37571569	37684463
8573	CASK	X		41514934	41923517
55869	HDAC8	X		72329516	72573101
55634	KRBOX4	X		46447292	46497422
54539	NDUFB11	X		47142216	47145038
7592	ZNF41	X		47445879	47482946
6872	TAF1	X		71366264	71532374
4841	NONO	X		71283192	71301168
10682	EBP	X		48521799	48528716
8473	OGT	X		71533104	71575892
29934	SNX12	X		71056332	71073426
3561	IL2RG	X		71107404	71112108
55086	RADX	X		106611930	106679439
27316	RBMX	X		136848004	136880764
79648	MCPH1		8	6406596	6648508
26260	FBXO25		8	406428	477967
1069	CETN2	X		152826994	152830757
6134	RPL10	X		154389955	154409168
79618	HMBOX1		8	28890395	29064764
55909	BIN3		8	22620418	22669148
51312	SLC25A37		8	23528956	23575463
91782	CHMP7		8	23243637	23262000
6770	STAR		8	38142700	38150992
11212	PLPBP		8	37762595	37779768
92140	MTDH		8	97644184	97730260
5440	POLR2K		8	100150623	100154003
83940	TATDN1		8	124488485	124539458
1030	CDKN2B		9	22002903	22009305
1029	CDKN2A		9	21967753	21995301
84641	MFSD14B		9	94374569	94461042
58499	ZNF462		9	106863166	107013634
7357	UGCG		9	111896814	111935369
401548	SNX30		9	112750760	112881671
2040	STOM		9	121338987	121370304
92399	MRRF		9	122264603	122331337
210	ALAD		9	113386312	113401290
23245	ASTN2		9	116425225	117415070
26301	GBGT1		9	133152948	133163933
56904	SH3GLB2		9	129007036	129028331

3934	LCN2	9	128149071	128153453
90678	LRSAM1	9	127451486	127503501
23412	COMMD3	10	22316386	22320306
22921	MSRB2	10	23095579	23122013
6251	RSU1	10	16590611	16817463
56288	PARD3	10	34109560	34815325
118924	FRA10AC1	10	93667883	93702592
4288	MKI67	10	128096659	128126423
9446	GSTO1	10	104235356	104267459
85450	ITPRIP	10	104309698	104338465
8038	ADAM12	10	126012381	126388455
27315	PGAP2	11	3797724	3826371
7762	ZNF215	11	6926404	7001004
8525	DGKZ	11	46332905	46380554
710	SERPING1	11	57597387	57614848
8501	SLC43A1	11	57484534	57515780
79096	C11orf49	11	46936689	47164385
10658	CELF1	11	47465933	47565569
26011	TENM4	11	78652829	79441030
871	SERPINH1	11	75562056	75572783
5058	PAK1	11	77322017	77474635
85463	ZC3H12C	11	110093392	110171841
54970	TTC12	11	113314579	113383544
472	ATM	11	108222832	108369102
55004	LAMTOR1	11	72085895	72103297
57325	KAT14	20	18138118	18188387
51524	TMEM138	11	61362344	61369509
932	MS4A3	11	60056587	60071115
79869	CPSF7	11	61402641	61430031
2206	MS4A2	11	60088261	60098467
80071	CCDC15	11	124954121	125041489
1111	CHEK1	11	125625665	125676255
84623	KIRREL3	11	126423358	127003460
10205	MPZL2	11	118253416	118264536
79980	DSN1	20	36751791	36773818
54915	YTHDF1	20	63195429	63216139
220064	LTO1	11	69653076	69675416
84304	NUDT22	11	64225941	64230686
2197	FAU	11	65120630	65122177
226	ALDOA	16	30064164	30070457
124446	TMEM219	16	29940885	29973050
22866	CNKSR2	X	21372801	21654695
2209	FCGR1A	1	149782671	149792518

55208	DCUN1D2	13	113455819	113490951
219854	TMEM218	11	125096545	125111763
26524	LATS2	13	20973036	21061586
10284	SAP18	13	21140514	21149084
27250	PDCD4	10	110871795	110900006
116966	WDR17	4	176065834	176182818
64407	RGS18	1	192158462	192185815
54545	MTMR12	5	32226994	32312987
134145	ATPSCKMT	5	10225507	10249897
26521	TIMM8B	11	112084800	112086798
3606	IL18	11	112143253	112164096
5305	PIP4K2A	10	22534854	22714578
2308	FOXO1	13	40555667	40666641
51232	CRIM1	2	36355778	36551135
196383	RILPL2	12	123410683	123436717
7316	UBC	12	124911604	124917368
3708	ITPR1	3	4493345	4847840
23657	SLC7A11	4	138164097	138242349
25819	NOCT	4	139015781	139045939
55293	UEVLD	11	18529609	18588747
144110	TMEM86A	11	18693122	18704785
121053	C12orf45	12	104986316	105074197
89910	UBE3B	12	109477402	109536705
144715	RAD9B	12	110501655	110533556
196463	PLBD2	12	113358566	113391629
5756	TWF1	12	43793723	43806328
22982	DIP2C	10	274190	689668
1456	CSNK1G3	5	123512099	123617045
9472	AKAP6	14	32329298	32837684
283635	FAM177A1	14	35044907	35113130
2132	EXT2	11	44095673	44251962
129787	TMEM18	2	663877	677406
80224	NUBPL	14	31489956	31861224
63894	VIPAS39	14	77426675	77457952
132321	C4orf33	4	129093317	129116640
55691	FRMD4A	10	13643706	14462142
23310	NCAPD3	11	134150113	134225454
57700	FAM160B1	10	114821744	114899832
79691	QTRT2	3	114005833	114088422
152485	ZNF827	4	145757627	145938823
4306	NR3C2	4	148078762	148444698
22944	KIN	10	7750962	7787993
150709	ANKAR	2	189674290	189761193

3628	INPP1	2	190343570	190371665
80014	WWC2	4	183099257	183320777
79682	CENPU	4	184694085	184734130
2180	ACSL1	4	184755595	184826818
196394	AMN1	12	31671142	31729121
636	BICD1	12	32106835	32383633
60558	GUF1	4	44678420	44700928
79668	PARP8	5	50665899	50846519
9531	BAG3	10	119651380	119677819
148534	TLCD4	1	95117355	95197607
130074	FAM168B	2	131047876	131093460
5784	PTPN14	1	214348700	214552449
4249	MGAT5	2	134119983	134454621
81615	TMEM163	2	134455759	134719000
253635	GPATCH11	2	37084451	37099244
57105	CYSLTR2	13	48653711	48711226
115761	ARL11	13	49628507	49633872
9050	PSTPIP2	18	45983536	46072272
115106	HAUS1	18	46104378	46128333
147339	C18orf25	18	46173553	46266992
5163	PDK1	2	172555373	172608669
5140	PDE3B	11	14643804	14872044
167555	FAM151B	5	80487969	80542563
7518	XRCC4	5	83077498	83353787
284306	ZNF547	19	57363477	57379565
374928	ZNF773	19	57499915	57518404
10172	ZNF256	19	57940833	57947706
64421	DCLRE1C	10	14897359	14954432
55301	OLAH	10	15032227	15073853
9397	NMT2	10	15102584	15168693
152137	CCDC50	3	191329085	191398659
814	CAMK4	5	111223653	111494886
114908	TMEM123	11	102396332	102470384
79925	SPEF2	5	35617844	35814611
4154	MBNL1	3	152243828	152465780
53918	PELO	5	52787916	52804044
200132	TCTEX1D1	1	66752459	66779047
118932	ANKRD22	10	88819896	88851844
10160	FARP1	13	98142562	98455176
24138	IFIT5	10	89414568	89420997
56978	PRDM8	4	80183879	80204329
9987	HNRNPDL	4	82422564	82430408
5796	PTPRK	6	127968779	128520674

51351	ZNF117	7	64971772	65006684
25859	PART1	5	60487713	60548813
1360	CPB1	3	148791102	148860187
10283	CWC27	5	64768930	65018750
3140	MR1	1	181033425	181061938
6728	SRP19	5	112861188	112898371
9425	CDYL	6	4706159	4955551
23589	CARHSP1	16	8852942	8869012
55024	BANK1	4	101411286	102074812
51061	TXNDC11	16	11679080	11742878
55289	ACOXL	2	110732573	111118548
64682	ANAPC1	2	111611639	111884690
60592	SCOC	4	140257286	140385726
3192	HNRNPU	1	244840638	244864560
5903	RANBP2	2	108719482	108785809
10225	CD96	3	111292719	111665750
50807	ASAP1	8	130052104	130443674
NA	LINC00467	1	211382736	211435570
124402	UBALD1	16	4608883	4615027
25827	FBXL2	3	33277025	33403662
7342	UBP1	3	33388336	33441371
22934	RPIA	2	88691673	88750929
2960	GTF2E1	3	120742637	120783069
136895	C7orf31	7	25134692	25180356
55022	PID1	2	228850526	229271287
9320	TRIP12	2	229763837	229923239
79047	KCTD15	19	33795933	33815763
148103	ZNF599	19	34758073	34773229
8526	DGKE	17	56834107	56869567
221302	ZUP1	6	116635618	116668794
26289	AK5	1	77282019	77559966
404550	C16orf74	16	85690084	85751129
219899	TBCEL	11	121024072	121090775
90268	OTULIN	5	14664664	14699850
84959	UBASH3B	11	122655722	122814473
26207	PITPNC1	17	67377281	67697261
5578	PRKCA	17	66302613	66810743
79705	LRRK1	15	100919327	101078257
5169	ENPP3	6	131628442	131747418
375056	MIA3	1	222618097	222668007
84976	DISP1	1	222815022	223005995
81559	TRIM11	1	228393673	228406835
115362	GBP5	1	89258950	89272804

9184	BUB3	10	123154402	123170467
118856	MMP21	10	125753580	125775821
518	ATP5MC3	2	175176258	175184607
135295	SRSF12	6	89095959	89118071
6921	ELOC	8	73939169	73972287
23643	LY96	8	73991392	74029079
10950	BTG3	21	17593653	17612945
54149	C21orf91	21	17788974	17819386
27342	RABGEF1	7	66682164	66811464
522	ATP5PF	21	25716503	25735673
11096	ADAMTS5	21	26917922	26967088
80746	TSEN2	3	12484432	12539623
146857	SLFN13	17	35435096	35448837
7476	WNT7A	3	13816258	13880071
201163	FLCN	17	17212212	17237188
285381	DPH3	3	16257061	16264969
92106	OXNAD1	3	16265160	16350299
23228	PLCL2	3	16802651	17090604
91369	ANKRD40	17	50693198	50707914
7566	ZNF18	17	11977439	11997475
113612	CYP2U1	4	107931549	107953461
5799	PTPRN2	7	157539056	158587823
51582	AZIN1	8	102826308	102893864
528	ATP6V1C1	8	103021063	103073051
55529	PIP4P2	8	90993802	91040872
112495	GTF3C6	6	110958706	110967890
64210	MMS19	10	97458324	97498794
118813	ZFYVE27	10	97737121	97760907
152992	TRMT44	4	8436140	8493531
81894	SLC25A28	10	99610522	99620609
6782	HSPA13	21	14371115	14383484
64092	SAMSN1	21	14485228	14658821
29761	USP25	21	15730025	15880069
54819	ZCCHC10	5	132996985	133026604
4343	MOV10	1	112673141	112700746
1622	DBI	2	119366921	119372560
9337	CNOT8	5	154857553	154876793
118788	PIK3AP1	10	96593315	96720514
11326	VSIG4	X	66021738	66040125
9601	PDIA4	7	149003062	149028662
130535	KCTD18	2	200488958	200519784
130540	FLACC1	2	201288271	201357398
1836	SLC26A2	5	149960758	149993455

10670	RRAGA		9	19049427	19051025
92370	PXYLP1		3	141228726	141367753
55005	RMND1		6	151404762	151452158
6503	SLA		8	133036728	133102912
7411	VBP1	X		155197007	155239841
2334	AFF2	X		148500617	149000663
137492	VPS37A		8	17246931	17302427
23362	PSD3		8	18527303	19084730
91748	MIDEAS		14	73715122	73790285
132	ADK		10	74151202	74709963
3778	KCNMA1		10	76869601	77638369
10538	BATF		14	75522455	75546993
9508	ADAMTS3		4	72280969	72569221
29104	N6AMT1		21	28872191	28885371
571	BACH1		21	29194071	29630751
7074	TIAM1		21	31118416	31559977
122416	ANKRD9		14	102501767	102509799
118980	SFXN2		10	102714538	102743492
9556	ATP5MPL		14	103912288	103928269
122402	TDRD9		14	103928456	104052667
9791	PTDSS1		8	96261902	96336995
159091	FAM122C	X		134796395	134854835
286151	FBXO43		8	100133351	100145817
9246	UBE2L6		11	57551656	57568284
25921	ZDHHC5		11	57667747	57701182
27020	NPTN		15	73560014	73634134
23522	KAT6B		10	74824927	75032624
142891	SAMD8		10	75099586	75182123
5603	MAPK13		6	36127809	36144524
93594	TBC1D31		8	123041968	123152153
29028	ATAD2		8	123319850	123416350
78994	PRR14		16	30650717	30656413
64319	FBRS		16	30658431	30670810
5261	PHKG2		16	30748293	30761176
64645	MFSD14A		1	100038095	100083377
163786	SASS6		1	100083563	100132955
3681	ITGAD		16	31393335	31426505
701	BUB1B		15	40161023	40221123
5147	PDE6D		2	231732433	231786272
6396	SEC13		3	10293131	10321112
9941	EXOG		3	38496127	38542161
9372	ZFYVE9		1	52142094	52346686
115548	FCHO2		5	72956041	73090522

11030	RBPMS		8	30384511	30572256
23648	SSBP3		1	54225432	54413479
6483	ST3GAL2		16	70375977	70439237
197258	FCSK		16	70454595	70480274
7567	ZNF19		16	71464555	71565089
9133	CCNB2		15	59105126	59125045
4643	MYO1E		15	59132434	59372871
26060	APPL1		3	57227726	57278105
1831	TSC22D3	X		107713221	107777342
3772	KCNJ15		21	38155549	38307357
2078	ERG		21	38380027	38661780
150082	LCA5L		21	39405844	39445805
4599	MX1		21	41420304	41459214
257397	TAB3	X		30827442	30975084
124565	SLC38A10		17	81244811	81295547
445815	PALM2AKAP2		9	109498325	110172512
9162	DGKI		7	137381037	137847092
203197	TMEM268		9	114611206	114646422
254048	UBN2		7	139230356	139308236
57728	WDR19		4	39182504	39285810
92749	DRC1		2	26401920	26456711
8764	TNFRSF14		1	2555639	2565382
11079	RER1		1	2391775	2405442
6497	SKI		1	2228319	2310213
26100	WIPI2		7	5190196	5233840
26119	LDLRAP1		1	25543606	25568886
116987	AGAP1		2	235494043	236131800
100310846	ANKRD61		7	6031376	6036552
5051	PAFAH2		1	25959767	25998117
144406	CFAP251		12	121918592	122003927
138639	PTPDC1		9	94030794	94109856
4690	NCK1		3	136862208	136951606
3242	HPD		12	121839527	121863596
26504	CNNM4		2	96760902	96811874
199221	DZIP1L		3	138061990	138115818
2176	FANCC		9	95099054	95426796
22808	MRAS		3	138347648	138405534
10163	WASF2		1	27404230	27490167
171586	ABHD3		18	21650901	21704780
55179	FAIM		3	138608606	138633376
114928	GPRASP2	X		102712176	102717733
26053	AUTS2		7	69598296	70793506
57477	SHROOM4	X		50591647	50814302

3017	H2BC5		6	26158146	26171349
995	CDC25C		5	138285265	138338355
8365	H4C8		6	26277609	26285638
9669	EIF5B		2	99337371	99401326
23382	AHCYL2		7	129225023	129430211
9334	B4GALT5		20	49632945	49713878
911	CD1C		1	158289923	158294774
653361	NCF1		7	74773962	74789376
130617	ZFAND2B		2	219195237	219209651
222068	TMED4		7	44577894	44582287
84919	PPP1R15B		1	204403381	204411817
26958	COPG2		7	130506238	130668748
85414	SLC45A3		1	205657851	205680509
115992	RNF166		16	88696499	88706408
7391	USF1		1	161039251	161045977
124044	SPATA2L		16	89696357	89701705
4817	NIT1		1	161118086	161125445
92822	ZNF276		16	89720400	89740925
1896	EDA	X		69616067	70039472
978	CDA		1	20589086	20618903
65018	PINK1		1	20633458	20651511
2039	DMTN		8	22048995	22082527
4720	NDUFS2		1	161197104	161214395
2207	FCER1G		1	161215234	161220699
84134	TOMM40L		1	161225939	161230746
4359	MPZ		1	161304735	161309968
51735	RAPGEF6		5	131423921	131635231
54461	FBXW5		9	136940435	136944738
56683	CFAP298		21	32592079	32612603
3460	IFNGR2		21	33403413	33479348
6651	SON		21	33543038	33577481
1827	RCAN1		21	34513142	34615113
65264	UBE2Z		17	48908407	48929056
861	RUNX1		21	34787801	36004667
873	CBR1		21	36069941	36073166
NA	TUBBP5		9	138150075	138177433
23515	MORC3		21	36320189	36386148
8208	CHAF1B		21	36385392	36419015
3141	HLCS		21	36748626	36990236
201176	ARHGAP27		17	45393902	45434421
5763	PTMS		12	6765516	6770952
23569	PADI4		1	17308195	17364004
51094	ADIPOR1		1	202940826	202958572

5710	PSMD4	1	151254709	151267479
130951	M1AP	2	74557883	74648338
11189	CELF3	1	151700058	151716803
8659	ALDH4A1	1	18871430	18902724
57519	STARD9	15	42575606	42720998
117145	THEM4	1	151870866	151909637
267	AMFR	16	56361452	56425545
112950	MED8	1	43383917	43389808
266747	RGL4	22	23688136	23699176
149483	CCDC17	1	45620044	45624057
60313	GPBP1L1	1	45627304	45688113
9813	EFCAB14	1	46674659	46719146
10417	SPON2	4	1166932	1208962
84303	CHCHD6	3	126704240	126960420
7791	ZYX	7	143381295	143391111
7773	ZNF230	19	44002957	44013924
7673	ZNF222	19	44025342	44033110
4882	NPR2	9	35791591	35809732
353355	ZNF233	19	44259880	44275317
10020	GNE	9	36214441	36277056
2909	ARHGAP35	19	46860997	47005077
808	CALM3	19	46601074	46610782
1676	DFFA	1	10456522	10472529
79140	CCDC28B	1	32200595	32205387
55108	BSDC1	1	32364633	32394731
118424	UBE2J2	1	1253909	1273864
126549	ANKLE1	19	17281645	17287646
9619	ABCG1	21	42199689	42297244
53347	UBASH3A	21	42403447	42447684
5152	PDE9A	21	42653621	42775509
8566	PDXK	21	43719094	43762307
2539	G6PD	X	154531391	154547572
1476	CSTB	21	43772511	43776330
56894	AGPAT3	21	43865223	43987592
4047	LSS	21	46188141	46228824
54058	C21orf58	21	46300181	46323875
5116	PCNT	21	46324141	46445769
10444	ZER1	9	128729786	128772414
84885	ZDHHC12	9	128720870	128724127
9442	MED27	9	131852928	132079867
162989	DEDD2	19	42198598	42220140
26097	CHTOP	1	153633982	153646306
643	CXCR5	11	118883892	118897787

103	ADAR	1	154582057	154628013
1141	CHRNB2	1	154567778	154580013
200186	CRTC2	1	153947669	153958615
2224	FDPS	1	155308748	155320666
79957	PAQR6	1	156243320	156248117
23218	NBEAL2	3	46979666	47009704
56893	UBQLN4	1	156035299	156053798
115352	FCRL3	1	157674321	157700769
3101	HK3	5	176880869	176899346
9592	IER2	19	13150411	13154911
84124	ZNF394	7	99473877	99504857
4061	LY6E	8	143017982	143023832
51160	VPS28	8	144423601	144428563
54784	ALKBH4	7	102456238	102464863
51149	MRNIP	5	179835133	179862173
8878	SQSTM1	5	179806398	179838078
222229	LRWD1	7	102464956	102473168
5701	PSMC2	7	103344254	103369395
126321	MFSD12	19	3538261	3574290
373856	USP41	22	20350578	20390758
1173	AP2M1	3	184174689	184184091
57125	PLXDC1	17	39063313	39154394
2232	FDXR	17	74862497	74873031
29115	SAP30BP	17	75667251	75708062
84765	ZNF577	19	51804816	51890950
55260	TMEM143	19	48332356	48364059
162417	NAGS	17	44004622	44009068
126119	JOSD2	19	50505998	50511220
80174	DBF4B	17	44708608	44752264
91010	FMNL3	12	49636499	49708165
29127	RACGAP1	12	49976923	50033136
147841	SPC24	19	11131520	11155808
246	ALOX15	17	4630919	4642294
115950	ZNF653	19	11483427	11505839
8741	TNFSF13	17	7558292	7561608
899	CCNF	16	2429394	2458854
51005	AMDHD2	16	2520357	2531422
146439	BICDL2	16	3027682	3036944
124222	PAQR4	16	2969270	2973484
114984	FLYWCH2	16	2883213	2899382
7627	ZNF75A	16	3305406	3318852
10825	NEU3	11	74988279	75018893
220002	CYB561A3	11	61348754	61362283

	6811	STX5	11	62806860	62832051
	4041	LRP5	11	68312591	68449275
	10158	PDZK1IP1	1	47183582	47191044
	6886	TAL1	1	47216290	47232220
	79699	ZYG11B	1	52726453	52827336
	7809	BSND	1	54998933	55017172
	23358	USP24	1	55066359	55215364
	79707	NOL9	1	6521347	6554513
	55225	RAVER2	1	64745095	64833232
	116362	RBP7	1	9997206	10016021
	553115	PEF1	1	31629866	31644896
	127733	UBXN10	1	20186096	20196050
	149233	IL23R	1	67138907	67259979
	7398	USP1	1	62436297	62451804
	91624	NEXN	1	77888513	77943895
	11080	DNAJB4	1	77979175	78017964
	2634	GBP2	1	89106132	89150456
	115361	GBP4	1	89181144	89198942
	164045	HFM1	1	91260766	91404856
	2672	GFI1	1	92473043	92486925
	10092	ARPC5	1	183620846	183635783
	84838	ZNF496	1	247297412	247331846
	2215	FCGR3B	1	161623196	161631963
	148304	C1orf74	1	209779208	209784559
	467	ATF3	1	212565334	212620777
	64783	RBM15	1	110338506	110346681
	25992	SNED1	2	240998618	241095568
	148362	BROX	1	222712553	222735196
	55220	KLHDC8A	1	205336061	205357090
NA		C1orf147	1	206491116	206497728
	9261	MAPKAPK2	1	206684905	206734281
	11009	IL24	1	206897443	206904139
	9214	FCMR	1	206903317	206923247
	824	CAPN2	1	223701593	223776018
	128308	MRPL55	1	228106679	228109312
	84542	KIAA1841	2	61065871	61138034
	27185	DISC1	1	231626790	232041272
	84661	DPY30	2	31867809	32039805
	79568	MAIP1	2	199955317	200008540
	130162	CLHC1	2	55172547	55232563
	112942	CFAP36	2	55519604	55545079
	200420	ALMS1P1	2	73641083	73697509
	56997	COQ8A	1	226897536	226987545

6443	SGCB	4	52020706	52038299
3625	INHBB	2	120346136	120351803
23248	RPRD2	1	150363091	150476566
1520	CTSS	1	150730188	150765792
79005	SCNM1	1	151156664	151170296
6944	VPS72	1	151176304	151195321
10602	CDC42EP3	2	37641882	37738468
6280	S100A9	1	153357854	153361023
6283	S100A12	1	153373711	153375621
151195	CCNYL1	2	207711540	207761839
7855	FZD5	2	207762598	207769906
1293	COL6A3	2	237324003	237414207
55249	YY1AP1	1	155659443	155689000
56983	POGLUT1	3	119468963	119494708
55356	SLC22A15	1	115976513	116070054
476	ATP1A1	1	116372668	116410261
6565	SLC15A2	3	121894401	121944188
92703	TMEM183A	1	203007374	203024848
3490	IGFBP7	4	57030773	57110385
10109	ARPC2	2	218217141	218254356
6746	SSR2	1	156009048	156020951
152110	NEK10	3	27110085	27369460
285025	CCDC141	2	178829757	179050086
64343	AZI2	3	28315003	28349050
2199	FBLN2	3	13549125	13638422
79411	GLB1L	2	219236598	219245478
151246	SGO2	2	200510008	200584096
23122	CLASP2	3	33496245	33718356
6708	SPTA1	1	158610704	158686715
4332	MNDA	1	158831351	158849506
3428	IFI16	1	158999968	159055155
9447	AIM2	1	159062484	159147096
130026	ICA1L	2	202773150	202871985
151987	PPP4R2	3	72996803	73069198
131450	CD200R1	3	112921205	112975103
25871	NEPRO	3	113002444	113019861
152185	SPICE1	3	113442718	113515187
57577	CCDC191	3	113964137	114056594
5783	PTPN13	4	86594315	86815171
132200	C3orf49	3	63819299	63848636
80145	THOC7	3	63833870	63863868
152926	PPM1K	4	88257620	88284769
5806	PTX3	3	157436850	157443633

323	APBB2	4	40810027	41216714
26577	PCOLCE2	3	142815922	142889206
2919	CXCL1	4	73869393	73871308
57047	PLSCR2	3	146391421	146495991
1359	CPA3	3	148865296	148897203
2992	GYG1	3	148991408	149031775
6259	RYK	3	134065303	134250859
54861	SNRK	3	43286512	43424764
22950	SLC4A1AP	2	27663471	27694976
56992	KIF15	3	44761721	44873376
51304	ZDHHC3	3	44915257	44976185
1230	CCR1	3	46201711	46208313
151636	DTX3L	3	122564338	122575203
349565	NMNAT3	3	139560180	139678017
131601	TPRA1	3	127571232	127598267
64769	MEAF6	1	37489993	37514766
59343	SENP2	3	185582496	185633551
8314	BAP1	3	52401008	52410008
91869	RFT1	3	53088483	53130453
51460	SFMBT1	3	52903572	53046750
55193	PBRM1	3	52545352	52685917
26043	UBXN7	3	196347662	196432430
165918	RNF168	3	196468783	196503768
6286	S100P	4	6693878	6697170
84448	ABLIM2	4	7965310	8158832
64789	EXO5	1	40508741	40516556
79078	C1orf50	1	42767249	42779491
114625	ERMAP	1	42817122	42844991
51058	ZNF691	1	42846573	42852477
23173	METAP1	4	98995659	99062809
993	CDC25A	3	48157146	48188417
820	CAMP	3	48223347	48225491
51385	ZNF589	3	48241100	48299253
79714	CCDC51	3	48432164	48440456
84126	ATRIP	3	48446710	48467645
51246	SHISA5	3	48467798	48504826
27152	INTU	4	127623271	127726737
63891	RNF123	3	49689538	49721529
80167	ABHD18	4	127965306	128039927
23132	RAD54L2	3	51541144	51668667
80335	WDR82	3	52254434	52288020
401152	C4orf3	4	119296419	119304445
3148	HMGB2	4	173331376	173334432

8819	SAP30	4	173369969	173377532
11341	SCRG1	4	173384701	173406380
4085	MAD2L1	4	120055623	120066858
152940	C4orf45	4	158893134	159038760
80155	NAA15	4	139301505	139420033
3600	IL15	4	141636583	141733987
729830	FAM160A1	4	151409176	151670503
27236	ARFIP1	4	152779937	152918463
23379	ICE1	5	5420664	5490220
10393	ANAPC10	4	144831908	145098541
55751	TMEM184C	4	147617386	147672044
4338	MOCS2	5	53095679	53110063
10085	EDIL3	5	83940554	84384880
401207	C5orf63	5	127042558	127073492
134266	GRPEL2	5	149345430	149354583
153642	ARSK	5	95555101	95605102
256987	SERINC5	5	80111651	80256048
836	CASP3	4	184627696	184649509
201973	PRIMPOL	4	184649667	184694963
253260	RICTOR	5	38937920	39074399
167153	TENT2	5	79612120	79686648
1879	EBF1	5	158695916	159099916
7098	TLR3	4	186069155	186088073
133957	CCDC127	5	196868	218153
116842	LEAP2	5	132873444	132875046
375519	GJB7	6	87282980	87329278
115004	CGAS	6	73413515	73452297
167838	TXLNB	6	139240061	139291998
10370	CITED2	6	139371807	139374648
153222	CREBRF	5	173056352	173139284
79722	ANKRD55	5	56099680	56233330
10466	COG5	7	107201555	107564514
10569	SLU7	5	160401641	160421711
6100	RP9	7	33094797	33109404
9232	PTTG1	5	160421855	160428739
285613	RELL2	5	141636950	141641064
8645	KCNK5	6	39188971	39229475
94120	SYTL3	6	158650014	158764876
2171	FABP5	8	81280536	81284777
117289	TAGAP	6	159034468	159045152
26266	SLC13A4	7	135681237	135729258
25798	BRI3	7	98252379	98310441
1508	CTSB	8	11842524	11869448

10395	DLC1	8	13083361	13604610
107	ADCY1	7	45574140	45723116
23514	SPIDR	8	47260878	47736306
1669	DEFA4	8	6935820	6938306
734	OSGIN2	8	89901849	89927888
23353	SUN1	7	816615	896435
55074	OXR1	8	106359476	106752694
115330	GPR146	7	1044546	1059261
79778	MICALL2	7	1428465	1459470
761	CA3	8	85373436	85449040
1020	CDK5	7	151053815	151057897
83590	TMUB1	7	151081085	151083493
7534	YWHAZ	8	100916523	100953388
25962	VIRMA	8	94487689	94553529
91147	TMEM67	8	93754844	93819234
57462	MYORG	9	34366666	34376898
51271	UBAP1	9	34179005	34252523
55335	NIPSNAP3B	9	104764129	104777764
19	ABCA1	9	104781006	104928155
4783	NFIL3	9	91409045	91423832
2395	FXN	9	69035751	69079076
137835	TMEM71	8	132685007	132760712
138050	HGSNAT	8	43140464	43202855
55582	KIF27	9	83834099	83921465
84267	C9orf64	9	83938311	83956986
3190	HNRNPK	9	83968083	83980616
203286	ANKS6	9	98731329	98796965
1536	CYBB	X	37780059	37813461
58526	MID1IP1	X	38801432	38806537
654817	NCF1C	7	75156639	75172044
158405	KIAA1958	9	112486827	112669397
55342	STRBP	9	123109500	123268586
84270	CARD19	9	93096217	93113283
195828	ZNF367	9	96385941	96418370
139324	HDX	X	84317874	84502479
30968	STOML2	9	35099776	35103195
9833	MELK	9	36572862	36677683
220213	OTUD1	10	23439458	23442390
220972	MARCHF8	10	45454585	45594906
7253	TSHR	14	80954989	81146302
3636	INPPL1	11	72223701	72239147
221035	REEP3	10	63521401	63625128
221150	SKA3	13	21153595	21176552

221154	MICU2		13	21492691	21604181
122769	LRR1		14	49598761	49614672
6166	RPL36AL		14	49618530	49620626
161436	EML5		14	88612431	88792752
123016	TTC8		14	88824153	88881078
122945	NOXRED1		14	77394021	77423517
89890	KBTBD6		13	41127569	41132802
11164	NUDT5		10	12165330	12196144
509	ATP5F1C		10	7788147	7807815
83860	TAF3		10	7818505	8016631
51266	CLEC1B		12	9985642	10013424
120939	TMEM52B		12	10170542	10191801
8328	GFI1B		9	132944000	132991687
9790	BMS1		10	42782795	42834937
65991	FUNDC2	X		155025980	155060304
57447	NDRG2		14	21016763	21070872
840	CASP7		10	113679162	113730907
55088	CCDC186		10	114120862	114174232
53349	ZFYVE1		14	72969451	73027131
10023	FRAT1		10	97319271	97321915
80019	UBTD1		10	97498924	97571206
144455	E2F7		12	77021248	77065569
143872	ARHGAP42		11	100687288	100993941
120071	LARGE2		11	45921621	45929096
5702	PSMC3		11	47418769	47426473
123036	TC2N		14	91779746	91867536
53981	CPSF2		14	92121969	92172145
122509	IFI27L1		14	94081301	94103846
3429	IFI27		14	94104836	94116698
79789	CLMN		14	95181940	95319908
783	CACNB2		10	18140424	18543557
9200	HACD1		10	17589032	17617374
221079	ARL5B		10	18659431	18681639
85459	CEP295		11	93661682	93730358
154810	AMOTL1		11	94706431	94876748
83700	JAM3		11	134069071	134152001
51762	RAB8B		15	63189560	63267776
6692	SPINT1		15	40844018	40858207
2200	FBN1		15	48408313	48645721
29117	BRD7		16	50313487	50368988
1152	CKB		14	103519667	103522833
27343	POLL		10	101578882	101588270
25911	DPCD		10	101570560	101609662

8879	SGPL1		10	70815948	70881184
25820	ARIH1		15	72474330	72602987
1353	COX11		17	54951902	54968703
252983	STXBP4		17	54968727	55173632
116159	CYYR1		21	26466209	26573286
322	APBB1		11	6395124	6419414
160140	C11orf65		11	108308519	108467531
54765	TRIM44		11	35662775	35818007
1200	TPP1		11	6612768	6619448
8642	DCHS1		11	6621330	6655809
118490	MSS51		10	73423579	73433561
374868	ATP9B		18	79069285	79378287
51700	CYB5R2		11	7665100	7677222
122618	PLD4		14	104924713	104937761
84460	ZMAT1	X		101882288	101932079
55839	CENPN		16	81006498	81033114
7702	ZNF143		11	9460319	9528524
5598	MAPK7		17	19377721	19383544
50810	HDGFL3		15	83112738	83207823
4176	MCM7		7	100092728	100101940
26253	CLEC4E		12	8533305	8540905
338339	CLEC4D		12	8509475	8522366
146227	BEAN1		16	66427295	66493529
7084	TK2		16	66508003	66552544
90701	SEC11C		18	59139866	59158832
201161	CENPV		17	16342534	16353656
51647	CIAO2B		16	66932065	66934423
80063	ATF7IP2		16	10326434	10483638
780776	TVP23A		16	10760919	10818794
9382	COG1		17	73192632	73208507
144100	PLEKHA7		11	16777297	17014414
644353	ZCCHC18	X		104112131	104115842
567	B2M		15	44711487	44718877
113201	GOLM2		15	44288719	44415758
4837	NNMT		11	114257787	114313536
116285	ACSM1		16	20623237	20698890
162394	SLFN5		17	35243071	35273655
117155	CATSPER2		15	43628503	43668118
NA	ZNF667-AS1		19	56477250	56504362
9665	MARF1		16	15594387	15643154
113174	SAAL1		11	18069935	18106087
219539	YPEL4		11	57645087	57649944
5479	PPIB		15	64155817	64163022

84191	CIAO2A	15	64072565	64093857
63901	FAM111A	11	59142748	59155039
9768	PCLAF	15	64364304	64387687
89797	NAV2	11	19350724	20121601
54386	TERF2IP	16	75647773	75761872
11318	GPR182	12	56994492	56998447
9880	ZBTB39	12	56998836	57006546
219988	PATL1	11	59636716	59669037
91419	ATP23	12	57906039	57959148
54948	MRPL16	11	59806140	59810778
79837	PIP4K2C	12	57591174	57603418
81628	TSC22D4	7	100463359	100479232
84689	MS4A14	11	60378530	60417756
2038	EPB42	15	43197227	43221018
4088	SMAD3	15	67063763	67195169
4141	MARS1	12	57475445	57517569
114785	MBD6	12	57520710	57530148
4824	NKX3-1	8	23678697	23682938
150290	DUSP18	22	30652051	30667890
7008	TEF	22	41367333	41399326
5245	PHB	17	49404049	49414905
138429	PIP5KL1	9	127920881	127930785
124460	SNX20	16	50666300	50681353
125336	LOXHD1	18	46476972	46657220
83473	KATNAL2	18	46917492	47102243
84064	HDHD2	18	47107408	47150500
125058	TBC1D16	17	79932343	80035872
762	CA4	17	60149942	60170899
4218	RAB8A	19	16111889	16134234
54815	GATAD2A	19	19385826	19508931
4597	MVD	16	88651935	88663161
81620	CDT1	16	88803789	88809258
84790	TUBA1C	12	49188736	49274600
84436	ZNF528	19	52397849	52418412
29946	SERTAD3	19	40440844	40444335
148137	PROSER3	19	35758143	35771028
558	AXL	19	41219223	41261766
84807	NFKBID	19	35887653	35902303
83696	TRAPPC9	8	139727725	140458579
90522	YIF1B	19	38303558	38317273
1613	DAPK3	19	3958453	3971123
10036	CHAF1A	19	4402640	4445018
80700	UBXN6	19	4444999	4457794

729359	PLIN4	19	4502180	4518465
83547	RILP	17	1646145	1649866
124997	WDR81	17	1716523	1738599
55611	OTUB1	11	63985853	64001811
148156	ZNF558	19	8806170	8832314
7001	PRDX2	19	12796820	12801800
7775	ZNF232	17	5105541	5123116
11314	CD300A	17	74466399	74484794
92162	TMEM88	17	7855066	7856099
2125	EVPL	17	76004502	76027306
7083	TK1	17	78174091	78187233
90850	ZNF598	16	1997654	2009821
21	ABCA3	16	2275881	2340746
54442	KCTD5	16	2682523	2709030
55048	VPS37C	11	61130257	61161615
220001	VWCE	11	61258286	61295316
5866	RAB3IL1	11	61897301	61920269
2495	FTH1	11	61959718	61967634
6520	SLC3A2	11	62856102	62888875
89849	ATG16L2	11	72814406	72843674
26005	C2CD3	11	74012714	74171210
9881	TRANK1	3	36826819	36945057
199223	TTC21A	3	39107704	39138903
54986	ULK4	3	41246599	41962130
8772	FADD	11	70203296	70207390
116071	BATF2	11	64987945	64997018
283234	CCDC88B	11	64340204	64357534
55209	SETD5	3	9397615	9479240
149603	RNF187	1	228487382	228496188
54541	DDIT4	10	72273924	72276036
29063	ZCCHC4	4	25312774	25370383
28511	NKIRAS2	17	42011382	42025644
7266	DNAJC7	17	41976421	42021376
440957	SMIM4	3	52534013	52579237
388753	COA6	1	234373456	234385080
3800	KIF5C	2	148875227	149026759
4247	MGAT2	14	49620799	49623481
27249	MMADHC	2	149569637	149587778
3008	H1-4	6	26156329	26157115
115294	PCMTD1	8	51817575	51899186
200845	KCTD6	3	58492096	58502360
378	ARF4	3	57571363	57598220
11259	FILIP1L	3	99830141	100114513

6890	TAP1	6	32845209	32853978
84289	ING5	2	241687085	241729478
197259	MLKL	16	74671855	74700960
399	RHOH	4	40191053	40246967
11031	RAB31	18	9708275	9862551
80346	REEP4	8	22138020	22141951
7148	TNXB	6	32041153	32115334
2237	FEN1	11	61792911	61797238
148738	HJV	1	146017468	146036746
124790	HEXIM2	17	45160700	45170040
6575	SLC20A2	8	42416475	42541926
79089	TMUB2	17	44186970	44191929
6774	STAT3	17	42313324	42388482
8754	ADAM9	8	38996869	39105261
8313	AXIN2	17	65528563	65561648
90075	ZNF30	19	34926903	34945170
753	LDLRAD4	18	13217498	13652755
9122	SLC16A4	1	110362851	110391082
3575	IL7R	5	35852695	35879603
29100	TMEM208	16	67227103	67229278
10768	AHCYL1	1	109984765	110023742
134218	DNAJC21	5	34929559	34958964
54910	SEMA4C	2	96859718	96870757
26505	CNNM3	2	96816245	96835382
2948	GSTM4	1	109656099	109674836
9677	PPIP5K1	15	43533462	43590253
10098	TSPAN5	4	98470367	98658611
9925	ZBTB5	9	37438102	37465450
9836	LCMT2	15	43323649	43330582
22847	ZNF507	19	32345594	32387667
27065	NSG1	4	4348140	4419058
10713	USP39	2	85602856	85649283
79155	TNIP2	4	2741648	2756342
388969	C2orf68	2	85605254	85612066
51255	RNF181	2	85595725	85597708
10791	VAMP5	2	85584431	85593406
57507	ZNF608	5	124636913	124748807
80723	SLC35G2	3	136818647	136855888
3635	INPP5D	2	233059967	233207903
56947	MFF	2	227325151	227357836
3965	LGALS9	17	27629798	27649560
521	ATP5ME	4	672436	674330
7386	UQCRFS1	19	29205320	29213151

84179	SLC49A3		4	681829	689271
1285	COL4A3		2	227164624	227314792
65110	UPF3A		13	114281601	114305817
NA			6	118501430	118502906
79663	HSPBAP1		3	122739999	122793831
6940	ZNF354A		5	178711512	178730659
255738	PCSK9		1	55039447	55064852
10421	CD2BP2		16	30350773	30355308
10636	RGS14		5	177357924	177372596
10960	LMAN2		5	177331567	177351668
148823	GCSAML		1	247507058	247577690
53917	RAB24		5	177301198	177303744
27166	PRELID1		5	177303799	177306949
7059	THBS3		1	155195588	155209051
11238	CA5B	X		15688830	15788411
55974	SLC50A1		1	155135344	155138857
3627	CXCL10		4	76021118	76023497
79628	SH3TC2		5	148923639	149063163
8706	B3GALNT1		3	161083883	161105411
7881	KCNAB1		3	156037701	156539138
64805	P2RY12		3	151336843	151384753
25942	SIN3A		15	75369379	75455842
6036	RNASE2		14	20955487	20956436
6037	RNASE3		14	20891399	20892348
6039	RNASE6		14	20781268	20782467
3576	CXCL8		4	73740541	73743716
6335	SCN9A		2	166195185	166376001
6383	SDC2		8	96493813	96611790
25932	CLIC4		1	24745382	24844321
151258	SLC38A11		2	164896186	164955525
1880	GPR183		13	99294539	99307399
5093	PCBP1		2	70087477	70089203
9022	CLIC3		9	136994608	136996568
27247	NFU1		2	69396113	69437628
150468	CKAP2L		2	112736349	112764664
123207	C15orf40		15	82988441	83011641
83640	RAMAC		15	82986210	82991057
200558	APLF		2	68467572	68655862
7798	LUZP1		1	23084023	23177808
284004	HEXD		17	82418318	82442645
699	BUB1		2	110637528	110678063
201255	LRRC45		17	82023305	82031151
10555	AGPAT2		9	136673143	136687457

7555	CNBP		3	129167827	129183922
4493	MT1E		16	56625475	56627112
5986	RFNG		17	82047902	82051831
7360	UGP2		2	63840940	63891562
686	BTD		3	15601341	15722311
55454	CSGALNACT2		10	43138445	43185302
6091	ROBO1		3	78597239	79767998
81844	TRIM56		7	101085481	101097967
51327	AHSP		16	31527900	31528803
9185	REPS2	X		16946658	17153272
4584	MUC3A		7	100949534	100968347
94056	SYAP1	X		16719612	16765340
3684	ITGAM		16	31259967	31332892
8460	TPST1		7	66205317	66420543
23252	OTUD3		1	19882395	19912945
51621	KLF13		15	31326835	31435665
83443	SF3B5		6	144094884	144095573
126917	IFFO2		1	18904280	18956676
10477	UBE2E3		2	180967248	181076585
116840	CNTROB		17	7932101	7949920
9283	GPR37L1		1	202122917	202133592
7324	UBE2E1		3	23805955	23891640
220988	HNRNPA3		2	177212563	177223958
23235	SIK2		11	111602449	111730855
57484	RNF150		4	140859807	141212877
2993	GYPA		4	144109303	144140751
55031	USP47		11	11841423	11961887
8427	ZNF282		7	149195546	149226238
10826	FAXDC2		5	154818492	154859252
11337	GABARAP		17	7240008	7242449
983	CDK1		10	60778331	60794852
7314	UBB		17	16380798	16382745
4798	NFRKB		11	129863636	129895590
10678	B3GNT2		2	62196115	62224731
2353	FOS		14	75278826	75282230
6419	SETMAR		3	4303304	4317567
7779	SLC30A1		1	211571568	211579161
136	ADORA2B		17	15944917	15975746
4255	MGMT		10	129467190	129770983
152518	NFXL1		4	47847233	47914667
929	CD14		5	140631728	140633701
57148	RALGAPB		20	38472816	38578859
84305	PYM1		12	55901413	55932618

51237	MZB1	5	139387467	139390081
345275	HSD17B13	4	87303789	87322886
5036	PA2G4	12	56104537	56113910
79071	ELOVL6	4	110045846	110199199
23204	ARL6IP1	16	18791669	18801572
133418	EMB	5	50396192	50443248
9229	DLGAP1	18	3496032	4455307
6773	STAT2	12	56341597	56360167
375611	SLC26A5	7	103352730	103446207
28991	COMMD5	8	144841042	144853736
6444	SGCD	5	155870344	156767788
80305	TRABD	22	50185915	50199598
3799	KIF5B	10	32009015	32056425
94031	HTRA3	4	8269754	8307098
3344	FOXN2	2	48314637	48379295
84669	USP32	17	60177327	60422470
2850	GPR27	3	71753855	71756496
NA		4	6663396	6676755
84864	RIOX2	3	97941818	97972457
11236	RNF139	8	124474880	124488618
79042	TSEN34	19	54189938	54194536
4696	NDUFA3	19	54102728	54109257
126014	OSCAR	19	54094668	54102692
85315	PAQR8	6	52361421	52407777
90338	ZNF160	19	53066606	53103436
1084	CEACAM3	19	41796587	41811554
2358	FPR2	19	51752026	51770531
238	ALK	2	29192774	29921586
55006	TRMT61B	2	28849821	28870309
3643	INSR	19	7112255	7294414
27094	KCNMB3	3	179236691	179267002
155066	ATP6V0E2	7	149872968	149891204
5581	PRKCE	2	45651345	46187990
10474	TADA3	3	9779860	9793011
29071	C1GALT1C1	X	120625674	120630054
79095	C9orf16	9	128160265	128163924
55657	ZNF692	1	248850006	248859144
203245	NAIF1	9	128061233	128068206
81831	NETO2	16	47077703	47143945
49861	CLDN20	6	155264013	155276548
51282	SCAND1	20	35953617	35959472
116844	LRG1	19	4536402	4540036
79801	SHCBP1	16	46578591	46621379

283742	FAM98B		15	38454127	38487710
90594	ZNF439		19	11848726	11883750
55636	CHD7		8	60678740	60868028
157570	ESCO2		8	27771949	27812640
1184	CLCN5	X		49922596	50099235
11076	TPPP		5	659862	693352
64979	MRPL36		5	1798385	1801366
9	NAT1		8	18170477	18223689
171023	ASXL1		20	32358330	32439319
24149	ZNF318		6	43307134	43369647
55144	LRRC8D		1	89821014	89936611
23566	LPAR3		1	84811602	84893206
598	BCL2L1		20	31664452	31723989
201514	ZNF584		19	58401504	58418327
80176	SPSB1		1	9292894	9369532
284307	ZIK1		19	57578456	57593890
27197	GPR82	X		41724181	41730130
344887	NMRAL2P		3	185959943	185980872
2857	GPR34	X		41688973	41697275
10287	RGS19		20	64073181	64079988
57687	VAT1L		16	77788564	77980107
23261	CAMTA1		1	6785454	7769706
83695	RHNO1		12	2876258	2889524
85442	KNDC1		10	133160219	133226412
170394	PWWP2B		10	132397168	132417859
163255	ZNF540		19	37551406	37614179
4815	NINJ2		12	564296	663779
6241	RRM2		2	10120698	10211725
51112	TRAPPC12		2	3379675	3485094
719	C3AR1		12	8056844	8066359
5728	PTEN		10	87863625	87971930
84978	FRMD5		15	43870761	44195271
83660	TLN2		15	62390526	62844631
51030	TVP23B		17	18781111	18806714
653464	SRGAP2C		1	121184811	121392874
91647	ATPAF2		17	17977409	18039209
83450	DRC3		17	17972813	18016889
149840	SHLD1		20	5750393	5864395
NA	OR52P2P		11	4431323	4432277
115196	ZNF554		19	2819868	2836735
83452	RAB33B		4	139453232	139476609
10869	USP19		3	49108046	49120938
5859	QARS1		3	49095932	49105130

94103	ORMDL3	17	39921041	39927601
51315	KRCC1	2	88027205	88064252
54205	CYCS	7	25118656	25125260
55106	SLFN12	17	35411060	35433283
257019	FRMD3	9	83242990	83538546
6641	SNTB1	8	120535745	120813273
10892	MALT1	18	58671465	58754477
3669	ISG20	15	88636153	88656483
154141	MBOAT1	6	20099684	20212469
10663	CXCR6	3	45940933	45948351
1051	CEBPB	20	50190830	50192668
566	AZU1	19	825097	832018
64581	CLEC7A	12	10116777	10130258
375444	C5orf34	5	43486709	43515148
682	BSG	19	571277	583493
55352	COPRS	17	31851871	31859291
160364	CLEC12A	12	9951316	9995694
10248	POP7	7	100706121	100707486
8801	SUCLG2	3	67360460	67654612
80765	STARD5	15	81309053	81324183
2783	GNB2	7	100673567	100679174
171024	SYNPO2	4	118850688	119061247
10978	CLP1	11	57648992	57661865
221421	RSPH9	6	43645036	43672600
54676	GTPBP2	6	43605316	43629264
55277	FGGY	1	59296638	59810647
79694	MANEA	6	95577535	95609452
4299	AFF1	4	86935002	87141054
9158	FIBP	11	65883740	65888531
57571	CARNS1	11	67414968	67425607
348938	NIPAL4	5	157460019	157474717
10125	RASGRP1	15	38488103	38565575
30008	EFEMP2	11	65866441	65873592
121275	OR10AD1	12	48202083	48203387
253725	WASHC2C	10	45727200	45792964
64393	ZMAT3	3	178960121	179072215
91607	SLFN11	17	35350305	35373701
80198	MUS81	11	65857126	65867653
79612	NAA16	13	41311267	41377030
80011	PSME3IP1	16	57152466	57186116
339122	RAB43	3	129087569	129122801
326624	RAB37	17	74670578	74747335
254122	SNX32	11	65833834	65856896

54961	SSH3	11	67303478	67312607
6670	SP3	2	173880850	173965702
1657	DMXL1	5	119037772	119249138
254042	METAP1D	2	171999943	172082430
51162	EGFL7	9	136658856	136672678
26960	NBEA	13	34942287	35673022
84153	RNASEH2C	11	65714005	65720818
26579	MYEOV	11	69294151	69367726
338692	ANKRD13D	11	67289300	67302485
4615	MYD88	3	38138478	38143022
23133	PHF8	X	53936676	54048958
541471	MIR4435-2H	2	111006015	111523376
344558	SH3RF3	2	109129205	109504634
156	GRK2	11	67266473	67286556
5970	RELA	11	65653597	65663090
283450	HECTD4	12	112160188	112382439
10855	HPSE	4	83292461	83335153
3310	HSPA6	1	161524540	161526894
51504	TRMT112	11	64316460	64317559
54674	LRRN3	7	111091006	111125454
150684	COMMD1	2	61888724	62147247
65059	RAPH1	2	203394345	203535335
54625	PARP14	3	122680839	122730840
10800	CYSLTR1	X	78271468	78327691
165631	PARP15	3	122577628	122639047
1163	CKS1B	1	154974653	154979251
126868	MAB21L3	1	116111755	116135240
91749	MFSD4B	6	111259327	111445354
81839	VANGL1	1	115641970	115698224
2745	GLRX	5	95751319	95822726
158399	ZNF483	9	111525159	111577844
653784	MZT2A	2	131464900	131492743
8658	TNKS	8	9555912	9782346
56977	STOX2	4	183797692	184023526
4296	MAP3K11	11	65597756	65615382
10221	TRIB1	8	125430358	125438403
4973	OLR1	12	10158301	10172138
1605	DAG1	3	49468703	49535618
339834	IHO1	3	49198428	49258106
440574	MICOS10	1	19484403	19629821
10534	ZNRD2	11	65570460	65573942
6599	SMARCC1	3	47585269	47782106
147687	ZNF417	19	57900296	57916610

5797	PTPRM	18	7566782	8406861
79834	PEAK1	15	77100656	77420144
8793	TNFRSF10D	8	23135588	23164027
90441	ZNF622	5	16451519	16465800
286205	SCAI	9	124942608	125143528
64802	NMNAT1	1	9943428	9985501
78999	LRFN4	11	66856647	66860475
7388	UQCRH	1	46303698	46316776
NA		11	65455258	65466720
3267	AGFG1	2	227472152	227561214
1267	CNP	17	41966763	41977740
91646	TDRD12	19	32719753	32829580
284131	ENDOV	17	80415165	80438086
57674	RNF213	17	80260866	80398786
162466	PHOSPHO1	17	49223366	49230787
26996	GPR160	3	170037995	170085392
83759	RBM4B	11	66664998	66677887
115123	MARCHF3	5	126867714	127030558
126074	SWSAP1	19	11374666	11376951
353189	SLCO4C1	5	102233986	102296284
5936	RBM4	11	66638667	66668374
9973	CCS	11	66593153	66606019
84984	CEP19	3	196706277	196712250
2787	GNG5	1	84498325	84506581
253827	MSRB3	12	65278643	65491430
23592	LEMD3	12	65169583	65248355
81793	TLR10	4	38772238	38782990
NA		15	41892777	41899528
10594	PRPF8	17	1650629	1684867
5306	PITPNA	17	1517718	1562792
23051	ZHX3	20	41178448	41317672
378108	STAG3L3	7	72969696	73005922
340024	SLC6A19	5	1201595	1225111
9156	EXO1	1	241847967	241895148
253959	RALGAPA1	14	35538352	35809304
3981	LIG4	13	108207439	108218368
488	ATP2A2	12	110280756	110351093
55055	ZWILCH	15	66504959	66550130
6124	RPL4	15	66498015	66524532
158038	LINGO2	9	27948078	28670286
79647	AKIRIN1	1	38991276	39006059
7368	UGT8	4	114598770	114678225
153339	TMEM167A	5	83052846	83077863

285590	SH3PXD2B	5	172325000	172454525
55196	RESF1	12	31959370	31993107
9975	NR1D2	3	23945286	23980617
25855	BRMS1	11	66337333	66345125
132720	FAM241A	4	112145454	112195256
2015	ADGRE1	19	6887566	6940459
171389	NLRP6	11	278365	285359
126328	NDUFA11	19	5891276	5904006
81876	RAB1B	11	66268590	66277492
125988	MICOS13	19	5678421	5680516
155185	AMZ1	7	2679522	2775500
26259	FBXW8	12	116910950	117031148
1488	CTBP2	10	124984317	125161170
9435	CHST2	3	143119771	143124014
545	ATR	3	142449007	142578733
125144	SNHG29	17	16438767	16478678
11065	UBE2C	20	45812576	45816957
80124	VCPIP1	8	66628487	66667231
201158	TVP23C	17	15502264	15563595
65108	MARCKSL1	1	32333839	32336233
80851	SH3BP5L	1	248810446	248825915
5708	PSMD2	3	184299198	184309050
22843	PPM1E	17	58755854	58985179
1466	CSRP2	12	76858709	76879023
55486	PARL	3	183829271	183884933
1649	DDIT3	12	57516588	57521737
5095	PCCA	13	100089015	100530437
10540	DCTN2	12	57530051	57547224
10025	MED16	19	867630	893218
9776	ATG13	11	46617527	46674818
23015	GOLGA8A	15	34379068	34437466
9537	TP53I11	11	44885903	44951306
9134	CCNE2	8	94879770	94896678
27257	LSM1	8	38163335	38176730
8665	EIF3F	11	7970251	8001862
285598	ARL10	5	176365487	176401865
1212	CLTB	5	176392455	176416569
374403	TBC1D10C	11	67403915	67410089
79772	MCTP1	5	94703741	95284575
57804	POLD4	11	67350772	67356972
143630	UBQLNL	11	5514393	5518431
10008	KCNE3	11	74454841	74467729
10589	DRAP1	11	65919274	65921563

7352	UCP3	11	74000277	74009085
83638	C11orf68	11	65916810	65919062
11007	CCDC85B	11	65890673	65891635
6199	RPS6KB2	11	67428460	67435401
116028	RMI2	16	11249619	11381662
146691	TOM1L2	17	17843511	17972422
54998	AURKAIP1	1	1373730	1375495
401505	TOMM5	9	37582646	37592604
55508	SLC35E3	12	68746125	68793964
169841	ZNF169	9	94259298	94301829
NA	OR52B3P	11	4378282	4379209
202309	GAPT	5	58491435	58497090
79666	PLEKHF2	8	95133785	95156685
2	A2M	12	9067664	9116229
57633	LRRN1	3	3799431	3849834
63893	UBE2O	17	76389456	76453152
93129	ORAI3	16	30949068	30956461
84747	UNC119B	12	120710458	120723640
163259	DENND2C	1	114582848	114670422
116068	LYSMD3	5	90515611	90529584
113829	SLC35A4	5	140564828	140569100
8636	SSNA1	9	137188660	137190370
664	BNIP3	10	131966455	131982013
79915	ATAD5	17	30831966	30895869
25914	RTTN	18	70003031	70205726
339487	ZBTB8OS	1	32600172	32650903
7694	ZNF135	19	58059239	58086310
1351	COX8A	11	63974620	63976543
NA		7	1838586	1849931
11145	PLAAT3	11	63573195	63616883
653583	PHLDB3	19	43474954	43504935
100128398		19	58002061	58011232
220441	RNF152	18	61808067	61894247
NA	SCAND2P	15	84631451	84647478
80230	RUFY1	5	179550554	179610012
374819	LRRC37A3	17	64854312	64919480
284207	METRNL	17	83079609	83095122
55884	WSB2	12	118032687	118062430
170082	TCEANC	X	13653189	13681964
51301	GCNT4	5	75025346	75052558
23625	FAM89B	11	65572349	65574198
54503	ZDHHC13	11	19117099	19176422
8408	ULK1	12	131894622	131923150

NA	NAP1L4P1		1	116532936	116534092
374907	B3GNT8		19	41425359	41428730
162517	FBXO39		17	6776215	6797101
NA	DLGAP1-AS1		18	3593732	3598363
64925	CCDC71		3	49162535	49166331
NA			12	31111652	31201235
100049716	NINJ2-AS1		12	630858	664196
219285	SAMD9L		7	93130056	93148385
10425	ARIH2		3	48918821	48986382
79413	ZBED2		3	111592900	111595346
475	ATOX1		5	151742316	151772532
9332	CD163		12	7470813	7503893
126069	ZNF491		19	11797667	11809622
3725	JUN		1	58780791	58784047
2629	GBA		1	155234452	155244699
28976	ACAD9		3	128879596	128916067
57104	PNPLA2		11	818914	825573
57085	AGTRAP		1	11736084	11754802
168451	THAP5		7	108554543	108569750
283229	CRACR2B		11	826144	831991
5441	POLR2L		11	837356	842529
389289	ANXA2R		5	43039081	43043170
10949	HNRNPA0		5	137745651	137754363
400728	FAM87B		1	817371	819837
66005	CHID1		11	867859	915058
253639	ZNF620		3	40477131	40518736
8269	TMEM187	X		153972754	153983194
374969	SVBP		1	42807052	42817397
1176	AP3S1		5	115841592	115914081
151188	ARL6IP6		2	152717893	152762396
60626	RIC8A		11	207511	215113
140461	ASB8		12	48147789	48181213
440836	ODF3B		22	50529710	50532580
285601	GPR150		5	95620087	95622142
259173	ALS2CL		3	46668995	46693704
25915	NDUFAF3		3	49020459	49023495
205327	C2orf69		2	199911293	199955935
83983	TSSK6		19	19512418	19515548
9659	PDE4DIP		1	148808181	149048286
1662	DDX10		11	108665069	108940927
4729	NDUFV2		18	9102630	9134345
85460	ZNF518B		4	10439880	10457426
84552	PARD6G		18	80157232	80247514

146547	PRSS36	16	31138926	31150083
125919	ZNF543	19	57320472	57330770
360200	TMPRSS9	19	2360238	2426239
282679	AQP11	11	77589391	77610356
100653515	CEP295NL	17	78870910	78903217
221294	NT5DC1	6	116100851	116249497
2731	GLDC	9	6532467	6645729
84987	COX14	12	50112082	50120457
55175	KLHL11	17	41848518	41865423
767	CA8	8	60185412	60281400
940	CD28	2	203706475	203738912
2859	GPR35	2	240605430	240631259
84875	PARP10	8	143977153	144012772
23512	SUZ12	17	31937007	32001038
115207	KCTD12	13	76880175	76886405
2907	GRINA	8	143990056	143993415
NA	GLULP4	9	34917175	34918296
9377	COX5A	15	74919791	74938083
26873	OPLAH	8	144051266	144063965
54512	EXOSC4	8	144078648	144080648
6879	TAF7	5	141260225	141320784
51341	ZBTB7A	19	4043303	4066899
80010	RMI1	9	83980798	84004074
55030	FBXO34	14	55271421	55361918
93621	MRFAP1	4	6640091	6642745
285237	C3orf38	3	88149959	88168729
283849	EXOC3L1	16	67184379	67190185
NA	C9orf106	9	129321016	129324905
374470	C12orf42	12	103237591	103496010
1537	CYC1	8	144095039	144097525
5187	PER1	17	8140472	8156506
160335	TMTC2	12	82686880	83134870
811	CALR	19	12938578	12944489
100506127	GVQW3	11	76381303	76414619
5886	RAD23A	19	12945855	12953642
90480	GADD45GIP1	19	12953119	12957223
79730	NSUN7	4	40749925	40811184
54734	RAB39A	11	107928448	107963482
1198	CLK3	15	74598500	74645414
2624	GATA2	3	128479427	128493201
10620	ARID3B	15	74541177	74598131
255520	ELMOD2	4	140524168	140553770
285908	LINC00174	7	66376044	66493566

50628	GEMIN4	17	744421	753999
81858	SHARPIN	8	144098633	144108124
144132	DNHD1	11	6497260	6593758
79571	GCC1	7	127580628	127593611
4261	CIITA	16	10866222	10943021
247	ALOX15B	17	8039034	8049134
201164	PLD6	17	17200995	17206333
23580	CDC42EP4	17	73283624	73312005
84232	MAF1	8	144104461	144107611
2205	FCER1A	1	159289714	159308224
729614		1	15834474	15848147
9582	APOBEC3B	22	38982347	38992804
51268	PIPOX	17	28950513	29057216
644997	PIK3CD-AS1	1	9652610	9654586
9495	AKAP5	14	64465499	64474503
163732	CITED4	1	40861054	40862363
154664	ABCA13	7	48171458	48647497
7710	ZNF154	19	57697367	57709194
55017	C14orf119	14	23095505	23100456
10194	TSHZ1	18	75210755	75289950
118672	PSTK	10	122954381	122997513
122809	SOCS4	14	55027230	55049489
284273	ZADH2	18	75195113	75209139
284417	TMEM150B	19	55312801	55334048
255043	TMEM86B	19	55226638	55228784
54952	TRNAU1AP	1	28553085	28578545
9282	MED14	X	40648305	40735858
81889	FAHD1	16	1826941	1840207
8575	PRKRA	2	178431414	178451512
223082	ZNRF2	7	30284597	30367689
285848	PNPLA1	6	36243203	36312229
386618	KCTD4	13	45192853	45194717
222166	MTURN	7	30134986	30162762
23060	ZNF609	15	64460742	64686068
NA	EMC3-AS1	3	9986893	10006990
NA	ATP5F1EP2	13	27945260	27945415
90411	MCFD2	2	46901870	46941855
283254	HARBI1	11	46602861	46617909
54494	C11orf71	11	114391443	114400511
NA	OR6K4P	1	158724113	158725067
23526	ARHGAP45	19	1065923	1086628
51276	ZNF571	19	37554782	37594792
3753	KCNE1	21	34446688	34512210

168620	BHLHA15		7	98211427	98212979
401563	C9orf139		9	137027464	137037957
8334	H2AC6		6	26124145	26139116
8347	H2BC4		6	26114873	26123926
151963	MB21D2		3	192796815	192917856
84333	PCGF5		10	91163012	91284337
5551	PRF1		10	70597348	70602759
NA	LINC02363		4	184334292	184353977
NA	SMG1P3		16	21446683	21520444
80045	GPR157		1	9100305	9129102
253832	ZDHHC20		13	21372571	21459370
124961	ZFP3		17	5078467	5096374
389362	PSMG4		6	3231403	3303373
NA	PDIA3P1		1	147172744	147179622
6748	SSR4	X		153793516	153798499
126375	ZNF792		19	34956354	34964229
404093	CUEDC1		17	57861243	57955412
23760	PITPNB		22	27851669	27920134
90843	TCEAL8	X		103252995	103255192
255252	LRRC57		15	42537820	42548802
64928	MRPL14		6	44113451	44127452
166379	BBS12		4	122732702	122744942
284406	ZFP82		19	36383120	36418644
64782	AEN		15	88621337	88632281
126003	TRAPPC5		19	7680833	7687703
25994	HIGD1A		3	42782908	42804490
NA			22	30475364	30492804
55526	DHTKD1		10	12068954	12123221
5430	POLR2A		17	7484366	7514616
23401	FRAT2		10	97332497	97334729
347736	NME9		3	138261437	138329886
388341	LRRC75A		17	16441577	16492193
91351	DDX60L		4	168356735	168537786
100287171	WASHC1		9	14475	73865
55076	TMEM45A		3	100492619	100577444
84282	RNF135		17	30970984	30999911
10586	MAB21L2		4	150582151	150584693
2187	FANCB	X		14835961	14873069
9966	TNFSF15		9	114784652	114806039
284459	ZNF875		19	37312837	37369365
26137	ZBTB20		3	114314500	115147288
205428	DIPK2A		3	143971823	144048719
90355	MACIR		5	103258763	103278660

57463	AMIGO1	1	109504178	109509738
169693	TMEM252	9	68536580	68540883
22820	COPG1	3	129249606	129277773
768211	RELL1	4	37590800	37686376
64864	RFX7	15	56087280	56245082
6517	SLC2A4	17	7281718	7288257
200205	IBA57	1	228165804	228182257
79673	ZNF329	19	58126248	58155110
5571	PRKAG1	12	49002274	49018807
64785	GINS3	16	58295080	58406147
119774	OR52K2	11	4449295	4450361
51363	CHST15	10	124007668	124093598
3418	IDH2	15	90083045	90102477
NA	OGFRP1	22	42269703	42279534
91304	TMEM259	19	1009648	1021179
84629	TNRC18	7	5306790	5425414
28955	DEXI	16	10928891	10942468
55505	NOP10	15	34341719	34343136
11022	TDRKH	1	151770107	151791416
64975	MRPL41	9	137551879	137552555
64764	CREB3L2	7	137874979	138002086
7318	UBA7	3	49805209	49813953
51021	MRPS16	10	73248843	73252693
81532	MOB2	11	1469457	1501247
202134	FAM153B	5	176060689	176116015
7325	UBE2E2	3	23203020	23591794
342945	ZSCAN22	19	58326994	58342332
NA	PDSS1P1	9	5084999	5086112
785	CACNB4	2	151832768	152099475
122553	TRAPPC6B	14	39147811	39170532
55666	NPLOC4	17	81556887	81648465
128553	TSHZ2	20	52972358	53495330
147968	CAPN12	19	38730187	38769904
23265	EXOC7	17	76081016	76121576
3838	KPNA2	17	68035708	68046854
654816	NCF1B	7	73220624	73235945
402415	XKRX	X	100913445	100929433
79598	CEP97	3	101724593	101770562
2242	FES	15	90883695	90895776
51218	GLRX5	14	95533503	95544724
201305	SPNS3	17	4433940	4488208
6304	SATB1	3	18345377	18445588
22906	TRAK1	3	42013802	42225890

23236	PLCB1		20	8077251	8968360
348235	SKA2		17	59109857	59155260
7267	TTC3		21	37073226	37203112
492311	IGIP		5	140125937	140129392
302	ANXA2		15	60347134	60402883
9628	RGS6		14	71932429	72566530
3214	HOXB4		17	48575507	48578350
389692	MAFA		8	143419182	143430732
79098	C1orf116		1	207018522	207032756
79087	ALG12		22	49900229	49918438
80781	COL18A1		21	45405165	45513720
83733	SLC25A18		22	17563450	17590995
28970	C11orf54		11	93741591	93764749
10473	HMGN4		6	26538366	26546933
221178	SPATA13		13	23979805	24307074
25904	CNOT10		3	32685145	32773875
389114	ZNF662		3	42906142	42919334
144608	C12orf60		12	14803666	14906586
5831	PYCR1		17	81932384	81942412
84316	NAA38		17	7856685	7885238
199675	MCEMP1		19	7676628	7679826
89874	SLC25A21		14	36677921	37172606
18	ABAT		16	8674596	8784575
57118	CAMK1D		10	12349547	12835545
145748	LYSMD4		15	99715697	99733561
166752	FREM3		4	143577302	143700675
389337	ARHGEF37		5	149551947	149634968
11251	PTGDR2		11	60850933	60855950
285753	CEP57L1		6	109095110	109163932
2842	GPR19		12	12660890	12696207
10856	RUVBL2		19	48993562	49015970
54880	BCOR	X		40049815	40177329
163351	GBP6		1	89364059	89388160
126075	CCDC159		19	11344684	11354944
391712	TRIM61		4	164954446	164977668
NA	OR7E38P		7	97966090	97967074
728340	GTF2H2C		5	69560208	69594723
4600	MX2		21	41361999	41409393
57634	EP400		12	131949942	132080460
84193	SETD3		14	99397748	99480889
128989	TANGO2		22	20017014	20067164
653604	H3C13		1	149813225	149813693
116541	MRPL54		19	3762682	3768575

220929	ZNF438	10	30820207	31031937
348327	ZNF530	19	57599885	57612722
4707	NDUFB1	14	92116122	92121917
64410	KLHL25	15	85759326	85794925
407738	TAFA1	3	68004247	68545621
10189	ALYREF	17	81887835	81891586
9241	NOG	17	56593699	56595611
7378	UPP1	7	48088628	48108736
84851	TRIM52	5	181254417	181261139
23585	TMEM50A	1	25338317	25362361
430	ASCL2	11	2268495	2270952
29110	TBK1	12	64452092	64502114
346389	MACC1	7	20134655	20217404
11200	CHEK2	22	28687743	28742422
NA	DOCK8-AS1	9	212824	215893
51807	TUBA8	22	18110331	18146554
389677	RBM12B	8	93729356	93741017
1233	CCR4	3	32951644	32956349
286826	LIN9	1	226231149	226309869
114781	BTBD9	6	38168451	38640148
54097	FAM3B	21	41304212	41357727
128239	IQGAP3	1	156525405	156572604
130502	TTC32	2	19896631	19901983
387893	KMT5A	12	123383773	123409353
28958	COA3	17	42795147	42798704
256435	ST6GALNAC3	1	76074746	76634603
8073	PTP4A2	1	31906421	31944856
112637020	SREBF2-AS1	22	41831215	41834665
29796	UQCR10	22	29767369	29770413
79174	CRELD2	22	49918167	49927540
152579	SCFD2	4	52872982	53366066
140739	UBE2F	2	237966827	238042782
11066	SNRNP35	12	123458088	123473154
3654	IRAK1	X	154010506	154019902
84319	CMSS1	3	99817837	100181732
220323	OAF	11	120211032	120230334
8338	H2AC20	1	149886918	149887411
317772	H2AC21	1	149887469	149887965
5463	POU6F1	12	51186936	51217708
10078	TSSC4	11	2400488	2403878
4070	TACSTD2	1	58575433	58577252
160365	CLECL1	12	9715860	9733299
254887	ZDHHC23	3	113947901	113965401

3009	H1-5		6	27866792	27867588
1435	CSF1		1	109910242	109930992
116447	TOP1MT		8	143304384	143359979
9735	KNTC1		12	122527246	122626396
253769	WDR27		6	169457212	169702067
10587	TXNRD2		22	19875517	19941820
4303	FOXO4	X		71095851	71103532
NA			11	33880643	33881800
57121	LPAR5		12	6618835	6635960
8303	SNN		16	11668455	11679152
81931	ZNF93		19	19900913	19963464
157313	CDCA2		8	25459199	25507911
9923	ZBTB40		1	22428838	22531157
8349	H2BC21		1	149884459	149886652
55911	APOBR		16	28494643	28498970
55967	NDUFA12		12	94897055	95003748
23762	OSBP2		22	30693782	30907824
79135	APOO	X		23833353	23907938
54732	TMED9		5	177592203	177597242
93517	SDR42E1		16	81988855	82011481
375287	RBM43		2	151247940	151261863
6612	SUMO3		21	44805617	44818779
83943	IMMP2L		7	110662644	111562517
752	FMNL1		17	45221444	45247319
391356	PTRHD1		2	24789734	24793382
11274	USP18		22	18150170	18177397
4700	NDUFA6		22	42085526	42090884
1133	CHRM5		15	33968497	34067458
140707	BRI3BP		12	124993645	125031231
8694	DGAT1		8	144314584	144326910
10509	SEMA4B		15	90160604	90229679
5813	PURA		5	140107777	140125619
8340	H2BC13		6	27807444	27807931
162387	MFSD6L		17	8797110	8799365
114659	LRRC37B		17	32007872	32053504
NA	LINC00482		17	81303771	81309248
10581	IFITM2		11	307631	315272
7127	TNFAIP2		14	103121476	103137439
9230	RAB11B		19	8389981	8404434
55015	PRPF39		14	45084107	45116282
283991	UBALD2		17	76265348	76271298
54033	RBM11		21	14216130	14228372
339230	CCDC137		17	81666737	81673904

729857	RGPD2	2	87755960	87825952
8651	SOCS1	16	11254408	11256204
6948	TCN2	22	30607003	30627271
23545	ATP6V0A2	12	123712353	123761755
5071	PRKN	6	161347417	162727775
9146	HGS	17	81683326	81703138
5600	MAPK11	22	50263713	50270767
123283	TARS3	15	101653596	101724473
64754	SMYD3	1	245749342	246507312
25840	METTL7A	12	50923472	50932510
400451	FAM174B	15	92617448	92809884
55010	PARPBP	12	102120185	102197520
246329	STAC3	12	57243453	57251188
3665	IRF7	11	612553	615983
113146	AHNAK2	14	104937244	104978374
NA	WASH3P	15	101961603	101976543
89782	LMLN	3	197960200	198043720
5034	P4HB	17	81843161	81860624
5719	PSMD13	11	236966	252984
5087	PBX1	1	164555584	164899296
7332	UBE2L3	22	21549447	21624034
6490	PMEL	12	55954105	55973317
333929	SNAI3	16	88677688	88686507
4733	DRG1	22	31399604	31530634
51479	ANKFY1	17	4163821	4263995
3434	IFIT1	10	89392546	89406487
51227	PIGP	21	37059170	37073170
127602	DNAH14	1	224896262	225399292
2124	EVI2B	17	31303770	31314105
140691	TRIM69	15	44728988	44767829
8519	IFITM1	11	313506	315272
3916	LAMP1	13	113297239	113323672
2865	FFAR3	19	35358460	35360489
84197	POMK	8	43093498	43131180
283897	C16orf54	16	29742463	29745990
200942	KLHDC8B	3	49171598	49176486
10308	ZNF267	16	31873807	31917357
8660	IRS2	13	109752695	109786583
57711	ZNF529	19	36534774	36605276
84529	C15orf41	15	36579626	36810248
51741	WWOX	16	78099430	79212667
NA	CIDECP1	3	10014238	10026365
146909	KIF18B	17	44924709	44947773

64757	MTARC1		1	220786913	220819659
388567	ZNF749		19	57435325	57447101
114792	KLHL32		6	96924620	97140754
57496	MRTFB		16	14071319	14266773
151888	BTLA		3	112463966	112499472
64222	TOR3A		1	179082070	179098023
148254	ZNF555		19	2841437	2860471
340591	CA5BP1	X		15674932	15703724
153768	PRELID2		5	145471799	145835369
23621	BACE1		11	117285207	117316259
55922	NKRF	X		119588337	119606443
342035	GLDN		15	51341655	51408005
2204	FCAR		19	54874235	54891420
6228	RPS23		5	82273320	82278396
54331	GNG2		14	51826195	51979342
11118	BTN3A2		6	26365159	26378320
NA	ANKRD20A5I		18	14179097	14252140
252884	ZNF396		18	35366694	35377337
84065	TMEM222		1	27322145	27336400
257106	ARHGAP30		1	161046946	161069970
4051	CYP4F3		19	15640897	15662825
23131	GPATCH8		17	44395281	44503430
221491	SMIM29		6	34246381	34249108
347240	KIF24		9	34252381	34311371
5138	PDE2A		11	72576141	72674591
55615	PRR5		22	44668547	44737681
80829	ZFP91		11	58579063	58621550
8372	HYAL3		3	50292831	50299405
84307	ZNF397		18	35241030	35267133
53373	TPCN1		12	113221050	113298585
7293	TNFRSF4		1	1211340	1214153
10614	HEXIM1		17	45148475	45152099
129293	TRABD2A		2	84821650	84907008
54821	ERCC6L	X		72204657	72239027
8784	TNFRSF18		1	1203508	1206592
23390	ZDHHC17		12	76763588	76853696
55893	ZNF395		8	28345590	28402701
5465	PPARA		22	46150521	46243756
129080	EMID1		22	29205896	29259597
83715	ESPN		1	6424776	6461367
353345	GPR141		7	37683796	37833788
91582	RPS19BP1		22	39529093	39532761
957	ENTPD5		14	73958010	74019399

4673	NAP1L1		12	76036585	76084735
4501	MT1X		16	56682470	56684196
2650	GCNT1		9	76419850	76651203
91404	SESTD1		2	179101678	179264832
23048	FNBP1		9	129887187	130043194
79659	DYNC2H1		11	103109410	103479863
4059	BCAM		19	44809071	44821421
222194	RSBN1L		7	77696459	77783022
349136	WDR86		7	151375909	151410727
2057	EPOR		19	11377207	11384342
51616	TAF9B	X		78129748	78139650
3010	H1-6		6	26107412	26108135
51547	SIRT7		17	81911939	81921323
9742	IFT140		16	1510427	1612072
57335	ZNF286A		17	15699577	15720787
9636	ISG15		1	1001138	1014540
387032	ZKSCAN4		6	28241697	28252269
145173	B3GLCT		13	31199975	31332276
51393	TRPV2		17	16415571	16437003
84281	C2orf88		2	189879609	190203484
166348	KBTBD12		3	127915232	127987671
2175	FANCA		16	89737549	89816657
7621	ZNF70		22	23738682	23751112
388335	TMEM220		17	10699015	10730023
3006	H1-2		6	26055740	26056470
550631	CCDC157		22	30356635	30378658
729956	SHISA7		19	55428740	55442863
341350	OVCH1		12	29412474	29497686
126432	RINL		19	38867830	38878275
100141515	C17orf99		17	78146353	78166177
285386	TPRG1		3	188947214	189325304
353116	RILPL1		12	123470054	123533719
375790	AGRN		1	1020120	1056118
4810	NHS	X		17375200	17735994
253012	HEPACAM2		7	93188534	93226469
389541	LAMTOR4		7	100148907	100155944
123879	DCUN1D3		16	20854925	20900358
390927	ZNF793		19	37506939	37548762
10383	TUBB4B		9	137241287	137243707
285180	RUFY4		2	218034960	218090581
57801	HES4		1	998962	1000172
374872	PEAK3		19	2274631	2282175
5359	PLSCR1		3	146515180	146544856

375341	C3orf62		3	49268596	49277232
84527	ZNF559		19	9323772	9351162
81442	OR6N2		1	158774222	158781204
440093	H3-5		12	31791185	31792298
6402	SELL		1	169690665	169711702
389792	IER5L		9	129175552	129178261
3014	H2AX		11	119093854	119095467
284325	C19orf54		19	40740856	40751553
348645	C22orf34		22	49414524	49657542
3040	HBA2		16	172876	173710
388115	CCDC9B		15	40331452	40340967
27158	NDOR1		9	137205685	137217009
344838	PAQR9		3	142949164	142963674
144535	CFAP54		12	96489571	96875555
1201	CLN3		16	28474111	28495575
6613	SUMO2		17	75165586	75182959
84247	RTL6		22	44492583	44498233
375743	PTAR1		9	69709522	69760011
283726	SAXO2		15	82262810	82284930
6006	RHCE		1	25362249	25430192
51114	ZDHHC9	X		129803288	129843909
643155	SMIM15		5	61157704	61162468
340277	FAM221A		7	23680130	23703249
401024	FSIP2		2	185738804	185833290
440603	BCL2L15		1	113876816	113887581
NA	TMSB4XP2		2	3617548	3617757
147694	ZNF548		19	57389850	57402992
4520	MTF1		1	37809574	37859592
441168	CALHM6		6	116461370	116463771
1269	CNR2		1	23870515	23913362
389206	BEND4		4	42110853	42152878
342125	TMC3		15	81331088	81374213
400499			16	11359845	11527245
120892	LRRK2		12	40196744	40369285
401494	HACD4		9	20999509	21031640
158293	FAM120AOS		9	93431441	93453581
135458	HUS1B		6	655939	656963
4697	NDUFA4		7	10931943	10940153
728780	ANKDD1B		5	75611182	75681773
51136	RNFT1		17	59952240	59964761
5649	RELN		7	103471784	103989658
3005	H1-0		22	37805093	37807436
83862	TMEM120A		7	75986831	75994656

196528	ARID2		12	45729706	45908040
222698	NKAPL		6	28259297	28260958
126231	ZNF573		19	37735833	37817300
84503	ZNF527		19	37371061	37393066
7581	ZNF33A		10	38010650	38065088
4128	MAOA	X		43654907	43746824
145853	C15orf61		15	67521131	67530146
100507206	LINC00943		12	126723412	126749027
55629	PNRC2		1	23956839	23963462
285016	ALKAL2		2	279558	288851
80317	ZKSCAN3		6	28349947	28369172
132660	LIN54		4	82909973	83012926
165186	TOGARAM2		2	28956611	29061373
100131211	NEMP2		2	190504338	190534722
9437	NCR1		19	54906148	54916140
282991	BLOC1S2		10	100273280	100286680
23424	TDRD7		9	97412096	97496125
653319	KIAA0895L		16	67175599	67184040
8644	AKR1C3		10	5035354	5107686
26010	SPATS2L		2	200305881	200482264
58500	ZNF250		8	144876497	144902168
7633	ZNF79		9	127424374	127445372
6275	S100A4		1	153543613	153550136
36	ACADSB		10	123008979	123058290
1510	CTSE		1	206009264	206023895
441191	RNF216P1		7	4973988	5040675
NA	EEF1A1P5		9	133019486	133020874
9901	SRGAP3		3	8980591	9363053
57573	ZNF471		19	56507850	56530221
84163	GTF2IRD2		7	74796144	74851551
60491	NIF3L1		2	200889327	200903938
84628	NTNG2		9	132161676	132244526
8295	TRRAP		7	98877933	99050831
647135	SRGAP2B		1	144887265	145095528
2526	FUT4		11	94543840	94549898
5657	PRTN3		19	840999	848175
91661	ZNF765		19	53389793	53430413
9819	TSC22D2		3	150408335	150466431
79693	YRDC		1	37802945	37808208
285971	ZNF775		7	150368790	150398630
140465	MYL6B		12	56152256	56159647
6817	SULT1A1		16	28605196	28623625
55660	PRPF40A		2	152651593	152717997

6536	SLC6A9		1	43991500	44031467
165679	SPTSSB		3	161344792	161372880
4122	MAN2A2		15	90902218	90922584
440184	CCDC196		14	66411704	66498677
3048	HBG2		11	5253188	5505605
55966	AJAP1		1	4654609	4792534
7516	XRCC2		7	152644776	152676141
4646	MYO6		6	75749192	75919537
65267	WNK3	X		54192823	54358642
57001	SDHAF3		7	97117698	97181763
23660	ZKSCAN5		7	99504662	99534700
51284	TLR7	X		12867072	12890361
100287569	LINC00173		12	116533422	116536518
84941	HSH2D		19	16134028	16158575
7582	ZNF33B		10	42574185	42638570
51321	AMZ2		17	68206076	68257712
8329	H2AC13		6	27808199	27808701
390036	OR52K1		11	4482646	4493497
8969	H2AC11		6	27133042	27135291
29966	STRN3		14	30893799	31026401
1146	CHRNG		2	232539692	232548115
64771	ILRUN		6	34587288	34696859
160760	PPTC7		12	110533245	110583318
140612	ZFP28		19	56538948	56556808
343990	CRACDL		2	98793846	98936259
445571	CBWD3		9	68232003	68300035
23365	ARHGEF12		11	120336413	120489937
9260	PDLIM7		5	177483394	177497606
57522	SRGAP1		12	63844700	64162217
57181	SLC39A10		2	195575977	195737702
837	CASP4		11	104942866	104969366
160	AP2A1		19	49767001	49807114
199704	ZNF585A		19	37106734	37172741
307	ANXA4		2	69644425	69827112
11152	WDR45	X		49074433	49101170
353088	ZNF429		19	21496682	21556270
29950	SERTAD1		19	40421589	40425992
57541	ZNF398		7	149126416	149183042
221785	ZSCAN25		7	99616946	99632408
126068	ZNF441		19	11767000	11784078
284390	ZNF763		19	11965037	11980617
8364	H4C3		6	26103933	26104337
4097	MAFG		17	81918270	81927735

1778	DYNC1H1	14	101964573	102056443
84619	ZGPAT	20	63707465	63736142
123096	SLC25A29	14	100291116	100306547
80055	PGAP1	2	196833004	196927796
400720	ZNF772	19	57466663	57477570
203102	ADAM32	8	39106990	39284917
23507	LRRC8B	1	89524829	89597864
8356	H3C12	6	27890315	27893106
341676	NEK5	13	52033611	52129092
400931	MIRLET7BHG	22	46053869	46113928
140688	NOL4L	20	32443059	32585074
6583	SLC22A4	5	132294394	132344190
9583	ENTPD4	8	23385783	23457695
10438	C1D	2	68041130	68110948
8363	H4C11	6	27824092	27824480
246778	IL27	16	28499362	28512051
128486	FITM2	20	44302840	44311202
100129940	HMGA2-AS1	12	65851340	65882167
124411	ZNF720	16	31713229	31794869
84301	DDI2	1	15617458	15669044
26020	LRP10	14	22871740	22881713
57162	PELI1	2	64092652	64144420
219927	MRPL21	11	68891276	68903835
104	ADARB1	21	45073853	45226560
344787	ZNF860	3	31981750	31991723
3064	HTT	4	3041422	3243960
128372	OR6N1	1	158747814	158772195
79674	VEPH1	3	157259742	157533619
4217	MAP3K5	6	136557046	136792477
373156	GSTK1	7	143244093	143270854
50861	STMN3	20	63639705	63657682
2996	GYPE	4	143870864	143905563
1305	COL13A1	10	69801867	69964275
79788	ZNF665	19	53159213	53193386
64078	SLC28A3	9	84275457	84340758
400823	FAM177B	1	222737207	222750805
NA	IRF1-AS1	5	132410636	132488702
10533	ATG7	3	11272309	11557665
1991	ELANE	19	851014	856247
56882	CDC42SE1	1	151050971	151070325
1803	DPP4	2	161992245	162074215
80380	PDCD1LG2	9	5510531	5571282
163059	ZNF433	19	12014732	12035741

10794	ZNF460		19	57280051	57294069
1379	CR1L		1	207645113	207738416
5253	PHF2		9	93576584	93679587
80217	CFAP43		10	104129888	104232364
79892	MCMBP		10	119829404	119892556
122773	KLHDC1		14	49693105	49753150
6884	TAF13		1	109062486	109076003
55210	ATAD3A		1	1512162	1534685
NA			17	17858227	17860041
259283	MDS2		1	23581495	23640568
28512	NKIRAS1		3	23889951	23946591
23303	KIF13B		8	29067278	29263124
85236	H2BC12		6	27146361	27146855
84671	ZNF347		19	53124072	53159075
89848	FCHSD1		5	141639302	141651418
58491	ZNF71		19	56595300	56626481
6277	S100A6		1	153534599	153536244
127687	C1orf122		1	37806979	37809454
3792	KEL		7	142941114	142962363
NA	FCGR1B		1	121087345	121097161
6191	RPS4X	X		72255679	72277248
10793	ZNF273		7	64870172	64930966
7637	ZNF84		12	133037292	133063304
63934	ZNF667		19	56439325	56478065
2870	GRK6		5	177403204	177442901
23607	CD2AP		6	47477789	47627263
54830	NUP62CL	X		107123427	107206433
65251	ZNF649		19	51889235	51905040
57209	ZNF248		10	37776526	37858106
57708	MIER1		1	66924895	66988619
258010	SVIP		11	22813799	22830299
84775	ZNF607		19	37696371	37719761
51170	HSD17B11		4	87336515	87391188
7789	ZXDA	X		57906708	57910820
83694	RPS6KL1		14	74903951	74923302
777	CACNA1E		1	181317690	181808084
11325	DDX42		17	63773603	63819317
55315	SLC29A3		10	71319259	71381423
59286	UBL5		19	9827892	9830115
9931	HELZ		17	67070444	67245989
7745	ZKSCAN8		6	28141883	28159460
4635	MYL4		17	47200446	47223679
79973	ZNF442		19	12345944	12365905

415116	PIM3	22	49960768	49964072
439	GET3	19	12737139	12748323
444	ASPH	8	61500556	61714640
200734	SPRED2	2	65310851	65432637
7574	ZNF26	12	132986365	133032952
10724	OGA	10	101784443	101818465
4494	MT1F	16	56657731	56660698
441478	NRARP	9	137299631	137302271
374900	ZNF568	19	36916329	36998700
158586	ZXDB	X	57591652	57597545
388558	ZNF808	19	52527652	52564464
7975	MAFK	7	1530702	1543043
5350	PLN	6	118548296	118561716
283518	KCNRG	13	50015254	50020922
11169	WDHD1	14	54938949	55027105
285989	ZNF789	7	99472890	99503650
1500	CTNND1	11	57753243	57819546
131870	NUDT16	3	131381671	131388830
987	LRBA	4	150264435	151015727
11177	BAZ1A	14	34752731	34875647
26112	CCDC69	5	151181052	151224093
4194	MDM4	1	204516379	204558120
27347	STK39	2	167954020	168247595
221477	C6orf89	6	36871870	36928964
338811	TAFA2	12	61708259	62279150
55705	IPO9	1	201829149	201884291
619518	SSBP3-AS1	1	54236440	54239063
112770	GLMP	1	156290089	156295689
10455	ECI2	6	4115689	4135597
10497	UNC13B	9	35161992	35405338
9646	CTR9	11	10751246	10801625
51734	MSRB1	16	1938210	1943326
6526	SLC5A3	21	34073578	34106260
63906	GPATCH3	1	26890488	26900467
4736	RPL10A	6	35468401	35470785
4512	MT-CO1	MT	5904	7445
4860	PNP	14	20468954	20477089
22887	FOXJ3	1	42176539	42335877
113402	SFT2D1	6	166319728	166342576
22876	INPP5F	10	119726042	119829147
9824	ARHGAP11A	15	32615144	32639941
56670	SUCNR1	3	151873643	151884619
3151	HMGN2	1	26472440	26476642

51465	UBE2J1		6	89326625	89352722
6263	RYR3		15	33310962	33866121
51714	SELENOT		3	150602875	150630445
1066	CES1		16	55802851	55833337
91300	R3HDM4		19	896503	913245
2869	GRK5		10	119207571	119459745
55253	TYW1		7	66995173	67239519
830	CAPZA2		7	116811070	116922049
7150	TOP1		20	41028822	41124487
221002	RASGEF1A		10	43194535	43267065
51490	SPOUT1		9	128819651	128829794
9851	KIAA0753		17	6578147	6640711
9937	DCLRE1A		10	113834725	113854383
79065	ATG9A		2	219219380	219229717
9737	GPRASP1	X		102651092	102659083
9755	TBKBP1		17	47694081	47712050
154467	CCDC167		6	37482938	37499893
23381	SMG5		1	156249224	156282825
7052	TGM2		20	38127385	38166578
259230	SGMS1		10	50305586	50625163
NA	RN7SKP291		1	55376526	55376852
26768	SNORA73B		1	28508559	28508762
NA	RN7SKP79		5	6848127	6848462
NA	RN7SKP180		17	72663823	72664152
26835	RNU4-1		12	120293097	120293237
NA	RNU6-1082P		9	117707996	117708102
NA	RN7SKP80		22	42565048	42565330
NA	RN7SKP47		9	74799895	74800222
NA	RNA5SP88		2	24564630	24564738
NA	RN7SKP230		5	109699500	109699834
NA			5	132468147	132468257
NA	CARNMT1-AS		9	74952968	74996293
170958	ZNF525		19	53365693	53392217
NA			2	68361214	68365584
116228	COX20		1	244835616	244845057
128387	TATDN3		1	212791828	212816830
345930	ECT2L		6	138795911	138904070
9293	GPR52		1	174447964	174449545
2214	FCGR3A		1	161541759	161550737
391114	OR6K3		1	158716327	158720720
92565	FANK1		10	125896564	126009592
399818	EEF1AKMT2		10	124748149	124791887
8528	DDO		6	110391771	110415575

574406	ADAMTSL4-A		1	150560202	150574552
440689	H2BC18		1	149782689	149812373
84852	ATP1A1-AS1		1	116378437	116421301
NA	SNHG5		6	85650491	85678932
439996	IFIT1B		10	89378056	89385205
90196	SYS1		20	45361937	45376798
3633	INPP5B		1	37860697	37947057
29935	RPA4	X		96883908	96885467
9935	MAFB		20	40685848	40689236
399761	BMS1P1		10	46786674	46811989
659	BMPR2		2	202376327	202567751
10471	PFDN6		6	33289302	33298401
339229	OXLD1		17	81665036	81666635
5696	PSMB8		6	32840717	32844679
5089	PBX2		6	32184733	32190202
177	AGER		6	32180968	32184322
6048	RNF5		6	32178405	32180793
494513	PJVK		2	178451346	178462102
643008	SMIM5		17	75633434	75641404
8859	STK19		6	31971091	31982821
7936	NELFE		6	31952087	31959038
100129029	PKP4-AS1		2	158658337	158735002
80736	SLC44A4		6	31863192	31879046
4758	NEU1		6	31857659	31862822
114769	CARD16		11	105041326	105101431
7918	GPANK1		6	31661228	31666283
7917	BAG6		6	31639028	31652705
730051	ZNF814		19	57848731	57889074
4277	MICB		6	31494881	31511124
90233	ZNF551		19	57681969	57717301
100507436	MICA		6	31399784	31415315
390980	ZNF805		19	57240632	57262728
28973	MRPS18B		6	30617840	30626395
11025	LILRB3		19	54216278	54223506
400997	ACOXL-AS1		2	111098345	111116407
3133	HLA-E		6	30489509	30494194
10107	TRIM10		6	30151943	30161211
NA		X		48568014	48574860
11138	TBC1D8		2	101007617	101252866
3134	HLA-F		6	29722775	29738528
5987	TRIM27		6	28903002	28923988
100268168			5	172954786	172959392
64901	RANBP17		5	170861870	171300015

83538	TTC25	17	41930617	41966503
51471	NAT8B	2	73700576	73701340
401431	ATP6V0E2-A!	7	149867697	149880610
57126	CD177	19	43353686	43363172
728568	C12orf73	12	103940763	103965708
NA	LINC02397	12	92466451	92492091
84912	SLC35B4	7	134289332	134316930
645121	CCNI2	5	132747426	132754403
286144	TRIQK	8	92883532	93017673
55851	PSENEN	19	35745600	35747519
5699	PSMB10	16	67934506	67936864
1874	E2F4	16	67192155	67198918
5150	PDE7A	8	65714334	65842322
100113407	TMEM170B	6	11537749	11583524
56953	NT5M	17	17303335	17347663
54458	PRR13	12	53441678	53446645
54809	SAMD9	7	93099513	93118023
255919	CNEP1R1	16	50024410	50037088
4676	NAP1L4	11	2944431	2992377
7114	TMSB4X	X	12975110	12977227
388931	MFSD2B	2	24010081	24063321
8110	DPF3	14	72619296	72894116
6453	ITSN1	21	33642400	33899861
162073	ITPRIPL2	16	19113932	19121629
9946	CRYZL1	21	33589341	33643926
3823	KLRC3	12	10412315	10420595
400941	LINC00487	2	6728177	6770311
93978	CLEC6A	12	8455962	8478330
10215	OLIG2	21	33025935	33029196
1667	DEFA1	8	6977649	6980080
220164	DOK6	18	69400888	69849087
653082	ZDHHC11B	5	710355	784729
440248	HERC2P9	15	28589492	28685264
3039	HBA1	16	176680	177522
3042	HBM	16	153892	166764
10866	HCP5	6	31463170	31478936
NA	HLA-H	6	29887752	29890482
131873	COL6A6	3	130560334	130678155
3105	HLA-A	6	29941260	29945884
201562	HACD2	3	123490820	123585053
131965	METTL6	3	15381275	15440566
26864	RNVU1-7	1	148038753	148038916
677807	SNORA22	7	65755526	65755659

NA		18	2778941	2779057
NA		4	168471409	168471539
103625684	RNU6-2	19	1021522	1021628
101954271	RNU6-9	19	893484	893590
693220	MIR635	17	68424451	68424548
NA	SNORD12C	20	49278940	49279028
55652	SLC48A1	12	47753916	47782751
NA	IGKC	2	88857161	88857683
NA	IGKV4-1	2	88885397	88886153
NA	IGKV6-21	2	89159751	89160366
NA	IGKV3D-11	2	90172802	90173414
NA	IGLV8-61	22	22098700	22099212
NA	IGLV6-57	22	22195799	22196276
NA	IGLV1-47	22	22357739	22358260
NA	IGLV7-46	22	22369614	22370087
NA	IGLV5-45	22	22375986	22376505
NA	IGLV1-44	22	22380766	22381347
NA	IGLV7-43	22	22395018	22395489
NA	IGLV1-40	22	22409766	22410282
NA	IGLV1-36	22	22431958	22432465
NA	IGLV3-25	22	22686726	22687271
NA	IGLV2-23	22	22697789	22698407
NA	IGLV3-21	22	22711689	22713203
NA	IGLV3-19	22	22720623	22721145
NA	IGLV2-18	22	22734607	22735089
NA	IGLV3-16	22	22747383	22747921
NA	IGLV2-14	22	22758700	22759218
NA	IGLV2-11	22	22792491	22793007
NA	IGLV3-10	22	22811747	22812281
NA	IGLV3-9	22	22819010	22819756
NA	IGLV3-1	22	22880706	22881396
NA	IGLJ2	22	22899481	22899655
NA	IGLC2	22	22900976	22901437
NA	IGLJ3	22	22904850	22905025
NA	IGLC3	22	22906342	22906803
NA	IGLC7	22	22922594	22923034
445347	TRGC1	7	38257879	38265678
NA	TRGV9	7	38317017	38318861
NA	TRGV8	7	38330343	38330935
NA	TRAV2	14	21712321	21712843
NA	TRAV5	14	21749178	21749705
NA	TRAV8-3	14	21852558	21853006
NA	TRAV13-2	14	21918188	21918756

NA	TRDV1	14	22096032	22096619
NA	TRAV29DV5	14	22163238	22163870
NA	TRAV41	14	22320188	22320691
NA	TRDV2	14	22422371	22423042
NA	TRAJ46	14	22493403	22493465
NA	TRAJ44	14	22494821	22494883
NA	TRAJ43	14	22495913	22495966
NA	TRAJ42	14	22496887	22496952
NA	TRAJ40	14	22499689	22499749
NA	TRAJ39	14	22501601	22501663
NA	TRAJ38	14	22502231	22502292
NA	TRAJ35	14	22506644	22506702
NA	TRAJ34	14	22507666	22507723
NA	TRAJ33	14	22508602	22508658
NA	TRAJ32	14	22509341	22509406
NA	TRAJ31	14	22510968	22511024
NA	TRAJ30	14	22512852	22512908
NA	TRAJ29	14	22513939	22513998
NA	TRAJ28	14	22515623	22515688
NA	TRAJ27	14	22516273	22516331
NA	TRAJ25	14	22518812	22518871
NA	TRAJ23	14	22520416	22520478
NA	TRAJ22	14	22522040	22522102
NA	TRAJ21	14	22523600	22523654
NA	TRAJ20	14	22524325	22524381
NA	TRAJ19	14	22525263	22525322
NA	TRAJ18	14	22525650	22525715
NA	TRAJ17	14	22526844	22526906
NA	TRAJ16	14	22528527	22528586
NA	TRAJ14	14	22530327	22530378
NA	TRAJ13	14	22531076	22531138
NA	TRAJ12	14	22531939	22531998
NA	TRAJ11	14	22532502	22532561
NA	TRAJ10	14	22533497	22533560
NA	TRAJ9	14	22535554	22535614
NA	TRAJ8	14	22536145	22536204
NA	TRAJ7	14	22537622	22537680
NA	TRAJ6	14	22539073	22539134
NA	TRAJ5	14	22540247	22540306
NA	TRAJ4	14	22542199	22542261
NA	TRAJ3	14	22543179	22543240
NA	IGHE	14	105597691	105601728
NA	IGHG4	14	105620506	105626066

NA	IGHG2	14	105639559	105644790
NA	IGHA1	14	105703995	105708665
NA	IGHG1	14	105736343	105743071
NA	IGHG3	14	105764503	105771405
NA	IGHV6-1	14	105939756	105940253
NA	IGHV1-3	14	106005095	106005574
NA	IGHV2-5	14	106037902	106038365
NA	IGHV3-7	14	106062151	106062683
NA	IGHV3-11	14	106116635	106117204
NA	IGHV3-13	14	106129540	106130072
NA	IGHV3-15	14	106153624	106154163
NA	IGHV1-18	14	106184901	106185394
NA	IGHV3-20	14	106210936	106211453
NA	IGHV3-21	14	106235064	106235594
NA	IGHV3-23	14	106268606	106269140
NA	IGHV3-33	14	106359793	106360324
NA	IGHV4-34	14	106373663	106374145
NA	IGHV4-39	14	106421711	106422218
NA	IGHV3-48	14	106537810	106538344
NA	IGHV3-49	14	106556936	106557477
NA	IGHV4-61	14	106639119	106639657
677841	SNORA74B	5	173020728	173020928
339263	LINC02693	17	21428263	21574517
NA		22	49548681	49556473
5462	POU5F1B	8	127322183	127420066
163486	DENND1B	1	197504748	197775696
NA	HNRNPA1P3	12	9027532	9029089
400798	C1orf220	1	178542752	178548889
375035	SFT2D2	1	168225938	168253021
11116	CEP43	6	166999317	167094789
NA		6	151225550	151225947
2532	ACKR1	1	159203307	159206500
1427	CRYGS	3	186538441	186546702
NA		1	40364766	40365183
NA		6	87408012	87511634
NA	STRADBP1	7	150512559	150513839
NA	MARCKSL1P	10	103175554	103176094
4893	NRAS	1	114704469	114716771
NA		4	187970273	187971284
51239	ANKRD39	2	96836611	96858016
1147	CHUK	10	100188300	100229596
83463	MXD3	5	177301461	177312757
NA	LINC00671	17	42874670	42898704

84986	ARHGAP19		10	97222173	97292673
3931	LCAT		16	67939750	67944131
NA	RPL18AP3		12	104265309	104265836
2086	ERV3-1		7	64990356	65006743
286467	FIRRE	X		131688779	131830862
10011	SRA1		5	140537340	140558252
51075	TMX2		11	57712593	57740973
4722	NDUFS3		11	47565336	47584562
54741	LEPROT		1	65420587	65436007
5500	PPP1CB		2	28751640	28802940
54978	SLC35F6		2	26764284	26781231
NA	PIGCP1		11	33075566	33076460
1192	CLIC1		6	31730581	31739763
23564	DDAH2		6	31727038	31730617
NA	RAB5CP1		10	74423435	74424014
10720	UGT2B11		4	69199951	69214748
7693	ZNF134		19	57614233	57624724
51202	DDX47		12	12813316	12829981
91664	ZNF845		19	53333749	53356906
NA	TMX2P1		9	37885683	37886390
NA	YBX1P10		9	35971344	35972318
NA	TAF9BP1		3	25754925	25755670
NA			3	25749488	25749896
NA	RPL13AP7		21	25361821	25381756
1241	LTB4R		14	24311450	24318036
2592	GALT		9	34638133	34651035
3672	ITGA1		5	52787916	52959209
6150	MRPL23		11	1947278	1984522
101060200	ZNF891		12	133106817	133130473
NA	PAXIP1-AS2		7	154928460	154952188
220729			3	197578213	197627906
NA	PLEKHM1P1		17	64779259	64837154
NA	ZNF788P		19	12092263	12137235
NA	RPS4XP1		12	100009052	100009838
255809	C19orf38		19	10836575	10869790
339210	C17orf67		17	56791913	56838773
51024	FIS1		7	101239458	101252316
NA	RAD51AP1P1		3	136899076	136900076
100505854	APTR		7	77657659	77696267
92482	BBIP1		10	110898730	110919201
NA	FAM185BP		7	77083787	77122116
10809	STARD10		11	72754729	72793681
NA	COPS8P2		3	68145004	68145630

400954	EML6	2	54723499	54972025
NA	RPSAP6	10	74371535	74372416
NA		9	121238117	121240089
NA	KRT8P26	11	65726939	65728214
414152	C10orf105	10	71711701	71737824
100271715	ARHGEF33	2	38889841	38975449
NA	SEPTIN7P2	7	45723780	45768985
NA		4	15730962	15731627
253128	LINC00612	12	9055589	9065070
219537	SMTNL1	11	57542641	57550274
283551	LINC01588	14	49927571	50105043
100130742	LRRC69	8	91101832	91219236
NA	PPIAP29	6	24976419	24976982
79680	RTL10	22	19846138	19854896
100130357		6	13264861	13295586
678655	CD27-AS1	12	6439001	6451567
137886	UBXN2B	8	58411359	58451501
644019	CBWD6	9	41131306	41199261
NA		5	34164698	34244796
728411	LINC00680	6	57946074	57961501
440270	GOLGA8B	15	34525207	34588503
NA		15	30470779	30507623
64601	VPS16	20	2840703	2866732
NA	GPX1P2	21	27143344	27143949
4636	MYL5	4	673580	682033
NA	ACTG1P3	20	1160584	1161697
55628	ZNF407	18	74597870	75065671
114044	MCM3AP-AS	21	46229196	46259390
79716	NPEPL1	20	58689131	58719238
400123	LINC00598	13	40079106	40535807
NA		22	20450122	20451824
388795	EFCAB8	20	32858923	32961609
NA	GUSBP9	5	71197646	71208130
56900	TMEM167B	1	109090764	109096934
100129094	BTNL10	1	228510425	228512305
NA	PDZK1P1	1	147993862	148014956
10437	IFI30	19	18173162	18178117
730101		6	52664366	52669155
NA		6	118452469	118454992
285780	LY86-AS1	6	6346465	6622771
NA	MTCO2P33	6	24947880	24948344
NA		6	26202156	26202654
NA		6	75610958	75611945

51192	CKLF	16	66552563	66566251
NA	PTGES3P2	2	25822469	25822950
NA		6	87173368	87174303
NA	NIP7P3	6	116137058	116137572
NA	H2AZP3	13	99215372	99215758
NA		6	156976294	156977072
NA	BRD7P4	6	111430260	111431781
NA		6	90116700	90117105
NA		6	35607628	35607934
NA	NPM1P37	6	69705287	69706160
NA	HSPA8P15	6	151411259	151413945
375190	FAM228B	2	24076526	24169640
101928464	ZNF433-AS1	19	11987617	12046275
NA		6	669081	670307
645191	LINGO3	19	2289784	2292024
NA	H2BC19P	1	149842875	149846486
NA	ASS1P1	6	25023247	25024483
NA	RPL21P28	1	212051524	212052006
NA	LINC01881	2	242088633	242160153
NA	RPS18P9	6	149592687	149601878
NA	RNU4ATAC1(	12	781133	781258
101929145	PPP3CB-AS1	10	73495525	73520070
2189	FANCG	9	35073835	35079942
84226	C2orf16	2	27537386	27582721
1052	CEBPD	8	47736913	47738164
NA	ARIH2OS	3	48917782	48918823
221786	FAM200A	7	99546300	99558536
10626	TRIM16	17	15627960	15684311
645843	TMEM14EP	3	152339695	152340990
80830	APOL6	22	35648446	35668404
7311	UBA52	19	18571730	18577550
9220	TIAF1	17	29073521	29078857
149069	DCDC2B	1	32209094	32216196
NA	RN7SKP110	6	90001222	90001506
NA	RN7SKP16	1	33336566	33336864
NA	RN7SKP26	18	45989653	45989945
NA	RN7SKP271	20	35643143	35643462
109616976	SNORA79B	14	20323179	20323326
100302139	MIR1537	1	235853000	235853060
NA	RN7SKP97	8	61631680	61632012
NA	RNA5SP39	13	108301212	108301331
NA	RNY3P8	13	95310830	95310955
105374219	LINC02068	3	172560888	172595607

NA	LINC01825		2	195532945	195540287
NA			20	53168643	53179550
NA			15	32519848	32536926
NA			2	28307063	28310560
NA	RABGAP1L-I		1	174896958	174897996
284391	ZNF844		19	12064731	12081565
NA	MTND5P28		2	120215181	120217279
NA	CCR5AS		3	46364955	46407059
3045	HBD		11	5232678	5243657
NA	IFITM3P2		12	31754720	31755121
NA	CCDC18-AS1		1	93262186	93346025
NA			1	143745249	143760669
NA			1	120267334	120341871
NA			10	8970125	8973468
NA			1	219222248	219225497
441307			7	520391	525238
100505783	OSER1-DT		20	44210907	44226027
NA	LINC02471		12	40156113	40211419
NA	LGALSL-DT		2	64395220	64453967
101927027	CHROMR		2	178413635	178440243
NA			7	90590619	90597353
NA	HNRNPRP1		10	21624592	21626286
NA	YES1P1		22	25647261	25649232
NA	IGKV3D-15		2	90114838	90115402
NA		X		46454097	46457199
NA	SNHG14		15	24978583	25420336
NA			11	5205041	5207308
NA			2	195448532	195481827
NA			7	127350128	127351523
100874055	LINC00570		2	11372612	11403175
100996485	C5orf66		5	135033280	135358219
NA	MTCYBP11		2	143093056	143094164
NA	IFIT6P		10	89362294	89363487
NA	OLFM5P		11	5518441	5524955
101929331			9	129282458	129312665
NA	IGHV4-59		14	106627249	106627825
NA	STK24-AS1		13	98577244	98578830
100506637	PRKAR2A-AS		3	48847572	48851981
NA	LINC00539		13	21302862	21348721
NA	MKRN5P	X		74161062	74162528
NA	RPL4P7		2	197028711	197030143
NA	TTC21B-AS1		2	165933857	165949891
NA			7	3337889	3338601

NA		3	172522228	172522444
NA	TNK2-AS1	3	195908076	195913986
NA	RTCA-AS1	1	100251528	100266179
NA		3	30626423	30626797
NA	IGHV3-74	14	106810442	106811131
100505696	SH3BP5-AS1	3	15254184	15264515
100874253	LINC00853	1	47179250	47180339
NA	MTND5P24	2	143094789	143096555
NA	RPL29P11	3	37016523	37017014
NA	YBX1P1	14	66012830	66013789
NA	ARL14EPP1	2	37148530	37149304
NA		10	99430919	99463259
401399	PRRT4	7	128350325	128361685
NA		9	6902670	6978859
NA	OR52K3P	11	4393783	4476361
NA		2	172480840	172556596
NA		1	246605873	246608102
NA		1	33307348	33349245
NA		22	18076527	18078884
NA	LRRK2-DT	12	40186009	40224915
NA		2	37816915	37817961
NA		9	63802144	63814159
NA	RBMS1P1	12	66234079	66235234
NA	TNPO1P1	10	84390908	84392045
554203	JPX	X	73944182	74070408
NA	MTCO3P5	2	143098898	143099933
100874337	PLCB1-IT1	20	8248704	8256918
400759	GBP1P1	1	89407679	89426243
NA	CYP4A22-AS1	1	47096653	47179271
NA	IGKV6D-21	2	90021567	90022185
NA		22	21885282	21885673
100191040	C2CD4D	1	151837819	151840557
NA	EIF2S2P5	1	114468315	114469282
348262	MCRIP1	17	81822361	81833302
NA		17	20056287	20056630
65010	SLC26A6	3	48625723	48635493
NA		X	80709928	80710705
440823	MIAT	22	26646411	26676475
401264	TRAM2-AS1	6	52576787	52643058
2074	ERCC6	10	49454470	49539538
102724231	C3orf86	3	44421127	44439946
NA		8	118282139	118400605
NA		1	27669468	27703063

NA		2	64143239	64252859
NA	DHDDS-AS1	1	26462756	26468014
NA	RIMKLBP2	1	219199914	219200567
51406	NOL7	6	13615335	13632739
NA	C5orf67	5	56511567	56606232
NA		2	230121370	230174223
NA	NRIR	2	6819463	6840464
101928527	PIGBOS1	15	55317184	55319161
101927621	LINC01534	19	36682636	36687449
NA		21	38237217	38238201
101928118		1	95161676	95233982
NA	LINC00623	1	120913184	121009291
389634	LINC00937	12	8295986	8396803
NA	AHCTF1P1	2	161500885	161507705
440465	BAIAP2-DT	17	81029130	81034881
647310	TEX22	14	105398538	105450106
29994		2	159670708	159712435
NA		6	6692741	6739030
84222	TMEM191A	22	20701114	20704606
143502	OR52I2	11	4581743	4593340
101928114	LINC01811	3	33956972	34677247
100506714	NUP50-DT	22	45133020	45163781
NA		9	104927553	104928892
NA	TMEM252-D	9	68541036	68644442
NA	PSPC1-AS2	13	19674624	19675884
NA		1	89661212	89661353
NA		1	19608114	19608568
NA	ADAM1B	12	111927018	111929017
79134	TMEM185B	2	120217479	120223400
NA	RPSAP21	1	236819634	236820518
NA	USF1P1	21	33334571	33335096
NA		X	51395959	51465661
NA	FTLP3	20	4023917	4024444
NA	OR52M2P	11	4514966	4515924
NA		10	5712174	5744067
NA	FAM66C	12	8180209	8216151
440498	HSBP1L1	18	79964582	79970822
104472714	DISC1-IT1	1	231925834	231945233
NA	LINC02611	2	98761098	98775361
NA	ZNF451-AS1	6	57114894	57174236
NA		7	23205787	23208045
NA	MTND6P11	2	143094233	143094715
NA		1	246108626	246113866

NA	LINC01359	1	64972225	65002489	
NA		2	172427774	172428603	
NA	CENPCP1	12	89500093	89502670	
NA		1	114459934	114460360	
NA	RPS2P36	10	96620877	96621707	
56967	C14orf132	14	96039324	96093889	
NA		8	115509602	115511325	
NA	NSA2P5	2	170077224	170078294	
NA	RPL7AP22	2	158548425	158549224	
100505538	RBM26-AS1	13	79406293	79427317	
NA		9	121369893	121463370	
NA		9	35772163	35790432	
NA		X	55908123	56209271	
440584	SLC2A1-AS1	1	42959049	42996814	
NA	MTND3P9	2	143098484	143098829	
NA		11	74745716	74746114	
NA		17	16040472	16041273	
NA	MTCO3P43	2	120211727	120212862	
NA		7	111411319	111411883	
NA		11	31618124	31767953	
NA	MTATP6P11	9	5099560	5100229	
NA	NA	NA	NA	NA	
NA		21	36360630	36362040	
NA	PTPN2P1	1	178746683	178747754	
386597	RNF144A-AS	2	6911754	6918734	
NA	ITPRIP-AS1	10	104323369	104327004	
55009	FAM174C	19	1275530	1279244	
NA		21	41441056	41445708	
NA		20	34014969	34017749	
157489	SDAD1P1	8	26375913	26382953	
100128731	OST4	2	27070472	27071654	
105374580	PRKCE-AS1	2	45674701	45675476	
728537	C2orf92	2	97664217	97703064	
NA		6	99424922	99432323	
NA	ITPKB-AS1	1	226668897	226676345	
NA	RPL7P52	22	40106325	40107066	
NA		2	143295421	143598002	
NA	PSMD10P1	20	50913731	50914411	
51716	CES1P1	16	55760566	55793960	
NA	LINC00659	20	62774121	62776996	
1719	DHFR	5	80626226	80654983	
NA		22	36445395	36454944	
400946	LINC00954	2	19868860	19885047	

643837	LINC01128	1	825138	859446
NA	AK3P5	10	32944481	32944999
NA		1	95243167	95278940
NA		1	160670778	160699761
NA	SATB1-AS1	3	18445024	18920401
NA	RAB1AP1	2	190992639	190993567
NA	VDAC1P8	6	143490424	143506406
NA		1	92930696	92934098
NA	RPS29P14	7	33036212	33036382
101927482	HECW2-AS1	2	196260024	196264204
NA	OR51AB1P	11	5291761	5292380
NA	CALM2P2	10	70163685	70164124
NA	BTBD6P1	1	23901471	23902737
6171	RPL41	12	56116590	56117967
100873997	KCNQ5-AS1	6	73130646	73143514
NA		2	206798325	206926876
NA		10	32347397	32374488
NA	GK-IT1	X	30671635	30672166
NA		2	177111036	177212662
NA	DPY19L1P1	7	32580949	32761787
NA	RALGAPA1P1	9	105520128	105526359
NA	RLIMP1	13	40618738	40621348
197135	PATL2	15	44665732	44711316
NA		11	1763009	1763749
NA		7	152120001	152121717
NA	PRDX3P1	22	38722743	38723505
401093	MBNL1-AS1	3	152245262	152269557
NA	RPSAP22	2	85490930	85491780
NA	ITGB1-DT	10	32958437	33082102
NA		7	139049456	139050006
NA		20	1361622	1362585
146542	ZNF688	16	30569346	30572734
100131801	PET100	19	7629793	7631956
100874111	STEAP3-AS1	2	119244422	119270714
NA		12	40286289	40286662
NA	RPL7P46	16	11027024	11027763
NA	B3GALNT1P1	2	69597353	69598951
NA	MIR181A1HG	1	198777861	198937471
NA	ALMS1-IT1	2	73456764	73459482
645784	ANKRD36BP2	2	88765807	88806612
NA		20	18289343	18359300
NA	FNTAP2	13	21530690	21531323
NA	UBBP1	2	136329441	136329913

NA			3	42601963	42654388
84320	ACBD6		1	180269653	180502954
NA	MTND5P14		9	5109496	5110762
NA	NFE4		7	102973522	102988856
158257	LINC02603		9	94176492	94259545
NA			2	100104919	100107504
104169671	LINC01036		1	187070700	187686628
NA			6	524171	525581
NA			3	50260303	50263358
NA			13	33355206	33676796
NA	ACTG1P14		9	6834456	6835577
NA	PHBP12		1	50780340	50781150
NA			1	44030437	44115913
NA	RPS23P8	X		70962964	70963293
388550	CEACAM22P		19	44537058	44620821
26226	FBXW4P1		22	23262767	23265005
NA			13	44234118	44256596
NA			1	89629725	89676386
NA			2	47924181	48314224
101927354			7	7255154	7278071
NA	OCLNP1		5	71074225	71093193
NA			9	116567636	116586328
NA	HNRNPA1P6[illegible]		1	100941017	100941995
NA	DSTNP1		21	46653558	46654022
3281	HSBP1		16	83807978	83819737
NA	OR52B5P		11	5557096	5564327
NA	RPL6P6		2	210088226	210089064
NA	SPATA20P1		21	38238227	38238664
NA			21	29058073	29060095
NA	YBX1P2		7	105582258	105583256
NA	CFAP58-DT		10	104351591	104353575
NA	RPS3P2		20	38537758	38538458
NA	TBL1XR1-AS[illegible]		3	177037405	177047923
NA	ACTG1P10	X		53142832	53143913
NA	LINC01712		1	83445967	83480024
101929147	WARS2-AS1		1	119140391	119275973
8847	DLEU2		13	49956670	50125720
NA			9	83831586	83868532
101927596	ANKRD44-AS[illegible]		2	197197991	197199273
105373934	LINC01891		2	234444590	234454595
NA	NANOGP5		9	100175178	100176054
NA			20	53397661	53412910
NA	LINC00899		22	46039907	46044853

NA	ARHGAP15-A		2	143629133	143656179
NA	MTCO2P5		2	143100930	143101355
NA			6	46096004	46129857
NA	TMEM44-AS		3	194584004	194590260
101929074	PIK3CD-AS2		1	9672426	9687555
NA		X		81363268	81364170
NA	DARS-AS1		2	135985176	136022593
NA			1	95120147	95138554
400761	LRRC8C-DT		1	89581291	89632916
641518	LEF1-AS1		4	108167525	108258037
NA	FAAHP1		1	46432129	46482493
NA			6	81724722	81736353
NA			20	50570975	50578041
NA			9	94332459	94363013
51372	TMA7		3	48440257	48444208
NA	IGHV3-43		14	106470264	106470800
100289178	GNG12-AS1		1	67832303	68202987
NA	VIPR1-AS1		3	42506465	42533258
NA	FTLP2	X		30630298	30630825
NA	OR52U1P		11	5719290	5720212
388789	SMIM26		20	18567347	18569563
NA			1	113698884	113699631
NA			2	38668202	38671421
NA			22	24101689	24103354
NA			7	66848496	66858136
NA	KANTR	X		53094145	53170914
NA		X		125203805	125204338
NA			1	202438396	202439957
NA	PRPS1P2		9	125150653	125151589
NA			4	183494884	183504494
100874205	ATP11A-AS1		13	112745449	112754693
101929591	KIZ-AS1		20	21154023	21218289
NA			2	201101382	201103136
101926943			7	74688864	74729001
NA	MTND4P22		2	143096839	143098007
101929947	ITGA6-AS1		2	172464262	172466022
NA	DUX4L50		9	63817748	63818462
201229	LYRM9		17	27878314	27894752
NA	TRBV29-1		7	142740206	142740894
NA	POLHP1		2	216156342	216156811
8490			1	163244505	163321894
101927560	LINC01725		1	83575776	83861023
NA			1	149785659	149793020

90625	ERVH48-1		21	42916803	42925646
NA	EEF1A1P14		1	194188967	194190342
100128420	LINC00892	X		136639539	136642429
NA	CTAGE7P		10	130106046	130108481
101929023	LINC01762		1	116423724	116478842
NA			17	45238028	45241734
NA			1	101025844	101090513
NA	HNRNPA1P4		6	166334568	166335471
NA			1	9660828	9662626
2876	GPX1		3	49357176	49358358
NA	FAM53B-AS		10	124703625	124714217
NA	UBE2FP3		1	111437514	111438037
NA	TLR8-AS1	X		12902817	12908333
100506915	CHRM3-AS2		1	239703381	239770130
158801	NKAPP1	X		120120953	120245267
NA	IATPR		10	33096257	33116672
NA	HOTAIRM1		7	27095647	27100265
643376	BTBD18		11	57743514	57751781
NA	STXBP5-AS1		6	146824539	147204614
NA			1	231520729	231528618
NA	KRT8P39		12	68705634	68707066
NA	EIF4EP1		20	5548840	5549481
728431	LINC01137		1	37350934	37474411
NA			21	25928754	25952614
730227	LINC01136		1	203298758	203305309
8341	H2BC15		6	27837760	27865798
NA	NA	NA		NA	NA
NA			1	64941979	64942365
440567	UQCRHL		1	15807169	15809348
NA			6	80441295	80466674
101929526	DTD1-AS1		20	18674395	18698709
NA	EDDM3CP		2	134319654	134320078
NA	MTND4P26		2	120213631	120214989
203569	PAGE2	X		55089018	55092842
NA			3	36880184	36880729
7726	TRIM26		6	30184455	30213427
NA		X		10847578	11111220
NA	RBPMSLP		21	15744004	15745080
NA	LANCL1-AS1		2	210324759	210469246
NA			5	132468890	132473043
100996590	LINC01505		9	105993310	106740875
NA	BCAS2P2		1	100393033	100394026
NA			7	64835280	64836882

NA		2	170700368	170813670
NA	CTBP2P8	1	68161761	68163090
NA		2	102438713	102440475
100505881	MAGI2-AS3	7	79452877	79471208
101928941	RHOXF1P1	X	119978713	120025138
101927015	LINC01506	9	68543541	68546589
NA	PTGES3P1	1	89104285	89104767
NA	CSNK1G2P1	9	5040945	5041940
NA	C2CD4D-AS1	1	151841877	151850385
NA	LINC01934	2	181086076	181409321
100507495	SDCBP2-AS1	20	1325405	1378735
NA	UPF3AP3	9	99998301	99999069
NA		7	7738524	7742574
729978	NPIPB2	16	11927259	11976643
NA		3	197850400	197850954
NA		11	72940498	72941112
105755953	LINC02676	10	8897989	8914596
NA	TPT1P9	9	118082869	118083382
NA		1	64918443	64918683
101409253	LINC00676	13	109728282	109730007
NA	OR52X1P	11	6066873	6067786
NA		11	1760348	1762486
100505635	DAAM2-AS1	6	39881804	39900071
NA	ZRANB2-AS1	1	71048855	71067184
NA		1	48435967	48437223
NA		22	49612657	49615716
NA	RPSAP16	1	179968686	179969506
NA		2	165794851	165810010
101928153	ITGA9-AS1	3	37693655	37861802
NA		11	111670956	111672404
105371807		17	48294335	48308762
NA	LINC00957	7	44039171	44042306
NA		2	169100743	169101210
NA		22	45792418	45793636
NA		6	155253139	155256724
NA		2	230520594	230580006
100132891	MSC-AS1	8	71828167	72118393
100507612	LINC00402	13	74231457	74259976
NA		5	71220356	71259238
NA		2	38601598	38602178
284749	LINC00494	20	48359884	48370636
NA	FBXO30-DT	6	145799409	145887430
NA	LINC00894	X	149938613	150224580

NA	PHACTR2-AS	6	143554325	143562031
9674	KIAA0040	1	175156986	175192999
NA		14	99262948	99264735
NA		1	100995473	100996260
NA		2	190880797	190882059
57000	GSN-AS1	9	121280768	121285530
104326055	APOA1-AS	11	116836117	116856018
NA	MTCO2P11	9	5098341	5099325
645676	ASH1L-AS1	1	155562042	155563944
100126791	EGOT	3	4749192	4751590
284900	TTC28-AS1	22	27919376	28008581
NA		7	157197600	157198511
100874212	MYCBP2-AS1	13	77026767	77129717
100874058	COX10-AS1	17	13755574	14069495
NA	RPL23AP37	2	64347193	64347741
400945	LINC01376	2	18986451	19348067
NA		2	37562486	37646698
100885782	MYO16-AS1	13	109163902	109201483
NA	MTND4P14	9	5107937	5109290
NA		2	117833937	117841658
NA	MTND4LP12	2	143098123	143098417
342618	SLFN14	17	35543985	35560819
NA	TRHDE-AS1	12	72249964	72276954
112935969	SCAT8	6	63806836	63822642
NA	SUCLG2P2	12	94548241	94549536
NA	DDX11L2	2	113599036	113601261
259285	TAS2R39	7	143183419	143184435
NA	MFF-DT	2	227221052	227325711
NA		2	102433957	102435340
NA		22	20058030	20070569
NA	RNF217-AS1	6	124644434	124963165
NA	YWHAQP5	2	98694109	98695490
NA	TCF3P1	9	5110913	5112849
NA	RAD23BP1	3	18538673	18539899
NA		X	51856968	51858066
NA	KRT18P5	22	20482815	20484105
100506881	MKLN1-AS	7	131309469	131328312
NA	SDR42E1P5	2	102411243	102412108
NA	TMSB4XP1	1	42500205	42500339
NA		10	26577676	26622338
81571	MIR600HG	9	123109494	123115477
NA	HNRNPA1P7(	12	68035767	68036853
NA	NF1P8	2	131189834	131198007

101927547	ANKRD44-IT1	2	197250858	197302519
NA		9	131132852	131167505
NA	EDRF1-AS1	10	125725634	125752110
NA		9	68788307	68789670
NA	NANOGP4	7	29212302	29213214
NA		1	42891094	42892430
NA	DDX10P1	10	30919012	30921235
91368	CDKN2AIPNL	5	134402065	134411881
NA		2	64450096	64450524
100499405	LINC00987	12	9240003	9257960
NA	MAPK6P3	13	42068932	42071093
NA	OR51R1P	11	4418060	4419011
100506866	TTN-AS1	2	178521183	178779963
100289098		7	66493607	66495758
NA	CDC42P6	4	22727375	22727950
NA	TUSC8	13	44400250	44405984
NA		22	25580241	25581382
5863	RGL2	6	33291654	33298942
105372751	LINC01684	21	24306939	24547942
387723	C10orf143	10	130020025	130110830
105378580	LINC01409	1	778747	810065
101927891	PKN2-AS1	1	87620803	88685204
400958	LINC02245	2	64901840	65056168
3833	KIFC1	6	33391823	33409896
NA	KRR1P1	13	20070250	20072136
NA		5	56842016	56862164
NA	MTCO3P11	9	5100236	5101009
NA		2	112641832	112645720
NA	EEF1A1P30	X	120210461	120212680
285550	FAM200B	4	15681662	15690447
NA		7	16970320	17299368
NA	ADAMTSL4-A	1	150548562	150557724
NA	LINC00211	2	37820498	37876274
NA	LINC01473	2	186032884	186422432
100874158	LINC00398	13	30803206	30810645
101929010	SIRPG-AS1	20	1633508	1648472
100506334	LINC00649	21	33915534	33977691
11191	PTENP1	9	33673504	33677499
NA		1	116013813	116017705
101927851		1	234957342	234970062
NA		2	64330481	64332801
NA		1	78666272	78666695
NA		2	54661011	54680045

NA	ZDHHC4P1		13	42933858	42934892
55592	GOLGA2P5		12	100156357	100173659
NA	RNF14P3		7	98998538	98999930
103352539	LINC01410		9	62801461	62813486
339751	MAP3K20-AS		2	173166446	173282036
100506215	PAXBP1-AS1		21	32728097	32747065
NA			2	64338067	64341647
NA	OR7E7P		7	97946987	97947998
NA	CCR12P		13	99404684	99417224
NA			10	20070805	20091784
389860	PAGE2B	X		55075030	55078909
NA			1	153533603	153535115
NA			4	139436368	139436494
NA	RN7SL546P		14	102582888	102583177
NA			7	139172516	139174266
81567	TXNDC5		6	7881517	7910788
10432	RBM14		11	66616626	66629934
142686	ASB14		3	57268342	57292685
100506826	MYLK-AS1		3	123585300	123644568
NA			7	25689580	25689959
NA			10	13631143	13668445
4830	NME1		17	51153559	51162428
60489	APOBEC3G		22	39077067	39087743
283507	SUGT1P3		13	40882577	40921774
1668	DEFA3		8	7015869	7018297
NA	IGKV1-6		2	88966262	88966767
NA	RN7SL674P		2	11584773	11585047
158	ADSL		22	40346500	40390463
NA			11	5254392	5678434
NA			3	152118173	152151724
NA	ZBTB20-AS3		3	114873114	114876514
NA	RN7SL368P		19	43660500	43660776
NA	IGKV3-20		2	89142574	89143160
11027	LILRA2		19	54572920	54590287
NA	YBX1P3		3	114930541	114931449
107986114			3	113019468	113184377
5698	PSMB9		6	32844136	32859851
NA	ETF1P2		7	151501878	151503463
101928085	ERICH6-AS1		3	150703564	150723005
NA		X		129794994	129796963
NA			3	158732263	158784070
90639	COX19		7	898778	975549
NA	RN7SL587P		14	77547566	77547846

728358	DEFA1B	8	6996766	6999195
100126793	GHRLOS	3	10285754	10294903
NA	IGKV1-17	2	89117342	89117844
NA	FAM214BP1	3	114231398	114233021
54596	L1TD1	1	62194849	62212328
358	AQP1	7	30911853	30925517
NA	IGKV1-8	2	88992409	88992931
57461	ISY1	3	129127415	129161063
NA		3	136736500	136737229
NA		5	177264766	177265241
NA	IGKV1-16	2	89099859	89100361
NA	RN7SL128P	6	20421583	20421887
730091	LINC00886	3	156747343	156817062
NA	RPL4P1	14	21750269	21751546
NA	RPS27AP10	5	141588935	141589142
NA	HMGN1P8	3	167694355	167694642
285286	LINC00877	3	71943592	72279503
NA	IGKV1D-16	2	90100236	90100738
27297	CRCP	7	66114604	66154568
101927056	LINC02614	3	125827238	125916384
NA	IGKV2-24	2	89176328	89177160
101927111	SUCLG2-AS1	3	67654669	67947713
NA	IGKV3-11	2	89027171	89027731
9551	ATP5MF	7	99448475	99466186
NA		3	114214313	114236204
NA	RPS15AP24	8	30117384	30117779
387036	GUSBP2	6	26871484	26956554
10093	ARPC4	3	9792495	9807726
NA		12	95467397	95467861
100131117	ZBTB20-AS1	3	114351771	114388978
NA		5	98954394	98954972
10552	ARPC1A	7	99325898	99366262
NA	IGKV1-9	2	89009982	89010515
101927974		7	107739999	107744581
100131434	LINC00893	X	149527591	149540959
80301	PLEKHO2	15	64841883	64868002
5297	PI4KA	22	20707691	20859417
25764	HYPK	15	43796142	43804427
644090		7	151806490	151810820
NA	IGKV1-33	2	89266494	89268506
NA	SLC5A4-AS1	22	32205115	32278382
NA	RPL9P32	19	29956369	29956988
NA	RN7SL124P	2	151372003	151372287

NA	RPL5P13	4	142286917	142287625
285972	LINC00996	7	150433654	150448140
101929754	ZBTB20-AS5	3	114445521	114529452
NA		3	101576489	101576947
NA	RPL12P32	12	31948334	31948827
NA	IGKV1-39	2	89319625	89320146
5273	SERPINB10	18	63897174	63936111
NA	RPL21P11	14	90366697	90367179
NA		5	72878868	72879185
2790	GNG10	9	111661605	111670226
1270	CNTF	11	58622665	58625733
100874131	ZBTB20-AS4	3	115100423	115103061
9907	AP5Z1	7	4775615	4794397
NA	RPL5P11	4	173419245	173420137
30816	ERVW-1	7	92468380	92477986
NA	UBA52P3	14	58141279	58141660
150000	ABCC13	21	14236206	14362754
9553	MRPL33	2	27771717	27988087
NA		3	157174201	157381265
NA	IGKV2-30	2	89244781	89245596
NA	IGKV1-12	2	89040224	89040745
NA		7	128651185	128652334
154881	KCTD7	7	66628767	66649067
NA	RN7SL141P	3	172237326	172237591
100887750	MRPS31P5	13	52167709	52194465
401115	C4orf48	4	2041993	2043970
NA	IGKV1-5	2	88947301	88947957
NA	DNAJB6P7	3	125344085	125344797
NA	RN7SL172P	3	121653994	121654296
NA	RN7SL834P	2	230507101	230507386
NA	RPS29P12	5	180945756	180945907
116143	WDR92	2	68122936	68157549
NA		7	106372251	106770207
NA	RPL7P18	5	94825961	94826694
64968	MRPS6	21	34073224	34143034
147007	TMEM199	17	28357642	28363683
NA		7	92457564	92495601
NA	RPL9P33	19	51621136	51621701
NA	IGKV2-28	2	89221698	89222461
105377105	FLNB-AS1	3	58162547	58170636
85014	TMEM141	9	136791344	136793317
104502416	FOXP1-AS1	3	71289769	71305853
NA	RN7SL151P	9	21699314	21699596

NA	RN7SL597P	13	41121876	41122171
NA	IGKV3-15	2	89085177	89085787
405754	ERVFRD-1	6	11102489	11111725
27350	APOBEC3C	22	39014257	39020352
NA	IGKV1-27	2	89213423	89213928
151516	ASPRV1	2	69960089	69962265
NA	RN7SL396P	8	119862662	119862956
NA	FCGR2C	1	161581339	161605662
7335	UBE2V1	20	50081124	50115959
NA	CD46P1	1	207645234	207657410
3043	HBB	11	5225464	5229395
NA	GABPB1-AS1	15	50354959	50372202
100507300	ALKBH3-AS1	11	43909289	43920944
144571	A2M-AS1	12	9065163	9068689
NA	MALINC1	5	140071312	140109274
100506020	ARAP1-AS2	11	72700474	72705607
100130231	LINC00861	8	125859721	126292788
NA	EEF1A1P4	12	19456244	19457648
386627	SAP30L-AS1	5	154325568	154445850
101928784	RORA-AS1	15	60479152	60630637
728769	SCAMP1-AS1	5	78342365	78360507
101928100		12	10363638	10398506
92285	ZNF585B	19	37181579	37218153
145837	DRAIC	15	69462921	69843120
109729137	MEF2C-AS2	5	88676033	88779088
NA		12	92145573	92226557
644873	LINC01184	5	127939152	128083172
100996286	LINC02273	4	152090459	152104720
NA	LINC01513	5	10479371	10482309
152195	NUDT16P1	3	131361898	131365122
101929241	LINC02325	14	97427071	97582146
100507053		4	99088805	99301356
100505676	RRS1-AS1	8	66419589	66428977
388011	LINC01550	14	97924637	97978124
727677	CASC8	8	127289817	127482139
NA	UBR5-AS1	8	102239394	102253750
NA	LINC02458	12	89010681	89309566
286190	LACTB2-AS1	8	70608577	70663279
100505658	DIAPH1-AS1	5	141558311	141565263
100507632	LINC00968	8	56496048	56559823
55766	H2AJ	12	14774383	14778002
NA	LINC00920	16	66408524	66412135
390595	UBAP1L	15	65092770	65115197

102546294		5	148088125	148383907
NA		14	61294909	61322838
NA	LY6E-DT	8	142981738	143018437
100131067	CKMT2-AS1	5	81201341	81301565
100507266	STX18-AS1	4	4542131	4787359
100505894	TMEM161B-/	5	88268891	88439337
100533182	SEC24B-AS1	4	109347475	109433817
283663	LINC00926	15	57300365	57307769
3640	INSL3	19	17816512	17821574
NA		4	52945649	52958087
NA	RRN3P1	16	21796106	21820410
NA	GAPDHP60	4	87207092	87208086
NA		4	87166844	87167734
NA	KRT8P4	8	89412621	89415071
NA		5	111277517	111302567
100507602	TRIM52-AS1	5	181261169	181272307
NA	CCNL2P1	4	42561457	42562425
NA	TRAJ51	14	22487183	22487245
441864	TARM1	19	54069895	54081365
NA	SEMA6A-AS1	5	116447547	116508276
NA	LINC02062	5	98929171	98995013
NA		X	77910741	77964890
NA		5	157375741	157384950
NA		5	134399495	134401921
100526737	RBM14-RBM	11	66616626	66646469
285407		11	71673885	71818238
NA		4	80182637	80197410
102467147	LINC01948	5	56451627	56481946
NA		4	173322206	173329694
NA	HIGD1AP14	4	109673843	109674124
NA		4	139426299	139430884
100507291		3	139389761	139782699
NA		12	1380060	1391502
643036		4	184798296	184799046
389273	LINC02213	5	10504996	10522084
100506229	LINC01093	4	184892858	184899553
NA		5	94788789	94793599
NA		5	177856262	177871227
NA		4	39066974	39182258
NA		4	16178939	16183120
NA	PDLIM1P4	3	98782188	98783193
NA		4	153720327	153727722
NA		5	111265809	111270089

NA		6	53503109	53507660
101929412	LINC02212	5	10493291	10502728
NA		4	120639090	120650796
729288	ZNF286B	17	18658429	18682228
NA	RBBP4P1	5	14797125	14798400
NA		4	17586267	17614585
100129917		4	760202	781859
NA	TNRC18P1	4	140641840	140645489
102723526	LINC02218	5	17444010	17486829
106144599	LINC01259	4	38509767	38518056
NA		4	150579089	150581545
100506564	THEGL	4	56530606	56603507
163050	ZNF564	19	12525373	12551542
100507388	PARM1-AS1	4	74955974	74970362
677770	SCARNA22	4	1974636	1974760
NA		12	8788253	8795789
NA		4	142566019	142660950
101929215		5	80052374	80083654
105374104	LINC02021	3	130111669	130201336
NA	EEF1A1P19	5	43495073	43496454
NA	NCOA4P2	4	88508591	88510403
25907	TMEM158	3	45224466	45226287
NA	HMGB1P3	2	230515023	230515638
NA		9	112581779	112582590
100506142	RHOQ-AS1	2	46568256	46580238
NA		5	36666214	36725195
NA	EEF1A1P13	5	14651941	14653329
NA		12	65281657	65286728
NA	NCAPGP1	8	120635558	120638204
389203	SMIM20	4	25861830	25929874
NA		22	30653877	30654814
2994	GYPB	4	143996104	144019380
101927124		14	31420286	31452883
27239	GPR162	12	6821624	6829972
NA	KRT8P33	5	123400922	123402344
90423	ATP6V1E2	2	46490750	46542577
NA	NTAN1P2	8	86481754	86483002
339874		3	131325092	131381656
NA		11	1734821	1763954
NA	NAIPP1	5	70462244	70479215
NA		5	32103445	32121941
100419170		4	153640168	153681180
NA		5	288833	290321

121952	METTL21EP	13	102880099	102896033
105370401		14	22197446	22482959
NA		8	22481588	22553444
NA		4	184813619	184821300
100128494		11	72014291	72020910
NA	MTND5P5	4	101972423	101972798
NA		11	35212550	35214007
NA	MIR3945HG	4	184844554	184855751
25850	ZNF345	19	36850361	36913029
NA		4	105137280	105140619
644974	ALG1L2	3	130081831	130113227
NA	LINC02384	12	68332888	68451663
NA		4	101976894	102036903
NA	HSPD1P11	5	95768999	95770700
162972	ZNF550	19	57535257	57559863
NA		5	157363382	157365501
NA	IGKV1D-39	2	89862482	89862981
378938	MALAT1	11	65497688	65506516
NA		11	318640	325631
NA	RNU6-638P	17	78696694	78696797
NA	RNU6-1016P	6	75644084	75644177
NA		5	65961183	65961319
NA		8	66112667	66126632
NA		8	141124717	141130064
NA		8	81149107	81165266
168455	CCDC71L	7	106654360	106661158
NA		12	19147074	19154659
NA	SUMO2P19	8	102242040	102242619
NA		8	122807746	122816517
NA	LINC00534	8	90221341	90687863
NA		8	23483016	23483534
NA		8	24295814	24912073
NA	FAM86HP	3	130099092	130111472
NA	CA3-AS1	8	85440596	85464915
NA	IGLV3-13	22	22762294	22762516
NA		8	73052178	73063061
552889	ATXN7L3B	12	74537835	74545430
5591	PRKDC	8	47773111	47960178
NA		8	27732915	27758552
NA	VPS13B-DT	8	98958277	99013743
NA		5	157199242	157224380
NA		8	1761054	1764508
NA		8	23493009	23494198

NA		8	64801236	64817573
NA	IGHEP2	9	5113549	5114804
103164619	CASC19	8	127072694	127227541
NA	LINC01485	5	173778527	173809039
105375624		8	86333274	86343429
101929415		8	56445807	56556513
101927003	PKIA-AS1	8	78268637	78558503
NA		8	22492631	22493044
101929719		5	140157319	140173051
100049587	SIGLEC14	19	51642553	51646801
NA	PPIAP41	11	43466392	43466871
100133315		11	71865509	71928654
91056	AP5B1	11	65773898	65780976
NA		11	59752578	59754975
NA		19	17417031	17418288
NA		11	10302657	10303704
NA		11	6488186	6489377
728975		11	65177606	65181834
NA	SMG1P6	16	29425800	29447026
NA	WAC-AS1	10	28522652	28533066
NA		11	78388061	78392405
8790	FPGT	1	74198212	74234086
102724957		11	15605484	15705376
NA	CASP1P2	11	105063345	105071541
387751	GVINP1	11	6713536	6746439
84769	MPV17L2	19	18193218	18196948
100499484	SUGT1P4-ST	9	97238449	97297314
NA		11	310139	311141
105376569	LINC02751	11	15552855	15604169
NA	RSF1-IT2	11	77717712	77718411
NA	STX16-NPEPI	20	58651434	58715410
404734	ANKHD1-EIF4	5	140401908	140549569
55845	BRK1	3	10115675	10127190
NA		11	287305	288987
NA		11	68050740	68053762
112268389	FAM66D	8	12115767	12177550
NA		11	36425447	36426122
NA		11	9242448	9245509
57132	CHMP1B	18	11851413	11854444
NA		11	59565923	59639861
100287520	GLYATL1B	11	59086307	59094786
NA	HSPE1P18	11	118208932	118209211
NA	DDX18P5	11	130295641	130296946

440068	CARD17	11	105092469	105101431
283194		11	58933643	59058659
23741	EID1	15	48878134	48880173
NA		11	327171	330122
101927708		11	3481520	3581211
259289	TAS2R43	12	11091287	11092313
NA		11	118510273	118531094
NA	CD44-AS1	11	35210343	35214985
NA		11	38498995	38500186
81343	OR10V2P	11	59748811	59749574
NA		11	65745729	65771585
100885789	IFNG-AS1	12	67989445	68234686
NA	LINC02422	12	31876969	31959316
NA	VDAC2P2	12	9036344	9037244
NA		12	8548361	8567613
497189	TIFAB	5	135444226	135452351
NA		12	9867027	9869808
101927901	LINC02421	12	67709047	67729475
NA	LINC02824	12	126688341	126720333
84334	COA8	14	103562960	103607523
3	A2MP1	12	9228533	9275817
NA		12	66251745	66257434
NA	LINC00944	12	126729787	126772519
7556	ZNF10	12	133130575	133159465
90649	ZNF486	19	20167214	20200488
85027	SMIM3	5	150777946	150796734
3145	HMBS	11	119084866	119093549
7768	ZNF225	19	44112181	44134822
NA		12	68828118	68828553
NA	HSPA8P5	12	4097451	4099519
NA	LINC02390	12	9704077	9709350
374443		12	9648047	9658412
387837	CLEC12B	12	10010627	10018796
NA	KLRA1P	12	10588063	10599669
59348	ZNF350	19	51964340	51986856
NA	BTG1P1	12	9135084	9135591
NA	PLBD1-AS1	12	14567393	14624191
388327	C17orf100	17	6651762	6693202
84290	CAPNS2	16	55566684	55567687
NA	TPT1P12	12	9155881	9156372
105369609	PARP11-AS1	12	3871579	3910382
NA	KRT17P8	12	9127783	9128645
NA		11	114210616	114356571

NA		12	31953889	31955268
3240	HP	16	72054505	72061055
NA		12	9658567	9662085
NA		11	60906789	60909742
57189	DENND11	7	141656728	141702166
100506606		12	29277955	29317848
NA		2	143765940	143776119
256021	LINC01619	12	91984976	92142914
NA		12	29142969	29158537
10778	ZNF271P	18	35290282	35328390
NA		2	143674315	143741294
100506551		12	116977442	116987337
NA		2	143162078	143172172
101926933		14	22929607	22956374
NA		12	93003415	93215679
2342	FNTB	14	64986895	65062652
NA		12	42431665	42433357
NA		12	47706083	47742294
NA		12	112907628	113017751
100127967	KRT73-AS1	12	52601467	52615305
101055625	OVCH1-AS1	12	29389289	29487488
255783	INAFM1	19	47274885	47275723
NA		12	71849228	71850428
NA		12	95996521	96011489
NA		12	62139999	62145643
1523	CUX1	7	101815904	102283958
NA		12	47237734	47279021
NA		12	92858079	92859273
NA		1	234979647	234980804
NA		12	49232790	49264756
643246	MAP1LC3B2	12	116548105	116576606
NA	MKRN9P	12	87782163	87784568
101929162		12	107736555	107759968
23601	CLEC5A	7	141927357	141947007
91612	CHURC1	14	64914361	64944591
NA		12	89561129	89594878
NA		12	55716036	55724703
NA	KLF17P1	12	62234390	62235515
101927267		12	49292631	49324576
NA	LINC02207	15	93835490	93878822
NA	ASB9P1	15	92795647	92796439
112268124		14	34874343	34876459
NA		14	89349377	89366081

105369203	LINC01579	15	93718542	94070898
NA		14	20783888	20784293
NA		15	93882082	93886743
90799	CEP95	17	64506865	64542461
NA		14	61535385	61545484
NA	KRT18P7	14	70594552	70595770
29018	FOXN3-AS2	14	89576216	89577477
NA		14	39385404	39387102
102723809		14	64513952	64540368
NA	CAP2P1	14	89290205	89291594
NA	DDX18P1	14	69083398	69085389
NA		15	101168530	101170821
NA		15	49877299	49883525
NA	PRKXP1	15	100553529	100558954
NA		15	58587507	58591676
109729181	ZNF710-AS1	15	90074512	90082207
NA		14	75423683	75427741
NA		15	38502853	38504981
NA	TMC3-AS1	15	81324338	81518200
NA		15	99805793	99916818
NA		8	29055935	29056685
NA		15	51752485	51756475
NA		15	100645434	100646655
NA		15	45041716	45058707
NA	LINC02352	15	31215622	31224445
NA		15	98954149	99105824
NA		15	48312353	48331856
NA	CYCSP38	15	60558080	60558401
NA		15	61834682	61935738
NA	TYRO3P	15	76258986	76261690
NA		15	60763593	60765625
NA		7	77043721	77198626
NA		15	85415228	85415633
NA		15	51859185	51861293
NA	ST20-AS1	15	79922771	79926993
100506686	IQCH-AS1	15	67290636	67521844
NA	LINC01220	14	75294404	75298109
NA	HSP90B2P	15	99257632	99260015
105370683	LINC00677	14	103120847	103123007
NA		16	72665123	72822781
NA	HMGB1P6	15	71164770	71165415
NA		16	29215385	29221040
NA		8	134792020	134798272

NA		1	247187281	247188526
NA		1	40256427	40257967
29890	RBM15B	3	51391285	51397908
NA		4	47840122	47844339
NA		16	53362019	53363678
NA		15	83179182	83439445
NA		17	81228707	81233983
NA		15	68184032	68229718
100506655	LCMT1-AS2	16	25140577	25149032
643802		16	53364982	53373083
NA		6	110477907	110479436
NA		16	53998313	53999969
102724163		16	83789967	83807834
100134229	KDM7A-DT	7	140177184	140179640
NA		3	47379089	47380999
NA		16	50664903	50671639
NA		20	32856621	32858751
NA		3	195913078	195954485
4360	MRC1	10	17809348	17911164
NA		15	92779757	92781492
NA		8	100913247	100914388
NA		11	73238975	73242335
NA		16	29139661	29216706
4500	MT1L	16	56617476	56618818
NA		17	82293716	82294910
NA	ERVK13-1	16	2660348	2682379
NA		16	19476916	19487899
NA		15	52577842	52598709
197187	SNAI3-AS1	16	88663298	88687278
NA		16	47196311	47202429
150967	LINC01963	2	216217045	216220192
NA		16	78895361	78899644
NA		16	68212401	68221671
9236	CCPG1	15	55340032	55408510
NA		4	47431960	47438959
NA	LINC02555	12	40140926	40142876
NA		9	33697459	33700986
NA		1	111431046	111433068
NA		16	8962706	8966990
NA		1	109895973	109897861
NA		16	53298224	53299792
NA		15	56192353	56193262
NA	ZNNT1	8	101166805	101169629

NA		2	231809846	231812352
NA		11	107312132	107316271
101927155	LMO7-AS1	13	75604700	75635994
100507421	TMEM178B	7	141074064	141480380
NA		16	89492017	89504460
83481	EPPK1	8	143857324	143878467
NA		3	131455126	131458598
NA		16	81738248	81767868
NA		16	31361278	31361695
NA		15	66488658	66492109
5175	PECAM1	17	64319415	64413776
NA		16	67549214	67563958
NA	LINC01003	7	152463786	152465549
NA		3	30697661	30699576
NA		16	75458252	75460017
NA		16	1317891	1322845
NA		16	66549280	66551189
NA	DLGAP1-AS5	18	4264602	4295405
NA	CCNYL7	16	23500905	23501630
NA		15	25345633	25347235
NA		15	65766460	65767398
NA		13	75549773	75807120
100506060	SMG1P7	16	70219574	70246612
NA		16	87396704	87400295
8139	GAN	16	81314944	81390809
NA	YBX3P1	16	31568386	31569475
NA		4	173467833	173470041
NA		16	89420075	89422226
NA	H2BC20P	1	149844498	149850937
NA		3	20388249	20390562
NA		19	34811589	34814345
NA		19	27757184	27760849
100287616	LOXL1-AS1	15	73908071	73928248
NA		16	53373479	53385019
NA	MFSD1P1	17	4301372	4302698
NA		16	67929614	67936017
NA	MMP25-AS1	16	3037400	3059370
NA		17	6653464	6655009
NA	DLGAP1-AS2	18	3603000	3608336
NA		16	3076911	3087111
10418	SPON1	11	13962723	14268133
NA		16	31118078	31118747
101928855		17	80801640	80805632

NA		17	46983287	47100323
101927131		16	11465260	11473174
2969	GTF2I	7	74650231	74760692
100294362	RNF213-AS1	17	80351828	80415168
NA	RYKP1	17	4222091	4223828
NA		17	5425139	5432876
NA		16	68224713	68227734
NA	TAPT1-AS1	4	16226685	16320140
NA		17	38903704	38904600
NA	ANTXRLP1	10	46233885	46273557
23666	UBBP4	17	22090743	22205154
NA	CHORDC1P4	18	2945550	2946890
NA	RN7SL4P	3	15738515	15738809
NA		17	47603860	47649420
NA		18	9112404	9115877
200030	NBPF11	1	148102046	148152322
NA		17	28861072	28861966
NA		17	57989039	57994850
NA	MAGOH2P	17	10716002	10716511
NA	LINC00909	18	74590317	74598885
NA		17	30971652	30973312
100422997	MIR4257	1	150551929	150552014
NA	PRKCA-AS1	17	66398069	66416854
100847051	MIR5194	8	130008334	130008453
NA	KRT8P34	17	39835037	39836080
100422896	MIR3140	4	152489327	152489416
100616157	MIR4772	2	102432289	102432366
102724532		17	47891229	47895812
NA		18	24113637	24121003
NA		15	64696622	64696861
NA	SNORD3B-1	17	19061912	19062669
NA		9	21858910	21861926
NA		18	24076794	24099320
NA		17	28670054	28672804
NA		17	58330884	58332508
NA		18	76884935	76888724
NA	ARGFXP2	17	32150433	32151504
NA		17	64750420	64751311
100287932	TIMM23	10	45972489	46003742
100847048	MIR5690	6	35664717	35664789
NA	FCGR1CP	1	143874793	143883575
101929693	RARA-AS1	17	40340867	40343136
NA		17	29775747	29776701

NA	RN7SL5P	9	9442060	9442380
9554	SEC22B	1	120150898	120176520
100422841	MIR3174	15	90006755	90006841
NA		18	2948238	2960756
10628	TXNIP	1	145992435	145996579
NA		17	64778842	64778937
100616194	MIR4477B	9	63819574	63819654
23380	SRGAP2	1	206203345	206464443
NA		18	2688564	2833065
101927275	NDUFV2-AS1	18	9121265	9136645
NA	ZNF415P1	18	9785108	9787805
NA		8	103383078	103383854
284565	NBPF15	1	144421386	144461674
NA		17	14327335	14329474
NA		17	8965896	8967070
105371967		18	3653030	3656282
645158		18	2652170	2655395
NA		17	17235433	17236118
80022	MYO15B	17	75588058	75626849
NA		18	9506242	9509726
NA		19	30037423	30038181
NA	ACTBP9	18	62442028	62443126
NA		18	35595901	35597599
100128568		19	5978403	6020363
NA		19	30028741	30029916
NA		17	68413623	68524949
NA	LRRC37A16P	17	68125777	68152468
NA		17	35409602	35410228
NA	ZNF561-AS1	19	9621249	9647203
339192		17	45190931	45222222
NA	RNF157-AS1	17	76138432	76154650
NA		18	13514520	13522888
NA	SNRPGP4	17	69318733	69318961
284408	ZNF790-AS1	19	36797502	36831596
NA		19	6067953	6077119
100130522	PARD6G-AS1	18	80147924	80179839
NA		18	61585746	61607006
NA		18	46028306	46029829
NA		17	35459481	35460550
NA	SNHG30	17	35568076	35574900
148145	LINC00906	19	28883430	28970874
400617	KCNJ2-AS1	17	70166961	70169402
NA		18	13500641	13501289

647946	MIR924HG	18	39206917	39800322
100507433	ZNF571-AS1	19	37548914	37587348
NA		19	19788755	19790531
NA		17	78903263	78905024
NA		18	13561399	13565035
NA		18	13471012	13472709
NA		17	76545668	76557683
NA		17	35403837	35404373
NA		18	62300036	62300998
101927151		19	27793431	27984984
NA		17	42509784	42511519
NA		19	37091341	37092564
7767	ZNF224	19	44094339	44109886
NA		19	37337236	37337743
NA		18	13461020	13470823
NA		18	13486462	13490676
100506374	EDDM13	19	56272748	56310454
NA		18	47105946	47108062
NA		17	68189884	68192802
NA		17	35406684	35409768
NA	RUNDC3A-AS	17	44299574	44315315
NA		19	567210	572228
400685	LINC01801	19	34788527	34832869
55957	LIN37	19	35748361	35754519
NA		19	23817599	23874701
NA		19	23323968	23329101
100505812	CARD8-AS1	19	48255675	48258199
390937	ERFL	19	41907705	41928516
149013	NBPF12	1	146938744	146996202
NA		19	21444241	21463908
NA		19	57304305	57308562
NA		19	21587432	21594628
NA		19	21637974	21656300
NA	BNIP3P25	19	21318161	21318719
NA		19	24033401	24077377
105372482		19	58257270	58278808
NA		19	21569738	21587322
NA		19	48204071	48214751
326342	ADGRE4P	19	6952500	6997872
NA		19	41757898	41786893
NA		19	58428632	58431148
NA		19	53197111	53214522
NA		19	16633797	16635269

NA		19	34837889	34872479
200298	LINC00528	22	17777322	17779481
NA		19	39341773	39341945
8517	IKBKG	X	154541199	154565046
100293516	ZNF587B	19	57819721	57846238
NA		12	48198357	48230275
NA		19	52300693	52345229
NA		11	58611119	58612642
400818	NBPF9	1	149054027	149103561
10984	KCNQ1OT1	11	2608328	2699994
NA	ZNF528-AS1	19	52388842	52397783
NA	IPO5P1	19	23255053	23257939
NA		3	169769649	169772043
NA		6	106100140	106100593
NA		8	47527397	47528148
NA		12	123968023	123968579
NA		3	9958717	9962539
101927972		6	5451683	5458075
NA		12	123966077	123966629
NA		X	49273054	49275768
NA		11	325703	326294
101927837		16	67481314	67505977
NA		12	123969990	123970344
NA		8	97144170	97144723
NA		14	103687576	103688127
NA		21	37100814	37101343
NA		12	123960717	123961244
101927950		6	5664985	5695272
728841	NBPF8	1	120419850	120467739
NA	BNIP3P10	19	19870723	19871299
NA		2	178548884	178550681
NA		7	7735743	7736851
767846	PFN1P2	1	120432204	120434052
NA	KNOP1P2	12	45880950	45882266
NA		2	178578790	178580906
NA		1	220207618	220208282
8148	TAF15	17	35713791	35864615
NA		2	61471188	61484130
NA	SRGAP2D	1	143975087	144068350
339929	LPP-AS2	3	188151206	188154057
NA		11	333192	333688
NA	HMGB1P51	15	58672296	58672933
NA		7	150676833	150677918

NA	CTHRC1P1	X	79177200	79177668
NA		19	34849046	34860747
NA	ARMCX5-GPI	X	102599512	102714671
NA		7	130930209	130932206
140809	SRXN1	20	646615	653200
NA	DPRXP2	6	35989515	35991374
101060226	NBPF19	1	149475338	149556361
NA		14	39370279	39370756
NA		2	111210995	111212476
128077	LIX1L	1	145933423	145958017
284021	MILR1	17	64449037	64468643
NA	ATP2B1-AS1	12	89708959	89712590
NA		4	184474802	184477304
NA		15	81303215	81309391
NA	LINC02772	1	157273760	157283968
NA		6	111297126	111298510
101929623	LINC01215	3	108125821	108138610
NA		6	45421079	45422005
NA		8	30155830	30156232
NA		3	3152942	3153435
NA		2	6905724	6906301
NA		3	16314439	16314987
NA		8	67343975	67345087
NA		3	53858994	53861576
NA		6	87151159	87155285
NA		2	37208875	37212677
NA		16	3006120	3007388
NA		8	38382364	38383461
NA		5	96803688	96806105
NA		8	65714334	65714778
723790	H2AC19	1	149851061	149851624
NA		2	196151263	196154881
NA		1	58812808	58813342
NA		8	97132835	97133379
NA		6	3068045	3068894
101929243		6	34696317	34697470
NA		6	57908560	57913911
NA		6	3311662	3313650
9757	KMT2B	19	35717973	35738878
NA		5	44826076	44828592
NA		6	111309203	111313517
NA		6	35220370	35224630
100133941	CD24	6	106969831	106975627

NA		6	24706747	24707151
NA		6	25992641	26001775
NA		1	48227888	48229561
NA		3	16339308	16339871
NA		3	16345126	16346440
NA		1	226045561	226061898
100506497		7	28979967	29013367
152687	ZNF595	4	53286	88208
84740	AFAP1-AS1	4	7754077	7778928
8447	DOC2B	17	142789	181650
105373098	KLHDC7B-DT	22	50542305	50542906
NA		22	38742625	38743115
NA		3	179405448	179405944
NA		2	71067519	71068125
NA		3	122416207	122443180
NA		2	183178806	183215414
55802	DCP1A	3	53283429	53347586
NA		7	135168403	135169547
NA		6	166999405	167139141
NA		2	105334027	105337475
100129550	LINC02035	3	122886941	122892416
NA		22	38736610	38736792
NA		4	82344876	82346842
NA		4	1167778	1168174
NA		22	50191724	50192402
NA		2	73750256	73750786
NA		4	139411927	139454034
NA		22	50199090	50200837
NA		22	50541108	50543011
NA		2	61199979	61200769
NA		7	130936464	130939661
NA		7	129604548	129611630
202781	PAXIP1-AS1	7	155003448	155005703
NA		1	110370154	110373003
NA		2	87477495	87478034
NA		4	109692004	109692703
NA		4	163529771	163530697
NA		4	140756528	140757921
NA		7	131493964	131497694
NA		17	39566915	39567559
9326	ZNHIT3	17	36486629	36499310
NA		15	90024955	90026003
8342	H2BC14	6	27815022	27815489

8339	H2BC8		6	26215159	26216692
NA			20	62596732	62603355
NA			15	45702640	45703183
8355	H3C8		6	26269405	26271815
NA			10	69098496	69098794
NA			9	98115885	98116135
NA		X		135297046	135298585
729438	CASTOR2		7	74964705	75031528
256302	NATD1		17	21238870	21253410
NA	RNVU1-27		1	148522601	148522765
30837	SOCS7		17	38351844	38405593
NA			17	56914186	56914533
NA			13	50125816	50128463
8344	H2BC6		6	26172059	26184655
NA			12	121800797	121803403
NA			7	35847038	35847426
NA			14	70556206	70557478
NA	MIR223HG	X		66015461	66020422
NA			12	116948738	116951422
NA			17	68131462	68131907
NA			13	111182111	111182692
8348	H2BC17		6	27893425	27893891
8353	H3C6		6	26224199	26227473
NA			13	112686769	112689815
NA	RN7SL600P		1	150568971	150569269
85235	H2AC12		6	27147106	27147562
11276	SYNRG		17	37514807	37609472
79873	NUDT18		8	22106874	22109419
7549	ZNF2		2	95165432	95184317
8368	H4C13		6	27873148	27873534
NA			1	120489860	120701289
NA			3	25618132	25618312
NA	LINC02340		13	27178263	27251288
6351	CCL4		17	36103827	36105621
8354	H3C11		6	27871845	27872346
NA			12	29277397	29277882
NA			12	46388856	46392126
NA			15	49343608	49344254
NA			20	3921279	3923400
8345	H2BC9		6	26251651	26253710
8350	H3C1		6	26020451	26020958
85376	RIMBP3		22	18605815	18611919
57636	ARHGAP23		17	38419280	38512385

NA		6	26602733	26606661
NA		12	62393607	62393880
NA		17	82244770	82245591
NA		13	19863858	19865048
NA	STMN1P1	12	31715342	31715781
NA		12	32000375	32001222
6029	RN7SL1	14	49586580	49586878
8294	H4C9	6	27138588	27139881
102723475	KCNE1B	21	7816675	7829926
NA		20	62651272	62652186
NA		2	32521927	32523547
8331	H2AC14	6	27814302	27814777
3018	H2BC3	6	26043227	26043713
NA		19	57449689	57453011
NA		2	32526504	32529507
NA		16	14150833	14153235
NA		20	37526642	37527060
NA		10	147820	148751
NA		13	33846190	33850825
NA	TRAJ36	14	22505110	22505167
NA	IGHV4-4	14	106011922	106012420
NA		13	95744726	95745765
NA		12	31729117	31731204
8332	H2AC16	6	27865317	27865798
NA		16	968375	969012
102466865	MIR7848	8	133046481	133046581
NA		1	219270774	219273387
NA	STAG3L2	7	74843754	74890610
3012	H2AC8	6	26216975	26217483
441250	TYW1B	7	72558744	72828200
NA		13	32782874	32788178
8360	H4C4	6	26188710	26189112
8343	H2BC7	6	26199520	26200715
101929698		20	32509959	32520285
4082	MARCKS	6	113857345	113863475
NA	RN7SL473P	1	150566562	150566860
342372	PKD1L3	16	71929538	71999978
NA		20	62648961	62650767
NA		17	32127595	32128454
NA		14	56117316	56117990
8968	H3C7	6	26250142	26250635
NA		12	64451591	64452901
NA		18	62525919	62526325

11056	DDX52	17	37609739	37643446
6753	SSTR3	22	37204237	37212477
NA	IGLV2-8	22	22822658	22823289
NA		20	48821688	48849458
NA		13	52454775	52455331
NA		8	128009551	128011938
100507240	VWA8-AS1	13	41955808	41981565
NA		16	4532216	4533670
8335	H2AC4	6	26033092	26033618
NA		15	58732188	58733149
101060146	CHMP1B2P	X	80228489	80335364
31	ACACA	17	37084992	37406836
8346	H2BC10	6	26272931	26273412
8359	H4C1	6	26021649	26022050
NA	TRAJ37	14	22503750	22503814
NA		17	68126666	68129586
NA	BACE1-AS	11	117288453	117293571
378707	RN7SL3	14	49853616	49853914
100289211	LINC00624	1	147258885	147517875
8357	H3C10	6	27810064	27811300
NA	IGKV1D-12	2	90159840	90160335
NA		17	17181504	17183257
NA		22	49933198	49934074
NA		19	11856032	11858302
NA		17	40348049	40350539
NA		2	112188364	112190635
100859930	HEIH	5	180826871	180831605
NA		17	69433084	69435533
NA		15	77063397	77064910
NA		2	136230753	136233245
NA		17	54956760	54958426
NA	LINC01727	20	21569976	21666621
NA		17	51183191	51186403
NA		6	49721931	49724007
NA		16	78550739	78553296
NA		15	55171378	55176739
NA		12	100026248	100027540
100130890	TSTD3	6	99520976	99589899
NA		22	44102779	44116232
NA		16	24098239	24099971
NA		17	58095672	58101479
NA		15	78906127	78906809
NA		18	62551203	62552505

NA		7	127349833	127350293
NA		3	108032456	108039916
NA		22	22264601	22273020
NA		17	57448218	57450291
NA		12	131896500	131898239
NA	PRAL	17	6772831	6776116
NA		18	44986109	44989203
NA		3	146504570	146506560
NA		17	78897264	78900152
NA		11	124789240	124792818
NA		16	53478957	53481550
NA		2	216211404	216213519
NA		16	50072862	50074986
NA		17	67637774	67638973
NA		5	36667717	36669958
NA		19	24078481	24080669
NA		18	77108284	77110537
NA		14	35086035	35088059
102723897	WASH9P	1	185217	195411
NA		14	105370418	105372918
NA		12	70321542	70324274
NA		19	57293083	57294469
NA		2	32563328	32566436
NA		17	57277920	57280017
NA		17	78691266	78691979
NA		22	49534062	49534926
NA		2	178554561	178558966
NA		6	87356831	87358605
NA		18	79420836	79422780
NA		16	68290087	68292790
NA		16	10445309	10449747
NA		2	16541690	16545695
NA		5	143375628	143375883
NA		16	53487607	53489943
105377031	LINC02033	3	36819276	36822498
NA		16	53389886	53390563
NA		18	77002849	77006846
NA		10	22693953	22694639
NA		5	177476631	177479656
NA		16	81630193	81632038
NA		19	47487638	47489519
NA		17	67973934	67976072
NA		16	2683106	2684764

NA		4	99844138	99844696
NA		19	46946535	46949156
NA		18	79496058	79498348
NA		5	61114432	61116753
NA		22	18110759	18131154
NA		16	53391089	53392473
NA		17	32159311	32160371
NA		17	7858943	7866083
NA		12	47728151	47730598
642757	TBC1D22A-A	22	46914092	46916042
NA		19	6894994	6896832
NA		19	34949038	34951205
191585	PLAC4	21	41175231	41186788
NA		5	54643557	54645987
NA		3	47346950	47349073
NA		12	64709458	64710513
NA		16	78890231	78892302
NA		17	80339898	80342058
NA		3	187383101	187384849
NA	FRG1KP	9	65646982	65652679
NA		7	127359785	127364759
NA		13	73800074	73802203
NA		12	123087256	123092821
NA		22	45657019	45680130
NA		7	76043977	76045963
NA		16	53991012	53994454
NA		17	82249067	82251844
100861563	SCAANT1	3	63911518	63911772
359822	KCNIP4-IT1	4	21843341	21853188
440952	EIF1B-AS1	3	40061110	40309698
79447	PAGR1	16	29816152	29822489
283267	LINC00294	11	33076149	33079454
NA	GSEC	11	126340889	126355587
101805488	CCAT2	8	127400399	127402150
NA		4	386174	454070
NA		3	36823151	36825158
283521	TMEM272	13	51813347	51845177
101243555	PTENP1-AS	9	33676785	33688011
NA	LINC01127	2	101962056	101987167
NA		2	71373938	71376320
NA	HELLPAR	12	102197585	102402596
100129716	ARRDC3-AS1	5	91380349	91612566
103021294	ABALON	20	31721507	31723409

NA			6	39818751	39823227
NA	IGHV1-69-2		14	106737110	106737547
359809	PEG13		8	140094894	140100543
100033411	DUXB		16	75693893	75701461
NA			11	95203727	95204033
105221694	BISPR		19	17405686	17419324
NA			4	47831330	47897965
NA			3	101457241	101457732
NA			6	26195595	26199293
NA			7	133034607	133035920
101926918	GTF2IP12		4	119405523	119454638
NA			6	26160765	26172802
NA			13	108335354	108448316
NA			16	20711473	20732990
NA			1	58715609	58771295
NA			5	140569962	140594570
105377068	LINC02009		3	46416524	46423591
729732			8	12467693	12665588
NA			2	143601517	143608759
51182			10	14838166	14847018
100500853	MIR3605		1	33332393	33332492
NA	MTATP6P5		2	143099936	143100612
9997	SCO2		22	50523568	50526461
NA			19	23259906	23274251
NA			16	75660227	75677009
NA			3	39148281	39172952
NA			7	97851688	97972985
107984345	SMIM38		11	69155478	69159752
NA			6	4714098	4724885
NA			6	167114957	167121527
NA			2	87455476	87767359
NA			1	84574129	84583620
NA			4	86924630	86936202
NA			9	137217452	137219361
NA			9	117759455	117879988
NA		X		135327758	135330743
NA			7	50388489	50400097
NA	LINC02795		1	86288704	86320711
NA			1	120963645	120977585
NA			16	20720994	20722281
NA	NA	NA		NA	NA
55056	GABPB1-IT1		15	50348936	50354861
NA			17	44276368	44281182

112267968			6	159051674	159121495
NA	TBCEL-TECTA		11	121024125	121113108
105369347	RELA-DT		11	65663006	65671716
NA			13	109478739	109731700
NA			3	148368384	148591927
NA			11	1464582	1467632
NA			1	11907940	11914298
NA			1	151885251	151899333
NA			15	84717474	84746837
NA		X		7928443	8374386
NA			9	7177577	7203458
NA			11	72793624	72814054
NA			12	865288	867139
NA			7	97966377	97970404
NA			1	42658687	42682160
NA		X		3817528	3937855
NA			16	84817182	84856665
NA			2	61527340	61529160
NA			5	157341596	157460100
NA			3	11193439	11225877
NA			6	63572472	63583587
NA			10	12563151	12567351
NA			5	66085405	66093937
NA			3	178028130	178172449
NA			8	127663280	127670990
NA			2	197817423	197836326
NA			4	144010237	144012598
105373170			1	231767464	231847706
NA			7	106425278	106511544
NA			10	88990045	88994249
NA			21	34836286	34884882
NA			20	48657073	48659119
728392			17	5499427	5501006
100996717	NOTCH2NLC		1	149390614	149468030
NA			18	79185801	79191559
NA			4	184826607	184850920
NA			7	98989867	98993778
NA			6	135854053	135880942
NA			6	13671720	13678908
NA		X		102659983	102663455
NA			13	97278768	97295400
NA			6	118565660	118576840
NA			7	64947571	64950701

NA		5	133051065	133051893
NA		X	3819924	3823898
NA		15	60634503	60640915
NA		11	114159791	114161166
NA		15	81315806	81321258
8358	H3C2	6	26031589	26032099
NA		2	112239621	112244375
NA		6	111505307	111511071
NA		6	25012985	25015696
NA		5	177260340	177263909
NA		6	136784045	136786280
NA		1	184080657	184149652
NA		7	50641725	50643868
NA		5	81408517	81411867
NA		5	5390096	5422116
NA		13	99841879	99957167
100289495		6	166342632	166351466
NA		15	38493308	38499985
NA		2	43128819	43131999
NA		5	97183827	97216074
NA		15	83095333	83108754
NA		16	78793584	78796362
NA		2	212932894	212937520
NA		5	14728587	14734258
NA		12	19582317	19583274
8352	H3C3	6	26045384	26045869
NA		3	10149986	10153326
NA		4	2859983	2862673
NA		8	13147389	13207125
NA		2	112061084	112063709
NA		8	23492170	23494897
NA		9	62801534	62802699
NA		8	25179113	25184234
NA		4	168170097	168194861
NA		12	108532074	108539738
NA		8	67313043	67314637
NA		4	153633692	153655689
NA		10	98446346	98453805
NA		5	42983636	42993276
NA		6	285443	292013
NA		4	148445703	149024624
NA		2	200390666	200478018
NA		9	27495857	27498251

NA	4	14359400	14453625
NA	3	16361348	16370398
NA	17	80824170	80826900
NA	11	129761713	129814690
NA	17	81339183	81345966
NA	12	96229563	96235650
NA	6	35650997	35652856
NA	10	129621988	129626664
NA	8	22545560	22548837
NA	7	27873785	27876546
NA	15	60642054	60659791
NA	15	74040190	74043332
NA	7	121304657	121309842
NA	4	152448275	152513788
NA	6	44090787	44095585
NA	7	64801958	64804117
NA	1	4730211	4734992
NA	10	20095125	20099664
NA	5	147725551	147728464
729870	4	152934516	152936761
NA	17	40317698	40318676
NA	1	77431314	77437604
NA	3	59809269	59811310
NA	7	4725440	4728721
NA	6	61870097	61893882
NA	12	7838914	7844395
NA	3	3918709	4046722
NA	11	69072915	69162440
NA	1	10612222	10616452
NA	7	135170816	135172414
100302522	8	133054959	133057767
NA	17	82273748	82279645
NA	4	11426874	11467802
NA	6	90073051	90079734
NA	22	31490150	31491022
NA	13	40874685	40877861
NA	1	155626757	155637604
NA	7	6952625	7104482
NA	3	9693182	9721399
NA	14	68731234	68736678
NA	3	156143570	156540627
NA	10	35443411	35444569
NA	8	47941426	47943670

NA	4	173397770	173409559
NA	6	6725036	6733191
NA	3	16444540	16447914
NA	6	135882780	135888133
NA	2	151796688	151798688
NA	4	9034519	9381269
NA	1	161399998	161401868
NA	11	111320379	111323816
NA	19	2293173	2295781

	baseMean	log2FoldChai	lfcSE	stat	pvalue	padj
ENSG00000C	1123.22933	0.56002563	0.10203171	5.4887411	4.05E-08	1.30E-06
ENSG00000C	1327.19905	0.21004728	0.05857421	3.58600295	0.00033579	0.00220914
ENSG00000C	220.922444	0.38233134	0.09473421	4.03583186	5.44E-05	0.0005021
ENSG00000C	2744.597	0.24138317	0.09449927	2.55433885	0.01063897	0.0366532
ENSG00000C	1546.86631	-0.2469094	0.08162849	-3.0247946	0.00248802	0.01117989
ENSG00000C	8640.76609	1.43297425	0.22762493	6.29533082	3.07E-10	2.08E-08
ENSG00000C	5435.89248	0.23076079	0.1220243	1.89110528	0.05861029	0.13563695
ENSG00000C	222.960431	0.78751899	0.12549736	6.27518365	3.49E-10	2.27E-08
ENSG00000C	219.553356	-1.1295333	0.25650837	-4.4034948	1.07E-05	0.00013122
ENSG00000C	791.453398	-0.1708572	0.07512295	-2.274367	0.02294393	0.06681181
ENSG00000C	4397.63876	0.39115847	0.07871692	4.96917906	6.72E-07	1.29E-05
ENSG00000C	42251.0301	0.55287558	0.15298752	3.61386057	0.00030167	0.00202423
ENSG00000C	662.614685	0.13171021	0.07144889	1.84341853	0.06526793	0.14705077
ENSG00000C	15750.5686	0.20211416	0.06871346	2.94140552	0.00326727	0.0139625
ENSG00000C	1912.03232	0.82061797	0.19379919	4.23437261	2.29E-05	0.00024723
ENSG00000C	1676.91085	-0.3822466	0.09764421	-3.9146877	9.05E-05	0.0007589
ENSG00000C	2178.28493	-0.3339445	0.07312545	-4.5667341	4.95E-06	7.02E-05
ENSG00000C	1450.8767	0.2607442	0.09905454	2.63232972	0.00848015	0.03051464
ENSG00000C	957.869662	0.18773959	0.10539238	1.78133932	0.07485704	0.16241633
ENSG00000C	146.034047	0.77564638	0.21029671	3.68834287	0.00022572	0.00159114
ENSG00000C	1854.40921	0.18515433	0.05825019	3.17860503	0.00147986	0.0074131
ENSG00000C	1442.84177	-0.2233963	0.06348916	-3.5186529	0.00043374	0.00272609
ENSG00000C	4794.65835	-0.3131709	0.0768165	-4.0768698	4.56E-05	0.00043362
ENSG00000C	1507.01451	0.24470957	0.10850904	2.25519994	0.02412078	0.06956754
ENSG00000C	1736.86477	0.16711459	0.06794669	2.45949582	0.01391323	0.0453864
ENSG00000C	227.406816	-0.7165929	0.14487429	-4.9463084	7.56E-07	1.42E-05
ENSG00000C	65.0199869	-0.2697394	0.13378011	-2.0162889	0.04376978	0.10881976
ENSG00000C	10947.8345	0.53822224	0.12698239	4.23855808	2.25E-05	0.00024339
ENSG00000C	1068.41651	1.54627919	0.3491762	4.42836358	9.50E-06	0.00011972
ENSG00000C	99.0666073	-0.514255	0.27906262	-1.8427943	0.06535905	0.14711879
ENSG00000C	4908.06834	0.15851044	0.07833397	2.02352112	0.04301945	0.10757853
ENSG00000C	600.135992	-0.3474565	0.10100998	-3.4398238	0.00058209	0.00345747
ENSG00000C	4197.67371	0.30887628	0.13319581	2.31896394	0.02039699	0.06129252
ENSG00000C	3036.38657	-0.2306758	0.07682671	-3.0025464	0.00267731	0.01187576
ENSG00000C	304.253465	-0.2768862	0.12549579	-2.2063383	0.02736032	0.07641584
ENSG00000C	74.4978592	0.25760781	0.13274618	1.94060432	0.05230629	0.12406045
ENSG00000C	6059.01613	0.36957392	0.13869807	2.66459303	0.00770815	0.02819188
ENSG00000C	226.606267	0.18902091	0.0915438	2.06481381	0.03894062	0.10023681
ENSG00000C	148.528686	-0.286191	0.15420764	-1.855881	0.06347053	0.14403156
ENSG00000C	5522.9366	0.44266294	0.17079874	2.59172242	0.00954968	0.03361181
ENSG00000C	3157.93257	-0.308549	0.11126275	-2.7731563	0.00555154	0.02153863
ENSG00000C	2412.08317	-0.1546098	0.07920256	-1.9520804	0.05092866	0.12150915

ENSG00000(	1635.8567	-0.2526486	0.11254997	-2.2447683	0.02478301	0.07106719
ENSG00000(	227.953352	0.30598493	0.15547936	1.96801	0.04906689	0.1182546
ENSG00000(	1104.15675	-0.2725567	0.11214026	-2.4304986	0.01507806	0.04836933
ENSG00000(	2701.26687	-0.3180235	0.18751891	-1.6959543	0.08989453	0.18606974
ENSG00000(	278.721346	0.1870534	0.05586379	3.34838367	0.00081284	0.00455959
ENSG00000(	314.621103	-0.6475024	0.21697824	-2.9841814	0.00284338	0.01245602
ENSG00000(	2654.89788	-0.1569193	0.07965993	-1.9698645	0.0488539	0.11791843
ENSG00000(	5694.34452	0.27554028	0.14040523	1.96246443	0.04970844	0.1194239
ENSG00000(	46.9244729	0.75700323	0.1904225	3.97538755	7.03E-05	0.00061993
ENSG00000(	4125.43778	0.3394799	0.07678812	4.42099525	9.82E-06	0.00012312
ENSG00000(	42973.4494	0.58633112	0.16838315	3.48212475	0.00049745	0.00305423
ENSG00000(	695.013015	-0.6468117	0.11791219	-5.485537	4.12E-08	1.32E-06
ENSG00000(	2546.05142	-0.2432097	0.06759512	-3.598037	0.00032063	0.00213071
ENSG00000(	117.029893	-0.5589451	0.18198465	-3.071386	0.00213068	0.00988963
ENSG00000(	54.5794457	0.65119032	0.16734331	3.8913437	9.97E-05	0.00082292
ENSG00000(	667.602132	0.38807218	0.09688225	4.00560638	6.19E-05	0.00055661
ENSG00000(	775.937441	0.22970099	0.13535866	1.69698039	0.08970037	0.1857483
ENSG00000(	591.862032	-0.3733801	0.09490204	-3.9343738	8.34E-05	0.00071042
ENSG00000(	1031.6743	-0.4292188	0.20027821	-2.1431126	0.03210406	0.0864161
ENSG00000(	560.181976	-0.1809536	0.10114342	-1.7890788	0.07360213	0.1605351
ENSG00000(	3077.61831	0.20468067	0.08816721	2.32150545	0.02025958	0.06099001
ENSG00000(	4993.92552	0.20933376	0.09429112	2.22007932	0.02641338	0.07440223
ENSG00000(	12717.0244	0.34535264	0.14614878	2.36302106	0.01812664	0.0558051
ENSG00000(	611.900098	0.77196471	0.26761512	2.88460797	0.00391901	0.01615673
ENSG00000(	397.200328	-0.1752412	0.07220359	-2.4270427	0.01522246	0.04866016
ENSG00000(	1902.3344	0.26836276	0.05375315	4.99250286	5.96E-07	1.17E-05
ENSG00000(	3151.72462	-0.1801783	0.07068398	-2.5490682	0.01080112	0.03709715
ENSG00000(	1568.63524	1.94274951	0.34178445	5.68413665	1.31E-08	4.93E-07
ENSG00000(	591.57635	0.13239577	0.06663939	1.98674949	0.04695016	0.11466066
ENSG00000(	9552.84677	0.10436377	0.04456204	2.34198834	0.01918131	0.05845177
ENSG00000(	1448.30608	0.30518703	0.09206356	3.3149601	0.00091656	0.00503192
ENSG00000(	1462.2709	-0.6215922	0.23116322	-2.6889753	0.00716717	0.0265167
ENSG00000(	3133.11722	-0.1484874	0.0781039	-1.9011524	0.05728206	0.13309466
ENSG00000(	5207.16102	0.11473138	0.06519792	1.75973987	0.07845192	0.16792745
ENSG00000(	75.1879905	1.16104332	0.19637176	5.91247587	3.37E-09	1.59E-07
ENSG00000(	8762.9062	0.66498661	0.12895653	5.15667284	2.51E-07	5.73E-06
ENSG00000(	1227.28882	0.26064056	0.1493852	1.74475489	0.08102754	0.17214343
ENSG00000(	2041.78636	0.24013864	0.10533463	2.27976914	0.02262138	0.06604514
ENSG00000(	13520.7819	-0.658507	0.12992368	-5.0684139	4.01E-07	8.36E-06
ENSG00000(	6904.4971	0.19256806	0.08329949	2.31175566	0.02079115	0.06211619
ENSG00000(	589.324178	0.4085899	0.10687352	3.82311634	0.00013178	0.00103527
ENSG00000(	638.128723	-0.3305641	0.08375049	-3.9470104	7.91E-05	0.00068198
ENSG00000(	9710.29639	-0.3557852	0.0838212	-4.244573	2.19E-05	0.00023784

ENSG00000(	1811.69747	-0.1506573	0.05880567	-2.5619512	0.01040859	0.03599649
ENSG00000(	387.215767	-0.1425577	0.08600611	-1.65753	0.09741236	0.19767655
ENSG00000(	20.5233158	1.09978396	0.33925425	3.24176918	0.0011879	0.00621942
ENSG00000(	5623.9045	-0.1782922	0.05614826	-3.1753832	0.00149639	0.00745467
ENSG00000(	9483.57459	-0.447651	0.06419092	-6.9737425	3.09E-12	3.55E-10
ENSG00000(	675.372575	-0.1249401	0.07161767	-1.7445435	0.08106437	0.17219641
ENSG00000(	78.6874879	1.17200148	0.23545695	4.97756155	6.44E-07	1.25E-05
ENSG00000(	1835.71773	-0.132728	0.05290338	-2.5088752	0.01211163	0.04056904
ENSG00000(	1332.63659	-0.1776154	0.08489494	-2.0921793	0.03642248	0.09523233
ENSG00000(	255.155501	0.61699821	0.24556325	2.51258371	0.01198507	0.04025682
ENSG00000(	732.542163	0.85696547	0.13068017	6.55773173	5.46E-11	4.68E-09
ENSG00000(	130.00311	1.09821022	0.18477537	5.94348819	2.79E-09	1.34E-07
ENSG00000(	595.983464	-0.1813716	0.0781111	-2.3219694	0.02023458	0.06093087
ENSG00000(	7839.95777	1.70279672	0.40549819	4.19927087	2.68E-05	0.00028213
ENSG00000(	975.703434	-0.1797232	0.10199876	-1.7620132	0.07806706	0.16727078
ENSG00000(	991.627501	-0.2192156	0.09584845	-2.2871065	0.02218961	0.06516538
ENSG00000(	8779.48558	0.66067649	0.14259278	4.63330964	3.60E-06	5.35E-05
ENSG00000(	338.903507	-0.2631477	0.07081216	-3.7161369	0.00020229	0.00145347
ENSG00000(	1090.77948	-0.2112919	0.08623091	-2.4503033	0.01427359	0.04637397
ENSG00000(	1443.18615	0.28853719	0.07171297	4.02350066	5.73E-05	0.00052277
ENSG00000(	77.6028484	-0.3089824	0.17142088	-1.8024781	0.07147021	0.15711912
ENSG00000(	8960.08246	-0.3447997	0.14272475	-2.4158367	0.0156991	0.04979918
ENSG00000(	10081.3743	-0.1977327	0.11080824	-1.7844587	0.07434916	0.16158131
ENSG00000(	3179.78774	0.33551254	0.08689931	3.86093463	0.00011295	0.00091315
ENSG00000(	1263.98045	0.18538704	0.08070923	2.29697445	0.02162023	0.06397309
ENSG00000(	1627.11749	0.12501455	0.0706865	1.76857756	0.0769644	0.16562437
ENSG00000(	1569.17567	-0.312036	0.0994752	-3.136822	0.0017079	0.00830837
ENSG00000(	1688.88633	-0.1575232	0.07199918	-2.1878466	0.02868078	0.07926334
ENSG00000(	3682.25167	-0.1212226	0.0591185	-2.0505018	0.04031549	0.10279306
ENSG00000(	1630.11984	-0.105792	0.03651992	-2.8968303	0.00376954	0.01568781
ENSG00000(	45361.7332	0.41810211	0.22010103	1.89959179	0.05748671	0.13346508
ENSG00000(	596.115832	0.10831571	0.06545974	1.65469202	0.09798699	0.19848119
ENSG00000(	834.631369	0.6356787	0.21672474	2.93311558	0.00335579	0.01428182
ENSG00000(	433.940751	-0.3311579	0.10539575	-3.1420426	0.00167774	0.00820019
ENSG00000(	146949.79	-0.6619262	0.12517431	-5.2880356	1.24E-07	3.20E-06
ENSG00000(	1309.89282	-0.1523182	0.08141781	-1.8708216	0.06136981	0.1404969
ENSG00000(	441.071557	-0.3145243	0.08791819	-3.5774653	0.00034694	0.00226619
ENSG00000(	188.961267	0.26379662	0.09785998	2.69565388	0.00702506	0.02610879
ENSG00000(	549.91821	1.11219471	0.21628061	5.14236901	2.71E-07	6.08E-06
ENSG00000(	9540.07689	-0.3607135	0.12232482	-2.9488169	0.00318993	0.01372105
ENSG00000(	1024.2388	-0.5685995	0.15135618	-3.7566984	0.00017217	0.00128009
ENSG00000(	18776.8536	0.64529373	0.14274179	4.5207063	6.16E-06	8.37E-05
ENSG00000(	1962.35652	0.23854537	0.10664081	2.23690506	0.02529255	0.07208734

ENSG00000(	626.154349	-0.6701536	0.12455036	-5.3805836	7.42E-08	2.09E-06
ENSG00000(	938.50516	0.27933823	0.07049793	3.96236074	7.42E-05	0.00064766
ENSG00000(	2550.46971	0.06374836	0.03737689	1.70555566	0.08809084	0.18327905
ENSG00000(	2009.00951	0.95867515	0.18331323	5.22971071	1.70E-07	4.15E-06
ENSG00000(	1619.44381	-0.3014944	0.10972789	-2.7476553	0.00600231	0.02290397
ENSG00000(	3480.99512	0.28777678	0.14571615	1.97491341	0.04827798	0.11689838
ENSG00000(	5327.85044	0.13525883	0.08045493	1.68117515	0.0927289	0.190521
ENSG00000(	5953.03252	0.33986571	0.18530581	1.83408017	0.0666421	0.14905419
ENSG00000(	667.244567	0.16178147	0.07128609	2.26946764	0.0232399	0.06749729
ENSG00000(	882.667522	0.14804003	0.05871161	2.52147811	0.01168629	0.03946372
ENSG00000(	344.149155	0.59311709	0.1260444	4.70562021	2.53E-06	4.01E-05
ENSG00000(	12139.7932	0.30730852	0.13285655	2.31308525	0.02071795	0.06197196
ENSG00000(	2738.34138	0.2537736	0.09583324	2.64807495	0.00809516	0.02937739
ENSG00000(	1569.93658	0.61818489	0.15941142	3.87792094	0.00010535	0.00086229
ENSG00000(	595.587437	-0.2515368	0.0983544	-2.5574531	0.01054418	0.03637855
ENSG00000(	3335.1572	-0.1706575	0.08186778	-2.0845507	0.03711011	0.09664705
ENSG00000(	36113.5351	1.03835009	0.15323576	6.77616023	1.23E-11	1.24E-09
ENSG00000(	793.808491	-0.2771867	0.0852415	-3.2517815	0.00114684	0.00604596
ENSG00000(	2036.17699	-0.2009973	0.05842661	-3.4401674	0.00058135	0.0034545
ENSG00000(	3438.03089	-0.0855677	0.03726247	-2.2963519	0.02165577	0.06405209
ENSG00000(	36148.1108	-0.230573	0.13292217	-1.734647	0.08280335	0.17518585
ENSG00000(	161.590906	0.54522034	0.14012952	3.89083133	9.99E-05	0.00082325
ENSG00000(	4288.22861	0.62587811	0.15077486	4.15107723	3.31E-05	0.00033501
ENSG00000(	23827.644	-0.1713437	0.08666359	-1.9771134	0.04802881	0.11652878
ENSG00000(	24164.1739	0.67143332	0.11871944	5.65563063	1.55E-08	5.72E-07
ENSG00000(	5404.97269	-0.2706117	0.0919951	-2.9415882	0.00326534	0.01395838
ENSG00000(	18838.8082	0.59048236	0.16678026	3.54048118	0.0003994	0.00254669
ENSG00000(	1249.92697	-0.1984317	0.09575354	-2.0723167	0.03823591	0.09884433
ENSG00000(	601.362468	0.57566848	0.09584008	6.00655245	1.90E-09	9.47E-08
ENSG00000(	10203.1775	0.10156235	0.05325437	1.9071176	0.05650536	0.1317761
ENSG00000(	1064.9225	0.14193312	0.05667024	2.50454411	0.01226093	0.04099333
ENSG00000(	66.8767454	1.00083435	0.18594366	5.3824603	7.35E-08	2.08E-06
ENSG00000(	5186.10802	-0.1390382	0.07280659	-1.9096923	0.05617285	0.13106394
ENSG00000(	104.337386	0.49376816	0.16915731	2.91898797	0.0035117	0.01480616
ENSG00000(	559.550179	-0.1756845	0.08900425	-1.9738891	0.04839435	0.11712144
ENSG00000(	1819.06243	0.46029442	0.12273487	3.75031489	0.00017661	0.00130807
ENSG00000(	48189.3349	0.30662609	0.16650925	1.84149582	0.06554893	0.14731731
ENSG00000(	110.524735	0.56411436	0.22675692	2.48774924	0.01285543	0.04251979
ENSG00000(	5614.12871	-0.1647296	0.08265588	-1.9929573	0.04626612	0.11339153
ENSG00000(	4705.28481	-0.1457794	0.06010636	-2.4253578	0.01529331	0.04882198
ENSG00000(	3134.11403	0.24699515	0.1212928	2.03635462	0.04171476	0.10515429
ENSG00000(	359.990745	-0.1488313	0.08351796	-1.7820271	0.07474481	0.16228377
ENSG00000(	1886.28978	-0.1257576	0.04198169	-2.9955351	0.00273964	0.01208561

ENSG00000(	664.08429	-0.1688887	0.07871492	-2.1455746	0.03190693	0.08604537
ENSG00000(	5707.20152	0.15272942	0.08404368	1.81726243	0.06917694	0.15321301
ENSG00000(	16952.9714	0.56794706	0.17885104	3.17553115	0.00149562	0.00745467
ENSG00000(	5503.92525	0.23330442	0.10845067	2.15124922	0.03145653	0.08503654
ENSG00000(	6724.69799	0.24597219	0.10898989	2.25683488	0.02401839	0.06932745
ENSG00000(	340.070962	-0.3368808	0.1439247	-2.3406742	0.01924895	0.05857165
ENSG00000(	53615.3432	0.41573211	0.10485924	3.96466823	7.35E-05	0.00064375
ENSG00000(	3211.84143	0.22137352	0.09402096	2.35451232	0.01854703	0.0569743
ENSG00000(	1105.10788	-0.279246	0.13340168	-2.0932724	0.03632485	0.09506292
ENSG00000(	61.4849887	1.02703564	0.25168309	4.08066996	4.49E-05	0.00042828
ENSG00000(	3812.02024	0.27678765	0.09309073	2.97331074	0.00294606	0.01283982
ENSG00000(	2764.10403	-0.107175	0.0565952	-1.8937126	0.05826316	0.13489827
ENSG00000(	530.482214	0.4311115	0.18803175	2.29275906	0.02186188	0.06447507
ENSG00000(	1962.57699	-0.1255014	0.06048737	-2.0748364	0.0380017	0.09846728
ENSG00000(	1997.34385	0.17538754	0.07943601	2.20790981	0.02725056	0.07621214
ENSG00000(	180.107815	-1.0974456	0.4735672	-2.317402	0.02048185	0.06146909
ENSG00000(	96.0364408	0.94332219	0.15683522	6.01473447	1.80E-09	9.06E-08
ENSG00000(	301.850667	-0.72581	0.23908419	-3.0357925	0.00239904	0.01084398
ENSG00000(	3085.77036	-0.19703	0.05659281	-3.4815375	0.00049854	0.00305775
ENSG00000(	2215.38083	-0.1222596	0.05918333	-2.0657777	0.03884948	0.10005553
ENSG00000(	872.379661	0.23454206	0.12333915	1.90160269	0.05722312	0.13302171
ENSG00000(	983.308921	0.67141706	0.15859356	4.23357074	2.30E-05	0.00024793
ENSG00000(	9063.64511	-0.1219763	0.06028096	-2.0234627	0.04302547	0.10757853
ENSG00000(	20058.4934	0.29489909	0.15288002	1.9289577	0.05373611	0.12668431
ENSG00000(	1882.9908	-0.1389099	0.051132	-2.716692	0.00659379	0.0247729
ENSG00000(	2451.32181	0.32421093	0.16087826	2.01525639	0.04387779	0.10903217
ENSG00000(	4333.99614	-0.2551249	0.08889682	-2.8698986	0.00410603	0.01680342
ENSG00000(	3039.866	0.26034925	0.07315796	3.5587274	0.00037266	0.00240475
ENSG00000(	394.660484	-0.292749	0.11078414	-2.6425173	0.00822923	0.02974471
ENSG00000(	1028.62167	-0.1351057	0.06397405	-2.1118824	0.03469654	0.09182477
ENSG00000(	119.403408	-0.5295629	0.17058279	-3.1044335	0.00190644	0.00907014
ENSG00000(	3227.96226	0.78117276	0.1426921	5.47453417	4.39E-08	1.38E-06
ENSG00000(	1810.13302	-0.2106973	0.08607256	-2.4479027	0.01436905	0.04656921
ENSG00000(	163.992535	0.48963962	0.18649038	2.62554887	0.00865094	0.03100169
ENSG00000(	1047.37574	-0.2486268	0.08312045	-2.9911629	0.00277917	0.0122265
ENSG00000(	11593.3119	0.6193384	0.16444098	3.76632647	0.00016567	0.0012416
ENSG00000(	161.270962	0.57970842	0.18956314	3.05812842	0.00222724	0.01023344
ENSG00000(	668.932857	-0.2383835	0.0818739	-2.9115934	0.0035959	0.01509207
ENSG00000(	34.7875806	0.55306239	0.24194031	2.28594562	0.02225744	0.06529703
ENSG00000(	3376.54325	0.17685563	0.09736911	1.81634225	0.06931789	0.1534781
ENSG00000(	170.222935	0.26484648	0.11166109	2.37187793	0.01769794	0.05471404
ENSG00000(	9567.27815	-0.1977594	0.10687994	-1.8502948	0.06427107	0.14543813
ENSG00000(	3569.77076	-0.0998477	0.04469255	-2.2341014	0.0254764	0.07243899

ENSG00000C	282.374937	-0.2047758	0.07301244	-2.8046694	0.00503682	0.01994153
ENSG00000C	1401.89282	-0.2174469	0.08280129	-2.6261294	0.0086362	0.0309608
ENSG00000C	5430.74213	-0.1435607	0.05322023	-2.6974836	0.00698657	0.02599242
ENSG00000C	2684.98186	0.39519777	0.12034458	3.2838852	0.00102387	0.00552019
ENSG00000C	8380.67568	0.15242897	0.09048632	1.68455262	0.09207492	0.18958064
ENSG00000C	2918.18588	0.2570373	0.10112172	2.54186052	0.01102642	0.03768334
ENSG00000C	646.46015	0.28713904	0.09334686	3.07604402	0.00209767	0.00976508
ENSG00000C	3100.43703	-0.2070864	0.09011145	-2.2981141	0.0215553	0.06388535
ENSG00000C	849.352164	-0.2597094	0.12897951	-2.0135709	0.0440546	0.10935898
ENSG00000C	2529.08786	0.19462571	0.09214819	2.11209472	0.03467833	0.09179333
ENSG00000C	169.736782	0.76979078	0.15977134	4.81807801	1.45E-06	2.48E-05
ENSG00000C	3347.81094	0.28144501	0.14570372	1.93162546	0.05340575	0.12609014
ENSG00000C	1491.73922	-0.1067835	0.06073592	-1.7581603	0.07872023	0.16829336
ENSG00000C	310.546644	2.24647992	0.37289968	6.02435465	1.70E-09	8.60E-08
ENSG00000C	1829.49566	-0.2757533	0.08551425	-3.224647	0.00126128	0.00651415
ENSG00000C	3645.01716	0.15273926	0.05800146	2.63336896	0.00845425	0.03044411
ENSG00000C	32158.1776	0.23796476	0.13037327	1.8252573	0.06796221	0.15112334
ENSG00000C	1256.78481	-0.2136261	0.06856987	-3.1154516	0.00183663	0.00881367
ENSG00000C	2361.26375	1.15277035	0.26942869	4.2785732	1.88E-05	0.00020945
ENSG00000C	176.040887	0.2237159	0.1217959	1.83680976	0.06623799	0.14840546
ENSG00000C	4876.01892	0.30455461	0.08133214	3.74457857	0.0001807	0.00132932
ENSG00000C	6257.8328	0.17208177	0.10225102	1.68293461	0.09238775	0.19008967
ENSG00000C	192.920133	-0.3072353	0.1351021	-2.2740973	0.02296014	0.06684556
ENSG00000C	1227.40197	0.30684298	0.08285367	3.70343253	0.0002127	0.00151644
ENSG00000C	3193.09475	-0.1982969	0.06111774	-3.244507	0.00117654	0.00617556
ENSG00000C	294.079911	-0.1881815	0.10360858	-1.8162736	0.06932841	0.1534781
ENSG00000C	5655.65615	0.2346326	0.09387284	2.49947271	0.01243783	0.0413842
ENSG00000C	2171.23966	0.20943444	0.07218256	2.90145508	0.00371434	0.01548479
ENSG00000C	314.402188	-0.2315373	0.11655369	-1.9865294	0.04697457	0.11470094
ENSG00000C	1331.92749	-0.1675424	0.04070389	-4.1161275	3.85E-05	0.00037663
ENSG00000C	5665.7695	0.25491942	0.11632667	2.1914099	0.02842214	0.07874385
ENSG00000C	690.825349	0.20254221	0.12106517	1.6730015	0.094327	0.19281999
ENSG00000C	107.096439	0.77725048	0.21744223	3.57451478	0.00035088	0.00228663
ENSG00000C	75.327494	1.1827469	0.23158085	5.10727415	3.27E-07	7.05E-06
ENSG00000C	45382.3905	0.26072863	0.1291565	2.0187031	0.04351809	0.10841729
ENSG00000C	5749.08216	0.50314957	0.1437485	3.50020742	0.0004649	0.00289178
ENSG00000C	119.613271	-0.3952634	0.17825388	-2.2174183	0.02659452	0.07482518
ENSG00000C	642.304792	0.22711257	0.09010535	2.52052244	0.01171808	0.03952498
ENSG00000C	1135.15331	-0.3039663	0.09944702	-3.0565653	0.00223889	0.01027717
ENSG00000C	69.7071255	0.30427867	0.16879784	1.80262177	0.07144764	0.15711912
ENSG00000C	712.205006	-0.1702408	0.08783253	-1.9382427	0.05259362	0.12455841
ENSG00000C	70.9317298	0.46661642	0.12843845	3.6329962	0.00028015	0.0019081
ENSG00000C	2411.11253	0.13296388	0.06726084	1.97683949	0.04805978	0.1165831

ENSG00000(	1857.62623	0.12161955	0.05508761	2.2077479	0.02726185	0.076229
ENSG00000(	2961.23002	0.17221338	0.06253005	2.75408988	0.00588556	0.02255956
ENSG00000(	96.7326701	-0.7887148	0.36512828	-2.1601032	0.03076468	0.08355604
ENSG00000(	4509.14074	0.36742444	0.13356664	2.7508699	0.00594372	0.02272545
ENSG00000(	164.993296	-0.2431563	0.09732311	-2.4984437	0.012474	0.04148548
ENSG00000(	185.181901	0.23839437	0.06573858	3.62639951	0.0002874	0.00194924
ENSG00000(	3307.8715	0.56564584	0.16923575	3.34235429	0.00083071	0.00464267
ENSG00000(	22.3429362	1.57078753	0.34941024	4.49553944	6.94E-06	9.25E-05
ENSG00000(	3302.7765	-0.189262	0.08164347	-2.3181524	0.02044104	0.06141214
ENSG00000(	5679.92758	0.28539185	0.08773405	3.25292014	0.00114226	0.00602398
ENSG00000(	5294.54103	0.30822013	0.10191112	3.02440149	0.00249126	0.01119095
ENSG00000(	2661.86488	0.13914892	0.0792994	1.75472851	0.07930576	0.16915526
ENSG00000(	1281.47176	0.3403108	0.12240995	2.78009113	0.00543436	0.02117473
ENSG00000(	240.267043	-0.3490385	0.18041627	-1.9346286	0.05303589	0.12540084
ENSG00000(	14846.195	-0.3128895	0.08174915	-3.8274341	0.00012949	0.00102171
ENSG00000(	16262.8792	2.24563839	0.25224805	8.90250055	5.46E-19	2.64E-16
ENSG00000(	2886.96997	-0.2450664	0.08795461	-2.786282	0.00533165	0.02086428
ENSG00000(	6304.57856	0.31133579	0.12930053	2.40784632	0.01604693	0.05064748
ENSG00000(	60.7492124	0.71639598	0.23126952	3.09766713	0.0019505	0.0092343
ENSG00000(	320.799658	0.25329451	0.0953556	2.65631492	0.00789998	0.02875553
ENSG00000(	3344.72595	0.17923977	0.06977998	2.56864178	0.01020979	0.03545285
ENSG00000(	118.085299	-0.2223098	0.08885696	-2.5018836	0.01235345	0.04119808
ENSG00000(	3143.48332	-0.1922679	0.08755374	-2.1959983	0.02809207	0.07796356
ENSG00000(	60.0173504	-0.4248322	0.24589758	-1.7276796	0.08404569	0.17694665
ENSG00000(	11.162028	0.52402506	0.29580936	1.77149587	0.07647828	0.16489446
ENSG00000(	1017.99725	0.33341325	0.08893041	3.7491477	0.00017744	0.0013119
ENSG00000(	6126.88395	0.50051022	0.21830644	2.29269561	0.02186554	0.06447507
ENSG00000(	2125.41557	-0.1308607	0.06550749	-1.9976454	0.04575512	0.11247279
ENSG00000(	603.590319	-0.223801	0.10321134	-2.1683759	0.0301301	0.08231091
ENSG00000(	239.117945	-0.3548399	0.11210768	-3.1651702	0.00154992	0.00767649
ENSG00000(	6944.30131	0.47283499	0.14881423	3.17735072	0.00148627	0.00743614
ENSG00000(	1336.41293	0.6330149	0.1843966	3.4328989	0.00059716	0.00352819
ENSG00000(	3643.86823	0.36010947	0.12829374	2.80691387	0.00500186	0.01984716
ENSG00000(	2626.59631	0.25889232	0.12324434	2.10064268	0.03567234	0.09379043
ENSG00000(	13451.1509	1.56843036	0.21166116	7.41009998	1.26E-13	2.18E-11
ENSG00000(	937.79611	-0.252779	0.09907864	-2.5512965	0.0107323	0.03692206
ENSG00000(	560.077147	0.16052377	0.08138691	1.97235367	0.04856925	0.11744034
ENSG00000(	73356.9636	0.50287401	0.18029114	2.789233	0.0052833	0.02071988
ENSG00000(	2334.66404	0.43774466	0.13922182	3.14422453	0.00166528	0.00814877
ENSG00000(	19734.2971	0.59284871	0.17892651	3.3133644	0.00092181	0.00504886
ENSG00000(	16780.4204	-0.7350502	0.25345026	-2.9001752	0.00372954	0.01554369
ENSG00000(	80.5434124	0.49815103	0.23434747	2.12569413	0.03352874	0.08937885
ENSG00000(	11959.5357	0.34992569	0.12709811	2.75319358	0.0059017	0.02261251

ENSG00000(	33.6726692	1.34592047	0.25756171	5.22562329	1.74E-07	4.22E-06
ENSG00000(	6704.74086	0.28709385	0.10128551	2.83450081	0.00458973	0.0184289
ENSG00000(	487.904262	0.82724105	0.26795997	3.08718147	0.00202064	0.00947654
ENSG00000(	147.779921	-0.2633397	0.12017812	-2.1912452	0.02843405	0.07876179
ENSG00000(	3201.24164	-0.174295	0.04218694	-4.1314924	3.60E-05	0.00035738
ENSG00000(	16079.0342	0.42691595	0.10157986	4.20276192	2.64E-05	0.00027801
ENSG00000(	6484.61047	0.36971335	0.08822694	4.19048136	2.78E-05	0.00028908
ENSG00000(	3979.24733	-0.4400309	0.07726283	-5.6952464	1.23E-08	4.76E-07
ENSG00000(	616.315586	-0.900246	0.22228667	-4.0499327	5.12E-05	0.00047822
ENSG00000(	37110.3413	0.32921582	0.16444023	2.00203943	0.04528049	0.11169393
ENSG00000(	1751.28184	0.13045203	0.0718544	1.81550496	0.06944634	0.1536218
ENSG00000(	6813.42509	-0.2086479	0.08418317	-2.4784993	0.01319364	0.04343018
ENSG00000(	27214.1695	-0.5448737	0.120318	-4.5286133	5.94E-06	8.17E-05
ENSG00000(	267.173434	-0.2795798	0.11690649	-2.391482	0.01678051	0.05248255
ENSG00000(	9883.33451	-0.2161756	0.06021249	-3.5902116	0.00033041	0.00218097
ENSG00000(	1154.48879	-0.2024248	0.09630286	-2.1019607	0.03555672	0.09355433
ENSG00000(	2998.21105	-0.186278	0.05340124	-3.488272	0.00048615	0.00299571
ENSG00000(	492.420412	-0.2315111	0.12144177	-1.9063549	0.05660417	0.13196829
ENSG00000(	4964.33788	-0.2270664	0.06016762	-3.7738975	0.00016072	0.0012114
ENSG00000(	14686.4723	0.22694355	0.09874332	2.29831805	0.02154369	0.06386403
ENSG00000(	1924.32568	0.13252547	0.04614496	2.87193793	0.00407963	0.01670953
ENSG00000(	1393.52894	0.50582372	0.1383957	3.65490928	0.00025727	0.00177566
ENSG00000(	100.822426	0.98265341	0.32567809	3.0172537	0.00255076	0.01141226
ENSG00000(	2257.93623	0.25408246	0.10575548	2.40254662	0.01628136	0.05126452
ENSG00000(	704.29766	-0.261849	0.09719624	-2.6940236	0.00705952	0.02620995
ENSG00000(	13992.6129	0.30151746	0.16859815	1.78837939	0.07371482	0.16063577
ENSG00000(	6878.95868	0.11790696	0.0651798	1.80894943	0.07045886	0.15548172
ENSG00000(	292.623468	-0.1981135	0.0991512	-1.9980943	0.04570644	0.11238105
ENSG00000(	25648.2637	0.6694987	0.16382426	4.08668843	4.38E-05	0.00041953
ENSG00000(	1065.78366	-0.15084	0.04603629	-3.2765464	0.00105085	0.0056384
ENSG00000(	2095.59998	0.35064	0.15357676	2.28315794	0.02242107	0.06561982
ENSG00000(	7999.60505	0.48062237	0.15692045	3.06284092	0.00219247	0.01011859
ENSG00000(	2417.78396	-0.1518424	0.07956946	-1.9082998	0.05635249	0.13144074
ENSG00000(	108.293827	0.41882636	0.1914565	2.18757971	0.02870023	0.07930197
ENSG00000(	1123.75138	0.19934464	0.05983692	3.33146548	0.0008639	0.00477684
ENSG00000(	6718.53999	-0.1037991	0.06091242	-1.7040716	0.08836771	0.18370128
ENSG00000(	128.897451	-0.2509591	0.11697474	-2.1454125	0.03191988	0.08606427
ENSG00000(	7991.2403	0.23499835	0.0961046	2.4452352	0.01447577	0.04684174
ENSG00000(	2518.11948	-0.2378118	0.07670802	-3.1002207	0.00193377	0.00917604
ENSG00000(	5703.4298	0.28695287	0.15106234	1.89956588	0.05749011	0.13346508
ENSG00000(	70.4931541	1.44367711	0.31020083	4.65400794	3.26E-06	4.92E-05
ENSG00000(	12006.1275	-0.2228047	0.10190618	-2.186371	0.02878847	0.07947001
ENSG00000(	1838.19498	0.49565368	0.10950274	4.52640423	6.00E-06	8.21E-05

ENSG00000C	6294.63813	0.27057014	0.13276228	2.03800461	0.04154947	0.10488364
ENSG00000C	161.682068	2.25298759	0.39234669	5.74233874	9.34E-09	3.78E-07
ENSG00000C	131.005785	0.51027345	0.13678529	3.73047035	0.00019112	0.00138648
ENSG00000C	2055.95963	-0.1690528	0.10008019	-1.6891732	0.09118625	0.18820561
ENSG00000C	393.255596	-0.6070102	0.17153168	-3.5387645	0.000402	0.00255768
ENSG00000C	2147.8432	-0.1908615	0.06546743	-2.9153663	0.00355271	0.01495306
ENSG00000C	8487.011	-0.1450429	0.06803013	-2.1320391	0.03300363	0.08823097
ENSG00000C	76.1357343	-0.3884789	0.18199207	-2.1345923	0.03279432	0.08780103
ENSG00000C	3776.40987	-0.1468241	0.05129389	-2.8624089	0.00420434	0.01712832
ENSG00000C	1149.10201	0.61591183	0.11985953	5.13861376	2.77E-07	6.15E-06
ENSG00000C	2718.22821	-0.1894835	0.08687445	-2.181119	0.02917461	0.08031216
ENSG00000C	7256.65399	-0.3324697	0.10177496	-3.2667136	0.00108804	0.00579713
ENSG00000C	997.675738	-0.2586021	0.11117748	-2.3260294	0.02001698	0.06045154
ENSG00000C	8913.80607	0.28318895	0.15819892	1.79008139	0.07344083	0.16023152
ENSG00000C	2397.53318	-0.3807778	0.06289204	-6.0544662	1.41E-09	7.32E-08
ENSG00000C	6378.04842	0.4679112	0.14772481	3.16745159	0.00153781	0.00764002
ENSG00000C	3504.29122	-0.1093681	0.05577899	-1.9607398	0.04990938	0.11977687
ENSG00000C	116.646275	1.55565774	0.2790138	5.57555845	2.47E-08	8.45E-07
ENSG00000C	4992.91295	-0.142317	0.07300459	-1.9494258	0.0512446	0.12211049
ENSG00000C	47.6603581	-1.2754744	0.31887428	-3.9999286	6.34E-05	0.00056651
ENSG00000C	488.06669	0.25602624	0.12787087	2.00222481	0.04526056	0.1116828
ENSG00000C	582.848788	-0.2180038	0.07887812	-2.7638062	0.00571315	0.02205338
ENSG00000C	2110.85934	-0.2302146	0.08474612	-2.7165205	0.00659721	0.02477931
ENSG00000C	32.9803695	0.93022314	0.39039191	2.38279309	0.01718185	0.05347227
ENSG00000C	345.233701	0.45419444	0.13701441	3.31493926	0.00091663	0.00503192
ENSG00000C	727.135715	-0.1671474	0.10020065	-1.6681272	0.09529048	0.19424135
ENSG00000C	2556.41599	0.41977846	0.13178872	3.1852382	0.00144635	0.00729638
ENSG00000C	18048.2372	1.03368791	0.14451044	7.15303274	8.49E-13	1.17E-10
ENSG00000C	27842.9964	0.29869543	0.1425739	2.09502181	0.03616905	0.09478674
ENSG00000C	243.303926	-0.4906044	0.13623901	-3.6010569	0.00031693	0.00210901
ENSG00000C	589.510786	0.44052058	0.10332177	4.26357968	2.01E-05	0.0002218
ENSG00000C	1159.78592	0.09366336	0.05298954	1.76758203	0.0771308	0.16581006
ENSG00000C	1864.34064	0.39243847	0.07712515	5.08833335	3.61E-07	7.67E-06
ENSG00000C	1008.88699	0.22577196	0.05905841	3.82285846	0.00013191	0.00103579
ENSG00000C	1147.2565	0.10536164	0.06152276	1.71256367	0.08679284	0.18117693
ENSG00000C	4.70256192	4.33361391	1.57353296	2.75406618	0.00588599	0.02255956
ENSG00000C	2317.82321	-0.2663441	0.0738128	-3.6083732	0.00030812	0.00205704
ENSG00000C	55.0776653	0.59478069	0.28088535	2.11752121	0.03421564	0.09076761
ENSG00000C	2923.22927	-0.4727931	0.10043874	-4.7072788	2.51E-06	3.99E-05
ENSG00000C	5002.65588	0.34892496	0.15468894	2.2556555	0.02409221	0.06949899
ENSG00000C	2837.7328	-0.1056806	0.06384342	-1.6553086	0.09786191	0.19828329
ENSG00000C	558.873923	-0.152576	0.08776941	-1.7383732	0.08214508	0.17398184
ENSG00000C	4565.87541	0.21525197	0.11673634	1.84391575	0.06519542	0.14693311

ENSG00000C	9360.51296	1.57000343	0.2410096	6.51427761	7.30E-11	6.08E-09
ENSG00000C	3663.76	0.47172191	0.13613405	3.46512802	0.00052998	0.00320577
ENSG00000C	13216.1361	0.28887725	0.12740454	2.26740158	0.0233657	0.0677813
ENSG00000C	1903.92684	-0.1688391	0.07910896	-2.1342597	0.03282153	0.08785764
ENSG00000C	6661.61041	0.45020469	0.17115742	2.63035441	0.00852959	0.03066208
ENSG00000C	6771.66043	0.41469462	0.08048868	5.1522103	2.57E-07	5.81E-06
ENSG00000C	293.611852	0.3311229	0.1092149	3.03184739	0.00243062	0.01097643
ENSG00000C	7400.43215	0.25822575	0.13824641	1.86786579	0.06178078	0.1412146
ENSG00000C	6801.01676	0.23570792	0.06742582	3.49581083	0.00047262	0.00292978
ENSG00000C	4002.03671	0.11206599	0.05131514	2.18387768	0.02897124	0.07983761
ENSG00000C	1687.50208	0.72534687	0.15113973	4.79918054	1.59E-06	2.70E-05
ENSG00000C	1475.2225	0.30564607	0.10123946	3.01904072	0.00253576	0.01137326
ENSG00000C	912.97164	0.41873007	0.13491787	3.10359244	0.00191186	0.00909297
ENSG00000C	173.730932	-0.6336875	0.27310326	-2.320322	0.02032346	0.06110959
ENSG00000C	1728.25349	-0.2276477	0.0653964	-3.4810429	0.00049947	0.00305936
ENSG00000C	769.054541	-0.1731145	0.0900703	-1.9219933	0.0546066	0.12821488
ENSG00000C	10278.7435	0.22956142	0.12415314	1.84901817	0.06445519	0.14580923
ENSG00000C	2873.36765	0.26677739	0.06318614	4.22208712	2.42E-05	0.00025936
ENSG00000C	29.3279424	1.02926581	0.25908237	3.97273586	7.11E-05	0.00062611
ENSG00000C	446.154633	-0.1546619	0.06465462	-2.3921238	0.01675119	0.05242483
ENSG00000C	2131.38857	-0.2492655	0.06550926	-3.8050423	0.00014178	0.00109486
ENSG00000C	1144.55123	0.47415398	0.1366112	3.47082793	0.00051886	0.003149
ENSG00000C	110.886267	-0.5393311	0.17748259	-3.0387833	0.00237536	0.01075368
ENSG00000C	7658.69837	0.63584186	0.13393392	4.74742972	2.06E-06	3.36E-05
ENSG00000C	1679.95392	0.27055513	0.09542801	2.83517513	0.00458006	0.01839514
ENSG00000C	1520.23382	0.3739052	0.1118913	3.34168245	0.00083272	0.0046506
ENSG00000C	906.744032	-0.1682153	0.07335022	-2.2933163	0.0218298	0.06440902
ENSG00000C	722.57486	0.5199394	0.16794525	3.09588624	0.00196226	0.00927176
ENSG00000C	379.014212	0.42051372	0.14740515	2.852775	0.00433393	0.01757225
ENSG00000C	1940.08368	0.4914118	0.11911722	4.12544718	3.70E-05	0.00036416
ENSG00000C	2173.0665	0.24512498	0.11710264	2.09324894	0.03632694	0.09506292
ENSG00000C	7976.60098	0.4103947	0.19157262	2.14224086	0.03217411	0.08653445
ENSG00000C	45665.5822	-0.5637353	0.07650082	-7.3690098	1.72E-13	2.83E-11
ENSG00000C	825.363733	-0.1676108	0.05813253	-2.8832529	0.00393591	0.01621256
ENSG00000C	863.700113	-0.1684883	0.05003963	-3.367097	0.00075964	0.0043072
ENSG00000C	445.673279	0.30674715	0.09265749	3.310549	0.00093113	0.00509607
ENSG00000C	2304.57682	-0.342059	0.0913663	-3.7438204	0.00018124	0.00133148
ENSG00000C	13667.5677	0.15666469	0.07680724	2.03971259	0.04137896	0.10452607
ENSG00000C	504.550679	-0.2277406	0.07192795	-3.1662326	0.00154427	0.00765582
ENSG00000C	3428.60068	0.63881974	0.21235765	3.00822563	0.00262778	0.01170622
ENSG00000C	2332.00371	0.23032878	0.1258645	1.82997414	0.06725381	0.14998938
ENSG00000C	299.679225	-0.2559381	0.11504067	-2.2247621	0.02609721	0.07374105
ENSG00000C	525.798075	-0.3993604	0.10985659	-3.6352889	0.00027767	0.00189477

ENSG00000(	66.797928	0.9780639	0.26315914	3.71662529	0.0002019	0.00145231
ENSG00000(	550.385879	-0.2863318	0.11028413	-2.5963104	0.00942309	0.03327134
ENSG00000(	27.0927374	0.81263669	0.34595209	2.34898619	0.01882461	0.05766802
ENSG00000(	1598.15728	-0.2240681	0.05517447	-4.0610837	4.88E-05	0.0004595
ENSG00000(	1423.5671	-0.2477116	0.13996201	-1.7698491	0.07675228	0.16532965
ENSG00000(	98.5268311	-0.2998088	0.15776311	-1.9003731	0.05738417	0.13328917
ENSG00000(	2974.85231	0.4183566	0.07820077	5.34977619	8.81E-08	2.40E-06
ENSG00000(	242.404692	-0.4830095	0.1561203	-3.0938289	0.00197591	0.00932716
ENSG00000(	11824.9267	-0.158043	0.07958349	-1.9858769	0.04704699	0.1148197
ENSG00000(	348.665716	-0.4645663	0.15460189	-3.0049201	0.00265651	0.01180878
ENSG00000(	1761.42444	-0.1574331	0.06474761	-2.4314888	0.01503691	0.048248
ENSG00000(	1574.72609	0.20595617	0.11749206	1.75293692	0.07961284	0.16961046
ENSG00000(	432.959334	-0.1452475	0.07846104	-1.8512056	0.06413999	0.14525496
ENSG00000(	4879.03001	-0.2932593	0.0784699	-3.7372202	0.00018607	0.00135659
ENSG00000(	70.1508926	1.75672272	0.31054609	5.65688239	1.54E-08	5.70E-07
ENSG00000(	5769.06076	0.12740565	0.05655443	2.25279694	0.02427196	0.06989223
ENSG00000(	59.4803396	0.55579062	0.20346019	2.73169225	0.006301	0.02385848
ENSG00000(	3175.32756	0.62611052	0.18244781	3.43172395	0.00059976	0.00354062
ENSG00000(	2162.57138	-0.1629978	0.08351035	-1.9518272	0.05095873	0.12153231
ENSG00000(	1755.47241	-0.2416395	0.05524538	-4.3739311	1.22E-05	0.00014695
ENSG00000(	65.9347829	0.82599721	0.18158347	4.54885677	5.39E-06	7.55E-05
ENSG00000(	2285.60408	0.17330514	0.05196508	3.33503074	0.0008529	0.00473227
ENSG00000(	1515.35788	-0.1792757	0.10199635	-1.7576681	0.07880399	0.16833286
ENSG00000(	7067.94931	0.52740786	0.21077499	2.50223166	0.01234131	0.04116708
ENSG00000(	340.034957	0.29787417	0.14373254	2.0724198	0.03822631	0.09884433
ENSG00000(	5512.70473	-0.3276524	0.09333996	-3.5103118	0.00044758	0.00279367
ENSG00000(	565.314136	-0.2144902	0.11407742	-1.8802158	0.06007867	0.13814606
ENSG00000(	1756.05136	-0.2208376	0.08989179	-2.4567052	0.01402177	0.04566848
ENSG00000(	9726.5483	0.46537554	0.15969904	2.9140785	0.0035674	0.01499737
ENSG00000(	909.259618	-0.3423875	0.09123474	-3.75282	0.00017486	0.00129705
ENSG00000(	647.308591	-0.2305587	0.10769739	-2.1408011	0.03229009	0.08670741
ENSG00000(	506.278508	-0.1650458	0.07120354	-2.3179433	0.0204524	0.06142081
ENSG00000(	6268.53753	0.28922951	0.10362789	2.7910394	0.00525391	0.02061651
ENSG00000(	3727.42798	0.988962	0.22496926	4.39598736	1.10E-05	0.0001348
ENSG00000(	484.458821	0.34185656	0.09337784	3.66100317	0.00025123	0.00173726
ENSG00000(	9601.43369	-0.3140334	0.08533207	-3.6801335	0.00023311	0.00163145
ENSG00000(	45.7752854	1.83000993	0.32814226	5.57687975	2.45E-08	8.41E-07
ENSG00000(	34.5119363	-0.9847349	0.30440619	-3.2349371	0.0012167	0.0063381
ENSG00000(	826.762248	-0.1819735	0.08222894	-2.2130101	0.02689695	0.07551475
ENSG00000(	341.175425	-0.1609894	0.08828629	-1.8234931	0.06822874	0.15152994
ENSG00000(	344.885807	0.27371616	0.16594478	1.64944124	0.09905727	0.19989451
ENSG00000(	8402.06763	0.20604206	0.07093692	2.90458153	0.00367744	0.01538064
ENSG00000(	1967.55064	0.26985217	0.12000281	2.24871542	0.02453061	0.07053889

ENSG00000(	493.940688	0.4390674	0.18237362	2.407516	0.01606146	0.05068228
ENSG00000(	93.1327265	0.36389019	0.12486107	2.91436066	0.00356418	0.01498817
ENSG00000(	3194.6666	0.37176546	0.11641979	3.19331851	0.00140648	0.00712684
ENSG00000(	486.001611	0.39630011	0.08554286	4.63276644	3.61E-06	5.36E-05
ENSG00000(	115.604163	1.13792334	0.21132042	5.38482439	7.25E-08	2.06E-06
ENSG00000(	366.800468	0.38356713	0.19435979	1.97349014	0.04843975	0.11721173
ENSG00000(	2884.99706	0.23073855	0.13872474	1.6632833	0.09625574	0.19585069
ENSG00000(	707.426369	0.4951297	0.10362122	4.77826545	1.77E-06	2.94E-05
ENSG00000(	4693.91405	0.16620635	0.06763789	2.45729635	0.01399872	0.04560694
ENSG00000(	335.881227	-0.3513755	0.16082456	-2.1848373	0.02890078	0.07973445
ENSG00000(	4346.69381	0.72274419	0.14060098	5.14039242	2.74E-07	6.11E-06
ENSG00000(	6261.79103	0.16317925	0.09359155	1.74352536	0.08124189	0.17249762
ENSG00000(	11544.1917	-0.1215364	0.06590145	-1.844215	0.06515181	0.14685768
ENSG00000(	4493.2492	0.47089099	0.1580541	2.97930261	0.00288905	0.01263699
ENSG00000(	250.009631	-0.4590875	0.14739095	-3.1147603	0.00184094	0.00883105
ENSG00000(	346810.866	-0.489403	0.14803335	-3.306032	0.00094627	0.00516723
ENSG00000(	172.936094	0.3247708	0.13578104	2.39187144	0.01676271	0.05244955
ENSG00000(	1421.1227	-0.1550492	0.08268455	-1.8751896	0.06076665	0.13940245
ENSG00000(	2669.85675	-0.3013508	0.05201319	-5.7937379	6.88E-09	2.92E-07
ENSG00000(	740.859124	-0.1584545	0.07086496	-2.2360062	0.02535136	0.07217666
ENSG00000(	1295.60015	0.20632228	0.1163051	1.77397453	0.07606736	0.16420621
ENSG00000(	1026.46087	0.17337653	0.0645489	2.68597169	0.00723192	0.02670631
ENSG00000(	15084.6025	0.25134909	0.09643584	2.6063868	0.00915031	0.03251422
ENSG00000(	3008.67963	-0.2338406	0.07398401	-3.1606906	0.00157396	0.00778488
ENSG00000(	611.743215	-0.4243556	0.09516333	-4.4592342	8.23E-06	0.00010601
ENSG00000(	665.702531	0.60805599	0.22498639	2.70263452	0.00687923	0.02566558
ENSG00000(	227.99185	0.51235982	0.15405646	3.32579238	0.00088168	0.00486214
ENSG00000(	15962.9717	0.19430819	0.11708898	1.65949171	0.09701675	0.19712225
ENSG00000(	765.901186	-0.2866927	0.15630688	-1.8341655	0.06662943	0.14905197
ENSG00000(	809.794721	-0.2852406	0.10396211	-2.7436973	0.00607515	0.02311813
ENSG00000(	7025.92441	0.42795911	0.13993658	3.05823612	0.00222644	0.01023301
ENSG00000(	760.453505	-0.13585	0.05388745	-2.5209947	0.01170236	0.03949666
ENSG00000(	5039.4152	0.55745798	0.14665807	3.80107257	0.00014407	0.00110901
ENSG00000(	3737.59443	-0.3480863	0.15323353	-2.2716068	0.02311027	0.06718411
ENSG00000(	456.884973	0.99264488	0.41639977	2.3838747	0.01713143	0.05336123
ENSG00000(	3843.9671	-0.1417959	0.07507291	-1.8887757	0.05892189	0.13619233
ENSG00000(	6600.5421	-0.1791628	0.0689399	-2.5988255	0.00935433	0.03310925
ENSG00000(	95.3783144	0.74663987	0.17169941	4.34852898	1.37E-05	0.00016131
ENSG00000(	7172.22539	0.43055211	0.17717855	2.43004645	0.01509689	0.04839757
ENSG00000(	3362.93225	0.17067537	0.07290084	2.34119908	0.01922191	0.05852625
ENSG00000(	2200.00947	-0.1770086	0.09703165	-1.8242362	0.06811637	0.15134998
ENSG00000(	748.364309	-0.1868207	0.09065314	-2.0608303	0.03931923	0.1009424
ENSG00000(	6097.89977	0.22142714	0.10805924	2.04912731	0.04044967	0.10295052

ENSG00000(	22210.2409	-0.2833867	0.11624462	-2.437848	0.01477499	0.04755503
ENSG00000(	568.755077	-0.2867911	0.10040079	-2.8564626	0.00428391	0.01740512
ENSG00000(	16104.3882	0.45885754	0.22126645	2.07377817	0.03809992	0.09866884
ENSG00000(	77.2517341	1.17925498	0.27299977	4.31961901	1.56E-05	0.00018025
ENSG00000(	6481.35797	-0.1727487	0.07993608	-2.1610856	0.03068873	0.08344217
ENSG00000(	684.22058	-0.2494964	0.06475724	-3.8527959	0.00011678	0.00093953
ENSG00000(	765.514689	2.39792188	0.30961744	7.74478952	9.57E-15	2.10E-12
ENSG00000(	145.311583	1.10973585	0.50184961	2.21129164	0.02701565	0.07572209
ENSG00000(	3012.01736	0.13549824	0.05770745	2.34801979	0.01887352	0.057769
ENSG00000(	301.561789	-0.4824695	0.08450203	-5.7095617	1.13E-08	4.46E-07
ENSG00000(	10855.7394	0.16312453	0.04719721	3.45623243	0.00054778	0.00329142
ENSG00000(	68.9804333	-0.2443271	0.11039745	-2.2131591	0.02688668	0.07550054
ENSG00000(	1343.2825	-0.1465327	0.07473463	-1.9607069	0.04991323	0.11977687
ENSG00000(	627.289102	0.20054065	0.06679953	3.00212659	0.00268101	0.0118885
ENSG00000(	2121.31976	-0.4003283	0.13572834	-2.9494821	0.00318307	0.01369968
ENSG00000(	14372.6998	0.30222394	0.12771888	2.36632163	0.01796583	0.05542397
ENSG00000(	3260.01702	0.17576276	0.05520806	3.18364314	0.00145434	0.0073131
ENSG00000(	1689.3031	-0.3274877	0.07343896	-4.4593185	8.22E-06	0.00010601
ENSG00000(	1837.92843	-0.1647608	0.07170173	-2.2978637	0.02156955	0.06391254
ENSG00000(	1246.73326	-0.6612377	0.19820445	-3.3361398	0.0008495	0.00471885
ENSG00000(	123.318563	0.43982326	0.11704367	3.75777053	0.00017143	0.00127755
ENSG00000(	32.6819163	0.82972797	0.23476707	3.53426052	0.00040892	0.00259597
ENSG00000(	320.766196	-0.2721771	0.08305814	-3.2769474	0.00104936	0.00563248
ENSG00000(	65.4825783	1.26487683	0.34985714	3.61540944	0.00029987	0.0020131
ENSG00000(	135.16265	1.65527533	0.29997074	5.51812272	3.43E-08	1.13E-06
ENSG00000(	16426.9225	0.1369629	0.07747616	1.7678069	0.07709319	0.1657784
ENSG00000(	378.803286	0.53065811	0.1207007	4.39647921	1.10E-05	0.00013473
ENSG00000(	3952.90965	0.83799209	0.16750023	5.00293084	5.65E-07	1.12E-05
ENSG00000(	9554.35696	0.42221477	0.16748334	2.52093603	0.01170431	0.03949666
ENSG00000(	2398.48109	-0.5156517	0.26249147	-1.9644511	0.04947781	0.11906727
ENSG00000(	319.875441	0.28232397	0.149711	1.88579306	0.05932285	0.13690345
ENSG00000(	4142.23389	1.43201119	0.21379328	6.6981113	2.11E-11	1.95E-09
ENSG00000(	1115.54094	0.12424568	0.04950162	2.5099318	0.01207545	0.04046658
ENSG00000(	135.24692	1.96146608	0.33270471	5.89551635	3.74E-09	1.74E-07
ENSG00000(	370.814758	-0.400913	0.09707762	-4.1298189	3.63E-05	0.00035901
ENSG00000(	408.714417	-0.2735073	0.12992153	-2.1051733	0.03527623	0.09300214
ENSG00000(	519.788328	-0.569753	0.13133467	-4.3381771	1.44E-05	0.00016785
ENSG00000(	69.6193356	0.76015769	0.27907758	2.72382209	0.00645313	0.02432009
ENSG00000(	8112.79546	0.26539753	0.11081752	2.39490596	0.01662463	0.05209626
ENSG00000(	2226.13352	0.18345008	0.09756444	1.8802965	0.06006768	0.13814606
ENSG00000(	6219.42168	-0.2373776	0.0689556	-3.4424696	0.00057643	0.00342971
ENSG00000(	1417.20497	-0.4350823	0.14458502	-3.0091798	0.00261954	0.0116767
ENSG00000(	65.5656449	-0.750779	0.17491501	-4.2922505	1.77E-05	0.0001994

ENSG00000(	360.106435	-0.7010924	0.30025337	-2.3350026	0.01954329	0.05928048
ENSG00000(	674.889474	0.25513289	0.10899438	2.34078948	0.01924301	0.05856587
ENSG00000(	9940.26204	0.21977302	0.1189876	1.84702447	0.06474359	0.14614217
ENSG00000(	2648.61732	0.25815107	0.09165642	2.81650834	0.00485488	0.01934341
ENSG00000(	106.738482	-0.3321845	0.14526909	-2.2866838	0.02221428	0.06521074
ENSG00000(	12661.9588	0.33873465	0.09055483	3.74065824	0.00018354	0.00134289
ENSG00000(	3077.94755	-0.1185295	0.06891786	-1.7198655	0.08545689	0.17916267
ENSG00000(	1093.74483	0.28713179	0.16488793	1.74137548	0.08161778	0.17316884
ENSG00000(	364.090569	1.33000656	0.16329028	8.14504438	3.79E-16	9.99E-14
ENSG00000(	16023.3357	-0.6032624	0.16134623	-3.7389312	0.0001848	0.00135079
ENSG00000(	3183.30539	0.31482821	0.10835017	2.90565486	0.00366485	0.01534472
ENSG00000(	356.340571	-0.3619985	0.08069587	-4.4859612	7.26E-06	9.59E-05
ENSG00000(	17797.6001	0.39084256	0.12460505	3.13665098	0.00170889	0.00831042
ENSG00000(	344.038443	-0.2715434	0.11117763	-2.4424281	0.01458884	0.04716547
ENSG00000(	581.636836	-0.3399508	0.06626272	-5.1303476	2.89E-07	6.37E-06
ENSG00000(	4208.74421	-0.1861225	0.04900265	-3.7982122	0.00014574	0.0011201
ENSG00000(	427.660368	0.34563343	0.1009088	3.42520587	0.00061433	0.00361636
ENSG00000(	2994.68377	0.69853582	0.14134256	4.94214771	7.73E-07	1.45E-05
ENSG00000(	36397.4752	0.1948548	0.11257397	1.73090448	0.0834688	0.17617936
ENSG00000(	2602.86929	-0.4259404	0.24005162	-1.7743702	0.07600194	0.16413836
ENSG00000(	94.4662618	-0.26759	0.10867627	-2.462267	0.01380619	0.04508797
ENSG00000(	4302.52161	0.12606301	0.06967171	1.80938576	0.07039109	0.15538972
ENSG00000(	2828.13053	-0.3020594	0.13943353	-2.1663324	0.03028579	0.08265831
ENSG00000(	303.863159	0.54592856	0.14063755	3.88181224	0.00010368	0.00085052
ENSG00000(	681.613743	-0.3484538	0.092039	-3.7859366	0.00015313	0.00116391
ENSG00000(	755.98405	-0.1584722	0.08141704	-1.9464256	0.05160363	0.12277458
ENSG00000(	6471.91603	0.18623726	0.07896727	2.35841067	0.01835338	0.05645125
ENSG00000(	7507.712	0.44875705	0.1419395	3.16160796	0.00156901	0.0077657
ENSG00000(	578.144659	0.25272599	0.1168012	2.16372772	0.03048525	0.08304703
ENSG00000(	312.785871	-0.292959	0.09482844	-3.0893583	0.00200589	0.0094261
ENSG00000(	10051.4861	0.45059849	0.16360222	2.75423218	0.005883	0.02255956
ENSG00000(	2023.35867	0.12107733	0.07236996	1.67303288	0.09432082	0.19281999
ENSG00000(	12436.779	0.19443378	0.06644881	2.92606852	0.00343275	0.01453677
ENSG00000(	1163.05055	-0.1341092	0.06045035	-2.2185022	0.02652061	0.07464622
ENSG00000(	228.396852	-0.3120857	0.08849587	-3.5265573	0.000421	0.00265637
ENSG00000(	1634.34547	0.25977045	0.0882139	2.94477909	0.00323185	0.01385203
ENSG00000(	1516.50941	-0.1270692	0.05710958	-2.2250061	0.02608083	0.07372351
ENSG00000(	2327.05307	0.15854429	0.07780488	2.03771662	0.04157828	0.10493808
ENSG00000(	2925.38801	0.2625882	0.13370321	1.96396333	0.04953435	0.11916374
ENSG00000(	5065.83712	-0.1351911	0.06430516	-2.102336	0.03552385	0.09351882
ENSG00000(	1331.07129	-0.1551519	0.05586986	-2.7770234	0.00548592	0.02133545
ENSG00000(	8347.6203	0.48940109	0.2226958	2.19762152	0.02797609	0.07773103
ENSG00000(	7769.67737	-0.3736764	0.131364	-2.8445876	0.0044469	0.01793491

ENSG00000C	209.901636	-0.1925378	0.07088026	-2.7163813	0.00659998	0.0247833
ENSG00000C	3256.6152	-0.4017454	0.06926744	-5.7999178	6.63E-09	2.83E-07
ENSG00000C	9.61094051	5.21532589	0.83984298	6.20988214	5.30E-10	3.25E-08
ENSG00000C	3642.92136	-0.1903859	0.05828706	-3.2663496	0.00108944	0.00580245
ENSG00000C	7701.00001	-0.1615301	0.08445973	-1.9125106	0.05581073	0.13045016
ENSG00000C	3583.36807	0.46217355	0.14431941	3.2024351	0.00136271	0.00693661
ENSG00000C	1213.42195	-0.3447233	0.14677324	-2.3486795	0.01884012	0.05768001
ENSG00000C	93039.2431	0.2531974	0.13650561	1.85484973	0.06361769	0.1442639
ENSG00000C	659.403465	0.34939543	0.11649854	2.99914002	0.00270743	0.01198
ENSG00000C	1196.94265	0.16643912	0.08924096	1.86505301	0.06217397	0.14184478
ENSG00000C	1785.84351	-0.2574629	0.0698498	-3.6859505	0.00022785	0.00160314
ENSG00000C	266.564139	-0.2711846	0.1119259	-2.4228942	0.01539741	0.04904624
ENSG00000C	892.865436	-0.1872254	0.08349878	-2.2422536	0.02494499	0.07139037
ENSG00000C	2567.57227	0.37226534	0.11184232	3.32848365	0.0008732	0.00482275
ENSG00000C	220.925991	-0.4651411	0.14580687	-3.1901183	0.00142215	0.00719868
ENSG00000C	1609.32634	-0.2242946	0.07664904	-2.9262544	0.0034307	0.01453233
ENSG00000C	465.50639	-0.543309	0.165035	-3.2920832	0.00099448	0.00539186
ENSG00000C	2265.18157	-0.3334431	0.09676192	-3.4460159	0.00056892	0.00339033
ENSG00000C	947.551762	0.16780305	0.06090873	2.75499188	0.00586936	0.02253031
ENSG00000C	1439.3319	-0.2087289	0.10459976	-1.9955005	0.04598832	0.11280609
ENSG00000C	51.4356686	-0.2525479	0.13692566	-1.8444161	0.06512252	0.14683734
ENSG00000C	4872.88189	0.18507244	0.08273272	2.23699214	0.02528685	0.07208734
ENSG00000C	121.694639	0.74587862	0.19162724	3.89234137	9.93E-05	0.00082094
ENSG00000C	1766.02322	-0.3140315	0.08746557	-3.5903441	0.00033024	0.00218097
ENSG00000C	44.6874187	1.10330215	0.22080742	4.99667154	5.83E-07	1.15E-05
ENSG00000C	7733.27823	0.59558011	0.10210497	5.83301759	5.44E-09	2.41E-07
ENSG00000C	2994.07305	1.64837054	0.20947774	7.86895312	3.58E-15	8.36E-13
ENSG00000C	2460.76363	-0.0959805	0.05512938	-1.741004	0.08168288	0.17322214
ENSG00000C	1317.56401	-0.152939	0.08472808	-1.8050564	0.07106586	0.15648748
ENSG00000C	7500.28021	-0.2112949	0.10411924	-2.0293554	0.0424221	0.10652955
ENSG00000C	455.236743	0.42705626	0.17490747	2.44161241	0.01462184	0.04721947
ENSG00000C	1334.39637	0.440924	0.14409943	3.05985935	0.00221441	0.01019065
ENSG00000C	520.67591	2.21794857	0.41006667	5.40875117	6.35E-08	1.85E-06
ENSG00000C	2962.76306	0.19820883	0.07820676	2.5344208	0.01126334	0.03834829
ENSG00000C	1072.93539	-0.2351918	0.08730452	-2.6939245	0.00706162	0.02621102
ENSG00000C	19123.9801	0.33028729	0.13321523	2.47935072	0.01316218	0.04335442
ENSG00000C	569.704792	-0.2341374	0.07109009	-3.2935311	0.00098937	0.00536618
ENSG00000C	158.833592	-0.2702754	0.10636499	-2.5410182	0.01105302	0.03775644
ENSG00000C	535.694283	-0.1759439	0.05719034	-3.0764613	0.00209474	0.00975456
ENSG00000C	535.206757	0.31642373	0.08733736	3.62300543	0.0002912	0.0019658
ENSG00000C	5653.06648	-0.1625845	0.06840584	-2.376764	0.01746526	0.05413333
ENSG00000C	4434.84334	0.14631309	0.08527519	1.71577564	0.08620311	0.18033567
ENSG00000C	922.833132	-0.0915099	0.05355909	-1.708579	0.08752896	0.18239816

ENSG00000C	3767.34101	-0.2029279	0.06234427	-3.2549566	0.0011341	0.00599404
ENSG00000C	465.267667	4.44954347	0.58340089	7.62690554	2.40E-14	4.84E-12
ENSG00000C	11083.4398	0.63138968	0.15047482	4.19598233	2.72E-05	0.00028483
ENSG00000C	431.089383	1.23785765	0.17386412	7.11968445	1.08E-12	1.42E-10
ENSG00000C	1034.09334	-0.283015	0.07478293	-3.7844868	0.00015403	0.00116887
ENSG00000C	27048.2609	0.31191266	0.08970887	3.47694318	0.00050717	0.0030949
ENSG00000C	1278.76439	-0.3124879	0.07896023	-3.9575361	7.57E-05	0.00065929
ENSG00000C	15277.2861	-0.2164874	0.08871495	-2.4402579	0.01467678	0.04732305
ENSG00000C	89.7363882	-0.197892	0.10998721	-1.7992274	0.07198272	0.158026
ENSG00000C	398.184982	-0.1653535	0.07433709	-2.2243739	0.0261233	0.07377157
ENSG00000C	2612.14157	0.26152288	0.08729033	2.99601208	0.00273536	0.01207477
ENSG00000C	12664.8771	-0.2052873	0.11145082	-1.8419544	0.06548183	0.14722888
ENSG00000C	1200.63791	0.71229907	0.12323592	5.7799632	7.47E-09	3.15E-07
ENSG00000C	143.333053	0.54918323	0.16203947	3.38919426	0.00070098	0.00402982
ENSG00000C	3252.53926	-0.3318933	0.164526	-2.0172696	0.04366739	0.10864967
ENSG00000C	4465.03933	-0.3371796	0.0679352	-4.963253	6.93E-07	1.33E-05
ENSG00000C	225.832091	-0.2544522	0.11529963	-2.2068782	0.02732257	0.07633984
ENSG00000C	216.137832	0.92789289	0.24534181	3.7820415	0.00015555	0.00117856
ENSG00000C	41.8641663	1.34578198	0.28496073	4.72269275	2.33E-06	3.75E-05
ENSG00000C	1466.40803	-0.3094035	0.12288919	-2.5177435	0.01181093	0.0398011
ENSG00000C	417.021338	0.66346251	0.26057628	2.54613548	0.01089229	0.03735715
ENSG00000C	603.254086	-0.4477384	0.10681982	-4.1915297	2.77E-05	0.00028854
ENSG00000C	1044.10535	-0.2654264	0.07952678	-3.3375724	0.00084514	0.0047
ENSG00000C	12478.5797	4.64922017	0.41571085	11.1837836	4.90E-29	1.01E-25
ENSG00000C	353.704452	-0.1516933	0.07423939	-2.0432988	0.04102287	0.1038983
ENSG00000C	108.170391	-0.813194	0.21641756	-3.757523	0.0001716	0.00127758
ENSG00000C	7895.12428	0.11041744	0.04716036	2.34131887	0.01921575	0.05851977
ENSG00000C	141.93434	-0.3133688	0.1552679	-2.0182459	0.04356566	0.10847985
ENSG00000C	3187.13615	-0.4483698	0.19361492	-2.3157814	0.02057021	0.06165954
ENSG00000C	344.715685	0.34492214	0.14422905	2.39148866	0.0167802	0.05248255
ENSG00000C	418.47682	-0.219555	0.08169025	-2.6876529	0.00719562	0.02660615
ENSG00000C	3617.92538	0.15496101	0.08935144	1.73428658	0.08286725	0.17525515
ENSG00000C	108.130315	-0.1816626	0.09532889	-1.9056402	0.05669691	0.13208565
ENSG00000C	2160.28215	-0.204451	0.07915011	-2.5830792	0.00979228	0.0342659
ENSG00000C	249.564373	-0.2055354	0.08898062	-2.3098899	0.02089425	0.06238562
ENSG00000C	34514.6409	3.11490168	0.29471402	10.5692349	4.14E-26	6.00E-23
ENSG00000C	3308.54302	1.97721747	0.44017103	4.49193	7.06E-06	9.38E-05
ENSG00000C	3177.6613	-0.3032784	0.10205132	-2.971822	0.00296038	0.01288674
ENSG00000C	17798.97	-0.1305803	0.04688942	-2.7848572	0.00535513	0.02093356
ENSG00000C	2718.51815	0.36246866	0.06227021	5.82090022	5.85E-09	2.56E-07
ENSG00000C	17601.0277	-0.341535	0.11050156	-3.0907704	0.00199638	0.00939811
ENSG00000C	13050.8836	0.28001893	0.13692575	2.04504214	0.0408507	0.10356866
ENSG00000C	36.2968794	0.62501791	0.25618068	2.43975432	0.01469725	0.04737853

ENSG00000(	148.657988	-0.5675919	0.10235793	-5.5451673	2.94E-08	9.92E-07
ENSG00000(	504.591186	0.7353011	0.19274946	3.81480245	0.00013629	0.00106167
ENSG00000(	9934.90377	-0.2528591	0.06457526	-3.9157267	9.01E-05	0.00075783
ENSG00000(	24796.0865	0.24025701	0.13584207	1.76864949	0.07695239	0.16562437
ENSG00000(	265.109085	0.23601289	0.12346855	1.9115223	0.05593749	0.13064106
ENSG00000(	182.708366	1.49661321	0.29144427	5.13516085	2.82E-07	6.25E-06
ENSG00000(	750.706635	0.6159263	0.25379612	2.42685464	0.01523036	0.04867465
ENSG00000(	847.027261	-0.2728408	0.10088949	-2.7043527	0.00684376	0.02555298
ENSG00000(	27925.4917	0.19574294	0.08841168	2.21399422	0.02682918	0.0754268
ENSG00000(	106.593039	0.95165726	0.23861727	3.9882162	6.66E-05	0.00059023
ENSG00000(	397.204423	0.55249611	0.14196893	3.89166908	9.96E-05	0.00082275
ENSG00000(	1117.70883	0.35716011	0.11711687	3.04960437	0.00229143	0.0104653
ENSG00000(	2466.80569	-0.3630399	0.09590263	-3.7855057	0.0001534	0.00116531
ENSG00000(	10269.8293	0.76052907	0.2447961	3.10678589	0.00189133	0.00902198
ENSG00000(	171467.72	0.39403087	0.14644108	2.69071259	0.00712996	0.02640381
ENSG00000(	643.999905	-0.4362592	0.09789056	-4.4566015	8.33E-06	0.00010704
ENSG00000(	1017.41293	-0.1834543	0.06571554	-2.7916424	0.00524413	0.02059475
ENSG00000(	1293.84291	0.23600308	0.07304496	3.23092884	0.00123389	0.00641612
ENSG00000(	4157.59317	-0.5117872	0.07954561	-6.4338831	1.24E-10	9.79E-09
ENSG00000(	354.868562	-0.5278782	0.10055712	-5.2495355	1.52E-07	3.77E-06
ENSG00000(	59.2331747	1.31614979	0.27119089	4.85322271	1.21E-06	2.12E-05
ENSG00000(	1345.99645	-0.3791285	0.11144849	-3.4018269	0.00066937	0.00386958
ENSG00000(	2630.3969	0.23092265	0.07073173	3.26476735	0.00109554	0.00582494
ENSG00000(	3499.50784	0.37576241	0.15979067	2.35159172	0.01869328	0.0573506
ENSG00000(	205.422782	-0.3191918	0.09470503	-3.370379	0.00075065	0.00426868
ENSG00000(	58.5883638	-1.0139233	0.29714058	-3.4122682	0.00064425	0.00375431
ENSG00000(	2265.89907	0.2356042	0.05660208	4.16246514	3.15E-05	0.00032074
ENSG00000(	909.900211	-0.2064207	0.07666855	-2.6923783	0.00709444	0.02632436
ENSG00000(	614.939057	-1.4803998	0.2841074	-5.2107048	1.88E-07	4.50E-06
ENSG00000(	20751.4699	0.3201738	0.13652614	2.34514653	0.0190196	0.05806764
ENSG00000(	2138.62442	-0.4235242	0.11995426	-3.5307142	0.00041444	0.00262477
ENSG00000(	9073.01336	-0.1045639	0.05711726	-1.8306877	0.06714717	0.14981689
ENSG00000(	4469.55428	-0.2885888	0.06877488	-4.1961361	2.72E-05	0.00028483
ENSG00000(	28227.5152	0.16682016	0.09312461	1.79136489	0.07323476	0.15992643
ENSG00000(	1661.14981	0.24777415	0.08665772	2.85922766	0.00424674	0.01727675
ENSG00000(	69.1897702	0.6051168	0.15242631	3.96989746	7.19E-05	0.00063246
ENSG00000(	2359.72265	0.48499609	0.11212171	4.32562155	1.52E-05	0.00017646
ENSG00000(	2025.1688	0.50063781	0.17477055	2.86454329	0.00417611	0.01703246
ENSG00000(	92.4941871	0.5398546	0.1526291	3.53703592	0.00040464	0.00257222
ENSG00000(	131.143953	-1.0918329	0.25738326	-4.242051	2.21E-05	0.00023999
ENSG00000(	178.992428	-0.7835214	0.18416502	-4.2544528	2.10E-05	0.00022895
ENSG00000(	1888.57975	0.14574251	0.05231174	2.78603826	0.00533566	0.02087434
ENSG00000(	14372.2468	0.20592609	0.06034485	3.41248844	0.00064373	0.00375278

ENSG00000(	150.903342	0.37482955	0.12775507	2.93396995	0.00334657	0.01425094
ENSG00000(	212.011891	0.39295451	0.08694617	4.51951478	6.20E-06	8.41E-05
ENSG00000(	9591.42893	-0.2509793	0.0806559	-3.1117286	0.00185995	0.00891043
ENSG00000(	23187.3651	0.68776337	0.12591865	5.46196577	4.71E-08	1.45E-06
ENSG00000(	123.683337	1.05053635	0.19713865	5.32892136	9.88E-08	2.65E-06
ENSG00000(	3740.19218	0.20242409	0.11923289	1.69772014	0.0895606	0.18558515
ENSG00000(	25660.7242	0.28158951	0.13455818	2.09269699	0.03637621	0.09517468
ENSG00000(	799.154702	0.15992195	0.04140537	3.86234835	0.0001123	0.0009089
ENSG00000(	2409.94011	-0.2937504	0.13163052	-2.2316286	0.02563952	0.07284561
ENSG00000(	102.478307	1.56884666	0.26710417	5.87353862	4.27E-09	1.94E-07
ENSG00000(	2196.13888	-0.3486473	0.08905716	-3.9148715	9.05E-05	0.00075876
ENSG00000(	5194.81714	0.64302808	0.21478828	2.99377639	0.00275548	0.0121407
ENSG00000(	2137.50389	0.22902949	0.08095778	2.82899926	0.00466938	0.01870205
ENSG00000(	2769.33761	0.16649846	0.09317087	1.7870227	0.07393382	0.1610148
ENSG00000(	154.075052	1.47013099	0.23864337	6.16036803	7.26E-10	4.21E-08
ENSG00000(	415.62922	-0.1335199	0.07353472	-1.8157391	0.06941041	0.15359148
ENSG00000(	1164.77444	-0.2022656	0.07093511	-2.8514173	0.00435248	0.01763759
ENSG00000(	1775.65203	-0.2084587	0.06962571	-2.99399	0.00275355	0.01213958
ENSG00000(	8390.70255	0.25562603	0.11364435	2.249351	0.02449017	0.0704366
ENSG00000(	764.756917	0.19228742	0.07521034	2.55666211	0.01056818	0.036444
ENSG00000(	239.855308	-0.2461943	0.11519204	-2.1372508	0.0325776	0.08734992
ENSG00000(	2327.36653	-0.2408376	0.08744858	-2.7540476	0.00588632	0.02255956
ENSG00000(	1127.82064	-0.3574571	0.11584065	-3.0857653	0.00203029	0.00949719
ENSG00000(	1678.01819	0.28417817	0.0775239	3.66568438	0.00024668	0.00171151
ENSG00000(	2794.02597	0.23877379	0.11756035	2.03107407	0.04224748	0.10623837
ENSG00000(	2086.11151	0.10009584	0.0478776	2.09066107	0.03655846	0.09553073
ENSG00000(	459.377608	0.25389518	0.0819195	3.09932535	0.00193962	0.00919518
ENSG00000(	684.941443	2.02988033	0.37271894	5.44614215	5.15E-08	1.56E-06
ENSG00000(	24511.1734	1.15620895	0.3474752	3.32745747	0.00087642	0.00483686
ENSG00000(	3848.01036	0.59178028	0.19174539	3.08628159	0.00202677	0.0094958
ENSG00000(	381.946198	0.26580372	0.09237923	2.87731028	0.00401081	0.01646956
ENSG00000(	439.546174	-0.2450257	0.11553135	-2.1208588	0.03393369	0.09020134
ENSG00000(	11265.2636	-0.6091112	0.11046046	-5.5142913	3.50E-08	1.15E-06
ENSG00000(	2770.56959	0.15733125	0.06938838	2.2674006	0.02336576	0.0677813
ENSG00000(	1421.21861	-0.2407796	0.07724977	-3.1168967	0.00182766	0.00878767
ENSG00000(	177.696375	0.40431917	0.14187757	2.84977517	0.00437501	0.0176992
ENSG00000(	4132.54615	0.63537046	0.14007519	4.53592422	5.74E-06	7.94E-05
ENSG00000(	3252.60411	0.26283762	0.1055784	2.48950176	0.01279223	0.04234941
ENSG00000(	1502.50833	-0.2559466	0.07088481	-3.6107397	0.00030533	0.00204401
ENSG00000(	12568.2339	0.4357705	0.16301101	2.67325801	0.00751185	0.02755041
ENSG00000(	356.573928	0.21428462	0.12862162	1.66600779	0.09571186	0.19493572
ENSG00000(	338.164989	0.54356105	0.15659913	3.47103498	0.00051846	0.0031479
ENSG00000(	442.483893	0.34102276	0.08290621	4.11335607	3.90E-05	0.00037965

ENSG00000C	6573.36504	-0.4514128	0.13140033	-3.4354007	0.00059168	0.00350292
ENSG00000C	905.192722	-0.2864195	0.07865249	-3.6415823	0.00027097	0.00185867
ENSG00000C	3399.61306	0.39078748	0.10754293	3.63378118	0.0002793	0.00190373
ENSG00000C	60.7729648	-0.5412141	0.24195996	-2.2367918	0.02529995	0.07209291
ENSG00000C	110.51892	-0.8472542	0.21130239	-4.0096765	6.08E-05	0.00054915
ENSG00000C	925.08542	-0.3239578	0.09330826	-3.4719089	0.00051677	0.00314241
ENSG00000C	23805.4411	0.20599987	0.11908253	1.72989157	0.08364965	0.17636916
ENSG00000C	167.207722	0.2436493	0.09546214	2.55231336	0.01070102	0.03682321
ENSG00000C	82.8708602	-0.301313	0.13096954	-2.3006341	0.02141232	0.06357867
ENSG00000C	1651.14604	-0.3302197	0.08383715	-3.9388226	8.19E-05	0.00070067
ENSG00000C	2270.60158	-0.2203759	0.1253553	-1.7580105	0.07874572	0.16829336
ENSG00000C	8814.65867	-0.2902471	0.07043783	-4.1206144	3.78E-05	0.00037079
ENSG00000C	1607.97273	0.29601813	0.12159926	2.43437446	0.01491755	0.04791809
ENSG00000C	1695.18571	-0.3716702	0.10348902	-3.5913977	0.00032891	0.00217476
ENSG00000C	6851.37606	-0.1876695	0.09147026	-2.0517001	0.04019881	0.10267282
ENSG00000C	74.2481021	-0.3839404	0.1772556	-2.1660267	0.03030914	0.08268975
ENSG00000C	6570.80625	0.74702931	0.13260761	5.63338185	1.77E-08	6.36E-07
ENSG00000C	1767.53669	-0.2157856	0.07981913	-2.7034323	0.00686274	0.02561725
ENSG00000C	2148.64941	0.13860092	0.07482739	1.85227523	0.06398631	0.14500469
ENSG00000C	6162.1784	-0.1503963	0.07442318	-2.0208257	0.04329782	0.10809104
ENSG00000C	520.719237	-0.2781405	0.08880924	-3.1318869	0.00173687	0.00842666
ENSG00000C	69.1323562	0.7458801	0.29665514	2.51430024	0.01192688	0.04010789
ENSG00000C	750.262434	0.73880243	0.17628306	4.19100067	2.78E-05	0.00028862
ENSG00000C	10795.2772	-0.2600417	0.12682085	-2.0504646	0.04031911	0.10279306
ENSG00000C	1484.15058	-0.2661865	0.07150279	-3.7227424	0.00019707	0.00142435
ENSG00000C	9964.47943	-0.4021413	0.12878234	-3.1226434	0.00179235	0.00865813
ENSG00000C	53.1331119	-1.4821191	0.31439792	-4.7141506	2.43E-06	3.88E-05
ENSG00000C	364.394595	0.11870481	0.06134562	1.93501685	0.05298823	0.12531903
ENSG000001	1085.08389	-0.4035524	0.07098794	-5.6848016	1.31E-08	4.93E-07
ENSG000001	3185.31128	-0.1565693	0.05309103	-2.9490719	0.0031873	0.01371381
ENSG000001	322.194022	0.75305128	0.22568851	3.33668419	0.00084784	0.00471324
ENSG000001	1603.15218	-0.3729824	0.07408872	-5.0342667	4.80E-07	9.80E-06
ENSG000001	1990.11415	-0.1889598	0.09362407	-2.0182824	0.04356185	0.10847985
ENSG000001	106.32797	0.19530655	0.11612192	1.68190931	0.09258643	0.19036326
ENSG000001	9783.49702	-0.185299	0.07401771	-2.5034412	0.01229921	0.04107203
ENSG000001	1587.88153	-0.5294752	0.1083827	-4.8852371	1.03E-06	1.85E-05
ENSG000001	2683.64433	-0.1694188	0.09474163	-1.7882196	0.07374059	0.1606436
ENSG000001	2084.16382	-0.2756733	0.11582898	-2.380003	0.0173125	0.05374034
ENSG000001	55.2782861	-0.9714107	0.23461002	-4.1405336	3.46E-05	0.00034643
ENSG000001	10568.892	0.57192291	0.14611853	3.91410249	9.07E-05	0.0007603
ENSG000001	12149.9369	-0.2076429	0.08787038	-2.3630592	0.01812478	0.0558051
ENSG000001	2033.69138	-0.3837315	0.07339385	-5.228388	1.71E-07	4.17E-06
ENSG000001	7757.19387	0.20234937	0.11693118	1.73049968	0.08354104	0.1762741

ENSG000001	691.669366	-0.1389192	0.08331567	-1.6673842	0.09543803	0.19446002
ENSG000001	7209.90573	-0.4579008	0.10746609	-4.2608858	2.04E-05	0.00022398
ENSG000001	201.734921	0.27710354	0.08995673	3.08040929	0.00206716	0.00965098
ENSG000001	991.358896	-0.1909201	0.06010378	-3.1765082	0.0014906	0.00744373
ENSG000001	1069.90013	0.30164184	0.09270118	3.25391599	0.00113826	0.00600946
ENSG000001	77.5253793	-0.7358918	0.15604185	-4.7159898	2.41E-06	3.85E-05
ENSG000001	104.821418	0.85747845	0.22941257	3.73771348	0.0001857	0.00135461
ENSG000001	90782.4698	0.1680564	0.09989196	1.68238167	0.09249485	0.19025599
ENSG000001	124.561774	-0.2033984	0.0953163	-2.1339307	0.03284845	0.08791345
ENSG000001	256.760867	-0.2263581	0.08375014	-2.7027785	0.00687626	0.02566108
ENSG000001	3023.03795	0.26945949	0.09205789	2.92706577	0.00342177	0.0145072
ENSG000001	2673.3484	-0.1255389	0.05720906	-2.1943882	0.02820751	0.07822401
ENSG000001	8690.81925	0.67270346	0.12749675	5.27623994	1.32E-07	3.36E-06
ENSG000001	6700.26452	0.16651706	0.06096512	2.73134957	0.00630755	0.02387083
ENSG000001	6121.16211	-0.337385	0.09871247	-3.4178561	0.00063116	0.0036989
ENSG000001	613.345313	0.3708659	0.10189653	3.63963236	0.00027303	0.00186661
ENSG000001	207.357942	-0.3446572	0.09581655	-3.5970529	0.00032184	0.00213584
ENSG000001	5455.2746	-0.209357	0.07825683	-2.675255	0.00746724	0.0274007
ENSG000001	8245.53721	0.36841452	0.13410168	2.74727745	0.00600923	0.02291542
ENSG000001	108.469966	-0.3498051	0.13292682	-2.6315617	0.00849934	0.03056111
ENSG000001	291.881999	0.37641664	0.09299252	4.04781646	5.17E-05	0.00048133
ENSG000001	143.479414	0.39121016	0.16080514	2.4328212	0.0149817	0.0481028
ENSG000001	3143.11078	-0.2971883	0.0817145	-3.6369098	0.00027593	0.00188467
ENSG000001	225.831513	0.80938244	0.21003018	3.85364832	0.00011637	0.00093763
ENSG000001	1837.52209	-0.2782577	0.07754807	-3.5881969	0.00033297	0.00219462
ENSG000001	275.597051	-0.4377699	0.18921111	-2.3136587	0.02068645	0.06192703
ENSG000001	788.492868	0.1731639	0.10340712	1.674584	0.09401588	0.19250997
ENSG000001	2193.86636	-0.1687181	0.07575245	-2.227229	0.02593197	0.0734604
ENSG000001	9310.84166	-0.2685921	0.15427666	-1.7409769	0.08168763	0.17322214
ENSG000001	2726.72528	0.28216514	0.15442434	1.82720639	0.06766874	0.15070209
ENSG000001	2204.76488	-0.1343458	0.0595982	-2.2541919	0.0241841	0.06970852
ENSG000001	86.6564245	0.81339842	0.24596342	3.30698945	0.00094304	0.00515348
ENSG000001	6352.45025	0.7218723	0.18438751	3.91497392	9.04E-05	0.00075876
ENSG000001	2836.3004	0.19491015	0.08128392	2.397893	0.01648968	0.05175174
ENSG000001	134.202098	-0.566587	0.14511696	-3.9043475	9.45E-05	0.00078799
ENSG000001	15103.5147	0.36045043	0.17764596	2.02903818	0.0424544	0.10657372
ENSG000001	1673.50426	-0.5353024	0.06422098	-8.33532	7.73E-17	2.54E-14
ENSG000001	8583.62582	-0.663915	0.14751321	-4.5007158	6.77E-06	9.06E-05
ENSG000001	1300.95459	0.6054208	0.14782944	4.09540092	4.21E-05	0.00040648
ENSG000001	1762.88541	-0.1596618	0.08020479	-1.9906766	0.04651645	0.11379329
ENSG000001	4151.40276	-0.1956127	0.08277165	-2.3632812	0.01811392	0.05579764
ENSG000001	605.547562	0.50939263	0.12338071	4.1286245	3.65E-05	0.00036039
ENSG000001	2084.09411	-0.3099351	0.08404293	-3.6878191	0.00022618	0.00159297

ENSG000001	967.371174	-0.216169	0.08344348	-2.5906038	0.00958077	0.03368038
ENSG000001	2080.01549	0.147827	0.07103731	2.08097691	0.03743602	0.09733231
ENSG000001	880.453463	-0.1256013	0.07441865	-1.6877666	0.09145604	0.18854756
ENSG000001	7851.7581	-0.1463229	0.06043754	-2.4210595	0.01547534	0.04926199
ENSG000001	2757.40311	-0.2940016	0.06663923	-4.4118402	1.02E-05	0.00012735
ENSG000001	1231.82364	-0.2620668	0.0666504	-3.9319615	8.43E-05	0.00071671
ENSG000001	6973.23006	0.22712373	0.06101869	3.72219959	0.00019749	0.00142627
ENSG000001	792.31171	0.17620837	0.09849944	1.78892767	0.07362646	0.16056399
ENSG000001	514.412481	2.66598369	0.44384984	6.00649909	1.90E-09	9.47E-08
ENSG000001	5626.24164	1.01091716	0.30158634	3.35199915	0.0008023	0.00450722
ENSG000001	1428.81707	0.26855266	0.12667241	2.12005651	0.03400128	0.09036444
ENSG000001	91.0757242	0.55712646	0.26226015	2.1243276	0.03364276	0.08960887
ENSG000001	451.84718	-0.2031418	0.07414914	-2.7396377	0.00615069	0.0233749
ENSG000001	70.459529	0.4642615	0.16044374	2.89360939	0.00380842	0.01582235
ENSG000001	11941.2926	0.28456601	0.13463961	2.11353858	0.0345547	0.09154972
ENSG000001	1165.65838	0.55995047	0.13895116	4.02983643	5.58E-05	0.0005121
ENSG000001	2209.0408	0.22427799	0.12253302	1.83034728	0.06719803	0.14990727
ENSG000001	37.4185926	1.09814535	0.26767363	4.1025534	4.09E-05	0.00039596
ENSG000001	344.040074	0.21998344	0.11711325	1.87838216	0.0603289	0.13852255
ENSG000001	2211.89315	0.41844625	0.08120715	5.15282513	2.57E-07	5.80E-06
ENSG000001	2734.34022	0.6662557	0.10618306	6.27459489	3.51E-10	2.27E-08
ENSG000001	2301.06724	0.34751687	0.16437683	2.11414758	0.03450266	0.09147879
ENSG000001	3573.077	-0.1986739	0.07600983	-2.6137916	0.00895436	0.03194333
ENSG000001	10221.5982	0.87605797	0.17340522	5.05208531	4.37E-07	9.03E-06
ENSG000001	280.389971	-0.3326197	0.12714241	-2.6161195	0.00889354	0.03174199
ENSG000001	3805.41285	0.16816332	0.06374783	2.63794597	0.00834099	0.03008112
ENSG000001	2141.78899	0.40580567	0.17745513	2.28680725	0.02220708	0.06520347
ENSG000001	38.6992714	-0.420219	0.19920729	-2.1094559	0.03490525	0.09226449
ENSG000001	1475.80271	0.60319275	0.11301966	5.33706022	9.45E-08	2.55E-06
ENSG000001	18899.279	-0.1513947	0.04608714	-3.2849674	0.00101994	0.00550108
ENSG000001	8234.50636	0.25647461	0.14999578	1.70987876	0.0872883	0.18205378
ENSG000001	1018.20673	-0.2137567	0.06481408	-3.2979974	0.00097377	0.00529741
ENSG000001	13230.763	0.56748342	0.16621085	3.4142381	0.00063961	0.00373478
ENSG000001	923.222622	0.39378832	0.12874191	3.0587422	0.00222268	0.01022223
ENSG000001	579.118027	0.55065237	0.10209093	5.39374448	6.90E-08	1.98E-06
ENSG000001	2186.29713	-0.236568	0.12869048	-1.8382716	0.06602239	0.14815155
ENSG000001	5646.48954	-0.1550325	0.05282779	-2.9346766	0.00333895	0.01422689
ENSG000001	1070.98927	0.33512945	0.10466303	3.20198497	0.00136484	0.00694421
ENSG000001	6252.32146	-0.2370587	0.05073361	-4.6726162	2.97E-06	4.56E-05
ENSG000001	18065.8357	0.14862427	0.05312341	2.79771688	0.00514652	0.02025472
ENSG000001	1204.98678	0.26484749	0.06474654	4.09052758	4.30E-05	0.00041319
ENSG000001	2663.46425	0.13246328	0.05786167	2.28930973	0.02206136	0.06488083
ENSG000001	272.75797	0.27507221	0.10298661	2.67095127	0.00756366	0.02770538

ENSG000001	1313.2035	0.40159525	0.09272113	4.33121633	1.48E-05	0.00017255
ENSG000001	339.196379	0.66521395	0.13024286	5.10748875	3.26E-07	7.05E-06
ENSG000001	3277.8509	0.50793575	0.13503071	3.7616312	0.00016881	0.00126196
ENSG000001	880.167419	1.06023248	0.16851946	6.29145411	3.15E-10	2.10E-08
ENSG000001	1436.37005	0.33632203	0.10354308	3.24813637	0.00116164	0.00610839
ENSG000001	5031.65808	-0.162975	0.08859069	-1.8396403	0.06582107	0.14776846
ENSG000001	1270.28482	-0.2300265	0.08537444	-2.694325	0.00705314	0.02619297
ENSG000001	14758.7276	0.71400177	0.36129428	1.97623324	0.04812837	0.1166336
ENSG000001	3774.12222	-0.2320714	0.08998591	-2.5789753	0.00990939	0.03460879
ENSG000001	1125.74867	-0.1949382	0.08530199	-2.285271	0.02229694	0.06536135
ENSG000001	74.6648349	0.64205913	0.19757781	3.24965198	0.00115546	0.00607814
ENSG000001	1496.45314	-0.1362157	0.07732093	-1.7616924	0.07812128	0.16734333
ENSG000001	630.368937	1.26732498	0.29530405	4.29159362	1.77E-05	0.00019968
ENSG000001	2402.01674	-0.255855	0.08472477	-3.0198372	0.00252911	0.01134691
ENSG000001	2567.6165	-0.3918371	0.11206473	-3.4965245	0.00047136	0.00292321
ENSG000001	3093.81668	0.2918523	0.10027985	2.91037822	0.00360992	0.01513657
ENSG000001	4197.76002	-0.2928217	0.06371749	-4.5956252	4.31E-06	6.24E-05
ENSG000001	47.5886047	0.75612914	0.29065445	2.60147109	0.00928249	0.03291126
ENSG000001	1533.6191	-0.098767	0.04812203	-2.052429	0.04012799	0.10250999
ENSG000001	2092.06144	-0.2109297	0.04998041	-4.2202468	2.44E-05	0.00026124
ENSG000001	16157.7859	0.31968038	0.12245069	2.61068668	0.00903606	0.03217932
ENSG000001	3152.67687	-0.21052	0.05356508	-3.9301718	8.49E-05	0.00072125
ENSG000001	5371.83615	-0.2433953	0.09078573	-2.6809862	0.00734055	0.02701794
ENSG000001	2931.55706	0.24565764	0.08873271	2.76851265	0.00563128	0.02178381
ENSG000001	56.7211701	0.65855225	0.20543778	3.2056044	0.00134779	0.00688001
ENSG000001	289.18495	0.87596805	0.21539659	4.06676841	4.77E-05	0.00045048
ENSG000001	3556.62089	-0.2281272	0.06000043	-3.8020923	0.00014348	0.00110563
ENSG000001	6559.88082	0.13165976	0.06675273	1.97235031	0.04856963	0.11744034
ENSG000001	153.234933	0.30517178	0.09819534	3.10780309	0.00188484	0.00899691
ENSG000001	9810.95061	-0.2131949	0.12548875	-1.6989166	0.0893349	0.18529601
ENSG000001	60.7854358	-0.7372103	0.25076477	-2.939848	0.00328373	0.01401221
ENSG000001	23526.2288	0.43349621	0.16670212	2.60042415	0.00931086	0.0329957
ENSG000001	253.985266	0.28097019	0.097628	2.87796742	0.00400246	0.01644461
ENSG000001	2537.37266	-0.3086431	0.0497432	-6.2047297	5.48E-10	3.34E-08
ENSG000001	8156.69055	-0.1795605	0.09739223	-1.8436843	0.06522916	0.1469863
ENSG000001	703.612524	-0.4458665	0.14386334	-3.0992362	0.0019402	0.00919518
ENSG000001	3176.94715	-0.1478127	0.08201763	-1.8022061	0.07151298	0.15716813
ENSG000001	167.653472	0.82645539	0.14200252	5.82000516	5.88E-09	2.57E-07
ENSG000001	2819.33677	0.14949145	0.08293715	1.80246669	0.07147201	0.15711912
ENSG000001	1429.63382	-0.1380628	0.06808049	-2.0279356	0.04256682	0.10680039
ENSG000001	28207.7787	0.26461137	0.10054264	2.63183244	0.00849257	0.03055175
ENSG000001	1281.89373	0.1604764	0.06805717	2.35796447	0.01837545	0.0564953
ENSG000001	249.442285	0.27234648	0.13942011	1.9534232	0.05076947	0.12120918

ENSG000001	1431.40787	-0.1203965	0.07214623	-1.6687844	0.09516011	0.19405751
ENSG000001	256.873511	-0.2757439	0.14264909	-1.9330224	0.05323344	0.1257448
ENSG000001	352.051269	0.64522637	0.18144876	3.55597007	0.00037659	0.0024258
ENSG000001	1797.78442	-0.2040287	0.05464141	-3.7339574	0.00018849	0.00137084
ENSG000001	626.235875	-0.2772661	0.08010778	-3.4611625	0.00053785	0.00324253
ENSG000001	1628.39368	1.6110842	0.35267443	4.56819106	4.92E-06	6.98E-05
ENSG000001	725.184392	-0.1907749	0.07944958	-2.4012066	0.01634111	0.05138841
ENSG000001	62.8510827	0.28904037	0.15511963	1.86333846	0.06241466	0.14221472
ENSG000001	3597.49688	0.27167074	0.13261429	2.04857822	0.04050338	0.103051
ENSG000001	515.354763	-0.1570903	0.08589106	-1.828948	0.0674074	0.15025866
ENSG000001	1303.43742	0.19791414	0.06250822	3.16621001	0.00154439	0.00765582
ENSG000001	933.905229	-0.1190396	0.05887367	-2.0219501	0.0431815	0.10787557
ENSG000001	358.654569	-0.3486785	0.09161584	-3.8058756	0.0001413	0.00109293
ENSG000001	1114.97649	0.34817182	0.0988359	3.5227262	0.00042713	0.00268763
ENSG000001	12508.7485	0.24215353	0.12841182	1.88575733	0.05932766	0.13690345
ENSG000001	44909.7711	0.73433927	0.16846549	4.35898937	1.31E-05	0.00015529
ENSG000001	295.757201	0.27230209	0.12157035	2.23987251	0.0250992	0.07171837
ENSG000001	25213.2094	0.5361713	0.10156042	5.27933331	1.30E-07	3.33E-06
ENSG000001	152.55543	-0.153054	0.09045474	-1.6920509	0.09063627	0.18725722
ENSG000001	1850.66248	0.20080031	0.09848901	2.03880939	0.04146906	0.10471429
ENSG000001	2546.14966	-0.1285461	0.06658867	-1.9304502	0.05355108	0.12639206
ENSG000001	789.548065	-0.3397091	0.10810726	-3.1423337	0.00167607	0.00819481
ENSG000001	1896.86513	-0.2935778	0.09632492	-3.047787	0.00230533	0.01051554
ENSG000001	421.523468	0.60166295	0.12797197	4.70152127	2.58E-06	4.07E-05
ENSG000001	457.230593	-0.364265	0.15369785	-2.3700071	0.01778774	0.05492136
ENSG000001	986.535805	0.32345082	0.07421241	4.35844678	1.31E-05	0.0001555
ENSG000001	684.863249	0.21088009	0.06069977	3.47414973	0.00051247	0.00312236
ENSG000001	846.263394	0.59606408	0.18322245	3.25322625	0.00114103	0.00601968
ENSG000001	1037.5309	0.12797707	0.06268521	2.04158324	0.04119289	0.10421959
ENSG000001	517.395459	0.30160421	0.11914387	2.53142873	0.01135989	0.03855921
ENSG000001	12581.6307	0.30346009	0.17414972	1.74252412	0.08141677	0.17279297
ENSG000001	507.299322	-0.1947305	0.0874061	-2.2278819	0.02588839	0.07337145
ENSG000001	2709.34456	0.20271856	0.0753028	2.69204539	0.00710153	0.02633218
ENSG000001	2070.1212	0.25385817	0.12301581	2.0636223	0.03905354	0.1004338
ENSG000001	1833.34095	-0.2339326	0.08816017	-2.6534955	0.00796628	0.02896776
ENSG000001	131.593146	0.48042103	0.12977154	3.70205246	0.00021386	0.00152172
ENSG000001	9343.11767	0.3506373	0.13030426	2.69091213	0.0071257	0.02639477
ENSG000001	764.155559	0.993092	0.31836482	3.11935216	0.00181249	0.00872925
ENSG000001	27.5666753	0.65002678	0.28339037	2.29375038	0.02180484	0.06440339
ENSG000001	118.234063	-0.2664195	0.14010097	-1.9016252	0.05722017	0.13302171
ENSG000001	3403.08894	-0.206571	0.07338455	-2.8149111	0.00487907	0.01942378
ENSG000001	550.679649	1.00544558	0.16489355	6.09754328	1.08E-09	5.87E-08
ENSG000001	4141.47812	0.37947402	0.12539546	3.02621808	0.00247634	0.01113775

ENSG000001	2097.05033	-0.1542627	0.0902777	-1.7087579	0.08749581	0.18237811
ENSG000001	2187.03631	-0.3615602	0.08679623	-4.1656211	3.11E-05	0.00031701
ENSG000001	4145.36336	0.11751044	0.07017025	1.67464751	0.09400341	0.19250997
ENSG000001	3844.35346	0.3191729	0.16559441	1.92743763	0.05392511	0.12692333
ENSG000001	2170.31216	-0.5236909	0.09372617	-5.5874564	2.30E-08	8.02E-07
ENSG000001	1764.47922	-0.1576972	0.07972159	-1.9780989	0.04791755	0.11629779
ENSG000001	1236.49091	-0.6183789	0.15290527	-4.0441963	5.25E-05	0.00048788
ENSG000001	10470.8037	0.28014767	0.16692386	1.6782962	0.09328929	0.19148228
ENSG000001	175.59923	0.46646614	0.11150474	4.1833751	2.87E-05	0.00029678
ENSG000001	552.224399	-0.2116377	0.08670223	-2.4409719	0.0146478	0.04725064
ENSG000001	3397.86553	-0.137781	0.07888425	-1.7466225	0.08070284	0.17152905
ENSG000001	8117.00974	0.21012201	0.07893694	2.66189701	0.00777016	0.02838285
ENSG000001	681.019616	0.29054997	0.09410069	3.08764977	0.00201746	0.00946469
ENSG000001	832.573832	-0.1793284	0.09272707	-1.9339383	0.05312072	0.12556042
ENSG000001	131.94597	-0.5099174	0.14276918	-3.571621	0.00035478	0.00230997
ENSG000001	11442.1622	1.16122082	0.19016102	6.10651335	1.02E-09	5.61E-08
ENSG000001	2610.97815	0.22834404	0.10512974	2.17202135	0.02985405	0.0816191
ENSG000001	414.922591	0.21797875	0.07033572	3.09911873	0.00194097	0.00919518
ENSG000001	316.355805	0.25867895	0.06844918	3.77913891	0.00015737	0.00118866
ENSG000001	2600.33989	-0.2628928	0.04618965	-5.6915958	1.26E-08	4.81E-07
ENSG000001	8073.83435	0.27797271	0.13266489	2.09529964	0.03614436	0.09475632
ENSG000001	5208.66141	0.21424106	0.08936541	2.39735998	0.01651369	0.05180464
ENSG000001	768.833141	0.15502324	0.07742918	2.00212954	0.0452708	0.11168905
ENSG000001	1243.54338	-0.1442737	0.06861588	-2.1026284	0.03549826	0.09348542
ENSG000001	138.170873	0.49695651	0.27888828	1.78191967	0.07476233	0.16228377
ENSG000001	1257.17844	-0.3692096	0.12287269	-3.0048138	0.00265743	0.01180928
ENSG000001	877.591464	0.87165389	0.23020061	3.78649682	0.00015279	0.00116251
ENSG000001	256.568921	0.20859858	0.11677808	1.786282	0.07405361	0.16107887
ENSG000001	1509.66923	0.29207265	0.16437903	1.77682429	0.07559715	0.16350791
ENSG000001	539.211187	0.17924209	0.10083988	1.777492	0.07548733	0.16345424
ENSG000001	1377.82095	3.05226379	0.47910508	6.37076067	1.88E-10	1.42E-08
ENSG000001	4.55096778	-0.7587388	0.32130756	-2.3614099	0.0182056	0.05603239
ENSG000001	392.177216	-0.3207548	0.09617691	-3.3350499	0.00085284	0.00473227
ENSG000001	288.220606	0.30026986	0.11157657	2.69115521	0.00712051	0.02638427
ENSG000001	1099.78742	0.37560257	0.13976407	2.68740445	0.00720097	0.02661915
ENSG000001	479.77582	0.19595667	0.08733147	2.2438266	0.02484356	0.07117039
ENSG000001	3085.38926	0.16523895	0.06431016	2.56940652	0.01018729	0.03540015
ENSG000001	1756.82994	0.59581561	0.11987926	4.97013099	6.69E-07	1.29E-05
ENSG000001	1543.4117	-0.5924848	0.09316244	-6.359696	2.02E-10	1.50E-08
ENSG000001	3239.13623	0.17906039	0.08635648	2.07350267	0.03812552	0.09869987
ENSG000001	158.804226	-0.4046017	0.08981873	-4.504648	6.65E-06	8.93E-05
ENSG000001	3882.87057	-0.3313701	0.07526893	-4.4024814	1.07E-05	0.00013172
ENSG000001	1519.58665	-0.1934878	0.09119405	-2.1217156	0.03386162	0.09005936

ENSG000001	9615.75625	0.36866379	0.13568897	2.71697689	0.00658812	0.02476443
ENSG000001	97.928159	-0.3140466	0.09109234	-3.4475635	0.00056567	0.00337652
ENSG000001	702.175274	-0.1433333	0.07432527	-1.9284597	0.05379798	0.12676416
ENSG000001	1303.81447	-1.017791	0.22120355	-4.6011513	4.20E-06	6.10E-05
ENSG000001	516.833835	0.20487785	0.05577402	3.67335632	0.00023939	0.0016689
ENSG000001	2132.64658	-0.1716305	0.06038767	-2.8421446	0.00448112	0.0180478
ENSG000001	404.935828	0.51845374	0.10816055	4.79337198	1.64E-06	2.75E-05
ENSG000001	6220.92341	-0.3665883	0.21446887	-1.7092843	0.0873983	0.18223074
ENSG000001	947.207973	0.59269203	0.26650838	2.22391519	0.02615415	0.07382993
ENSG000001	1310.9353	-0.2320474	0.12144297	-1.9107524	0.0560364	0.13085096
ENSG000001	847.280159	-0.2335359	0.11604295	-2.0124953	0.04416775	0.1095085
ENSG000001	4299.75775	0.18958479	0.08178596	2.31806027	0.02044605	0.06141444
ENSG000001	1357.67319	-0.1894587	0.09264505	-2.0449954	0.04085531	0.10356866
ENSG000001	1162.95804	-0.1942729	0.09965797	-1.9493961	0.05124814	0.12211049
ENSG000001	87.0165936	0.44263913	0.15463787	2.86242391	0.00420414	0.01712832
ENSG000001	4564.44175	-0.3730008	0.05456423	-6.8359962	8.14E-12	8.61E-10
ENSG000001	17158.9796	0.52131732	0.1494268	3.48878059	0.00048523	0.00299359
ENSG000001	72.480088	-0.3900965	0.13066295	-2.9855172	0.00283099	0.01241301
ENSG000001	6345.95939	-0.2998992	0.12942046	-2.3172475	0.02049025	0.06146909
ENSG000001	585.235827	-0.2366665	0.06249526	-3.7869518	0.00015251	0.00116153
ENSG000001	1509.95167	-0.1358573	0.06522698	-2.0828397	0.03726584	0.09697687
ENSG000001	666.94568	-1.5219741	0.40520086	-3.7560978	0.00017258	0.00128216
ENSG000001	4774.92583	0.21811231	0.12131036	1.79796939	0.07218186	0.1583672
ENSG000001	6264.34223	-0.1931251	0.09149553	-2.1107594	0.03479299	0.09206321
ENSG000001	225.69507	-0.1588665	0.06540227	-2.4290664	0.01513776	0.04847958
ENSG000001	287.815331	-0.1868267	0.0810905	-2.3039286	0.02122665	0.06318276
ENSG000001	228.511043	0.12177999	0.04689955	2.59661326	0.00941479	0.03326634
ENSG000001	1799.90945	-0.1546498	0.08966441	-1.724762	0.08457037	0.17774133
ENSG000001	1166.22349	-0.3282108	0.1301981	-2.5208566	0.01170695	0.03949666
ENSG000001	61.7347817	-0.6372762	0.21595848	-2.9509199	0.00316829	0.01364824
ENSG000001	54.2571293	0.71204361	0.25894383	2.74979946	0.00596317	0.0227938
ENSG000001	1064.13512	0.25644052	0.11160273	2.2977979	0.02157329	0.06391254
ENSG000001	1661.80357	-0.232097	0.0799053	-2.9046505	0.00367663	0.01538064
ENSG000001	66482.9477	0.48585781	0.20231316	2.40151363	0.0163274	0.05136483
ENSG000001	1831.31174	0.18417536	0.04966506	3.708349	0.00020862	0.00149024
ENSG000001	1968.4451	0.16690144	0.09944434	1.67834025	0.09328069	0.19148228
ENSG000001	6775.69391	0.10870331	0.06157127	1.76548757	0.07748186	0.16636722
ENSG000001	1774.25408	0.26237593	0.06322077	4.150154	3.32E-05	0.00033566
ENSG000001	5252.27315	0.15077169	0.08166184	1.84629306	0.06484966	0.14629042
ENSG000001	8688.2489	0.23216089	0.1074022	2.16160272	0.03064881	0.08336638
ENSG000001	529.622827	-0.2275943	0.10992933	-2.0703694	0.03841777	0.09915521
ENSG000001	1066.44223	-0.3012953	0.08310292	-3.6255681	0.00028833	0.00195187
ENSG000001	8586.10091	0.20927262	0.11827312	1.76940132	0.07682692	0.16545141

ENSG000001	1031.64418	0.6251879	0.15300389	4.08609152	4.39E-05	0.00042033
ENSG000001	2307.86374	-0.154781	0.09049206	-1.7104378	0.08718495	0.1818906
ENSG000001	5105.25675	0.37071739	0.12945718	2.86362956	0.00418817	0.01707686
ENSG000001	8255.78549	0.45500744	0.09414254	4.83317567	1.34E-06	2.32E-05
ENSG000001	166.721918	-0.3037286	0.12044647	-2.5216897	0.01167927	0.03944918
ENSG000001	409.233431	-0.1551508	0.08469713	-1.8318307	0.06697665	0.14956122
ENSG000001	1145.94175	-0.2922875	0.06605924	-4.4246268	9.66E-06	0.00012149
ENSG000001	4612.10341	0.61523833	0.13034014	4.72025217	2.36E-06	3.79E-05
ENSG000001	5223.20693	0.1967327	0.08320552	2.36441898	0.01805838	0.0556612
ENSG000001	69.8613478	0.40382835	0.16534503	2.44233738	0.0145925	0.0471668
ENSG000001	18.1399694	2.20225524	0.47921796	4.59551897	4.32E-06	6.24E-05
ENSG000001	2767.30154	0.22839268	0.11784143	1.93813565	0.05260668	0.12456896
ENSG000001	318.184922	-0.1572292	0.08847924	-1.7770185	0.07556519	0.16350791
ENSG000001	182.227121	0.37093585	0.13857368	2.67681313	0.00743261	0.02728743
ENSG000001	6867.22023	0.30140981	0.11922974	2.52797508	0.01147225	0.03891325
ENSG000001	649.195789	0.38475595	0.09433234	4.07872772	4.53E-05	0.00043102
ENSG000001	1306.70214	-0.3044752	0.0667279	-4.5629372	5.04E-06	7.13E-05
ENSG000001	752.650381	-0.3724688	0.14884635	-2.5023714	0.01233644	0.04116031
ENSG000001	378.571699	0.2648527	0.1445472	1.83229211	0.06690791	0.14951333
ENSG000001	2079.97016	0.22509091	0.10418059	2.16058396	0.03072749	0.08348634
ENSG000001	21.2281029	0.57973332	0.2673055	2.16880433	0.03009754	0.08223748
ENSG000001	75.3619758	0.20210107	0.1125419	1.7957851	0.0725287	0.15886352
ENSG000001	281.060389	-0.172475	0.10000573	-1.7246512	0.08459036	0.17775754
ENSG000001	5614.81863	-0.1237351	0.06454573	-1.9170148	0.05523605	0.12933646
ENSG000001	275.860622	0.66782387	0.09929828	6.72543249	1.75E-11	1.67E-09
ENSG000001	3387.87306	-0.1468333	0.08509126	-1.7255974	0.08441987	0.17751239
ENSG000001	198.133819	0.17057668	0.08637093	1.97493155	0.04827592	0.11689838
ENSG000001	1321.12787	-0.1223761	0.06779451	-1.8051027	0.07105861	0.15648748
ENSG000001	724.242764	0.45620507	0.15813416	2.88492417	0.00391508	0.0161543
ENSG000001	22017.0971	0.26628646	0.12345266	2.15699254	0.03100625	0.08405454
ENSG000001	8108.24961	-0.2003572	0.08292453	-2.4161396	0.01568604	0.04977957
ENSG000001	1442.50867	-0.3393376	0.11890374	-2.8538845	0.00431882	0.01752571
ENSG000001	2391.57031	-0.1685446	0.06526739	-2.5823704	0.00981242	0.03431978
ENSG000001	1056.90831	-0.2125626	0.10259095	-2.0719431	0.03827075	0.09891674
ENSG000001	840.697132	-0.3024725	0.05488101	-5.5114242	3.56E-08	1.17E-06
ENSG000001	59.2104252	-0.7105158	0.20236747	-3.5110181	0.00044639	0.00278746
ENSG000001	4621.77742	-0.3223109	0.08445953	-3.8161583	0.00013555	0.001058
ENSG000001	4366.94777	0.13933934	0.07694868	1.81080879	0.07017045	0.154997
ENSG000001	6455.24459	-0.3287908	0.07656124	-4.2944813	1.75E-05	0.00019756
ENSG000001	127.194738	-0.3976441	0.1181721	-3.3649574	0.00076555	0.00433565
ENSG000001	65.2454742	0.48650838	0.19426751	2.50432196	0.01226863	0.04100962
ENSG000001	719.081029	-0.2330942	0.09092912	-2.5634717	0.01036312	0.03589062
ENSG000001	3875.04362	-0.4454455	0.10863242	-4.1004842	4.12E-05	0.00039898

ENSG000001	8767.55957	0.41940111	0.13762166	3.04749345	0.00230759	0.01051919
ENSG000001	751.756619	1.57276115	0.29971337	5.24755089	1.54E-07	3.80E-06
ENSG000001	1369.3188	-0.6574574	0.28253857	-2.3269652	0.01996712	0.0603261
ENSG000001	3675.78292	-0.1727927	0.08150808	-2.1199462	0.03401058	0.09037259
ENSG000001	19492.2112	-0.2009307	0.1109258	-1.8113969	0.07007943	0.15483311
ENSG000001	904.32161	-0.1742038	0.09314948	-1.8701533	0.06146253	0.14062029
ENSG000001	994.265239	-0.154418	0.07827106	-1.9728616	0.04851133	0.11734573
ENSG000001	105.090182	-0.2970082	0.17279614	-1.7188357	0.0856443	0.17947765
ENSG000001	5079.82994	0.13644732	0.06096499	2.23812569	0.02521286	0.0719438
ENSG000001	433.641708	-0.1778649	0.06644293	-2.6769579	0.0074294	0.02728743
ENSG000001	500.81213	-0.6523292	0.14560846	-4.4800225	7.46E-06	9.78E-05
ENSG000001	448.227972	-0.2465329	0.09300077	-2.6508699	0.00802848	0.02915732
ENSG000001	1495.09561	0.20107011	0.06708192	2.99738163	0.0027231	0.01203095
ENSG000001	18036.6871	-0.2449462	0.12715693	-1.92633	0.05406318	0.12722764
ENSG000001	1102.38883	-0.2996817	0.11680144	-2.5657364	0.01029571	0.03569128
ENSG000001	241.082705	-0.3282934	0.10366573	-3.1668464	0.00154102	0.00765069
ENSG000001	3376.05858	-0.7550688	0.27909176	-2.7054501	0.00682119	0.02548184
ENSG000001	3920.58263	-0.6659467	0.12559616	-5.3022851	1.14E-07	3.01E-06
ENSG000001	5760.01436	-0.3232184	0.10856132	-2.9772886	0.0029081	0.01271263
ENSG000001	3546.97917	0.14928423	0.08432038	1.77044072	0.07665375	0.16515213
ENSG000001	169.673655	-0.2529905	0.13468865	-1.8783355	0.06033528	0.13852255
ENSG000001	1800.96032	-0.2848468	0.12875863	-2.2122541	0.02694911	0.07559429
ENSG000001	13488.9556	0.30275729	0.1369806	2.21022027	0.02708988	0.07583596
ENSG000001	283.315321	-0.2949598	0.1607075	-1.8353827	0.06644901	0.14876321
ENSG000001	13543.7163	-0.3454438	0.07713441	-4.4784654	7.52E-06	9.84E-05
ENSG000001	38474.8769	0.59308182	0.15961993	3.71558763	0.00020273	0.00145467
ENSG000001	1346.43803	0.74823123	0.230576	3.24505248	0.00117429	0.00616598
ENSG000001	391.892071	-1.4799906	0.38287241	-3.865493	0.00011087	0.00089978
ENSG000001	6814.7514	-0.4444295	0.24004299	-1.851458	0.0641037	0.14519547
ENSG000001	68.9735081	0.31375854	0.1574535	1.99270608	0.04629364	0.11343298
ENSG000001	23762.3839	0.26406974	0.10291935	2.56579299	0.01029402	0.03569128
ENSG000001	8501.52879	-0.1456922	0.07290242	-1.9984551	0.04566735	0.11236923
ENSG000001	8892.01025	0.70680475	0.1672032	4.22722025	2.37E-05	0.00025427
ENSG000001	183.360977	-0.8757539	0.17198141	-5.0921428	3.54E-07	7.54E-06
ENSG000001	2821.2189	-0.2062134	0.0808938	-2.5491862	0.01079746	0.03709339
ENSG000001	1311.59236	-0.1981144	0.09208324	-2.1514705	0.03143908	0.08500896
ENSG000001	18520.1646	0.25997825	0.11382082	2.28410104	0.02236559	0.06550957
ENSG000001	1691.6184	-0.2248244	0.0968879	-2.3204595	0.02031604	0.06109994
ENSG000001	991.38248	0.45739383	0.22953933	1.99265996	0.04629869	0.11343298
ENSG000001	42.1443582	1.33409293	0.34521132	3.86456889	0.00011129	0.00090167
ENSG000001	1110.5596	-0.6114833	0.10825646	-5.6484691	1.62E-08	5.91E-07
ENSG000001	751.374981	-0.2509108	0.09542728	-2.629341	0.00855505	0.03071843
ENSG000001	8301.85909	-0.3114611	0.0925958	-3.3636639	0.00076915	0.00435092

ENSG000001	1125.01054	-0.1539469	0.08036313	-1.9156413	0.05541077	0.12966175
ENSG000001	9.71795987	1.30551053	0.42808136	3.04967853	0.00229086	0.0104653
ENSG000001	2947.21994	0.20049901	0.11874937	1.68842172	0.0913303	0.18844924
ENSG000001	98.4360028	-0.2668176	0.1013162	-2.6335136	0.00845065	0.03043871
ENSG000001	334.874109	-0.2171282	0.09634516	-2.2536495	0.02421822	0.06977912
ENSG000001	27309.4973	0.45980588	0.10924214	4.20905228	2.56E-05	0.00027181
ENSG000001	5915.30485	-0.1225477	0.06389323	-1.918008	0.05511	0.12916656
ENSG000001	107.13943	-0.3835991	0.09225173	-4.1581777	3.21E-05	0.00032636
ENSG000001	853.286952	-0.1718965	0.1016558	-1.6909658	0.09084333	0.18765826
ENSG000001	1770.02456	-0.234716	0.08225247	-2.8536044	0.00432263	0.01753626
ENSG000001	825.037648	-0.2964421	0.14598353	-2.0306545	0.04229005	0.10630852
ENSG000001	30.4886263	1.22082792	0.26622083	4.58577162	4.52E-06	6.51E-05
ENSG000001	488.548243	0.66879097	0.17036878	3.92554883	8.65E-05	0.00073096
ENSG000001	365.712275	-0.3375871	0.12384635	-2.7258546	0.00641352	0.02419602
ENSG000001	4654.4896	-0.1785833	0.10217046	-1.747896	0.08048205	0.17113509
ENSG000001	1144.67524	-0.4296381	0.09338949	-4.6004972	4.21E-06	6.11E-05
ENSG000001	1373.7379	-0.2343608	0.09338071	-2.5097348	0.01208219	0.04047979
ENSG000001	480.385394	0.62874968	0.18297148	3.43632605	0.00058966	0.00349526
ENSG000001	1775.08392	-0.2651071	0.07637452	-3.4711459	0.00051824	0.0031479
ENSG000001	3997.80592	0.25985706	0.09129821	2.84624474	0.00442382	0.01785674
ENSG000001	62314.4205	0.49818532	0.19238572	2.58951299	0.00961118	0.03374628
ENSG000001	284.885552	-0.2247634	0.09917897	-2.266241	0.02343663	0.06794606
ENSG000001	43.2195728	-1.0702321	0.33090037	-3.2343031	0.0012194	0.00634763
ENSG000001	1473.2069	0.25137847	0.126809	1.98233938	0.04744127	0.11545544
ENSG000001	2225.48757	-0.2831651	0.05791609	-4.8892296	1.01E-06	1.82E-05
ENSG000001	252.709049	-0.395534	0.13182558	-3.0004342	0.00269595	0.01194745
ENSG000001	12761.3334	0.70796286	0.15984904	4.42894663	9.47E-06	0.00011971
ENSG000001	488.230377	1.36629908	0.32403146	4.21656306	2.48E-05	0.00026404
ENSG000001	7136.00104	-0.151472	0.09099517	-1.6646161	0.09598938	0.19541851
ENSG000001	2243.62985	0.24885525	0.13781787	1.80568192	0.07096804	0.15634336
ENSG000001	1837.33247	0.74601783	0.20004326	3.72928255	0.00019203	0.00139163
ENSG000001	3895.65919	-0.1118087	0.04691676	-2.3831301	0.01716613	0.0534491
ENSG000001	76.8845986	1.59286824	0.35424114	4.49656477	6.91E-06	9.22E-05
ENSG000001	4374.2781	-0.1712814	0.10237285	-1.6731134	0.09430498	0.19281999
ENSG000001	12090.4603	-0.3660507	0.09980903	-3.667511	0.00024492	0.00170259
ENSG000001	541.550854	-0.3126843	0.08016065	-3.9007199	9.59E-05	0.00079759
ENSG000001	5803.36448	0.348428	0.10185527	3.42081467	0.00062434	0.0036678
ENSG000001	1660.79534	1.47254667	0.36489454	4.03554045	5.45E-05	0.0005021
ENSG000001	1189.47455	0.2358333	0.08477953	2.78172453	0.00540709	0.02109411
ENSG000001	4057.19961	-0.2251626	0.0562486	-4.0029905	6.25E-05	0.00056106
ENSG000001	3067.33435	0.35839528	0.09191846	3.89905655	9.66E-05	0.00080171
ENSG000001	1375.68034	-0.5306147	0.14793837	-3.5867281	0.00033485	0.00220501
ENSG000001	1411.94368	0.35778405	0.09517078	3.75938961	0.00017033	0.00127128

ENSG000001	3248.94213	0.14671505	0.07352189	1.99552866	0.04598525	0.11280609
ENSG000001	5130.60083	-0.3017709	0.09023367	-3.3443273	0.00082482	0.00461538
ENSG000001	666.74108	0.20295786	0.11228497	1.80752479	0.07068049	0.15587583
ENSG000001	1339.68822	-0.3520273	0.06857574	-5.133409	2.85E-07	6.28E-06
ENSG000001	29.851631	-0.8557563	0.40107249	-2.1336699	0.03286982	0.08794851
ENSG000001	1255.87639	-0.4309703	0.12084547	-3.5662925	0.00036207	0.00235003
ENSG000001	437.12653	0.30036878	0.11703815	2.56641769	0.0102755	0.0356383
ENSG000001	675.273383	0.34576709	0.09262685	3.73290328	0.00018929	0.00137521
ENSG000001	3190.08928	0.87332297	0.15040268	5.80656522	6.38E-09	2.73E-07
ENSG000001	1541.51703	-0.2707422	0.08614328	-3.1429285	0.00167267	0.00818093
ENSG000001	589.883133	0.4006192	0.08633551	4.64025982	3.48E-06	5.21E-05
ENSG000001	2555.67947	0.18260529	0.07990171	2.28537406	0.0222909	0.06535688
ENSG000001	1729.6441	0.18978795	0.06903495	2.74915775	0.00597486	0.02282041
ENSG000001	1494.55582	-0.2179561	0.10004422	-2.1785972	0.0293616	0.08071376
ENSG000001	1534.35106	-0.1171063	0.05214779	-2.2456617	0.02472568	0.07095899
ENSG000001	589.322539	0.37967661	0.12202066	3.11157653	0.00186091	0.00891208
ENSG000001	375.267133	-0.2100704	0.08860807	-2.3707821	0.01775049	0.05481803
ENSG000001	2704.05235	-0.1469215	0.06597409	-2.2269575	0.02595012	0.07349742
ENSG000001	116.609622	-0.3197725	0.12181508	-2.6250653	0.00866324	0.03103469
ENSG000001	375.708467	-0.6414815	0.22750547	-2.8196311	0.00480789	0.01919842
ENSG000001	4669.8638	0.57900501	0.12703794	4.55773295	5.17E-06	7.27E-05
ENSG000001	254.507377	0.29422638	0.10020809	2.93615385	0.00332309	0.01416348
ENSG000001	2128.61978	0.37388287	0.10012464	3.73417433	0.00018833	0.00137084
ENSG000001	2590.38032	0.85574528	0.17030511	5.02477743	5.04E-07	1.02E-05
ENSG000001	1021.26477	-0.4264154	0.09321819	-4.5743794	4.78E-06	6.83E-05
ENSG000001	5872.68636	-0.1999934	0.04674701	-4.2782062	1.88E-05	0.00020963
ENSG000001	581.933154	0.18555524	0.06257126	2.96550253	0.00302189	0.01311507
ENSG000001	2640.89474	0.45510867	0.11261302	4.04135023	5.31E-05	0.00049353
ENSG000001	436.410871	-0.554087	0.18774122	-2.9513339	0.00316405	0.01364213
ENSG000001	1463.23388	-0.2637149	0.10811095	-2.4392989	0.01471579	0.04739385
ENSG000001	5315.91645	0.14775309	0.05729536	2.5787967	0.00991451	0.03461835
ENSG000001	1055.79667	-0.1172938	0.06026467	-1.946311	0.0516174	0.12277458
ENSG000001	794.204651	-0.2410839	0.11430491	-2.1091295	0.0349334	0.09231671
ENSG000001	1131.04638	-0.2694515	0.07298365	-3.6919438	0.00022255	0.0015714
ENSG000001	1746.47358	0.38344899	0.0947392	4.04741662	5.18E-05	0.00048184
ENSG000001	1938.36891	-0.1761802	0.09169923	-1.9212835	0.05469598	0.12840392
ENSG000001	234.498117	0.69029406	0.28426218	2.42837109	0.01516682	0.04855727
ENSG000001	23483.4192	0.39730116	0.2073817	1.91579665	0.05539098	0.12963638
ENSG000001	7908.72555	0.78539537	0.14200172	5.53088628	3.19E-08	1.06E-06
ENSG000001	1180.46182	0.39095976	0.12481465	3.13232257	0.00173429	0.00842263
ENSG000001	4610.43874	0.25175044	0.11996273	2.09857222	0.03585463	0.09415585
ENSG000001	2808.76573	0.22618338	0.0823474	2.74669718	0.00601987	0.02294996
ENSG000001	272.089797	0.39599734	0.13062846	3.03147821	0.00243359	0.01098302

ENSG000001	2148.47465	0.45967693	0.09778369	4.7009573	2.59E-06	4.08E-05
ENSG000001	1053.1931	-0.5935343	0.11794691	-5.032216	4.85E-07	9.88E-06
ENSG000001	609.558342	0.32732775	0.08256599	3.96443786	7.36E-05	0.00064399
ENSG000001	2253.47715	-0.1963152	0.05801449	-3.3839004	0.00071464	0.00409857
ENSG000001	1922.7886	-0.1813565	0.06155245	-2.9463727	0.00321525	0.01378988
ENSG000001	27205.6219	0.29157817	0.13391264	2.17737611	0.02945252	0.08084097
ENSG000001	1336.65816	-0.3043744	0.08811144	-3.4544252	0.00055147	0.00330675
ENSG000001	1395.48727	-0.3171842	0.06410235	-4.9480906	7.49E-07	1.42E-05
ENSG000001	11717.3965	1.66871747	0.23112531	7.21996851	5.20E-13	7.69E-11
ENSG000001	7114.91985	-0.2615735	0.10127373	-2.5828363	0.00979918	0.03428175
ENSG000001	1890.77602	0.9197229	0.17662958	5.20707166	1.92E-07	4.55E-06
ENSG000001	12817.3411	0.33771598	0.09599846	3.51793128	0.00043492	0.00272996
ENSG000001	8805.56545	0.51780576	0.12038679	4.30118435	1.70E-05	0.00019243
ENSG000001	740.166674	-0.232418	0.04944502	-4.700533	2.59E-06	4.08E-05
ENSG000001	796.789195	0.31674338	0.05399991	5.86562797	4.47E-09	2.02E-07
ENSG000001	1566.65315	-0.3591732	0.15513184	-2.3152768	0.02059779	0.06171668
ENSG000001	1152.16905	-0.7136968	0.12764213	-5.5913894	2.25E-08	7.89E-07
ENSG000001	664.343997	-0.3029444	0.10302165	-2.9405898	0.00327588	0.01399106
ENSG000001	2537.50119	0.5665	0.20279366	2.79347985	0.00521443	0.02048859
ENSG000001	7921.19043	-0.1604581	0.07366989	-2.1780687	0.02940092	0.08074524
ENSG000001	14.9719003	1.32534327	0.32703048	4.05265972	5.06E-05	0.0004736
ENSG000001	529.918561	0.17859433	0.07911491	2.25740426	0.02398283	0.06923858
ENSG000001	1287.74157	0.14003888	0.07068605	1.98113893	0.0475757	0.11571714
ENSG000001	1133.32109	-0.2515048	0.06652071	-3.7808491	0.00015629	0.00118299
ENSG000001	1916.54324	-0.1763604	0.05880018	-2.9993163	0.00270586	0.01197967
ENSG000001	804.200768	-0.1375407	0.07090657	-1.9397457	0.05241061	0.12422651
ENSG000001	1432.57294	-0.2848357	0.12921545	-2.2043472	0.02749993	0.07670599
ENSG000001	3672.28196	0.32480133	0.11086197	2.92978136	0.00339201	0.01439789
ENSG000001	111609.776	0.29764777	0.18031465	1.65071314	0.09879716	0.19955621
ENSG000001	17955.446	-0.5826652	0.10871201	-5.3597133	8.34E-08	2.30E-06
ENSG000001	2565.10495	-0.1094084	0.04403053	-2.4848293	0.01296136	0.04281148
ENSG000001	407.473381	-0.2065641	0.08148454	-2.5350101	0.01124441	0.03829284
ENSG000001	283.092953	0.3356165	0.18208647	1.84317102	0.06530405	0.1470864
ENSG000001	1158.16532	-0.1671711	0.098208	-1.7022143	0.08871519	0.18423409
ENSG000001	2019.1655	-0.2597435	0.07674137	-3.3846605	0.00071266	0.00409047
ENSG000001	382.937171	0.41828616	0.1527914	2.73762898	0.00618838	0.02349964
ENSG000001	1457.57325	-0.2775653	0.06488623	-4.2777228	1.89E-05	0.00020993
ENSG000001	1500.04012	0.14584351	0.06735924	2.16515971	0.03037546	0.082825
ENSG000001	1200.0573	-0.3275532	0.10202166	-3.2106237	0.00132447	0.00678248
ENSG000001	1176.45662	0.20746748	0.09094804	2.28116505	0.02253868	0.06587712
ENSG000001	642.841298	-0.2367729	0.06353977	-3.7263742	0.00019425	0.00140567
ENSG000001	491.594327	-0.4865012	0.20956851	-2.3214423	0.02026298	0.06099001
ENSG000001	743.999446	-0.2908246	0.11988569	-2.4258494	0.01527261	0.04877306

ENSG000001	1801.65555	-0.1206039	0.06977894	-1.7283712	0.08392169	0.17683979
ENSG000001	2169.83225	0.17729439	0.09236195	1.91956082	0.0549134	0.12880997
ENSG000001	1079.95887	0.18959094	0.07995679	2.37116754	0.01773199	0.05478425
ENSG000001	4742.69437	0.20347041	0.1208802	1.68324011	0.09232862	0.19004067
ENSG000001	7529.96069	-0.2260466	0.09515377	-2.375593	0.01752077	0.05427057
ENSG000001	232.45062	0.48870814	0.17444185	2.80155333	0.00508572	0.02008091
ENSG000001	46765.8552	-0.3359238	0.11932718	-2.8151487	0.00487547	0.0194201
ENSG000001	273.653211	0.4023251	0.11119995	3.61803293	0.00029685	0.00199558
ENSG000001	11106.8717	0.26586973	0.12615752	2.10744257	0.03507924	0.09257065
ENSG000001	4244.45155	-0.1526348	0.07339585	-2.079611	0.03756123	0.0975528
ENSG000001	2520.46222	0.68297915	0.30208362	2.26089432	0.0237658	0.06870788
ENSG000001	7452.68233	-0.2269531	0.05488303	-4.1352143	3.55E-05	0.00035236
ENSG000001	3598.91203	-0.254214	0.06957433	-3.6538478	0.00025834	0.00178217
ENSG000001	911.726191	0.41022486	0.13915973	2.94787046	0.00319971	0.01375088
ENSG000001	513.566119	2.10851852	0.31598056	6.67293756	2.51E-11	2.28E-09
ENSG000001	6915.24573	0.56775454	0.1626928	3.48973358	0.0004835	0.00298572
ENSG000001	22405.0853	0.51676664	0.14517992	3.55949112	0.00037157	0.00239991
ENSG000001	2218.95223	0.26896241	0.07324243	3.67222138	0.00024045	0.00167553
ENSG000001	2210.31379	0.42834581	0.13636854	3.14108964	0.00168321	0.00822415
ENSG000001	4076.56484	0.26155597	0.11285514	2.31762568	0.02046967	0.06145492
ENSG000001	47374.2506	-0.194342	0.10844936	-1.7920071	0.07313183	0.15977572
ENSG000001	961.063031	-0.2561028	0.09923517	-2.5807661	0.00985814	0.03446304
ENSG000001	1309.33707	-0.1533088	0.08686465	-1.7649157	0.07757793	0.16640086
ENSG000001	274.361384	0.12831918	0.06884252	1.86395237	0.06232839	0.1420405
ENSG000001	1523.26857	-0.1469438	0.07388282	-1.9888766	0.04671483	0.11419807
ENSG000001	58.3456591	0.33311967	0.15972759	2.08554866	0.03701953	0.09648749
ENSG000001	2822.28016	0.12051687	0.04859227	2.48016571	0.01313213	0.04326705
ENSG000001	5048.93388	1.71612106	0.26835264	6.395022	1.61E-10	1.24E-08
ENSG000001	120.811442	3.21853165	0.48261874	6.66889079	2.58E-11	2.33E-09
ENSG000001	34.3341344	3.21633732	0.49548374	6.49130748	8.51E-11	6.96E-09
ENSG000001	330.084802	-0.157884	0.05486634	-2.8776112	0.00400699	0.01645853
ENSG000001	6396.87518	1.8449988	0.23585632	7.82255409	5.18E-15	1.18E-12
ENSG000001	135.564099	0.25944746	0.13496574	1.92232083	0.0545654	0.12813891
ENSG000001	183.806925	0.33350575	0.10926507	3.05226312	0.00227123	0.0103927
ENSG000001	4660.64376	0.15158604	0.07996994	1.8955378	0.05802119	0.13442395
ENSG000001	10910.2988	0.19773947	0.08131794	2.43168313	0.01502885	0.04823282
ENSG000001	14014.6637	-0.3094788	0.09329527	-3.3171966	0.00090926	0.00500281
ENSG000001	733.086939	0.39624498	0.103149	3.84148144	0.00012229	0.00097613
ENSG000001	1836.58714	-0.4292488	0.09973099	-4.3040667	1.68E-05	0.00019069
ENSG000001	2387.8333	-0.1948827	0.08711363	-2.2371094	0.02527919	0.07208734
ENSG000001	661.323952	0.26620435	0.12109406	2.19832704	0.02792581	0.07766581
ENSG000001	51.9430372	-0.7111303	0.19568773	-3.6340054	0.00027906	0.00190333
ENSG000001	3214.77845	0.27500477	0.0938585	2.92999321	0.00338969	0.01439651

ENSG000001	4585.53791	-0.2392226	0.09558329	-2.5027659	0.0123227	0.04112394
ENSG000001	1316.0795	0.32436302	0.11192919	2.89793044	0.00375634	0.0156399
ENSG000001	16035.9592	-0.2254915	0.13016533	-1.7323467	0.08321185	0.17577867
ENSG000001	163.580265	0.72863364	0.2378819	3.06300576	0.00219126	0.01011624
ENSG000001	148.370324	-0.185127	0.09882377	-1.8733042	0.06102639	0.13993182
ENSG000001	3027.63976	1.21094645	0.20683462	5.85466037	4.78E-09	2.14E-07
ENSG000001	987.157956	0.27464698	0.10062069	2.72952798	0.00634251	0.0239718
ENSG000001	13361.8374	0.55726503	0.14173163	3.93183241	8.43E-05	0.00071671
ENSG000001	14084.6289	-0.2339554	0.11930659	-1.9609594	0.04988376	0.11976276
ENSG000001	1743.16865	-0.1620858	0.07353723	-2.2041322	0.02751505	0.07671487
ENSG000001	661.000743	0.30365054	0.06333961	4.79400701	1.63E-06	2.75E-05
ENSG000001	51.007055	-1.5899882	0.27923212	-5.6941452	1.24E-08	4.76E-07
ENSG000001	872.759251	-0.7630924	0.13005978	-5.867244	4.43E-09	2.01E-07
ENSG000001	7609.6981	-0.1859863	0.07954512	-2.3381231	0.01938086	0.05889879
ENSG000001	4017.42427	0.15660017	0.09092226	1.7223523	0.08500572	0.17842333
ENSG000001	2216.86163	-0.1291685	0.06549988	-1.9720412	0.0486049	0.11746212
ENSG000001	4319.73793	0.10456621	0.04029438	2.59505688	0.00945753	0.03335229
ENSG000001	1917.5601	0.41378297	0.15338145	2.69773799	0.00698124	0.02599242
ENSG000001	1866.20624	0.45003218	0.09313739	4.83191722	1.35E-06	2.33E-05
ENSG000001	27.1519508	1.04856241	0.367088	2.85643334	0.0042843	0.01740512
ENSG000001	21797.0018	0.16484146	0.09609115	1.71546976	0.08625913	0.18042681
ENSG000001	3028.86024	0.22392012	0.11875263	1.88560136	0.05934869	0.13693017
ENSG000001	1319.64257	-0.5565082	0.13155493	-4.2302346	2.33E-05	0.00025126
ENSG000001	1322.16561	-0.1504391	0.05108526	-2.9448635	0.00323097	0.01385203
ENSG000001	3782.19384	0.45826652	0.22181152	2.06601762	0.03882682	0.10001495
ENSG000001	1974.89343	0.73916653	0.25038162	2.9521597	0.0031556	0.01361785
ENSG000001	156.390413	-0.3636677	0.13937072	-2.6093553	0.0090713	0.03227306
ENSG000001	1406.21497	-0.146672	0.0614635	-2.3863272	0.0170176	0.0531166
ENSG000001	449.86917	-0.1873295	0.09023664	-2.0759804	0.03789577	0.09822795
ENSG000001	240.831255	0.18172952	0.07663715	2.37129812	0.01772573	0.05477658
ENSG000001	1712.61217	0.21946355	0.12476799	1.75897316	0.07858207	0.16810668
ENSG000001	83.6399666	-0.4518327	0.19313478	-2.3394684	0.0193112	0.05871174
ENSG000001	2508.2588	-0.1111177	0.06077144	-1.8284533	0.06748155	0.15033143
ENSG000001	9493.08613	0.62403395	0.15929695	3.91742567	8.95E-05	0.00075339
ENSG000001	128.447924	1.69232369	0.27929826	6.05919889	1.37E-09	7.15E-08
ENSG000001	3666.4179	0.51794175	0.13190045	3.92676267	8.61E-05	0.00072941
ENSG000001	2313.73397	0.83400328	0.38688975	2.15566135	0.03111012	0.08427305
ENSG000001	89.819975	-0.252435	0.13406027	-1.8829963	0.05970087	0.13741444
ENSG000001	852.941631	0.95546575	0.27690881	3.45047072	0.00055961	0.0033445
ENSG000001	144.869083	0.25560053	0.11067725	2.30942252	0.02092015	0.06245006
ENSG000001	7768.02941	0.31579667	0.17476586	1.80697009	0.07076694	0.15600993
ENSG000001	2404.18447	-0.1829424	0.05935737	-3.0820504	0.0020558	0.00960102
ENSG000001	9738.05393	0.75794804	0.16967651	4.46701819	7.93E-06	0.00010287

ENSG000001	458.39164	0.10941006	0.06542603	1.67227105	0.09447089	0.1929271
ENSG000001	1068.52918	0.1766395	0.05678812	3.11050091	0.0018677	0.00892984
ENSG000001	10887.5016	0.48029919	0.11989695	4.00593334	6.18E-05	0.00055653
ENSG000001	584.7346	1.26114703	0.2128537	5.92494772	3.12E-09	1.48E-07
ENSG000001	3351.74468	-0.2482901	0.11799338	-2.1042716	0.03535476	0.09315832
ENSG000001	399.247032	-0.4454484	0.1043152	-4.270216	1.95E-05	0.00021584
ENSG000001	4177.75543	-0.4076704	0.10241648	-3.9805157	6.88E-05	0.00060856
ENSG000001	3277.61688	-0.5353239	0.09580385	-5.5877079	2.30E-08	8.02E-07
ENSG000001	719.621581	-0.1432027	0.06426527	-2.2283069	0.02586006	0.07334273
ENSG000001	3862.77316	1.5191796	0.37307774	4.07201885	4.66E-05	0.00044073
ENSG000001	437.152003	0.16193216	0.08106872	1.99746783	0.04577439	0.11249084
ENSG000001	2403.09895	0.13482019	0.0509814	2.64449741	0.00818123	0.02963038
ENSG000001	4647.58066	0.2286068	0.12955103	1.76460814	0.07762965	0.16648715
ENSG000001	143.632464	-0.3435554	0.1664565	-2.063935	0.03902388	0.10037978
ENSG000001	3940.05861	0.38768341	0.1148912	3.3743525	0.0007399	0.00422003
ENSG000001	10576.8793	-0.1235813	0.07032143	-1.7573777	0.07885346	0.16840685
ENSG000001	775.822668	-0.4970702	0.17146372	-2.8989818	0.00374377	0.015594
ENSG000001	4.91429096	0.48053603	0.27988506	1.71690492	0.08599655	0.18009116
ENSG000001	5334.44374	-0.5408918	0.14030261	-3.8551796	0.00011564	0.0009323
ENSG000001	181.733106	1.09571817	0.19637394	5.57975343	2.41E-08	8.31E-07
ENSG000001	4903.78829	-0.147338	0.07009173	-2.1020738	0.03554681	0.09354526
ENSG000001	1380.73388	0.15162701	0.09151169	1.65691404	0.09753685	0.19787375
ENSG000001	6118.26985	-0.4375319	0.11735695	-3.7282147	0.00019284	0.00139615
ENSG000001	10809.3244	0.25249188	0.0882851	2.85996024	0.00423694	0.01724173
ENSG000001	44862.2247	0.34703342	0.13758916	2.52224386	0.01166088	0.03942386
ENSG000001	643.165229	0.25978849	0.07198748	3.60880074	0.00030762	0.0020546
ENSG000001	69.7587119	-0.8073066	0.17997724	-4.4856038	7.27E-06	9.59E-05
ENSG000001	87.5086783	-0.2805141	0.1389503	-2.018809	0.04350708	0.10840851
ENSG000001	577.706667	0.51977228	0.21493811	2.41824154	0.01559572	0.04957981
ENSG000001	1675.7291	-0.0854807	0.04526371	-1.8885032	0.05895843	0.13619908
ENSG000001	1703.53925	0.76468242	0.1588429	4.81407987	1.48E-06	2.52E-05
ENSG000001	750.751018	0.40609268	0.09688723	4.19139534	2.77E-05	0.00028854
ENSG000001	2137.39097	-0.3141191	0.05245739	-5.9880816	2.12E-09	1.05E-07
ENSG000001	1217.89146	-0.2814218	0.03892036	-7.2307108	4.80E-13	7.25E-11
ENSG000001	14499.1908	0.42153597	0.15614394	2.69966277	0.00694098	0.02586265
ENSG000001	32002.6996	0.21639705	0.11951222	1.81066878	0.07019214	0.15501121
ENSG000001	6039.06002	-0.2061267	0.0545378	-3.77952	0.00015713	0.00118746
ENSG000001	3669.36497	0.6174191	0.18911774	3.26473387	0.00109567	0.00582494
ENSG000001	309.2031	0.66799735	0.21846153	3.05773445	0.00223017	0.01024366
ENSG000001	825.099488	0.82525589	0.21101175	3.9109475	9.19E-05	0.00076853
ENSG000001	1716.47516	0.21615448	0.09465561	2.28358862	0.02239572	0.0655713
ENSG000001	2102.6667	-0.1803001	0.07696427	-2.3426467	0.0191475	0.05837329
ENSG000001	348.348207	0.46465654	0.16566778	2.80474908	0.00503558	0.01994153

ENSG000001	109.148858	0.3109584	0.16827443	1.84792429	0.0646133	0.14597891
ENSG000001	291.502291	0.31765535	0.10063186	3.15660806	0.00159616	0.00787855
ENSG000001	1520.47685	-0.2123722	0.07413496	-2.8646706	0.00417443	0.01703246
ENSG000001	252.309844	0.43351116	0.16162064	2.68227595	0.00731231	0.02692767
ENSG000001	1048.39179	0.65123035	0.12970557	5.02083569	5.14E-07	1.04E-05
ENSG000001	13032.6611	0.42185693	0.10002693	4.2174336	2.47E-05	0.00026341
ENSG000001	2997.46309	0.32773417	0.08704906	3.76493626	0.00016659	0.00124667
ENSG000001	60.4312264	1.05983525	0.2168091	4.88833371	1.02E-06	1.82E-05
ENSG000001	7513.19551	-0.1794477	0.07680234	-2.3364868	0.01946588	0.05910757
ENSG000001	2293.62667	-0.1948074	0.08556123	-2.2768183	0.02279708	0.06647771
ENSG000001	1859.91934	0.15738099	0.04058647	3.87767081	0.00010546	0.00086269
ENSG000001	60.0331591	1.58777074	0.35960023	4.41537747	1.01E-05	0.0001256
ENSG000001	6114.60058	0.2308311	0.11560294	1.99675805	0.04585147	0.11264207
ENSG000001	96.5641004	0.2276809	0.09154713	2.48703479	0.01288128	0.04259555
ENSG000001	1065.71152	-0.251774	0.10775185	-2.3366098	0.01945948	0.05910191
ENSG000001	59414.6478	3.69032327	0.32146407	11.4797378	1.67E-30	4.83E-27
ENSG000001	43076.4874	2.91521937	0.29860421	9.76282056	1.63E-22	1.24E-19
ENSG000001	3.73592369	0.73423366	0.40082594	1.83180176	0.06698096	0.14956122
ENSG000001	2457.3161	-0.192718	0.05547243	-3.4741228	0.00051253	0.00312236
ENSG000001	1835.36935	-0.1940206	0.05858714	-3.3116584	0.00092745	0.00507783
ENSG000001	500.337567	0.10191852	0.04969706	2.05079586	0.04028683	0.10278889
ENSG000001	211.475585	-0.545694	0.18150495	-3.0064967	0.00264277	0.01175854
ENSG000001	47.0585025	1.07991795	0.28564008	3.78069468	0.00015639	0.0011831
ENSG000001	3512.73997	-0.1167215	0.03786076	-3.0829142	0.00204984	0.00957938
ENSG000001	3265.09284	-0.2165743	0.0660601	-3.2784429	0.00104381	0.00560688
ENSG000001	1702.2287	0.35270764	0.0965739	3.65220471	0.00026	0.00179106
ENSG000001	93.80442	0.1939921	0.07879525	2.46197699	0.01381735	0.04511426
ENSG000001	1169.71124	-0.2479775	0.11216733	-2.2107821	0.02705093	0.0757855
ENSG000001	2164.16119	0.25801322	0.13903939	1.85568431	0.06349857	0.14407263
ENSG000001	2151.30899	-0.188816	0.05718218	-3.302007	0.00095996	0.00522816
ENSG000001	2453.63106	0.2908216	0.05708938	5.09414504	3.50E-07	7.49E-06
ENSG000001	100.118042	0.80297937	0.18505568	4.33912307	1.43E-05	0.0001674
ENSG000001	2585.14562	-0.2530166	0.09609304	-2.6330377	0.00846249	0.03046624
ENSG000001	1839.20838	-0.3902355	0.1310081	-2.9787129	0.00289462	0.01265751
ENSG000001	1458.76913	-0.5012917	0.11722795	-4.2762136	1.90E-05	0.00021103
ENSG000001	410.510512	0.67866708	0.124127	5.46752188	4.56E-08	1.42E-06
ENSG000001	815.187873	0.35472898	0.09607699	3.69213248	0.00022238	0.0015714
ENSG000001	577.079948	-0.1455965	0.08720993	-1.6694946	0.0950194	0.19379784
ENSG000001	3344.22397	0.28960176	0.11223338	2.5803533	0.00986993	0.0344793
ENSG000001	6635.88831	0.45564152	0.09127149	4.99215612	5.97E-07	1.17E-05
ENSG000001	6781.14732	0.37275248	0.14819298	2.51531806	0.0118925	0.04001085
ENSG000001	723.350552	0.51727719	0.1200345	4.30940423	1.64E-05	0.00018693
ENSG000001	1218.2461	0.56673383	0.2406274	2.35523398	0.01851105	0.05688789

ENSG000001	115.670779	0.17195218	0.10018785	1.71629767	0.08610758	0.18021292
ENSG000001	239.780612	0.3402706	0.10079142	3.37598777	0.00073551	0.00420164
ENSG000001	9434.68814	0.44027788	0.19311448	2.27988025	0.02261479	0.0660392
ENSG000001	227.073754	-0.3605597	0.19139241	-1.8838769	0.05958163	0.13723983
ENSG000001	60.7232113	-0.409879	0.17581519	-2.331306	0.01973723	0.05978115
ENSG000001	344.127301	0.60485194	0.18989381	3.18521151	0.00144648	0.00729638
ENSG000001	14805.9311	0.22008326	0.10934518	2.01273863	0.04414213	0.10948247
ENSG000001	789.682636	0.24761574	0.10695772	2.31508063	0.02060852	0.06173529
ENSG000001	1391.56718	0.54836207	0.11085056	4.94685898	7.54E-07	1.42E-05
ENSG000001	1868.28264	0.63881517	0.1378449	4.63430386	3.58E-06	5.33E-05
ENSG000001	75549.5975	0.41808498	0.18039734	2.31757839	0.02047225	0.06145492
ENSG000001	277.029778	0.42552033	0.23816215	1.78668324	0.0739887	0.16107887
ENSG000001	230.857028	-0.4154173	0.15345229	-2.7071428	0.00678651	0.02537845
ENSG000001	150.528994	0.16404915	0.09123498	1.79809496	0.07216196	0.15834752
ENSG000001	2691.66938	-0.3231255	0.06963688	-4.6401491	3.48E-06	5.21E-05
ENSG000001	407.820104	-0.2550942	0.06470086	-3.9426707	8.06E-05	0.00069197
ENSG000001	1532.83037	1.66198346	0.26001878	6.39178229	1.64E-10	1.26E-08
ENSG000001	19629.7092	0.58916372	0.18161067	3.24410304	0.00117821	0.00617761
ENSG000001	32.8216851	0.68414959	0.33082107	2.06803514	0.03863672	0.09961384
ENSG000001	9039.54484	0.14896288	0.05601858	2.65916893	0.00783337	0.02855611
ENSG000001	1108.11936	-0.1675822	0.07463349	-2.2454022	0.02474232	0.07099268
ENSG000001	2647.86915	-0.2105373	0.10050217	-2.0948534	0.03618402	0.09480882
ENSG000001	1324.77226	0.61307596	0.18243665	3.36048685	0.00077805	0.00439098
ENSG000001	778.151113	-0.2110405	0.07667936	-2.7522462	0.0059188	0.02266006
ENSG000001	10481.5184	0.38998699	0.18116819	2.15262402	0.03134824	0.08482293
ENSG000001	672.605293	-0.4825604	0.10575088	-4.5631811	5.04E-06	7.13E-05
ENSG000001	7218.87683	0.14590922	0.08057813	1.8107794	0.07017501	0.154997
ENSG000001	3412.6638	1.02239923	0.26773456	3.81870468	0.00013415	0.00104997
ENSG000001	63240.9528	0.60610235	0.21851895	2.77368329	0.00554256	0.02152106
ENSG000001	864.341607	0.40060868	0.07047515	5.6843963	1.31E-08	4.93E-07
ENSG000001	15042.0918	0.26054062	0.08967709	2.90531964	0.00366878	0.01535673
ENSG000001	227.277452	0.77286159	0.14697741	5.25836976	1.45E-07	3.62E-06
ENSG000001	1249.38851	0.22452674	0.06869586	3.26841731	0.00108151	0.0057687
ENSG000001	12154.299	0.83383033	0.12550137	6.64399386	3.05E-11	2.75E-09
ENSG000001	989.970228	-0.1543628	0.08249824	-1.8711045	0.0613306	0.14045154
ENSG000001	149.074851	-0.7372994	0.23779419	-3.1005777	0.00193144	0.00916799
ENSG000001	8746.27452	0.20867418	0.1081321	1.92980787	0.05363065	0.12651344
ENSG000001	1161.87094	0.34128807	0.11173375	3.05447612	0.00225454	0.01033265
ENSG000001	246.95081	-0.3161293	0.09627679	-3.2835462	0.0010251	0.00552478
ENSG000001	1251.50077	-0.214389	0.10818045	-1.981772	0.04750477	0.11558642
ENSG000001	29078.3564	0.28020327	0.15526115	1.80472231	0.07111815	0.15657881
ENSG000001	287.986094	-0.1824242	0.08977581	-2.0319966	0.042154	0.1060401
ENSG000001	957.60555	-0.1205989	0.03011613	-4.0044619	6.22E-05	0.00055862

ENSG000001	1380.05379	-0.1842681	0.07267419	-2.5355375	0.01122749	0.03824421
ENSG000001	182.107	-0.4804432	0.21440886	-2.2407804	0.0250403	0.07160749
ENSG000001	2980.64414	0.13806913	0.0532766	2.59155297	0.00955438	0.03361959
ENSG000001	5369.40374	0.13458964	0.06952694	1.93579138	0.05289326	0.12512486
ENSG000001	1885.73166	-0.1571735	0.07194429	-2.1846561	0.02891407	0.07974074
ENSG000001	1914.78932	0.67078906	0.11235162	5.97044423	2.37E-09	1.16E-07
ENSG000001	537.931932	-0.154148	0.0842356	-1.8299625	0.06725554	0.14998938
ENSG000001	1258.64502	0.34027917	0.14423498	2.35920005	0.01831438	0.05634326
ENSG000001	64.5624384	1.45296766	0.240459	6.04247558	1.52E-09	7.82E-08
ENSG000001	1215.85883	-1.1779872	0.22945148	-5.1339272	2.84E-07	6.28E-06
ENSG000001	8001.50792	0.39959384	0.10790434	3.70322294	0.00021288	0.0015168
ENSG000001	2658.07486	0.64696727	0.13981858	4.62719081	3.71E-06	5.47E-05
ENSG000001	166.733474	-0.5314187	0.18461467	-2.8785289	0.00399535	0.01642468
ENSG000001	3176.0291	-0.2049401	0.09128197	-2.2451321	0.02475965	0.07102833
ENSG000001	7331.46896	0.2138419	0.10978197	1.94787808	0.05142955	0.12246219
ENSG000001	2648.81213	0.09791383	0.05444588	1.79836975	0.07211843	0.15827598
ENSG000001	132.598664	-0.2359592	0.09074827	-2.6001509	0.00931828	0.03300584
ENSG000001	1777.94102	-0.2017829	0.07387102	-2.7315565	0.00630359	0.02386207
ENSG000001	826.679965	0.15863565	0.081268	1.95200633	0.05093745	0.12151011
ENSG000001	60.3283933	0.92684657	0.25771511	3.59639974	0.00032265	0.00213925
ENSG000001	1814.16937	-0.2781626	0.06993664	-3.977351	6.97E-05	0.00061559
ENSG000001	4601.2266	-0.2880156	0.07582153	-3.7985984	0.00014552	0.00111954
ENSG000001	1922.20355	-0.1546719	0.07695565	-2.009883	0.04444357	0.1100041
ENSG000001	1131.45286	0.19923146	0.07318175	2.72242015	0.00648057	0.02439813
ENSG000001	2493.06224	0.39920753	0.08485123	4.70479374	2.54E-06	4.01E-05
ENSG000001	67.7486917	-0.2287023	0.12905342	-1.7721522	0.07636929	0.16478432
ENSG000001	3037.96898	0.28521361	0.14475675	1.9702958	0.04880448	0.11781878
ENSG000001	7660.85316	-0.1940048	0.1125942	-1.7230442	0.08488054	0.17821222
ENSG000001	1257.35415	0.18711937	0.10125082	1.84807755	0.06459113	0.14597008
ENSG000001	4090.42275	-0.3529015	0.0712853	-4.9505512	7.40E-07	1.40E-05
ENSG000001	84.8179789	-0.4698946	0.158079	-2.9725302	0.00295356	0.01286534
ENSG000001	2574.65407	0.57709351	0.23609735	2.44430319	0.01451322	0.04695245
ENSG000001	2560.71606	0.18535665	0.08564478	2.16424931	0.03044522	0.08296838
ENSG000001	714.921512	-0.447264	0.15245374	-2.9337687	0.00334874	0.01425599
ENSG000001	2129.89978	-0.2594923	0.06289591	-4.1257414	3.70E-05	0.00036403
ENSG000001	1471.50894	-0.1694605	0.06618976	-2.560222	0.01046053	0.03615885
ENSG000001	6457.33721	0.84653979	0.17984313	4.707101	2.51E-06	3.99E-05
ENSG000001	12111.8731	0.42885644	0.14591456	2.93909294	0.00329174	0.01404225
ENSG000001	3440.57483	-0.2606749	0.06235474	-4.1805147	2.91E-05	0.00030011
ENSG000001	2943.17388	0.83838737	0.15528749	5.39893696	6.70E-08	1.93E-06
ENSG000001	622.102282	-0.149288	0.08834955	-1.6897421	0.0910773	0.18808792
ENSG000001	467.842288	0.23986218	0.11701728	2.04980141	0.04038381	0.10290947
ENSG000001	89.1483569	0.36806366	0.21263798	1.73094034	0.08346241	0.17617936

ENSG000001	688.317183	0.3325798	0.17850591	1.8631305	0.06244391	0.14225899
ENSG000001	8703.30272	0.29578976	0.13698221	2.15932976	0.03082459	0.08365603
ENSG000001	4119.7307	-0.1815813	0.05440181	-3.3377809	0.0008445	0.00469828
ENSG000001	705.304225	0.24040637	0.11420324	2.10507497	0.03528478	0.09300776
ENSG000001	703.469888	0.19725126	0.11403597	1.72972842	0.08367881	0.17640496
ENSG000001	1523.87035	0.20807785	0.06772269	3.07249805	0.00212275	0.009866
ENSG000001	19283.0792	0.34623929	0.11388543	3.04024224	0.00236388	0.01072183
ENSG000001	2004.4672	-0.194879	0.08516468	-2.2882602	0.02212237	0.06500746
ENSG000001	864.623506	-0.2085847	0.116401	-1.7919496	0.07314103	0.15977572
ENSG000001	3166.99579	0.19500636	0.06395294	3.04921668	0.00229439	0.01047552
ENSG000001	335.001522	1.27553035	0.24310938	5.24673445	1.55E-07	3.81E-06
ENSG000001	16038.1219	0.54435929	0.10549134	5.1602271	2.47E-07	5.64E-06
ENSG000001	240.896136	0.20949589	0.10166531	2.06064284	0.03933713	0.1009424
ENSG000001	4111.50545	0.49226059	0.18005459	2.7339519	0.00625792	0.02371396
ENSG000001	17825.1584	0.46611659	0.21162444	2.20256502	0.02762542	0.07696336
ENSG000001	1920.6948	0.36879422	0.16009681	2.30357001	0.02124679	0.06322972
ENSG000001	1056.62181	0.25707902	0.04729071	5.43614227	5.44E-08	1.63E-06
ENSG000001	85.4729832	0.90347301	0.17060046	5.29584157	1.18E-07	3.09E-06
ENSG000001	1114.84674	0.26274611	0.12143236	2.16372391	0.03048554	0.08304703
ENSG000001	1591.24114	0.39983024	0.11331617	3.52844834	0.000418	0.00264091
ENSG000001	1660.37398	0.16719042	0.05445422	3.07029305	0.00213849	0.00992004
ENSG000001	2251.03345	0.2460723	0.09578689	2.5689561	0.01020054	0.0354377
ENSG000001	54276.6324	0.52642891	0.21218591	2.48097953	0.01310219	0.04319787
ENSG000001	1518.30916	-0.1999189	0.08921861	-2.2407754	0.02504063	0.07160749
ENSG000001	1136.36442	0.30967406	0.07480939	4.1395079	3.48E-05	0.0003475
ENSG000001	6371.17276	0.75653379	0.14684988	5.1517493	2.58E-07	5.81E-06
ENSG000001	4932.53101	0.27120619	0.11781889	2.30189061	0.02134134	0.06343291
ENSG000001	1161.56698	0.76024474	0.18131464	4.19295827	2.75E-05	0.0002878
ENSG000001	9767.84919	0.43974965	0.18532409	2.37286823	0.01765056	0.05461419
ENSG000001	169.38278	0.76488087	0.17040894	4.48850196	7.17E-06	9.50E-05
ENSG000001	111.237978	-0.5940161	0.22341838	-2.6587611	0.00784286	0.02857662
ENSG000001	12121.4739	0.22536769	0.10765836	2.09335985	0.03631704	0.09506292
ENSG000001	297.077587	0.38775895	0.13434703	2.8862487	0.00389864	0.01610482
ENSG000001	7.16903526	2.61341989	1.08388148	2.41116758	0.01590154	0.05028343
ENSG000001	81.9147708	0.28333662	0.13983236	2.02625931	0.04273822	0.10717476
ENSG000001	4804.31328	0.46545176	0.13308791	3.49732558	0.00046995	0.00291819
ENSG000001	73.5883515	0.42165064	0.17471419	2.4133738	0.0158056	0.05002747
ENSG000001	3015.4513	0.1031247	0.04933574	2.09026354	0.03659413	0.09557224
ENSG000001	2457.80316	-0.2392079	0.09959926	-2.4017035	0.01631893	0.05134934
ENSG000001	2539.54202	0.68333416	0.15332878	4.45665952	8.32E-06	0.00010704
ENSG000001	256.804553	0.21687553	0.10219993	2.1220712	0.03383176	0.08999646
ENSG000001	668.741045	0.3363984	0.14119524	2.38250525	0.01719529	0.0535026
ENSG000001	1244.87167	-0.2743381	0.08582515	-3.1964766	0.00139117	0.00705422

ENSG000001	655.377858	-0.2259104	0.13620577	-1.6585966	0.09719711	0.19732267
ENSG000001	130.348248	-0.3429259	0.13177712	-2.6023176	0.00925961	0.03284621
ENSG000001	7669.43921	-0.2230404	0.11551915	-1.9307658	0.05351203	0.12632047
ENSG000001	585.353142	0.41530509	0.18109361	2.29331716	0.02182975	0.06440902
ENSG000001	868.871124	0.375299	0.11963912	3.13692558	0.00170729	0.00830822
ENSG000001	5702.23195	0.30132637	0.12012764	2.50838511	0.01212844	0.04060789
ENSG000001	274.525453	-0.4453314	0.13730189	-3.2434469	0.00118093	0.00618962
ENSG000001	3290.34782	0.11894155	0.04323288	2.7511829	0.00593805	0.02270974
ENSG000001	1144.71489	0.1274969	0.06550087	1.94649157	0.05159572	0.12277458
ENSG000001	3277.38124	0.35574144	0.07901376	4.50227218	6.72E-06	9.01E-05
ENSG000001	2976.29271	-0.3650841	0.10511217	-3.473281	0.00051414	0.00312822
ENSG000001	1849.29069	-0.3375755	0.10775349	-3.1328495	0.00173118	0.00841034
ENSG000001	4639.30364	0.35782427	0.07574341	4.72416389	2.31E-06	3.73E-05
ENSG000001	3320.01407	0.25330969	0.07919495	3.19855868	0.00138116	0.00700838
ENSG000001	2444.58015	-0.4330686	0.08969271	-4.8283594	1.38E-06	2.37E-05
ENSG000001	7920.12899	-0.2564955	0.07944141	-3.2287381	0.00124338	0.00644927
ENSG000001	114.581807	0.44731021	0.18473103	2.42141351	0.01546028	0.04922485
ENSG000001	105.337681	-0.4593312	0.25830794	-1.7782311	0.07536591	0.16322709
ENSG000001	16490.6329	0.20401176	0.07414812	2.7514083	0.00593396	0.02270011
ENSG000001	3662.24285	-0.1064547	0.05011052	-2.1243983	0.03363685	0.08960887
ENSG000001	678.968999	-0.1179802	0.05379888	-2.1929858	0.0283084	0.07847375
ENSG000001	61.6182189	-0.5988269	0.17780033	-3.3679743	0.00075723	0.0042952
ENSG000001	12725.8793	-0.2881277	0.09143172	-3.1512882	0.00162552	0.00800167
ENSG000001	3512.01123	-0.175428	0.06351515	-2.7619868	0.00574508	0.02215304
ENSG000001	334.75302	0.29358818	0.10282592	2.85519623	0.00430102	0.01745838
ENSG000001	1294.84092	0.73559126	0.11976652	6.14187735	8.16E-10	4.67E-08
ENSG000001	444.925262	0.47324928	0.14804561	3.1966451	0.00139036	0.00705257
ENSG000001	2247.38558	0.38294955	0.10453506	3.66335994	0.00024893	0.00172629
ENSG000001	5334.06302	6.88288347	0.55073407	12.4976533	7.69E-36	5.57E-32
ENSG000001	1644.04768	0.58476487	0.13725315	4.26048406	2.04E-05	0.00022404
ENSG000001	32.328262	1.32480409	0.2800877	4.72996175	2.25E-06	3.64E-05
ENSG000001	14582.5035	0.25291796	0.10377982	2.43706289	0.0148071	0.0476161
ENSG000001	644.668143	-0.1563823	0.07688006	-2.0341081	0.0419407	0.10568707
ENSG000001	2424.35005	-0.2753232	0.0560152	-4.9151512	8.87E-07	1.64E-05
ENSG000001	1368.94175	-0.1325368	0.07459157	-1.7768337	0.07559561	0.16350791
ENSG000001	879.712231	0.39177726	0.10227107	3.83077313	0.00012774	0.00100961
ENSG000001	3998.61915	-0.400643	0.08476729	-4.7263867	2.29E-06	3.70E-05
ENSG000001	100.597037	-0.7455829	0.14933755	-4.9926012	5.96E-07	1.17E-05
ENSG000001	7016.43527	1.73860963	0.24026696	7.23615771	4.62E-13	7.04E-11
ENSG000001	5443.90622	-0.3067886	0.12164462	-2.5220074	0.01166872	0.03944116
ENSG000001	37984.1821	0.33965142	0.19256886	1.76379203	0.077767	0.16675703
ENSG000001	3607.34686	-0.1749237	0.0709354	-2.4659578	0.01366474	0.04471808
ENSG000001	2227.93505	-0.1039098	0.06206825	-1.6741217	0.09410669	0.19261406

ENSG000001	1056.58894	-0.1774605	0.10635988	-1.6684909	0.09521832	0.19414888
ENSG000001	3696.45092	-0.3105396	0.10396546	-2.9869496	0.00281776	0.01236832
ENSG000001	1492.02615	0.18803765	0.08164216	2.30319308	0.02126798	0.06326679
ENSG000001	1998.88705	0.47886965	0.14053613	3.40744868	0.00065573	0.00380745
ENSG000001	1096.81974	0.38345924	0.11163988	3.43478722	0.00059302	0.00350799
ENSG000001	271.502684	0.48907956	0.114964	4.25419768	2.10E-05	0.000229
ENSG000001	77.350062	0.66300864	0.24662806	2.68829362	0.00718182	0.02656192
ENSG000001	37355.1375	0.99125607	0.17188252	5.76705583	8.07E-09	3.35E-07
ENSG000001	516.295344	0.32280157	0.10077479	3.20319753	0.00135911	0.00692599
ENSG000001	1705.12663	0.2292031	0.04246467	5.39750095	6.76E-08	1.94E-06
ENSG000001	61.1588299	0.95575404	0.33568196	2.84720108	0.00441055	0.01782305
ENSG000001	2155.82767	0.23523642	0.04862276	4.8379899	1.31E-06	2.28E-05
ENSG000001	3297.07086	-0.2059547	0.07342294	-2.8050459	0.00503094	0.01992979
ENSG000001	1208.93503	0.18203493	0.05341465	3.40795869	0.00065451	0.00380187
ENSG000001	379.502423	-0.1420257	0.0675444	-2.102701	0.03549192	0.09348542
ENSG000001	503.492621	0.52771065	0.14823903	3.55986297	0.00037105	0.00239758
ENSG000001	1709.83678	0.25117474	0.0861666	2.91498971	0.003557	0.01496669
ENSG000001	149.809028	-0.2415421	0.1130648	-2.1363157	0.03265369	0.08748918
ENSG000001	437.227209	0.26503226	0.11539441	2.29675125	0.02163296	0.0639977
ENSG000001	154.322557	0.34427383	0.12079012	2.85018212	0.00436942	0.01768645
ENSG000001	4120.4094	0.34884604	0.10628175	3.2822759	0.00102973	0.00554354
ENSG000001	14365.8549	1.66972148	0.38557565	4.33046401	1.49E-05	0.00017286
ENSG000001	2230.68177	0.8175266	0.27039676	3.02343342	0.00249924	0.01122334
ENSG000001	4651.7134	1.79625341	0.36035011	4.984745	6.20E-07	1.21E-05
ENSG000001	11682.1332	1.36338376	0.30430643	4.48029894	7.45E-06	9.77E-05
ENSG000001	1974.55141	-0.29349	0.07299177	-4.0208643	5.80E-05	0.00052832
ENSG000001	70.3247384	0.30939948	0.11536498	2.68191863	0.00732013	0.0269496
ENSG000001	1725.01145	0.39164548	0.09064311	4.32074193	1.56E-05	0.00017965
ENSG000001	2017.3175	-0.3207494	0.1730089	-1.8539474	0.06374669	0.14452242
ENSG000001	4033.77594	-0.1348982	0.07263757	-1.8571416	0.06329101	0.14371425
ENSG000001	3297.22065	0.13872951	0.08195193	1.69281561	0.09049057	0.18711951
ENSG000001	5598.21342	0.2305405	0.0724116	3.18375088	0.0014538	0.0073131
ENSG000001	2937.49358	-0.3333654	0.07129872	-4.6756158	2.93E-06	4.51E-05
ENSG000001	1076.70054	0.26946671	0.10530116	2.55900987	0.01049708	0.03626786
ENSG000001	1818.2769	-0.2495281	0.08079843	-3.0882792	0.00201319	0.00944788
ENSG000001	12275.631	0.58602288	0.20996671	2.79102759	0.0052541	0.02061651
ENSG000001	71.3169387	2.31926164	0.47607779	4.87160225	1.11E-06	1.95E-05
ENSG000001	3113.99341	0.12897916	0.06258731	2.06078784	0.03932329	0.1009424
ENSG000001	1235.11713	0.41246526	0.15507678	2.65974867	0.0078199	0.02851419
ENSG000001	2247.02106	0.16720485	0.08309482	2.01221754	0.04419701	0.1095623
ENSG000001	1164.00694	0.22149212	0.06361238	3.48190294	0.00049786	0.00305487
ENSG000001	34058.2685	0.27300063	0.1105166	2.47022291	0.01350289	0.04427713
ENSG000001	803.493974	-0.1287285	0.05694088	-2.2607393	0.02377541	0.06872193

ENSG000001	847.587392	-0.5389092	0.08536874	-6.3127236	2.74E-10	1.92E-08
ENSG000001	46.7785453	-0.6135721	0.24421758	-2.5123994	0.01199133	0.04025918
ENSG000001	1667.24799	0.40281926	0.07431311	5.42056814	5.94E-08	1.74E-06
ENSG000001	85.3007914	0.40909297	0.18158336	2.25292109	0.02426412	0.06989223
ENSG000001	1483.94804	-0.1088029	0.06235659	-1.7448502	0.08101096	0.17213343
ENSG000001	650.820214	-0.3624605	0.08812736	-4.1129172	3.91E-05	0.00037994
ENSG000001	617.180547	0.31769661	0.15916782	1.99598522	0.04593553	0.11277004
ENSG000001	993.302938	-0.1273855	0.07246125	-1.7579815	0.07875065	0.16829336
ENSG000001	186.263216	0.21231005	0.08149535	2.60517977	0.00918261	0.03260501
ENSG000001	1378.00063	-0.1884678	0.05588543	-3.3723964	0.00074517	0.0042451
ENSG000001	1634.39446	0.24570944	0.05517094	4.45360258	8.44E-06	0.00010834
ENSG000001	11029.2329	0.32398623	0.0857691	3.77742384	0.00015846	0.00119625
ENSG000001	2890.58197	-0.3370127	0.10606874	-3.1773043	0.00148651	0.00743614
ENSG000001	4774.91178	0.2541819	0.10419469	2.43948992	0.01470801	0.04739159
ENSG000001	786.188238	-0.3191452	0.08801024	-3.6262282	0.00028759	0.00194962
ENSG000001	13440.9232	-0.4802692	0.15229589	-3.153527	0.0016131	0.00795135
ENSG000001	6621.60346	-0.2008642	0.06978725	-2.8782372	0.00399904	0.01643521
ENSG000001	3334.34656	0.22417307	0.13073903	1.71466055	0.08640748	0.18058066
ENSG000001	942.465426	-0.1552991	0.06884477	-2.2557868	0.02408399	0.06948909
ENSG000001	3806.97367	0.27952722	0.0650254	4.29873916	1.72E-05	0.00019426
ENSG000001	8451.96136	-0.1626874	0.05771729	-2.8186946	0.00482194	0.01923335
ENSG000001	15888.2252	0.503926	0.10057722	5.01033941	5.43E-07	1.09E-05
ENSG000001	10219.2922	0.33224257	0.14707644	2.25897891	0.0238847	0.06899653
ENSG000001	632.968201	-0.2795863	0.09855951	-2.8367257	0.00455787	0.01832638
ENSG000001	979.728246	-0.2022691	0.06119469	-3.3053376	0.00094862	0.00517614
ENSG000001	63.4109401	-0.6152762	0.14352951	-4.2867574	1.81E-05	0.0002036
ENSG000001	14277.6728	-0.1921899	0.04550384	-4.2235978	2.40E-05	0.00025782
ENSG000001	2658.85435	0.20290426	0.04902407	4.13886998	3.49E-05	0.00034813
ENSG000001	191.251257	-0.3209444	0.10706426	-2.9976805	0.00272043	0.01202283
ENSG000001	1755.94186	-0.2516545	0.11645989	-2.1608688	0.03070547	0.08344217
ENSG000001	141.722976	-0.544221	0.16193776	-3.36068	0.00077751	0.00438962
ENSG000001	2752.8177	1.27219468	0.21481953	5.92215552	3.18E-09	1.50E-07
ENSG000001	553.783397	0.33091313	0.09041977	3.65974295	0.00025247	0.00174342
ENSG000001	1462.95545	0.15302803	0.09083016	1.68477102	0.09203276	0.18954771
ENSG000001	3710.42296	0.22258214	0.11027606	2.01840842	0.04354874	0.10847501
ENSG000001	7559.76897	0.19085517	0.09952986	1.91756691	0.05516595	0.12926205
ENSG000001	94348.6791	0.32254499	0.16724415	1.92858752	0.05378209	0.12676416
ENSG000001	1005.4552	-0.258715	0.0848267	-3.0499246	0.00228899	0.01046075
ENSG000001	356.924511	0.36491385	0.1120422	3.25693219	0.00112623	0.00595682
ENSG000001	3661.191	-0.4151889	0.23293782	-1.7824022	0.07468366	0.16221943
ENSG000001	46955.4866	0.57442417	0.18189844	3.15793894	0.00158889	0.00784801
ENSG000001	1734.2511	0.2534476	0.09269296	2.7342703	0.00625187	0.02369724
ENSG000001	858.975905	0.27949563	0.05822195	4.8005201	1.58E-06	2.68E-05

ENSG000001	6738.32405	0.17578381	0.07670753	2.29161081	0.02192811	0.06458213
ENSG000001	1215.6538	0.48989805	0.15603946	3.13957792	0.00169191	0.00825556
ENSG000001	2812.16699	0.15363292	0.07606584	2.01973604	0.04341077	0.10822438
ENSG000001	2004.07094	0.68069099	0.12767999	5.33122676	9.76E-08	2.63E-06
ENSG000001	1143.66309	0.23706215	0.11599141	2.04379066	0.04097423	0.10382958
ENSG000001	12637.0894	-0.23502	0.12597996	-1.865535	0.06210645	0.14173537
ENSG000001	239.971179	-0.1516407	0.08832288	-1.7168903	0.08599921	0.18009116
ENSG000001	1224.5501	0.19780575	0.07619199	2.59614894	0.00942752	0.03327488
ENSG000001	2127.90991	0.07776056	0.03664278	2.12212522	0.03382722	0.08999646
ENSG000001	1577.91549	0.21286637	0.09780874	2.1763532	0.02952886	0.08094316
ENSG000001	456.173262	-0.3379262	0.08161221	-4.140633	3.46E-05	0.00034643
ENSG000001	4757.72321	0.31953363	0.07933993	4.02740015	5.64E-05	0.0005158
ENSG000001	1357.82313	-0.3461955	0.14364759	-2.4100335	0.01595106	0.05038878
ENSG000001	3517.41935	1.06196727	0.28479838	3.72883884	0.00019236	0.00139339
ENSG000001	354.529902	0.26264181	0.13941859	1.88383636	0.05958711	0.13723983
ENSG000001	370.229314	1.80221352	0.25423653	7.08872775	1.35E-12	1.77E-10
ENSG000001	1723.14937	-0.138901	0.05573306	-2.4922542	0.01269352	0.04208994
ENSG000001	1163.00938	0.20681666	0.10596274	1.95178666	0.05096354	0.12153231
ENSG000001	1614.94118	-0.2748313	0.13268003	-2.0713842	0.03832291	0.09896328
ENSG000001	7886.64313	0.28020706	0.0968696	2.89262123	0.00382042	0.01584494
ENSG000001	3522.54451	0.16938157	0.06977326	2.42760012	0.01519909	0.04860689
ENSG000001	1448.53153	0.33882854	0.08044883	4.21172724	2.53E-05	0.00026916
ENSG000001	1807.97651	0.27431799	0.15337162	1.78858382	0.07368187	0.16058811
ENSG000001	2189.31193	0.22324041	0.06777119	3.29403113	0.00098762	0.00535865
ENSG000001	724.021452	-0.2837456	0.09355478	-3.0329351	0.00242188	0.01094036
ENSG000001	2052.39603	0.1082932	0.06541616	1.65545013	0.09783322	0.19825289
ENSG000001	2878.51971	0.77699386	0.18763225	4.1410464	3.46E-05	0.00034637
ENSG000001	15760.2161	1.38860224	0.2441099	5.68843054	1.28E-08	4.86E-07
ENSG000001	3706.53664	0.13847843	0.07845505	1.76506715	0.07755248	0.16639733
ENSG000001	18041.0262	0.43280164	0.15961832	2.71147844	0.00669839	0.02508131
ENSG000001	76.5615779	-0.692313	0.1871678	-3.6988894	0.00021654	0.00153779
ENSG000001	9042.56605	0.09350074	0.04918464	1.90101506	0.05730004	0.13311509
ENSG000001	1541.61137	-0.119274	0.05546904	-2.1502802	0.03153306	0.08518143
ENSG000001	137.810413	0.99770254	0.3045582	3.27590108	0.00105325	0.0056492
ENSG000001	20271.0643	0.24620342	0.13430209	1.83320614	0.06677192	0.14927678
ENSG000001	161.607575	0.61008888	0.17027001	3.58306714	0.00033958	0.00223007
ENSG000001	971.949798	-0.2397321	0.0930546	-2.5762522	0.00998778	0.03482381
ENSG000001	3527.32373	0.37451191	0.10675694	3.50808032	0.00045135	0.00281565
ENSG000001	967.3889	0.64654436	0.10231695	6.31903457	2.63E-10	1.86E-08
ENSG000001	653.876966	-0.5090445	0.12070812	-4.2171517	2.47E-05	0.00026354
ENSG000001	61.916191	-1.0311705	0.19618034	-5.2562375	1.47E-07	3.66E-06
ENSG000001	963.355784	-0.3009332	0.09131471	-3.2955616	0.00098225	0.00533217
ENSG000001	572.684221	0.27880478	0.11399988	2.44565844	0.01445879	0.04680771

ENSG000001	686.342146	0.1707604	0.09686613	1.7628494	0.07792589	0.16699888
ENSG000001	8119.26539	-0.4988578	0.13230378	-3.7705483	0.00016289	0.00122332
ENSG000001	544.539142	-0.1301132	0.06426919	-2.0245035	0.04291838	0.10750365
ENSG000001	46.0117583	-0.5245725	0.21618986	-2.4264435	0.01524762	0.04871909
ENSG000001	2950.10773	-0.4557309	0.1423917	-3.2005441	0.00137168	0.0069676
ENSG000001	4041.94083	0.39062786	0.08627104	4.52791392	5.96E-06	8.18E-05
ENSG000001	18394.5015	0.27577804	0.11466833	2.4050061	0.0161722	0.05096511
ENSG000001	1737.04417	-0.2275493	0.0592642	-3.8395751	0.00012325	0.00098266
ENSG000001	1095.50599	-0.6342944	0.14374667	-4.4125851	1.02E-05	0.00012713
ENSG000001	45.0729825	1.30071004	0.29281448	4.44209596	8.91E-06	0.00011331
ENSG000001	1311.60302	0.07854662	0.04476654	1.75458312	0.07933064	0.16918342
ENSG000001	1975.07674	0.25171267	0.0595245	4.22872389	2.35E-05	0.00025276
ENSG000001	216.709342	0.74836685	0.19465297	3.84462073	0.00012074	0.00096585
ENSG000001	277.730151	1.26927243	0.30327099	4.18527477	2.85E-05	0.00029494
ENSG000001	4382.67366	0.32819571	0.10249975	3.2019171	0.00136516	0.00694421
ENSG000001	619.010881	0.28781334	0.07536402	3.81897566	0.00013401	0.00104938
ENSG000001	5231.24786	-0.1819173	0.07534496	-2.4144589	0.0157586	0.04992501
ENSG000001	1459.59624	0.16769487	0.0918658	1.82543309	0.0679357	0.15111078
ENSG000001	1956.01231	2.73161367	0.41428208	6.59360801	4.29E-11	3.72E-09
ENSG000001	506.914128	0.88834897	0.1660476	5.34996567	8.80E-08	2.40E-06
ENSG000001	3594.66537	0.26954925	0.1459324	1.84708299	0.06473511	0.14614217
ENSG000001	43.4026492	1.3640737	0.28165863	4.84300334	1.28E-06	2.22E-05
ENSG000001	1339.74997	0.28993416	0.08372385	3.46298183	0.00053422	0.00322606
ENSG000001	148.675774	-0.3207308	0.1578162	-2.0323058	0.04212271	0.10597979
ENSG000001	77.8611308	-0.3916477	0.18303626	-2.1397276	0.03237679	0.08690801
ENSG000001	22.5848366	0.46281168	0.23196134	1.99521044	0.04601993	0.11286453
ENSG000001	8851.25959	0.25356636	0.11758143	2.15651718	0.0310433	0.08412352
ENSG000001	748.32125	0.11555194	0.06909169	1.67244348	0.0944369	0.19290855
ENSG000001	63.4900306	-0.8105945	0.20802127	-3.8966906	9.75E-05	0.00080865
ENSG000001	92.3544019	-0.2216279	0.10244344	-2.1634171	0.03050911	0.08309561
ENSG000001	44.9880357	1.16578209	0.38213982	3.05066894	0.00228332	0.01043815
ENSG000001	4990.8199	0.16890094	0.10236228	1.65003105	0.09893659	0.19973444
ENSG000001	5.38502259	1.76658544	0.45587559	3.8751481	0.00010656	0.00086995
ENSG000001	5810.59357	0.4278493	0.11384224	3.75826493	0.0001711	0.00127569
ENSG000001	5232.85517	-0.4571452	0.12169023	-3.7566304	0.00017222	0.00128009
ENSG000001	3005.07691	0.24281024	0.10051458	2.41567181	0.01570621	0.04981083
ENSG000001	1721.94686	2.40159039	0.42876941	5.60112347	2.13E-08	7.51E-07
ENSG000001	25414.4122	0.49973493	0.12695969	3.9361702	8.28E-05	0.00070637
ENSG000001	5737.31146	0.4615711	0.07568331	6.09871698	1.07E-09	5.87E-08
ENSG000001	3630.55969	0.29207871	0.11287121	2.58771673	0.00966144	0.0338899
ENSG000001	434.763023	-0.2504524	0.08192467	-3.057106	0.00223485	0.0102619
ENSG000001	12319.4365	0.45255701	0.09527469	4.75002365	2.03E-06	3.33E-05
ENSG000001	6986.43698	-0.1458652	0.08190893	-1.7808216	0.0749416	0.16251623

ENSG000001	787.487351	0.26796649	0.10540929	2.54215238	0.01101721	0.03766078
ENSG000001	50.2511964	0.42327865	0.17683511	2.39363468	0.01668236	0.05226585
ENSG000001	21282.6945	-0.3293981	0.11088879	-2.9705268	0.00297289	0.01293343
ENSG000001	5916.9288	0.17374102	0.09484171	1.83190507	0.06696557	0.14956122
ENSG000001	613.655801	1.11778641	0.21399013	5.22354194	1.76E-07	4.25E-06
ENSG000001	16286.7169	0.38978906	0.17529002	2.22368088	0.02616992	0.07386006
ENSG000001	4082.15181	-0.3228334	0.08557096	-3.7726982	0.00016149	0.0012166
ENSG000001	927.824808	0.53252374	0.13931927	3.82232644	0.0001322	0.00103746
ENSG000001	630.722345	1.20090302	0.16630603	7.22104304	5.16E-13	7.69E-11
ENSG000001	37.9695	0.4077709	0.18553227	2.19784352	0.02796026	0.07770386
ENSG000001	1719.06363	0.31043288	0.18011926	1.72348515	0.08480083	0.17811464
ENSG000001	80.6303752	-0.2300059	0.11397656	-2.0180102	0.0435902	0.10852228
ENSG000001	2284.67236	0.14160174	0.037953	3.73097595	0.00019074	0.00138508
ENSG000001	196.505339	-0.2672863	0.09210917	-2.901843	0.00370974	0.01547454
ENSG000001	234.122036	-0.4002559	0.13494267	-2.9661181	0.00301585	0.01309277
ENSG000001	8870.64418	0.20386355	0.08845743	2.30465159	0.02118609	0.06311105
ENSG000001	4645.55071	-0.2427058	0.05787067	-4.1939337	2.74E-05	0.00028692
ENSG000001	5527.9566	-0.1481986	0.06228756	-2.3792647	0.01734721	0.05382503
ENSG000001	4211.31343	0.20804749	0.0747218	2.78429451	0.00536443	0.0209586
ENSG000001	458.885497	-0.1888938	0.09349751	-2.020308	0.04335145	0.10815094
ENSG000001	2271.43104	0.31094084	0.13532588	2.29771895	0.02157779	0.06391279
ENSG000001	848.677349	0.1646495	0.0873107	1.88578831	0.05932349	0.13690345
ENSG000001	813.800652	-0.2536429	0.10306992	-2.4608823	0.01385958	0.04522157
ENSG000001	1514.27477	0.09295289	0.04693203	1.98058532	0.0476378	0.1158112
ENSG000001	418.860153	-0.300406	0.17074678	-1.7593656	0.07851544	0.16798894
ENSG000001	188.567953	-0.4854747	0.13232468	-3.6688142	0.00024368	0.00169556
ENSG000001	1198.85856	-0.1522845	0.055344	-2.751598	0.00593053	0.02269296
ENSG000001	428577.177	0.38606395	0.16038761	2.40706834	0.01608116	0.05072235
ENSG000001	1026.66451	-0.3216444	0.09305586	-3.4564656	0.00054731	0.00328999
ENSG000001	749.499689	-0.3027925	0.09135609	-3.314421	0.00091833	0.00503743
ENSG000001	2467.74731	-0.3951507	0.07466006	-5.2926651	1.21E-07	3.14E-06
ENSG000001	36.2895265	0.81335374	0.25392071	3.20318004	0.00135919	0.00692599
ENSG000001	197.802819	0.68708158	0.14332648	4.7938217	1.64E-06	2.75E-05
ENSG000001	5645.18175	-0.1034814	0.04344747	-2.3817594	0.01723015	0.05357519
ENSG000001	198.833978	-0.1700094	0.08561803	-1.9856729	0.04706966	0.11485568
ENSG000001	609.020902	0.28269256	0.13690273	2.06491542	0.038931	0.10022987
ENSG000001	4264.34807	-0.1497436	0.03394867	-4.4108829	1.03E-05	0.0001278
ENSG000001	159.997183	-0.4315505	0.12798299	-3.3719362	0.00074642	0.00425037
ENSG000001	199.887576	0.46129404	0.13066052	3.53047752	0.00041481	0.00262532
ENSG000001	327.762097	-0.5432603	0.09485503	-5.727269	1.02E-08	4.08E-07
ENSG000001	84.698636	-0.2531521	0.15306447	-1.6538919	0.09814948	0.19872889
ENSG000001	2854.77759	-0.2127052	0.07788352	-2.7310687	0.00631293	0.02388494
ENSG000001	1367.86322	0.17372738	0.10041754	1.73005014	0.08362132	0.17633508

ENSG000001	1000.86163	-0.1923528	0.09309132	-2.0662802	0.03880203	0.09997443
ENSG000001	7911.48232	0.38603533	0.1095368	3.5242524	0.00042468	0.00267609
ENSG000001	5173.65702	0.24772978	0.0946508	2.61730264	0.00886277	0.03166839
ENSG000001	1612.11716	0.86321994	0.21855054	3.94974972	7.82E-05	0.00067623
ENSG000001	770.03254	-0.541298	0.13960045	-3.8774801	0.00010554	0.00086288
ENSG000001	1239.22215	0.24495583	0.07012127	3.4933172	0.00047706	0.00295475
ENSG000001	12.0464476	1.86818813	0.49190015	3.79790114	0.00014593	0.00112091
ENSG000001	62.9646016	0.74098229	0.21260103	3.4853185	0.00049155	0.00302539
ENSG000001	74174.5808	3.07620649	0.29936964	10.2756128	9.08E-25	1.01E-21
ENSG000001	66153.2217	2.38272448	0.30483797	7.81636375	5.44E-15	1.21E-12
ENSG000001	1144.16021	0.34527162	0.06859665	5.03336005	4.82E-07	9.83E-06
ENSG000001	569.700642	-0.2653299	0.10832536	-2.4493791	0.01431027	0.04643067
ENSG000001	228.673475	0.39450645	0.14703168	2.68313903	0.00729347	0.02687193
ENSG000001	1064.71786	-0.231691	0.08921062	-2.5971232	0.00940082	0.03324132
ENSG000001	1232.63414	0.2112519	0.11374699	1.85720871	0.06328147	0.14371425
ENSG000001	3230.61414	-0.1811763	0.07943176	-2.2809052	0.02255405	0.06590169
ENSG000001	264.496667	0.85951985	0.1807255	4.75594128	1.98E-06	3.25E-05
ENSG000001	575.005279	-0.1836964	0.09445062	-1.944894	0.05178774	0.1230118
ENSG000001	27813.3095	0.75234162	0.24830064	3.02996243	0.00244584	0.0110297
ENSG000001	68.2947973	0.82345475	0.37336825	2.2054761	0.0274207	0.07654022
ENSG000001	518.555193	-0.1894626	0.09481276	-1.9982818	0.04568612	0.11236923
ENSG000001	2114.91364	1.10592719	0.24762783	4.4660861	7.97E-06	0.00010323
ENSG000001	1619.53096	-0.1404037	0.07059659	-1.9888177	0.04672133	0.11419807
ENSG000001	242.316672	0.3025914	0.12675309	2.38725065	0.01697491	0.05301047
ENSG000001	3323.9897	0.53759724	0.08537348	6.29700504	3.03E-10	2.06E-08
ENSG000001	49.2519427	-0.6897734	0.20674799	-3.3363006	0.00084901	0.00471793
ENSG000001	2314.72119	1.03096709	0.14455553	7.13197941	9.89E-13	1.33E-10
ENSG000001	3477.14697	0.48668404	0.21410813	2.2730759	0.02302161	0.06695724
ENSG000001	2999.53529	0.12592871	0.03143959	4.00541856	6.19E-05	0.00055671
ENSG000001	869.936661	0.22139472	0.07556649	2.92980028	0.0033918	0.01439789
ENSG000001	4011.64653	-0.2830298	0.05764535	-4.9098461	9.11E-07	1.68E-05
ENSG000001	4031.77426	0.13443167	0.07903474	1.70091868	0.08895826	0.18465945
ENSG000001	628.078597	-0.3584979	0.11574792	-3.0972298	0.00195338	0.00924491
ENSG000001	509.057418	0.23915431	0.08538126	2.80101626	0.0050942	0.02010888
ENSG000001	331.245446	0.34014073	0.14852036	2.29019595	0.02200996	0.0647691
ENSG000001	1298.574	0.24322678	0.06854936	3.54819898	0.00038788	0.00248714
ENSG000001	531.600641	0.64696722	0.13600285	4.75701231	1.96E-06	3.24E-05
ENSG000001	7099.73806	0.40642429	0.16081242	2.52731899	0.01149371	0.0389769
ENSG000001	2289.80796	0.32021831	0.09479065	3.37816331	0.00072972	0.00417348
ENSG000001	603.395652	0.18237332	0.07481955	2.43750898	0.01478885	0.04758908
ENSG000001	1023.4209	0.50219911	0.13828245	3.63169086	0.00028157	0.00191328
ENSG000001	2170.96605	-0.1842934	0.06321399	-2.9153896	0.00355245	0.01495306
ENSG000001	585.647714	0.32741976	0.08270096	3.95908037	7.52E-05	0.00065583

ENSG000001	12327.0073	0.32111019	0.1378767	2.32896626	0.01986085	0.06003648
ENSG000001	117.868477	-0.3638203	0.08829496	-4.1205102	3.78E-05	0.00037079
ENSG000001	2365.8995	-0.2281448	0.0999539	-2.2824997	0.02245985	0.06569269
ENSG000001	618.41189	0.18667471	0.10091363	1.84984634	0.0643357	0.14556165
ENSG000001	7818.67389	-0.4601767	0.14239848	-3.2316122	0.00123094	0.0064031
ENSG000001	3634.25619	0.1351421	0.08001034	1.68905802	0.09120831	0.18822433
ENSG000001	158.282266	-0.4868476	0.1680013	-2.89788	0.00375694	0.0156399
ENSG000001	377.402974	-0.2029926	0.11160811	-1.8187982	0.06894222	0.15280997
ENSG000001	632.472343	0.25484116	0.13284399	1.91834911	0.05506676	0.12908611
ENSG000001	744.530072	0.36212874	0.0849721	4.26173706	2.03E-05	0.00022347
ENSG000001	1306.86433	-0.6626273	0.14771287	-4.4859142	7.26E-06	9.59E-05
ENSG000001	28481.1743	0.29380807	0.1152399	2.54953422	0.01078669	0.03707398
ENSG000001	950.813797	-0.4447941	0.1628738	-2.7309123	0.00631593	0.02389004
ENSG000001	851.351285	0.15881922	0.06477182	2.45198022	0.01420725	0.04618848
ENSG000001	1315.22863	-0.2221494	0.08698435	-2.5539009	0.01065236	0.03668189
ENSG000001	3020.54617	-0.1608524	0.04018223	-4.0030721	6.25E-05	0.00056106
ENSG000001	2267.66424	0.28721972	0.05593685	5.13471382	2.83E-07	6.26E-06
ENSG000001	1099.49462	-0.2035029	0.08693263	-2.3409265	0.01923596	0.05855669
ENSG000001	658.260669	-0.1222662	0.06333857	-1.930359	0.05356237	0.12639813
ENSG000001	33253.9296	0.87493728	0.15958591	5.48254698	4.19E-08	1.33E-06
ENSG000001	5681.31081	-0.1533495	0.08545956	-1.7944103	0.0727477	0.15919879
ENSG000001	142.134078	0.80813755	0.21353686	3.78453425	0.000154	0.00116887
ENSG000001	279.114984	2.61163863	0.30809442	8.47674751	2.32E-17	8.60E-15
ENSG000001	17.7396286	2.00886548	0.5015027	4.00569223	6.18E-05	0.00055661
ENSG000001	5865.25214	0.45559902	0.12309854	3.7010919	0.00021467	0.00152674
ENSG000001	735.999536	-0.1645243	0.06010844	-2.7371244	0.00619789	0.02352955
ENSG000001	20506.9421	0.38573286	0.18378313	2.09884808	0.0358303	0.09412031
ENSG000001	280.300803	0.22307164	0.11077383	2.01375759	0.04403499	0.10934777
ENSG000001	1820.28848	0.11631073	0.03732494	3.11616652	0.00183219	0.00879778
ENSG000001	513.003821	-0.2288859	0.10656583	-2.1478355	0.03172682	0.08559152
ENSG000001	868.438767	-0.4609702	0.1352038	-3.4094472	0.00065095	0.00378573
ENSG000001	1233.04827	-0.287885	0.06174463	-4.6625108	3.12E-06	4.76E-05
ENSG000001	211.77413	0.56640019	0.19519274	2.90174826	0.00371087	0.01547476
ENSG000001	1655.73373	0.18759667	0.09020017	2.07978168	0.03754556	0.0975528
ENSG000001	2912.23976	1.2894579	0.18072915	7.13475344	9.70E-13	1.31E-10
ENSG000001	968.375748	0.42893966	0.1313606	3.26536	0.00109325	0.00581635
ENSG000001	48.6666998	0.72645788	0.20434165	3.55511416	0.00037782	0.00243154
ENSG000001	2160.45076	-0.2591619	0.0571779	-4.5325537	5.83E-06	8.02E-05
ENSG000001	13028.6195	-0.6782052	0.15718176	-4.314783	1.60E-05	0.00018361
ENSG000001	53.2425173	1.14366765	0.35066588	3.26141696	0.00110857	0.00587596
ENSG000001	2589.57972	0.342441	0.10103562	3.3893097	0.00070069	0.00402972
ENSG000001	833.309392	0.24717579	0.06554461	3.77110798	0.00016252	0.00122159
ENSG000001	6068.71152	-0.2832705	0.04925482	-5.7511218	8.87E-09	3.62E-07

ENSG000001	844.962508	-0.3751835	0.07834514	-4.7888544	1.68E-06	2.81E-05
ENSG000001	529.141577	0.71087696	0.16279764	4.3666295	1.26E-05	0.00015057
ENSG000001	957.340033	0.34327568	0.08422309	4.07579073	4.59E-05	0.00043507
ENSG000001	25985.467	1.20122686	0.16293753	7.37231543	1.68E-13	2.79E-11
ENSG000001	1862.17581	0.22275564	0.10677739	2.0861687	0.03696334	0.09636278
ENSG000001	3778.49589	-0.1225202	0.06784103	-1.8059903	0.07091985	0.15626098
ENSG000001	580.649477	0.11175797	0.05917037	1.88874889	0.05892548	0.13619233
ENSG000001	76.6355848	0.22550943	0.09441208	2.3885654	0.0169143	0.05285536
ENSG000001	1138.43179	0.23092883	0.08717101	2.64914709	0.00806952	0.02929169
ENSG000001	152.897825	1.15455902	0.35825746	3.22270752	0.00126985	0.00655607
ENSG000001	1309.3179	0.22457837	0.11067445	2.02918001	0.04243996	0.10655592
ENSG000001	30425.9758	0.25439577	0.12253542	2.07609988	0.03788471	0.09821687
ENSG000001	137.486077	0.22109297	0.079613	2.77709621	0.00548469	0.02133545
ENSG000001	341.184711	-0.2500061	0.12341739	-2.0256956	0.04279598	0.10728413
ENSG000001	868.651008	0.31831196	0.13256617	2.40115535	0.0163434	0.05138841
ENSG000001	572.791021	0.65722388	0.35681775	1.84190354	0.06548926	0.14722888
ENSG000001	1255.52942	0.20340061	0.06633988	3.06603809	0.00216916	0.0100398
ENSG000001	209.296093	-0.3089424	0.17014787	-1.8157291	0.06941194	0.15359148
ENSG000001	2013.40776	-0.2214262	0.09279148	-2.3862776	0.0170199	0.0531166
ENSG000001	25962.6723	0.18236939	0.09358429	1.94871795	0.05132912	0.12226322
ENSG000001	417.876987	-0.172841	0.08711731	-1.984003	0.04725551	0.11513464
ENSG000001	2078.59528	-0.1740185	0.05261563	-3.3073529	0.00094182	0.00514874
ENSG000001	2478.41683	-0.2243965	0.10939673	-2.0512174	0.04024578	0.10273844
ENSG000001	2928.72435	-0.311458	0.12423898	-2.5069264	0.01217861	0.0407463
ENSG000001	437.788359	0.2182503	0.12548327	1.73927803	0.08198587	0.17372083
ENSG000001	176.414228	-0.7140285	0.13998765	-5.1006538	3.38E-07	7.25E-06
ENSG000001	57.5226148	0.33307249	0.19480786	1.70974869	0.08731235	0.18207774
ENSG000001	1041.49158	0.25784767	0.13410288	1.9227601	0.05451019	0.12803
ENSG000001	78.3123709	2.83441965	0.35998611	7.8736918	3.44E-15	8.18E-13
ENSG000001	87.5124977	0.49793233	0.13605711	3.65973025	0.00025248	0.00174342
ENSG000001	249.24284	0.29042731	0.11638182	2.49546971	0.01257905	0.04177732
ENSG000001	5262.59529	0.30933621	0.14959169	2.06787028	0.03865222	0.09963608
ENSG000001	368.980722	-0.2136675	0.06196161	-3.4483857	0.00056395	0.00336765
ENSG000001	2131.52668	-0.3050004	0.15057146	-2.0256189	0.04280386	0.10728413
ENSG000001	806.128274	-0.1002029	0.05246293	-1.9099759	0.05613632	0.13103985
ENSG000001	3935.58837	0.35753061	0.08606957	4.15397213	3.27E-05	0.00033126
ENSG000001	36.5142826	2.14069887	0.40229107	5.32126868	1.03E-07	2.75E-06
ENSG000001	182.503044	0.60712783	0.20314189	2.98868854	0.00280178	0.01231846
ENSG000001	2525.35567	1.15863238	0.18938748	6.11778774	9.49E-10	5.27E-08
ENSG000001	272.315928	0.60442223	0.19206563	3.14695678	0.00164979	0.00809365
ENSG000001	141.479516	0.34389566	0.11227271	3.06303878	0.00219102	0.01011624
ENSG000001	23523.0235	0.42602806	0.12999427	3.27728343	0.00104811	0.00562787
ENSG000001	435.828495	-0.3949887	0.09787825	-4.0355102	5.45E-05	0.0005021

ENSG000001	1330.99828	0.92012622	0.1888124	4.87322982	1.10E-06	1.94E-05
ENSG000001	1756.01101	0.24930354	0.10044258	2.48205035	0.01306288	0.04309372
ENSG000001	3637.73374	0.78040958	0.1537128	5.07706293	3.83E-07	8.04E-06
ENSG000001	97642.1208	0.74668234	0.17847687	4.18363635	2.87E-05	0.00029678
ENSG000001	300.228617	0.20868565	0.09255175	2.25479973	0.0241459	0.06962613
ENSG000001	1200.32034	-0.0960317	0.04851057	-1.9796035	0.04774809	0.11592544
ENSG000001	727.749887	-0.1093726	0.05867412	-1.8640696	0.06231193	0.14202837
ENSG000001	252.520791	0.90113428	0.20753428	4.34209849	1.41E-05	0.00016541
ENSG000001	1663.32888	-0.133483	0.07884656	-1.6929469	0.09046557	0.18711803
ENSG000001	101.613746	0.5666727	0.15419678	3.67499705	0.00023785	0.00165981
ENSG000001	3537.22433	-0.5714921	0.09844887	-5.8049639	6.44E-09	2.75E-07
ENSG000001	2182.59584	-0.2670789	0.0813965	-3.2812083	0.00103363	0.00556044
ENSG000001	9212.8692	0.25671184	0.14414014	1.78098785	0.07491444	0.16251623
ENSG000001	264.385624	0.43414871	0.10510547	4.13060064	3.62E-05	0.00035853
ENSG000001	1861.62373	0.1694172	0.09289943	1.82366234	0.06820314	0.15149629
ENSG000001	40.7184551	0.38190477	0.22196419	1.72056934	0.085329	0.17897224
ENSG000001	1679.96098	-0.1895261	0.09286333	-2.0409145	0.04125933	0.10432527
ENSG000001	4899.66347	0.94434859	0.1361398	6.93660936	4.02E-12	4.55E-10
ENSG000001	688.882972	0.70159166	0.15231563	4.60616973	4.10E-06	5.98E-05
ENSG000001	1053.17035	-0.226783	0.1283952	-1.7662888	0.07734741	0.16615243
ENSG000001	160.532883	-0.2178515	0.07449045	-2.9245563	0.00344948	0.01459479
ENSG000001	354.048768	0.23004473	0.0918363	2.50494336	0.0122471	0.040966
ENSG000001	188.186969	0.45703328	0.16838774	2.71417191	0.00664417	0.02491692
ENSG000001	2748.92169	-0.2011899	0.07768636	-2.5897718	0.00960396	0.0337291
ENSG000001	971.763563	-0.1339046	0.07372874	-1.8161798	0.0693428	0.1534865
ENSG000001	16422.5515	0.18723134	0.07477973	2.50377146	0.01228774	0.041064
ENSG000001	50.5899837	0.54976881	0.18001582	3.05400281	0.0022581	0.01034242
ENSG000001	5778.40892	0.5181289	0.13268062	3.90508359	9.42E-05	0.00078604
ENSG000001	337.188443	0.25291748	0.13008417	1.94426028	0.05186407	0.12315273
ENSG000001	281.549445	-0.5897295	0.25841811	-2.2820751	0.02248491	0.0657527
ENSG000001	935.316224	0.49515426	0.10952176	4.52105841	6.15E-06	8.36E-05
ENSG000001	26345.7447	0.39881878	0.1718454	2.3207999	0.02029765	0.06105731
ENSG000001	434.07429	0.17138575	0.0844487	2.02946585	0.04241086	0.1065198
ENSG000001	1102.30324	0.20474945	0.05894403	3.4736252	0.00051348	0.00312553
ENSG000001	140.530736	-0.5202749	0.12575353	-4.1372587	3.51E-05	0.0003499
ENSG000001	147.359561	-0.6363942	0.21856382	-2.9117088	0.00359458	0.01509207
ENSG000001	50.5993934	-0.4354479	0.25847674	-1.6846694	0.09205237	0.18956115
ENSG000001	95.0434517	0.65474207	0.17716915	3.69557605	0.00021939	0.0015557
ENSG000001	123.216449	1.59861625	0.25960382	6.15790717	7.37E-10	4.25E-08
ENSG000001	514.279515	-0.2262796	0.1175837	-1.924413	0.05430284	0.12764648
ENSG000001	6696.08023	0.90493049	0.14773135	6.12551406	9.04E-10	5.12E-08
ENSG000001	4730.44227	1.77299694	0.25981396	6.82410193	8.85E-12	9.27E-10
ENSG000001	4497.54383	0.47670468	0.17180988	2.77460582	0.00552686	0.02147739

ENSG000001	4306.18961	0.58655525	0.15221935	3.85335523	0.00011651	0.00093823
ENSG000001	10046.4703	0.26417514	0.13762075	1.91958802	0.05490996	0.12880997
ENSG000001	220.947837	1.03888307	0.2535183	4.09786231	4.17E-05	0.00040275
ENSG000001	8757.92178	0.70414591	0.20303105	3.46816867	0.00052402	0.00317501
ENSG000001	3805.80869	0.37237969	0.12335913	3.01866326	0.00253893	0.01138039
ENSG000001	1482.3779	0.30290029	0.0856798	3.53525897	0.00040738	0.00258731
ENSG000001	2559.61088	0.24058962	0.09423579	2.55306	0.01067811	0.0367531
ENSG000001	2481.72572	1.25951317	0.32763924	3.84420736	0.00012094	0.00096641
ENSG000001	540.390181	1.06356282	0.58230039	1.82648479	0.06777727	0.15087687
ENSG000001	17.7737506	0.5569677	0.228775	2.43456543	0.01490968	0.04790396
ENSG000001	131.007944	1.07285643	0.19920214	5.38576755	7.21E-08	2.05E-06
ENSG000001	2041.5535	0.31046048	0.12006791	2.58570739	0.00971794	0.03406334
ENSG000001	25.2296798	-1.2805697	0.19093999	-6.7066607	1.99E-11	1.86E-09
ENSG000001	74.2437262	-0.3209055	0.13752982	-2.3333525	0.01962965	0.05948018
ENSG000001	1766.99064	0.16790878	0.07713406	2.17684341	0.02949225	0.08087342
ENSG000001	142.679308	0.67190227	0.1328525	5.05750563	4.25E-07	8.82E-06
ENSG000001	1988.56562	1.23518907	0.27374013	4.51226894	6.41E-06	8.65E-05
ENSG000001	1496.30579	0.17754474	0.08507973	2.08680425	0.03690583	0.09626482
ENSG000001	58710.7657	0.38790855	0.18965704	2.04531577	0.04082374	0.1035569
ENSG000001	6637.88343	0.24059082	0.09230802	2.60639142	0.00915018	0.03251422
ENSG000001	4041.39741	-0.1750908	0.05827325	-3.0046521	0.00265885	0.01181194
ENSG000001	2717.96363	-0.3191982	0.06131302	-5.2060431	1.93E-07	4.57E-06
ENSG000001	1866.39919	-0.1600089	0.04652403	-3.4392745	0.00058328	0.00346308
ENSG000001	84.3773771	-0.2292317	0.11022271	-2.0797137	0.0375518	0.0975528
ENSG000001	7831.94146	0.46040922	0.13894381	3.31363607	0.00092091	0.00504886
ENSG000001	1803.17598	-0.1534877	0.09284584	-1.6531454	0.09830128	0.19897871
ENSG000001	139.719323	-0.3832838	0.17217992	-2.2260659	0.02600977	0.07361973
ENSG000001	5690.25874	-0.1515208	0.06692189	-2.2641433	0.0235653	0.06823717
ENSG000001	16114.8402	0.74421	0.16313508	4.56192498	5.07E-06	7.15E-05
ENSG000001	1713.8934	-0.3672103	0.1228907	-2.9881049	0.00280713	0.01233452
ENSG000001	3778.90922	-0.2279178	0.08766512	-2.5998688	0.00932594	0.03302491
ENSG000001	464.630865	-0.1602494	0.04274195	-3.7492303	0.00017738	0.0013119
ENSG000001	10674.2589	0.19874149	0.07521994	2.64213834	0.00823844	0.02977059
ENSG000001	1984.21651	0.67635929	0.13413834	5.04225163	4.60E-07	9.47E-06
ENSG000001	203.037825	-0.6172689	0.20083235	-3.0735531	0.00211526	0.00983749
ENSG000001	2561.53412	-0.2357119	0.06527827	-3.6108791	0.00030516	0.00204386
ENSG000001	844.847155	-0.2211093	0.11320268	-1.9532157	0.05079404	0.12124062
ENSG000001	11871.0033	2.02236463	0.24404751	8.28676605	1.16E-16	3.51E-14
ENSG000001	748.944249	-0.2944281	0.11446772	-2.5721501	0.01010691	0.03517145
ENSG000001	2076.48238	0.17631157	0.04564236	3.86289352	0.00011205	0.00090738
ENSG000001	22064.3146	0.61310767	0.13466563	4.55281482	5.29E-06	7.43E-05
ENSG000001	204.628459	-0.3808656	0.13917208	-2.7366521	0.00620679	0.02355701
ENSG000001	859.489911	0.29289999	0.06406898	4.57163468	4.84E-06	6.89E-05

ENSG000001	3232.48158	0.24460814	0.1324179	1.84724374	0.06471182	0.14613877
ENSG000001	5286.0529	-0.1745511	0.07087713	-2.4627281	0.01378844	0.04506049
ENSG000001	84.2811408	-0.3824809	0.14288613	-2.6768232	0.00743238	0.02728743
ENSG000001	947.816505	2.07165581	0.40771292	5.08116303	3.75E-07	7.91E-06
ENSG000001	30.5259038	1.77527538	0.39096032	4.5408071	5.60E-06	7.79E-05
ENSG000001	3951.61522	-0.2176138	0.07246545	-3.0030005	0.00267332	0.01186168
ENSG000001	233.668822	-0.6685408	0.17860191	-3.7431897	0.0001817	0.0013328
ENSG000001	2035.55213	0.53949988	0.1170349	4.60973503	4.03E-06	5.90E-05
ENSG000001	1851.78715	-0.1288065	0.0446506	-2.8847654	0.00391705	0.0161555
ENSG000001	280.164292	0.99081839	0.14520951	6.82337109	8.89E-12	9.27E-10
ENSG000001	1904.14694	0.15847022	0.0743278	2.13204497	0.03300315	0.08823097
ENSG000001	6661.30507	0.61557159	0.17011583	3.61854392	0.00029627	0.00199257
ENSG000001	1000.69842	0.22289418	0.05484999	4.06370487	4.83E-05	0.00045495
ENSG000001	325.90504	-0.1464814	0.08655601	-1.6923303	0.09058301	0.18721165
ENSG000001	29.4884192	-0.8729921	0.23725052	-3.6796214	0.00023358	0.00163394
ENSG000001	754.589496	0.12288761	0.06003207	2.04703253	0.04065489	0.10327493
ENSG000001	46.0131901	0.61102729	0.2580088	2.36824205	0.01787284	0.05516059
ENSG000001	2119.56372	0.69787651	0.11378781	6.1331393	8.62E-10	4.91E-08
ENSG000001	4113.60083	-0.2658084	0.07505531	-3.5415	0.00039786	0.00253911
ENSG000001	194.567941	0.62409614	0.15602751	3.99991079	6.34E-05	0.00056651
ENSG000001	37.0234974	0.85796514	0.24840815	3.45385266	0.00055264	0.00331197
ENSG000001	27037.1941	0.3608924	0.20937085	1.72369936	0.08476213	0.17806685
ENSG000001	1918.73114	0.34257829	0.16852944	2.03275045	0.04207774	0.10592185
ENSG000001	378.018065	-0.5385593	0.1258215	-4.2803437	1.87E-05	0.00020811
ENSG000001	1961.35468	-0.1320754	0.06267361	-2.1073529	0.03508701	0.09257065
ENSG000001	830.230958	-0.236816	0.07079782	-3.344961	0.00082294	0.00460841
ENSG000001	100.103146	1.0773938	0.31649925	3.40409589	0.00066383	0.00384764
ENSG000001	1018.21906	0.14781594	0.07213001	2.04929882	0.0404329	0.10294873
ENSG000001	5158.81892	0.31842283	0.12108368	2.62977503	0.00854414	0.03069914
ENSG000001	5204.26379	0.294887	0.10571854	2.78935928	0.00528124	0.02071741
ENSG000001	142.95679	0.40865645	0.17749373	2.30237118	0.02131425	0.06336539
ENSG000001	99.3505411	-0.4050095	0.17379901	-2.3303327	0.01978857	0.05987408
ENSG000001	277.103207	0.36676128	0.10383599	3.53212087	0.00041224	0.002614
ENSG000001	1473.68891	-0.2336551	0.0716909	-3.2592022	0.00111726	0.00591368
ENSG000001	5161.96735	2.00551441	0.2976102	6.73872876	1.60E-11	1.54E-09
ENSG000001	1415.93168	-0.3893311	0.09512386	-4.0928861	4.26E-05	0.00041037
ENSG000001	64.6164303	0.75771043	0.20797597	3.64325949	0.00026921	0.00184747
ENSG000001	582.595426	0.14866796	0.06604438	2.25103117	0.02438356	0.07018571
ENSG000001	15521.5702	-0.1799479	0.08227422	-2.1871722	0.02872995	0.07935383
ENSG000001	92.4969563	1.39269222	0.2669424	5.21720118	1.82E-07	4.37E-06
ENSG000001	1881.34886	0.5934609	0.1040907	5.7013826	1.19E-08	4.64E-07
ENSG000001	2453.46474	-0.1489576	0.07442163	-2.001536	0.04533466	0.11175915
ENSG000001	793.451574	-0.2108012	0.11799915	-1.7864634	0.07402425	0.16107887

ENSG000001	1344.58736	0.17393935	0.08860682	1.96304694	0.04964072	0.11938
ENSG000001	63.2750415	0.63118445	0.21098338	2.99163122	0.00277491	0.01221518
ENSG000001	1069.88668	0.14979162	0.06525958	2.2953199	0.0217148	0.06421357
ENSG000001	781.334585	-0.2530408	0.07873992	-3.2136283	0.00131069	0.00672141
ENSG000001	515.133811	-0.2966605	0.09372439	-3.1652427	0.00154954	0.00767649
ENSG000001	257.723966	0.56675684	0.15239311	3.71904506	0.00019998	0.0014399
ENSG000001	2435.82265	-0.2213512	0.1239573	-1.7857051	0.07414701	0.16121383
ENSG000001	5970.65698	-0.4279356	0.16275594	-2.6293086	0.00855587	0.03071843
ENSG000001	906.99242	0.14149631	0.08264229	1.71215393	0.08686831	0.18130833
ENSG000001	999.357389	-0.1702817	0.08814648	-1.9318036	0.05338375	0.12605874
ENSG000001	649.218418	-0.2468379	0.12994782	-1.8995153	0.05749676	0.13346508
ENSG000001	1665.46227	-0.1896776	0.08674404	-2.1866361	0.0287691	0.0794468
ENSG000001	4569.95201	0.65032778	0.21542655	3.01879125	0.00253785	0.0113791
ENSG000001	90.8850993	0.82019371	0.21326501	3.84588973	0.00012012	0.00096192
ENSG000001	245.039701	-0.3439811	0.10074819	-3.4142653	0.00063954	0.00373478
ENSG000001	182.179328	-0.4238228	0.17856922	-2.3734372	0.01762339	0.05454177
ENSG000001	2505.90165	0.4542788	0.1234203	3.68074614	0.00023255	0.00162832
ENSG000001	10252.6036	0.21770167	0.11142388	1.95381513	0.05072308	0.12117832
ENSG000001	125.916574	0.35126595	0.14456099	2.42988068	0.01510379	0.048409
ENSG000001	3768.82367	0.38760311	0.09011482	4.30121373	1.70E-05	0.00019243
ENSG000001	772.586995	0.18492119	0.06908018	2.67690665	0.00743053	0.02728743
ENSG000001	9017.17953	-0.4515443	0.11429819	-3.950581	7.80E-05	0.00067509
ENSG000001	15274.8798	0.50371726	0.14473175	3.48035071	0.00050076	0.00306484
ENSG000001	4987.71028	-0.1608011	0.09625511	-1.6705717	0.09480631	0.19347218
ENSG000001	68.4149608	-0.3942899	0.12416977	-3.1754098	0.00149625	0.00745467
ENSG000001	829.896302	0.16853184	0.06771097	2.48898878	0.0128107	0.04239119
ENSG000001	92.6166509	0.40837625	0.22836919	1.78822829	0.07373918	0.1606436
ENSG000001	2003.09416	-0.3250724	0.07898929	-4.1153979	3.87E-05	0.00037707
ENSG000001	1601.89949	0.25509553	0.06990016	3.64942692	0.00026283	0.00180882
ENSG000001	1379.66313	0.15960493	0.07033134	2.26932869	0.02324835	0.06750827
ENSG000001	2498.22744	-0.5151132	0.11616097	-4.4344774	9.23E-06	0.00011708
ENSG000001	104.589585	0.3626847	0.16176745	2.24201279	0.02496055	0.07142078
ENSG000001	5438.02142	0.1766715	0.09947854	1.77597595	0.07573688	0.16376055
ENSG000001	712.959663	-0.1764712	0.06665203	-2.6476492	0.00810536	0.02940704
ENSG000001	110.111783	0.50180767	0.12489465	4.01784752	5.87E-05	0.00053442
ENSG000001	1353.94474	0.20625415	0.05907254	3.49154043	0.00048024	0.00297066
ENSG000001	5099.79326	-0.1922145	0.07649998	-2.5126091	0.0119842	0.04025682
ENSG000001	644.427703	0.47260931	0.11540718	4.0951466	4.22E-05	0.00040666
ENSG000001	2011.03631	-0.3003204	0.10074337	-2.981044	0.00287267	0.01257294
ENSG000001	11002.1857	0.14004747	0.05838496	2.39869094	0.0164538	0.05166149
ENSG000001	123.727275	-0.5948209	0.12742554	-4.6679883	3.04E-06	4.65E-05
ENSG000001	1982.88109	0.13576555	0.07999744	1.69712373	0.08967327	0.18574445
ENSG000001	1664.68801	-0.1790287	0.08419685	-2.1263112	0.03347736	0.08929967

ENSG000001	2010.51936	-0.1435366	0.07534857	-1.9049677	0.05678427	0.13225622
ENSG000001	253.297848	-0.442836	0.14735108	-3.0053123	0.00265308	0.01179718
ENSG000001	332.489743	-0.4832521	0.15483741	-3.121029	0.0018022	0.00869414
ENSG000001	14032.7594	-0.3999209	0.11420087	-3.5019079	0.00046194	0.00287709
ENSG000001	3168.15114	-0.2703362	0.11496305	-2.3515048	0.01869765	0.05735187
ENSG000001	39512.3401	0.74979899	0.17043777	4.39925356	1.09E-05	0.00013325
ENSG000001	1966.07865	-0.3478227	0.08451747	-4.1153945	3.87E-05	0.00037707
ENSG000001	11331.1524	-0.3657143	0.16879027	-2.1666787	0.03025936	0.0826173
ENSG000001	489.292195	-0.8342144	0.14666114	-5.6880399	1.29E-08	4.86E-07
ENSG000001	3211.84215	-0.2650318	0.10957879	-2.4186413	0.01557859	0.04953623
ENSG000001	2113.62697	-0.4513919	0.10267985	-4.3961101	1.10E-05	0.0001348
ENSG000001	985.600443	0.1225024	0.04514169	2.71373097	0.00665302	0.02494365
ENSG000001	2283.33369	0.1075852	0.06046069	1.77942385	0.0751703	0.16292521
ENSG000001	670.483182	-0.345839	0.09931986	-3.4820729	0.00049755	0.00305423
ENSG000001	35157.5119	3.10593742	0.32118586	9.67021852	4.04E-22	2.92E-19
ENSG000001	1764.35509	-0.1187484	0.06811647	-1.7433146	0.08127868	0.17255045
ENSG000001	936.204586	0.24045091	0.09034929	2.66134804	0.00778285	0.02840768
ENSG000001	1822.45314	-0.2155292	0.09756089	-2.209176	0.0271624	0.0760096
ENSG000001	1370.60006	-0.1485935	0.05710632	-2.6020507	0.00926681	0.03286373
ENSG000001	116.238395	1.73762884	0.3027054	5.74032976	9.45E-09	3.81E-07
ENSG000001	2960.83837	0.4460052	0.11429174	3.90233994	9.53E-05	0.00079318
ENSG000001	3434.01916	0.97294625	0.2329882	4.17594651	2.97E-05	0.00030533
ENSG000001	238.926627	0.33723356	0.18817379	1.79213884	0.07311073	0.15977572
ENSG000001	342.056962	-0.1807045	0.09253495	-1.9528243	0.05084042	0.12131859
ENSG000001	14.9840666	0.85114707	0.2930541	2.90440258	0.00367955	0.01538401
ENSG000001	8596.73235	-0.2161784	0.05696737	-3.7947755	0.00014778	0.00113093
ENSG000001	9108.71548	0.27089234	0.11651513	2.32495409	0.02007441	0.06061235
ENSG000001	2113.44227	-0.2838615	0.0860476	-3.2988886	0.00097068	0.00528261
ENSG000001	6609.18925	0.27166748	0.10673527	2.54524555	0.01092009	0.03740822
ENSG000001	238.999025	-0.2881016	0.11050811	-2.6070632	0.00913225	0.03247396
ENSG000001	1489.48242	-0.2402109	0.09440327	-2.5445194	0.01094282	0.03746837
ENSG000001	12.1456648	1.15897337	0.4613605	2.51207757	0.01200227	0.04027725
ENSG000001	178.276769	0.15777583	0.06349094	2.48501311	0.01295467	0.04279914
ENSG000001	273.447931	0.38415771	0.12634739	3.0404879	0.00236195	0.01071644
ENSG000001	41488.6824	0.23460294	0.13085082	1.79290382	0.0729883	0.15962886
ENSG000001	1818.73272	0.08808393	0.04755501	1.85225344	0.06398944	0.14500469
ENSG000001	3781.61354	-0.5783792	0.11665971	-4.9578322	7.13E-07	1.36E-05
ENSG000001	1854.22132	0.79857574	0.19573686	4.07984341	4.51E-05	0.00042952
ENSG000001	1979.06573	0.18534284	0.09704256	1.90991291	0.05614443	0.13103985
ENSG000001	2254.69048	0.14718016	0.07356398	2.0007096	0.04542369	0.11191601
ENSG000001	501.307263	0.16337347	0.07519004	2.17280737	0.02979482	0.08151795
ENSG000001	3386.66941	-0.3346287	0.13255334	-2.524483	0.01158686	0.03921936
ENSG000001	411.051397	-0.2480882	0.14623654	-1.6964858	0.08979392	0.18588804

ENSG000001	3225.16903	0.14755619	0.08326854	1.77205223	0.07638589	0.16479558
ENSG000001	1032.6744	-0.1910155	0.04069398	-4.693951	2.68E-06	4.17E-05
ENSG000001	1085.69947	-0.2481027	0.0661792	-3.7489523	0.00017757	0.0013119
ENSG000001	9968.52548	0.27583069	0.05661458	4.87207838	1.10E-06	1.95E-05
ENSG000001	9694.4097	-0.2467929	0.0689264	-3.5805282	0.0003429	0.00224693
ENSG000001	1835.65296	-0.2826664	0.05600297	-5.0473463	4.48E-07	9.23E-06
ENSG000001	18645.9573	-0.3979233	0.10607461	-3.7513528	0.00017588	0.001304
ENSG000001	988.209664	0.2940855	0.10035649	2.93040828	0.00338517	0.01438422
ENSG000001	122.837542	1.04750878	0.21154071	4.95180712	7.35E-07	1.39E-05
ENSG000001	83.2751566	0.36301253	0.21271724	1.7065496	0.0879058	0.18301411
ENSG000001	11249.1739	0.21146348	0.08745957	2.4178427	0.01561282	0.04960153
ENSG000001	3211.24967	-0.2914401	0.079565	-3.6629186	0.00024936	0.00172844
ENSG000001	71.7315525	0.23888906	0.14203294	1.68192722	0.09258295	0.19036326
ENSG000001	1193.08722	-0.245272	0.07850242	-3.1243872	0.00178176	0.00862135
ENSG000001	3601.77222	-0.3719617	0.08214482	-4.5281208	5.95E-06	8.18E-05
ENSG000001	1761.86044	-0.4181405	0.09787978	-4.2719799	1.94E-05	0.00021442
ENSG000001	57.7718739	-0.5434525	0.18922691	-2.871962	0.00407932	0.01670953
ENSG000001	493.163832	0.43618989	0.11443504	3.81168131	0.00013802	0.00107215
ENSG000001	5482.75527	0.40093232	0.23526051	1.70420576	0.08834265	0.18369743
ENSG000001	22632.6967	0.62960801	0.1768159	3.56081113	0.00036971	0.00239214
ENSG000001	538.186581	0.21530103	0.07441538	2.89323301	0.00381298	0.01582264
ENSG000001	803.762826	-0.2044813	0.12375996	-1.6522414	0.09848536	0.19921214
ENSG000001	2032.81239	0.36510974	0.18629628	1.95983377	0.05001522	0.11994215
ENSG000001	262.000668	-0.3482388	0.13130629	-2.6521105	0.00799904	0.02906497
ENSG000001	3885.42707	-0.1815092	0.0927664	-1.9566266	0.05039138	0.12060541
ENSG000001	6938.40937	0.29442031	0.06182839	4.76189472	1.92E-06	3.16E-05
ENSG000001	20.2834022	-0.9770372	0.48790037	-2.0025341	0.04522731	0.11161978
ENSG000001	260.72189	-0.5179629	0.25138602	-2.0604285	0.03935759	0.10096927
ENSG000001	5782.49442	0.14658449	0.0765884	1.91392554	0.05562967	0.13008992
ENSG000001	14250.6141	0.59260022	0.15284342	3.87717188	0.00010568	0.00086348
ENSG000001	61.4683021	2.03047739	0.32400123	6.26688166	3.68E-10	2.37E-08
ENSG000001	11394.5708	1.20263339	0.19803821	6.07273408	1.26E-09	6.62E-08
ENSG000001	1074.49415	0.31988934	0.11296914	2.83165252	0.00463081	0.0185784
ENSG000001	1248.4978	-0.086235	0.05027678	-1.7152045	0.08630774	0.18050241
ENSG000001	87.0951389	0.25513204	0.13030099	1.9580208	0.05022757	0.12031198
ENSG000001	1402.34064	-0.3743516	0.10567681	-3.54242	0.00039647	0.00253362
ENSG000001	1538.95861	1.03470409	0.21945297	4.71492414	2.42E-06	3.87E-05
ENSG000001	783.199253	0.20932085	0.0911175	2.29726272	0.02160379	0.06395056
ENSG000001	112.379407	-0.5124695	0.16316689	-3.1407692	0.00168505	0.00823037
ENSG000001	2806.37234	0.4784543	0.1162197	4.11680887	3.84E-05	0.00037578
ENSG000001	5946.67974	0.40940325	0.11618893	3.52359955	0.00042573	0.00268153
ENSG000001	477.750036	0.50050856	0.1515543	3.3025032	0.00095826	0.00522088
ENSG000001	1140.5518	0.73602878	0.13455974	5.46990339	4.50E-08	1.41E-06

ENSG000001	6502.23476	0.34319394	0.15455522	2.22052637	0.02638306	0.07434572
ENSG000001	4333.54932	0.76973133	0.15724436	4.89512826	9.82E-07	1.77E-05
ENSG000001	883.784906	0.28515193	0.07430113	3.83778697	0.00012415	0.00098869
ENSG000001	95.4402394	0.99252404	0.20334562	4.88097078	1.06E-06	1.88E-05
ENSG000001	1219.33404	0.34331816	0.13923296	2.46578227	0.01367144	0.04472858
ENSG000001	409.630241	0.35570675	0.0918452	3.87289441	0.00010755	0.00087582
ENSG000001	5077.35472	0.10494684	0.05414193	1.93836518	0.05257869	0.1245434
ENSG000001	2318.45627	0.20979902	0.07343048	2.85711075	0.00427517	0.01737776
ENSG000001	5858.48258	0.08629974	0.05159885	1.67251287	0.09442323	0.19290855
ENSG000001	1052.62016	0.20519439	0.05207136	3.94063797	8.13E-05	0.00069662
ENSG000001	3467.45763	-0.2203035	0.07198987	-3.0602016	0.00221188	0.01018548
ENSG000001	233.017022	-0.4329689	0.16388017	-2.6419848	0.00824218	0.02977666
ENSG000001	7597.09688	0.11273065	0.05443818	2.07080126	0.03837737	0.09908626
ENSG000001	408.468477	-0.1974929	0.0969664	-2.0367145	0.04167866	0.10512674
ENSG000001	60.1762115	1.69708422	0.29162987	5.81930857	5.91E-09	2.57E-07
ENSG000001	2034.80616	-0.2512947	0.07080341	-3.5491893	0.00038642	0.00247921
ENSG000001	654.50198	0.46913677	0.1014567	4.62400955	3.76E-06	5.55E-05
ENSG000001	2070.35471	-0.1327093	0.07710554	-1.7211386	0.08522568	0.17878141
ENSG000001	2723.02117	0.14287356	0.08414911	1.69786181	0.08953385	0.18558515
ENSG000001	2122.41073	0.23689199	0.08455366	2.80167622	0.00508379	0.02007874
ENSG000001	4590.70885	0.25444573	0.12467495	2.0408729	0.04126346	0.10432527
ENSG000001	941.033644	0.56830686	0.25292022	2.24698078	0.02464126	0.07075485
ENSG000001	2930.16714	-0.1830261	0.08828321	-2.0731702	0.03815644	0.09873211
ENSG000001	12576.3245	-0.2046453	0.0768888	-2.6615753	0.00777759	0.02840282
ENSG000001	148.247506	0.44131345	0.22571487	1.95518116	0.05056169	0.12087248
ENSG000001	8650.61937	0.45511706	0.16468347	2.76358685	0.00571699	0.02206233
ENSG000001	868.637783	0.39811104	0.10804549	3.68466152	0.00022901	0.0016105
ENSG000001	418.028059	0.38960118	0.09791947	3.97879193	6.93E-05	0.00061224
ENSG000001	283.112961	0.8980827	0.17879102	5.0230861	5.08E-07	1.03E-05
ENSG000001	4807.91127	0.36315135	0.08531013	4.25683716	2.07E-05	0.0002267
ENSG000001	172.764747	2.42539818	0.36182124	6.70330523	2.04E-11	1.89E-09
ENSG000001	5386.9646	0.35883628	0.08579196	4.18263317	2.88E-05	0.00029754
ENSG000001	6567.09135	0.27514532	0.05865961	4.69054101	2.72E-06	4.23E-05
ENSG000001	38.9840587	0.41528039	0.22487026	1.8467555	0.06478258	0.1461861
ENSG000001	17.1313276	1.83192609	0.39746855	4.6089838	4.05E-06	5.92E-05
ENSG000001	6125.24648	0.19342165	0.06656111	2.9059259	0.00366168	0.01533587
ENSG000001	1691.71196	0.19642192	0.07210798	2.7239969	0.00644971	0.02431355
ENSG000001	9399.91137	0.6435731	0.12638888	5.09200727	3.54E-07	7.54E-06
ENSG000001	70.8294455	0.88108751	0.18058932	4.87895698	1.07E-06	1.90E-05
ENSG000001	8900.66581	-0.1194709	0.06017966	-1.9852369	0.04711813	0.11495458
ENSG000001	493.830148	-0.2703685	0.07922435	-3.4126947	0.00064324	0.00375145
ENSG000001	93.6697618	0.58137324	0.34657068	1.67750268	0.09344423	0.19163739
ENSG000001	78.9682943	-0.7450403	0.28954989	-2.5730982	0.01007926	0.03508367

ENSG000001	1764.44984	-0.199004	0.1022815	-1.9456502	0.05169678	0.12289913
ENSG000001	11013.073	0.24128207	0.08869345	2.72040473	0.00652021	0.0245346
ENSG000001	44.7954752	0.82450691	0.29575089	2.78784258	0.00530603	0.02079583
ENSG000001	7.12483754	1.34322042	0.55067265	2.43923576	0.01471836	0.04739385
ENSG000001	241.569276	-0.3319449	0.13937919	-2.3815955	0.01723782	0.05357741
ENSG000001	1195.83123	0.10919056	0.06616119	1.65037166	0.09886694	0.19964949
ENSG000001	2000.58911	-0.2607582	0.10043199	-2.5963664	0.00942155	0.03327134
ENSG000001	1225.18992	0.21957152	0.07975886	2.7529422	0.00590623	0.0226239
ENSG000001	549.711297	0.27623993	0.12409654	2.2260083	0.02601363	0.07361973
ENSG000001	43704.2789	1.02300499	0.14855848	6.88621047	5.73E-12	6.15E-10
ENSG000001	84.1368592	-0.7277943	0.14443784	-5.0388066	4.68E-07	9.63E-06
ENSG000001	4559.75362	0.41885176	0.14194867	2.95072695	0.00317027	0.01365271
ENSG000001	153.847627	0.35609365	0.1932784	1.84238722	0.06541853	0.14720693
ENSG000001	760.080515	0.23123458	0.12586349	1.83718549	0.06618252	0.14833961
ENSG000001	2498.76119	-0.1776941	0.10642	-1.6697439	0.09497005	0.19375174
ENSG000001	214.080083	0.67536166	0.20638669	3.27231211	0.00106672	0.00570873
ENSG000001	10467.4239	-0.3798985	0.10303679	-3.6870185	0.0002269	0.00159721
ENSG000001	738.242892	-0.2164455	0.11014878	-1.9650285	0.04941094	0.11894746
ENSG000001	858.116355	-0.1801735	0.09432229	-1.9101902	0.05610873	0.13099874
ENSG000001	157.28961	0.39830124	0.19436161	2.04927941	0.0404348	0.10294873
ENSG000001	4647.3144	-0.1483645	0.06545113	-2.266799	0.02340251	0.06786072
ENSG000001	326.437915	0.31269277	0.08844862	3.53530422	0.00040731	0.00258731
ENSG000001	10817.625	1.14979057	0.19404109	5.92550056	3.11E-09	1.48E-07
ENSG000001	1120.22948	-0.3075418	0.11451138	-2.6856878	0.00723807	0.02672219
ENSG000001	998.693405	-0.5298839	0.08358098	-6.3397664	2.30E-10	1.68E-08
ENSG000001	648.420823	0.24535142	0.12121021	2.02418122	0.04295151	0.10754209
ENSG000001	3345.81896	-0.2860097	0.1418334	-2.0165188	0.04374575	0.10877869
ENSG000001	3893.54376	-0.103087	0.05215427	-1.9765785	0.04808929	0.11660448
ENSG000001	974.523555	0.19263384	0.09018083	2.13608409	0.03267256	0.08752355
ENSG000001	128.794128	-1.4810555	0.34889446	-4.2449956	2.19E-05	0.00023775
ENSG000001	5659.31385	-0.2269839	0.10869442	-2.0882757	0.03677297	0.09593554
ENSG000001	2502.1893	-0.2940414	0.14362317	-2.0473119	0.04062747	0.10323982
ENSG000001	1782.68025	1.96998868	0.28499542	6.91235199	4.77E-12	5.23E-10
ENSG000001	28.3551286	1.05246732	0.25490162	4.12891573	3.64E-05	0.00036018
ENSG000001	458.790972	0.2593169	0.05718669	4.53456725	5.77E-06	7.98E-05
ENSG000001	174.691745	-0.5347371	0.3200295	-1.6708993	0.09474156	0.19339454
ENSG000001	966.992241	-0.2789778	0.06925545	-4.0282439	5.62E-05	0.00051465
ENSG000001	193.013246	-0.318872	0.11389034	-2.7998156	0.00511318	0.02016186
ENSG000001	24365.8797	1.83435039	0.23286643	7.87726409	3.35E-15	8.08E-13
ENSG000001	15762.8446	-0.4059645	0.17063943	-2.3790781	0.017356	0.05384077
ENSG000001	160.544242	0.9312663	0.2148794	4.33390214	1.46E-05	0.00017073
ENSG000001	4824.84787	-0.1466791	0.07323926	-2.0027391	0.0452053	0.11158445
ENSG000001	503.201881	-0.3530771	0.09219258	-3.829778	0.00012826	0.00101258

ENSG000001	1017.09028	-0.1338317	0.07869305	-1.7006798	0.08900312	0.18472611
ENSG000001	324.040341	-0.4285752	0.10285755	-4.1666864	3.09E-05	0.00031575
ENSG000001	1847.07159	0.16479556	0.05312268	3.10216953	0.00192108	0.0091278
ENSG000001	9182.5448	-0.416291	0.09489441	-4.3868862	1.15E-05	0.00013951
ENSG000001	2974.98973	-0.192047	0.07780126	-2.4684306	0.0135707	0.04444917
ENSG000001	17221.4456	0.66256215	0.14113091	4.69466363	2.67E-06	4.16E-05
ENSG000001	2115.62782	-0.2858046	0.04822965	-5.9259101	3.11E-09	1.48E-07
ENSG000001	92.0981282	0.42314272	0.20251387	2.08945053	0.03666719	0.09572852
ENSG000001	267.633523	0.4073767	0.10964652	3.7153637	0.00020291	0.00145524
ENSG000001	2230.63449	-0.201732	0.03796545	-5.3135674	1.07E-07	2.86E-06
ENSG000001	7804.7925	-0.1765078	0.090233	-1.9561341	0.05044936	0.12066368
ENSG000001	4550.31841	0.90578378	0.15717869	5.76276448	8.27E-09	3.42E-07
ENSG000001	4078.34417	-0.1828187	0.08931483	-2.0469023	0.04066768	0.10327493
ENSG000001	1990.53921	-0.2853894	0.07007467	-4.0726471	4.65E-05	0.00043983
ENSG000001	639.605786	-0.337408	0.10748524	-3.13911	0.00169462	0.00826319
ENSG000001	674.893961	-0.1563121	0.09337432	-1.6740371	0.09412331	0.19261406
ENSG000001	801.234241	-0.1751467	0.08485742	-2.0640122	0.03901656	0.10037877
ENSG000001	431.514423	0.16954574	0.10128116	1.67401071	0.09412849	0.19261406
ENSG000001	488.272196	-0.3803657	0.11625705	-3.2717645	0.00106879	0.00571558
ENSG000001	18305.1345	-0.2602327	0.1162496	-2.2385683	0.02518402	0.07191816
ENSG000001	14.823169	6.16604029	2.75776135	2.23588611	0.02535923	0.07217666
ENSG000001	187.957974	-1.3275186	0.28769691	-4.6142955	3.94E-06	5.79E-05
ENSG000001	82.8782624	0.47549136	0.14983511	3.17343088	0.00150649	0.00749467
ENSG000001	970.479648	-0.3235917	0.11397168	-2.8392293	0.00452227	0.0181933
ENSG000001	948.452655	-0.2544166	0.0843152	-3.0174465	0.00254914	0.01141226
ENSG000001	6232.34613	0.33501869	0.09669013	3.46486968	0.00053049	0.00320617
ENSG000001	429.546511	-0.2838634	0.13262875	-2.1402857	0.03233168	0.08680301
ENSG000001	3149.73937	-0.1709396	0.07701064	-2.2196883	0.02643993	0.07446254
ENSG000001	126.586462	1.07533352	0.23361267	4.60306156	4.16E-06	6.06E-05
ENSG000001	8833.65456	0.24568571	0.09483726	2.59060326	0.00958079	0.03368038
ENSG000001	569.532813	0.76121435	0.18768241	4.05586409	4.99E-05	0.00046806
ENSG000001	192.464834	0.38097256	0.1935894	1.9679412	0.0490748	0.1182546
ENSG000001	1759.57157	-0.5202966	0.08016299	-6.4904841	8.56E-11	6.96E-09
ENSG000001	3497.6616	0.21755692	0.08706254	2.498858	0.01245942	0.04144653
ENSG000001	467.241246	0.14330994	0.06165784	2.32427757	0.02011062	0.06067109
ENSG000001	2300.73759	-0.3496806	0.0882575	-3.9620497	7.43E-05	0.00064811
ENSG000001	1670.87752	0.07118417	0.03484528	2.04286443	0.04106586	0.10397084
ENSG000001	38882.0137	0.27338542	0.15605202	1.75188644	0.07979334	0.16994503
ENSG000001	380.913806	-0.4221623	0.15335926	-2.752767	0.00590939	0.02263002
ENSG000001	54.7811991	0.24270896	0.14465914	1.67779898	0.09338635	0.19157293
ENSG000001	24843.3737	0.26378535	0.10274506	2.56737732	0.0102471	0.03556536
ENSG000001	6388.07076	-0.247669	0.07907393	-3.1321196	0.00173549	0.00842562
ENSG000001	8788.03188	0.50954745	0.17594542	2.89605414	0.00377887	0.01572215

ENSG000001	118.426094	0.23085484	0.10104273	2.28472499	0.02232896	0.06542873
ENSG000001	5406.48367	-0.2146384	0.08121668	-2.642787	0.00822267	0.02973587
ENSG000001	1749.8717	0.25121526	0.0751891	3.34111251	0.00083443	0.00465836
ENSG000001	233.657739	0.24668686	0.10316896	2.39109576	0.01679817	0.05252646
ENSG000001	5720.35746	-0.1660153	0.05501937	-3.0173975	0.00254955	0.01141226
ENSG000001	4325.19127	-0.1385023	0.04723267	-2.9323403	0.00336418	0.01431332
ENSG000001	2045.40755	-0.177858	0.07023149	-2.5324541	0.01132672	0.0384807
ENSG000001	4124.75114	0.18207187	0.08734452	2.08452528	0.03711241	0.09664705
ENSG000001	1760.67802	-0.30429	0.16871898	-1.8035317	0.07130475	0.15687044
ENSG000001	940.850256	0.20710355	0.05005856	4.1372259	3.52E-05	0.0003499
ENSG000001	333.854687	1.18424533	0.25052825	4.72699312	2.28E-06	3.69E-05
ENSG000001	6780.27323	0.16724154	0.07856473	2.12871004	0.03327826	0.088834
ENSG000001	1085.21422	-0.1487614	0.05414767	-2.7473275	0.00600831	0.02291542
ENSG000001	1407.89506	-0.1428915	0.08064598	-1.7718368	0.07642165	0.16482891
ENSG000001	529.820706	-0.2602227	0.09841643	-2.6440982	0.00819089	0.02965794
ENSG000001	105.516076	-0.173115	0.08907322	-1.9435139	0.05195409	0.12334628
ENSG000001	146.268418	-0.2135514	0.11326224	-1.8854602	0.05936773	0.13695228
ENSG000001	1219.03311	0.16327567	0.07517235	2.17201771	0.02985433	0.0816191
ENSG000001	4078.48011	0.35327366	0.1367039	2.58422518	0.00975981	0.03418413
ENSG000001	517.601376	0.16943421	0.07061231	2.39949957	0.0164175	0.05156988
ENSG000001	2202.92665	0.17175422	0.06023883	2.85122119	0.00435517	0.01763861
ENSG000001	142.018495	0.48703018	0.21856087	2.22835029	0.02585717	0.07334273
ENSG000001	3416.83528	0.44424589	0.13163438	3.37484698	0.00073857	0.00421577
ENSG000001	4951.8394	-0.2629273	0.08491782	-3.0962557	0.00195981	0.00926625
ENSG000001	421.157229	-0.2340852	0.12567068	-1.8626873	0.06250628	0.14233393
ENSG000001	624.263034	-0.1595649	0.08470552	-1.8837598	0.05959747	0.13724187
ENSG000001	11021.2305	0.60294719	0.15508405	3.88787352	0.00010113	0.00083051
ENSG000001	2155.71779	-0.1833353	0.07330809	-2.5008883	0.01238822	0.04130453
ENSG000001	3279.04496	-0.1434539	0.0738938	-1.941352	0.05221559	0.12388584
ENSG000001	26200.0606	0.31091439	0.13442371	2.31294317	0.02072576	0.06197196
ENSG000001	25.942031	1.26980894	0.28800826	4.40893242	1.04E-05	0.00012863
ENSG000001	337.736168	0.14262291	0.05150953	2.76886436	0.0056252	0.02177193
ENSG000001	3327.72736	1.13354074	0.190415	5.95300132	2.63E-09	1.28E-07
ENSG000001	52905.4943	1.80906758	0.21516097	8.40797265	4.17E-17	1.47E-14
ENSG000001	3730.00642	0.22195601	0.09701882	2.28776233	0.02215137	0.06507946
ENSG000001	1520.50093	-0.1380522	0.06543797	-2.1096647	0.03488725	0.09226127
ENSG000001	553.925404	-0.1878085	0.07057458	-2.6611351	0.00778777	0.02841849
ENSG000001	220.428963	0.19823975	0.10029902	1.97648729	0.04809961	0.11660448
ENSG000001	3828.97114	0.21530092	0.08420233	2.55694725	0.01055952	0.03642281
ENSG000001	2560.68279	0.65357148	0.15247765	4.28634292	1.82E-05	0.00020367
ENSG000001	10275.8053	0.15879394	0.09338198	1.70047733	0.08904118	0.18477631
ENSG000001	1954.89481	-0.2213149	0.09567235	-2.3132591	0.0207084	0.0619584
ENSG000001	3901.92676	-0.3148063	0.08179042	-3.8489392	0.00011863	0.00095213

ENSG000001	441.031913	-0.3073407	0.11720983	-2.6221413	0.00873792	0.03127903
ENSG000001	2564.34045	-0.255981	0.05805153	-4.4095486	1.04E-05	0.00012837
ENSG000001	100.886431	-0.4738905	0.10190204	-4.6504515	3.31E-06	4.99E-05
ENSG000001	11404.5096	0.15677881	0.09281251	1.68919915	0.09118127	0.18820561
ENSG000001	1050.30376	0.41629413	0.12949184	3.214829	0.00130522	0.00669811
ENSG000001	487.586771	-0.1853104	0.07365292	-2.5159959	0.01186966	0.03994325
ENSG000001	8081.20257	2.18764809	0.39985825	5.47105899	4.47E-08	1.40E-06
ENSG000001	168.592207	-0.4867322	0.14608208	-3.3319091	0.00086252	0.00477105
ENSG000001	898.120926	-0.5597486	0.19637374	-2.850425	0.00436608	0.01767788
ENSG000001	28.6858288	0.5534248	0.27227176	2.03261916	0.04209101	0.10592373
ENSG000001	8130.80449	2.77383997	0.29752825	9.32294666	1.13E-20	6.83E-18
ENSG000001	102.858994	0.49110659	0.13917268	3.52875727	0.00041752	0.00264014
ENSG000001	3575.96144	0.33553636	0.09795828	3.42529874	0.00061412	0.00361636
ENSG000001	42.2435622	-0.4427699	0.19937589	-2.2207795	0.0263659	0.07434075
ENSG000001	3020.41705	0.24243041	0.08936154	2.71291656	0.00666939	0.02498096
ENSG000001	1697.56307	-0.1479222	0.08438146	-1.7530178	0.07959896	0.16960584
ENSG000001	1412.93098	-0.3142272	0.05968564	-5.2647029	1.40E-07	3.52E-06
ENSG000001	175.687401	-0.2697384	0.12733282	-2.1183731	0.03414348	0.09064259
ENSG000001	2242.17751	0.39877334	0.10467458	3.80964826	0.00013916	0.00107985
ENSG000001	434.721766	-0.171639	0.10193865	-1.6837479	0.09223041	0.1898738
ENSG000001	1002.35879	0.41508757	0.15029432	2.76183134	0.00574782	0.02215769
ENSG000001	9236.54336	0.45207706	0.14378086	3.14420883	0.00166537	0.00814877
ENSG000001	1611.01585	0.25420797	0.09863808	2.57717893	0.00996104	0.03476404
ENSG000001	204.03428	-0.176572	0.08996099	-1.9627623	0.0496738	0.11941461
ENSG000001	588.554456	-0.1686276	0.06485236	-2.6001773	0.00931756	0.03300584
ENSG000001	12691.1477	-0.3378899	0.19188536	-1.7608947	0.07825622	0.16760761
ENSG000001	5369.20373	0.39057622	0.12301439	3.17504501	0.00149813	0.00745823
ENSG000001	2506.15378	0.3121295	0.14168172	2.20303296	0.02759243	0.07688622
ENSG000001	3001.72117	0.17777984	0.08784036	2.02389702	0.04298075	0.10755953
ENSG000001	186.389534	-0.6456117	0.12254163	-5.2685097	1.38E-07	3.45E-06
ENSG000001	760.687615	-0.2615116	0.14043188	-1.8621956	0.06257553	0.14246922
ENSG000001	2547.26958	0.16495037	0.09262801	1.78078275	0.07494795	0.16251623
ENSG000001	700.986535	0.21582423	0.0976207	2.21084491	0.02704658	0.0757855
ENSG000001	2030.23265	0.15595677	0.06713074	2.32317982	0.02016949	0.06078452
ENSG000001	2047.29012	0.57549956	0.09892538	5.81751194	5.97E-09	2.58E-07
ENSG000001	506.387059	-0.154274	0.06723371	-2.2945925	0.02175649	0.06431059
ENSG000001	33562.7946	0.69435655	0.23096338	3.00634913	0.00264405	0.01176063
ENSG000001	232.526262	0.3358366	0.16598426	2.02330394	0.04304182	0.10760084
ENSG000001	480.089006	1.69867613	0.31346996	5.41894384	6.00E-08	1.75E-06
ENSG000001	1006.02958	-0.2766555	0.10343764	-2.6746118	0.00748158	0.02744636
ENSG000001	1975.31905	0.55745753	0.27528623	2.02501062	0.04286627	0.1074215
ENSG000001	111.435293	1.55487797	0.35443561	4.38691234	1.15E-05	0.00013951
ENSG000001	2141.26838	0.21495545	0.10032902	2.14250534	0.03215284	0.0864992

ENSG000001	34.6300975	0.49193611	0.22142482	2.2216846	0.02630463	0.07418243
ENSG000001	15667.8742	2.98181157	0.32916445	9.05872897	1.32E-19	7.08E-17
ENSG000001	284.718368	-0.1924445	0.09511864	-2.0232049	0.04305203	0.10760778
ENSG000001	27590.697	0.3327956	0.17425538	1.90981543	0.05615698	0.13104803
ENSG000001	238.9125	-0.4848326	0.11396902	-4.2540735	2.10E-05	0.000229
ENSG000001	38.4785679	1.21466808	0.30098659	4.03562192	5.45E-05	0.0005021
ENSG000001	1453.95654	0.17837798	0.08300186	2.14908409	0.03162773	0.085356
ENSG000001	451.217334	1.60289098	0.27814163	5.7628589	8.27E-09	3.42E-07
ENSG000001	3908.87796	-0.2650173	0.05121576	-5.1745264	2.28E-07	5.27E-06
ENSG000001	4702.7329	-0.1438453	0.07250228	-1.9840106	0.04725466	0.11513464
ENSG000001	699.525406	0.52106123	0.11919445	4.37152253	1.23E-05	0.00014846
ENSG000001	774.407173	-0.4100637	0.19958076	-2.0546255	0.03991519	0.102113
ENSG000001	3946.28491	0.37019512	0.1529345	2.4206123	0.01549439	0.04931179
ENSG000001	44.6065967	2.25907728	0.39710173	5.68891326	1.28E-08	4.86E-07
ENSG000001	2033.54278	-0.2604879	0.08377231	-3.1094751	0.0018742	0.008955
ENSG000001	140.914231	-0.6390418	0.2490834	-2.5655737	0.01030053	0.03569948
ENSG000001	24431.4906	0.16947715	0.09602044	1.76501119	0.07756189	0.16639733
ENSG000001	180.386374	-0.288379	0.05796616	-4.9749546	6.53E-07	1.26E-05
ENSG000001	28592.1587	-0.1750161	0.09698647	-1.8045419	0.07114639	0.1566172
ENSG000001	536.541689	-0.1608734	0.07014868	-2.2933205	0.02182956	0.06440902
ENSG000001	609.576118	0.62924714	0.37997169	1.6560369	0.09771435	0.19815058
ENSG000001	3285.81897	-0.1504791	0.08040597	-1.8714922	0.06127688	0.1403729
ENSG000001	948.545985	0.25923854	0.09832946	2.63642795	0.0083784	0.0301935
ENSG000001	131.810886	0.448171	0.19125033	2.34337376	0.01911023	0.05828418
ENSG000001	716.377667	0.27130338	0.0758976	3.57459767	0.00035077	0.00228663
ENSG000001	1998.61862	-0.3640732	0.11784802	-3.0893453	0.00200598	0.0094261
ENSG000001	160.110535	0.91711867	0.23211676	3.95110928	7.78E-05	0.00067426
ENSG000001	2495.08139	0.53822639	0.1635665	3.29056624	0.00099986	0.00541899
ENSG000001	3438.29092	-0.1774263	0.09315162	-1.9047044	0.0568185	0.13231468
ENSG000001	4504.29756	0.18881569	0.07880064	2.39611891	0.01656971	0.05194665
ENSG000001	24167.9268	-0.1724544	0.07116331	-2.4233611	0.01537763	0.04900478
ENSG000001	2138.8065	-0.3937124	0.08345926	-4.7174199	2.39E-06	3.84E-05
ENSG000001	845.768066	-0.1507314	0.09005748	-1.6737243	0.0941848	0.19269244
ENSG000001	10158.214	0.39165837	0.11921308	3.28536395	0.00101851	0.00549948
ENSG000001	1539.63053	-0.747613	0.1467889	-5.0931169	3.52E-07	7.51E-06
ENSG000001	1788.76074	0.19489049	0.07717832	2.52519753	0.01156333	0.03916716
ENSG000001	5757.79644	0.13589132	0.06594106	2.06079971	0.03932215	0.1009424
ENSG000001	159.041493	0.26849164	0.08676882	3.09433317	0.00197256	0.00931436
ENSG000001	245.496979	0.7011702	0.21336506	3.28624658	0.00101532	0.00548432
ENSG000001	410.292179	-0.2750028	0.13609174	-2.0207164	0.04330913	0.10809104
ENSG000001	2338.40509	-0.0758533	0.04429059	-1.7126281	0.08678098	0.18117693
ENSG000001	3825.7774	-0.3573935	0.10570667	-3.3809929	0.00072224	0.00413605
ENSG000001	3791.94441	0.10303117	0.05995778	1.71839516	0.08572456	0.17959389

ENSG000001	2445.53086	0.36646693	0.18201927	2.01334132	0.04407873	0.10940014
ENSG000001	2656.87851	0.32857531	0.13413974	2.44950023	0.01430546	0.04642545
ENSG000001	503.599374	0.2555518	0.11402859	2.24112052	0.02501827	0.07157181
ENSG000001	2555.11809	0.24480748	0.09390139	2.60706986	0.00913207	0.03247396
ENSG000001	4648.53631	0.55065945	0.16843324	3.26930392	0.00107812	0.00575278
ENSG000001	2227.66825	0.23285964	0.09433316	2.46848138	0.01356877	0.04444917
ENSG000001	16849.3101	0.21935407	0.1326294	1.65388725	0.09815042	0.19872889
ENSG000001	922.202115	-0.2828695	0.14842864	-1.9057608	0.05668125	0.13207998
ENSG000001	1206.01014	0.1922126	0.07304132	2.63155966	0.00849939	0.03056111
ENSG000001	2092.54391	0.23375447	0.10346841	2.25918698	0.02387176	0.0689729
ENSG000001	8965.89318	-0.2094961	0.11372096	-1.842194	0.06544678	0.14722888
ENSG000001	788.188101	0.25602294	0.09884269	2.59020603	0.00959185	0.03369475
ENSG000001	144.966885	-0.2632437	0.07178526	-3.6671005	0.00024532	0.00170451
ENSG000001	3926.0542	-0.139381	0.06806758	-2.0476855	0.04059083	0.10318297
ENSG000001	42.6535872	-0.377735	0.161726	-2.3356483	0.01950958	0.05920303
ENSG000001	1347.64976	0.23098153	0.10391489	2.22279536	0.0262296	0.07398525
ENSG000001	45808.8948	4.80843722	0.44149286	10.8913136	1.27E-27	2.30E-24
ENSG000001	10419.7181	3.6266161	0.38302492	9.46835543	2.84E-21	1.87E-18
ENSG000001	916.557142	0.45908538	0.08449027	5.43358888	5.52E-08	1.64E-06
ENSG000001	1334.33892	0.22869909	0.08606232	2.65736589	0.00787539	0.02867323
ENSG000001	253.835746	-0.3342756	0.1157801	-2.887159	0.00388738	0.01607651
ENSG000001	1172.58708	0.32675167	0.07836324	4.16970575	3.05E-05	0.00031202
ENSG000001	1237.99644	0.43572236	0.16814523	2.59134536	0.00956015	0.03362415
ENSG000001	3089.28237	-0.1348844	0.04232659	-3.1867533	0.00143879	0.00727213
ENSG000001	551.508613	-0.2419469	0.12914544	-1.8734451	0.06100694	0.13990938
ENSG000001	792.922989	1.2516322	0.37979713	3.29552833	0.00098237	0.00533217
ENSG000001	902.420243	0.4611376	0.11057997	4.17017307	3.04E-05	0.00031184
ENSG000001	1090.13628	0.26557339	0.05227206	5.08059955	3.76E-07	7.92E-06
ENSG000001	213.130609	-1.2322953	0.2363671	-5.2134805	1.85E-07	4.45E-06
ENSG000001	231.766596	0.75572546	0.25157034	3.00403248	0.00266427	0.01182877
ENSG000001	1133.52113	0.88796642	0.17590174	5.04808216	4.46E-07	9.21E-06
ENSG000001	1708.43851	0.30346432	0.07669079	3.95698505	7.59E-05	0.00066041
ENSG000001	1116.07766	0.16491974	0.09390365	1.75626544	0.07904309	0.16869436
ENSG000001	1212.3916	-0.1532838	0.09029035	-1.6976764	0.08956885	0.18558515
ENSG000001	783.218975	0.11731775	0.06181225	1.89796919	0.05770014	0.13383863
ENSG000001	285.51657	0.47780986	0.13648182	3.50090484	0.00046368	0.00288546
ENSG000001	3022.42604	-0.2100848	0.07422002	-2.8305677	0.00464655	0.01862089
ENSG000001	3100.73651	-0.1714587	0.08891071	-1.928437	0.0538008	0.12676416
ENSG000001	36635.3626	0.57831021	0.29598992	1.95381725	0.05072283	0.12117832
ENSG000001	216.624002	0.4120232	0.14772386	2.78914459	0.00528475	0.02071993
ENSG000001	1663.59877	0.15940866	0.07063962	2.25664656	0.02403017	0.06934762
ENSG000001	12605.0278	0.26568292	0.14742411	1.80216735	0.07151908	0.15716813
ENSG000001	207.818117	-0.4766899	0.19916577	-2.3934329	0.01669154	0.052272

ENSG000001	53.9969656	0.99692197	0.24310008	4.10087065	4.12E-05	0.00039858
ENSG000001	2540.37944	-0.2035772	0.07638599	-2.6651118	0.00769627	0.02815554
ENSG000001	1754.7726	0.19070658	0.09649221	1.97639351	0.04811023	0.11660914
ENSG000001	2365.98906	-0.1349812	0.08142036	-1.6578317	0.09735144	0.19758059
ENSG000001	2814.171	-0.3147388	0.13416499	-2.3459084	0.01898077	0.05799924
ENSG000001	1665.94413	1.90331982	0.25274371	7.53063178	5.05E-14	9.26E-12
ENSG000001	10841.9847	0.43899676	0.15602891	2.81356032	0.00489962	0.01950022
ENSG000001	1009.91697	-0.5953463	0.28860985	-2.0628065	0.03913101	0.10058392
ENSG000001	1809.41979	0.27146075	0.08987833	3.0203138	0.00252513	0.01133257
ENSG000001	1209.95773	1.40012946	0.25796666	5.42755973	5.71E-08	1.69E-06
ENSG000001	3006.55736	0.33504392	0.11301667	2.96455305	0.00303123	0.01314773
ENSG000001	8883.43436	0.26642751	0.12238982	2.17687641	0.02948979	0.08087342
ENSG000001	205.734488	-0.5855146	0.1535967	-3.8120262	0.00013783	0.00107123
ENSG000001	2607.45469	-0.232225	0.0826925	-2.808296	0.00498044	0.01978927
ENSG000001	22774.7183	-0.417736	0.10566133	-3.9535369	7.70E-05	0.00066846
ENSG000001	2478.41818	0.23027881	0.13537551	1.70103739	0.08893597	0.18463963
ENSG000001	11495.3647	0.28484658	0.13271532	2.14629763	0.03184924	0.08590578
ENSG000001	5251.42311	-0.2249801	0.04997155	-4.5021627	6.73E-06	9.01E-05
ENSG000001	16292.0947	0.68269515	0.139405	4.89720719	9.72E-07	1.76E-05
ENSG000001	816.998084	0.70697198	0.18501499	3.82116051	0.00013283	0.00104182
ENSG000001	831.615412	-0.2491027	0.11274035	-2.2095255	0.02713811	0.07595631
ENSG000001	244.472899	0.27420545	0.15514268	1.7674405	0.07715449	0.16583636
ENSG000001	1588.34939	0.32978861	0.10808646	3.05115572	0.00227962	0.01042782
ENSG000001	535.554707	-0.1269566	0.04896144	-2.5929925	0.00951448	0.03351089
ENSG000001	1079.49622	-0.3789951	0.07773621	-4.8753998	1.09E-06	1.93E-05
ENSG000001	19010.3199	2.8858282	0.31322443	9.21329208	3.16E-20	1.83E-17
ENSG000001	92.5110739	-0.640483	0.33294031	-1.9237173	0.05439003	0.12778921
ENSG000001	589.988986	-0.5514635	0.17305824	-3.1865777	0.00143967	0.00727213
ENSG000001	11581.6542	0.70481742	0.1642962	4.28991923	1.79E-05	0.00020104
ENSG000001	878.012518	0.26045069	0.11211307	2.32310722	0.02017339	0.06078452
ENSG000001	1010.48178	0.55757782	0.09002884	6.1933248	5.89E-10	3.52E-08
ENSG000001	1956.18181	0.16926273	0.10065301	1.68164599	0.09263751	0.19043256
ENSG000001	89.9140083	-0.4274718	0.1071759	-3.9885068	6.65E-05	0.00058986
ENSG000001	28.2667504	0.9719903	0.26114348	3.72205463	0.00019761	0.00142638
ENSG000001	5974.0498	-0.0826623	0.04868383	-1.6979417	0.08951877	0.18558364
ENSG000001	581.160674	0.33904079	0.0787983	4.30264086	1.69E-05	0.00019162
ENSG000001	3604.74117	-0.1396993	0.07305579	-1.9122271	0.05584707	0.13051404
ENSG000001	843.244415	0.29608921	0.0570747	5.18774932	2.13E-07	4.99E-06
ENSG000001	2122.36843	-0.2965802	0.15643393	-1.8958814	0.05797573	0.13434011
ENSG000001	5228.86917	0.60539632	0.12363866	4.89649695	9.76E-07	1.77E-05
ENSG000001	11366.178	0.30832564	0.13419413	2.29760906	0.02158405	0.06391826
ENSG000001	1988.73239	-0.3585479	0.08853588	-4.049747	5.13E-05	0.0004783
ENSG000001	3354.64321	0.48869897	0.20566784	2.37615649	0.01749404	0.05421088

ENSG000001	5871.89935	-0.2255453	0.0556408	-4.0535966	5.04E-05	0.00047218
ENSG000001	904.591041	0.17433442	0.04636992	3.75964453	0.00017015	0.00127064
ENSG000001	13331.3487	0.24033556	0.13542121	1.77472614	0.07594312	0.16403577
ENSG000001	623.312181	-0.2062531	0.0871007	-2.3679845	0.01788528	0.05518724
ENSG000001	148.021904	1.02993598	0.20112572	5.12085659	3.04E-07	6.64E-06
ENSG000001	184.534286	0.64573359	0.12231741	5.27916359	1.30E-07	3.33E-06
ENSG000001	961.003654	0.68689664	0.10837546	6.33811991	2.33E-10	1.68E-08
ENSG000001	977.324607	-0.1984787	0.09633282	-2.0603435	0.03936572	0.10097223
ENSG000001	85.5414123	1.2492225	0.2491265	5.01441036	5.32E-07	1.07E-05
ENSG000001	1120.59361	0.13520824	0.04819487	2.80544879	0.00502466	0.019917
ENSG000001	2146.24818	-0.3035578	0.06032832	-5.0317634	4.86E-07	9.89E-06
ENSG000001	469.422415	-0.2423328	0.10110848	-2.3967605	0.01654073	0.05186701
ENSG000001	132.579776	1.07317553	0.2169941	4.94564391	7.59E-07	1.43E-05
ENSG000001	5204.17506	-0.1241043	0.06603109	-1.8794822	0.06017869	0.13831646
ENSG000001	17604.5375	0.23482106	0.11900979	1.97312393	0.04848144	0.11729303
ENSG000001	235.70655	-0.7748681	0.19599473	-3.9535151	7.70E-05	0.00066846
ENSG000001	19475.2032	0.40271608	0.11091458	3.63086684	0.00028247	0.0019185
ENSG000001	642.460684	-0.1426224	0.07058678	-2.0205261	0.04332884	0.10811314
ENSG000001	17060.2788	0.32036303	0.11244672	2.84902061	0.0043854	0.01773133
ENSG000001	1618.88184	-0.3081435	0.06589743	-4.6761082	2.92E-06	4.51E-05
ENSG000001	197.611044	-0.3691946	0.09626136	-3.8353347	0.00012539	0.00099429
ENSG000001	5921.27825	-0.1617472	0.08218864	-1.9679997	0.04906808	0.1182546
ENSG000001	1017.91371	-0.2763527	0.1090675	-2.5337768	0.01128406	0.03839177
ENSG000001	31256.0058	0.59533874	0.22113492	2.6921969	0.0070983	0.02632696
ENSG000001	73.8349979	-0.4007708	0.17011606	-2.3558672	0.01847952	0.05680307
ENSG000001	22552.0487	0.57351899	0.15218084	3.76866758	0.00016412	0.00123129
ENSG000001	12779.8222	0.2451847	0.13407527	1.82870933	0.06744316	0.15031525
ENSG000001	4311.4073	0.36672819	0.16129211	2.2736896	0.02298466	0.06686319
ENSG000001	356.329085	0.46711063	0.10693014	4.36837191	1.25E-05	0.00014987
ENSG000001	335.184356	-0.3891781	0.11729037	-3.3180736	0.00090641	0.00498902
ENSG000001	378.687208	-0.2222988	0.08730727	-2.5461662	0.01089133	0.03735715
ENSG000001	1027.66141	-0.2435892	0.06982485	-3.4885746	0.0004856	0.00299359
ENSG000001	4786.06196	0.42283423	0.13484324	3.13574663	0.00171417	0.00833329
ENSG000001	6779.75421	-0.1447664	0.06393443	-2.2642949	0.02355598	0.06822382
ENSG000001	189.233711	0.53814401	0.13793835	3.90133716	9.57E-05	0.00079602
ENSG000001	3409.98694	0.1760448	0.10087521	1.74517399	0.08095459	0.17203889
ENSG000001	9665.34907	0.292944	0.16885246	1.73491101	0.08275657	0.17515886
ENSG000001	14972.4311	0.91605836	0.15186158	6.03219282	1.62E-09	8.31E-08
ENSG000001	1813.62609	0.30852683	0.08548739	3.60903335	0.00030734	0.00205371
ENSG000001	105.700678	-0.5887505	0.16448816	-3.5792881	0.00034453	0.00225439
ENSG000001	4318.22798	0.33263641	0.108457	3.06698895	0.00216227	0.01001112
ENSG000001	1335.92544	-0.1748711	0.07081741	-2.4693234	0.01353688	0.0443685
ENSG000001	1101.90558	0.36846511	0.15978024	2.30607441	0.02110647	0.06290259

ENSG000001	191.66272	-0.9582411	0.21377372	-4.4825017	7.38E-06	9.69E-05
ENSG000001	1504.9673	-0.198029	0.05790299	-3.4200129	0.00062618	0.00367416
ENSG000001	87.233974	0.42940086	0.20457534	2.09898642	0.0358181	0.09410533
ENSG000001	111.122906	-0.3321262	0.19673774	-1.6881671	0.09137916	0.18849635
ENSG000001	25460.3033	0.42874816	0.1895481	2.2619491	0.02370055	0.06857397
ENSG000001	1398.93477	0.44319831	0.12367542	3.58356022	0.00033894	0.00222789
ENSG000001	6389.69433	0.36485479	0.14341461	2.5440559	0.01095735	0.03749154
ENSG000001	1554.78227	-0.2887737	0.11617751	-2.4856245	0.01293243	0.04273544
ENSG000001	2442.22426	0.16500298	0.08189582	2.01479119	0.04392653	0.10911586
ENSG000001	2542.60207	-0.3264672	0.1751112	-1.8643424	0.06227363	0.1419827
ENSG000001	239.605812	0.2126967	0.10634437	2.00007485	0.04549218	0.11204441
ENSG000001	778.676576	-0.2265777	0.06853166	-3.3061759	0.00094579	0.00516652
ENSG000001	6754.08107	0.97711645	0.14539552	6.72040291	1.81E-11	1.70E-09
ENSG000001	394.972308	-0.6342661	0.12035005	-5.2701772	1.36E-07	3.43E-06
ENSG000001	85.505475	-0.591112	0.13524258	-4.3707536	1.24E-05	0.00014874
ENSG000001	1303.70964	-0.5793095	0.1108094	-5.2279819	1.71E-07	4.17E-06
ENSG000001	2583.49892	0.29633748	0.13681579	2.16595966	0.03031427	0.08268975
ENSG000001	47.0915129	0.97384248	0.19918992	4.88901496	1.01E-06	1.82E-05
ENSG000001	2618.64067	-0.218483	0.05178991	-4.21864	2.46E-05	0.00026258
ENSG000001	11015.5716	0.23573924	0.12389787	1.90268997	0.05708101	0.13277659
ENSG000001	1013.09287	-0.1823152	0.08370179	-2.1781515	0.02939476	0.08074524
ENSG000001	24028.5116	0.18062106	0.09055517	1.99459695	0.04608686	0.11300953
ENSG000001	14129.533	0.17787522	0.09951409	1.78743753	0.0738668	0.16089435
ENSG000001	49.6087219	0.85948883	0.22671172	3.79110891	0.00014998	0.00114473
ENSG000001	315.894322	0.48198332	0.14919533	3.23055242	0.00123551	0.00642227
ENSG000001	169.412513	0.6600089	0.20245179	3.26007935	0.00111381	0.00589758
ENSG000001	2009.29248	-0.1504595	0.08061672	-1.8663559	0.06199159	0.14154984
ENSG000001	6126.41272	-0.2532344	0.05797421	-4.3680529	1.25E-05	0.00014997
ENSG000001	80.7295479	0.53530784	0.16587513	3.22717354	0.0012502	0.00647305
ENSG000001	2518.76958	-0.247777	0.06529158	-3.7949308	0.00014768	0.00113082
ENSG000001	2767.42219	2.55953172	0.30187428	8.47880013	2.28E-17	8.60E-15
ENSG000001	1680.17258	0.14781202	0.07367711	2.00621355	0.04483347	0.11079878
ENSG000001	474.56013	1.04529383	0.16506403	6.33265652	2.41E-10	1.72E-08
ENSG000001	4423.50775	0.42221301	0.11104065	3.80232823	0.00014334	0.00110516
ENSG000001	1182.5508	0.35956461	0.14647481	2.45478809	0.01409677	0.0458921
ENSG000001	28.8430569	1.33681624	0.25281892	5.28764314	1.24E-07	3.21E-06
ENSG000001	874.130764	0.0967015	0.05526593	1.74974877	0.08016167	0.17062918
ENSG000001	272.397745	0.57325344	0.12812505	4.47417133	7.67E-06	0.00010002
ENSG000001	15810.0691	1.18632229	0.2029683	5.84486473	5.07E-09	2.26E-07
ENSG000001	1563.32449	-0.3089944	0.08238572	-3.7505816	0.00017642	0.00130735
ENSG000001	4289.34826	-0.1787929	0.04957692	-3.6063742	0.00031051	0.00207008
ENSG000001	1778.98297	-0.4024289	0.10060326	-4.000158	6.33E-05	0.00056651
ENSG000001	2007.96434	-0.1799257	0.08043538	-2.2368978	0.02529302	0.07208734

ENSG000001	497.961254	-0.2622179	0.06563612	-3.9950241	6.47E-05	0.00057705
ENSG000001	894.088454	-0.1553644	0.05351112	-2.903404	0.0036913	0.01541536
ENSG000001	7332.73954	0.16443958	0.08376357	1.96313967	0.04962995	0.11937391
ENSG000001	7082.84929	0.11503788	0.06046882	1.902433	0.05711457	0.13283332
ENSG000001	928.344436	0.28209429	0.09796575	2.87951947	0.00398282	0.01638247
ENSG000001	723.123517	0.33939259	0.11607882	2.92381145	0.00345774	0.01462549
ENSG000001	1935.10428	-0.108422	0.05461844	-1.9850805	0.04713551	0.11495828
ENSG000001	3499.32615	-0.1610302	0.06358821	-2.5323901	0.01132879	0.0384807
ENSG000001	2860.91432	1.06139588	0.17614308	6.02575962	1.68E-09	8.56E-08
ENSG000001	1729.27706	-0.2834895	0.06849595	-4.1387782	3.49E-05	0.00034813
ENSG000001	3248.01026	0.14034041	0.06038597	2.32405649	0.02012247	0.06068155
ENSG000001	5809.37204	1.32597188	0.21037007	6.30304426	2.92E-10	2.01E-08
ENSG000001	1607.0809	-0.2704253	0.12527536	-2.1586471	0.03087756	0.08376838
ENSG000001	4078.57113	0.55748638	0.10826615	5.14922139	2.62E-07	5.88E-06
ENSG000001	56.8629784	-0.6069225	0.23615153	-2.5700555	0.01016822	0.03534238
ENSG000001	158.835403	-0.4644292	0.14884134	-3.1202973	0.00180669	0.00871287
ENSG000001	500.876582	0.34097629	0.19676209	1.73293693	0.08310688	0.17565937
ENSG000001	19946.8205	0.5623478	0.1975241	2.8469832	0.00441357	0.01782779
ENSG000001	2068.01098	0.32421131	0.0833599	3.88929567	0.00010054	0.00082706
ENSG000001	1323.95959	-0.2740608	0.05763575	-4.7550481	1.98E-06	3.26E-05
ENSG000001	5306.04794	-0.139243	0.06712569	-2.0743626	0.03804565	0.09856355
ENSG000001	4228.27282	0.57308206	0.0937088	6.11556295	9.62E-10	5.32E-08
ENSG000001	1037.83537	-0.1488889	0.07261662	-2.0503423	0.04033104	0.10279306
ENSG000001	589.141841	0.22753931	0.12697761	1.79196409	0.07313872	0.15977572
ENSG000001	3346.98347	0.25209343	0.06623218	3.80620752	0.00014111	0.00109263
ENSG000001	2188.64042	0.15479533	0.05109408	3.02961393	0.00244867	0.01103385
ENSG000001	4637.66028	-0.3796253	0.0706024	-5.3769463	7.58E-08	2.13E-06
ENSG000001	4626.8592	0.33484031	0.15046404	2.22538433	0.02605545	0.07370455
ENSG000001	99.2773012	-0.5001851	0.16991725	-2.9436984	0.00324316	0.01388405
ENSG000001	40.8570159	0.96158448	0.24335933	3.95129488	7.77E-05	0.00067426
ENSG000001	3216.5452	-0.2232205	0.04665987	-4.7839931	1.72E-06	2.86E-05
ENSG000001	3173.89027	-0.3929127	0.11545191	-3.4032583	0.00066587	0.00385398
ENSG000001	620.038322	-0.1766067	0.08662509	-2.0387483	0.04147516	0.10471429
ENSG000001	1016.43335	-0.2589284	0.07129101	-3.6319921	0.00028124	0.00191194
ENSG000001	245.596934	-0.5579913	0.22967963	-2.4294329	0.01512246	0.04845811
ENSG000001	488.132885	-0.4539467	0.10763109	-4.2176167	2.47E-05	0.00026339
ENSG000001	29386.5337	0.34711561	0.16962017	2.04642883	0.0407142	0.10336954
ENSG000001	786.457442	-0.5303066	0.10258703	-5.1693335	2.35E-07	5.40E-06
ENSG000001	3986.23676	0.33560714	0.1730313	1.93957477	0.05243139	0.12425545
ENSG000001	8039.90106	0.98370218	0.15182502	6.47918375	9.22E-11	7.46E-09
ENSG000001	2154.78142	-0.1207121	0.05685774	-2.1230541	0.03374932	0.08984315
ENSG000001	669.4637	0.61588119	0.21849878	2.81869396	0.00482195	0.01923335
ENSG000001	444.259054	-0.3089824	0.09756087	-3.1670727	0.00153982	0.00764736

ENSG000001	69.3007074	-0.2888514	0.11399617	-2.5338689	0.01128109	0.03839069
ENSG000001	4266.09224	0.13702238	0.07922851	1.7294581	0.08372714	0.17648117
ENSG000001	3344.24019	-0.2351655	0.10695438	-2.1987457	0.02789601	0.07759783
ENSG000001	5475.40087	0.3395253	0.16377656	2.07310069	0.03816291	0.09873211
ENSG000001	140.398838	-0.1715062	0.09390359	-1.8264076	0.06778888	0.15087687
ENSG000001	832.328538	0.31521459	0.09290561	3.39284783	0.0006917	0.00398278
ENSG000001	4727.05864	0.46238305	0.2059988	2.24459103	0.0247944	0.07108578
ENSG000001	2570.58497	0.21870384	0.10115595	2.16204627	0.03061461	0.08330463
ENSG000001	14145.4916	0.99556236	0.17453384	5.70412234	1.17E-08	4.59E-07
ENSG000001	1819.50981	0.66970343	0.19055351	3.51451644	0.00044056	0.00275694
ENSG000001	402.669702	0.35856064	0.194869	1.84000865	0.06576697	0.14766988
ENSG000001	37233.0042	0.81465436	0.23067096	3.5316729	0.00041294	0.00261691
ENSG000001	238.512793	0.30826301	0.15907175	1.93788657	0.05263706	0.12462055
ENSG000001	360.711526	-0.2814775	0.07233305	-3.8914088	9.97E-05	0.00082292
ENSG000001	1151.73446	-0.1454417	0.08672355	-1.6770725	0.09352831	0.1917284
ENSG000001	9080.49685	-0.2280377	0.06348481	-3.5920035	0.00032815	0.0021707
ENSG000001	830.03409	0.23860236	0.079281	3.009578	0.00261611	0.011665
ENSG000001	923.006697	-0.5131835	0.14724254	-3.4852939	0.0004916	0.00302539
ENSG000001	65.1687689	0.28247931	0.16121116	1.75223179	0.07973396	0.16984354
ENSG000001	659.179332	0.40054684	0.10783802	3.71433782	0.00020374	0.00146043
ENSG000001	166.882009	0.86985049	0.19463253	4.46919376	7.85E-06	0.0001021
ENSG000001	1336.55626	-0.1097874	0.06333291	-1.7334973	0.08300731	0.17550014
ENSG000001	9235.07932	2.64337125	0.2966585	8.91048535	5.08E-19	2.54E-16
ENSG000001	24.9789655	-1.3494851	0.28139098	-4.7957653	1.62E-06	2.73E-05
ENSG000001	388.141763	0.40533472	0.11345013	3.5728007	0.00035318	0.00230062
ENSG000001	197.557884	0.24175712	0.11732302	2.06061114	0.03934015	0.1009424
ENSG000001	344.893946	0.3919059	0.14275257	2.74535095	0.00604462	0.02302615
ENSG000001	20622.2598	0.8925385	0.12722269	7.01556075	2.29E-12	2.74E-10
ENSG000001	1014.25494	1.11569584	0.24596693	4.53595879	5.73E-06	7.94E-05
ENSG000001	725.448266	-0.414719	0.07493962	-5.5340412	3.13E-08	1.05E-06
ENSG000001	4817.61381	0.57866602	0.15155762	3.81812543	0.00013447	0.00105187
ENSG000001	715.340927	0.63149004	0.29871681	2.11400905	0.03451449	0.09149341
ENSG000001	835.839033	0.41840683	0.0768479	5.44460946	5.19E-08	1.57E-06
ENSG000001	132.68664	0.50834114	0.15737459	3.23013471	0.00123732	0.00642935
ENSG000001	119.415026	0.91447844	0.21325123	4.288268	1.80E-05	0.00020238
ENSG000001	3161.98299	0.11988668	0.0383765	3.12396059	0.00178434	0.00863098
ENSG000001	23379.7219	-0.3577467	0.12112142	-2.9536208	0.0031407	0.01356568
ENSG000001	539.572086	-0.2819601	0.08876105	-3.17662	0.00149002	0.00744373
ENSG000001	1133.70227	-0.1501671	0.08187865	-1.8340202	0.066651	0.14905419
ENSG000001	115.930572	0.55100887	0.1855843	2.96904892	0.00298723	0.0129841
ENSG000001	30482.0331	0.1890259	0.10971153	1.72293552	0.08490019	0.17822765
ENSG000001	857.745941	0.56481478	0.10310457	5.4780771	4.30E-08	1.36E-06
ENSG000001	61.4907386	-0.3611773	0.18531369	-1.9490051	0.05129482	0.12220161

ENSG000001	790.727679	0.62682046	0.23022743	2.72261415	0.00647677	0.02439015
ENSG000001	277.985709	0.56042903	0.18161594	3.08579203	0.00203011	0.00949719
ENSG000001	90.450031	-0.3263088	0.12558737	-2.5982609	0.00936973	0.03315566
ENSG000001	1253.49834	0.10411067	0.04857785	2.1431718	0.03209931	0.0864161
ENSG000001	2389.01805	0.61829682	0.0910861	6.78804765	1.14E-11	1.16E-09
ENSG000001	38435.2755	5.14030727	0.43070468	11.9346445	7.81E-33	3.77E-29
ENSG000001	69.8368746	1.04313025	0.27844276	3.74630047	0.00017946	0.00132368
ENSG000001	21024.0209	4.42154206	0.38959996	11.3489286	7.51E-30	1.81E-26
ENSG000001	1243.31587	0.26940032	0.05072452	5.31104763	1.09E-07	2.89E-06
ENSG000001	436.61167	-0.2526884	0.08700568	-2.9042751	0.00368105	0.01538574
ENSG000001	775.664163	0.29222037	0.1045363	2.79539601	0.00518362	0.02039518
ENSG000001	142.479193	-0.2037201	0.11535167	-1.7660789	0.07738262	0.16617877
ENSG000001	1464.24718	1.34002398	0.20709063	6.47071266	9.75E-11	7.85E-09
ENSG000001	71.6438746	0.26579454	0.15767934	1.68566497	0.09186035	0.18927334
ENSG000001	1036.46663	-0.1819693	0.08671881	-2.0983837	0.03587127	0.09415964
ENSG000001	2701.89001	-0.1483827	0.08415414	-1.7632257	0.07786243	0.16691226
ENSG000001	778.387358	0.42315093	0.11121747	3.80471644	0.00014197	0.00109572
ENSG000001	242.557216	0.83424483	0.18908511	4.41200695	1.02E-05	0.00012735
ENSG000001	984.771707	0.30119768	0.15682706	1.92057219	0.05478566	0.12859364
ENSG000001	2986.48172	-0.2221283	0.11179791	-1.9868732	0.04693646	0.11466066
ENSG000001	100.865252	1.39313553	0.2604001	5.34998078	8.80E-08	2.40E-06
ENSG000001	7134.69015	0.45269649	0.15961777	2.83612842	0.00456641	0.01835223
ENSG000001	1867.95728	0.33229232	0.14781488	2.24803027	0.02457426	0.07063637
ENSG000001	4.29559768	1.18997886	0.46769682	2.54433815	0.0109485	0.03747848
ENSG000001	830.771564	-0.1256757	0.05083299	-2.4723252	0.01342374	0.04408743
ENSG000001	3185.71247	0.35312323	0.11561935	3.05418805	0.00225671	0.01033931
ENSG000001	5291.42729	-0.3190732	0.07261177	-4.3942356	1.11E-05	0.00013533
ENSG000001	924.159979	0.1833081	0.06227769	2.94339918	0.0032463	0.01389338
ENSG000001	34.4497477	0.95288643	0.28181881	3.38120243	0.00072169	0.00413575
ENSG000001	5170.9657	0.26005761	0.14712275	1.76762343	0.07712388	0.16581006
ENSG000001	204.37696	0.22921488	0.12161222	1.88480131	0.05945667	0.13711378
ENSG000001	1958.77867	-0.2332235	0.10719123	-2.1757704	0.02957243	0.08102319
ENSG000001	1149.93544	-0.1743607	0.08144153	-2.1409308	0.03227962	0.08669537
ENSG000001	285.75463	0.20652209	0.1014035	2.03663674	0.04168646	0.10512674
ENSG000001	683.139857	0.22802499	0.11542543	1.97551785	0.04820941	0.11681046
ENSG000001	345.703902	0.30338987	0.10868345	2.79150026	0.00524643	0.02059758
ENSG000001	1880.26618	0.53321756	0.11333224	4.70490636	2.54E-06	4.01E-05
ENSG000001	17.8494528	-1.3491568	0.34017526	-3.9660639	7.31E-05	0.00064116
ENSG000001	2262.46707	0.35202965	0.11758452	2.99384354	0.00275487	0.0121407
ENSG000001	673.315982	0.66191706	0.25803464	2.56522557	0.01031087	0.03572677
ENSG000001	2295.15119	0.25481904	0.08899541	2.86328286	0.00419276	0.01709075
ENSG000001	955.249578	0.1501053	0.08134502	1.84529187	0.06499509	0.14659566
ENSG000001	7275.92327	0.49092938	0.13292503	3.69328009	0.00022138	0.00156598

ENSG000001	5337.20746	0.32057821	0.1297723	2.47031307	0.01349949	0.04427599
ENSG000001	1335.39715	-0.4250135	0.10414244	-4.081079	4.48E-05	0.00042781
ENSG000001	738.737946	-0.2985704	0.10566628	-2.8255974	0.00471925	0.01887571
ENSG000001	7152.70658	0.31196837	0.18748753	1.66394196	0.09612403	0.19563765
ENSG000001	2265.41043	0.23319952	0.13244531	1.76072317	0.07828527	0.16764504
ENSG000001	394.976204	0.49596792	0.09649017	5.14008777	2.75E-07	6.11E-06
ENSG000001	25565.135	1.48584601	0.20077161	7.40067768	1.35E-13	2.31E-11
ENSG000001	3112.04252	0.1650029	0.07715629	2.13855405	0.0324718	0.08711463
ENSG000001	3337.18144	0.26347581	0.0760351	3.46518643	0.00052986	0.00320577
ENSG000001	3266.48746	0.42038436	0.12089313	3.47732225	0.00050645	0.00309229
ENSG000001	152.846168	-0.7973274	0.12920106	-6.1712136	6.78E-10	3.99E-08
ENSG000001	1474.01516	0.36829538	0.09333326	3.9460249	7.95E-05	0.00068397
ENSG000001	94.2691479	0.36996203	0.13818327	2.67732861	0.00742118	0.02728004
ENSG000001	4360.25148	2.16985996	0.25772488	8.41928783	3.79E-17	1.37E-14
ENSG000001	16741.4458	3.31169795	0.31757085	10.4282178	1.84E-25	2.23E-22
ENSG000001	1129.12469	0.28216982	0.08813007	3.20174291	0.00136599	0.00694597
ENSG000001	9914.93299	-0.3973104	0.04920455	-8.0746684	6.77E-16	1.72E-13
ENSG000001	483.477644	0.33604482	0.13086199	2.5679329	0.0102307	0.03551692
ENSG000001	4154.02901	-0.3327263	0.09159761	-3.6324779	0.00028071	0.00191036
ENSG000001	318.217531	0.22469724	0.09842195	2.28299927	0.02243041	0.06561982
ENSG000001	6884.60082	0.72962212	0.14679615	4.97030824	6.68E-07	1.29E-05
ENSG000001	275.681596	-0.184761	0.09943319	-1.8581416	0.06314891	0.14348131
ENSG000001	348.697377	0.28590797	0.13106111	2.18148605	0.02914749	0.08027749
ENSG000001	414.644547	0.61175116	0.27928221	2.19044079	0.02849228	0.07887784
ENSG000001	4131.64649	-0.5164218	0.12597774	-4.0993099	4.14E-05	0.00040075
ENSG000001	5317.64754	-0.1289919	0.06510516	-1.9812851	0.04755931	0.11569971
ENSG000001	1084.7929	-0.2990069	0.09282371	-3.2212345	0.0012764	0.00658712
ENSG000001	2997.7042	0.52874065	0.12780865	4.13697082	3.52E-05	0.00034992
ENSG000001	3764.43986	-0.1545771	0.06746142	-2.2913409	0.0219437	0.0646135
ENSG000001	5105.2232	1.2967406	0.24901604	5.20745809	1.91E-07	4.55E-06
ENSG000001	1129.4775	0.22626173	0.07957909	2.84323109	0.00446587	0.01799846
ENSG000001	102.908028	0.93538314	0.22087567	4.23488541	2.29E-05	0.00024685
ENSG000001	578.288342	0.21790604	0.05753601	3.78729852	0.00015229	0.00116059
ENSG000001	6604.9031	-0.4486965	0.15570682	-2.8816752	0.00395567	0.01628086
ENSG000001	1011.64115	-0.2012818	0.07947844	-2.5325327	0.01132418	0.0384807
ENSG000001	2372.52701	-0.1746341	0.07910876	-2.2075191	0.02727782	0.07624421
ENSG000001	5587.62034	0.270676	0.1063253	2.5457346	0.01090481	0.03737353
ENSG000001	2116.86297	0.22921627	0.09869633	2.3224396	0.02020928	0.06086732
ENSG000001	499.056334	-0.1417592	0.07944111	-1.7844567	0.07434949	0.16158131
ENSG000001	7192.30558	0.37223004	0.1090311	3.41398054	0.00064021	0.0037353
ENSG000001	4726.66329	0.28010454	0.16121272	1.73748411	0.08230176	0.17428819
ENSG000001	2190.11034	0.70181435	0.21240023	3.30420706	0.00095245	0.00519315
ENSG000001	70.1806458	0.28172138	0.12350112	2.2811242	0.0225411	0.06587712

ENSG000001	826.267874	-0.2585373	0.08674521	-2.9804212	0.00287852	0.01259473
ENSG000001	194.285581	0.52329655	0.1188912	4.40147418	1.08E-05	0.00013211
ENSG000001	707.854709	0.3240287	0.08222086	3.9409549	8.12E-05	0.00069611
ENSG000001	92.7736738	-0.2692917	0.16098183	-1.6728079	0.09436513	0.19287071
ENSG000001	327.553755	0.43038796	0.16940733	2.54055098	0.0110678	0.0377891
ENSG000001	503.338353	0.19844377	0.08165429	2.43029204	0.01508666	0.0483862
ENSG000001	713.818151	0.27290453	0.11630323	2.34649141	0.0189511	0.05793303
ENSG000001	4372.84279	0.23404388	0.0821184	2.85007852	0.00437084	0.01768727
ENSG000001	1958.49008	-0.4032128	0.14548703	-2.771469	0.0055804	0.02162795
ENSG000001	188.915897	-0.2596488	0.06979505	-3.720161	0.0001991	0.00143426
ENSG000001	9429.03359	0.19298658	0.09940414	1.94143413	0.05220564	0.12388584
ENSG000001	848.794292	0.18286326	0.06532871	2.7991257	0.00512412	0.02019948
ENSG000001	1383.60457	-0.3592579	0.11154315	-3.2207972	0.00127835	0.00658733
ENSG000001	9869.24608	-0.2447585	0.10931707	-2.2389776	0.02515738	0.07187042
ENSG000001	125.999798	0.30258247	0.16881873	1.79235134	0.0730767	0.15977572
ENSG000001	3517.79925	0.25943086	0.12511641	2.07351579	0.0381243	0.09869987
ENSG000001	3695.27259	0.31998869	0.13432979	2.3821127	0.01721363	0.05353668
ENSG000001	402.500723	-0.1807117	0.09266381	-1.9501861	0.05115394	0.12192614
ENSG000001	280.222648	0.28556255	0.10354372	2.7578936	0.00581751	0.02238468
ENSG000001	88.06783	-0.4709495	0.14090736	-3.3422632	0.00083098	0.00464267
ENSG000001	146.342748	0.82548968	0.18189279	4.53833095	5.67E-06	7.87E-05
ENSG000001	271.229939	0.895457	0.21974218	4.07503462	4.60E-05	0.00043614
ENSG000001	657.842147	0.16601114	0.0940547	1.76504896	0.07755554	0.16639733
ENSG000001	5176.51462	-0.2006466	0.06628774	-3.0269029	0.00247073	0.011116
ENSG000001	284.750241	1.27036248	0.18889597	6.72519648	1.75E-11	1.67E-09
ENSG000001	1030.12578	-0.3217832	0.08503873	-3.7839606	0.00015435	0.00117073
ENSG000001	4283.0792	0.47272664	0.1305777	3.6202708	0.00029429	0.001983
ENSG000001	4452.87325	0.11117429	0.05172105	2.14949795	0.03159495	0.0852993
ENSG000001	1303.27952	-0.213843	0.09635397	-2.2193478	0.02646308	0.07449875
ENSG000001	8831.84997	-0.6028326	0.11289652	-5.3396917	9.31E-08	2.53E-06
ENSG000001	47684.3901	-0.1798625	0.08624509	-2.0854815	0.03702561	0.09648749
ENSG000001	3498.70848	-0.0855443	0.04213258	-2.0303606	0.0423199	0.10634665
ENSG000001	2694.67877	0.27579777	0.0932338	2.95813057	0.00309511	0.01338473
ENSG000001	96.6753497	-0.3354662	0.11339086	-2.9584941	0.00309146	0.01337295
ENSG000001	2617.05599	0.55132214	0.19389319	2.84343226	0.00446305	0.01799505
ENSG000001	61.3724285	-0.2453873	0.13507176	-1.8167179	0.06926032	0.15337424
ENSG000001	1155.53006	-0.0966734	0.04670757	-2.0697581	0.03847501	0.09928525
ENSG000001	1824.59097	0.09188914	0.05001745	1.83714144	0.06618902	0.14833961
ENSG000001	489.129164	-0.3485578	0.09494703	-3.6710763	0.00024153	0.00168143
ENSG000001	3341.97192	-0.1469211	0.06860397	-2.1415833	0.03222703	0.0866023
ENSG000001	693.297067	-0.3679541	0.1243571	-2.958851	0.00308788	0.01336146
ENSG000001	1498.1965	0.31681983	0.12543419	2.52578531	0.011544	0.03911998
ENSG000001	972.36548	0.28243111	0.16684184	1.6928075	0.09049211	0.18711951

ENSG000001	3260.64093	-0.2217571	0.05594219	-3.9640405	7.37E-05	0.00064428
ENSG000001	72.490958	-0.4231149	0.14337725	-2.95106	0.00316685	0.01364611
ENSG000001	2966.66681	0.86485979	0.18541978	4.66433406	3.10E-06	4.73E-05
ENSG000001	1991.91231	0.44222747	0.12392211	3.56859202	0.0003589	0.00233264
ENSG000001	3555.31325	0.36643377	0.14434619	2.53857594	0.01113046	0.03795834
ENSG000001	1073.67223	-0.6497134	0.18489006	-3.5140529	0.00044132	0.00276057
ENSG000001	775.508012	-0.1941455	0.0930541	-2.0863728	0.03694487	0.09633195
ENSG000001	435.494734	-0.2453998	0.11515704	-2.1310016	0.03308901	0.08841027
ENSG000001	2901.52095	0.30808135	0.09815786	3.13863142	0.00169739	0.00827391
ENSG000001	3924.58921	0.68143236	0.20755566	3.28313066	0.00102661	0.00553087
ENSG000001	4523.84199	0.4129893	0.15505	2.66358785	0.00773122	0.02825485
ENSG000001	106.07939	0.16521107	0.09718348	1.69999128	0.08913257	0.18491529
ENSG000001	14997.3774	0.24601816	0.14183039	1.73459419	0.08281271	0.17518585
ENSG000001	1476.56162	-0.2690013	0.08768905	-3.0676725	0.00215733	0.00999144
ENSG000001	328.135606	-0.3082755	0.11159673	-2.7624063	0.0057377	0.02213066
ENSG000001	2909.91078	0.44543568	0.12577238	3.54160178	0.00039771	0.00253911
ENSG000001	808.429058	-1.2190094	0.28306964	-4.3063939	1.66E-05	0.00018899
ENSG000001	1481.15556	0.1596822	0.07389604	2.16090324	0.03070281	0.08344217
ENSG000001	4959.26703	0.58499584	0.09629179	6.07524099	1.24E-09	6.55E-08
ENSG000001	1762.90722	-0.3889835	0.0666876	-5.8329221	5.45E-09	2.41E-07
ENSG000001	75.8380296	0.21167496	0.12614597	1.67801597	0.09334398	0.19151314
ENSG000001	1163.49597	0.17567201	0.10603469	1.6567409	0.09757187	0.19791707
ENSG000001	13017.9111	0.2771442	0.10896804	2.54335321	0.01097942	0.03755817
ENSG000001	2578.03498	0.19232339	0.06222862	3.09059384	0.00199757	0.00939875
ENSG000001	1871.31447	1.49213062	0.23705362	6.29448579	3.08E-10	2.08E-08
ENSG000001	2509.10747	-0.2380035	0.11157779	-2.1330728	0.03291875	0.0880528
ENSG000001	1232.01927	0.15491296	0.07611713	2.03519185	0.04183157	0.10543041
ENSG000001	5943.68912	0.25318873	0.14479017	1.74865965	0.08034987	0.1709293
ENSG000001	766.550059	-0.2980371	0.12359623	-2.411377	0.01589241	0.0502693
ENSG000001	1036.77309	-0.1418261	0.08322834	-1.7040601	0.08836986	0.18370128
ENSG000001	1089.67486	-0.3707015	0.1846315	-2.0077911	0.0446655	0.11043796
ENSG000001	129.202994	0.93490196	0.20294118	4.60676324	4.09E-06	5.97E-05
ENSG000001	5852.75426	0.21732374	0.10655461	2.03955276	0.04139489	0.10454808
ENSG000001	647.725123	0.09696117	0.05428256	1.78623081	0.07406189	0.16107887
ENSG000001	10770.6558	1.04797754	0.20450654	5.12442053	2.98E-07	6.53E-06
ENSG000001	81.6680686	-0.4166804	0.17095795	-2.4373271	0.01479629	0.04759188
ENSG000001	8845.67749	-0.2297458	0.07703884	-2.9822077	0.00286178	0.01253282
ENSG000001	546.102813	0.47799734	0.11451467	4.17411465	2.99E-05	0.00030758
ENSG000001	41.3114231	1.45191602	0.26157908	5.55058147	2.85E-08	9.66E-07
ENSG000001	145.062704	-0.2108409	0.11684755	-1.80441	0.07116706	0.1566389
ENSG000001	866.347921	-0.1995574	0.11969748	-1.6671814	0.09547835	0.19451481
ENSG000001	8003.81352	0.66299944	0.17217013	3.85083884	0.00011771	0.00094582
ENSG000001	3503.96092	-0.2077212	0.06762895	-3.0714838	0.00212998	0.00988963

ENSG000001	761.852053	-0.2026436	0.08232935	-2.4613774	0.01384047	0.04516937
ENSG000001	4093.39969	0.38588352	0.06461415	5.97212053	2.34E-09	1.15E-07
ENSG000001	372.848221	0.21804919	0.08384056	2.60076032	0.00930174	0.03297145
ENSG000001	72115.5746	0.35439916	0.1610321	2.20079816	0.02775032	0.07726674
ENSG000001	64.1401064	-0.3718091	0.21425491	-1.7353584	0.08267733	0.17503238
ENSG000001	886.411711	-0.3967155	0.09203297	-4.3105805	1.63E-05	0.00018632
ENSG000001	2048.62518	0.21513043	0.05225023	4.11731059	3.83E-05	0.00037521
ENSG000001	588.879753	-0.3810185	0.08717977	-4.3704923	1.24E-05	0.00014879
ENSG000001	29.2762493	0.94813307	0.23692366	4.00185048	6.28E-05	0.00056342
ENSG000001	918.904295	1.11681316	0.18963989	5.88912571	3.88E-09	1.80E-07
ENSG000001	1117.56382	0.27593648	0.06476552	4.26054618	2.04E-05	0.00022404
ENSG000001	27101.7986	0.56762327	0.10032439	5.65787889	1.53E-08	5.68E-07
ENSG000001	537.161211	0.31229827	0.14224155	2.19554882	0.02812425	0.07802299
ENSG000001	7559.68194	0.70105235	0.21831018	3.21126727	0.00132151	0.0067697
ENSG000001	232.432339	0.4326863	0.10936223	3.95645098	7.61E-05	0.00066149
ENSG000001	737.427214	-0.3481179	0.08038604	-4.3305761	1.49E-05	0.00017286
ENSG000001	4377.43181	-0.3577934	0.09397116	-3.8074808	0.00014039	0.00108819
ENSG000001	10127.2128	-0.2455366	0.11029463	-2.2261884	0.02600156	0.07361436
ENSG000001	3183.02206	0.21648111	0.10737045	2.01620749	0.04377828	0.10882224
ENSG000001	153.85352	0.15831217	0.07863858	2.01316164	0.04409763	0.10942829
ENSG000001	2371.83803	-0.1766699	0.07733884	-2.2843617	0.02235028	0.06547796
ENSG000001	860.951989	-0.2980188	0.08065439	-3.6950102	0.00021988	0.0015584
ENSG000001	3378.65359	-0.1712543	0.09514619	-1.7999075	0.07187525	0.15783791
ENSG000001	865.913222	0.4255503	0.19012573	2.23825735	0.02520428	0.07193349
ENSG000001	1045.00262	-0.1936048	0.08844717	-2.1889322	0.02860177	0.07908743
ENSG000001	1351.67056	-0.1717268	0.05977659	-2.8728099	0.00406839	0.01668235
ENSG000001	2121.48802	-0.22517	0.1168092	-1.9276734	0.05389577	0.12687488
ENSG000001	1081.47069	-0.179511	0.08809886	-2.037609	0.04158905	0.10494697
ENSG000001	1624.69686	0.38934294	0.10974016	3.54786206	0.00038837	0.00248843
ENSG000001	720.918286	0.29148782	0.07535693	3.86809558	0.00010969	0.00089123
ENSG000001	949.521337	-0.1095036	0.06390341	-1.7135803	0.08660583	0.18089081
ENSG000001	4259.67083	-0.3988505	0.17902924	-2.2278511	0.02589045	0.07337145
ENSG000001	1351.93819	-0.1737729	0.07740755	-2.2449092	0.02477396	0.07105532
ENSG000001	573.301914	0.33185658	0.06316552	5.25376162	1.49E-07	3.70E-06
ENSG000001	1939.38571	-0.1143867	0.05561388	-2.0568013	0.03970534	0.10173528
ENSG000001	1877.45262	-0.3910029	0.09928878	-3.9380373	8.22E-05	0.00070214
ENSG000001	1750.38626	-0.1459426	0.07894416	-1.8486816	0.06450381	0.14585089
ENSG000001	387.208176	0.34254097	0.17693915	1.93592528	0.05287685	0.12510648
ENSG000001	731.742544	-0.1431786	0.07362395	-1.9447292	0.05180758	0.12303875
ENSG000001	870.332836	0.25792429	0.14588422	1.7680068	0.07705977	0.16575573
ENSG000001	22240.2416	0.25517719	0.09480465	2.69161063	0.00711079	0.02635978
ENSG000001	2630.82707	-0.5054272	0.09413857	-5.3689706	7.92E-08	2.20E-06
ENSG000001	8972.66989	-0.3405367	0.09695153	-3.5124427	0.00044401	0.00277376

ENSG000001	23923.3488	0.45264708	0.15752595	2.87347619	0.00405982	0.01665663
ENSG000001	138.45041	0.40198593	0.13417446	2.99599443	0.00273551	0.01207477
ENSG000001	1652.14194	-0.1985012	0.11450621	-1.7335413	0.08299951	0.17550014
ENSG000001	134.09771	-0.3354096	0.11122095	-3.0157056	0.00256382	0.01146006
ENSG000001	3208.63478	-0.2235788	0.08827944	-2.5326259	0.01132117	0.0384807
ENSG000001	1200.38843	-0.2959584	0.09595278	-3.0844175	0.00203951	0.00953725
ENSG000001	1726.74183	-0.1119689	0.04535235	-2.4688663	0.01355419	0.04441518
ENSG000001	1578.8083	-0.1772	0.08220644	-2.1555491	0.03111889	0.08428106
ENSG000001	355.136092	-0.2396795	0.09555175	-2.5083738	0.01212883	0.04060789
ENSG000001	26194.4638	0.32654275	0.1752902	1.86286938	0.06248065	0.14229805
ENSG000001	2645.1579	-0.1854526	0.03682804	-5.0356356	4.76E-07	9.75E-06
ENSG000001	1813.9257	-0.1145897	0.05099354	-2.2471415	0.02463099	0.07075485
ENSG000001	3433.4074	-0.1159908	0.05456618	-2.1256906	0.03352903	0.08937885
ENSG000001	11587.6191	1.91221399	0.24226693	7.8930046	2.95E-15	7.24E-13
ENSG000001	528.84233	0.53345957	0.16292136	3.27433775	0.0010591	0.00567635
ENSG000001	133.569126	-0.5900238	0.12997845	-4.5393969	5.64E-06	7.84E-05
ENSG000001	4592.35131	-0.1903276	0.05558533	-3.4240621	0.00061693	0.00363014
ENSG000001	1748.20612	0.26040779	0.09672263	2.69231517	0.00709579	0.02632436
ENSG000001	302.259929	1.23373918	0.20467476	6.02780327	1.66E-09	8.48E-08
ENSG000001	111.847268	-0.5431476	0.11070941	-4.9060655	9.29E-07	1.70E-05
ENSG000001	7744.18619	-0.2517408	0.08954513	-2.8113292	0.00493373	0.01961979
ENSG000001	2226.69351	-0.3617237	0.10574737	-3.4206399	0.00062474	0.00366867
ENSG000001	7920.15944	-0.2300885	0.08310438	-2.7686689	0.00562858	0.02177918
ENSG000001	42.8244135	1.26527346	0.23948572	5.28329405	1.27E-07	3.28E-06
ENSG000001	52.5024292	0.90935349	0.25305964	3.59343547	0.00032635	0.00216168
ENSG000001	5176.45132	0.19609387	0.08816581	2.22414856	0.02613845	0.07379999
ENSG000001	2841.55912	0.48784417	0.17669085	2.76100412	0.00576239	0.02220798
ENSG000001	66125.8062	1.76455338	0.31218831	5.65220838	1.58E-08	5.81E-07
ENSG000001	31824.4226	0.51354201	0.13303809	3.86011274	0.00011333	0.00091521
ENSG000001	150.700334	-1.1581144	0.25807018	-4.487595	7.20E-06	9.53E-05
ENSG000001	3530.60529	0.24632909	0.10925104	2.25470706	0.02415172	0.06962906
ENSG000001	1488.17535	-0.6673226	0.2407837	-2.7714607	0.00558054	0.02162795
ENSG000001	3362.77592	-0.3605396	0.1399238	-2.576685	0.00997528	0.03478861
ENSG000001	2462.50049	-0.1945922	0.07015538	-2.7737315	0.00554174	0.02152106
ENSG000001	129.60988	0.2977664	0.09932945	2.99776543	0.00271967	0.01202283
ENSG000001	951.62153	-0.368482	0.18922979	-1.9472728	0.05150204	0.12257434
ENSG000001	111949.454	0.27218068	0.15132907	1.79860144	0.07208175	0.15821944
ENSG000001	9944.98657	0.35031791	0.16827388	2.08183181	0.03735784	0.09714647
ENSG000001	149.04896	-1.035709	0.28443009	-3.6413484	0.00027121	0.00185948
ENSG000001	2128.92993	-0.2822002	0.1259481	-2.2406068	0.02505156	0.07162461
ENSG000001	2613.23531	0.32336802	0.09989522	3.23707194	0.00120763	0.00630221
ENSG000001	6359.58644	-0.9051557	0.18766258	-4.8233146	1.41E-06	2.42E-05
ENSG000001	434.374181	-0.125652	0.07095267	-1.7709274	0.07657278	0.16500221

ENSG000001	364.063207	-0.2544341	0.110308	-2.3065789	0.02107831	0.0628445
ENSG000001	309.846965	1.04089596	0.22819861	4.56135986	5.08E-06	7.16E-05
ENSG000001	18029.0869	0.20722073	0.12552709	1.65080485	0.09877843	0.19955419
ENSG000001	758.902696	-0.27575	0.08171471	-3.3745453	0.00073938	0.00421873
ENSG000001	14767.3382	0.32961636	0.14741897	2.23591548	0.02535731	0.07217666
ENSG000001	10993.3022	0.87029561	0.16966088	5.12961851	2.90E-07	6.38E-06
ENSG000001	101.660911	0.75089007	0.18507481	4.05722462	4.97E-05	0.00046564
ENSG000001	640.895011	0.36480975	0.11279375	3.2343082	0.00121938	0.00634763
ENSG000001	226.077483	-0.4288526	0.11155279	-3.8443915	0.00012085	0.00096622
ENSG000001	144.663289	1.00891373	0.22287607	4.52679255	5.99E-06	8.20E-05
ENSG000001	4326.02194	0.75378233	0.11756494	6.41162502	1.44E-10	1.12E-08
ENSG000001	1643.11423	0.16351668	0.07394183	2.21142315	0.02700655	0.07571973
ENSG000001	2941.84591	-0.1141274	0.04408926	-2.5885544	0.00963797	0.03382396
ENSG000001	4417.88867	0.24666425	0.08649022	2.85193221	0.00434544	0.01761397
ENSG000001	9912.94592	0.1935733	0.10891434	1.77729861	0.07551912	0.16346116
ENSG000001	60346.0694	0.2306468	0.11149303	2.06871058	0.03857325	0.09948562
ENSG000001	3436.34939	-0.3426826	0.10125054	-3.3845016	0.00071308	0.00409122
ENSG000001	104.677176	-0.6916792	0.15401126	-4.4910949	7.09E-06	9.41E-05
ENSG000001	1709.44664	0.21250568	0.0555811	3.82334411	0.00013165	0.00103487
ENSG000001	170.292249	0.3625759	0.09756232	3.71635193	0.00020212	0.00145316
ENSG000001	2732.55777	-0.3145403	0.06154248	-5.1109461	3.21E-07	6.94E-06
ENSG000001	4878.674	0.44608387	0.12318887	3.62113785	0.00029331	0.00197821
ENSG000001	473.762198	-0.4973463	0.1505106	-3.3043935	0.00095182	0.00519165
ENSG000001	431.156736	-0.3256479	0.07773983	-4.1889455	2.80E-05	0.00029083
ENSG000001	2180.34125	0.57609681	0.11973967	4.81124456	1.50E-06	2.55E-05
ENSG000001	66.3683407	-0.4631598	0.24468822	-1.8928571	0.05837688	0.13513996
ENSG000001	1696.01728	0.16707488	0.08679434	1.92495139	0.05423544	0.12752943
ENSG000001	43391.758	0.37881482	0.15886963	2.38443818	0.01710522	0.05329104
ENSG000001	71.1241191	1.14618089	0.21738166	5.27266609	1.34E-07	3.39E-06
ENSG000001	787.380496	0.2816008	0.10023024	2.80953941	0.00496124	0.01971839
ENSG000001	1877.3315	-0.2302235	0.05626109	-4.0920556	4.28E-05	0.00041157
ENSG000001	3095.00385	-0.1431484	0.0780891	-1.8331421	0.06678145	0.14927678
ENSG000001	1223.07697	0.10893743	0.04874824	2.23469453	0.02543741	0.07234232
ENSG000001	122.647195	0.38261037	0.14512424	2.63643324	0.00837827	0.0301935
ENSG000001	928.195562	0.15790034	0.07876963	2.00458409	0.04500753	0.11119101
ENSG000001	63.5882566	0.64526308	0.16950856	3.80666952	0.00014085	0.00109118
ENSG000001	7185.11131	0.17553682	0.09555116	1.83709777	0.06619546	0.14833961
ENSG000001	341.239351	0.82109041	0.17724611	4.63248762	3.61E-06	5.36E-05
ENSG000001	468.38164	-0.8019172	0.22285587	-3.5983672	0.00032022	0.00212898
ENSG000001	499.932666	0.59320635	0.17346255	3.41979506	0.00062668	0.00367561
ENSG000001	4097.34595	-0.3531289	0.10933968	-3.22965	0.00123942	0.00643565
ENSG000001	7573.90206	0.24893077	0.13389361	1.85916838	0.06300327	0.14319555
ENSG000001	2669.97116	0.11808594	0.05245117	2.25134996	0.02436338	0.07014155

ENSG000001	4986.66778	0.69331994	0.15197366	4.56210607	5.06E-06	7.15E-05
ENSG000001	115543.616	0.33909996	0.14169724	2.39313036	0.0167053	0.05230381
ENSG000001	5101.84714	-0.1556163	0.07658635	-2.031907	0.04216308	0.10604453
ENSG000001	5398.5264	0.24359323	0.06039229	4.03351526	5.49E-05	0.00050478
ENSG000001	194.658054	-0.4438781	0.12600681	-3.5226513	0.00042725	0.00268763
ENSG000001	3595.71922	-0.1392188	0.06573708	-2.1178128	0.03419092	0.09073528
ENSG000001	110.690171	0.85868318	0.22620873	3.79597728	0.00014706	0.00112725
ENSG000001	1126.54334	0.19144644	0.08476891	2.25845107	0.02391755	0.0690639
ENSG000001	115.054591	1.32372456	0.27071132	4.8898013	1.01E-06	1.82E-05
ENSG000001	70.3527177	0.3614011	0.20186471	1.79031344	0.07340354	0.16019841
ENSG000001	2247.14126	-0.1347295	0.07216318	-1.8670122	0.06189989	0.14144222
ENSG000001	363.992862	0.51997143	0.20988086	2.47745998	0.01323212	0.0435384
ENSG000001	365.866728	-0.1507912	0.07597462	-1.9847574	0.04717147	0.11502185
ENSG000001	3991.50923	0.17992763	0.10141313	1.77420448	0.07602933	0.16417304
ENSG000001	1228.11069	0.15392616	0.0699881	2.19931903	0.02785525	0.0775142
ENSG000001	51990.0861	1.62621384	0.29389627	5.53329179	3.14E-08	1.05E-06
ENSG000001	16.5713275	1.0832281	0.37705719	2.87284826	0.00406789	0.01668235
ENSG000001	1410.93269	0.12557867	0.06163494	2.03745906	0.04160406	0.10496657
ENSG000001	631.527162	0.31955375	0.13754101	2.3233343	0.0201612	0.06078387
ENSG000001	6707.75094	-0.1381886	0.06008624	-2.2998376	0.02145742	0.06367343
ENSG000001	2788.17324	0.20701031	0.07335279	2.82211917	0.00477074	0.01905536
ENSG000001	36.8811532	1.19628321	0.30190991	3.96238466	7.42E-05	0.00064766
ENSG000001	6619.67559	0.32812953	0.05742431	5.71412192	1.10E-08	4.36E-07
ENSG000001	4185.97373	0.36047931	0.12342055	2.92073974	0.00349201	0.01474033
ENSG000001	1759.52733	0.16306833	0.08567196	1.90340365	0.0569879	0.13258128
ENSG000001	656.252171	0.32645531	0.10924164	2.98837794	0.00280463	0.01232725
ENSG000001	20125.9155	0.3873614	0.12849427	3.01462002	0.00257301	0.01149052
ENSG000001	998.8362	-0.1564506	0.07829217	-1.9982912	0.0456851	0.11236923
ENSG000001	1672.26204	0.1942403	0.10173876	1.90920638	0.05623547	0.13118894
ENSG000001	4415.98582	0.21493459	0.09780871	2.19749962	0.02798478	0.07774028
ENSG000001	1851.97585	0.69784869	0.1196841	5.83075529	5.52E-09	2.44E-07
ENSG000001	9133.34922	-0.1966676	0.06200153	-3.1719793	0.00151404	0.00752965
ENSG000001	705.655944	-0.3429985	0.09752522	-3.5170236	0.00043641	0.00273796
ENSG000001	3129.83026	0.38797609	0.17976859	2.15819734	0.03091249	0.08383176
ENSG000001	5152.14386	-0.3191837	0.1079956	-2.9555249	0.00312138	0.01348624
ENSG000001	2830.87171	-0.2811193	0.05542331	-5.0722222	3.93E-07	8.22E-06
ENSG000001	750.104177	-0.1985244	0.06280451	-3.1609899	0.00157234	0.00777954
ENSG000001	417.720731	-0.3739728	0.17391877	-2.1502727	0.03153365	0.08518143
ENSG000001	42.1734313	-0.8699487	0.17827418	-4.8798356	1.06E-06	1.89E-05
ENSG000001	176.591648	0.35571572	0.19360425	1.83733423	0.06616057	0.14833961
ENSG000001	3583.51357	0.1498966	0.08343284	1.79661395	0.07239693	0.15871887
ENSG000001	1593.63504	0.49041168	0.10043564	4.88284513	1.05E-06	1.87E-05
ENSG000001	2546.86945	1.31389586	0.21790949	6.02954867	1.64E-09	8.42E-08

ENSG000001	18363.713	0.2203531	0.09269647	2.37714671	0.01744715	0.05408877
ENSG000001	100.309603	0.60321261	0.16709807	3.60993164	0.00030628	0.0020485
ENSG000001	3964.09487	0.32560734	0.16210929	2.00856677	0.0445831	0.11031176
ENSG000001	1208.36001	0.17789702	0.08037206	2.2134186	0.0268688	0.0754796
ENSG000001	195.004387	0.88072015	0.15233659	5.78140905	7.41E-09	3.14E-07
ENSG000001	5244.47984	0.10435236	0.02690273	3.87887581	0.00010494	0.0008594
ENSG000001	296.661002	0.32350422	0.10015539	3.23002296	0.0012378	0.00642956
ENSG000001	367.603705	-0.2055599	0.08667373	-2.3716521	0.01770876	0.05473582
ENSG000001	2431.92813	0.24640872	0.13101656	1.88074486	0.06000663	0.13805242
ENSG000001	4320.5674	-0.1525022	0.05014848	-3.0410137	0.00235783	0.01070781
ENSG000001	14772.5475	0.45859808	0.18185609	2.52176365	0.01167681	0.03944918
ENSG000001	180.167817	-0.2172863	0.1097373	-1.9800583	0.04769697	0.11584008
ENSG000001	233.942871	1.20709745	0.22656307	5.32786505	9.94E-08	2.66E-06
ENSG000001	154.925726	-0.5349477	0.16489215	-3.2442277	0.0011777	0.00617715
ENSG000001	616.139515	-0.1730378	0.09076962	-1.9063409	0.05660599	0.13196829
ENSG000001	602.831255	-0.5572907	0.23224246	-2.3996072	0.01641267	0.05156964
ENSG000001	4529.85362	0.3474236	0.1526332	2.27619944	0.02283408	0.06655831
ENSG000001	1394.75867	-0.1279598	0.0432068	-2.9615671	0.00306078	0.01325605
ENSG000001	798.881875	-0.2014024	0.11313093	-1.7802592	0.07503355	0.1626775
ENSG000001	239.115029	-1.7045619	0.40190259	-4.2412314	2.22E-05	0.00024069
ENSG000001	668.506773	0.36257136	0.0868935	4.17259482	3.01E-05	0.0003092
ENSG000001	275.867929	0.56166667	0.19323953	2.90658277	0.003654	0.01530813
ENSG000001	11.088228	-0.3676079	0.20064106	-1.832167	0.06692654	0.1495319
ENSG000001	559.431418	-0.1236853	0.06773161	-1.8261098	0.06783373	0.15095349
ENSG000001	89.6520401	0.49493227	0.20786304	2.38104986	0.01726338	0.05363382
ENSG000001	1391.05008	-0.1912045	0.08409399	-2.2736994	0.02298407	0.06686319
ENSG000001	1035.45229	-0.1594864	0.06019398	-2.6495402	0.00806014	0.02926497
ENSG000001	205.811671	-0.3902377	0.11744773	-3.3226501	0.00089167	0.00491536
ENSG000001	2017.4564	-0.2183111	0.06271337	-3.4810938	0.00049937	0.00305936
ENSG000001	996.030776	0.14061067	0.06462371	2.17583732	0.02956743	0.08102319
ENSG000001	704.591669	-0.1218941	0.06821321	-1.7869577	0.07394433	0.1610148
ENSG000001	2335.59325	-0.1592082	0.07785235	-2.0450014	0.04085472	0.10356866
ENSG000001	1376.82469	0.16163319	0.06704593	2.41078324	0.01591831	0.05031824
ENSG000001	417.197287	-0.4136514	0.24250872	-1.7057177	0.08806064	0.18326886
ENSG000001	442.004966	-0.2604658	0.08579789	-3.0358064	0.00239893	0.01084398
ENSG000001	5324.53185	0.75836989	0.1740548	4.35707534	1.32E-05	0.00015627
ENSG000001	52.2118207	-0.6863751	0.20386621	-3.3667916	0.00076048	0.00431029
ENSG000001	552.706357	-0.3027089	0.07346301	-4.1205616	3.78E-05	0.00037079
ENSG000001	3502.21274	0.24286749	0.0694895	3.49502441	0.00047402	0.00293717
ENSG000001	12781.8055	0.0936768	0.04886563	1.9170283	0.05523434	0.12933646
ENSG000001	2632.09618	0.25008072	0.11162046	2.24045587	0.02506134	0.07163845
ENSG000001	189.37155	-0.2411733	0.12962058	-1.8606099	0.06279929	0.14286641
ENSG000001	2346.57662	0.16397856	0.09461996	1.73302287	0.0830916	0.17565272

ENSG000001	202.586246	-0.4597567	0.16917772	-2.7175962	0.0065758	0.02472455
ENSG000001	4630.55008	0.40072425	0.08758006	4.57551923	4.75E-06	6.81E-05
ENSG000001	19.8871036	0.61955742	0.28296689	2.18950495	0.02856016	0.07899027
ENSG000001	68.5979454	0.41960831	0.16162986	2.59610635	0.00942869	0.03327488
ENSG000001	2735.12065	0.16483674	0.08563983	1.92476732	0.05425847	0.12756289
ENSG000001	77.7887274	-0.4699823	0.23296713	-2.017376	0.04365629	0.1086495
ENSG000001	3782.41888	0.32464003	0.11025904	2.9443393	0.00323645	0.01386353
ENSG000001	1786.92201	0.69875263	0.12825584	5.44811543	5.09E-08	1.55E-06
ENSG000001	1485.88005	-0.2819445	0.08134788	-3.4659106	0.00052844	0.00320046
ENSG000001	1546.85364	0.29553005	0.10420303	2.83609831	0.00456684	0.01835223
ENSG000001	150.788136	1.33027286	0.23416394	5.68094661	1.34E-08	5.01E-07
ENSG000001	36.9789836	0.93714639	0.25102002	3.73335311	0.00018895	0.00137345
ENSG000001	3142.50742	-0.1236018	0.06445833	-1.9175456	0.05516865	0.12926205
ENSG000001	212.823209	0.32161039	0.1371176	2.34550775	0.01900118	0.05803714
ENSG000001	74.04392	-0.4299705	0.21847634	-1.9680414	0.04906328	0.1182546
ENSG000001	5695.72387	0.58767935	0.13200803	4.45184533	8.51E-06	0.00010905
ENSG000001	168.592621	0.2041693	0.10879441	1.87665239	0.06056575	0.13896358
ENSG000001	1839.53674	0.45868141	0.19635456	2.33598554	0.019492	0.05917207
ENSG000001	1467.69508	0.17857171	0.10018744	1.78237623	0.0746879	0.16221943
ENSG000001	1381.2935	-0.1934328	0.11279094	-1.7149678	0.08635113	0.18052177
ENSG000001	1517.51655	0.43984041	0.12876541	3.41582751	0.00063589	0.00372205
ENSG000001	183.005761	-0.176232	0.09049923	-1.9473321	0.05149493	0.12257434
ENSG000001	234.368014	1.07043881	0.20276244	5.27927558	1.30E-07	3.33E-06
ENSG000001	1155.22582	0.30206891	0.07047014	4.28648095	1.82E-05	0.00020367
ENSG000001	598.247805	-0.418351	0.16299991	-2.5665718	0.01027094	0.035631
ENSG000001	1086.7514	-0.2506471	0.10178113	-2.4626088	0.01379303	0.04506532
ENSG000001	228.625604	-0.225045	0.07280753	-3.0909581	0.00199512	0.00939638
ENSG000001	3777.44443	0.34409365	0.13147792	2.61712116	0.00886749	0.0316724
ENSG000001	75.9830727	-1.0065871	0.28904709	-3.4824329	0.00049688	0.00305271
ENSG000001	1474.27559	0.84039412	0.22454916	3.7425841	0.00018214	0.00133466
ENSG000001	122.767385	-0.4714121	0.17696173	-2.6639213	0.00772356	0.02823397
ENSG000001	1018.59548	0.48039424	0.1989851	2.41422222	0.01576884	0.04992501
ENSG000001	744.096246	0.15457895	0.07622763	2.02785988	0.04257455	0.1068013
ENSG000001	127.389195	1.09646664	0.2607628	4.20484298	2.61E-05	0.00027567
ENSG000001	2414.60199	0.24701291	0.09335783	2.64587235	0.00814806	0.02953237
ENSG000001	2762.01763	0.65816399	0.23006524	2.8607711	0.00422612	0.01720253
ENSG000001	3916.39936	-0.2447419	0.08021699	-3.0509982	0.00228082	0.01043
ENSG000001	1509.80889	0.85327275	0.19307128	4.41947017	9.89E-06	0.00012357
ENSG000001	227.699043	0.37312245	0.13414902	2.78140265	0.00541246	0.02110071
ENSG000001	1242.04796	-0.1907636	0.06567724	-2.9045615	0.00367768	0.01538064
ENSG000001	713.245451	-0.3631673	0.07062473	-5.1422113	2.72E-07	6.08E-06
ENSG000001	564.37848	0.28826853	0.11465829	2.5141534	0.01193185	0.04011528
ENSG000001	2680.17825	0.16093873	0.07832093	2.05486238	0.0398923	0.102113

ENSG000001	1515.7045	-0.1638441	0.04459574	-3.6739845	0.0002388	0.0016656
ENSG000001	14734.091	0.33329884	0.16170971	2.06109359	0.03929411	0.10093153
ENSG000001	3497.65195	-0.1384967	0.06527813	-2.1216397	0.033868	0.09005979
ENSG000001	4364.62842	0.27631744	0.11748951	2.35184773	0.01868042	0.05732329
ENSG000001	1639.44019	-0.2645423	0.08745315	-3.0249598	0.00248666	0.01117725
ENSG000001	5313.2179	-0.2419975	0.06812012	-3.5525112	0.00038157	0.00245246
ENSG000001	327.850227	-0.2969664	0.17062528	-1.7404597	0.08177833	0.17335713
ENSG000001	411.441909	-0.2230585	0.10514246	-2.1214881	0.03388074	0.09007714
ENSG000001	65.2945637	-0.3394569	0.15727476	-2.1583684	0.0308992	0.08381141
ENSG000001	505.174461	0.36546239	0.10364812	3.52599161	0.0004219	0.00266089
ENSG000001	5506.66384	0.51695047	0.09623157	5.37194269	7.79E-08	2.18E-06
ENSG000001	547.225014	-0.4474857	0.16052164	-2.7876972	0.00530841	0.02079583
ENSG000001	8600.99392	0.20452524	0.08743368	2.33920434	0.01932486	0.05874093
ENSG000001	72.4902262	-1.1329933	0.18243297	-6.2104636	5.28E-10	3.25E-08
ENSG000001	12440.2411	-0.3734533	0.0847846	-4.4047305	1.06E-05	0.00013059
ENSG000001	935.689002	0.10387523	0.06174906	1.68221574	0.09252701	0.19029512
ENSG000001	2073.34679	3.30907449	0.60751071	5.44694017	5.12E-08	1.56E-06
ENSG000001	40.81487	1.01557678	0.39098908	2.59745562	0.00939173	0.03322538
ENSG000001	7381.67841	0.2591306	0.0924672	2.80240556	0.00507231	0.0200498
ENSG000001	4465.76863	0.30637461	0.12638751	2.4240892	0.01534684	0.04894971
ENSG000001	894.05449	0.21445969	0.06979868	3.07254657	0.00212241	0.009866
ENSG000001	3653.46586	0.16213383	0.08796105	1.84324565	0.06529315	0.14708473
ENSG000001	807.537102	0.37886189	0.091637	4.13437695	3.56E-05	0.00035316
ENSG000001	2252.836	0.18625202	0.08705739	2.1394165	0.03240195	0.08694334
ENSG000001	92.718353	0.34736839	0.11070086	3.13790143	0.00170162	0.00828897
ENSG000001	5904.01561	0.90087427	0.1445415	6.23263417	4.59E-10	2.89E-08
ENSG000001	5387.6851	0.13881129	0.06571769	2.11223641	0.03466618	0.09178343
ENSG000001	1321.48695	0.37011855	0.09888985	3.74273534	0.00018203	0.00133453
ENSG000001	2782.46839	-0.3001962	0.10059221	-2.9842889	0.00284238	0.01245541
ENSG000001	1710.33415	0.30864478	0.11767206	2.62292334	0.00871789	0.03121504
ENSG000001	277.07075	0.16233388	0.09521554	1.70490951	0.0882113	0.18347697
ENSG000001	167.363404	0.23087823	0.11801106	1.9564117	0.05041667	0.12060541
ENSG000001	2352.64384	-0.1443204	0.06672137	-2.1630312	0.03053878	0.08312953
ENSG000001	19405.9168	0.46683761	0.19331954	2.41484964	0.01574171	0.04989064
ENSG000001	172.90713	1.21545171	0.25018699	4.8581731	1.18E-06	2.07E-05
ENSG000001	4506.75558	0.18220692	0.10518895	1.73218692	0.08324029	0.17578747
ENSG000001	4970.91354	-0.2927981	0.08735102	-3.3519716	0.00080238	0.00450722
ENSG000001	3859.31556	-0.1256331	0.06847147	-1.8348242	0.06653175	0.14887943
ENSG000001	1034.59073	0.15560407	0.0599136	2.59714098	0.00940033	0.03324132
ENSG000001	23352.9254	0.24144529	0.12973629	1.86104675	0.06273757	0.14274843
ENSG000001	10088.3563	0.13271735	0.07043399	1.88427987	0.05952713	0.13721074
ENSG000001	2060.29787	-0.2329891	0.05960821	-3.9086736	9.28E-05	0.00077535
ENSG000001	3771.64662	-0.1202854	0.06968005	-1.7262526	0.08430197	0.17735734

ENSG000001	548.519033	-0.1994157	0.09654733	-2.0654705	0.0388785	0.10011249
ENSG000001	15312.1271	0.8164831	0.1976889	4.13014135	3.63E-05	0.00035875
ENSG000001	1067.9691	0.20919731	0.07250377	2.88533036	0.00391003	0.01614267
ENSG000001	140.204689	-0.5331834	0.16286584	-3.2737581	0.00106127	0.0056838
ENSG000001	863.794409	-0.175383	0.08321359	-2.1076242	0.03506351	0.09255924
ENSG000001	555.713352	0.51524444	0.16488937	3.12478862	0.00177933	0.00861247
ENSG000001	36.7523025	0.95677116	0.23280387	4.10977344	3.96E-05	0.00038455
ENSG000001	1427.25153	-0.3319166	0.05257768	-6.3128794	2.74E-10	1.92E-08
ENSG000001	61.198996	0.2957824	0.15650793	1.88988758	0.058773	0.13594833
ENSG000001	891.218408	-0.271936	0.16188834	-1.6797751	0.09300109	0.19102605
ENSG000001	618.580014	-0.243475	0.08681845	-2.8044156	0.00504079	0.01994153
ENSG000001	1345.48597	-0.3817138	0.08908018	-4.2850591	1.83E-05	0.00020467
ENSG000001	9055.95737	-0.1731109	0.05631177	-3.0741507	0.00211103	0.00982096
ENSG000001	31422.3479	0.17613312	0.10020616	1.75770747	0.0787973	0.16833286
ENSG000001	10517.9152	0.28536429	0.11550002	2.47068596	0.01348542	0.04423987
ENSG000001	66.5980136	-0.3945716	0.22448389	-1.7576832	0.07880143	0.16833286
ENSG000001	20.0812595	1.99267273	0.41759905	4.7717368	1.83E-06	3.03E-05
ENSG000001	182.367979	-0.3653875	0.14380198	-2.5409072	0.01105653	0.03775952
ENSG000001	4984.05292	-0.1894017	0.08283078	-2.2866107	0.02221856	0.06521074
ENSG000001	485.935092	-0.2015398	0.09553484	-2.1095947	0.03489328	0.09226127
ENSG000001	3374.45455	-0.3030461	0.1816411	-1.6683784	0.09524064	0.19416707
ENSG000001	16441.5478	-0.2744865	0.11824327	-2.3213706	0.02026685	0.06099001
ENSG000001	2636.62238	0.40374869	0.10704517	3.77175992	0.0001621	0.00121929
ENSG000001	9225.56977	-0.1997492	0.10765176	-1.8555122	0.06352312	0.14410577
ENSG000001	121140.632	0.49705168	0.17394145	2.85758042	0.00426884	0.01735693
ENSG000001	6040.15351	-0.2862884	0.08585098	-3.3347139	0.00085387	0.00473404
ENSG000001	16504.45	0.36861008	0.17063863	2.16017962	0.03075877	0.08355565
ENSG000001	2730.37258	-0.083475	0.04999499	-1.6696669	0.09498528	0.19375553
ENSG000001	92.8509525	0.30705487	0.1234902	2.48647156	0.01290169	0.0426533
ENSG000001	206.244263	-0.3060514	0.1145709	-2.6712843	0.00755616	0.0276849
ENSG000001	76.8271807	0.69652211	0.16882589	4.125683	3.70E-05	0.00036403
ENSG000001	66.48504	-0.3112981	0.14970772	-2.0793724	0.03758313	0.09759219
ENSG000001	342.695816	0.75674342	0.22048226	3.43221915	0.00059866	0.00353561
ENSG000001	7502.28112	-0.1429577	0.08257869	-1.731169	0.08342162	0.17614474
ENSG000001	129.809983	0.23938812	0.12784677	1.87246129	0.06114281	0.14015442
ENSG000001	1157.4585	0.1183493	0.03621138	3.26828984	0.001082	0.00576918
ENSG000001	72.0114697	-0.7216553	0.24626441	-2.9304084	0.00338517	0.01438422
ENSG000001	1047.37051	-0.1722929	0.08265986	-2.0843596	0.03712747	0.09666888
ENSG000001	80.024601	0.68380772	0.16643771	4.10849028	3.98E-05	0.00038644
ENSG000001	5385.64876	0.47951663	0.14316725	3.34934579	0.00081003	0.00454663
ENSG000001	1634.65737	-0.2355432	0.0695572	-3.3863244	0.00070836	0.00406897
ENSG000001	125.338537	0.37047425	0.12659975	2.92634273	0.00342973	0.01453233
ENSG000001	6205.48567	0.43947485	0.12830089	3.42534527	0.00061402	0.00361636

ENSG000001	2152.32965	0.3598842	0.13684206	2.62992381	0.0085404	0.03069332
ENSG000001	6488.39189	0.44852888	0.12078782	3.71336166	0.00020452	0.00146463
ENSG000001	2313.84768	-0.1904565	0.04567791	-4.1695535	3.05E-05	0.00031202
ENSG000001	2498.97528	-0.1598291	0.06485818	-2.4642859	0.01372866	0.04489549
ENSG000001	4832.62689	-0.265301	0.06988692	-3.7961459	0.00014696	0.00112725
ENSG000001	4214.48424	1.87791249	0.38312392	4.90157982	9.51E-07	1.73E-05
ENSG000001	2089.41155	0.22551314	0.08405102	2.68305074	0.00729539	0.0268722
ENSG000001	5693.64975	-0.128518	0.07244504	-1.7740069	0.07606201	0.16420621
ENSG000001	1314.68458	-0.1438765	0.07756919	-1.8548147	0.0636227	0.1442639
ENSG000001	16798.0094	0.2761635	0.14500249	1.90454312	0.05683949	0.13233609
ENSG000001	87.0732363	-0.3574305	0.14402835	-2.4816679	0.01307691	0.04312432
ENSG000001	523.978658	0.22437538	0.08769022	2.55872742	0.01050561	0.03628868
ENSG000001	1354.1753	0.47221798	0.17404866	2.71313763	0.00666494	0.02497543
ENSG000001	222.282546	0.37845231	0.09867433	3.83536747	0.00012538	0.00099429
ENSG000001	278.832931	-0.2658832	0.14791367	-1.7975566	0.0722473	0.15848677
ENSG000001	729.36773	0.24897848	0.10232667	2.43317282	0.01496716	0.04806676
ENSG000001	107.534716	-1.2205658	0.23139437	-5.2748294	1.33E-07	3.37E-06
ENSG000001	3325.5485	-0.1626629	0.06061861	-2.6833823	0.00728816	0.02686606
ENSG000001	4183.08679	-0.1781861	0.09107147	-1.9565525	0.0504001	0.12060541
ENSG000001	691.435214	1.22139398	0.24748071	4.93530986	8.00E-07	1.49E-05
ENSG000001	3654.42521	0.4506362	0.10459909	4.30822314	1.65E-05	0.00018773
ENSG000001	383.22795	-0.0934855	0.05491908	-1.7022414	0.08871013	0.18423409
ENSG000001	1681.56201	-0.1996654	0.09001552	-2.2181215	0.02654655	0.07470471
ENSG000001	10927.5389	0.38059542	0.15875159	2.3974274	0.01651065	0.05180464
ENSG000001	76.5732405	-1.2662399	0.27199419	-4.6553932	3.23E-06	4.90E-05
ENSG000001	1645.96959	0.3336634	0.10634007	3.13770155	0.00170278	0.00829183
ENSG000001	5069.95668	0.47768626	0.19081509	2.50339869	0.01230069	0.04107203
ENSG000001	1261.16551	0.31303	0.09180958	3.4095569	0.00065069	0.00378573
ENSG000001	584.779338	-0.2964579	0.08497271	-3.4888603	0.00048508	0.00299359
ENSG000001	701.415135	-0.2267935	0.11438892	-1.9826523	0.04740628	0.11542434
ENSG000001	283.98497	-0.4870292	0.102238	-4.7636808	1.90E-06	3.14E-05
ENSG000001	8378.41692	2.2914282	0.36886587	6.21209052	5.23E-10	3.25E-08
ENSG000001	3474.85895	-0.3375032	0.09175794	-3.6781902	0.00023489	0.00164234
ENSG000001	596.77545	-0.188513	0.08122742	-2.3208055	0.02029735	0.06105731
ENSG000001	462.537157	-0.2579704	0.0805494	-3.2026353	0.00136176	0.00693422
ENSG000001	679.495348	0.22760674	0.08698408	2.61664818	0.00887978	0.0317085
ENSG000001	1045.23552	0.17507121	0.08867498	1.97430227	0.04834738	0.11704688
ENSG000001	1354.06513	0.43849129	0.1684342	2.60333874	0.00923207	0.03275655
ENSG000001	310.082918	-0.6863049	0.11295129	-6.0761138	1.23E-09	6.53E-08
ENSG000001	314.792248	-0.7821666	0.13462792	-5.8098394	6.25E-09	2.69E-07
ENSG000001	1437.55664	-0.078442	0.04675488	-1.6777278	0.09340026	0.19157433
ENSG000001	106.248804	0.28412125	0.16468039	1.72528889	0.08447542	0.1775933
ENSG000001	1837.3739	-0.1831559	0.08412435	-2.1772044	0.02946532	0.08086078

ENSG000001	1226.2358	-0.1601942	0.06956996	-2.3026345	0.02129942	0.06333429
ENSG000001	134.907245	0.52874493	0.28298193	1.86847597	0.06169576	0.14108705
ENSG000001	565.097336	0.24286454	0.0764546	3.17658521	0.0014902	0.00744373
ENSG000001	684.404678	-0.1986011	0.08189983	-2.4249268	0.01531148	0.04886921
ENSG000001	1037.05918	-0.3377246	0.08389726	-4.0254547	5.69E-05	0.00051975
ENSG000001	6748.99261	-0.1911884	0.07320238	-2.6117787	0.00900725	0.03210039
ENSG000001	95.6723217	0.2979361	0.110448	2.69752365	0.00698573	0.02599242
ENSG000001	467.16043	-0.1496036	0.08416732	-1.7774553	0.07549336	0.16345424
ENSG000001	134.363645	1.22314813	0.36088102	3.38933899	0.00070061	0.00402972
ENSG000001	175.693238	0.49346504	0.17853783	2.76392426	0.00571108	0.02205338
ENSG000001	8673.39643	-0.1953152	0.09785049	-1.9960575	0.04592767	0.11277004
ENSG000001	232.694157	-0.9624254	0.28059382	-3.4299594	0.00060367	0.00356192
ENSG000001	123.753319	0.90194518	0.20573622	4.38398832	1.17E-05	0.00014068
ENSG000001	120.559755	-1.180066	0.24446916	-4.8270546	1.39E-06	2.38E-05
ENSG000001	323.770345	-0.4485711	0.12439729	-3.6059555	0.00031101	0.00207206
ENSG000001	677.230029	0.15919929	0.06408142	2.48432842	0.0129796	0.04285222
ENSG000001	564.515258	-0.3648218	0.10927089	-3.3386911	0.00084174	0.00468831
ENSG000001	2670.88817	-0.3274574	0.0636121	-5.1477222	2.64E-07	5.92E-06
ENSG000001	2688.5487	-0.2650258	0.04553082	-5.8207996	5.86E-09	2.56E-07
ENSG000001	1473.18728	0.10722701	0.05721495	1.87410824	0.06091551	0.13972181
ENSG000001	2387.76227	-0.1508585	0.04715221	-3.1993944	0.00137717	0.00699299
ENSG000001	8138.44313	0.27791287	0.11820058	2.35119727	0.01871311	0.05738071
ENSG000001	333.168921	-0.2603001	0.10304307	-2.5261288	0.01153272	0.0390909
ENSG000001	5170.75189	-0.2315918	0.11038055	-2.098121	0.03589446	0.09420346
ENSG000001	814.461636	-0.2491249	0.10309683	-2.4164168	0.01567411	0.04976348
ENSG000001	4908.91957	0.11570444	0.06795462	1.70267217	0.08862944	0.18416159
ENSG000001	115.961963	-0.5701084	0.17765057	-3.2091559	0.00133125	0.00680758
ENSG000001	689.763735	-0.4102455	0.24126195	-1.7004154	0.08905282	0.18477631
ENSG000001	208.701959	0.41500952	0.14852708	2.79416732	0.00520335	0.02045062
ENSG000001	1386.97063	0.44267239	0.08838167	5.00864457	5.48E-07	1.09E-05
ENSG000001	1076.88463	0.95052733	0.23940529	3.97036901	7.18E-05	0.0006316
ENSG000001	788.864087	-0.1149534	0.05744462	-2.0011161	0.04537987	0.11184387
ENSG000001	219.211966	-0.1714469	0.10377518	-1.6520996	0.09851424	0.19923467
ENSG000001	686.080925	0.41492118	0.17009054	2.43941361	0.01471112	0.04739159
ENSG000001	7135.77147	-0.2289434	0.05858346	-3.9079877	9.31E-05	0.0007771
ENSG000001	34.5256217	-0.5205069	0.26286801	-1.9801073	0.04769147	0.11584008
ENSG000001	5025.95147	0.80717872	0.22379917	3.60671012	0.0003101	0.00206836
ENSG000001	1605.58051	-0.2168989	0.07082409	-3.0625016	0.00219495	0.01012362
ENSG000001	5619.06106	0.13357918	0.0657806	2.03067753	0.04228772	0.10630852
ENSG000001	61.0375019	-0.586951	0.17708227	-3.3145666	0.00091785	0.00503672
ENSG000001	785.744979	-0.1363303	0.07366825	-1.8505974	0.06422749	0.14538495
ENSG000001	253.16293	0.80734879	0.17663283	4.57077424	4.86E-06	6.91E-05
ENSG000001	533.190668	0.49758815	0.12316365	4.04005688	5.34E-05	0.00049594

ENSG000001	110.264386	0.17895056	0.10129203	1.76667953	0.07728191	0.16603635
ENSG000001	10317.2625	-0.2658979	0.08454352	-3.1451014	0.00166029	0.00813689
ENSG000001	2349.90705	-0.3552527	0.0873747	-4.0658527	4.79E-05	0.00045137
ENSG000001	222.38127	-0.349946	0.10451565	-3.3482639	0.0008132	0.00455959
ENSG000001	167.979083	0.44285116	0.19513012	2.26951715	0.0232369	0.06749729
ENSG000001	7011.22475	0.26868054	0.13553814	1.98232423	0.04744296	0.11545544
ENSG000001	49820.024	0.36060804	0.12082994	2.98442613	0.00284111	0.01245359
ENSG000001	102.060472	0.59060458	0.23821576	2.4792842	0.01316464	0.04335442
ENSG000001	174.83277	0.37487078	0.11005531	3.40620348	0.00065873	0.00382333
ENSG000001	36.7377214	0.55677823	0.24889084	2.23703791	0.02528386	0.07208734
ENSG000001	826.176625	0.17920399	0.08148571	2.19920755	0.02786317	0.07752136
ENSG000001	140.293335	-0.356949	0.1349582	-2.6448856	0.00817185	0.02960381
ENSG000001	743.355854	-0.3003329	0.13708601	-2.1908356	0.02846369	0.07882882
ENSG000001	685.59864	-0.4502468	0.11111823	-4.0519615	5.08E-05	0.0004744
ENSG000001	1456.20347	-0.2435952	0.10215212	-2.3846323	0.0170962	0.05327439
ENSG000001	386.878787	0.23962297	0.13018677	1.84060924	0.06567884	0.14749487
ENSG000001	433.054825	0.15469838	0.07003119	2.20899246	0.02717517	0.07603063
ENSG000001	118.907134	-0.3483058	0.12950324	-2.6895529	0.00715478	0.0264822
ENSG000001	898.453797	0.21555704	0.11723766	1.83863303	0.06596918	0.14805507
ENSG000001	41.9858699	-0.5057773	0.22996622	-2.1993549	0.0278527	0.0775142
ENSG000001	1172.20001	0.18630859	0.09127394	2.0412025	0.0412307	0.10429705
ENSG000001	410.543008	0.21400124	0.08785701	2.4357902	0.0148593	0.04776906
ENSG000001	1549.96164	-0.2205328	0.08196965	-2.69042	0.00713621	0.02642022
ENSG000001	139.058292	0.33572269	0.18431014	1.82150964	0.06852942	0.15210445
ENSG000001	162.894658	-0.2260587	0.10373599	-2.1791732	0.0293188	0.08061255
ENSG000001	1750.44956	0.16462987	0.05870136	2.80453256	0.00503896	0.01994153
ENSG000001	1946.18224	0.39855468	0.11306062	3.52514143	0.00042326	0.00266829
ENSG000001	646.492169	0.64809504	0.12114028	5.3499548	8.80E-08	2.40E-06
ENSG000001	355.822807	0.38811416	0.12462838	3.11417166	0.00184462	0.0088399
ENSG000001	468.442751	0.49854574	0.14637953	3.40584339	0.0006596	0.00382684
ENSG000001	1830.62067	-0.0912229	0.05187771	-1.7584224	0.07867566	0.16823236
ENSG000001	1416.35421	0.16561251	0.08409466	1.96935834	0.04891196	0.11799959
ENSG000001	1746.69054	0.26233548	0.06875518	3.81550123	0.00013591	0.00105968
ENSG000001	649.927106	-0.2392188	0.09739498	-2.4561714	0.01404261	0.04572609
ENSG000001	3237.09934	0.45352422	0.16279067	2.78593488	0.00533736	0.02087536
ENSG000001	353.879498	-0.574971	0.14632832	-3.9293214	8.52E-05	0.00072338
ENSG000001	1074.23352	0.34584178	0.06586647	5.25064969	1.52E-07	3.75E-06
ENSG000001	253.160969	0.34595996	0.0797918	4.33578359	1.45E-05	0.00016941
ENSG000001	657.625081	-0.1297311	0.07174863	-1.8081333	0.07058575	0.15569058
ENSG000001	1278.08379	-0.1711801	0.08587798	-1.993294	0.04622926	0.11332035
ENSG000001	2148.65376	0.32900413	0.13575622	2.42349212	0.01537209	0.04899789
ENSG000001	149.349834	-0.3122167	0.1609228	-1.9401647	0.05235968	0.12414644
ENSG000001	137.521673	0.54908962	0.15879332	3.45788864	0.00054443	0.00327946

ENSG000001	99841.8085	0.82933489	0.22935887	3.61588322	0.00029933	0.00201128
ENSG000001	465.208862	0.87315444	0.1591776	5.48541041	4.13E-08	1.32E-06
ENSG000001	243.73106	-0.2986974	0.10232394	-2.9191347	0.00351004	0.0148035
ENSG000001	380.122291	-0.2009482	0.09937379	-2.0221449	0.04316138	0.1078439
ENSG000001	1233.89228	-0.1568437	0.08325528	-1.8838885	0.05958007	0.13723983
ENSG000001	266.581968	0.22997538	0.12718507	1.80819469	0.0705762	0.15569058
ENSG000001	286.487308	-0.2764004	0.12504953	-2.210327	0.02708248	0.07582988
ENSG000001	15184.5436	0.29941521	0.11862371	2.52407565	0.0116003	0.03923733
ENSG000001	4463.33166	-0.0795318	0.03712633	-2.1421938	0.03217789	0.08653445
ENSG000001	492.316314	-0.8846597	0.12012783	-7.3643191	1.78E-13	2.90E-11
ENSG000001	12420.9127	0.39983612	0.18728663	2.13488873	0.0327701	0.08775239
ENSG000001	89.1478052	0.95753006	0.21218093	4.51279973	6.40E-06	8.65E-05
ENSG000001	63.0126057	0.4666513	0.26811918	1.74046221	0.08177789	0.17335713
ENSG000001	4609.17455	-0.3929124	0.06335776	-6.201488	5.59E-10	3.38E-08
ENSG000001	1044.63642	-0.1915827	0.08945809	-2.1415913	0.03222639	0.0866023
ENSG000001	3484.26761	-0.2042845	0.10126233	-2.0173792	0.04365595	0.1086495
ENSG000001	364.852964	0.26631301	0.11338873	2.34867264	0.01884046	0.05768001
ENSG000001	516.283341	-1.0018167	0.17464129	-5.7364253	9.67E-09	3.89E-07
ENSG000001	2954.65915	0.49127709	0.13685549	3.58975056	0.00033099	0.00218258
ENSG000001	3625.0807	0.17458825	0.08901949	1.96123636	0.04985146	0.11970795
ENSG000001	4834.18395	0.44783771	0.19568401	2.28857596	0.022104	0.06497985
ENSG000001	324.258614	0.75042345	0.16012309	4.68654103	2.78E-06	4.31E-05
ENSG000001	3812.589	0.59117147	0.14635803	4.03921439	5.36E-05	0.00049709
ENSG000001	170.872626	0.35226142	0.10187756	3.45769383	0.00054482	0.00327989
ENSG000001	678.824033	0.15551307	0.05659931	2.74761408	0.00600306	0.02290397
ENSG000001	111.907032	0.54876172	0.14802588	3.70720117	0.00020956	0.00149627
ENSG000001	506.766585	-0.2059641	0.0843454	-2.4419129	0.01460967	0.04721176
ENSG000001	994.962257	0.19782089	0.0662215	2.98726068	0.0028149	0.01236304
ENSG000001	277.053106	0.97544637	0.16876079	5.78005331	7.47E-09	3.15E-07
ENSG000001	257.67023	-0.2672196	0.0856017	-3.1216622	0.00179833	0.00868125
ENSG000001	159.650572	0.29909009	0.13271175	2.25368201	0.02421618	0.06977912
ENSG000001	1675.78051	0.22829545	0.05706357	4.00072172	6.31E-05	0.00056577
ENSG000001	295.2596	3.93385756	0.57178166	6.87999961	5.99E-12	6.38E-10
ENSG000001	1011.95723	-0.400845	0.13844834	-2.8952678	0.00378835	0.01575706
ENSG000001	83.1783674	-0.3330169	0.16861672	-1.9749935	0.04826888	0.11689838
ENSG000001	1398.93061	-0.2802173	0.13193581	-2.1238913	0.03367923	0.08967305
ENSG000001	2695.73465	-0.3403258	0.16946145	-2.0082784	0.04461371	0.11036866
ENSG000001	13595.4856	-0.2976739	0.11855979	-2.5107495	0.01204752	0.04039139
ENSG000001	550.555949	-0.2214489	0.08025873	-2.7591876	0.00579453	0.02231502
ENSG000001	28310.4232	1.21169358	0.19468473	6.2238758	4.85E-10	3.03E-08
ENSG000001	1005.10082	-0.4018876	0.07157134	-5.6152025	1.96E-08	6.99E-07
ENSG000001	674.870089	0.45248591	0.17660062	2.5621989	0.01040117	0.03597942
ENSG000001	394.879067	0.34836457	0.13612651	2.55912373	0.01049364	0.03626463

ENSG000001	40.6984435	1.86642338	0.37731349	4.9466118	7.55E-07	1.42E-05
ENSG000001	783.714202	1.28953999	0.28360358	4.5469807	5.44E-06	7.61E-05
ENSG000001	7580.65617	2.12226079	0.25429214	8.3457586	7.08E-17	2.38E-14
ENSG000001	10564.5063	-0.2049961	0.07015212	-2.9221651	0.00347607	0.01468587
ENSG000001	565.763315	0.14300755	0.05521019	2.59023838	0.00959095	0.03369475
ENSG000001	3966.67539	0.88210411	0.23558794	3.74426677	0.00018092	0.00132979
ENSG000001	726.341648	0.29417143	0.10129314	2.90415953	0.0036824	0.01538574
ENSG000001	63.3883405	-0.8077456	0.19207173	-4.2054374	2.61E-05	0.00027514
ENSG000001	603.137734	0.16638702	0.0849462	1.95873417	0.05014393	0.12019111
ENSG000001	4425.54932	0.42256035	0.07398739	5.71124831	1.12E-08	4.43E-07
ENSG000001	660.346025	0.29435067	0.06508464	4.52258261	6.11E-06	8.32E-05
ENSG000001	915.085672	-0.2348402	0.04508539	-5.2087872	1.90E-07	4.53E-06
ENSG000001	2922.99743	0.30473962	0.09724256	3.13380901	0.00172553	0.0083857
ENSG000001	46.4931269	1.17017413	0.22571738	5.18424472	2.17E-07	5.05E-06
ENSG000001	390.648444	-0.1699537	0.09879467	-1.720272	0.08538302	0.17905961
ENSG000001	96.8941322	0.51748482	0.20813172	2.48633325	0.0129067	0.04266015
ENSG000001	16581.7869	0.21562787	0.12736118	1.6930423	0.09044742	0.1871072
ENSG000001	18662.1708	-0.204828	0.06653459	-3.0785192	0.00208032	0.00970303
ENSG000001	127.925532	-0.2432364	0.12422044	-1.9581026	0.05021797	0.12030887
ENSG000001	6061.15913	0.19853608	0.10615713	1.87020956	0.06145472	0.14062029
ENSG000001	4265.84159	-0.2567584	0.1094349	-2.3462206	0.01896488	0.05796292
ENSG000001	18682.6311	0.26934276	0.12129096	2.22063355	0.02637579	0.07434572
ENSG000001	145.673628	0.3052144	0.08573255	3.56007599	0.00037075	0.00239671
ENSG000001	4470.91342	-0.2907607	0.10158483	-2.8622453	0.00420651	0.01713234
ENSG000001	784.265835	0.20572993	0.06553106	3.1394264	0.00169279	0.00825705
ENSG000001	887.320557	0.1903192	0.07863258	2.42036078	0.01550512	0.04933508
ENSG000001	355.257351	-0.3233968	0.05784056	-5.5911762	2.26E-08	7.89E-07
ENSG000001	2031.11951	-0.2248247	0.11237296	-2.0007012	0.0454246	0.11191601
ENSG000001	733.668971	-0.8052202	0.22065064	-3.6492993	0.00026296	0.00180886
ENSG000001	11525.5314	0.21295455	0.09292735	2.29162398	0.02192735	0.06458213
ENSG000001	768.322329	-0.4956906	0.12851154	-3.8571683	0.00011471	0.00092527
ENSG000001	175.340207	-0.2133661	0.11661301	-1.8296936	0.06729577	0.150056
ENSG000001	734.092155	0.49728822	0.16480998	3.01734284	0.00255001	0.01141226
ENSG000001	4498.53619	0.32048649	0.10460861	3.06367212	0.00218638	0.01010122
ENSG000001	2312.01342	-0.2460084	0.06904353	-3.5630906	0.00036651	0.00237464
ENSG000001	393.971979	0.31752333	0.09895801	3.2086672	0.00133352	0.00681194
ENSG000001	1332.30728	-0.1000041	0.04751317	-2.1047661	0.03531167	0.09306171
ENSG000001	200.722148	-0.2434437	0.13805784	-1.7633457	0.0778422	0.16689358
ENSG000001	296.528507	-0.6323418	0.21128511	-2.9928363	0.00276398	0.01217075
ENSG000001	39.9623416	1.37468393	0.34917179	3.93698451	8.25E-05	0.00070439
ENSG000001	170.316089	-0.3645536	0.14118947	-2.5820169	0.00982248	0.03434666
ENSG000001	3246.00507	-0.3157451	0.08980153	-3.5160329	0.00043805	0.0027448
ENSG000001	2280.85895	0.21927414	0.11517182	1.90388705	0.0569249	0.13247726

ENSG000001	390.132045	-0.4711059	0.18787744	-2.5075172	0.01215827	0.04069704
ENSG000001	661.567019	1.45681663	0.29165533	4.9949941	5.88E-07	1.16E-05
ENSG000001	174.728582	-0.5732614	0.29942488	-1.9145416	0.05555099	0.12992689
ENSG000001	2668.52055	-0.4628696	0.0755994	-6.1226626	9.20E-10	5.19E-08
ENSG000001	1682.39439	-0.3372117	0.121582	-2.7735334	0.00554511	0.02152521
ENSG000001	2311.44728	0.24816866	0.10833432	2.29076683	0.0219769	0.06469811
ENSG000001	46.3170645	0.30607399	0.15520889	1.97201327	0.04860809	0.11746212
ENSG000001	70.2151647	-1.2464711	0.2614376	-4.7677575	1.86E-06	3.08E-05
ENSG000001	43.5930757	0.38656841	0.21064027	1.83520656	0.06647509	0.14877581
ENSG000001	27.177549	2.60052782	0.46592003	5.58148968	2.38E-08	8.26E-07
ENSG000001	4653.75396	0.34234214	0.09133945	3.74802059	0.00017824	0.00131538
ENSG000001	155.675211	-0.2569304	0.08391391	-3.0618333	0.00219986	0.01014302
ENSG000001	240.650961	0.9151102	0.36012789	2.54107007	0.01105138	0.03775644
ENSG000001	29249.3892	0.92515204	0.23245023	3.98000058	6.89E-05	0.00060951
ENSG000001	4188.72896	0.16099817	0.08844018	1.82041882	0.06869525	0.15233247
ENSG000001	603.919338	0.27841402	0.07830051	3.55571137	0.00037696	0.00242711
ENSG000001	764.629834	1.21252254	0.22205899	5.46036243	4.75E-08	1.46E-06
ENSG000001	150.999982	0.23249553	0.1213467	1.91596093	0.05537007	0.12962931
ENSG000001	72.1706208	-0.3521539	0.14425548	-2.4411821	0.01463927	0.04723365
ENSG000001	120.693647	0.34403418	0.09950001	3.45762949	0.00054495	0.00327989
ENSG000001	421.926417	0.29239727	0.10728249	2.72548939	0.00642062	0.0242165
ENSG000001	875.956751	0.68905754	0.12012559	5.73614261	9.69E-09	3.89E-07
ENSG000001	138.011929	-0.766791	0.24351593	-3.1488332	0.00163924	0.00805824
ENSG000001	55.7840907	-0.9285399	0.28924849	-3.2101806	0.00132652	0.00679054
ENSG000001	1966.33273	0.2517868	0.10496318	2.39881075	0.01644841	0.05165579
ENSG000001	103.987855	-0.8709782	0.22843478	-3.8128092	0.0001374	0.00106899
ENSG000001	3536.62699	0.38689885	0.11537824	3.35330868	0.00079852	0.00449247
ENSG000001	1492.44569	0.36007267	0.0924271	3.89574782	9.79E-05	0.00081041
ENSG000001	10065.6165	0.33040409	0.17850378	1.85096413	0.06417471	0.14531089
ENSG000001	26.3891689	-0.3890855	0.20796644	-1.870905	0.06135825	0.14049265
ENSG000001	143.060962	-0.3418088	0.15733064	-2.1725505	0.02981417	0.08155549
ENSG000001	1301.85668	0.58175913	0.17649268	3.29622236	0.00097994	0.005327
ENSG000001	4457.48047	-0.1886589	0.07444151	-2.5343234	0.01126647	0.03834994
ENSG000001	1018.45717	-0.2189213	0.07309982	-2.9948261	0.00274601	0.01211005
ENSG000001	1434.98991	0.15496566	0.09036482	1.71488934	0.08636552	0.18052177
ENSG000001	338.172181	-0.4139867	0.11042763	-3.7489407	0.00017758	0.0013119
ENSG000001	1362.33779	0.96545105	0.23787287	4.05868501	4.93E-05	0.00046334
ENSG000001	3827.5159	0.38838791	0.11626219	3.34062109	0.00083591	0.00466308
ENSG000001	3497.71275	0.36261947	0.15903115	2.28017895	0.02259708	0.06600078
ENSG000001	3871.19429	-0.1198596	0.0587464	-2.0402878	0.04132167	0.10441777
ENSG000001	900.773449	-0.2563176	0.07618216	-3.3645361	0.00076672	0.00433888
ENSG000001	4999.87583	0.14215183	0.06506816	2.18466045	0.02891375	0.07974074
ENSG000001	3418.46284	1.14960455	0.21154432	5.43434382	5.50E-08	1.64E-06

ENSG000001	4287.44912	0.51070859	0.12505751	4.08378978	4.43E-05	0.00042396
ENSG000001	476.010531	0.50377461	0.14735402	3.41880462	0.00062897	0.00368752
ENSG000001	7051.41607	1.17648658	0.19929534	5.90323179	3.56E-09	1.67E-07
ENSG000001	1513.6124	0.91457376	0.13605138	6.72226727	1.79E-11	1.69E-09
ENSG000001	805.368199	0.24200419	0.09125687	2.65190092	0.008004	0.02907572
ENSG000001	5209.32658	-0.1381711	0.06603785	-2.0923021	0.0364115	0.09523233
ENSG000001	164.066361	-0.2567667	0.07337387	-3.4994293	0.00046626	0.00289899
ENSG000001	314.18932	0.25116139	0.06446332	3.89619049	9.77E-05	0.0008094
ENSG000001	15195.3697	0.6569394	0.16661512	3.94285589	8.05E-05	0.00069197
ENSG000001	475.572691	0.1883949	0.07830992	2.4057604	0.01613885	0.05087107
ENSG000001	2010.78174	0.48348579	0.13462808	3.59126989	0.00032907	0.00217484
ENSG000001	348.730791	0.67000203	0.29425391	2.27695197	0.02278909	0.06646781
ENSG000001	6850.42459	0.51984308	0.21523677	2.41521502	0.01572592	0.04985152
ENSG000001	496.033523	0.2709886	0.12710767	2.13196106	0.03301005	0.08823184
ENSG000001	438.4932	0.3443312	0.10756762	3.20106736	0.0013692	0.00695984
ENSG000001	33561.2113	0.54383034	0.1902092	2.85911696	0.00424822	0.01727793
ENSG000001	1790.98371	0.30225349	0.11944487	2.53048532	0.01139048	0.03864495
ENSG000001	1126.75907	0.45286696	0.10718345	4.22515761	2.39E-05	0.00025623
ENSG000001	418.148424	-0.1630808	0.085943	-1.8975456	0.05775597	0.133938
ENSG000001	202.744222	0.26906286	0.1619219	1.66168301	0.09657635	0.19633758
ENSG000001	60.6854054	0.57598464	0.16359638	3.52076647	0.0004303	0.00270563
ENSG000001	176.863665	-0.1699025	0.08256352	-2.0578395	0.03960554	0.10151548
ENSG000001	55.7940863	1.25937469	0.38701738	3.25405204	0.00113771	0.00600877
ENSG000001	1245.50643	0.67004073	0.12418392	5.39555161	6.83E-08	1.96E-06
ENSG000001	298.65367	0.15449166	0.08608953	1.79454643	0.072726	0.15917533
ENSG000001	2524.79167	0.20131783	0.06742789	2.98567611	0.00282952	0.01241301
ENSG000001	1968.13999	0.29864306	0.0890486	3.3537087	0.00079736	0.00448869
ENSG000001	13282.1394	0.35329531	0.15474623	2.28306241	0.02242669	0.06561982
ENSG000001	20.692572	-0.494148	0.29800777	-1.6581717	0.09728282	0.197469
ENSG000001	1669.92043	-0.3206877	0.12730226	-2.5191045	0.01176537	0.03967527
ENSG000001	2211.02832	-0.4241455	0.0600381	-7.0646048	1.61E-12	2.03E-10
ENSG000001	199.894315	-0.3403641	0.12176716	-2.795204	0.0051867	0.02039622
ENSG000001	204.728806	0.29465742	0.12045746	2.44615335	0.01443896	0.04675395
ENSG000001	1269.74874	0.81911594	0.13927374	5.8813381	4.07E-09	1.87E-07
ENSG000001	527.032617	0.8217154	0.24437983	3.36245177	0.00077254	0.00436495
ENSG000001	81.3366527	-0.9698955	0.25578973	-3.7917687	0.00014958	0.0011423
ENSG000001	35.8067648	0.95804047	0.36489667	2.62551168	0.00865188	0.03100169
ENSG000001	1187.56058	0.72371231	0.1544492	4.68576289	2.79E-06	4.32E-05
ENSG000001	453.75639	0.21142024	0.0991899	2.13146952	0.03305048	0.08832361
ENSG000001	4202.87423	-0.2565273	0.06187132	-4.1461415	3.38E-05	0.00033993
ENSG000001	13081.2238	0.24815017	0.13209409	1.8785865	0.06030098	0.13850947
ENSG000001	336.083793	0.44500701	0.08504951	5.23232874	1.67E-07	4.10E-06
ENSG000001	925.611186	-0.1630821	0.07001206	-2.3293433	0.01984089	0.05999477

ENSG000001	1952.68778	-0.200105	0.0698342	-2.8654305	0.00416443	0.01700875
ENSG000001	247.700998	-0.2814259	0.16394591	-1.7165777	0.08605636	0.18018479
ENSG000001	8064.72488	-0.1988799	0.10801499	-1.841225	0.0655886	0.14736073
ENSG000001	1673.86806	-0.2099736	0.09078986	-2.3127424	0.02073681	0.0619835
ENSG000001	22197.5132	1.08969542	0.20536962	5.30602043	1.12E-07	2.97E-06
ENSG000001	2908.33269	0.47201084	0.14102085	3.34709965	0.00081662	0.00457654
ENSG000001	1225.45279	-0.1474905	0.08307894	-1.7753054	0.07584747	0.16393495
ENSG000001	9687.07866	0.50426441	0.15907079	3.17006281	0.00152406	0.00757689
ENSG000001	560.244873	-0.2235219	0.08948182	-2.4979583	0.01249109	0.0415328
ENSG000001	159.280687	0.30344904	0.08509901	3.56583529	0.0003627	0.00235202
ENSG000001	676.35646	0.18597388	0.11229753	1.65608164	0.09770529	0.19815058
ENSG000001	909.696694	-0.229731	0.10310318	-2.2281662	0.02586944	0.07335497
ENSG000001	340.754	0.75613554	0.13315547	5.67859183	1.36E-08	5.07E-07
ENSG000001	785.934188	-0.1957061	0.06669372	-2.9344002	0.00334193	0.01423538
ENSG000001	172.464113	0.23064745	0.11987951	1.92399396	0.05435534	0.12774916
ENSG000001	224.56164	1.1255571	0.20488089	5.49371441	3.94E-08	1.27E-06
ENSG000001	5140.31516	0.2063103	0.12020561	1.71631171	0.08610501	0.18021292
ENSG000001	461.994897	0.29395727	0.05574206	5.27352719	1.34E-07	3.39E-06
ENSG000001	80.4617639	1.31422443	0.25317991	5.19087176	2.09E-07	4.91E-06
ENSG000001	958.800657	0.13584431	0.05121864	2.65224337	0.00799589	0.02906456
ENSG000001	2421.36426	-0.2178258	0.05036918	-4.3245848	1.53E-05	0.00017697
ENSG000001	91.8306308	-0.7581436	0.15801782	-4.7978359	1.60E-06	2.71E-05
ENSG000001	10611.2742	0.34131116	0.09778006	3.49060103	0.00048194	0.00297985
ENSG000001	22.6213118	-0.5758311	0.30214043	-1.9058394	0.05667105	0.13207745
ENSG000001	71.4967502	0.60581185	0.28856318	2.09940799	0.03578095	0.09402478
ENSG000001	453.850158	0.26288144	0.11255718	2.33553689	0.01951539	0.05920827
ENSG000001	514.739187	-0.1758691	0.0655068	-2.6847463	0.00725849	0.02678393
ENSG000001	2107.41286	-0.4960329	0.11389701	-4.3551007	1.33E-05	0.00015717
ENSG000001	150.299036	-0.3418301	0.15020937	-2.275691	0.02286451	0.06662071
ENSG000001	379.654472	-0.4501127	0.14294542	-3.1488435	0.00163918	0.00805824
ENSG000001	86.1968669	0.34841138	0.10043127	3.4691523	0.0005221	0.00316547
ENSG000001	74.2535155	0.67699455	0.20265983	3.34054632	0.00083614	0.00466308
ENSG000001	387.879249	-0.2862798	0.07233018	-3.9579585	7.56E-05	0.00065852
ENSG000001	8571.66077	0.26834601	0.12998586	2.06442464	0.03897747	0.10031385
ENSG000001	29.0821244	-1.5809745	0.37370955	-4.23049	2.33E-05	0.00025116
ENSG000001	145.669988	-0.5918007	0.26102783	-2.2671938	0.02337839	0.06780435
ENSG000001	287.051691	-0.4479328	0.18583548	-2.4103728	0.01593623	0.0503639
ENSG000001	143.842407	1.17873448	0.26531824	4.44271929	8.88E-06	0.00011308
ENSG000001	383.927607	-0.3738924	0.14615881	-2.5581246	0.01052384	0.03632877
ENSG000001	77473.3142	0.40483612	0.23735822	1.70559133	0.08808419	0.18327905
ENSG000001	24376.5164	0.67819472	0.2165417	3.13193582	0.00173658	0.00842666
ENSG000001	89.428376	1.16388766	0.3237644	3.59485992	0.00032457	0.00215096
ENSG000001	1236.74315	-0.4466413	0.13866893	-3.2209182	0.00127781	0.00658733

ENSG000001	11318.93	-0.4288648	0.17964232	-2.3873263	0.01697142	0.05301047
ENSG000001	48514.7734	3.08720451	0.31249053	9.87935373	5.12E-23	4.36E-20
ENSG000001	2312.81585	0.29224981	0.11294473	2.58754704	0.0096662	0.03389839
ENSG000001	1709.15656	0.56963025	0.15912298	3.57981133	0.00034384	0.0022509
ENSG000001	155.116615	-0.3341091	0.12794943	-2.6112587	0.00902096	0.03214134
ENSG000001	2129.95625	0.49095662	0.1272624	3.85782945	0.0001144	0.00092328
ENSG000001	2180.37329	0.47578813	0.18225298	2.61059185	0.00903857	0.03218033
ENSG000001	351.853783	0.81512376	0.18655702	4.36930086	1.25E-05	0.00014948
ENSG000001	137.804588	-0.2213462	0.1079979	-2.0495415	0.0404092	0.10293793
ENSG000001	2373.87395	-0.1830569	0.08495658	-2.1547111	0.03118445	0.08442704
ENSG000001	44.8510484	1.52232053	0.27644338	5.50680768	3.65E-08	1.19E-06
ENSG000001	97.2686345	-0.2490057	0.10309722	-2.415251	0.01572437	0.04985152
ENSG000001	2293.70205	0.21844513	0.07973973	2.73947666	0.00615371	0.02338022
ENSG000001	624.431446	-0.2465895	0.06506334	-3.7899911	0.00015065	0.00114929
ENSG000001	641.638572	-0.1326669	0.0672649	-1.9723054	0.04857476	0.11744034
ENSG000001	6950.01081	0.10150415	0.05070772	2.00174931	0.0453117	0.11173296
ENSG000001	5005.49627	-0.4419398	0.12234201	-3.6123306	0.00030346	0.00203433
ENSG000001	8291.86071	-0.5672295	0.12390507	-4.5779361	4.70E-06	6.75E-05
ENSG000001	229.429332	-0.5932796	0.24318004	-2.4396722	0.0147006	0.04737876
ENSG000001	1491.10053	-0.2748735	0.07917226	-3.4718415	0.0005169	0.00314241
ENSG000001	1484.93832	-0.255197	0.1141653	-2.2353289	0.02539576	0.07226643
ENSG000001	805.9361	0.28150412	0.09144685	3.07833596	0.0020816	0.00970587
ENSG000001	143.536268	-0.2459694	0.13904487	-1.7689931	0.07689502	0.16554884
ENSG000001	573.000277	0.84218211	0.27451599	3.06787993	0.00215583	0.0099877
ENSG000001	66.737123	0.98275212	0.27107434	3.62539704	0.00028852	0.00195225
ENSG000001	730.848304	-0.2943034	0.05061441	-5.8146168	6.08E-09	2.62E-07
ENSG000001	2352.72382	0.18512895	0.06049944	3.06001099	0.00221329	0.01018872
ENSG000001	59.9986353	1.2664291	0.25439683	4.97816381	6.42E-07	1.25E-05
ENSG000001	237.973998	-0.5131026	0.19555086	-2.623883	0.00869336	0.03113492
ENSG000001	4801.70628	0.28422289	0.133019	2.13670891	0.03262167	0.08743575
ENSG000001	166.592752	0.43922932	0.22583651	1.94489954	0.05178707	0.1230118
ENSG000001	4304.49919	0.18927696	0.09234432	2.04968705	0.04039498	0.10291982
ENSG000001	621.741451	-0.5890154	0.16905576	-3.4841486	0.00049371	0.00303707
ENSG000001	38.4321606	0.84501716	0.27006866	3.12889756	0.00175463	0.00850431
ENSG000001	717.915356	0.60511965	0.21609235	2.80028268	0.00510579	0.02014915
ENSG000001	952.468425	1.24366493	0.22852673	5.44209826	5.27E-08	1.59E-06
ENSG000001	1642.17926	0.23623093	0.06586045	3.58684043	0.00033471	0.00220501
ENSG000001	2990.72604	-0.3814219	0.09900349	-3.8526105	0.00011687	0.00093953
ENSG000001	2757.77817	-0.1538111	0.03833815	-4.01196	6.02E-05	0.00054591
ENSG000001	96.633807	0.58533853	0.23660309	2.47392598	0.01336374	0.04392025
ENSG000001	976.60977	-0.3813116	0.06951598	-5.4852372	4.13E-08	1.32E-06
ENSG000001	12810.7365	0.65741167	0.21191373	3.10226087	0.00192049	0.0091278
ENSG000001	1285.22185	-0.2200696	0.10115773	-2.1755092	0.02959198	0.08103951

ENSG000001	566.908905	-0.7502365	0.17781236	-4.2192597	2.45E-05	0.00026206
ENSG000001	1758.03261	-0.3385505	0.0622147	-5.441648	5.28E-08	1.59E-06
ENSG000001	3635.03472	-0.2763657	0.08006198	-3.4518967	0.00055666	0.00332962
ENSG000001	3498.12345	0.16299531	0.08202765	1.98707754	0.0469138	0.11462983
ENSG000001	6589.05655	0.22352664	0.10953682	2.04065293	0.04128534	0.10436236
ENSG000001	672.978693	-0.2419805	0.13257864	-1.8251843	0.06797322	0.15112462
ENSG000001	562.263232	-1.0706384	0.23406511	-4.5741048	4.78E-06	6.83E-05
ENSG000001	14993.3101	0.31601964	0.12332696	2.56245387	0.01039354	0.03597156
ENSG000001	57.3133497	-0.6642727	0.3301909	-2.0117839	0.04424272	0.1096381
ENSG000001	10346.9882	0.32170436	0.09283559	3.46531296	0.00052961	0.00320577
ENSG000001	1173.59913	0.23725201	0.13426558	1.76703514	0.07722234	0.16593297
ENSG000001	2887.25648	0.14729518	0.08470588	1.73890161	0.08205207	0.17383568
ENSG000001	4663.32476	0.1967755	0.06738819	2.92002951	0.00349998	0.01476668
ENSG000001	74.4970289	0.32158072	0.1806786	1.77984951	0.07510059	0.16279849
ENSG000001	27985.9262	0.6305818	0.1533223	4.11278605	3.91E-05	0.00037994
ENSG000001	707.434823	0.35066824	0.07920788	4.42718904	9.55E-06	0.00012027
ENSG000001	537.117803	0.32429554	0.11970411	2.70914287	0.00674573	0.02523247
ENSG000001	4339.08954	-0.1992382	0.07038765	-2.8305846	0.0046463	0.01862089
ENSG000001	12372.7657	-0.1114317	0.05618681	-1.9832352	0.04734117	0.11532394
ENSG000001	1321.6937	-0.2391191	0.07972652	-2.9992415	0.00270653	0.01197967
ENSG000001	491.772818	-0.1484524	0.07600586	-1.9531707	0.05079938	0.12124062
ENSG000001	2860.01222	0.31984377	0.13726485	2.33012144	0.01979973	0.05989533
ENSG000001	12632.2786	-0.125253	0.07303412	-1.7149925	0.08634659	0.18052177
ENSG000001	142.391881	-0.2717812	0.11867761	-2.2900803	0.02201666	0.06477567
ENSG000001	155.810231	1.62548913	0.30271163	5.36976105	7.88E-08	2.20E-06
ENSG000001	188.745533	0.25846705	0.12093008	2.13732636	0.03257146	0.08734963
ENSG000001	3391.28734	-0.3397795	0.05840676	-5.8174692	5.97E-09	2.58E-07
ENSG000001	4658.61123	0.1949255	0.1005266	1.93904389	0.05249599	0.12436786
ENSG000001	3684.43818	0.34298049	0.12449982	2.75486743	0.00587159	0.02253292
ENSG000001	9858.66199	0.11178791	0.06061183	1.84432489	0.06513581	0.14684445
ENSG000001	1304.26285	-0.2856465	0.15946628	-1.7912659	0.07325063	0.15993699
ENSG000001	4043.04652	0.4220639	0.25547862	1.65205176	0.098524	0.19923467
ENSG000001	2375.48036	-0.1976968	0.09183396	-2.1527632	0.03133729	0.08480915
ENSG000001	2365.8462	-0.1799089	0.09157879	-1.9645256	0.04946917	0.11906628
ENSG000001	86.0782328	0.55352804	0.15594131	3.54959209	0.00038583	0.00247652
ENSG000001	43.0657763	0.64106806	0.17702756	3.62128961	0.00029314	0.00197797
ENSG000001	1692.80933	-0.2810276	0.13565802	-2.0715886	0.03830382	0.09895922
ENSG000001	1465.91924	0.15853003	0.09259556	1.71206953	0.08688386	0.18131467
ENSG000001	3064.30198	-0.2181185	0.05368665	-4.0628075	4.85E-05	0.00045641
ENSG000001	2506.82132	0.50274396	0.19130607	2.62795617	0.00858996	0.03081791
ENSG000001	221.451899	-0.3946866	0.23103479	-1.7083428	0.08757276	0.18243695
ENSG000001	628.301328	0.6835497	0.17639626	3.87508043	0.00010659	0.00086995
ENSG000001	8122.47454	0.14694899	0.06832448	2.15075162	0.03149581	0.08511095

ENSG000001	1790.4176	-0.5828651	0.17130606	-3.402478	0.00066778	0.00386345
ENSG000001	7092.36721	-0.0959583	0.0434107	-2.210476	0.02707215	0.07581561
ENSG000001	604.961403	-0.7810545	0.25271034	-3.0907104	0.00199678	0.00939811
ENSG000001	94.6366771	-0.554733	0.19291412	-2.8755441	0.00403332	0.0165526
ENSG000001	3338.15938	-0.2131381	0.12594567	-1.6923022	0.09058837	0.18721165
ENSG000001	117.590231	-0.3361731	0.17426791	-1.9290593	0.05372351	0.12668214
ENSG000001	1569.69179	0.23290003	0.10766418	2.16320817	0.03052517	0.08310809
ENSG000001	1566.05264	-0.1204708	0.05889937	-2.0453659	0.04081879	0.1035569
ENSG000001	1126.93708	0.79775592	0.14509535	5.4981494	3.84E-08	1.25E-06
ENSG000001	188.566211	0.35622789	0.1003055	3.55142932	0.00038314	0.00246147
ENSG000001	132.332942	-0.4440582	0.1317555	-3.3703197	0.00075081	0.00426868
ENSG000001	762.794294	-0.1981029	0.05765509	-3.4360003	0.00059037	0.00349803
ENSG000001	1468.93831	-0.5454648	0.2452025	-2.2245484	0.02611157	0.07376722
ENSG000001	1771.8245	-0.1749376	0.07585108	-2.3063304	0.02109218	0.06287291
ENSG000001	17774.5539	-0.1487906	0.08280091	-1.7969684	0.07234063	0.15864346
ENSG000001	1136.55335	-0.2146206	0.06440396	-3.3324133	0.00086096	0.00476671
ENSG000001	385.505189	0.27557762	0.0973444	2.83095518	0.00464092	0.01860865
ENSG000001	112.286933	0.30931764	0.06870779	4.5019297	6.73E-06	9.02E-05
ENSG000001	864.084374	0.30766075	0.09782919	3.14487666	0.00166157	0.00814039
ENSG000001	1564.70735	-0.3001701	0.0751308	-3.9953005	6.46E-05	0.00057673
ENSG000001	143.645324	-0.5112969	0.16568295	-3.0859961	0.00202872	0.00949719
ENSG000001	126.328561	0.40073393	0.13070587	3.06592147	0.00217	0.01004051
ENSG000001	3985.10805	0.66105487	0.21284212	3.1058461	0.00189735	0.00903879
ENSG000001	1810.91089	-0.3439685	0.13512014	-2.5456493	0.01090747	0.03737382
ENSG000001	372.327577	0.83917749	0.27977708	2.99945044	0.00270467	0.01197877
ENSG000001	358.904695	0.18561138	0.09551359	1.94329808	0.05198015	0.12338792
ENSG000001	1441.34999	-0.2025801	0.06240423	-3.2462554	0.00116934	0.00614269
ENSG000001	2062.96834	-0.2822761	0.08722263	-3.2362716	0.00121102	0.0063138
ENSG000001	9785.7418	-0.4398639	0.1751783	-2.5109497	0.01204069	0.04037811
ENSG000001	6500.82241	0.36621133	0.11340049	3.22936286	0.00124066	0.0064398
ENSG000001	881.496163	-0.1431372	0.08431412	-1.6976664	0.08957075	0.18558515
ENSG000001	218.462374	0.73010283	0.24907534	2.93125291	0.00337598	0.01435509
ENSG000001	164.582847	0.3432074	0.10777287	3.18454342	0.00144983	0.00730307
ENSG000001	90465.178	-0.2803834	0.13247302	-2.1165323	0.03429956	0.0909736
ENSG000001	237.933377	-0.4319087	0.13602282	-3.1752666	0.00149699	0.0074551
ENSG000001	2046.53529	-0.151777	0.0781839	-1.9412817	0.05222412	0.12388584
ENSG000001	2934.58513	0.21988172	0.11469478	1.91710314	0.05522483	0.12933646
ENSG000001	4498.97562	0.19019459	0.10590665	1.79587005	0.07251519	0.15885793
ENSG000001	480.396382	0.20748304	0.08227197	2.52191639	0.01167174	0.03944217
ENSG000001	5368.81956	0.33096399	0.08958723	3.69432097	0.00022048	0.00156187
ENSG000001	6969.92355	-0.1558834	0.08047116	-1.9371334	0.05272904	0.12479752
ENSG000001	143.254562	-0.4708616	0.24012537	-1.9608989	0.04989082	0.11976276
ENSG000001	16991.065	0.20513839	0.09798254	2.09362182	0.03629368	0.09504457

ENSG000001	3176.92779	-0.137459	0.06688153	-2.0552612	0.03985378	0.10204344
ENSG000001	708.148637	-0.1513942	0.07692276	-1.9681323	0.04905282	0.1182546
ENSG000001	82995.6811	0.96703321	0.16510931	5.85692702	4.72E-09	2.11E-07
ENSG000001	4506.33914	-0.1024612	0.05675393	-1.8053583	0.07101863	0.15643104
ENSG000001	56.4319275	1.4180481	0.22715228	6.24272012	4.30E-10	2.72E-08
ENSG000001	7115.89379	-0.141698	0.08266781	-1.7140649	0.08651681	0.18073503
ENSG000001	911.27795	-0.1615964	0.0734187	-2.2010253	0.02773424	0.07723681
ENSG000001	120.747963	-0.2748483	0.09047322	-3.0378971	0.00238235	0.01077862
ENSG000001	860.187653	0.65937199	0.2217075	2.97406262	0.00293885	0.01281997
ENSG000001	8600.85028	0.24503037	0.13833707	1.77125598	0.07651814	0.16493354
ENSG000001	1019.02269	-0.3874145	0.19515724	-1.9851402	0.04712887	0.11495828
ENSG000001	59816.6842	0.70082581	0.14067139	4.98200677	6.29E-07	1.22E-05
ENSG000001	896.872317	-0.3171343	0.08771459	-3.6155256	0.00029974	0.0020131
ENSG000001	83.3147623	0.87321226	0.17024246	5.12922723	2.91E-07	6.38E-06
ENSG000001	24056.759	0.62269017	0.15591147	3.99387025	6.50E-05	0.00057809
ENSG000001	2844.96122	0.1371898	0.03831275	3.5807872	0.00034256	0.00224693
ENSG000001	10275.4975	3.31256167	0.3725424	8.89177096	6.01E-19	2.81E-16
ENSG000001	2750.84526	-0.3023657	0.11442098	-2.6425724	0.00822789	0.02974471
ENSG000001	1107.43551	-0.1861503	0.07847354	-2.3721405	0.01768536	0.05469613
ENSG000001	328.431247	0.44348808	0.14270786	3.10766393	0.00188572	0.00899818
ENSG000001	2390.19652	0.38156273	0.10782882	3.53859677	0.00040226	0.00255818
ENSG000001	13044.0276	0.30241324	0.14848867	2.03660824	0.04168932	0.10512674
ENSG000001	863.742471	0.35884691	0.10923001	3.28524101	0.00101895	0.00549983
ENSG000001	35944.7116	-0.2303381	0.11994965	-1.9202896	0.05482133	0.12865651
ENSG000001	3214.07342	0.51910481	0.14981329	3.46501167	0.00053021	0.00320581
ENSG000001	307.316461	0.93710621	0.24049614	3.89655407	9.76E-05	0.00080865
ENSG000001	2267.92872	0.44238809	0.08912222	4.96383609	6.91E-07	1.32E-05
ENSG000001	3.42907698	3.39871847	0.87750596	3.87315714	0.00010743	0.00087573
ENSG000001	14257.2581	-0.314121	0.09647373	-3.2560265	0.00112983	0.00597368
ENSG000001	2812.9992	-0.1397256	0.06383455	-2.1888711	0.02860621	0.07908743
ENSG000001	32.2840918	-0.4250165	0.20330353	-2.0905517	0.03656827	0.09553913
ENSG000001	97.0686695	-0.3336753	0.10538823	-3.1661534	0.00154469	0.00765582
ENSG000001	695.990793	-0.7498428	0.19088527	-3.9282381	8.56E-05	0.00072537
ENSG000001	1385.23199	-0.1987471	0.10035339	-1.9804717	0.04765055	0.1158112
ENSG000001	83.2349276	0.38669788	0.17241159	2.2428764	0.02490479	0.07130349
ENSG000001	408.042529	0.4289719	0.1345671	3.18779168	0.00143364	0.00724925
ENSG000001	1219.95179	-0.1804888	0.05606745	-3.2191364	0.00128577	0.00660755
ENSG000001	5674.48436	-0.1973596	0.06127788	-3.2207324	0.00127863	0.00658733
ENSG000001	382.43393	-0.1603162	0.07431021	-2.157391	0.03097521	0.08398613
ENSG000001	2103.03091	-0.3083729	0.11440264	-2.6955055	0.00702819	0.02611372
ENSG000001	5601.17568	-0.3136606	0.13855618	-2.2637795	0.02358768	0.06828832
ENSG000001	262.031025	0.45440898	0.14176887	3.20528051	0.00134931	0.00688533
ENSG000001	153.864523	-0.1734975	0.08221642	-2.1102539	0.03483649	0.09216148

ENSG000001	177.574157	0.25621151	0.08616936	2.97334829	0.0029457	0.01283982
ENSG000001	697.900519	0.66580702	0.12310363	5.40850848	6.36E-08	1.85E-06
ENSG000001	2554.34191	-0.4111278	0.1148227	-3.5805446	0.00034288	0.00224693
ENSG000001	270.206948	-0.2193167	0.10969372	-1.9993552	0.04556994	0.11217871
ENSG000001	1711.07562	-0.3633668	0.11432488	-3.1783702	0.00148105	0.00741654
ENSG000001	58.6272615	0.6930418	0.21190581	3.27051819	0.00107351	0.00573236
ENSG000001	4951.91661	0.14744441	0.05304899	2.77940063	0.00544593	0.02121409
ENSG000001	877.758182	-1.7264638	0.40361257	-4.2775273	1.89E-05	0.00020995
ENSG000001	1121.74651	0.25588973	0.12019728	2.12891453	0.03326133	0.08880518
ENSG000001	858.744862	0.14717204	0.07698537	1.91168846	0.05591616	0.1306123
ENSG000001	1522.69561	0.2826425	0.16918169	1.67064478	0.09479186	0.19346995
ENSG000001	326.963277	0.22055654	0.10640052	2.07288974	0.03818254	0.09875924
ENSG000001	1352.90566	0.81198397	0.20245913	4.01060681	6.06E-05	0.00054746
ENSG000001	589.685425	-0.2312188	0.08800336	-2.6273858	0.00860437	0.03086198
ENSG000001	1289.81209	0.28419435	0.10853081	2.61855926	0.0088302	0.03157035
ENSG000001	329.036505	0.99452541	0.33172219	2.99806714	0.00271698	0.01201492
ENSG000001	252.133085	0.18170331	0.09795325	1.8550003	0.06359618	0.14424893
ENSG000001	686.335093	-0.2017121	0.11846661	-1.7026916	0.08862581	0.18416159
ENSG000001	3163.41515	-0.3088056	0.08295279	-3.7226671	0.00019713	0.00142435
ENSG000001	328.120259	-0.3623413	0.11320142	-3.2008548	0.00137021	0.00696253
ENSG000001	423.209819	-0.2098925	0.08728075	-2.4047967	0.01618147	0.05098323
ENSG000001	3307.53344	-0.3792193	0.06960052	-5.4485119	5.08E-08	1.55E-06
ENSG000001	91.4779164	-0.3516328	0.14307347	-2.4577077	0.01398269	0.0455822
ENSG000001	1516.77885	-0.5612736	0.27191295	-2.0641664	0.03900194	0.10035898
ENSG000001	364.537986	0.14963454	0.07798531	1.91875284	0.05501562	0.1289871
ENSG000001	729.278359	0.4432415	0.09603479	4.61542618	3.92E-06	5.77E-05
ENSG000001	61.914311	-0.7850329	0.19679733	-3.9890424	6.63E-05	0.00058889
ENSG000001	187.373801	-0.2478992	0.06699186	-3.7004368	0.00021523	0.00152994
ENSG000001	31.9575133	2.21529177	0.4295473	5.15727081	2.51E-07	5.72E-06
ENSG000001	66.8150788	0.58670862	0.23423601	2.50477555	0.01225291	0.04097598
ENSG000001	1160.32401	-0.2210597	0.08677084	-2.5476268	0.01084584	0.03722428
ENSG000001	126.860547	-0.2874838	0.17032165	-1.6878874	0.09143285	0.18854756
ENSG000001	1233.94194	-0.1114344	0.06312233	-1.7653718	0.0775013	0.16638432
ENSG000001	1843.43098	-0.3893971	0.08349544	-4.6636932	3.11E-06	4.74E-05
ENSG000001	23695.0067	-0.1948667	0.11284123	-1.7269106	0.08418372	0.17716001
ENSG000001	126.38679	-0.4370059	0.11290451	-3.8705794	0.00010858	0.00088269
ENSG000001	55.8218703	0.76805533	0.16873826	4.55175562	5.32E-06	7.46E-05
ENSG000001	1349.80094	0.59798769	0.1877319	3.18532813	0.0014459	0.00729638
ENSG000001	131.797321	0.43455262	0.13083245	3.32144373	0.00089553	0.00493478
ENSG000001	963.885329	-0.6377651	0.12758211	-4.9988599	5.77E-07	1.14E-05
ENSG000001	3278.70387	-0.2248304	0.04958353	-4.5343775	5.78E-06	7.98E-05
ENSG000001	121.466985	0.27986739	0.15636656	1.78981609	0.07348348	0.16030044
ENSG000001	82.6106579	-0.7399815	0.19937458	-3.7115138	0.00020602	0.00147391

ENSG000001	1016.40438	0.13296006	0.05318609	2.4999027	0.01242274	0.04136251
ENSG000001	3744.09123	-0.1509988	0.06434079	-2.3468599	0.01893237	0.0579002
ENSG000001	338.358341	1.6029218	0.22697104	7.06223045	1.64E-12	2.05E-10
ENSG000001	932.368275	0.25158855	0.07235206	3.4772827	0.00050652	0.00309229
ENSG000001	1004.68823	0.1381992	0.05329958	2.59287593	0.00951771	0.03351089
ENSG000001	37795.0349	0.68047867	0.1526299	4.4583577	8.26E-06	0.00010635
ENSG000001	7363.31875	0.82745499	0.18774826	4.40725779	1.05E-05	0.00012935
ENSG000001	27946.6994	0.21684506	0.11543417	1.87851706	0.06031047	0.13850947
ENSG000001	2032.45141	0.93149624	0.17873675	5.21155425	1.87E-07	4.48E-06
ENSG000001	1752.62893	-0.3920061	0.10897821	-3.5971058	0.00032178	0.00213584
ENSG000001	1775.46447	0.10006905	0.03431357	2.91631141	0.00354197	0.01492076
ENSG000001	186603.228	0.64888195	0.25010554	2.59443255	0.00947472	0.03340479
ENSG000001	386.045913	-0.1688838	0.07687798	-2.1967775	0.02803634	0.07783873
ENSG000001	854.9713	-0.5113363	0.12217274	-4.1853548	2.85E-05	0.00029494
ENSG000001	305.907944	0.96942178	0.26953772	3.59660899	0.00032239	0.00213851
ENSG000001	3239.02633	-0.1782999	0.0741471	-2.4046775	0.01618674	0.05098876
ENSG000001	3382.32679	0.20447638	0.10008549	2.04301716	0.04105074	0.10395072
ENSG000001	793.803891	0.18900475	0.10933482	1.72867838	0.08386667	0.17674956
ENSG000001	355.442819	0.18009127	0.09161493	1.96574152	0.04932848	0.11880659
ENSG000001	1715.65251	-0.1883288	0.08161039	-2.307657	0.02101822	0.06269116
ENSG000001	2865.21122	0.1482088	0.0739147	2.00513286	0.04494885	0.11106497
ENSG000001	74.8221091	0.52139378	0.2997143	1.73963595	0.08192296	0.17361293
ENSG000001	90.2266923	0.38684773	0.15017935	2.575905	0.00999781	0.0348474
ENSG000001	152.966253	-0.6011545	0.1370927	-4.3850217	1.16E-05	0.00014013
ENSG000001	11265.9452	-0.6193964	0.12918461	-4.7946605	1.63E-06	2.74E-05
ENSG000001	13329.6314	0.2459968	0.08765393	2.80645475	0.00500899	0.01987002
ENSG000001	1048.04251	0.43183626	0.12405528	3.48099873	0.00049955	0.00305936
ENSG000001	523.630612	0.22480613	0.10144461	2.21604799	0.02668822	0.07505964
ENSG000001	637.853931	0.15797179	0.05979832	2.64174301	0.00824806	0.02979049
ENSG000001	312.79555	0.2149149	0.09997318	2.14972564	0.03157692	0.08526652
ENSG000001	80.0286461	0.24026446	0.11593841	2.07234567	0.03823322	0.09884433
ENSG000001	1839.40304	0.60749982	0.14661853	4.1434042	3.42E-05	0.00034354
ENSG000001	45.2889873	1.36173689	0.35784438	3.8053885	0.00014158	0.00109392
ENSG000001	110.668005	0.25685759	0.08519458	3.01495228	0.0025702	0.01148148
ENSG000001	482.17342	0.50711205	0.10124157	5.00893123	5.47E-07	1.09E-05
ENSG000001	372.53992	0.23087693	0.07420173	3.11147637	0.00186154	0.00891216
ENSG000001	35.8676318	0.5620128	0.28535804	1.96950048	0.04889565	0.11797989
ENSG000001	2476.08443	0.30019565	0.13626426	2.20304022	0.02759191	0.07688622
ENSG000001	3189.60613	-0.582838	0.10991903	-5.3024303	1.14E-07	3.01E-06
ENSG000001	184.140958	1.44252895	0.38840858	3.71394712	0.00020405	0.00146197
ENSG000001	2214.92213	-0.2944191	0.06966629	-4.2261343	2.38E-05	0.00025531
ENSG000001	114533.282	0.24359763	0.1021343	2.38507181	0.01707579	0.05323634
ENSG000001	208.757625	0.31593501	0.12427692	2.54218579	0.01101616	0.03766078

ENSG000001	385.126406	0.25132873	0.08702574	2.88798143	0.00387723	0.01604382
ENSG000001	2850.0077	0.23011085	0.09733507	2.36411026	0.01807343	0.05568478
ENSG000001	2884.35391	0.08823039	0.03666121	2.4066414	0.01609997	0.05075958
ENSG000001	36272.8942	0.29810151	0.16178279	1.84260335	0.06538695	0.14715872
ENSG000001	75.0721519	0.25676193	0.11749079	2.18537918	0.02886106	0.0796552
ENSG000001	12032.7442	0.59160127	0.14963251	3.9536948	7.70E-05	0.00066846
ENSG000001	84284.8709	0.46580224	0.19985384	2.3307145	0.01976842	0.05983809
ENSG000001	303610.935	0.97981078	0.19880862	4.92841191	8.29E-07	1.54E-05
ENSG000001	10258.1783	1.34093194	0.26765695	5.00989031	5.45E-07	1.09E-05
ENSG000001	2258.05097	0.47511768	0.20182188	2.35414357	0.01856544	0.05700668
ENSG000001	299.114199	0.70071103	0.10339406	6.77709191	1.23E-11	1.24E-09
ENSG000001	3842.02248	0.30674757	0.09900519	3.09829803	0.00194636	0.00921767
ENSG000001	22097.2179	-0.3906018	0.12438389	-3.140292	0.00168779	0.00823824
ENSG000001	4078.26238	-0.3351997	0.06024034	-5.5643726	2.63E-08	8.97E-07
ENSG000001	101.577491	0.26666825	0.12054465	2.21219485	0.0269532	0.07559429
ENSG000001	43.6036828	0.52941515	0.15682661	3.37579922	0.00073602	0.00420286
ENSG000001	67.7917725	-0.556654	0.16957226	-3.2826949	0.0010282	0.00553737
ENSG000001	1861.31801	0.72375412	0.18601055	3.89093061	9.99E-05	0.00082325
ENSG000001	6611.18477	-0.3602748	0.08817634	-4.0858446	4.39E-05	0.0004205
ENSG000001	6706.01604	0.42387992	0.12742938	3.32639066	0.00087979	0.00485356
ENSG000001	1132.5347	0.09965505	0.04154778	2.3985651	0.01645945	0.05166805
ENSG000001	694.907263	0.20155136	0.12056082	1.6717816	0.0945674	0.19309343
ENSG000001	428.261164	0.23578759	0.12450608	1.89378379	0.05825371	0.13489795
ENSG000001	92571.6748	0.40398883	0.19676059	2.05319991	0.04005319	0.10235502
ENSG000001	44883.1509	0.32992462	0.090425	3.64859979	0.00026367	0.00181293
ENSG000001	10056.5773	0.20473932	0.07076684	2.89315351	0.00381395	0.01582264
ENSG000001	1246.4873	0.13100206	0.07590627	1.72583974	0.08437625	0.17747624
ENSG000001	139.641246	0.50857966	0.16785414	3.02989042	0.00244642	0.0110297
ENSG000001	1455.82328	-0.3434098	0.18396612	-1.866701	0.06194335	0.14148941
ENSG000001	1687.59991	0.13360597	0.06115922	2.1845599	0.02892113	0.07974504
ENSG000001	726.127183	0.63118163	0.13360312	4.7243029	2.31E-06	3.73E-05
ENSG000001	1297.13727	0.46710626	0.18743082	2.49215288	0.01269714	0.04209232
ENSG000001	89.5636385	-1.3410018	0.25113047	-5.3398609	9.30E-08	2.53E-06
ENSG000001	16.2689921	0.71330047	0.19288031	3.69815086	0.00021718	0.00154076
ENSG000001	162.483775	0.70942527	0.17761976	3.9940673	6.49E-05	0.00057797
ENSG000001	107.691044	0.31307257	0.14777969	2.11850871	0.03413201	0.09062874
ENSG000001	1520.34158	-0.1447638	0.07120601	-2.033028	0.0420497	0.10589345
ENSG000001	3019.58962	0.14456454	0.06219034	2.32454981	0.02009605	0.06063974
ENSG000001	142458.294	0.54016293	0.15086647	3.58040403	0.00034306	0.00224693
ENSG000001	34191.0096	1.12585146	0.17895287	6.29132961	3.15E-10	2.10E-08
ENSG000001	1661.99526	1.61122147	0.22478539	7.16782099	7.62E-13	1.08E-10
ENSG000001	264.113012	0.80003436	0.20569378	3.88944365	0.00010047	0.00082703
ENSG000001	577.476781	-0.2005369	0.1064795	-1.8833379	0.05965458	0.13735155

ENSG000001	2425.35583	0.28217711	0.13736055	2.05428051	0.03994855	0.10215969
ENSG000001	441.001974	-0.5751568	0.14988484	-3.8373245	0.00012438	0.00098898
ENSG000001	2088.18067	-0.1618679	0.05839933	-2.7717417	0.00557573	0.02162086
ENSG000001	6798.48702	0.66095521	0.19092266	3.46190027	0.00053638	0.00323635
ENSG000001	1177.09724	0.40954888	0.08140651	5.0309108	4.88E-07	9.92E-06
ENSG000001	91.246756	0.6143541	0.19699145	3.11868411	0.00181661	0.00874325
ENSG000001	4609.96953	0.21754177	0.05807018	3.74618711	0.00017954	0.00132368
ENSG000001	3441.56906	0.46350806	0.12835952	3.61101417	0.000305	0.00204374
ENSG000001	1348.21543	-0.1944023	0.06736567	-2.8857768	0.00390449	0.01612438
ENSG000001	12174.5061	0.23020446	0.09816503	2.34507615	0.01902319	0.05806764
ENSG000001	165.965987	0.48214646	0.19161708	2.5161977	0.01186286	0.03993894
ENSG000001	54.3494771	2.53375047	0.35194873	7.19920339	6.06E-13	8.86E-11
ENSG000001	2339.72089	0.1956465	0.0788064	2.48262199	0.01304194	0.0430384
ENSG000001	683.647672	-0.1327657	0.04048715	-3.2792063	0.00104099	0.00559796
ENSG000001	417.326382	-0.2794215	0.08082547	-3.4570972	0.00054603	0.00328365
ENSG000001	46.9669572	-1.0014081	0.21423481	-4.6743482	2.95E-06	4.54E-05
ENSG000001	65.6392714	-0.9263727	0.19695363	-4.7035068	2.56E-06	4.04E-05
ENSG000001	1096.42506	-0.1554935	0.07341039	-2.1181407	0.03416315	0.09067819
ENSG000001	30.1465236	2.31106625	0.45123128	5.12168892	3.03E-07	6.62E-06
ENSG000001	158.613563	-0.7344526	0.25385165	-2.8932355	0.00381295	0.01582264
ENSG000001	3355.12179	0.84091549	0.26401568	3.18509674	0.00144706	0.00729673
ENSG000001	640.872559	0.45952635	0.16979772	2.70631643	0.00680342	0.02542857
ENSG000001	354.828822	-0.9489649	0.28028362	-3.3857309	0.00070989	0.00407617
ENSG000001	4265.71298	0.42947912	0.16620682	2.58400418	0.00976606	0.03418413
ENSG000001	2661.68104	0.11989277	0.06115064	1.96061335	0.04992414	0.11978322
ENSG000001	19126.7022	1.15215551	0.19018048	6.0582218	1.38E-09	7.17E-08
ENSG000001	14.4088736	0.47833903	0.25355867	1.88650237	0.05922729	0.13675896
ENSG000001	794.274532	0.17326101	0.08420075	2.05771347	0.03961765	0.10152854
ENSG000001	15029.4957	1.05758744	0.16901159	6.25748477	3.91E-10	2.50E-08
ENSG000001	3229.9916	0.18954363	0.10375382	1.82685938	0.06772092	0.1507951
ENSG000001	93.2110207	-0.4642608	0.12367437	-3.7538966	0.00017411	0.00129215
ENSG000001	1874.85326	0.12721353	0.05890306	2.15971016	0.03079511	0.08362302
ENSG000001	2302.85367	-0.2675995	0.05902045	-4.5340132	5.79E-06	7.98E-05
ENSG000001	1352.72867	-0.204001	0.05163249	-3.9510204	7.78E-05	0.00067426
ENSG000001	1988.07151	0.14277773	0.07125317	2.00380883	0.04509054	0.1113771
ENSG000001	695.970428	-0.1219185	0.07376368	-1.652826	0.09836627	0.19902823
ENSG000001	56.867081	0.71410424	0.22676927	3.14903439	0.00163811	0.00805816
ENSG000001	576.525955	-0.291117	0.09269357	-3.1406383	0.0016858	0.00823128
ENSG000001	1395.36184	0.38434345	0.1268535	3.0298213	0.00244698	0.0110297
ENSG000001	2212.39696	-0.1410895	0.07379169	-1.9119973	0.05587654	0.13056183
ENSG000001	1658.61247	0.21447106	0.11466592	1.87039928	0.06142839	0.1406088
ENSG000001	2720.64882	-0.1216225	0.05546798	-2.1926624	0.02833171	0.07852335
ENSG000001	299.29261	1.18186651	0.30796323	3.83768709	0.0001242	0.00098869

ENSG000001	441.768097	0.55848047	0.12632191	4.42108953	9.82E-06	0.00012312
ENSG000001	6831.97638	0.94952961	0.34093156	2.7851033	0.00535107	0.02092332
ENSG000001	294.698294	0.56376578	0.13403196	4.20620405	2.60E-05	0.00027441
ENSG000001	876.929946	0.20069793	0.08397496	2.38997349	0.01684959	0.05266451
ENSG000001	1683.57242	-0.1821583	0.06243923	-2.917369	0.00352998	0.01487458
ENSG000001	5697.13587	0.32604665	0.08741938	3.72968388	0.00019172	0.00139012
ENSG000001	215.900665	0.33458379	0.14752072	2.26804616	0.02332639	0.0676942
ENSG000001	1406.18268	-0.3578922	0.05397043	-6.6312645	3.33E-11	2.98E-09
ENSG000001	61.3249321	1.54239213	0.297629	5.1822643	2.19E-07	5.09E-06
ENSG000001	2820.55124	1.81636617	0.32244764	5.63305768	1.77E-08	6.36E-07
ENSG000001	2035.06297	-0.252259	0.08254447	-3.0560368	0.00224284	0.01028878
ENSG000001	109.868858	0.1788407	0.09031067	1.98028308	0.04767173	0.1158176
ENSG000001	330.598908	0.23517017	0.07938441	2.96242269	0.00305228	0.01322322
ENSG000001	724.383662	0.25356661	0.05582922	4.54182568	5.58E-06	7.76E-05
ENSG000001	52128.9246	1.02200768	0.1937191	5.27571981	1.32E-07	3.36E-06
ENSG000001	46.7063004	-0.6490235	0.2141367	-3.0308839	0.00243839	0.01100123
ENSG000001	4433.63608	-0.1654803	0.06667211	-2.482002	0.01306465	0.04309372
ENSG000001	487.806818	-0.2482312	0.080794	-3.0723971	0.00212347	0.00986617
ENSG000001	1397.5812	-0.1114783	0.05533968	-2.0144362	0.04396376	0.1091896
ENSG000001	2882.98906	-0.3068055	0.08090915	-3.7919754	0.00014945	0.00114195
ENSG000001	157.052683	0.45060142	0.12666914	3.55731009	0.00037467	0.00241453
ENSG000001	10769.0698	-0.304813	0.06181386	-4.9311424	8.18E-07	1.52E-05
ENSG000001	8813.06279	0.55393904	0.10441272	5.30528299	1.12E-07	2.97E-06
ENSG000001	8197.3323	1.47412583	0.23100162	6.38145234	1.75E-10	1.34E-08
ENSG000001	552.553494	1.08621722	0.25194655	4.31130031	1.62E-05	0.00018627
ENSG000001	26.5966932	1.32536368	0.31184052	4.25013297	2.14E-05	0.00023271
ENSG000001	183.575019	0.51191371	0.17364029	2.94812741	0.00319705	0.01374761
ENSG000001	666.393168	0.28490281	0.05991603	4.75503484	1.98E-06	3.26E-05
ENSG000001	343.163518	0.37588281	0.16384834	2.2940898	0.02178534	0.06436962
ENSG000001	2251.4218	0.27196333	0.15252078	1.78312309	0.07456627	0.16200383
ENSG000001	558.89113	0.55539517	0.12663958	4.38563646	1.16E-05	0.00014002
ENSG000001	2474.62103	0.39137598	0.11954651	3.27383851	0.00106097	0.0056838
ENSG000001	70.5401926	0.37404246	0.13446439	2.78172138	0.00540714	0.02109411
ENSG000001	538.432429	0.15967963	0.09239976	1.72813905	0.0839633	0.17685029
ENSG000001	83.396589	0.53772425	0.14538574	3.69860377	0.00021679	0.00153876
ENSG000001	6597.4696	0.19805688	0.11461539	1.72801296	0.0839859	0.17687218
ENSG000001	318.464113	0.20115867	0.10597415	1.89818616	0.05767156	0.13380653
ENSG000001	1461.27698	-0.1462668	0.07346192	-1.9910565	0.04647468	0.11372951
ENSG000001	1079.31438	0.15636101	0.0680311	2.29837536	0.02154043	0.06386403
ENSG000001	347.767498	0.98032896	0.17469802	5.61156308	2.01E-08	7.12E-07
ENSG000001	20.1173007	-1.2890596	0.26745949	-4.8196444	1.44E-06	2.46E-05
ENSG000001	174.093367	-0.8625568	0.17573279	-4.908343	9.18E-07	1.69E-05
ENSG000001	2201.45848	0.43343481	0.10578788	4.09720663	4.18E-05	0.0004036

ENSG000001	8384.14151	0.58845724	0.15981685	3.68207253	0.00023135	0.00162143
ENSG000001	139.400998	-0.4201587	0.25052441	-1.6771168	0.09351964	0.1917284
ENSG000001	789.614877	0.14149959	0.08429157	1.67869219	0.09321205	0.19137797
ENSG000001	3078.69981	0.13439009	0.07068277	1.90131338	0.05726098	0.13306702
ENSG000001	90.2884451	0.54341093	0.18598589	2.92178576	0.00348031	0.01469948
ENSG000001	761.922567	0.12295479	0.05667482	2.16947812	0.0300464	0.08211323
ENSG000001	724.46062	-0.1320216	0.07506594	-1.7587419	0.07862135	0.16816589
ENSG000001	374.448423	-0.3020888	0.14589009	-2.0706602	0.03839056	0.09910265
ENSG000001	381.430058	0.38975516	0.19361607	2.01303108	0.04411136	0.10944362
ENSG000001	898.64941	0.63209429	0.12713728	4.9717464	6.64E-07	1.28E-05
ENSG000001	98.1476648	-0.1752771	0.07893357	-2.2205651	0.02638043	0.07434572
ENSG000001	573.112983	0.20894622	0.10445975	2.00025574	0.04547266	0.11201536
ENSG000001	883.017829	0.32116734	0.08643375	3.71576322	0.00020259	0.00145438
ENSG000001	116.71444	0.40600796	0.15374308	2.64082105	0.00827054	0.02986423
ENSG000001	2948.05365	-0.4077465	0.06274043	-6.4989424	8.09E-11	6.70E-09
ENSG000001	8893.04643	0.40425513	0.14974788	2.69957166	0.00694288	0.02586308
ENSG000001	1484.17254	-0.1719995	0.080774	-2.1293921	0.03322183	0.08871608
ENSG000001	400.340609	-0.15143	0.06237713	-2.4276529	0.01519688	0.04860689
ENSG000001	5211.88591	0.14085806	0.0792025	1.77845472	0.0753292	0.16317197
ENSG000001	3793.68593	0.14583069	0.07912926	1.84294264	0.06533739	0.14711575
ENSG000001	3878.96802	0.51253519	0.09222423	5.55748956	2.74E-08	9.31E-07
ENSG000001	267.511752	-0.2760207	0.0857463	-3.2190394	0.00128621	0.00660755
ENSG000001	288.452661	0.82365027	0.20178133	4.08189543	4.47E-05	0.00042687
ENSG000001	1433.81577	0.13434439	0.05490237	2.44696892	0.01440633	0.04667959
ENSG000001	512.651024	0.32535645	0.11178702	2.9105028	0.00360848	0.01513492
ENSG000001	78.2763364	0.62526151	0.17115967	3.65308908	0.0002591	0.00178575
ENSG000001	166.671299	-0.8434613	0.19191038	-4.3950794	1.11E-05	0.00013492
ENSG000001	931.195136	0.37641094	0.11001874	3.42133475	0.00062315	0.00366228
ENSG000001	3673.06181	0.24008806	0.14470149	1.65919544	0.09707642	0.19713289
ENSG000001	210.145403	-0.204304	0.10727474	-1.9044933	0.05684597	0.13233609
ENSG000001	9019.40515	0.41726061	0.15000315	2.78167906	0.00540785	0.02109411
ENSG000001	7622.1781	0.17042803	0.10087055	1.68957168	0.09110993	0.1881285
ENSG000001	1397.3637	0.4095666	0.10212223	4.01055276	6.06E-05	0.00054746
ENSG000001	626.11819	0.20766732	0.08560771	2.42580163	0.01527462	0.04877306
ENSG000001	81.6014678	0.29492056	0.08168843	3.61031011	0.00030583	0.00204646
ENSG000001	2712.56917	0.16470173	0.07709176	2.13643743	0.03264378	0.0874788
ENSG000001	586.344739	-0.3905344	0.07865169	-4.9653659	6.86E-07	1.32E-05
ENSG000001	234.393587	2.57923087	0.45629487	5.6525528	1.58E-08	5.81E-07
ENSG000001	2630.14253	0.32748666	0.15295335	2.14108846	0.03226691	0.08667786
ENSG000001	2653.74343	-0.2177521	0.10018828	-2.1734287	0.02974806	0.08141753
ENSG000001	206.468358	1.00685975	0.32528009	3.09536234	0.00196573	0.00928512
ENSG000001	189.30267	0.24056563	0.11638697	2.06694639	0.03873921	0.09984253
ENSG000001	472.113617	0.52569396	0.17744974	2.96249496	0.00305157	0.01322322

ENSG000001	866.66632	0.35766618	0.10927363	3.27312443	0.00106366	0.00569445
ENSG000001	10838.6441	0.36136546	0.15679759	2.30466199	0.02118551	0.06311105
ENSG000001	2824.69808	0.11924363	0.06094934	1.95643841	0.05041353	0.12060541
ENSG000001	68.0404997	-0.3408155	0.1373779	-2.4808612	0.01310654	0.04320237
ENSG000001	473.447373	0.46059436	0.11285696	4.08122233	4.48E-05	0.00042781
ENSG000001	4300.58738	-0.1444744	0.0690572	-2.0920978	0.03642977	0.09523233
ENSG000001	122.009441	-0.5435117	0.14189533	-3.8303707	0.00012795	0.0010107
ENSG000001	4667.14647	1.50917025	0.23086051	6.5371521	6.27E-11	5.31E-09
ENSG000001	6721.4828	0.61835373	0.20779887	2.97573183	0.0029229	0.01275425
ENSG000001	323.439004	-0.1763151	0.07924919	-2.2248193	0.02609337	0.07374105
ENSG000001	9738.00854	0.40739727	0.13277439	3.06834212	0.0021525	0.00997739
ENSG000001	2.20808998	1.40764754	0.49286978	2.85602326	0.00428984	0.01742273
ENSG000001	3815.18971	-0.2484544	0.07387334	-3.3632487	0.00077031	0.00435577
ENSG000001	625.537146	0.55187249	0.26971251	2.0461509	0.04074153	0.10340268
ENSG000001	499.680988	-0.3890278	0.12063112	-3.2249376	0.00126	0.00650986
ENSG000001	1015.11529	-0.3218887	0.12550146	-2.5648205	0.01032292	0.03575995
ENSG000001	27656.4106	0.32687933	0.11380477	2.87228157	0.0040752	0.01670554
ENSG000001	6283.89541	0.40356081	0.19449661	2.07489897	0.0379959	0.09846728
ENSG000001	86.0505339	0.82053698	0.20784558	3.94782015	7.89E-05	0.00068048
ENSG000001	809.819127	1.72287583	0.24188999	7.12255955	1.06E-12	1.41E-10
ENSG000001	41.1977062	-0.4289386	0.21460849	-1.9987028	0.04564052	0.11233337
ENSG000001	76.5549351	0.30399464	0.13665986	2.22446181	0.02611739	0.07376928
ENSG000001	3075.6233	-0.2915049	0.14432104	-2.0198364	0.04340036	0.1082211
ENSG000001	10582.8361	-0.2836136	0.11344718	-2.4999617	0.01242067	0.04136251
ENSG000001	1146.81519	-0.1170577	0.06273458	-1.8659201	0.06205254	0.14163465
ENSG000001	68.6655992	1.36946527	0.27863936	4.91483063	8.89E-07	1.64E-05
ENSG000001	838.754188	0.31467466	0.16531261	1.9035127	0.05697368	0.13256949
ENSG000001	26.632535	1.06709764	0.30105306	3.54455003	0.00039328	0.00251768
ENSG000001	216.773872	-0.3232365	0.1245513	-2.5952081	0.00945337	0.03334573
ENSG000001	382.227219	-0.2801749	0.11440028	-2.4490754	0.01432234	0.04645678
ENSG000001	769.779704	-0.3608368	0.08721891	-4.1371395	3.52E-05	0.0003499
ENSG000001	1062.90624	0.1698282	0.09177785	1.85042689	0.06425205	0.14541781
ENSG000001	38.1016317	1.43141642	0.29455389	4.85960792	1.18E-06	2.06E-05
ENSG000001	2086.87026	0.28520688	0.08374485	3.4056647	0.00066003	0.00382782
ENSG000001	327.889431	0.28381925	0.08611904	3.29566215	0.0009819	0.00533217
ENSG000001	5413.93064	0.27340113	0.07579044	3.60733015	0.00030936	0.00206437
ENSG000001	64.6079241	0.79121398	0.17559195	4.50598102	6.61E-06	8.90E-05
ENSG000001	1429.37022	0.16563195	0.07284611	2.27372387	0.02298259	0.06686319
ENSG000001	6899.1587	-0.2214221	0.04077192	-5.430749	5.61E-08	1.66E-06
ENSG000001	110.655699	-0.1500122	0.08240286	-1.8204734	0.06868694	0.15233247
ENSG000001	124.756128	0.78499517	0.19801745	3.9642726	7.36E-05	0.00064404
ENSG000001	278.282268	0.59845061	0.28376788	2.10894414	0.0349494	0.09232531
ENSG000001	2915.44428	0.59392913	0.11290468	5.26044736	1.44E-07	3.59E-06

ENSG000001	298.296945	0.51312882	0.09014374	5.69234031	1.25E-08	4.80E-07
ENSG000001	632.111992	-0.5399566	0.0734707	-7.3492778	1.99E-13	3.21E-11
ENSG000001	599.982328	-0.3264891	0.06394003	-5.1061768	3.29E-07	7.08E-06
ENSG000001	60.0272769	-0.3748474	0.15436764	-2.4282768	0.01517076	0.04855917
ENSG000001	1297.80432	0.15521546	0.06217216	2.49654296	0.01254105	0.04167978
ENSG000001	546.367021	-0.1997386	0.06826825	-2.9257911	0.00343582	0.01454549
ENSG000001	1946.81996	-0.1638819	0.06507735	-2.5182635	0.01179351	0.03975163
ENSG000001	1593.44102	-0.1360645	0.07842854	-1.7348845	0.08276126	0.17515886
ENSG000001	84.3268776	-0.2862771	0.1046117	-2.7365683	0.00620837	0.02355701
ENSG000001	930.769534	-0.5247307	0.2141191	-2.4506486	0.01425991	0.04633991
ENSG000001	3835.12209	-0.179055	0.08385969	-2.1351734	0.03274685	0.08770634
ENSG000001	1268.57161	-0.1742922	0.08666133	-2.0111873	0.04430568	0.10970028
ENSG000001	716.88646	-0.2100065	0.12216903	-1.7189829	0.08561748	0.1794474
ENSG000001	1333.97711	0.348907	0.13891822	2.51159993	0.01201853	0.04031312
ENSG000001	3050.39097	-0.1013616	0.04034379	-2.512447	0.01198971	0.04025918
ENSG000001	526.302823	-0.6602744	0.13234701	-4.9889635	6.07E-07	1.19E-05
ENSG000001	292.866153	-0.2810551	0.14533574	-1.933833	0.05313366	0.12557053
ENSG000001	1105.98994	0.36630896	0.10411724	3.51823553	0.00043443	0.0027292
ENSG000001	1807.3411	0.23299798	0.13125062	1.77521442	0.07586249	0.16393495
ENSG000001	112.39386	-0.8730711	0.14688586	-5.9438748	2.78E-09	1.34E-07
ENSG000001	35.9946104	1.15785748	0.26184096	4.42198763	9.78E-06	0.00012288
ENSG000001	780.38726	0.17806329	0.0673566	2.64359088	0.00820317	0.02968758
ENSG000001	8646.59155	6.84038772	0.58355104	11.7220042	9.83E-32	3.56E-28
ENSG000001	2979.41689	0.37023659	0.13395491	2.76388959	0.00571169	0.02205338
ENSG000001	1118.79552	0.26073279	0.11348956	2.29741656	0.02159502	0.06393767
ENSG000001	677.782058	0.3367051	0.1200191	2.80542921	0.00502496	0.019917
ENSG000001	2352.53389	0.1556041	0.07640671	2.03652399	0.04169777	0.10512975
ENSG000001	1625.15314	0.18181175	0.05965906	3.04751304	0.00230744	0.01051919
ENSG000001	72.6170602	-0.3022685	0.11964434	-2.5263915	0.0115241	0.03907082
ENSG000001	180.053986	-0.5617545	0.18475022	-3.0406162	0.00236095	0.01071523
ENSG000001	307.707988	-0.3884226	0.11847279	-3.2785806	0.00104331	0.00560622
ENSG000001	140.901209	0.43563347	0.15119773	2.88121702	0.00396143	0.01629912
ENSG000001	184.531093	0.24671342	0.13081798	1.88592895	0.05930453	0.13690345
ENSG000001	8703.70344	0.44418129	0.11506714	3.8601924	0.0001133	0.00091521
ENSG000001	449.913148	-0.2096989	0.09746849	-2.151453	0.03144046	0.08500896
ENSG000001	1981.07194	0.11852605	0.05751652	2.06073046	0.03932876	0.1009424
ENSG000001	198.167707	-0.9279659	0.20760814	-4.4697953	7.83E-06	0.00010191
ENSG000001	824.420541	-0.1921132	0.05769933	-3.3295569	0.00086984	0.00480603
ENSG000001	2057.46	-0.1416882	0.04986379	-2.8415054	0.00449011	0.01807899
ENSG000001	2623.61555	-0.1521248	0.06614937	-2.2997171	0.02146425	0.06368065
ENSG000001	1637.18498	-0.2240717	0.07293405	-3.0722511	0.00212451	0.00986783
ENSG000001	2843.85413	-0.2737066	0.08848504	-3.0932526	0.00197976	0.00934225
ENSG000001	1139.86893	0.554843	0.16458687	3.37112546	0.00074862	0.00425971

ENSG000001	2540.19978	-0.2211234	0.06948508	-3.182315	0.00146103	0.00734163
ENSG000001	2542.86765	0.24117214	0.09974514	2.41788372	0.01561106	0.04960153
ENSG000001	3424.18987	-0.0980285	0.05701969	-1.7192036	0.0855773	0.17938914
ENSG000001	967.069115	-0.1692132	0.09514409	-1.7784945	0.07532267	0.16317197
ENSG000001	210.466508	-0.2826101	0.14013838	-2.0166502	0.04373203	0.10876705
ENSG000001	2067.01661	-0.3442833	0.07845933	-4.3880472	1.14E-05	0.000139
ENSG000001	2114.72261	-0.7473738	0.13652914	-5.4740973	4.40E-08	1.38E-06
ENSG000001	4789.8052	-0.1717796	0.08270236	-2.0770823	0.03779397	0.09801669
ENSG000001	138.154801	0.30940903	0.0858664	3.60337725	0.00031411	0.00209122
ENSG000001	440.663287	-0.4309618	0.21044684	-2.0478417	0.04057551	0.10318017
ENSG000001	1205.77626	0.18359927	0.11066527	1.65905053	0.09710561	0.19716454
ENSG000001	127.235725	0.388601	0.19859116	1.95678899	0.05037228	0.12060541
ENSG000001	846.190946	-1.1772457	0.19241866	-6.118147	9.47E-10	5.27E-08
ENSG000001	1723.83717	0.1745152	0.10082499	1.73087251	0.0834745	0.17617936
ENSG000001	1979.43853	0.55807387	0.14347455	3.88970645	0.00010037	0.0008266
ENSG000001	527.613153	0.27488555	0.09039611	3.04090032	0.00235872	0.01070848
ENSG000001	79.53373	0.57059577	0.14664715	3.89094349	9.99E-05	0.00082325
ENSG000001	288.892509	0.1805488	0.08315414	2.17125447	0.02991194	0.08176119
ENSG000001	1808.07201	-0.121159	0.06116031	-1.9810074	0.04759044	0.11571714
ENSG000001	1260.73877	0.14934537	0.07454612	2.00339553	0.04513485	0.11144854
ENSG000001	758.500343	-0.1324297	0.07346713	-1.80257	0.07145577	0.15711912
ENSG000001	1424.89216	-0.1793857	0.06872057	-2.6103645	0.00904458	0.03219306
ENSG000001	1090.47086	0.37976286	0.1552949	2.44543033	0.01446794	0.04682686
ENSG000001	83.1493708	0.2908503	0.1054763	2.75749427	0.00582462	0.02240014
ENSG000001	15197.2959	1.16006216	0.29225614	3.96933377	7.21E-05	0.00063319
ENSG000001	1333.04565	-0.1426321	0.05022042	-2.8401221	0.00450963	0.01815253
ENSG000001	1459.3672	1.58843411	0.28908183	5.49475595	3.91E-08	1.27E-06
ENSG000001	145.379718	-0.1168438	0.0640675	-1.8237611	0.06818819	0.15148632
ENSG000001	160.819628	0.70525993	0.21451909	3.28763242	0.00101034	0.00546351
ENSG000001	968.361214	0.61436225	0.1609424	3.81728025	0.00013493	0.00105434
ENSG000001	297.225424	-0.3343512	0.11657689	-2.868074	0.00412979	0.01688156
ENSG000001	1796.41914	0.18096755	0.08789788	2.05883861	0.0395097	0.10130567
ENSG000001	3902.40031	-0.1426652	0.03454224	-4.1301684	3.62E-05	0.00035875
ENSG000001	787.71077	-0.3421235	0.10756804	-3.1805314	0.00147005	0.00737929
ENSG000001	133.183554	-0.2210945	0.0947007	-2.3346657	0.01956089	0.05930905
ENSG000001	1537.52589	-0.1871221	0.05295623	-3.5335241	0.00041006	0.00260207
ENSG000001	160.122595	-0.7414217	0.16196127	-4.5777714	4.70E-06	6.75E-05
ENSG000001	571.412737	-0.1869259	0.08582987	-2.1778658	0.02941603	0.08077143
ENSG000001	161.696548	-0.403028	0.15594511	-2.5844223	0.00975423	0.034174
ENSG000001	100278.063	1.03928799	0.12555247	8.27771861	1.26E-16	3.61E-14
ENSG000001	9979.08143	0.57685203	0.18489111	3.11995541	0.00180878	0.00871718
ENSG000001	165.679016	0.76395661	0.22676874	3.36887969	0.00075474	0.00428492
ENSG000001	5091.38563	0.28930159	0.1042876	2.77407475	0.0055359	0.02150671

ENSG000001	1178.00495	0.35334361	0.10652346	3.31704985	0.00090973	0.00500354
ENSG000001	6980.62687	0.43978486	0.09621642	4.5707881	4.86E-06	6.91E-05
ENSG000001	46.3189136	1.78447221	0.26932427	6.62573856	3.46E-11	3.05E-09
ENSG000001	1553.05893	-0.1165045	0.05611501	-2.0761739	0.03787787	0.09821671
ENSG000001	61.8149421	-0.6583365	0.31324068	-2.1016955	0.03557996	0.0935815
ENSG000001	576.322758	0.45206613	0.15513728	2.91397481	0.00356859	0.014998
ENSG000001	4917.13107	-0.1284432	0.06801528	-1.8884467	0.05896601	0.13619908
ENSG000001	277.420411	0.47698629	0.15232875	3.13129518	0.00174037	0.00844083
ENSG000001	556.050552	-0.5085851	0.09434649	-5.3906096	7.02E-08	2.01E-06
ENSG000001	8634.37855	0.14462558	0.06864207	2.10695237	0.03512171	0.09262847
ENSG000001	6066.76309	0.55956979	0.12812251	4.36745877	1.26E-05	0.00015025
ENSG000001	19947.2186	0.47651843	0.16365875	2.91165882	0.00359515	0.01509207
ENSG000001	589.738155	0.3446702	0.16573248	2.0796781	0.03755507	0.0975528
ENSG000001	25569.8222	-0.2249011	0.08828369	-2.5474816	0.01085036	0.03723096
ENSG000001	23.0145295	1.64281448	0.27154227	6.04994015	1.45E-09	7.50E-08
ENSG000001	1185.69727	-0.1630029	0.07816433	-2.0853872	0.03703417	0.09648749
ENSG000001	3683.90062	-0.1700962	0.0870358	-1.9543249	0.05066281	0.12107425
ENSG000001	4080.95813	-0.2467408	0.05525881	-4.4651846	8.00E-06	0.00010357
ENSG000001	9292.92063	0.19484183	0.06662042	2.92465636	0.00344837	0.01459436
ENSG000001	5614.04824	-0.3352136	0.0910308	-3.68242	0.00023103	0.00162001
ENSG000001	336.432777	-0.9798151	0.37416741	-2.6186544	0.00882773	0.03156933
ENSG000001	747.336999	0.50504546	0.10515713	4.80276955	1.56E-06	2.65E-05
ENSG000001	633.275105	-0.2245816	0.12349911	-1.818488	0.06898958	0.15289155
ENSG000001	808.326052	-0.2460449	0.05337044	-4.6101351	4.02E-06	5.89E-05
ENSG000001	374.483358	0.25533107	0.11728744	2.17696847	0.02948292	0.08087342
ENSG000001	1264.08157	0.20050669	0.0915756	2.18952082	0.02855901	0.07899027
ENSG000001	463.915301	0.54592158	0.1215984	4.48954566	7.14E-06	9.46E-05
ENSG000001	70.4633185	0.6485971	0.26592079	2.43906132	0.01472547	0.04740619
ENSG000001	8340.04056	-0.5871429	0.14328081	-4.0978473	4.17E-05	0.00040275
ENSG000001	768.751966	-0.2647933	0.10232157	-2.5878546	0.00965757	0.03388454
ENSG000001	884.96504	-0.1685284	0.08383254	-2.0102983	0.04439963	0.10991412
ENSG000001	187.52769	0.50552426	0.22584775	2.23834094	0.02519883	0.07193211
ENSG000001	581.598582	-0.1518716	0.07519061	-2.0198207	0.04340199	0.1082211
ENSG000001	509.033036	0.14511513	0.07730829	1.87709676	0.06050483	0.1388458
ENSG000001	115.110853	0.32154235	0.18897406	1.70151584	0.08884617	0.18447964
ENSG000001	687.347515	0.16703646	0.09315352	1.79313094	0.07295199	0.15957353
ENSG000001	7099.25139	0.41251547	0.1762572	2.34041775	0.01926218	0.05859958
ENSG000001	473.455645	1.66666355	0.26545021	6.27862955	3.42E-10	2.25E-08
ENSG000001	728.008883	-0.2088934	0.06895273	-3.0295166	0.00244945	0.01103397
ENSG000001	1130.54074	0.39376573	0.09548379	4.12390152	3.73E-05	0.00036611
ENSG000001	3613.44352	0.52544637	0.16290709	3.22543581	0.00125781	0.00650318
ENSG000001	6152.45442	-0.1437578	0.0711804	-2.0196263	0.04342217	0.10823416
ENSG000001	680.900768	-0.1667362	0.05542873	-3.0081195	0.0026287	0.01170671

ENSG000001	65.4180765	-0.608089	0.12393633	-4.906463	9.27E-07	1.70E-05
ENSG000001	826.727727	-0.116883	0.05168209	-2.2615759	0.02372362	0.0686133
ENSG000001	808.044014	0.21432296	0.10010013	2.14108585	0.03226712	0.08667786
ENSG000001	5527.94468	0.24806406	0.09754189	2.54315414	0.01098568	0.0375707
ENSG000001	2225.17945	0.67703661	0.27332354	2.47705195	0.01324726	0.04357699
ENSG000001	937.541057	-0.0852095	0.04609196	-1.8486847	0.06450335	0.14585089
ENSG000001	3276.43315	0.16720421	0.08804104	1.899162	0.05754318	0.13355143
ENSG000001	5857.07548	-0.2527543	0.11017208	-2.2941778	0.02178029	0.06436782
ENSG000001	48.1679353	0.69801155	0.2133979	3.27093917	0.00107191	0.00572805
ENSG000001	525.467256	-0.2330247	0.07711122	-3.0219303	0.00251168	0.01127573
ENSG000001	158.944209	0.78477914	0.23262983	3.37351043	0.00074216	0.00422962
ENSG000001	220.745261	0.10636916	0.0585011	1.81824201	0.06902715	0.15292802
ENSG000001	28557.3752	-0.3160096	0.15040924	-2.1009986	0.03564109	0.09372527
ENSG000001	1158.7404	0.46462046	0.18863192	2.46310621	0.01377391	0.04502316
ENSG000001	14042.6636	0.45268007	0.11212531	4.03726936	5.41E-05	0.00050026
ENSG000001	456.571038	0.19947392	0.09098734	2.19232599	0.02835598	0.07857556
ENSG000001	717.097713	0.27991504	0.09201065	3.0422027	0.00234854	0.01067229
ENSG000001	254.577313	0.32815267	0.07828143	4.19196041	2.77E-05	0.00028844
ENSG000001	308.88442	-0.6972138	0.19571641	-3.5623675	0.00036753	0.00237906
ENSG000001	68.9670116	1.29402591	0.30477155	4.24588813	2.18E-05	0.00023698
ENSG000001	1718.3028	0.18013697	0.07496753	2.40286666	0.01626712	0.05123081
ENSG000001	736.569858	-0.2055802	0.08934453	-2.3009828	0.0213926	0.06354616
ENSG000001	776.586832	-0.1862525	0.06857339	-2.716105	0.0066055	0.02479475
ENSG000001	1639.79718	-0.2432939	0.10687684	-2.2763948	0.02282239	0.06653813
ENSG000001	1379.44458	-0.1704013	0.09469329	-1.7995076	0.07193842	0.15795269
ENSG000001	85703.7133	-0.38602	0.09290032	-4.1552066	3.25E-05	0.00033017
ENSG000001	644.144451	-0.4627021	0.17068508	-2.7108528	0.00671104	0.02511614
ENSG000001	2063.00175	-0.2174592	0.09598323	-2.2655956	0.02347616	0.06803692
ENSG000001	2868.03328	0.79389675	0.20252609	3.91997279	8.86E-05	0.0007459
ENSG000001	135.365572	1.04737864	0.26244103	3.99091036	6.58E-05	0.00058463
ENSG000001	800.674402	-0.1594936	0.07656602	-2.0830861	0.03724337	0.09693582
ENSG000001	65.1361669	-0.2707248	0.1036227	-2.6126014	0.0089856	0.03203111
ENSG000001	444.69297	-0.1488022	0.06432154	-2.3134119	0.0207	0.06194608
ENSG000001	38.0099065	-0.951864	0.21535371	-4.4200025	9.87E-06	0.00012337
ENSG000001	1894.21379	-0.7706033	0.37904612	-2.0330067	0.04205184	0.10589345
ENSG000001	3852.38088	-0.2046143	0.09218973	-2.2194916	0.0264533	0.07448571
ENSG000001	3599.93992	-0.2148057	0.10931461	-1.9650228	0.0494116	0.11894746
ENSG000001	2545.67496	-0.3726203	0.12438937	-2.9955961	0.00273909	0.01208561
ENSG000001	287.273375	-0.1518759	0.08461773	-1.7948474	0.07267802	0.15911841
ENSG000001	20921.5306	0.39168561	0.12326251	3.17765404	0.00148472	0.00743231
ENSG000001	546.571635	0.23159274	0.09697997	2.3880471	0.01693817	0.05291854
ENSG000001	2137.28904	0.43413239	0.10622446	4.08693436	4.37E-05	0.00041937
ENSG000001	44.6974409	-0.6112831	0.27619888	-2.2131989	0.02688394	0.07550054

ENSG000001	2934.08288	-0.3130245	0.08267988	-3.7859816	0.0001531	0.00116391
ENSG000001	330.888755	0.95182692	0.22775653	4.17914209	2.93E-05	0.00030171
ENSG000001	1883.00697	-0.1186647	0.0719255	-1.6498286	0.09897799	0.19979018
ENSG000001	1768.26278	-0.2969865	0.0832351	-3.5680435	0.00035966	0.00233648
ENSG000001	92.6111278	0.32331789	0.13079576	2.47192953	0.0134386	0.04412625
ENSG000001	389.024768	-0.1479585	0.06682833	-2.2140083	0.02682821	0.0754268
ENSG000001	6428.81854	-0.2789029	0.08483961	-3.2874138	0.00101112	0.00546571
ENSG000001	2127.34459	-0.4231537	0.11855893	-3.5691424	0.00035815	0.00232954
ENSG000001	602.504974	-0.6315763	0.30738811	-2.0546542	0.03991242	0.102113
ENSG000001	5370.23172	0.51605136	0.17107021	3.0166057	0.00255622	0.01142962
ENSG000001	157.400215	-0.1351841	0.07706356	-1.7541894	0.07939805	0.16927735
ENSG000001	4732.46362	0.32370161	0.12310505	2.62947471	0.00855169	0.03071843
ENSG000001	42.8418318	-1.1049817	0.28412148	-3.8891171	0.00010061	0.0008272
ENSG000001	59268.566	0.44270765	0.14722184	3.00707858	0.00263772	0.01173966
ENSG000001	2264.3722	0.34299504	0.07107258	4.82598255	1.39E-06	2.39E-05
ENSG000001	21763.1475	0.71683531	0.15664513	4.57617351	4.74E-06	6.79E-05
ENSG000001	797.727926	1.41409862	0.18641487	7.58576074	3.31E-14	6.38E-12
ENSG000001	608.412161	0.33861054	0.18482933	1.83201739	0.06694883	0.14955863
ENSG000001	3177.07997	-0.4119725	0.16546129	-2.4898423	0.01277998	0.04231853
ENSG000001	508.359812	-0.2489915	0.10301759	-2.4169802	0.01564987	0.04970832
ENSG000001	3119.96183	2.06838325	0.32817075	6.30276549	2.92E-10	2.01E-08
ENSG000001	20878.0475	-0.1922388	0.05879153	-3.2698376	0.00107609	0.00574405
ENSG000001	26053.7843	0.33702486	0.13534677	2.49008421	0.01277128	0.0422994
ENSG000001	29.0691611	2.02468534	0.38358197	5.27836417	1.30E-07	3.34E-06
ENSG000001	2036.78267	-0.1941434	0.08163697	-2.3781307	0.01740066	0.05395619
ENSG000001	864.889129	-0.2467241	0.05731659	-4.3045851	1.67E-05	0.00019039
ENSG000001	3008.17315	-0.1759688	0.04003434	-4.3954468	1.11E-05	0.0001348
ENSG000001	7690.89244	0.21794553	0.12323141	1.76858743	0.07696275	0.16562437
ENSG000001	370.82207	-0.2851089	0.09132314	-3.1219789	0.0017964	0.00867481
ENSG000001	2754.30601	0.12610597	0.07296753	1.72824763	0.08394384	0.17685029
ENSG000001	3229.24815	0.16543682	0.06216177	2.66139193	0.00778183	0.02840768
ENSG000001	3652.6998	-0.3174013	0.05619112	-5.6486032	1.62E-08	5.91E-07
ENSG000001	395.374552	0.34766485	0.2016627	1.72399179	0.08470932	0.17798172
ENSG000001	414.02894	0.49035939	0.1114629	4.3993057	1.09E-05	0.00013325
ENSG000001	177.625231	-0.5605417	0.16244569	-3.4506409	0.00055926	0.00334377
ENSG000001	881.658805	-0.1995535	0.12081369	-1.6517456	0.09858641	0.19933306
ENSG000001	1852.96293	-0.2719335	0.06011767	-4.523355	6.09E-06	8.30E-05
ENSG000001	3710.8878	0.38381259	0.08831167	4.3461142	1.39E-05	0.00016281
ENSG000001	412.817903	-0.1735339	0.07659592	-2.2655759	0.02347736	0.06803692
ENSG000001	1012.262	0.16518645	0.05758487	2.86857381	0.00412327	0.01686443
ENSG000001	25.7497399	0.78126406	0.25734075	3.03591271	0.00239809	0.01084398
ENSG000001	11919.9693	0.2012233	0.12173076	1.65301943	0.09832691	0.19900279
ENSG000001	4710.32573	0.13424903	0.06446989	2.08235252	0.03731028	0.09704435

ENSG000001	274.877724	-0.2306003	0.13750872	-1.676987	0.09354503	0.19173554
ENSG000001	6861.53293	0.77823976	0.16261094	4.78590035	1.70E-06	2.85E-05
ENSG000001	1690.03022	-0.2182825	0.07781338	-2.8052056	0.00502845	0.01992537
ENSG000001	7722.19577	0.46607892	0.12284579	3.79401621	0.00014823	0.0011338
ENSG000001	3124.32375	-0.2025738	0.10280283	-1.9705083	0.04878014	0.11779928
ENSG000001	698.056785	-0.186214	0.06154995	-3.0254117	0.00248295	0.01116402
ENSG000001	1561.55543	0.26806656	0.07222983	3.71130013	0.0002062	0.00147442
ENSG000001	4078.94084	-0.4269521	0.1004061	-4.2522525	2.12E-05	0.00023069
ENSG000001	13121.4822	0.34344428	0.16510664	2.08013604	0.03751306	0.0974976
ENSG000001	7169.19716	-0.2817398	0.06690525	-4.2110265	2.54E-05	0.0002698
ENSG000001	573.089778	0.28849199	0.1290834	2.23492701	0.02542214	0.07232461
ENSG000001	205.435657	0.32475921	0.0967965	3.35507192	0.00079344	0.00447087
ENSG000001	64.8944448	-0.4897629	0.2228042	-2.1981762	0.02793655	0.07767542
ENSG000001	240.298516	-0.3031386	0.07681468	-3.9463633	7.93E-05	0.00068341
ENSG000001	693.472501	-0.1985221	0.06224898	-3.1891619	0.00142686	0.00721751
ENSG000001	6391.15305	0.26179428	0.10017012	2.61349679	0.00896209	0.03196303
ENSG000001	25133.7351	0.27789345	0.15324848	1.81335215	0.06977752	0.15428373
ENSG000001	791.052343	-0.1000612	0.04942756	-2.0244005	0.04292896	0.1075042
ENSG000001	678.792468	0.45591949	0.14805441	3.0794051	0.00207414	0.00967734
ENSG000001	375.117966	-0.6951663	0.18176616	-3.824509	0.00013103	0.00103055
ENSG000001	868.881347	0.48452087	0.14034949	3.45224522	0.00055594	0.0033267
ENSG000001	169.20964	-0.2455546	0.13408221	-1.8313733	0.06704484	0.14968079
ENSG000001	998.08163	-0.1596138	0.07336297	-2.1756731	0.02957971	0.08102319
ENSG000001	26471.647	-0.3521568	0.14586892	-2.4142005	0.01576978	0.04992501
ENSG000001	1044.25348	0.27169812	0.09484793	2.86456549	0.00417582	0.01703246
ENSG000001	3869.03133	-0.2594476	0.06255889	-4.1472537	3.36E-05	0.00033876
ENSG000001	1321.56541	-0.4704809	0.14233258	-3.3055038	0.00094806	0.00517503
ENSG000001	2445.61035	-0.3418981	0.06183207	-5.5294623	3.21E-08	1.07E-06
ENSG000001	584.84667	-0.6175977	0.22795871	-2.7092525	0.0067435	0.02523065
ENSG000001	9570.20997	0.33553383	0.16270826	2.06218075	0.03919053	0.10070117
ENSG000001	129.103894	-0.4224802	0.12942904	-3.264184	0.0010978	0.00583412
ENSG000001	358.996918	-0.1891645	0.08254682	-2.2916025	0.02192859	0.06458213
ENSG000001	994.540774	-0.3858736	0.08622922	-4.4749751	7.64E-06	9.97E-05
ENSG000001	836.043004	-0.1927007	0.10223561	-1.8848683	0.05944762	0.13711378
ENSG000001	479.903939	-0.2197792	0.08912566	-2.4659473	0.01366514	0.04471808
ENSG000001	585.726201	-0.5672518	0.20240417	-2.8025698	0.00506972	0.02004506
ENSG000001	663.954673	0.13861306	0.05537225	2.50329443	0.01230431	0.04107203
ENSG000001	1071.23134	-0.2438392	0.07074092	-3.4469331	0.00056699	0.00338163
ENSG000001	483.585835	-0.1783352	0.06165444	-2.8924955	0.00382195	0.01584675
ENSG000001	5379.46401	0.18127997	0.06570291	2.75908587	0.00579633	0.02231502
ENSG000001	639.761036	-0.3046847	0.07807385	-3.9025186	9.52E-05	0.00079305
ENSG000001	2890.62627	0.31471674	0.09439081	3.33418858	0.00085549	0.00473936
ENSG000001	2048.77539	0.70605624	0.15090523	4.67880547	2.89E-06	4.46E-05

ENSG000001	367.891756	1.49964121	0.33448555	4.483426	7.35E-06	9.66E-05
ENSG000001	5148.72312	0.20277544	0.12284469	1.65066509	0.09880698	0.19955621
ENSG000001	1973.66638	0.1470848	0.049426	2.97585868	0.00292169	0.01275281
ENSG000001	14640.1819	0.8611875	0.18151893	4.74433981	2.09E-06	3.41E-05
ENSG000001	1404.70484	-0.4848728	0.12541992	-3.8659955	0.00011064	0.00089843
ENSG000001	488.202323	0.61874692	0.19577981	3.16042256	0.0015754	0.00778938
ENSG000001	633.221212	0.5966299	0.11134285	5.358493	8.39E-08	2.32E-06
ENSG000001	1191.20569	0.2558573	0.07476172	3.42230342	0.00062093	0.00365073
ENSG000001	895.840357	0.59719405	0.13781071	4.3334372	1.47E-05	0.00017095
ENSG000001	88.2832983	-0.5064602	0.2891892	-1.7513109	0.07989237	0.17010593
ENSG000001	3309.43142	-0.2519333	0.07850723	-3.2090457	0.00133176	0.00680778
ENSG000001	3356.50128	-0.2510131	0.07466851	-3.3616997	0.00077464	0.00437515
ENSG000001	574.249552	-0.5929314	0.07813619	-7.588435	3.24E-14	6.34E-12
ENSG000001	183.25503	-0.2036464	0.09952022	-2.0462813	0.0407287	0.10338824
ENSG000001	850.289479	0.41797784	0.17716247	2.35929116	0.01830988	0.05634139
ENSG000001	1022.73373	0.11063012	0.05932552	1.86479806	0.06220972	0.14188164
ENSG000001	25.193346	4.56340426	0.83624434	5.45702262	4.84E-08	1.49E-06
ENSG000001	78705.3955	0.41577196	0.18540273	2.24253416	0.02492687	0.07135261
ENSG000001	1336.67101	-0.139046	0.07365376	-1.8878333	0.05904833	0.13636747
ENSG000001	19190.0263	0.24994122	0.13048098	1.91553754	0.05542399	0.12967172
ENSG000001	9519.82349	0.18448267	0.08458276	2.18109075	0.0291767	0.08031216
ENSG000001	96.5179309	-0.9483543	0.24679544	-3.8426734	0.0001217	0.00097193
ENSG000001	3022.32792	0.7237011	0.14843017	4.87570074	1.08E-06	1.93E-05
ENSG000001	2323.27517	0.35245755	0.099828	3.53064824	0.00041454	0.00262477
ENSG000001	1264.35139	0.18498892	0.10211872	1.81150831	0.0700622	0.15483311
ENSG000001	963.512767	-0.3285781	0.08622606	-3.8106588	0.0001386	0.00107602
ENSG000001	265.571953	2.12769126	0.34526424	6.16250115	7.16E-10	4.19E-08
ENSG000001	696.177925	0.38456478	0.13290905	2.89344318	0.00381043	0.01582264
ENSG000001	50.662285	0.44054433	0.21079608	2.08990759	0.0366261	0.09563849
ENSG000001	97.4427853	0.48026154	0.16869402	2.84693884	0.00441418	0.01782779
ENSG000001	224.706061	0.58123904	0.19096836	3.0436406	0.00233734	0.01063142
ENSG000001	115.746746	0.54883757	0.20966123	2.61773513	0.00885155	0.03163888
ENSG000001	74.7951183	-0.4411238	0.17531192	-2.5162228	0.01186202	0.03993894
ENSG000001	474.76718	0.29338371	0.09016688	3.25378572	0.00113878	0.00601003
ENSG000001	3879.10546	-0.3952863	0.08388776	-4.7120847	2.45E-06	3.91E-05
ENSG000001	2651.62878	0.96115948	0.26508149	3.62590196	0.00028795	0.00195117
ENSG000001	550.193294	0.88965525	0.38017331	2.34013077	0.01927699	0.05863232
ENSG000001	1489.32036	-0.1754063	0.05965838	-2.9401784	0.00328023	0.01400552
ENSG000001	4875.55353	0.61703488	0.19776463	3.12004677	0.00180822	0.00871718
ENSG000001	564.135927	0.43876486	0.26204462	1.67438987	0.094054	0.19256081
ENSG000001	98.7305754	0.54882912	0.25447228	2.15673437	0.03102637	0.08409335
ENSG000001	133.597474	-0.4858319	0.2620999	-1.8536136	0.06379446	0.14460809
ENSG000001	10001.8096	0.56845896	0.18388026	3.09146264	0.00199173	0.00938569

ENSG000001	2608.07029	-0.3161657	0.15873593	-1.9917716	0.04639612	0.11359483
ENSG000001	7804.38834	0.24392698	0.10606104	2.29987352	0.02145539	0.06367343
ENSG000001	3813.27996	-0.2623874	0.10563681	-2.4838635	0.01299656	0.04289843
ENSG000001	2456.08485	0.52352389	0.16301149	3.2115766	0.00132009	0.00676481
ENSG000001	688.167544	-0.7794162	0.21857021	-3.5659765	0.0003625	0.00235181
ENSG000001	462.668967	0.36924815	0.08467963	4.3605309	1.30E-05	0.00015445
ENSG000001	52.340905	1.2595698	0.23431205	5.37560822	7.63E-08	2.14E-06
ENSG000001	159.253771	0.13679357	0.08061009	1.69697824	0.08970078	0.1857483
ENSG000001	618.850005	-0.2402809	0.08451751	-2.8429717	0.0044695	0.01800604
ENSG000001	787.164351	-0.1845334	0.08486974	-2.1743134	0.0296816	0.08125423
ENSG000001	4281.50277	0.33610176	0.09874415	3.40376374	0.00066464	0.00384839
ENSG000001	256.684238	1.17730726	0.2663528	4.42010467	9.87E-06	0.00012337
ENSG000001	109.839482	0.23470632	0.11995632	1.95659817	0.05039473	0.12060541
ENSG000001	18714.9612	0.17882734	0.06020859	2.97013014	0.00297674	0.01294626
ENSG000001	133.26043	1.08969258	0.24605532	4.42864866	9.48E-06	0.00011972
ENSG000001	3655.1304	-0.2600998	0.08637242	-3.0113759	0.00260067	0.01160328
ENSG000001	5037.91341	0.21007076	0.0646233	3.25069711	0.00115122	0.00606465
ENSG000001	12097.6796	-0.0957288	0.05261735	-1.8193399	0.0688596	0.15265019
ENSG000001	5070.69235	0.6423343	0.18346396	3.50114707	0.00046326	0.00288408
ENSG000001	231.224303	-0.1507984	0.08836649	-1.7065111	0.08791297	0.18301411
ENSG000001	15343.276	0.82456432	0.10777586	7.65073258	2.00E-14	4.14E-12
ENSG000001	4389.6599	0.48017357	0.24891899	1.92903554	0.05372645	0.12668214
ENSG000001	21323.933	0.36145651	0.16065545	2.24988638	0.02445616	0.07036671
ENSG000001	1520.92679	1.45709482	0.38974796	3.73855667	0.00018508	0.00135212
ENSG000001	2478.34192	0.22294001	0.08731192	2.55337411	0.01066848	0.03672869
ENSG000001	1664.91775	-0.2576424	0.08409681	-3.0636413	0.00218661	0.01010122
ENSG000001	622.646627	-0.1567839	0.07374035	-2.1261608	0.03348987	0.0893166
ENSG000001	408.942988	-0.2405906	0.06543716	-3.6766662	0.0002363	0.00165138
ENSG000001	647.94208	-0.2305824	0.08250901	-2.7946335	0.00519586	0.0204267
ENSG000001	150.214708	0.41765704	0.14184789	2.9444007	0.00323581	0.01386353
ENSG000001	8714.61377	0.27335548	0.15033971	1.81825205	0.06902562	0.15292802
ENSG000001	1640.40034	-0.4201281	0.10738871	-3.9122181	9.15E-05	0.00076538
ENSG000001	15164.7455	-0.1302016	0.07208631	-1.8061899	0.07088869	0.15621606
ENSG000001	958.323173	-0.232481	0.12554785	-1.8517325	0.06406424	0.14512879
ENSG000001	4027.68419	-0.2410561	0.07329208	-3.2889786	0.00100552	0.00544558
ENSG000001	328.351109	0.21576211	0.10285712	2.09768767	0.03593274	0.09428685
ENSG000001	241.959706	-0.4068917	0.21851347	-1.86209	0.06259041	0.1424807
ENSG000001	390.580556	-0.2876184	0.0653372	-4.4020623	1.07E-05	0.00013187
ENSG000001	99.5265231	-0.6055227	0.27807419	-2.1775581	0.02943895	0.08081904
ENSG000001	2271.49147	0.25519763	0.07253946	3.51805276	0.00043473	0.0027299
ENSG000001	6053.50006	-0.1442194	0.04297951	-3.3555391	0.0007921	0.00446507
ENSG000001	1030.0486	-0.3148953	0.06737068	-4.6740709	2.95E-06	4.54E-05
ENSG000001	3023.39826	0.28053646	0.11569457	2.42480242	0.01531672	0.04887518

ENSG000001	489.530417	0.34752902	0.08162949	4.25739557	2.07E-05	0.00022647
ENSG000001	62.6191225	0.82593559	0.2746126	3.00763914	0.00263286	0.01172163
ENSG000001	1458.32723	-0.1608945	0.06353186	-2.5325004	0.01132522	0.0384807
ENSG000001	979.874955	-0.3137709	0.05857289	-5.35693	8.46E-08	2.33E-06
ENSG000001	3352.78028	-0.2342245	0.09564028	-2.4490153	0.01432474	0.04645678
ENSG000001	261.672185	-0.139353	0.07737196	-1.8010787	0.07169047	0.15747989
ENSG000001	140.619649	-0.2849464	0.17039578	-1.6722623	0.09447262	0.1929271
ENSG000001	117.170442	0.25043072	0.0889644	2.81495435	0.00487842	0.01942378
ENSG000001	934.237723	0.36413162	0.09541697	3.81621463	0.00013551	0.001058
ENSG000001	126.916612	1.84725896	0.28458175	6.4911365	8.52E-11	6.96E-09
ENSG000001	1527.51898	0.38737463	0.13497805	2.86990831	0.00410591	0.01680342
ENSG000001	9905.27374	0.36000719	0.18487519	1.94729849	0.05149896	0.12257434
ENSG000001	6402.38259	-0.078917	0.04315834	-1.8285453	0.06746775	0.15033143
ENSG000001	831.499655	0.53187207	0.1490231	3.56905796	0.00035827	0.00232954
ENSG000001	65.3702577	0.89359671	0.22255346	4.01520022	5.94E-05	0.00053914
ENSG000001	42.0202113	1.78814286	0.38080595	4.69567999	2.66E-06	4.14E-05
ENSG000001	252.572024	0.4148715	0.23283314	1.78184041	0.07477526	0.16228752
ENSG000001	695.934713	-0.2478951	0.0755353	-3.2818446	0.0010313	0.00554996
ENSG000001	73.4861366	0.21917053	0.10779727	2.03317324	0.04203502	0.10588791
ENSG000001	2179.23338	1.5201075	0.33146629	4.58600935	4.52E-06	6.51E-05
ENSG000001	72.1446436	0.35004418	0.11878585	2.94685074	0.00321028	0.01378154
ENSG000001	88.2440104	-0.3997081	0.20375266	-1.9617317	0.04979373	0.11958916
ENSG000001	376.944735	-0.6601754	0.34643935	-1.9056016	0.05670192	0.13208565
ENSG000001	516.134348	-0.1483206	0.07557855	-1.9624692	0.04970788	0.1194239
ENSG000001	103.445755	0.52179487	0.18760096	2.78140836	0.00541236	0.02110071
ENSG000001	2228.18309	-0.1622981	0.09400705	-1.7264463	0.08426714	0.17730982
ENSG000001	320.566464	0.47876481	0.15487026	3.0913928	0.0019922	0.00938569
ENSG000001	6688.82337	0.48162602	0.1987097	2.42376706	0.01536045	0.04898237
ENSG000001	4577.74647	0.16987444	0.05396791	3.14769322	0.00164564	0.00807876
ENSG000001	59.9822426	-0.8367834	0.34588277	-2.4192687	0.01555175	0.04947259
ENSG000001	8960.0057	0.24029296	0.08209846	2.92688772	0.00342372	0.01451126
ENSG000001	28241.6001	1.36516264	0.19034063	7.17220817	7.38E-13	1.06E-10
ENSG000001	500.545577	0.17654534	0.10530622	1.67649489	0.0936413	0.19190571
ENSG000001	1018.60148	-0.4854567	0.11329537	-4.2848766	1.83E-05	0.00020467
ENSG000001	160.847883	0.45550296	0.16403626	2.77684316	0.00548897	0.02134156
ENSG000001	227.88578	-0.1695978	0.07856297	-2.1587502	0.03086955	0.08376235
ENSG000001	437.375312	-0.3605615	0.10186607	-3.5395644	0.00040079	0.00255241
ENSG000001	770.287573	0.29690227	0.07625003	3.89379862	9.87E-05	0.00081649
ENSG000001	4445.54072	0.22609771	0.11016095	2.05243058	0.04012783	0.10250999
ENSG000001	17873.7367	0.25275062	0.10825449	2.33478198	0.01955481	0.05930303
ENSG000001	56.6849842	1.82030286	0.39053663	4.66102979	3.15E-06	4.79E-05
ENSG000001	6146.239	0.32169358	0.14706454	2.18743132	0.02871105	0.07931675
ENSG000001	2681.69413	0.58430264	0.19790438	2.95244921	0.00315264	0.01360914

ENSG000001	917.734041	-0.292556	0.10010845	-2.9223903	0.00347356	0.01467954
ENSG000001	412.143219	0.3036335	0.13528631	2.24437709	0.02480815	0.07111113
ENSG000001	417.58512	0.80713369	0.14875231	5.42602448	5.76E-08	1.70E-06
ENSG000001	8108.22211	0.18006447	0.10745239	1.67576048	0.09378512	0.19214609
ENSG000001	959.894388	0.29249053	0.17041935	1.71629885	0.08610736	0.18021292
ENSG000001	45.8666995	-0.4745183	0.15191533	-3.1235711	0.00178671	0.00863664
ENSG000001	1512.58692	-0.4608025	0.10057111	-4.5818578	4.61E-06	6.63E-05
ENSG000001	743.64581	0.18687746	0.06806318	2.74564695	0.00603917	0.02301144
ENSG000001	1100.83781	0.35156176	0.18372413	1.91353071	0.05568015	0.13018694
ENSG000001	444.834393	-0.1876441	0.11181684	-1.6781385	0.09332006	0.19149118
ENSG000001	1354.10494	-0.1386543	0.07046438	-1.9677224	0.04909999	0.11829562
ENSG000001	31.2399655	0.82896881	0.29252354	2.83385332	0.00459904	0.01846117
ENSG000001	11254.2722	0.49064474	0.14710578	3.33531933	0.00085201	0.00473099
ENSG000001	6108.86623	-0.3338571	0.11922883	-2.8001375	0.00510808	0.02015273
ENSG000001	346.265583	-0.3473572	0.15702229	-2.2121524	0.02695614	0.07559429
ENSG000001	23924.2244	0.35050209	0.18544521	1.89005746	0.05875028	0.13591748
ENSG000001	117171.736	0.34242521	0.19330401	1.77143357	0.07648863	0.16489446
ENSG000001	968.314622	-0.2279412	0.08275399	-2.7544437	0.0058792	0.02255614
ENSG000001	358.861399	-0.8658993	0.22682588	-3.8174627	0.00013483	0.00105413
ENSG000001	2024.42736	0.18875228	0.07733464	2.44072119	0.01465797	0.04727292
ENSG000001	1606.5977	-0.4119472	0.12689216	-3.2464352	0.0011686	0.00614269
ENSG000001	577.071312	0.52108972	0.15118876	3.44661675	0.00056765	0.00338419
ENSG000001	7701.59854	0.17902936	0.0863593	2.07307567	0.03816524	0.09873211
ENSG000001	2431.47978	0.15422574	0.08360205	1.84476026	0.06507242	0.14674722
ENSG000001	86.6660473	0.24074455	0.09280795	2.59400788	0.00948643	0.03343794
ENSG000001	301.157476	0.59669334	0.1333268	4.47541919	7.63E-06	9.96E-05
ENSG000001	705.035847	0.44640178	0.11110831	4.01771729	5.88E-05	0.00053442
ENSG000001	91.1506591	-0.413413	0.16592466	-2.4915706	0.01271797	0.04214208
ENSG000001	184.74712	1.11467791	0.22673217	4.91627599	8.82E-07	1.63E-05
ENSG000001	47.8550552	1.50200336	0.31979202	4.69681308	2.64E-06	4.13E-05
ENSG000001	432.839852	-0.3587674	0.14044843	-2.5544425	0.0106358	0.03665101
ENSG000001	1248.49949	0.19762367	0.07166387	2.75764711	0.0058219	0.02239561
ENSG000001	2428.94362	0.58791194	0.16544149	3.5535942	0.00038001	0.00244347
ENSG000001	48.337774	1.19794941	0.26481565	4.52371084	6.08E-06	8.30E-05
ENSG000001	219.261242	0.24157229	0.10333789	2.33769332	0.01940316	0.0589542
ENSG000001	311.744172	0.77731941	0.13466789	5.77212119	7.83E-09	3.27E-07
ENSG000001	4201.69527	-0.1783991	0.06094562	-2.9271857	0.00342045	0.01450585
ENSG000001	1959.95924	-0.1628569	0.0876921	-1.8571448	0.06329055	0.14371425
ENSG000001	36.0068412	0.81916288	0.26004733	3.15005302	0.00163241	0.00803285
ENSG000001	752.924731	0.31453668	0.09991257	3.14811906	0.00164325	0.00807247
ENSG000001	1884.71251	0.23572422	0.06011557	3.92118437	8.81E-05	0.00074259
ENSG000001	145.845677	-0.3137254	0.08317402	-3.7719163	0.000162	0.00121916
ENSG000001	3836.73862	-0.4190186	0.0946207	-4.4284027	9.49E-06	0.00011972

ENSG000001	1235.82023	-0.457915	0.11416428	-4.0110183	6.05E-05	0.00054706
ENSG000001	235.22787	0.46110454	0.14805899	3.11432986	0.00184363	0.00883809
ENSG000001	278.320724	-0.373787	0.12955945	-2.8850616	0.00391337	0.01615185
ENSG000001	79.4355536	-0.5025576	0.18877568	-2.6621946	0.0077633	0.02836492
ENSG000001	238.757763	-0.351601	0.19428055	-1.8097593	0.07033312	0.15529886
ENSG000001	16814.4587	0.25031476	0.12798061	1.95588039	0.05047925	0.1206953
ENSG000001	761.255621	-0.247448	0.09038419	-2.7377354	0.00618638	0.02349819
ENSG000001	69.3817356	0.80056834	0.21738373	3.68274274	0.00023074	0.00161874
ENSG000001	3090.8632	-0.1075043	0.04813555	-2.2333663	0.0255248	0.07256082
ENSG000001	105.846612	-0.57137	0.21755659	-2.6263053	0.00863173	0.03095246
ENSG000001	320.286954	0.33127962	0.11260633	2.94192711	0.00326177	0.01395006
ENSG000001	321.684904	-0.2798257	0.14472483	-1.9335016	0.05317444	0.12562592
ENSG000001	232.178303	-0.187364	0.11220964	-1.6697674	0.09496538	0.19375174
ENSG000001	27.0871282	0.41503335	0.23669183	1.7534756	0.0795204	0.16951326
ENSG000001	673.547666	1.70396147	0.29886574	5.7014279	1.19E-08	4.64E-07
ENSG000001	1014.31047	-0.2075474	0.09800599	-2.117701	0.0342004	0.09074381
ENSG000001	6057.06279	0.43520097	0.17406666	2.50019709	0.01241242	0.04136132
ENSG000001	3662.16205	0.69633421	0.21618107	3.22106934	0.00127713	0.00658712
ENSG000001	616.578784	0.52718388	0.18739407	2.81323678	0.00490455	0.01951449
ENSG000001	1073.25269	0.45805607	0.17814957	2.57118812	0.01013503	0.03525237
ENSG000001	1051.51719	-0.1674194	0.06232586	-2.6861943	0.00722711	0.02669533
ENSG000001	2850.84462	-0.3286766	0.08504341	-3.86481	0.00011118	0.00090129
ENSG000001	34.9050625	0.31978116	0.18510217	1.72759273	0.08406127	0.17695373
ENSG000001	276.626023	0.39251635	0.11834953	3.31658556	0.00091125	0.00500805
ENSG000001	205.366046	0.72341476	0.16832128	4.29782105	1.72E-05	0.00019476
ENSG000001	683.682559	-0.2499696	0.07495841	-3.3347766	0.00085368	0.00473404
ENSG000001	240.119049	-0.3826193	0.10450219	-3.6613522	0.00025089	0.00173676
ENSG000001	239.291745	-0.2164929	0.0857659	-2.5242301	0.0115952	0.03922925
ENSG000001	1606.25213	0.5218721	0.17024528	3.06541304	0.0021737	0.01005117
ENSG000001	7697.07186	-0.1647965	0.06799921	-2.4235061	0.01537149	0.04899789
ENSG000001	765.446203	-0.2059581	0.09460952	-2.1769275	0.02948597	0.08087342
ENSG000001	1584.44201	0.52853371	0.12048857	4.38658804	1.15E-05	0.00013959
ENSG000001	1304.02279	0.24394718	0.09588138	2.54426018	0.01095095	0.03747848
ENSG000001	2330.73225	0.33980572	0.17736521	1.91585329	0.05538377	0.12963638
ENSG000001	781.429033	0.96540012	0.17507398	5.51424091	3.50E-08	1.15E-06
ENSG000001	470.471069	1.21163471	0.22852039	5.30208583	1.14E-07	3.01E-06
ENSG000001	165.219811	0.3530165	0.11522416	3.06373692	0.00218591	0.01010122
ENSG000001	242.984738	0.71091304	0.1987205	3.57745192	0.00034696	0.00226619
ENSG000001	15592.6766	0.95108716	0.15146755	6.2791481	3.40E-10	2.25E-08
ENSG000001	637.634147	-0.5273957	0.2245425	-2.3487568	0.01883621	0.05768001
ENSG000001	133.410305	1.90072746	0.40458188	4.69800445	2.63E-06	4.12E-05
ENSG000001	24358.3937	0.62077186	0.1385205	4.48144401	7.41E-06	9.73E-05
ENSG000001	1096.41943	-0.183086	0.08417918	-2.1749563	0.02963339	0.08113759

ENSG000001	1490.7109	-0.1117099	0.05087384	-2.1958219	0.0281047	0.07798367
ENSG000001	223.556285	-0.1813439	0.10175947	-1.7820837	0.07473558	0.16228377
ENSG000001	126.860285	0.40428629	0.10661456	3.79203628	0.00014942	0.00114195
ENSG000001	1127.82988	-0.1298494	0.07142705	-1.8179301	0.06907481	0.15301021
ENSG000001	35.6622065	0.98963819	0.36702265	2.69639541	0.00700944	0.0260708
ENSG000001	19833.9846	-0.1477874	0.08233152	-1.7950281	0.07264922	0.1590794
ENSG000001	50.5450509	0.7374273	0.18400634	4.00761902	6.13E-05	0.00055327
ENSG000001	187.071819	1.62570255	0.24940728	6.51826417	7.11E-11	5.96E-09
ENSG000001	635.464748	0.29050123	0.13043002	2.22725739	0.02593008	0.0734604
ENSG000001	7983.28407	0.89439676	0.14453377	6.18815089	6.09E-10	3.61E-08
ENSG000001	234.090788	0.35835475	0.12836746	2.79163229	0.00524429	0.02059475
ENSG000001	6630.06234	0.53177899	0.14253579	3.73084541	0.00019084	0.00138511
ENSG000001	620.687572	-0.2968832	0.1481653	-2.0037295	0.04509904	0.11137911
ENSG000001	13867.1754	-0.2547367	0.10309557	-2.4708789	0.01347814	0.04422602
ENSG000001	3364.85669	-0.2385264	0.08248859	-2.8916292	0.0038325	0.01587903
ENSG000001	861.370769	0.28636285	0.08386819	3.41443958	0.00063913	0.00373478
ENSG000001	498.41174	0.22274227	0.10681305	2.0853469	0.03703782	0.09648749
ENSG000001	1931.40677	0.16093342	0.08633489	1.86406007	0.06231327	0.14202837
ENSG000001	1293.55206	-0.1571234	0.06402207	-2.4542075	0.01411955	0.0459353
ENSG000001	2845.3991	0.1821136	0.09385478	1.94037639	0.05233396	0.12410577
ENSG000001	220.581639	0.18927722	0.11317401	1.67244411	0.09443678	0.19290855
ENSG000001	2619.50856	-0.1013754	0.05917717	-1.7130834	0.08669719	0.18105382
ENSG000001	1078.65284	-0.1899866	0.08606771	-2.2074079	0.02728558	0.07625118
ENSG000001	327.896983	0.74533623	0.18277061	4.07798722	4.54E-05	0.00043211
ENSG000001	7254.74208	0.23655066	0.06679145	3.54163102	0.00039766	0.00253911
ENSG000001	396.815069	0.39585663	0.17258847	2.29364465	0.02181092	0.06440578
ENSG000001	143.298925	0.57144556	0.13000682	4.39550435	1.11E-05	0.0001348
ENSG000001	12554.8768	0.26766945	0.10885632	2.45892435	0.0139354	0.04544847
ENSG000001	8062.67929	0.34441275	0.12595261	2.73446301	0.00624821	0.02368957
ENSG000001	153.448455	0.39914298	0.13133904	3.03902761	0.00237343	0.01075368
ENSG000001	13437.998	-0.2975844	0.10963708	-2.7142681	0.00664224	0.02491613
ENSG000001	1351.39477	-0.2665849	0.11202375	-2.3797179	0.0173259	0.0537704
ENSG000001	257.979216	0.21911755	0.08065108	2.71685813	0.00659049	0.02476689
ENSG000001	8277.50881	0.23098917	0.12632874	1.82847686	0.06747802	0.15033143
ENSG000001	307.409123	-0.2794291	0.12348144	-2.2629242	0.02364036	0.0684135
ENSG000001	204.945542	0.44974681	0.19715895	2.28113819	0.02254027	0.06587712
ENSG000001	10988.1396	0.21063656	0.0656251	3.20969528	0.00132876	0.00679962
ENSG000001	316.279507	-0.4511585	0.10491207	-4.3003485	1.71E-05	0.000193
ENSG000001	2017.39504	0.15174913	0.0751288	2.019853	0.04339864	0.1082211
ENSG000001	17838.4275	0.30834655	0.1074178	2.87053479	0.00409778	0.01677913
ENSG000001	34777.897	0.57160637	0.13102968	4.36241904	1.29E-05	0.00015325
ENSG000001	3474.46563	-0.1573596	0.05153318	-3.0535596	0.00226144	0.01035444
ENSG000001	471.168536	-0.194457	0.08301771	-2.3423552	0.01916247	0.05840661

ENSG000001	968.224415	0.86894458	0.16281484	5.3370108	9.45E-08	2.55E-06
ENSG000001	8.68888468	1.65678457	0.71089092	2.33057494	0.01977578	0.05984788
ENSG000001	963.002126	-0.2461824	0.0817352	-3.0119514	0.00259574	0.01158488
ENSG000001	6531.78943	-0.3270526	0.08329539	-3.9264191	8.62E-05	0.0007295
ENSG000001	51.4069531	0.59256917	0.17883739	3.31345244	0.00092152	0.00504886
ENSG000001	925.623489	-0.1964685	0.07214365	-2.7232962	0.00646341	0.02434618
ENSG000001	3338.79511	0.31811311	0.14084477	2.25860796	0.02390778	0.06904945
ENSG000001	486.278628	0.20575665	0.08494939	2.42210859	0.01543074	0.04914159
ENSG000001	11024.4054	0.77945103	0.16830205	4.63126293	3.63E-06	5.39E-05
ENSG000001	561.352882	0.27658939	0.13668416	2.02356585	0.04301484	0.10757853
ENSG000001	549.012836	0.29186658	0.10038773	2.90739302	0.00364455	0.01527295
ENSG000001	1506.68789	0.28133762	0.12235189	2.29941378	0.02148145	0.06371864
ENSG000001	38880.434	1.60352275	0.21885053	7.32702235	2.35E-13	3.69E-11
ENSG000001	1893.69941	0.60888903	0.1391632	4.3753595	1.21E-05	0.00014611
ENSG000001	42.8481992	1.36112599	0.29182461	4.66419193	3.10E-06	4.73E-05
ENSG000001	5535.67209	0.63324012	0.12128428	5.22112294	1.78E-07	4.30E-06
ENSG000001	4370.27523	0.19768339	0.08699323	2.27239965	0.02306238	0.06706237
ENSG000001	76.377956	0.92858911	0.39380979	2.3579635	0.0183755	0.0564953
ENSG000001	154.846955	0.18656968	0.11025171	1.6922157	0.09060485	0.18721901
ENSG000001	76.2156444	0.73447084	0.22638741	3.24430959	0.00117736	0.00617715
ENSG000001	2899.9143	0.3402416	0.18211329	1.86829643	0.06172076	0.14112195
ENSG000001	236.832673	0.64422894	0.18642563	3.4556887	0.00054889	0.00329538
ENSG000001	447.49759	1.44008663	0.28370482	5.07600335	3.85E-07	8.07E-06
ENSG000001	597.28006	0.77142453	0.26093851	2.95634608	0.00311308	0.01345841
ENSG000001	214.061677	2.15181821	0.38822181	5.54275452	2.98E-08	1.00E-06
ENSG000001	690.935362	-0.2758958	0.07295545	-3.7817019	0.00015576	0.00117956
ENSG000001	362.118363	0.40791439	0.09969846	4.09148155	4.29E-05	0.00041232
ENSG000001	768.469445	0.24508926	0.0943818	2.59678522	0.00941007	0.03325781
ENSG000001	372.015078	-0.1490768	0.06020502	-2.476152	0.0132807	0.04367708
ENSG000001	25791.8626	0.258648	0.10090475	2.56328864	0.01036858	0.03590096
ENSG000001	988.247276	-0.2129793	0.0805602	-2.643729	0.00819983	0.02968289
ENSG000001	386.924759	-0.3510309	0.11135181	-3.1524488	0.00161907	0.00797806
ENSG000001	620.465549	-0.1794757	0.10222265	-1.7557334	0.07913395	0.16886338
ENSG000001	3829.59453	-0.299342	0.06505322	-4.6014933	4.19E-06	6.10E-05
ENSG000001	994.232647	-0.710318	0.12467553	-5.6973331	1.22E-08	4.72E-07
ENSG000001	574.907456	-0.3024494	0.09292674	-3.2547081	0.00113509	0.0059971
ENSG000001	150.591334	0.26530535	0.08143855	3.25773654	0.00112305	0.00594214
ENSG000001	1736.71229	0.24274432	0.06696176	3.62511843	0.00028883	0.00195344
ENSG000001	525.98461	-0.2319428	0.09094598	-2.5503353	0.01076194	0.03701523
ENSG000001	16264.4537	0.58919641	0.16462807	3.5789547	0.00034497	0.00225625
ENSG000001	10686.615	0.30992473	0.08779869	3.52994714	0.00041564	0.00262944
ENSG000001	253.746291	-0.2751677	0.12414329	-2.2165326	0.02665505	0.07498091
ENSG000001	202.807918	0.2246543	0.11187114	2.00815251	0.04462709	0.1103829

ENSG000001	5699.22982	0.52991089	0.22753442	2.32892632	0.01986297	0.06003648
ENSG000001	207.775949	-0.4591339	0.11438623	-4.0138918	5.97E-05	0.00054179
ENSG000001	732.676381	0.17392295	0.09736896	1.78622582	0.0740627	0.16107887
ENSG000001	271.802235	0.50788504	0.15038934	3.3771345	0.00073245	0.00418718
ENSG000001	893.486342	-0.2097113	0.06606002	-3.1745577	0.00150065	0.0074682
ENSG000001	1261.00682	0.58308259	0.12258899	4.7564025	1.97E-06	3.24E-05
ENSG000001	1063.14122	-0.209217	0.11729342	-1.7837062	0.07447142	0.16182203
ENSG000001	1113.52511	0.31706417	0.09983613	3.17584595	0.001494	0.00745304
ENSG000001	147.194319	0.55472826	0.16407395	3.38096496	0.00072232	0.00413605
ENSG000001	5620.34281	0.3080742	0.1451284	2.12276981	0.03377315	0.08989005
ENSG000001	6381.76359	-0.6422314	0.13793167	-4.6561562	3.22E-06	4.89E-05
ENSG000001	4066.14018	0.44966446	0.13642681	3.29601234	0.00098068	0.00532898
ENSG000001	2816.75829	0.50352537	0.29751164	1.69245605	0.09055905	0.18720448
ENSG000001	22509.9886	-0.1541213	0.08648858	-1.7819842	0.07475182	0.16228377
ENSG000001	434.864375	-0.2449803	0.07327306	-3.343388	0.00082762	0.00462746
ENSG000001	78346.184	1.4835373	0.1791471	8.28111276	1.22E-16	3.61E-14
ENSG000001	1173.07482	-0.4396906	0.22272721	-1.9741217	0.0483679	0.11707699
ENSG000001	379.507578	-0.2427294	0.10694511	-2.2696631	0.02322803	0.06748988
ENSG000001	519.158736	-0.18222	0.09732959	-1.8721955	0.06117957	0.14021648
ENSG000001	1473.31207	0.68075464	0.19576663	3.47737837	0.00050634	0.00309229
ENSG000001	335.817987	-0.430907	0.10243089	-4.2068069	2.59E-05	0.00027388
ENSG000001	762.886378	-0.3414259	0.06881905	-4.961212	7.01E-07	1.34E-05
ENSG000001	617.729561	0.39180613	0.1076446	3.63981216	0.00027284	0.00186661
ENSG000001	1250.32795	0.38493059	0.20018085	1.92291413	0.05449083	0.12800531
ENSG000001	1182.74679	-0.1380299	0.06592356	-2.0937866	0.036279	0.09502329
ENSG000001	1920.14478	-0.1681287	0.07839634	-2.1445984	0.03198497	0.08617561
ENSG000001	2698.26629	-0.421907	0.10448276	-4.0380538	5.39E-05	0.00049923
ENSG000001	1592.69336	0.4093599	0.13703737	2.98721354	0.00281533	0.01236304
ENSG000001	434.872455	-0.6862628	0.13887791	-4.9414828	7.75E-07	1.45E-05
ENSG000001	308.540709	0.42631474	0.17550264	2.4291072	0.01513606	0.04847958
ENSG000001	8983.16219	0.37531368	0.13984248	2.68383168	0.00727837	0.02684259
ENSG000001	7774.01968	0.49336857	0.16653727	2.96251153	0.0030514	0.01322322
ENSG000001	683.318355	0.27109174	0.08669472	3.12696932	0.00176618	0.00855743
ENSG000001	589.907827	0.49968846	0.16704105	2.99141111	0.00277691	0.01222028
ENSG000001	3751.42001	0.1615488	0.0570864	2.82989992	0.00465626	0.01865464
ENSG000001	2690.91496	-0.2967639	0.08521654	-3.4824683	0.00049681	0.00305271
ENSG000001	455.67522	-0.2109923	0.09213686	-2.2899879	0.02202202	0.06477827
ENSG000001	59.5477466	1.23998969	0.24777903	5.00441739	5.60E-07	1.11E-05
ENSG000001	264.24444	-0.2354358	0.07274983	-3.2362391	0.00121116	0.0063138
ENSG000001	53.4420419	1.10513668	0.25200517	4.38537307	1.16E-05	0.00014002
ENSG000001	3418.65382	-0.1273714	0.06155186	-2.0693339	0.03851477	0.09937016
ENSG000001	274.969521	0.23097072	0.11402488	2.02561697	0.04280405	0.10728413
ENSG000001	957.141333	0.18356848	0.0540307	3.39748482	0.00068008	0.00392056

ENSG000001	56.3164652	0.3204926	0.18230452	1.75800687	0.07874634	0.16829336
ENSG000001	446.73253	-0.3232227	0.11427394	-2.8284901	0.00467681	0.01872665
ENSG000001	232.518419	-0.2288969	0.09922042	-2.306953	0.02105744	0.06279522
ENSG000001	830.532454	0.21544403	0.10669808	2.01919322	0.04346714	0.10832762
ENSG000001	300.783045	-0.3002914	0.1377655	-2.1797286	0.02927758	0.08054393
ENSG000001	218.589642	-0.453133	0.09642681	-4.6992428	2.61E-06	4.10E-05
ENSG000001	4385.37079	0.24870534	0.09397536	2.6464952	0.00813307	0.02950018
ENSG000001	43.2918538	0.88342296	0.27751745	3.18330597	0.00145604	0.00731909
ENSG000001	38355.3995	0.4919353	0.16999085	2.89389282	0.00380498	0.01581261
ENSG000001	1249.55747	0.61262055	0.1063909	5.75820423	8.50E-09	3.49E-07
ENSG000001	3693.82578	0.16780324	0.09431208	1.77923387	0.07520143	0.1629683
ENSG000001	668.12451	0.16378922	0.09721074	1.68488814	0.09201015	0.1895281
ENSG000001	678.922193	0.27369898	0.14577414	1.87755501	0.06044207	0.13874571
ENSG000001	792.917969	0.30258749	0.08513207	3.55433032	0.00037894	0.00243772
ENSG000001	736.705281	0.19083166	0.11516639	1.65700828	0.0975178	0.1978628
ENSG000001	265.137985	-0.8063927	0.24909156	-3.2373346	0.00120652	0.00630095
ENSG000001	3240.3845	-0.4657158	0.1808473	-2.5751881	0.01001856	0.03490593
ENSG000001	1061.36651	-0.1412435	0.06533925	-2.1616943	0.03064174	0.08336281
ENSG000001	73.8350836	0.36101802	0.1400897	2.57704905	0.00996478	0.03476422
ENSG000001	3940.18765	0.80384751	0.22532051	3.56757364	0.0003603	0.00233962
ENSG000001	460.204561	0.10336141	0.05674959	1.82135966	0.0685522	0.1521084
ENSG000001	865.923073	-0.5043319	0.23531041	-2.1432623	0.03209204	0.08641587
ENSG000001	548.93729	-0.1966133	0.10134972	-1.9399493	0.05238585	0.12418815
ENSG000001	346.032432	0.60949453	0.20987407	2.90409645	0.00368315	0.01538574
ENSG000001	2020.92626	-0.3429261	0.12559002	-2.7305203	0.00632344	0.02391222
ENSG000001	3200.13896	0.23073772	0.1285062	1.79553765	0.07256808	0.15892574
ENSG000001	188.137135	0.8531484	0.17189605	4.96316472	6.94E-07	1.33E-05
ENSG000001	4898.44191	0.40560649	0.13177695	3.07797745	0.00208411	0.00971131
ENSG000001	79.4053411	-0.2332136	0.08645529	-2.697505	0.00698612	0.02599242
ENSG000001	814.719328	0.41299583	0.07825989	5.27723514	1.31E-07	3.34E-06
ENSG000001	16475.311	-0.2685824	0.12731471	-2.1095943	0.03489332	0.09226127
ENSG000001	3308.10156	-0.6951342	0.13377375	-5.1963424	2.03E-07	4.79E-06
ENSG000001	8178.66013	0.30556159	0.14325466	2.13299577	0.03292507	0.08805345
ENSG000001	818.897334	0.84795312	0.12890679	6.578033	4.77E-11	4.11E-09
ENSG000001	246.807827	-0.3058873	0.09048332	-3.3805937	0.00072329	0.00414001
ENSG000001	950.306829	-0.2023707	0.08497998	-2.381393	0.0172473	0.05359538
ENSG000001	1912.87713	-0.179647	0.09701168	-1.8518074	0.06405349	0.14512714
ENSG000001	7113.77514	-0.3250202	0.08801398	-3.6928249	0.00022178	0.00156802
ENSG000001	1531.50806	-0.3413908	0.15430004	-2.2125127	0.02693126	0.0755853
ENSG000001	411.557725	-0.1762277	0.07198835	-2.4480028	0.01436505	0.04656668
ENSG000001	117.416395	0.45118897	0.10297934	4.3813544	1.18E-05	0.00014227
ENSG000001	5499.79533	0.26075234	0.05603944	4.65301458	3.27E-06	4.94E-05
ENSG000001	311.51043	0.60676939	0.11162555	5.43575726	5.46E-08	1.63E-06

ENSG000001	2971.96882	0.12247569	0.04736529	2.58576911	0.0097162	0.03406334
ENSG000001	32.122086	-0.5379167	0.18865502	-2.8513245	0.00435375	0.01763781
ENSG000001	1716.22117	-0.1827719	0.03927522	-4.6536196	3.26E-06	4.93E-05
ENSG000001	3716.62389	-0.1052215	0.05175962	-2.0328879	0.04206385	0.10590528
ENSG000001	4291.0809	-0.3689702	0.08802537	-4.1916348	2.77E-05	0.00028854
ENSG000001	2399.52777	0.58105873	0.14017747	4.14516474	3.40E-05	0.00034115
ENSG000001	348.362382	-0.2771366	0.14884576	-1.8619047	0.06261652	0.14251777
ENSG000001	179.528644	1.30762988	0.23253975	5.62325313	1.87E-08	6.72E-07
ENSG000001	79.3963641	-0.1960349	0.11498988	-1.7048013	0.0882315	0.18349264
ENSG000001	5530.42694	0.23527732	0.1412912	1.66519445	0.09587397	0.19523843
ENSG000001	6465.15546	0.65889541	0.15861527	4.15404783	3.27E-05	0.00033126
ENSG000001	193.781186	0.33125097	0.14655184	2.26029895	0.0238027	0.06878711
ENSG000001	225.358079	-0.1192006	0.07002589	-1.7022359	0.08871116	0.18423409
ENSG000001	19845.4716	-0.3481708	0.11997315	-2.9020725	0.00370703	0.01546766
ENSG000001	4537.86226	0.22849245	0.122862	1.85974867	0.06292109	0.14305365
ENSG000001	559.120951	-0.3058136	0.08901652	-3.4354708	0.00059153	0.00350292
ENSG000001	519.119862	0.33128169	0.11671861	2.83829371	0.00453554	0.01824165
ENSG000001	837.701286	0.21776272	0.07847501	2.77493059	0.00552135	0.02146171
ENSG000001	3242.45622	0.12649758	0.06465529	1.95649249	0.05040717	0.12060541
ENSG000001	313.369792	1.15202289	0.2127763	5.41424436	6.15E-08	1.80E-06
ENSG000001	3246.37211	-0.289835	0.06904932	-4.1975063	2.70E-05	0.00028392
ENSG000001	71.1810187	-0.3458069	0.16514253	-2.0939907	0.03626081	0.09499283
ENSG000001	390.233206	0.27521311	0.13285808	2.07148196	0.03831378	0.09895922
ENSG000001	2953.60458	-0.1841293	0.09550314	-1.9279925	0.05385606	0.12682261
ENSG000001	1165.20145	0.21967858	0.12430039	1.76732011	0.07717463	0.16585505
ENSG000001	35.3013849	0.59589876	0.19185193	3.10603483	0.00189614	0.00903879
ENSG000001	44.8793627	0.83430888	0.24201919	3.44728396	0.00056625	0.00337863
ENSG000001	2645.27323	-0.2029888	0.09812917	-2.0685874	0.03858482	0.09949774
ENSG000001	4581.43505	0.37627654	0.13639034	2.75882106	0.00580103	0.02232718
ENSG000001	445.736699	-0.4523454	0.12767155	-3.5430404	0.00039554	0.00252918
ENSG000001	121.259718	-0.6841321	0.29117194	-2.349581	0.01879456	0.05760032
ENSG000001	75.1517932	-0.2762635	0.1279356	-2.159395	0.03081953	0.08365603
ENSG000001	302.814246	1.00851967	0.2889658	3.4901005	0.00048284	0.00298289
ENSG000001	2177.78934	-0.2951492	0.09046385	-3.2626206	0.00110387	0.00585933
ENSG000001	1698.01992	-0.2991406	0.06927957	-4.3178762	1.58E-05	0.00018127
ENSG000001	983.599079	-0.1589313	0.08899476	-1.7858506	0.07412345	0.16118679
ENSG000001	279.851757	0.34851982	0.08769824	3.97408015	7.07E-05	0.00062297
ENSG000001	11.4242605	0.87674681	0.33447724	2.62124504	0.00876093	0.0313459
ENSG000001	1102.77988	0.6271923	0.34399742	1.82324714	0.06826597	0.15157823
ENSG000001	138.88736	0.5840502	0.13432264	4.34811432	1.37E-05	0.00016147
ENSG000001	2578.10329	0.15341505	0.06637543	2.31132299	0.02081502	0.06217468
ENSG000001	61.3643591	-0.308165	0.16881128	-1.8254998	0.06792564	0.15111078
ENSG000001	451.565606	0.39756544	0.13407109	2.96533297	0.00302355	0.01311837

ENSG000001	789.785802	-0.1161234	0.06419217	-1.8089962	0.0704516	0.15548172
ENSG000001	528.812167	0.19997171	0.08836134	2.26311303	0.02362872	0.06839347
ENSG000001	81.8647976	-0.2962704	0.13865312	-2.136774	0.03261637	0.08743575
ENSG000001	442.251709	-0.1879825	0.09133308	-2.0582082	0.03957015	0.10144272
ENSG000001	19.8798741	1.15574931	0.30202979	3.82660704	0.00012992	0.00102403
ENSG000001	474.162712	0.45622144	0.07172092	6.36106506	2.00E-10	1.50E-08
ENSG000001	66.876521	-0.574222	0.2241044	-2.5622967	0.01039825	0.03597788
ENSG000001	3657.07605	-0.2245238	0.13518331	-1.6608842	0.0967367	0.19663597
ENSG000001	291.278024	-0.2035122	0.09396108	-2.1659197	0.03031732	0.08268975
ENSG000001	5386.264	0.1614367	0.07236122	2.23098377	0.0256822	0.07292941
ENSG000001	750.725355	-0.2501079	0.04783229	-5.228852	1.71E-07	4.17E-06
ENSG000001	2161.61671	-0.2129335	0.06719203	-3.1690287	0.00152949	0.00760129
ENSG000001	136.413592	-0.4114327	0.11548541	-3.5626376	0.00036715	0.00237768
ENSG000001	957.78896	-0.3391918	0.09678376	-3.5046358	0.00045723	0.002849
ENSG000001	523.775287	0.3774217	0.16509446	2.28609548	0.02224867	0.06528591
ENSG000001	48.6986075	-0.505561	0.22647692	-2.2322846	0.02559616	0.07273667
ENSG000001	303.218416	-0.560535	0.11975118	-4.6808304	2.86E-06	4.42E-05
ENSG000001	157.503946	0.1609553	0.07963102	2.02126374	0.04325247	0.10799699
ENSG000001	653.985269	0.45370654	0.1953418	2.32262898	0.02019909	0.06084929
ENSG000001	1256.62862	0.14797628	0.06209955	2.38288813	0.01717741	0.05346996
ENSG000001	1544.80792	-0.2991183	0.11019633	-2.7144129	0.00663934	0.0249117
ENSG000001	250.529881	0.31557626	0.17088187	1.84675099	0.06478324	0.1461861
ENSG000001	218.847156	0.21458	0.08682552	2.47139324	0.01345877	0.04418247
ENSG000001	142.862544	-0.5842639	0.284168	-2.0560509	0.03977761	0.10190244
ENSG000001	71.0970615	0.4573212	0.14339857	3.18916139	0.00142686	0.00721751
ENSG000001	2708.13786	0.16725201	0.08942632	1.87027722	0.06144533	0.14062029
ENSG000001	121.03325	1.14685022	0.23716731	4.83561672	1.33E-06	2.30E-05
ENSG000001	460.814109	0.09004856	0.04739334	1.90002563	0.05742976	0.13337366
ENSG000001	1098.73942	-0.3513568	0.07130935	-4.9272189	8.34E-07	1.54E-05
ENSG000001	58.2490281	0.39466607	0.20451636	1.92975309	0.05363744	0.12651344
ENSG000001	511.690767	-0.3081994	0.18420717	-1.673113	0.09430506	0.19281999
ENSG000001	1126.94888	-0.2967973	0.08692672	-3.4143393	0.00063937	0.00373478
ENSG000001	1633.33815	-0.3139265	0.07083309	-4.4319187	9.34E-06	0.00011827
ENSG000001	888.457018	0.24416294	0.07561379	3.22907966	0.00124189	0.00644388
ENSG000001	329.159789	0.16241175	0.06582833	2.46720133	0.01361738	0.04458191
ENSG000001	84.3736661	0.80553472	0.1976148	4.07628749	4.58E-05	0.00043442
ENSG000001	1122.63798	-0.212802	0.0928362	-2.2922305	0.02189235	0.0645357
ENSG000001	73.3091383	3.20428759	0.37288044	8.59333787	8.45E-18	3.40E-15
ENSG000001	123.288095	0.35344759	0.18274699	1.93408166	0.05310309	0.12553924
ENSG000001	19.3079722	1.36754659	0.32111609	4.25872951	2.06E-05	0.00022547
ENSG000001	473.953653	0.43861781	0.12392286	3.53944221	0.00040097	0.00255241
ENSG000001	84.6274167	-0.3084114	0.08337838	-3.698937	0.0002165	0.00153779
ENSG000001	182.437063	-0.4292519	0.14946143	-2.8719907	0.00407895	0.01670953

ENSG000001	467.396837	-0.210722	0.08875843	-2.3741067	0.01759147	0.05446625
ENSG000001	1011.18323	0.20520825	0.10455526	1.96267745	0.04968367	0.11941461
ENSG000001	23252.9064	1.86140411	0.23061364	8.07152647	6.94E-16	1.73E-13
ENSG000001	1696.49568	0.66668149	0.13110941	5.08492493	3.68E-07	7.79E-06
ENSG000001	75.3590287	0.32992151	0.17527458	1.88231242	0.05979361	0.13760604
ENSG000001	1166.50623	0.1783301	0.07303217	2.44180191	0.01461416	0.04721574
ENSG000001	5723.63825	1.00080868	0.2533443	3.9503895	7.80E-05	0.00067523
ENSG000001	66.9455398	-0.4068641	0.13783618	-2.9517945	0.00315933	0.01362585
ENSG000001	41576.3606	-0.3856722	0.13301553	-2.8994525	0.00373815	0.01557508
ENSG000001	42.9281791	0.72091748	0.21924992	3.28810829	0.00100863	0.00545836
ENSG000001	777.298047	0.32257363	0.14617742	2.20672681	0.02733315	0.07635468
ENSG000001	2489.04352	-0.0983869	0.04319877	-2.2775399	0.022754	0.06637882
ENSG000001	645.448	0.38447503	0.14132162	2.72056769	0.00651699	0.02452888
ENSG000001	33407.8859	0.38468399	0.15672307	2.45454601	0.01410626	0.0459127
ENSG000001	14069.5412	0.37596831	0.14379321	2.61464575	0.008932	0.03187142
ENSG000001	394.099491	0.35849193	0.2131936	1.68153232	0.09265956	0.19043256
ENSG000001	473.327383	-0.5888874	0.111686	-5.272706	1.34E-07	3.39E-06
ENSG000001	5565.61622	-0.4804084	0.06362321	-7.5508351	4.32E-14	8.24E-12
ENSG000001	85.2255641	0.18987472	0.09138752	2.07768747	0.03773815	0.09790699
ENSG000001	516.957956	0.23404448	0.10347449	2.2618568	0.02370625	0.06857677
ENSG000001	434.153106	0.15008714	0.05915678	2.53710803	0.01117725	0.03809994
ENSG000001	308.991891	0.23204914	0.08046461	2.88386593	0.00392826	0.01618564
ENSG000001	2616.63732	0.14490749	0.07296816	1.98590017	0.04704441	0.1148197
ENSG000001	387.124833	0.40291964	0.14834728	2.71605689	0.00660646	0.02479475
ENSG000001	3705.79656	-0.1807451	0.06266864	-2.884139	0.00392485	0.01617621
ENSG000001	1176.95291	-0.3453021	0.08579476	-4.0247461	5.70E-05	0.00052066
ENSG000001	6269.45591	1.40725214	0.18815756	7.47911547	7.48E-14	1.32E-11
ENSG000001	2839.71313	-0.3287593	0.09067279	-3.6257764	0.00028809	0.0019512
ENSG000001	920.611687	-0.3515038	0.16395867	-2.1438562	0.03204441	0.08630366
ENSG000001	673.822708	-0.3940503	0.09045862	-4.3561385	1.32E-05	0.00015675
ENSG000001	501.896055	-0.2553684	0.09293249	-2.7478916	0.00599798	0.02290269
ENSG000001	94.4445978	0.42007711	0.19052038	2.20489335	0.02746158	0.07662479
ENSG000001	23302.7263	0.189269	0.10817587	1.7496416	0.08018018	0.17064349
ENSG000001	196.593967	0.28512513	0.14338168	1.98857432	0.04674821	0.11424448
ENSG000001	6708.51086	-0.2148554	0.08833912	-2.4321655	0.01500885	0.0481793
ENSG000001	570.283196	0.16192081	0.0893578	1.81204998	0.06997847	0.15470443
ENSG000001	50.8027646	0.68395631	0.1614602	4.23606757	2.27E-05	0.00024574
ENSG000001	909.511275	-0.2166568	0.05672951	-3.8191197	0.00013393	0.00104934
ENSG000001	79.7569883	1.86125042	0.39594275	4.70080694	2.59E-06	4.08E-05
ENSG000001	635.518198	0.44618604	0.11502664	3.87898014	0.0001049	0.0008594
ENSG000001	145.59157	-0.2721072	0.12486749	-2.1791676	0.02931922	0.08061255
ENSG000001	66.7549278	-0.9681825	0.23328848	-4.1501514	3.32E-05	0.00033566
ENSG000001	1761.37162	-0.2990667	0.14302139	-2.0910626	0.03652245	0.09545386

ENSG000001	21330.0431	1.27435939	0.19757909	6.44986985	1.12E-10	8.91E-09
ENSG000001	1112.71838	0.23658459	0.07658516	3.08917005	0.00200717	0.00942832
ENSG000001	166.719233	-0.2991369	0.17312105	-1.7279062	0.08400505	0.17688679
ENSG000001	47.8482744	1.73827328	0.44898934	3.8715246	0.00010816	0.00088026
ENSG000001	1708.38165	-0.1616898	0.09562432	-1.6908859	0.09085859	0.18766302
ENSG000001	90.2161257	0.45038247	0.15045636	2.99344257	0.00275849	0.01215029
ENSG000001	1186.65794	0.5166285	0.23116762	2.23486531	0.02542619	0.07232461
ENSG000001	7855.21945	0.40046019	0.11626605	3.44434339	0.00057245	0.00340997
ENSG000001	785.855253	-0.1799124	0.09248423	-1.9453307	0.05173519	0.12292728
ENSG000001	28968.1427	0.42454896	0.09891688	4.29197709	1.77E-05	0.00019949
ENSG000001	53.7717557	-0.6941106	0.19546071	-3.5511516	0.00038355	0.00246298
ENSG000001	4559.52861	-0.2862412	0.07260351	-3.9425254	8.06E-05	0.00069198
ENSG000001	21.6373191	1.97717159	0.39682645	4.98245923	6.28E-07	1.22E-05
ENSG000001	751.191214	0.24790845	0.11380912	2.17828274	0.02938499	0.08074524
ENSG000001	1340.87855	-0.1083078	0.06155439	-1.7595459	0.07848483	0.16797307
ENSG000001	3148.59735	-0.3534334	0.0950032	-3.7202262	0.00019904	0.00143426
ENSG000001	201.623392	0.74091043	0.17710613	4.18342635	2.87E-05	0.00029678
ENSG000001	8320.72905	-0.1042201	0.06297514	-1.6549401	0.09793664	0.19840695
ENSG000001	1098.52303	0.30862297	0.08213566	3.75747851	0.00017163	0.00127758
ENSG000001	627.683722	-0.2561867	0.0868104	-2.9511065	0.00316638	0.01364611
ENSG000001	665.861112	2.54396751	0.34724876	7.32606644	2.37E-13	3.69E-11
ENSG000001	74.9510234	0.83195283	0.20614622	4.03574142	5.44E-05	0.0005021
ENSG000001	76.6791181	-0.3938061	0.1680392	-2.3435372	0.01910186	0.05827092
ENSG000001	3155.5871	0.58268153	0.27352671	2.13025462	0.0331506	0.08854217
ENSG000001	1899.85572	-0.3610639	0.09736349	-3.7084117	0.00020856	0.00149024
ENSG000001	7656.92928	0.39075107	0.12662243	3.08595471	0.002029	0.00949719
ENSG000001	964.808161	0.34512838	0.10691802	3.22797195	0.00124671	0.00645963
ENSG000001	1783.44059	-0.2456614	0.08650465	-2.8398629	0.00451329	0.01816224
ENSG000001	11089.434	-0.2033382	0.10595887	-1.91903	0.05498053	0.1289257
ENSG000001	1131.89572	-0.517136	0.11590791	-4.4616108	8.13E-06	0.00010513
ENSG000001	3345.64802	-0.2845053	0.1022022	-2.7837487	0.00537346	0.02098824
ENSG000001	1056.14922	0.26047879	0.12721098	2.04761253	0.04059798	0.10318297
ENSG000001	791.939408	0.67583256	0.24391446	2.77077695	0.00559227	0.02166762
ENSG000001	62.6181641	0.64000074	0.19537863	3.27569478	0.00105402	0.00565124
ENSG000001	7944.09791	0.13773702	0.06716253	2.05080167	0.04028627	0.10278889
ENSG000001	969.113219	-1.4664909	0.27119942	-5.4074263	6.39E-08	1.85E-06
ENSG000001	1296.44038	0.26198827	0.1022417	2.56244061	0.01039394	0.03597156
ENSG000001	95.4178782	0.62898687	0.21132361	2.97641547	0.00291639	0.01274092
ENSG000001	1149.08318	0.5305143	0.1082843	4.89927273	9.62E-07	1.75E-05
ENSG000001	951.165981	-0.4627005	0.0874637	-5.2902004	1.22E-07	3.17E-06
ENSG000001	32.5053118	0.42977338	0.21032244	2.04340243	0.04101262	0.1038905
ENSG000001	3184.02969	0.17747381	0.09896679	1.79326627	0.07293036	0.1595503
ENSG000001	17.3977661	1.24584361	0.26041892	4.7839981	1.72E-06	2.86E-05

ENSG000001	514.677079	-0.338881	0.14796775	-2.2902356	0.02200767	0.0647691
ENSG000001	295.347956	1.39725518	0.38559384	3.62364501	0.00029048	0.00196277
ENSG000001	259.164055	0.31195027	0.08875299	3.51481443	0.00044006	0.00275504
ENSG000001	430.241281	-0.3578324	0.11708705	-3.0561225	0.0022422	0.01028878
ENSG000001	1173.13323	-0.656696	0.27273129	-2.4078501	0.01604677	0.05064748
ENSG000001	165.629874	0.24767892	0.14314665	1.73024609	0.08358632	0.17631705
ENSG000001	435.988122	1.21225521	0.46632915	2.59956991	0.00933407	0.03304561
ENSG000001	4712.81299	0.47462519	0.14710749	3.22638364	0.00125365	0.00648631
ENSG000001	19563.1298	-0.3199884	0.18566874	-1.7234373	0.08480947	0.17811464
ENSG000001	2666.45036	-0.3704223	0.08667159	-4.2738601	1.92E-05	0.00021295
ENSG000001	186.912266	0.36298016	0.13672228	2.65487204	0.00793385	0.02887156
ENSG000001	160.936658	-0.3327294	0.15459733	-2.1522329	0.03137902	0.0848745
ENSG000001	151.669071	-0.4293452	0.17440417	-2.4617828	0.01382484	0.04512853
ENSG000001	189.081588	-0.1887176	0.08165441	-2.3111748	0.0208232	0.06218629
ENSG000001	353.279673	0.46722135	0.20415356	2.28857801	0.02210388	0.06497985
ENSG000001	295.506072	2.33286797	0.38360493	6.08143379	1.19E-09	6.37E-08
ENSG000001	386.425322	-0.1707614	0.07933324	-2.1524568	0.0313614	0.08484268
ENSG000001	412.099744	-0.4430905	0.20343251	-2.1780712	0.02940074	0.08074524
ENSG000001	4550.59653	0.15159734	0.07405955	2.04696552	0.04066147	0.10327493
ENSG000001	778.434624	-0.2075711	0.06832209	-3.0381256	0.00238055	0.01077382
ENSG000001	1190.08534	-0.5099312	0.0976792	-5.2204684	1.78E-07	4.31E-06
ENSG000001	1357.86075	0.23359811	0.12797378	1.82535915	0.06794685	0.15111237
ENSG000001	436.900761	-0.3264842	0.0968732	-3.3702217	0.00075108	0.00426868
ENSG000001	662.414959	0.35916007	0.13991798	2.56693287	0.01026025	0.03560244
ENSG000001	6055.39967	-0.2117154	0.12076356	-1.7531398	0.07957801	0.16958615
ENSG000001	959.109261	0.19570573	0.0578945	3.38038579	0.00072384	0.00414151
ENSG000001	58.2476153	0.77761148	0.24720113	3.14566308	0.00165711	0.00812403
ENSG000001	167.716195	-0.4930371	0.23598268	-2.0892937	0.03668129	0.09574809
ENSG000001	2056.38593	-0.1905078	0.06434895	-2.9605429	0.00307097	0.01329623
ENSG000001	658.765446	0.24510997	0.09990761	2.45336636	0.01415261	0.04603253
ENSG000001	485.059383	0.50079537	0.23706334	2.1124961	0.03464392	0.09175835
ENSG000001	2550.28352	-0.3736179	0.07832567	-4.7700568	1.84E-06	3.05E-05
ENSG000001	60.1089028	0.36116273	0.14792517	2.44152327	0.01462545	0.0472206
ENSG000001	1947.82077	0.10934981	0.06519652	1.677234	0.09349673	0.19171793
ENSG000001	231.095232	0.39073042	0.06924417	5.64279165	1.67E-08	6.06E-07
ENSG000001	276.380871	0.16770925	0.06344649	2.64331784	0.00820979	0.02970411
ENSG000001	835.368764	1.16595974	0.30468371	3.82678722	0.00012983	0.00102384
ENSG000001	50.4242015	1.80716671	0.39719898	4.54977684	5.37E-06	7.52E-05
ENSG000001	2661.08017	0.50690254	0.13065239	3.87977998	0.00010455	0.00085718
ENSG000001	2652.74023	0.36027283	0.17044505	2.1137183	0.03453933	0.09153861
ENSG000001	15407.3467	0.71654864	0.1762224	4.06616094	4.78E-05	0.00045107
ENSG000001	14253.8613	-0.4648158	0.20779416	-2.2369048	0.02529256	0.07208734
ENSG000001	1616.08744	0.53218279	0.18894923	2.81653859	0.00485442	0.01934341

ENSG000001	78.0899893	0.92237766	0.22309482	4.13446473	3.56E-05	0.00035316
ENSG000001	426.310061	-0.5478139	0.14959286	-3.6620321	0.00025022	0.00173361
ENSG000001	453.417431	-0.4889872	0.23185747	-2.1089991	0.03494466	0.09232531
ENSG000001	92.5811915	0.54085815	0.20650067	2.61915934	0.00881468	0.03153042
ENSG000001	4158.22118	0.27993405	0.12298709	2.27612549	0.0228385	0.06655831
ENSG000001	45.2586019	0.56994689	0.25638208	2.22303714	0.0262133	0.0739595
ENSG000001	1323.06544	0.18791006	0.11387246	1.65018006	0.09890611	0.19970075
ENSG000001	67.9735704	0.90747808	0.18137365	5.00336221	5.63E-07	1.12E-05
ENSG000001	4815.60587	0.18597512	0.1087594	1.70996831	0.08727173	0.18204544
ENSG000001	441.816813	-0.3406298	0.09096846	-3.7444827	0.00018077	0.00132932
ENSG000001	2850.02995	0.60523159	0.14483276	4.17883085	2.93E-05	0.00030191
ENSG000001	3816.39126	0.13806226	0.08016614	1.7222017	0.08503299	0.17845471
ENSG000001	2321.27717	-0.2373357	0.03985406	-5.955119	2.60E-09	1.27E-07
ENSG000001	715.345401	0.33904409	0.1140596	2.97251679	0.00295369	0.01286534
ENSG000001	267.876132	-0.2590855	0.11189606	-2.3154121	0.02059039	0.06170727
ENSG000001	713.7129	0.34869462	0.2023685	1.72306769	0.08487629	0.17821222
ENSG000001	92.3137796	0.35507276	0.1138215	3.11955782	0.00181123	0.00872605
ENSG000001	1496.51502	0.19762172	0.09044158	2.18507586	0.02888328	0.07970136
ENSG000001	822.269352	-0.1207088	0.06554356	-1.8416568	0.06552536	0.14728718
ENSG000001	333.607855	0.43692376	0.11602557	3.76575386	0.00016605	0.00124381
ENSG000001	51.0383854	0.77160711	0.19146102	4.03010028	5.58E-05	0.00051185
ENSG000001	3809.87362	-0.1478529	0.05338877	-2.7693624	0.00561661	0.0217503
ENSG000001	760.525046	-0.3180704	0.17654999	-1.8015884	0.07161019	0.1573274
ENSG000001	2712.82888	0.1971017	0.10700734	1.84194571	0.06548309	0.14722888
ENSG000001	720.399101	-0.4299104	0.08909888	-4.8250934	1.40E-06	2.40E-05
ENSG000001	70.4934617	0.74272775	0.25712887	2.88854282	0.00387031	0.01601978
ENSG000001	26072.106	0.32713902	0.15971278	2.04829577	0.04053103	0.10310325
ENSG000001	23736.9601	1.61069742	0.22863613	7.04480708	1.86E-12	2.28E-10
ENSG000001	73.9712935	0.2125634	0.1092187	1.94621812	0.05162854	0.12277458
ENSG000001	296.405334	0.40007174	0.23093285	1.7324159	0.08319954	0.17577829
ENSG000001	45.6179237	2.73271563	0.42899444	6.37004901	1.89E-10	1.42E-08
ENSG000001	1475.53986	-0.1063504	0.04629844	-2.297062	0.02161523	0.06397138
ENSG000001	2648.69065	0.33607747	0.11797067	2.84882213	0.00438814	0.01773744
ENSG000001	864.518737	-0.1683956	0.08355173	-2.0154647	0.04385598	0.10899667
ENSG000001	88.375163	0.42300354	0.14963921	2.82682286	0.00470123	0.01880883
ENSG000001	99.1631246	-0.320773	0.13666089	-2.3472189	0.01891414	0.05785666
ENSG000001	746.536994	0.1994118	0.1015977	1.9627591	0.04967417	0.11941461
ENSG000001	24073.2311	0.33675598	0.14720529	2.2876622	0.0221572	0.06508341
ENSG000001	72.4308741	0.75015727	0.23411851	3.20417758	0.00135449	0.00690689
ENSG000001	274.183454	-0.5434338	0.14928887	-3.6401495	0.00027248	0.00186639
ENSG000001	1523.94422	-0.2851912	0.09599322	-2.9709518	0.00296878	0.01291943
ENSG000001	1475.2874	0.19580878	0.06771673	2.89158652	0.00383302	0.01587903
ENSG000001	91.5485877	0.4780996	0.14066701	3.39880391	0.00067681	0.00390637

ENSG000001	437.27853	0.85501134	0.2270994	3.76492112	0.0001666	0.00124667
ENSG000001	1034.30616	-0.4319357	0.14176577	-3.0468264	0.00231271	0.01053925
ENSG000001	227.070935	0.92997719	0.23618079	3.9375649	8.23E-05	0.0007031
ENSG000001	353.847467	1.10800274	0.28904682	3.83329849	0.00012644	0.00100147
ENSG000001	2852.14651	0.27203913	0.08599612	3.16338854	0.00155944	0.00772099
ENSG000001	1656.96609	-0.1424604	0.07367475	-1.9336393	0.05315749	0.12560635
ENSG000001	418.898219	-0.3328938	0.10340367	-3.2193621	0.00128476	0.00660713
ENSG000001	184.321247	0.33289619	0.107389	3.09990965	0.0019358	0.00918267
ENSG000001	942.626667	-0.1324111	0.07391127	-1.7914876	0.07321508	0.15990757
ENSG000001	2542.79539	0.26163186	0.05502715	4.75459595	1.99E-06	3.26E-05
ENSG000001	803.075243	0.20132802	0.06337201	3.17692332	0.00148846	0.00744139
ENSG000001	270.211558	0.50179454	0.11479595	4.37118667	1.24E-05	0.00014856
ENSG000001	525.040747	0.34482496	0.20672769	1.66801534	0.09531268	0.19425926
ENSG000001	22937.8601	0.32359284	0.16519379	1.95886808	0.05012824	0.12017339
ENSG000001	121.597053	-0.1987176	0.08082907	-2.458492	0.01395219	0.04549299
ENSG000001	8992.15088	0.23179047	0.13397581	1.73009188	0.08361386	0.17633508
ENSG000001	67.6227422	-0.4970189	0.15353582	-3.2371528	0.00120729	0.00630221
ENSG000001	5616.79783	0.46812665	0.12238415	3.82505937	0.00013074	0.00102881
ENSG000001	366.796072	-0.3974468	0.10065345	-3.9486661	7.86E-05	0.00067889
ENSG000001	2216.08676	0.21272284	0.0891748	2.38545916	0.01705782	0.05320056
ENSG000001	154.148445	0.18406923	0.10781607	1.70725227	0.08777517	0.18279013
ENSG000001	35.225234	0.93475098	0.22577139	4.14025442	3.47E-05	0.00034661
ENSG000001	13834.7188	0.52748494	0.10003271	5.27312467	1.34E-07	3.39E-06
ENSG000001	479.893341	-0.3551777	0.1329162	-2.6721929	0.00753573	0.02761703
ENSG000001	166.347114	0.30400371	0.07922166	3.83738132	0.00012435	0.00098898
ENSG000001	211.100563	-0.834025	0.15802889	-5.2776744	1.31E-07	3.34E-06
ENSG000001	1125.13326	0.34203005	0.14253742	2.3995807	0.01641386	0.05156964
ENSG000001	2377.34898	-0.3947122	0.09501704	-4.1541204	3.27E-05	0.00033126
ENSG000001	7607.8479	-0.2515624	0.07279975	-3.4555385	0.00054919	0.00329561
ENSG000001	419.845719	-0.6895902	0.17190823	-4.0113854	6.04E-05	0.00054655
ENSG000001	539.832382	-0.1964128	0.09832405	-1.9976071	0.04575928	0.11247279
ENSG000001	73.8766827	0.87731352	0.25632675	3.42263734	0.00062017	0.00364773
ENSG000001	1068.25912	-0.1886301	0.07237004	-2.6064668	0.00914817	0.03251422
ENSG000001	264.843358	-0.343759	0.06719733	-5.1156646	3.13E-07	6.80E-06
ENSG000001	108.506779	0.25669261	0.14324708	1.79195707	0.07313984	0.15977572
ENSG000001	215.77803	0.48749546	0.21907372	2.22525763	0.02606395	0.07370455
ENSG000001	237.283572	0.41593856	0.13420819	3.09920396	0.00194041	0.00919518
ENSG000001	204.359607	-0.6215511	0.16627146	-3.7381708	0.00018536	0.00135351
ENSG000001	5741.0469	0.31817035	0.0535102	5.9459759	2.75E-09	1.33E-07
ENSG000001	4772.26944	0.42749685	0.20952296	2.04033415	0.04131706	0.10441777
ENSG000001	637.957352	0.41662304	0.14299288	2.91359288	0.00357296	0.01501201
ENSG000001	24669.1758	0.42342872	0.16041599	2.63956681	0.00830121	0.02996003
ENSG000001	1975.0018	-0.2932781	0.08169641	-3.5898526	0.00033086	0.00218258

ENSG000001	343.602774	-0.2572494	0.08579192	-2.9985267	0.00271288	0.01200047
ENSG000001	497.295808	-0.9812411	0.22907807	-4.2834355	1.84E-05	0.00020571
ENSG000001	6280.69554	-0.302539	0.09275365	-3.2617473	0.00110728	0.00587157
ENSG000001	8497.03686	-0.3592164	0.14714413	-2.4412557	0.01463629	0.04723365
ENSG000001	75.5583208	-0.7444676	0.20103772	-3.7031239	0.00021296	0.0015168
ENSG000001	2758.30586	-0.1820922	0.05617443	-3.2415497	0.00118882	0.00622196
ENSG000001	219.222038	-0.6168221	0.1575302	-3.9155799	9.02E-05	0.00075785
ENSG000001	459.394198	0.37289124	0.10977638	3.39682562	0.00068172	0.00392845
ENSG000001	3838.43855	-0.2803064	0.09503293	-2.9495709	0.00318216	0.01369968
ENSG000001	499.984514	-0.2165949	0.12697201	-1.7058474	0.0880365	0.18324494
ENSG000001	508.706265	-0.3453113	0.1121877	-3.0779786	0.0020841	0.00971131
ENSG000001	530.687439	-0.4981348	0.13206287	-3.7719516	0.00016198	0.00121916
ENSG000001	6580.45627	0.30087066	0.15422531	1.95085141	0.05107472	0.12175737
ENSG000001	48.6754719	-0.5327743	0.26951717	-1.976773	0.04806729	0.1165831
ENSG000001	1035.36814	-0.2108165	0.06959838	-3.0290432	0.0024533	0.01104784
ENSG000001	7625.92891	0.23041605	0.1287177	1.79008835	0.07343971	0.16023152
ENSG000001	768.325465	-0.1129939	0.05582901	-2.0239271	0.04297766	0.10755953
ENSG000001	535.198773	0.33870132	0.13045144	2.59637865	0.00942122	0.03327134
ENSG000001	19780.9038	0.45004981	0.22895551	1.96566491	0.04933733	0.11880816
ENSG000001	958.613831	0.34638258	0.10759692	3.21926096	0.00128521	0.00660713
ENSG000001	4811.62981	0.31533883	0.12692297	2.48448987	0.01297372	0.04284255
ENSG000001	10157.7883	-0.1682508	0.06748935	-2.4929979	0.01266696	0.04202112
ENSG000001	6122.76179	0.37763184	0.06994357	5.39909308	6.70E-08	1.93E-06
ENSG000001	7693.70686	0.4280744	0.1208938	3.54091271	0.00039875	0.00254365
ENSG000001	83.0719431	-0.543483	0.18474103	-2.9418642	0.00326243	0.01395006
ENSG000001	64.5360254	1.14743147	0.25244892	4.54520253	5.49E-06	7.65E-05
ENSG000001	800.937622	0.31076883	0.12945081	2.40067127	0.01636503	0.05143853
ENSG000001	2643.66075	1.10825138	0.24132201	4.59241726	4.38E-06	6.33E-05
ENSG000001	852.314459	-0.1910164	0.06416138	-2.9771249	0.00290965	0.01271558
ENSG000001	1988.66275	0.41875545	0.22950912	1.82456993	0.06806595	0.15126116
ENSG000001	6368.22119	-0.3334669	0.16502678	-2.0206837	0.04331252	0.10809104
ENSG000001	157.098595	0.30168567	0.12861701	2.3456124	0.01899585	0.05803308
ENSG000001	810.961019	-1.4996849	0.2793669	-5.3681551	7.95E-08	2.20E-06
ENSG000001	101.764712	0.27331592	0.12167735	2.24623492	0.02468896	0.07086765
ENSG000001	88.2272985	-0.3847812	0.13291513	-2.8949387	0.00379233	0.01576907
ENSG000001	195.828466	1.33570494	0.29980083	4.45530774	8.38E-06	0.00010759
ENSG000001	774.437374	-0.2843435	0.08324947	-3.4155589	0.00063651	0.00372271
ENSG000001	133.306996	-0.1686956	0.09778692	-1.7251345	0.08450323	0.177626
ENSG000001	660.358597	0.46202256	0.10600918	4.35832577	1.31E-05	0.0001555
ENSG000001	7903.51163	0.10819	0.06331719	1.70869885	0.08750675	0.18237811
ENSG000001	1826.87091	0.34734309	0.19646935	1.76792502	0.07707344	0.16576053
ENSG000001	1468.00253	-0.376691	0.07793732	-4.8332556	1.34E-06	2.32E-05
ENSG000001	171.489603	1.28873623	0.29585084	4.35603373	1.32E-05	0.00015675

ENSG000001	2856.67093	-0.5326243	0.17200841	-3.0965014	0.00195819	0.0092616
ENSG000001	2354.91144	0.26004994	0.09756895	2.66529417	0.0076921	0.02814738
ENSG000001	27.1072047	-0.6428597	0.27840906	-2.3090472	0.02094096	0.06248644
ENSG000001	2877.26393	-0.128252	0.07371656	-1.7397995	0.08189424	0.17357744
ENSG000001	47204.7845	1.42423402	0.20589198	6.91738456	4.60E-12	5.09E-10
ENSG000001	2709.17053	-0.2438757	0.06633424	-3.6764674	0.00023649	0.00165181
ENSG000001	352.998854	0.17886857	0.0974449	1.83558676	0.0664188	0.14875298
ENSG000001	166.850656	-0.3349431	0.19036796	-1.7594509	0.07850096	0.16798277
ENSG000001	28.9632227	-0.8470786	0.17940628	-4.7215662	2.34E-06	3.77E-05
ENSG000001	438.721761	0.18084512	0.0832562	2.17215209	0.02984419	0.0816191
ENSG000001	2509.10059	0.74662218	0.17331237	4.30795666	1.65E-05	0.00018781
ENSG000001	2167.50769	-0.440184	0.06959697	-6.3247582	2.54E-10	1.80E-08
ENSG000001	3018.77719	-0.8529878	0.21885476	-3.8975064	9.72E-05	0.0008064
ENSG000001	200.991984	-0.2101536	0.09039678	-2.3247912	0.02008313	0.06062603
ENSG000001	904.22862	0.34667267	0.08555306	4.05213668	5.08E-05	0.00047435
ENSG000001	272.929551	-0.5467957	0.10574875	-5.1707059	2.33E-07	5.37E-06
ENSG000001	1603.10202	-0.1918823	0.10115603	-1.8968943	0.0578419	0.1341158
ENSG000001	56.0663456	-0.3776521	0.18959234	-1.9919165	0.04638022	0.11357509
ENSG000001	266.715488	-1.2024799	0.27895083	-4.3107235	1.63E-05	0.00018632
ENSG000001	5889.28241	0.44359912	0.11752147	3.77462203	0.00016025	0.00120851
ENSG000001	9399.06643	0.14581759	0.08712707	1.67361987	0.09420533	0.19270723
ENSG000001	2124.34881	0.56763337	0.10025226	5.66205089	1.50E-08	5.56E-07
ENSG000001	35.4554923	0.45081187	0.21949603	2.05384979	0.03999023	0.10223783
ENSG000001	1420.32171	-0.3127004	0.09757983	-3.20456	0.00135269	0.00690015
ENSG000001	5471.2989	0.43754987	0.25225706	1.73453962	0.08282238	0.17518585
ENSG000001	99.3844868	0.1949354	0.10887376	1.79047183	0.07337809	0.160167
ENSG000001	461.855986	-0.6654589	0.21855201	-3.0448536	0.00232794	0.01059862
ENSG000001	150.356322	0.66216995	0.14039578	4.71645192	2.40E-06	3.85E-05
ENSG000001	42.32809	1.29486864	0.21234789	6.09786454	1.07E-09	5.87E-08
ENSG000001	975.321941	-0.3071238	0.17915555	-1.7142857	0.08647626	0.18069834
ENSG000001	1230.33093	-0.1246218	0.05346268	-2.3310058	0.01975306	0.05980408
ENSG000001	45.0393708	1.47017641	0.28251425	5.20390171	1.95E-07	4.61E-06
ENSG000001	1981.60011	-0.4361641	0.06301636	-6.9214418	4.47E-12	5.02E-10
ENSG000001	31.709793	1.42210178	0.34211042	4.15685023	3.23E-05	0.00032803
ENSG000001	2616.17598	-0.231324	0.09412994	-2.4574961	0.01399093	0.04559879
ENSG000001	634.348476	0.13572406	0.08000151	1.69651881	0.08978767	0.18588804
ENSG000001	220.876629	0.50210923	0.20172355	2.4890958	0.01280685	0.04238811
ENSG000001	96540.5476	-0.2177311	0.09948216	-2.1886447	0.02862268	0.07911786
ENSG000001	19166.6805	0.32658574	0.13976958	2.33660105	0.01945994	0.05910191
ENSG000001	117.228896	0.2813848	0.0916838	3.06907856	0.0021472	0.00995727
ENSG000001	1092.02474	0.38218688	0.11627251	3.28699271	0.00101263	0.00547185
ENSG000001	256.737726	-0.2284067	0.08369339	-2.729089	0.00635096	0.02398627
ENSG000001	1027.20639	0.35454401	0.0954942	3.71272802	0.00020504	0.00146758

ENSG000001	281.58765	-0.2187217	0.08537922	-2.5617676	0.0104141	0.03600693
ENSG000001	1450.13877	-0.2559812	0.06564253	-3.8996248	9.63E-05	0.00080029
ENSG000001	221.999749	0.43036779	0.09919179	4.33874418	1.43E-05	0.00016755
ENSG000001	49.081372	0.72553527	0.180661	4.01600375	5.92E-05	0.00053798
ENSG000001	1441.56728	0.14563559	0.08553714	1.7026007	0.08864282	0.18416299
ENSG000001	536.613643	-0.1846586	0.09179493	-2.0116422	0.04425766	0.10965636
ENSG000001	952.800826	0.20691217	0.0826545	2.5033382	0.01230279	0.04107203
ENSG000001	3055.65696	-0.1089149	0.05006056	-2.1756636	0.02958043	0.08102319
ENSG000001	1910.45733	-0.3267716	0.10452034	-3.1263922	0.00176965	0.00856851
ENSG000001	6959.44084	0.1514364	0.06024054	2.51386197	0.01194172	0.04013913
ENSG000001	1108.57414	-0.7604523	0.17674909	-4.3024398	1.69E-05	0.00019164
ENSG000001	913.990333	-0.4566538	0.13052452	-3.4986058	0.0004677	0.00290671
ENSG000001	40.2093901	1.13845592	0.58072239	1.96041336	0.0499475	0.1198194
ENSG000001	1965.06861	0.33273962	0.18934404	1.75732822	0.07886188	0.16840685
ENSG000001	803.082569	-0.2363662	0.11410533	-2.0714742	0.03831451	0.09895922
ENSG000001	6460.87308	0.14130493	0.07603503	1.85841865	0.06310959	0.14341468
ENSG000001	1192.44774	-0.2257452	0.1073776	-2.1023494	0.03552268	0.09351882
ENSG000001	841.186633	0.29049687	0.08543731	3.40011714	0.00067357	0.0038892
ENSG000001	1567.89519	-0.2107742	0.08300018	-2.5394426	0.01110293	0.03787334
ENSG000001	100.185146	0.31248992	0.13055911	2.39347458	0.01668964	0.052272
ENSG000001	293.453403	0.18121851	0.0819526	2.21125993	0.02701784	0.07572209
ENSG000001	67.5007647	-0.4181953	0.16729825	-2.4996994	0.01242987	0.04137068
ENSG000001	6640.17283	0.58499161	0.21390544	2.73481415	0.00624155	0.02367268
ENSG000001	974.726384	-0.4500511	0.10929274	-4.1178503	3.82E-05	0.00037459
ENSG000001	239.83945	0.45055826	0.23916805	1.88385643	0.0595844	0.13723983
ENSG000001	2509.29052	-0.3767236	0.19582839	-1.9237433	0.05438677	0.12778921
ENSG000001	216.281881	0.34244434	0.1036449	3.30401535	0.00095311	0.00519475
ENSG000001	29752.0156	-0.3256917	0.08302653	-3.9227429	8.75E-05	0.0007391
ENSG000001	56.5980096	1.45613564	0.27486305	5.29767698	1.17E-07	3.08E-06
ENSG000001	3775.62295	-0.1916518	0.09346768	-2.0504611	0.04031946	0.10279306
ENSG000001	3644.2322	0.48410126	0.19412408	2.49377235	0.01263936	0.04194877
ENSG000001	48.2577653	0.54689035	0.29829894	1.83336337	0.06674855	0.14924931
ENSG000001	1214.34288	-0.2654947	0.05402899	-4.9139311	8.93E-07	1.64E-05
ENSG000001	112.880045	0.15062524	0.08277208	1.81975905	0.06879571	0.15253189
ENSG000001	28252.1572	0.3774547	0.18371963	2.05451478	0.03992589	0.10211978
ENSG000001	2349.3815	-0.1182939	0.06080337	-1.9455151	0.05171302	0.12291493
ENSG000001	144.07313	0.31816909	0.0925805	3.43667482	0.0005889	0.00349362
ENSG000001	5840.56574	0.15159864	0.06546204	2.31582524	0.02056781	0.06165954
ENSG000001	399.915538	-0.1468649	0.05818203	-2.5242319	0.01159514	0.03922925
ENSG000001	11840.8531	0.26576492	0.09796366	2.71289292	0.00666987	0.02498096
ENSG000001	1605.35506	-0.1583661	0.07954865	-1.9908084	0.04650195	0.11377703
ENSG000001	3568.28024	-0.2521337	0.11856704	-2.1265072	0.03346105	0.08927261
ENSG000001	993.521222	0.66503458	0.15334804	4.33676615	1.45E-05	0.00016879

ENSG000001	92.1541064	-0.3634253	0.12958867	-2.8044528	0.00504021	0.01994153
ENSG000001	67.0855698	0.41734049	0.17058113	2.44658058	0.01442186	0.04670901
ENSG000001	260.438194	0.24589547	0.11402873	2.15643439	0.03104976	0.08412529
ENSG000001	297.806315	-0.2402043	0.12089933	-1.9868123	0.0469432	0.11466066
ENSG000001	1777.47295	4.3678098	0.41203878	10.6004823	2.96E-26	4.77E-23
ENSG000001	163.654429	0.61633968	0.13766554	4.47708028	7.57E-06	9.89E-05
ENSG000001	52.1135053	0.37374549	0.21935888	1.70380836	0.0884169	0.18377268
ENSG000001	688.855328	0.33288299	0.08036649	4.14206197	3.44E-05	0.00034508
ENSG000001	1067.83742	-0.5544136	0.07886935	-7.029519	2.07E-12	2.50E-10
ENSG000001	280.511638	0.23925128	0.10544732	2.26891767	0.02327333	0.06756729
ENSG000001	458.61006	-0.1946711	0.1157829	-1.6813461	0.0926957	0.1904798
ENSG000001	1723.86104	-0.2210155	0.0868129	-2.5458834	0.01090016	0.03737353
ENSG000001	1391.60796	-0.3997361	0.07742371	-5.1629675	2.43E-07	5.57E-06
ENSG000001	1260.27316	-0.4245056	0.08274619	-5.1302134	2.89E-07	6.37E-06
ENSG000001	573.326291	0.4159022	0.14374068	2.89342023	0.00381071	0.01582264
ENSG000001	1149.63224	-0.2206044	0.07780824	-2.8352319	0.00457924	0.01839514
ENSG000001	1213.84436	-0.612285	0.12230037	-5.0064035	5.55E-07	1.11E-05
ENSG000001	1034.127	-0.1703014	0.06826561	-2.4946887	0.01260677	0.0418502
ENSG000001	1273.7387	-0.2083607	0.10710512	-1.9453851	0.05172865	0.12292728
ENSG000001	23.0313062	1.6171418	0.39854722	4.05759146	4.96E-05	0.00046521
ENSG000001	856.769711	0.26157265	0.07461066	3.50583487	0.00045518	0.00283742
ENSG000001	267.794366	0.81843191	0.1840478	4.44684437	8.71E-06	0.00011132
ENSG000001	185287.309	0.53471108	0.21583129	2.47744925	0.01323252	0.0435384
ENSG000001	60634.133	0.18206152	0.10457476	1.74097005	0.08168883	0.17322214
ENSG000001	2284.39488	0.22491518	0.08391473	2.68028253	0.007356	0.02706793
ENSG000001	5846.17966	-0.3334202	0.09504571	-3.5079982	0.00045149	0.00281565
ENSG000001	587.663952	-0.229956	0.09544899	-2.409203	0.0159874	0.05048158
ENSG000001	353.812038	0.83572523	0.21921165	3.81241252	0.00013762	0.00107013
ENSG000001	12130.7571	0.42991625	0.15859112	2.7108469	0.00671116	0.02511614
ENSG000001	70.7516068	0.36864851	0.16019664	2.30122498	0.02137892	0.06351854
ENSG000001	4665.80203	0.49194575	0.13121329	3.74920682	0.00017739	0.0013119
ENSG000001	731.998399	1.18295687	0.22835981	5.18023231	2.22E-07	5.14E-06
ENSG000001	991.712005	1.27185653	0.22110907	5.75216798	8.81E-09	3.61E-07
ENSG000001	1621.52338	-0.1430614	0.06237046	-2.2937361	0.02180566	0.06440339
ENSG000001	733.561187	-0.3311331	0.10416168	-3.1790297	0.00147769	0.0074048
ENSG000001	346.494111	0.71003925	0.15814929	4.48967714	7.13E-06	9.46E-05
ENSG000001	2749.94179	0.21912719	0.06424753	3.41067112	0.00064803	0.00377442
ENSG000001	605.502837	-0.2582823	0.082748	-3.121312	0.00180047	0.00868868
ENSG000001	287.648484	-0.2143457	0.08326311	-2.5743175	0.01004381	0.03497707
ENSG000001	187.581594	0.56318536	0.15296117	3.68188451	0.00023152	0.00162185
ENSG000001	337.56909	-0.190834	0.08756634	-2.179308	0.0293088	0.08061255
ENSG000001	51.2322061	0.66291646	0.18953285	3.49763357	0.00046941	0.00291607
ENSG000001	1028.09774	0.52548509	0.14405168	3.64789279	0.0002644	0.00181706

ENSG000001	88.9806822	0.69362684	0.12701086	5.46116163	4.73E-08	1.46E-06
ENSG000001	4690.43464	1.91099088	0.26677746	7.16323971	7.88E-13	1.11E-10
ENSG000001	155.083831	-0.402547	0.12253603	-3.2851314	0.00101935	0.00549992
ENSG000001	16990.0196	0.31757203	0.11264502	2.81922841	0.00481392	0.01921194
ENSG000001	433.934952	0.54268886	0.12437976	4.36316068	1.28E-05	0.00015285
ENSG000001	173.157925	-0.2233422	0.12941356	-1.7258023	0.08438299	0.17747624
ENSG000001	11574.2289	-0.1731499	0.07133909	-2.4271398	0.01521839	0.04865788
ENSG000001	30085.9255	-0.2101911	0.12408588	-1.6939167	0.09028111	0.18678984
ENSG000001	1253.65178	0.25087791	0.06920827	3.62497037	0.00028899	0.00195365
ENSG000001	5663.90996	0.61125046	0.1188756	5.14193358	2.72E-07	6.08E-06
ENSG000001	497.928038	-0.1691385	0.09542281	-1.7725166	0.07630885	0.16470296
ENSG000001	38387.1977	3.90085818	0.38512464	10.12882	4.12E-24	3.98E-21
ENSG000001	4962.5417	0.12084199	0.06003677	2.0127996	0.04413572	0.10948247
ENSG000001	77.7869823	-0.209062	0.10819788	-1.9322188	0.05333251	0.12595827
ENSG000001	18109.4843	-0.2027101	0.06260248	-3.2380524	0.00120349	0.00628965
ENSG000001	1085.84622	-0.173538	0.09446414	-1.8370782	0.06619836	0.14833961
ENSG000001	7539.13302	-0.3215357	0.10812336	-2.9737859	0.0029415	0.01282768
ENSG000001	298.351211	-0.3504101	0.0984578	-3.5589879	0.00037229	0.00240344
ENSG000001	51726.4803	0.45143971	0.15752203	2.86588306	0.00415848	0.01699404
ENSG000001	183.375677	-0.2919947	0.13056343	-2.2364198	0.02532428	0.07213387
ENSG000001	157.25473	0.93267218	0.13771427	6.77251683	1.27E-11	1.26E-09
ENSG000001	37573.2517	1.62372324	0.19619042	8.27626176	1.27E-16	3.61E-14
ENSG000001	10889.9935	-0.3010416	0.11920955	-2.5253141	0.01155949	0.03916333
ENSG000001	101.527265	1.49543208	0.30322108	4.93182089	8.15E-07	1.52E-05
ENSG000001	59.1344757	0.49581174	0.18548545	2.67304932	0.00751652	0.02756057
ENSG000001	2414.64287	1.05238517	0.24658487	4.26784167	1.97E-05	0.00021777
ENSG000001	1635.93558	0.40044727	0.11971422	3.34502687	0.00082275	0.00460841
ENSG000001	2727.02295	0.58355371	0.1603488	3.63927708	0.0002734	0.00186831
ENSG000001	82.8556819	0.47318373	0.18049778	2.6215488	0.00875312	0.03132571
ENSG000001	546.68713	0.19131481	0.11419852	1.6752827	0.09387878	0.19228359
ENSG000001	274.668173	-0.2055216	0.11732597	-1.7517144	0.07982292	0.16998305
ENSG000001	609.211051	-0.2336537	0.12586958	-1.8563158	0.06340856	0.1439135
ENSG000001	180.008407	-0.3061047	0.12184438	-2.5122595	0.01199608	0.04026581
ENSG000001	118.951299	-0.3173714	0.09412416	-3.3718381	0.00074668	0.00425037
ENSG000001	33.4197174	-1.6760284	0.34473617	-4.8617713	1.16E-06	2.04E-05
ENSG000001	3035.59408	-0.2592348	0.080516	-3.2196682	0.00128339	0.00660311
ENSG000001	28.3927791	1.19178116	0.48201547	2.47249568	0.01341733	0.0440764
ENSG000001	139.738076	0.54874627	0.11256386	4.87497731	1.09E-06	1.93E-05
ENSG000001	208.677121	-0.2642175	0.08308337	-3.1801485	0.001472	0.00738394
ENSG000001	696.892399	0.16288182	0.07308776	2.22857878	0.02584194	0.07332006
ENSG000001	4101.30561	-0.3520153	0.13808757	-2.5492179	0.01079648	0.03709339
ENSG000001	82.2436822	1.64616961	0.26856372	6.12953082	8.81E-10	5.01E-08
ENSG000001	352.067026	-0.2203771	0.08459871	-2.6049705	0.00918822	0.03261693

ENSG000001	55.647684	-0.3675017	0.19207851	-1.913289	0.05571107	0.13023822
ENSG000001	3353.86055	0.63074432	0.26076658	2.41880815	0.01557145	0.04952439
ENSG000001	837.362971	-0.1922041	0.06535879	-2.9407538	0.00327415	0.01398778
ENSG000001	356.076896	-0.2483903	0.1308736	-1.8979408	0.05770388	0.13383863
ENSG000001	2492.36131	0.26804506	0.07062696	3.79522305	0.00014751	0.00113008
ENSG000001	89.8832189	-0.6888113	0.17446354	-3.9481678	7.88E-05	0.0006799
ENSG000001	203.206549	-0.1750646	0.07352908	-2.3808892	0.0172709	0.05363421
ENSG000001	153.673345	-0.2309514	0.07219285	-3.1990903	0.00137862	0.00699792
ENSG000001	11435.1772	0.35355124	0.16868526	2.09592256	0.03608906	0.09464559
ENSG000001	289.525701	0.47594127	0.1101952	4.31907435	1.57E-05	0.00018044
ENSG000001	461.67484	-0.3181517	0.07899179	-4.0276557	5.63E-05	0.00051556
ENSG000001	19.4855801	2.41209696	0.40087407	6.01709404	1.78E-09	8.96E-08
ENSG000001	359.89276	0.77254461	0.2014232	3.83543022	0.00012534	0.00099429
ENSG000001	142.278788	-0.4820069	0.19007348	-2.5358977	0.01121595	0.03821389
ENSG000001	14533.5397	0.20608849	0.11536518	1.78640114	0.07403433	0.16107887
ENSG000001	279.483918	0.64926457	0.16864984	3.84977867	0.00011822	0.0009494
ENSG000001	51414.8494	0.21575711	0.09311194	2.31717981	0.02049394	0.06146909
ENSG000001	103.971959	-0.266567	0.13007832	-2.0492809	0.04043465	0.10294873
ENSG000001	16971.4823	0.41633316	0.22385666	1.85982033	0.06291095	0.14305365
ENSG000001	3411.6059	-0.2769776	0.06353862	-4.3592004	1.31E-05	0.00015526
ENSG000001	176.350023	-0.4316987	0.18639104	-2.3160915	0.02055327	0.06163429
ENSG000001	1302.39622	-0.2077421	0.07374591	-2.8169984	0.00484748	0.01932455
ENSG000001	66.8626566	1.1234802	0.27518771	4.0825959	4.45E-05	0.00042586
ENSG000001	58.3457516	0.56291542	0.13800314	4.07900453	4.52E-05	0.00043079
ENSG000001	78.2978414	1.20294737	0.23158878	5.19432493	2.05E-07	4.83E-06
ENSG000001	3053.33792	-0.1226474	0.05268879	-2.327771	0.01992427	0.06020919
ENSG000001	3406.78779	-0.1831836	0.08607045	-2.1282989	0.03331231	0.08890852
ENSG000001	159.488552	-0.3293509	0.14876738	-2.2138648	0.02683808	0.07542823
ENSG000001	97.060901	0.72785212	0.16472662	4.41854593	9.94E-06	0.00012388
ENSG000001	981.679609	-0.7968026	0.17654986	-4.5131872	6.39E-06	8.64E-05
ENSG000001	2612.45676	0.30455061	0.09796347	3.10881794	0.00187837	0.00897198
ENSG000001	1128.83899	0.2071796	0.08954836	2.31360563	0.02068936	0.06192703
ENSG000001	3111.17971	-0.2060151	0.10430902	-1.9750461	0.04826292	0.11689838
ENSG000001	606.59242	0.55957916	0.14731722	3.79846393	0.0001456	0.00111956
ENSG000001	579.892812	0.49668587	0.20903601	2.37607804	0.01749776	0.05421088
ENSG000001	2945.2551	-0.386867	0.17244852	-2.2433767	0.02487253	0.07122521
ENSG000001	3191.39646	0.32267638	0.14069855	2.29338807	0.02182567	0.06440902
ENSG000001	1396.00447	-0.5768401	0.09361696	-6.161705	7.20E-10	4.19E-08
ENSG000001	749.685721	-0.2829881	0.07387238	-3.83077	0.00012774	0.00100961
ENSG000001	79.4989097	-0.4766119	0.22664411	-2.1029087	0.03547375	0.09345484
ENSG000001	366.850275	-0.467944	0.27768622	-1.6851538	0.09195889	0.18944945
ENSG000001	764.007733	0.77808784	0.20396704	3.81477246	0.00013631	0.00106167
ENSG000001	2253.06912	0.20859671	0.08802579	2.36972254	0.01780144	0.05495194

ENSG000001	490.908616	-0.2509305	0.07297137	-3.4387524	0.0005844	0.00346834
ENSG000001	10736.2381	-0.2472203	0.09291474	-2.6607222	0.00779732	0.0284462
ENSG000001	2420.31795	0.87121261	0.19163257	4.54626588	5.46E-06	7.62E-05
ENSG000001	180.911471	0.69674918	0.18745235	3.7169402	0.00020165	0.00145122
ENSG000001	3949.95712	-0.1872009	0.06131092	-3.053304	0.00226337	0.01035999
ENSG000001	1066.5038	-0.1528825	0.08234516	-1.8566058	0.06336726	0.14384229
ENSG000001	205.424449	0.24968896	0.12874036	1.93947688	0.0524433	0.12426334
ENSG000001	758.235871	0.19578979	0.09487928	2.06356749	0.03905874	0.1004338
ENSG000001	1547.64962	0.4587948	0.12264618	3.74079968	0.00018344	0.00134282
ENSG000001	12798.5237	-0.2103823	0.07839089	-2.6837596	0.00727994	0.02684259
ENSG000001	83.3909749	-0.4241884	0.15543324	-2.729071	0.0063513	0.02398627
ENSG000001	81.6007811	-1.2441575	0.35224466	-3.5320832	0.0004123	0.002614
ENSG000001	272.11656	0.42736744	0.11765665	3.63232722	0.00028088	0.00191036
ENSG000001	228.272233	0.41349283	0.24919	1.65934764	0.09704576	0.1971259
ENSG000001	6010.28559	-0.2269736	0.08408181	-2.6994383	0.00694566	0.0258668
ENSG000001	485.492765	0.17391154	0.09835678	1.7681703	0.07703244	0.16572155
ENSG000001	5869.45952	0.17375072	0.07909301	2.19678975	0.02803547	0.07783873
ENSG000001	305.411783	-0.2837137	0.09733588	-2.9147902	0.00355928	0.01497191
ENSG000001	3468.447	0.88575299	0.21474619	4.12465054	3.71E-05	0.00036517
ENSG000001	6134.29981	-0.2324342	0.06468497	-3.5933269	0.00032648	0.00216168
ENSG000001	64.5088081	0.58037046	0.16196136	3.58338845	0.00033917	0.00222834
ENSG000001	91.7687872	-0.2467267	0.09840795	-2.5071825	0.01216979	0.0407262
ENSG000001	44161.9222	4.07433911	0.44430595	9.1701205	4.72E-20	2.63E-17
ENSG000001	681.381795	0.11057302	0.05577874	1.98235048	0.04744003	0.11545544
ENSG000001	821.915131	0.22863481	0.09737773	2.3479168	0.01887874	0.05777277
ENSG000001	441.263064	0.21550315	0.09203353	2.34157218	0.01920271	0.05850467
ENSG000001	229.188876	-0.3995098	0.09249036	-4.3194752	1.56E-05	0.00018025
ENSG000001	1559.68772	-0.2611181	0.06755393	-3.865328	0.00011094	0.00089988
ENSG000001	869.379511	-0.2293977	0.0673921	-3.4039261	0.00066425	0.00384764
ENSG000001	970.467926	0.73791507	0.15036931	4.90735155	9.23E-07	1.69E-05
ENSG000001	5081.48516	0.15390458	0.04568459	3.36885099	0.00075482	0.00428492
ENSG000001	2559.85949	0.81315499	0.19550484	4.15925764	3.19E-05	0.00032505
ENSG000001	68.6578723	0.25720597	0.12484615	2.06018344	0.03938101	0.10099357
ENSG000001	301.112429	0.18148524	0.07734168	2.34653872	0.0189487	0.05793303
ENSG000001	1000.49513	-0.1613003	0.04532809	-3.5585073	0.00037297	0.0024057
ENSG000001	13.6379481	0.96060201	0.37325188	2.57360259	0.01006458	0.03504099
ENSG000001	329.196804	0.26480766	0.07749262	3.41719835	0.00063269	0.00370635
ENSG000001	284.457868	0.40338618	0.18127148	2.22531517	0.02606009	0.07370455
ENSG000001	9156.02438	-0.2045934	0.1019499	-2.0068029	0.04477065	0.11066241
ENSG000001	121.35024	0.97101327	0.19368737	5.0133019	5.35E-07	1.08E-05
ENSG000001	1658.59728	2.4695885	0.28783454	8.57988944	9.50E-18	3.72E-15
ENSG000001	564.430799	0.43502038	0.19864702	2.18991651	0.02853029	0.0789378
ENSG000001	29.3921002	0.75884489	0.35308185	2.14920388	0.03161824	0.08534628

ENSG000001	1005.0401	0.14619655	0.08026656	1.82138795	0.0685479	0.1521084
ENSG000001	297.779363	0.15139204	0.08969691	1.68781765	0.09144624	0.18854756
ENSG000001	10385.1356	0.30056603	0.14628824	2.05461512	0.03991619	0.102113
ENSG000001	650.187086	-0.2958457	0.11928422	-2.4801747	0.0131318	0.04326705
ENSG000001	482.063408	0.16135866	0.0787811	2.04819015	0.04054137	0.10311145
ENSG000001	200.014014	-0.306842	0.1842813	-1.6650742	0.09589796	0.19525983
ENSG000001	627.253555	0.75301149	0.21535535	3.49659993	0.00047123	0.00292321
ENSG000001	368.669269	1.52682025	0.21020402	7.26351583	3.77E-13	5.81E-11
ENSG000001	536.147054	2.41701217	0.38314563	6.30833813	2.82E-10	1.96E-08
ENSG000001	114.499575	0.18447205	0.10575179	1.74438704	0.08109162	0.17222905
ENSG000001	11805.5253	1.78648569	0.25408376	7.03108973	2.05E-12	2.49E-10
ENSG000001	3086.93921	0.31099904	0.16219189	1.91747586	0.05517751	0.12926205
ENSG000001	591.46573	0.32391628	0.08847107	3.66126802	0.00025097	0.00173676
ENSG000001	43.315624	-0.3316977	0.17085442	-1.9414056	0.05220909	0.12388584
ENSG000001	42.3336256	0.76416865	0.22552961	3.38832959	0.0007032	0.00404094
ENSG000001	197688.114	0.66175748	0.17463342	3.7894092	0.00015101	0.00115138
ENSG000001	105.209368	0.93430532	0.27626536	3.38191262	0.00071983	0.0041267
ENSG000001	1413067.17	-0.9276513	0.28659423	-3.2368109	0.00120873	0.0063057
ENSG000001	437.657075	0.18360581	0.10322842	1.77863621	0.07529942	0.16315626
ENSG000001	5500.0607	0.34314248	0.1680608	2.04177581	0.04117377	0.10418943
ENSG000001	2119.2377	0.24748544	0.07712428	3.20891742	0.00133236	0.00680842
ENSG000001	50.4450308	0.74579362	0.21406275	3.48399526	0.00049399	0.00303752
ENSG000001	648.989356	0.36352774	0.09166566	3.96580068	7.31E-05	0.00064148
ENSG000001	1272.36351	-0.2166249	0.10946653	-1.9789142	0.04782567	0.11609425
ENSG000001	4393.56418	0.35736711	0.16838555	2.12231461	0.03381133	0.08997515
ENSG000001	104.00475	0.77258359	0.17617127	4.38541199	1.16E-05	0.00014002
ENSG000001	94.6959585	0.28057076	0.0910207	3.08249397	0.00205274	0.00958982
ENSG000001	209.105582	-0.1966053	0.083971	-2.3413474	0.01921428	0.05851977
ENSG000001	301.590086	-0.2742464	0.15850247	-1.7302343	0.08358842	0.17631705
ENSG000001	165.687998	0.9383903	0.18857167	4.97630572	6.48E-07	1.26E-05
ENSG000001	943.037862	-0.2370529	0.12831807	-1.8473851	0.06469135	0.1461153
ENSG000001	3408.47021	0.40047953	0.12647095	3.16657335	0.00154246	0.00765406
ENSG000001	301.862779	-0.2531583	0.10164473	-2.4906193	0.01275207	0.04224542
ENSG000001	1333.77179	0.16852025	0.08597717	1.96005816	0.04998899	0.1198991
ENSG000001	758.471508	-0.7495011	0.17387443	-4.3105884	1.63E-05	0.00018632
ENSG000001	442.068765	0.38582439	0.12269145	3.14467223	0.00166273	0.00814333
ENSG000001	964.722662	0.21312275	0.06480993	3.28842759	0.00100749	0.00545421
ENSG000001	10771.5511	1.0606465	0.19947957	5.31706823	1.05E-07	2.81E-06
ENSG000001	34686.8047	0.5871004	0.17807657	3.29689862	0.00097759	0.00531618
ENSG000001	1252.05684	-0.1583158	0.05887647	-2.6889477	0.00716776	0.0265167
ENSG000001	6613.59364	0.5722258	0.12201862	4.68965979	2.74E-06	4.24E-05
ENSG000001	3229.78448	0.29936867	0.0688351	4.34907025	1.37E-05	0.00016116
ENSG000001	2153.99517	-0.1702821	0.07991267	-2.1308522	0.03310132	0.08842686

ENSG000001	3750.41303	-0.1797072	0.07145282	-2.5150476	0.01190163	0.04003226
ENSG000001	1000.22637	0.75512776	0.1856984	4.06642042	4.77E-05	0.00045086
ENSG000001	268.219037	-0.1965999	0.1035331	-1.898909	0.05757644	0.13360723
ENSG000001	837.160335	0.22122823	0.10723811	2.06296283	0.03911616	0.10056359
ENSG000001	104.279025	0.59646642	0.26093552	2.28587664	0.02226148	0.06529703
ENSG000001	2031.96458	0.56252218	0.1638259	3.43365842	0.00059549	0.00351976
ENSG000001	4546.3091	0.27054411	0.15926506	1.69870344	0.08937507	0.18532458
ENSG000001	1619.49352	-0.1180793	0.06999135	-1.6870561	0.09159256	0.18874843
ENSG000001	4807.4863	-0.352681	0.08466814	-4.1654512	3.11E-05	0.00031702
ENSG000001	1828.24032	-0.2187517	0.06653601	-3.2877183	0.00101003	0.00546351
ENSG000001	182.739075	0.63499442	0.16727913	3.79601695	0.00014704	0.00112725
ENSG000001	5764.50118	0.36349598	0.15055438	2.41438323	0.01576187	0.04992501
ENSG000001	140.027008	0.27595794	0.14980785	1.84207926	0.06546356	0.14722888
ENSG000001	94.6568618	2.71907171	0.50070831	5.43045058	5.62E-08	1.66E-06
ENSG000001	1668.68912	0.59161748	0.35733672	1.65563026	0.09779672	0.19823437
ENSG000001	135.220067	-0.1841067	0.10139916	-1.8156633	0.06942203	0.15359148
ENSG000001	227.308636	0.50945304	0.19980409	2.54976287	0.01077962	0.03705847
ENSG000001	2946.74211	-0.4381187	0.05411395	-8.0962248	5.67E-16	1.47E-13
ENSG000001	2460.21481	0.15130878	0.0759584	1.99199521	0.04637158	0.11357509
ENSG000001	66.093507	0.57854744	0.15802125	3.66120023	0.00025104	0.00173676
ENSG000001	864.474488	-0.6068239	0.18851944	-3.2188932	0.00128686	0.00660858
ENSG000001	7406.94688	-0.1896047	0.08237684	-2.3016744	0.02135354	0.06345616
ENSG000001	810.27016	0.59488209	0.27737405	2.14469269	0.03197743	0.08617561
ENSG000001	494.420043	0.36970714	0.17065115	2.16644976	0.03027683	0.08264943
ENSG000001	385.665422	-0.4576666	0.09127216	-5.0143072	5.32E-07	1.07E-05
ENSG000001	176.00876	0.19806716	0.06455307	3.0682841	0.00215292	0.00997739
ENSG000001	1576.51887	0.57167894	0.11539186	4.95423984	7.26E-07	1.38E-05
ENSG000001	3724.10795	1.18170892	0.18797601	6.28648784	3.25E-10	2.16E-08
ENSG000001	585.669544	0.7922232	0.13206592	5.99869535	1.99E-09	9.90E-08
ENSG000001	2131.41331	3.17181154	0.36008165	8.80858987	1.27E-18	5.74E-16
ENSG000001	44785.5669	0.26560316	0.12553952	2.11569368	0.03437087	0.09114603
ENSG000001	208.759945	0.87242802	0.35657808	2.44666758	0.01441838	0.04670818
ENSG000001	666.285428	-0.3617841	0.11372414	-3.1812424	0.00146645	0.00736376
ENSG000001	858.479321	-0.1330086	0.06864419	-1.9376528	0.05266559	0.12466772
ENSG000001	12787.7596	0.41675139	0.17518289	2.37895023	0.01736202	0.05384791
ENSG000001	1107.40774	0.18405143	0.05450074	3.37704461	0.00073269	0.00418718
ENSG000001	109.821289	-0.8994199	0.17896625	-5.0256398	5.02E-07	1.02E-05
ENSG000001	333.90811	-0.1988688	0.09209732	-2.1593328	0.03082435	0.08365603
ENSG000001	7319.61046	-0.2040583	0.07433246	-2.7452114	0.00604719	0.02302989
ENSG000001	1156.58394	0.3337393	0.17975205	1.85666485	0.06335885	0.14384229
ENSG000001	366.579296	-0.2566881	0.06085703	-4.2178876	2.47E-05	0.00026327
ENSG000001	797.957411	0.2943464	0.12797422	2.3000444	0.0214457	0.06366474
ENSG000001	7030.12541	0.38076963	0.09503825	4.00648821	6.16E-05	0.00055557

ENSG000001	141.885979	0.49115775	0.14428997	3.40396314	0.00066416	0.00384764
ENSG000001	2343.80722	-0.1778119	0.09016296	-1.9721173	0.04859621	0.11746212
ENSG000001	1965.69475	0.30045532	0.10305926	2.91536464	0.00355273	0.01495306
ENSG000001	1016.60908	-0.2244326	0.0732826	-3.0625631	0.0021945	0.01012362
ENSG000001	2903.41209	-0.201568	0.06982635	-2.8867038	0.003893	0.01609072
ENSG000001	782.502745	0.43924657	0.12787602	3.4349409	0.00059268	0.00350743
ENSG000001	12729.7332	2.10091765	0.24048489	8.73617321	2.41E-18	1.06E-15
ENSG000001	1674.66298	-0.2247351	0.059123	-3.8011457	0.00014403	0.00110901
ENSG000001	2170.97157	-0.156713	0.08992656	-1.7426772	0.08139002	0.17276149
ENSG000001	83.536835	1.15553747	0.22936779	5.037924	4.71E-07	9.64E-06
ENSG000001	761.494659	0.30195161	0.10245689	2.94710891	0.0032076	0.01377662
ENSG000001	772.507191	-0.1742189	0.09184994	-1.8967775	0.05785732	0.13413009
ENSG000001	205.357593	-0.1615743	0.06636691	-2.4345615	0.01490985	0.04790396
ENSG000001	45.220271	2.2708377	0.48244743	4.70691224	2.51E-06	3.99E-05
ENSG000001	8771.73498	-0.2572397	0.10095609	-2.5480349	0.01083316	0.03718958
ENSG000001	88.1174158	0.66880769	0.25140573	2.66027229	0.00780775	0.02847706
ENSG000001	306.39582	-0.3550726	0.10105582	-3.5136287	0.00044203	0.00276379
ENSG000001	1017.06559	-0.2641252	0.08599771	-3.0713053	0.00213125	0.00988963
ENSG000001	723.441607	-0.280426	0.09603623	-2.9200017	0.00350029	0.01476668
ENSG000001	1407.98558	-0.1541534	0.06607373	-2.3330518	0.01964543	0.05951553
ENSG000001	229.655351	0.70601307	0.133311	5.29598513	1.18E-07	3.09E-06
ENSG000001	294.462926	-0.1899913	0.1015016	-1.8718056	0.0612335	0.14031788
ENSG000001	5492.51714	0.19638188	0.07109094	2.76240374	0.00573775	0.02213066
ENSG000001	176.007524	-0.1885139	0.10199252	-1.8483108	0.0645574	0.14592651
ENSG000001	2005.59099	-0.0858378	0.03877683	-2.2136366	0.02685378	0.07545205
ENSG000001	443.563482	-0.4256693	0.21871126	-1.9462615	0.05162333	0.12277458
ENSG000001	5541.45521	0.28499307	0.07302449	3.90270553	9.51E-05	0.0007929
ENSG000001	80.8785551	-0.7258339	0.24386878	-2.9763298	0.00291721	0.01274092
ENSG000001	11989.807	0.48914162	0.17409566	2.80961415	0.00496009	0.01971839
ENSG000001	31.0605916	-1.6523558	0.36616303	-4.5126232	6.40E-06	8.65E-05
ENSG000001	37.0628033	0.63377503	0.25035389	2.53151663	0.01135704	0.03855859
ENSG000001	9.76293786	0.98893907	0.52925035	1.86856573	0.06168326	0.14108073
ENSG000001	425.927374	0.34443997	0.10275597	3.3520191	0.00080224	0.00450722
ENSG000001	849.466744	0.20432597	0.04934778	4.14052987	3.47E-05	0.00034643
ENSG000001	287.117461	0.14389875	0.07609783	1.89097039	0.0586283	0.13565694
ENSG000001	9431.86457	0.39798792	0.16710317	2.38168979	0.01723341	0.05357519
ENSG000001	1115.89694	0.43216167	0.16591948	2.60464702	0.0091969	0.03263974
ENSG000001	1065.59618	0.6550925	0.17783344	3.68374197	0.00022983	0.00161503
ENSG000001	1893.23279	0.43349772	0.16304522	2.6587576	0.00784294	0.02857662
ENSG000001	25058.8023	0.72457875	0.1526658	4.74617607	2.07E-06	3.38E-05
ENSG000001	274.653015	-0.15348	0.07822832	-1.9619497	0.04976834	0.11954799
ENSG000001	2417.55973	0.1059084	0.0439952	2.40727197	0.0160722	0.05070512
ENSG000001	1879.52872	0.29633349	0.10489962	2.82492422	0.00472918	0.01890498

ENSG000001	165.939668	-0.275362	0.15762448	-1.7469496	0.08064608	0.17143356
ENSG000001	131.908518	0.76989969	0.190593	4.03949607	5.36E-05	0.00049681
ENSG000001	388.301814	-0.47533	0.1710727	-2.7785261	0.00546061	0.02125125
ENSG000001	447.453454	0.32336098	0.07564356	4.27479882	1.91E-05	0.00021221
ENSG000001	7566.54451	0.25015837	0.13662964	1.83092308	0.06711202	0.14976719
ENSG000001	188.675144	-0.3582356	0.15335938	-2.3359223	0.01949529	0.05917207
ENSG000001	69.2494221	0.70809695	0.13269823	5.33614478	9.49E-08	2.56E-06
ENSG000001	656.488196	0.15815886	0.07253758	2.18037114	0.02922996	0.08044348
ENSG000001	13625.0442	0.31940748	0.18583988	1.71872415	0.08566461	0.17949425
ENSG000001	65.9432918	0.87343576	0.28406129	3.07481445	0.00210634	0.00980228
ENSG000001	151.109831	0.21065809	0.09037196	2.33101168	0.01975274	0.05980408
ENSG000001	20418.6137	0.62787012	0.13646803	4.60085853	4.21E-06	6.10E-05
ENSG000001	8636.17938	-0.1985449	0.10162829	-1.9536376	0.05074409	0.12118707
ENSG000001	2403.59414	0.29296323	0.09055237	3.23529068	0.00121519	0.00633254
ENSG000001	522.429855	-0.2837111	0.122614	-2.3138554	0.02067565	0.06191157
ENSG000001	781.335323	0.44112614	0.11006689	4.00780056	6.13E-05	0.00055319
ENSG000001	234.324591	0.32417569	0.13102488	2.47415374	0.01335522	0.04390222
ENSG000001	959.697473	0.77266472	0.21228971	3.63967102	0.00027299	0.00186661
ENSG000001	162.72783	-0.3539414	0.1298241	-2.7263148	0.00640459	0.02416861
ENSG000001	1337.7641	-0.1475494	0.07410576	-1.991065	0.04647374	0.11372951
ENSG000001	1104.82791	-0.1123147	0.04507679	-2.4916293	0.01271587	0.04214208
ENSG000001	18377.6321	-0.2255474	0.11233964	-2.0077279	0.04467222	0.11043796
ENSG000001	457.462581	0.4457451	0.15879388	2.80706727	0.00499948	0.01984525
ENSG000001	1936.96598	0.21615618	0.11505995	1.87863969	0.06029371	0.13850947
ENSG000001	202.358632	0.4791508	0.13203044	3.6290935	0.00028442	0.00192991
ENSG000001	2092.57135	0.37810095	0.10880906	3.47490329	0.00051104	0.00311722
ENSG000001	618.95299	-0.224838	0.12280148	-1.8309061	0.06711456	0.14976719
ENSG000001	8198.49146	0.12638055	0.06353744	1.98907192	0.04669326	0.11416799
ENSG000001	9375.39032	-0.2559976	0.08105708	-3.1582389	0.00158725	0.00784262
ENSG000001	323.854481	1.06965279	0.22124663	4.8346625	1.33E-06	2.31E-05
ENSG000001	6363.6945	0.16593616	0.06865728	2.41687642	0.01565433	0.04971159
ENSG000001	5180.67127	-0.2859376	0.09388661	-3.0455631	0.00232245	0.01057697
ENSG000001	171.64299	0.48869771	0.22563443	2.16588276	0.03032015	0.08268975
ENSG000001	4122.27336	-0.1790606	0.06473878	-2.7658935	0.00567671	0.02194781
ENSG000001	2648.58559	0.64967717	0.19495918	3.33237535	0.00086108	0.00476671
ENSG000001	6608.48343	0.11194606	0.0630732	1.77485949	0.07592109	0.16401265
ENSG000001	493.373012	0.23042852	0.08513795	2.7065313	0.00679902	0.02541867
ENSG000001	1108.85722	0.1802659	0.05498243	3.27860894	0.0010432	0.00560622
ENSG000001	132.262216	0.85862237	0.17413982	4.93064937	8.20E-07	1.52E-05
ENSG000001	339.956708	-0.4078686	0.10101598	-4.0376646	5.40E-05	0.00049974
ENSG000001	375.51735	0.19600513	0.0969456	2.02180535	0.04319646	0.10789434
ENSG000001	483.794376	0.15228827	0.08677306	1.75501792	0.07925624	0.16909747
ENSG000001	6264.27546	0.33947874	0.182351	1.8616774	0.06264858	0.14256832

ENSG000001	12192.522	0.3431948	0.18005497	1.90605566	0.05664298	0.13203329
ENSG000001	751.944769	0.35487231	0.10630491	3.33824945	0.00084308	0.00469397
ENSG000001	84.2785117	-1.4119191	0.37284926	-3.7868364	0.00015258	0.00116153
ENSG000001	513.965323	-0.2612491	0.08447602	-3.0925835	0.00198422	0.00935724
ENSG000001	526.783483	-0.1870437	0.0948686	-1.9716085	0.04865431	0.11753794
ENSG000001	695.998693	0.28116765	0.09542339	2.94652754	0.00321364	0.01378988
ENSG000001	1639.30355	-0.3719833	0.10047029	-3.7024208	0.00021355	0.00152026
ENSG000001	42.1902276	0.67739116	0.24720052	2.74024978	0.00613925	0.02334366
ENSG000001	319.100071	0.13884298	0.06898702	2.01259588	0.04415716	0.10950099
ENSG000001	6344.46791	0.36998405	0.13950082	2.65219992	0.00799692	0.02906456
ENSG000001	16741.4603	0.19456459	0.07403051	2.62816751	0.00858462	0.03081403
ENSG000001	2604.73316	-0.2339632	0.06457355	-3.6232048	0.00029098	0.0019652
ENSG000001	2708.13538	-0.3203292	0.13546496	-2.3646645	0.01804641	0.05564886
ENSG000001	40.1215241	-1.0260068	0.30568422	-3.356427	0.00078957	0.00445422
ENSG000001	8184.67504	-0.2565838	0.10473523	-2.449833	0.01429225	0.04641376
ENSG000001	265.684502	-0.3502022	0.13088957	-2.6755545	0.00746057	0.02738316
ENSG000001	77.9083776	-0.6680311	0.16020523	-4.169846	3.05E-05	0.00031202
ENSG000001	410.617451	0.26631296	0.09685391	2.74963555	0.00596616	0.02279919
ENSG000001	62.6572279	0.85940689	0.28968692	2.96667484	0.00301039	0.01307692
ENSG000001	4183.9464	1.01764981	0.14262925	7.13493057	9.68E-13	1.31E-10
ENSG000001	3018.88163	0.30880524	0.1181737	2.61314691	0.00897127	0.0319879
ENSG000001	103.867371	1.92661055	0.42416619	4.54211246	5.57E-06	7.76E-05
ENSG000001	1852.36779	-0.17314	0.1006531	-1.7201654	0.08540239	0.17907431
ENSG000001	729.584069	-0.1496705	0.06312901	-2.3708671	0.01774641	0.05481711
ENSG000001	518.630009	0.13587579	0.06158753	2.20622224	0.02736844	0.07641592
ENSG000001	56681.3902	0.20779423	0.10492657	1.98037772	0.0476611	0.1158112
ENSG000001	1208.49888	-0.5602397	0.12999366	-4.3097464	1.63E-05	0.00018688
ENSG000001	129.27077	0.68985444	0.29982357	2.30086798	0.02139909	0.06355242
ENSG000001	9380.54616	-0.2004206	0.09208073	-2.1765748	0.0295123	0.08091309
ENSG000001	650.575968	0.17314766	0.09405349	1.8409488	0.06562906	0.14742878
ENSG000001	12.6710017	1.03279532	0.35269491	2.92829662	0.00340825	0.01446259
ENSG000001	436.74657	0.73693983	0.20547107	3.58658685	0.00033503	0.0022052
ENSG000001	1174.10689	0.24799858	0.09329956	2.6580896	0.0078585	0.02862612
ENSG000001	134.943748	-0.4126657	0.14970716	-2.7564863	0.00584261	0.02244546
ENSG000001	563.231062	0.41717742	0.10618313	3.92884852	8.54E-05	0.0007243
ENSG000001	332.689044	0.19114746	0.09596152	1.99191776	0.04638008	0.11357509
ENSG000001	92.7292707	0.2034327	0.1002676	2.02889781	0.0424687	0.10659114
ENSG000001	97.5180575	0.70098655	0.17599514	3.98298806	6.81E-05	0.00060263
ENSG000001	8967.09865	0.62003132	0.15440186	4.01569852	5.93E-05	0.00053833
ENSG000001	99.375291	0.42154203	0.1755088	2.40182852	0.01631335	0.05134294
ENSG000001	1242.77571	0.10883384	0.05442946	1.9995393	0.04555003	0.11214877
ENSG000001	887.728836	-0.1757739	0.10288568	-1.7084388	0.08755495	0.18242608
ENSG000001	159.407298	-0.6918171	0.19408399	-3.5645243	0.00036452	0.00236275

ENSG000001	120.603306	0.19269687	0.07299549	2.63984624	0.00829436	0.0299428
ENSG000001	120.346975	-0.193341	0.1051953	-1.8379244	0.06607355	0.14822042
ENSG000001	209.302699	-0.4480352	0.13507803	-3.3168625	0.00091034	0.00500499
ENSG000001	5399.11226	0.30159013	0.0742051	4.06427745	4.82E-05	0.00045413
ENSG000001	388.353384	-0.2911946	0.08412591	-3.4614144	0.00053735	0.00324085
ENSG000001	61184.8333	0.74960303	0.15389845	4.87076399	1.11E-06	1.96E-05
ENSG000001	6274.53078	-0.5171569	0.23905512	-2.1633374	0.03051524	0.08309666
ENSG000001	750.189184	0.22827044	0.10833889	2.10700364	0.03511726	0.09262847
ENSG000001	4735.29995	0.2260548	0.07181033	3.14794253	0.00164424	0.00807461
ENSG000001	826.960076	0.94728406	0.21202586	4.46777607	7.90E-06	0.0001026
ENSG000001	49504.3755	-0.2590667	0.12187599	-2.1256583	0.03353172	0.08937885
ENSG000001	171.616425	0.43115721	0.12556693	3.43368444	0.00059544	0.00351976
ENSG000001	210.816794	0.2899248	0.10572705	2.74220082	0.0061029	0.02321153
ENSG000001	926.067152	-0.1813046	0.09595134	-1.8895471	0.05881856	0.136024
ENSG000001	1248.53394	0.3065904	0.1443061	2.12458386	0.03362135	0.08958479
ENSG000001	503.029221	-0.2888481	0.11369935	-2.5404548	0.01107084	0.03779054
ENSG000001	3136.88669	0.18983637	0.10207504	1.85977262	0.0629177	0.14305365
ENSG000001	542.310873	0.15559683	0.08213962	1.894297	0.0581856	0.1347833
ENSG000001	4469.96836	0.35855503	0.10971397	3.26808907	0.00108276	0.00577115
ENSG000001	1718.25669	-0.1391157	0.08424154	-1.6513911	0.09865874	0.19939582
ENSG000001	183.87037	-0.2834374	0.12633508	-2.2435364	0.02486224	0.07120983
ENSG000001	16.1858951	0.60569896	0.29526923	2.05134466	0.04023339	0.10273844
ENSG000001	14515.4008	0.36637033	0.13642254	2.68555563	0.00724093	0.02672596
ENSG000001	5628.37846	0.33660315	0.05206919	6.46453593	1.02E-10	8.13E-09
ENSG000001	1019.20748	1.06036583	0.22384532	4.73704704	2.17E-06	3.52E-05
ENSG000001	4856.44359	-0.2853675	0.0489763	-5.8266458	5.66E-09	2.49E-07
ENSG000001	5093.15711	-0.1874847	0.0391652	-4.7870219	1.69E-06	2.84E-05
ENSG000001	520.639042	-0.3629051	0.09709119	-3.7377761	0.00018566	0.00135461
ENSG000001	3427.9928	0.37079199	0.10901531	3.40128377	0.0006707	0.00387418
ENSG000001	1482.77367	-0.1644423	0.08229847	-1.9981205	0.04570359	0.11238105
ENSG000001	5232.86784	-0.218552	0.10203457	-2.1419408	0.03219825	0.08655766
ENSG000001	638.714272	-0.2933416	0.10732757	-2.7331428	0.00627331	0.02376608
ENSG000001	7432.05731	0.25036668	0.1221076	2.05037762	0.04032759	0.10279306
ENSG000001	2675.19402	1.28663725	0.27642745	4.65452054	3.25E-06	4.92E-05
ENSG000001	7730.35266	0.22389178	0.10820414	2.06916087	0.03853099	0.09939432
ENSG000001	59.3247627	-0.9746429	0.22825355	-4.2700011	1.95E-05	0.00021584
ENSG000001	26530.5027	0.33818327	0.09888319	3.42002801	0.00062615	0.00367416
ENSG000001	382.319209	0.60338266	0.14885423	4.0535137	5.05E-05	0.00047218
ENSG000001	99.1728376	-0.1922635	0.11425878	-1.6827023	0.09243273	0.1901552
ENSG000001	458.291106	-0.3980771	0.09952359	-3.9998261	6.34E-05	0.00056651
ENSG000001	1212.50666	0.19110956	0.11190558	1.70777513	0.08767807	0.18263009
ENSG000001	546.845744	-0.5642824	0.18171649	-3.1052901	0.00190092	0.00904892
ENSG000001	2146.0986	-0.2073367	0.05277444	-3.9287332	8.54E-05	0.0007243

ENSG000001	161.445375	0.25285455	0.11969613	2.11247056	0.03464611	0.09175835
ENSG000001	1511.03974	-0.2540899	0.11885278	-2.1378544	0.03252856	0.08725074
ENSG000001	1026.0516	-0.570105	0.23273753	-2.4495619	0.01430301	0.04642545
ENSG000001	5262.88489	0.54293099	0.17465915	3.10851736	0.00188029	0.00897815
ENSG000001	1668.00217	-0.1733283	0.0923353	-1.8771619	0.0604959	0.1388458
ENSG000001	736.644763	0.4329757	0.1359059	3.18584925	0.0014433	0.00728792
ENSG000001	9693.80224	0.51150089	0.14247223	3.59017947	0.00033045	0.00218097
ENSG000001	70.9308672	0.57363449	0.12547211	4.57180878	4.84E-06	6.89E-05
ENSG000001	14907.7109	0.21302182	0.07009996	3.0388293	0.00237499	0.01075368
ENSG000001	22502.5438	-0.1184831	0.05189787	-2.2830049	0.02243008	0.06561982
ENSG000001	441.238841	0.39651014	0.21780912	1.82044784	0.06869083	0.15233247
ENSG000001	1027.46761	-0.1253389	0.05169742	-2.4244718	0.01533067	0.04890894
ENSG000001	23131.8448	-0.734052	0.21611789	-3.3965352	0.00068245	0.00393106
ENSG000001	78044.3469	-0.5569318	0.17258799	-3.2269443	0.0012512	0.00647592
ENSG000001	1489.85047	0.377943	0.14117694	2.67708739	0.00742653	0.02728743
ENSG000001	789.567379	-0.2474257	0.09232566	-2.6799229	0.00736391	0.02708327
ENSG000001	73712.3981	-1.0224409	0.19720523	-5.1846541	2.16E-07	5.05E-06
ENSG000001	3693.30651	-0.1542105	0.07463279	-2.0662574	0.03880418	0.09997443
ENSG000001	4236.06769	0.81492225	0.24441343	3.33419587	0.00085546	0.00473936
ENSG000001	35381.8909	0.48578951	0.18987192	2.55851154	0.01051213	0.03630257
ENSG000001	61.1682693	-1.2738968	0.49455363	-2.5758517	0.00999935	0.0348474
ENSG000001	23.9347257	0.56633675	0.27974501	2.02447486	0.04292132	0.10750365
ENSG000001	100.743272	-0.5812352	0.28889879	-2.0118991	0.04423058	0.10962675
ENSG000001	57353.514	-0.5882781	0.18876064	-3.1165296	0.00182993	0.00879494
ENSG000001	139.254273	0.34583492	0.11378338	3.03941511	0.00237038	0.01074459
ENSG000001	57179.0262	-0.8325882	0.19431509	-4.2847327	1.83E-05	0.00020467
ENSG000001	3146.9521	-0.1026781	0.05597324	-1.8344138	0.06659259	0.14899256
ENSG000001	254.475825	-0.3601904	0.20592855	-1.749104	0.08027304	0.17081604
ENSG000001	1076.1845	-0.1246854	0.07243222	-1.7214079	0.08517683	0.1787048
ENSG000001	136711.875	-0.7211842	0.18620363	-3.8730943	0.00010746	0.00087573
ENSG000001	2169.48709	0.4094411	0.15214466	2.69113024	0.00712104	0.02638427
ENSG000001	3298.60795	-0.1225981	0.05509962	-2.2250263	0.02607947	0.07372351
ENSG000001	256.505298	-0.4543091	0.14399113	-3.1551185	0.00160433	0.0079108
ENSG000001	2860.0977	0.29147359	0.09049031	3.22104746	0.00127723	0.00658712
ENSG000001	9595.74347	-0.3856628	0.12017153	-3.2092694	0.00133073	0.00680729
ENSG000001	930.106194	-0.472133	0.07966976	-5.9261248	3.10E-09	1.48E-07
ENSG000001	96.9835162	1.11448249	0.24033965	4.63711455	3.53E-06	5.28E-05
ENSG000001	4549.49758	0.16689607	0.06633101	2.51610945	0.01186583	0.03993967
ENSG000001	373.306037	-0.43745	0.18104575	-2.4162403	0.01568171	0.04977671
ENSG000001	7816.60794	0.48027334	0.12769477	3.76110438	0.00016916	0.00126389
ENSG000001	13658.9756	0.18691641	0.06018523	3.10568593	0.00189838	0.00904072
ENSG000001	624.982869	0.33023381	0.09537696	3.46240649	0.00053537	0.00323161
ENSG000001	22562.252	-0.8281234	0.1597278	-5.1845912	2.16E-07	5.05E-06

ENSG000001	3290.49684	0.33146133	0.10198379	3.25013753	0.00115349	0.00606997
ENSG000001	369.080278	0.67542495	0.28689834	2.35423096	0.01856107	0.05700536
ENSG000001	20352.1489	-0.3070234	0.13558538	-2.2644284	0.02354778	0.0682137
ENSG000001	1150.01023	0.25343758	0.10379781	2.44164667	0.01462045	0.04721947
ENSG000001	411.394484	-0.1188023	0.06792964	-1.7489026	0.08030786	0.17086503
ENSG000001	1129.12616	-0.3213161	0.1040861	-3.0870219	0.00202173	0.00947823
ENSG000001	144238.845	-0.981091	0.19252066	-5.0960298	3.47E-07	7.42E-06
ENSG000001	76156.3349	-0.7098343	0.20034846	-3.5429985	0.0003956	0.00252918
ENSG000001	575.837986	-0.1712029	0.10134969	-1.6892296	0.09117544	0.18820561
ENSG000001	352.865547	-0.6361324	0.28734405	-2.2138354	0.0268401	0.07542823
ENSG000001	6031.62155	0.52366462	0.14401626	3.63614941	0.00027674	0.00188934
ENSG000001	46267.5231	-0.805186	0.18615302	-4.3253986	1.52E-05	0.00017646
ENSG000001	1413.26887	-0.1400865	0.05961183	-2.3499787	0.01877449	0.05755099
ENSG000001	856.341492	0.81658124	0.17647531	4.62716985	3.71E-06	5.47E-05
ENSG000001	187.83258	0.80894365	0.20037663	4.03711568	5.41E-05	0.00050027
ENSG000001	919.911133	-0.1085219	0.06221564	-1.7442865	0.08110915	0.17224102
ENSG000001	326.428091	0.27406747	0.10559304	2.59550688	0.00944515	0.03332487
ENSG000001	2027.02382	0.18076852	0.10877131	1.66191361	0.0965301	0.19627109
ENSG000001	14.8625792	1.56748955	0.31961466	4.9043106	9.38E-07	1.71E-05
ENSG000001	4637.916	-0.2483695	0.11401371	-2.1784178	0.02937495	0.08073513
ENSG000001	918.009408	-0.5399084	0.15711645	-3.4363583	0.00058959	0.00349526
ENSG000001	4370.99014	0.69861125	0.17264826	4.04644236	5.20E-05	0.00048354
ENSG000001	219.15285	-0.4636824	0.12085953	-3.8365396	0.00012478	0.00099156
ENSG000001	70036.3956	-0.6648037	0.17285037	-3.8461227	0.00012	0.00096154
ENSG000001	1042.32477	-0.236805	0.10145516	-2.3340858	0.01959123	0.05938861
ENSG000001	4627.19227	-0.1803988	0.10524685	-1.7140542	0.08651877	0.18073503
ENSG000001	10321.9258	0.27954249	0.10832278	2.58064357	0.00986163	0.03446695
ENSG000001	539.644363	-0.1069144	0.05086047	-2.1021126	0.03554341	0.09354526
ENSG000002	133.187351	0.67432478	0.15886532	4.24463168	2.19E-05	0.00023784
ENSG000002	2231.93911	-0.3331111	0.13977994	-2.3831109	0.01716702	0.0534491
ENSG000002	859.329326	0.32098971	0.17199021	1.8663255	0.06199584	0.14154984
ENSG000002	331.166296	0.50384661	0.17207131	2.92812678	0.00341011	0.01446625
ENSG000002	24.0178422	0.44639059	0.25135889	1.77590927	0.07574787	0.16376055
ENSG000002	885.053864	0.18405108	0.08390982	2.19343908	0.02827576	0.07839826
ENSG000002	9234.09111	0.68834847	0.21320897	3.22851556	0.00124434	0.00645198
ENSG000002	271.294453	0.26582093	0.10841393	2.45190742	0.01421012	0.04618848
ENSG000002	48949.5321	0.53305335	0.19184242	2.77859995	0.00545937	0.02125125
ENSG000002	86.8830374	0.90018684	0.16232556	5.54556425	2.93E-08	9.92E-07
ENSG000002	64.485403	0.70724148	0.23654689	2.98985742	0.00279108	0.01227515
ENSG000002	38.9603204	0.35157005	0.17373243	2.02362939	0.0430083	0.10757853
ENSG000002	629.929783	1.17429266	0.2073172	5.66423179	1.48E-08	5.50E-07
ENSG000002	295.198238	0.86867764	0.20343242	4.27010433	1.95E-05	0.00021584
ENSG000002	95.3189579	0.20713287	0.0942441	2.19783389	0.02796095	0.07770386

ENSG000002	110.510115	0.2305406	0.12319302	1.87137711	0.06129283	0.14038723
ENSG000002	502.373643	0.27810703	0.10189912	2.72923877	0.00634807	0.02398627
ENSG000002	610.94815	-0.1354149	0.07245489	-1.8689543	0.06162917	0.14097928
ENSG000002	891.601441	0.47423631	0.25412044	1.86618719	0.06201518	0.14157169
ENSG000002	456.547965	0.7586377	0.26589706	2.85312559	0.00432915	0.01755779
ENSG000002	32.1468131	1.36046366	0.32503088	4.18564434	2.84E-05	0.00029488
ENSG000002	3853.74785	0.46073624	0.13245163	3.47852434	0.00050418	0.0030845
ENSG000002	1456.6492	-0.3614263	0.07983208	-4.5273312	5.97E-06	8.19E-05
ENSG000002	244.774766	-0.4145915	0.08112715	-5.1103919	3.21E-07	6.95E-06
ENSG000002	13449.3451	0.26413472	0.1348795	1.95830137	0.05019466	0.12027291
ENSG000002	1393.69942	-0.1569361	0.05930826	-2.6461088	0.00814236	0.02952651
ENSG000002	45310.5867	0.26930679	0.16047149	1.67822197	0.09330377	0.19148488
ENSG000002	1206.91421	-0.3132452	0.06126864	-5.1126517	3.18E-07	6.90E-06
ENSG000002	545.271548	-0.2625816	0.0792074	-3.3151146	0.00091606	0.00503192
ENSG000002	1418.448	0.40418423	0.11299726	3.5769382	0.00034764	0.00226962
ENSG000002	318.234965	-0.1419968	0.0857608	-1.6557304	0.09777643	0.19823437
ENSG000002	70.9872271	-0.4624321	0.21049431	-2.1968866	0.02802854	0.07783873
ENSG000002	891.830701	-0.4416282	0.12789176	-3.4531407	0.0005541	0.00331704
ENSG000002	3157.0644	0.35563072	0.11063721	3.21438624	0.00130724	0.00670607
ENSG000002	564.157277	-0.2661943	0.12807692	-2.0783944	0.03767305	0.09779338
ENSG000002	782.424821	-0.1890612	0.10691028	-1.7684099	0.07699241	0.16566004
ENSG000002	638.677111	0.27415808	0.1374873	1.9940611	0.04614538	0.11313388
ENSG000002	74.7678571	-0.6560741	0.38977942	-1.6831932	0.0923377	0.19004067
ENSG000002	8.55530066	1.41795077	0.45579789	3.11091998	0.00186505	0.00892307
ENSG000002	494.816491	-0.2835089	0.08413973	-3.3695006	0.00075305	0.00427818
ENSG000002	374.022115	0.27414812	0.099321	2.76022299	0.00577619	0.02225524
ENSG000002	691.312685	-0.3212587	0.09254626	-3.4713309	0.00051789	0.00314707
ENSG000002	1884.62131	1.26983647	0.16966054	7.48457167	7.18E-14	1.28E-11
ENSG000002	963.423756	0.16393206	0.07504869	2.18434266	0.02893708	0.07977383
ENSG000002	5072.41128	-0.3432992	0.12685877	-2.7061527	0.00680677	0.02543455
ENSG000002	3591.50647	-0.2277852	0.10440489	-2.1817488	0.02912808	0.08025456
ENSG000002	3744.14992	0.48404546	0.14348286	3.37354212	0.00074208	0.00422962
ENSG000002	156.940301	-0.6790691	0.16138666	-4.2077154	2.58E-05	0.00027298
ENSG000002	180.189864	0.54893217	0.11657918	4.70866374	2.49E-06	3.97E-05
ENSG000002	428.713495	-0.2100474	0.11440543	-1.8359912	0.06635896	0.14865352
ENSG000002	161.349196	0.22642112	0.11276016	2.00798861	0.0446445	0.11040712
ENSG000002	43044.9168	1.57012358	0.3313444	4.7386453	2.15E-06	3.50E-05
ENSG000002	118.264905	-0.3262061	0.07743872	-4.2124421	2.53E-05	0.00026851
ENSG000002	923.05794	-0.3326139	0.0934931	-3.5576304	0.00037422	0.00241266
ENSG000002	46193.483	0.33963885	0.12732238	2.66755022	0.00764065	0.02797322
ENSG000002	331.460591	-0.2230216	0.05133968	-4.3440396	1.40E-05	0.00016422
ENSG000002	46124.0246	-0.3169935	0.10356625	-3.0607797	0.00220761	0.01016907
ENSG000002	1885.98526	0.26806616	0.15289165	1.75330809	0.07954914	0.16954956

ENSG000002	5093.96547	0.25995842	0.12224636	2.12651263	0.0334606	0.08927261
ENSG000002	1472.4924	-0.1959005	0.0977954	-2.0031667	0.0451594	0.11149016
ENSG000002	386.943083	0.19445752	0.06462845	3.00885306	0.00262236	0.01168567
ENSG000002	151.307157	-0.4384542	0.21053053	-2.0826159	0.03728624	0.09701254
ENSG000002	39.6546657	1.45422314	0.31085713	4.67810776	2.90E-06	4.47E-05
ENSG000002	96.5785321	0.49935932	0.16833199	2.96651467	0.00301196	0.01307981
ENSG000002	1527.53388	-0.3129363	0.05046359	-6.2012296	5.60E-10	3.38E-08
ENSG000002	525.008117	-0.4282283	0.12563148	-3.4086068	0.00065296	0.00379589
ENSG000002	13.402034	1.52832384	0.61426859	2.48803842	0.01284499	0.04249493
ENSG000002	153.854284	-0.547369	0.16049803	-3.4104409	0.00064858	0.003775
ENSG000002	101.305767	-0.1708857	0.10166022	-1.6809499	0.09277264	0.19058384
ENSG000002	72.6716266	0.26455045	0.12221451	2.16464024	0.03041525	0.08290229
ENSG000002	57.260518	0.53405255	0.19879191	2.68649036	0.0072207	0.02667848
ENSG000002	143.424906	0.61057905	0.22152212	2.75628933	0.00584613	0.02245303
ENSG000002	35.4504862	0.55833453	0.22040301	2.53324369	0.01130124	0.03844118
ENSG000002	855.736216	0.27660151	0.1507196	1.835206	0.06647517	0.14877581
ENSG000002	143.197983	0.28669146	0.17278834	1.65920607	0.09707427	0.19713289
ENSG000002	292.474049	-0.3578492	0.13274534	-2.6957569	0.00702289	0.02610742
ENSG000002	34.2559911	-1.0105127	0.20776681	-4.8636869	1.15E-06	2.02E-05
ENSG000002	3018.49054	0.2799191	0.1554289	1.8009463	0.07171134	0.15750186
ENSG000002	5416.46702	0.44968945	0.08589564	5.23530017	1.65E-07	4.04E-06
ENSG000002	6744.23751	0.22723673	0.13297108	1.70891844	0.08746606	0.18234577
ENSG000002	11856.0319	1.50739806	0.20404512	7.38757206	1.50E-13	2.52E-11
ENSG000002	2192.1107	0.36944543	0.13216879	2.79525456	0.00518589	0.02039622
ENSG000002	227.691203	0.69684142	0.26636328	2.61613175	0.00889322	0.03174199
ENSG000002	1397.71795	0.16314283	0.06042842	2.69976984	0.00693875	0.02586098
ENSG000002	47.5974012	1.11130162	0.2280714	4.87260407	1.10E-06	1.95E-05
ENSG000002	62240.601	0.23603788	0.07822397	3.01746233	0.00254901	0.01141226
ENSG000002	96.1286766	0.3138094	0.12924568	2.42800692	0.01518205	0.04857385
ENSG000002	167.863325	0.87344584	0.12666873	6.89551299	5.37E-12	5.80E-10
ENSG000002	479.465064	0.24327185	0.08034218	3.02794682	0.00246221	0.01108455
ENSG000002	766.963147	0.34938888	0.12810708	2.72731898	0.00638513	0.02410145
ENSG000002	112.337886	1.4281998	0.22760614	6.27487384	3.50E-10	2.27E-08
ENSG000002	296.128265	4.39167979	0.44123641	9.95312203	2.44E-23	2.21E-20
ENSG000002	95.3089021	1.02216461	0.22025828	4.64075444	3.47E-06	5.21E-05
ENSG000002	63.5425038	0.78508364	0.16037444	4.89531637	9.81E-07	1.77E-05
ENSG000002	250.361277	0.96534391	0.21780779	4.4320909	9.33E-06	0.00011827
ENSG000002	174.264819	-1.5531621	0.31025275	-5.0061186	5.55E-07	1.11E-05
ENSG000002	4531.30413	-0.1830013	0.0616633	-2.9677514	0.00299987	0.01303512
ENSG000002	1386.81291	-0.24047	0.07363683	-3.2656206	0.00109224	0.00581313
ENSG000002	74.2502962	-0.7293355	0.2658624	-2.7432818	0.00608285	0.02314133
ENSG000002	571567.631	-1.0186986	0.30456776	-3.3447353	0.00082361	0.00461038
ENSG000002	1809.67858	0.41972954	0.11899206	3.52737427	0.0004197	0.00264934

ENSG000002	37.1800315	-0.4758028	0.24017577	-1.9810608	0.04758446	0.11571714
ENSG000002	1254.96347	-0.2139134	0.08776212	-2.4374226	0.01479238	0.04758988
ENSG000002	21.1159661	0.75400274	0.25591051	2.94635322	0.00321545	0.01378988
ENSG000002	380.991637	-0.8715109	0.15100052	-5.7715756	7.85E-09	3.27E-07
ENSG000002	402.121815	0.1967996	0.05627696	3.49698324	0.00047055	0.00292068
ENSG000002	145.134339	-0.2211763	0.0820331	-2.6961832	0.00701391	0.02608072
ENSG000002	91.08417	0.4234053	0.15306508	2.76617832	0.00567175	0.02193451
ENSG000002	1384.88088	-0.5055596	0.22232507	-2.2739657	0.02296805	0.06685515
ENSG000002	85.0298163	-0.4971299	0.28693635	-1.7325441	0.08317673	0.17577829
ENSG000002	196.539604	-0.2892008	0.12918964	-2.2385758	0.02518353	0.07191816
ENSG000002	455.55019	-0.4255332	0.13038452	-3.2636785	0.00109976	0.00584239
ENSG000002	356.731904	-0.768051	0.16533089	-4.6455384	3.39E-06	5.10E-05
ENSG000002	1180.84591	-0.3516632	0.11184577	-3.1441801	0.00166553	0.00814877
ENSG000002	62845.0655	1.48256512	0.38017533	3.89968784	9.63E-05	0.00080029
ENSG000002	252.77515	1.38540456	0.34222563	4.0482198	5.16E-05	0.00048112
ENSG000002	628.594342	1.3994143	0.33382619	4.19204466	2.76E-05	0.00028844
ENSG000002	243.815624	1.41745261	0.32787832	4.32310565	1.54E-05	0.00017802
ENSG000002	660.455313	1.21093826	0.30712788	3.94278197	8.05E-05	0.00069197
ENSG000002	3446.75996	1.40781421	0.33451436	4.20853149	2.57E-05	0.0002722
ENSG000002	108.845473	1.57826422	0.37398484	4.22012888	2.44E-05	0.00026124
ENSG000002	48.3815293	1.85529113	0.39024149	4.75421292	1.99E-06	3.26E-05
ENSG000002	678.420978	1.44092729	0.36623639	3.93441864	8.34E-05	0.00071042
ENSG000002	470.254528	1.14880854	0.45567716	2.52110188	0.0116988	0.03949666
ENSG000002	223.861639	1.33669184	0.43262832	3.0897003	0.00200359	0.00942347
ENSG000002	1140.29619	2.37190854	0.40269363	5.89010694	3.86E-09	1.79E-07
ENSG000002	205.644969	0.68973281	0.33459363	2.06140448	0.03926447	0.10087327
ENSG000002	5.17116875	1.03321971	0.32831524	3.14703552	0.00164935	0.00809365
ENSG000002	76.9806493	1.74776418	0.3669482	4.76297244	1.91E-06	3.15E-05
ENSG000002	24.1034258	2.05796933	0.37575658	5.47686829	4.33E-08	1.37E-06
ENSG000002	900.300887	1.82135826	0.38135083	4.77606997	1.79E-06	2.97E-05
ENSG000002	308.766509	1.03508705	0.36244396	2.85585403	0.00429212	0.01742713
ENSG000002	14.1065091	1.3555402	0.39021703	3.47381094	0.00051312	0.00312468
ENSG000002	52.3715123	1.98373486	0.38490085	5.15388541	2.55E-07	5.78E-06
ENSG000002	22.0900504	1.2870623	0.40186816	3.20269789	0.00136147	0.00693422
ENSG000002	73.5865871	2.16798338	0.39835407	5.44235277	5.26E-08	1.59E-06
ENSG000002	229.062673	2.29157724	0.42680805	5.36910508	7.91E-08	2.20E-06
ENSG000002	1394.20594	2.15842097	0.38221295	5.64716859	1.63E-08	5.94E-07
ENSG000002	1525.85796	1.73401487	0.34746601	4.99045904	6.02E-07	1.18E-05
ENSG000002	3112.79956	2.0657292	0.4088353	5.05271728	4.36E-07	9.01E-06
ENSG000002	1942.08461	1.68689204	0.386134	4.36867009	1.25E-05	0.00014979
ENSG000002	176.260555	1.42088616	0.35835879	3.96498207	7.34E-05	0.0006433
ENSG000002	52.6070667	1.1716094	0.33033583	3.54672211	0.00039006	0.00249812
ENSG000002	1537.75853	1.48229332	0.37524287	3.95022382	7.81E-05	0.00067529

ENSG000002	1349.85986	1.68653474	0.37804055	4.46125355	8.15E-06	0.00010521
ENSG000002	514.93105	2.15973812	0.40525836	5.32928705	9.86E-08	2.65E-06
ENSG000002	423.910456	2.26747091	0.41972	5.40234175	6.58E-08	1.90E-06
ENSG000002	28.6511684	1.36560309	0.40998551	3.33085696	0.00086579	0.00478547
ENSG000002	2050.1738	1.91714754	0.36203986	5.29540452	1.19E-07	3.09E-06
ENSG000002	346.736959	1.79870606	0.33955874	5.29718679	1.18E-07	3.08E-06
ENSG000002	8190.51544	1.86696932	0.36198198	5.15763055	2.50E-07	5.71E-06
ENSG000002	494.133887	1.65994849	0.3754636	4.42106369	9.82E-06	0.00012312
ENSG000002	4055.312	1.66424892	0.3573922	4.65664593	3.21E-06	4.88E-05
ENSG000002	133.457012	1.7084661	0.44774537	3.81570915	0.00013579	0.00105936
ENSG000002	822.350695	-0.717905	0.21127854	-3.3979078	0.00067903	0.00391606
ENSG000002	266.241221	-1.0039418	0.25934249	-3.8711044	0.00010834	0.00088129
ENSG000002	149.7406	-0.5202972	0.16995636	-3.0613577	0.00220336	0.01015591
ENSG000002	117.14	-0.3980242	0.18831027	-2.1136616	0.03454418	0.09153861
ENSG000002	87.6970539	-0.2992235	0.16235881	-1.8429766	0.06533243	0.14711575
ENSG000002	95.5364734	-0.4217171	0.17497703	-2.4101284	0.01594691	0.05038666
ENSG000002	260.857795	-0.3641284	0.16342797	-2.2280668	0.02587606	0.0733594
ENSG000002	136.801405	-0.3048423	0.15809172	-1.9282625	0.05382248	0.12676416
ENSG000002	163.219232	-0.3423009	0.15840415	-2.1609338	0.03070046	0.08344217
ENSG000002	286.034653	0.45485153	0.26181326	1.7373128	0.08233197	0.1743267
ENSG000002	311.609383	-0.3416743	0.16889422	-2.0230075	0.04307237	0.10764006
ENSG000002	161.797759	-0.4001805	0.22889073	-1.7483474	0.08040389	0.17101912
ENSG000002	158.789781	0.65852804	0.25997636	2.53303048	0.01130811	0.03845554
ENSG000002	111.534065	-0.4408366	0.15840008	-2.7830577	0.00538492	0.02102166
ENSG000002	180.831103	-0.4587759	0.17118182	-2.6800507	0.0073611	0.02707981
ENSG000002	128.012771	-0.4257245	0.17850926	-2.3848874	0.01708435	0.05324892
ENSG000002	67.866223	-0.3222268	0.17341992	-1.8580725	0.06315872	0.14348131
ENSG000002	159.758639	-1.4927849	0.37396747	-3.9917506	6.56E-05	0.00058292
ENSG000002	83.680805	-0.4942425	0.21729364	-2.2745372	0.02293371	0.06680889
ENSG000002	1152.4293	-0.5419097	0.20275027	-2.672794	0.00752224	0.02757458
ENSG000002	657.965111	1.4176896	0.44410118	3.19226716	0.00141161	0.00715033
ENSG000002	712.679624	0.7970182	0.3985189	1.99995082	0.04550557	0.11205835
ENSG000002	3730.67748	1.79101416	0.4008155	4.46842542	7.88E-06	0.00010238
ENSG000002	4628.72814	2.19862364	0.46396623	4.73875793	2.15E-06	3.50E-05
ENSG000002	823.737875	1.30819589	0.40718609	3.21277157	0.00131461	0.00673911
ENSG000002	62.3665481	1.52530239	0.45694085	3.33807405	0.00084361	0.00469513
ENSG000002	84.6762488	1.04279204	0.34630968	3.01115473	0.00260256	0.01160816
ENSG000002	23.8804819	0.95400569	0.36919732	2.58399953	0.00976619	0.03418413
ENSG000002	82.6767259	1.11243827	0.35622852	3.12282202	0.00179126	0.00865577
ENSG000002	49.6310522	1.25182906	0.37349033	3.35170407	0.00080316	0.00450983
ENSG000002	33.5399091	0.97059625	0.3693341	2.62796274	0.00858979	0.03081791
ENSG000002	36.6731677	0.96249911	0.34536501	2.78690393	0.00532143	0.02083554
ENSG000002	18.9062061	1.2811248	0.38177801	3.35567994	0.0007917	0.00446453

ENSG000002	121.023424	1.08678301	0.38471011	2.82494006	0.00472895	0.01890498
ENSG000002	32.7683915	0.96745462	0.35208714	2.74777043	0.0060002	0.02290397
ENSG000002	78.6473947	1.16362308	0.41293258	2.81794928	0.00483314	0.01927271
ENSG000002	37.0085336	1.26251864	0.39109743	3.22814358	0.00124596	0.00645806
ENSG000002	17.9470855	0.95787343	0.39416296	2.43014572	0.01509275	0.04839502
ENSG000002	38.2647587	1.12238958	0.40056915	2.80198706	0.00507889	0.02006487
ENSG000002	65.2322363	1.25090356	0.36222029	3.45343313	0.0005535	0.00331482
ENSG000002	8.3101978	1.48406229	0.45716441	3.24623322	0.00116943	0.00614269
ENSG000002	194.507792	0.39783206	0.14494292	2.74474988	0.0060557	0.02305018
ENSG000002	335.538981	-0.2541185	0.11155667	-2.2779321	0.02273063	0.06632398
ENSG000002	224.102624	0.60581594	0.19188901	3.15711646	0.00159338	0.0078675
ENSG000002	65.2693496	-0.3767988	0.21040487	-1.7908276	0.07332097	0.16006644
ENSG000002	13945.8191	-1.0110121	0.17189103	-5.8817034	4.06E-09	1.87E-07
ENSG000002	216.473393	0.24713538	0.07467185	3.30961925	0.00093423	0.0051111
ENSG000002	1311.72465	-0.2792746	0.09162772	-3.0479271	0.00230426	0.01051395
ENSG000002	513.043884	0.24094697	0.07652384	3.14865217	0.00164025	0.0080605
ENSG000002	3258.95139	-0.2061926	0.07511584	-2.744995	0.00605118	0.02303902
ENSG000002	983.958933	0.33103358	0.14240759	2.32455019	0.02009603	0.06063974
ENSG000002	79.9940148	0.46892149	0.15123353	3.10064495	0.001931	0.00916799
ENSG000002	887.942715	0.74667262	0.1832701	4.07416501	4.62E-05	0.00043726
ENSG000002	2009.00496	-0.17885	0.07630589	-2.3438556	0.01908556	0.05823347
ENSG000002	267.620707	-0.2965656	0.09830434	-3.0168107	0.00255449	0.01142542
ENSG000002	106.718416	-0.2228557	0.13301454	-1.6754234	0.09385119	0.19225427
ENSG000002	2404.6235	0.50957452	0.13982233	3.64444312	0.00026797	0.00183986
ENSG000002	50.7451781	0.92789759	0.25239617	3.67635371	0.00023659	0.00165181
ENSG000002	2166.28263	0.28930069	0.09084858	3.18442739	0.00145041	0.00730346
ENSG000002	56.8571459	0.2121268	0.11000231	1.92838501	0.05380726	0.12676416
ENSG000002	2070.96648	0.35509423	0.1139452	3.11635966	0.00183099	0.00879494
ENSG000002	2923.76612	0.60436535	0.15919712	3.79633339	0.00014685	0.00112725
ENSG000002	604.057857	-0.540279	0.19718874	-2.7399082	0.00614564	0.0233618
ENSG000002	453.576674	1.3552428	0.25747566	5.26357642	1.41E-07	3.53E-06
ENSG000002	1900.56818	0.84929193	0.19220935	4.41857759	9.94E-06	0.00012388
ENSG000002	593.859752	-0.2509066	0.11221666	-2.2359127	0.02535749	0.07217666
ENSG000002	66.4383929	1.20796484	0.27767081	4.35034873	1.36E-05	0.00016035
ENSG000002	3452.61099	0.36414958	0.10481153	3.47432732	0.00051214	0.00312236
ENSG000002	135.360683	0.44143553	0.07717494	5.71993381	1.07E-08	4.24E-07
ENSG000002	1507.27395	-0.3561889	0.09359518	-3.8056335	0.00014144	0.00109342
ENSG000002	1549.01947	-0.0826839	0.04967503	-1.6644965	0.09601325	0.19543965
ENSG000002	494.244996	0.34430687	0.09901318	3.4773843	0.00050633	0.00309229
ENSG000002	3679.01783	0.2789146	0.09127528	3.05575197	0.00224497	0.0102953
ENSG000002	11704.2589	-0.4346579	0.11801501	-3.6830728	0.00023044	0.00161743
ENSG000002	754.611628	-0.1816741	0.07833859	-2.319088	0.02039026	0.06128501
ENSG000002	92.8177133	0.72881511	0.14892829	4.89373194	9.89E-07	1.78E-05

ENSG000002	8972.86128	0.61805341	0.11256533	5.49061978	4.01E-08	1.29E-06
ENSG000002	694.375318	0.47764124	0.16175188	2.95292535	0.00314778	0.01359222
ENSG000002	4868.68091	0.6223736	0.19148969	3.25016769	0.00115337	0.00606997
ENSG000002	1075.53794	-0.2256253	0.06603465	-3.4167709	0.00063369	0.00371067
ENSG000002	67.6375111	-0.5655561	0.22221045	-2.5451372	0.01092348	0.03741098
ENSG000002	317.106047	0.25030792	0.13582548	1.84286429	0.06534883	0.14711864
ENSG000002	180.745812	0.48484182	0.21004429	2.30828377	0.02098336	0.06260006
ENSG000002	2838.5161	0.20945002	0.07756001	2.70048998	0.00692374	0.0258117
ENSG000002	1171.29869	-0.151177	0.06719619	-2.2497859	0.02446254	0.07037109
ENSG000002	212.331947	3.90134956	0.78977542	4.93982147	7.82E-07	1.46E-05
ENSG000002	1108.8601	0.35675404	0.20130139	1.77223829	0.07635501	0.16477805
ENSG000002	399.338145	0.98952964	0.18254206	5.42083101	5.93E-08	1.74E-06
ENSG000002	427.127901	-0.2389067	0.06883943	-3.4704915	0.00051951	0.00315163
ENSG000002	207.67616	-0.2037872	0.11262971	-1.809356	0.07039571	0.15538972
ENSG000002	4516.00023	0.18778986	0.06920683	2.71345857	0.00665849	0.02495771
ENSG000002	75.9221373	0.3309057	0.1443626	2.29218434	0.02189501	0.0645357
ENSG000002	1030.16149	0.19630161	0.06060773	3.23888721	0.00119997	0.00627354
ENSG000002	3854.77417	0.32219634	0.09102476	3.53965615	0.00040065	0.00255241
ENSG000002	4831.64918	-0.2617087	0.07006801	-3.7350667	0.00018767	0.00136687
ENSG000002	460.880505	0.26251949	0.11001919	2.38612458	0.01702698	0.05312726
ENSG000002	1177.30593	0.21968635	0.06052841	3.62947508	0.000284	0.00192797
ENSG000002	8690.44094	-0.2046486	0.09107467	-2.2470416	0.02463737	0.07075485
ENSG000002	910.576129	0.17755318	0.10459204	1.69757836	0.08958737	0.18559306
ENSG000002	4356.84149	0.61863533	0.11297254	5.4759799	4.35E-08	1.37E-06
ENSG000002	213.438188	0.58996432	0.12085407	4.88162544	1.05E-06	1.88E-05
ENSG000002	7399.50396	0.34380858	0.14411288	2.38568943	0.01704714	0.05317871
ENSG000002	148.749851	-0.2008352	0.10758113	-1.866826	0.06192589	0.14147931
ENSG000002	3677.33802	0.20177463	0.12129646	1.66348327	0.09621574	0.1957968
ENSG000002	117.038253	0.64694253	0.17155594	3.77102969	0.00016258	0.00122159
ENSG000002	436.08922	0.40798673	0.14237717	2.86553471	0.00416306	0.01700795
ENSG000002	214.429573	-0.2335691	0.09505227	-2.4572704	0.01399973	0.04560694
ENSG000002	139.63284	0.18599615	0.08679126	2.14302849	0.03211081	0.08641823
ENSG000002	1723.53001	0.5161602	0.21827921	2.36467871	0.01804572	0.05564886
ENSG000002	1033.75415	-0.1242345	0.06532552	-1.9017754	0.05720052	0.13301188
ENSG000002	1697.54112	0.37475805	0.17243179	2.1733698	0.0297525	0.08141753
ENSG000002	127.665668	-0.7774889	0.44126337	-1.7619611	0.07807586	0.16727078
ENSG000002	24.9969254	-0.7330672	0.33915567	-2.1614477	0.03066078	0.08338326
ENSG000002	75.8699267	0.20097597	0.11456652	1.75422956	0.07939118	0.16927735
ENSG000002	5878.40456	0.19604771	0.09870491	1.98620017	0.0470111	0.11477079
ENSG000002	65.8149037	1.62227051	0.26326668	6.16208073	7.18E-10	4.19E-08
ENSG000002	800.530337	0.72118191	0.20880967	3.45377641	0.0005528	0.00331197
ENSG000002	79.379583	-0.3181253	0.15085902	-2.1087592	0.03496537	0.09235065
ENSG000002	2872.34051	-0.3932267	0.08471022	-4.6420215	3.45E-06	5.18E-05

ENSG000002	109.608001	0.2010214	0.09053903	2.22027352	0.02640021	0.07437958
ENSG000002	506.2315	-0.1972669	0.08820295	-2.2365109	0.02531832	0.07213108
ENSG000002	198.893409	0.21878263	0.12208735	1.79201712	0.07313022	0.15977572
ENSG000002	5.36481961	0.71479633	0.29969825	2.38505344	0.01707664	0.05323634
ENSG000002	239.66208	0.45901551	0.16507732	2.78060923	0.0054257	0.02114666
ENSG000002	1487.56807	3.72387624	0.3647597	10.2091219	1.80E-24	1.87E-21
ENSG000002	30.6538729	0.60663631	0.2578771	2.35242414	0.01865149	0.05724665
ENSG000002	80.790448	1.23314275	0.27963115	4.40989053	1.03E-05	0.00012828
ENSG000002	1172.19301	-0.216834	0.06515522	-3.3279609	0.00087484	0.00482997
ENSG000002	266.94054	0.6538001	0.16928685	3.86208442	0.00011242	0.00090937
ENSG000002	639.364161	0.20896035	0.07053004	2.96271427	0.00304939	0.01322256
ENSG000002	527.255233	0.3514922	0.05302564	6.62872177	3.39E-11	3.01E-09
ENSG000002	401.609806	0.39962203	0.12904422	3.09678351	0.00195633	0.00925582
ENSG000002	6997.28844	0.42057957	0.16751685	2.51067018	0.01205022	0.04039139
ENSG000002	164.319954	0.50800972	0.16757359	3.03156191	0.00243292	0.01098302
ENSG000002	16.1493102	-1.4168174	0.35974288	-3.9384167	8.20E-05	0.00070144
ENSG000002	143.996374	-0.3698335	0.15989225	-2.3130168	0.02072172	0.06197196
ENSG000002	136.962594	0.85154904	0.25400983	3.3524255	0.00080107	0.00450507
ENSG000002	411.755568	0.74684104	0.18805427	3.97141236	7.14E-05	0.00062922
ENSG000002	1137.55795	-0.1318096	0.05873185	-2.2442616	0.02481558	0.07111835
ENSG000002	674.299496	-0.2141363	0.05800155	-3.691906	0.00022258	0.0015714
ENSG000002	3037.63713	0.11965818	0.05718454	2.09249177	0.03639455	0.09520545
ENSG000002	603.717386	0.36799759	0.09173749	4.01141996	6.04E-05	0.00054655
ENSG000002	86.2392588	0.23803055	0.1354771	1.75697994	0.07892123	0.16845909
ENSG000002	2142.4067	0.39934702	0.1109111	3.60060468	0.00031748	0.00211171
ENSG000002	427.471804	0.13786913	0.07707957	1.78865989	0.07366961	0.16058811
ENSG000002	5047.38369	0.18011245	0.09137518	1.97113105	0.04870889	0.11764682
ENSG000002	310.360319	0.32499631	0.10510521	3.09210471	0.00198743	0.0093693
ENSG000002	1194.60792	-0.4273223	0.15487678	-2.7591118	0.00579587	0.02231502
ENSG000002	268.597196	0.54486565	0.12057703	4.51881804	6.22E-06	8.43E-05
ENSG000002	25.8374303	1.11111099	0.32529987	3.41565149	0.0006363	0.00372271
ENSG000002	135.399039	0.190134	0.07717545	2.46365899	0.01375269	0.04496394
ENSG000002	26.8261097	0.60286978	0.27411608	2.19932289	0.02785497	0.0775142
ENSG000002	4294.54092	0.40092231	0.16691092	2.40201369	0.0163051	0.05132811
ENSG000002	115.15866	1.64949607	0.25962855	6.35329221	2.11E-10	1.54E-08
ENSG000002	113.548417	0.89618319	0.16976164	5.2790678	1.30E-07	3.33E-06
ENSG000002	46.8314456	-0.3543159	0.18799633	-1.8846957	0.05947093	0.13712484
ENSG000002	3.38556248	0.96584619	0.37571569	2.57068365	0.0101498	0.03529355
ENSG000002	100.266289	0.5990698	0.1939313	3.08908262	0.00200776	0.00942832
ENSG000002	989.998928	-0.1108294	0.06142732	-1.804237	0.07119416	0.15667475
ENSG000002	214.899651	-0.3593851	0.14668993	-2.4499642	0.01428704	0.04640726
ENSG000002	449.718499	0.15083277	0.08260576	1.82593528	0.06786001	0.15098879
ENSG000002	225.14605	-0.2952941	0.1259021	-2.3454265	0.01900533	0.05803756

ENSG000002	31.4014819	-0.6969634	0.23706479	-2.9399703	0.00328244	0.0140108
ENSG000002	183.238918	0.3999499	0.10684314	3.74333729	0.00018159	0.00133269
ENSG000002	1526.93948	0.64440309	0.23466077	2.74610494	0.00603075	0.02298538
ENSG000002	8813.74052	-0.179494	0.07705524	-2.3294198	0.01983684	0.05999477
ENSG000002	397.971774	1.17369041	0.63113593	1.8596476	0.0629354	0.14306373
ENSG000002	391.831107	0.52429382	0.16281802	3.22012149	0.00128136	0.00659904
ENSG000002	3043.82583	-0.3253888	0.1158772	-2.8080488	0.00498427	0.01979903
ENSG000002	459.530115	-0.1241603	0.07325765	-1.6948443	0.09010495	0.18647864
ENSG000002	163.898707	0.39473461	0.21683322	1.82045263	0.0686901	0.15233247
ENSG000002	11810.5169	0.44090544	0.23850842	1.84859486	0.06451634	0.14585645
ENSG000002	329.353859	-0.4022532	0.14505941	-2.7730239	0.0055538	0.02154162
ENSG000002	65.7594692	0.85081494	0.32762149	2.59694482	0.0094057	0.03325047
ENSG000002	36.9618154	1.44771175	0.26142382	5.53779583	3.06E-08	1.03E-06
ENSG000002	14798.2408	1.15703629	0.20025548	5.77780075	7.57E-09	3.17E-07
ENSG000002	233.140735	0.41031134	0.15677202	2.61724859	0.00886418	0.03166839
ENSG000002	62.9938147	1.14235373	0.34532939	3.30801189	0.00093961	0.00513858
ENSG000002	533.908829	0.93959332	0.12575776	7.47145398	7.93E-14	1.38E-11
ENSG000002	46.4923178	1.00832526	0.27847269	3.62091255	0.00029357	0.00197901
ENSG000002	114.661154	0.82911237	0.28293973	2.93034979	0.00338581	0.01438422
ENSG000002	38.8879168	1.12620993	0.24690304	4.561345	5.08E-06	7.16E-05
ENSG000002	235.005489	1.91683602	0.37716043	5.08228293	3.73E-07	7.89E-06
ENSG000002	16.3478856	2.79344853	0.44848129	6.22868463	4.70E-10	2.95E-08
ENSG000002	128.515586	0.36686618	0.09093936	4.03418486	5.48E-05	0.00050387
ENSG000002	484.583788	-0.1119476	0.05608833	-1.9959159	0.04594308	0.11277004
ENSG000002	52.5299026	0.22252797	0.11554555	1.92588959	0.05411816	0.12733633
ENSG000002	320.724032	0.37655307	0.10706884	3.51692495	0.00043658	0.00273796
ENSG000002	99.8946999	0.21167111	0.12459445	1.69888073	0.08934166	0.18529601
ENSG000002	56.1871787	0.78421015	0.19724712	3.97577491	7.02E-05	0.0006193
ENSG000002	422.62156	1.4380332	0.50130497	2.86857956	0.00412319	0.01686443
ENSG000002	1383.23663	0.27412145	0.10755233	2.54872633	0.01081171	0.03712473
ENSG000002	332.903342	0.27734353	0.09407942	2.94797237	0.00319866	0.01375043
ENSG000002	2660.45842	-0.4130898	0.14418154	-2.865067	0.00416921	0.01702349
ENSG000002	1379.31384	0.38910524	0.12318734	3.15864642	0.00158504	0.00783433
ENSG000002	63.0474325	1.43527159	0.22934035	6.25826029	3.89E-10	2.50E-08
ENSG000002	336.542528	1.22829362	0.24856021	4.94163407	7.75E-07	1.45E-05
ENSG000002	691.601291	0.86428043	0.16043498	5.38710704	7.16E-08	2.04E-06
ENSG000002	241.177469	0.46243877	0.2055131	2.2501669	0.02443835	0.07032944
ENSG000002	44.7707234	1.36056444	0.40969746	3.32090036	0.00089728	0.00494252
ENSG000002	84.009874	0.59470389	0.25071011	2.37207779	0.01768837	0.05469613
ENSG000002	580.593001	0.39848738	0.10177568	3.91534978	9.03E-05	0.00075814
ENSG000002	137.557057	0.8111479	0.19689059	4.11979016	3.79E-05	0.0003717
ENSG000002	36.518952	1.5593697	0.27383442	5.69457155	1.24E-08	4.76E-07
ENSG000002	403.213257	-0.3123832	0.17541347	-1.7808391	0.07493873	0.16251623

ENSG000002	58.6218396	1.24512267	0.39095981	3.18478429	0.00144862	0.00729953
ENSG000002	12671.7303	0.29828873	0.17381602	1.71611761	0.08614052	0.18023075
ENSG000002	135.379439	-0.2185729	0.09454132	-2.3119298	0.02078155	0.06210032
ENSG000002	151.139493	0.85278631	0.1400381	6.08967376	1.13E-09	6.12E-08
ENSG000002	100.462827	0.26341377	0.15735714	1.6739868	0.09413319	0.19261406
ENSG000002	52.1892886	0.3637606	0.18869167	1.92780425	0.05387948	0.12685714
ENSG000002	69.2411511	0.34032211	0.11723166	2.902988	0.00369621	0.01542696
ENSG000002	140.330294	1.48969576	0.30064326	4.95502794	7.23E-07	1.37E-05
ENSG000002	405.22133	0.81546587	0.1431374	5.69708462	1.22E-08	4.72E-07
ENSG000002	211.977205	0.36683482	0.10815767	3.39166719	0.00069469	0.00399839
ENSG000002	2656.10508	0.93922571	0.20764423	4.52324502	6.09E-06	8.30E-05
ENSG000002	464.783305	0.32991853	0.11771526	2.80268267	0.00506795	0.02004352
ENSG000002	1960.78139	0.38707101	0.21726799	1.78153718	0.07482474	0.16237057
ENSG000002	147.595426	1.29509602	0.44482166	2.91149498	0.00359704	0.01509207
ENSG000002	1985.28109	0.78678984	0.24739267	3.18032807	0.00147108	0.00738192
ENSG000002	780.584828	0.65656527	0.15964288	4.11271253	3.91E-05	0.00037994
ENSG000002	130.012395	1.21195397	0.27089675	4.47385945	7.68E-06	0.00010008
ENSG000002	103.848764	1.06206416	0.28353004	3.74586116	0.00017978	0.00132405
ENSG000002	27.5176391	1.83626465	0.36102162	5.08630105	3.65E-07	7.74E-06
ENSG000002	220.543109	0.24996996	0.11204618	2.23095482	0.02568412	0.07292941
ENSG000002	70.2793977	-0.3579082	0.0955475	-3.7458672	0.00017977	0.00132405
ENSG000002	85.2641934	0.97498026	0.18876873	5.16494583	2.41E-07	5.52E-06
ENSG000002	340.179817	0.95417272	0.16613706	5.74328628	9.29E-09	3.77E-07
ENSG000002	152.844711	2.57496699	0.36816854	6.99398982	2.67E-12	3.13E-10
ENSG000002	122.249416	-0.2043318	0.11093142	-1.8419647	0.06548031	0.14722888
ENSG000002	24.8245983	2.14264978	0.48475117	4.42010235	9.87E-06	0.00012337
ENSG000002	112.703979	-0.3359153	0.20339474	-1.6515435	0.09862764	0.19936076
ENSG000002	214.054802	-0.3437921	0.11985531	-2.8683926	0.00412563	0.01686933
ENSG000002	2683.66628	-0.5930437	0.23349644	-2.5398405	0.0110903	0.03783919
ENSG000002	1616.99473	0.33625739	0.08565786	3.92558694	8.65E-05	0.00073096
ENSG000002	153.982234	-0.7739367	0.23440476	-3.3017108	0.00096097	0.00523171
ENSG000002	128.905913	0.88105553	0.49035493	1.79677101	0.07237198	0.15868819
ENSG000002	6586.72789	0.39109842	0.18330253	2.13362254	0.03287369	0.08794851
ENSG000002	94.9825774	0.18839813	0.09212719	2.04497861	0.04085697	0.10356866
ENSG000002	85.4402195	0.67017717	0.21362231	3.13720596	0.00170566	0.00830307
ENSG000002	1017.15228	-0.1471164	0.07392539	-1.9900659	0.04658368	0.11393851
ENSG000002	426.894689	0.16581028	0.05793459	2.86202553	0.00420943	0.01713941
ENSG000002	1732.52963	0.5065142	0.26939868	1.88016586	0.06008548	0.13814606
ENSG000002	491.023201	4.05866704	0.38582273	10.5195125	7.02E-26	9.25E-23
ENSG000002	5300.67658	0.30085269	0.17951038	1.67596263	0.09374551	0.19209211
ENSG000002	64.5187517	1.04603688	0.26129569	4.00326875	6.25E-05	0.00056106
ENSG000002	75.3559048	-0.3971768	0.19330015	-2.0547152	0.03990652	0.102113
ENSG000002	853.43703	1.20767859	0.18807035	6.42141939	1.35E-10	1.06E-08

ENSG000002	1196.87334	3.66130966	0.38143341	9.59881746	8.09E-22	5.58E-19
ENSG000002	91.1303849	0.96041974	0.50642138	1.89648341	0.05789615	0.13419865
ENSG000002	271.765807	0.3333855	0.09907148	3.36510048	0.00076516	0.0043351
ENSG000002	596.442763	0.88121783	0.24603371	3.58169553	0.00034137	0.0022408
ENSG000002	1275.96195	-0.4423575	0.12498002	-3.5394254	0.000401	0.00255241
ENSG000002	89.7273389	0.31816696	0.13862753	2.29512105	0.02172619	0.06423414
ENSG000002	338.940816	0.29945755	0.0940607	3.18366288	0.00145424	0.0073131
ENSG000002	96.7007301	0.67229335	0.16030591	4.19381521	2.74E-05	0.00028692
ENSG000002	99.3690555	-0.2877126	0.13648962	-2.1079448	0.03503576	0.09250284
ENSG000002	333.909797	0.7016443	0.18718411	3.74841812	0.00017795	0.00131397
ENSG000002	75.2156157	0.16638245	0.09829582	1.69267065	0.09051817	0.18714668
ENSG000002	59.78414	0.83079748	0.25256657	3.28941986	0.00100394	0.00543908
ENSG000002	92.8677753	-0.1920511	0.09654506	-1.989238	0.04667494	0.11414245
ENSG000002	105.589336	0.26881595	0.13298684	2.02137266	0.0432412	0.10798747
ENSG000002	63.7395849	-0.3470479	0.15188186	-2.2849855	0.02231368	0.06539718
ENSG000002	157.458749	0.61726119	0.13731942	4.49507566	6.95E-06	9.26E-05
ENSG000002	309.743443	-0.241822	0.12185124	-1.9845676	0.0471926	0.11503036
ENSG000002	79.4867302	-0.2411947	0.14127874	-1.7072255	0.08778014	0.18279013
ENSG000002	170.737094	0.70417032	0.16595281	4.24319607	2.20E-05	0.00023913
ENSG000002	631.884798	-0.2614359	0.12057562	-2.1682317	0.03014106	0.08232533
ENSG000002	96.5976239	-0.9114741	0.22621163	-4.0292982	5.59E-05	0.00051295
ENSG000002	503.895561	-0.3060459	0.09415488	-3.2504519	0.00115222	0.00606768
ENSG000002	227.664699	0.72688566	0.17133769	4.24241532	2.21E-05	0.00023978
ENSG000002	81.077014	0.19286369	0.08950413	2.1548021	0.03117732	0.08442353
ENSG000002	42.8061207	0.51147212	0.19392479	2.63747666	0.00835254	0.03011528
ENSG000002	17.9133496	1.75940366	0.336744	5.22475138	1.74E-07	4.23E-06
ENSG000002	53.4907292	1.00428963	0.18272021	5.49632476	3.88E-08	1.26E-06
ENSG000002	399.850009	0.32809564	0.16599985	1.97648163	0.04810025	0.11660448
ENSG000002	228.901704	0.29367176	0.14103002	2.08233512	0.03731187	0.09704435
ENSG000002	70.7764364	0.98131603	0.26214506	3.74340842	0.00018154	0.00133269
ENSG000002	159.464602	0.26165918	0.12812842	2.04216346	0.04113532	0.1041285
ENSG000002	101.32538	1.74632351	0.22881228	7.63212311	2.31E-14	4.71E-12
ENSG000002	1034.44174	0.51614528	0.18732351	2.75536837	0.00586261	0.02251037
ENSG000002	10882.8409	-0.8232669	0.17140907	-4.8029364	1.56E-06	2.65E-05
ENSG000002	222.654668	0.2252631	0.126741	1.77734978	0.07551071	0.16346116
ENSG000002	1542.91909	0.32234862	0.09611877	3.35364887	0.00079753	0.00448869
ENSG000002	204.832923	3.99319743	0.42359832	9.42684916	4.23E-21	2.66E-18
ENSG000002	204.589315	0.49578189	0.2069507	2.39565219	0.01659083	0.05200158
ENSG000002	159.984082	-0.1665942	0.08609604	-1.9349809	0.05299264	0.12531903
ENSG000002	33.4759762	0.55454823	0.30254873	1.83292205	0.06681416	0.14932687
ENSG000002	5545.13447	0.24634225	0.11689533	2.10737465	0.03508512	0.09257065
ENSG000002	121.79395	0.43624758	0.12532452	3.48094344	0.00049965	0.00305936
ENSG000002	96.2982994	0.41914603	0.21271756	1.97043456	0.04878859	0.11780005

ENSG000002	63.3120234	0.26566707	0.12670813	2.09668529	0.03602144	0.09450247
ENSG000002	275.702152	-0.3886518	0.12803244	-3.035573	0.00240079	0.01084849
ENSG000002	119.202379	0.24784783	0.13182882	1.88007316	0.06009811	0.13815317
ENSG000002	108.661407	0.27312646	0.14684714	1.85993716	0.06289442	0.14305365
ENSG000002	167.168	0.44237645	0.10660714	4.14959494	3.33E-05	0.00033624
ENSG000002	395.030574	-0.3280272	0.10405837	-3.152338	0.00161969	0.00797837
ENSG000002	418.532687	-0.4595081	0.17159212	-2.6779093	0.00740833	0.0272397
ENSG000002	21.110583	2.08085305	0.33315604	6.24588117	4.21E-10	2.68E-08
ENSG000002	3972.9165	0.6791587	0.16110948	4.21551055	2.49E-05	0.00026508
ENSG000002	1431.86836	0.24437743	0.12236661	1.99709238	0.04581515	0.11257192
ENSG000002	956.409671	0.79223497	0.38813798	2.04111684	0.04123921	0.10430037
ENSG000002	404.01775	0.17864733	0.09578308	1.86512421	0.062164	0.14184435
ENSG000002	47.019833	0.45843181	0.23979538	1.91176251	0.05590666	0.13061116
ENSG000002	29.3341867	0.69667459	0.27089079	2.57179135	0.01011738	0.03519946
ENSG000002	262.87874	-0.6110588	0.34659762	-1.7630208	0.07789697	0.1669616
ENSG000002	86.0498441	0.25920375	0.14511129	1.78624107	0.07406023	0.16107887
ENSG000002	27.1340756	0.75634167	0.2977286	2.54037286	0.01107343	0.03779054
ENSG000002	2366.70277	0.93913038	0.14571585	6.44494317	1.16E-10	9.15E-09
ENSG000002	51.9338166	0.98093505	0.22046809	4.44932899	8.61E-06	0.00011024
ENSG000002	143.675662	0.72630674	0.25959475	2.79784838	0.00514443	0.02025198
ENSG000002	49.9764409	1.15911596	0.16573955	6.99359918	2.68E-12	3.13E-10
ENSG000002	148.205236	0.22778207	0.12606045	1.80692735	0.07077361	0.15600993
ENSG000002	28.9382245	0.75621506	0.24185742	3.12669776	0.00176782	0.00856247
ENSG000002	40.5263149	1.36008341	0.23657345	5.7490957	8.97E-09	3.65E-07
ENSG000002	74.2296259	0.36045791	0.12945146	2.78450256	0.00536099	0.02095081
ENSG000002	1351.52335	0.44956024	0.19103411	2.35329822	0.0186077	0.05712435
ENSG000002	5580.11298	0.65886784	0.39704861	1.65941353	0.09703249	0.1971259
ENSG000002	260.321762	0.2549375	0.09841636	2.59039766	0.00958651	0.03369233
ENSG000002	89.4755838	-0.929843	0.25689574	-3.6195346	0.00029513	0.00198635
ENSG000002	14.3008674	1.05224722	0.50796326	2.07150263	0.03831185	0.09895922
ENSG000002	21.9160995	-0.5279098	0.26081717	-2.024061	0.04296388	0.10755447
ENSG000002	93.4973783	0.77062141	0.20598568	3.7411407	0.00018319	0.00134167
ENSG000002	205.42698	1.22038607	0.16043759	7.60660914	2.81E-14	5.58E-12
ENSG000002	39.2943747	1.56816238	0.29340065	5.34478164	9.05E-08	2.47E-06
ENSG000002	32.2630312	2.14770639	0.54132773	3.967479	7.26E-05	0.00063775
ENSG000002	842.110953	0.744913	0.25659747	2.90304112	0.00369558	0.01542696
ENSG000002	59.1756976	0.55857578	0.18732066	2.98192291	0.00286444	0.0125407
ENSG000002	51.2191956	1.10753561	0.17423115	6.35670272	2.06E-10	1.52E-08
ENSG000002	33.6630968	0.8833659	0.31242849	2.8274179	0.0046925	0.01877909
ENSG000002	58.9924874	0.49332232	0.2561927	1.92559086	0.05415548	0.12740345
ENSG000002	55.7264757	0.68026948	0.22386139	3.03879763	0.00237524	0.01075368
ENSG000002	981.14246	0.36831969	0.15071041	2.44389027	0.01452985	0.04699573
ENSG000002	16.9258253	-0.3742853	0.20855837	-1.7946311	0.0727125	0.15916984

ENSG000002	140.799585	0.3029047	0.14136583	2.14270087	0.03213713	0.08647298
ENSG000002	2354.60735	-0.1919539	0.07950685	-2.4143066	0.01576519	0.04992501
ENSG000002	1.70751087	1.04196996	0.50686783	2.05570348	0.03981111	0.10195218
ENSG000002	410.04379	0.24078942	0.10797554	2.23003686	0.025745	0.07307362
ENSG000002	47.6819789	1.44868874	0.26234239	5.52212982	3.35E-08	1.11E-06
ENSG000002	2598.20973	0.30513668	0.07312374	4.17288107	3.01E-05	0.00030903
ENSG000002	58.7810213	0.28765414	0.14302568	2.01120626	0.04430367	0.10970028
ENSG000002	126.633003	0.2456614	0.08990628	2.73241635	0.00628716	0.02381232
ENSG000002	227.776342	-0.399349	0.16440667	-2.4290318	0.0151392	0.04847958
ENSG000002	117.330495	-0.3036843	0.10302789	-2.9475928	0.00320259	0.01375915
ENSG000002	335.515812	1.56443405	0.22365795	6.99476163	2.66E-12	3.13E-10
ENSG000002	30.9791151	-0.4977464	0.27860617	-1.786559	0.07400879	0.16107887
ENSG000002	49.4550385	1.92415372	0.37899477	5.0769928	3.83E-07	8.04E-06
ENSG000002	3626.81425	-0.3872757	0.1389229	-2.7877022	0.00530833	0.02079583
ENSG000002	497.487758	1.17751131	0.47159317	2.49687951	0.01252915	0.0416498
ENSG000002	13.0567412	0.89760985	0.39936668	2.24758319	0.02460278	0.07070432
ENSG000002	22.7009166	1.36540071	0.32539617	4.196118	2.72E-05	0.00028483
ENSG000002	6985.98063	0.43261887	0.16829375	2.57061755	0.01015174	0.03529355
ENSG000002	45.6637927	0.7751384	0.18482155	4.19398277	2.74E-05	0.00028692
ENSG000002	46.0389284	0.82276258	0.2607327	3.15557883	0.0016018	0.00790238
ENSG000002	208.396911	0.18917201	0.08784595	2.15345177	0.03128319	0.08467855
ENSG000002	869.705487	0.68084179	0.13513617	5.03819052	4.70E-07	9.64E-06
ENSG000002	205.673638	0.40622874	0.16447503	2.46985054	0.01351695	0.04431321
ENSG000002	101.920353	0.19143896	0.0958011	1.99829619	0.04568456	0.11236923
ENSG000002	164.715603	0.35169869	0.11055178	3.18130272	0.00146614	0.00736376
ENSG000002	253.947822	0.43940696	0.15935872	2.75734497	0.00582728	0.02240442
ENSG000002	110.03263	0.43683553	0.11679236	3.74027486	0.00018382	0.00134426
ENSG000002	151.638494	0.55960407	0.11406079	4.90619137	9.29E-07	1.70E-05
ENSG000002	237.635812	0.66266532	0.09591222	6.90908117	4.88E-12	5.31E-10
ENSG000002	13.1560059	-1.1931639	0.6016545	-1.983138	0.04735201	0.11533097
ENSG000002	54.9203559	0.50117641	0.25419827	1.97159645	0.04865569	0.11753794
ENSG000002	62.4161586	1.55969736	0.43167151	3.61315795	0.00030249	0.00202879
ENSG000002	55.6005073	-0.7182455	0.18880272	-3.8042114	0.00014226	0.00109738
ENSG000002	124.932921	0.39401663	0.22382389	1.76038685	0.07834224	0.16774224
ENSG000002	63.1127988	0.48808212	0.21104269	2.31271743	0.02073818	0.0619835
ENSG000002	92.9778832	1.09633553	0.22378784	4.89899504	9.63E-07	1.75E-05
ENSG000002	466.454919	0.41735216	0.09308643	4.48349087	7.34E-06	9.66E-05
ENSG000002	57.3317796	0.66857377	0.20435483	3.27163188	0.00106929	0.00571615
ENSG000002	35.5609993	0.75550697	0.17524412	4.31116884	1.62E-05	0.00018627
ENSG000002	508.764866	0.23505965	0.11088735	2.11980574	0.03402243	0.09038749
ENSG000002	122.027649	0.60876167	0.28387605	2.14446294	0.03199581	0.08618879
ENSG000002	698.604211	-0.2670857	0.14807659	-1.8036998	0.07127837	0.15683624
ENSG000002	9.38720041	1.75326092	0.53587524	3.27177073	0.00106876	0.00571558

ENSG000002	66.8287107	0.35830349	0.12955654	2.7656148	0.00568156	0.02196072
ENSG000002	88.9272626	0.39723369	0.19747454	2.01156913	0.04426538	0.10965672
ENSG000002	174.052295	-0.2864268	0.16189895	-1.7691702	0.07686547	0.16550982
ENSG000002	118.157126	1.24131678	0.22141724	5.60623368	2.07E-08	7.32E-07
ENSG000002	119.320197	0.32127841	0.10569496	3.03967561	0.00236833	0.01073866
ENSG000002	632.282643	1.01377009	0.16095381	6.29851585	3.01E-10	2.05E-08
ENSG000002	642.974971	0.78326892	0.15760365	4.96986547	6.70E-07	1.29E-05
ENSG000002	75.309647	0.23311264	0.10848457	2.14880924	0.03164952	0.0853989
ENSG000002	183.597821	0.60590017	0.23996674	2.52493398	0.011572	0.03918738
ENSG000002	142.110545	0.68355855	0.20898939	3.27078115	0.00107251	0.00572914
ENSG000002	71.2074247	1.09555478	0.2670157	4.10296009	4.08E-05	0.00039553
ENSG000002	134.458869	1.4326052	0.24791694	5.77856935	7.53E-09	3.16E-07
ENSG000002	36.1111589	0.52702028	0.30271834	1.74095919	0.08169073	0.17322214
ENSG000002	54.0814124	1.11198811	0.19439826	5.72015461	1.06E-08	4.24E-07
ENSG000002	152.594286	0.9565848	0.20284456	4.71585133	2.41E-06	3.85E-05
ENSG000002	145.640881	0.45707662	0.25749351	1.77509956	0.07588145	0.16395146
ENSG000002	1770.91057	-0.4900623	0.1979624	-2.4755321	0.01330378	0.04374304
ENSG000002	370.372965	-0.6860429	0.34361687	-1.9965345	0.04587577	0.11268266
ENSG000002	733.042015	0.36447756	0.19394193	1.87931281	0.06020179	0.13832567
ENSG000002	55.9275836	0.54636187	0.23554883	2.31952698	0.02036648	0.06122623
ENSG000002	103.955981	0.31742189	0.12717359	2.49597334	0.0125612	0.04173719
ENSG000002	284.842521	0.52819271	0.17229931	3.06555332	0.00217268	0.01004967
ENSG000002	38.542743	0.53970182	0.15862798	3.40231162	0.00066818	0.00386426
ENSG000002	236.687256	0.20340623	0.10619434	1.91541503	0.0554396	0.1296873
ENSG000002	75.5095608	-0.3580139	0.13325933	-2.6865951	0.00721844	0.02667692
ENSG000002	27.4083483	2.65184875	0.33905334	7.82133204	5.23E-15	1.18E-12
ENSG000002	67.7880639	0.90678864	0.22252955	4.07491329	4.60E-05	0.00043614
ENSG000002	98.0655259	0.40661106	0.15896696	2.55783375	0.01053264	0.0363474
ENSG000002	801.922435	-0.533673	0.26739134	-1.9958499	0.04595027	0.11277004
ENSG000002	71.5860497	0.37208357	0.12404247	2.99964656	0.00270293	0.01197473
ENSG000002	91.5321571	0.56012632	0.14265167	3.92653187	8.62E-05	0.0007295
ENSG000002	20.5386703	4.25547838	0.51316237	8.29265473	1.11E-16	3.51E-14
ENSG000002	49.7101769	0.58358217	0.20241355	2.88311815	0.0039376	0.01621489
ENSG000002	2237.79034	0.72985588	0.14011521	5.20896964	1.90E-07	4.53E-06
ENSG000002	678.406512	1.5739601	0.36234553	4.34380991	1.40E-05	0.00016426
ENSG000002	254.327884	1.45400213	0.82366149	1.76529089	0.07751489	0.16638883
ENSG000002	143.292689	0.52744736	0.2347418	2.2469256	0.02464478	0.07075485
ENSG000002	36.7822226	-0.9630703	0.28103338	-3.4268896	0.00061054	0.00359839
ENSG000002	392.770821	0.93560107	0.23226159	4.02822127	5.62E-05	0.00051465
ENSG000002	183.328064	0.41835189	0.13958841	2.99703891	0.00272616	0.01204081
ENSG000002	444.211372	0.81984551	0.15219836	5.38669086	7.18E-08	2.04E-06
ENSG000002	21.3997513	1.40038515	0.29971344	4.67241354	2.98E-06	4.56E-05
ENSG000002	22.2395769	0.96005041	0.34050241	2.81951136	0.00480968	0.0192003

ENSG000002	207.790608	0.24782341	0.10755603	2.30413305	0.02121518	0.06316158
ENSG000002	2483.84258	0.16162582	0.07960796	2.03027213	0.04232889	0.10635077
ENSG000002	71.1690903	0.29171038	0.14572775	2.00174895	0.04531174	0.11173296
ENSG000002	285.212625	0.24119851	0.11549025	2.08847504	0.036755	0.09590594
ENSG000002	393.095505	0.22025963	0.08828544	2.49485787	0.01260076	0.04183983
ENSG000002	1519.16916	0.23453588	0.12446406	1.88436625	0.05951545	0.13720566
ENSG000002	79530.6794	0.46028439	0.20199704	2.27866896	0.02268675	0.0662093
ENSG000002	83.3360831	0.21341074	0.11530952	1.85076426	0.06420347	0.14535329
ENSG000002	309.318896	0.25536458	0.08375773	3.0488479	0.00229721	0.01048508
ENSG000002	227.059716	0.81761093	0.14877601	5.49558312	3.89E-08	1.26E-06
ENSG000002	59.4532349	3.79339109	0.63757748	5.94969433	2.69E-09	1.31E-07
ENSG000002	550.786427	-0.2828866	0.0738111	-3.8325749	0.00012681	0.00100369
ENSG000002	81.4432596	-0.4727673	0.10540072	-4.4854271	7.28E-06	9.59E-05
ENSG000002	58.0535682	0.48083871	0.16453595	2.92239302	0.00347353	0.01467954
ENSG000002	983.191536	-0.2653777	0.09950882	-2.6668767	0.00765598	0.02802226
ENSG000002	188.380651	1.02716672	0.1969717	5.21479353	1.84E-07	4.42E-06
ENSG000002	162.842357	-0.2236382	0.09581886	-2.333969	0.01959735	0.05939472
ENSG000002	195.486711	0.30631707	0.11831469	2.58900278	0.00962543	0.03378813
ENSG000002	21.4720138	1.94752519	0.36257749	5.37133513	7.82E-08	2.18E-06
ENSG000002	48.7788076	1.08196596	0.23036448	4.69675682	2.64E-06	4.13E-05
ENSG000002	98.7826182	-0.1826793	0.09760462	-1.8716254	0.06125845	0.14035287
ENSG000002	1129.37808	-0.4014124	0.09677904	-4.1477203	3.36E-05	0.00033854
ENSG000002	26.1657777	0.57627155	0.21471171	2.68393162	0.0072762	0.02684245
ENSG000002	56.4508816	0.85417051	0.17740131	4.81490532	1.47E-06	2.51E-05
ENSG000002	51.7565363	0.36188408	0.19209586	1.88387241	0.05958224	0.13723983
ENSG000002	137.419744	0.78029652	0.21473831	3.63370891	0.00027938	0.00190373
ENSG000002	37.2605004	0.37969634	0.20048325	1.89390553	0.05823755	0.13488208
ENSG000002	129.442427	0.46925236	0.14388382	3.26132822	0.00110892	0.00587596
ENSG000002	146.853896	-0.5720599	0.27888572	-2.0512341	0.04024415	0.10273844
ENSG000002	208.429425	0.20559543	0.10440248	1.96925816	0.04892345	0.11800767
ENSG000002	310.879398	0.36201231	0.116419	3.10956386	0.00187364	0.008955
ENSG000002	16554.1801	0.45445474	0.14304977	3.17689947	0.00148859	0.00744139
ENSG000002	396.552785	0.18608463	0.07142236	2.60541116	0.00917641	0.03259098
ENSG000002	37.9646943	0.49483277	0.28812379	1.71743114	0.08590043	0.1799363
ENSG000002	29.546362	-0.9730241	0.3886717	-2.5034601	0.01229855	0.04107203
ENSG000002	110.686803	0.66215343	0.20307361	3.26065722	0.00111154	0.00588772
ENSG000002	100.26108	0.20779877	0.09321626	2.22921164	0.02579983	0.0732149
ENSG000002	8.93492366	1.37154401	0.37020461	3.70482696	0.00021154	0.00150961
ENSG000002	397.902486	0.88777781	0.22064052	4.02363906	5.73E-05	0.00052277
ENSG000002	154.972251	0.6125156	0.08854379	6.91765771	4.59E-12	5.09E-10
ENSG000002	57.9637645	-0.7884522	0.36059545	-2.1865285	0.02877697	0.07945338
ENSG000002	104.719911	0.56078423	0.15072422	3.720598	0.00019875	0.00143392
ENSG000002	85.6394277	0.39144785	0.23308693	1.67940716	0.09307272	0.19114609

ENSG000002	142.626419	1.63984448	0.33470487	4.89937438	9.61E-07	1.75E-05
ENSG000002	28.5004658	0.71649361	0.25180818	2.84539447	0.00443565	0.0178995
ENSG000002	309.214858	0.26497845	0.09385467	2.82328455	0.00475344	0.0189967
ENSG000002	40.0709005	0.91250239	0.26163042	3.4877534	0.0004871	0.00300024
ENSG000002	345.05975	-0.3168894	0.13620605	-2.3265445	0.01998952	0.0603812
ENSG000002	48.819912	0.55196471	0.1878267	2.93869135	0.00329601	0.01405632
ENSG000002	742.428391	-0.4131387	0.21403927	-1.9302004	0.05358201	0.12642388
ENSG000002	25.6458061	1.40711058	0.33735407	4.17102003	3.03E-05	0.0003109
ENSG000002	98.3552098	0.66758298	0.1716628	3.88892039	0.00010069	0.0008274
ENSG000002	24.9083232	0.53281661	0.18842035	2.82780824	0.00468679	0.01876139
ENSG000002	385.591429	0.52665283	0.20687348	2.54577254	0.01090362	0.03737353
ENSG000002	24.1904129	0.91457669	0.31925012	2.86476537	0.00417318	0.01703246
ENSG000002	93.3807171	0.8189443	0.21983816	3.72521445	0.00019515	0.00141145
ENSG000002	289.721959	0.20986399	0.10244007	2.04865143	0.04049621	0.10305087
ENSG000002	182.306233	0.18715038	0.1041278	1.79731415	0.07228576	0.15854713
ENSG000002	27.6688332	1.54456514	0.34127207	4.52590555	6.01E-06	8.22E-05
ENSG000002	77.5768753	0.84704107	0.24508409	3.45612418	0.000548	0.00329142
ENSG000002	531.242796	0.52993681	0.11756281	4.50769104	6.55E-06	8.83E-05
ENSG000002	31.0602078	1.09636996	0.23899863	4.58734825	4.49E-06	6.48E-05
ENSG000002	40.3583208	1.09421848	0.26597736	4.11395343	3.89E-05	0.00037892
ENSG000002	40.7229127	0.59452343	0.29792918	1.99551931	0.04598627	0.11280609
ENSG000002	2792.70789	0.2645272	0.11885833	2.22556726	0.02604318	0.07368898
ENSG000002	92.5335177	0.34113825	0.16542058	2.0622479	0.03918414	0.10070117
ENSG000002	41.0761169	1.01666679	0.2529931	4.01855545	5.86E-05	0.00053319
ENSG000002	29.6791321	-0.4945743	0.20244021	-2.4430634	0.01456318	0.04709302
ENSG000002	61.9793396	1.27979074	0.23658288	5.40948166	6.32E-08	1.84E-06
ENSG000002	1429.71114	0.25612748	0.11325768	2.2614579	0.02373092	0.06862072
ENSG000002	368.610788	1.19313526	0.21386171	5.57900355	2.42E-08	8.32E-07
ENSG000002	639.93694	1.11890401	0.23084118	4.84707284	1.25E-06	2.18E-05
ENSG000002	78.1951039	0.14679615	0.08339616	1.7602268	0.07836936	0.16777552
ENSG000002	95.7398467	0.28029296	0.06902692	4.0606328	4.89E-05	0.00046009
ENSG000002	86.4158954	0.47253917	0.22411786	2.10844045	0.03499291	0.09240654
ENSG000002	79.5036896	0.76912562	0.09195961	8.36373275	6.08E-17	2.10E-14
ENSG000002	530.336038	0.91330585	0.23350692	3.91125812	9.18E-05	0.00076798
ENSG000002	24.3031463	4.07144629	0.47244646	8.6177941	6.83E-18	2.83E-15
ENSG000002	29.8855889	3.97245839	0.48557263	8.18097668	2.82E-16	7.70E-14
ENSG000002	166.339249	0.21146544	0.09446812	2.23848483	0.02518946	0.07191952
ENSG000002	33.8545385	1.22458651	0.2993304	4.09108637	4.29E-05	0.00041275
ENSG000002	712.366563	0.78921328	0.18267223	4.32037916	1.56E-05	0.0001798
ENSG000002	251.228104	-1.0007252	0.25516413	-3.9218882	8.79E-05	0.00074129
ENSG000002	82.8118791	0.47556048	0.11222117	4.23770722	2.26E-05	0.00024413
ENSG000002	115.37904	0.21317157	0.08857604	2.4066504	0.01609957	0.05075958
ENSG000002	164.610965	1.41310177	0.30122231	4.69122544	2.72E-06	4.22E-05

ENSG000002	16.1536938	0.88430435	0.52389959	1.68792717	0.09142521	0.18854756
ENSG000002	3332.496	-0.3686566	0.07386577	-4.9908993	6.01E-07	1.18E-05
ENSG000002	233.957852	0.28174124	0.07673923	3.67141097	0.00024122	0.00168004
ENSG000002	777.916234	0.35390735	0.11500092	3.07743051	0.00208794	0.00972602
ENSG000002	257.294736	0.21189611	0.11219962	1.88856355	0.05895034	0.13619908
ENSG000002	388.947211	0.62460837	0.18362473	3.40154818	0.00067005	0.00387198
ENSG000002	1384.84836	0.23321231	0.09863443	2.3644108	0.01805878	0.0556612
ENSG000002	208.603512	0.77763818	0.15360524	5.0625759	4.14E-07	8.61E-06
ENSG000002	2006.75366	-0.1546443	0.08222912	-1.8806514	0.06001936	0.13805978
ENSG000002	5121.13474	0.46171232	0.1155996	3.99406505	6.50E-05	0.00057797
ENSG000002	169.015795	0.65441244	0.17010026	3.84721592	0.00011947	0.00095779
ENSG000002	627.014009	-0.2194716	0.07582217	-2.8945565	0.00379695	0.01578375
ENSG000002	2424.33647	2.75646755	0.50694138	5.43744832	5.40E-08	1.62E-06
ENSG000002	14.6530604	1.33179703	0.41113283	3.23933519	0.00119809	0.00626821
ENSG000002	91.00227	0.25700254	0.107035	2.40110745	0.01634554	0.05138841
ENSG000002	86.5566641	0.34862656	0.14677322	2.37527355	0.01753595	0.05430596
ENSG000002	56.117956	1.42635664	0.28070438	5.08134801	3.75E-07	7.91E-06
ENSG000002	114.992256	-0.2886628	0.1083567	-2.6640052	0.00772164	0.02823397
ENSG000002	31.8824923	1.03700596	0.22930874	4.52231324	6.12E-06	8.32E-05
ENSG000002	69.1298981	1.42921229	0.33453844	4.27219158	1.94E-05	0.00021438
ENSG000002	807.282776	0.20382488	0.07499359	2.7178973	0.00656982	0.02470847
ENSG000002	5690.88193	0.61130128	0.15151466	4.03460167	5.47E-05	0.00050373
ENSG000002	103.383774	0.32571173	0.08616662	3.78002232	0.00015681	0.00118568
ENSG000002	647.488343	0.21185174	0.11695303	1.81142591	0.07007495	0.15483311
ENSG000002	194.47721	-0.32826	0.10536558	-3.115439	0.00183671	0.00881367
ENSG000002	64.0335922	1.34445401	0.34181615	3.9332665	8.38E-05	0.00071328
ENSG000002	48.6013236	0.47241415	0.14178366	3.33193646	0.00086244	0.00477105
ENSG000002	3.26091544	-0.7621659	0.31290906	-2.4357424	0.01486126	0.04776906
ENSG000002	107.370757	-0.4709948	0.15103495	-3.1184492	0.00181805	0.00874474
ENSG000002	30.2306139	0.72309592	0.33166891	2.18017397	0.02924457	0.08046839
ENSG000002	1220.04542	0.32417246	0.06938903	4.6718114	2.99E-06	4.57E-05
ENSG000002	84.1386497	0.83664772	0.18050417	4.63506033	3.57E-06	5.32E-05
ENSG000002	393.655706	0.26488984	0.14745148	1.7964543	0.0724223	0.15875046
ENSG000002	1071.37897	-0.2126372	0.06632181	-3.2061431	0.00134527	0.00686956
ENSG000002	273.548828	0.48067649	0.11915281	4.03411797	5.48E-05	0.00050387
ENSG000002	22.3575811	1.26475549	0.39819242	3.17624197	0.00149196	0.00744709
ENSG000002	41.2903079	1.25771571	0.34545825	3.64071693	0.00027188	0.00186316
ENSG000002	1232.24903	0.37034168	0.10402446	3.56014024	0.00037066	0.00239671
ENSG000002	58.6120497	0.67672324	0.23884094	2.8333637	0.00460609	0.01848435
ENSG000002	14.4635542	1.34234473	0.39000966	3.4418243	0.00057781	0.00343623
ENSG000002	64.4735953	0.69369151	0.19659245	3.52857651	0.0004178	0.00264079
ENSG000002	47.5532695	1.79282257	0.43229186	4.14725038	3.36E-05	0.00033876
ENSG000002	276.950454	0.43060577	0.15772018	2.73018824	0.00632982	0.02393008

ENSG000002	59.4109599	0.2522563	0.12668736	1.9911718	0.046462	0.11372951
ENSG000002	914.141663	0.48882627	0.10592987	4.61462144	3.94E-06	5.79E-05
ENSG000002	120.024584	0.62869276	0.22697105	2.76992494	0.00560692	0.02171857
ENSG000002	169.065221	0.63842245	0.18403168	3.46909001	0.00052222	0.00316547
ENSG000002	54.0606813	0.74807304	0.26254711	2.84929071	0.00438168	0.01772123
ENSG000002	1196.66717	0.16777027	0.07916739	2.11918408	0.03407491	0.09049372
ENSG000002	142.184528	-0.1866292	0.10501946	-1.7770918	0.07555313	0.16350791
ENSG000002	86.9164136	-0.527161	0.17081269	-3.0861931	0.00202737	0.0094958
ENSG000002	50.1781909	-0.6721606	0.26012541	-2.5839867	0.00976656	0.03418413
ENSG000002	440.512758	0.21571872	0.12176114	1.77165489	0.07645186	0.16486426
ENSG000002	96.9961303	0.50264936	0.15873877	3.16651918	0.00154275	0.00765406
ENSG000002	2126.29817	0.13557721	0.08211698	1.65102532	0.0987334	0.19951888
ENSG000002	200.265883	1.47084611	0.35040577	4.19755104	2.70E-05	0.00028392
ENSG000002	614.199782	-0.2623156	0.1295677	-2.0245447	0.04291414	0.10750365
ENSG000002	153.542459	0.76792408	0.16940908	4.53295714	5.82E-06	8.02E-05
ENSG000002	188.404771	0.62905066	0.21767779	2.88982475	0.00385457	0.01595916
ENSG000002	136.116113	0.29555351	0.11852936	2.4935047	0.01264889	0.04197079
ENSG000002	234.306405	-0.2500648	0.14821042	-1.6872279	0.09155954	0.18870723
ENSG000002	43.4523624	1.80641698	0.62044647	2.9114792	0.00359722	0.01509207
ENSG000002	6192.38235	0.21005801	0.09521314	2.20618714	0.0273709	0.07641592
ENSG000002	237.849151	0.45341495	0.19000271	2.38636049	0.01701606	0.0531166
ENSG000002	968.818621	0.374183	0.0852131	4.39114412	1.13E-05	0.00013715
ENSG000002	93.1012971	0.24678409	0.13236762	1.86438422	0.06226777	0.1419827
ENSG000002	127.550634	0.1831374	0.10706389	1.71054307	0.0871655	0.18187621
ENSG000002	105.156582	0.35730325	0.09871691	3.6194736	0.0002952	0.00198635
ENSG000002	97.4580529	1.47360037	0.36873012	3.99641981	6.43E-05	0.00057437
ENSG000002	113.778432	1.24618473	0.37554619	3.31832615	0.00090559	0.00498641
ENSG000002	682.250409	0.32087991	0.10271993	3.12383305	0.00178512	0.00863184
ENSG000002	2436.61575	-0.2185126	0.09924801	-2.2016823	0.02768776	0.07712221
ENSG000002	70.8609274	0.57326774	0.16590265	3.45544654	0.00054938	0.00329561
ENSG000002	282.124839	1.42364381	0.34335398	4.14628606	3.38E-05	0.00033993
ENSG000002	46.6285009	0.46873677	0.19686946	2.38095216	0.01726795	0.05363421
ENSG000002	548.070044	0.37813428	0.10919192	3.46302428	0.00053414	0.00322606
ENSG000002	60.9540207	1.36444138	0.20641702	6.6101205	3.84E-11	3.37E-09
ENSG000002	420.824534	0.27258099	0.13536424	2.01368544	0.04404257	0.10934785
ENSG000002	728.207434	0.20111864	0.11328901	1.77527049	0.07585324	0.16393495
ENSG000002	324.106329	0.43976051	0.09997343	4.39877395	1.09E-05	0.00013343
ENSG000002	481.612131	-0.6020031	0.35800709	-1.6815397	0.09265813	0.19043256
ENSG000002	44.3508752	1.19565591	0.26660366	4.48476934	7.30E-06	9.61E-05
ENSG000002	50.5963643	0.72638483	0.25890061	2.80565127	0.0050215	0.01991417
ENSG000002	83.4643523	0.27482091	0.11405473	2.40955301	0.01597208	0.05044418
ENSG000002	1291.57742	-0.2655664	0.08934808	-2.9722679	0.00295609	0.0128719
ENSG000002	1891.66875	-0.1406004	0.06266082	-2.2438321	0.02484321	0.07117039

ENSG000002	749.334571	-0.110859	0.05428727	-2.0420819	0.04114341	0.10413078
ENSG000002	273.889637	0.22139366	0.09612113	2.30327765	0.02126323	0.06326563
ENSG000002	709.121935	0.10166626	0.05785866	1.7571486	0.07889248	0.16843341
ENSG000002	66.0842111	-0.2488076	0.13909925	-1.7887053	0.07366229	0.16058811
ENSG000002	3169.63653	-0.1432457	0.04852486	-2.9520062	0.00315717	0.01362057
ENSG000002	214.819786	0.31997679	0.10474601	3.05478743	0.0022522	0.0103252
ENSG000002	1348.07189	0.71085386	0.19915125	3.56941696	0.00035778	0.00232844
ENSG000002	53.9967333	1.53228502	0.35960935	4.26097106	2.04E-05	0.00022398
ENSG000002	2205.85154	0.83658958	0.24055547	3.47774081	0.00050566	0.00309222
ENSG000002	920.831711	0.20883286	0.07656116	2.72766049	0.00637852	0.02408279
ENSG000002	229.365713	0.25172295	0.11734281	2.14519283	0.03193743	0.08609557
ENSG000002	57.8847736	1.51035291	0.38047942	3.9696048	7.20E-05	0.00063286
ENSG000002	1816.88602	0.69754103	0.21665192	3.21963923	0.00128352	0.00660311
ENSG000002	49.3511347	0.70400093	0.13770666	5.11232315	3.18E-07	6.90E-06
ENSG000002	9536.02757	0.36929346	0.09245321	3.99438242	6.49E-05	0.0005779
ENSG000002	6.15496628	-0.6069831	0.36504728	-1.6627521	0.09636208	0.19601199
ENSG000002	95.2052072	0.44877762	0.13957517	3.21531125	0.00130303	0.00668923
ENSG000002	32.9340379	0.56785508	0.26727516	2.12460853	0.03361929	0.08958479
ENSG000002	50.1562144	-0.3255705	0.18332498	-1.77592	0.0757461	0.16376055
ENSG000002	85.2720549	-0.2574038	0.15510937	-1.6594987	0.09701535	0.19712225
ENSG000002	125.75347	0.86934344	0.16009554	5.43015414	5.63E-08	1.66E-06
ENSG000002	97.2881139	0.70327527	0.09974814	7.05050976	1.78E-12	2.21E-10
ENSG000002	10502.4651	-0.3431238	0.13309131	-2.5781084	0.00993428	0.03467902
ENSG000002	95.8989681	0.26444961	0.11532749	2.2930319	0.02184617	0.06444419
ENSG000002	410.087423	0.41466264	0.09535743	4.34850875	1.37E-05	0.00016131
ENSG000002	44518.5665	0.49420308	0.11216854	4.4058974	1.05E-05	0.00013
ENSG000002	203.560648	-0.2403357	0.11563594	-2.0783828	0.03767411	0.09779338
ENSG000002	590.710895	0.34765986	0.09102809	3.81925897	0.00013385	0.00104931
ENSG000002	38.7607562	0.49411645	0.20652781	2.39249357	0.01673432	0.05238334
ENSG000002	574.276539	-0.6521125	0.21714313	-3.0031459	0.00267204	0.01185965
ENSG000002	756.586722	0.31280205	0.11123979	2.81196185	0.00492403	0.01958662
ENSG000002	53.5570797	0.55244512	0.21317782	2.59147562	0.00955653	0.03361959
ENSG000002	709.440729	0.53239952	0.20529393	2.5933524	0.00950453	0.03348545
ENSG000002	52.6108554	-0.3957316	0.16888055	-2.3432635	0.01911588	0.05828915
ENSG000002	123.757348	0.20594137	0.08918213	2.30922231	0.02093125	0.06247033
ENSG000002	61.1295214	0.23368481	0.11366566	2.05589633	0.03979251	0.10192258
ENSG000002	1068.53839	0.52273976	0.19482283	2.68315453	0.00729313	0.02687193
ENSG000002	331.797453	0.57319353	0.12726972	4.50376993	6.68E-06	8.96E-05
ENSG000002	76.9931342	-1.3152783	0.28371245	-4.6359555	3.55E-06	5.30E-05
ENSG000002	79.865859	0.7548057	0.16983727	4.44428785	8.82E-06	0.00011236
ENSG000002	364.943297	0.51576477	0.12532528	4.11540874	3.86E-05	0.00037707
ENSG000002	270.62287	0.55806886	0.30371981	1.83744635	0.06614403	0.14833961
ENSG000002	480.080449	0.33148712	0.14911659	2.22300641	0.02621537	0.0739595

ENSG000002	44.1971944	0.49478842	0.2363725	2.09325711	0.03632621	0.09506292
ENSG000002	1258.60828	0.22603728	0.11021592	2.05085865	0.04028071	0.10278889
ENSG000002	71.8822954	0.31024776	0.18191834	1.70542323	0.08811552	0.18330407
ENSG000002	89.6817033	-0.3930237	0.17830327	-2.2042431	0.02750725	0.0767079
ENSG000002	131.074266	0.18632802	0.06895435	2.70219379	0.00688836	0.02569302
ENSG000002	110.171661	0.36218177	0.11331501	3.19623835	0.00139232	0.00705757
ENSG000002	53.2555161	0.63375131	0.18029111	3.51515568	0.0004395	0.00275269
ENSG000002	1970.03594	-0.1878742	0.04175587	-4.4993481	6.82E-06	9.11E-05
ENSG000002	54.7806323	2.36462887	0.3766492	6.27806691	3.43E-10	2.25E-08
ENSG000002	142.77402	0.1583192	0.08563804	1.84870174	0.06450089	0.14585089
ENSG000002	391.997705	-0.1983473	0.11044551	-1.795884	0.07251297	0.15885793
ENSG000002	3467.53147	0.27661624	0.12530351	2.20756981	0.02727428	0.07624421
ENSG000002	283.659994	0.70449479	0.17965568	3.92136108	8.81E-05	0.00074248
ENSG000002	77.9658763	0.39656372	0.13712711	2.89194257	0.00382868	0.01587012
ENSG000002	7897.68427	0.53411958	0.16770193	3.18493395	0.00144787	0.0072983
ENSG000002	186.966545	0.43557529	0.14736699	2.95571821	0.00311942	0.01348181
ENSG000002	41.4147436	0.84134327	0.2606843	3.22744123	0.00124903	0.00646931
ENSG000002	43.488376	1.00654679	0.21804274	4.61628201	3.91E-06	5.75E-05
ENSG000002	243.641424	-0.2442812	0.11040985	-2.2124946	0.02693251	0.0755853
ENSG000002	56.1422179	0.38055502	0.13972652	2.72357036	0.00645805	0.0243323
ENSG000002	6749.804	-0.7670487	0.19581885	-3.917134	8.96E-05	0.00075386
ENSG000002	174.495208	0.23204757	0.10069894	2.30436956	0.02120191	0.06313505
ENSG000002	177.026344	-0.2448699	0.08612493	-2.8431944	0.00446638	0.01799846
ENSG000002	498.40263	0.37501324	0.14929167	2.51195019	0.0120066	0.04028246
ENSG000002	34.6634192	1.00070073	0.3171262	3.15552844	0.00160208	0.00790238
ENSG000002	15.3969226	-1.8305602	0.28046312	-6.5269195	6.71E-11	5.65E-09
ENSG000002	72.7293515	0.736854	0.20285902	3.63234528	0.00028086	0.00191036
ENSG000002	173.739901	0.86983474	0.16726426	5.20036211	1.99E-07	4.69E-06
ENSG000002	899.850762	-0.1869642	0.09938161	-1.881276	0.05993438	0.1379081
ENSG000002	102.396259	1.31940379	0.39498337	3.34040343	0.00083657	0.00466308
ENSG000002	84.7963569	0.71453081	0.18584083	3.84485375	0.00012062	0.00096546
ENSG000002	30.8177686	1.2155353	0.30848643	3.94032019	8.14E-05	0.00069713
ENSG000002	208.286095	0.75845528	0.23517642	3.22504818	0.00125951	0.00650966
ENSG000002	366.811669	0.27680361	0.15815182	1.75023975	0.08007695	0.17047389
ENSG000002	102.926692	2.13982084	0.29122847	7.34756741	2.02E-13	3.21E-11
ENSG000002	214.258164	0.62654422	0.22195782	2.82280758	0.00476051	0.01901973
ENSG000002	68.2836941	-0.3953333	0.2279178	-1.7345432	0.08282174	0.17518585
ENSG000002	6773.5358	1.13199047	0.38615744	2.93142214	0.00337414	0.01435148
ENSG000002	1271.93553	0.2484459	0.10789497	2.30266435	0.02129774	0.06333429
ENSG000002	104.308004	-0.3539958	0.17012686	-2.0807754	0.03745447	0.0973628
ENSG000002	31.5917311	1.0309897	0.25556935	4.03408976	5.48E-05	0.00050387
ENSG000002	124.231312	0.32348397	0.14484583	2.23329847	0.02552927	0.07256082
ENSG000002	337.234544	0.37921572	0.10148474	3.73667732	0.00018647	0.00135883

ENSG000002	413.250175	0.46614168	0.12765405	3.65160116	0.00026061	0.00179442
ENSG000002	113.652558	-0.2203528	0.0962967	-2.2882691	0.02212185	0.06500746
ENSG000002	164.714962	-0.3298286	0.12411825	-2.657374	0.0078752	0.02867323
ENSG000002	1054.57829	-0.65311	0.17396518	-3.754257	0.00017386	0.00129096
ENSG000002	292.937796	-0.1990373	0.08714963	-2.2838574	0.02237991	0.06553826
ENSG000002	351.869664	0.99752965	0.20680268	4.8235818	1.41E-06	2.42E-05
ENSG000002	44.9910053	1.4597357	0.23612235	6.18211566	6.32E-10	3.74E-08
ENSG000002	92.7605649	-0.4278958	0.19786931	-2.1625173	0.03057832	0.08322152
ENSG000002	67.022647	1.08956821	0.23645632	4.60790485	4.07E-06	5.94E-05
ENSG000002	110.268258	-0.9298079	0.29816463	-3.118438	0.00181812	0.00874474
ENSG000002	260.473523	0.43484759	0.07951334	5.46886305	4.53E-08	1.41E-06
ENSG000002	53.8287174	0.5106078	0.23258096	2.19539815	0.02813505	0.078038
ENSG000002	215.105087	0.6403428	0.1746819	3.66576491	0.0002466	0.00171151
ENSG000002	131.005867	0.21830886	0.12970532	1.68311406	0.09235301	0.19004518
ENSG000002	41.760027	1.36362513	0.28853217	4.72607655	2.29E-06	3.70E-05
ENSG000002	248.227813	0.85771048	0.24803959	3.45795799	0.00054429	0.00327946
ENSG000002	600.141315	0.68386371	0.24434061	2.7988131	0.00512908	0.02020893
ENSG000002	62.0207549	1.32762918	0.21836791	6.07978157	1.20E-09	6.41E-08
ENSG000002	44.991053	1.30255906	0.27664183	4.70846755	2.50E-06	3.97E-05
ENSG000002	1422.65395	0.48957925	0.08944383	5.47359469	4.41E-08	1.38E-06
ENSG000002	23158.5879	0.55274098	0.12826576	4.30934168	1.64E-05	0.00018693
ENSG000002	210.238311	-0.2738368	0.0819764	-3.340434	0.00083648	0.00466308
ENSG000002	367.132076	0.59605676	0.16351829	3.64519942	0.00026718	0.00183533
ENSG000002	165.351034	-0.1981624	0.09048767	-2.1899384	0.02852871	0.0789378
ENSG000002	179.451904	0.25407493	0.1075224	2.36299538	0.0181279	0.0558051
ENSG000002	569.453341	0.64226848	0.16785484	3.82633279	0.00013007	0.00102462
ENSG000002	50.9779084	1.30767755	0.25543513	5.11941146	3.06E-07	6.68E-06
ENSG000002	4.30510094	1.24960364	0.52666524	2.37267159	0.01765996	0.05463161
ENSG000002	85.9520378	1.29880531	0.33196228	3.91250868	9.13E-05	0.0007649
ENSG000002	50.7889847	2.50101297	0.48506274	5.1560608	2.52E-07	5.73E-06
ENSG000002	93.9979671	0.3830466	0.15555398	2.46246745	0.01379847	0.04507293
ENSG000002	2643.83574	0.84506483	0.18516603	4.56382211	5.02E-06	7.11E-05
ENSG000002	365.882266	0.31436127	0.17502873	1.79605528	0.07248573	0.15884143
ENSG000002	162.444068	0.68325684	0.24591004	2.77848289	0.00546134	0.02125125
ENSG000002	15.3319322	3.03628193	0.6637963	4.57411697	4.78E-06	6.83E-05
ENSG000002	33.3371593	-0.3880921	0.21287658	-1.8230848	0.06829055	0.15159748
ENSG000002	9589.93839	0.15425878	0.08959255	1.72178135	0.08510913	0.17858865
ENSG000002	60.8114407	0.33632967	0.12199883	2.75682688	0.00583652	0.022434
ENSG000002	223.705669	1.77197604	0.41414804	4.27860545	1.88E-05	0.00020945
ENSG000002	42.3576153	0.86541982	0.26620508	3.25095162	0.00115019	0.00606143
ENSG000002	21.3560159	1.72778083	0.38090982	4.53593139	5.73E-06	7.94E-05
ENSG000002	232.720551	0.40917488	0.12235733	3.3440977	0.00082551	0.00461742
ENSG000002	5104.97551	-0.2370213	0.04802236	-4.9356448	7.99E-07	1.49E-05

ENSG000002	68.191446	0.24999369	0.09574898	2.61092799	0.00902969	0.03216453
ENSG000002	20.798828	1.50143832	0.62799973	2.39082639	0.0168105	0.05255367
ENSG000002	38.3756916	2.63822954	0.45818268	5.75802981	8.51E-09	3.49E-07
ENSG000002	62.9275989	-0.2925416	0.16938477	-1.7270832	0.08415273	0.17712053
ENSG000002	45.4332974	1.54576849	0.25824251	5.98572433	2.15E-09	1.07E-07
ENSG000002	56.3164197	1.06840537	0.23200924	4.60501213	4.12E-06	6.01E-05
ENSG000002	14.6490733	1.67503446	0.46813645	3.57809023	0.00034611	0.0022627
ENSG000002	94.8451797	0.94904666	0.13190093	7.19514746	6.24E-13	9.04E-11
ENSG000002	146.485986	0.40182553	0.13957486	2.87892473	0.00399034	0.01640874
ENSG000002	25683.4932	0.9537913	0.17262605	5.52518742	3.29E-08	1.09E-06
ENSG000002	116.302954	0.65165666	0.18671474	3.49011895	0.00048281	0.00298289
ENSG000002	151.536548	1.33678524	0.18666499	7.16141399	7.98E-13	1.11E-10
ENSG000002	44.1297556	0.96462479	0.21945834	4.39548021	1.11E-05	0.0001348
ENSG000002	1123.67636	0.24391342	0.08615489	2.83110371	0.00463877	0.01860516
ENSG000002	218.525022	-0.2741344	0.16494587	-1.661966	0.09651959	0.19627109
ENSG000002	700.433212	0.42571394	0.12482113	3.41059206	0.00064822	0.00377442
ENSG000002	29.3827965	0.69172561	0.33848345	2.04360246	0.04099284	0.10385855
ENSG000002	192.649773	0.66418585	0.31831191	2.08658816	0.03692538	0.09629846
ENSG000002	62.2235895	-0.333187	0.15865422	-2.1000827	0.03572156	0.09390279
ENSG000002	80.0893975	0.81756071	0.21220382	3.85271446	0.00011682	0.00093953
ENSG000002	552.02804	0.76293405	0.37273012	2.04688062	0.04066981	0.10327493
ENSG000002	3206.59426	0.28497606	0.14580564	1.95449267	0.05064298	0.12104683
ENSG000002	57.0613028	-0.4789097	0.15062705	-3.1794404	0.0014756	0.00739688
ENSG000002	2272.8367	0.21348411	0.10213234	2.09026947	0.0365936	0.09557224
ENSG000002	585.918155	0.46969534	0.12811963	3.66606861	0.00024631	0.00171058
ENSG000002	239.992925	0.17583196	0.07291581	2.41143825	0.01588974	0.0502693
ENSG000002	1363.79803	0.52963001	0.14743785	3.59222539	0.00032787	0.00216985
ENSG000002	495.22226	0.4170218	0.13699226	3.04412678	0.00233357	0.01061759
ENSG000002	76.4932608	0.68140533	0.1305389	5.219941	1.79E-07	4.31E-06
ENSG000002	178.881983	1.07248207	0.17267354	6.21103902	5.26E-10	3.25E-08
ENSG000002	8484.35861	0.23095739	0.11788423	1.95918816	0.05009076	0.1201034
ENSG000002	228.146596	2.00472581	0.26565444	7.54636667	4.48E-14	8.42E-12
ENSG000002	252.843777	0.19942691	0.10518259	1.89600676	0.05795915	0.13432318
ENSG000002	3866.43547	0.21306825	0.09379849	2.27155297	0.02311352	0.06718411
ENSG000002	79.5351039	0.56279893	0.14681715	3.83333241	0.00012642	0.00100147
ENSG000002	122.103558	-0.390396	0.11018208	-3.5431898	0.00039532	0.00252918
ENSG000002	540.387023	1.75388087	0.28320124	6.19305514	5.90E-10	3.52E-08
ENSG000002	48.5699999	1.79030536	0.27127427	6.59961348	4.12E-11	3.60E-09
ENSG000002	3526.93179	0.30959054	0.16385907	1.88937085	0.05884215	0.13604314
ENSG000002	71.291766	0.74085837	0.24572238	3.01502202	0.00256961	0.01148148
ENSG000002	41.9932497	0.68189239	0.27359091	2.49237956	0.01268904	0.04208472
ENSG000002	56.4894727	1.81946258	0.23636956	7.69753329	1.39E-14	3.00E-12
ENSG000002	104.860681	-0.1585639	0.09152451	-1.7324752	0.08318898	0.17577829

ENSG000002	61.6756284	0.36445041	0.14860858	2.45241832	0.01418996	0.04614364
ENSG000002	276.364251	0.84000163	0.18025885	4.65997431	3.16E-06	4.81E-05
ENSG000002	316.62537	1.30910423	0.32861533	3.98369797	6.79E-05	0.0006012
ENSG000002	71.1414792	-0.5416619	0.21019	-2.5770107	0.00996589	0.03476422
ENSG000002	210.026993	0.37982094	0.09144615	4.15349292	3.27E-05	0.00033172
ENSG000002	85.6859873	0.449701	0.16061301	2.79990395	0.00511178	0.02016182
ENSG000002	74.3715023	0.35326357	0.16301903	2.16700815	0.03023423	0.08256425
ENSG000002	574.609165	1.13807966	0.21952547	5.18427155	2.17E-07	5.05E-06
ENSG000002	131.81725	-0.1867058	0.10356204	-1.8028403	0.0714133	0.1570854
ENSG000002	301.652806	0.12026883	0.06441241	1.86716872	0.06187803	0.14141458
ENSG000002	9.60521831	-0.7714523	0.25352754	-3.0428738	0.00234331	0.01065186
ENSG000002	252.854934	-0.3145853	0.13217341	-2.3800951	0.01730817	0.05373842
ENSG000002	1257.64633	0.44823066	0.16260046	2.75663835	0.00583989	0.02244098
ENSG000002	38.9621004	0.86277154	0.24708581	3.49178911	0.0004798	0.00296917
ENSG000002	77.8866856	0.32397286	0.13130241	2.46737933	0.01361061	0.04456982
ENSG000002	1104.67549	0.48953746	0.19580267	2.50015723	0.01241382	0.04136132
ENSG000002	131.227278	0.55881645	0.17731504	3.15154562	0.00162409	0.00799734
ENSG000002	127.661669	0.35013873	0.12130116	2.88652409	0.00389523	0.01609532
ENSG000002	217.680082	-0.9439695	0.21418997	-4.4071603	1.05E-05	0.00012935
ENSG000002	97.0170747	0.5168324	0.23872112	2.16500495	0.03038731	0.08284172
ENSG000002	21.8077562	0.95852908	0.3220928	2.97594072	0.00292091	0.01275281
ENSG000002	192.405708	5.14329865	0.75391191	6.822148	8.97E-12	9.28E-10
ENSG000002	1170.39188	0.46225645	0.08252115	5.6016727	2.12E-08	7.50E-07
ENSG000002	64.0863242	-0.4755845	0.25836643	-1.8407365	0.06566019	0.14747583
ENSG000002	40.3061955	0.46212898	0.15382241	3.00430195	0.00266191	0.01182192
ENSG000002	378.241217	0.57020492	0.22053366	2.58556873	0.00972185	0.0340688
ENSG000002	144.360644	-0.1920223	0.11626107	-1.6516476	0.09860641	0.19934566
ENSG000002	939.909094	1.74793304	0.35715229	4.89408318	9.88E-07	1.78E-05
ENSG000002	727.043943	-0.2103578	0.07394463	-2.8448019	0.00444391	0.01792783
ENSG000002	1028.70066	-0.1405413	0.06568925	-2.1394871	0.03239624	0.08694334
ENSG000002	98.2294652	0.1494678	0.08117119	1.84138976	0.06556446	0.14732936
ENSG000002	127.671178	0.13761985	0.07367671	1.86788817	0.06177766	0.1412146
ENSG000002	183.793464	0.32865644	0.1629245	2.01723149	0.04367136	0.10864967
ENSG000002	68.4198006	-0.2439448	0.11880978	-2.0532387	0.04004943	0.10235502
ENSG000002	121.960044	-0.4032826	0.13246208	-3.0445136	0.00233057	0.01060727
ENSG000002	59.4991292	0.30224496	0.13711432	2.20432822	0.02750127	0.07670599
ENSG000002	943.744835	0.37060581	0.08567797	4.32556695	1.52E-05	0.00017646
ENSG000002	499.106597	0.78448733	0.18840568	4.16382005	3.13E-05	0.00031907
ENSG000002	5639.93523	0.75158358	0.28412223	2.64528254	0.00816227	0.0295765
ENSG000002	196.602641	0.57849352	0.1531644	3.77694503	0.00015876	0.00119792
ENSG000002	57.9518448	-0.6163597	0.29180766	-2.1122122	0.03466825	0.09178343
ENSG000002	143.065471	-0.2684408	0.11984133	-2.2399688	0.02509295	0.07171465
ENSG000002	64.710367	0.43355524	0.25920128	1.67265856	0.09439453	0.1929036

ENSG000002	6038.42615	0.21653766	0.11032982	1.96263945	0.04968808	0.11941461
ENSG000002	70.7298901	0.58500389	0.13473643	4.3418389	1.41E-05	0.00016548
ENSG000002	77.7459124	0.97316196	0.2583019	3.76753696	0.00016487	0.00123624
ENSG000002	167.133132	1.3854812	0.3184156	4.35117246	1.35E-05	0.00015988
ENSG000002	255.980836	1.15296865	0.23294086	4.94961964	7.44E-07	1.41E-05
ENSG000002	61.8076229	0.24313715	0.1473786	1.64974522	0.09899506	0.19979679
ENSG000002	103.286858	0.8813561	0.19825022	4.44567536	8.76E-06	0.00011183
ENSG000002	366.613454	0.86229518	0.17720713	4.86602971	1.14E-06	2.00E-05
ENSG000002	544.884895	0.51792178	0.20161851	2.56882062	0.01020453	0.03544305
ENSG000002	3825.72769	0.43385094	0.13946761	3.1107648	0.00186604	0.00892481
ENSG000002	218.903062	0.41131643	0.1148803	3.58039122	0.00034308	0.00224693
ENSG000002	239.455333	-0.28571	0.13042983	-2.1905264	0.02848608	0.07887574
ENSG000002	164.840016	0.51830947	0.10398297	4.98456097	6.21E-07	1.21E-05
ENSG000002	343.523154	1.38309786	0.37442288	3.6939459	0.0002208	0.00156341
ENSG000002	86.7918345	0.23093719	0.11019015	2.09580612	0.03609939	0.09465555
ENSG000002	55.9666286	1.09996757	0.30504764	3.60588787	0.00031109	0.00207206
ENSG000002	106.098072	0.4048808	0.18487242	2.19005513	0.02852024	0.0789378
ENSG000002	58.193962	0.83667996	0.21602579	3.8730558	0.00010748	0.00087573
ENSG000002	716.178209	0.55803178	0.32851063	1.69867191	0.08938102	0.18532458
ENSG000002	81.5422693	1.2183205	0.38822496	3.13818178	0.00169999	0.00828383
ENSG000002	453.320694	0.25987352	0.10022735	2.59284044	0.00951869	0.03351089
ENSG000002	70.3694285	0.44222649	0.1267603	3.48868276	0.00048541	0.00299359
ENSG000002	142.94438	0.44403782	0.21411788	2.07380078	0.03809782	0.09866884
ENSG000002	42.4052918	0.70657013	0.33335674	2.11956156	0.03404304	0.09042565
ENSG000002	48.9862766	0.46104987	0.22191586	2.0775886	0.03774727	0.0979131
ENSG000002	88.0383078	1.16680198	0.22692916	5.1417015	2.72E-07	6.08E-06
ENSG000002	28.2081124	1.88452224	0.33444522	5.63477111	1.75E-08	6.33E-07
ENSG000002	547.93125	0.18370527	0.07835256	2.34459807	0.0190476	0.05812989
ENSG000002	73.3692955	0.68298926	0.14780946	4.62074114	3.82E-06	5.64E-05
ENSG000002	99.3623309	0.54102771	0.16711429	3.23747122	0.00120594	0.0063002
ENSG000002	295.846055	-0.4063908	0.13084366	-3.1059266	0.00189684	0.00903879
ENSG000002	598.752631	0.27875634	0.16154444	1.72557066	0.08442468	0.17751239
ENSG000002	148.421692	0.47400456	0.2104059	2.25281017	0.02427112	0.06989223
ENSG000002	181.291317	-0.3099014	0.12541567	-2.4709941	0.01347381	0.0442218
ENSG000002	70.0794998	0.76340744	0.18807584	4.05904055	4.93E-05	0.00046293
ENSG000002	55.7645272	1.06004031	0.17419341	6.08542138	1.16E-09	6.26E-08
ENSG000002	31.5865758	1.14251011	0.25591735	4.46437147	8.03E-06	0.00010387
ENSG000002	59.608155	-0.4655062	0.11653068	-3.994709	6.48E-05	0.00057746
ENSG000002	2040.70635	-0.2491332	0.14166685	-1.758585	0.07864802	0.16819809
ENSG000002	217.137093	0.38179807	0.12901605	2.9593067	0.00308332	0.0133457
ENSG000002	200.523196	0.32980233	0.12634708	2.61028844	0.00904659	0.03219306
ENSG000002	2818.37206	-0.2501768	0.04803395	-5.208332	1.91E-07	4.53E-06
ENSG000002	153.518738	-0.2805536	0.12845385	-2.1840811	0.02895629	0.07981159

ENSG000002	488.168819	-0.1621191	0.09237792	-1.7549551	0.07926699	0.16909747
ENSG000002	210.805068	0.18416107	0.10398715	1.7709984	0.07656097	0.1650013
ENSG000002	91.8850247	0.55711572	0.20243685	2.752047	0.0059224	0.02266786
ENSG000002	83.9727979	0.73961018	0.14494312	5.10276146	3.35E-07	7.19E-06
ENSG000002	277.157749	-0.2527785	0.12466922	-2.0275936	0.04260174	0.10685101
ENSG000002	935.024093	0.83836954	0.19398805	4.32175878	1.55E-05	0.00017897
ENSG000002	21.7722442	0.72631738	0.26857335	2.70435387	0.00684374	0.02555298
ENSG000002	19.3730822	1.37017974	0.51921972	2.63892085	0.00831704	0.03000223
ENSG000002	81.1303358	0.23952242	0.13291162	1.80211796	0.07152685	0.15716813
ENSG000002	176.181064	0.72869593	0.15228213	4.7851703	1.71E-06	2.85E-05
ENSG000002	520.937986	-0.3989637	0.13033299	-3.0611102	0.00220518	0.01016108
ENSG000002	89.5740805	0.25576645	0.14015191	1.82492304	0.06801264	0.15118907
ENSG000002	267.63882	0.66224303	0.11641634	5.68857444	1.28E-08	4.86E-07
ENSG000002	184.068005	0.25404765	0.09514106	2.67022092	0.00758014	0.0277587
ENSG000002	43.1699364	-0.4338503	0.24005804	-1.8072726	0.07071979	0.15593876
ENSG000002	195.209656	1.06106037	0.21174691	5.01098398	5.42E-07	1.09E-05
ENSG000002	524.327147	-0.5990503	0.17546892	-3.4139969	0.00064017	0.0037353
ENSG000002	236.120368	0.94169763	0.22824422	4.12583348	3.69E-05	0.00036403
ENSG000002	80.1231733	-0.4589386	0.18558726	-2.4728993	0.01340219	0.04403664
ENSG000002	516.011198	0.68975857	0.23891287	2.88707159	0.00388846	0.01607651
ENSG000002	781.785579	0.33316639	0.15987659	2.08389725	0.03716951	0.09676096
ENSG000002	576.190223	0.32317287	0.11061265	2.92166275	0.00348168	0.014701
ENSG000002	77.3291784	1.0112797	0.17244599	5.86432717	4.51E-09	2.03E-07
ENSG000002	487.531307	-0.1796611	0.08377283	-2.1446221	0.03198307	0.08617561
ENSG000002	76.5727729	0.44407731	0.1431561	3.10204951	0.00192186	0.00912851
ENSG000002	466.176928	0.29246776	0.1427224	2.0492072	0.04044186	0.10294873
ENSG000002	107.81065	0.2576633	0.14598538	1.76499387	0.0775648	0.16639733
ENSG000002	88.951956	-0.2920724	0.08278055	-3.5282734	0.00041828	0.00264151
ENSG000002	2302.95622	0.24857783	0.15013611	1.65568312	0.09778601	0.19823437
ENSG000002	381.132473	1.18791157	0.22941814	5.17793228	2.24E-07	5.18E-06
ENSG000002	896.062647	-0.21137	0.11186543	-1.8895028	0.05882449	0.136024
ENSG000002	375.167742	-0.5590104	0.09461159	-5.9084771	3.45E-09	1.62E-07
ENSG000002	32.3403663	1.95699385	0.27993637	6.99085231	2.73E-12	3.17E-10
ENSG000002	123.979453	-0.1458465	0.08775745	-1.6619276	0.09652729	0.19627109
ENSG000002	80.8486235	-0.4394639	0.20908135	-2.1018797	0.03556382	0.09355602
ENSG000002	107.2652	0.55139152	0.31905779	1.72818697	0.08395471	0.17685029
ENSG000002	99.1235542	1.33389766	0.23725411	5.62223193	1.89E-08	6.73E-07
ENSG000002	76.9918313	0.44720161	0.12693012	3.52321108	0.00042635	0.00268429
ENSG000002	164.774081	0.97543369	0.19119288	5.10183073	3.36E-07	7.22E-06
ENSG000002	18.2120094	1.05426263	0.26791121	3.93511951	8.32E-05	0.00070905
ENSG000002	97.9525165	-0.1566708	0.06968814	-2.2481702	0.02456534	0.07062473
ENSG000002	310.260659	0.82246649	0.1162247	7.07652073	1.48E-12	1.91E-10
ENSG000002	100.307896	0.19826492	0.1077868	1.83941739	0.06585382	0.14781907

ENSG000002	331.171772	0.1845736	0.11113478	1.66080859	0.0967519	0.19663927
ENSG000002	983.363394	0.51341412	0.20133045	2.55010657	0.010769	0.03703074
ENSG000002	115.506257	-0.3661965	0.10242952	-3.5751074	0.00035008	0.00228351
ENSG000002	181.707003	-0.1909841	0.11568885	-1.6508426	0.09877071	0.19955419
ENSG000002	702.352592	0.28451155	0.09169628	3.10275994	0.00191725	0.00911559
ENSG000002	1064.80975	0.54698161	0.17551418	3.11645259	0.00183041	0.00879494
ENSG000002	78.9090844	-0.3491164	0.1889289	-1.8478718	0.06462088	0.14597891
ENSG000002	269.418894	-0.3080488	0.11431782	-2.6946701	0.00704583	0.02617257
ENSG000002	1121.50726	0.61474741	0.09704682	6.33454477	2.38E-10	1.71E-08
ENSG000002	109.568152	-0.5052277	0.15470461	-3.2657572	0.00109172	0.00581247
ENSG000002	11513.8393	0.83508573	0.18848338	4.4305536	9.40E-06	0.00011892
ENSG000002	311.308136	0.59714489	0.16654823	3.58541722	0.00033654	0.0022131
ENSG000002	221.784249	0.1738144	0.10387006	1.67338311	0.0942519	0.19277528
ENSG000002	47.0940198	0.38946125	0.19986734	1.9485988	0.05134336	0.12227703
ENSG000002	98.2902118	-0.7093455	0.19381361	-3.6599364	0.00025228	0.00174342
ENSG000002	135.566092	0.47562866	0.15612899	3.04638269	0.00231613	0.01055149
ENSG000002	144.918882	-0.5875031	0.1532956	-3.8324847	0.00012686	0.00100369
ENSG000002	22.3491346	-0.4627787	0.25053733	-1.8471447	0.06472617	0.14614217
ENSG000002	538.82441	-0.329073	0.09785211	-3.3629631	0.00077111	0.00435857
ENSG000002	362.282895	1.95586402	0.19880774	9.83796714	7.73E-23	6.22E-20
ENSG000002	2029.61644	-0.3367672	0.08129718	-4.1424214	3.44E-05	0.00034478
ENSG000002	48.7619465	-0.4487077	0.23367556	-1.9202167	0.05483053	0.12865726
ENSG000002	1223.38605	-0.2195149	0.12293371	-1.7856364	0.07415815	0.16121386
ENSG000002	126.963172	0.97875178	0.18902377	5.17792972	2.24E-07	5.18E-06
ENSG000002	482.066179	0.21842286	0.1182986	1.84636896	0.06483865	0.14628836
ENSG000002	78.601376	-0.6445447	0.249782	-2.5804289	0.00986777	0.0344793
ENSG000002	237.842597	-0.6840257	0.24443214	-2.7984277	0.00513521	0.02022118
ENSG000002	126.902779	1.326823	0.17337074	7.65309629	1.96E-14	4.12E-12
ENSG000002	5488.35784	-0.1375838	0.06399681	-2.1498546	0.03156671	0.08525484
ENSG000002	118.244037	0.43611967	0.09419444	4.62999379	3.66E-06	5.42E-05
ENSG000002	271.201911	-0.2253037	0.09013337	-2.4996699	0.01243091	0.04137068
ENSG000002	304.21214	0.12406976	0.0635859	1.95121485	0.05103149	0.12167433
ENSG000002	62.3468837	1.06900886	0.220062	4.85776221	1.19E-06	2.07E-05
ENSG000002	79.5511851	0.34734334	0.18645613	1.86286899	0.0624807	0.14229805
ENSG000002	5581.81627	0.34345877	0.13242528	2.59360424	0.00949757	0.03346907
ENSG000002	1197.78809	0.19066747	0.10966489	1.73863733	0.08209858	0.17390877
ENSG000002	855.602201	0.23836974	0.08191097	2.9101075	0.00361304	0.01514531
ENSG000002	105.403052	0.78878996	0.30632672	2.57499563	0.01002414	0.03491696
ENSG000002	70.4321271	0.68827411	0.20516201	3.35478335	0.00079427	0.0044738
ENSG000002	991.585176	0.75834231	0.17575796	4.31469682	1.60E-05	0.00018361
ENSG000002	160.652169	0.163385	0.08512844	1.91927626	0.05494938	0.12887351
ENSG000002	1817.25181	0.26200816	0.07609218	3.4432995	0.00057466	0.00342176
ENSG000002	37.4849205	0.80754597	0.19625505	4.11477812	3.88E-05	0.00037782

ENSG000002	191.424701	0.42767825	0.15262644	2.80212423	0.00507673	0.02006182
ENSG000002	4571.32984	0.88693088	0.18887642	4.69582645	2.66E-06	4.14E-05
ENSG000002	334.224589	0.25458356	0.12480302	2.03988298	0.04136198	0.1045014
ENSG000002	169.449042	0.39546012	0.20133327	1.96420651	0.04950615	0.11911569
ENSG000002	1477.47523	0.3435361	0.06110389	5.62216406	1.89E-08	6.73E-07
ENSG000002	105.087126	0.4192362	0.1339208	3.13047869	0.00174522	0.0084615
ENSG000002	82.5857798	0.66640667	0.30984366	2.1507836	0.03149328	0.08511095
ENSG000002	489.62814	-0.3475493	0.16507335	-2.1054236	0.03525445	0.09296164
ENSG000002	135.364585	0.37616593	0.14727841	2.5541145	0.01064582	0.03666811
ENSG000002	96.9271666	0.27493744	0.11929988	2.30459101	0.02118949	0.06311105
ENSG000002	1167.31423	0.23974831	0.09938507	2.41231721	0.01585148	0.05016174
ENSG000002	10839.3853	0.2294093	0.09630387	2.38213998	0.01721235	0.05353668
ENSG000002	110.211013	0.53274434	0.1356857	3.92631151	8.63E-05	0.0007295
ENSG000002	47.9896523	-0.4684549	0.19086307	-2.4544031	0.01411187	0.04592064
ENSG000002	55.7471905	2.06364769	0.24894277	8.28964694	1.14E-16	3.51E-14
ENSG000002	64.1619079	0.57892482	0.14892122	3.8874568	0.0001013	0.00083146
ENSG000002	95.5945208	0.62482666	0.28355381	2.2035559	0.02755559	0.07681313
ENSG000002	145.363626	0.94994247	0.19666078	4.83036051	1.36E-06	2.35E-05
ENSG000002	4866.48139	0.32119958	0.07851688	4.09083471	4.30E-05	0.00041292
ENSG000002	48.6764661	0.29125083	0.17348642	1.6788106	0.09318896	0.19135769
ENSG000002	252.511024	0.30052402	0.12464109	2.41111521	0.01590382	0.05028343
ENSG000002	67.408474	0.44386801	0.22922567	1.93638005	0.05282117	0.12499515
ENSG000002	141.104258	1.64065188	0.24236778	6.76926558	1.29E-11	1.28E-09
ENSG000002	244.067039	0.38568336	0.08293132	4.65063566	3.31E-06	4.99E-05
ENSG000002	111.281054	0.48173999	0.10856843	4.43720146	9.11E-06	0.00011571
ENSG000002	290.134104	1.10235532	0.23813511	4.62911706	3.67E-06	5.43E-05
ENSG000002	81.1487481	0.92695304	0.25165874	3.68337319	0.00023017	0.00161631
ENSG000002	14.1410351	-1.6590894	0.39987255	-4.1490454	3.34E-05	0.00033682
ENSG000002	27.1785459	-1.2628755	0.43731652	-2.8877838	0.00387966	0.01604932
ENSG000002	79.770955	-0.3777345	0.12005537	-3.1463359	0.0016533	0.00810811
ENSG000002	50.4238241	1.01738527	0.1328076	7.6605952	1.85E-14	3.94E-12
ENSG000002	51.1147081	-0.3707848	0.19228375	-1.928321	0.05381521	0.12676416
ENSG000002	99.1536337	0.29126908	0.09362603	3.11098388	0.00186465	0.00892307
ENSG000002	113.058177	0.93594049	0.21080881	4.43975987	9.01E-06	0.00011445
ENSG000002	230.778495	0.58641338	0.23226704	2.52473784	0.01157846	0.03920009
ENSG000002	59.4113006	1.03794278	0.16444027	6.31197452	2.75E-10	1.92E-08
ENSG000002	32.3575768	0.99857172	0.23798409	4.19596005	2.72E-05	0.00028483
ENSG000002	1136.9747	0.23262983	0.09099479	2.55651828	0.01057255	0.03645039
ENSG000002	98.0633186	0.90786938	0.19851286	4.573353	4.80E-06	6.85E-05
ENSG000002	206.200135	-0.1364082	0.0759434	-1.7961826	0.07246548	0.15882109
ENSG000002	35.7371646	0.5810169	0.2294572	2.53213627	0.01133699	0.03849953
ENSG000002	121.966333	0.32668904	0.12981728	2.51652971	0.01185169	0.03991989
ENSG000002	1690.16938	0.33201796	0.11120908	2.98552918	0.00283088	0.01241301

ENSG000002	203.842421	0.33957838	0.1024828	3.31351581	0.00092131	0.00504886
ENSG000002	154.698588	-0.2918437	0.14581201	-2.0015069	0.04533779	0.11175915
ENSG000002	48.2851793	0.72083228	0.23282765	3.09599094	0.00196156	0.00927151
ENSG000002	193.837768	0.63983949	0.30789079	2.07813779	0.03769667	0.09783441
ENSG000002	111.173322	0.2351689	0.1078021	2.18148714	0.0291474	0.08027749
ENSG000002	886.93953	-0.2025272	0.07005037	-2.8911652	0.00383816	0.01589579
ENSG000002	89.1159893	1.00558962	0.31007033	3.24310169	0.00118236	0.00619488
ENSG000002	82.5401698	0.55129996	0.16510886	3.33900883	0.00084078	0.00468476
ENSG000002	611.541806	-0.2358677	0.13003206	-1.8139194	0.06969013	0.15411401
ENSG000002	110.106502	0.37554677	0.1094928	3.42987647	0.00060386	0.00356192
ENSG000002	116.469374	0.29537974	0.14659108	2.01499123	0.04390557	0.10908248
ENSG000002	84.7997849	0.59385161	0.1386986	4.28159761	1.86E-05	0.0002071
ENSG000002	47.1490162	0.63679478	0.3553675	1.79193307	0.07314369	0.15977572
ENSG000002	597.574572	-1.0391941	0.27745915	-3.7453951	0.00018011	0.00132584
ENSG000002	23.9998347	1.69901501	0.35292881	4.81404446	1.48E-06	2.52E-05
ENSG000002	57.0970453	-0.5035163	0.161443	-3.1188489	0.00181559	0.00874126
ENSG000002	582.863895	0.28445215	0.05599613	5.07985382	3.78E-07	7.94E-06
ENSG000002	735.857339	0.25507457	0.14516638	1.75711873	0.07889757	0.16843341
ENSG000002	487.310343	-0.1599159	0.07929837	-2.0166355	0.04373356	0.10876705
ENSG000002	70.6591248	1.22709938	0.18123993	6.7705797	1.28E-11	1.27E-09
ENSG000002	76.6915504	0.78447288	0.2100897	3.73399016	0.00018847	0.00137084
ENSG000002	68.6816659	0.49939977	0.14652409	3.40831177	0.00065366	0.00379847
ENSG000002	1460.09106	0.56700008	0.10241235	5.53644256	3.09E-08	1.04E-06
ENSG000002	89.103179	0.42174812	0.12520373	3.36849474	0.0007558	0.00428878
ENSG000002	32.7461956	0.82073922	0.20802682	3.94535293	7.97E-05	0.00068549
ENSG000002	77.7987287	0.18754213	0.09588454	1.95591633	0.05047501	0.1206953
ENSG000002	604.0516	0.20961974	0.08501877	2.46557011	0.01367954	0.04474498
ENSG000002	722.918117	-0.6899823	0.2134106	-3.2331209	0.00122446	0.00637166
ENSG000002	107.987184	0.24914613	0.08190898	3.04174378	0.00235212	0.01068522
ENSG000002	72.917587	2.56500024	0.34003606	7.5433183	4.58E-14	8.51E-12
ENSG000002	161.601535	-0.32133	0.17480712	-1.8381973	0.06603334	0.14815316
ENSG000002	356.167163	-0.3340836	0.11348423	-2.9438773	0.00324129	0.01388013
ENSG000002	143.244792	0.27494555	0.12605802	2.18110321	0.02917578	0.08031216
ENSG000002	418.454148	0.29212697	0.14720212	1.98452961	0.04719683	0.11503036
ENSG000002	114.210726	-0.2150229	0.09736894	-2.2083316	0.02722116	0.07614462
ENSG000002	34.4374136	0.51660429	0.22003868	2.34778857	0.01888524	0.05778045
ENSG000002	69.4186533	0.22829716	0.13528885	1.68747942	0.0915112	0.18863444
ENSG000002	179.54095	0.76675374	0.20788003	3.68844346	0.00022563	0.00159114
ENSG000002	151.070052	0.1196851	0.06110704	1.95861396	0.05015801	0.12020499
ENSG000002	65.1123688	1.34674477	0.2680545	5.02414535	5.06E-07	1.02E-05
ENSG000002	720.818798	0.27326229	0.07904203	3.45717725	0.00054587	0.00328365
ENSG000002	136.780956	-0.2501029	0.09046218	-2.7647235	0.00569711	0.02200906
ENSG000002	206.939298	0.96947584	0.22260761	4.35508854	1.33E-05	0.00015717

ENSG000002	663.507726	-0.3522164	0.18496286	-1.9042546	0.05687703	0.13238713
ENSG000002	103.565076	0.51914299	0.16858328	3.07944537	0.00207386	0.00967734
ENSG000002	212.927297	0.26530013	0.13109951	2.02365457	0.04300571	0.10757853
ENSG000002	73.613479	0.41794008	0.15376319	2.71807622	0.00656627	0.02470152
ENSG000002	157.587665	0.19782911	0.10898387	1.81521462	0.06949093	0.15369697
ENSG000002	2248.4909	-0.4215817	0.09312114	-4.527239	5.98E-06	8.19E-05
ENSG000002	160.613695	0.1951514	0.09842702	1.9827015	0.04740078	0.11542434
ENSG000002	101.646502	0.39542623	0.13748964	2.87604391	0.00402694	0.0165311
ENSG000002	779.944292	1.70380495	0.30505879	5.58516912	2.33E-08	8.11E-07
ENSG000002	172.993348	0.46989198	0.13017363	3.60973239	0.00030651	0.00204913
ENSG000002	354.920705	-0.2755069	0.15251205	-1.8064595	0.07084659	0.15614705
ENSG000002	85.4425706	0.28367968	0.14580269	1.94564091	0.05169789	0.12289913
ENSG000002	67.8727324	0.53932299	0.22717863	2.374004	0.01759636	0.05446976
ENSG000002	28.9653459	1.4837223	0.29922654	4.958525	7.10E-07	1.35E-05
ENSG000002	92.2355839	0.28358189	0.12071752	2.34913625	0.01881702	0.05765697
ENSG000002	211.50776	0.35519842	0.14130545	2.51369222	0.01194747	0.04014914
ENSG000002	2185.99721	-0.2727383	0.07160603	-3.8088736	0.0001396	0.00108266
ENSG000002	1338.78647	0.71739039	0.15751904	4.55430902	5.26E-06	7.39E-05
ENSG000002	480.400266	0.53366391	0.09737587	5.48045347	4.24E-08	1.35E-06
ENSG000002	83.8578092	-0.498802	0.18851517	-2.6459513	0.00814615	0.02953237
ENSG000002	67.7812545	0.24733002	0.14003687	1.76617783	0.07736602	0.16616776
ENSG000002	37.2384986	0.62035035	0.20121285	3.08305533	0.00204887	0.00957792
ENSG000002	18.4186182	1.65615987	0.39506338	4.19213718	2.76E-05	0.00028844
ENSG000002	63.3658097	0.47910034	0.19158075	2.50077497	0.01239219	0.04130825
ENSG000002	170.097975	-0.130764	0.06250424	-2.0920827	0.03643112	0.09523233
ENSG000002	479.332038	0.34231856	0.12298463	2.78342546	0.00537882	0.0210035
ENSG000002	127.919981	-0.3185536	0.15179938	-2.0985173	0.03585947	0.09415585
ENSG000002	183.683645	-0.3051744	0.07250484	-4.2090213	2.56E-05	0.00027181
ENSG000002	124.455654	-0.2539971	0.10404574	-2.4412057	0.01463831	0.04723365
ENSG000002	229.365442	0.32179848	0.16020019	2.00872722	0.04456607	0.11028846
ENSG000002	520.159885	0.43246478	0.09772873	4.42515526	9.64E-06	0.0001213
ENSG000002	354.760592	0.58717939	0.18464998	3.17995903	0.00147296	0.00738621
ENSG000002	204.216797	-0.220694	0.07970253	-2.768971	0.00562336	0.02177062
ENSG000002	155.177485	-0.3320805	0.08998819	-3.6902675	0.00022402	0.00158079
ENSG000002	102.400884	0.8407678	0.25770293	3.26254656	0.00110416	0.00585933
ENSG000002	100.025173	0.37536082	0.1115634	3.36455156	0.00076668	0.00433888
ENSG000002	277.309192	-0.2656075	0.11621536	-2.285477	0.02228487	0.06535241
ENSG000002	1290.24194	-0.3340638	0.06775996	-4.9301068	8.22E-07	1.52E-05
ENSG000002	298.633635	0.86312248	0.14711564	5.86696623	4.44E-09	2.01E-07
ENSG000002	33.4589804	0.99246183	0.32136454	3.08827424	0.00201323	0.00944788
ENSG000002	24.5298416	23.0075536	1.42972165	16.0923307	2.89E-58	4.18E-54
ENSG000002	37.7767474	1.47068865	0.3733727	3.93892926	8.18E-05	0.00070067
ENSG000002	182.244154	-0.4464269	0.15197462	-2.9375094	0.0033086	0.01410586

ENSG000002	55.3321056	0.58888457	0.16117749	3.65364032	0.00025855	0.00178277
ENSG000002	191.863736	0.59704916	0.11095609	5.38094965	7.41E-08	2.09E-06
ENSG000002	146.039744	-0.6191202	0.16121712	-3.8402882	0.00012289	0.00098035
ENSG000002	56.1091045	0.42718375	0.14171059	3.01448004	0.0025742	0.01149228
ENSG000002	120.558176	0.22859691	0.07768144	2.94274804	0.00325313	0.01391852
ENSG000002	196.449815	1.87932271	0.2293639	8.19362901	2.53E-16	7.06E-14
ENSG000002	989.034589	1.20025686	0.1953995	6.14257903	8.12E-10	4.67E-08
ENSG000002	76.8832422	0.21788148	0.12906099	1.68820555	0.09137178	0.18849635
ENSG000002	24.2415407	1.76684005	0.39221467	4.50477809	6.64E-06	8.93E-05
ENSG000002	6.30526752	0.67985185	0.39031695	1.74179433	0.08154444	0.17303856
ENSG000002	241.10807	0.34542797	0.11819017	2.92264551	0.00347071	0.01467608
ENSG000002	81.0162482	0.22267461	0.10947557	2.03401183	0.0419504	0.10569313
ENSG000002	52.6789421	-0.6225118	0.21579615	-2.8847215	0.0039176	0.0161555
ENSG000002	72.1796769	0.50604565	0.13452878	3.76161636	0.00016882	0.00126196
ENSG000002	2205.33043	0.09457638	0.05187352	1.82321119	0.06827141	0.15157823
ENSG000002	2037.56132	0.18115351	0.08846877	2.04765489	0.04059383	0.10318297
ENSG000002	63.6990438	0.32684229	0.12790336	2.55538471	0.01060705	0.03656063
ENSG000002	157.850106	0.65803233	0.17513066	3.75737949	0.0001717	0.00127758
ENSG000002	220.996455	1.01363377	0.17257313	5.87364762	4.26E-09	1.94E-07
ENSG000002	63.0833691	0.32189477	0.16004335	2.01129739	0.04429405	0.10970028
ENSG000002	146.764267	-0.3609854	0.19666896	-1.8354976	0.06643199	0.14875298
ENSG000002	323.115308	0.38954012	0.09144405	4.25987409	2.05E-05	0.00022448
ENSG000002	74.1140315	0.5898719	0.19544033	3.01816871	0.00254307	0.01139545
ENSG000002	79.7266109	1.12743045	0.16718574	6.74358025	1.55E-11	1.50E-09
ENSG000002	91.0613759	0.21344215	0.09183931	2.32408274	0.02012106	0.06068155
ENSG000002	42.7723433	0.58052466	0.20995049	2.76505497	0.00569132	0.02199257
ENSG000002	66.5020372	1.23242	0.20133839	6.1211376	9.29E-10	5.20E-08
ENSG000002	139.590637	0.21889667	0.10425769	2.0995733	0.03576639	0.09400358
ENSG000002	462.850179	0.29730893	0.09360589	3.17617751	0.0014923	0.00744709
ENSG000002	74.7501238	0.2405375	0.12492577	1.92544348	0.0541739	0.12742609
ENSG000002	140.019486	0.48785059	0.10730001	4.54660352	5.45E-06	7.62E-05
ENSG000002	367.145286	0.93460684	0.15360079	6.0846487	1.17E-09	6.26E-08
ENSG000002	128.432585	0.48767586	0.17370752	2.80745394	0.00499348	0.0198302
ENSG000002	143.593721	1.45063837	0.20520018	7.06938162	1.56E-12	2.00E-10
ENSG000002	135.438968	0.41915521	0.11152613	3.75835857	0.00017103	0.00127569
ENSG000002	59.5272282	0.32789505	0.12817939	2.5580949	0.01052474	0.03632877
ENSG000002	322.345974	-0.1735131	0.1049278	-1.6536426	0.09820016	0.1988018
ENSG000002	250.857802	0.14047869	0.0848541	1.65553226	0.09781658	0.19824689
ENSG000002	436.257124	-0.3240114	0.0844293	-3.8376654	0.00012421	0.00098869
ENSG000002	298.298473	0.30208044	0.15995333	1.88855358	0.05895167	0.13619908
ENSG000002	118.960246	0.42617464	0.12130377	3.51328427	0.0004426	0.00276618
ENSG000002	79.0451819	0.34633641	0.16418465	2.1094324	0.03490728	0.09226449
ENSG000002	22.235247	0.50820376	0.30747628	1.65282267	0.09836696	0.19902823

ENSG000002	470.37488	0.39694363	0.13228598	3.0006478	0.00269406	0.01194273
ENSG000002	209.22841	0.43469323	0.19788662	2.19667825	0.02804343	0.0778435
ENSG000002	85.7346529	0.83709258	0.22602631	3.70351835	0.00021263	0.00151644
ENSG000002	184.197238	-0.3008054	0.05945601	-5.0592929	4.21E-07	8.75E-06
ENSG000002	119.620529	0.61788222	0.26877358	2.29889488	0.02151091	0.06379295
ENSG000002	71.1880268	0.7058126	0.13760031	5.12944054	2.91E-07	6.38E-06
ENSG000002	842.391266	0.14004182	0.07451663	1.87933657	0.06019855	0.13832567
ENSG000002	43.6864986	0.70104087	0.26898848	2.60621151	0.00915499	0.03252289
ENSG000002	757.179146	0.371498	0.10084921	3.68369752	0.00022988	0.00161503
ENSG000002	601.712386	-0.4396008	0.13129327	-3.3482354	0.00081328	0.00455959
ENSG000002	308.694938	1.16463931	0.39910119	2.9181554	0.00352109	0.01484143
ENSG000002	49.6282644	0.34337529	0.18800131	1.82645156	0.06778227	0.15087687
ENSG000002	115.347615	0.6980261	0.22570574	3.09263772	0.00198386	0.00935724
ENSG000002	941.014122	0.20752373	0.12557751	1.65255483	0.0984215	0.19911077
ENSG000002	176.937037	1.61453746	0.26506392	6.09112502	1.12E-09	6.08E-08
ENSG000002	68.8763861	0.37965641	0.16734487	2.26870658	0.02328618	0.06759103
ENSG000002	12.8441439	-0.9172986	0.36693143	-2.4999183	0.0124222	0.04136251
ENSG000002	1326.27705	-0.2126447	0.0840188	-2.530918	0.01137644	0.03860636
ENSG000002	30271.6178	0.7106263	0.19375176	3.6677153	0.00024473	0.00170205
ENSG000002	121.626996	-0.2128165	0.08808592	-2.4160102	0.01569162	0.04978636
ENSG000002	56.8188892	1.02593564	0.40656778	2.52340612	0.01162241	0.03930295
ENSG000002	44.643334	-0.6051976	0.33166179	-1.8247432	0.06803979	0.15122621
ENSG000002	43.8905793	0.78710997	0.32146226	2.44852996	0.01434405	0.04650901
ENSG000002	374.044921	-0.2284462	0.1289329	-1.7718221	0.07642409	0.16482891
ENSG000002	17.3095926	-1.3120619	0.44524744	-2.9468151	0.00321065	0.01378154
ENSG000002	219.78899	0.74947356	0.23138535	3.23907096	0.0011992	0.00627176
ENSG000002	53.1412665	0.36840896	0.20071501	1.83548292	0.06643417	0.14875298
ENSG000002	305.816031	0.24918721	0.10725722	2.32326747	0.02016479	0.06078387
ENSG000002	64.942984	1.28490353	0.21760533	5.90474292	3.53E-09	1.66E-07
ENSG000002	157.822722	0.31587563	0.16408699	1.92504985	0.05422312	0.12752116
ENSG000002	1671.89481	0.27851679	0.10553251	2.63915625	0.00831127	0.02998887
ENSG000002	891.344474	0.64220859	0.09814827	6.54324891	6.02E-11	5.13E-09
ENSG000002	369.505877	-0.0916537	0.05296417	-1.7304846	0.08354373	0.1762741
ENSG000002	109.600011	0.2064425	0.12182346	1.69460383	0.0901506	0.18654646
ENSG000002	94.1194618	-0.4734283	0.23340947	-2.0283164	0.04252797	0.1067214
ENSG000002	88.149251	0.63345528	0.14737087	4.29837524	1.72E-05	0.00019443
ENSG000002	824.027166	-0.2747609	0.12097394	-2.2712402	0.02313244	0.06722561
ENSG000002	130.650324	0.33152696	0.19332347	1.71488214	0.08636684	0.18052177
ENSG000002	351.968208	0.29873731	0.14301755	2.08881577	0.03672431	0.09584311
ENSG000002	456.611317	0.45329938	0.1227348	3.69332391	0.00022134	0.00156598
ENSG000002	101.794534	-0.2116725	0.10348223	-2.045496	0.04080598	0.10354813
ENSG000002	545.976437	0.37941281	0.17713529	2.14193804	0.03219847	0.08655766
ENSG000002	519.766333	0.45918666	0.20433444	2.24723082	0.02462528	0.07075485

ENSG000002	1170.08746	-0.6698373	0.16234992	-4.1258863	3.69E-05	0.00036403
ENSG000002	170.318943	-0.1502953	0.08492178	-1.769809	0.07675897	0.16532965
ENSG000002	90.1035463	0.22026606	0.12601732	1.74790307	0.08048081	0.17113509
ENSG000002	57.0248505	-0.2271166	0.12697937	-1.7886104	0.07367759	0.16058811
ENSG000002	67.504387	1.74196314	0.54710435	3.18396873	0.00145271	0.0073125
ENSG000002	173.13001	0.15231892	0.09134996	1.66742184	0.09543056	0.19446002
ENSG000002	229.216343	0.47524159	0.1698073	2.79871127	0.0051307	0.02020893
ENSG000002	222.717309	0.31375229	0.09114217	3.44244924	0.00057647	0.00342971
ENSG000002	7.80797877	0.692488	0.38288367	1.80861198	0.07051131	0.15557376
ENSG000002	21.289604	1.23171581	0.5374196	2.29190712	0.021911	0.06456971
ENSG000002	185.93587	0.36945947	0.1777985	2.07796736	0.03771237	0.09785761
ENSG000002	682.341972	0.82520571	0.17205349	4.79621605	1.62E-06	2.73E-05
ENSG000002	65.4084091	0.80067015	0.22254795	3.59774211	0.00032099	0.00213214
ENSG000002	74.7226087	1.47324659	0.26106265	5.64326836	1.67E-08	6.06E-07
ENSG000002	127.836603	1.69859588	0.27747025	6.12172247	9.26E-10	5.20E-08
ENSG000002	73.8595646	-0.5846956	0.20978077	-2.7871743	0.00531699	0.02082379
ENSG000002	69.2146119	0.27909269	0.13139679	2.12404502	0.03366638	0.0896553
ENSG000002	59.872665	1.37066607	0.2211711	6.19731102	5.74E-10	3.45E-08
ENSG000002	93.8531543	0.30818322	0.11693794	2.6354426	0.00840276	0.03027377
ENSG000002	323.792111	-0.4180138	0.09760516	-4.2827022	1.85E-05	0.00020623
ENSG000002	190.330817	1.73266946	0.25488579	6.79782676	1.06E-11	1.09E-09
ENSG000002	68.3641558	0.32649743	0.16493074	1.97960327	0.04774812	0.11592544
ENSG000002	80.3115493	0.56771384	0.22556544	2.51684762	0.011841	0.03989316
ENSG000002	151.711076	0.31950305	0.15556535	2.05381891	0.03999322	0.10223783
ENSG000002	22871.4148	-0.3002253	0.15126958	-1.9847036	0.04717746	0.11502185
ENSG000002	73.1202807	0.29073454	0.16638278	1.74738362	0.08057082	0.17129871
ENSG000002	157.694458	0.21421566	0.12819639	1.67099609	0.09472245	0.19338277
ENSG000002	463.948416	0.32374289	0.13747008	2.35500613	0.0185224	0.05691071
ENSG000002	817.594668	0.76570273	0.19317666	3.96374358	7.38E-05	0.00064469
ENSG000002	734.559782	1.50305681	0.36029143	4.17178069	3.02E-05	0.00031008
ENSG000002	87.5141878	0.32634397	0.12293837	2.65453317	0.00794182	0.02888606
ENSG000002	2053.6505	0.34711165	0.15691174	2.21214583	0.02695659	0.07559429
ENSG000002	10.5639262	1.09160687	0.47108682	2.31720952	0.02049232	0.06146909
ENSG000002	201.254823	0.51238537	0.21671337	2.36434593	0.01806194	0.0556612
ENSG000002	108.361601	0.82059057	0.1715422	4.78360768	1.72E-06	2.87E-05
ENSG000002	584.424879	0.51449629	0.14135429	3.6397642	0.00027289	0.00186661
ENSG000002	98.4617135	0.41788257	0.11882851	3.51668593	0.00043697	0.00273924
ENSG000002	709.874676	0.22131283	0.09034471	2.44964911	0.01429955	0.04642545
ENSG000002	71.5032139	0.63032817	0.15968585	3.94730125	7.90E-05	0.00068155
ENSG000002	580.698361	-0.2043539	0.08416018	-2.4281541	0.01517589	0.04856487
ENSG000002	98.1861004	0.40081929	0.12907905	3.10522337	0.00190135	0.00904892
ENSG000002	108.662431	0.27887565	0.12230034	2.28025235	0.02259273	0.06600078
ENSG000002	74.8203358	0.89777872	0.17419788	5.15378679	2.55E-07	5.78E-06

ENSG000002	1309.97815	0.6862166	0.16533156	4.15054817	3.32E-05	0.00033555
ENSG000002	28.0442771	0.58300944	0.29487352	1.97715089	0.04802458	0.11652878
ENSG000002	412.297788	-0.3875219	0.12143435	-3.1912049	0.00141681	0.00717417
ENSG000002	87.5717373	0.45351542	0.12529638	3.6195414	0.00029513	0.00198635
ENSG000002	455.665284	0.37257034	0.12306807	3.0273518	0.00246707	0.01110295
ENSG000002	291.007161	-0.2725769	0.16474892	-1.654499	0.09802616	0.19853278
ENSG000002	78.1934628	1.12439397	0.20581546	5.46311728	4.68E-08	1.45E-06
ENSG000002	135.783009	2.64592086	0.31928163	8.28710651	1.16E-16	3.51E-14
ENSG000002	456.973017	1.6724526	0.22256138	7.51456788	5.71E-14	1.03E-11
ENSG000002	262.107358	0.30242033	0.15905732	1.90132916	0.05725891	0.13306702
ENSG000002	4.13635845	3.81640431	0.75480584	5.05614039	4.28E-07	8.87E-06
ENSG000002	27.7382909	1.47256211	0.29435966	5.00259485	5.66E-07	1.12E-05
ENSG000002	406.106999	0.51087399	0.11005352	4.64205037	3.45E-06	5.18E-05
ENSG000002	84.1672133	-0.3631618	0.13208532	-2.7494483	0.00596957	0.02280621
ENSG000002	276.025558	-0.3402485	0.099208	-3.4296477	0.00060437	0.00356347
ENSG000002	193.103766	0.78310103	0.21050075	3.72018163	0.00019908	0.00143426
ENSG000002	433.452905	0.35772253	0.09626426	3.716047	0.00020236	0.00145347
ENSG000002	243.899911	0.24259832	0.1400467	1.73226732	0.08322598	0.17578287
ENSG000002	83.0481185	-0.5281048	0.28746275	-1.8371242	0.06619156	0.14833961
ENSG000002	97.6386377	0.59451053	0.13226442	4.49486369	6.96E-06	9.26E-05
ENSG000002	257.654126	0.19310276	0.10344858	1.86665459	0.06194984	0.14148941
ENSG000002	80.9433762	0.37781565	0.12724084	2.96929542	0.00298483	0.01297758
ENSG000002	201.39455	0.6157403	0.13970207	4.40752458	1.05E-05	0.00012935
ENSG000002	212.619944	0.49745899	0.13070583	3.80594326	0.00014126	0.00109293
ENSG000002	299.896429	0.31625258	0.13878458	2.27872995	0.02268312	0.0662093
ENSG000002	93.8026246	-0.212633	0.12412755	-1.7130199	0.08670887	0.18105382
ENSG000002	479.109842	1.23638142	0.19916287	6.20789122	5.37E-10	3.28E-08
ENSG000002	634.733757	0.32208641	0.12905706	2.49568998	0.01257124	0.04176097
ENSG000002	90.9955063	0.30633272	0.12669542	2.41786729	0.01561177	0.04960153
ENSG000002	548.488794	1.07007758	0.16833791	6.3567238	2.06E-10	1.52E-08
ENSG000002	44.2616896	0.25539525	0.15322974	1.66674726	0.09556467	0.1946633
ENSG000002	159.738933	0.55381641	0.16970542	3.26339852	0.00110085	0.00584602
ENSG000002	104.659499	0.92566772	0.13721382	6.74616951	1.52E-11	1.49E-09
ENSG000002	80.4249335	0.52982663	0.11592645	4.57036877	4.87E-06	6.91E-05
ENSG000002	88.4130464	0.78689454	0.19549049	4.02523187	5.69E-05	0.00051992
ENSG000002	127.816756	0.42584456	0.17766354	2.39691593	0.01653372	0.05185624
ENSG000002	717.182491	0.24586807	0.13837191	1.77686402	0.07559061	0.16350791
ENSG000002	142.811066	-0.3165969	0.11619553	-2.7246909	0.00643617	0.02426882
ENSG000002	58.7307685	0.6958438	0.14813735	4.69728785	2.64E-06	4.13E-05
ENSG000002	490.353842	0.48616622	0.11133259	4.3667915	1.26E-05	0.00015057
ENSG000002	441.071041	0.22465286	0.12156289	1.84803815	0.06459682	0.14597008
ENSG000002	104.745016	0.96226058	0.14411885	6.67685436	2.44E-11	2.24E-09
ENSG000002	101.550702	0.1686698	0.08308322	2.03013082	0.04234324	0.1063684

ENSG000002	34.2255335	1.5133423	0.23726772	6.37820554	1.79E-10	1.36E-08
ENSG000002	198.931151	0.50591272	0.12055379	4.19657245	2.71E-05	0.00028483
ENSG000002	170.739633	0.73861364	0.16429827	4.49556561	6.94E-06	9.25E-05
ENSG000002	168.785156	0.68495797	0.14760419	4.64050483	3.48E-06	5.21E-05
ENSG000002	104.880051	0.22068471	0.12071753	1.8281082	0.06753331	0.15042361
ENSG000002	182.20613	-0.5602406	0.27323883	-2.0503697	0.04032837	0.10279306
ENSG000002	443.519847	0.35678768	0.11484809	3.10660524	0.00189249	0.00902452
ENSG000002	205.195747	0.61522881	0.13338531	4.61241801	3.98E-06	5.84E-05
ENSG000002	523.526529	-0.3909263	0.0879473	-4.4450067	8.79E-06	0.00011208
ENSG000002	890.321812	0.38988679	0.20703095	1.88322953	0.05966927	0.13736353
ENSG000002	344.250483	0.2053605	0.10438718	1.96729613	0.04914908	0.1183942
ENSG000002	69.2296802	-0.3554942	0.19819694	-1.7936413	0.07287044	0.15944329
ENSG000002	700.409096	-0.1403113	0.07084921	-1.9804218	0.04765615	0.1158112
ENSG000002	303.06465	0.51081055	0.1199945	4.25694982	2.07E-05	0.0002267
ENSG000002	2593.545	0.92090099	0.30101265	3.05934321	0.00221823	0.01020498
ENSG000002	167.286307	0.47834925	0.21693396	2.20504549	0.0274509	0.07660975
ENSG000002	253.492146	0.37960072	0.12708862	2.98689774	0.00281824	0.01236832
ENSG000002	145.279718	0.21483151	0.1185644	1.8119395	0.06999555	0.15471856
ENSG000002	98.7625061	0.88066571	0.18096957	4.86637442	1.14E-06	2.00E-05
ENSG000002	58.0166266	0.3217569	0.13278022	2.42322909	0.01538322	0.04901182
ENSG000002	25.5827925	4.27414757	0.76587585	5.58073164	2.40E-08	8.28E-07
ENSG000002	7.91824014	3.60387713	1.06039922	3.3986041	0.00067731	0.00390766
ENSG000002	188.947353	-0.2945615	0.10770922	-2.7347839	0.00624212	0.02367268
ENSG000002	1007.45336	0.3947795	0.20042127	1.96974856	0.04886719	0.11793087
ENSG000002	320.046386	-0.5996359	0.25902114	-2.3150075	0.02061252	0.06173529
ENSG000002	1389.28882	1.23790145	0.17514143	7.06801019	1.57E-12	2.00E-10
ENSG000002	12.7995003	-1.3817153	0.28426574	-4.8606466	1.17E-06	2.05E-05
ENSG000002	57.0996353	0.40944472	0.12715708	3.2199915	0.00128194	0.00659969
ENSG000002	214.964043	1.39085672	0.23630411	5.8858761	3.96E-09	1.83E-07
ENSG000002	36.2710666	0.45165857	0.2468901	1.82939119	0.06734103	0.1501338
ENSG000002	49.1724544	1.28447456	0.23314349	5.50937353	3.60E-08	1.18E-06
ENSG000002	56.3659079	1.3481365	0.25354965	5.31705127	1.05E-07	2.81E-06
ENSG000002	134.689112	1.10579589	0.24830167	4.45343719	8.45E-06	0.00010834
ENSG000002	39.6672422	0.46674047	0.27195649	1.71623216	0.08611956	0.18021292
ENSG000002	275.051641	0.93145528	0.18373556	5.06954282	3.99E-07	8.32E-06
ENSG000002	280.703481	2.20111295	0.40242885	5.46957045	4.51E-08	1.41E-06
ENSG000002	548.594338	0.265759	0.12664396	2.09847364	0.03586333	0.09415585
ENSG000002	19.2644137	1.65068123	0.47256207	3.49304637	0.00047754	0.00295648
ENSG000002	19.6591936	0.80679513	0.35615875	2.26526829	0.02349622	0.06807795
ENSG000002	462.312233	1.72295506	0.30136532	5.71716433	1.08E-08	4.30E-07
ENSG000002	80.4994272	0.58907345	0.1825948	3.22612387	0.00125479	0.00648988
ENSG000002	65.8428778	1.64849149	0.20181054	8.16851038	3.12E-16	8.38E-14
ENSG000002	48.6477988	1.14289678	0.29790743	3.83641586	0.00012484	0.00099156

ENSG000002	62.7890474	0.84711339	0.30402477	2.78633017	0.00533085	0.02086428
ENSG000002	399.923155	0.46122504	0.20668517	2.2315343	0.02564576	0.07284904
ENSG000002	339.369675	0.54828091	0.19769446	2.77337523	0.00554781	0.02152991
ENSG000002	75.1869209	0.77006261	0.27053413	2.84645273	0.00442093	0.01785006
ENSG000002	262.70407	0.52031194	0.20989247	2.47894528	0.01317715	0.04338577
ENSG000002	889.796991	0.2880534	0.15377463	1.873218	0.06103829	0.13993697
ENSG000002	187.816064	0.41769955	0.10244585	4.07727167	4.56E-05	0.00043316
ENSG000002	62.8417819	0.38079552	0.20420026	1.86481408	0.06220747	0.14188164
ENSG000002	108.458487	0.87758788	0.14467319	6.06600215	1.31E-09	6.88E-08
ENSG000002	336.199751	0.33680735	0.1327772	2.53663535	0.01119235	0.03814244
ENSG000002	56.4486941	-0.8456063	0.27649867	-3.0582652	0.00222622	0.01023301
ENSG000002	68.2084929	0.906179	0.23684765	3.82599949	0.00013024	0.00102545
ENSG000002	329.370369	0.44223193	0.08530416	5.18417785	2.17E-07	5.05E-06
ENSG000002	135.707304	0.39023599	0.14042013	2.7790601	0.00545164	0.02123064
ENSG000002	110.077535	0.22987869	0.12153646	1.8914381	0.05856589	0.13555584
ENSG000002	784.227138	0.61960677	0.16100856	3.84828461	0.00011895	0.00095415
ENSG000002	26.0003217	0.74609897	0.28104705	2.65471199	0.00793761	0.02887801
ENSG000002	47.2514878	0.51063126	0.21718268	2.35116018	0.01871498	0.05738071
ENSG000002	262.529494	-0.2362884	0.11837097	-1.9961684	0.0459156	0.11276136
ENSG000002	74.3025448	0.39072732	0.12840727	3.04287536	0.00234329	0.01065186
ENSG000002	33.5035314	0.81826197	0.19524263	4.19100051	2.78E-05	0.00028862
ENSG000002	12.9738159	-1.4040544	0.34684609	-4.0480618	5.16E-05	0.00048113
ENSG000002	500.301955	1.60158451	0.2875527	5.56970788	2.55E-08	8.72E-07
ENSG000002	52.5310287	0.77864838	0.23871116	3.26188513	0.00110674	0.00587087
ENSG000002	562.848791	0.54411926	0.17470966	3.11441998	0.00184307	0.00883809
ENSG000002	154.520155	1.43214192	0.16540079	8.65861583	4.78E-18	2.03E-15
ENSG000002	183.569531	-0.9751031	0.31588057	-3.086936	0.00202231	0.00947823
ENSG000002	69.9261365	0.74324761	0.26556524	2.79873826	0.00513027	0.02020893
ENSG000002	1489.91379	0.9257989	0.13334866	6.94269391	3.85E-12	4.39E-10
ENSG000002	31.8009398	1.90389886	0.30044022	6.33703048	2.34E-10	1.69E-08
ENSG000002	120.337149	1.11660618	0.21289071	5.2449737	1.56E-07	3.84E-06
ENSG000002	77.6372023	0.37891604	0.14029176	2.70091438	0.00691491	0.02578542
ENSG000002	49.4453495	0.46812447	0.23054514	2.03051112	0.04230461	0.10632667
ENSG000002	386.441363	1.01875588	0.15874134	6.41771015	1.38E-10	1.08E-08
ENSG000002	77.9708973	1.01306126	0.26781234	3.78272809	0.00015512	0.00117593
ENSG000002	307.421426	0.79507775	0.24950695	3.18659558	0.00143958	0.00727213
ENSG000002	79.2032876	0.49080378	0.14260976	3.44158611	0.00057831	0.00343785
ENSG000002	1012.28407	0.7894799	0.17483869	4.51547606	6.32E-06	8.55E-05
ENSG000002	39.7781923	0.55600117	0.28459396	1.95366468	0.05074089	0.12118707
ENSG000002	24.4255264	1.43327022	0.30433732	4.70947899	2.48E-06	3.96E-05
ENSG000002	891.563687	0.34600888	0.1309093	2.64311913	0.00821461	0.02971413
ENSG000002	19.6095752	1.04069484	0.24186995	4.30270409	1.69E-05	0.00019162
ENSG000002	70.9061906	0.23718318	0.1212184	1.95665995	0.05038746	0.12060541

ENSG000002	421.073582	0.75935935	0.14451462	5.25455022	1.48E-07	3.69E-06
ENSG000002	117.361955	0.37464156	0.13000895	2.88165979	0.00395587	0.01628086
ENSG000002	62.4811203	0.36604436	0.1665255	2.19812795	0.02793999	0.07767542
ENSG000002	148.501049	0.40067828	0.165139	2.42630919	0.01525327	0.04872637
ENSG000002	36.8625856	0.3991131	0.1952636	2.04397079	0.04095643	0.10380263
ENSG000002	46.7340739	0.70749208	0.31106505	2.27441846	0.02294084	0.06681181
ENSG000002	54.0879993	0.3683149	0.20115118	1.83103526	0.06709528	0.14976719
ENSG000002	80.5326907	0.3881875	0.1529216	2.53847388	0.01113371	0.03796048
ENSG000002	27.5995189	0.62576007	0.28307628	2.21057047	0.0270656	0.07581193
ENSG000002	55.5338902	-0.4933618	0.2353663	-2.0961447	0.03606936	0.09461105
ENSG000002	27.4646945	0.32903241	0.19787863	1.66279906	0.09635266	0.19601199
ENSG000002	181.1868	0.32801899	0.14706411	2.23044898	0.02571765	0.07301031
ENSG000002	219.261518	0.65369546	0.18948109	3.44992453	0.00056074	0.00334989
ENSG000002	68.2119699	0.49560919	0.22260669	2.22638944	0.02598811	0.07359064
ENSG000002	3.27859958	3.657712	1.45236732	2.51844829	0.01178732	0.03974003
ENSG000002	25.3522951	0.47133096	0.23936706	1.96907192	0.04894483	0.11803959
ENSG000002	78.7539233	0.89384266	0.15678528	5.70106247	1.19E-08	4.64E-07
ENSG000002	82.416109	0.67422254	0.2079324	3.24250839	0.00118482	0.00620555
ENSG000002	533.123989	0.72911544	0.35166472	2.0733255	0.038142	0.09872487
ENSG000002	70.4666997	3.34904156	0.37465911	8.93890325	3.93E-19	2.03E-16
ENSG000002	61.4894471	0.54438072	0.15223329	3.57596371	0.00034894	0.00227707
ENSG000002	57.3983036	0.54347954	0.17590493	3.08962092	0.00200412	0.00942347
ENSG000002	111.698709	0.96564857	0.17724237	5.44818141	5.09E-08	1.55E-06
ENSG000002	168.073051	-0.5917419	0.14163972	-4.1777964	2.94E-05	0.00030307
ENSG000002	173.700283	-0.6524679	0.31141824	-2.0951498	0.03615768	0.09477406
ENSG000002	156.640499	1.30679917	0.23362793	5.5935057	2.23E-08	7.82E-07
ENSG000002	77.4526156	0.56211689	0.14914535	3.76891996	0.00016396	0.00123069
ENSG000002	114.239999	0.68327526	0.24341547	2.80703299	0.00500001	0.01984525
ENSG000002	295.325951	1.02685279	0.19914598	5.15628171	2.52E-07	5.73E-06
ENSG000002	138.648373	1.26258287	0.23459497	5.38196912	7.37E-08	2.08E-06
ENSG000002	335.451538	0.90914331	0.21350398	4.2582031	2.06E-05	0.00022583
ENSG000002	119.107411	1.54016475	0.34618496	4.44896487	8.63E-06	0.00011033
ENSG000002	408.966543	0.81562621	0.14947563	5.45658334	4.85E-08	1.49E-06
ENSG000002	318.527933	0.53360261	0.13990395	3.81406391	0.0001367	0.00106415
ENSG000002	88.8538845	0.43062956	0.11675762	3.68823533	0.00022581	0.00159114
ENSG000002	95.6850173	0.65864376	0.18563203	3.54811489	0.000388	0.00248714
ENSG000002	72.7304411	0.91115362	0.17019354	5.35363238	8.62E-08	2.37E-06
ENSG000002	142.110142	-0.3130385	0.16023901	-1.9535721	0.05075185	0.12118707
ENSG000002	54.0799162	0.77879572	0.24524012	3.17564561	0.00149503	0.00745467
ENSG000002	42.6191257	-0.5391499	0.23041567	-2.3399014	0.01928883	0.05865603
ENSG000002	4338.75087	0.38783755	0.14730959	2.63280576	0.00846828	0.03047948
ENSG000002	189.372671	-0.1865508	0.09177947	-2.0325984	0.04209311	0.10592373

entrez	symbol	chromosome	start	end
8813	DPM1	20	50934867	50958555
57147	SCYL3	1	169849631	169894267
55732	C1orf112	1	169662007	169854080
4800	NFYA	6	41072945	41099976
81887	LAS1L	X	65512582	65534775
51056	LAP3	4	17577192	17607972
29916	SNX11	17	48103357	48123074
3382	ICA1	7	8113184	8262687
79007	DBNDD1	16	90004871	90020128
57679	ALS2	2	201700267	201780956
843	CASP10	2	201182898	201229406
8837	CFLAR	2	201116154	201176687
55471	NDUFAF7	2	37231631	37253403
10181	RBM5	3	50088919	50119021
952	CD38	4	15778275	15853232
2288	FKBP4	12	2794970	2805423
23028	KDM1A	1	23019443	23083689
5965	RECQL	12	21468910	21501669
4706	NDUFAB1	16	23581014	23596316
85413	SLC22A16	6	110424687	110476641
996	CDC27	17	47117703	47189422
1856	DVL2	17	7225342	7234517
292	SLC25A5	X	119468422	119471396
5439	POLR2J	7	102473118	102478907
79657	RPAP3	12	47661249	47706030
6296	ACSM3	16	20610243	20797581
81691	REXO5	16	20806429	20849668
10943	MSL3	X	11758159	11775772
4353	MPO	17	58269855	58280935
5244	ABCB4	7	87401696	87480435
23077	MYCBP2	13	77044657	77327094
5164	PDK2	17	50094737	50112152
51573	GDE1	16	19501693	19522123
9020	MAP3K14	17	45263119	45317029
4293	MAP3K9	14	70722526	70809534
55971	BAIAP2L1	7	98291650	98401090
80853	KDM7A	7	140084746	140176983
79017	GGCT	7	30496621	30504841
6915	TBXA2R	19	3594507	3606875
3475	IFRD1	7	112422968	112481017
54623	PAF1	19	39385629	39391154
60528	ELAC2	17	12991612	13018065

115098	CCDC124		19	17933015	17943991
90273	CEACAM21		19	41549518	41586844
79090	TRAPPC6A		19	45162928	45178237
4145	MATK		19	3777970	3802129
54879	ST7L		1	112523514	112620825
8277	TKTL1	X		154295795	154330350
55692	LUC7L		16	188969	229463
29116	MYLIP		6	16129086	16148248
1870	E2F2		1	23506438	23531233
5689	PSMB1		6	170535120	170553307
65220	NADK		1	1751232	1780457
9265	CYTH3		7	6161776	6272644
6856	SYPL1		7	106090503	106112576
10157	AASS		7	122073549	122144255
55200	PLEKHG6		12	6310436	6328506
51188	SS18L2		3	42581840	42595114
4257	MGST1		12	16347142	16609259
1407	CRY1		12	106991364	107093549
4784	NFIX		19	12995608	13098796
23221	RHOBTB2		8	22987417	23020199
54497	HEATR5B		2	36968383	37084372
7095	SEC62		3	169966635	169998373
6224	RPS20		8	56067295	56074581
10178	TENM1	X		124375903	124963817
29954	POMT2		14	77274956	77320884
51534	VTA1		6	142147162	142224685
9031	BAZ1B		7	73440406	73522293
51513	ETV7		6	36354091	36387800
83932	SPRTN		1	231337104	231355023
7756	ZNF207		17	32350117	32381886
83930	STARD3NL		7	38178222	38230671
928	CD9		12	6199715	6238271
9918	NCAPD2		12	6493356	6531955
25900	IFFO1		12	6538375	6556083
56920	SEMA3G		3	52433035	52445103
23166	STAB1		3	52495338	52524495
3423	IDS	X		149476988	149521096
7752	ZNF200		16	3222325	3236221
920	CD4		12	6786858	6820799
695	BTK	X		101349447	101390796
3077	HFE		6	26087281	26098343
22955	SCMH1		1	41027200	41242154
2534	FYN		6	111660332	111873452

6924	ELOA		1	23743155	23762059
54903	MKS1		17	58205441	58219605
3730	ANOS1	X		8528874	8732137
54476	RNF216		7	5620047	5781739
5725	PTBP1		19	797075	812327
23395	LARS2		3	45388561	45554726
54443	ANLN		7	36389821	36453791
2844	RABGAP1		9	122940833	123104866
23177	CEP68		2	65056366	65087004
27033	ZBTB32		19	35704558	35717038
672	BRCA1		17	43044295	43170245
7869	SEMA3B		3	50267558	50277546
4007	PRICKLE3	X		49174802	49186528
4057	LTF		3	46435645	46485234
11183	MAP4K5		14	50418501	50561126
5704	PSMC4		19	39971165	39981764
51667	NUB1		7	151341699	151378449
55703	POLR3B		12	106357748	106510198
23357	ANGEL1		14	76786009	76826246
1774	DNASE1L1	X		154401236	154412112
10046	MAMLD1	X		150361422	150514178
923	CD6		11	60971680	61020377
823	CAPN1		11	65180566	65212006
4190	MDH1		2	63588609	63607197
10463	SLC30A9		4	41990502	42090461
1355	COX15		10	99710868	99732127
10771	ZMYND11		10	134465	254637
64132	XYLT2		17	50346126	50363138
23386	NUDCD3		7	44379119	44490658
55830	GLT8D1		3	52694488	52706032
6556	SLC11A1		2	218382029	218396894
63932	CXorf56	X		119538149	119565409
8685	MARCO		2	118942166	118994660
56981	PRDM11		11	45095806	45235110
972	CD74		5	150400041	150412929
23154	NCDN		1	35557473	35567274
55734	ZFP64		20	52051663	52204308
4331	MNAT1		14	60734742	60969965
23034	SAMD4A		14	54566117	54793315
864	RUNX3		1	24899511	24965121
58473	PLEKHB1		11	73646178	73662819
1992	SERPINB1		6	2832332	2841959
6683	SPAST		2	32063547	32157637

	2273	FHL1	X	136146702	136211359
	55778	ZNF839	14	102317377	102342702
	64429	ZDHHC6	10	112424428	112446917
	57476	GRAMD1B	11	123358428	123627774
	6050	RNH1	11	494512	507300
	9821	RB1CC1	8	52622458	52745843
	23071	ERP44	9	99979185	100099000
	330	BIRC3	11	102317450	102339403
	51022	GLRX2	1	193090866	193106114
	51071	DERA	12	15911302	16037381
	1244	ABCC2	10	99782640	99852594
	23304	UBR2	6	42564062	42693504
	25901	CCDC28A	6	138773509	138793319
	58528	RRAGD	6	89364616	89412273
	3298	HSF2	6	122399551	122433119
	51230	PHF20	20	35771974	35950370
	1890	TYMP	22	50525752	50530032
	10953	TOMM34	20	44942130	44960397
	11231	SEC63	6	107867756	107958208
	23633	KPNA6	1	32108056	32176563
	7431	VIM	10	17228241	17237593
NA		RTEL1-TNFR:	20	63659300	63698684
	355	FAS	10	88990531	89017059
	960	CD44	11	35138870	35232402
	11119	BTN3A1	6	26402237	26415208
	5583	PRKCH	14	61187559	61550976
	3459	IFNGR1	6	137197485	137219449
	11285	B4GALT7	5	177600132	177610330
	7444	VRK2	2	57907629	58159920
	6642	SNX1	15	64094123	64146090
	9519	TBPL1	6	133952170	133990432
	56938	ARNTL2	12	27332854	27425289
	9774	BCLAF1	6	136256627	136289851
	3149	HMGB3	X	150980509	150990771
	23279	NUP160	11	47778087	47848555
	578	BAK1	6	33572547	33580293
	2896	GRN	17	44345246	44353106
	55166	CENPQ	6	49463370	49493107
	6301	SARS1	1	109213918	109238182
	8498	RANBP3	19	5916139	5978142
	5926	ARID4A	14	58298504	58373887
	8100	IFT88	13	20567069	20691437
	55596	ZCCHC8	12	122471599	122501073

10061	ABCF2	7	151211484	151227205
54480	CHPF2	7	151232489	151238827
9846	GAB2	11	78215293	78418348
535	ATP6V0A1	17	42458844	42522611
8554	PIAS1	15	68054179	68198603
321	APBA2	15	28884483	29118315
9168	TMSB10	2	84905656	84906671
25897	RNF19A	8	100257060	100336218
26751	SH3YL1	2	217730	266398
55789	DEPDC1B	5	60596912	60700190
8439	NSMAF	8	58583508	58659853
159	ADSS2	1	244408494	244451909
26228	STAP1	4	67558727	67607337
5981	RFC1	4	39287456	39366375
55773	TBC1D23	3	100260992	100325251
9172	MYOM2	8	2045046	2165552
51121	RPL26L1	5	172958729	172969771
2324	FLT4	5	180601506	180649624
54888	NSUN2	5	6599239	6633291
54455	FBXO42	1	16246840	16352480
4238	MFAP3	5	154038906	154220478
55300	PI4K2B	4	25160663	25279204
27430	MAT2B	5	163503114	163519354
1462	VCAN	5	83471618	83582303
23274	CLEC16A	16	10944564	11182186
9765	ZFYVE16	5	80408013	80483379
79567	RIPOR1	16	67518418	67546788
11284	PNKP	19	49859882	49878351
23035	PHLPP2	16	71637835	71724701
1497	CTNS	17	3636459	3663103
65078	RTN4R	22	20241415	20283246
5685	PSMA4	15	78540405	78552417
55341	LSG1	3	194640791	194672463
140876	RIPOR3	20	50586108	50691542
55775	TDP1	14	89954939	90044764
822	CAPG	2	85394753	85418432
7038	TG	8	132866958	133134903
54802	TRIT1	1	39838110	39883511
153	ADRB1	10	114043866	114046904
5256	PHKA2	X	18892298	18984114
54875	CNTLN	9	17134982	17503923
3309	HSPA5	9	125234853	125241343
22884	WDR37	10	1049538	1132384

56474	CTPS2	X		16587999	16712936
51606	ATP6V1H		8	53715543	53843558
5431	POLR2B		4	56977722	57031158
116984	ARAP2		4	35948221	36244514
7175	TPR		1	186311652	186375693
23247	KIAA0556		16	27550133	27780369
57102	C12orf4		12	4487735	4538508
4134	MAP4		3	47850690	48089272
9734	HDAC9		7	18086949	19002416
26262	TSPAN17		5	176647387	176659054
57545	CC2D2A		4	15469865	15601557
50484	RRM2B		8	102204502	102238961
168850	ZNF800		7	127346790	127431924
608	TNFRSF17		16	11965210	11968068
92017	SNX29		16	11976734	12574287
55187	VPS13D		1	12230030	12512047
10659	CELF2		10	10798397	11336675
23518	R3HDM1		2	135531455	135725270
1298	COL9A2		1	40300489	40317813
1161	ERCC8		5	60873831	60945073
9563	H6PD		1	9234774	9271337
9341	VAMP3		1	7771296	7781432
8863	PER3		1	7784320	7845177
5982	RFC2		7	74231499	74254458
57492	ARID1B		6	156776020	157210779
89801	PPP1R3F	X		49269793	49301461
3074	HEXB		5	74640023	74722647
51528	JKAMP		14	59484443	59505410
9498	SLC4A8		12	51391317	51515763
5601	MAPK9		5	180233143	180292099
84067	FAM160A2		11	6211345	6234711
9454	HOMER3		19	18929201	18941261
5888	RAD51		15	40694774	40732340
10721	POLQ		3	121431427	121546641
1535	CYBA		16	88643289	88651054
23593	HEBP2		6	138403531	138422197
54477	PLEKHA5		12	19129752	19376400
6307	MSMO1		4	165327667	165343164
8574	AKR7A2		1	19303965	19312146
4826	NNAT		20	37521206	37523690
55850	USE1		19	17215346	17219829
23101	MCF2L2		3	183178043	183428778
55745	AP5M1		14	57268924	57298742

29945	ANAPC4	4	25377213	25418498
27095	TRAPPC3	1	36136570	36156053
954	ENTPD2	9	137048107	137054061
51742	ARID4B	1	235131634	235328219
23596	OPN3	1	241590102	241677376
10806	SDCCAG8	1	243256034	243500091
23095	KIF1B	1	10210805	10381603
2296	FOXC1	6	1609915	1613897
25771	TBC1D22A	22	46762617	47175699
23201	FAM168A	11	73400487	73598189
84957	RELT	11	73376399	73397474
2581	GALC	14	87837820	87993665
51602	NOP58	2	202265736	202303661
3757	KCNH2	7	150944961	150978321
26999	CYFIP2	5	157266079	157395595
5610	EIF2AK2	2	37099210	37157065
57602	USP36	17	78787381	78841441
58508	KMT2C	7	152134922	152436005
55283	MCOLN3	1	85018082	85048500
54969	HPF1	4	169729470	169757944
54542	RC3H2	9	122844556	122905359
55540	IL17RB	3	53846568	53865794
6646	SOAT1	1	179293714	179358680
5318	PKP2	12	32790755	32896777
4438	MSH4	1	75796882	75913242
9474	ATG5	6	106045423	106325791
57458	TMCC3	12	94567122	94650557
57095	PITHD1	1	23778418	23788232
57504	MTA3	2	42494569	42756947
8975	USP13	3	179653040	179789401
23200	ATP11B	3	182793503	182921629
5218	CDK14	7	90466424	91210590
54432	YIPF1	1	53851719	53889798
64718	UNKL	16	1363205	1414751
64761	PARP12	7	140023749	140062951
5832	ALDH18A1	10	95605941	95656711
5188	GATB	4	151670504	151761007
4084	MXD1	2	69897688	69942945
5128	CDK17	12	96278261	96400480
6515	SLC2A3	12	7919230	7936187
8531	YBX3	12	10699089	10723323
55359	STYK1	12	10618923	10674318
11054	OGFR	20	62804835	62814000

10076	PTPRU	1	29236516	29326813
30	ACAA1	3	38103129	38137242
586	BCAT1	12	24810024	24949101
10787	NCKAP1	2	182909115	183038858
6433	SFSWAP	12	131711081	131799737
10188	TNK2	3	195863364	195911945
65056	GPBP1	5	57173948	57264679
1431	CS	12	56271699	56300391
4058	LTK	15	41503637	41513887
81671	VMP1	17	59707192	59842255
10513	APPBP2	17	60443158	60526242
1975	EIF4B	12	53006158	53042209
6141	RPL18	19	48615328	48619184
770	CA11	19	48637946	48646187
11338	U2AF2	19	55654146	55674715
10838	ZNF275	X	153334147	153360110
8776	MTMR1	X	150692971	150765108
57143	ADCK1	14	77800109	77935014
6047	RNF4	4	2462220	2625320
841	CASP8	2	201233443	201287711
51768	TM7SF3	12	26971579	27014434
10402	ST3GAL6	3	98732236	98821201
9914	ATP2C2	16	84368527	84464187
8625	RFXANK	19	19192229	19201869
54929	TMEM161A	19	19119169	19138513
9170	LPAR2	19	19623655	19628930
10147	SUGP2	19	18990888	19034023
6558	SLC12A2	5	128083766	128189677
10347	ABCA7	19	1039997	1065572
11218	DDX20	1	111755245	111768000
55711	FAR2	12	29149103	29340980
22904	SBNO2	19	1107637	1174268
10362	HMG20B	19	3572777	3579088
10203	CALCRL	2	187341964	187448460
6882	TAF11	6	34877462	34888071
8943	AP3D1	19	2100988	2164468
9351	SLC9A3R2	16	2025356	2039026
2773	GNAI3	1	109548615	109600195
3843	IPO5	13	97953658	98024296
5586	PKN2	1	88684222	88836255
55388	MCM10	10	13161554	13211104
1606	DGKA	12	55927319	55954027
55854	ZC3H15	2	186486253	186509361

51167	CYB5R4		6	83859656	83967423
1308	COL17A1		10	104031286	104085880
119391	GSTO2		10	104268873	104304950
5588	PRKCQ		10	6427143	6580301
7089	TLE2		19	2997639	3047635
51665	ASB1		2	238426742	238452250
83641	FAM107B		10	14518557	14774897
4199	ME1		6	83210402	83431051
8621	CDK13		7	39950121	40099580
10797	MTHFD2		2	74198610	74217565
55810	FOXJ2		12	8032716	8055517
4904	YBX1		1	42682418	42703805
5141	PDE4A		19	10416773	10469630
5525	PPP2R5A		1	212285410	212361853
1994	ELAVL1		19	7958573	8005659
57609	DIP2B		12	50504985	50748657
6602	SMARCD1		12	50085200	50100707
259266	ASPM		1	197084127	197146694
64834	ELOVL1		1	43363398	43368074
2263	FGFR2		10	121478332	121598458
55631	LRRC40		1	70144805	70205579
51015	ISOC1		5	129094749	129114028
55623	THUMPD1		16	20702816	20742084
26153	KIF26A		14	104138723	104180894
10734	STAG3		7	100177563	100221488
4649	MYO9A		15	71822291	72118577
3422	IDI1		10	1039152	1049170
6672	SP100		2	230415942	230545606
1316	KLF6		10	3775996	3785281
4756	NEO1		15	73051710	73305205
9399	STOML1		15	73978926	73994622
54505	DHX29		5	55256055	55307694
30836	DNTTIP2		1	93866284	93879918
79091	METTL22		16	8621683	8649654
7158	TP53BP1		15	43403061	43510728
7544	ZFY	Y		2935281	2982506
55699	IARS2		1	220094132	220148041
89795	NAV3		12	77324641	78213010
3421	IDH3G	X		153785766	153794523
6093	ROCK1		18	20946906	21111813
865	CBFB		16	67028984	67101058
8692	HYAL2		3	50317790	50322782
9759	HDAC4		2	239048168	239401654

3430	IFI35		17	43006740	43014456
4205	MEF2A		15	99565417	99716466
7030	TFE3	X		49028726	49043410
4943	TBC1D25	X		48539714	48562609
2182	ACSL4	X		109624244	109733403
56850	GRIPAP1	X		48973720	49002264
55771	PRR11		17	59155732	59206709
23250	ATP11A		13	112690329	112887168
9741	LAPTM4A		2	20032650	20051628
51447	IP6K2		3	48688003	48740353
55133	SRBD1		2	45388680	45612165
27248	ERLEC1		2	53787044	53818819
5837	PYGM		11	64746389	64759974
54549	SDK2		17	73334384	73644445
55746	NUP133		1	229440259	229508341
10681	GNB5		15	52115105	52191369
5873	RAB27A		15	55202966	55319113
7353	UFD1		22	19449911	19479202
2984	GUCY2C		12	14612632	14696599
57661	PHRF1		11	576470	612222
8518	ELP1		9	108867517	108934124
4925	NUCB2		11	17208153	17349980
5217	PFN2		3	149964904	150050788
27071	DAPP1		4	99816827	99870190
23446	SLC44A1		9	105244622	105439171
54916	TMEM260		14	56488354	56650606
23293	SMG6		17	2059839	2303785
8218	CLTCL1		22	19179473	19291719
10272	FSTL3		19	676392	683392
5423	POLB		8	42338454	42371808
55062	WIPI1		17	68420948	68457513
3340	NDST1		5	150485818	150558211
26986	PABPC1		8	100685816	100722809
29105	CFAP20		16	58113592	58129381
1459	CSNK2A2		16	58157907	58198106
8891	EIF2B3		1	44850522	44986722
6949	TCOF1		5	150357629	150400308
998	CDC42		1	22025511	22101360
56852	RAD18		3	8775402	8963773
6160	RPL31		2	101002229	101024032
23161	SNX13		7	17790761	17940501
79658	ARHGAP10		4	147732063	148072776
6196	RPS6KA2		6	166409364	166906451

9319	TRIP13	5	892884	919357
6596	HLTF	3	149030127	149086554
140469	MYO3B	2	170178145	170655171
51109	RDH11	14	67676800	67695793
5566	PRKACA	19	14091688	14118084
22859	ADGRL1	19	14147743	14206187
23503	ZFYVE26	14	67727374	67816590
22905	EPN2	17	19215615	19336715
26469	PTPN18	2	130356045	130375405
55679	LIMS2	2	127638381	127681786
224	ALDH3A2	17	19648136	19685760
7037	TFRC	3	196027183	196082096
64398	MPP5	14	67241342	67336061
8243	SMC1A	X	53374149	53422728
3161	HMMR	5	163460203	163491941
55743	CHFR	12	132822187	132956304
8974	P4HA2	5	132191838	132295315
2213	FCGR2B	1	161663147	161678654
4775	NFATC3	16	68084751	68229259
23291	FBXW11	5	171861549	172006873
2121	EVC	4	5711201	5814305
51009	DERL2	17	5471254	5486811
54847	SIDT1	3	113532296	113629578
10335	MRVI1	11	10573091	10693988
79041	TMEM38A	19	16661139	16690023
8907	AP1M1	19	16197578	16245907
5817	PVR	19	44643798	44666162
7515	XRCC1	19	43543311	43580473
80216	ALPK1	4	112285509	112442621
5151	PDE8A	15	84980440	85139145
54475	NLE1	17	35128730	35142304
6389	SDHA	5	218241	257082
5927	KDM5A	12	280057	389320
10170	DHRS9	2	169064789	169096167
9175	MAP3K13	3	185282941	185489094
6480	ST6GAL1	3	186930502	187078553
771	CA12	15	63321378	63381846
59277	NTN4	12	95657807	95791189
617	BCS1L	2	218658764	218663443
9187	SLC24A1	15	65611366	65660995
8578	SCARF1	17	1633858	1645744
23140	ZZEF1	17	4004445	4143030
56005	MYDGF	19	4641374	4670362

64431	ACTR6	12	100199122	100241865
54962	TIPIN	15	66336191	66386746
8672	EIF4G3	1	20806292	21176888
79023	NUP37	12	102073103	102120120
51512	GTSE1	22	46296870	46330810
10512	SEMA3C	7	80742538	80922359
55020	TTC38	22	46267961	46294008
38	ACAT1	11	108116695	108147603
27332	ZNF638	2	71276561	71435069
119	ADD2	2	70607618	70768225
9605	VPS9D1	16	89707134	89720898
4140	MARK3	14	103385392	103503831
5250	SLC25A3	12	98593591	98606379
64778	FNDC3B	3	172039578	172401669
6624	FSCN1	7	5592816	5606655
60	ACTB	7	5527148	5563784
284403	WDR62	19	36054881	36105108
1739	DLG1	3	197042560	197299330
9733	SART3	12	108522214	108561400
22920	KIFAP3	1	169921326	170085208
4175	MCM6	2	135839626	135876443
10179	RBM7	11	114400030	114414203
11176	BAZ2A	12	56595596	56636816
25930	PTPN23	3	47381011	47413435
7374	UNG	12	109097597	109110992
5362	PLXNA2	1	208022242	208244384
10615	SPAG5	17	28577565	28599025
88455	ANKRD13A	12	109999186	110039763
7163	TPD52	8	80034745	80231232
32	ACACB	12	109116595	109268226
55824	PAG1	8	80967810	81112068
55094	GPATCH1	19	33080899	33130542
22978	NT5C2	10	103088017	103193306
55796	MBNL3	X	132369317	132489968
5909	RAP1GAP	1	21596215	21669363
5609	MAP2K7	19	7903843	7914478
7155	TOP2B	3	25597905	25664907
29089	UBE2T	1	202331657	202341980
4660	PPP1R12B	1	202348699	202592706
23032	USP33	1	77695987	77759852
6626	SNRPA	19	40750637	40765389
56915	EXOSC5	19	41386374	41397359
4034	LRCH4	7	100574011	100586129

832	CAPZB		1	19338775	19485539
7107	GPR137B		1	236142505	236221865
8530	CST7		20	24949269	24959928
273	AMPH		7	38383704	38631420
369	ARAF	X		47561100	47571920
56922	MCCC1		3	183015218	183116075
27074	LAMP3		3	183122215	183163839
10529	NEBL		10	20779973	21174187
5289	PIK3C3		18	41955234	42087830
7289	TULP3		12	2877223	2941138
5527	PPP2R5C		14	101761798	101927989
1906	EDN1		6	12290361	12297194
8028	MLLT10		10	21524646	21743630
55063	ZCWPW1		7	100400826	100428992
27334	P2RY10	X		78945332	78963727
4898	NRDC		1	51789191	51878937
7419	VDAC3		8	42391624	42405937
57690	TNRC6C		17	77959240	78108835
9139	CBFA2T2		20	33490075	33650036
58476	TP53INP2		20	34704339	34713439
57644	MYH7B		20	34955810	35002437
7161	TP73		1	3652516	3736201
51619	UBE2D4		7	43926436	43956136
114880	OSBPL6		2	178194481	178402893
6507	SLC1A3		5	36606355	36688334
7520	XRCC5		2	216107464	216206303
56925	LXN		3	158645822	158672648
11262	SP140		2	230203110	230313215
8569	MKNK1		1	46557408	46616843
7145	TNS1		2	217799588	218033982
10411	RAPGEF3		12	47734363	47771040
634	CEACAM1		19	42507304	42561234
29843	SENP1		12	48042897	48106079
51207	DUSP13		10	75094432	75109221
3991	LIPE		19	42401514	42427388
5050	PAFAH1B3		19	42297033	42303546
2037	EPB41L2		6	130839347	131063322
26002	MOXD1		6	132296055	132401475
8417	STX7		6	132445867	132513198
55183	RIF1		2	151409883	151508013
6595	SMARCA2		9	1980290	2193624
27244	SESN1		6	108986437	109094819
11043	MID2	X		107825755	107927193

50509	COL5A3	19	9959561	10010504
9933	PUM3	9	2720469	2844095
5663	PSEN1	14	73136418	73223691
56650	CLDND1	3	98497912	98523066
5891	MOK	14	102224500	102305190
3320	HSP90AA1	14	102080742	102139699
22839	DLGAP4	20	36306336	36528637
22997	IGSF9B	11	133896438	133956985
10403	NDC80	18	2571557	2616635
6932	TCF7	5	134114681	134151865
28962	OSTM1	6	108041409	108165854
55218	EXD2	14	69191511	69244020
9262	STK17B	2	196133583	196176503
8555	CDC14B	9	96490241	96619843
65985	AACS	12	125065434	125143333
9812	DELE1	5	141923855	141942047
51668	HSPB11	1	53916574	53945929
6604	SMARCD3	7	151238764	151277896
2533	FYB1	5	39105252	39274528
55437	STRADB	2	201387858	201480846
114899	C1QTNF3	5	34019448	34043832
4200	ME2	18	50879080	50954257
7188	TRAF5	1	211326615	211374946
29093	MRPL22	5	154941073	154969411
25929	GEMIN5	5	154887411	154938211
23085	ERC1	12	990509	1495933
7514	XPO1	2	61477849	61538626
11342	RNF13	3	149812708	149962139
57226	LYRM2	6	89568144	89638749
594	BCKDHB	6	80106647	80346270
79670	TUT7	9	86287733	86354454
22894	DIS3	13	72752169	72782096
1540	CYLD	16	50742050	50801935
79891	ZNF671	19	57719751	57727624
55659	ZNF416	19	57571566	57578911
54807	ZNF586	19	57769655	57819939
9422	ZNF264	19	57191500	57222846
25978	CHMP2B	3	87227271	87255556
10269	ZMPSTE24	1	40258236	40294180
56910	STARD7	2	96184859	96208825
84273	NOA1	4	56963350	56977606
3034	HAL	12	95972662	95996365
2950	GSTP1	11	67583742	67586656

57613	FAM234B	12	13044381	13142521
51729	WBP11	12	14784582	14803486
79570	NKAIN1	1	31179745	31239887
200081	TXLNA	1	32179675	32198285
3030	HADHA	2	26190635	26244672
966	CD59	11	33703010	33736479
63035	BCORL1	X	129981107	130058083
2219	FCN1	9	134903232	134917912
55892	MYNN	3	169772831	169789716
9522	SCAMP1	5	78360611	78480739
5550	PREP	6	105273220	105454062
57531	HACE1	6	104728094	104859919
81929	SEH1L	18	12947133	12987536
57590	WDFY1	2	223855716	223945357
5016	OVGP1	1	111414319	111427735
4216	MAP3K4	6	160991727	161117385
5243	ABCB1	7	87503017	87713323
231	AKR1B1	7	134442356	134459284
4528	MTIF2	2	55236595	55269347
23259	DDHD2	8	38225218	38275558
22996	TTC39A	1	51287258	51345116
2060	EPS15	1	51354263	51519266
4998	ORC1	1	52372829	52404423
10523	CHERP	19	16517894	16542437
8438	RAD54L	1	46246461	46278480
3301	DNAJA1	9	33025273	33039907
51510	CHMP5	9	33264879	33282070
4799	NFX1	9	33290512	33371157
27292	DIMT1	5	62347284	62403939
27102	EIF2AK1	7	6022247	6059175
51314	NME8	7	37848597	37900397
80271	ITPKC	19	40717112	40740860
4680	CEACAM6	19	41750977	41772211
54469	ZFAND6	15	80059568	80138393
55787	TXLNG	X	16786432	16844519
7462	LAT2	7	74199652	74229834
9183	ZW10	11	113733187	113773735
79796	ALG9	11	111782195	111871581
8310	ACOX3	4	8366282	8440723
7205	TRIP6	7	100867387	100873454
51593	SRRT	7	100875103	100888664
581	BAX	19	48954815	48961798
51701	NLK	17	28041737	28196381

	94005	PIGS	17	28553383	28571794
	9509	ADAMTS2	5	179110853	179345461
	9489	PGS1	17	78378649	78425114
	1071	CETP	16	56961923	56983845
	55239	OGFOD1	16	56451521	56479104
	6452	SH3BP2	4	2793071	2841098
	8602	NOP14	4	2937933	2963406
	118	ADD1	4	2843857	2930076
	79944	L2HGDH	14	50237563	50312229
	57544	TXNDC16	14	52430596	52552522
	51637	RTRAF	14	51989514	52010694
	10992	SF3B2	11	66050729	66069308
	51290	ERGIC2	12	29337352	29381189
	6790	AURKA	20	56369389	56392337
	57091	CASS4	20	56412112	56460387
	8570	KHSRP	19	6413348	6424794
	2767	GNA11	19	3094362	3123999
	22974	TPX2	20	31739271	31801805
NA		FER1L4	20	35558737	35607562
	23348	DOCK9	13	98793429	99086625
	55287	TMEM40	3	12733528	12769457
	23368	PPP1R13B	14	103733195	103847575
	8455	ATRN	20	3471018	3651118
	6614	SIGLEC1	20	3686970	3707128
	79175	ZNF343	20	2481817	2524702
	57593	EBF4	20	2692874	2760108
	57506	MAVS	20	3846799	3876123
	9762	LZTS3	20	3162617	3173592
	54997	TESC	12	117038923	117099479
	51575	ESF1	20	13714322	13784886
	10741	RBBP9	20	18486540	18497225
	9962	SLC23A2	20	4852356	5010293
	55184	DZANK1	20	18383367	18467281
	84678	KDM2B	12	121429096	121581023
	55617	TASP1	20	13389392	13638932
	4938	OAS1	12	112906783	112933222
	22895	RPH3A	12	112570380	112898881
	5037	PEBP1	12	118136124	118145584
	2521	FUS	16	31180138	31191605
	3476	IGBP1	X	70133447	70166324
	53827	FXYD5	19	35154730	35169881
	57655	GRAMD1A	19	34994784	35026471
	9843	HEPH	X	66162549	66268867

29965	CDIP1		16	4510669	4538828
113540	CMTM1		16	66566393	66579137
23193	GANAB		11	62624826	62646726
51291	GMIP		19	19629476	19643657
55285	RBM41	X		107064420	107118823
332	BIRC5		17	78214186	78225636
3902	LAG3		12	6772512	6778455
57062	DDX24		14	94048287	94081212
393	ARHGAP4	X		153907367	153934999
5996	RGS1		1	192575763	192580024
51650	MRPS33		7	141002610	141015228
4708	NDUFB2		7	140690777	140722790
10726	NUDC		1	26900238	26946871
11213	IRAK3		12	66189195	66254622
4069	LYZ		12	69348381	69354234
7023	TFAP4		16	4257186	4273075
10081	PDCD7		15	65117379	65133808
51726	DNAJB11		3	186567403	186585800
8761	PABPC4		1	39560816	39576790
1947	EFNB1	X		68829021	68842160
24137	KIF4A	X		70290104	70420886
57605	PITPNM2		12	122983480	123150015
55763	EXOC1		4	55853648	55905086
58484	NLRC4		2	32224453	32265732
54517	PUS7		7	105439661	105522271
4897	NRCAM		7	108147623	108456717
1738	DLD		7	107891162	107931730
23335	WDR7		18	56651343	57036606
3568	IL5RA		3	3066326	3126613
54918	CMTM6		3	32481312	32502852
3655	ITGA6		2	172427354	172506459
55573	CDV3		3	133573730	133590261
54890	ALKBH5		17	18183078	18209954
22861	NLRP1		17	5499427	5619424
9552	SPAG7		17	4959226	4967817
23594	ORC6		16	46689643	46698394
55012	PPP2R3C		14	35085467	35122517
54930	HAUS4		14	22946228	22957161
84502	JPH4		14	23568035	23578790
23428	SLC7A8		14	23125295	23183674
51310	SLC22A17		14	23346306	23352912
23256	SCFD1		14	30622311	30735850
3183	HNRNPC		14	21209136	21269494

8487	GEMIN2	14	39114223	39136973
825	CAPN3	15	42359498	42412949
7430	EZR	6	158765741	158819368
4637	MYL6	12	56158346	56163496
63967	CLSPN	1	35720218	35769978
117584	RFFL	17	35005990	35089319
201294	UNC13D	17	75827225	75844717
79157	MFSD11	17	76735865	76781449
1808	DPYSL2	8	26514031	26658178
8318	CDC45	22	19479457	19520612
1312	COMT	22	19941733	19969975
55350	VNN3	6	132722787	132736935
55862	ECHDC1	6	127288712	127343609
9209	LRRFIP2	3	37052626	37183689
990	CDC6	17	40287879	40304657
139596	UPRT	X	75156388	75304885
8697	CDC23	5	138187650	138213343
8086	AAAS	12	53307456	53324864
4214	MAP3K1	5	56815549	56896152
29911	HOOK2	19	12763003	12872740
55151	TMEM38B	9	105694541	105776629
23511	NUP188	9	128947699	129007096
1384	CRAT	9	129094794	129111189
54187	NANS	9	98056732	98083077
55357	TBC1D2	9	98199011	98255649
55280	CWF19L1	10	100232298	100267680
1390	CREM	10	35126791	35212958
10321	CRISP3	6	49727384	49744437
2289	FKBP5	6	35573585	35728583
6732	SRPK1	6	35832966	35921342
55168	MRPS18A	6	43671303	43687791
114327	EFHC1	6	52362123	52529886
3326	HSP90AB1	6	44246166	44253888
988	CDC5L	6	44387706	44450425
23411	SIRT1	10	67884656	67918390
80173	IFT74	9	26947039	27062930
3717	JAK2	9	4984390	5129948
3594	IL12RB1	19	18058995	18098944
25	ABL1	9	130713016	130887675
51100	SH3GLB1	1	86704570	86748184
8317	CDC7	1	91500851	91525764
6319	SCD	10	100347233	100364826
11018	TMED1	19	10832067	10836318

3983	ABLIM1	10	114431113	114768061
79956	ERMP1	9	5765076	5833117
22931	RAB18	10	27504174	27542239
8829	NRP1	10	33177492	33336262
23555	TSPAN15	10	69451465	69507666
79629	OCEL1	19	17226213	17229219
4650	MYO9B	19	17075781	17214537
57828	CATSPERG	19	38335775	38370943
112755	STX1B	16	30989256	31010638
9739	SETD1A	16	30957754	30984664
513	ATP5F1D	19	1241746	1244825
4670	HNRNPM	19	8444767	8489114
4713	NDUFB7	19	14566078	14572066
9524	TECR	19	14517085	14565980
5434	POLR2E	19	1086574	1095380
610	HCN2	19	589881	617159
4616	GADD45B	19	2476122	2478259
84861	KLHL22	22	20441519	20495844
9342	SNAP29	22	20859007	20891214
1399	CRKL	22	20917407	20953747
6598	SMARCB1	22	23786931	23838009
91319	DERL3	22	23834503	23839128
5008	OSM	22	30262829	30266851
23523	CABIN1	22	24011192	24178628
83874	TBC1D10A	22	30291990	30326947
10291	SF3A1	22	30331988	30356919
2687	GGT5	22	24219654	24245142
200312	RNF215	22	30368811	30421771
23384	SPECC1L	22	24270817	24417739
23759	PPIL2	22	21666009	21700015
51733	UPB1	22	24494107	24528390
6634	SNRPD3	22	24555958	24582052
23481	PES1	22	30576625	30607083
339665	SLC35E4	22	30635781	30669016
4242	MFNG	22	37469063	37486393
6576	SLC25A1	22	19175581	19178739
157	GRK3	22	25564675	25729294
23616	SH3BP1	22	37634654	37656119
23544	SEZ6L	22	26169462	26383597
3956	LGALS1	22	37675636	37679802
113791	PIK3IP1	22	31281594	31292534
23598	PATZ1	22	31325804	31346346
11078	TRIOBP	22	37697048	37776556

129138	ANKRD54	22	37830855	37849327
51386	EIF3L	22	37848868	37889407
79879	CCDC134	22	41800679	41826299
9681	DEPDC5	22	31753867	31908033
9463	PICK1	22	38056311	38075701
23331	TTC28	22	27978014	28679865
79019	CENPM	22	41938737	41947152
10521	DDX17	22	38483438	38507660
6942	TCF20	22	42160013	42343616
25776	CBY1	22	38656636	38673854
51493	RTCB	22	32387582	32412248
9929	JOSD1	22	38685543	38701556
9567	GTPBP1	22	38705742	38738299
9701	PPP6R2	22	50343304	50445090
1727	CYB5R3	22	42617840	42649392
25807	RHBDD3	22	29259872	29268209
25809	TTLL1	22	43039516	43089419
162	AP1B1	22	29327680	29423179
10043	TOM1	22	35299275	35347992
4744	NEFH	22	29480218	29491390
1120	CHKB	22	50578959	50601455
638	BIK	22	43110750	43129712
4174	MCM5	22	35400134	35425431
164668	APOBEC3H	22	39097224	39104067
23170	TTLL12	22	43166622	43187134
5155	PDGFB	22	39223359	39244982
55954	ZMAT5	22	29730956	29767011
10454	TAB1	22	39399778	39437060
84164	ASCC2	22	29788609	29838304
8897	MTMR3	22	29883169	30030868
54471	MIEF1	22	39499432	39518132
80832	APOL4	22	36189124	36204840
8542	APOL1	22	36253071	36267530
25828	TXN2	22	36467046	36481640
80020	FOXRED2	22	36487190	36507101
4689	NCF4	22	36860988	36878017
6767	ST13	22	40824535	40856639
3560	IL2RB	22	37125843	37175054
9978	RBX1	22	40951347	40973309
83746	L3MBTL2	22	41205282	41231271
5905	RANGAP1	22	41245611	41286251
84844	PHF5A	22	41459717	41468692
50	ACO2	22	41469117	41528989

171568	POLR3H	22	41525799	41544606
55687	TRMU	22	46330875	46357340
5372	PMM1	22	41576900	41589871
64781	CERK	22	46684410	46738252
23774	BRD1	22	49773283	49827512
9889	ZBED4	22	49853844	49890080
23351	KHNYN	14	24429286	24441843
2287	FKBP3	14	45115599	45135319
1511	CTSG	14	24573518	24576250
2999	GZMH	14	24606480	24609699
10419	PRMT5	14	22920525	22929408
1690	COCH	14	30874514	30895065
11154	AP4S1	14	31025106	31130996
5427	POLE2	14	49643555	49688422
51199	NIN	14	50719763	50831121
5706	PSMC6	14	52707178	52728590
80821	DDHD1	14	53036745	53153323
1033	CDKN3	14	54396849	54420218
10668	CGRRF1	14	54509812	54539292
51382	ATP6V1D	14	67294371	67360265
5684	PSMA3	14	58244843	58272012
283578	TMED8	14	77335029	77377094
10598	AHSA1	14	77457870	77469472
9517	SPTLC2	14	77505997	77616773
5641	LGMN	14	92703807	92748679
22938	SNW1	14	77717599	77761207
3705	ITPK1	14	92936914	93116320
51676	ASB2	14	93934166	93976739
2079	ERH	14	69380128	69398299
6430	SRSF5	14	69726900	69772005
23405	DICER1	14	95086228	95158010
79882	ZC3H14	14	88562970	88627596
22990	PCNX1	14	70907405	71115382
51527	GSKIP	14	96363452	96387288
7443	VRK1	14	96797382	96931722
9252	RPS6KA5	14	90847861	91060641
55671	PPP4R3A	14	91457611	91510554
60686	C14orf93	14	22985894	23010166
7528	YY1	14	100238298	100282788
22985	ACIN1	14	23058564	23095614
51550	CINP	14	102341102	102362916
6729	SRP54	14	34981957	35029567
5106	PCK2	14	24094053	24110598

80344	DCAF11	14	24114195	24125242
5687	PSMA6	14	35278633	35317493
5721	PSME2	14	24143362	24147570
9985	REC8	14	24171853	24180257
51292	GMPR2	14	24232422	24239242
63935	PCIF1	20	45934683	45948023
2937	GSS	20	34928432	34956027
4318	MMP9	20	46008908	46016561
5834	PYGB	20	25248085	25298012
26090	ABHD12	20	25294742	25390835
9837	GINS1	20	25407673	25452628
55245	UQCC1	20	35302566	35412031
4605	MYBL2	20	43667019	43716495
57446	NDRG3	20	36651766	36746090
4773	NFATC2	20	51386957	51562831
80336	PABPC1L	20	44910060	44959035
23394	ADNP	20	50888916	50931437
5203	PFDN4	20	54208087	54228052
1477	CSTF1	20	56392371	56406362
8480	RAE1	20	57351223	57379211
7165	TPD52L2	20	63865228	63891545
51497	NELFCD	20	58981208	58995133
24148	PRPF6	20	63981132	64033100
26164	MTG2	20	62183029	62203568
28231	SLCO4A1	20	62642503	62685785
4923	NTSR1	20	62708836	62762771
54994	GID8	20	62938147	62948475
55738	ARFGAP1	20	63272785	63289790
5753	PTK6	20	63528001	63537376
994	CDC25B	20	3786772	3806121
140862	ISM1	20	13221274	13300651
11237	RNF24	20	3927309	4015558
79133	NDUFAF5	20	13785007	13821580
1457	CSNK2A1	20	472498	543835
81502	HM13	20	31514410	31577923
9751	SNPH	20	1266280	1309328
10483	SEC23B	20	18507520	18561415
140711	TLDC2	20	36876121	36894235
51340	CRNKL1	20	20034368	20056046
23509	POFUT1	20	32207880	32238658
25939	SAMHD1	20	36890229	36951843
63905	MANBAL	20	37289638	37317260
182	JAG1	20	10637684	10673999

51654	CDK5RAP1		20	33358839	33401561
6640	SNTA1		20	33407957	33443763
1869	E2F1		20	33675477	33686385
58490	RPRD1B		20	38033725	38127780
11264	PXMP4		20	33702758	33720319
671	BPI		20	38304150	38337505
79913	ACTR5		20	38748460	38772520
81610	FAM83D		20	38926312	38953106
116092	DNTTIP1		20	45791954	45811427
128869	PIGU		20	34560542	34698790
10005	ACOT8		20	45841721	45857405
22850	ADNP2		18	80109262	80147523
79863	RBFA		18	80034389	80050651
64863	METTL4		18	2537525	2571509
9663	LPIN2		18	2916994	3013315
23347	SMCHD1		18	2655726	2805017
8736	MYOM1		18	3066807	3219968
10627	MYL12A		18	3247481	3256236
79959	CEP76		18	12661833	12702777
55125	CEP192		18	12991362	13125052
8731	RNMT		18	13726660	13764558
4092	SMAD7		18	48919853	48952052
54941	RNF125		18	32018825	32073219
5932	RBBP8		18	22798261	23026488
340547	VSIG1	X		108044970	108079184
5716	PSMD10	X		108084207	108091549
115201	ATG4A	X		108091668	108154671
412	STS	X		7147237	7804358
79576	NKAP	X		119920672	119943751
55916	NXT2	X		109535781	109544690
51311	TLR8	X		12906620	12923169
6839	SUV39H1	X		48695554	48709016
331	XIAP	X		123859724	123913979
215	ABCD1	X		153724856	153744755
28952	CCDC22	X		49235470	49250520
6855	SYP	X		49187804	49200259
5355	PLP2	X		49171898	49175235
660	BMX	X		15464246	15556529
5358	PLS3	X		115561174	115650861
340554	ZC3H12B	X		65366638	65507887
5931	RBBP7	X		16839283	16870362
3750	KCND1	X		48961378	48971844
2332	FMR1	X		147911919	147951125

10084	PQBP1	X		48890197	48903143
2010	EMD	X		154379273	154381523
6901	TAZ	X		154411524	154421726
6102	RP2	X		46837043	46882358
8237	USP11	X		47232866	47248328
27336	HTATSF1	X		136497079	136512346
959	CD40LG	X		136648158	136660390
7076	TIMP1	X		47582408	47586789
5303	PIN4	X		72181353	72302926
64840	PORCN	X		48508962	48520814
10916	MAGED2	X		54807599	54816015
5935	RBM3	X		48574449	48581162
51260	PBDC1	X		76173040	76178314
56271	BEX4	X		103215108	103217246
54602	NDFIP2		13	79481124	79556075
10673	TNFSF13B		13	108251240	108308484
22862	FNDC3A		13	48975912	49209779
81602	CDADC1		13	49247925	49293485
81617	CAB39L		13	49308650	49444064
8874	ARHGEF7		13	111114559	111305737
143	PARP4		13	24420931	24512778
55578	SUPT20H		13	37009312	37059713
10240	MRPS31		13	40729128	40771190
3839	KPNA3		13	49699320	49792682
2321	FLT1		13	28300346	28495145
28984	RGCC		13	41457550	41470871
160851	DGKH		13	42040036	42256578
84056	KATNAL1		13	30202630	30307551
79758	DHRS12		13	51767993	51804162
1203	CLN5		13	76990660	77019143
10562	OLFM4		13	53028813	53052057
10232	MSLN		16	760734	768865
23361	ZNF629		16	30778456	30787205
3299	HSF4		16	67164681	67169945
79153	GDPD3		16	30104810	30113537
79767	ELMO3		16	67199111	67204029
5257	PHKB		16	47461123	47701523
57149	LYRM1		16	20899868	20925006
9688	NUP93		16	56730118	56850286
83752	LONP2		16	48244300	48363122
51090	PLLP		16	57248547	57284672
10664	CTCF		16	67562467	67639177
65057	ACD		16	67657512	67660815

79650	USB1	16	57999546	58021618
3038	HAS3	16	69105653	69118719
79613	TANGO6	16	68843531	69085182
55512	SMPD3	16	68358327	68448508
84138	SLC7A6OS	16	68284503	68310946
79726	WDR59	16	74871362	75000173
56942	CMC2	16	80966448	81020270
8131	NPRL3	16	84271	138860
54550	NECAB2	16	83968632	84002776
4833	NME4	16	396725	410367
64788	LMF1	16	853634	981318
1186	CLCN7	16	1444934	1475084
79791	FBXO31	16	87326987	87392142
10273	STUB1	16	680224	682870
9028	RHBDL1	16	675666	678268
7329	UBE2I	16	1308880	1327018
4210	MEFV	16	3242028	3256627
29904	EEF2K	16	22206278	22288738
2935	GSPT1	16	11868128	11916082
7727	ZNF174	16	3401215	3409364
56061	UBFD1	16	23557721	23574389
260429	PRSS33	16	2783953	2787948
6923	ELOB	16	2771414	2777280
23062	GGA2	16	23463542	23521995
57478	USP31	16	23061406	23149270
9093	DNAJA3	16	4425805	4456775
51283	BFAR	16	14632931	14669236
4150	MAZ	16	29806106	29811164
50615	IL21R	16	27402174	27452042
51760	SYT17	16	19167971	19268332
79838	TMC5	16	19410496	19499113
9738	CCP110	16	19523811	19553408
57020	VPS35L	16	19555240	19706793
366	AQP9	15	58138169	58185911
79719	AAGAB	15	67200667	67255195
114294	LACTB	15	63121833	63142061
8925	HERC1	15	63608618	63833948
9325	TRIP4	15	64387748	64455303
8766	RAB11A	15	65726054	65891989
1512	CTSH	15	78921058	78949574
2184	FAH	15	80152490	80186946
26015	RPAP1	15	41517176	41544269
64397	ZNF106	15	42412823	42491141

79895	ATP8B4	15	49858238	50182817
90427	BMF	15	40087890	40108892
23312	DMXL2	15	51447711	51622833
80208	SPG11	15	44562696	44663678
7782	SLC30A4	15	45479606	45522755
79848	CSPP1	8	67062426	67196263
55756	INTS9	8	28767661	28890242
4683	NBN	8	89933331	90003228
1666	DECR1	8	90001405	90053633
793	CALB1	8	90058608	90095475
6422	SFRP1	8	41261962	41309473
55284	UBE2W	8	73780097	73878910
10940	POP1	8	98117293	98159835
54332	GDAP1	8	74321130	74488872
3646	EIF3E	8	108201216	108435333
9694	EMC2	8	108443601	108489196
54108	CHRAC1	8	140511311	140517154
83988	NCALD	8	101686542	102124907
64089	SNX16	8	81799581	81842866
79792	GSDMD	8	143553207	143563062
63898	SH2D4A	8	19313693	19396218
90459	ERI1	8	9002147	9116746
23516	SLC39A14	8	22367249	22434129
23484	LEPROTL1	8	30095408	30177208
10671	DCTN6	8	30156319	30183639
2936	GSR	8	30678066	30727846
7993	UBXN8	8	30732247	30767006
5516	PPP2CB	8	30774457	30814314
4173	MCM4	8	47960185	47978160
427	ASAH1	8	18055992	18084998
4125	MAN2B1	19	12646511	12666742
3783	KCNN4	19	43766533	43781257
2997	GYS1	19	48968130	48993310
1891	ECH1	19	38815422	38831841
3191	HNRNPL	19	38836388	38852347
10382	TUBB4A	19	6494319	6502848
1209	CLPTM1	19	44954585	44993341
11129	CLASRP	19	45039045	45070956
23370	ARHGEF18	19	7395113	7472484
10848	PPP1R13L	19	45379638	45406349
92960	PEX11G	19	7476875	7497449
2068	ERCC2	19	45349837	45370918
8175	SF3A2	19	2236824	2248655

8677	STX10	19	13144058	13150383
56729	RETN	19	7669049	7670455
2208	FCER2	19	7688758	7702146
79735	TBC1D17	19	49877425	49888750
166	TLE5	19	3052910	3063107
6618	SNAPC2	19	7920338	7923250
9466	IL27RA	19	14031762	14053218
55723	ASF1B	19	14119512	14136613
26017	FAM32A	19	16185380	16192046
9441	MED26	19	16574907	16629062
93145	OLFM2	19	9853718	9936552
79744	ZNF419	19	57487711	57496097
10775	POP4	19	29606283	29617237
6217	RPS16	19	39433137	39435949
2091	FBL	19	39834458	39846379
9149	DYRK1B	19	39825350	39834201
1178	CLC	19	39731255	39738029
2821	GPI	19	34359480	34402413
23646	PLD3	19	40348456	40380439
1155	TBCB	19	36114289	36125947
5438	POLR2I	19	36113709	36115213
6510	SLC1A5	19	46774883	46788594
25865	PRKD2	19	46674275	46717127
27134	TJP3	19	3708362	3750813
11100	HNRNPUL1	19	41262496	41307787
22898	DENND3	8	141117278	141195808
1089	CEACAM4	19	41618971	41627074
27181	SIGLEC8	19	51450847	51458456
973	CD79A	19	41877279	41881372
7087	ICAM5	19	10289952	10296778
7297	TYK2	19	10350529	10380572
11140	CDC37	19	10391133	10420121
8775	NAPA	19	47487637	47515091
5802	PTPRS	19	5158495	5340803
10945	KDELR1	19	48382575	48391551
83743	GRWD1	19	48445841	48457022
22900	CARD8	19	48180770	48255946
3978	LIG1	19	48115445	48170603
8778	SIGLEC5	19	51611927	51645545
3036	HAS1	19	51713112	51723991
1628	DBP	19	48630030	48637379
587	BCAT2	19	48795062	48811029
5518	PPP2R1A	19	52190048	52229518

2639	GCDH	19	12891160	12914207
10990	LILRB5	19	54249421	54257301
1777	DNASE2	19	12875209	12881466
79165	LENG1	19	54155161	54159721
26121	PRPF31	19	54115410	54131719
3718	JAK3	19	17824780	17848071
27106	ARRDC2	19	18001132	18014102
5864	RAB3A	19	18196784	18204042
51477	ISYNA1	19	18434388	18438167
23373	CRTC1	19	18683678	18782333
51599	LSR	19	35248330	35267964
3249	HPN	19	35040506	35066571
6324	SCN1B	19	35030470	35040449
80714	PBX4	19	19561707	19618693
2931	GSK3A	19	42230190	42242625
64763	ZNF574	19	42068477	42081552
23094	SIPA1L3	19	37907208	38208369
199731	CADM4	19	43622368	43639850
56006	SMG9	19	43727983	43754962
23080	AVL9	7	32495426	32588726
10135	NAMPT	7	106248298	106286326
5445	PON2	7	95404862	95435329
3696	ITGB8	7	20330702	20415754
26959	HBP1	7	107168961	107202522
79872	CBLL1	7	107743949	107761667
51678	MPP6	7	24573268	24694193
56829	ZC3HAV1	7	139043515	139109720
79989	TTC26	7	139133744	139191986
4967	OGDH	7	44606572	44709066
11033	ADAP1	7	897900	955407
22797	TFEC	7	115935148	116159896
94239	H2AZ2	7	44826791	44848087
857	CAV1	7	116525001	116561184
10049	DNAJB6	7	157335381	157417439
3955	LFNG	7	2512529	2529177
11112	HIBADH	7	27525442	27662883
8887	TAX1BP1	7	27739331	27844564
2887	GRB10	7	50590063	50793462
10392	NOD1	7	30424527	30478784
835	CASP2	7	143288215	143307696
51142	CHCHD2	7	56101573	56106476
3315	HSPB1	7	76302673	76304295
8896	BUD31	7	99408641	99419616

7586	ZKSCAN1	7	100015572	100041689
8662	EIF3B	7	2354086	2380745
4521	NUDT1	7	2242222	2251146
6878	TAF6	7	100107070	100119841
10842	PPP1R17	7	31687215	31708455
3268	AGFG2	7	100539203	100568220
23658	LSM5	7	32485338	32495283
1174	AP1S1	7	101154456	101161596
56913	C1GALT1	7	7156934	7248616
8985	PLOD3	7	101205977	101218420
6119	RPA3	7	7636518	7718607
10467	ZNHIT1	7	101218165	101224190
24146	CLDN15	7	101232092	101238820
113263	GLCCI1	7	7968796	8094272
4899	NRF1	7	129611720	129757082
2146	EZH2	7	148807383	148884321
95681	CEP41	7	130393771	130442433
643641	ZNF862	7	149838375	149867479
4232	MEST	7	130486171	130506465
27075	TSPAN13	7	16753755	16784536
26157	GIMAP2	7	150685697	150693641
5683	PSMA2	7	42916861	42932185
55744	COA1	7	43608456	43729717
644	BLVRA	7	43758680	43807342
55665	URGCP	7	43875894	43926411
55069	TMEM248	7	66921225	66958551
6009	RHEB	7	151466012	151520120
51422	PRKAG2	7	151556124	151877125
165	AEBP1	7	44104345	44114562
5425	POLD2	7	44114681	44124358
10652	YKT6	7	44200968	44214294
26608	TBL2	7	73567537	73578791
7461	CLIP2	7	74289407	74405935
10927	SPIN1	9	88388430	88478694
54981	NMRK1	9	75060573	75088217
23731	TMEM245	9	109015135	109119947
158471	PRUNE2	9	76611376	76906114
1955	MEGF9	9	120600811	120714470
9830	TRIM14	9	98069275	98119222
7464	CORO2A	9	98120975	98192637
7046	TGFBR1	9	99104038	99154192
10952	SEC61B	9	99222064	99230615
727	C5	9	120952335	121050275

944	TNFSF8	9	114893343	114930595
2022	ENG	9	127815012	127854756
55848	PLGRKT	9	5357971	5437925
54662	TBC1D13	9	128787253	128810430
23081	KDM4C	9	6720863	7175648
81704	DOCK8	9	214854	465259
7016	TESK1	9	35605262	35610041
8939	FUBP3	9	130578965	130638352
23586	DDX58	9	32455302	32526208
8721	EDF1	9	136862119	136866308
56654	NPDC1	9	137039463	137046179
23064	SETX	9	132261356	132354986
20	ABCA2	9	137007234	137028922
51104	ABHD17B	9	71862452	71910931
51010	EXOSC3	9	37766978	37801437
9124	PDLIM1	10	95237572	95291012
2625	GATA3	10	8045378	8075198
23268	DNMBP	10	99875577	100009947
10613	ERLIN1	10	100150094	100186033
8661	EIF3A	10	119033670	119080823
9033	PKD2L1	10	100288149	100330264
1787	TRDMT1	10	17137336	17202054
79009	DDX50	10	68901286	68946847
5599	MAPK8	10	48306639	48439360
11196	SEC23IP	10	119892730	119944657
11101	ATE1	10	121740421	121928801
59338	PLEKHA1	10	122374696	122442602
64072	CDH23	10	71396920	71815947
64115	VSIR	10	71747556	71773520
9806	SPOCK2	10	72059034	72089032
5532	PPP3CB	10	73436428	73496024
9562	MINPP1	10	87504875	87553461
59	ACTA2	10	88935074	88991339
84445	LZTS2	10	100996618	101007836
6468	FBXW4	10	101610664	101695295
22849	CPEB3	10	92046692	92291078
51684	SUFU	10	102503972	102633535
91452	ACBD5	10	27195214	27242130
64077	LHPP	10	124461823	124617888
56647	BCCIP	10	125823546	125853695
55149	MTPAP	10	30309801	30374448
9148	NEURL1	10	103493979	103592552
9644	SH3PXD2A	10	103594027	103855543

	10531	PITRM1	10	3137728	3172841
	1326	MAP3K8	10	30434021	30461833
	10539	GLRX3	10	130136391	130184521
	8036	SHOC2	10	110919593	111013666
	219771	CCNY	10	35247025	35572669
	27338	UBE2S	19	55399745	55407788
	6158	RPL28	19	55385932	55403250
	64081	PBLD	10	68282660	68333049
	57532	NUFIP2	17	29255839	29294148
	28964	GIT1	17	29573469	29594054
	10900	RUNDC3A	17	44308413	44318670
	7343	UBTF	17	44205033	44221626
	5709	PSMD3	17	39980807	39997959
	51195	RAPGEFL1	17	40177010	40195656
	5414	SEPTIN4	17	58520250	58544368
	5023	P2RX1	17	3896592	3916476
	3837	KPNB1	17	47649476	47685505
	80279	CDK5RAP3	17	47967810	47981781
	2584	GALK1	17	75751594	75765236
	9969	MED13	17	61942605	62065278
	5216	PFN1	17	4945652	4949061
	10743	RAI1	17	17681458	17811453
	642	BLMH	17	30248203	30292056
	51003	MED31	17	6643311	6651634
	1819	DRG2	17	18087892	18107970
	27077	B9D1	17	19334308	19378193
	55813	UTP6	17	31860904	31901708
	3959	LGALS3BP	17	78971238	78979947
	6347	CCL2	17	34255218	34257203
	6355	CCL8	17	34319435	34321402
	5193	PEX12	17	35574795	35578863
	79132	DHX58	17	42101404	42112714
NA		HSD17B1P1	17	42546764	42548706
	3292	HSD17B1	17	42549214	42555213
	6945	MLX	17	42567068	42573239
	2145	EZH1	17	42700275	42745049
	84687	PPP1R9B	17	50133737	50150677
	51264	MRPL27	17	50367857	50373207
	10493	VAT1	17	43014607	43025123
	55379	LRRC59	17	50375059	50397523
	239	ALOX12	17	6996049	7010754
	4355	MPP2	17	43875357	43909711
	1845	DUSP3	17	43766125	43778977

9343	EFTUD2	17	44849948	44899445
9120	SLC16A6	17	68267026	68291267
5573	PRKAR1A	17	68511780	68551319
54757	FAM20A	17	68535113	68601367
29098	RANGRF	17	8288654	8290092
5608	MAP2K6	17	69414697	69553865
25979	DHRS7B	17	21123364	21193265
26118	WSB1	17	27294076	27315926
9368	SLC9A3R1	17	74748628	74769353
54868	TMEM104	17	74776499	74839753
90410	IFT20	17	28328325	28335489
5376	PMP22	17	15229777	15265326
230	ALDOC	17	28573115	28576948
6830	SUPT6H	17	28662198	28702679
55161	TMEM33	4	41935129	41960803
57606	SLAIN2	4	48341529	48426201
54940	OCIAD1	4	48805212	48861817
26511	CHIC2	4	54009789	54064605
8649	LAMTOR3	4	99878336	99894428
374	AREG	4	74445136	74455005
7323	UBE2D3	4	102794383	102868896
1998	ELF2	4	139028112	139177218
23158	TBC1D9	4	140620782	140756385
27309	ZNF330	4	141220887	141234697
11275	KLHL2	4	165207561	165323156
6164	RPL34	4	108620566	108630412
7466	WFS1	4	6269849	6303265
80273	GRPEL1	4	7058895	7068064
54433	GAR1	4	109815510	109824740
2483	FRG1	4	189940870	189963202
51809	GALNT7	4	173168811	173323967
23321	TRIM2	4	153152342	153339317
57620	STIM2	4	26857601	27025381
683	BST1	4	15703065	15738313
64151	NCAPG	4	17810979	17844865
10553	HTATIP2	11	20363685	20383783
7704	ZBTB16	11	114059711	114256765
79864	JHY	11	122882683	122963862
27087	B3GAT1	11	134378504	134412242
29907	SNX15	11	65027439	65040572
23130	ATG2A	11	64894546	64917211
5007	OSBP	11	59574398	59615774
81622	UNC93B1	11	67991100	68004982

83480	PUS3	11	125893485	125903221
55572	FOXRED1	11	126269055	126278131
64231	MS4A6A	11	60172014	60184666
51338	MS4A4A	11	60185702	60317944
1374	CPT1A	11	68754620	68844410
79080	CCDC86	11	60842113	60851081
27339	PRPF19	11	60890547	60906585
79073	TMEM109	11	60914158	60923443
10612	TRIM3	11	6448613	6474459
2352	FOLR3	11	72114869	72139892
24145	PANX1	11	94128841	94181968
22897	CEP164	11	117314557	117413266
50862	RNF141	11	10511673	10541230
57562	CEP126	11	101915010	102001062
329	BIRC2	11	102347211	102378670
1656	DDX6	11	118747763	118791164
5818	NECTIN1	11	119623408	119729200
6506	SLC1A2	11	35251205	35420063
921	CD5	11	61102489	61127852
4192	MDK	11	46380756	46383837
8567	MADD	11	47269161	47330031
833	CARS1	11	3000922	3057613
975	CD81	11	2376177	2397397
29125	C11orf21	11	2295628	2303049
10312	TCIRG1	11	68039025	68050895
5715	PSMD9	12	121888732	121918297
10536	P3H3	12	6828407	6839847
8496	PPFIBP1	12	27523431	27695564
969	CD69	12	9752486	9760901
11108	PRDM4	12	107732871	107761272
9976	CLEC2B	12	9852984	9870136
84274	COQ5	12	120503279	120534434
83892	KCTD10	12	109448655	109477359
81566	CSRNP2	12	51061205	51083664
10645	CAMKK2	12	121237675	121298308
51411	BIN2	12	51281038	51324668
10728	PTGES3	12	56663341	56688408
8825	LIN7A	12	80792520	80937925
79611	ACSS3	12	80936414	81261210
2735	GLI1	12	57459785	57472268
10988	METAP2	12	95473520	95515839
2004	ELK3	12	96194375	96269824
6539	SLC6A12	12	190077	214570

81029	WNT5B	12	1529891	1647212
55110	MAGOHB	12	10604193	10613609
55846	ITFG2	12	2812622	2859791
2305	FOXM1	12	2857680	2877174
57097	PARP11	12	3791047	3873448
10094	ARPC3	12	110434823	110450422
51699	VPS29	12	110491083	110502111
10635	RAD51AP1	12	4538798	4560048
10019	SH2B3	12	111405923	111451623
1389	CREBL2	12	12611827	12645108
80724	ACAD10	12	111686053	111757107
55507	GPRC5D	12	12940775	12952147
4055	LTBR	12	6375045	6391571
79676	OGFOD2	12	122974580	122980043
8099	CDK2AP1	12	123250112	123272334
4940	OAS3	12	112938444	112973251
4939	OAS2	12	112978395	113011723
420	ART4	12	14825569	14843526
1967	EIF2B1	12	123620406	123633766
79794	C12orf49	12	116710171	116738070
5985	RFC5	12	118013588	118033130
283383	ADGRD1	12	130953907	131141469
3458	IFNG	12	68154768	68159740
4848	CNOT2	12	70242994	70355257
11052	CPSF6	12	69239569	69274358
51258	MRPL51	12	6491886	6493841
4839	NOP2	12	6556863	6568691
1108	CHD4	12	6570082	6614524
23074	UHRF1BP1L	12	100028455	100142874
50813	COPS7A	12	6724014	6731875
51147	ING4	12	6650301	6663142
83461	CDCA3	12	6844793	6852066
8078	USP5	12	6852128	6866632
2026	ENO2	12	6913745	6923698
1822	ATN1	12	6924463	6942321
50856	CLEC4A	12	8123632	8138607
1337	COX6A1	12	120438090	120440737
55188	RIC8B	12	106774621	106889316
26127	FGFR1OP2	12	26938470	26966650
10384	BTN3A3	6	26440472	26453415
51567	TDP2	6	24649979	24666930
51389	RWDD1	6	116571409	116597675
4117	MAK	6	10762723	10838555

55003	PAK1IP1	6	10694972	10709782
57150	SMIM8	6	87322583	87399749
4739	NEDD9	6	11183298	11382348
84830	ADTRP	6	11712054	11807046
79632	FAM184A	6	118959763	119149387
2570	GABRR2	6	89254464	89315299
57515	SERINC1	6	122443351	122471807
51020	HDDC2	6	125219962	125302078
135114	HINT3	6	125956770	125980244
135112	NCOA7	6	125781161	125932034
9750	RIPOR2	6	24804282	25042170
23328	SASH1	6	148272304	148552048
10090	UST	6	148747030	149076990
54516	MTRF1L	6	152987362	153002709
5467	PPARD	6	35342558	35428191
2178	FANCE	6	35452338	35467102
116369	SLC26A8	6	35943516	36024868
1432	MAPK14	6	36027677	36111236
6005	RHAG	6	49605175	49636839
6428	SRSF3	6	36594353	36605600
29074	MRPL18	6	159789812	159798436
4172	MCM3	6	52264014	52284881
221692	PHACTR1	6	12716805	13290484
22858	CILK1	6	53001279	53061802
79865	TREML2	6	41189749	41201149
7915	ALDH5A1	6	24494867	24537207
202	CRYBG1	6	106360717	106572017
8876	VNN1	6	132680849	132714055
8875	VNN2	6	132743870	132763459
55856	ACOT13	6	24667035	24705065
81688	C6orf62	6	24704861	24719998
51053	GMNN	6	24774931	24786099
10767	HBS1L	6	134960378	135103056
10475	TRIM38	6	25962802	25991231
9841	ZBTB24	6	109462594	109483219
117247	SLC16A10	6	111087503	111231194
51696	HECA	6	139135080	139180802
9749	PHACTR2	6	143536845	143831185
7957	EPM2A	6	145382535	145736023
51596	CUTA	6	33416442	33418317
896	CCND3	6	41934934	42050357
84498	FAM120B	6	170290703	170407065
6908	TBP	6	170554302	170572870

5528	PPP2R5D	6	42984553	43012342
5754	PTK7	6	43076307	43161719
23113	CUL9	6	43182184	43224587
56940	DUSP22	6	291630	351355
55770	EXOC2	6	485154	693139
1347	COX7A2	6	75237675	75250323
2762	GMDS	6	1623806	2245605
7422	VEGFA	6	43770184	43786487
7272	TTK	6	80003887	80042527
2030	SLC29A1	6	44219553	44234142
11120	BTN2A1	6	26457904	26476621
55603	TENT5A	6	81491439	81752774
53405	CLIC5	6	45880827	46080348
57666	FBRSL1	12	132489551	132585188
55914	ERBIN	5	65926475	66082549
23438	HARS2	5	140691430	140699305
55722	CEP72	5	612340	667168
11044	TENT4A	5	6713432	6757044
3157	HMGCS1	5	43287470	43313512
10112	KIF20A	5	138178719	138187723
23530	NNT	5	43602692	43707405
3313	HSPA9	5	138553756	138575416
23107	MRPS27	5	72219409	72320646
5201	PFDN1	5	140245035	140303113
10307	APBB3	5	140558268	140564781
8622	PDE8B	5	77210449	77428256
57396	CLK4	5	178602664	178630615
3702	ITK	5	157142933	157255185
411	ARSB	5	78777209	78986087
9685	CLINT1	5	157785743	157859175
7060	THBS4	5	79991311	80083287
79908	BTNL8	5	180899077	180950906
7265	TTC1	5	160009113	160065543
5924	RASGRF2	5	80960363	81230162
900	CCNG1	5	163437569	163446151
29102	DROSHA	5	31400497	31532196
4001	LMNB1	5	126776623	126837020
57561	ARRDC3	5	91368631	91383317
64083	GOLPH3	5	32124716	32174319
10923	SUB1	5	32531633	32604079
5810	RAD1	5	34905260	34918989
55299	BRIX1	5	34915677	34925996
5618	PRLR	5	35048756	35230487

10723	SLC12A7		5	1050384	1112063
7903	ST8SIA4		5	100806933	100903282
6500	SKP1		5	134148935	134176964
80006	TRAPPC13		5	65625004	65666233
23548	TTC33		5	40512333	40755963
5917	RARS1		5	168486451	168519301
9555	MACROH2A1		5	135333900	135399914
10915	TCERG1		5	146447311	146511961
4090	SMAD5		5	136132845	136188747
1452	CSNK1A1		5	149492982	149551471
5159	PDGFRB		5	150113839	150155872
8992	ATP6V0E1		5	172983771	173035445
662	BNIP1		5	173144442	173164387
80315	CPEB4		5	173888349	173961980
3274	HRH2		5	175658030	175710756
1627	DBN1		5	177456608	177474401
23567	ZNF346		5	177022696	177081189
90249	UNC5A		5	176810519	176880898
10051	SMC4		3	160399274	160434954
58515	SELENOK		3	53884417	53891962
51300	TIMMDC1		3	119498547	119525090
51185	CRBN		3	3144628	3179727
604	BCL6		3	187721377	187745725
7337	UBE3A		15	25333728	25439051
9616	RNF7		3	141738249	141747560
54464	XRN1		3	142306607	142448062
8850	KAT2B		3	20040446	20154404
11235	PDCD10		3	167683298	167734939
5210	PFKFB4		3	48517684	48562015
1294	COL7A1		3	48564073	48595267
3280	HES1		3	194136148	194138732
7375	USP4		3	49277144	49340712
1894	ECT2		3	172750682	172821474
8287	USP9Y	Y		12537650	12860839
3373	HYAL1		3	50299890	50312381
6152	RPL24		3	101681091	101686718
57415	C3orf14		3	62318973	62336213
8087	FXR1		3	180868141	180982753
868	CBLB		3	105655461	105869552
59345	GNB4		3	179396088	179451476
84223	IQCG		3	197889077	197960142
8723	SNX4		3	125446650	125520202
54946	SLC41A3		3	126006355	126101561

5361	PLXNA1	3	126988594	127037392
50512	PODXL2	3	127629185	127672802
22937	SCAP	3	47413681	47477126
57501	KIAA1257	3	128909866	129002690
11222	MRPL3	3	131462212	131502983
1154	CISH	3	50606490	50611831
93	ACVR2B	3	38453890	38493142
57599	WDR48	3	39052013	39096671
51122	COMMD2	3	149738472	149752495
64689	GORASP1	3	39096659	39108363
84836	ABHD14B	3	51968510	51983409
7433	VIPR1	3	42489299	42537573
25981	DNAH1	3	52316319	52400491
6747	SSR3	3	156540140	156555184
27086	FOXP1	3	70952817	71583989
1981	EIF4G1	3	184314495	184335358
1933	EEF1B2	2	206159585	206162928
8745	ADAM23	2	206443532	206621127
55233	MOB1A	2	74152528	74178898
55683	KANSL3	2	96593170	96642787
81562	LMAN2L	2	96705929	96740064
6242	RTKN	2	74425835	74442422
4691	NCL	2	231453531	231483641
10120	ACTR1B	2	97655939	97664044
57669	EPB41L5	2	120013077	120179119
51639	SF3B6	2	24067586	24076373
9540	TP53I3	2	24077433	24085861
10254	STAM2	2	152116801	152175763
9381	OTOF	2	26457203	26558756
2820	GPD2	2	156435290	156613735
1058	CENPA	2	26764289	26801067
9595	CYTIP	2	157414619	157488961
90	ACVR1	2	157736444	157876330
2976	GTF3C2	2	27325849	27357034
8890	EIF2B4	2	27364352	27370457
10213	PSMD14	2	161308425	161411717
5496	PPM1G	2	27381195	27409591
92840	REEP6	19	1491166	1497927
64135	IFIH1	2	162267074	162318684
6209	RPS15	19	1438358	1440494
25801	GCA	2	162318840	162371595
7841	MOGS	2	74461057	74465410
64427	TTC31	2	74483073	74494559

374291	NDUFS7	19	1383527	1395589
6711	SPTBN1	2	54456317	54671446
27429	HTRA2	2	74529377	74533348
2591	GALNT3	2	165747588	165794659
56655	POLE4	2	74958643	74970128
55120	FANCL	2	58159243	58241372
2335	FN1	2	215360440	215436073
6772	STAT1	2	190908460	191020960
55825	PECR	2	215996329	216082955
25972	UNC50	2	98608579	98618515
3485	IGFBP2	2	216632828	216664436
84173	ELMOD3	2	85354394	85391752
10575	CCT4	2	61868085	61888671
2677	GGCX	2	85544720	85561547
23301	EHBP1	2	62673851	63046487
10190	TXNDC9	2	99318982	99340702
80219	COQ10B	2	197453423	197475308
9486	CHST10	2	100391860	100417668
25843	MOB4	2	197515571	197553699
3336	HSPE1	2	197500140	197503449
51652	CHMP3	2	86503430	86563479
7850	IL1R2	2	101991960	102028544
3554	IL1R1	2	102064544	102179874
8809	IL18R1	2	102311529	102398775
8807	IL18RAP	2	102418689	102452565
80146	UXS1	2	106093303	106194339
10058	ABCB6	2	219209772	219218994
8576	STK16	2	219245455	219250337
23178	PASK	2	241106099	241150264
10494	STK25	2	241492670	241509730
4953	ODC1	2	10439968	10448327
57448	BIRC6	2	32357028	32618899
26003	GORASP2	2	170928464	170967130
10153	CEBPZ	2	37201612	37231596
23683	PRKD3	2	37250502	37324808
25797	QPCT	2	37342827	37373322
6382	SDC1	2	20200797	20225433
6654	SOS1	2	38981549	39124345
8942	KYNU	2	142877657	143055833
9167	COX7A2L	2	42333546	42425088
5000	ORC4	2	147930396	148021604
5341	PLEK	2	68365282	68397453
54980	C2orf42	2	70149880	70248615

51449	PCYOX1	2	70257386	70281185
84634	KISS1R	19	917287	921005
7341	SUMO1	2	202206182	202238599
116444	GRIN3B	19	1000419	1009732
2956	MSH6	2	47695530	47810063
607	BCL9	1	147541501	147626216
1718	DHCR24	1	54849627	54887195
23341	DNAJC16	1	15526813	15592379
79906	MORN1	1	2321253	2391707
9731	CEP104	1	3812086	3857214
127428	TCEANC2	1	54053584	54112519
9528	TMEM59	1	54026681	54053504
6146	RPL22	1	6185020	6209389
6814	STXBP3	1	108746674	108809523
148479	PHF13	1	6613731	6624030
271	AMPD2	1	109616104	109632053
6429	SRSF4	1	29147743	29181900
80267	EDEM3	1	184690237	184754907
79084	WDR77	1	111439890	111449256
515	ATP5PB	1	111448864	111462773
3065	HDAC1	1	32292083	32333635
829	CAPZA1	1	112619805	112671616
127544	RNF19B	1	32936445	32964685
10067	SCAMP3	1	155255979	155262430
55223	TRIM62	1	33145399	33182059
58512	DLGAP3	1	34865436	34929650
6421	SFPQ	1	35176378	35193145
54856	GON4L	1	155749662	155859400
85440	DOCK7	1	62454298	62688386
6723	SRM	1	11054584	11060020
26232	FBXO2	1	11637018	11655785
26270	FBXO6	1	11664200	11674354
54823	SWT1	1	185157080	185291781
10459	MAD2L2	1	11674480	11691650
60672	MIIP	1	12019466	12032045
9887	SMG7	1	183472216	183598246
4688	NCF2	1	183555562	183590876
23169	SLC35D1	1	66999350	67054148
5321	PLA2G4A	1	186828949	186988981
79971	WLS	1	68098473	68233120
5997	RGS2	1	192809039	192812275
6738	RO60	1	193059422	193091777
10286	BCAS2	1	114567557	114581629

23207	PLEKHM2	1	15684320	15734769
10745	PHTF1	1	113696831	113759489
7709	ZBTB17	1	15941869	15976132
965	CD58	1	116514534	116571039
252839	TMEM9	1	201134772	201171574
55700	MAP7D1	1	36155579	36180849
10352	WARS2	1	119031216	119140671
64960	MRPS15	1	36455718	36464384
8443	GNPAT	1	231241207	231277973
7257	TSNAX	1	231528541	231566524
54955	C1orf109	1	37681570	37692249
3964	LGALS8	1	236518000	236552981
4610	MYCL	1	39895426	39902256
57568	SIPA1L2	1	232397965	232561558
8564	KMO	1	241532134	241595642
127396	ZNF684	1	40531573	40548167
2117	ETV3	1	157121191	157138474
34	ACADM	1	75724347	75787575
6504	SLAMF1	1	160608106	160647295
962	CD48	1	160678746	160711831
6390	SDHB	1	17018722	17054032
80135	RPF1	1	84479259	84498352
1486	CTBS	1	84549611	84574480
59349	KLHL12	1	202891116	202928636
54680	ZNHIT6	1	85649417	85708433
5929	RBBP5	1	205086142	205122015
2635	GBP3	1	89006679	89022894
2633	GBP1	1	89052319	89065230
8934	RAB29	1	205767986	205775482
1889	ECE1	1	21217247	21345572
3399	ID3	1	23557926	23559501
51107	APH1A	1	150265399	150269580
64175	P3H1	1	42746335	42767084
991	CDC20	1	43358981	43363203
533	ATP6V0B	1	43974487	43978295
8704	B4GALT2	1	43978943	43991170
79033	ERI3	1	44221070	44355260
5052	PRDX1	1	45511036	45523047
8548	BLZF1	1	169367970	169396540
2166	FAAH	1	46394317	46413848
1266	CNN3	1	94896949	94927223
5825	ABCD3	1	94418389	94518666
51611	DPH5	1	100989623	101026088

58155	PTBP2	1	96721665	96823738
9588	PRDX6	1	173477330	173488815
55157	DARS2	1	173824653	173858808
3664	IRF6	1	209785617	209806175
11123	RCAN3	1	24502351	24541040
25949	SYF2	1	25222276	25232502
57035	RSRP1	1	25242249	25338213
56181	MTFR1L	1	25818640	25832942
57134	MAN1C1	1	25617468	25786207
4751	NEK2	1	211658657	211675630
79947	DHDDS	1	26432282	26471306
25936	NSL1	1	212726153	212791782
1063	CENPF	1	214603195	214664571
200010	SLC5A9	1	48222685	48248644
114883	OSBPL9	1	51577179	51798427
51060	TXNDC12	1	52020131	52055191
57488	ESYT2	7	158730995	158830253
10274	STAG1	3	136336236	136752403
4317	MMP8	11	102711796	102727050
11133	KPTN	19	47475150	47484265
6230	RPS25	11	119015712	119018691
9928	KIF14	1	200551497	200620751
83479	DDX59	1	200623896	200669907
23271	CAMSAP2	1	200739558	200860704
9331	B4GALT6	18	31622247	31685836
55259	CASC1	12	25108289	25195162
4033	LRMP	12	25004342	25108334
57558	USP35	11	78188812	78215232
6785	ELOVL4	6	79914814	79947553
90025	UBE3D	6	82892390	83065841
84251	SGIP1	1	66533383	66751139
23469	PHF3	6	63635802	63779336
79747	ADGB	6	146598967	146815462
7128	TNFAIP3	6	137867214	137883312
6446	SGK1	6	134169246	134318112
81847	RNF146	6	127266610	127288567
383	ARG1	6	131470832	131584332
26234	FBXL5	4	15604381	15681679
80306	MED28	4	17614641	17634105
8673	VAMP8	2	85561562	85582031
64858	DCLRE1B	1	113905213	113914086
103910	MYL12B	18	3261479	3278431
6185	RPN2	20	37178410	37241619

5311	PKD2	4	88007635	88077777
8987	STBD1	4	76306026	76311599
10983	CCNI	4	77047155	77075989
64747	MFSD1	3	158732198	158829719
5493	PPL	16	4882507	4960741
29855	UBN1	16	4846665	4882401
894	CCND2	12	4273762	4305353
22936	ELL2	5	95885098	95961851
4709	NDUFB3	2	201071433	201085750
23314	SATB2	2	199269500	199471266
140803	TRPM6	9	74722495	74888094
80235	PIGZ	3	196946356	196969060
205564	SENP5	3	196867856	196934714
80212	CCDC92	12	123918660	123972831
84886	C1orf198	1	230837119	230869589
23307	FKBP15	9	113161006	113221318
6418	SET	9	128683424	128696400
51552	RAB14	9	121178133	121223014
11064	CNTRL	9	121074863	121177610
22954	TRIM32	9	116687302	116701300
10783	NEK6	9	124257606	124353307
4702	NDUFA8	9	122144058	122159779
81932	HDHD3	9	113373419	113376999
92400	RBM18	9	122237622	122264840
57864	SLC46A2	9	112878920	112890876
79695	GALNT12	9	98807670	98850081
85365	ALG2	9	99216425	99221942
1441	CSF3R	1	36466043	36483278
2531	KDSR	18	63327726	63367228
84878	ZBTB45	19	58513530	58538911
56252	YLPM1	14	74763316	74859435
5228	PGF	14	74941834	74955626
83982	IFI27L2	14	94127779	94130253
91754	NEK9	14	75079353	75127344
112752	IFT43	14	75902136	76084585
55640	FLVCR2	14	75578620	75663214
1743	DLST	14	74881891	74903743
7043	TGFB3	14	75958099	75982991
81892	SLIRP	14	77708071	77761104
4329	ALDH6A1	14	74056847	74084492
114818	KLHL29	2	23385179	23708611
54867	TMEM214	2	27032910	27041694
54454	ATAD2B	2	23748664	23927123

64225	ATL2	2	38293954	38377285
51646	YPEL5	2	30146941	30160533
84272	YIPF4	2	32277904	32316594
29094	LGALSL	2	64453969	64461381
53335	BCL11A	2	60450520	60554467
9419	CRIPT	2	46616416	46630176
4072	EPCAM	2	47345158	47387601
83401	ELOVL3	10	102226299	102229589
3437	IFIT3	10	89327997	89340971
3433	IFIT2	10	89283694	89309271
51076	CUTC	10	99702558	99756134
84795	PYROXD2	10	98383565	98415182
3070	HELLS	10	94501434	94613905
26123	TCTN3	10	95663396	95694143
404636	DENND10	10	119104086	119137984
55717	WDR11	10	120851305	120909524
159686	CFAP58	10	104353833	104455102
2805	GOT1	10	99396870	99430624
1843	DUSP1	5	172768096	172771195
7010	TEK	9	27109141	27230174
79886	CAAP1	9	26840685	26892803
29126	CD274	9	5450503	5470566
348995	NUP43	6	149724315	149749665
25902	MTHFD1L	6	150865549	151101887
5110	PCMT1	6	149749443	149811420
80177	MYCT1	6	152697895	152724567
80231	CXorf21	X	30558809	30577766
84418	CYSTM1	5	140175156	140282052
54853	WDR55	5	140664676	140674124
63931	MRPS14	1	175010789	175023425
6950	TCP1	6	159778498	159789703
399979	SNX19	11	130875436	130916509
79684	MSANTD2	11	124766498	124800706
51248	PDZD11	X	70286595	70290514
84955	NUDCD1	8	109240919	109334118
56943	ENY2	8	109334324	109345954
84930	MASTL	10	27154824	27187953
84898	PLXDC2	10	19816239	20289856
6883	TAF12	1	28589323	28643085
9617	MTRF1	13	41216369	41263577
29103	DNAJC15	13	43023203	43114213
80209	PROSER1	13	39009865	39038089
11193	WBP4	13	41061509	41084006

1997	ELF1	13	40931924	41061440
4093	SMAD9	13	36844831	36920765
10808	HSPH1	13	31134973	31162388
29880	ALG5	13	36949738	37000261
7045	TGFBI	5	136028988	136063818
51247	PAIP2	5	139341587	139369720
1958	EGR1	5	138465479	138469303
80298	MTERF2	12	106977277	106987160
8835	SOCS2	12	93569814	93583487
51019	WASHC3	12	102012840	102062149
10174	SORBS3	8	22544986	22575788
2185	PTK2B	8	27311482	27459391
2053	EPHX2	8	27490781	27545564
81790	RNF170	8	42849637	42897290
29914	UBIAD1	1	11273206	11296049
23435	TARDBP	1	11012344	11025739
10987	COPS5	8	67043079	67083783
8165	AKAP1	17	57085092	57121346
8161	COIL	17	56938199	56961050
7706	TRIM25	17	56836387	56914080
81558	FAM117A	17	49710332	49789180
23397	NCAPH	2	96335766	96377091
117854	TRIM6	11	5596109	5612958
85320	ABCC11	16	48166910	48247568
113	ADCY7	16	50246137	50318135
84902	CEP89	19	32875925	32971991
79887	PLBD1	12	14503661	14567883
79912	PYROXD1	12	21437615	21471250
55269	PSPC1	13	19674752	19783019
256051	ZNF549	19	57527325	57557542
65982	ZSCAN18	19	58083838	58118427
10520	ZNF211	19	57630395	57644041
89884	LHX4	1	180230264	180278984
81627	TRMT1L	1	185118101	185157072
1475	CSTA	3	122325248	122341969
8702	B4GALT4	3	119211732	119240946
81930	KIF18A	11	28020619	28108156
1408	CRY2	11	45847118	45883248
847	CAT	11	34438934	34472060
10804	GJB6	13	20221962	20232365
64786	TBC1D15	12	71839707	71927248
51538	ZCCHC17	1	31296982	31364953
10657	KHDRBS1	1	32013868	32060850

55116	TMEM39B		1	32072031	32102866
9034	CCRL2		3	46407166	46412997
84265	POLR3GL		1	145964690	145978848
8743	TNFSF10		3	172505508	172523475
5290	PIK3CA		3	179148114	179240093
23244	PDS5A		4	39822863	39977956
11019	LIAS		4	39459004	39485109
7579	ZSCAN20		1	33472645	33496507
55791	LRIF1		1	110947190	110963965
57413	TMIGD3		1	111483348	111563962
79712	GTDC1		2	143938068	144332568
7852	CXCR4		2	136114349	136118149
84083	ZRANB3		2	135136916	135531218
92	ACVR2A		2	147844517	147930826
51426	POLK		5	75511756	75601144
2322	FLT3		13	28003274	28100592
219402	MTIF3		13	27435643	27450591
387496	RASL11A		13	27270830	27275192
130916	MTERF4		2	241072169	241102332
54440	SASH3	X		129779949	129795201
4952	OCRL	X		129539849	129592561
57179	KIAA1191		5	176346062	176361807
51744	CD244		1	160830160	160862887
4063	LY9		1	160796074	160828261
54537	SHLD2		10	87094161	87191468
84293	PRXL2A		10	80407829	80437115
7755	ZNF205		16	3112560	3120517
57489	ODF2L		1	86346824	86396342
5737	PTGFR		1	78303884	78540701
100505741	SPATA1		1	84506300	84566194
343099	CCDC18		1	93179919	93278730
80148	SLC66A2		18	79902420	79951657
27241	BBS9		7	33109557	33877180
80820	EEPD1		7	36153254	36301538
55975	KLHL7		7	23105758	23177914
11335	CBX3		7	26201162	26213607
3624	INHBA		7	41667168	41705834
11328	FKBP9		7	32957404	33006930
51251	NT5C3A		7	33014114	33062797
10124	ARL4A		7	12686856	12690958
51622	CCZ1		7	5898725	5926550
152007	GLIPR2		9	36136536	36163913
8434	RECK		9	36036913	36124455

48	ACO1		9	32384603	32454769
79269	DCAF10		9	37800554	37867666
78996	CYREN		7	135092363	135170795
5552	SRGN		10	69088103	69104805
55847	CISD1		10	58269162	58289586
11319	ECD		10	73130155	73169055
8034	SLC25A16		10	68477998	68527523
11130	ZWINT		10	56357227	56361273
9904	RBM19		12	113816738	113966325
11113	CIT		12	119685791	119877320
84329	HVCN1		12	110627841	110704950
79039	DDX54		12	113157174	113185479
23389	MED13L		12	115953872	116277719
1031	CDKN2C		1	50960745	50974634
3709	ITPR2		12	26335352	26833194
64168	NECAB1		8	90791741	90959393
11059	WWP1		8	86342547	86478420
23597	ACOT9	X		23701055	23766475
10549	PRDX4	X		23664262	23686397
10755	GIPC1		19	14477760	14496149
57213	SPRYD7		13	49912702	49936490
84650	EBPL		13	49660674	49691486
540	ATP7B		13	51930436	52012125
23091	ZC3H13		13	45954465	46052759
466	ATF1		12	50763710	50821162
3071	NCKAP1L		12	54497752	54548243
4327	MMP19		12	55835433	55842966
5204	PFDN5		12	53295291	53299450
65244	SPATS2		12	49366584	49527425
3164	NR4A1		12	52022832	52059507
5153	PDE1B		12	54549601	54579239
4778	NFE2		12	54292111	54301015
64375	IKZF4		12	56007659	56038435
23583	SMUG1		12	54121277	54189008
25895	EEF1AKMT3		12	57771492	57782541
1757	SARDH		9	133663560	133739955
1621	DBH		9	133636363	133659329
6491	STIL		1	47250139	47314147
55355	HJURP		2	233833416	233854566
79828	METTL8		2	171317405	171434802
9111	NMI		2	151270470	151289894
7130	TNFAIP6		2	151357592	151380046
29994	BAZ2B		2	159318979	159616569

206358	SLC36A1		5	151437046	151492379
9926	LPGAT1		1	211743457	211830763
50486	G0S2		1	209675412	209676390
3759	KCNJ2		17	70168673	70180044
57826	RAP2C	X		132203024	132219480
5393	EXOSC9		4	121801318	121817021
79934	COQ8B		19	40691530	40718207
5208	PFKFB2		1	207034366	207081024
722	C4BPA		1	207104233	207144972
23682	RAB38		11	88113251	88175443
1164	CKS2		9	89311195	89316703
2181	ACSL3		2	222860942	222944639
23363	OBSL1		2	219550728	219571859
79843	FAM124B		2	224378698	224402107
6560	SLC12A4		16	67943474	67969601
200232	FAM209A		20	56517187	56526152
6590	SLPI		20	45252239	45254564
79183	TTPAL		20	44475874	44494603
57580	PREX1		20	48624252	48827999
8202	NCOA3		20	47501887	47656877
51604	PIGT		20	45416084	45456934
57727	NCOA5		20	46060991	46089962
9217	VAPB		20	58389229	58451101
84612	PARD6B		20	50731580	50756795
514	ATP5F1E		20	59025475	59032345
84181	CHD6		20	41402083	41618384
84969	TOX2		20	43914852	44069616
6431	SRSF6		20	43457893	43466046
57169	ZNFX1		20	49237946	49278426
128611	ZNF831		20	59123381	59259113
6780	STAU1		20	49113339	49188367
27304	MOCS3		20	50958818	50963929
8675	STX16		20	58651253	58679526
NA	PPP4R1L		20	58228940	58309451
56937	PMEPA1		20	57648392	57711536
55661	DDX27		20	49219295	49244077
55653	BCAS4		20	50794894	50882676
81030	ZBP1		20	57603846	57620576
23096	IQSEC2	X		53225828	53321350
10617	STAMBP		2	73828916	73873659
55577	NAGK		2	71064344	71079808
400961	PAIP2B		2	71182738	71227103
11017	SNRNP27		2	69893956	69905575

10396	ATP8A1		4	42408373	42657105
23326	USP22		17	20999596	21043760
27076	LYPD3		19	43460787	43465608
1088	CEACAM8		19	42580243	42595055
7180	CRISP2		6	49692358	49713590
55809	TRERF1		6	42224931	42452051
29993	PACSIN1		6	34466061	34535229
10385	BTN2A2		6	26383096	26394874
56897	WRNIP1		6	2765393	2786952
NA	BTN2A3P		6	26421391	26432383
89845	ABCC10		6	43427366	43450427
4835	NQO2		6	2987987	3028869
221443	OARD1		6	41033627	41097787
222643	UNC5CL		6	41026895	41039221
4337	MOCS1		6	39899578	39934551
9477	MED20		6	41905354	41921139
26212	OR2B6		6	27957241	27958182
9587	MAD2L1BP		6	43629540	43640952
116138	KLHDC3		6	43014103	43021298
27232	GNMT		6	42960754	42963880
1769	DNAH8		6	38715311	39030792
54210	TREM1		6	41267926	41286682
1026	CDKN1A		6	36676460	36687337
6659	SOX4		6	21593751	21598619
6239	RREB1		6	7107597	7251980
83732	RIOK1		6	7389793	7418037
51299	NRN1		6	5997999	6007605
51000	SLC35B3		6	8411463	8435611
6310	ATXN1		6	16299112	16761491
7913	DEK		6	18223860	18264548
745	MYRF		11	61752642	61788518
7471	WNT1		12	48978322	48982620
26231	LRRC29		16	67207139	67227048
583	BBS2		16	56466836	56520087
4502	MT2A		16	56608584	56609497
2806	GOT2		16	58707131	58734342
78995	HROB		17	44141906	44162476
65109	UPF3B	X		119805311	119852998
23157	SEPTIN6	X		119615724	119693370
55026	TMEM255A	X		120258650	120311556
4694	NDUFA1	X		119871832	119876662
8603	FAM193A		4	2536631	2732565
9953	HS3ST3B1		17	14301081	14349404

51081	MRPS7		17	75261674	75266376
10485	C1orf61		1	156404250	156456763
7270	TTF1		9	132375548	132406851
9329	GTF3C4		9	132670035	132694953
64794	DDX31		9	132592997	132670401
4987	OPRL1		20	64080082	64100643
56731	SLC2A4RG		20	63739776	63744050
79144	PPDPF		20	63520765	63522206
51141	INSIG2		2	118088452	118110997
84172	POLR1B		2	112541915	112579818
79085	SLC25A23		19	6436079	6465203
8192	CLPP		19	6361531	6370242
9349	RPL23		17	38847860	38853764
970	CD70		19	6583183	6604103
9322	TRIP10		19	6737925	6751530
2354	FOSB		19	45467995	45475179
6633	SNRPD2		19	45687454	45692569
56261	GPCPD1		20	5544439	5611006
27111	SDCBP2		20	1309909	1329139
80025	PANK2		20	3888839	3929882
63908	NAPB		20	23374519	23421519
9491	PSMF1		20	1113240	1189415
10616	RBCK1		20	407498	432139
6628	SNRPB		20	2461634	2470853
80023	NRSN2		20	346782	359660
8195	MKKS		20	10401009	10434222
631	BFSP1		20	17493905	17569220
11034	DSTN		20	17570075	17609919
6629	SNRPB2		20	16729961	16742564
92667	MGME1		20	17969018	17991122
83541	FAM110A		20	833715	857463
128646	SIRPD		20	1534251	1557705
56926	NCLN		19	3185563	3209575
10236	HNRNPR		1	23303771	23344336
10893	MMP24		20	35226690	35276998
8894	EIF2S2		20	34088309	34112243
51614	ERGIC3		20	35542021	35557634
140823	ROMO1		20	35699272	35700984
101410538	MMP24OS		20	35201745	35278131
8242	KDM5C	X		53191321	53225422
154796	AMOT	X		112774503	112840815
192669	AGO3		1	35930718	36072500
7517	XRCC3		14	103697609	103715504

10426	TUBGCP3		13	112485011	112588205
23263	MCF2L		13	112894378	113099742
79414	LRFN3		19	35935358	35946624
826	CAPNS1		19	36139953	36150353
79171	RBM42		19	35629030	35637686
2867	FFAR2		19	35443907	35451767
7067	THRA		17	40058290	40093867
1236	CCR7		17	40553769	40565472
9572	NR1D1		17	40092793	40100589
58506	SCAF1		19	49642209	49658642
57479	PRR12		19	49591182	49626439
84081	NSRP1		17	30115521	30186475
22826	DNAJC8		1	28199456	28233029
27245	AHDC1		1	27534035	27604431
2537	IFI6		1	27666064	27672198
171017	ZNF384		12	6666477	6689572
10436	EMG1		12	6970913	6997428
8409	UXT	X		47651796	47659180
2002	ELK1	X		47635521	47650604
9787	DLGAP5		14	55148112	55191608
55288	RHOT1		17	32142454	32253374
2123	EVI2A		17	31317560	31321884
4974	OMG		17	31272013	31297539
55112	WDR60		7	158856558	158956747
83543	AIF1L		9	131096476	131123152
286336	FAM78A		9	131258076	131276510
8021	NUP214		9	131125586	131234663
8273	SLC10A3	X		154487306	154490690
3188	HNRNPH2	X		101408222	101414133
55906	ZC4H2	X		64915802	65034713
821	CANX		5	179678628	179731641
6003	RGS13		1	192636138	192660306
64768	IPPK		9	92613183	92670131
83744	ZNF484		9	92844182	92878038
89846	FGD3		9	92947523	93036236
79717	PPCS		1	42456117	42473385
64919	BCL11B		14	99169287	99272197
1350	COX7C		5	86617928	86620962
92797	HELB		12	66302493	66343643
5908	RAP1B		12	68610855	68671901
117177	RAB3IP		12	69738681	69823204
8445	DYRK2		12	67648338	67665406
65999	LRRC61		7	150323263	150338156

84286	TMEM175	4	932387	958656
54850	FBXL12	19	9810267	9827816
23065	EMC1	1	19215660	19251527
23352	UBR4	1	19074510	19210266
50809	HP1BP3	1	20740266	20787323
79939	SLC35E1	19	16549831	16572415
10365	KLF2	19	16324826	16328685
10975	UQCR11	19	1597169	1605473
9088	PKMYT1	16	2968024	2980479
117166	WFIKKN1	16	629239	634117
23499	MACF1	1	39081316	39487177
6597	SMARCA4	19	10960825	11079426
83460	EMC6	17	3668812	3669668
79066	METTL16	17	2405562	2511891
14	AAMP	2	218264123	218270257
1892	ECHS1	10	133362485	133373354
90485	ZNF835	19	56661980	56671783
7979	SEM1	7	96481626	96709891
3092	HIP1	7	75533298	75738962
79689	STEAP4	7	88270892	88306891
7978	MTERF1	7	91692008	91880702
64921	CASD1	7	94509219	94557019
57622	LRFN1	19	39306568	39315336
79644	SRD5A3	4	55346242	55373100
7533	YWHAH	22	31944522	31957603
468	ATF4	22	39519695	39522685
53947	A4GALT	22	42692121	42721298
11135	CDC42EP1	22	37560480	37569405
8459	TPST2	22	26521996	26596717
23780	APOL2	22	36226209	36239954
3976	LIF	22	30240453	30246759
200315	APOBEC3A	22	38952741	38992778
200316	APOBEC3F	22	39040604	39055972
51234	EMC4	15	34225013	34230156
7732	RNF112	17	19411125	19417276
92521	SPECC1	17	20009344	20319026
9732	DOCK4	7	111726110	112206407
25913	POT1	7	124822386	124929983
222256	CDHR3	7	105876796	106036432
4289	MKLN1	7	131110096	131496632
3663	IRF5	7	128937457	128950038
4698	NDUFA5	7	123536997	123557904
8520	HAT1	2	171922448	171983686

57705	WDFY4		10	48684876	48982956
29767	TMOD2		15	51751597	51816363
90416	CCDC32		15	40528683	40565057
8854	ALDH1A2		15	57953424	58497866
54629	MINDY2		15	58771192	58861900
90417	KNSTRN		15	40382721	40394246
10776	ARPP19		15	52547045	52569883
25847	ANAPC13		3	134477706	134486716
1315	COPB1		11	14443440	14500027
5682	PSMA1		11	14504874	14643635
25870	SUMF2		7	56064002	56080670
23022	PALLD		4	168497052	168928457
60559	SPCS3		4	176319966	176332245
26297	SERGEF		11	17788048	18013047
79733	E2F8		11	19224063	19241620
1635	DCTD		4	182890060	182917936
84817	TXNDC17		17	6640985	6644541
9513	FXR2		17	7591230	7614897
10749	KIF1C		17	4997950	5028401
9526	MPDU1		17	7583529	7592789
51105	PHF20L1		8	132775358	132848807
23639	LRRC6		8	132570416	132675592
65095	KRI1		19	10553085	10566031
3609	ILF3		19	10654261	10692417
10053	AP1M2		19	10572671	10587315
1032	CDKN2D		19	10566460	10569059
25983	NGDN		14	23469689	23509862
11035	RIPK3		14	24336025	24340022
196883	ADCY4		14	24318349	24335093
58533	SNX6		14	34561093	34630160
6035	RNASE1		14	20801228	20802855
1603	DAD1		14	22564907	22589224
7011	TEP1		14	20365667	20413501
64097	EPB41L4A		5	112142441	112419313
1036	CDO1		5	115804733	115816659
7905	REEP5		5	112876385	112922289
81533	ITFG1		16	47154387	47464149
79651	RHBDF2		17	76470891	76501790
15	AANAT		17	76453351	76470117
9459	ARHGEF6	X		136665547	136780932
79649	MAP7D3	X		136213220	136256482
2258	FGF13	X		138614727	139222777
1028	CDKN1C		11	2883213	2885773

83855	KLF16		19	1852399	1863579
23383	MAU2		19	19320829	19358754
25759	SHC2		19	416583	460996
6861	SYT5		19	55171196	55180289
2593	GAMT		19	1397026	1401570
8226	PUDP	X		6667865	7148158
55274	PHF10		6	169703902	169725566
84766	CRACR2A		12	3606633	3764819
9754	STARD8	X		68647666	68725842
6303	SAT1	X		23783173	23786226
23677	SH3BP4		2	234952017	235055714
158747	MOSPD2	X		14873421	14922327
57572	DOCK6		19	11199295	11262524
3949	LDLR		19	11089462	11133820
84337	ELOF1		19	11551147	11559236
26526	TSPAN16		19	11296139	11326996
5589	PRKCSH		19	11435288	11450968
51337	THEM6		8	142727223	142736927
10452	TOMM40		19	44890569	44903689
10912	GADD45G		9	89605012	89606555
9667	SAFB2		19	5586999	5624046
148229	ATP8B3		19	1782075	1812276
684	BST2		19	17402939	17405630
376497	SLC27A1		19	17468769	17506168
51429	SNX9		6	157700387	157945077
83861	RSPH3		6	158972871	159000202
81	ACTN4		19	38647649	38731589
4705	NDUFA10		2	239892450	240025345
10095	ARPC1B		7	99374249	99394816
59285	CACNG6		19	53992288	54012669
23149	FCHO1		19	17747718	17788568
55201	MAP1S		19	17719242	17734513
113730	KLHDC7B		22	50545899	50551023
9518	GDF15		19	18374731	18389176
79230	ZNF557		19	7069703	7087968
10439	OLFM1		9	135075422	135121179
157922	CAMSAP1		9	135808487	135907546
140685	ZBTB46		20	63743666	63832038
85441	HELZ2		20	63558086	63574239
4046	LSP1		11	1853084	1892267
7140	TNNT3		11	1919703	1938706
10844	TUBGCP2		10	133278630	133312337
10298	PAK4		19	39125770	39182816

64793	CEP85		1	26234200	26278808
6874	TAF4		20	61953469	62065810
83549	UCK1		9	131523801	131531264
10155	TRIM28		19	58544064	58550722
23211	ZC3H4		19	47064187	47113776
9535	GMFG		19	39328353	39342372
57470	LRRC47		1	3778559	3796498
27293	SMPDL3B		1	27935000	27959157
93974	ATP5IF1		1	28236109	28246906
57693	ZNF317		19	9140397	9163424
8666	EIF3G		19	10115014	10119918
55337	SHFL		19	10086122	10093252
1786	DNMT1		19	10133345	10231286
1736	DKC1	X		154762742	154777689
55422	ZNF331		19	53519527	53580269
7776	ZNF236		18	76822607	76970727
91574	C12orf65		12	123233436	123257960
54897	CASZ1		1	10636604	10796650
22927	HABP4		9	96450169	96491336
7317	UBA1	X		47190861	47215128
NA	TXLNGY	Y		19567313	19606274
9590	AKAP12		6	151239967	151358559
54869	EPS8L1		19	55072020	55087923
2686	GGT7		20	34844720	34872856
23229	ARHGEF9	X		63634967	63809274
529	ATP6V1E1		22	17592136	17628749
126299	ZNF428		19	43607224	43619629
23199	GSE1		16	85169525	85676204
51659	GINS2		16	85676198	85690073
5119	CHMP1A		16	89644435	89657721
1349	COX7B	X		77899440	77907376
2161	F12		5	177402140	177409576
4772	NFATC1		18	79395856	79529325
51132	RLIM	X		74582976	74614624
93323	HAUS8		19	17049729	17075625
23180	RFTN1		3	16313574	16514026
64145	RBSN		3	15070073	15099163
6533	SLC6A6		3	14402576	14489349
3748	KCNC3		19	50311937	50333515
9476	NAPSA		19	50358477	50365830
4245	MGAT1		5	180784782	180815652
10197	PSME3		17	42824385	42843760
6155	RPL27		17	42998273	43002959

29893	PSMC3IP	17	42572315	42577831
47	ACLY	17	41866917	41930542
84313	VPS25	17	42773449	42779599
54882	ANKHD1	5	140401814	140539856
80762	NDFIP1	5	142108779	142154440
7322	UBE2D2	5	139526431	139628434
60412	EXOC4	7	133253073	134066589
116983	ACAP3	1	1292390	1309609
79652	TMEM204	16	1528688	1555580
84240	ZCCHC9	5	81301587	81313297
7153	TOP2A	17	40388525	40417896
10948	STARD3	17	39637090	39664201
10401	PIAS3	1	145848522	145859836
64087	MCCC2	5	71587288	71658706
57343	ZNF304	19	57351307	57359898
79149	ZSCAN5A	19	56219670	56368383
7691	ZNF132	19	58432814	58440153
55829	SELENOS	15	101270817	101277500
22856	CHSY1	15	101175727	101252048
55145	THAP1	8	42836674	42843325
55860	ACTR10	14	58200080	58235636
145447	ABHD12B	14	50872053	50904970
2643	GCH1	14	54842008	54902826
3337	DNAJB1	19	14514769	14529770
5989	RFX1	19	13961530	14007039
54862	CC2D1A	19	13906201	13930879
6737	TRIM21	11	4384897	4393702
10489	LRRC41	1	46261196	46303608
22907	DHX30	3	47802909	47850195
5894	RAF1	3	12583601	12664226
5468	PPARG	3	12287368	12434356
51478	HSD17B7	1	162790702	162812817
85363	TRIM5	11	5663195	5938619
10346	TRIM22	11	5689697	5737089
23167	EFR3A	8	131904093	132013642
80895	ILKAP	2	238170402	238203708
8864	PER2	2	238244044	238290102
51540	SCLY	2	238060924	238099413
5901	RAN	12	130872037	130877678
84674	CARD6	5	40841308	40860175
23108	RAP1GAP2	17	2755705	3037741
10514	MYBBP1A	17	4538897	4555631
6117	RPA1	17	1829702	1900082

5176	SERPINF1	17	1762029	1777565
7326	UBE2G1	17	4269259	4366628
60678	EEFSEC	3	128153481	128408646
25957	PNISR	6	99398050	99425331
23480	SEC61G	7	54752250	54759974
55915	LANCL2	7	55365448	55433742
3512	JCHAIN	4	70655541	70681817
91107	TRIM47	17	75874164	75878581
10462	CLEC10A	17	7074537	7080307
3000	GUCY2D	17	8002615	8020342
54739	XAF1	17	6755447	6775647
10247	RIDA	8	98102344	98117171
157680	VPS13B	8	99013266	99877580
4147	MATN2	8	97868840	98036724
6388	SDF2	17	28648346	28662189
54496	PRMT7	16	68310974	68358563
7014	TERF2	16	69355567	69408571
63926	ANKEF1	20	9986126	10058303
5111	PCNA	20	5114953	5126626
29107	NXT1	20	23350791	23354771
54453	RIN2	20	19757606	20002459
9826	ARHGEF11	1	156934840	157045370
23208	SYT11	1	155859567	155885199
25974	MMACHC	1	45500300	45513382
114034	TOE1	1	45340052	45343973
55544	RBM38	20	57391396	57409333
51526	OSER1	20	44195939	44210771
5509	PPP1R3D	20	59936663	59940305
8546	AP3B1	5	78002326	78294698
84327	ZBED3	5	77072072	77087285
10207	PATJ	1	61742406	62178675
51164	DCTN4	5	150708440	150759109
9205	ZMYM5	13	19823482	19863649
10208	USPL1	13	30617693	30660770
51371	POMP	13	28659104	28678959
1024	CDK8	13	26254104	26405238
241	ALOX5AP	13	30713478	30764426
4628	MYH10	17	8474207	8630761
1118	CHIT1	1	203212827	203273641
254428	SLC41A1	1	205789094	205813748
9911	TMCC2	1	205227946	205285632
8900	CCNA1	13	36431520	36442870
23111	SPART	13	36301638	36370180

122042	RXFP2		13	31739526	31803389
94240	EPSTI1		13	42886388	42992271
5994	RFXAP		13	36819222	36829104
7178	TPT1		13	45333471	45341284
79710	MORC4	X		106813871	107000212
55859	BEX1	X		103062651	103064171
84923	FAM104A		17	73207353	73236753
2048	EPHB2		1	22710839	22921500
10250	SRRM1		1	24631716	24673281
55643	BTBD2		19	1985438	2034881
84250	SLF1		5	94618669	94739436
85329	LGALS12		11	63506052	63516774
5920	PLAAT4		11	63536808	63546462
54979	PLAAT2		11	63552770	63563379
22880	MORC2		22	30925130	30968774
9215	LARGE1		22	33162226	33922841
55303	GIMAP4		7	150567369	150573953
57180	ACTR3B		7	152759749	152855378
694	BTG1		12	92140278	92145846
80195	TMEM254		10	80078646	80092557
83857	TMTC1		12	29500840	29784759
51520	LARS1		5	146110566	146182696
3612	IMPA1		8	81656914	81686331
85444	LRRCC1		8	85107215	85146080
29080	CCDC59		12	82223681	82358805
23075	SWAP70		11	9664077	9752993
10894	LYVE1		11	10556966	10611689
81846	SBF2		11	9778667	10294207
3295	HSD17B4		5	119452473	119637199
196441	ZFC3H1		12	71609599	71667725
51669	SARAF		8	30063003	30083208
4221	MEN1		11	64803510	64811294
11161	ERG28		14	75649791	75660876
80017	DGLUCY		14	91060333	91225632
23508	TTC9		14	70641916	70675366
1965	EIF2S1		14	67360151	67386516
8932	MBD2		18	54151606	54224669
92259	MRPS36		5	69217760	69230158
891	CCNB1		5	69167135	69178245
8536	CAMK1		3	9757347	9769992
7428	VHL		3	10141778	10153667
8553	BHLHE40		3	4979437	4985323
55207	ARL8B		3	5122245	5180912

79768	KATNBL1		15	34140674	34210096
10451	VAV3		1	107571161	107965180
84722	PSRC1		1	109279556	109283186
26191	PTPN22		1	113813811	113871753
6272	SORT1		1	109309568	109397918
10542	LAMTOR5		1	110401249	110407942
4853	NOTCH2		1	119911553	120100779
9398	CD101		1	117001750	117036476
8774	NAPG		18	10525905	10552764
51303	FKBP11		12	48921518	48926474
377	ARF3		12	48935723	48957487
79022	TMEM106C		12	47963569	47968878
NA	PLEKHA8P1		12	45173064	45216041
10971	YWHAQ		2	9583967	9630997
29841	GRHL1		2	9951693	10002277
9475	ROCK2		2	11179759	11348330
91543	RSAD2		2	6865806	6898239
129607	CMPK2		2	6840570	6866635
285148	IAH1		2	9473658	9496543
3939	LDHA		11	18394560	18408425
89796	NAV1		1	201622885	201826969
10440	TIMM17A		1	201955503	201970664
6210	RPS15A		16	18781295	18790383
84991	RBM17		10	6089034	6117457
3559	IL2RA		10	6010689	6062370
79746	ECHDC3		10	11742366	11764070
3601	IL15RA		10	5943639	5978187
902	CCNH		5	87318416	87412930
59340	HRH4		18	24460637	24479961
2012	EMP1		12	13196723	13219941
4607	MYBPC3		11	47331406	47352702
1643	DDB2		11	47214465	47239217
53	ACP2		11	47239302	47248906
8933	RTL8C	X		135032355	135033546
51634	RBMX2	X		130401987	130413656
143689	PIWIL4		11	94543840	94621421
9698	PUM1		1	30931506	31065991
8565	YARS1		1	32775237	32818153
1912	PHC2		1	33323623	33431052
55143	CDCA8		1	37692481	37709719
29889	GNL2		1	37566816	37595937
192670	AGO4		1	35808016	35857890
51361	HOOK1		1	59814786	59876322

1573	CYP2J2	1	59893308	59926773
23318	TUT4	1	52408282	52553487
51444	RNF138	18	32091874	32131561
55250	ELP2	18	36129444	36180557
25941	TPGS2	18	36777647	36829216
26519	TIMM10	11	57528464	57530803
9704	DHX34	19	47349315	47382704
9415	FADS2	11	61792980	61867354
746	TMEM258	11	61768501	61792802
6947	TCN1	11	59852800	59866489
55858	TMEM165	4	55395957	55453397
79587	CARS2	13	110641412	110713603
9743	ARHGAP32	11	128965060	129279324
3703	STT3A	11	125591712	125625215
2113	ETS1	11	128458761	128587558
324	APC	5	112707498	112846239
26578	OSTF1	9	75088514	75147265
29979	UBQLN1	9	83659968	83707958
301	ANXA1	9	73151865	73170393
1514	CTSL	9	87726109	87731469
51280	GOLM1	9	86026146	86100173
29968	PSAT1	9	78297125	78330093
84868	HAVCR2	5	157085832	157142869
84749	USP30	12	109023089	109088023
23014	FBXO21	12	117141988	117190531
8638	OASL	12	121019111	121039242
8739	HRK	12	116856144	116881441
1840	DTX1	12	113056709	113098028
10906	TRAFD1	12	112125538	112153604
57639	CCDC146	7	77122434	77329533
85025	TMEM60	7	77793728	77798434
50640	PNPLA8	7	108470417	108569666
29923	HILPDA	7	128455849	128458418
375616	KCP	7	128862451	128910719
26136	TES	7	116210506	116258783
22832	CEP162	6	84124241	84227643
10492	SYNCRIP	6	85607785	85643792
23595	ORC3	6	87590067	87667453
79899	PRR5L	11	36296288	36465204
4005	LMO2	11	33858576	33892076
51317	PHF21A	11	45929323	46121178
55226	NAT10	11	34105617	34147670
79056	PRRG4	11	32829927	32858120

4076	CAPRIN1	11	34051731	34102610
85406	DNAJC14	12	55820960	55830824
967	CD63	12	55725323	55729707
10220	GDF11	12	55743122	55757264
3679	ITGA7	12	55684568	55716043
5959	RDH5	12	55720367	55724705
2647	BLOC1S1	12	55716037	55720087
1019	CDK4	12	57747727	57756013
10024	TROAP	12	49323236	49331731
6302	TSPAN31	12	57738013	57750219
7024	TFCP2	12	51093656	51173135
93058	COQ10A	12	56266858	56270966
9700	ESPL1	12	53268299	53293638
3178	HNRNPA1	12	54280193	54287088
10956	OS9	12	57693955	57721557
9053	MAP7	6	136342281	136550819
8763	CD164	6	109366514	109382467
6610	SMPD2	6	109440724	109443919
64780	MICAL1	6	109444062	109465968
85021	REPS1	6	138903493	138988261
7006	TEC	4	48135783	48269838
10574	CCT7	2	73233420	73253021
10322	SMYD5	2	73214222	73227221
8291	DYSF	2	71453722	71686768
27345	KCNMB4	12	70366290	70434292
9958	USP15	12	62260338	62417431
2799	GNS	12	64713445	64759431
4193	MDM2	12	68808177	68850686
55336	FBXL8	16	67159932	67164570
92922	CCDC102A	16	57512181	57536571
9816	URB2	1	229626247	229660200
22796	COG2	1	230642481	230693982
6041	RNASEL	1	182573634	182589256
1660	DHX9	1	182839347	182887982
57710	KIAA1614	1	180912897	180951614
9857	CEP350	1	179954773	180114875
80896	NPL	1	182789293	182830384
3431	SP110	2	230167293	230225729
65080	MRPL44	2	223957463	223967714
5270	SERPINE2	2	223975045	224039318
1329	COX5B	2	97646062	97648383
51455	REV1	2	99400475	99490035
84804	MFSD9	2	102714630	102736888

10913	EDAR	2	108894471	108989372
9392	TGFBRAP1	2	105264391	105329735
375248	ANKRD36	2	97113153	97264521
84101	USP44	12	95516560	95551476
10154	PLXNC1	12	94148577	94307675
55332	DRAM1	12	101877580	102012130
23325	WASHC4	12	105107324	105169130
2317	FLNB	3	58008400	58172251
79621	RNASEH2B	13	50909747	51024120
9882	TBC1D4	13	75283503	75482169
79866	BORA	13	72727749	72756198
23143	LRCH1	13	46553168	46753040
51131	PHF11	13	49495610	49528981
4008	LMO7	13	75620434	75859870
10253	SPRY2	13	80335976	80341126
9805	SCRN1	7	29920104	29990289
55501	CHST12	7	2403588	2448484
10643	IGF2BP3	7	23310209	23470491
11014	KDELR2	7	6445953	6484190
313	AOAH	7	36512941	36724549
28969	BZW2	7	16646131	16706523
28988	DBNL	7	44044640	44069456
83605	CCM2	7	44999475	45076469
NA		14	20919361	20920299
10588	MTHFS	15	79833585	79897379
57538	ALPK3	15	84816680	84873482
55316	RSAD1	17	50478860	50485974
10238	DCAF7	17	63550477	63594279
83990	BRIP1	17	61679139	61863528
11143	KAT7	17	49788681	49835026
64108	RTP4	3	187368385	187372076
4711	NDUFB5	3	179604690	179627647
57129	MRPL47	3	179588285	179604649
10010	TANK	2	161136908	161236230
6498	SKIL	3	170357678	170396835
3586	IL10	1	206767602	206774541
26750	RPS6KC1	1	213051233	213274774
150472	CBWD2	2	113437691	113496204
3557	IL1RN	2	113107214	113134016
55627	SMPD4	2	130151392	130182750
55339	WDR33	2	127701027	127811187
79595	SAP130	2	127941217	128028120
92856	IMP4	2	130342877	130347967

9394	HS6ST1	2	128236716	128318868
8027	STAM	10	17644151	17716824
10006	ABI1	10	26746593	26861087
10730	YME1L1	10	27110112	27155266
64215	DNAJC1	10	21756548	22003769
25934	NIPSNAP3A	9	104747683	104760120
56262	LRRC8A	9	128882112	128918039
1025	CDK9	9	127785679	127790792
7295	TXN	9	110243810	110256507
4957	ODF2	9	128455186	128501292
23392	ECPAS	9	111360692	111484745
27348	TOR1B	9	129803157	129811281
9314	KLF4	9	107484852	107490482
1861	TOR1A	9	129812942	129824244
153090	DAB2IP	9	121567057	121785530
6812	STXBP1	9	127579370	127696027
1318	SLC31A2	9	113150976	113164140
7099	TLR4	9	117704175	117724735
55014	STX17	9	99906654	99974534
9128	PRPF4	9	113275642	113294009
10868	USP20	9	129834698	129881838
9550	ATP6V1G1	9	114587769	114598879
54881	TEX10	9	100302077	100352942
54534	MRPL50	9	101387633	101398618
5695	PSMB7	9	124353465	124415444
2800	GOLGA1	9	124878275	124948492
10541	ANP32B	9	97983341	98015943
11224	RPL35	9	124857880	124861981
5168	ENPP2	8	119557086	119673453
79075	DSCC1	8	119833976	119855894
79139	DERL1	8	123013170	123042302
4609	MYC	8	127735434	127742951
64425	POLR1E	9	37485948	37503697
55833	UBAP2	9	33921693	34048949
25822	DNAJB5	9	34989641	34998900
51754	TMEM8B	9	35814451	35865518
6194	RPS6	9	19375715	19380236
116113	FOXP4	6	41546426	41602384
63971	KIF13A	6	17759183	17987635
23070	CMTR1	6	37433219	37482827
93643	TJAP1	6	43477523	43506556
9856	KIAA0319	6	24544104	24646191
63027	SLC22A23	6	3268962	3457022

55227	LRRC1	6	53794497	53924125
8737	RIPK1	6	3063991	3115187
3159	HMGA1	6	34236873	34246231
10211	FLOT1	6	30727709	30742732
9656	MDC1	6	30699807	30717447
7172	TPMT	6	18128311	18155077
255488	RNF144B	6	18387350	18468870
129685	TAF8	6	42050513	42087461
51439	FAM8A1	6	17600302	17611715
132864	CPEB2	4	15002674	15070153
85462	FHDC1	4	152936352	152979696
7097	TLR2	4	153684070	153705702
4647	MYO7A	11	77128246	77215241
338699	ANKRD42	11	83193712	83260694
10068	IL18BP	11	71998613	72005715
4926	NUMA1	11	72002864	72080693
60492	CCDC90B	11	83259097	83286407
54843	SYTL2	11	85694224	85811159
2615	LRRC32	11	76657524	76670747
29088	MRPL15	8	54135241	54148514
8836	GGH	8	63015079	63038806
96764	TGS1	8	55773446	55826445
55601	DDX60	4	168216294	168318804
7225	TRPC6	11	101451564	101872562
84259	DCUN1D5	11	103050686	103092194
64776	C11orf1	11	111878935	111885975
53826	FXYD6	11	117836976	117877486
834	CASP1	11	105025443	105035250
838	CASP5	11	104994235	105023168
5607	MAP2K5	15	67542703	67807117
58472	SQOR	15	45631148	45691281
7057	THBS1	15	39581079	39599466
51103	NDUFAF1	15	41387353	41402519
9493	KIF23	15	69414246	69448427
57082	KNL1	15	40594020	40664342
56965	PARP6	15	72241181	72272999
6176	RPLP1	15	69452814	69456205
27229	TUBGCP4	15	43369221	43409771
55177	RMDN3	15	40735884	40755851
55075	UACA	15	70654554	70763558
5330	PLCB2	15	40278176	40307935
80021	TMEM62	15	43123279	43185144
56924	PAK6	15	40217428	40277487

51187	RSL24D1	15	55180806	55197049
51332	SPTBN5	15	41848146	41894053
54874	FNBP1L	1	93448131	93554661
56267	KYAT3	1	88935773	88992953
2959	GTF2B	1	88852633	88891944
10964	IFI44L	1	78619922	78646145
9411	ARHGAP29	1	94148988	94275068
10561	IFI44	1	78649796	78664078
8634	RTCA	1	100266207	100292769
26160	IFT172	2	27444377	27489789
85465	SELENOI	2	26308547	26395891
3795	KHK	2	27086747	27100762
87178	PNPT1	2	55634061	55693863
51626	DYNC2LI1	2	43774039	43810010
8884	SLC5A6	2	27199587	27212958
10128	LRPPRC	2	43886224	43995989
79847	MFSD13A	10	102461395	102477045
3832	KIF11	10	92593130	92655395
1847	DUSP5	10	110497907	110511533
51063	CALHM2	10	103446786	103452402
55165	CEP55	10	93496612	93529092
953	ENTPD1	10	95711779	95877266
54536	EXOC6	10	92826831	93059493
5950	RBP4	10	93591687	93601744
51163	DBR1	3	138160988	138174921
23317	DNAJC13	3	132417502	132539032
310	ANXA7	10	73375101	73414076
51008	ASCC1	10	72096032	72217134
140766	ADAMTS14	10	70672506	70762441
6229	RPS24	10	78033760	78056813
1763	DNA2	10	68414064	68472121
471	ATIC	2	215311956	215349773
50485	SMARCAL1	2	216412414	216483053
580	BARD1	2	214725646	214809683
54529	ASNSD1	2	189661385	189670831
29081	METTL5	2	169810081	169824931
6741	SSB	2	169791933	169812064
65061	CDK15	2	201790461	201895550
9360	PPIG	2	169584344	169641406
57520	HECW2	2	196189099	196593684
3417	IDH1	2	208236227	208266074
29789	OLA1	2	174072447	174248599
9541	CIR1	2	174348022	174395712

6744	ITPRID2	2	181891730	181930738
150864	FAM117B	2	202634969	202769757
10152	ABI2	2	203328239	203447728
30061	SLC40A1	2	189560590	189583758
57337	SENP7	3	101324205	101513241
10063	COX17	3	119654513	119677454
83666	PARP9	3	122527924	122564577
9101	USP8	15	50424380	50514421
84888	SPPL2A	15	50702266	50765709
60490	PPCDC	15	75023586	75117462
8482	SEMA7A	15	74409289	74433958
10144	FAM13A	4	88725955	89111398
8916	HERC3	4	88592434	88708541
55008	HERC6	4	88378739	88443097
51191	HERC5	4	88457119	88506163
51138	COPS4	4	83034447	83075818
3184	HNRNPD	4	82352498	82374503
153020	RASGEF1B	4	81426393	82044244
22872	SEC31A	4	82818509	82901166
55212	BBS7	4	121824329	121870487
84162	KIAA1109	4	122152333	122362758
55132	LARP1B	4	128061286	128222931
8654	PDE5A	4	119494397	119628804
11107	PRDM5	4	120684919	120922870
27163	NAAA	4	75913660	75941013
9908	G3BP2	4	75642782	75724525
55752	SEPTIN11	4	76949703	77040384
950	SCARB2	4	76158737	76234536
246175	CNOT6L	4	77713387	77819615
306	ANXA3	4	78551747	78610451
27068	PPA2	4	105369077	105474067
1062	CENPE	4	103105349	103198445
57117	INTS12	4	105682627	105895986
51176	LEF1	4	108047545	108168956
3033	HADH	4	107989714	108035174
10427	SEC24B	4	109433772	109540896
5998	RGS3	9	113444731	113597743
91445	RNF185	22	31160182	31207019
121506	ERP27	12	14914039	14938537
2120	ETV6	12	11649674	11895377
23710	GABARAPL1	12	10212458	10223128
121512	FGD4	12	32399529	32646050
84920	ALG10	12	34022468	34029694

121536	AEBP2	12	19404045	19720801
254013	ETFBKMT	12	31647160	31673114
85437	ZCRB1	12	42312086	42326118
144165	PRICKLE1	12	42456757	42590355
51279	C1RL	12	7089587	7109238
4704	NDUFA9	12	4649095	4694317
9746	CLSTN3	12	7129698	7158945
55080	TAPBPL	12	6451690	6466517
939	CD27	12	6444867	6451718
5830	PEX5	12	7188685	7218574
9169	SCAF11	12	45919131	45992120
84298	LLPH	12	66116555	66130750
92979	MARCHF9	12	57755103	57760411
1848	DUSP6	12	89347235	89352501
113675	SDSL	12	113422380	113438276
9818	NUP58	13	25301556	25349800
9107	MTMR6	13	25246201	25288009
222484	LNX2	13	27545913	27620529
85478	CCDC65	12	48904110	48931840
8608	RDH16	12	56951431	56959374
94	ACVRL1	12	51906908	51923361
53831	GPR84	12	54362445	54364487
79035	NABP2	12	56222015	56229854
6601	SMARCC2	12	56162359	56189567
675	BRCA2	13	32315086	32400266
11226	GALNT6	12	51351247	51392867
51380	CSAD	12	53157663	53180909
55716	LMBR1L	12	49096551	49110900
60314	C12orf10	12	53299695	53307177
23344	ESYT1	12	56118250	56144671
7009	TMBIM6	12	49707725	49764934
283337	ZNF740	12	53180704	53195142
115825	WDFY2	13	51584455	51767709
144983	HNRNPA1L2	13	52642425	52643796
10161	LPAR6	13	48389567	48444704
283385	MORN3	12	121648742	121672631
65082	VPS33A	12	122229564	122266494
8562	DENR	12	122752824	122771064
122060	SLAIN1	13	77697687	77764242
64062	RBM26	13	79311824	79406477
10150	MBNL2	13	97221434	97394120
84945	ABHD13	13	108218392	108234243
55647	RAB20	13	110523066	110561722

8451	CUL4A	13	113208193	113267108
64403	CDH24	14	23047062	23057538
161253	REM2	14	22883222	22887678
81542	TMX1	14	51240162	51257655
57161	PELI2	14	56117814	56301524
6252	RTN1	14	59595976	59870776
122830	NAA30	14	57390544	57415906
376267	RAB15	14	64945814	64973226
8477	GPR65	14	88005135	88014811
122953	JDP2	14	75427716	75474111
123041	SLC24A4	14	92322581	92501483
122616	CLBA1	14	104985775	105010482
9990	SLC12A6	15	34229784	34338060
6938	TCF12	15	56918623	57299281
6652	SORD	15	45023147	45077185
256586	LYSMD2	15	51723011	51751585
3067	HDC	15	50241947	50265965
2958	GTF2A2	15	59638062	59657541
6727	SRP14	15	40035690	40039181
22893	BAHD1	15	40439721	40468236
85455	DISP2	15	40358219	40378621
54939	COMMD4	15	75336020	75343224
9051	PSTPIP1	15	76993359	77037475
2108	ETFA	15	76215353	76311472
597	BCL2A1	15	79960892	79971196
10099	TSPAN3	15	77041404	77083984
80349	WDR61	15	78277835	78299703
10499	NCOA2	8	70109782	70403808
79661	NEIL1	15	75346955	75357115
55466	DNAJA4	15	78264086	78282196
91947	ARRDC4	15	97960703	97973833
80119	PIF1	15	64815632	64825668
9960	USP3	15	63504511	63594640
585	BBS4	15	72686179	72738475
5371	PML	15	73994673	74047827
170691	ADAMTS17	15	99971437	100342005
10066	SCAMP2	15	74843730	74873365
55215	FANCI	15	89243949	89317261
90381	TICRR	15	89575469	89631056
55070	DET1	15	88494440	88546675
4240	MFGE8	15	88898683	88913381
55784	MCTP2	15	94231538	94483952
64784	CRTC3	15	90529923	90645345

79631	EFL1	15	82130233	82262734
23478	SEC11A	15	84669538	84716460
5373	PMM2	16	8788823	8849325
3687	ITGAX	16	31355134	31382999
7041	TGFB1I1	16	31471585	31477960
79068	FTO	16	53701692	54158512
7385	UQCRC2	16	21953288	21983660
55114	ARHGAP17	16	24919389	25015666
91807	MYLK3	16	46702282	46790407
85407	NKD1	16	50548396	50649249
54957	TXNL4B	16	72044289	72094431
84166	NLRC5	16	56989485	57083531
3801	KIFC3	16	57758217	57863053
146225	CMTM2	16	66579448	66588275
8996	NOL3	16	67170154	67175735
23174	ZCCHC14	16	87406246	87493024
3394	IRF8	16	85899162	85922606
6187	RPS2	16	1962058	1964841
2588	GALNS	16	88813734	88856970
2622	GAS8	16	90019629	90044975
8533	COPS3	17	17246616	17281273
79018	GID4	17	18039408	18068405
57610	RANBP10	16	67723070	67806652
64180	DPEP3	16	67975663	67980549
28987	NOB1	16	69741871	69754926
5636	PRPSAP2	17	18840085	18931287
10140	TOB1	17	50862223	50867978
90507	SCRN2	17	47837692	47841289
22901	ARSG	17	68259182	68422731
51651	PTRH2	17	59674636	59707626
10939	AFG3L2	18	12328944	12377227
3613	IMPA2	18	11981025	12030877
25800	SLC39A6	18	36108531	36129385
83608	C18orf21	18	35972625	35979286
29919	RMC1	18	23503470	23531822
27043	PELP1	17	4669774	4704337
4864	NPC1	18	23506184	23586506
84225	ZMYND15	17	4739833	4746119
55135	WRAP53	17	7686071	7703502
432	ASGR1	17	7173431	7179564
23533	PIK3R5	17	8878911	8965712
7157	TP53	17	7661779	7687550
396	ARHGDIA	17	81867721	81871378

9123	SLC16A3	17	82228397	82261129
79092	CARD14	17	80169992	80209331
94015	TTYH2	17	74213571	74262020
10966	RAB40B	17	82654973	82698698
9775	EIF4A3	17	80134369	80147151
79672	FN3KRP	17	82716706	82730328
3607	FOXK2	17	82519713	82644662
201292	TRIM65	17	75880335	75896951
57332	CBX8	17	79792132	79801683
6398	SECTM1	17	82321024	82334074
56270	WDR45B	17	82614562	82648553
54808	DYM	18	49041474	49461347
4089	SMAD4	18	51028394	51085045
54877	ZCCHC2	18	62523025	62587709
5366	PMAIP1	18	59899996	59904305
10609	P3H4	17	41801947	41812604
162427	RETREG3	17	42579513	42610623
84299	MIEN1	17	39728496	39730532
773	CACNA1A	19	13206442	13633025
90378	SAMD1	19	14087851	14091036
23476	BRD4	19	15235519	15332545
4782	NFIC	19	3314403	3469217
5211	PFKL	21	44300051	44327376
117286	CIB3	19	16161368	16173525
63946	DMRTC2	19	41844743	41852333
90324	CCDC97	19	41310172	41324873
641649	TMEM91	19	41350911	41384083
10410	IFITM3	11	319676	327537
80162	PGGHG	11	289126	296107
1291	COL6A1	21	45981769	46005050
6647	SOD1	21	31659666	31668931
1292	COL6A2	21	46098112	46132848
351	APP	21	25880550	26171128
10055	SAE1	19	47113274	47210636
79760	GEMIN7	19	45079195	45091518
81794	ADAMTS10	19	8580240	8610735
4542	MYO1F	19	8520778	8577577
91662	NLRP12	19	53793741	53824394
59283	CACNG8	19	53963040	53990215
10498	CARM1	19	10871553	10923075
5694	PSMB6	17	4796144	4798502
89790	SIGLEC10	19	51410020	51417803
25888	ZNF473	19	50025714	50053414

80110	ZNF614	19	52012765	52030240
6518	SLC2A5	1	9035106	9088478
473	RERE	1	8352397	8848921
5195	PEX14	1	10472288	10630758
6135	RPL11	1	23691742	23696835
79932	KIAA0319L	1	35433492	35557950
10733	PLK4	4	127880893	127899224
9064	MAP3K6	1	27354067	27366961
113451	AZIN2	1	33081104	33123492
11004	KIF2C	1	44739818	44767767
148932	MOB3C	1	46607715	46616891
22823	MTF2	1	93079235	93139079
85369	STRIP1	1	110031577	110074641
5686	PSMA5	1	109399042	109426448
128346	C1orf162	1	111473792	111478512
963	CD53	1	110871188	110899922
223	ALDH9A1	1	165662216	165698863
481	ATP1B1	1	169105697	169132722
261726	TIPRL	1	168178962	168202109
29922	NME7	1	169132531	169367948
57645	POGK	1	166839447	166856344
8804	CREG1	1	167529117	167553805
10223	GPA33	1	167052836	167166479
7371	UCK2	1	165827614	165911618
54499	TMCO1	1	165724293	165827755
6375	XCL1	1	168576605	168582069
5451	POU2F1	1	167220876	167427345
2212	FCGR2A	1	161505430	161524013
83540	NUF2	1	163266576	163355764
5202	PFDN2	1	161100556	161118055
27005	USP21	1	161159450	161165723
5546	PRCC	1	156750610	156800815
51093	RRNAD1	1	156728442	156736960
1382	CRABP2	1	156699606	156705816
27	ABL2	1	179099330	179229684
6004	RGS16	1	182598623	182604389
26092	TOR1AIP1	1	179882042	179925000
23179	RGL1	1	183636085	183928531
6097	RORC	1	151806071	151831845
7286	TUFT1	1	151540305	151583583
10262	SF3B4	1	149923317	149927803
81609	SNX27	1	151612006	151699091
9869	SETDB1	1	150926263	150964744

54507	ADAMTSL4	1	150549369	150560937
4170	MCL1	1	150574551	150579738
29956	CERS2	1	150960583	150975004
2029	ENSA	1	150600851	150629612
10500	SEMA6C	1	151131685	151146664
405	ARNT	1	150809713	150876708
84072	HORMAD1	1	150698060	150720895
55204	GOLPH3L	1	150646230	150697154
51514	DTL	1	212035553	212107400
8444	DYRK3	1	206635536	206684419
1939	EIF2D	1	206571292	206612465
55061	SUSD4	1	223220819	223364178
11221	DUSP10	1	221701424	221742089
8751	ADAM15	1	155050566	155062775
10899	JTB	1	153974269	153977674
6279	S100A8	1	153390032	153391073
91181	NUP210L	1	153992685	154155116
23557	SNAPIN	1	153658703	153661852
11000	SLC27A3	1	153774354	153780157
9898	UBAP2L	1	154220179	154271510
10456	HAX1	1	154272589	154275875
3782	KCNN3	1	154697455	154870281
25912	C1orf43	1	154206696	154220637
6016	RIT1	1	155897808	155911404
65123	INTS3	1	153728050	153774808
51097	SCCPDH	1	246724409	246768137
1130	LYST	1	235661041	235883640
116841	SNAP47	1	227728539	227781231
6726	SRP9	1	225777813	225790468
8560	DEGS1	1	224175756	224193441
29097	CNIH4	1	224356858	224379459
3707	ITPKB	1	226631690	226739323
79169	C1orf35	1	228100726	228105411
129642	MBOAT2	2	8852690	9003709
142	PARP1	1	226360691	226408093
29920	PYCR2	1	225919877	225924340
2052	EPHX1	1	225810092	225845563
9580	SOX13	1	204073115	204127743
55224	ETNK2	1	204131062	204152044
127833	SYT2	1	202590596	202710454
10130	PDIA6	2	10783391	10837977
92906	HNRNPLL	2	38561969	38603586
130589	GALM	2	38666081	38741237

805	CALM2	2	47160083	47176921
494143	CHAC2	2	53767804	53775196
6233	RPS27A	2	55231903	55235853
51542	VPS54	2	63892146	64019428
6637	SNRPG	2	70281362	70293740
9391	CIAO1	2	96266159	96274173
51002	TPRKB	2	73729104	73737400
23233	EXOC6B	2	72175984	72826041
94097	SFXN5	2	72942036	73075619
113419	TEX261	2	70985942	70994873
5899	RALB	2	120240064	120294710
80775	TMEM177	2	119679167	119686507
284958	NT5DC4	2	112721486	112742879
129804	FBLN7	2	112138385	112188218
84524	ZC3H8	2	112211529	112255136
3899	AFF3	2	99545419	100192428
339745	SPOPL	2	138501770	138573547
83607	AMMECR1L	2	127861630	127885956
8502	PKP4	2	158456964	158682879
57282	SLC4A10	2	161424332	161985282
79634	SCRN3	2	174395730	174429575
83879	CDCA7	2	173354820	173368997
165215	FAM171B	2	186694060	186765959
151194	METTL21A	2	207580631	207625928
65065	NBEAL1	2	203014879	203226378
285362	SUMF1	3	3700814	4467273
84236	RHBDD1	2	226835581	226999215
57007	ACKR3	2	236567787	236582354
64708	COPS7B	2	231781671	231809254
2177	FANCD2	3	10026414	10101930
9686	VGLL4	3	11556069	11771350
114790	STK11IP	2	219597857	219616451
85403	EAF1	3	15427598	15450635
114884	OSBPL10	3	31657890	32077580
84892	POMGNT2	3	43079232	43106079
64651	CSRNP1	3	39141855	39154562
23066	CAND2	3	12796472	12871916
55164	SHQ1	3	72749277	72861914
9039	UBA3	3	69054730	69080408
10550	ARL6IP5	3	69084937	69106092
7110	TMF1	3	69019827	69052339
26018	LRIG1	3	66378797	66501263
91775	NXPE3	3	101779202	101828231

90102	PHLDB2	3	111732497	111976517
64422	ATG3	3	112532510	112562046
66000	TMEM108	3	133038391	133397792
116931	MED12L	3	151085697	151437072
83939	EIF2A	3	150546678	150586016
7220	TRPC1	3	142724034	142807888
4026	LPP	3	188153284	188890671
9711	RUBCN	3	197668867	197749727
9730	DCAF1	3	51395867	51500002
7873	MANF	3	51385291	51389397
55840	EAF2	3	121835183	121886526
286676	ILDR1	3	121987323	122022247
8893	EIF2B5	3	184135038	184146127
10861	SLC26A1	4	979073	993440
79966	SCD5	4	82629539	82798796
51316	PLAC8	4	83090048	83137075
93587	TRMT10A	4	99546709	99564039
57563	KLHL8	4	87160103	87240314
100996939	PYURF	4	88520998	88523776
817	CAMK2D	4	113451032	113761927
92610	TIFA	4	112274537	112285904
166378	SPATA5	4	122923078	123319433
890	CCNA2	4	121816444	121823933
57721	METTL14	4	118685392	118715433
54532	USP53	4	119212587	119295517
80854	SETD7	4	139495941	139606699
92345	NAF1	4	163110073	163166890
55016	MARCHF1	4	163524298	164384050
6423	SFRP2	4	153780591	153789083
285533	RNF175	4	153710160	153760024
56034	PDGFC	4	156760454	156971799
83853	ROPN1L	5	10441524	10472029
6715	SRD5A1	5	6633427	6674386
4651	MYO10	5	16661907	16936288
54491	OTULINL	5	14581792	14616180
3001	GZMA	5	55102646	55110252
5295	PIK3R1	5	68215756	68301821
10184	LHFPL2	5	78485215	78770021
54826	GIN1	5	103086000	103120138
64924	SLC30A5	5	69093949	69131069
64839	FBXL17	5	107859035	108382098
51397	COMMD10	5	116085016	116412762
9140	ATG12	5	115828200	115841837

81555	YIPF5	5	144158162	144170714
23092	ARHGAP26	5	142770377	143229011
9879	DDX46	5	134758771	134855133
81545	FBXO38	5	148383935	148442836
78991	PCYOX1L	5	149358037	149369653
10146	G3BP1	5	151771045	151812785
23138	N4BP3	5	178113532	178126081
26240	FAM50B	6	3849373	3851320
340156	MYLK4	6	2663629	2750922
51256	TBC1D7	6	13266542	13328583
153527	ZMAT2	5	140698680	140706686
26249	KLHL3	5	137617500	137736089
54540	FAM193B	5	177519789	177554563
27242	TNFRSF21	6	47231532	47309905
22838	RNF44	5	176526712	176538025
4594	MMUT	6	49430360	49463253
23500	DAAM2	6	39792298	39904877
51557	LGSN	6	63275951	63319983
221472	FGD2	6	37005646	37029069
55023	PHIP	6	78934419	79078254
57038	RARS2	6	87513938	87589987
256380	SCML4	6	107702154	107824317
93663	ARHGAP18	6	129576132	129710177
58527	ABRACL	6	139028745	139043302
113115	MTFR2	6	136231024	136250335
6993	DYNLT1	6	158636474	158644743
9589	WTAP	6	159725585	159756319
79624	ARMT1	6	151452258	151470101
2768	GNA12	7	2728105	2844308
84310	C7orf50	7	996986	1138260
NA	WASH2P	2	113588550	113599043
221960	CCZ1B	7	6794134	6826770
79034	C7orf26	7	6590021	6608726
9586	CREB5	7	28299321	28825894
113130	CDCA5	11	65066300	65084164
5814	PURB	7	44876299	44885530
4191	MDH2	7	76048051	76067508
908	CCT6A	7	56051685	56063989
222255	ATXN7L1	7	105605067	105876604
56996	SLC12A9	7	100826820	100867010
64599	GIGYF1	7	100679507	100694037
89122	TRIM4	7	99876958	99919600
56257	MEPCE	7	100428790	100434118

84928	TMEM209		7	130164713	130207770
55281	TMEM140		7	135148072	135166215
11011	TLK2		17	62458658	62615481
2041	EPHA1		7	143390289	143408856
64434	NOM1		7	156949712	156973176
54892	NCAPG2		7	158631169	158704804
401409	RAB19		7	140404058	140427974
51631	LUC7L2		7	139340359	139423457
347404	LANCL3	X		37571569	37684463
56548	CHST7	X		46573765	46598496
7592	ZNF41	X		47445879	47482946
9203	ZMYM3	X		71239624	71255146
4841	NONO	X		71283192	71301168
8473	OGT	X		71533104	71575892
3561	IL2RG	X		71107404	71112108
7552	ZNF711	X		85244032	85273362
56000	NXF3	X		103075810	103093143
55086	RADX	X		106611930	106679439
27316	RBMX	X		136848004	136880764
50814	NSDHL	X		152830967	152869729
7739	ZNF185	X		152914442	152973480
6134	RPL10	X		154389955	154409168
55909	BIN3		8	22620418	22669148
9046	DOK2		8	21908873	21913690
51312	SLC25A37		8	23528956	23575463
91782	CHMP7		8	23243637	23262000
80005	DOCK5		8	25184689	25418082
11212	PLPBP		8	37762595	37779768
84296	GINS4		8	41529218	41545030
137872	ADHFE1		8	66432492	66468907
28957	MRPS28		8	79918717	80030289
29967	LRP12		8	104489231	104589024
5440	POLR2K		8	100150623	100154003
8667	EIF3H		8	116642130	116766925
83940	TATDN1		8	124488485	124539458
7553	ZNF7		8	144827464	144847509
7436	VLDLR		9	2621787	2660056
50808	AK3		9	4709556	4742043
1029	CDKN2A		9	21967753	21995301
203228	C9orf72		9	27535640	27573866
10280	SIGMAR1		9	34634722	34637809
220869	CBWD5		9	65668805	65734041
84641	MFSD14B		9	94374569	94461042

7357	UGCG	9	111896814	111935369
2040	STOM	9	121338987	121370304
210	ALAD	9	113386312	113401290
54107	POLE3	9	113407235	113410675
25792	CIZ1	9	128161251	128204383
3934	LCN2	9	128149071	128153453
90678	LRSAM1	9	127451486	127503501
9919	SEC16A	9	136440096	136483759
92715	DPH7	9	137554444	137578925
4851	NOTCH1	9	136494433	136546048
254427	PROSER2	10	11823339	11872277
23412	COMMD3	10	22316386	22320306
22921	MSRB2	10	23095579	23122013
23590	PDSS1	10	26697701	26746798
221061	FAM171A1	10	15211643	15371289
80013	MINDY3	10	15778170	15860507
92211	CDHR1	10	84194537	84219621
2746	GLUD1	10	87050202	87094843
54788	DNAJB12	10	72332830	72355149
4288	MKI67	10	128096659	128126423
9446	GSTO1	10	104235356	104267459
6877	TAF5	10	103367976	103389065
23082	PPRC1	10	102132994	102150333
85450	ITPRIP	10	104309698	104338465
8038	ADAM12	10	126012381	126388455
84280	BTBD10	11	13388001	13463297
133	ADM	11	10305073	10307397
27315	PGAP2	11	3797724	3826371
64852	TUT1	11	62575045	62591637
51144	HSD17B12	11	43680680	43856617
85456	TNKS1BP1	11	57299638	57324952
710	SERPING1	11	57597387	57614848
6749	SSRP1	11	57325986	57335892
8501	SLC43A1	11	57484534	57515780
79096	C11orf49	11	46936689	47164385
51501	HIKESHI	11	86302211	86345943
23052	ENDOD1	11	95089846	95132645
79780	CCDC82	11	96352769	96389956
871	SERPINH1	11	75562056	75572783
726	CAPN5	11	77066961	77126155
92105	INTS4	11	77878720	77994671
85463	ZC3H12C	11	110093392	110171841
4863	NPAT	11	108157215	108222638

60496	AASDHPPT	11	106075501	106098699
89944	GLB1L2	11	134331874	134378341
128710	SLX4IP	20	10435305	10636829
57325	KAT14	20	18138118	18188387
26007	TKFC	11	61333210	61353295
9219	MTA2	11	62593214	62601865
6094	ROM1	11	62611722	62615120
3619	INCENP	11	62123998	62153169
932	MS4A3	11	60056587	60071115
9651	PLCH2	1	2425980	2505532
79869	CPSF7	11	61402641	61430031
2206	MS4A2	11	60088261	60098467
1111	CHEK1	11	125625665	125676255
9638	FEZ1	11	125442881	125592568
84866	TMEM25	11	118531041	118547280
79980	DSN1	20	36751791	36773818
140710	SOGA1	20	36777447	36863538
149986	LSM14B	20	62122461	62135374
54915	YTHDF1	20	63195429	63216139
220064	LTO1	11	69653076	69675416
740	MRPL49	11	65122183	65127371
2197	FAU	11	65120630	65122177
7108	TM7SF2	11	65111845	65116408
738	VPS51	11	65089324	65111862
8479	HIRIP3	16	29992330	29996074
9344	TAOK2	16	29973868	29992261
22866	CNKSR2	X	21372801	21654695
51348	KLRF1	12	9827481	9845007
51267	CLEC1A	12	10069554	10111627
51503	CWC15	11	94962620	94973586
2209	FCGR1A	1	149782671	149792518
55208	DCUN1D2	13	113455819	113490951
57536	KIAA1328	18	36829106	37232172
3176	HNMT	2	137964020	138016364
27250	PDCD4	10	110871795	110900006
2823	GPM6A	4	175632934	176002664
64407	RGS18	1	192158462	192185815
54545	MTMR12	5	32226994	32312987
22948	CCT5	5	10249929	10266389
85458	DIXDC1	11	111927144	112022653
1737	DLAT	11	112025408	112064404
26521	TIMM8B	11	112084800	112086798
3606	IL18	11	112143253	112164096

5805	PTS	11	112226367	112269955
5305	PIP4K2A	10	22534854	22714578
2308	FOXO1	13	40555667	40666641
51232	CRIM1	2	36355778	36551135
23457	ABCB9	12	122920951	122981649
196383	RILPL2	12	123410683	123436717
7316	UBC	12	124911604	124917368
23657	SLC7A11	4	138164097	138242349
25819	NOCT	4	139015781	139045939
7068	THRB	3	24117153	24495756
55293	UEVLD	11	18529609	18588747
144110	TMEM86A	11	18693122	18704785
90488	TMEM263	12	106955719	106978778
121551	BTBD11	12	107318421	107659642
196463	PLBD2	12	113358566	113391629
5756	TWF1	12	43793723	43806375
1977	EIF4E	4	98871684	98930637
93081	TEX30	13	102765888	102773811
1456	CSNK1G3	5	123512099	123617045
9472	AKAP6	14	32329298	32837684
283635	FAM177A1	14	35044907	35113130
51562	MBIP	14	36298564	36320637
2132	EXT2	11	44095673	44251962
10873	ME3	11	86441108	86672636
80224	NUBPL	14	31489956	31861224
63894	VIPAS39	14	77426675	77457952
132320	SCLT1	4	128864921	129093600
132321	C4orf33	4	129093317	129116640
55691	FRMD4A	10	13643706	14462142
5800	PTPRO	12	15322257	15602175
27034	ACAD8	11	134253548	134265855
29087	THYN1	11	134248279	134253370
143187	VTI1A	10	112446998	112818744
5860	QDPR	4	17460261	17512206
57700	FAM160B1	10	114821744	114899832
4306	NR3C2	4	148078762	148444698
22944	KIN	10	7750962	7787993
5281	PIGF	2	46580937	46617055
3628	INPP1	2	190343570	190371665
54842	MFSD6	2	190408355	190509205
6868	ADAM17	2	9488486	9556732
80014	WWC2	4	183099257	183320777
79682	CENPU	4	184694085	184734130

	2180	ACSL1	4	184755595	184826818
	196394	AMN1	12	31671142	31729121
	636	BICD1	12	32106835	32383633
	60485	SAV1	14	50632058	50668306
	51594	NBAS	2	15166914	15561334
	26272	FBXO4	5	41925254	41941743
	56477	CCL28	5	43376645	43412391
	79668	PARP8	5	50665899	50846519
	7073	TIAL1	10	119571802	119597029
	9531	BAG3	10	119651380	119677819
	144423	GLT1D1	12	128853427	128984968
	121256	TMEM132D	12	129071725	129904025
	148534	TLCD4	1	95117355	95197607
	130074	FAM168B	2	131047876	131093460
NA		SMPD4BP	2	131492813	131521573
	4249	MGAT5	2	134119983	134454621
	79596	OBI1	13	78614289	78659155
	57105	CYSLTR2	13	48653711	48711226
	115761	ARL11	13	49628507	49633872
	57724	EPG5	18	45847609	45967329
	9050	PSTPIP2	18	45983536	46072272
	115106	HAUS1	18	46104378	46128333
	147339	C18orf25	18	46173553	46266992
	83734	ATG10	5	81972023	82276857
	134359	POC5	5	75674124	75717448
	167555	FAM151B	5	80487969	80542563
	117143	TADA1	1	166856510	166876264
	143884	CWF19L2	11	107326345	107457844
	7518	XRCC4	5	83077498	83353787
	374928	ZNF773	19	57499915	57518404
	79723	SUV39H2	10	14878820	14904315
	64421	DCLRE1C	10	14897359	14954432
	55301	OLAH	10	15032227	15073853
	9397	NMT2	10	15102584	15168693
	116412	ZNF837	19	58367618	58381030
	152137	CCDC50	3	191329085	191398659
	814	CAMK4	5	111223653	111494886
	678	ZFP36L2	2	43222402	43226606
	5213	PFKM	12	48105139	48146404
	114908	TMEM123	11	102396332	102470384
	23171	GPD1L	3	32105689	32168709
	25780	RASGRP3	2	33436324	33564750
	160897	GPR180	13	94601857	94634661

200132	TCTEX1D1	1	66752459	66779047
118932	ANKRD22	10	88819896	88851844
24138	IFIT5	10	89414568	89420997
9987	HNRNPDL	4	82422564	82430408
9453	GGPS1	1	235327350	235344532
51351	ZNF117	7	64971772	65006684
9412	MED21	12	27022546	27066343
166647	ADGRA3	4	22345071	22516066
10283	CWC27	5	64768930	65018750
3140	MR1	1	181033425	181061938
6728	SRP19	5	112861188	112898371
9425	CDYL	6	4706159	4955551
51061	TXNDC11	16	11679080	11742878
55289	ACOXL	2	110732573	111118222
64682	ANAPC1	2	111611639	111884690
1070	CETN3	5	90392261	90409786
283349	RASSF3	12	64507001	64697564
3192	HNRNPU	1	244840638	244864560
4929	NR4A2	2	156324437	156342348
5937	RBMS1	2	160272151	160493807
10225	CD96	3	111292719	111665750
51571	CYRIB	8	129839593	130017129
NA	LINC00467	1	211382736	211435570
64795	RMND5A	2	86720291	86778041
2960	GTF2E1	3	120742637	120783069
10428	CFDP1	16	75293698	75433503
136895	C7orf31	7	25134692	25180356
221895	JAZF1	7	27830573	28180795
55022	PID1	2	228850526	229271285
9320	TRIP12	2	229763838	229923239
79047	KCTD15	19	33795933	33815763
148103	ZNF599	19	34758073	34773229
255231	MCOLN2	1	84925583	84997113
1105	CHD1	5	98853985	98928957
124540	MSI2	17	57255851	57684689
221302	ZUP1	6	116635618	116668794
5529	PPP2R5E	14	63371364	63543377
125206	SLC5A10	17	18950345	19022595
26289	AK5	1	77282019	77559966
123872	DNAAF1	16	84145287	84178767
404550	C16orf74	16	85690084	85751129
56172	ANKH	5	14704800	14871778
84959	UBASH3B	11	122655722	122814473

64221	ROBO3	11	124865432	124881471
2838	GPR15	3	98531978	98534681
284	ANGPT1	8	107249482	107498055
26207	PITPNC1	17	67377281	67697261
5578	PRKCA	17	66302613	66810743
79705	LRRK1	15	100919327	101078257
10349	ABCA10	17	69147214	69244846
5169	ENPP3	6	131628442	131747418
55286	C4orf19	4	37453925	37623495
7345	UCHL1	4	41256413	41268455
375056	MIA3	1	222618097	222668007
84976	DISP1	1	222872271	223005995
5239	PGM5	9	68328308	68531061
115362	GBP5	1	89258950	89272804
518	ATP5MC3	2	175176258	175184607
6921	ELOC	8	73939169	73972287
23643	LY96	8	73991392	74029079
10950	BTG3	21	17593653	17612945
91133	L3MBTL4	18	5954706	6415237
27342	RABGEF1	7	66682164	66811464
54148	MRPL39	21	25585656	25607517
522	ATP5PF	21	25716503	25735673
9510	ADAMTS1	21	26835755	26845409
11096	ADAMTS5	21	26917922	26967088
146857	SLFN13	17	35435096	35448837
7476	WNT7A	3	13816258	13880071
201163	FLCN	17	17212212	17237188
285381	DPH3	3	16257061	16264969
9989	PPP4R1	18	9546791	9615240
124739	USP43	17	9644698	9729687
51560	RAB6B	3	133824235	133895882
2047	EPHB1	3	134597801	135260467
84532	ACSS1	20	25006230	25058980
91369	ANKRD40	17	50693198	50707914
7566	ZNF18	17	11977439	11997475
113612	CYP2U1	4	107931549	107953461
5799	PTPRN2	7	157539056	158587823
528	ATP6V1C1	8	103021063	103073051
55529	PIP4P2	8	90993802	91040872
64210	MMS19	10	97458324	97498794
55361	PI4K2A	10	97640686	97676434
118813	ZFYVE27	10	97737121	97760907
64092	SAMSN1	21	14485228	14658821

29761	USP25		21	15730025	15880069
54819	ZCCHC10		5	132996985	133026604
4343	MOV10		1	112673141	112700746
1622	DBI		2	119366921	119372560
84365	NIFK		2	121726945	121736911
23367	LARP1		5	154712843	154817605
342357	ZKSCAN2		16	25236001	25257845
138241	C9orf85		9	71911510	71986054
118788	PIK3AP1		10	96593315	96720514
129831	RBM45		2	178112424	178139011
7273	TTN		2	178525989	178830802
11326	VSIG4	X		66021738	66040125
285172	FAM126B		2	200973718	201071671
133522	PPARGC1B		5	149730298	149855022
9443	MED7		5	157137424	157159019
6503	SLA		8	133036728	133102912
51643	TMBIM4		12	66135846	66170072
7411	VBP1	X		155197007	155239841
116442	RAB39B	X		155258235	155264491
1193	CLIC2	X		155276211	155334657
23362	PSD3		8	18527303	19084730
145483	FAM161B		14	73931501	73950414
3778	KCNMA1		10	76869601	77638369
10538	BATF		14	75522455	75546993
137682	NDUFAF6		8	94895767	95116455
128338	DRAM2		1	111117333	111140216
10600	USP16		21	29024629	29054488
571	BACH1		21	29194071	29630751
7102	TSPAN7	X		38561370	38688920
7074	TIAM1		21	31118416	31559977
57466	SCAF4		21	31671000	31732118
23552	CDK20		9	87966441	87974753
119392	SFR1		10	104122058	104126385
9556	ATP5MPL		14	103912288	103928269
122402	TDRD9		14	103928456	104052667
5097	PCDH1		5	141853111	141879246
153769	SH3RF2		5	145936578	146081791
7381	UQCRB		8	96222947	96235546
51001	MTERF3		8	96239398	96261610
9791	PTDSS1		8	96261902	96336995
6156	RPL30		8	98024851	98046469
159091	FAM122C	X		134796395	134854835
6832	SUPV3L1		10	69180234	69209099

159090	FAM122B	X		134769566	134797232
80201	HKDC1		10	69220332	69267552
3098	HK1		10	69269984	69401884
219743	TYSND1		10	70137981	70146700
9246	UBE2L6		11	57551656	57568284
142891	SAMD8		10	75099586	75182123
9131	AIFM1	X		130129362	130165887
5603	MAPK13		6	36127809	36144524
93594	TBC1D31		8	123041968	123152153
55093	WDYHV1		8	123416726	123470028
29028	ATAD2		8	123319850	123416350
114907	FBXO32		8	123497889	123541206
286053	NSMCE2		8	125091679	125367120
115509	ZNF689		16	30602558	30624012
64645	MFSD14A		1	100038095	100083377
3681	ITGAD		16	31393335	31426505
23355	VPS8		3	184812143	185052614
2585	GALK2		15	49155656	49367869
701	BUB1B		15	40161023	40221123
5147	PDE6D		2	231732433	231786272
7862	BRPF1		3	9731729	9748015
9372	ZFYVE9		1	52142094	52346686
23049	SMG1		16	18804860	18926408
134285	TMEM171		5	73120569	73131809
6854	SYN2		3	12004388	12192032
7804	LRP8		1	53242364	53328469
22976	PAXIP1		7	154943687	155003124
23648	SSBP3		1	54225432	54413479
4323	MMP14		14	22836560	22849027
8321	FZD1		7	91264433	91271326
NA	ZFHX2-AS1		14	23511760	23560778
221481	ARMC12		6	35737032	35749079
11269	DDX19B		16	70289663	70335305
6483	ST3GAL2		16	70375977	70439237
146433	IL34		16	70579895	70660682
3815	KIT		4	54657918	54740715
55799	CACNA2D3		3	54122547	55074557
9133	CCNB2		15	59105126	59125045
4643	MYO1E		15	59132434	59372871
1831	TSC22D3	X		107713221	107777342
3772	KCNJ15		21	38155549	38307357
2078	ERG		21	38380027	38661780
90139	TSPAN18		11	44726465	44932423

84187	TMEM164	X		110002631	110182734
4599	MX1		21	41420304	41459214
257397	TAB3	X		30827442	30975084
445815	PALM2AKAP2		9	109498325	110172512
114991	ZNF618		9	113876282	114056591
203197	TMEM268		9	114611206	114646422
84255	SLC37A3		7	140293693	140404433
114793	FMNL2		2	152335174	152649826
150946	GAREM2		2	26173088	26189663
121665	SPPL3		12	120762510	120904358
92749	DRC1		2	26401920	26456711
127281	PRXL2B		1	2586491	2591469
8764	TNFRSF14		1	2555639	2565382
55229	PANK4		1	2508537	2526597
64897	C12orf43		12	121000486	121016502
11079	RER1		1	2391775	2405442
6497	SKI		1	2228319	2310213
26119	LDLRAP1		1	25543606	25568886
116987	AGAP1		2	235494043	236131800
5051	PAFAH2		1	25959767	25998117
9577	BABAM2		2	27889941	28338901
63875	MRPL17		11	6680385	6683340
138639	PTPDC1		9	94030794	94109856
79623	GALNT14		2	30910467	31155202
3242	HPD		12	121839527	121863596
26504	CNNM4		2	96760902	96811874
2140	EYA3		1	27970344	28088637
199221	DZIP1L		3	138061990	138115818
22808	MRAS		3	138347648	138405534
171586	ABHD3		18	21650901	21704780
388591	RNF207		1	6205475	6221299
8450	CUL4B	X		120523858	120575794
26053	AUTS2		7	69598296	70793506
57477	SHROOM4	X		50591647	50814302
3017	H2BC5		6	26158146	26171349
8365	H4C8		6	26277609	26285638
129531	MITD1		2	99161427	99181058
9669	EIF5B		2	99337371	99401326
55571	CNOT11		2	101252886	101270316
3745	KCNB1		20	49293394	49484297
23382	AHCYL2		7	129225023	129430211
9334	B4GALT5		20	49632945	49713878
9825	SPATA2		20	49903391	49915529

911	CD1C		1	158289923	158294774
90121	TSR2	X		54440404	54448032
124245	ZC3H18		16	88570381	88631966
130617	ZFAND2B		2	219195237	219209651
84919	PPP1R15B		1	204403381	204411817
26958	COPG2		7	130506238	130668748
85414	SLC45A3		1	205657851	205680509
115992	RNF166		16	88696499	88706408
3362	HTR6		1	19664875	19680966
7391	USF1		1	161039251	161045977
124044	SPATA2L		16	89696357	89701705
9191	DEDD		1	161120974	161132688
92822	ZNF276		16	89720400	89740925
1896	EDA	X		69616067	70039472
2207	FCER1G		1	161215234	161220699
84134	TOMM40L		1	161225939	161230746
4359	MPZ		1	161304735	161309972
57805	CCAR2		8	22604632	22621514
56990	CDC42SE2		5	131245493	131398447
51735	RAPGEF6		5	131423921	131635236
56683	CFAP298		21	32592079	32612603
3460	IFNGR2		21	33402896	33479348
6651	SON		21	33543038	33577481
9900	SV2A		1	149903318	149917844
714	C1QC		1	22643633	22648110
149473	CCDC24		1	43991359	43996528
861	RUNX1		21	34787801	36004667
23515	MORC3		21	36320189	36386148
201176	ARHGAP27		17	45393902	45434421
83440	ADPGK		15	72751369	72785846
5763	PTMS		12	6765516	6770952
23569	PADI4		1	17308195	17364004
51706	CYB5R1		1	202961873	202967275
23400	ATP13A2		1	16985958	17011928
130951	M1AP		2	74557883	74648338
11189	CELF3		1	151700058	151716803
117145	THEM4		1	151870866	151909637
197131	UBR1		15	42942897	43106113
267	AMFR		16	56361452	56425545
266747	RGL4		22	23688136	23699176
64843	ISL2		15	76336773	76342475
149483	CCDC17		1	45620044	45624057
60313	GPBP1L1		1	45627304	45688113

221188	ADGRG5		16	57542643	57591681
9813	EFCAB14		1	46674659	46719146
51673	TPPP3		16	67389809	67393518
29800	ZDHHC1		16	67394152	67416833
146206	CARMIL2		16	67644988	67657569
9715	FAM131B		7	143353400	143362770
29	ABR		17	1003518	1229738
150275	CCDC117		22	28772674	28789301
7773	ZNF230		19	44002957	44013924
7673	ZNF222		19	44025342	44033110
4882	NPR2		9	35792154	35809732
10020	GNE		9	36214441	36277056
115650	TNFRSF13C		22	41922023	41926818
2909	ARHGAP35		19	46860997	47005077
808	CALM3		19	46601074	46610782
1676	DFFA		1	10456522	10472529
79140	CCDC28B		1	32200386	32205387
55721	IQCC		1	32205661	32208687
56063	TMEM234		1	32214472	32222359
149076	ZNF362		1	33256492	33300719
2063	NR2F6		19	17231883	17245940
131076	CCDC58		3	122359591	122383231
9619	ABCG1		21	42199689	42297244
53347	UBASH3A		21	42403447	42447684
5152	PDE9A		21	42653621	42775509
10785	WDR4		21	42843094	42879568
4731	NDUFV3		21	42879644	42913304
23076	RRP1B		21	43659560	43696079
2539	G6PD	X		154531391	154547572
1476	CSTB		21	43772511	43776318
8568	RRP1		21	43789513	43805293
326	AIRE		21	44285838	44298648
81543	LRRC3		21	44455510	44462196
3689	ITGB2		21	44885953	44931989
26086	GPSM1		9	136327476	136359605
27433	TOR2A		9	127731524	127735313
84885	ZDHHC12		9	128720870	128724127
57551	TAOK1		17	29390464	29551904
284086	NEK8		17	28725897	28743455
9159	PCSK7		11	117204337	117232525
6294	SAFB		19	5623035	5668478
643	CXCR5		11	118883892	118897787
51043	ZBTB7B		1	155002630	155018522

6464	SHC1	1	154962298	154974395
55823	VPS11	11	119067692	119081978
103	ADAR	1	154582057	154628013
55585	UBE2Q1	1	154548577	154559028
1141	CHRNB2	1	154567778	154580013
200186	CRTC2	1	153947669	153958615
23623	RUSC1	1	155320894	155331114
10712	FAM189B	1	155247205	155255483
79957	PAQR6	1	156243320	156248117
9673	SLC25A44	1	156193932	156212796
4000	LMNA	1	156082573	156140089
23218	NBEAL2	3	46979666	47009704
56893	UBQLN4	1	156035299	156053798
2264	FGFR4	5	177086905	177098144
3101	HK3	5	176880869	176899346
84124	ZNF394	7	99473877	99504857
4061	LY6E	8	143017982	143023832
51160	VPS28	8	144423601	144428563
84939	PWWP3A	19	1354711	1378431
9401	RECQL4	8	144511288	144517845
84449	ZNF333	19	14689801	14733746
80228	ORAI2	7	102433106	102456825
54784	ALKBH4	7	102456238	102464863
6132	RPL8	8	144789765	144792587
222229	LRWD1	7	102464956	102473168
222235	FBXL13	7	102813230	103074843
5701	PSMC2	7	103344254	103369395
373856	USP41	22	20350578	20390758
1173	AP2M1	3	184174689	184184091
55324	ABCF3	3	184186023	184194012
93099	DMKN	19	35497220	35513658
622	BDH1	3	197509783	197573323
57125	PLXDC1	17	39063313	39154394
93210	PGAP3	17	39671122	39696797
2905	GRIN2C	17	74842023	74861504
2232	FDXR	17	74862497	74873031
5635	PRPSAP1	17	76309478	76384521
6427	SRSF2	17	76734115	76737333
55260	TMEM143	19	48332356	48364059
126133	ALDH16A1	19	49453225	49471050
25946	ZNF385A	12	54369133	54391298
400709	SIGLEC16	19	49969673	49975814
162417	NAGS	17	44004622	44009068

92591	ASB16	17	44170447	44179084
80174	DBF4B	17	44708608	44752264
91010	FMNL3	12	49636499	49708165
160622	GRASP	12	52006946	52015889
125950	RAVER1	19	10316212	10333638
147841	SPC24	19	11131520	11155808
221496	LEMD2	6	33771202	33789130
246	ALOX15	17	4630919	4642294
NA	ADCY10P1	6	41101022	41140835
400569	MED11	17	4731428	4733608
433	ASGR2	17	7101322	7115700
1973	EIF4A1	17	7572706	7579006
6154	RPL26	17	8377516	8383213
339123	JMJD8	16	681670	684528
51005	AMDHD2	16	2520357	2531422
146439	BICDL2	16	3027682	3036944
124222	PAQR4	16	2969270	2973484
115	ADCY9	16	3953387	4116442
220002	CYB561A3	11	61348754	61362283
80150	ASRGL1	11	62337448	62393412
10629	TAF6L	11	62771357	62787342
84447	SYVN1	11	65121780	65134533
4041	LRP5	11	68312591	68449275
10158	PDZK1IP1	1	47183582	47191044
65260	COA7	1	52684449	52698347
4116	MAGOH	1	53226900	53238518
26027	ACOT11	1	54542257	54639192
25973	PARS2	1	54756898	54764523
7809	BSND	1	54998933	55017172
8613	PLPP3	1	56494761	56645301
79707	NOL9	1	6521347	6554513
57643	ZSWIM5	1	45016399	45306209
10691	GMEB1	1	28668732	28719353
57190	SELENON	1	25800176	25818221
3716	JAK1	1	64833229	65067754
55225	RAVER2	1	64745095	64833232
11330	CTRC	1	15438442	15449242
116362	RBP7	1	9997206	10016021
284723	SLC25A34	1	15736258	15741392
9249	DHRS3	1	12567910	12618210
5928	RBBP4	1	32651142	32686211
127733	UBXN10	1	20186096	20196050
55450	CAMK2N1	1	20482391	20486210

83941	TM2D1	1	61681046	61725423
8880	FUBP1	1	77944055	77979110
91624	NEXN	1	77888513	77943895
113802	HENMT1	1	108648290	108661526
148423	C1orf52	1	85249953	85259672
2634	GBP2	1	89106132	89150456
115361	GBP4	1	89181144	89198942
10092	ARPC5	1	183620846	183635783
84838	ZNF496	1	247297412	247331846
93185	IGSF8	1	160091340	160098943
5824	PEX19	1	160276812	160286348
2215	FCGR3B	1	161623196	161631963
148304	C1orf74	1	209779208	209784559
28982	FLVCR1	1	212858275	212899363
467	ATF3	1	212565334	212620777
51278	IER5	1	181088700	181092900
148362	BROX	1	222712553	222735196
51205	ACP6	1	147629652	147670524
64216	TFB2M	1	246540561	246566261
163882	CNST	1	246566444	246668595
129285	PPP1R21	2	48440598	48515391
55220	KLHDC8A	1	205336065	205357090
NA	C1orf147	1	206491116	206497728
11009	IL24	1	206897443	206904139
9214	FCMR	1	206903317	206923247
824	CAPN2	1	223701593	223776018
5966	REL	2	60881521	60931612
150962	PUS10	2	60940222	61018259
5194	PEX13	2	61017225	61051990
84542	KIAA1841	2	61065871	61138034
130132	RFTN2	2	197568224	197676045
27185	DISC1	1	231626790	232041272
92291	CAPN13	2	30722771	30820542
51072	MEMO1	2	31865060	32011230
84661	DPY30	2	31867809	32039805
79568	MAIP1	2	199955317	200008540
NA	ALMS1P1	2	73641083	73697509
3020	H3-3A	1	226061851	226073212
56997	COQ8A	1	226897536	226987545
3625	INHBB	2	120346136	120351803
23248	RPRD2	1	150363091	150476566
1520	CTSS	1	150730188	150765792
149428	BNIPL	1	151036321	151047720

388695	LYSMD1	1	151159748	151165948
79005	SCNM1	1	151156664	151170296
2071	ERCC3	2	127257290	127294166
284996	RNF149	2	101271219	101308701
388962	BOLA3	2	74135400	74147912
10602	CDC42EP3	2	37641882	37738468
6282	S100A11	1	152032506	152047907
6280	S100A9	1	153357854	153361023
6283	S100A12	1	153373711	153375621
7039	TGFA	2	70447284	70554193
51023	MRPS18C	4	83455932	83469735
151556	GPR155	2	174431571	174487094
57326	PBXIP1	1	154944076	154956123
90780	PYGO2	1	154957026	154963853
127579	DCST2	1	155018520	155033781
149095	DCST1	1	155033824	155050930
1293	COL6A3	2	237324003	237414207
55356	SLC22A15	1	115976513	116070054
476	ATP1A1	1	116372668	116410261
317649	EIF4E3	3	71675414	71754773
92703	TMEM183A	1	203007374	203024848
3490	IGFBP7	4	57030773	57110385
80128	TRIM46	1	155173787	155184971
3577	CXCR1	2	218162841	218166962
10109	ARPC2	2	218217141	218254356
6746	SSR2	1	156009048	156020951
64320	RNF25	2	218663892	218672002
57650	CIP2A	3	108549864	108589644
8320	EOMES	3	27715949	27722711
57703	CWC22	2	179944876	180007297
64343	AZI2	3	28315003	28349050
50852	TRAT1	3	108822770	108855005
2199	FBLN2	3	13549125	13638422
23114	NFASC	1	204828651	205022822
151246	SGO2	2	200510008	200584096
5274	SERPINI1	3	167735243	167825568
23122	CLASP2	3	33496245	33718356
8802	SUCLG1	2	84423528	84460045
4332	MNDA	1	158831351	158849506
3428	IFI16	1	158999968	159055155
9447	AIM2	1	159062484	159147096
200916	RPL22L1	3	170864875	170870208
151742	PPM1L	3	160755602	161078902

151987	PPP4R2	3	72996803	73069198
131450	CD200R1	3	112921205	112975103
25871	NEPRO	3	113002444	113019861
23001	WDFY3	4	84669610	84966391
80145	THOC7	3	63833870	63863868
6314	ATXN7	3	63863867	64003462
9861	PSMD6	3	64010549	64024010
152926	PPM1K	4	88257620	88284769
8833	GMPS	3	155870650	155944020
57018	CCNL1	3	157146508	157160760
5806	PTX3	3	157436850	157443633
8820	HESX1	3	57197838	57227606
201895	SMIM14	4	39546336	39638902
11102	RPP14	3	58306247	58324695
57406	ABHD6	3	58237532	58295693
1776	DNASE1L3	3	58192257	58214697
132014	IL17RE	3	9902612	9916402
78987	CRELD1	3	9933816	9945406
26577	PCOLCE2	3	142815922	142889206
6374	CXCL5	4	73995642	73998677
5473	PPBP	4	73986439	73988190
2919	CXCL1	4	73869393	73871308
1359	CPA3	3	148865296	148897203
2992	GYG1	3	148991408	149031775
11073	TOPBP1	3	133598175	133662380
1230	CCR1	3	46201711	46208313
79442	LRRC2	3	46515385	46580099
54859	ELP6	3	47495640	47513712
151636	DTX3L	3	122564338	122575203
7707	ZNF148	3	125225561	125375354
349565	NMNAT3	3	139560180	139678017
55689	YEATS2	3	183697797	183812624
64769	MEAF6	1	37492575	37514774
79753	SNIP1	1	37534449	37554293
59343	SENP2	3	185582496	185633551
55764	IFT122	3	129440036	129520507
116832	RPL39L	3	187120948	187180908
51460	SFMBT1	3	52903572	53046750
26354	GNL3	3	52681156	52694497
55193	PBRM1	3	52545352	52685917
57654	UVSSA	4	1347208	1395989
7884	SLBP	4	1692731	1712344
131540	ZDHHC19	3	196197452	196211437

200931	SLC51A	3	196211487	196243178
6286	S100P	4	6693878	6697170
64789	EXO5	1	40508741	40516556
114625	ERMAP	1	42817122	42844991
23173	METAP1	4	98995659	99062809
3015	H2AZ1	4	99948086	99950355
56898	BDH2	4	103077592	103099870
10424	PGRMC2	4	128269237	128288829
993	CDC25A	3	48157146	48188417
820	CAMP	3	48223347	48225491
51385	ZNF589	3	48241100	48299253
5364	PLXNB1	3	48403854	48430086
79714	CCDC51	3	48432164	48440456
84126	ATRIP	3	48446710	48467645
51246	SHISA5	3	48467798	48504826
10252	SPRY1	4	123396795	123403760
327	APEH	3	49674014	49683971
84315	MON1A	3	49907160	49930173
23132	RAD54L2	3	51541144	51668667
1849	DUSP7	3	52048919	52056571
25886	POC1A	3	52075226	52154690
80335	WDR82	3	52254434	52288020
401152	C4orf3	4	119296419	119304445
3148	HMGB2	4	173331376	173334432
8819	SAP30	4	173369969	173377532
11341	SCRG1	4	173384701	173406380
4085	MAD2L1	4	120055623	120066858
26269	FBXO8	4	174236658	174283667
55314	TMEM144	4	158201604	158255416
51313	GASK1B	4	158124474	158173318
3600	IL15	4	141636583	141733987
27236	ARFIP1	4	152779937	152918463
10393	ANAPC10	4	144831908	145098541
4338	MOCS2	5	53095679	53110063
91942	NDUFAF2	5	60945205	61153026
25836	NIPBL	5	36876769	37066413
153733	CCDC112	5	115267190	115296693
133619	PRRC1	5	127517640	127555085
55255	WDR41	5	77425970	77620611
4724	NDUFS4	5	53560633	53683338
11082	ESM1	5	54977867	55022671
22836	RHOBTB3	5	95713522	95824383
836	CASP3	4	184627696	184649509

253260	RICTOR	5	38937920	39074399
1879	EBF1	5	158695916	159099916
57763	ANKRA2	5	73552190	73565667
134510	UBLCP1	5	159263290	159286036
7098	TLR3	4	186069155	186088073
84340	GFM2	5	74721206	74767147
133957	CCDC127	5	196868	218153
23305	ACSL6	5	131949973	132012243
134549	SHROOM1	5	132822141	132830898
27089	UQCRQ	5	132866630	132868847
116842	LEAP2	5	132873444	132875046
10559	SLC35A1	6	87470623	87512336
115004	CGAS	6	73413515	73452297
167838	TXLNB	6	139240061	139291998
10370	CITED2	6	139371807	139374648
153222	CREBRF	5	173056352	173139284
94081	SFXN1	5	175477062	175529742
57107	PDSS2	6	107152562	107459564
9263	STK17A	7	43582758	43650713
79685	SAP30L	5	154445997	154461053
10569	SLU7	5	160401641	160421711
6100	RP9	7	33094797	33109404
9232	PTTG1	5	160421855	160428739
819	CAMLG	5	134738548	134752157
285613	RELL2	5	141636950	141641064
8645	KCNK5	6	39188971	39229475
222223	KIAA1324L	7	86876906	87059654
25862	USP49	6	41789896	41895361
94120	SYTL3	6	158650014	158764876
26266	SLC13A4	7	135681237	135729258
25798	BRI3	7	98252379	98310441
1508	CTSB	8	11842524	11869448
5828	PEX2	8	76980258	77001044
90390	MED30	8	117520713	117540262
5260	PHKG1	7	56080283	56092996
23514	SPIDR	8	47260878	47736306
54919	DNAAF5	7	726699	786475
1669	DEFA4	8	6935820	6938306
734	OSGIN2	8	89901849	89927888
23353	SUN1	7	816615	896435
2852	GPER1	7	1082208	1093815
79778	MICALL2	7	1428465	1459470
1345	COX6C	8	99873200	99893707

	25879	DCAF13	8	103414714	103443453
	94241	TP53INP1	8	94925972	94949378
	55656	INTS8	8	94813311	94881746
	91147	TMEM67	8	93754844	93819234
	318	NUDT2	9	34329506	34343711
	11168	PSIP1	9	15464066	15510995
	55335	NIPSNAP3B	9	104764129	104777764
	19	ABCA1	9	104781006	104928155
	4783	NFIL3	9	91409045	91423832
	2395	FXN	9	69035563	69100178
	137835	TMEM71	8	132685007	132760712
	57094	CPA6	8	67422038	67746378
	138050	HGSNAT	8	43140464	43202855
	55582	KIF27	9	83834099	83921465
	203286	ANKS6	9	98731329	98796965
	2203	FBP1	9	94603133	94640249
	1536	CYBB	X	37780059	37813461
NA		NCF1C	7	75156639	75172044
	158401	SHOC1	9	111686173	111795008
	158405	KIAA1958	9	112486847	112669397
	65268	WNK2	9	93184916	93320572
	139324	HDX	X	84317874	84502479
	360	AQP3	9	33441156	33447596
	7415	VCP	9	35056064	35072627
	84720	PIGO	9	35088688	35096601
	9833	MELK	9	36572862	36677683
	220972	MARCHF8	10	45454585	45594906
	7253	TSHR	14	80954989	81146302
	1073	CFL2	14	34709113	34714823
	219654	ZCCHC24	10	79382325	79445624
	51084	CRYL1	13	20403666	20525873
	221035	REEP3	10	63521401	63625128
	221150	SKA3	13	21153595	21176552
	221154	MICU2	13	21492691	21604181
	122769	LRR1	14	49598761	49614672
	6166	RPL36AL	14	49618530	49620626
	11067	DEPP1	10	44970981	44978809
	9147	NEMF	14	49782083	49852821
	382	ARF6	14	49893082	49897054
	123016	TTC8	14	88824153	88881078
	122945	NOXRED1	14	77394021	77423517
	222389	BEND7	10	13438484	13528974
	509	ATP5F1C	10	7788147	7807815

8559	PRPF18		10	13586939	13630859
83860	TAF3		10	7818505	8016631
7417	VDAC2		10	75210154	75231448
84858	ZNF503		10	75397830	75401764
63877	FAM204A		10	118297925	118342328
10495	ENOX2	X		130623369	130903317
23203	PMPCA		9	136410641	136423761
10807	ENTR1		9	136401922	136410614
11092	SPACA9		9	132878027	132890201
8328	GFI1B		9	132944000	132991687
9188	DDX21		10	68956170	68985068
9790	BMS1		10	42782795	42834937
282974	STK32C		10	132207492	132331847
65991	FUNDC2	X		155025980	155060304
90809	PIP4P1		14	20457719	20461612
57447	NDRG2		14	21016763	21070872
51222	ZNF219		14	21090077	21104722
840	CASP7		10	113679162	113730907
55088	CCDC186		10	114120862	114174232
6297	SALL2		14	21521080	21537216
144455	E2F7		12	77021248	77065569
122961	ISCA2		14	74493756	74497106
3429	IFI27		14	94104836	94116698
79789	CLMN		14	95181940	95319908
221079	ARL5B		10	18659431	18681639
85459	CEP295		11	93661682	93730358
79101	TAF1D		11	93729948	93784391
27291	R3HCC1L		10	98134624	98244897
154810	AMOTL1		11	94706431	94876748
161742	SPRED1		15	38252836	38357249
83700	JAM3		11	134069071	134152001
112937	GLB1L3		11	134274245	134319564
84706	GPT2		16	46884362	46931289
51762	RAB8B		15	63189560	63267776
27079	RPUSD2		15	40569299	40574949
29117	BRD7		16	50313487	50368934
1152	CKB		14	103519667	103522833
8945	BTRC		10	101354033	101557321
9529	BAG5		14	103556544	103562831
8539	API5		11	43311963	43344529
79803	HPS6		10	102065349	102068036
9221	NOLC1		10	102152176	102163871
9318	COPS2		15	49106068	49155661

8879	SGPL1	10	70815948	70881184
10576	CCT2	12	69585426	69601570
25820	ARIH1	15	72474330	72602987
1353	COX11	17	54951902	54968703
252983	STXBP4	17	54968727	55173632
6609	SMPD1	11	6390440	6394998
322	APBB1	11	6395124	6419414
54765	TRIM44	11	35662775	35818007
3611	ILK	11	6603708	6610874
8642	DCHS1	11	6621330	6655809
8495	PPFIBP2	11	7513298	7657127
7275	TUB	11	8019244	8106243
122618	PLD4	14	104924713	104937761
9866	TRIM66	11	8612037	8682694
254225	RNF169	11	74748849	74842413
124359	CDYL2	16	80597907	80804598
55839	CENPN	16	81006498	81033114
56672	AKIP1	11	8911139	8920084
23300	ATMIN	16	81035842	81047350
440026	TMEM41B	11	9280654	9314636
123169	LEO1	15	51938025	51971778
5598	MAPK7	17	19377721	19383544
50810	HDGFL3	15	83112738	83207823
8509	NDST2	10	73801911	73811798
26253	CLEC4E	12	8533305	8540905
7551	ZNF3	7	100064033	100082548
338339	CLEC4D	12	8509475	8522366
7589	ZSCAN21	7	100049774	100065040
146227	BEAN1	16	66427295	66493529
90701	SEC11C	18	59139866	59158832
201161	CENPV	17	16342534	16353656
51647	CIAO2B	16	66932065	66934423
10904	BLCAP	20	37492472	37527931
80063	ATF7IP2	16	10326434	10483638
780776	TVP23A	16	10760919	10818794
9382	COG1	17	73192632	73208507
144100	PLEKHA7	11	16777297	17014414
80095	ZNF606	19	57977053	58003349
644353	ZCCHC18	X	104112131	104115846
567	B2M	15	44711487	44718877
162394	SLFN5	17	35243071	35273655
219539	YPEL4	11	57645087	57649944
5479	PPIB	15	64155817	64163022

84191	CIAO2A	15	64072565	64093857
63901	FAM111A	11	59142748	59155039
9768	PCLAF	15	64364304	64387687
124491	TMEM170A	16	75443054	75465497
348093	RBPMS2	15	64739891	64775589
348094	ANKDD1A	15	64911902	64958691
54386	TERF2IP	16	75647773	75761872
5347	PLK1	16	23677656	23690367
4665	NAB2	12	57089043	57095476
23339	VPS39	15	42158701	42208316
219988	PATL1	11	59636716	59669037
6809	STX3	11	59713456	59805882
54893	MTMR10	15	30938941	30991628
7529	YWHAB	20	44885702	44908532
84419	C15orf48	15	45430529	45448761
115752	DIS3L	15	66293217	66333898
4088	SMAD3	15	67063763	67195169
10982	MAPRE2	18	34976928	35143470
4141	MARS1	12	57475445	57517569
2923	PDIA3	15	43746410	43773278
4824	NKX3-1	8	23678697	23682938
150290	DUSP18	22	30652051	30667890
7008	TEF	22	41367333	41399326
5090	PBX3	9	125747345	125967377
2793	GNGT2	17	49202791	49210574
5245	PHB	17	49404049	49414905
6632	SNRPD1	18	21612314	21633524
162461	TMEM92	17	50271406	50281485
399665	FAM102A	9	127940582	127980989
80221	ACSF2	17	50426158	50474845
10999	SLC27A4	9	128340527	128361470
51148	CERCAM	9	128411751	128437351
57171	DOLPP1	9	129081111	129090438
10229	COQ7	16	19067614	19080095
1398	CRK	17	1420689	1463162
26263	FBXO22	15	75903876	75942511
64127	NOD2	16	50693588	50734041
125336	LOXHD1	18	46476972	46657220
257160	RNF214	11	117232625	117286454
10632	ATP5MG	11	118401606	118431496
915	CD3D	11	118338954	118342744
6786	STIM1	11	3854527	4093210
55128	TRIM68	11	4598672	4608231

64122	FN3K	17	82735615	82751196
126298	IRGQ	19	43584369	43596135
7769	ZNF226	19	44165073	44178381
9726	ZNF646	16	31074422	31084196
762	CA4	17	60149942	60170899
55181	SMG8	17	59209400	59215247
4218	RAB8A	19	16111889	16134234
2879	GPX4	19	1103926	1106791
126306	JSRP1	19	2252252	2269759
55295	KLHL26	19	18637025	18671721
81620	CDT1	16	88803789	88809258
124045	SPATA33	16	89657802	89671272
6137	RPL13	16	89560657	89566828
147015	DHRS13	17	28897781	28903079
8085	KMT2D	12	49018975	49059774
84790	TUBA1C	12	49188736	49274600
55762	ZNF701	19	52555457	52587174
53916	RAB4B	19	40778216	40796942
29785	CYP2S1	19	41193210	41207539
558	AXL	19	41219223	41261766
84807	NFKBID	19	35887653	35902303
3903	LAIR1	19	54351384	54370558
116115	ZNF526	19	42220312	42228201
83696	TRAPPC9	8	139727725	140458579
10653	SPINT2	19	38244035	38292615
1938	EEF2	19	3976056	3985463
126259	TMIGD2	19	4292232	4302431
84717	HDGFL2	19	4472297	4502208
729359	PLIN4	19	4502180	4518465
10501	SEMA6B	19	4542593	4559684
55720	TSR1	17	2322396	2336657
374875	HSD11B1L	19	5680604	5688523
124936	CYB5D2	17	4143168	4187310
3816	KLK1	19	50819146	50823787
3848	KRT1	12	52674736	52680407
84926	SPRYD3	12	53064316	53079404
10263	CDK2AP2	11	67506497	67508649
7001	PRDX2	19	12796820	12801800
7775	ZNF232	17	5105541	5123116
11314	CD300A	17	74466399	74484794
3396	MRPL58	17	75012670	75021261
10476	ATP5PD	17	75038863	75046985
2125	EVPL	17	76004502	76027306

6730	SRP68	17	76038775	76072517
7083	TK1	17	78174091	78187233
84514	GHDC	17	42188799	42194532
83986	FAM234A	16	234521	272183
1775	DNASE1L2	16	2235816	2238711
146434	ZNF597	16	3432414	3443504
1642	DDB1	11	61299451	61342596
55048	VPS37C	11	61130257	61161615
220001	VWCE	11	61258286	61295316
7439	BEST1	11	61950063	61965515
26580	BSCL2	11	62690275	62709845
6520	SLC3A2	11	62856102	62888875
117245	PLAAT5	11	63461404	63491194
89849	ATG16L2	11	72814406	72843674
26005	C2CD3	11	74012714	74171210
9881	TRANK1	3	36826820	36945098
199223	TTC21A	3	39107704	39138903
8772	FADD	11	70203296	70207390
4054	LTBP3	11	65538805	65558930
10004	NAALADL1	11	65044818	65058553
116071	BATF2	11	64987945	64997018
7536	SF1	11	64764606	64778786
283234	CCDC88B	11	64340204	64357534
55872	PBK	8	27809624	27838082
84309	NUDT16L1	16	4693694	4695859
5867	RAB4A	1	229271062	229305894
93487	MAPK1IP1L	14	55051647	55070194
3516	RBPJ	4	26163455	26435131
92181	UBTD2	5	172209646	172283764
28511	NKIRAS2	17	42011382	42025644
7266	DNAJC7	17	41976421	42021376
359948	IRF2BP2	1	234604269	234609525
64943	NT5DC2	3	52524385	52535054
388753	COA6	1	234373456	234385080
3800	KIF5C	2	148875227	149026759
648	BMI1	10	22321099	22331484
57215	THAP11	16	67842320	67844195
27249	MMADHC	2	149569637	149587778
200845	KCTD6	3	58492096	58502360
136647	MPLKIP	7	40126027	40134622
8309	ACOX2	3	58505136	58537283
3660	IRF2	4	184387729	184474550
378	ARF4	3	57571363	57598220

84879	MFSD2A	1	39955112	39969968
6890	TAP1	6	32845209	32853978
84289	ING5	2	241687085	241729478
197259	MLKL	16	74671855	74700960
399	RHOH	4	40191053	40246967
91949	COG7	16	23388493	23453189
51362	CDC40	6	110180141	110254275
10963	STIP1	11	64185272	64204543
11031	RAB31	18	9708275	9862551
11273	ATXN2L	16	28823035	28837237
2237	FEN1	11	61792911	61797238
124790	HEXIM2	17	45160700	45170040
2675	GFRA2	8	21690398	21812357
3622	ING2	4	183505058	183512429
6575	SLC20A2	8	42416475	42541926
79089	TMUB2	17	44186970	44191929
6774	STAT3	17	42313324	42388568
90204	ZSWIM1	20	45881227	45885266
8754	ADAM9	8	38996869	39105261
8313	AXIN2	17	65528563	65561648
4725	NDUFS5	1	39026318	39034636
90075	ZNF30	19	34926903	34945170
753	LDLRAD4	18	13217498	13652755
3575	IL7R	5	35852695	35879603
29100	TMEM208	16	67227103	67229278
10768	AHCYL1	1	109984765	110023742
54910	SEMA4C	2	96859718	96870757
26505	CNNM3	2	96816245	96835382
2948	GSTM4	1	109656099	109674836
54790	TET2	4	105145875	105279816
79867	TCTN2	12	123671113	123708399
9677	PPIP5K1	15	43533462	43590253
116236	ABHD15	17	29560547	29567037
9925	ZBTB5	9	37438102	37465450
9836	LCMT2	15	43323649	43330582
27065	NSG1	4	4348140	4419058
166793	ZBTB49	4	4290251	4321786
85476	GFM1	3	158644527	158695581
55308	DDX19A	16	70346861	70373383
388969	C2orf68	2	85605254	85612066
129303	TMEM150A	2	85598547	85603196
51255	RNF181	2	85595725	85597708
10791	VAMP5	2	85584431	85593406

57507	ZNF608	5	124636913	124748807
3635	INPP5D	2	233059967	233207903
56947	MFF	2	227325151	227357836
3965	LGALS9	17	27629798	27649560
27036	SIGLEC7	19	51142299	51153526
54951	COMMD8	4	47450787	47463702
521	ATP5ME	4	672436	674330
7386	UQCRFS1	19	29205320	29213151
84179	SLC49A3	4	681829	689441
1285	COL4A3	2	227164624	227314792
5604	MAP2K1	15	66386837	66491544
3187	HNRNPH1	5	179614178	179634784
113189	CHST14	15	40470984	40473158
84632	AFAP1L2	10	114294824	114404756
6940	ZNF354A	5	178711512	178730659
23099	ZBTB43	9	126805006	126838210
255738	PCSK9	1	55039447	55064852
23214	XPO6	16	28097979	28211920
27301	APEX2	X	55000363	55009057
10636	RGS14	5	177357924	177372596
10960	LMAN2	5	177331567	177351668
148823	GCSAML	1	247507058	247577690
53917	RAB24	5	177301198	177303744
27166	PRELID1	5	177303799	177306949
7059	THBS3	1	155195588	155209051
11238	CA5B	X	15688830	15788411
3627	CXCL10	4	76021118	76023497
51068	NMD3	3	161104696	161253532
8706	B3GALNT1	3	161083883	161105411
114787	GPRIN1	5	176595802	176610156
65008	MRPL1	4	77862830	77952785
64805	P2RY12	3	151336843	151384753
23251	MINAR1	15	79432336	79472304
8738	CRADD	12	93677375	93894840
25942	SIN3A	15	75369379	75455842
6036	RNASE2	14	20955487	20956436
6037	RNASE3	14	20891399	20892348
5780	PTPN9	15	75463251	75579315
6039	RNASE6	14	20781268	20782467
3576	CXCL8	4	73740541	73743716
6335	SCN9A	2	166195185	166376001
6383	SDC2	8	96493813	96611790
1043	CD52	1	26317958	26320523

1880	GPR183	13	99294539	99307399
9839	ZEB2	2	144364364	144521057
5093	PCBP1	2	70087477	70089203
3094	HINT1	5	131159027	131171735
9022	CLIC3	9	136994608	136996568
27247	NFU1	2	69396113	69437628
150468	CKAP2L	2	112736349	112764664
83640	RAMAC	15	82986210	82991057
23119	HIC2	22	21417371	21451463
7798	LUZP1	1	23084023	23177808
284004	HEXD	17	82418318	82442645
699	BUB1	2	110637528	110678063
83985	SPNS1	16	28974221	28984548
7555	CNBP	3	129167827	129183922
4493	MT1E	16	56625475	56627112
2873	GPS1	17	82050691	82057470
7360	UGP2	2	63840940	63891562
3185	HNRNPF	10	43385617	43409166
55454	CSGALNACT2	10	43138445	43185302
57099	AVEN	15	33866227	34039204
81844	TRIM56	7	101085481	101097967
9185	REPS2	X	16946658	17153272
3684	ITGAM	16	31259967	31332892
8460	TPST1	7	66205317	66420543
163590	TOR1AIP2	1	179839967	179877803
8019	BRD3	9	134030305	134068535
92595	ZNF764	16	30553764	30558498
65988	ZNF747	16	30530367	30535347
79724	ZNF768	16	30524004	30526821
83756	TAS1R3	1	1331280	1335314
10746	MAP3K2	2	127298730	127388465
126917	IFFO2	1	18904280	18956676
1107	CHD3	17	7884796	7912760
214	ALCAM	3	105366909	105576900
7532	YWHAG	7	76326799	76358991
9196	KCNAB3	17	7921859	7929803
285596	FAM153A	5	177707981	177783398
375484	SIMC1	5	176238367	176345991
2848	GPR25	1	200872981	200874178
7324	UBE2E1	3	23805955	23891640
220988	HNRNPA3	2	177212563	177223958
23235	SIK2	11	111602449	111730855
9121	SLC16A5	17	75087727	75106162

56985	ADPRM	17	10697594	10711558
2172	FABP6	5	160187367	160238735
114825	PWWP2A	5	160061801	160119450
7988	ZNF212	7	149239651	149255606
2720	GLB1	3	32996609	33097202
23587	ELP5	17	7251416	7259940
152189	CMTM8	3	32238679	32370321
11337	GABARAP	17	7240008	7242449
9482	STX8	17	9250471	9576591
983	CDK1	10	60778331	60794852
10678	B3GNT2	2	62196115	62224731
2353	FOS	14	75278826	75282230
10972	TMED10	14	75131469	75176612
10446	LRRN2	1	204617170	204685738
55890	GPRC5C	17	74424851	74451653
196410	METTL7B	12	55681678	55684611
160518	DENND5B	12	31382226	31591136
79697	RIOX1	14	73490933	73493394
202051	SPATA24	5	139396563	139404088
51237	MZB1	5	139387467	139390081
9963	SLC23A1	5	139367196	139384553
4862	NPAS2	2	100820139	100996829
140807	KRT72	12	52585589	52601538
53343	NUDT9	4	87422573	87459455
345275	HSD17B13	4	87303789	87322886
5036	PA2G4	12	56104537	56113910
79071	ELOVL6	4	110045846	110199199
5209	PFKFB3	10	6144934	6254644
23204	ARL6IP1	16	18791669	18801572
1000	CDH2	18	27950966	28177130
133418	EMB	5	50396192	50443248
6773	STAT2	12	56341597	56360167
134492	NUDCD2	5	163446526	163460102
26145	IRF2BP1	19	45883608	45886141
121355	GTSF1	12	54455950	54473602
7564	ZNF16	8	144930358	144950888
162979	ZNF296	19	45071500	45076587
5429	POLH	6	43576185	43620523
3799	KIF5B	10	32009015	32056425
11214	AKAP13	15	85380571	85749358
94031	HTRA3	4	8269754	8307098
84669	USP32	17	60177327	60422470
2850	GPR27	3	71753855	71756496

NA		4	6663396	6676755
51499	TRIAP1	12	120443964	120446384
27258	LSM3	3	14178817	14201122
6203	RPS9	19	54200809	54249003
79042	TSEN34	19	54189938	54194536
2941	GSTA4	6	52977948	52995304
85315	PAQR8	6	52361421	52407777
NA	TPT1-AS1	13	45341345	45417975
26115	TANC2	17	63009556	63427699
120526	DNAJC24	11	31369840	31432835
90338	ZNF160	19	53066606	53103436
112464	CAVIN3	11	6318946	6320532
1084	CEACAM3	19	41796587	41811554
1901	S1PR1	1	101236865	101243713
5569	PKIA	8	78516340	78605267
2358	FPR2	19	51752026	51770531
2357	FPR1	19	51745172	51804110
53838	C11orf24	11	68261338	68272001
NA	SIGLEC17P	19	51167328	51173524
55669	MFN1	3	179347709	179394936
155066	ATP6V0E2	7	149872968	149891204
5581	PRKCE	2	45651345	46187990
10474	TADA3	3	9779860	9793011
55657	ZNF692	1	248850006	248859144
64080	RBKS	2	27781379	27890681
84233	TMEM126A	11	85647967	85656547
55863	TMEM126B	11	85628573	85636539
219738	FAM241B	10	69630247	69633596
79801	SHCBP1	16	46578591	46621379
4884	NPTX1	17	80466834	80477843
90594	ZNF439	19	11848726	11883750
51013	EXOSC1	10	97435909	97446017
55636	CHD7	8	60678740	60868028
157570	ESCO2	8	27771949	27812640
64979	MRPL36	5	1798385	1801366
9	NAT1	8	18170477	18223689
171023	ASXL1	20	32358330	32439319
24149	ZNF318	6	43307134	43369647
23566	LPAR3	1	84811602	84893206
6902	TBCA	5	77691166	77868780
5356	PLRG1	4	154535005	154550400
201514	ZNF584	19	58401504	58418327
22883	CLSTN1	1	9728926	9823984

51523	CXXC5		5	139647299	139683882
80176	SPSB1		1	9292894	9369532
284307	ZIK1		19	57578456	57593890
27197	GPR82	X		41724181	41730130
2857	GPR34	X		41688973	41697275
10287	RGS19		20	64073181	64079988
6919	TCEA2		20	64049836	64072347
203859	ANO5		11	21799934	22283357
8841	HDAC3		5	141620876	141636849
2628	GATM		15	45361124	45402327
92342	METTL18		1	169792529	169794963
163255	ZNF540		19	37551406	37614179
148268	ZNF570		19	37467585	37488652
55138	FAM90A1		12	8221260	8227618
6241	RRM2		2	10120698	10211725
51112	TRAPPC12		2	3379675	3485094
6227	RPS21		20	62387103	62388520
719	C3AR1		12	8056844	8066359
5728	PTEN		10	87863625	87971930
6201	RPS7		2	3575260	3580920
246243	RNASEH1		2	3541430	3558333
5621	PRNP		20	4686350	4701590
83660	TLN2		15	62390526	62844631
51030	TVP23B		17	18781111	18806714
653464	SRGAP2C		1	121184811	121392874
91647	ATPAF2		17	17977409	18039209
83450	DRC3		17	17972813	18016889
115196	ZNF554		19	2819868	2836735
83452	RAB33B		4	139453232	139476609
5859	QARS1		3	49095932	49105130
9451	EIF2AK3		2	88556741	88627464
51315	KRCC1		2	88027205	88064252
54205	CYCS		7	25118656	25125260
926	CD8B		2	86815339	86861924
55106	SLFN12		17	35411060	35433283
257019	FRMD3		9	83242990	83538546
79736	TEFM		17	30897336	30906238
28998	MRPL13		8	120380761	120445402
3669	ISG20		15	88636153	88656483
10663	CXCR6		3	45940933	45948351
566	AZU1		19	825097	832018
64581	CLEC7A		12	10116777	10130258
1798	DPAGT1		11	119096503	119108331

25988	HINFP		11	119121580	119136059
55352	COPRS		17	31851871	31859291
199857	ALG14		1	94974405	95072951
8801	SUCLG2		3	67360460	67654614
10231	RCAN2		6	46220736	46491972
3603	IL16		15	81159575	81314058
171024	SYNPO2		4	118850688	119061247
221421	RSPH9		6	43645036	43672600
150678	COPS9		2	240126563	240136807
54676	GTPBP2		6	43605316	43629264
55277	FGGY		1	59296638	59810647
4299	AFF1		4	86935002	87141054
57571	CARNS1		11	67414968	67425607
5499	PPP1CA		11	67398183	67421183
3054	HCFC1	X		153947557	153971818
118487	CHCHD1		10	73782047	73783652
30008	EFEMP2		11	65866441	65873592
253725	WASHC2C		10	45727200	45792961
64393	ZMAT3		3	178960121	179072215
91607	SLFN11		17	35350305	35373701
55222	LRRC20		10	70298970	70382650
80198	MUS81		11	65857126	65867653
79612	NAA16		13	41311267	41377030
90288	EFCAB12		3	129401321	129428651
80011	PSME3IP1		16	57152466	57186116
339122	RAB43		3	129087569	129122801
55871	CBWD1		9	121038	179147
326624	RAB37		17	74670578	74747335
167227	DCP2		5	112976702	113022195
254122	SNX32		11	65833834	65856896
6169	RPL38		17	74203582	74210655
5916	RARG		12	53210567	53232980
283848	CES4A		16	66988589	67009758
6670	SP3		2	173880850	173965702
254042	METAP1D		2	171999943	172082430
51162	EGFL7		9	136658856	136672678
55191	NADSYN1		11	71453109	71524107
1717	DHCR7		11	71428193	71452868
84153	RNASEH2C		11	65714005	65720818
338692	ANKRD13D		11	67289300	67302485
4615	MYD88		3	38138478	38143022
23133	PHF8	X		53936676	54048958
253558	LCLAT1		2	30447226	30644225

NA	MIR4435-2H(		2	111006015	111523376
NA	UNC93B3		3	75619506	75623849
79877	DCAKD		17	45023340	45061109
5970	RELA		11	65653597	65663090
132884	EVC2		4	5542772	5709548
55731	FAM222B		17	28755978	28855232
10855	HPSE		4	83292461	83335153
27235	COQ2		4	83261536	83284914
3310	HSPA6		1	161524540	161526894
64318	NOC3L		10	94333226	94362959
150684	COMMD1		2	61888724	62147247
4580	MTX1		1	155208699	155213824
54625	PARP14		3	122680839	122730840
10800	CYSLTR1	X		78271468	78327691
126868	MAB21L3		1	116111755	116135240
2745	GLRX		5	95751319	95822726
2804	GOLGB1		3	121663199	121749767
144195	SLC2A14		12	7812512	7891148
8658	TNKS		8	9555912	9782346
79660	PPP1R3B		8	9136255	9151574
10221	TRIB1		8	125430358	125438403
10089	KCNK7		11	65592836	65595996
713	C1QB		1	22652762	22661637
712	C1QA		1	22636628	22639678
4973	OLR1		12	10158301	10172138
1605	DAG1		3	49468703	49535618
64801	ARV1		1	230978981	231000733
51126	NAA20		20	20017310	20033655
440574	MICOS10		1	19484403	19629821
254102	EHBP1L1		11	65576046	65592650
79102	RNF26		11	119334527	119337309
26472	PPP1R14B		11	64244479	64246943
10534	ZNRD2		11	65570460	65573942
6599	SMARCC1		3	47585269	47782106
7423	VEGFB		11	64234538	64238793
8793	TNFRSF10D		8	23135588	23164027
4485	MST1		3	49683947	49689501
90441	ZNF622		5	16451519	16465800
257364	SNX33		15	75647912	75662301
64859	NABP1		2	191678068	191741097
1106	CHD2		15	92900189	93027996
29903	CCDC106		19	55641062	55653161
51134	CEP83		12	94306449	94459988

27284	SULT1B1		4	69721167	69787961
5091	PC		11	66848417	66958386
64802	NMNAT1		1	9943428	9985501
78999	LRFN4		11	66856647	66860475
9986	RCE1		11	66842835	66846552
7388	UQCRH		1	46303698	46316776
1964	EIF1AX	X		20124525	20141838
256643	BCLAF3	X		19912860	19970298
NA			11	65455258	65466720
3267	AGFG1		2	227472152	227561214
924	CD7		17	82314868	82317608
1267	CNP		17	41966763	41977740
3728	JUP		17	41754604	41786931
10209	EIF1		17	41688885	41692668
284131	ENDOV		17	80415165	80438086
57674	RNF213		17	80260866	80398786
220359	TIGD3		11	65354751	65357613
10276	NET1		10	5412557	5459056
23333	DPY19L1		7	34928876	35038271
26996	GPR160		3	170037995	170085392
27333	GOLIM4		3	168008689	168095924
83759	RBM4B		11	66664998	66677887
84833	ATP5MD		10	103389041	103396492
353189	SLCO4C1		5	102233986	102296284
5936	RBM4		11	66638667	66668374
9973	CCS		11	66593153	66606019
375387	NRROS		3	196639694	196662004
2787	GNG5		1	84498325	84506581
8722	CTSF		11	66563464	66568841
253827	MSRB3		12	65278643	65491430
7096	TLR1		4	38790677	38856817
10333	TLR6		4	38823715	38856817
54931	TRMT10C		3	101561868	101566446
6403	SELP		1	169588849	169630193
54872	PIGG		4	499210	540200
10594	PRPF8		17	1650629	1684867
23051	ZHX3		20	41178448	41317672
23612	PHLDA3		1	201464278	201469237
201232	SLC16A13		17	7036015	7040117
9156	EXO1		1	241847967	241895148
488	ATP2A2		12	110280756	110351093
55055	ZWILCH		15	66504959	66550130
400073	C12orf76		12	110027028	110073634

400986	ANKRD36C	2	95836919	95991831
246330	PELI3	11	66466327	66477337
388730	TMEM81	1	205083129	205084460
65003	MRPL11	11	66435075	66466738
3177	SLC29A2	11	66362521	66372214
11041	B4GAT1	11	66345374	66347629
153339	TMEM167A	5	83052846	83077863
3952	LEP	7	128241278	128257629
55196	RESF1	12	31959370	31993107
51574	LARP7	4	112637107	112657592
25855	BRMS1	11	66337333	66345125
132720	FAM241A	4	112145454	112195256
9610	RIN1	11	66330241	66336840
152815	THAP6	4	75513946	75550473
9662	CEP135	4	55948871	56033361
57124	CD248	11	66314494	66317044
2015	ADGRE1	19	6887566	6940459
201626	PDE12	3	57556274	57566848
254263	CNIH2	11	66278175	66285301
171389	NLRP6	11	278365	285359
51319	RSRC1	3	158105855	158545730
9934	P2RY14	3	151212117	151278542
26259	FBXW8	12	116910950	117031148
6580	SLC22A1	6	160121815	160158718
9435	CHST2	3	143119771	143124014
NA	SNHG29	17	16438767	16478678
11065	UBE2C	20	45812576	45816957
80124	VCPIP1	8	66628487	66667231
201158	TVP23C	17	15502264	15563595
56945	MRPS22	3	139005806	139357223
55690	PACS1	11	66070272	66244744
65108	MARCKSL1	1	32333839	32336233
80851	SH3BP5L	1	248810446	248825915
1649	DDIT3	12	57516588	57521737
5095	PCCA	13	100089015	100530437
79797	ZNF408	11	46701030	46705912
9793	CKAP5	11	46743048	46846308
392	ARHGAP1	11	46677080	46700619
23015	GOLGA8A	15	34379068	34437466
22845	DOLK	9	128945530	128947603
9134	CCNE2	8	94879770	94896678
85007	PHYKPL	5	178208471	178232802
27257	LSM1	8	38163335	38176730

56674	TMEM9B	11	8947202	8965011
56675	NRIP3	11	8980576	9004049
84285	EIF1AD	11	65996545	66002176
4087	SMAD2	18	47808957	47931146
8665	EIF3F	11	7970251	8001862
79772	MCTP1	5	94703741	95284575
23529	CLCF1	11	67364168	67374177
143630	UBQLNL	11	5514393	5518431
387787	LIPT2	11	74490519	74493724
10008	KCNE3	11	74454841	74467729
10589	DRAP1	11	65919274	65921563
79836	LONRF3	X	118974614	119018355
7352	UCP3	11	74000277	74009085
7351	UCP2	11	73974672	73983246
83638	C11orf68	11	65916810	65919062
80227	PAAF1	11	73876699	73931114
51642	MRPL48	11	73787872	73865133
54968	TMEM70	8	73972437	73982783
6199	RPS6KB2	11	67428460	67435401
116028	RMI2	16	11249619	11381662
146691	TOM1L2	17	17843511	17972422
126695	KDF1	1	26949562	26960468
146712	B3GNTL1	17	82942155	83051810
22877	MLXIP	12	122078756	122147344
55508	SLC35E3	12	68746125	68793964
2810	SFN	1	26863149	26864456
NA	OR52B3P	11	4378282	4379209
23399	CTDNEP1	17	7243591	7252491
202309	GAPT	5	58491435	58497090
10458	BAIAP2	17	81035122	81117432
54084	TSPEAR	21	44497893	44711572
379	ARL4D	17	43398993	43401137
57633	LRRN1	3	3799431	3849834
63893	UBE2O	17	76389456	76453152
84747	UNC119B	12	120710458	120723640
126792	B3GALT6	1	1232237	1235041
79898	ZNF613	19	51927147	51948759
26471	NUPR1	16	28532708	28539008
257101	ZNF683	1	26361634	26374522
55057	CRYBG2	1	26321698	26360080
284001	CCDC57	17	82101460	82212830
124535	HSF5	17	58420167	58488408
8877	SPHK1	17	76376584	76387860

664	BNIP3	10	131966455	131982013
114926	SMIM19	8	42541155	42555193
342908	ZNF404	19	43872365	43901385
25914	RTTN	18	70003031	70205726
414149	ACBD7	10	15075475	15088776
339487	ZBTB8OS	1	32600172	32650903
7694	ZNF135	19	58059239	58086310
6051	RNPEP	1	201982372	202006147
84277	DNAJC30	7	73680918	73683453
161176	SYNE3	14	95407266	95475836
1196	CLK2	1	155262868	155278491
254531	LPCAT4	15	34358633	34367196
284346	ZNF575	19	43525497	43536130
112869	SGF29	16	28553915	28591790
11145	PLAAT3	11	63573195	63616883
148252	DIRAS1	19	2714567	2721372
653583	PHLDB3	19	43474954	43504935
124817	CNTD1	17	42798800	42811587
84002	B3GNT5	3	183253253	183298504
51115	RMDN1	8	86468257	86514357
51320	MEX3C	18	51174550	51218304
NA	C20orf197	20	60055853	60072956
NA	SCAND2P	15	84631451	84647478
666	BOK	2	241551424	241574131
374819	LRRC37A3	17	64854312	64919480
55884	WSB2	12	118032694	118062430
7298	TYMS	18	657653	673578
170082	TCEANC	X	13653189	13681964
9240	PNMA1	14	73711783	73714384
733	C8G	9	136945185	136946975
51301	GCNT4	5	75025346	75052558
51078	THAP4	2	241584405	241637158
23403	FBXO46	19	45710629	45730896
54503	ZDHHC13	11	19117099	19176422
84942	WDR73	15	84639285	84654343
NA	MRVI1-AS1	11	10541258	10599936
80324	PUS1	12	131929200	131945896
162517	FBXO39	17	6776215	6797101
3737	KCNA2	1	110519837	110631474
NA	C8orf31	8	143039209	143060684
NA	DLGAP1-AS1	18	3593732	3598363
29928	TIMM22	17	997129	1003671
3090	HIC1	17	2054154	2063241

64110	MAGEF1	3	184710364	184712064
NA	NINJ2-AS1	12	630858	664196
219285	SAMD9L	7	93130056	93148385
NA	ZFAS1	20	49278178	49299600
375316	RBM44	2	237798389	237842808
475	ATOX1	5	151742316	151772532
9332	CD163	12	7470813	7503893
126069	ZNF491	19	11797667	11809622
6181	RPLP2	11	809965	812880
83903	HASPIN	17	3723903	3726699
3725	JUN	1	58780791	58784047
23283	CSTF2T	10	51695486	51699595
2629	GBA	1	155234452	155244699
23765	IL17RA	22	17084954	17115694
57085	AGTRAP	1	11736084	11754802
283229	CRACR2B	11	826144	831991
6666	SOX12	20	325595	330224
10949	HNRNPA0	5	137745651	137754363
253639	ZNF620	3	40477131	40518736
374969	SVBP	1	42807052	42817397
285267	ZNF619	3	40477113	40491053
1176	AP3S1	5	115841592	115914081
7334	UBE2N	12	93405673	93441947
158056	MAMDC4	9	136850943	136860799
51272	BET1L	11	167784	207428
55272	IMP3	15	75639085	75648706
440836	ODF3B	22	50529710	50532580
3615	IMPDH2	3	49024325	49029408
259173	ALS2CL	3	46668995	46693704
9659	PDE4DIP	1	148808181	149048286
1662	DDX10	11	108665069	108940927
167465	ZNF366	5	72439903	72507410
5339	PLEC	8	143915147	143976734
146547	PRSS36	16	31138926	31150083
11180	WDR6	3	49007062	49015953
90637	ZFAND2A	7	1152071	1160759
100653515	CEP295NL	17	78870910	78903217
221294	NT5DC1	6	116100851	116249497
2731	GLDC	9	6532467	6645729
84987	COX14	12	50112082	50120457
NA	RPL10P16	19	12643275	12643919
767	CA8	8	60185412	60281400
4094	MAF	16	79585843	79600737

	84875	PARP10	8	143977153	144012772
	63899	NSUN3	3	94062980	94131832
	2814	GP5	3	194394821	194398354
	151176	ERFE	2	238158970	238168900
	4351	MPI	15	74890005	74902219
NA		ADORA2A-AS	22	24429206	24495074
	26873	OPLAH	8	144051266	144063965
	6879	TAF7	5	141260225	141320784
	5198	PFAS	17	8247618	8270491
	79415	CYBC1	17	82442586	82450829
	399668	SMIM10L2A X		135421944	135428075
	51341	ZBTB7A	19	4043303	4066899
	127254	ERICH3	1	74568117	74673792
	80010	RMI1	9	83980711	84004074
	55030	FBXO34	14	55271421	55361918
	27335	EIF3K	19	38619082	38636955
	9212	AURKB	17	8204733	8210600
	93621	MRFAP1	4	6640091	6642745
	285237	C3orf38	3	88149959	88168729
	23212	RRS1	8	66429014	66430733
	283849	EXOC3L1	16	67184379	67190185
NA		C9orf106	9	129321016	129324905
	374470	C12orf42	12	103237591	103496010
	5187	PER1	17	8140472	8156506
	2193	FARSA	19	12922479	12934037
	168537	GIMAP7	7	150514872	150521073
	285343	TCAIM	3	44338119	44409451
	144348	ZNF664	12	123971845	124015439
	811	CALR	19	12938578	12944489
	9500	MAGED1 X		51803007	51902354
	5886	RAD23A	19	12945855	12953642
	90480	GADD45GIP1	19	12953119	12957223
	79730	NSUN7	4	40749925	40811184
	54734	RAB39A	11	107928448	107963482
	1198	CLK3	15	74598500	74645414
	2624	GATA2	3	128479427	128493201
	255520	ELMOD2	4	140524168	140553770
	1960	EGR3	8	22687659	22693480
NA		LINC00174	7	66376044	66493566
	50628	GEMIN4	17	744421	753999
	85474	LBX2	2	74497517	74503316
	79571	GCC1	7	127580628	127593611
	132228	LSMEM2	3	50279087	50288114

161882	ZFPM1		16	88453280	88537031
247	ALOX15B		17	8039034	8049134
201164	PLD6		17	17200995	17206333
100128927	ZBTB42		14	104800596	104804712
2205	FCER1A		1	159289714	159308224
340390	WDR97		8	144107726	144118328
9582	APOBEC3B		22	38982347	38992804
NA	PCBP1-AS1		2	69962263	70103220
91663	MYADM		19	53864763	53876435
9792	SERTAD2		2	64631621	64751005
NA	PIK3CD-AS1		1	9652610	9654586
9495	AKAP5		14	64465499	64474503
126326	GIPC3		19	3585478	3593541
284023	RNF227		17	7913324	7916276
163732	CITED4		1	40861054	40862363
154664	ABCA13		7	48171458	48647497
23042	PDXDC1		16	14974591	15139339
7710	ZNF154		19	57697367	57709194
22928	SEPHS2		16	30443631	30445874
84922	FIZ1		19	55591376	55601970
10194	TSHZ1		18	75210755	75289950
122809	SOCS4		14	55027230	55049489
197407	ZNF48		16	30378106	30400108
284417	TMEM150B		19	55312801	55334048
1731	SEPTIN1		16	30378133	30395991
54952	TRNAU1AP		1	28553085	28578545
122011	CSNK1A1L		13	37103259	37105664
66004	LYNX1		8	142771197	142777810
9282	MED14	X		40648305	40735858
223082	ZNRF2		7	30284597	30367689
285848	PNPLA1		6	36243203	36312229
23060	ZNF609		15	64460742	64686068
NA	ATP5F1EP2		13	27945260	27945415
90411	MCFD2		2	46901870	46941855
283254	HARBI1		11	46602861	46617909
54494	C11orf71		11	114391443	114400511
3753	KCNE1		21	34446688	34512210
168620	BHLHA15		7	98211427	98212979
401563	C9orf139		9	137027464	137037957
2529	FUT7		9	137030174	137032088
8334	H2AC6		6	26124145	26139116
5551	PRF1		10	70597348	70602759
55432	YOD1		1	207043849	207052980

NA	LINC02363		4	184334292	184353977
80045	GPR157		1	9100305	9129102
166012	CHST13		3	126524155	126543291
NA	WDFY3-AS2		4	84965534	85011277
253832	ZDHHC20		13	21372573	21459370
389840	MAP3K15	X		19360056	19515261
5464	PPA1		10	70202835	70233911
NA	CSNK1G2-AS		19	1952531	1954586
6748	SSR4	X		153793516	153798499
126375	ZNF792		19	34956354	34964229
404093	CUEDC1		17	57861243	57955412
23510	KCTD2		17	75032575	75065889
23760	PITPNB		22	27851669	27920134
64928	MRPL14		6	44113451	44127452
284406	ZFP82		19	36383120	36418644
286006	LSMEM1		7	112480853	112491062
1728	NQO1		16	69706996	69726668
64782	AEN		15	88621337	88632281
79147	FKRP		19	46746046	46776988
25994	HIGD1A		3	42784298	42804531
390072	OR52N4		11	5754243	5755905
79813	EHMT1		9	137618992	137870016
2149	F2R		5	76716126	76735770
4869	NPM1		5	171387116	171411137
64219	PJA1	X		69160851	69165793
389197	C4orf50		4	5897373	6200555
23401	FRAT2		10	97332497	97334729
91351	DDX60L		4	168356735	168537786
163183	SYNE4		19	36003307	36008813
100287171	WASHC1		9	14475	73865
55076	TMEM45A		3	100492619	100577444
57621	ZBTB2		6	151364115	151391559
84282	RNF135		17	30970984	30999911
79777	ACBD4		17	45132600	45144181
NA	RPL24P4		6	42956345	42956765
399664	MEX3D		19	1554669	1568058
51649	MRPS23		17	57834781	57850056
53829	P2RY13		3	151326312	151329549
9966	TNFSF15		9	114784652	114806039
285973	ATG9B		7	151012209	151024499
284459	ZNF875		19	37312837	37369365
286451	YIPF6	X		68498562	68537282
26137	ZBTB20		3	114314501	115147271

90355	MACIR		5	103258763	103278660
57463	AMIGO1		1	109504178	109509738
169693	TMEM252		9	68536580	68540883
NA	CELF2-AS1		10	11316833	11319884
84967	LSM10		1	36391238	36397908
55343	SLC35C1		11	45804072	45813016
79673	ZNF329		19	58126248	58155110
NA	LINC02724		11	64449074	64451657
84890	ADO		10	62804720	62808479
5571	PRKAG1		12	49002274	49018807
64963	MRPS11		15	88467453	88480776
6635	SNRPE		1	203861599	203871152
219790	RTKN2		10	62183035	62268844
51363	CHST15		10	124007668	124093598
NA	OGFRP1		22	42269703	42279534
84629	TNRC18		7	5306790	5425414
161291	TMEM30B		14	61277370	61281761
55505	NOP10		15	34341719	34343136
11022	TDRKH		1	151770107	151791416
64764	CREB3L2		7	137874979	138002086
NA	TP53TG1		7	87322943	87345528
56963	RGMA		15	93035273	93089204
7318	UBA7		3	49805209	49813953
348378	SHISAL2A		1	52633168	52669683
5890	RAD51B		14	67819779	68730218
23641	LDOC1	X		141111605	141177129
2131	EXT1		8	117794490	118111826
6472	SHMT2		12	57229573	57234935
10159	ATP6AP2	X		40579372	40606848
25825	BACE2		21	41167801	41282530
7325	UBE2E2		3	23203020	23591794
338707	B4GALNT4		11	369499	382117
65265	C8orf33		8	145052465	145066685
342945	ZSCAN22		19	58326994	58342332
143879	KBTBD3		11	106051098	106077459
785	CACNB4		2	151832768	152099475
122553	TRAPPC6B		14	39147811	39170532
128553	TSHZ2		20	52972358	53495330
23265	EXOC7		17	76081016	76121576
NA	NCF1B		7	73220624	73235945
79598	CEP97		3	101724593	101770562
3985	LIMK2		22	31212239	31280080
84975	MFSD5		12	53251251	53254406

55256	ADI1	2	3497366	3519531
201305	SPNS3	17	4433940	4488208
6304	SATB1	3	18345377	18445588
1436	CSF1R	5	150053291	150113372
2049	EPHB3	3	184561785	184582408
22906	TRAK1	3	42013802	42225890
23236	PLCB1	20	8077251	8968360
348235	SKA2	17	59109857	59155260
7267	TTC3	21	37073226	37203112
283870	BRICD5	16	2209253	2211950
492311	IGIP	5	140125937	140129392
164091	PAQR7	1	25861210	25871253
338442	HCAR2	12	122701293	122703357
79098	C1orf116	1	207018522	207032756
55794	DDX28	16	68020916	68023232
29035	C16orf72	16	9091644	9121635
79087	ALG12	22	49900229	49918438
NA	PRKCZ-AS1	1	2181794	2184389
222487	ADGRG3	16	57668277	57689378
28970	C11orf54	11	93741591	93764749
80254	CEP63	3	134485721	134575017
6734	SRPRA	11	126262938	126269144
10473	HMGN4	6	26538366	26546933
221178	SPATA13	13	23979805	24307074
389114	ZNF662	3	42906142	42919334
5831	PYCR1	17	81932384	81942412
84316	NAA38	17	7856685	7885238
199675	MCEMP1	19	7676628	7679826
161	AP2A2	11	924894	1012245
6546	SLC8A1	2	40097270	40611053
57118	CAMK1D	10	12349482	12835545
4703	NEB	2	151485336	151734487
11251	PTGDR2	11	60850933	60855950
285753	CEP57L1	6	109095110	109163932
2842	GPR19	12	12660890	12696207
338773	TMEM119	12	108589851	108598320
2188	FANCF	11	22622533	22625823
390637	GDPGP1	15	90233808	90245811
NA	LINC01547	21	44932814	44939937
9403	SELENOF	1	86862445	86914424
27439	TMEM121B	22	17116297	17121367
54880	BCOR	X	40049815	40177329
163351	GBP6	1	89364059	89388160

2275	FHL3	1	37996770	38005606
126075	CCDC159	19	11344684	11354944
54101	RIPK4	21	41739369	41767089
29933	GPR132	14	105049395	105065445
4600	MX2	21	41361999	41409393
57634	EP400	12	131949942	132080460
8624	PSMG1	21	39174769	39183488
54039	PCBP3	21	45643694	45942454
84133	ZNRF3	22	28883572	29057488
119559	SFXN4	10	119140767	119165714
220929	ZNF438	10	30820207	31031937
56941	HMCES	3	129278828	129306186
1232	CCR3	3	46163604	46266706
348327	ZNF530	19	57599885	57612722
4707	NDUFB1	14	92116122	92121917
64410	KLHL25	15	85759326	85794925
10189	ALYREF	17	81887835	81891586
359845	RFLNB	17	439978	445939
9241	NOG	17	56593699	56595611
7378	UPP1	7	48088628	48108736
23585	TMEM50A	1	25338317	25362361
29110	TBK1	12	64452092	64502114
346389	MACC1	7	20134655	20217404
10607	TBL3	16	1972053	1982929
83999	KREMEN1	22	29073078	29168333
10293	TRAIP	3	49828601	49856574
80139	ZNF703	8	37695782	37700019
NA	DOCK8-AS1	9	212824	215893
51807	TUBA8	22	18110331	18146554
1233	CCR4	3	32951644	32956349
114781	BTBD9	6	38168451	38640148
128239	IQGAP3	1	156525405	156572604
10766	TOB2	22	41433494	41446801
6331	SCN5A	3	38548057	38649687
5613	PRKX	X	3604340	3713649
387893	KMT5A	12	123383773	123409353
256435	ST6GALNAC3	1	76074746	76634603
71	ACTG1	17	81509971	81523847
23258	DENND5A	11	9138825	9265350
56616	DIABLO	12	122207668	122227456
55803	ADAP2	17	30906344	30959322
NA	SREBF2-AS1	22	41831215	41834665
29796	UQCR10	22	29767369	29770413

54954	FAM120C	X		54068324	54183281
8508	NIPSNAP1		22	29554808	29581327
220074	LRTOMT		11	72080331	72110782
388581	C1QTNF12		1	1242446	1246722
79174	CRELD2		22	49918167	49927540
152579	SCFD2		4	52872982	53366066
140739	UBE2F		2	237966827	238042782
5504	PPP1R2		3	195514428	195543386
64061	TSPYL2	X		53082367	53088540
3654	IRAK1	X		154010506	154019902
116448	OLIG1		21	33070141	33072413
5463	POU6F1		12	51186936	51217708
116534	MRGPRE		11	3225030	3232417
1435	CSF1		1	109910242	109930992
116447	TOP1MT		8	143304384	143359979
9276	COPB2		3	139355600	139389680
80764	THAP7		22	20999104	21002196
9735	KNTC1		12	122527246	122626396
10587	TXNRD2		22	19875517	19941820
348013	TMEM255B		13	113759226	113816995
374659	HDDC3		15	90929968	90935196
340542	BEX5	X		102153708	102155977
9021	SOCS3		17	78356778	78360077
57121	LPAR5		12	6618835	6635960
90134	KCNH7		2	162371407	162838767
4753	NELL2		12	44508275	44921848
81931	ZNF93		19	19900913	19963464
10801	SEPTIN9		17	77280569	77500596
157313	CDCA2		8	25459199	25507911
9923	ZBTB40		1	22428838	22531157
8349	H2BC21		1	149884459	149886652
5413	SEPTIN5		22	19714503	19724224
56478	EIF4ENIF1		22	31436977	31496108
55328	RNLS		10	88273864	88584530
55911	APOBR		16	28494643	28498970
25923	ATL3		11	63624087	63671921
79135	APOO	X		23833353	23907938
54732	TMED9		5	177592203	177597242
25880	TMEM186		16	8780384	8797642
155435	RBM33		7	155644451	155781485
90135	BTBD6		14	105248490	105251093
8971	H1-10		3	129314771	129316286
375287	RBM43		2	151247940	151261863

83943	IMMP2L	7	110662644	111562517
3714	JAG2	14	105140982	105168824
391356	PTRHD1	2	24789734	24793382
79050	NOC4L	12	132144457	132152473
11274	USP18	22	18150170	18177397
4700	NDUFA6	22	42085526	42090884
57537	SORCS2	4	7192538	7742836
113277	TMEM106A	17	43211835	43220041
140707	BRI3BP	12	124993645	125031231
2157	F8	X	154835788	155026940
23764	MAFF	22	38200767	38216507
2972	BRF1	14	105209286	105315589
7469	NELFA	4	1982717	2041903
80789	INTS5	11	62646848	62653302
51065	RPS27L	15	63125872	63158021
11124	FAF1	1	50437028	50960267
131583	FAM43A	3	194685883	194689037
3297	HSF1	8	144291591	144314720
5813	PURA	5	140107777	140125619
83881	MIXL1	1	226223618	226227054
114659	LRRC37B	17	32007872	32053504
NA	LINC00482	17	81303771	81309248
10581	IFITM2	11	307631	315272
7127	TNFAIP2	14	103123442	103137439
353274	ZNF445	3	44431705	44477670
9230	RAB11B	19	8389981	8404434
7625	ZNF74	22	20394115	20408461
285600	KIAA0825	5	94152966	94618597
283991	UBALD2	17	76265348	76271298
54033	RBM11	21	14216130	14228372
84614	ZBTB37	1	173868082	173903549
8651	SOCS1	16	11254408	11256204
6948	TCN2	22	30607003	30627271
23545	ATP6V0A2	12	123712353	123761755
126282	TNFAIP8L1	19	4639516	4655568
5600	MAPK11	22	50263713	50270767
93349	SP140L	2	230327184	230403732
123283	TARS3	15	101653596	101724473
64754	SMYD3	1	245749342	246507312
400451	FAM174B	15	92617448	92809884
374920	ZSWIM9	19	48170680	48197620
55010	PARPBP	12	102120185	102197520
246329	STAC3	12	57243453	57251188

4582	MUC1	1	155185824	155192916
3665	IRF7	11	612553	615983
5148	PDE6G	17	81650459	81663112
6667	SP1	12	53380176	53416446
64976	MRPL40	22	19431902	19436075
89782	LMLN	3	197960200	198043720
5034	P4HB	17	81843161	81860624
677	ZFP36L1	14	68787660	68796253
4733	DRG1	22	31399604	31530634
51479	ANKFY1	17	4163821	4263995
79943	ZNF696	8	143289676	143299952
3434	IFIT1	10	89392546	89406487
159013	CXorf38	X	40626921	40647561
79581	SLC52A2	8	144354135	144361272
10320	IKZF1	7	50304068	50405101
5833	PCYT2	17	81900958	81911432
10134	BCAP31	X	153700492	153724565
54584	GNB1L	22	19783223	19854939
2124	EVI2B	17	31303770	31314105
79896	THNSL1	10	25016612	25026664
140691	TRIM69	15	44728988	44767829
8519	IFITM1	11	313506	315272
3916	LAMP1	13	113297239	113323672
2865	FFAR3	19	35358460	35360489
84197	POMK	8	43093506	43123434
200942	KLHDC8B	3	49171598	49176486
55599	RNPC3	1	103525691	103555239
10308	ZNF267	16	31873807	31917357
402573	C7orf61	7	100456620	100464260
55217	TMLHE	X	155489011	155669944
84924	ZNF566	19	36445119	36489902
57711	ZNF529	19	36534774	36605276
NA	MATN1-AS1	1	30718504	30726827
84529	C15orf41	15	36579626	36810248
3852	KRT5	12	52514575	52520530
23396	PIP5K1C	19	3630183	3700468
8529	CYP4F2	19	15878023	15898077
130355	C2orf76	2	119302225	119366834
51741	WWOX	16	78099430	79212667
NA	CIDECP1	3	10014238	10026365
283149	BCL9L	11	118893875	118925608
146909	KIF18B	17	44924709	44947773
84937	ZNRF1	16	74999024	75110994

128178	EDARADD		1	236348257	236502915
64757	MTARC1		1	220786913	220819659
55330	BLOC1S4		4	6716174	6717664
57496	MRTFB		16	14071319	14266773
64222	TOR3A		1	179082070	179098023
4675	NAP1L3	X		93670930	93673578
NA	CA5BP1	X		15674932	15703724
200035	NUDT17		1	145845630	145848954
342510	CD300E		17	74609885	74623738
728621	CCDC30		1	42463221	42657190
55922	NKRF	X		119588337	119606443
342035	GLDN		15	51341655	51408005
2204	FCAR		19	54874235	54891420
4674	NAP1L2	X		73212299	73214851
11118	BTN3A2		6	26365159	26378320
[truncated: 1,378,134 more chars]
